# Identification and comparative genomic analysis of prophage sequences and CRISPR‒Cas immunity in *Methylococcus* genomes: insights into industrial methane bioconversion

**DOI:** 10.1186/s13068-026-02738-6

**Published:** 2026-01-29

**Authors:** Irina Nizovtseva, Alexey Rezaykin, Aleksandra Korenskaia, Maksim Zakhartsev, Alina Chigireva, Ilya Starodumov, Dmitrii Chernushkin

**Affiliations:** 1 Otto-Schott-Institut Für Materialforschung, Friedrich-Schiller University of Jena, 07743 Jena, Germany; 2 Laboratory of Multiphase Physical and Biological Media Modeling, Ural Federal University, Yekaterinburg, 620000 Russia; 3 Department of Medical Physics and Digital Technologies, Ural State Medical University, Yekaterinburg, 620028 Russia; 4 The Federal Research Centre “Fundamentals of Biotechnology” of the Russian Academy of Sciences, Moscow, 119071 Russia; 5 NPO Biosintez Ltd, Moscow, 109390 Russia; 6 Tübingen, 72076 Germany

**Keywords:** Bacteriophages, Prophages, Methane, *Methylococcus*, Gaprin, Bioinformatics, Genomic analysis, CRISPR‒Cas, Comparative genomics, Biotechnology, Industrial bioconversion

## Abstract

**Background:**

*Methylococcus* species utilize methane as the sole carbon and energy source, converting it into biomass and other metabolic end products. Owing to this metabolic capacity, they hold particular promise in industrial C1 biotechnology, especially for the production of protein-rich feed. However, the industrial cultivation of *Methylococcus*-based consortia on methane is inherently nonsterile, exposing the process to potential biological risks that may compromise the stability, duration and productivity of cultivation. One of the most critical threats is bacteriophage infection, whose triggers for rapid phage-mediated lysis and resulting economic losses remain incompletely understood. Elucidating these processes is paramount for devising strategies to mitigate or prevent detrimental outcomes.

**Results:**

In this investigation, nine publicly accessible genomes of *Methylococcus* species were examined, culminating in the identification of eleven prophage sequences distributed variably among the genomes. Sequence annotations revealed that nine prophages are potentially functional and intact, whereas the rest carry incomplete gene sets indicative of nonviability. Phylogenetic analyses corroborated the substantial diversity of prophages, which formed distinct clusters related to γ-proteobacteria phages. Furthermore, comparative genomic analyses demonstrated a high degree of structural conservation despite the presence of rearrangements. The annotation of the CRISPR‒Cas systems provided insights into additional dimensions of phage‒bacteria interactions. Examination of prophage integration sites did not reveal any disruption of metabolic gene structures, thus suggesting minimal risk of deleterious phenotypic outcomes.

**Conclusions:**

These findings considerably advance the current understanding of the genetic diversity and biological properties of prophages infecting *Methylococcus* species, underscoring the importance of holistic approaches for the detection and analysis of these elements. Our findings underscore the need for routine prophage monitoring in industrial methanotrophic consortia, with the pipeline established here serving as a foundational framework for future refinement and industrial adaptation.

**Supplementary Information:**

The online version contains supplementary material available at 10.1186/s13068-026-02738-6.

## Background

Representatives of the genus *Methylococcus* are obligate aerobic methanotrophic bacteria belonging to the class Gammaproteobacteria. They are immotile, gram-negative cocci that are thermotolerant or moderately thermophilic, with optimal growth at 40–46 °C for *Methylococcus capsulatus* and up to 60 °C for thermophilic species. Owing to their capacity to oxidize methane—one of the principal greenhouse gases—they play a pivotal role in the global carbon cycle [1]. In natural environments, methanotrophs typically inhabit strata abundant in natural gas that interfaces with oxygen-bearing phases (e.g., air and water), where oxygen diffusion is sufficient to sustain methane oxidation. These habitats include swamps, soils and landfill covers; wastewater from coal mines and geothermal fields; and sediments from rivers and ponds [2].

*Methylococcus* species oxidize methane as their sole carbon and energy source to synthesize biomass and other metabolic end products. Owing to their high protein content, they are extensively employed in industrial biotechnology as producers of bacterial single-cell protein (BSCP). To stabilize and enhance the efficiency of methane-based bacterial cultivation at the industrial scale, *Methylococci* are cocultured with heterotrophic “satellite” bacteria to form active industrial consortia. These stellitebacteria metabolize the accumulating end products of *Methylococcus* species, thereby alleviating the kinetic inhibition of producer growth. The high biomass density of methanotrophic consortia attainable in the bioreactor permits operation under nonsterile conditions, substantially reducing operational costs.

BSCP derived from methane is known as a “bioprotein” in Western countries and a “gaprin” in Eastern Europe. It comprises the inactivated biomass of a nonpathogenic, methane-oxidizing bacterial community cultivated aerobically on natural gas. This biomass functions as a highly concentrated, nutritionally complete protein supplement for agricultural and aquaculture feeds, containing up to 75% (w/w) crude protein. The amino acid profile of gaprin is more complete and richer in essential amino acids than soybean or meat-and-bone meal, feed yeasts, and other plant-based meals. Moreover, it closely resembles the amino acid composition of fish meal, facilitating its substitution in feed mixtures and conferring synergistic benefits when used concurrently.

From a technological perspective, the production of BSCP from methane by methanotrophs is relatively straightforward, as it does not necessitate removing the substrate from the cultivation broth containing the bacterial biomass, thereby simplifying downstream processing. Conversely, methane-based cultivation apparatuses require complex engineering solutions to meet the heightened mass- and heat-transfer demands of bioreactors. In all gaprin production technologies, the process is continuous (employing a flow-through cultivation mode) and proceeds through several sequential stages: (i) preparation of the nutrient substrate; (ii) cultivation of biomass on methane; (iii) separation of the cultivation broth; (iv) thickening of the microbial suspension; (v) inactivation of bacterial biomass; (vi) drying, packaging, and packing of the final product; and (vii) treatment of process water. Thus, the duration of a single cultivation process is a crucial economic parameter in gaprin production technology.

From an economic standpoint, large-scale production of gaprin is more cost-effective than conventional methods for producing high-protein feed ingredients of comparable nutritional value (such as fish meal, plant-based proteins, or yeast-derived proteins). This advantage stems from the absence of requirements for fleets, farmland, irrigation, fertilizers, and associated logistics and field management expenses, as well as its independence from seasonal and climatic conditions and its lower specific water consumption. Moreover, gaprin offers high nutritional value. When properly configured, methane-based industrial consortia centered on *Methylococcus* exhibit robust process productivity, marked by elevated specific growth rates at high biomass densities in the bioreactor. Gaprin can also be produced according to planned schedules and has favorable storage characteristics (with a shelf life of up to two years). Consequently, gaprin constitutes an attractive alternative to fish meal, plant proteins, yeast proteins, and related feedstuffs.

Hence, the industrial production of gaprin is a continuous, nonsterile process (i.e., an open aseptic process), which entails biological risks to both its stability and efficiency. One major concern is bacteriophage infection, since phages can infiltrate production lines even when sterile gases and water are used, where they can enter through various sources, such as equipment, raw materials, personnel, or ambient air [3]. Once phages switch to the lytic cycle, cultivation failure ensues. In large-volume bioreactors, such failure constitutes a significant production setback: an immediate loss of substantial amounts of the product, process downtime, equipment cleaning, and the logistical challenge of rapidly disposing of large volumes of lysate, among other issues.

**Fig. 1:**
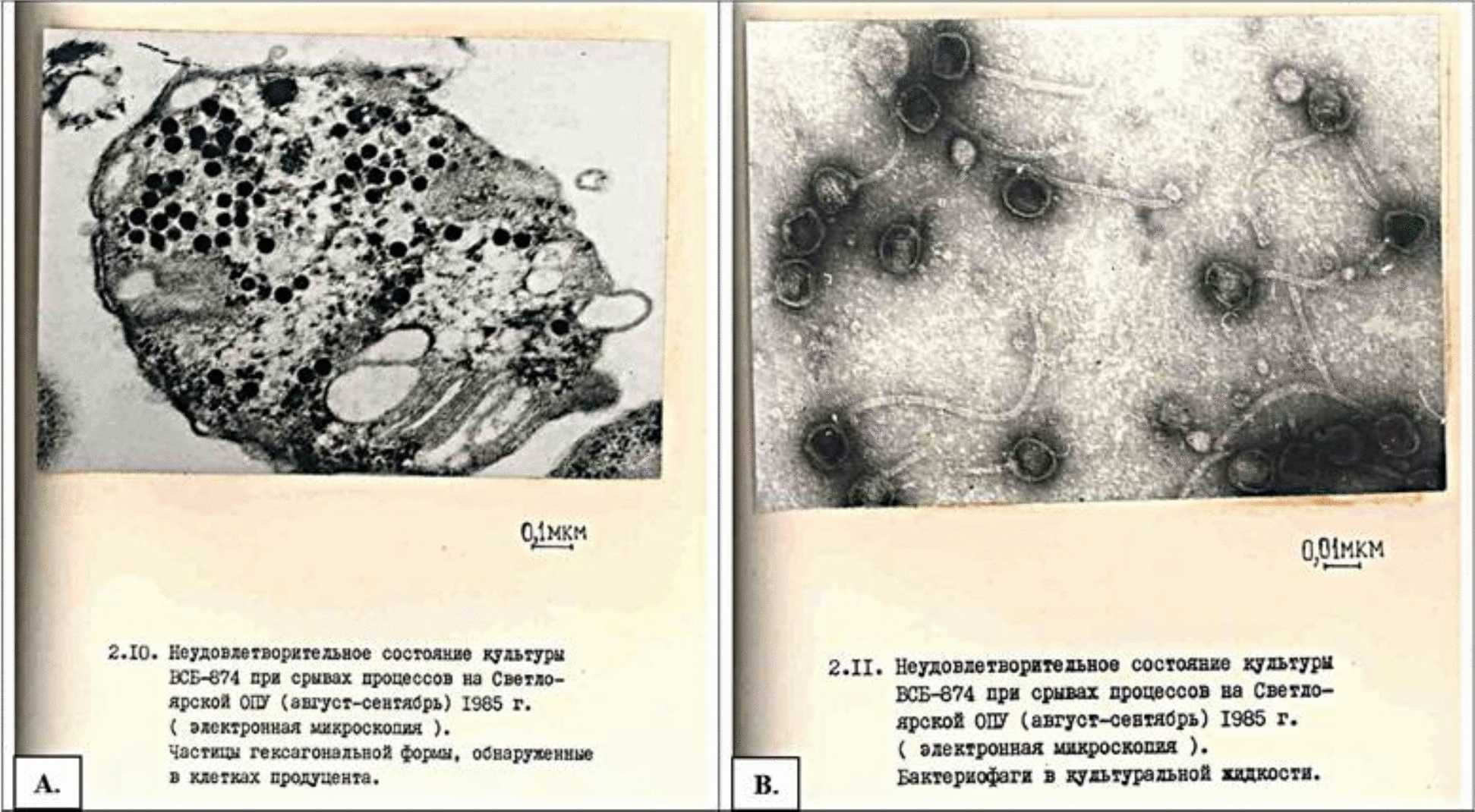
Historical evidence of phage-related disruption in VSB-874 culture, as documented by electron microscopy. **A** hexagonal-shaped viral particles within producer cells. **B** free bacteriophages in the culture medium

Historical records further substantiate these concerns. Indeed, this challenge is by no means a recent discovery; indeed, classical references kindly made available by the authors of this study confirm that it has preoccupied practitioners since the earliest days of industrial gaprin production—the advent of modern comparative methodologies now enables a far more nuanced understanding of phage-related disruptions. The archived reports labelled 2.10 and 2.11 (Fig. 1) from the Album of the VSB-874 culture, a gaprin producer, and its associated microflora were cultivated under laboratory, pilot, and semiindustrial conditions (originally in Russian, *“Aльбoм кyльтypы BCБ-874—пpoдyцeнтa гaпpинa и coпyтcтвyющeй микpoфлopы, выpaщивaeмoй в кaмepaльныx, oпытныx и oпытнo-пpoмышлeнныx ycлoвияx”*) serve as particularly compelling historical evidence. Document 2.10, Fig. 1A “Unsatisfactory condition of the VSB-874 culture during process failure at the Svetloyarsk pilot production facility (August–September 1985) (electron microscopy). Hexagonal-shaped particles detected within producer cells” (originally in Russia, *“Heyдoвлeтвopитeльнoe cocтoяниe кyльтypы BCБ-874 пpи cpывax пpoцeccoв нa Cвeтлoяpcкoм OПУ (aвгycт–ceнтябpь) 1985 г. (элeктpoннaя микpocкoпия). Чacтицы гeкcaгoнaльнoй фopмы, oбнapyжeнныe в клeткax пpoдyцeнтa”*) details hexagon-shaped particles within producer cells, while 2.11, Fig. 1B (Unsatisfactory condition of the VSB-874 culture during process failure at the Svetloyarsk pilot production facility (August–September 1985) (electron microscopy). The bacteriophages detected in the culture fluid, originally in Russia, were *“Heyдoвлeтвopитeльнoe cocтoяниe кyльтypы BCБ-874 пpи cpывax пpoцeccoв нa Cвeтлoяpcкoм OПУ (aвгycт–ceнтябpь) 1985 г. (элeктpoннaя микpocкoпия). Бaктepиoфaги в кyльтypaльнoй жидкocти”*) describes bacteriophages detected in the culture fluid. These findings underscore both the longstanding and pervasive nature of phage infestations in large-scale methane-based bioprocesses.

Building upon these historical insights, contemporary genomic methodologies now provide an unprecedented depth of understanding and allow for a precise evaluation of prophage–host interactions. The use of high-resolution genomic analyses, coupled with robust comparative approaches, significantly advances our ability to detect, monitor, and predict potential phage induction events that might disrupt large-scale production processes. In addition to these capabilities, state-of-the-art computational tools empower researchers with predictive analytics and real-time optimization strategies. Specifically, when combined with contemporary technologies such as computational fluid dynamics (CFD), machine learning (ML), computer vision (CV), and deep learning [4–11], scientists and engineers now have powerful resources at their disposal to predict and prevent conditions leading to phage-induced culture failure. Thus, an inherently biological challenge becomes manageable and findings may inform future strategies for improving stability in methane-based bioproduction systems, such as reliability, economical and sustainability aspects in gaprin production.

**Fig. 2:**
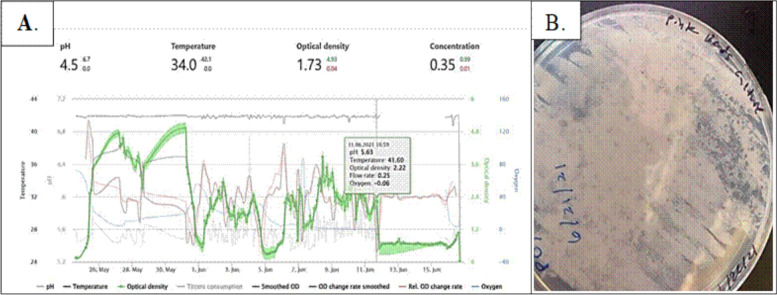
Real-world example of process disruption using laboratory-cultivated *Methylocystis*. **A** Monitoring data indicating abrupt changes in key operational parameters (temperature, pH, optical density, and dissolved oxygen), indicative of imminent culture instability and failure. **B** Morphological abnormalities in *Methylocystis* cultures postfailure, as evidenced by atypical colony formation and significant discoloration. These images underscore the critical importance of ongoing microbial monitoring, diagnostics, and proactive management strategies

To illustrate the practical importance of proactive monitoring, we present a real-world scenario demonstrating a severe case of process disruption documented during laboratory-scale cultivation of another methanotrophic bacterium, *Methylocystis* (Fig. 2). The data clearly illustrate how quickly and unexpectedly phage-induced or other microbial disturbances can arise, causing catastrophic culture collapse even under controlled laboratory conditions. Early sampling and preliminary measurements did not clearly indicate any deviation, highlighting the difficulty of identifying early indicators of culture instability without continuous, sensitive monitoring systems. When the optical density decreased and severe instability in pH and temperature became apparent, the process was compromised. Comprehensive microbiological diagnostics performed in collaboration with specialists from San Diego State University revealed underlying microbial stress consistent with contamination, confirming these suspicions. Such practical experiences further reinforce the value of routine, proactive phage and microbial assessments, which enable operators to intervene before irreversible damage occurs. Although acknowledging the differences between *Methylococcus* and the laboratory-grown *Methylocystis* example, we reiterate that the critical relevance lies in the shared vulnerability of methane-based SCP production processes to a variety of microbial disturbances and contamination issues, particularly bacteriophages. The results reinforce the relevance of prophage awareness in designing more robust microbial production platforms. We recognize that practical industrial scenarios may necessitate slightly modified methodologies for prophage and bacteriophage detection, particularly when lysis is already suspected. Nevertheless, we believe that the comprehensive algorithm developed within the scope of this study provides a robust reference framework. Future research objectives include refining this framework to ensure optimal relevance and effectiveness under specific industrial conditions, enhancing both predictive capability and operational robustness for commercial gaprin production.

Building upon these practical insights and emphasizing the broader relevance of our investigation, we emphasize that the advanced genomic and computational tools discussed above bring us significantly closer to addressing one of the most persistent biological challenges: the threat posed by prophages introduced as latent viral genomes within library-derived bacterial cultures. Prophages represent the latent form of a virus within a bacterial cell. Under conditions of host-cell damage or other stress factors, prophage induction may occur, triggering rapid onset of the lytic cycle and, in turn, cultivation failure. Moreover, prophage infection can pose an additional threat to culture productivity by leading to the loss of certain metabolic reactions and thus diminished biomass growth efficiency. As discussed above, establishing a prophage monitoring pipeline could be a promising future application of this framework. The identification of prophage sequences in sequenced bacterial genomes poses considerable technical challenges. At present, there are no uniform criteria for detecting or determining the functional status of prophages. Prophages may reside within the bacterial genome in diverse forms, ranging from unmodified, inducible sequences to nonviable remnants produced by deletions, insertions, or genetic rearrangements. Moreover, the pool of prophage genes that are sufficiently conserved, reliably distinct from bacterial genes, and thus capable of serving as clear prophage signatures remains quite limited [12, 13].

Bacteriophages are viruses that act as obligate parasites of bacteria and are considered the most numerous biological entities on Earth, with a global population estimated at approximately 10^31^ particles [14, 15]. The modes of phage–host interaction can vary significantly. Certain bacteriophages follow a lytic life cycle, rapidly killing the bacterial host through replication and subsequent release of progeny virions. Others are lysogenic, integrating their genomes into the bacterial chromosome for an indefinite period and forming prophages. In this state, the integrated viral genome is passed on to daughter cells during bacterial replication, potentially persisting in the population indefinitely. Notably, lysogenic phages can switch to the lytic cycle under specific induction conditions; if such induction is possible, they are classified as functional or inducible prophages. However, genetic rearrangements—such as deletions, insertions, or translocations—can abrogate a prophage’s ability to induce lysis, rendering it nonfunctional or cryptic [12, 16].

In earlier work, two putative prophages measuring 58.5 Kbp and 45 Kbp in length were identified in the genome of *Methylococcus capsulatus* Bath, whereas potentially complete prophages were found in the genomes of *Methylococcus capsulatus* strains IO1 and KN2 [2, 17]. These discoveries underscore the breadth of prophage diversity in *Methylococcus* and highlight the need for more refined strategies to detect and characterize such elements in industrially relevant methanotrophs.

To further elucidate which phages may infect Methylococcus species, examining the population’s (or its ancestors’) infection history with various prophages would be informative. This record may be preserved through the clustered regularly interspaced short palindromic repeats (CRISPR‒Cas) system, which stores short segments of foreign DNA (phages or plasmids) within the bacterial genome [18]. These approximately 30-nucleotide segments, called *spacers*, alternate with direct repeats, collectively forming a CRISPR array. On one side of this array lies the leader sequence, where new spacers are added. If the same foreign DNA reenters the cell, the CRISPR‒Cas system recognizes and cleaves it via Cas endonucleases, thereby safeguarding the cell against phages. When comparing these spacer sequences with bacteriophage genomes is widely employed to identify potential phage hosts [19, 20].

In our study, publicly available genomes of the genus *Methylococcus* were analysed, including the following strains of *Methylococcus capsulatus*: Bath, BH, KN2, IO1, Mc(Nor), and MIR; *Methylococcus mesophilus* 16–5; *Methylococcus geothermalis* IM1; and *Methylococcus* sp. Mc7. An initial screening of these *Methylococcus* genomes was performed, followed by comparative and phylogenetic analyses of the detected prophage sequences. The primary objective of this study was to identify and characterize prophage sequences and CRISPR–Cas immune elements in publicly available *Methylococcus* genomes as a foundation for evaluating the risk of phage infection in open bioprocesses. Identifying and examining prophages is critical for elucidating the evolutionary history of methanotrophs and their interactions with the surrounding environment. Prophages can affect bacterial metabolism, virulence, and other phenotypic traits; they may also serve as reservoirs of novel genes and functions. These findings are particularly relevant in open-system industrial operations, where the likelihood of culture infection is increased and close monitoring of prophages becomes paramount.

Consequently, there is a need to establish a pipeline for identifying and monitoring prophage insertions within the metagenome of the methane-oxidizing community employed in industrial feed–biomass production. Such a pipeline would aid in detecting factors that trigger phage-induced lysis of the biomass, potentially helping to prevent process failure once it is validated and implemented in an industrial setting. Hence, in addition to the fundamental interest in questions pertaining to the evolutionary dynamics of methanotrophic bacteria and their phage interactions, our study aims to establish foundational knowledge and methodologies that could guide future industrial applications, with the long-term goal of improving the stability and profitability of large-scale methane-based cultivation systems.

## Materials and methods

### Methylococcus genomes

Nine complete, unannotated genomes of methanotrophic bacteria from the genus *Methylococcus* were obtained from the RefSeq database of the National Center for Biotechnology Information (NCBI) (https://www.ncbi.nlm.nih.gov/refseq/). These included the genomes of six strains of *Methylococcus capsulatus*, one genome each of *M. geothermalis* and *M. mesophilus*, and one genome with an undetermined species assignment. The strains were isolated between 2004 and 2020 in four countries (Russia, the USA, the United Kingdom, and South Korea) from various environmental sources (including sediment and sludge, landfill and rice paddy soils, and hot springs). The set also included the genome of strain Bath (NCIMB 11132), which is stored in the National Collection of Industrial and Marine Bacteria (Aberdeen, United Kingdom) and is recognized as both the first fully sequenced and the most extensively characterized genome of *M. capsulatus*. The genome sizes of the analysed strains ranged from 3187 to 4443 kb. More detailed information can be found in Table S1 [see Supplementary Materials 1]. Genome annotation was performed with Prokka [50].

A species‑level phylogenetic tree was constructed on the basis of 1627 single-copy genes shared among the analysed strains. These genes were identified via OrthoFinder [51], after which the respective sequences for each strain were concatenated into a single, combined amino acid sequence. The resulting sequences were aligned with MAFFT [52] and trimmed of gaps via trimAl [53]. A phylogenetic tree was then constructed with IQ-TREE (with partitioning of the alignment into 1627 segments and model selection for each segment, parameters -bb 1000 -alrt 1000) and visualized via iTOL [54].

### Prophage identification in bacterial genomes

Searching for new prophages requires multiple lines of evidence [21], so at the first stage, each bacterial genome was investigated for potential prophage sequences via four programs: (1) PHASTEST [22] (default parameters with the “deep” gene annotation mode); (2) Phigaro (version 2.4.0) [23] (default parameters, employing the pVOGs database of prokaryotic viral orthologous groups [27]; (3) VIBRANT (version 1.2.0) [24] (default parameters); and (4) PhiSpy (version 4.2.21) [25] (default parameters, using the universal PhiSpy training dataset).

All of these tools initially predict protein-coding genes via Prodigal [26] and then annotate these genes as bacterial, viral, or unknown according to their respective pipelines. PHASTEST identifies phage proteins by searching for homology against the virus sequences in NCBI RefSeq; Phigaro implements a similar approach but relies on the hidden Markov model (HMM) database of prokaryotic virus orthologous groups (pVOGs) [27]. VIBRANT uses a hybrid methodology that combines neural networks with homology searches, whereas PhiSpy likewise employs a hybrid strategy. PhiSpy computes a feature set—including protein length, transcriptional strand orientation, AT/GC ratios, frequency of unique phage-specific 12-nt “words”, and phage integration sites—to feed a random forest classifier that assigns a phage probability to each gene; in parallel, it searches for homologues via HMM profiles from three databases: KEGG [28], Pfam [29], and virus orthologous groups (VOGs) [30]. Each program then clusters its potential phage genes into putative prophage sequences, taking into account the number and proximity of phage-like genes, as well as other relevant features. PHASTEST and VIBRANT additionally provide an overall “score” for the identified regions.

This set of programs was selected to leverage distinct yet complementary search algorithms. The quality of the putative prophage sequences was evaluated with CheckV [31]. Pairwise alignment of sequences to detect disruptions in syntenic blocks within the bacterial genome at potential phage integration sites was performed with ProgressiveMauve [32].

### Prophage annotation and morphological classification

All prophage sequences were annotated via Pharokka v1.5.0 [36]. Specifically, coding sequences (CDSs) were predicted via PHANOTATE v1.5.0 [55], tRNAs were identified via tRNAscan-SE v2.0 [56], and tmRNAs were predicted via Aragorn v1.2.38 [57]. Functional annotation was obtained by mapping each CDS to the PHROGs [58], VFDB [59], and CARD [60] databases via MMseqs2 [61]. Phage morphology was determined on the basis of the presence or absence of characteristic structural proteins. A tail sheath protein indicates a myoviral (contractile-tailed) morphology, whereas a tail tape measure protein without a tail sheath protein indicates a siphoviral morphology [62, 63].

### Prophage genome and proteome analyses

Phylogenetic analysis was conducted via the VICTOR web service (https://victor.dsmz.de), which applies a genome-based method for classifying and inferring the phylogeny of prokaryotic viruses [64]. All pairwise comparisons of nucleotide sequences were performed via the Genome BLAST Distance Phylogeny (GBDP) method [65] under the conditions recommended for prokaryotic viruses [64]. The resulting intergenomic distances were used to compute branch lengths in the phylogenetic tree via the balanced minimum evolution approach. Branch support was determined from 100 pseudoreplicates via FASTME, including SPR postprocessing [66]. The trees were midpoint-rooted [67] and visualized with ggtree [68]. Taxonomic boundaries at the species, genus, and family levels were defined via the OPTSIL program [69], applying recommended clustering thresholds [64] and an F value (the proportion of linkages required to merge clusters) of 0.5 [64]. A pairwise genome similarity matrix was computed with the VIRIDIC web server (http://rhea.icbm.uni-oldenburg.de/VIRIDIC/) [33] via default parameters. In accordance with ICTV recommendations for virus taxonomy, the intergenomic similarity threshold for species demarcation was set at 95%, and for genus demarcation, it was set at 70%. Proteome-based phylogenetic trees were constructed with VipTree 3.3 [35], employing default parameters and linear branch-length scaling. Reference virus genomes of prokaryotic dsDNA viruses were included in the analysis for comparative purposes. Comparative alignments of prophage sequences and intergenomic comparison diagrams were generated via Clinker (v. 0.0.31) [69] with default settings.

### CRISPR analysis

The genomes were screened for CRISPR‒Cas systems via CRISPRCasFinder [39]. For each identified CRISPR array containing at least one spacer located adjacent to *cas* genes, forward or reverse-complement spacers were extracted depending on the orientation predicted by CRISPRCasFinder. The spacer sequences were compared against the predicted prophage genomes via SpacePHARER [41], a tool that offers high accuracy and sensitivity for identifying CRISPR–phage matches. The algorithm operates on both the protein and nucleotide levels, performing searches with the VTML40 substitution matrix (gap open cost = 16; gap extension cost = 2) at the protein level, followed by nucleotide re-alignment (match reward = 1, mismatch penalty = 1, gap open cost = 10, and gap extension cost = 2) to prioritize near-perfect (up to 1–2 mismatches) nucleotide matches. The combined score for multiple spacers matching the same phage genome is then evaluated for a false discovery rate (FDR) < 0.05, as calculated against a null model database, ensuring the accuracy of the tool’s predictions. The annotations of the spacer-mapping loci were retained from the prophage annotations described in the section *Prophage Annotation and Morphological Classification*. All phages listed in Table S2 [see Supplementary Materials 1] were used as putative prophage genomes. The same software was then employed to compare these spacers with prophage genomes from the PhageScope database [37], which incorporates data from RefSeq [70], GenBank [71], EMBL [72], DDBJ [73], PhagesDB [74], GOV2 [75], GVD [76], GPD [77], MGV [31, 78], CHVD [79], STV [80], TemPhD [81], IGVD [82] and IMG/VR [83].

### Analysis of prophage integration sites in the bacterial genome

For prophages with refined boundaries (*Bath-R1, Bath-R2, KN2-R1, IO1-R1, McNor-R1,* and *McNor-R2*), as determined via synteny analysis of insertion regions with the *MIR* strain genome, annotated genome records produced by Prokka were examined. In the genomes of the *Bath*, *KN2*, *IO1*, and *Mc (Nor)* strains, the genes nearest to the prophage boundaries were analysed. Genes located within 1000 nucleotides of the boundary and annotated as anything other than “hypothetical protein” were compared with their homologues in the MIR strain, including comparisons of their lengths. To detect any loss of gene functionality resulting from prophage insertion, the set of genes annotated at the boundaries of the prophage insertion was checked against annotations in the homologous region of MIR.

In addition, for any genes whose metabolic function could be compromised by prophage insertion (due to disruption of the gene itself or its regulatory region), the presence of duplicated copies was assessed on the basis of the number of annotated gene products in the respective genome.

## Results

### Predicting prophages in the genomes of Methylococcus species

**Fig. 3:**
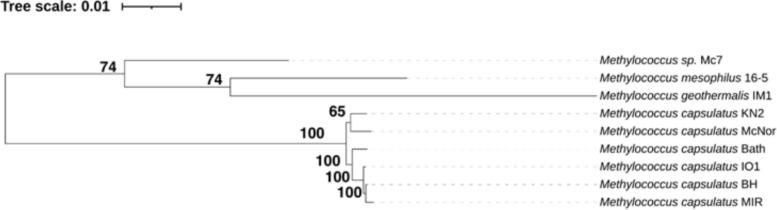
Species-level phylogenetic tree constructed by IQ-TREE, which uses the amino acid sequences of 1.627 single-copy orthologous genes

The dataset examined in this study comprises nine complete, unannotated genomes of *Methylococcus* isolates obtained from the RefSeq database. To situate the identified bacteriophages within an evolutionary context, a species-level phylogenetic tree was constructed (see Fig. 3).

Because the discovery of novel prophages necessitates multiple complementary approaches [21], each *Methylococcus* genome was initially screened for potential prophage regions via four bioinformatic algorithms: PHASTEST [22], Phigaro (version 2.4.0) [23], VIBRANT [24], and PhiSpy [25]. As a result of this initial search, a set of putative prophage sequences is obtained. However, the regions identified by different algorithms often overlap only partially, vary in length, or encode distinct sets of genes. Therefore, in the second stage, any overlapping or closely related sequences are merged, while low-quality regions that fail to meet the selection criteria are discarded. These criteria include the following: (1) the prophage sequence is identified by more than one algorithm; (2) its length exceeds 10 kb; (3) it contains at least ten phage-specific genes; (4) it has an average or higher PHASTEST/VIBRANT score; and (5) it harbors characteristic prophage attachment sites (attR, attL).

Next, the size and gene complement of the merged prophage sequences are refined via CheckV [31], and any regions that fail to pass CheckV verification for putative prophage status are removed from further consideration. Finally, the boundaries of the remaining prophage sequences are adjusted by pairwise alignment with “uninfected” reference genomes of closely related bacterial strains, taking into account any disruptions to bacterial genes or syntenic blocks. If a prophage disrupts a gene or syntenic region, the prophage boundary is shifted accordingly. This alignment process highlights syntenic blocks between the surrounding genome region and a phage-free reference genome, clarifying the precise site of prophage insertion. Pairwise alignments are performed with ProgressiveMauve [32].

Through this pipeline (see Fig. 11), the final set of predicted prophage regions is derived. Once these regions were delineated, they served as the foundation for subsequent comparative and phylogenetic analyses. In total, 68 potential prophage regions were detected, with the largest contribution—21 regions (30.9%)—identified by Phigaro, followed by PhiSpy, with 19 regions (27.9%), and PHASTEST and VIBRANT, with 14 regions (20.6%) each. The size of the detected regions ranged from 51.1 kb (24.4–62.2) via VIBRANT to 27.2 kb (11.4–46.2) via Phigaro, although no statistically significant variation among the algorithms was observed. The detailed results from this initial stage are provided in Table S2 [see Supplementary Materials 1].

The regions identified by different algorithms often overlap only partially, showing variability in both size and gene content. Accordingly, any overlapping or closely situated regions were merged, while low-quality regions that did not satisfy the specified selection criteria were discarded. The size and gene complement of each merged prophage region were then refined via CheckV [31], and sequences failing to meet the requirements for putative prophages were removed from subsequent analysis. This pipeline ultimately yielded 11 prophage regions in five (55.6%) *Methylococcus* strains.

In the final step, prophage boundaries were adjusted via pairwise alignment of each prophage sequence (extended by 20 kb on both sides) against an uninfected genome from a closely related bacterial strain via the ProgressiveMauve program [32]. Because no prophage sequences were detected in *M. capsulatus* MIR at earlier stages, this strain served as the uninfected reference. In cases where the prophage sequence disrupted bacterial genes or syntenic blocks of the host genome, the prophage boundaries were correspondingly expanded or contracted. The final set of prophage sequences obtained through this comprehensive search is listed in Table 1. Each genome contained between one and three prophage regions, ranging in length from 29.1 to 71.4 kb and encoding 33–90 genes. The strain *M. mesophilus* 16–5 presented the highest overall fraction of prophage DNA in its genome (3.82%), with three prophage regions identified.

**Table 1:** Predicted prophage regions

Species, Strain	Region name	Region position (length (kb))	Total protein/phage protein	CheckV	
				Completeness (%)	Quality
M. capsulatus, str. Bath	Bath-R1	2,818,869–2,875,600 (56.7)	57/17	76.28	Medium-quality
	Bath-R2	3,090,981–3,136,586 (45.6)	63/27	100.0	High-quality
M. capsulatus, str. KN2	KN2-R1	1,828,593–1,899,999 (71.4)	81/22	60.97	Medium-quality
	KN2-R2	2,309,080–2,345,124 (36.0)	45/16	60.91	Medium-quality
M. capsulatus, str. IO1	IO1-R1	2,502,234–2,549,530 (47.3)	65/26	100.0	High-quality
M. capsulatus, str. Mc (Nor)	McNor-R1	491,588–520,685 (29.1)	33/13	70.28	Medium-quality
	McNor-R2	1,493,609–1,539,485 (45.9)	58/21	73.14	Medium-quality
	McNor-R3	1,900,782–1,941,935 (41.2)	53/16	69.54	Medium-quality
M. mesophilus, str. 16–5	16–5-R1	1,897,271–1,964,996 (67.7)	90/20	100.0	High-quality
	16–5-R2	2,518,532–2,564,410 (45.9)	60/29	100.0	High-quality
	16–5-R3	3,229,289–3,281,743 (52.5)	68/18	100.0	High-quality
M. capsulatus, str. MIR	No prophage regions found				
M. geothermalis, str. IM1	No prophage regions found				
M. capsulatus, str. BH	No prophage regions found				
M. sp., str. Mc7	No prophage regions found				

### Genome‑based phylogeny of Methylococcus-infecting prophages

**Fig. 4:**
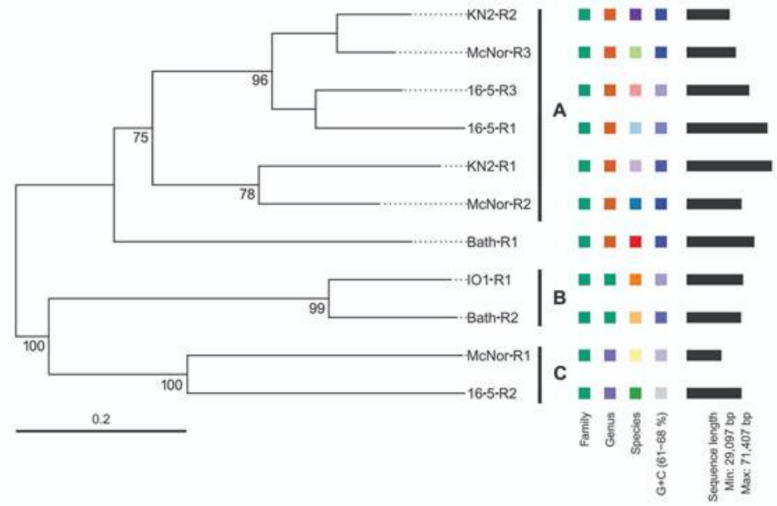
Phylogenetic tree constructed via the VICTOR service on the basis of 11 predicted prophage regions. The coloured squares on the right-hand side indicate, according to the VICTOR predictions, the family-, genus-, and species-level groupings, as well as the %G + C content and genome size. The phylogenetic distance and level of branch support delineate three clusters of closely related prophage regions (labelled A, B, and C, respectively)

A phylogenetic analysis was performed on the sequences obtained, employing the VICTOR suite of programs—a genome-based methodology for classifying and inferring the phylogeny of prokaryotic viruses. This approach yielded a phylogenetic tree with an average support value of 82% (see Fig. 4). Using OPTSIL clustering, eleven species-level groups were delineated, corresponding to the eleven prophage regions under study. At the genus level, three groups were identified, and at the family level, a single group emerged. On the basis of phylogenetic distance and branch support, three clusters of closely related prophage regions could be distinguished (labelled A, B, and C in Fig. 4):

Cluster A, the largest and most heterogeneous, comprises two pairs of regions—*KN2-R2* with *McNor-R3* and *16-5-R3* with *16-5-R1* (each pair demonstrating 96% support)—to which a closely related pair, *KN2-R1* and *McNor-R2*, is also added. Although this latter grouping has a 75% support value, it is sufficiently robust to be considered valid.

Cluster B, supported at the 99% level, unites the *IO1-R1* and *Bath-R2* regions and finally,

Cluster C, which is supported at 100%, comprises *16-5-R2* and *McNor-R1*.

**Fig. 5:**
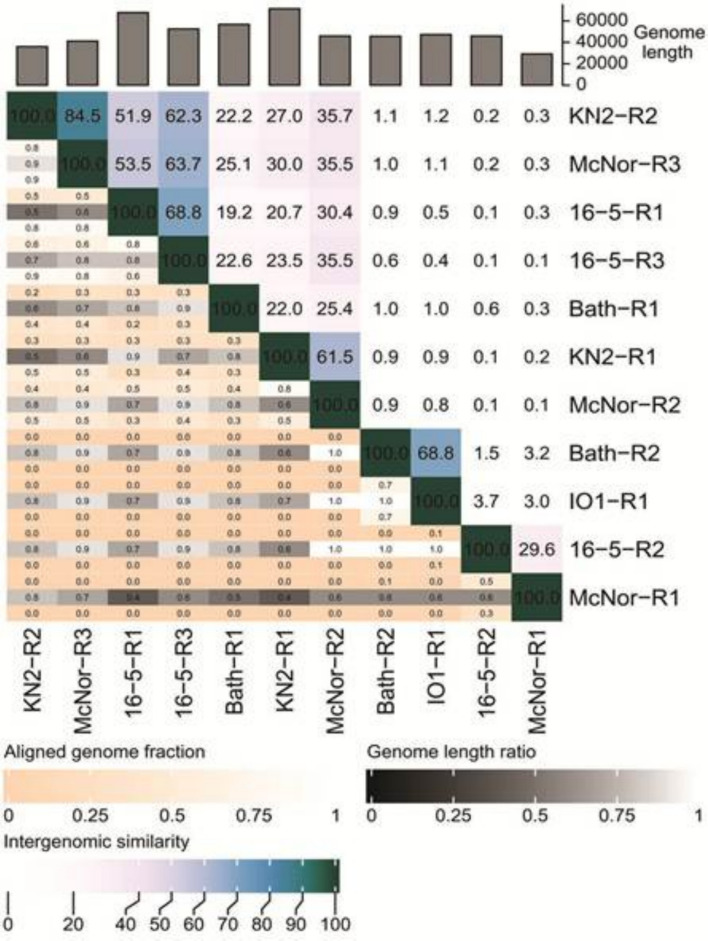
Intergenomic similarity analysis of 11 prophage sequences via VIRIDIC. The represented heatmap is divided into an upper-right section, which displays intergenomic similarity values, and a lower-left section, which shows alignment indicators. In the right half, both color saturation and numerical values reflect the degree of similarity between each pair of genomes. In the left half, three alignment indicators are listed for every pair of genomes, in top-down order: (i) the aligned fraction of the genome shown in the row, (ii) the ratio of the lengths of the two compared genomes, and (iii) the aligned fraction of the genome shown in the column, where darker colors correspond to lower values

To assess the intergenomic similarity among the eleven identified prophage regions, the bioinformatic tool VIRIDIC [33] was employed. This measure of intergenomic similarity serves as a standard used by the International Committee on Taxonomy of Viruses (ICTV) for classifying phages at the genus or species level [34]. The results (Fig. 5) closely mirror those obtained from the VICTOR-based phylogenetic analysis. In the upper-left portion of the heatmap, one can discern the region corresponding to phylogenetic Cluster A (Fig. 4). The highest degree of intergenomic similarity in this region was observed between KN2-R2 and.

McNor-R3 (84.5%), as well as between 16 and 5-R1 and 16-5-R3 (68.8%); both pairs were grouped together in the phylogenetic tree and assigned to the same genus-level cluster. The regions KN2-R1 and McNor-R2, which display 61.5% similarity, also lie in this section of the heatmap. Phylogenetic cluster B, encompassing IO1-R1 and Bath-R2 at 68.8% intergenomic similarity, is represented by a relatively isolated area in the lower-right portion of the heatmap (Fig. 5). Conversely, although 16-5-R2 and McNor-R1 were grouped in cluster C and assigned by VICTOR to the same genus, their mutual similarity was notably low, at just 29.6%.

### Proteome‑based classification of Methylococcus strains infecting prophages

**Fig. 6:**
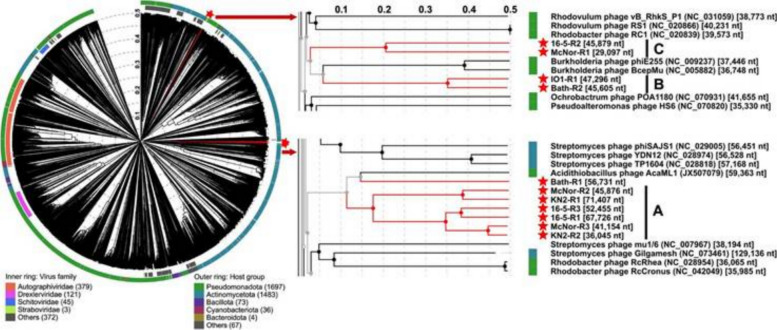
ViPTree proteome tree of closely related dsDNA bacteriophages. The analysed prophage regions, highlighted in red, are grouped into three clusters. Labels A, B, and C correspond to the phylogenetic clusters identified in the genome-based phylogenetic analysis (see Fig. 4)

For proteomic classification of the identified prophages, the global proteome-tree construction tool ViPTree [35] was used. This method integrates the analysed bacteriophage genomes alongside various reference viral genomes into a single dendrogram, thereby clarifying the phylogenetic relationships both among the newly characterized prophage regions and with previously described phages. The resulting tree is shown in Fig. 6, where all detected regions are grouped into three clusters, mirroring the structure obtained via the VICTOR algorithm.

Cluster A features three pairs of grouped regions—*16-5-R3* with *16-5-R1*, *KN2-R2* with *McNor-R3*, and *KN2-R1* with *McNor-R2*—indicating a shared evolutionary origin and suggesting that they belong to the same genus. Notably, *Bath-R1* also resides near this cluster but maintains a substantial distance from the remaining sequences. Among the reference phages in this group, the closest is *Acidithiobacillus phage AcaML1* (JX507079), which is assigned to the class *Caudoviricetes* with a head–tail morphology.

Clusters B and C each encompass two paired prophage regions: *IO1-R1* with *Bath-R2* and *16-5-R2* with *McNor-R1*. Although pairwise groupings within these clusters are strongly supported, the distance between them precludes combining all four regions into a single genus. The most closely related phages described for these clusters—*Burkholderia phage phiE255* (NC009237) and *Burkholderia cenocepacia phage BcepMu* (NC005882)—belong to the genus *Bcepmuvirus* within the class *Caudoviricetes*.

### Comparative genomic analysis of Methylococcus prophages

Ten prophage sequences that formed three distinct clusters in the genome- and proteome-based analyses were examined in greater depth to assess the presence of functional genes, their synteny, and overall similarity. Annotation of these sequences was performed via Pharokka v1.5.0 [36], while the online bacteriophage database PhageScope [37] was employed for additional taxonomic and functional annotation. Comparative alignments and intergenomic comparison diagrams were generated with Clinker (v. 0.0.31) [38]. On the basis of the presence of specific tail-encoding genes, all examined sequences were classified as temperate bacteriophages belonging to the class *Caudoviricetes*.

**Fig. 7:**
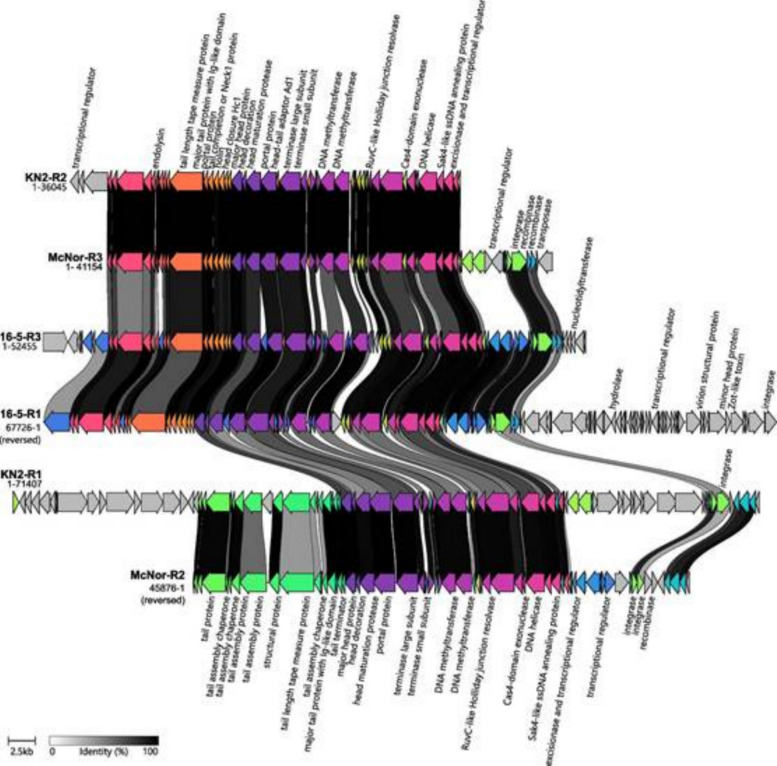
Comparative alignment of prophage sequences from Cluster A. The sequences are presented alongside their assigned name and genome length. Protein genes are indicated by arrows, which are coloured to reflect the homologous groups identified via Clinker. The lines connecting the arrows indicate the percentage amino acid identity, as shown in the legend (greater than 40%)

Cluster A (Fig. 7)—the largest and most heterogeneous group—consists of six prophage sequences paired into three subclusters (KN2-R2 with McNor-R3, 16-5-R1 with 16-5-R3, and KN2-R1 with McNor-R2). Their lengths range from 36.0 to 71.4 kb, encoding 45–81 proteins, of which 16–22 are annotated as proviral. Each prophage genome contains a *tail length tape measure protein* but lacks a *tail sheath protein*, which is indicative of siphoviral morphology. All six members of Cluster A displayed substantial gene synteny, as illustrated in the figure. Five of these genomes harbor an *integrase* at a characteristic locus, which is consistent with temperate phages. Beyond that region, the sequences exhibit a relatively uniform arrangement of genes involved in metabolic regulation, nucleic acid replication, packaging (*terminase*), and core structural proteins (including *portal*, *head*, *neck*, and *tail* proteins). Moreover, *endolysin*-related proteins were identified, suggesting the potential for a lytic developmental cycle. Quality assessments with CheckV indicated 100% completeness and high-quality designations for only two sequences, 16-5-R1 and 16-5-R3, whereas the remaining prophage genomes received medium-quality ratings, with completeness ranging between 61 and 73%.

**Fig. 8:**
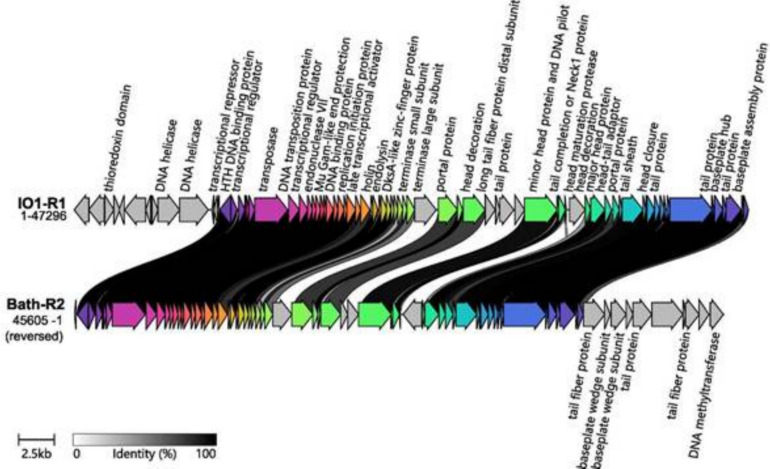
Comparative alignment of prophage sequences from Cluster B. The sequences are presented alongside their assigned name and genome length. Protein genes are indicated by arrows, which are coloured to reflect the homologous groups identified via Clinker. The lines connecting the arrows indicate the percentage amino acid identity, as shown in the legend (greater than 40%)

Cluster B (Fig. 8) comprises two prophage sequences, Bath-R2 and IO1-R1, measuring 45.5 and 47.3 kb, respectively. Each sequence encodes 63 or 65 proteins, 27 and 26 of which, respectively, are definitively phage related. Both prophages carry a *tail sheath protein*, indicating a myoviral morphology. According to CheckV, these sequences exhibit 100% completeness and high-quality status. Their annotated genes show marked synteny, and both genomes include all key elements required for the lytic cycle: *transposase* (serving as an integrase), genes for metabolic regulation and nucleic acid replication, packaging (*terminase*) genes, and structural genes (including *portal*, *head*, *neck*, *tail*, and *baseplate* proteins).

**Fig. 9:**
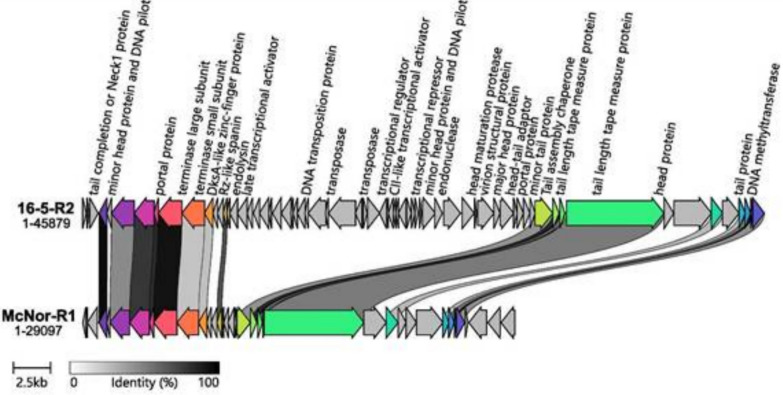
Comparative alignment of prophage sequences from Cluster C. The sequences are presented alongside their assigned name and genome length. Protein genes are indicated by arrows, which are coloured to reflect the homologous groups identified via Clinker. The lines connecting the arrows indicate the percentage amino acid identity, as shown in the legend (greater than 40%)

Cluster C (Fig. 9) consists of two prophage sequences—16-5-R2 and McNor-R1—whose lengths differ considerably, at 45.9 kb and 29.1 kb. The disparity in genome size is reflected in the number of protein-coding genes (60 versus 33, of which 29 and 13, respectively, are putative viral genes). Both sequences encode a *tail length tape measure protein* but lack a *tail sheath protein*, which is consistent with siphoviral morphology. Comparative alignment of the annotated genes suggested that, unlike 16-5-R2, the McNor-R1 genome has lost approximately half of its central region, including *transposase* (integrase) genes, several metabolic and nucleic acid replication genes, and certain structural components. This substantial deletion casts doubt on McNor-R1’s ability to complete a lytic cycle. CheckV categorizes McNor-R1 as having 70% completeness and medium quality—an assessment that may be somewhat generous, given the extensive genetic deletions. In contrast, 16-5-R2 retains all major gene blocks essential for lysogenic phage reproduction. These include a *transposase*, syntenic *large* and *small terminase* subunits, a *portal* protein, genes encoding structural proteins (such as *head*, *neck*, and *tail*), and various nucleic acid metabolism genes, along with a dedicated lysis module (Rz-like spanin and *endolysin*). CheckV rates 16-5-R2 at 100% completeness and high-quality status.

### CRISPR‑Cas and spacer prediction (phage enumeration and threat analysis)

To investigate the interaction history between *Methylococcus* species and various bacteriophages, the CRISPR‒Cas system was analysed for potential phage targets. CRISPRCasFinder [39] was used to identify *cas* genes, direct repeats, and spacers in CRISPR arrays. The analysis revealed the presence of CRISPR‒Cas in all the genomes except for 16-5. Among all the strains of *M. capsulatus*, two systems—classified according to [40] as Type I–E and Type I–C—were identified, each of which was distinguished by a specific set of *Cas* proteins. The *IM1* and *Mc7* strains had a greater number of arrays, with an additional Type I–F system present. The repeat consensus sequences within each system were conserved across strains (see the Table S4 Supplementary Materials 1), suggesting a monophyletic origin of the CRISPR arrays. The *IM1* strain was also found to harbor a Type III-A system, whereas the *Mc7* genome contains two CRISPR arrays with identical direct repeats but no adjacent *cas* genes, indicating a loss of Type III-A *cas* proteins. Among the genomes that do encode CRISPR‒Cas systems, the number of spacers varies from 69 in strain *Bath* to 197 in strain *IM1* [see Table S3 in Supplementary Materials 1], with average array lengths of 50 spacers for Types I–E and 46 for Types I–C. array sizes also vary substantially between strains. Analysis of the similarity of the spacer sequences revealed few identical sequences. Notably, the Type I-E arrays of strains *BH*, *MIR*, and *IO1* share the highest number of identical spacers: 58 are common to all three strains, and 24 are shared by at least two of them. Moreover, four identical spacers are shared between ‘KN2_TypeIE’ and ‘McNor_TypeIE’, and 21 are shared between ‘Bath_TypeIC’ and ‘MIR_TypeIC’. Overall, these findings indicate that the genomes of *Methylococcus* species harbor multiple CRISPR‒Cas defense systems, most of which feature predominantly unique spacer sequences.

**Fig. 10:**
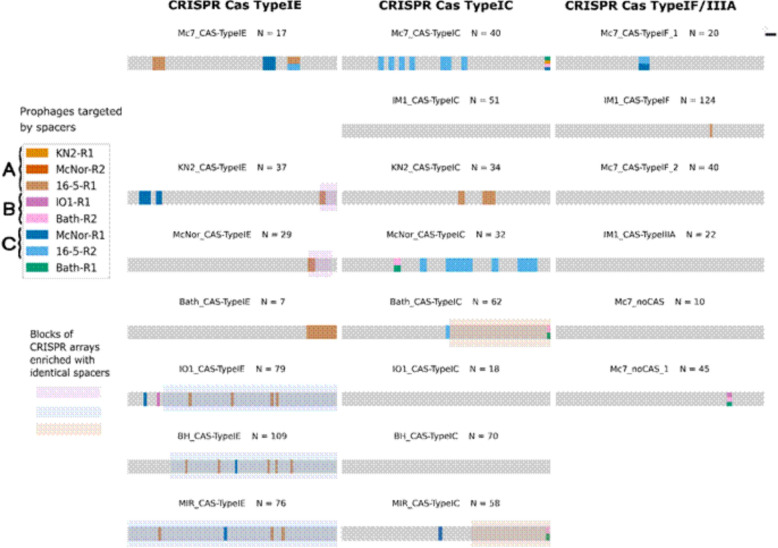
CRISPR arrays identified in *Methylococcus* strains, grouped according to the types of adjacent Cas proteins. The rows of the first two columns replicate the topology of the species-level phylogenetic tree. Spacers targeting predicted prophage sequences are highlighted in color, with N denoting the total number of spacers in each array. Vertical divisions within a spacer cell indicate that the spacer matches more than one prophage sequence. The semitransparent boxes highlight blocks containing predominantly identical spacers. The spacers in each array are oriented from left to right relative to the predicted leader sequence such that those at the left edge are considered the most recently acquired

To ascertain which prophages might be targeted by the spacers in *Methylococcus* strains, the SpacePHARER program [41] was used to compare all the spacers against the set of predicted prophages. In the majority of the CRISPR arrays, spacers that matched one or more of the predicted prophages were identified (see Fig. 10). In most instances, the spacers targeted prophages located in genomes other than those of the host strain. An exception was observed in *Bath*, which contained a single spacer directed against its own prophage; however, the spacer-prophage alignment did not exhibit 100% identity, thus preventing self-targeting. The most frequently targeted prophages were 16-5-R1, McNor-R1, and 16-5-R2 (see Fig. 10), which appeared in 8, 6, and 4 genomes, respectively, and belong—according to Sects. “Prophage identification in bacterial genomes” and “Prophage annotation and morphological classification”—to clusters A and C. Because CRISPR arrays are polarized (new spacers are added near the leader sequence) and spacer orientation is predicted by the CRISPR‒Cas software, it is possible to gauge the relative recency of spacer acquisition. As shown in Fig. 10, spacers against 16-5-R1 are typically situated closer to the distal end of the array, whereas those against McNor-R1 can be found nearer the leader sequence. For strains *IO1* and *KN2*, where the array orientation is further corroborated by shared spacer blocks with other strains—reflecting events in an ancestral strain—this positioning suggests a recent encounter with a phage related to McNor-R1. Overall, 57 spacers (5.4% of the total) matched one of the predicted prophages. In order to explore the biological significance of these matches, the functional annotations of the targeted prophage genes were examined (see Supplementary Table 3). Of the 57 identified spacer–phage matches, 29 spacers mapped to unannotated proteins, whereas the remaining spacers targeted recognizable functional categories, predominantly tail (13) and head/packaging (8) proteins—key determinants of host recognition and virion assembly. Notably, all spacers targeting the *McNor-R1* prophage mapped to genes encoding tail proteins, while *16-5-R2* was primarily targeted in its head and packaging region, suggesting selective pressure on structural modules essential for infection. All 18 spacers targeting the *16-5-R1* prophage corresponded to hypothetical proteins. The loci of these mappings are unique to this phage compared with other members of Cluster A (Fig. 7), which may highlight the functional importance of these unannotated genes and warrants their further characterization. In contrast, spacers positioned closest to the leader sequences in the Type I-E arrays of strains KN2 and IO1 matched *McNor-R1* tail protein genes. The placement of these spacers near the leader regions—where newly acquired spacers are added—suggests recent encounters with this phage lineage. Such patterns imply ongoing CRISPR-mediated surveillance and adaptation against recurrent phage infections in methane-oxidizing consortia.

Finally, to determine potential targets for the remaining spacers, these were compared with prophage genomic sequences in the PhageScope database [37], which contains 873,718 prophage entries. Only one spacer—from the *Mc7* genome—produced a match, found in the GOV2 database entry for a phage designated Station125_MXL_ALL_assembly_NODE_1449_length_19610_cov_33.798006, belonging to the order *Caudovirales* and detected in the genome of *Pseudomonas aeruginosa*. Hence, the majority of spacers have yet to be assigned to a target. These results indicate that while *Methylococcus* genomes possess numerous spacers in their CRISPR‒Cas arrays, only a small fraction can currently be matched to known bacteriophages. The genus has likely encountered a multitude of yet-undescribed phages, making this extensive spacer repertoire an invaluable resource for future metagenomic studies aimed at assessing the risk of *Methylococcus* infection in open-system industrial consortia.

### Identification of potential disruptions to metabolic genes

An analysis of prophage integration sites in the bacterial genome was performed to assess potential disruptions to metabolic genes. Only prophages with clearly defined boundaries, as determined by the alignments described—namely, Bath-R1, Bath-R2, KN2-R1, IO1-R1, McNor-R1, and McNor-R2—were included in this study. The genes flanking each prophage insertion were compared with their homologous counterparts in the prophage-free *MIR* strain. If a gene was disrupted by prophage integration, its annotation would be missing in the corresponding region of the prophage-bearing strain; however, no such cases were detected. For annotated genes, gene length was also compared to that of the homologue in strain *MIR*. The analysis revealed that prophage integrations did not compromise the integrity of metabolic genes; in all instances, prophages were inserted between bacterial genes. Many of the genes flanking the prophage insertion sites were annotated as hypothetical proteins. Nevertheless, three RNA-coding genes in the *Methylococcus capsulatus* genome were found in close proximity to functionally intact prophage insertions (Table 2).

**Table 2:** List of genes at (or near) which phage integrations were detected

Species, strain	Region name	Genes neighboring to prophage	Comments
M. capsulatus, str. Bath	Bath-R1	ssrA (transfer-messenger RNA, SsrA)	The length of the ssrA gene is preserved, the gene is located close to the left border of Bath-R1, there are no duplicates of the ssrA gene
	Bath-R2	tRNA-Thr	The length of the tRNA-Thr gene is preserved, the gene is located close to the left border of Bath-R2, there are three duplicates of the tRNA-Thr gene
M. capsulatus, str. KN2	KN2-R1	tRNA-Phe	The length of the tRNA-Phe gene is preserved, the gene is located close to the right border of KN2-R1, there are no duplicates of the tRNA-Phe gene

These findings indicate that prophage integration is unlikely to impair bacterial metabolic activity, as no disruption of metabolic genes was observed. Bacterial genes can be duplicated and may acquire new functions over the course of evolution, enabling adaptation to diverse conditions. Therefore, if random prophage integration affects one copy of a duplicated bacterial gene, the remaining copies remain active, preserving metabolic functionality. Consequently, a search was conducted for duplicated genes flanking the prophage insertion sites. Gene duplication was inferred on the basis of bacterial gene annotations in the respective genomes. This analysis identified three duplicated *tRNA-Thr* loci in *Methylococcus capsulatus Bath* (Table 2). No other duplicated genes were found in the remaining annotated genomic regions.

## Discussion

Identifying prophage sequences in sequenced bacterial genomes poses considerable technical challenges. To date, no uniform criteria exist for detecting or determining the functional status of prophages. They may appear in the bacterial genome in various forms, ranging from unmodified, inducible sequences to nonviable fragments that have arisen through deletions, insertions, or genetic rearrangements. Moreover, the number of prophage genes that remain sufficiently conserved and reliably distinguishable from bacterial genes—thus serving as clear prophage signatures—is quite limited [12, 13].

According to the proteome-based phylogenetic tree constructed via ViPTree, the prophage sequences identified in this study were most similar to those of previously described bacteriophages: Acidithiobacillus phage AcaML1 (JX507079), Burkholderia phage phiE255 (NC009237), and Burkholderia cenocepacia phage BcepMu (NC005882). The genome sizes of these reference phages, namely, 59.4 kb, 37.4 kb, and 36.7 kb, are comparable to those of most of the prophages identified in our study. Comparative genomic alignment revealed conserved synteny maps among the prophage genomic regions within all three described clusters. The observed genomic features of the identified prophages suggest that several of them may retain the ability to produce infectious particles.

As fully characterized bacteriophage genomes of no single genus are available in current databases, it was not possible to directly compare gene composition and synteny with those of viable bacteriophages. However, the presence and proper arrangement of all essential gene blocks required for the initiation and progression of the lytic reproductive cycle allow indirect inference of prophage inducibility. The essential gene sets identified included integrases located at the beginning of the prophage sequence; structural genes encoding head, portal, neck, and tail proteins; conserved synteny of large and small terminase subunits along with portal proteins; lysis-related genes, including endolysin and/or Rz-like spanin; and genes involved in nucleic acid metabolism, such as polymerases, helicases, and ligases. The comparative alignment of prophage sequences confirmed the presence of these essential gene blocks in the following sequences: Cluster A (McNor-R2, McNor-R3, KN2-R1, 16-5-R1, 16-5-R3), Cluster B (IO1-R1, Bath-R2), and Cluster C (16-5-R2).

The Bath-R1 sequence deserves special attention. According to genomic and proteomic analyses, this sequence is closely associated with Cluster A but maintains significant phylogenetic distance. Gene annotation indicated that Bath-R1 possesses siphoviral morphology and includes all essential genetic elements necessary for the induction of a lytic reproductive cycle (annotated sequences provided in the Supplementary materials 2). Conversely, comparative genomic analysis indicated that the KN2-R2 sequence, classified within Cluster A, has lost the right-side genomic region, including regulatory genes and integrase, thus casting doubt on its capacity for full prophage induction. Likewise, the comparative alignment of annotated genes in Cluster C revealed that the McNor-R1 sequence, in contrast to 16-5-R2, has experienced a deletion of approximately 50% of its central genome, encompassing integrative genes (transposases), certain genes involved in nucleic acid metabolism and regulation, and structural genes. This finding strongly questions McNor-R1’s potential to execute a complete lytic reproductive cycle. Therefore, we conclude that there are no objective grounds to exclude the inducibility potential in 9 out of 11 prophage sequences identified in this study. These results are consistent with previous findings obtained during the characterization of strains Bath, IO1, and KN2 [2, 17].

Analysis of the CRISPR‒Cas system revealed a markedly high average number of spacers in *Methylococcus* strains—122—compared with the reported averages of 9.36 and 37.93 spacers per genome in *Streptococcus mutans* [19]. Likewise, the CAS-Type I-E arrays in *Methylococcus* strains comprised 7–109 spacers (average of 50), in contrast to 2–32 spacers (average of 11) for the same array type across 1048 *E. coli* isolates [42]. These numbers are also greater than the average 40 Type I CRISPR spacers noted in a survey of 811 bacterial genomes, yet they fall within the standard deviation, indicating that they are not extreme outliers [43]. According to the model proposed by Martynov and colleagues [44], the optimal number of spacers in a bacterial genome increases with viral diversity, although an overabundance of spacers within an arraymay reduces overall CRISPR efficiency. Similarly, Bradde [45] demonstrated that the number of spacers must balance phage diversity for optimal CRISPR system performance, although increasing the number of spacers by adding more CRISPR arrays can maintain system efficacy. Hence, the variability observed in CRISPR array numbers and lengths among *Methylococcus* strains likely reflects differences in phage burden, with strains *Bath* and *Mc (Nor)* appearing to harbor lower phage diversity, whereas *IM1*, *Mc7*, and *BH* exhibit a broader phage range. The relatively low number of spacers in the Bath strain aligns with its long-term laboratory cultivation—at least 15 years prior to its sequencing [46, 47]. In contrast, most other strains were isolated from natural environments, supporting the hypothesis that a high phage load—and a correspondingly large number of spacers—may be typical for *Methylococcus* in situ. However, the relatively low spacer counts observed in the environmentally derived Mc (Nor) and KN2 strains indicate that these cases may not fully align with the general trend. This could reflect a lower local phage burden in their respective environments or suggest that additional factors—such as host-specific dynamics or evolutionary constraints—may also contribute to shaping the composition of the CRISPR array.

Because self-targeting spacers are known to induce severe toxicity in bacterial cells, resulting in morphological alterations, growth suppression, and genomic instability [48], such events tend to be purged over evolutionary time. The sole genome analysed here that lacks a CRISPR‒Cas system is strain 16–5, which contains three prophage sequences, two of which are still targeted by spacers found in other members of this genus. Given that self-targeting can favour genetic deletions and destabilize CRISPR‒Cas loci, it is plausible that this strain lost its CRISPR system as a consequence of prophage invasion. None of the other strains in the genus exhibit self-targeting spacers, although *Bath* has a spacer whose sequence deviates slightly from self-complementarity. In an industrial context, such autoimmunity would be highly undesirable, rendering the absence of self-targeting spacers a beneficial attribute for strains employed in large-scale processes.

An examination of the spacer repertoire against the predicted prophage genomes revealed clear evidence of immune defences against 16-5-R1, 16-5-R2, and McNor-R1, with each strain bearing protection against at least one of these phages. Because of the polarized architecture of CRISPR arrays—where new spacers are added adjacent to the leader sequence—it is possible to distinguish earlier from more recent infection events. Admittedly, the orientation of each array cannot be definitively confirmed without additional evidence; thus, only those arrays whose orientation was corroborated by conserved spacer blocks shared with other strains were utilized for assessing infection chronology. For example, shared spacer blocks inferred to originate from a common ancestor were expected on the distal side from the leader, allowing us to correct the orientation of the MIR_CAS-TypeIC array. On the basis of the proximity of these spacers to the leader sequences—using array orientation supported by shared spacer blocks—strains *KN2* and *IO1* appear to have engaged in relatively recent encounters with a phage related to McNor-R1—as indicated by three spacers in *KN2* that target the “tail length tape measure protein” and one spacer in *IO1* with the same annotation but different sequences. Given that these events likely occurred independently within strains of similar origin, namely, activated sludge in Moscow, a bacteriophage identical to or closely related to McNor-R1 represents a common threat in this environment. The distribution of spacer targets provides insight into selective pressures shaping CRISPR–phage interactions in *Methylococcus*, but it also warrants caution in interpretation because a large fraction of matches map to unannotated loci. Of the 57 spacer–prophage matches, many (≈50%) target hypothetical proteins, although the remainder map to identifiable functional categories—notably tail and head/packaging components. The latter targets, including the exclusive association of *McNor-R1* spacers with tail-associated genes and the clustering of *16-5-R2* spacers in head/packaging modules, are consistent with CRISPR systems preferentially targeting infection-critical regions (e.g., adsorption and assembly). At the same time, the prevalence of hits to unannotated regions (particularly for *16-5-R1*) suggests the presence of novel or rapidly evolving phage loci whose functions remain to be determined. Overall, these patterns indicate that CRISPR–Cas activity in *Methylococcus* reflects both targeted responses against known structural modules and unresolved interactions with uncharacterized phage genes, underlining the need for functional follow-up of the hypothetical targets to fully understand host–phage co-evolution.

Despite having conducted a thorough search for CRISPR–Cas targets—both among the *Methylococcus* prophages and in databases containing over 800,000 phage sequences—potential targets were identified for only a small fraction (5.5%) of the spacers. The remaining spacers may target other mobile genetic elements, such as plasmids, or potentially undiscovered phages. According to the literature, the relative contributions of phages versus plasmids to spacer origin can vary substantially, sometimes favouring plasmids (by several orders of magnitude) or, conversely, phages (see, for example, [49]). Thus, the ~ 5% match rate observed here implies that the prophages characterized in this work represent only a subset of the infection events recorded in the CRISPR arrays, highlighting the existence of additional, yet-undescribed phage and plasmid threats. Further metagenomic analyses, comparing identified spacers to the phages and plasmids present in a given community, could clarify this ratio and potentially uncover additional phages that may threaten industrial processes reliant on *Methylococcus* species.

Depending on the molecular mechanism of integration, prophages may be incorporated into the bacterial genome either at specific sites (site-specific integration) or randomly (random integration); the outcome largely depends on both the phage type and the bacterial host. In the former scenario, specialized “attachment sites” (att-sites) enable a temperate phage integrase to recognize and recombine with a homologous sequence in the bacterial genome. For example, the *Escherichia coli* λ‑phage integrates at attB (between the gal and bio genes), whereas phage P22 in *Salmonella* inserts at a tRNA-Leu locus, and phage φ80 similarly employs a specific tRNA site. Such targeted integrations provide stable maintenance within the host cell and support an effective lysogenic cycle. In contrast, transposed phages typically lack specific att-sites and randomly insert themselves throughout the genome, often relying on nonspecific recombinases or DNA repair systems. For example, phage Mu behaves like a transposon, inserting at random locations in the bacterial genome; some phages utilize nonspecific IS elements (insertion sequences) for integration.

This raises the questions of where precisely the identified prophages have inserted and whether their integration disrupts metabolic genes. We therefore examined the genomic regions flanking the prophage boundaries (left and right) for which alignments allowed clear boundary definitions—namely, *Bath-R1*, *Bath-R2*, *KN2-R1*, *IO1-R1*, *McNor-R1*, and *McNor-R2*. Our analysis revealed that most prophage insertions occurred between bacterial genes, many of which are annotated as hypothetical proteins. Nonetheless, three RNA-coding genes in the *Methylococcus capsulatus* genome—SsrA, tRNA-Thr, and tRNA-Phe—were found in close proximity to prophage insertions.

The bacterial genes flanking each prophage boundary were compared against homologous loci in the prophage‑free *Methylococcus capsulatus* MIR strain to assess gene annotations and gene length. This analysis revealed no instances of random disruption of bacterial metabolic genes—that is, there was no loss of integrity in any flanking genes. Furthermore, the identified attachment sites (*att-sites*) for prophage insertions Bath-R1, Bath-R2, and KN2-R1 (Table 2), located near the *SsrA*, *tRNA-Thr*, and *tRNA-Phe* genes, align with the well‑documented preference of prophages for tRNA‑associated regions. Therefore, *tRNA-Thr* can be considered an integration target because (i) it is both conserved and widespread among bacteria; (ii) prophage integrase systems readily recognize it; (iii) it avoids disruption of essential genes, thereby stabilizing the lysogenic state; (iv) it is present in many bacteria, often in multiple copies; and (v) it participates in stress responses, potentially triggering a prophage’s transition to the lytic cycle under adverse conditions.

From these results, it can be inferred that the identified prophage insertions (Table 1) involved site-specific integration and did not compromise any bacterial metabolic genes (Table 2). Consequently, no metabolic impairment would be expected to manifest as a phenotypic change in the bacterium. The observed metabolic differences among methanotrophic strains may therefore be caused by factors other than prophage integration.

Furthermore, it is necessary to note that identified prophage insertions—Bath-R2, Bath-R1, and KN2-R1—are potentially functionally active under particular conditions. On the other hand, their integration near genes (*SsrA*, *tRNA-Thr*, and *tRNA-Phe*) that are involved in the regulation of metabolic activity and response to various stressors stabilizes their maintenance within the host cell and promotes an effective lysogenic cycle under appropriate stressful conditions. Below is a brief overview of the functional activity associated with these genes (*SsrA*, *tRNA-Thr*, and *tRNA-Phe*), highlighting their regulatory mechanisms and potential to trigger prophage induction under various stress conditions:

- The SsrA gene encodes *tmRNA* (also known as 10Sa RNA or SsrA RNA), which performs a dual role akin to both tRNA and mRNA. It participates in protein quality control by rescuing stalled ribosomes when the mRNA is truncated (lacking a stop codon) and by adding a small peptide tag (SsrA tag) to any incomplete protein, marking it for proteolytic degradation (ClpXP, Lon). *SsrA* also influences gene expression and stress responses (e.g., antibiotic resistance, heat shock, and mechanical damage). This explains, in part, why prophages may switch to a lytic cycle (phage‑induced lysis) under stressful conditions. Thus, *SsrA* is involved in ribosome rescue during stalled translation events and in tagging incomplete proteins for degradation, thus linking translational quality control to stress responses.
- The tRNA-Thr gene encodes a transfer RNA that delivers threonine to the ribosome during protein synthesis. Certain bacteria incorporate *tRNA-Thr*-associated elements into regulatory mechanisms (such as antitermination structures in leader sequences), which may also contribute to stress adaptation by modulating threonine availability. *tRNA-Thr* is often a hotspot for prophage and transposon integrations. Different variants of *tRNA-Thr* exist across species because of modifications in the coding sequences of tRNA-modification systems. *tRNA-Thr* may be upregulated under numerous stressors (*e.g.,* amino acid starvation; exposure to antibiotics; oxidative stress; heat-shock stress; pH stress; and exposure to heavy metals), possibly influencing prophage induction when it is overexpressed.
- The tRNA-Phe gene encodes a transfer RNA responsible for supplying phenylalanine to the ribosome. Some bacteria deploy *tRNA-Phe* in attenuation mechanisms, such as regulating phenylalanine synthetase genes in response to intracellular phenylalanine levels. Like other tRNAs, *tRNA-Phe* can be crucial for stress adaptation, and it frequently serves as an integration site for prophages, transposons, or pathogenicity islands (as observed in certain *Escherichia coli* and *Salmonella* strains). Different species may carry modified *tRNA-Phe* variants that increase fitness under diverse environmental conditions. *tRNA-Phe* can also participate in attenuation mechanisms and respond to amino acid limitations, which may indirectly affect prophage dynamics.

The presence of prophages integrated near stress-response genes suggests that various stress conditions could potentially induce these prophages to enter the lytic cycle (a hypothesis that remains untested in our study).

Various environmental or mechanical stress conditions could trigger prophage induction and disrupt the cultivation process; however, we present this as a potential scenario rather than a confirmed outcome.

Given the inherent difficulty in tracing such disruptions to their exact triggers retrospectively, proactive elimination or careful mitigation of stress-inducing factors remains critical. Where these measures are impractical or insufficient, it may become necessary to select alternative *Methylococcus* strains devoid of such prophage vulnerabilities.

Addressing these multifaceted challenges requires the integration of advanced computational methods that can anticipate and prevent prophage induction. Computational fluid dynamics (CFD), coupled with artificial intelligence (AI), computer vision (CV), machine learning (ML), and other emerging technologies, provides powerful tools for modelling and optimizing bioreactor conditions. By simulating flow dynamics, temperature distributions, pressure changes, and mixing patterns within industrial-scale bioreactors, researchers can pinpoint and mitigate conditions likely to trigger prophage activation. These sophisticated tools facilitate the design of more robust reactors, refine the selection of mechanical components to minimize shear stress or cavitation risk, and establish responsive control strategies. Such multidisciplinary integration of computational modelling with genomic and biological insights could enhance the stability, efficiency, and economic viability of methane-based bioprocesses, providing a path to optimize production for industrial stakeholders once these approaches are tested and refined.

Future research efforts should continue investigating prophages infecting *Methylococcus* species, with clear benefits for both fundamental microbiology and applied industrial biotechnology. Expanded metagenomic surveys across diverse methanotrophic microbial communities, including those in large-scale industrial bioreactors, promise to identify novel prophage varieties and reveal previously unrecognized pathways of prophage–host interactions. The integration of multiomics approaches (including transcriptomics, proteomics, and metabolomics) with advanced computational modelling techniques, such as CFD and AI-driven analytics, will substantially advance our understanding of specific stress conditions and genetic triggers that prompt prophage induction. Additionally, exploiting prophages as vectors for targeted genetic engineering offers the potential to increase the metabolic capabilities of *Methylococcus* strains. This approach could foster the development of novel, robust strains optimized for increased productivity, improved stress resistance, and increased metabolic versatility, thus contributing significantly to more resilient and sustainable methane-based biotechnological solutions.

## Conclusions

**Fig. 11:**
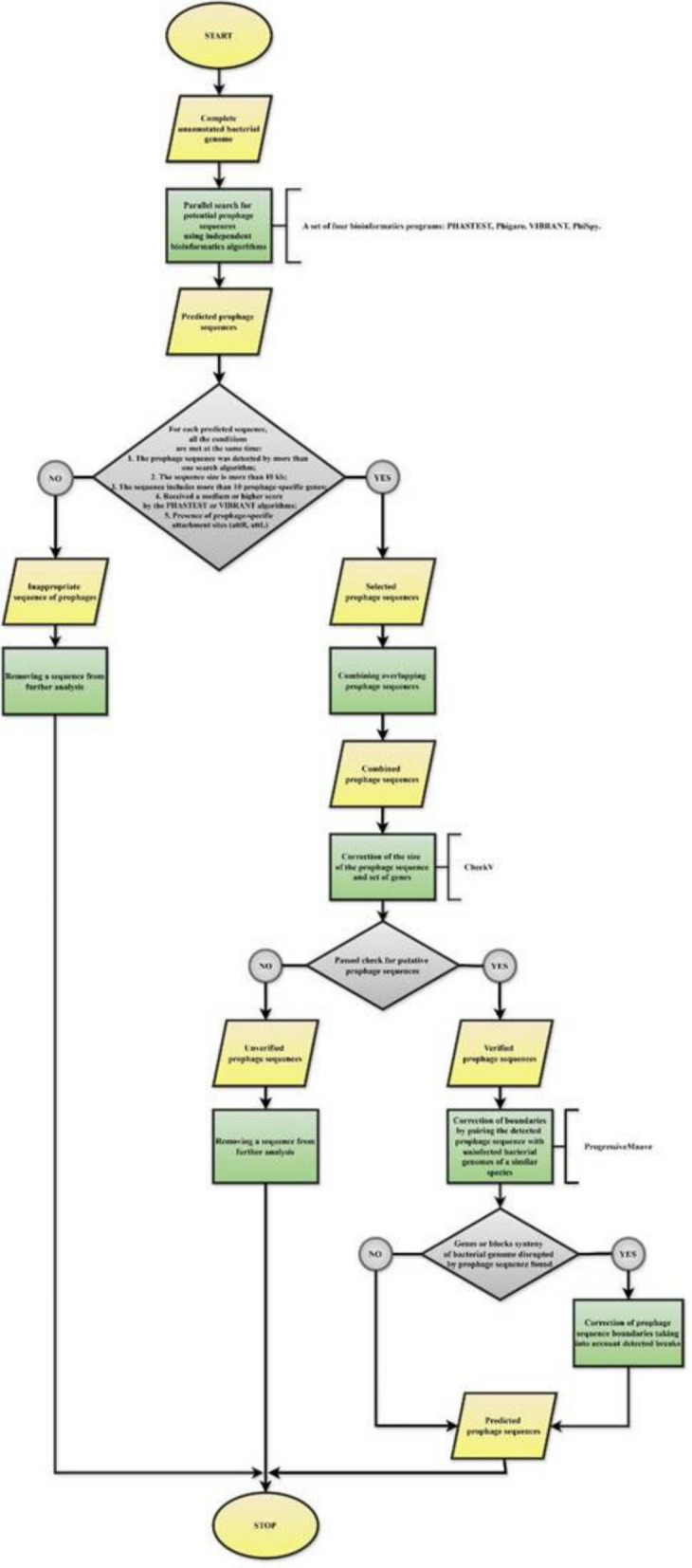
Block diagram of the prophage identification algorithm

This study developed a robust, multistage pipeline to detect, classify, and analyse prophages and associated CRISPR‒Cas immunity in nine publicly available genomes of Methylococcus (see Fig. 11). Eleven prophages were identified, nine of which appear potentially functional (i.e., predicted inducible based on genomic features). We note that inducibility was not experimentally confirmed in this study, so this finding serves as a testable hypothesis rather than a definitive conclusion. CRISPR‒Cas analyses provided evidence of prior exposure and adaptive immune responses to these prophages. Particularly significant is the discovery of prophages adjacent to stress-sensitive genomic loci (e.g., tRNA-Thr), suggesting that environmental and mechanical stressors in bioreactors may trigger prophage induction, increasing cultivation stability. Our findings underscore the need for routine prophage monitoring in industrial methanotrophic consortia, with the pipeline established here serving as a foundational framework for future refinement and industrial adaptation. Integrating advanced metagenomics with computational modelling approaches will further elucidate prophage–host dynamics, increase bioreactor design, and help prevent phage-mediated cultivation failures, although such benefits remain to be demonstrated on various scales. Ultimately, a deeper understanding and proactive management of prophages can convert these genetic elements from risks into valuable tools, bolstering both the ecological and economic potential of methane bioconversion processes.

## Supplementary Information

Supplementary Material 1.

Supplementary Material 2.

Supplementary Material 3.

## Data Availability

The original contributions presented in this study are included in the article/Supplementary Material. Further inquiries can be directed to the corresponding authors.

## Abbreviations

NCBI: National Centre for Biotechnology Information
DNA: Deoxyribonucleic acid
dsDNA: Double-stranded deoxyribonucleic acid
PHASTEST: PHAge Search Tool with Enhanced Sequence Translation
VICTOR: Viral comparison and tree-building online resources
VIRIDIC: Virus Intergenomic Distance Calculator
CRISPR–Cas: Clustered regularly interspersed palindromic repeats
ICTV: International Committee on Taxonomy of Viruses
BLAST: Basic Local Alignment Search Tool
OPTSIL: Clustering by Optimizing Silhouette Widths
CDS: Coding sequence
GBDP: Genome BLAST distance phylogeny
pVOGs: Prokaryotic Virus Orthologous Groups
KEGG: Kyoto Encyclopedia of Genes and Genomes
VOG: Virus Orthologous Groups
VIBRANT: Virus identification via iterative annotation

## References

[1] Rani A, Pundir A, Verma M, Joshi S, Verma G, Andjelković S, et al. Methanotrophy: a biological method to mitigate global methane emission. Microbiol Res. 2024;15(2):634–54. 10.3390/microbiolres15020042.

[2] Oshkin IY, Danilova OV, However SY, et al. Expanding characterized diversity and the pool of complete genome sequences of *Methylococcus* species, the bacteria of high environmental and biotechnological relevance. Front Microbiol. 2021;12:756830. 10.3389/fmicb.2021.756830. 34691008 10.3389/fmicb.2021.756830 PMC8527097

[3] Woern C, Grossmann L. Microbial gas fermentation technology for sustainable food protein production. Biotechnol Adv. 2023;69:108240. 10.1016/j.biotechadv.2023.108240. 37647973 10.1016/j.biotechadv.2023.108240

[4] Mikushin P, Martynenko N, Nizovtseva I, Makhaeva K, Nikishina M, Chernushkin D, et al. Novel framework for artificial bubble image generation and boundary detection using superformula regression and computer vision techniques. Mathematics. 2025;13(1):127. 10.3390/math13010127.

[5] Nizovtseva I, Mikushin P, Starodumov I, Makhaeva K, Kraev S, Chernushkin D. Bubble Detection in Multiphase Flows Through Computer Vision and Deep Learning for Applied Modeling. Mathematics. 2024;12(23):3864. 10.3390/math12233864.

[6] Mikushin P, Starodumov I, Shuvaev A, et al. Assessment of otr measurement techniques in the bubble environment of an industrial fermenter. Eur Phys J Spec Top. 2024;233:3519–34. 10.1140/epjs/s11734-024-01378-x.

[7] Nizovtseva I, Palmin V, Simkin I, Starodumov I, Mikushin P, Nozik A, et al. Assessing the mass transfer coefficient in jet bioreactors with classical computer vision methods and neural networks algorithms. Algorithms. 2023;16(3):125. 10.3390/a16030125.

[8] Nizovtseva IG, Starodumov IO, Shchelyaev AY, et al. Simulation of two-phase air–liquid flows in a closed bioreactor loop: numerical modelling, experiments, and verification. Math Meth Appl Sci. 2022;45(13):8216–29. 10.1002/mma.8132.

[9] Zhang YZ, Liu Y, Bai Z, Fujimoto K, Uematsu S, Imoto S. Zero-shot-capable identification of phage-host relationships with whole-genome sequence representation by contrastive learning. Brief Bioinform. 2023;24(5):bbad239. 10.1093/bib/bbad239. 37466138 10.1093/bib/bbad239 PMC10516345

[10] Sweet T Jr, Sindi S, Sistrom M. Going through phages: a computational approach to revealing the role of prophage in *Staphylococcus aureus*. Access Microbiol. 2023;5(6):acmi000424. 10.1099/acmi.0.000424. 37424556 10.1099/acmi.0.000424 PMC10323782

[11] López-Leal G, Camelo-Valera LC, Hurtado-Ramírez JM, Verleyen J, Castillo-Ramírez S, Reyes-Muñoz A. Mining of thousands of prokaryotic genomes reveals high abundance of prophages with a strictly narrow host range. mSystems. 2022;7(4):e0032622. 10.1128/msystems.00326-22. 35880895 10.1128/msystems.00326-22 PMC9426530

[12] Canchaya C, Proux C, Fournous G, Bruttin A, Brüssow H. Prophage genomics [published correction appears in Microbiol Mol Biol Rev. 2003 Sep;67(3):473]. Microbiol Mol Biol Rev. 2003;67(2):238–76. 10.1128/MMBR.67.2.238-276.2003. 12794192 10.1128/MMBR.67.2.238-276.2003 PMC156470

[13] Roux S, Krupovic M, Daly RA, et al. Cryptic inoviruses revealed as pervasive in bacteria and archaea across Earth’s biomes. Nat Microbiol. 2019;4(11):1895–906. 10.1038/s41564-019-0510-x. 31332386 10.1038/s41564-019-0510-x PMC6813254

[14] Hendrix RW, Smith MC, Burns RN, Ford ME, Hatfull GF. Evolutionary relationships among diverse bacteriophages and prophages: all the world’s a phage. Proc Natl Acad Sci U S A. 1999;96(5):2192–7. 10.1073/pnas.96.5.2192. 10051617 10.1073/pnas.96.5.2192 PMC26759

[15] Mushegian AR. Are there 1031 virus particles on earth, or more, or fewer? J Bacteriol. 2020;202(9):e00052-20. 10.1128/JB.00052-20. 32071093 10.1128/JB.00052-20 PMC7148134

[16] Casjens S. Prophages and bacterial genomics: what have we learned thus far? Mol Microbiol. 2003;49(2):277–300. 10.1046/j.1365-2958.2003.03580.x. 12886937 10.1046/j.1365-2958.2003.03580.x

[17] Ward N, Larsen Ø, Sakwa J, et al. Genomic insights into methanotrophy: the complete genome sequence of *Methylococcus capsulatus* (Bath). PLoS Biol. 2004;2(10):e303. 10.1371/journal.pbio.0020303. 15383840 10.1371/journal.pbio.0020303 PMC517821

[18] Ishino Y, Shinagawa H, Makino K, Amemura M, Nakata A. Nucleotide sequence of the iap gene, responsible for alkaline phosphatase isozyme conversion in *Escherichia coli*, and identification of the gene product. J Bacteriol. 1987;169(12):5429–33. 10.1128/jb.169.12.5429-5433.1987. 3316184 10.1128/jb.169.12.5429-5433.1987 PMC213968

[19] Walker AR, Shields RC. Investigating CRISPR spacer targets and their impact on genomic diversification of *Streptococcus mutans*. Front Genet. 2022;13:997341. 10.3389/fgene.2022.997341. 36186424 10.3389/fgene.2022.997341 PMC9522601

[20] Stern A, Mick E, Tirosh I, Sagy O, Sorek R. CRISPR targeting reveals a reservoir of common phages associated with the human gut microbiome. Genome Res. 2012;22(10):1985–94. 10.1101/gr.138297.112. 22732228 10.1101/gr.138297.112 PMC3460193

[21] Ho SFS, Wheeler NE, Millard AD, van Schaik W. Gauge your phage: benchmarking of bacteriophage identification tools in metagenomic sequencing data. Microbiome. 2023;11(1):84. 10.1186/s40168-023-01533-x. 37085924 10.1186/s40168-023-01533-x PMC10120246

[22] Wishart DS, Han S, Saha S, et al. PHASTEST: faster than PHASTER, better than PHAST. Nucleic Acids Res. 2023;51(W1):W443–50. 10.1093/nar/gkad382. 37194694 10.1093/nar/gkad382 PMC10320120

[23] Starikova EV, Tikhonova PO, Prianichnikov NA, et al. Phigaro: high-throughput prophage sequence annotation. Bioinformatics. 2020;36(12):3882–4. 10.1093/bioinformatics/btaa250. 32311023 10.1093/bioinformatics/btaa250

[24] Kieft K, Zhou Z, Anantharaman K. VIBRANT: automated recovery, annotation and curation of microbial viruses, and evaluation of viral community function from genomic sequences. Microbiome. 2020;8(1):90. 10.1186/s40168-020-00867-0. 32522236 10.1186/s40168-020-00867-0 PMC7288430

[25] Akhter S, Aziz RK, Edwards RA. PhiSpy: a novel algorithm for finding prophages in bacterial genomes that combines similarity- and composition-based strategies. Nucleic Acids Res. 2012;40(16):e126. 10.1093/nar/gks406. 22584627 10.1093/nar/gks406 PMC3439882

[26] Hyatt D, Chen GL, Locascio PF, Land ML, Larimer FW, Hauser LJ. Prodigal: prokaryotic gene recognition and translation initiation site identification. BMC Bioinform. 2010;11(1):119. 10.1186/1471-2105-11-119. 10.1186/1471-2105-11-119 PMC2848648 20211023

[27] Grazziotin AL, Koonin EV, Kristensen DM. Prokaryotic virus orthologous groups (pVOGs): a resource for comparative genomics and protein family annotation. Nucleic Acids Res. 2017;45(D1):D491–8. 10.1093/nar/gkw975. 27789703 10.1093/nar/gkw975 PMC5210652

[28] Kanehisa M, Goto S. KEGG: kyoto encyclopedia of genes and genomes. Nucleic Acids Res. 2000;28(1):27–30. 10.1093/nar/28.1.27. 10592173 10.1093/nar/28.1.27 PMC102409

[29] El-Gebali S, Mistry J, Bateman A, et al. The Pfam protein families database in 2019. Nucleic Acids Res. 2019;47(D1):D427–32. 10.1093/nar/gky995. 30357350 10.1093/nar/gky995 PMC6324024

[30] Trgovec-Greif L, Hellinger H-J, Mainguy J, Pfundner A, Frishman D, Kiening M, et al. VOGDB-database of virus orthologous groups. Viruses. 2024;16:1191. 10.3390/v16081191. 39205165 10.3390/v16081191 PMC11360334

[31] Nayfach, S., Camargo, A.P., Schulz, F. et al. CheckV assesses the quality and completeness of metagenome-assembled viral genomes. Nat Biotechnol 39, 578–585 (2021). 10.1038/s41587-020-00774-7. 33349699 10.1038/s41587-020-00774-7 PMC8116208

[32] Darling AE, Mau B, Perna NT. ProgressiveMauve: multiple genome alignment with gene gain, loss and rearrangement. PLoS ONE. 2010;5(6):e11147. 10.1371/journal.pone.0011147. 20593022 10.1371/journal.pone.0011147 PMC2892488

[33] Moraru C, Varsani A, Kropinski AM. VIRIDIC - a novel tool to calculate the intergenomic similarities of prokaryote-infecting viruses. Viruses. 2020;12(11):1268. 10.3390/v12111268. 33172115 10.3390/v12111268 PMC7694805

[34] Turner D, Kropinski AM, Adriaenssens EM. A roadmap for genome-based phage taxonomy. Viruses. 2021;13(3):506. 10.3390/v13030506. 33803862 10.3390/v13030506 PMC8003253

[35] Nishimura Y, Yoshida T, Kuronishi M, Uehara H, Ogata H, Goto S. Viptree: the viral proteomic tree server. Bioinformatics. 2017;33(15):2379–80. 10.1093/bioinformatics/btx157. 28379287 10.1093/bioinformatics/btx157

[36] Bouras G, Nepal R, Houtak G, Psaltis AJ, Wormald PJ, Vreugde S. Pharokka: a fast scalable bacteriophage annotation tool. Bioinformatics. 2023;39(1):btac776. 10.1093/bioinformatics/btac776. 36453861 10.1093/bioinformatics/btac776 PMC9805569

[37] Wang RH, Yang S, Liu Z, et al. Phagescope: a well-annotated bacteriophage database with automatic analyses and visualizations. Nucleic Acids Res. 2024;52(D1):D756–61. 10.1093/nar/gkad979. 37904614 10.1093/nar/gkad979 PMC10767790

[38] Gilchrist CLM, Chooi YH. Clinker & clustermap.js: automatic generation of gene cluster comparison figures. Bioinformatics. 2021;37(16):2473–5. 10.1093/bioinformatics/btab007. 33459763 10.1093/bioinformatics/btab007

[39] Couvin D, Bernheim A, Toffano-Nioche C, et al. CRISPRCasFinder, an update of CRISRFinder, includes a portable version, enhanced performance and integrates search for Cas proteins. Nucleic Acids Res. 2018;46(W1):W246–51. 10.1093/nar/gky425. 29790974 10.1093/nar/gky425 PMC6030898

[40] Makarova KS, Wolf YI, Iranzo J, et al. Evolutionary classification of CRISPR‒Cas systems: a burst of class 2 and derived variants. Nat Rev Microbiol. 2020;18(2):67–83. 10.1038/s41579-019-0299-x. 31857715 10.1038/s41579-019-0299-x PMC8905525

[41] Zhang R, Mirdita M, Levy Karin E, Norroy C, Galiez C, Söding J. SpacePHARER: sensitive identification of phages from CRISPR spacers in prokaryotic hosts. Bioinformatics. 2021;37(19):3364–6. 10.1093/bioinformatics/btab222. 33792634 10.1093/bioinformatics/btab222 PMC8504623

[42] Dion W, Tao Y, Chambers M, et al. SON-dependent nuclear speckle rejuvenation alleviates proteinopathies. bioRxiv. 2024. 10.1101/2024.04.18.590103.39569145

[43] Toms A, Barrangou R. On the global CRISPR array behavior in class i systems. Biol Direct. 2017;12(1):20. 10.1186/s13062-017-0193-2. 28851439 10.1186/s13062-017-0193-2 PMC5575924

[44] Martynov A, Severinov K, Ispolatov I. Optimal number of spacers in CRISPR arrays. PLoS Comput Biol. 2017;13(12):e1005891. 10.1371/journal.pcbi.1005891. 29253874 10.1371/journal.pcbi.1005891 PMC5749868

[45] Bradde S, Nourmohammad A, Goyal S, Balasubramanian V. The size of the immune repertoire of bacteria. Proc Natl Acad Sci U S A. 2020;117(10):5144–51. 10.1073/pnas.1903666117. 32071241 10.1073/pnas.1903666117 PMC7071851

[46] Smith DD, Dalton H. Solubilisation of methane monooxygenase from *Methylococcus capsulatus* (Bath). Eur J Biochem. 1989;182(3):667–71. 10.1111/j.1432-1033.1989.tb14877.x. 2502395 10.1111/j.1432-1033.1989.tb14877.x

[47] Woodland MP, Dalton H. Purification and characterization of component A of the methane monooxygenase from Methylococcus capsulatus (Bath). J Biol Chem. 1984;259(1):53–9. 6323414

[48] Vercoe RB, Chang JT, Dy RL, et al. Cytotoxic chromosomal targeting by CRISPR/Cas systems can reshape bacterial genomes and expel or remodel pathogenicity islands. PLoS Genet. 2013;9(4):e1003454. 10.1371/journal.pgen.1003454. 23637624 10.1371/journal.pgen.1003454 PMC3630108

[49] Martínez Arbas S, Narayanasamy S, Herold M, et al. Roles of bacteriophages, plasmids and CRISPR immunity in microbial community dynamics revealed using time-series integrated meta-omics. Nat Microbiol. 2021;6(1):123–35. 10.1038/s41564-020-00794-8. 33139880 10.1038/s41564-020-00794-8 PMC7752763

[50] Seemann T. Prokka: rapid prokaryotic genome annotation. Bioinformatics. 2014;30(14):2068–9. 10.1093/bioinformatics/btu153. 24642063 10.1093/bioinformatics/btu153

[51] Emms DM, Kelly S. OrthoFinder: phylogenetic orthology inference for comparative genomics. Genome Biol. 2019;20(1):238. 10.1186/s13059-019-1832-y. 31727128 10.1186/s13059-019-1832-y PMC6857279

[52] Katoh K, Standley DM. MAFFT multiple sequence alignment software version 7: improvements in performance and usability. Mol Biol Evol. 2013;30(4):772–80. 10.1093/molbev/mst010. 23329690 10.1093/molbev/mst010 PMC3603318

[53] Capella-Gutiérrez S, Silla-Martínez JM, Gabaldón T. TrimAl: a tool for automated alignment trimming in large-scale phylogenetic analyses. Bioinformatics. 2009;25(15):1972–3. 10.1093/bioinformatics/btp348. 19505945 10.1093/bioinformatics/btp348 PMC2712344

[54] Letunic I, Bork P. Interactive tree of life (iTOL) v6: recent updates to the phylogenetic tree display and annotation tool. Nucleic Acids Res. 2024;52(W1):W78–82. 10.1093/nar/gkae268. 38613393 10.1093/nar/gkae268 PMC11223838

[55] McNair K, Zhou C, Dinsdale EA, Souza B, Edwards RA. PHANOTATE: a novel approach to gene identification in phage genomes. Bioinformatics. 2019;35(22):4537–42. 10.1093/bioinformatics/btz265. 31329826 10.1093/bioinformatics/btz265 PMC6853651

[56] Chan PP, Lin BY, Mak AJ, Lowe TM. tRNAscan-SE 2.0: improved detection and functional classification of transfer RNA genes. Nucleic Acids Res. 2021;49(16):9077–96. 10.1093/nar/gkab688. 34417604 10.1093/nar/gkab688 PMC8450103

[57] Laslett D, Canback B. ARAGORN, a program to detect tRNA genes and tmRNA genes in nucleotide sequences. Nucleic Acids Res. 2004;32(1):11–6. 10.1093/nar/gkh152. 14704338 10.1093/nar/gkh152 PMC373265

[58] Terzian P, Olo Ndela E, Galiez C, et al. PHROG: families of prokaryotic virus proteins clustered using remote homology. NAR Genomics Bioinform. 2021;3(3):lqab067. 10.1093/nargab/lqab067. 10.1093/nargab/lqab067 PMC8341000 34377978

[59] Chen L, Yang J, Yu J, et al. VFDB: a reference database for bacterial virulence factors. Nucleic Acids Res. 2005;33:D325–8. 10.1093/nar/gki008. 15608208 10.1093/nar/gki008 PMC539962

[60] Alcock BP, Raphenya AR, Lau TTY, et al. CARD 2020: antibiotic resistome surveillance with the comprehensive antibiotic resistance database. Nucleic Acids Res. 2020;48(D1):D517–25. 10.1093/nar/gkz935. 31665441 10.1093/nar/gkz935 PMC7145624

[61] Steinegger M, Söding J. MMseqs2 enables sensitive protein sequence searching for the analysis of massive datasets. Nat Biotechnol. 2017;35(11):1026–8. 10.1038/nbt.3988. 29035372 10.1038/nbt.3988

[62] Pell LG, Kanelis V, Donaldson LW, Howell PL, Davidson AR. The phage λ major tail protein structure reveals a common evolution for long-tailed phages and the type VI bacterial secretion system. Proc Natl Acad Sci U S A. 2009;106(11):4160–5. 10.1073/pnas.0900044106. 19251647 10.1073/pnas.0900044106 PMC2657425

[63] Veesler D, Cambillau C. A common evolutionary origin for tailed-bacteriophage functional modules and bacterial machineries. Microbiol Mol Biol Rev. 2011;75(3):423–33. 10.1128/MMBR.00014-11. 21885679 10.1128/MMBR.00014-11 PMC3165541

[64] Meier-Kolthoff JP, Göker M. VICTOR: genome-based phylogeny and classification of prokaryotic viruses. Bioinformatics. 2017;33(21):3396–404. 10.1093/bioinformatics/btx440. 29036289 10.1093/bioinformatics/btx440 PMC5860169

[65] Meier-Kolthoff JP, Auch AF, Klenk HP, Göker M. Genome sequence-based species delimitation with confidence intervals and improved distance functions. BMC Bioinformatics. 2013;14(1):60. 10.1186/1471-2105-14-60. 23432962 10.1186/1471-2105-14-60 PMC3665452

[66] Lefort V, Desper R, Gascuel O. FastME 2.0: a comprehensive, accurate, and fast distance-based phylogeny inference program. Mol Biol Evol. 2015;32(10):2798–800. 10.1093/molbev/msv150. 26130081 10.1093/molbev/msv150 PMC4576710

[67] Farris JS. Estimating phylogenetic trees from distance matrices. Am Nat. 1972;106(951):645–68. 10.1086/282802.

[68] Yu G. Using ggtree to visualize data on tree-like structures. Curr Protoc Bioinformatics. 2020;69(1):e96. 10.1002/cpbi.96. 32162851 10.1002/cpbi.96

[69] Göker M, García-Blázquez G, Voglmayr H, Tellería MT, Martín MP. Molecular taxonomy of phytopathogenic fungi: a case study in Peronospora. PLoS ONE. 2009;4(7):e6319. 10.1371/journal.pone.0006319. 19641601 10.1371/journal.pone.0006319 PMC2712678

[70] O’Leary NA, Wright MW, Brister JR, et al. Reference sequence (RefSeq) database at NCBI: current status, taxonomic expansion, and functional annotation. Nucleic Acids Res. 2016;44(D1):D733–45. 10.1093/nar/gkv1189. 26553804 10.1093/nar/gkv1189 PMC4702849

[71] Benson DA, Cavanaugh M, Clark K, et al. GenBank. Nucleic Acids Res. 2018;46(D1):D41–7. 10.1093/nar/gkx1094. 29140468 10.1093/nar/gkx1094 PMC5753231

[72] Kanz C, Aldebert P, Althorpe N, Baker W, Baldwin A, Bates K, et al. The EMBL nucleotide sequence database. Nucleic Acids Res. 2005;33:D29-33. 10.1093/nar/gki098. 15608199 10.1093/nar/gki098 PMC540052

[73] Ogasawara O, Kodama Y, Mashima J, Kosuge T, Fujisawa T. DDBJ database updates and computational infrastructure enhancement. Nucleic Acids Res. 2020;48(D1):D45–50. 10.1093/nar/gkz982. 31724722 10.1093/nar/gkz982 PMC7145692

[74] Russell DA, Hatfull GF. PhagesDB: the actinobacteriophage database. Bioinformatics. 2017;33(5):784–6. 10.1093/bioinformatics/btw711. 28365761 10.1093/bioinformatics/btw711 PMC5860397

[75] Gregory AC, Zayed AA, Conceição-Neto N, et al. Marine DNA viral macro- and microdiversity from pole to pole. Cell. 2019;177(5):1109-1123.e14. 10.1016/j.cell.2019.03.040. 31031001 10.1016/j.cell.2019.03.040 PMC6525058

[76] Gregory AC, Zablocki O, Zayed AA, Howell A, Bolduc B, Sullivan MB. The gut virome database reveals age-dependent patterns of virome diversity in the human gut. Cell Host Microbe. 2020;28(5):724-740.e8. 10.1016/j.chom.2020.08.003. 32841606 10.1016/j.chom.2020.08.003 PMC7443397

[77] Camarillo-Guerrero LF, Almeida A, Rangel-Pineros G, Finn RD, Lawley TD. Massive expansion of human gut bacteriophage diversity. Cell. 2020;184:1098-1109.e9. 10.1016/j.cell.2021.01.029 PMC7895897 33606979

[78] Stephen, Nayfach D, Páez-Espino L, Call SJ, Low H, Sberro NN, Ivanova AD, Proal MA, Fischbach AS, Bhatt P, Hugenholtz NC, Kyrpides (2021) Metagenomic compendium of 189680 DNA viruses from the human gut microbiome. Nat Microbiol 6(7):960–970. 10.1038/s41564-021-00928-6 10.1038/s41564-021-00928-6 PMC8241571 34168315

[79] Tisza MJ, Buck CB. A catalog of tens of thousands of viruses from human metagenomes reveals hidden associations with chronic diseases. Proc Natl Acad Sci U S A. 2021;118(23):e2023202118. 10.1073/pnas.2023202118. 34083435 10.1073/pnas.2023202118 PMC8201803

[80] Santos-Medellin C, Zinke LA, Ter Horst AM, Gelardi DL, Parikh SJ, Emerson JB. Viromes outperform total metagenomes in revealing the spatiotemporal patterns of agricultural soil viral communities. ISME J. 2021;15(7):1956–70. 10.1038/s41396-021-00897-y. 33612831 10.1038/s41396-021-00897-y PMC8245658

[81] Zhang X, Wang R, Xie X, Hu Y, Wang J, Sun Q, et al. Mining bacterial NGS data vastly expands the complete genomes of temperate phages. NAR Genom Bioinform. 2022;4:lqac057. 10.1093/nargab/lqac057. 35937545 10.1093/nargab/lqac057 PMC9346568

[82] Shah SA, Deng L, Thorsen J, et al. Expanding known viral diversity in the healthy infant gut. Nat Microbiol. 2023;8(5):986–98. 10.1038/s41564-023-01345-7. 37037943 10.1038/s41564-023-01345-7 PMC10159846

[83] Camargo AP, Nayfach S, Chen IA, et al. IMG/VR v4: an expanded database of uncultivated virus genomes within a framework of extensive functional, taxonomic, and ecological metadata. Nucleic Acids Res. 2023;51(D1):D733–43. 10.1093/nar/gkac1037. 36399502 10.1093/nar/gkac1037 PMC9825611

