# Supplementary material for: Identification and comparative genomic analysis of prophage sequences and CRISPR‒Cas immunity in Methylococcus genomes: insights into industrial methane bioconversion
Source: Biotechnol Biofuels Bioprod. 2026 Jan 29;19:21. doi: 10.1186/s13068-026-02738-6 (PMC12924245; doi:10.1186/s13068-026-02738-6)
Supplement: Supplementary file 1 — Supplementary Material 1. [file 13068_2026_2738_MOESM1_ESM.pdf]

Supplementary materials

Table S1. Methanotrophic Bacterial Strains Used in This Study

Num	Species, strain	RefSeq accession number	Genome size (bp)	Isolation source	Collection date	Region of isolation	Project / published
**1**	Methylococcus capsulatus, str. Bath	NC_002977	3 304 561	National Collection of Industrial and Marine Bacteria (NCIMB 11132)	2004-10-01	-	DOI: 10.1371/journal.pbio.0020303
**2**	Methylococcus sp., str. Mc7	NZ_CP079095	4 003 396	landfill cover soil	2020-06-11	Russia, Khanty-Mansiysk	Oshkin IY, 2021 Oct 6;12:756830. doi: 10.3389/fmicb.2021.756830
**3**	Methylococcus capsulatus, str. BH	NZ_CP079096	3 187 959	lake sediment	2020-11-14	Russia, Krasnodar region	Oshkin IY, 2021 Oct 6;12:756830. doi: 10.3389/fmicb.2021.756830
**4**	Methylococcus capsulatus, str. KN2	NZ_CP079097	3 552 732	activated sludge	2020-10-20	Russia, Moscow	Oshkin IY, 2021 Oct 6;12:756830. doi: 10.3389/fmicb.2021.756830
**5**	Methylococcus capsulatus, str. IO1	NZ_CP079098	3 255 256	activated sludge	2020-09-10	Russia, Moscow	Oshkin IY, 2021 Oct 6;12:756830. doi: 10.3389/fmicb.2021.756830
**6**	Methylococcus capsulatus, str. MIR	NZ_CP097161	3 187 097	activated sludge	2020-10-21	Russia, Irkutsk	Draft genome sequence of Methylococcus capsulatus MIR, an obligate methanotroph displaying robust growth on methanol; Unpublished
**7**	Methylococcus capsulatus, str. Mc(Nor)	NZ_OX458332	3 398 174	soil of a functioning landfill methane biofilter	2016	United Kingdom, Norfolk	Draft genome sequence of a biofilter Methylococcus isolate; Unpublished
**8**	Methylococcus geothermalis, str. IM1	NZ_CP046565	3 371 031	hot spring soil	2016-04-01	South Korea	DOI: 10.1099/ijsem.0.004442
**9**	Methylococcus mesophilus, str. 16-5	NZ_CP110921	4 343 116	Rice field soil	2016-06-24	South Korea	A novel Methylococcus sp. isolated from rice field; Unpublished

Table S2. Predicted Prophage Regions

Reg. num.	Algorithm	Region position (length (kb)	Total protein / phage protein	Attachment sites attL - attR	Completeness (Score); morphology
		**Methylococcus capsulatus, str. Bath (NC_002977)**			
1	PHASTEST	2819754 - 2873113 (53.3)	51 / 31	2819754..2819769 - 2873113..2873128	intact (150)
	Phigaro	2824960 - 2874998 (50.0)	50 / 35		Siphoviridae
	VIBRANT	2816780 - 2878183 (61.4)	61 / 38		high quality draft
	PhiSpy	2820020 - 2851790 (31.8)	37 / 33	2820934..2820949 - 2849773..2849788	
2	PHASTEST	3093885 - 3142838 (48.9)	59 / 42	3099890..3099907 - 3142838..3142855	intact (150)
	Phigaro	3092748 - 3138558 (45.8)	63 / 48		Myoviridae
	VIBRANT	3091049 - 3141096 (50.0)	67 / 42		high quality draft
	PhiSpy	3096072 - 3128377 (32.3)	43 / 42	3095155..3095168 - 3126981..3126994	
		**Methylococcus sp., str. Mc7 (NZ_CP079095)**			
1	PHASTEST	1825683 - 1851097 (25.4)	18 / 7	1825683..1825695 - 1851097..1851109	incomplete (60)
2	PhiSpy	938889 - 972163 (33.3)	44 / 43	938508..938519 - 969521..969532	
3	PhiSpy	1444321 - 1460832 (16.5)	24 / 24	1443453..1443499 - 1457149..1457195	
4	PhiSpy	1629311 - 1649204 (19.9)	24 / 24	1629089..1629102 - 1647424..1647437	
5	PhiSpy	2001894 - 2019505 (17.6)	22 / 12	2001914..2001927 - 2021408..2021421	
6	PhiSpy	2542546 - 2557313 (14.8)	23 / 21	2545514..2545554 - 2557443..2557483	
		**Methylococcus capsulatus, str. KN2 (NZ_CP079097)**			
1	PHASTEST	1847198 - 1868022 (20.8)	26 / 19	no	intact (130)
	Phigaro	1826812 - 1881123 (54.3)	59 / 38		Siphoviridae
	VIBRANT	1816647 - 1881124 (64.5)	64 / 16		medium quality draft
	PhiSpy	1848851 - 1883280 (34.4)	42 / 41	1848308..1848321 - 1880960..1880973	
	PHASTEST	2313376 - 2334848 (21.5)	26 / 16	no	intact (100)

**2**	Phigaro	2317958 - 2347495 (29.5)	37 / 24		Siphoviridae
	PhiSpy	2309079 - 2358084 (49.0)	59 / 52	2308062..2308073 - 2356010..2356021	
**3**	VIBRANT	2860568 - 2870561 (10.0)	13 / 13		low quality draft
	PhiSpy	2857626 - 2870866 (13.2)	22 / 21	2855685..2855729 - 2869236..2869280	
**4**	Phigaro	2258052 - 2304686 (46.6)	33 / 21		Siphoviridae
**5**	Phigaro	2488117 - 2496470 (8.4)	7 / 5		Tectiviridae
**6**	VIBRANT	2968034 - 2975097 (7.1)	8 / 8		low quality draft
**Methylococcus capsulatus, str. IO1 (NZ_CP079098)**					
**1**	PHASTEST	2508110 - 2556599 (48.5)	62 / 45	2514769..2514781 - 2547456..2547468	intact (150)
	Phigaro	2509623 - 2560215(50.6)	68 / 51		Myoviridae
	VIBRANT	2512440 - 2569933 (57.5)	75 / 44		high quality draft
	PhiSpy	2514897 - 2554412 (39.5)	55 / 53	2515378..2515391 - 2551541..2551554	
**2**	VIBRANT	830933 - 839256 (8.3)	13 / 3		low quality draft
	PhiSpy	827273 - 839561 (12.3)	20 / 19	827159..827172 - 836574..836587	
**3**	Phigaro	104180 - 121683 (17.5)	15 / 9		Siphoviridae / Tectiviridae
**Methylococcus capsulatus, str. Mc(Nor) (NZ_OX458332)**					
**1**	PHASTEST	493441 - 523538 (30.1)	30 / 21	no	questionable (90)
	Phigaro	494765 - 503693 (8.9)	13 / 8		Siphoviridae
	VIBRANT	492712 - 521922 (29.2)	31 / 16		low quality draft
**2**	PHASTEST	1518704 - 1541006 (22.3)	28 / 22	no	intact (150)
	Phigaro	1500468 - 1541775 (41.3)	52 / 36		Siphoviridae
	VIBRANT	1497043 - 1540680 (43.6)	55 / 32		medium quality draft
	PhiSpy	1497834 - 1544167 (46.3)	61 / 60	1500355..1500371 - 1542412..1542428	
**3**	PHASTEST	1901642 - 1923114 (21.5)	27 / 16	no	intact (100)
	Phigaro	1906224 - 1933389 (27.2)	35 / 23		Siphoviridae
	VIBRANT	1882881 - 1941936 (59.1)	64 / 32		high quality draft

	PhiSpy	1885328 - 1925146 (39.8)	43 / 40	1888240..1888257 - 1921358..1921375	
**4**	Phigaro	1562044 - 1575952 (13.9)	16 / 9		Siphoviridae
			**Methylococcus mesophilus, str. 16-5 (NZ_CP110921)**		
**1**	PHASTEST	8405 - 43171 (34.7)	45 / 23	8405..8416 - 42194..42205	incomplete (30)
	Phigaro	17968 - 30426 (12.5)	18 / 11		Siphoviridae
	VIBRANT	799 - 43171 (42.4)	57 / 30		low quality draft
**2**	PHASTEST	1783651 - 1805198 (21.5)	27 / 13	1783651..1783676 - 1805198..1805223	incomplete (10)
	Phigaro	1793947 - 1802118 (8.2)	13 / 11		Siphoviridae
	PhiSpy	1778947 - 1798732 (19.8)	25 / 13	1777232..1777244 - 1800508..1800520	
**3**	PHASTEST	1921503 - 1966951 (45.4)	53 / 28	1921503..1921514 - 1966951..1966962	incomplete (40)
	Phigaro	1924104 - 1957254 (33.1)	46 / 28		Siphoviridae
	VIBRANT	1897271 - 1994813 (97.5)	113 / 61		medium quality draft
	PhiSpy	1921494 - 1958308 (36.8)	51 / 45	1923010..1923023 - 1958426..1958439	
**4**	PHASTEST	2514364 - 2561403 (47.1)	52 / 38	2514364..2514379 - 2561403..2561418	incomplete (30)
	Phigaro	2520489 - 2561608 (41.1)	53 / 36		Siphoviridae
	VIBRANT	2516963 - 2569198 (52.2)	65 / 39		medium quality draft
	PhiSpy	2519661 - 2557660 (38.0)	51 / 48	2517762..2517775 - 555724..2555737	
**5**	PHASTEST	3238455 - 3278741 (40.2)	41 / 27	3238547..3238560 - 3277160..3277173	incomplete (10)
	Phigaro	3213273 - 3273803 (60.5)	55 / 36		Siphoviridae
	VIBRANT	3177406 - 3285509 (108.1)	123 / 73		high quality draft
	PhiSpy	3242229 - 3289935 (47.7)	61 / 51	3245186..3245200 - 3288226..3288240	
**6**	PhiSpy	446781 - 460166 (13.4)	17 / 15	446578..446621 - 460261..460304	
			**Methylococcus capsulatus, str. BH (NZ_CP079096)**		
**1**	Phigaro	1958526 - 1974301 (15.8)	14 / 7		Siphoviridae
			**Methylococcus capsulatus, str. MIR (NZ_CP097161)**		
**1**	Phigaro	2338624 - 2357160 (18.5)	14 / 8		Siphoviridae

**Methylococcus geothermalis, str. IM1 (NZ_CP046565)**				
**1**	Phigaro	8392 - 14236 (5.8)	7 / 6	Unknown
**2**	Phigaro	932615 - 942864 (10.2)	13 / 8	Myoviridae

Table S3. Number of Spacers in the Different CRISPR-Cas Systems Found in the Analyzed Genomes

Species, strain	CAS-Type-IE	CAS-TypeIC	CAS-TypeIF	CAS-TypeIIIA	Number of spacers in strain
**MIR**	76	58	0	0	134
**Mc7**	17	40	20+40	(without CAS)10+45	172
**Bath**	7	62	0	0	69
**BH**	109	70	0	0	179
**IO1**	79	18	0	0	97
**KN2**	37	34	0	0	71
**Mc(Nor)**	29	32	0	0	61
**16_5**	no cas	no cas	no cas	no cas	no cas
**IM1**		51	124	22	197
**Average number of spacers in CRISPR**	50	46	-	-	122

Table S4. The file shows the CheckV results (quality_summary.tsv) for the 11 prophage regions identified in this paper

contig_id	con-tig_length	pro-virus	provi-ral_length	gene_-count	viral_-genes	host_-genes	check-v_quali-ty	miu-vig_quali-ty	com-plete-ness	complete-ness_method	contam-ination	kmer_freq	warnings
Bath-R1	56731	Yes	43192	57	17	6	Medium-quality	Genome-fragment	72.76	AAI-based (high-confidence)	23.87	1.0	
Bath-R2	45605	No	NA	63	27	1	High-quality	High-qual-ity	100.0	AAI-based (medi-um-confidence)	0.0	1.0	
KN2-R1	71407	Yes	34677	81	22	18	Medium-quality	Genome-fragment	58.61	AAI-based (high-confidence)	51.44	1.0	low-confi-dence Pro-virus
KN2-R2	36045	No	NA	45	16	0	Medium-quality	Genome-fragment	60.92	AAI-based (high-confidence)	0.0	1.0	
IO1-R1	47296	Yes	36329	65	26	10	High-quality	High-qual-ity	97.95	AAI-based (medi-um-confidence)	23.19	1.0	
McNor-R1	29097	No	NA	33	13	1	Medium-quality	Genome-fragment	75.71	AAI-based (medi-um-confidence)	0.0	1.0	
McNor-R2	45876	Yes	42975	58	21	2	Medium-quality	Genome-fragment	72.66	AAI-based (medi-um-confidence)	6.32	1.0	
McNor-R3	41154	No	NA	53	16	2	Medium-quality	Genome-fragment	69.54	AAI-based (high-confidence)	0.0	1.0	
16-5-R1	67726	No	NA	90	20	5	High-quality	High-qual-ity	100.0	AAI-based (high-confidence)	0.0	1.0	
16-5-R2	45879	No	NA	60	29	1	High-quality	High-qual-ity	100.0	AAI-based (medi-um-confidence)	0.0	1.0	
16-5-R3	52455	No	NA	68	18	2	Medium-quality	Genome-fragment	88.67	AAI-based (high-confidence)	0.0	1.0	
