# Supplementary material for: Identification and comparative genomic analysis of prophage sequences and CRISPR‒Cas immunity in Methylococcus genomes: insights into industrial methane bioconversion
Source: Biotechnol Biofuels Bioprod. 2026 Jan 29;19:21. doi: 10.1186/s13068-026-02738-6 (PMC12924245; doi:10.1186/s13068-026-02738-6)
Supplement: Supplementary file 2 — Supplementary Material 2. [file 13068_2026_2738_MOESM2_ESM.pdf]

Data file 1. The file contains all the GenBank sequences for the 11 prophage regions identified in this paper, which have been annotated using the Pharokka

```
LOCUS       Bath-R1              56731 bp    DNA     linear   PHG 16-FEB-2025
DEFINITION  Bath-R1.
ACCESSION   Bath-R1
VERSION     Bath-R1
KEYWORDS    .
SOURCE      .
  ORGANISM  .
            .
FEATURES             Location/Qualifiers
     CDS             complement(3..317)
                     /ID="YLSDQRHL_CDS_0001"
                     /transl_table=11
                     /phrog="No_PHROGs_HMM"
                     /top_hit="No_MMseqs_PHROG_hit"
                     /locus_tag="YLSDQRHL_CDS_0001"
                     /function="unknown function"
                     /product="hypothetical protein"
                     /source="PHANOTATE_1.5.1"
                     /score="-0.1069506150138596"
                     /phase="0"
                     /translation="LTDVTPPAAAAACGRLFCASSRPSRRRKAVSDYNQWSLSPCQARR
                     WPPVPTTDRREKPRTERPCRGGNGARTACASARRRPETAPTLALRARKKANRERLALGY
                     W"
     CDS             complement(310..555)
                     /ID="YLSDQRHL_CDS_0002"
                     /transl_table=11
                     /phrog="14161"
                     /top_hit="No_MMseqs_PHROG_hit"
                     /locus_tag="YLSDQRHL_CDS_0002"
                     /function="other"
                     /product="recombinase"
                     /source="PHANOTATE_1.5.1"
                     /score="-5.628707506581835"
                     /phase="0"
                     /translation="MRALGLAHHWQRLLHEGRVASAAEIAQAEGLDVSTVHRLLRLTLL
                     APEVIERLLGSPDLAIEKVLGRPWPYGWREQVRLLD"
     CDS             complement(509..679)
                     /ID="YLSDQRHL_CDS_0003"
                     /transl_table=11
                     /phrog="No_PHROGs_HMM"
                     /top_hit="No_MMseqs_PHROG_hit"
                     /locus_tag="YLSDQRHL_CDS_0003"
                     /function="unknown function"
                     /product="hypothetical protein"
                     /source="PHANOTATE_1.5.1"
                     /score="-0.0465286857945133"
                     /phase="0"
                     /translation="LAAGAARHPPRGDRAARRAEGGGGAFDGGRPAAPRGQGHGADACA
                     RAGAPLATAAP"
     CDS             complement(771..1154)
```

```
                /ID="YLSDQRHL_CDS_0004"
                /transl_table=11
                /phrog="14161"
                /top_hit="No_MMseqs_PHROG_hit"
                /locus_tag="YLSDQRHL_CDS_0004"
                /function="other"
                /product="recombinase"
                /source="PHANOTATE_1.5.1"
                /score="-20.42808106950166"
                /phase="0"
                /translation="MKDGVRETFVPLTLRRRGVRRLVQHQAEDRDTHDSTLIEGMARAF
                HWQRLLDSGAMPSGSAIARAEGLHHSVVNELLRLTLLAPDIVEMLMAGRQPRRMSLIWF
                QRHPLPVDWVAQREIVRRFEEGA"
CDS             complement(1151..2530)
                /ID="YLSDQRHL_CDS_0005"
                /transl_table=11
                /phrog="95"
                /top_hit="No_MMseqs_PHROG_hit"
                /locus_tag="YLSDQRHL_CDS_0005"
                /function="integration and excision"
                /product="integrase"
                /source="PHANOTATE_1.5.1"
                /score="-55475.5971928414"
                /phase="0"
                /translation="MTEIASTKARKRCAVYCRVSSDERLDQEFNSIDAQKEAGHAFIAS
                QRSEGWISVADDYDDPGFSGGNTERPALKRLMVDIQRGLIDIVVVYKIDRLTRSLPDFS
                KMVEVFERHGVSFVSVTQQFNTTTSMGRLTLNILLSFAQFEREVTGERIRDKIAAAKKK
                GLWMGGVPTIGYDVVNRQLVVNQAEAAVVRRIFEEMLTIGSPTQIAARLTAEGITTKAW
                TTQDGRVRTGTRIDKKYIHKVLRNRIYLGELSNRGQWYPGVHEPIIERELWDQVHAVLA
                RDSHARSVDTKIRSRNDALLRGLLYAPSGERMYPTYSRKNGRKYRYYVSKSESRFGAPG
                KSYERLPAGEIEAAVVAQIRTVLTSPESVAAVVRHVQRQGALIDEATVVMAMGRLNDVW
                DQLFPVEQHRIVNLMIERIDLVHTDEMQGIRVKWRELGWDALIGEFAPRSIGAELLEVE
                A"
CDS             complement(2530..2973)
                /ID="YLSDQRHL_CDS_0006"
                /transl_table=11
                /phrog="11806"
                /top_hit="No_MMseqs_PHROG_hit"
                /locus_tag="YLSDQRHL_CDS_0006"
                /function="unknown function"
                /product="hypothetical protein"
                /source="PHANOTATE_1.5.1"
                /score="-21.96077928452664"
                /phase="0"
                /translation="MNEKQASVAARIAELSHLPMAELWVLWDRYFERRPQFPNRTHVES
                RIAYKMQEEVFGGLAPETRQRLEAIGAKHSKIKLRAKPRVFNFAPGTVLLREWGEREHR
                VTVTAEGRFEYEGRSFKSLTAVARHITGQHWSGPLFFGLKGGA"
CDS             complement(2973..3296)
                /ID="YLSDQRHL_CDS_0007"
                /transl_table=11
                /phrog="No_PHROGs_HMM"
                /top_hit="No_MMseqs_PHROG_hit"
                /locus_tag="YLSDQRHL_CDS_0007"
```

/function="unknown function"
/product="hypothetical protein"
/source="PHANOTATE_1.5.1"
/score="-8.738184917275364"
/phase="0"
/translation="MTTPRSRRKRRQWQADTRRPLPCVAILFGGSVPLSLTVAFPGAVM
KNIELASPLEMSPGARAGEITTILAAAIVRTLGSSGLEQSAARLGFLPDQRVHTTPSQQ
EKL"
CDS complement(3250..5496)
/ID="YLSDQRHL_CDS_0008"
/transl_table=11
/phrog="37634"
/top_hit="No_MMseqs_PHROG_hit"
/locus_tag="YLSDQRHL_CDS_0008"
/function="unknown function"
/product="hypothetical protein"
/source="PHANOTATE_1.5.1"
/score="-64385237.203159645"
/phase="0"
/translation="MGKKTLTNSHCLLELTEKAQAGVLKAFSGLPECQALARGFDWSRD
DGALPAALVERIKHLRKEQRDPAEREALRVLRLASPRGAAILATVAEQLNDSDLIALFL
SQDGCEIGRSVWMRTHSDESARLFDVAESILNTGDLRGNKRLHDAFDVPCDDAPPFIWS
DSVKKELETHLTTAMRLAEPCEVIHVPLADEARDGETKTVHYLVVRFAGEQVTAVQVIN
RNRRSFCYFPARDATLIYAPHRKMVEVYAHTLSTRAPLANVLSKHGFKMPLSNRPLDRS
RYDLSRFARPLKDEKPRIDGAKVEHLYLIEAKALLGHATDAVTLHIDSGAELHEVIDER
WGNHPFAQPGALLGVTLVADLVFEGETSVTPLSIVLAEPGRCSLSGEKDQRLRRVGMQL
LEALGVRKPLHPGSGVDDPNLIAQVARLLECATSPLDGFALAKLGIDIDRLEDEGILTE
GERITETVVQLDDGAPFTVKLERCADLNQVRYRDPLTGMDVVLPAKLARRWKVQLNWLR
EEIITALGSALKGVRGRHLDDEPVFLGEMDIDGHAVALYFAAKMSSERQYARVDAALRL
RPRAVPGIVLTTASIPFPFAGTNVVIPIEDVLSDGGDGSAIDTTRLKVAYRHGQLAAMG
GTTVSLKVSADGYSATLYLPGKAPWKVTNKAKIAVLQRLVDAYTAGTPHVNTKKLMEDT
GCASPANLFSKNSPWRDYLVKVKGAHAWQLNLPTLDAPVDDDTEVETEEAAMAG"
CDS complement(5515..6135)
/ID="YLSDQRHL_CDS_0009"
/transl_table=11
/phrog="No_PHROGs_HMM"
/top_hit="No_MMseqs_PHROG_hit"
/locus_tag="YLSDQRHL_CDS_0009"
/function="unknown function"
/product="hypothetical protein"
/source="PHANOTATE_1.5.1"
/score="-132.10790561682398"
/phase="0"
/translation="MNWRPRPRNSAWGIDWMSSHFTVVYDACVLYPAPLRDLLMHLALS
DLYRARWSDMIHDEWTRNVLVSRPDLTQDQLNRTRQLMNAHVRDCLVTGFEYLIPSIDL
PDPDDRHVVAAAIHSGASLIVTFNLKDFPPEALRPYNLAAQHPDDFIVDLLDLHPAGVL
EAAASHRRSLKNPPKTADEYLDTLLAQGLTQSVAVMRQWIVAM"
CDS complement(6092..6553)
/ID="YLSDQRHL_CDS_0010"
/transl_table=11
/phrog="66"
/top_hit="No_MMseqs_PHROG_hit"
/locus_tag="YLSDQRHL_CDS_0010"

/function="integration and excision"
/product="excisionase and transcriptional regulator"
/source="PHANOTATE_1.5.1"
/score="-40.43280449798307"
/phase="0"
/translation="MNTPAIPKTLPSAEDIALARESGRVLSTVLQTRAETQQIDFHDEK
GAVRTVSIPTSALRLLLDVLTEIGQGNAVSIIPIHAELTTQEAADVLNVSRPFLVQLLE
KGDIPFHKIGTHRRVRYQDVIAYKNRIDAERRKALDELAAQAQELGMGY"
CDS complement(6550..6645)
/ID="YLSDQRHL_CDS_0011"
/transl_table=11
/phrog="No_PHROGs_HMM"
/top_hit="No_MMseqs_PHROG_hit"
/locus_tag="YLSDQRHL_CDS_0011"
/function="unknown function"
/product="hypothetical protein"
/source="PHANOTATE_1.5.1"
/score="-1.1175762589685632"
/phase="0"
/translation="MLAPRRFGYCDFGYFVYNGFRPNFDPTRRAS"
CDS complement(6668..7072)
/ID="YLSDQRHL_CDS_0012"
/transl_table=11
/phrog="No_PHROGs_HMM"
/top_hit="No_MMseqs_PHROG_hit"
/locus_tag="YLSDQRHL_CDS_0012"
/function="unknown function"
/product="hypothetical protein"
/source="PHANOTATE_1.5.1"
/score="-6.638548518848701"
/phase="0"
/translation="MVRFVMAAPSLVRPGEGRVARDAPLEGLDFGDQVGDARPELAEDG
AELLLAPAAQGFDGKPPAGGELVLVKGDARGTVRAGPAGFPAGLNRRDLVLHCGTPLVW
ESLLNCSTLWACASAFWAPMSRARRGKCNP"
CDS 7056..7535
/ID="YLSDQRHL_CDS_0013"
/transl_table=11
/phrog="2480"
/top_hit="No_MMseqs_PHROG_hit"
/locus_tag="YLSDQRHL_CDS_0013"
/function="unknown function"
/product="hypothetical protein"
/source="PHANOTATE_1.5.1"
/score="-27.232123609983535"
/phase="0"
/translation="MTNLTILPADLAAMSVSDLASLPPAQKQEISRNLDEALDWLKKAR
AKFDAALDAAYGEQARAARLAAGKDFGVIHLSDGPLRVTVDIPKRVSWDQEQLAAIARR
IAASGEKVEDYLDIEFSVSESRFNNWPAALRAQFEAARTVKPGKPSFRLALISED"
CDS 7539..8297
/ID="YLSDQRHL_CDS_0014"
/transl_table=11
/phrog="130"
/top_hit="No_MMseqs_PHROG_hit"

```
                /locus_tag="YLSDQRHL_CDS_0014"
                /function="transcription regulation"
                /product="anti-repressor Ant"
                /source="PHANOTATE_1.5.1"
                /score="-558.3775992506239"
                /phase="0"
                /translation="MSTELIPFDFEGRPVRVVTDAQGEPWFVAADVAQSLEYRMASDMT
                RSLDDDEKGTQIVRTPSGNQEMLVINESGLYSAILKSRKPEAKRFKRWVTHEVLPAIRK
                TGAYAAGATLPALPVPTQDRVSSILLIGEAVAKVPGVKAGIAMAATLTCIQENTGLAVE
                TLRRALPAANAPICSLNATQLGKLINRSAKATNQLLAATGLQFRNKRDEWELTEAGEAW
                AEAMPYSRNGHSGYQILWNPAVVEQLKEVA"
CDS                8297..9115
                /ID="YLSDQRHL_CDS_0015"
                /transl_table=11
                /phrog="124"
                /top_hit="No_MMseqs_PHROG_hit"
                /locus_tag="YLSDQRHL_CDS_0015"
                /function="DNA"
                /function=" RNA and nucleotide metabolism"
                /product="Sak4-like ssDNA annealing protein"
                /source="PHANOTATE_1.5.1"
                /score="-387.8996271945424"
                /phase="0"
                /translation="MALPIITADQRLREKQGVKLVLLGKSGIGKTSQLKTLPEGSTLFV
                DLEAGDLAVKDWHGDCVRPTTWPEFRDLVVFLAGPNPALPADAPFSEAHYRHVCERYGD
                PAQLAKYDTYFVDSITVLARLALIWAKTQPQSFSERTGKPDTRGAYGLLGTEMLTALTH
                LQHARGKHVVFVAILDERIDDFNRKVFVPQIEGAKTAAELPGIVDEVVTLAEIKAEDGS
                AYRAFVTHTLNPYGYPAKDRSGQLELLEPPNLRALIDKCAAATRIPTSKE"
CDS                9119..9745
                /ID="YLSDQRHL_CDS_0016"
                /transl_table=11
                /phrog="2300"
                /top_hit="No_MMseqs_PHROG_hit"
                /locus_tag="YLSDQRHL_CDS_0016"
                /function="unknown function"
                /product="hypothetical protein"
                /source="PHANOTATE_1.5.1"
                /score="-42.05377675139183"
                /phase="0"
                /translation="MTAWNDFNDAEQQQSFDLIPKGTVARVRMTIKPGGYDDPAQGWTG
                GYATQSFDTGSIYLACEFVVLEGEYAKRKLWSNVGLHSPKGQTWASMGRSFIRAALNSA
                RNVLPQDNSPQAAAARRIQGFHELDGIEFVARIDIEKDARGELRNVVKLAVEPDQPDYA
                RVMGLPPKTPGGSSGAPAAAIPSRVMPAPAAAQRPAVPGKPAWAQ"
CDS                9807..10280
                /ID="YLSDQRHL_CDS_0017"
                /transl_table=11
                /phrog="21342"
                /top_hit="No_MMseqs_PHROG_hit"
                /locus_tag="YLSDQRHL_CDS_0017"
                /function="unknown function"
                /product="hypothetical protein"
                /source="PHANOTATE_1.5.1"
                /score="-41.91751806385074"
```

/phase="0"
/translation="MSGKCWVCKRQARGFGHTDNRHGVGDPRRYPIDWVFCSRRCQEAF
HALYGNWLRVKEGRADIEEVAMIDPSDVELAAMKKCLKAFGEAAGEIGFGKPLGDYSEA
EALQVIDAIVTCYTEAMVEHHEATKYPPVRGLTNPVSDPFADMKDDLPWEVKP"
CDS 10280..11008
/ID="YLSDQRHL_CDS_0018"
/transl_table=11
/phrog="1990"
/top_hit="No_MMseqs_PHROG_hit"
/locus_tag="YLSDQRHL_CDS_0018"
/function="DNA"
/function=" RNA and nucleotide metabolism"
/product="Cas4-domain exonuclease"
/source="PHANOTATE_1.5.1"
/score="-165.3018718619659"
/phase="0"
/translation="MDFNSSASLSGQVTALVDLGMQLARSLQGTRQYLGASRLGASCER
VLQYEYAQAPVDPGRETEGRMLRIFERGHVIEDCMVTWLRDAGFDLRTRKADGEQFGFI
ALDGRLQGHVDGVIVGGPEGFGYPALWENKCLGAKSWRELEKNRLASARPIYAAQVALY
QAYLELHEHPALFTAVNADTMEIYAELVPFDAELAQRMSDRAVKVISATEAGELLPRSF
SEPTHFECRMCPWQDRCWRV"
CDS 11012..11284
/ID="YLSDQRHL_CDS_0019"
/transl_table=11
/phrog="66"
/top_hit="No_MMseqs_PHROG_hit"
/locus_tag="YLSDQRHL_CDS_0019"
/function="integration and excision"
/product="excisionase and transcriptional regulator"
/source="PHANOTATE_1.5.1"
/score="-9.791192636237016"
/phase="0"
/translation="MKNNDERTLTAVSEMVGEPMIDAKQAAAALRLPYYWFADHAMRSK
YRIPHYLLGGLVRYRLSELSAWAARSAAVQGREAREEDTPAGEAE"
CDS 11281..13548
/ID="YLSDQRHL_CDS_0020"
/transl_table=11
/phrog="6088"
/top_hit="No_MMseqs_PHROG_hit"
/locus_tag="YLSDQRHL_CDS_0020"
/function="DNA"
/function=" RNA and nucleotide metabolism"
/product="DNA primase"
/source="PHANOTATE_1.5.1"
/score="-2571397.21575563"
/phase="0"
/translation="MIDFNDIPQPEAQDRQARREEIRAALLARLESVLFALLPAGKKRG
GKFLIGDVLGSPGDSLEVVLDGEKAGLWTDRATGDGGDIFGLIAAHLGADVHADFPRVL
DVAADLVGRAHPAPPRKARSAPPVDGLGPATAKWDYFDPAGKLIAVVYRYDPPGRKKEF
RPWDARRRKMAPPEPRPLYNQPGLVAADTVVLVEGEKCAQALIDIGVAATTAMHGANAP
VDKTDWSPLAGKAVLIWPDRDKPGWEYAMAAAQAALAVGAMSCDVLLPPDDRSEGWDAA
DAVAEGFDVAGFLAFGPRMSIKPASVTPTQEPSVWATDDALALAFTSRYAEDWRYCAAW
GKWLVWDGRRWQADETLLVHHLIRAICREAALKADSHRLAAKLAASSTVGGVERLARTD

RRHASTSEEWDADLFALNTPSGIVDLKTGRIKPHDRRERMTKLATATPHGDCARWRAFL
EDITGGDTALQAYLQRMVGYCLTGATSAHALFFLYGTGANGKSVFVNTLATILGDYATS
APMDTFMEARGDRHPTDLAGLRGARFVSSIETEQGRRWNESKVKAITGGDKVSARFMRQ
DFFEYLPQFKLVIAGNHKPAIRNVDEAMKRRLHLIPFTVTIPPEKRDGQLTEKLLAERD
GILAWAVEGCLAWQRDGLNPPASVVSATEEYFDEEDAIGDFLDEEAQRFEQARVAVADV
FQRWQEWATRRGEYVGTSRWLAQQLANRGFGRARLHGGVKGLAGLSLKPRDYGTRLPYR
DD"
CDS 13684..14160
/ID="YLSDQRHL_CDS_0021"
/transl_table=11
/phrog="159"
/top_hit="No_MMseqs_PHROG_hit"
/locus_tag="YLSDQRHL_CDS_0021"
/function="DNA"
/function=" RNA and nucleotide metabolism"
/product="RuvC-like Holliday junction resolvase"
/source="PHANOTATE_1.5.1"
/score="-28.193140504421304"
/phase="0"
/translation="MTTTILALDLGTTTGWALRGGDGHITSGSESFRPQRFEGGGMRFL
RFKRWLTELKGHVDGIGALYFEEVRRHASTDAAHAYGGFLATLTAWCEHHQIPYQGVPV
GTIKKHATGKGNAGKEGVIASVRARGHAPSDDNEADALALLLWAIQNHDDGQEV"
CDS 14162..14371
/ID="YLSDQRHL_CDS_0022"
/transl_table=11
/phrog="19772"
/top_hit="No_MMseqs_PHROG_hit"
/locus_tag="YLSDQRHL_CDS_0022"
/function="unknown function"
/product="hypothetical protein"
/source="PHANOTATE_1.5.1"
/score="-0.9769933899216008"
/phase="0"
/translation="VKIPTPRYRCPLGRLQPDTTDLDAIKQRGWRDQHILVVNAFDERL
DFIEREIVRRIGERLYGQGGTNHG"
CDS 14364..14780
/ID="YLSDQRHL_CDS_0023"
/transl_table=11
/phrog="6836"
/top_hit="No_MMseqs_PHROG_hit"
/locus_tag="YLSDQRHL_CDS_0023"
/function="unknown function"
/product="hypothetical protein"
/source="PHANOTATE_1.5.1"
/score="-44.29037083380136"
/phase="0"
/translation="MAEWTIDDVAARFEEAATTGRRLPPVRVQGYFNTWPAFVRKEWEA
FAADEKVYRPFPPSPEAIDRMLETMRWVQWLEVEQRHLVWMRAKRYGWRDITIRFACDR
TTAWRRWQKALEIVAARLNEKGLPNFAKAATMKP"
CDS 14777..15013
/ID="YLSDQRHL_CDS_0024"
/transl_table=11
/phrog="No_PHROGs_HMM"

/top_hit="No_MMseqs_PHROG_hit"
/locus_tag="YLSDQRHL_CDS_0024"
/function="unknown function"
/product="hypothetical protein"
/source="PHANOTATE_1.5.1"
/score="-5.272074621140132"
/phase="0"
/translation="MSTMNISLPDALKAFVDEQVSQRGYGTSSEYVRELIRKDQDRQRL
RGLLLAGAASAPTAPADSDYFDSLRARVRTARG"

CDS 15010..15345
/ID="YLSDQRHL_CDS_0025"
/transl_table=11
/phrog="2455"
/top_hit="No_MMseqs_PHROG_hit"
/locus_tag="YLSDQRHL_CDS_0025"
/function="unknown function"
/product="hypothetical protein"
/source="PHANOTATE_1.5.1"
/score="-11.19967496229587"
/phase="0"
/translation="MKAKPVIPRALATQDVEEAIRYYLSEDAKQAALGFIDALEQAYSH
IGRHPTTGSPRYAHELDLPGLRSWPLKRYPHIVFYVERDDHIDVWRVLHGMRDIPAWMR
AGDDTPR"

CDS 15406..15537
/ID="YLSDQRHL_CDS_0026"
/transl_table=11
/phrog="No_PHROGs_HMM"
/top_hit="No_MMseqs_PHROG_hit"
/locus_tag="YLSDQRHL_CDS_0026"
/function="unknown function"
/product="hypothetical protein"
/source="PHANOTATE_1.5.1"
/score="-0.0652309281945003"
/phase="0"
/translation="LPCLSVSTLGNPATKQPGRGSISAIFWTAVQAAKTAQGKRGPS"

CDS 15601..15735
/ID="YLSDQRHL_CDS_0027"
/transl_table=11
/phrog="No_PHROGs_HMM"
/top_hit="No_MMseqs_PHROG_hit"
/locus_tag="YLSDQRHL_CDS_0027"
/function="unknown function"
/product="hypothetical protein"
/source="PHANOTATE_1.5.1"
/score="-0.0748758007380286"
/phase="0"
/translation="LQGLQFAPPPLSAHVTSPPTAFRRRVFFLAAPKRRCACLPRNRF"

CDS 15798..17141
/ID="YLSDQRHL_CDS_0028"
/transl_table=11
/phrog="498"
/top_hit="No_MMseqs_PHROG_hit"
/locus_tag="YLSDQRHL_CDS_0028"

/function="other"
/product="DNA methyltransferase"
/source="PHANOTATE_1.5.1"
/score="-14976.467059669108"
/phase="0"
/translation="MHSETQVAKIAASIVEYGWTSPILVDGDNGIIAGHGRLAAARKLG
LAEVPVIELAHLTPTQKRALVLADNRLALEAGWDEELLALELAELSDAGYDLALTGFDD
GEIEAFLSEPQPHEDTSAEADAGKDDTTDDVPDAPVVPVSRPGDVWCLGSHRLICGDAT
DPAVVAALMRGERAKLCFTSPPYGNQRDYTTGGIADWDALMRGVFGHLPMAEDGQVLVN
LGLIHRDNEVIPYWDAWLGWMRAQGWRRFAWYVWDQGPGMPGDWAGRFAPSFEFVFHFN
RQSRKPNKIVPCKHAGQDSHLRADGSSTAMRNKDGEVGGWTHAGLPTQDTRIPDSVIRV
MRHKGKIGKGIDHPAVFPVALPQFVMEAYSDEGAVVFEPFGGSGTTMLAAERTGRVCRS
VEIAPEYVDVAIKRFQQNHPDVAVTLLASGQSFAEVAAEREREAEVVA"
CDS 17138..18403
/ID="YLSDQRHL_CDS_0029"
/transl_table=11
/phrog="498"
/top_hit="No_MMseqs_PHROG_hit"
/locus_tag="YLSDQRHL_CDS_0029"
/function="other"
/product="DNA methyltransferase"
/source="PHANOTATE_1.5.1"
/score="-14049.570343929288"
/phase="0"
/translation="MTNSWFADKIEQWPTAKLVPYARNARTHSDEQIAQIAASIAEFGF
TNPILAGSDGVIVAGHGRLAAAQKLGLDIVPVVVLDHLTPTQRRALVIADNRIAENAGW
DDALLRVELKALQDEGFDLDLTGFDAGALADLLAGDEPANEGQTDDDVVPEVSETPVSR
PGDVWLLGPHRLLCGDATVADSYARLLAGDPVDMVFTDPPYNVNYANSAKDKMRGKDRA
ILNDNLGDGFHDFLLAALTPTVANCRGAIYVAMSSSELDTLQAAFRAAGGHWSTFIIWA
KNTFTLGRADYQRQYEPILYGWPEGAQRHWCGDRDQGDVWQIKKPQRNDLHPTMKPVEL
VERAIRNSSRPGAVVLDPFGGSGTTLIAAEKAGRVARLIELDPKYVDVIVRRWQDWTGK
QATREADGLAFDQAATSSATIL"
CDS complement(18370..18960)
/ID="YLSDQRHL_CDS_0030"
/transl_table=11
/phrog="10315"
/top_hit="No_MMseqs_PHROG_hit"
/locus_tag="YLSDQRHL_CDS_0030"
/function="unknown function"
/product="hypothetical protein"
/source="PHANOTATE_1.5.1"
/score="-83.190884301461"
/phase="0"
/translation="MTTIQLTPAQHAIVAYAIEHTDGKIVWFPDNVKGGARKKVLDGLF
RRALITSIGADWFVAAEGYDAMGCPRPAPAPVEPDEELEAAVSAAEATWAQRRSDAKPR
TRENERSEVSRGEAERVRSIRQNSKQAEVIRMLKRPEGATVRQICEATGWQAHTVRGTF
AGAFKKKLGLVLVSEKLDGGERVYRIVAEDVAA"
CDS 19071..19607
/ID="YLSDQRHL_CDS_0031"
/transl_table=11
/phrog="57"
/top_hit="No_MMseqs_PHROG_hit"
/locus_tag="YLSDQRHL_CDS_0031"

/function="head and packaging"
/product="terminase small subunit"
/source="PHANOTATE_1.5.1"
/score="-32.15457980382507"
/phase="0"
/translation="MGISIRAYARHRGVTDTAVHKAIRAGRITPEADGTIDPEKADREW
ARNSGPPNTGTRTKVPKVAVPDAPGMGSEGPAALPTGGASLLQARTVNEVVKAQTNKVR
LARLKGELVDRNQAIAHVFKLARAERDAWLNWPARISAQMAARLGVDPHTMHVALEAAV
REHLQELGELRPRVD"
CDS 19607..21577
/ID="YLSDQRHL_CDS_0032"
/transl_table=11
/phrog="15"
/top_hit="No_MMseqs_PHROG_hit"
/locus_tag="YLSDQRHL_CDS_0032"
/function="head and packaging"
/product="terminase large subunit"
/source="PHANOTATE_1.5.1"
/score="-640999.0411576249"
/phase="0"
/translation="MDFEYEGAAEIERAWREGLTPDPLLTVSEWSDRHRVLSSKASAEP
GRWRTSRTPYLKAIMDCLSPTSPVERVVFMKAAQLGATEMGSNWIGYVIHHAPGPMMAV
WPTVEMARRNSKQRIDPLIEESPALLELIAPARSRDSGNTILAKEFRGGVLVMTGANSA
VGLRSMPVRYLFLDEVDGYPLDVEGEGDAISLAEARTRTFARRKIFIVSTPTISGASAI
EREYEASDQRRYFVPCPHCSHRQWLRFEQLRWDKGQPETAAYVCESCDTAIAEHHKTWM
LEHGEWRAMAPENGARTAGFHLSSLYSPVGWRSWRDIAAAWENAVSKESGSAAAIKTFK
NTELGETWVEEGETPDWQRLIERREDYRIGSVPRGGLLLVGGADVQKDRIEVSIWAFGR
GKESWLVEHRVLMGDTARDTVWKRLGELIADTWTHESGAAMPLARFALDTGFATQEAYA
FVRACRDPRVMAVKGVPRGAALIGTPTAVDVSQAGKKLRRGIKVFAVAVGIAKLELYNN
LRKSALVAEDGTTVAYPAGFVHLPKIDAEFIQQLCAEQLITRRDRNGFPVREWQKMRER
NESLDCYVYARAAAAAAGLDRFEERHWRELERQLGMERPPDEPSPPQPLNLDEATHSGG
LVVSDIRKTGRRVIKSRWLSR"
CDS 21585..21791
/ID="YLSDQRHL_CDS_0033"
/transl_table=11
/phrog="2545"
/top_hit="No_MMseqs_PHROG_hit"
/locus_tag="YLSDQRHL_CDS_0033"
/function="connector"
/product="head-tail adaptor Ad1"
/source="PHANOTATE_1.5.1"
/score="-3.2822859871484904"
/phase="0"
/translation="MTYTTTQLDALKRALGSGERRVTFGDKTVEYRSVEELQAAIRTVE
AELARNAGESPTRQIRVTTSKGF"
CDS 21791..23299
/ID="YLSDQRHL_CDS_0034"
/transl_table=11
/phrog="21"
/top_hit="No_MMseqs_PHROG_hit"
/locus_tag="YLSDQRHL_CDS_0034"
/function="head and packaging"
/product="portal protein"

/source="PHANOTATE_1.5.1"
/score="-17715.844688020476"
/phase="0"
/translation="MAWFSRIRSLFGQPPVHEAAGRGRRSLAWMPGNPGAVAAMLATSA
ELRIKSRDLVRRNAWAQAAIEAFVANAVGTGIKPQSLSPEERFKAEVQALWRDWTEEAD
AAGQTDFYGLQALACRAMLEGGECLIRLRPRRLEDGLVVPLQLQLLEPEHLPISLNTDL
PSGNVVRSGIEFDSLGRRLAYHLYRSHPEDGRLAPMSGQGGMDTVRVDAREIIHLYRVL
RPGQIRGEPWLSRALVKLNELDQYDDAELVRKKTAAMFAGFVTRQNPEDNLMGEGAADG
DGIALAGLEPGTLQILEPGEDIKFSDPADVGGSYSEFLRTQFRAVAAAIGITYEQLTGD
LTGVNYSSIRAGLLEFRRRCEMVQHSVLVHQLCRPVWAAWMRQAVLAGVLEAPGFARGG
PARRRQYLQVKWIPQGWQWVDPEKEFKALLLAIRAGLMSRSEAISAFGYDAEDVDREIA
ADNRRADDLGLVFDSDPRRTSKDGGSATANAAADAPQTGSSSTV"

CDS 23309..24535
/ID="YLSDQRHL_CDS_0035"
/transl_table=11
/phrog="53"
/top_hit="No_MMseqs_PHROG_hit"
/locus_tag="YLSDQRHL_CDS_0035"
/function="head and packaging"
/product="head maturation protease"
/source="PHANOTATE_1.5.1"
/score="-707.8806524487096"
/phase="0"
/translation="MTLLPHLAARLFGVPLAIHRPKLDVILAVLGPRIGVADLAAPPGF
APPMRAAPAATQRIAVIPVHGTLVRRTVGLEAESGLASYAGLAAQLDAAVASAQVDAIL
LDIDSPGGESGGVFDLADRIRAAAAVKPVWAVANDMAFSAAYALASAASKVFVSRTGGV
GSIGVIAMHVDQSAKDAKDGVRYTAVFAGERKNDLNPHEPISDEAHALLQAEVDRVYRL
FVDTVARHRGLDARAIEATEAGLFFGQDAVTAGLADAVGTFDDALSQLTESLSSSPMAV
AARGPFLNLQTESPMHDRTDPAAADRPAADPAGSPPQPATAAMAVADAIEIAQTCTLAG
RADLIAGFLEARTPPAKVRAQLLAAQAEASPEIVTRIAPDAAASAANNPLLEAAKQLAA
KSASLKKEI"

CDS 24537..24914
/ID="YLSDQRHL_CDS_0036"
/transl_table=11
/phrog="49"
/top_hit="No_MMseqs_PHROG_hit"
/locus_tag="YLSDQRHL_CDS_0036"
/function="head and packaging"
/product="head decoration"
/source="PHANOTATE_1.5.1"
/score="-10.870891991772778"
/phase="0"
/translation="MPVFVESLNLGDLLKYEAPNLYSRDRVTVAAGQNLPLGAVVGMIT
ATGKVKQIDPSATDGSQVAAGVLMQRCDAALAERDDGLMVARHAIVSDHALSWPTGITT
AEQQAAIAQLKALGILVRQGV"

CDS 24917..25933
/ID="YLSDQRHL_CDS_0037"
/transl_table=11
/phrog="29"
/top_hit="No_MMseqs_PHROG_hit"
/locus_tag="YLSDQRHL_CDS_0037"
/function="head and packaging"
/product="major head protein"

/source="PHANOTATE_1.5.1"
/score="-1827.2626359737635"
/phase="0"
/translation="MQNIFENPAFSMSALTAAINLLPNNYDRLGAMGLFVDKPQRFRSV
IVEEQNGVLTLLPTMPPGSPGTVGVRGKRKVRSFTIPHIPHDDVILPEEVQGIRAFGSE
TELQTVAGVMAQHLQTMRNKHAITLEHLRFGALKGQILDADGSVIYDLYNEFEITPKTF
TFDIADPGNGFDVKKACLDVIRYVEDNLQGERMTGLHAFVGEDFFDALTGHDEVKAAYD
RWQDGQALRTDMRAGFTFAGITFEEHRGRAVAPGNAVRRFIEPDEGHAFPLGTMDTFAT
YYAPADFNETANTVALPLYAKQEPRKFDRGTDLHTQSNPLPLCHRPALLVKLVMGGV"
CDS 25930..26232
/ID="YLSDQRHL_CDS_0038"
/transl_table=11
/phrog="10473"
/top_hit="No_MMseqs_PHROG_hit"
/locus_tag="YLSDQRHL_CDS_0038"
/function="unknown function"
/product="hypothetical protein"
/source="PHANOTATE_1.5.1"
/score="-7.584756686641125"
/phase="0"
/translation="MSLVEQIYDAAAHAGLLKECLWRPADGSPPQSHPVGFAAPDGTVL
DGLALSTEYAISYPASVFTGLASREAVEIDGATFLVRDIRAVGDGSEMRATLTRA"
CDS 26236..26682
/ID="YLSDQRHL_CDS_0039"
/transl_table=11
/phrog="4204"
/top_hit="No_MMseqs_PHROG_hit"
/locus_tag="YLSDQRHL_CDS_0039"
/function="connector"
/product="tail terminator"
/source="PHANOTATE_1.5.1"
/score="-14.544403444455092"
/phase="0"
/translation="MADNSIRERILLAVAAAVRPAAEAFGAGLHRSPAVAISREQCPAL
VVFPEIDAITERANDRVTRELTVRLVALTRAVPPAVPETAADRLLTAAHAALMTDGTLG
GLALGLREVDAEWEIEDADAVAVALPARYRITYRTLANDLSTLG"
CDS 26689..26889
/ID="YLSDQRHL_CDS_0040"
/transl_table=11
/phrog="3129"
/top_hit="No_MMseqs_PHROG_hit"
/locus_tag="YLSDQRHL_CDS_0040"
/function="unknown function"
/product="hypothetical protein"
/source="PHANOTATE_1.5.1"
/score="-2.3850624352640306"
/phase="0"
/translation="MTALVLTRSHTHAGKPYAPGDRIEVDVATADWLIAHDIAKPGPAA
PVANPDTTPEPKPLQRKEPKP"
CDS 26886..27638
/ID="YLSDQRHL_CDS_0041"
/transl_table=11
/phrog="807"

/top_hit="No_MMseqs_PHROG_hit"
/locus_tag="YLSDQRHL_CDS_0041"
/function="tail"
/product="major tail protein with Ig-like domain"
/source="PHANOTATE_1.5.1"
/score="-532.6933060844572"
/phase="0"
/translation="MSTYASFQGRVFLGKRDIAGLPIEVRSPGNVAELKLSLKTDVLEH
YESQTGQRSLDHRMVKQKSATVNLTIEEFTKENLALALYGNHVTGSGGTVTGEPVGGAA
PVVGDRYFLAHPKVSSLVVMDSAGTPATLTLGTHYTADTDFGALQFLEITGFTPPFKAS
YAYGVATEIGIFTQALPERYLRLEGVNTAQGNAKVLVELYRVAFDPLKEISFISDEYNK
FELEGSLLADSTKPFDALLGQFGRIVQL"
CDS 27652..28047
/ID="YLSDQRHL_CDS_0042"
/transl_table=11
/phrog="1442"
/top_hit="No_MMseqs_PHROG_hit"
/locus_tag="YLSDQRHL_CDS_0042"
/function="tail"
/product="tail assembly chaperone"
/source="PHANOTATE_1.5.1"
/score="-31.814846287395184"
/phase="0"
/translation="MSDLETLIPQSVELVIDGEPLAIKPLKVGQMPGFLRAITPVMQHL
TGGEIDWLALFSERGDDLLSAIAIAVGKPRAWVDELAADEAILLAAKVLEVNADFFTRT
VIPRIDGLFARVTAVQAKAAGSTPSNT"
CDS 28144..28872
/ID="YLSDQRHL_CDS_0043"
/transl_table=11
/phrog="6367"
/top_hit="No_MMseqs_PHROG_hit"
/locus_tag="YLSDQRHL_CDS_0043"
/function="unknown function"
/product="hypothetical protein"
/source="PHANOTATE_1.5.1"
/score="-206.7215842982257"
/phase="0"
/translation="MRGSCRSLRLVRAVSPASSTRPSTPSPTVRISVRIDSAAAQAQLR
RWGGEFRDKVKKAVARAIASEATELKQDVRGHVAGQMAVIKKSFLKGFTAKVLDKDPNR
LPALYVGSRIPWSGMHERGGLIAGRLLIPLHGRVGRKRFKAQIAELMRGGNAYFIKNAK
GNIVLMAENIKEHDRVLSGFKRRYRKAEGIKRLKRGADIPIAVLVPKVVLKKRLDVERL
VGGRIPRLSAAIERQIRTVD"
CDS 28890..32921
/ID="YLSDQRHL_CDS_0044"
/transl_table=11
/phrog="8010"
/top_hit="No_MMseqs_PHROG_hit"
/locus_tag="YLSDQRHL_CDS_0044"
/function="tail"
/product="tail length tape measure protein"
/source="PHANOTATE_1.5.1"
/score="-7036422917989.86"
/phase="0"

/translation="MANRISVLVALEGADEGLKRAIASAERSLGELSSTAKTAGEKAAA
GMAEVKAGMSAFGDQVANARTQLLAFLSINWAAGKVQEIVQIADAWNMMSARLKLATAG
QREYAVAQKELFDIAQRSGLAVQETATLYGKLQQAVRMLGGEQKDALTITESISQALRL
SGASAEESRSALLQFGQALASGVLRGEEFNSVVENSPRLAQALADGLNVPIGRLRKLAE
EGQLTADVVVNALLSQKDKLASEYAQLPQTVSQSFERLRNAFGQWINKLDESTGFTKKL
AEALTWLAQNLDTVMQWLKRIAEVGLAVLIYRLIPALITAWQTAGAAAVAAASATSAAW
ATANLSVSDAIAIVGKLRAAFAVLGAFLVGWEIGTWLSERFEIVRKAGIFMVEVLVKGV
EQLRYRWEVFAAVFTSDTIAEATKRHEARLAEMNRIFAEMYADATKGADAAKGAMNTAA
SAAEEIAKRLEAVRQGTQEAVGRGIEAVHAALEKLKSRLGEVEQAVGKANQTVNDATAK
MAEAYKGLTAVVEANLQRQIEAVKARYEQEKSTLELSKQSEAALITKSTQLLTEALTQQ
TTLRRQATTDALKLIDDESRAKIEAARRDGQTEAERAANVTRVENEILATKRQTLAQAL
SEYRQHIDALNAEANRHLAEIKRIEEEKRQLSMTTEERIRDIRRQGMTEFEATEDRKRQ
IAEYQEKAREALANGEFEQARQLAQKAMDLAAQVASSQTSEAKRGEEARKQSEQAVSQV
TQLEAQAREATRRQEYAQAEALMRQADQLRAELAQKAKDADAQIAQGKDGVNQAIQRIR
DSEEILNRALDAEAKAHQTAAQSALTARDQIKETLTQTETQIDQITAKLKDGLKVTIDA
DTARFDKAIADLDKALAEKEVLLKIQADLQEAEKKLQQYEQLLKEGKTLPVDADVSKAK
DALARLKTYADQNSQLELKVATEKAQAAITNVEGMIKALDRIQTESRHQISTNADAARA
EIMSLNGANTSSTHTIYVQKVEVNAAGGLVGGGVRRFGDGGAVAPAFPRMTGGTVPGSG
HHDTVPRTLEAGAFVIRKAAVRKYGSGALARLANGVAHFAVGGRVASLGNPGSTSTDPD
GKPSTPKRNREAVEALKMIDLGLQGMDEYTGWLEWNYGASVSLDMRRKTMESYGKQAQE
DRRTLEGFIDRKTLTGNERQTLERIKQTWRQAMAQPLLWGKDLERELIDYMEQHQGEFY
RRGGLSKSDTVPAMLTPGEFVVNKEAVARYGAGFFEAINNLSAPAQALAQRVQGFAAGG
LVPPAPSRITRPVLPSEGATTRSVRVELAAGDRKVDARIDARDEARLLQLLDAARTRTA
"

CDS 32932..33339
/ID="YLSDQRHL_CDS_0045"
/transl_table=11
/phrog="4408"
/top_hit="No_MMseqs_PHROG_hit"
/locus_tag="YLSDQRHL_CDS_0045"
/function="unknown function"
/product="hypothetical protein"
/source="PHANOTATE_1.5.1"
/score="-12.283895070615785"
/phase="0"
/translation="MQLKNLADEVAVLLPDDLLWTDEHAWSPAVASTSYLITGALLIQS
ALRQAGRPITLMGAPDMAWVTRATVEQLRAWAAIPVSAASGRFALTFADGRAFTVAFRH
GDTPIESEPVLGIPARSGGDFYRLTLRFLEI"

CDS 33342..36926
/ID="YLSDQRHL_CDS_0046"
/transl_table=11
/phrog="6128"
/top_hit="No_MMseqs_PHROG_hit"
/locus_tag="YLSDQRHL_CDS_0046"
/function="unknown function"
/product="hypothetical protein"
/source="PHANOTATE_1.5.1"
/score="-3151901559.2255316"
/phase="0"
/translation="MPIQTGDVKLLKSAVMADVPEGGGAPTGNTIADGVSNAIFPDISE
LDRAGGRVNLRKTFVSVQTDDTDTYFGANVIVAEPPKDARVSVTLFSTERTFDNREQAQ
LRIEAYLNKGPEWAGYLFENHIAGQRVIQLFQRPTDTVPNVGQTLVLVESEGQATQKEQ
YVRATAVSVVERTFTYDGDKDYKAAVVTVDISDALRYDFTGSPASRTFTRAANGTKVRD

TVVADAGTYVGVVPLTQAAAVGDFTIKGASIYTQLVPSAQTETPISFVPPYAAAGLPVP
GASSVSYTANHAWTPGLRFNLPGGCLPGSLTLQTDGITIFDDAGLLKTASGTIGTIDYA
NGILALNAGSMSNAKAVTYTPAAQILRAPQSSEIPVTPESRSQSYVGTVTPVPQPGTLS
ISYMAQGRWYVLSDAGNGTLKGLDASYGAGTVNRNTGAFVVTLGALPDVGSSLILTWNV
PTQETQQPQVTLKASQTLVLSPPEGKAVQPGSLTVSWEYTGTKTATAATNGVLSGAATG
VLRIASNRLEFAPNVLPSVGTQLTVSYVAGPKQEDAFAHPSRNGSGLVPVTATLGSIEP
GSLEVEWNTLTDTSVLGAYTLAQLLEMGVQAAWRDPTQIARDDGNGHVVLNGSSIGTVD
YATGQVTFNPDVTVLIPRPLYTSTAINGTGRWRLNYRGLAYVEAPSLYPNDESGYVKLR
YNSAGSTSSVTETFQFTPSFKLVPGVNAQIVPGTVVLTLPGAQPWGDNGQGTLREFTSS
GWVTRGTINYLSGDVTLTSWTAGTTNAFTRASCVTTVGENISSEYVFRTGAAPLRPGSL
SIQYARAVGGTQTVTAGIDGKIQATGITGSVDYENGLVRVRFGSFVTAAGNESEPWYSA
DRVGSDGKIFRPEPVAASTVRYSAVAYSYLPLDADLLGIDPVRLPSDGRVPIFRAGGFA
VVGHTGKITAPVANGQTINCGRVRLSRVRVVGHDGTVIHSGYTTDLEAGTVSFNNVTGY
SQPVTIEHRIEDMAVVRDVQINGEISFTRALTHEYPVATAGDPASGSYVSSALIAGDLF
ARVSLVFDQATWNGAWVDAMSGSAATATFNNTQYPIRVTNRGAVTERWVVRFTNSTAFE
VIGENVGVIATGNTSADCAPNNPATGVPYFHLPALGWGSGWATGNVLRFNTIGAQFPVW
VVRTVQQGPESVPNDNFTLLIRGDVDTP"

CDS 36836..38218
/ID="YLSDQRHL_CDS_0047"
/transl_table=11
/phrog="9998"
/top_hit="No_MMseqs_PHROG_hit"
/locus_tag="YLSDQRHL_CDS_0047"
/function="unknown function"
/product="hypothetical protein"
/source="PHANOTATE_1.5.1"
/score="-5824.674660898145"
/phase="0"
/translation="MGGAYGATGTGIGAQRQLHVADPRRRGHPLTPQTGTNDMPDLSVK
YFNSGMAGAPQISNNWGDLVSMLDACLVNGFALKAIDTLTFANGIATATITSGHAYQRD
QVVQVAGAEQPEYNGQFRVLTTTATTFTYAVTGTPASPATTATSLSAKVAPLGWEKPFA
GTNKAAYRSKNPASPQNLLLIDDSLKTPGYTTSWAKWANVGIVEDLADIDTIVGAQAPY
DPNNPTQNWKQVTANQWGWHKWYHARQTGYDTYGDSGGGNRNWVLVGDDRLFFLFCTFA
PGFNWYGRSCYCFGDITSFKPGDNYATVLAAHDLYWSNNNQYMSYPGEYGGASLIASLD
FSGHVLLRNHTQLGNPVRWAATSLNTNNGQQICGRGPMPFPNGADYSLWLLPTYVRQED
GHMRGLMPGMYWMPQDRPYSDQTIVDNVVGQTGKRFLLVRTQYSSETEGAQVAFDITGP
WR"

CDS 38291..39307
/ID="YLSDQRHL_CDS_0048"
/transl_table=11
/phrog="27013"
/top_hit="No_MMseqs_PHROG_hit"
/locus_tag="YLSDQRHL_CDS_0048"
/function="unknown function"
/product="hypothetical protein"
/source="PHANOTATE_1.5.1"
/score="-1410.063247565824"
/phase="0"
/translation="MSASHNAAQQALDLSASSTQSILRFNEAANGDFWFEADIELLTDP
SGRKHVGLWMTTGNAAEGYRFAHLDSAWSVSRWSSGFGDGSAVTGSVNDGARPMAGIAA
TAPTFNVGQRRVLRCEVITGAPDANGVPWSRLLQFSAGGVVLFQVADATYRGKLVPGVF
LYGATARVHAIAGDTPSGLPAFPATVAVNAADDLLPLAGGPTSVLPDPASPIGVAADCD
LMRRNSPASDLWNRPGGYDHDFHPIQSGRKDIHFSGHGVIAGTVKEKGQPDQPLVRRVQ

IISENANVLVAETWSDAAGNYRFEFIDPAQRYTVVSYDYKHLYRAVIADNLKPELLP"
CDS 39304..39699
/ID="YLSDQRHL_CDS_0049"
/transl_table=11
/phrog="3758"
/top_hit="No_MMseqs_PHROG_hit"
/locus_tag="YLSDQRHL_CDS_0049"
/function="head and packaging"
/product="virion structural protein"
/source="PHANOTATE_1.5.1"
/score="-12.368107163464396"
/phase="0"
/translation="MTVAITVEHNEARLAGTLAFLDAGPEPARLRIYGGTRPPTPATVP
SSEMLVEIRLTKPAGTISGGLLTLTQQEDGLITATGVATWARLVNGNDVTALDLDCSGT
DGNGDVKLASTALYLGGDARMVSAILG"
CDS 39668..39811
/ID="YLSDQRHL_CDS_0050"
/transl_table=11
/phrog="No_PHROGs_HMM"
/top_hit="No_MMseqs_PHROG_hit"
/locus_tag="YLSDQRHL_CDS_0050"
/function="unknown function"
/product="hypothetical protein"
/source="PHANOTATE_1.5.1"
/score="-1.2734664749224616"
/phase="0"
/translation="MPGWCRRSWGRPWQPPSLSRPTCCSTDPPPPMRICCSVPTSPRHA
TI"
CDS 40000..41661
/ID="YLSDQRHL_CDS_0051"
/transl_table=11
/phrog="6079"
/top_hit="No_MMseqs_PHROG_hit"
/locus_tag="YLSDQRHL_CDS_0051"
/function="tail"
/product="tail protein"
/source="PHANOTATE_1.5.1"
/score="-19021.49808109556"
/phase="0"
/translation="MQRPTVGEIRHPWQGTRATEEVATQPQQHAHATPAGWSGLWEGAS
GAPQGIAHRLPHVLQTAHQQRRAGHQDASRLQDATWFAHQDGSPLGLVRFAAFERATGV
RRATWFRHQDGSVTCRAGRASGWQDARVLVQHQGSDHQSATPCPKGWRGRYQNTRRPPP
GISLLVIPRPPQPRPCYTPSPHLLFAGLAVSQGDLLFVCENHIDPPPPDGEPVVVPVRR
VYFVVNNVTLHRLPDGVPVPVFNLSLSLDVASWAWGFEAQLPAKAESLVAPGNASGPVE
LVASINGTEFRVFAENISRERIFGEASIRVSGRGHNAVLAAPYAPVMTFRNAEARTARQ
LMDDVLTLNGIPLGWTVDWGLTDWNVPAGVFARQGTWIEALTAIASAAGGYLVPHPSDQ
RIRVRHRYPVAPWEWHTVTPDFVLPVDAVARESLRWIEKPAYNRVFVSGQDVGVLGQVT
RAGTAGDVLAPMVVDALITEAAAARQRGLAVLADTGHQIEVSLRLPVLAETGIIEPGAF
VEYQDGSVTRLGLVRSTQIEAGIPEVWQTLGVQGHA"
CDS 41654..41875
/ID="YLSDQRHL_CDS_0052"
/transl_table=11
/phrog="5076"

/top_hit="No_MMseqs_PHROG_hit"
/locus_tag="YLSDQRHL_CDS_0052"
/function="unknown function"
/product="hypothetical protein"
/source="PHANOTATE_1.5.1"
/score="-7.044953074787324"
/phase="0"
/translation="MHNLYVQFRQLIPDPPLQAGTVVEIGSGVVTVQLPGGGRIKARGT
GAIGQNVFVRDDTVEGIAPTLTLELIEI"
CDS 41962..42264
/ID="YLSDQRHL_CDS_0053"
/transl_table=11
/phrog="10540"
/top_hit="No_MMseqs_PHROG_hit"
/locus_tag="YLSDQRHL_CDS_0053"
/function="unknown function"
/product="hypothetical protein"
/source="PHANOTATE_1.5.1"
/score="-7.579466380295733"
/phase="0"
/translation="MTEPDNKPAHVENMLLLRKEDFDELLDRAAQRGAERCLAHLGLEN
GSAARDIRELRDLLEAWRDARRTAWQTVVKVVTTGILAALLVGAAIKLKLMGGSQ"
CDS 42261..42737
/ID="YLSDQRHL_CDS_0054"
/transl_table=11
/phrog="6637"
/top_hit="No_MMseqs_PHROG_hit"
/locus_tag="YLSDQRHL_CDS_0054"
/function="unknown function"
/product="hypothetical protein"
/source="PHANOTATE_1.5.1"
/score="-29.90651834795351"
/phase="0"
/translation="MIETLLGGFLGGAFRLAPEILKWLDRKGERGHELAMQDKALEFEK
LRGAQRMAEIGASADAAWNSGAIEALRDAVRTQGEKTGVRWADALSSSVRPVITYWFMA
LYCAAKTAAFAAAVTAGAGWGVAILHAWTEADQALWAGVLNFWFLGRVFDRGRP"
CDS 42712..42930
/ID="YLSDQRHL_CDS_0055"
/transl_table=11
/phrog="No_PHROGs_HMM"
/top_hit="No_MMseqs_PHROG_hit"
/locus_tag="YLSDQRHL_CDS_0055"
/function="unknown function"
/product="hypothetical protein"
/source="PHANOTATE_1.5.1"
/score="-0.1414111633182485"
/phase="0"
/translation="VCSTGGGRDRGAADSHRPGQALRGLPPRAEDRSGPCASVHLPGRL
LDDRVRTSLRLDACANHRGRSGSLSGP"
CDS 42938..43192
/ID="YLSDQRHL_CDS_0056"
/transl_table=11
/phrog="7"

/top_hit="No_MMseqs_PHROG_hit"
/locus_tag="YLSDQRHL_CDS_0056"
/function="lysis"
/product="endolysin"
/source="PHANOTATE_1.5.1"
/score="-5.387959355141708"
/phase="0"
/translation="MALAATLRYCPVLATESEGRLAAIVDFTFNLGAGRLQTSTLRRRV
NQRDWMAAGDEIQRWAYGGGKVLPGLVLRRKAERVLLGG"
CDS complement(43193..44497)
/ID="YLSDQRHL_CDS_0057"
/transl_table=11
/phrog="No_PHROGs_HMM"
/top_hit="No_MMseqs_PHROG_hit"
/locus_tag="YLSDQRHL_CDS_0057"
/function="unknown function"
/product="hypothetical protein"
/source="PHANOTATE_1.5.1"
/score="-6761454.887564009"
/phase="0"
/translation="MLDEYSIEQRATQIFDARTKEYFSEVLSCYSAGNYRSAVVMLWSV
AICDLLFKLQNLVDLHGDAKAKEILEDVGRLQQENERSPIWETKLVESVAEQTQLLDIA
ERENLLHLQRQRHLAAHPVVNANFQLHRPNRETARALIRNTLDGVLTKAPILSKQIVNE
LVEDLEQASGILIDDKKLKAYLESKYYNRFNPEVEKAVFKALWKFAFRLTDERCEKNRA
INYSALRLLYSRNPAQFRAKIEAEKDYFSTIATGGAPVVCLIHFLSREASIYGLLADHA
KTAIQHAAESDPSARCLAWFTATNLQAHADRLGEWISGKDYPHIDCAIWDEIEELSDSP
EWAKLVVRLFNKYYVASGSYNTADRRFSEVIRPNLDRYELDDCIDLVEGIQGNDQTYGR
GRARTDHQALRARILALDPNFDFTAYKTFVRYLDD"
CDS complement(44497..47025)
/ID="YLSDQRHL_CDS_0058"
/transl_table=11
/phrog="23807"
/top_hit="No_MMseqs_PHROG_hit"
/locus_tag="YLSDQRHL_CDS_0058"
/function="unknown function"
/product="hypothetical protein"
/source="PHANOTATE_1.5.1"
/score="-10620097843.316216"
/phase="0"
/translation="MESASALTSAKIDLLPHQVVLTHRVATASPRRYLIADEVGLGKTI
ETALILRELASRGELNRALMVVPAGLVNNWHRELNEVFNLDFEVFGSEGDITDRKTNAF
AKHDRLIASIDTLKRPARIKRLLDAPRWDLVVFDEAHHLTACRNGGKVRKTENYKLAEA
LKDHARDFLLLSATPHQGNHFQFWMLVQLLNPTLFRSPEEMLENRHRLNTVMFRRTKAD
ACQPDGSPLFARRWVHTESFVMNTGERLFYERLREYLEDGFDLARRQGSQGRALGFLMA
IFQKIAASSFAAVRRTLKRRLLMLTIHEALLRDKELDIEGRERLMDEARDLIHQEFQLP
RDSIGRSEVDRVLADLKYRLVKRLDEEALDLASDPYGSEYSATHAEDVASAVVEMHLPE
ERLRIGDLLKIFPVQRETKAQKLLDGLGYLWRQNPNEKVVVFATYLGTVDLIAREIEQA
YPGQGVVVLRGGDHGAKVAAERRFRQKDGPRVLVCTAAGREGINLQFARILFNFDLPWN
PMDVEQRIGRIHRYGQNHTAQVYNLVLSDTIEGRIFLLLDEKLTEIARTVGKVDDQGNV
AEDLRAQILGQLSERLNYDRLYQEALSDPELKRTRVELEAALSNSREARQVVFDLFQDL
EGFSLDDYKPFSDVSSSLDRLVRFLSAAAAERQQRLIKVDDETYDLVTVDGVRRARFTL
NRDTATGQDDVELLGLDHPLVQEELGRWRIVRPEDVGIAVTGDVDGPVLLSLWMVETSA
GNGERRIVVQPIAVKQDGTRVPAVERQAERYLQAPATSPRFTPEQRLDLFARAVEPTLQ

RELKHKGAVKGDGSYSAELIGYVEIVEQGA"
CDS complement(47004..47210)
/ID="YLSDQRHL_CDS_0059"
/transl_table=11
/phrog="No_PHROGs_HMM"
/top_hit="No_MMseqs_PHROG_hit"
/locus_tag="YLSDQRHL_CDS_0059"
/function="unknown function"
/product="hypothetical protein"
/source="PHANOTATE_1.5.1"
/score="-0.0770305237206217"
/phase="0"
/translation="LFLTLRATAICAHSSASVSAVFRLAPSGVSSRVPSASCVPSTEGL
TERAARGCPTKRTHCQLWRAPRR"
CDS complement(47284..47784)
/ID="YLSDQRHL_CDS_0060"
/transl_table=11
/phrog="No_PHROGs_HMM"
/top_hit="No_MMseqs_PHROG_hit"
/locus_tag="YLSDQRHL_CDS_0060"
/function="unknown function"
/product="hypothetical protein"
/source="PHANOTATE_1.5.1"
/score="-59.96763885067634"
/phase="0"
/translation="MRQTLDTGWRRRGTSWIWDEEARNIVCTAPEVWSLRQFLQAVGHW
SDDLPSNNGDTLVVAGLDGSLDLLSPKDAEVWLADVIKPAVLSFQSYYEGQAALSFWMP
SARERMQTNPATDAVTWLCEAPHRGSQLDFGRILWGEANEYPQEILLREGAKPAGLFHL
RIT"
CDS complement(47781..51113)
/ID="YLSDQRHL_CDS_0061"
/transl_table=11
/phrog="10183"
/top_hit="No_MMseqs_PHROG_hit"
/locus_tag="YLSDQRHL_CDS_0061"
/function="other"
/product="DNA methyltransferase"
/source="PHANOTATE_1.5.1"
/score="-9487139941510.426"
/phase="0"
/translation="MEVAQPGRLSNTDARRSFAARGAVALRRSHEIKGQKMNFWEQKQD
AKAQLREQVGKAVGAGKAVALESVDFSDPGRPKTCLEVDFPILPVNQVAVIEGNAGKPI
YQMSKWWARRRSSVFRSMLIAAAAKAPDDPAHAARLVWDNYYANHQKKGSFKHLKVADI
FMGGGTTLVEGSRLGMQMVGNDLNPVAWFVVKQELANVDLGEVKKLLADVEAEVKPQIM
PYYYCDGPEGEKGTWTHLPTKKVMPADFDPLVIPRDERKDYRYEGPEIIYTFWAKHGPC
QVTGCGHRTPIMSSPVMAVKTLSVKHWEHTCGKCGGEFHVEEEAARMAPDVPLYVAPSE
YPFSVLDRKKGVICPHCGHTALVNLGKGKNKKVELSLLVHPQWLAGEAKQDVSGQPYGG
SAQDDAASTARWNHARAAKIRLLEVRGALPDQVTCPETGVTFRTDKGTVPKKSNYACGA
CGTAQDVLTTVKASGKTGPMAAYAVQGYAPKRDEARKPYNGRFFAPLDERLARQYDAAS
EEWEARKDTDLKDYWPRSAVPYGFMTGIANGDIREGHGFTHWWTMFNPRQLLVHAQLLK
AIVEVGNYDWTVREYVLGAFQQYLRNQCLFSFWNPQRDTPEPMFSNNNYHPKSTVVENC
VFPALGRGNWASSVEGILEGRDWAIDPWEAVSAEALRRKDNALAGEISGKSEKVFPGDP
VGDVTVFQGSSTDLARIEAGSLDLVITDPPFGGLLHYSELADFFYVWLRLVLKGKYPEY

FSADYTPKSLEAVANKAREPEDPDGFYQRLLTQCWREAHRILKPGGILAFTFHHSEDEP
WVAVLESLFDAGFYLEATYPIRSDETKGEGEFGSKTIEYDIIHVCRKRTEEPKPVSWGR
MRREVMADVRQLQAMLENHAKEGLPAADIQVIRRGKALEYFSRHYGKVYVDEGRTITVK
DALVGINQLIDEDADKGKEPPPVNAEPITRQFLRTFGAAGELKRDQLQKFLRGTITTPD
DFVQRGWCAEKNKVFTRTNPLDFAREWSGKHRRRLTSDLDQALVLIGACFDGSGINASD
TLKNENFKPHVALKPLLEWLHRNGPEQATRNAASRAVSIYNTWHASQAVKPTQGSLFEE
YEL"
CDS complement(51017..52006)
/ID="YLSDQRHL_CDS_0062"
/transl_table=11
/phrog="No_PHROGs_HMM"
/top_hit="No_MMseqs_PHROG_hit"
/locus_tag="YLSDQRHL_CDS_0062"
/function="unknown function"
/product="hypothetical protein"
/source="PHANOTATE_1.5.1"
/score="-7609.760088945123"
/phase="0"
/translation="MGLDANILNSLTKESAAEAWRVIIDSAVEIASKPLAAGNAPSEVV
KRDRAIGALDHFLATSGWDLWSSFGDVVERTSDRPVRWWKEPYSAKAVLILDGLSLREL
PWLMQGAKERGFALHEVGANASELPGETNEFARALGFTSRSQLQNNGGGLAHKLQPAHT
ECVDMPWKDCEGLINSTPNWVFWHHWPDSKVHDGAGAGQGLEILTRDAAQQLSSDDFWA
FVERLATGRRLVITSDHGYAASGHFPDADGEVGQFLKKTFSSGRSASGSGDTGPFVPPV
ALQINSPHGAHLMALGRWKWRSQGGYPTLTHGGLSLLEVLSPFVEVTK"
CDS complement(52006..54747)
/ID="YLSDQRHL_CDS_0063"
/transl_table=11
/phrog="38717"
/top_hit="No_MMseqs_PHROG_hit"
/locus_tag="YLSDQRHL_CDS_0063"
/function="unknown function"
/product="hypothetical protein"
/source="PHANOTATE_1.5.1"
/score="-43404039954.57986"
/phase="0"
/translation="MLGLTLREEFRGKRLKGTAIELSNDTSTGATQIAAKQFLEITYPT
HDLLKGIEAVGPNQGRPVVVIGERGLGKSHLMAALYHAVNDAASTGAWLNAWAGTLGDA
NIGKIALRSGMYVIGESLHRQRYKFLWDLLFERHPHGTYIKGKWEGMGAAKTDIPSDKL
IVEMLEHRPTMLLLDEFQTWYDGLTNTKQYPWKNWAFNFIQILSEIAKERPDLLVLVIS
VRNGGSDAYQQVHRVNPVAIDFKAGGNAERIQQDRRRMLLHRLFDNRMQIANGTIESLI
AQHVSEYFRLLDVPPAEHDRKRREFVESWPFAPHLLRLLEEQVLIATDAQETRDMIRIL
ANLYKSRGDAVPVLTAADFRLDDDASGIGALLDSVSNEHHRTLREKAQQNIISVTEAVY
DHATRAPHLQEIVGALWLRSIAVGNLAGAEPATLQVDITRDKPVDDNAFQVELATIVEN
SFNIHQDGPRLVFREEENPRAKLMACARNDKLFTDGSDQAQLAKQIRYVIGGSDEVAKS
FRVIALPKSWQTDPWSALDEAEQPERWDDRLPILVLPEEPEKLDQTLGRWLKDHLQKRR
NTVRFLLPRSGSANAFLDRDLLILARAEMKAQEWSGQNPEYKKLHKEFEGTLRDNLKKR
FDRFAVLHRYDHQNPHQCVFSVESLKKQGAQIPEAIEEALTNDLFVPEDFEALVLEAAA
DNAPLGKLLRELQEPRPAGQDCIPWLGETAMKERILRLCARGKIAINLRGLEYLQTHAG
EDEDAAWKRLRPKLFYTGRQLDEVFLMEPSAVPTTGGATPPAPPPAGGTGGGLFGGGPT
PPTPPTPGDDTTGSGGTPPPGGIFGGGTTTAGAKPRIPLSNPPTSPLNLIGKLEGWGIG
PATQVAEVSIKVSAATGAQLKELLKKLPDGMTFELSLEKEDS"
CDS complement(54747..54992)
/ID="YLSDQRHL_CDS_0064"

/transl_table=11
/phrog="66"
/top_hit="No_MMseqs_PHROG_hit"
/locus_tag="YLSDQRHL_CDS_0064"
/function="integration and excision"
/product="excisionase and transcriptional regulator"
/source="PHANOTATE_1.5.1"
/score="-12.13798313505216"
/phase="0"
/translation="MVPAARAAAGEFFNGLLKVTERTIYRLAAAKKIPAFKVGGTWRFS
LGDIDRWIKQQSREVLGTGPDEGGAATDQSHDGERK"
CDS 55030..56130
/ID="YLSDQRHL_CDS_0065"
/transl_table=11
/phrog="8928"
/top_hit="No_MMseqs_PHROG_hit"
/locus_tag="YLSDQRHL_CDS_0065"
/function="unknown function"
/product="hypothetical protein"
/source="PHANOTATE_1.5.1"
/score="-13250.274375470855"
/phase="0"
/translation="MRGTQNFQGAMFSYISLEERVPARHPLRKLRALVDALLASMSAEF
EAVYARRGRPSVPPEMLLKALLLQILFSIRSERLLVEAIDYNLLYRWFVGLNLEDKVWD
HSTFSANRQRLFNEDLARVFFERVKYTADWAKLIGDEHFSVDGTLIEAWASQKSFKRKD
ASGSDDGAPPQGRNPEVDFKGETRRNDTHASTTDADARLFKKAAGDKSRLCHMGHILMD
NRHGLVVDVEITHASGTAERQAALKMLQRQKRKAGRLTVGADKGYDCRAFVQGCRKLGI
TPHVAAKAKHSAIDGRTQRHEGYKVSLRVRKRIEEAFGWIKTVGGLAKTKLIGHAKLAG
QALMCFAAYNLVRMGSLGGWWDAHHA"
CDS 56154..56378
/ID="YLSDQRHL_CDS_0066"
/transl_table=11
/phrog="No_PHROGs_HMM"
/top_hit="No_MMseqs_PHROG_hit"
/locus_tag="YLSDQRHL_CDS_0066"
/function="unknown function"
/product="hypothetical protein"
/source="PHANOTATE_1.5.1"
/score="-3.0750570709909324"
/phase="0"
/translation="MGEQPPKGEPKRLSEPRKTACAGLGPTQTGTWPLRWTLFQRPVKY
SATCLMVRISPSRVLIGMVIAKLSQTATG"
CDS 56486..56677
/ID="YLSDQRHL_CDS_0067"
/transl_table=11
/phrog="No_PHROGs_HMM"
/top_hit="No_MMseqs_PHROG_hit"
/locus_tag="YLSDQRHL_CDS_0067"
/function="unknown function"
/product="hypothetical protein"
/source="PHANOTATE_1.5.1"
/score="-2.443303898726528"
/phase="0"

/translation="MAFVSGPAADSPLIRPQVAKPSHPRPQGRGVTIWIGKRTRVRFPF
SPPIPGIVKTIPDQLRPF"

ORIGIN

1 caccaatacc ccaaggccaa ccgttctcgg ttggcctttt ttctcgcccg caacgccaat
61 gttggcgcgg tttccgggcg gcgccttgcg gacgcgcacg ccgtccgagc gccgttcccg
121 ccccgacagg gcctctctgt tctcggtttt tctctgcggt ccgtggtagg aaccggaggc
181 cacctccttg cctgacaagg acttagagac cactggttgt agtcagaaac tgccttgcgg
241 cggcgactcg ggcgcgacga agcgcaaaaa agccgcccgc aggcggctgc ggcggcgggc
301 ggcgtgacgt cagtcaagca accgcacctg ctcgcgccag ccgtagggcc aggggcggcc
361 cagcaccttc tcgatggcga ggtcgggcga tccgagcagc cgctcgatca cctcgggcgc
421 gagcagcgtc agccgcagca ggcgatgcac cgtggataca tccagcccct cggcctgggc
481 gatctcggcc gccgacgcca cccgcccctc atggagcagc cgttgccagt ggtgcgccag
541 cccgagcgca cgcatcagcg ccgtgtcctt ggcctcgggg cgcggcgggc cggcccccgt
601 cgaatgcgcc tccaccgccc tcggcgcgcc gggcggcgcg atcaccgcgc ggcggatgcc
661 gcgccgcacc agccgccaag gcacgaaggt ttccagtttc acgccgcccg ccggcgtggg
721 cagtctctgg gtgaccggcg ctccctcgaa gcgtccccgg tgtttcgtgc tcacgctccc
781 tcctcgaagc gtcgcacgat ctcgcgctgg gccacccagt ccaccggcag cgggtggcgc
841 tggaaccaga tcaggctcat gcgccgcggc tggcgcccgg ccatcagcat ctcgacgatg
901 tcgggcgcga gcagtgtcag gcgcagcaac tcgttgacca ccgagtgatg cagcccctcg
961 gcacgcgcga tggccgagcc gctcggcatc gcgccgctgt ccaacaaccg ctgccagtgg
1021 aaggcccgtg ccatcccttc gatgagcgtg ctgtcgtgcg tgtcccggtc ctcggcctga
1081 tgctggacca gccggcgcac gccgcgccgc cgcagggtca gcggcacgaa ggtttcgcga
1141 actccgtctt tcatgcttcg acctccagca gctcggcgcc gatgctcctg ggggcgaact
1201 cgccgatcaa ggcgtcccag cccagttcgc gccacttcac ccggatgccc tgcatctcgt
1261 cggtgtggac gaggtcgatg cgctcgatca tcaggttgac gatgcggtgc tgctcgaccg
1321 ggaaaagctg atcccacacg tcgttgaggc gccccatcgc catcacgacc gtggcctcgt
1381 cgatcagtgc gccctggcgt tggacgtgac gcacgacggc ggcgacggat tcggggctgg
1441 tgagcacggt ccggatctgg gcgaccaccg ccgcttcgat ctcgccagcg ggcaggcgct
1501 cgtagctctt gccgggtgcg ccgaaccggc tctcggactt ggagacgtag tagcggtact
1561 tgcggccgtt cttgcgcgag taggtcgggt acatccgctc gcccgagggc gcgtacagca
1621 gtccgcgcag cagcgcgtcg ttgcgcgagc ggatcttggt gtccaccgac cgcgcgtggc
1681 tgtcgcgggc cagcaccgcg tgaacctggt cccacagctc gcgctcgatg atcggctcgt
1741 gcacgccggg gtaccactgg ccccggttcg acaactcccc gaggtagatg cggttgcgca
1801 gcaccttgtg gatgtacttc ttgtcgatgc gcgtgccggt ccgcacccgc ccgtcctggg
1861 tcgtccacgc cttggtcgtg atgccctcgg cggtcagccg cgcggcgatc tgcgtcggcg
1921 agccgatggt caacatctcc tcgaagatgc ggcgcaccac cgccgcctcg gcctggttga
1981 ccaccagctg gcggttgacc acgtcgtagc cgatggtggg cacgccgccc atccacagcc
2041 ccttcttctt ggcggcggcg atcttgtcgc ggatgcgctc gccggtgacc tcgcgctcga
2101 actgggcgaa ggacagcagg atgttgagcg tgagccgccc catcgaggtc gtggtgttga
2161 actgctgggt caccgagacg aaggacacgc cgtggcgctc gaacacctcg accatcttgg
2221 aaaagtccgg gaggctcctc gtgagacggt cgatcttgta gaccaccacg atgtcgatca
2281 gcccgcgctg gatatcgacc atcaggcgct tcagggccgg gcgctcggtg ttgccgcccg
2341 agaagccggg atcatcgtag tcgtcggcga cagagatcca cccctcgctc ctttggctgg
2401 cgatgaaggc gtggcccgct tccttctgcg cgtcgatgga gttgaactcc tggtcgagcc
2461 gctcgtccga ggacacccgg cagtagaccg cacagcgctt gcgtgccttg gtggaggcga
2521 tctccgtcat caggcacctc ccttcaggcc gaagaacagc ggcccgctcc agtgctgccc
2581 ggtgatgtgt ctggccacgg cggtgaggct cttgaagctg cggccctcgt actcgaagcg
2641 cccctcggcg gtgaccgtga cgcggtgctc gcgctcgccc cattcgcgca gcagcacggt
2701 gcccggcgcg aagttgaaga cgcgcggctt ggcgcgcagc ttgatcttgg agtgcttcgc
2761 gccgatggcc tccaggcgct ggcgcgtctc gggcgccaag ccgccgaaga cctcctcctg
2821 catcttgtag gcgatgcggg attcgacatg ggtgcggttc gggaactgcg ggcgccgctc
2881 gaagtaccga tcccagagca cccagagttc ggccatcggc aggtgactca gctcggcgat
2941 ccgcgccgcg acggaagctt gtttttcgtt catcacaact tctcctgttg agagggggtt

3001 gtatgaacgc gctggtcggg caggaagcca aggcgagccg cgctctgttc cagcccggaa
3061 gacccgaggg tacggacgat ggcggccgca aggatggtgg tgatttcgcc ggcgcgggcg
3121 ccggggctca tctccaaggg agatgcaagt tcgatgttct tcatgacggc tccagggaat
3181 gcaaccgtca gagatagtgg aactgatcct ccgaacagga tggcaacgca gggcaatggc
3241 cgccgggtgt cagcctgcca ttgccgcctc ttccgtctcg acctcggtgt cgtcatcgac
3301 cggggcgtcg agcgtcggca gattcagttg ccacgcatgc gcgcctttga ctttcaccag
3361 atagtcgcgc cagggcgagt ttttggagaa caggttggcg ggcgaagcgc agccggtgtc
3421 ctccatcagc ttcttggtgt tcacgtgggg tgtgcccgcc gtgtaggcgt caaccagccg
3481 ctgcagcacg gcgatcttgg ccttgttcgt caccttccaa ggcgccttgc ccggcaggta
3541 cagcgtggcc gagtaaccgt cagcggacac cttcaggctg accgtagtgc cacccatcgc
3601 cgcgagttgc ccgtgccggt aggccacctt cagccgcgtc gtgtcgatgg ccgagccgtc
3661 accgccatcg gacaggacat cctcgatggg gatcaccacg ttggttcccg cgaacggaaa
3721 cgggatcgag gcggtggtga gcacgatgcc cggcacggcg cgcgggcgca gacgcagtgc
3781 ggcgtcgacc ctggcgtact gccgttcact ggacatcttc gcagcgaagt acagcgcgac
3841 cgcgtgtcca tcgatgtcca tctccccgag gaacacgggt tcgtcgtcga ggtgcctgcc
3901 acgtacgccc ttcaaggccg acccgagcgc ggtgatgatc tcctcgcgca gccagttcag
3961 ttgcaccttc cagcgacgtg ccagcttggc gggcaggacg acgtccatcc ccgtcagcgg
4021 gtcgcggtag cgcacctgat tcaggtcggc gcaacgctcc agcttcaccg tgaagggtgc
4081 accatcgtcc aactggacga ccgtttccgt gatccgttcc ccctcggtga ggatgccctc
4141 gtcttcgagg cggtcgatgt cgatgcccag cttggccagt gcgaagccgt ccagcggact
4201 ggtcgcgcat tcgagcagcc tggccacctg ggcgatcagg ttcggatcgt ccacacccga
4261 gccggggtgc aagggcttgc gcacacccag tgcctcgagc agttgcatcc cgacacggcg
4321 caggcgctga tccttctcac cggacaggct gcaccgcccc ggctcggcaa gcacgatgga
4381 caggggcgtg acgctcgtct cgccctcgaa cacgaggtcg gcgaccagcg tcacgccgag
4441 caaggcgccg ggctgcgcga agggatggtt gccccagcgc tcgtcgatca cctcatgcag
4501 ctctgcaccg ctgtcgatgt gcagcgtgac ggcgtccgtg gcgtgaccaa gcagagcctt
4561 ggcctcgatc agataaagat gctcgacctt cgcgccgtcg atgcgaggct tctcgtcctt
4621 gagcggtcgc gcgaagcggg acaggtcata gcgggagcgg tccagcggcc ggttcgacaa
4681 cggcatcttg aaaccgtgct tggacagcac gttcgccaac ggcgcccggg tggagagcgt
4741 gtgcgcgtag acctcgacca tcttgcgatg gggcgcgtag atgagcgtgg cgtcacgtgc
4801 cgggaagtag cagaagctgc ggcgattgcg gttgatcacc tgcaccgccg tgacctgctc
4861 gccggcaaag cggaccacca agtagtggac ggtcttggtc tcgccgtccc tagcctcatc
4921 ggcgagcggg acatggatga cctcgcaggg ctccgccagg cgcatcgctg tcgtgagatg
4981 ggtttccagc tccttcttga cggagtcgct ccagatgaat gggggcgcgt cgtcacaagg
5041 cacatcgaat gcgtcgtgca gccgcttgtt gccgcgaagg tcgccggtgt tcaggatcga
5101 ctcggcaacg tcgaacagcc gcgcagactc atccgagtgc gtacgcatcc agactgagcg
5161 gccgatctcg cagccgtctt gcgacaggaa cagggcgatc agatcgctgt cgttgagctg
5221 ttcggcgacg gtggcgagga tggccgcacc gcgcggcgac gcgagacgca gcacccgcag
5281 cgcctcgcgc tcggccggat cacgctgttc cttgcggaga tgcttgatgc gttcgaccag
5341 ggccgccgga agcgcgccat catcccgcga ccagtcgaag ccgcgggcca gcgcttggca
5401 ttcgggcagg ccgctgaagg ctttgaggac acctgcctgc gccttctcgg tcagttccag
5461 caggcagtgc gagttggtca gggtcttctt gcccatgctt tctcccttgg ccgttcacat
5521 ggccacgatc cattggcgca tcaccgccac cgattgcgtc agcccctgcg ccagcagggt
5581 gtccaggtat tcgtccgccg tcttgggcgg gttcttcagt gatcgccggt ggctggctgc
5641 agcctccagc actcctgccg gatgcagatc cagcagatcg acgatgaagt cgtccggatg
5701 ctgggcggcg aggttgtagg gcctgagcgc ctcgggcggg aagtccttga gattgaaggt
5761 cacgatcaga ctggccccgg agtggatggc ggccgccacc acatggcggt cgtccggatc
5821 gggcagatcg atcgagggaa tcaggtactc gaagccggtg accagacaat cccggacgtg
5881 ggcgttcatc agctggcgcg tccggttcag ctggtcttga gtgagatcgg gacggctgac
5941 cagtacgttg cgcgtccact catcgtgaat catgtcgctc cagcgcgccc ggtacagatc
6001 cgacagcgcc agatgcatca acaggtcgcg caggggtgcc ggatagagca cgcaggcgtc
6061 atagacgacg gtgaagtgcg aactcatcca gtcaataccc catgccgagt tcctgggcct
6121 gggccgccag ttcatcgagc gccttgcggc gctcggcgtc gatccggttc ttgtaggcga

6181 tcacgtcctg gtagcgcacg cggcgatgcg tgccgatctt gtggaacggg atatcgcctt
6241 tctccagcaa ctggacgagg aaggggcgcg agacgttgag cacgtcggca gcctcctgcg
6301 tcgtcagctc ggcatggatg gggatgatcg acacggcgtt gccctggccg atctcggtca
6361 ggacgtcgag cagcaggcgc aacgccgaag tcgggatcga tacggtgcgc accgcgccct
6421 tttcgtcatg gaagtcgatc tgctgggttt cggcacgggt ctggagcacg gtcgagagca
6481 cgcgcccgga ttcccgggcg agcgcgatgt cctcggccga aggcaacgtt ttcgggatgg
6541 cgggagtgtt catgaggctc tcctcgtcgg gtcaaagttt ggtctgaagc cattataaac
6601 gaaatatccg aaatcgcaat aaccgaaacg gcgcggcgca agcattgcga atcaacgact
6661 tagccgttca ggggttgcac ttgccccgac gagctcggct catcggcgcc caaaacgcgc
6721 tcgcgcaagc ccaaagcgtg gagcaattca atagggactc ccaaacaaga ggagtcccgc
6781 aatgcaaaac caagtccctt ctgttcaatc cggccggaaa cccagccggc cccgctcgga
6841 cggtgccacg cgcatcgccc ttgacgagaa cgagctcgcc gcccgctggg ggctttccgt
6901 caaaaccctg cgccgctggc gccaggagca gctcggcccc gtcttctgca agctcggggc
6961 gcgcgtcacc tacctgatcg ccgaagtcga ggccttcgag cggcgcgtct cgcgctactc
7021 gaccttcgcc cgggcgtacc agtgaggggg cggccatgac gaatctcacc atcctcccgg
7081 ctgacctcgc cgccatgtcg gtcagcgacc tggcctcgct gccgccggcg cagaagcagg
7141 agatcagtcg caacctcgac gaggcgctcg actggctgaa gaaggcgcgc gccaagttcg
7201 acgccgcgct cgatgccgcc tatggcgaac aggcccgcgc cgcgcgcctt gcggccggca
7261 aggacttcgg cgtcatccac ctcagcgacg gcccgctgcg cgtaaccgtc gacattccga
7321 agcgcgtgtc ctgggaccag gagcagctcg cggccatcgc ccggcgcatt gccgcctccg
7381 gcgaaaaggt cgaggactac ctcgacatcg agttctccgt ctcggaatcc cgcttcaaca
7441 actggcctgc ggcgctgcgc gcgcagttcg aggccgcccg caccgtcaag cccggcaagc
7501 cgtcgttccg gctggccctc atctcggagg actgatccat gagcaccgaa ctgattccgt
7561 ttgatttcga gggccgtccg gtccgtgtcg tcaccgatgc ccagggcgag ccgtggttcg
7621 ttgcggctga tgtcgcccag tccctcgaat accgcatggc cagcgacatg actcgctcgc
7681 tcgatgacga cgagaaaggt acgcagattg tgcgtacccc ctccggcaat caggagatgc
7741 tggtcatcaa cgagtccggc ctctactcgg cgatcctcaa gagccgcaag cccgaggcca
7801 agcgcttcaa gcgctgggtc acgcacgagg tgttgccggc catccgcaag accggggcgt
7861 atgccgcagg cgccacgctg ccggctctcc ccgtgccgac gcaggaccgc gtgtcgtcga
7921 tcctgttgat cggcgaggcc gtggccaagg tgccgggcgt caaggccggt atcgcgatgg
7981 ccgcaacgct gacctgcatc caggagaaca ccggcctcgc ggttgagacg ctgcgccgcg
8041 cgctgcccgc cgccaatgca ccgatctgct cgctcaacgc gactcagctc ggcaagctga
8101 tcaaccgctc ggccaaggcg acgaaccagt tgctggcggc caccggcctg caattccgca
8161 acaagcgcga cgagtgggag ctcaccgagg cgggcgaagc ctgggccgag gccatgccgt
8221 actcgcgcaa cggccacagc ggctaccaga tcctctggaa tccggcggtc gtcgagcagc
8281 tgaaggaggt ggcgtgatgg cactccccat catcaccgcc gaccagcggc tgcgcgagaa
8341 gcagggcgtc aagctcgtgc tgctgggcaa gagcggcatc ggcaagacca gccagctcaa
8401 gacgctgccg gaaggatcga cgctgttcgt cgatctcgag gccggcgatc tcgcggtcaa
8461 ggactggcac ggcgactgcg tgcgcccgac cacctggccc gaattccgcg acctcgtggt
8521 gttcctcgcc ggcccgaacc cggcgctgcc cgcggatgcg ccgttctccg aggcgcacta
8581 ccggcacgtc tgcgagcgtt acggcgaccc ggcgcaactg gccaagtacg acacctactt
8641 cgtcgacagc atcaccgtgc tcgcgcgcct ggccctgatc tgggccaaga cccagccgca
8701 gtccttcagc gagcgtaccg gcaagcctga cacccgcggc gcctacggcc tgctgggcac
8761 cgagatgctg accgctctca cgcacctgca gcacgcgcgc ggcaagcacg ttgtgttcgt
8821 ggccatcctc gacgagcgca tcgacgactt caaccgcaag gtgttcgtgc cgcagatcga
8881 gggtgccaag accgccgccg agctgcccgg catcgtcgac gaggtcgtga cgctcgccga
8941 gatcaaggcc gaggacggca gcgcctaccg cgccttcgtc acccacaccc tgaatcccta
9001 cggctacccg gccaaggacc gctccggcca gctcgagctg ctggagccgc cgaacctgcg
9061 cgcgctgatc gacaagtgcg ccgccgccac ccgaatcccg acatccaagg agtaagccat
9121 gaccgcatgg aacgatttca acgacgccga acaacagcag agcttcgacc tcatccccaa
9181 gggcaccgtc gcccgcgtgc gcatgaccat caagcccggt ggctacgacg atccggccca
9241 gggctggacg ggcggctacg ccacgcagag cttcgacacc gggtcgatct atctcgcctg
9301 cgagttcgtg gtgctggagg gcgagtacgc caaacgcaag ctctggtcga acgtggggct

9361 gcacagcccc aagggccaga cctgggcgag catgggccgc agcttcatcc gcgccgcgct
9421 caacagcgcg cgcaacgtgc tgccccagga caactcgccg caagccgccg ccgcacggcg
9481 catccaaggc ttccacgagc tggacggcat cgagttcgtc gcccgcatcg acatcgagaa
9541 ggacgcccgt ggcgagctgc ggaacgtcgt caagctggcg gtcgagcccg atcaaccgga
9601 ctacgcgcgt gtgatgggcc tgccgcccaa gacacctggc ggtagctcgg gcgcaccggc
9661 cgccgccatt ccgtcgcgcg tgatgcccgc ccctgcggcc gctcaacgtc ccgccgtgcc
9721 cggaaagccg gcctgggcgc agtgaggagg ggcgatgaaa atgagcgaag cgaagtttcg
9781 cgaaaccgaa ggcgagagcg aatgcaatga gcggcaaatg ctgggtctgc aaacgacagg
9841 cccggggctt cggtcacacc gacaaccgcc acggtgtcgg cgatccccgg cgctacccca
9901 tcgactgggt gttctgctcg cggcgctgcc aggaggcgtt ccacgcgctc tatggcaact
9961 ggctgcgggt gaaggaaggc cgtgcggaca tcgaggaggt cgccatgatc gatccgtctg
10021 atgtcgagct ggccgcgatg aagaagtgcc tcaaggcctt cggcgaggcg gccggcgaga
10081 tcggcttcgg caagcccctg ggcgactact ccgaggccga agcgctgcag gtcatcgacg
10141 ccatcgtcac ctgctacacg gaagcgatgg tcgagcacca tgaggcgacc aagtacccgc
10201 cggtgcgggg gctcacgaac ccggttagcg atcccttcgc cgacatgaag gacgatctgc
10261 cgtgggaggt gaagccgtga tggacttcaa ctcctcggcc agcctgtccg gccaggtcac
10321 agcgctggtg gacctcggga tgcagcttgc gcgctccctc caaggcacgc ggcaatacct
10381 cggcgcctcg cggctgggcg ccagctgcga gcgcgtgctg cagtacgagt acgcgcaggc
10441 gccggtcgac cccggccggg aaaccgaagg ccggatgctg cgcatcttcg agcgcggcca
10501 cgtcatcgag gattgcatgg tgacgtggtt gcgtgatgcc ggcttcgact tgcgcacgcg
10561 caaggccgac ggcgagcagt tcggcttcat cgcgctggac ggccgactgc agggtcacgt
10621 cgacggcgtc atcgtcggcg gccccgaggg cttcggctac ccggcactgt gggagaacaa
10681 gtgcctgggc gcgaagtcct ggcgcgagct ggagaagaac cggctggcgt ctgcccgccc
10741 gatctacgcc gcgcaggtcg cgctctacca ggcctatctc gaactgcacg agcacccggc
10801 actgttcacg gcggtgaacg ccgacacgat ggagatctac gccgagctcg tgcccttcga
10861 cgccgagctc gcgcagcgca tgtccgaccg cgctgtgaag gtcatctccg cgaccgaagc
10921 cggcgagctg ctaccgcgct cgttctccga acccacccat ttcgaatgcc ggatgtgccc
10981 gtggcaggac cggtgctgga gggtatgaca catgaagaac aacgatgaac gaacgctgac
11041 cgccgtcagc gagatggtgg gcgagccgat gatcgacgcc aagcaggccg ccgccgcgct
11101 gcgcctgccc tactactggt tcgccgacca cgcgatgcgc agcaagtacc ggattccgca
11161 ttacctgttg ggcggactgg tgcgttatcg gctgtccgaa ctgtccgcgt gggccgcccg
11221 cagcgccgcg gttcaagggc gcgaggcccg cgaagaggac acacctgccg gggaggccga
11281 atgatcgact tcaacgacat cccgcaaccc gaggcacaag accgccaggc ccgccgcgag
11341 gagattcgcg ccgcactgct cgcccgcctg gaatcggtgc tgttcgctct tctccccgca
11401 ggcaagaagc gtggcggcaa gttcctgatc ggcgacgtgc tcggcagccc tggcgacagc
11461 ctggaagtcg tgctcgacgg cgagaaagct ggtctctgga cggatcgcgc caccggcgat
11521 ggcggtgaca tcttcggctt gatcgcggcg catcttggcg cagacgtcca cgccgacttc
11581 ccgcgcgtgc tcgacgtcgc cgccgatctg gtcggacgcg cgcatccggc gccgccacgc
11641 aaggccagat cggcgccgcc ggtggacgga ctcggcccgg ccacggccaa gtgggactac
11701 tttgaccccg ccggcaaact gatcgcggtc gtctatcgct acgacccgcc cgggcgcaag
11761 aaggagttcc gtccctggga cgccaggcgc cgcaagatgg cgccgcccga gccgcgcccg
11821 ctctacaacc agccgggcct cgtggccgcc gacacggtcg ttctggtcga gggcgagaaa
11881 tgcgcgcagg cgctaatcga catcggcgtc gcggcgacca cggcgatgca cggcgcgaac
11941 gcgcccgtgg acaagaccga ctggtcaccg ctggcgggca aggccgtgct gatctggccc
12001 gaccgcgaca agccgggctg ggagtatgcg atggcggctg cgcaggcggc acttgccgtc
12061 ggagccatgt cctgcgacgt gctgctgccg cccgacgacc ggtccgaggg ttgggacgcg
12121 gccgacgccg tggccgaggg cttcgatgtc gcgggcttcc ttgcctttgg tccgcgcatg
12181 agcatcaagc ccgccagcgt cacgccgacg caggagcctt ccgtctgggc gacggacgat
12241 gcgctggcgc tggccttcac gtcgcgctac gccgaggact ggcgctactg cgcggcctgg
12301 ggcaagtggc tggtctggga cggtcgccgc tggcaggccg acgaaacctt gctcgttcac
12361 cacctgatcc gcgcgatctg ccgcgaggcc gcgctcaagg ccgactcgca tcggctggcg
12421 gccaagctcg cggccagcag cacggtcggc ggcgtcgaac gcctcgcccg caccgaccgg
12481 cggcacgcct ccacctccga ggaatgggac gctgacctgt tcgcgctcaa cacgccgagc

12541 ggcatcgtcg atctgaagac cggccgcatc aagccgcacg accggcgcga gcgcatgacc
12601 aagctggcca ccgccacgcc gcacggtgac tgcgcgcgtt ggcgcgcgtt cctcgaagac
12661 atcaccggcg gcgacaccgc cttgcaggct tacctgcaac ggatggtcgg ctattgcctc
12721 acgggcgcga ccagcgcgca cgcgctgttc ttcctctatg gcacgggcgc gaacggcaag
12781 tcggtgtttg tgaacacgct ggcgacgatc ctgggcgatt acgccaccag cgcaccgatg
12841 gacaccttca tggaagcgcg cggcgatcgc catccgaccg acctcgccgg cctgcgcggc
12901 gcgcgcttcg tgtcttccat cgaaaccgag cagggccggc gctggaacga atccaaggtc
12961 aaggccatca ccggcggcga caaggtgtcc gcacgcttca tgcgccagga cttcttcgag
13021 tacctgccgc agttcaagct ggtgatcgcg ggcaaccaca agcccgccat ccgcaacgtg
13081 gacgaggcga tgaagcgccg gctgcacctg atcccgttca cggtgacgat cccgcccgag
13141 aagcgcgatg gtcagctcac cgaaaagctg ctggccgagc gcgacggcat cctcgcgtgg
13201 gcggtcgaag gctgcctggc gtggcagcgc gacgggctga atccccccgc cagcgtggtg
13261 tcggccaccg aggagtattt cgacgaggaa gacgcgatcg gcgacttcct cgacgaggaa
13321 gcgcagcgct ttgagcaggc ccgcgtggcc gtggccgacg tgttccagcg ctggcaggag
13381 tgggccacgc ggcgcggcga gtatgtgggc accagtcgct ggctggcgca gcagcttgcc
13441 aatcggggtt tcggtcgcgc gcgcctgcac ggtggcgtca aaggcctcgc aggcctatcg
13501 ctcaaaccaa gggactacgg cacgcgcttg ccgtaccgcg atgactgacc cgcggtgacc
13561 gaaggtgacc cgcccaggga ttgaatctct ttacgcgcgt gcgtgcgcgc aggcgtaacg
13621 cccaatccac aaaccggtca ccgtcggtca cccggacgag aacacgaacg aaggactgca
13681 acgatgacaa cgacgattct tgctctcgat ctgggcacca cgaccggctg ggcgctgcgc
13741 ggcggcgacg gccacatcac cagcggctcc gagagctttc gtccccaacg cttcgaaggc
13801 ggcggcatgc gcttcctccg gttcaagcgc tggctcaccg aactcaaggg tcatgtagac
13861 ggcatcggcg cgctgtattt cgaggaggtg cgtcgccacg cctcgaccga cgcggcgcac
13921 gcctatggcg ggtttctcgc cacgctcacc gcctggtgcg agcaccacca gatcccgtac
13981 cagggcgtcc cggtcggcac gatcaagaag cacgccaccg gcaagggcaa tgccggcaag
14041 gagggcgtga tcgcctccgt ccgcgcgcgc ggtcacgcac cgtccgacga caacgaagcc
14101 gacgccctgg ccctgttgct ctgggccatc cagaaccacg acgacgggca ggaggtgtga
14161 ggtgaagatt ccgacaccgc gataccgctg ccccctcggt cggctgcaac ccgacaccac
14221 cgacctggac gccatcaagc aacggggttg gcgcgatcag cacatcctgg tggtcaacgc
14281 cttcgacgaa cgtctggatt tcatcgaacg tgaaatcgtg cgacgcatcg gcgaacggct
14341 gtacggccag ggagggacga atcatggctg agtggacgat tgacgacgtg gcggcacgct
14401 tcgaggaagc cgccaccaca ggacgccgcc ttcctcccgt gcgtgtgcag ggctacttca
14461 acacgtggcc ggctttcgtg cgcaaggagt gggaggcctt cgcagccgac gagaaggtct
14521 accgcccctt cccaccgagc cccgaggcca tcgaccggat gctggagacc atgcgctggg
14581 tgcaatggct ggaagtcgag cagcgtcacc tcgtgtggat gcgcgccaag cgttacggct
14641 ggagggacat caccatccgt ttcgcctgcg accgcacgac ggcgtggcgg cgctggcaga
14701 aggcgctgga gatcgtggcg gcacggctga acgagaaggg tttgccaaat tttgccaaag
14761 cggctacgat gaagccatga gtacgatgaa tatttccctg cccgacgcgc tcaaggcctt
14821 tgtcgatgag caagtcagcc aacgcggcta cggcaccagc agcgagtatg tgcgcgaact
14881 gatccgcaag gatcaggatc gccagcgctt gcgcggcttg ctgctggcgg gcgccgcctc
14941 ggcaccaacc gcccccgccg atagcgacta cttcgattcg ctgcgagccc gcgtgcggac
15001 tgcccgggga tgaaggccaa gcccgtcatt cccagggcgc tggccacgca ggatgtcgaa
15061 gaagcaatcc gctactacct gagcgaagac gccaagcagg ctgcgctcgg cttcattgat
15121 gccctggagc aggcctacag ccacattggc cggcacccca cgacgggttc cccccgctat
15181 gcgcacgaac tcgacctgcc cggcctgcgc agctggccgc tgaagcgcta tccgcacatc
15241 gtgttctacg tcgagcgcga cgaccatatc gacgtctggc gcgtgctgca tggtatgcgg
15301 gatatccccg cgtggatgcg cgccggcgat gacacacctc gctgacggaa tgcttcccaa
15361 aatcctgggc aatttagggt aacgcctgcc gcatttgtcc tcgccttgcc ctgcctgtcc
15421 gtttcgacgc tcggcaaccc cgcaacaaaa cagcccggtc ggggtagtat ttccgctatc
15481 ttctggacag cggtgcaggc agcgaagacg gcccaaggca aaaggggtcc ttcctgacga
15541 aatcgcgatg cggggggcgc gagcgcggca ttcgcctagc gtccgactgc aaaccgaggt
15601 ttgcagggtt tgcagtttgc acccccgccc ctgtccgcac acgtcacgag cccgcccacg
15661 gctttccgtc ggcgggtttt ctttttggct gcgccgaaac ggcgctgcgc ttgtcttccg

15721 aggaaccgat tctgaacacg ctcaacgtcg aataccgcaa ggtcgagacg ctgattccct
15781 acgcgcgcaa tccgcgcatg cacagcgaga cgcaggtcgc caagatcgcg gccagcatcg
15841 tcgagtacgg ctggaccagc ccgatcctcg tcgatggcga caacggcatc atcgcgggcc
15901 acgggcggct cgcggccgcg cgcaagttgg gactcgcgga agtgccggtg atcgaactgg
15961 cgcacctcac gccgacgcaa aagcgcgcgc tcgtcctcgc cgacaaccgt ctcgcgctcg
16021 aggcgggctg ggacgaggaa ctgttggcgc tcgaactggc cgaactgtcc gacgccggct
16081 acgacctcgc gctgaccggc ttcgacgatg gagagatcga agccttcctg tcggagccgc
16141 agccgcacga ggacaccagc gccgaggccg acgccggcaa ggacgatacg accgacgatg
16201 tgcccgacgc acccgtcgtt ccggtctcgc gcccaggcga cgtgtggtgc ctcggatcgc
16261 atcgactgat ctgcggagac gccaccgatc cggccgtggt cgcggcgctc atgcggggcg
16321 agcgggcgaa gctctgcttc acctcgccgc cgtacggcaa ccagcgtgac tacaccaccg
16381 gcggcatcgc cgactgggac gcgctgatgc gcggtgtatt cggccatctg ccgatggccg
16441 aggacggcca ggtgctggtc aatctcggac tgatccaccg cgacaacgag gtgattccgt
16501 attgggatgc gtggctcggc tggatgcgcg cccagggctg gcggcgcttc gcctggtacg
16561 tctgggacca aggacccggc atgccgggtg actgggcggg acgcttcgca ccgagcttcg
16621 agttcgtttt tcacttcaac cgacagagcc gcaagcccaa caagatcgtg ccctgcaagc
16681 acgcgggcca agacagccat ctgcgggcag acggatcgtc cacggcgatg cgcaacaagg
16741 atggcgaggt gggcggctgg acgcacgcgg gcctgcccac gcaggacaca cgcatccccg
16801 actcggtgat ccgcgtgatg cggcacaagg gcaagatcgg caagggcatc gaccacccgg
16861 ccgtgtttcc ggtggccctg ccgcagttcg tcatggaggc gtactcggac gaaggcgctg
16921 tggtgttcga gcccttcggc ggcagcggca cgaccatgct cgccgccgag cgcaccggcc
16981 gcgtgtgccg cagcgtcgag atcgcgcccg agtatgtcga tgtcgcgatc aagcgcttcc
17041 agcaaaacca tccggacgtg gcggtgacgc tgcttgcctc tggtcagtcc ttcgccgagg
17101 tggcggccga gcgtgagcgt gaagcggagg tggtcgcatg accaactcct ggtttgccga
17161 caagatcgag cagtggccga ccgccaagct cgtcccctac gcccgcaatg cgcggacaca
17221 ctcggacgaa cagatcgcgc agatcgccgc ctcgattgcc gagttcggat tcaccaatcc
17281 gatcctcgcc ggcagcgacg gcgtgatcgt cgccggccat gggcgtcttg ccgccgcgca
17341 gaagttgggg ctcgacatcg tgccggtggt cgtgctcgat cacctcaccc caacccagcg
17401 gcgcgcactg gtgatcgcgg acaaccggat cgccgagaac gccggatggg acgatgcgtt
17461 gctgcgcgtc gaactgaaag cactccagga cgagggtttc gacctggatc tgaccggctt
17521 cgatgccggc gcgctggccg atctgctggc aggcgatgag cccgccaacg aggggcagac
17581 cgacgacgac gtggtgccgg aggtcagcga gacgccggtg tcgcgaccgg gcgacgtctg
17641 gctgctcggc ccgcaccgcc tgttgtgcgg cgatgcgacc gtggccgaca gctacgcgcg
17701 cctgctcgca ggcgatccgg tggacatggt gttcaccgat ccgccataca acgtgaacta
17761 cgcgaacagc gccaaggaca agatgcgtgg taaggatcgg gcaatcttga acgacaacct
17821 gggtgacggc ttccacgact tcctgctggc ggccctgacg ccgacggtcg cgaactgccg
17881 gggcgcgatc tacgtggcga tgtcgtcgag cgaactggac acgctgcagg cggccttccg
17941 tgccgctggc gggcactggt cgacgttcat catctgggcc aagaacacct tcacgctggg
18001 gcgcgccgac taccagcgcc agtacgagcc gatcctctac ggctggcccg agggcgcgca
18061 gcgccactgg tgcggcgacc gcgaccaggg cgatgtgtgg caaatcaaga agccgcagcg
18121 caatgacctc cacccgacga tgaagccagt ggaactggtc gagcgggcca tccgcaactc
18181 cagtcgcccc ggtgccgtgg tgctcgaccc ctttggcggt tccggcacca cgctgatcgc
18241 ggccgagaag gcaggacgcg tcgcgcgact gatcgagctc gacccgaagt atgtcgatgt
18301 gatcgtgcgc cgctggcagg actggaccgg caagcaggcc acccgcgagg cggatggcct
18361 ggcgtttgat caggcggcta cgtcttccgc gacgatcctg tagacgcgct cgccgccgtc
18421 aagcttctcg gagacgagga ccagcccgag cttcttcttg aaggccccgg cgaaggtgcc
18481 gcgcaccgtg tgcgcttgcc agcctgtggc ttcgcagatc tggcgcaccg ttgcgccctc
18541 cgggcgcttg agcatgcgga tgacctcggc ctgcttgctg ttttgccgga tgctgcgcac
18601 cctctcggct tcgcctcgcg aaacttcgct acgctcgttt tcgcgcgtgc ggggcttggc
18661 gtcggagcgc cgttgcgccc aggtcgcctc ggcggcggag acggccgcct ccagttcctc
18721 atccggttcc accggagcgg gggcgggccg gggacaaccc atcgcgtcgt agccttcggc
18781 ggcgacgaac cagtcggcac caatggatgt gatcagcgcg cgtctgaaaa ggccgtccag
18841 caccttcttg cgggcgccgc ctttgacgtt gtcggggaac cagacgatct tgccgtcggt

18901 gtgttcgatg gcataggcca cgattgcatg ctgggcgggt gtcagttgga tggtggtcat
18961 cgcttgctcc ttgcgtgggt tgatcgggtg acgtgatgaa cgcgctgttt ccgacggaag
19021 ccaagcgaat gccgcgaaga agttgaacaa atcagccgac tgatcagtga atgggaatct
19081 cgatccgcgc ctatgcgcgc caccgtggcg tgaccgatac agccgtgcac aaggcgatcc
19141 gcgcgggacg catcacgccg gaggctgacg gcaccatcga tccagagaag gccgaccgcg
19201 aatgggcccg caactcggga ccgcccaata ccggcacccg cacgaaggtg ccgaaggtcg
19261 ccgtgcccga cgcaccgggt atggggagtg agggcccggc ggccctgcca acgggtggcg
19321 cttcgctgct gcaggcgcgt accgtcaacg aggtggtgaa ggcgcagacg aacaaagtac
19381 gcctcgcccg cctcaagggg gagctggtcg accgcaatca agccatcgcc cacgtgttca
19441 agctcgcgcg agccgagcgc gatgcgtggc tcaactggcc ggcgcgcatt tcggcgcaga
19501 tggcggctcg gcttggcgtt gatccgcaca cgatgcacgt cgcgctggag gccgccgtgc
19561 gcgagcacct gcaggagctg ggcgagttgc gcccgcgggt ggactgatgg acttcgagta
19621 tgaaggcgct gccgagatcg agcgcgcatg gcgcgagggt ctgacgcccg atccgctgct
19681 caccgtctcc gagtggtcgg accgccaccg tgtgctctcc agcaaggcat cggccgagcc
19741 gggccgctgg cgcaccagcc gcacgccgta tctcaaggcg atcatggact gcctgtcccc
19801 gacttcgccg gtcgagcgtg tggtgttcat gaaggcggcg cagctcggcg cgaccgagat
19861 gggctcgaac tggatcggtt acgtgatcca ccacgcgccg gggccgatga tggcggtgtg
19921 gccgacggtg gagatggcca ggcgcaactc caagcagcgc atcgatccgc tgatcgagga
19981 gtcgcccgct ctgctcgaac tgatcgctcc ggcccgttca cgcgactccg gcaacaccat
20041 cctcgccaag gagttccgtg gcggtgtgct cgtaatgacg ggcgcgaaca gcgccgtggg
20101 cctgcgctcg atgccggtgc gctacctgtt cctcgacgag gtcgacggct atccgctcga
20161 cgtcgaggga gaaggcgacg cgatctcgct ggcggaagcg cgcacgcgca cctttgcgcg
20221 gcgcaagatc ttcatcgtct ccacgcccac gatttcgggg gcgtcggcga tcgagcgcga
20281 gtacgaagcg agcgatcagc gccgctactt cgtgccgtgc ccgcactgct cgcaccggca
20341 gtggctgcgc ttcgagcagt tgcgctggga caaggggcag ccggagacag cggcctatgt
20401 ctgcgaatcc tgcgacacgg cgattgccga gcaccacaag acgtggatgc tggagcacgg
20461 agagtggcgc gcgatggcac ccgagaacgg cgccaggacg gcaggattcc acctgtcctc
20521 gctctacagc ccggtcggct ggcgcagctg gcgcgacatc gcggctgcct gggagaacgc
20581 ggtcagcaag gagtccggct cggccgccgc gatcaagacc ttcaagaata ccgagctcgg
20641 cgaaacgtgg gtcgaggaag gcgaaacgcc cgactggcag cggctgatcg agcgccgcga
20701 ggattaccgg atcggatcgg tgccacgggg cggcctgttg cttgtcggcg gcgccgacgt
20761 gcagaaggat cgcatcgaag tgtccatctg ggccttcggg cgcggcaagg aatcctggct
20821 cgtcgagcac cgcgtgctga tgggcgacac ggcgcgcgat acggtgtgga agcgcctcgg
20881 tgagctgatc gccgacacct ggacccacga gtcgggagcc gcgatgccct tggcccggtt
20941 cgcgctcgac accggcttcg ccacgcaaga ggcctacgcc ttcgtgcgtg cctgccgcga
21001 tccgcgcgtg atggcggtca agggtgtgcc gcgcggcgcg gcgctgatcg ggacgccgac
21061 ggcggtcgat gtctcccaag cgggcaagaa gctgcgccgg ggcatcaagg tctttgcggt
21121 cgccgtcggc atcgccaagc tcgagctcta caacaacctg cgcaagagcg cgctggtggc
21181 cgaggatggc actaccgtgg cttacccggc cggcttcgtc catctgccga agatcgacgc
21241 ggagttcatc cagcagctct gcgccgagca actgatcacg cgccgcgacc gcaacggctt
21301 cccggtgcgc gagtggcaga agatgcgcga gcgcaacgaa tcgctcgact gctacgtgta
21361 cgcccgcgcg gctgcagcgg cggcgggact ggatcgcttc gaggagcgcc actggcgcga
21421 actggagcga caactcggga tggaacggcc gccggatgag ccgtccccgc cacagccatt
21481 gaacctggac gaggccaccc acagcggtgg cctcgtcgtt tccgacatcc gcaaaaccgg
21541 ccgccgcgtg atcaagagcc gctggctgtc ccgatgagga atccatgacc tacaccacca
21601 cccagctcga cgcgctcaag cgcgccctag gcagcggcga gcgccgcgtc accttcggcg
21661 acaagacggt cgagtaccgc tcggtcgagg agttgcaggc cgcgatccgc accgtcgaag
21721 ccgagcttgc gcgcaatgca ggcgagagcc cgacgcgtca gatccgggtc accacctcga
21781 agggattctg atggcctggt tctccagaat ccgaagcctg ttcgggcaac cgccggtgca
21841 tgaggccgcc ggccgtggcc gccgctcgct cgcgtggatg cccggcaacc cgggcgcggt
21901 ggccgcgatg ctggcaacca gcgccgaact gcgcatcaag agccgcgacc tggtgcgccg
21961 caatgcatgg gcgcaggccg ccatcgaggc cttcgtggcg aacgcggtcg gcaccggcat
22021 caagccgcaa agcctctcgc ccgaggagcg cttcaaggcc gaggtgcagg cgctgtggcg

22081 cgattggacc gaggaagcgg acgctgccgg gcagaccgac ttctacggcc tgcaggcact
22141 ggcctgccgc gcgatgctcg aaggcggcga gtgcctgatc cggctgcggc cacgccgcct
22201 ggaagatggt ctggtcgtgc ccctgcagct ccagttgctg gagcccgagc acctgccgat
22261 cagtctcaac accgatcttc cttcgggcaa cgtcgtgcgc tccggcatcg agttcgacag
22321 cttgggacgg cgcctggcct accacctgta tcgctcgcat cccgaggacg ggcgtctcgc
22381 gccgatgtca ggacagggcg ggatggacac ggtgcgcgtc gatgcgcgcg agatcatcca
22441 tctgtaccgc gtccttcgcc ccggccagat ccggggcgaa ccctggcttt cgcgggccct
22501 ggtcaagctc aacgagctag atcagtacga cgacgccgag ctggtgcgca agaagaccgc
22561 cgcgatgttc gccggcttcg tcacccgcca gaaccccgag gacaacctga tgggcgaagg
22621 ggccgccgat ggcgacggca tcgcgctcgc cgggctggag ccgggcacgc tgcagatcct
22681 ggagcccggc gaggacatca agttctccga ccccgccgac gtgggcggct cgtattccga
22741 gttcctgcgc acgcagttcc gcgcggtggc cgccgccatc ggcatcacct acgagcagct
22801 gaccggcgac ctcaccggcg tgaactactc atccatccgc gccgggctgc tggagttccg
22861 ccgccgttgc gagatggtgc agcactcggt gctggtccat cagctgtgcc gcccggtgtg
22921 ggcggcgtgg atgaggcagg ccgtgctcgc cggtgtgctg gaggcgcccg gttttgcacg
22981 cggcgggccg gcgcgccgcc gccagtacct gcaggtgaag tggattccgc agggctggca
23041 gtgggtcgat cccgagaagg agttcaaggc gctgctgctc gcgatccgtg cgggactgat
23101 gagccgctcg gaagcgatct cggctttcgg ctacgacgcc gaggacgtgg atcgcgaaat
23161 cgccgccgac aaccggcgcg ccgacgacct tggcctggtt ttcgattccg acccgcgccg
23221 cacctcgaag gacggtggct ctgccaccgc gaacgctgct gccgacgccc cgcagaccgg
23281 cagctcgtcg actgtctgaa ggacttccat gaccctgctg ccgcacttgg cggcacgcct
23341 gttcggtgtg ccgctggcga tccatcgccc caaacttgac gtgatcctgg ccgtgctcgg
23401 cccccgcatc ggcgtggccg accttgccgc accgcccgga ttcgcgccgc ccatgcgtgc
23461 cgcacccgcc gcgacgcaga ggatcgcggt catcccggtt cacggcacgc tggtgcggcg
23521 caccgtaggt ctggaggctg agtcgggcct tgccagctac gcaggactcg cagcccaact
23581 ggatgcggcg gtcgcgagtg cgcaggtgga cgccatcctg ctcgacatcg actcgccggg
23641 cggcgaatcg ggcggggtgt ttgatcttgc tgatcgcatt cgcgccgcag ccgccgtcaa
23701 gccggtctgg gccgtggcca acgacatggc cttctcggcc gcctacgccc tggcctcggc
23761 tgcgagcaag gtcttcgtct cgcgcaccgg cggagtgggc tcgatcggcg tcatcgcgat
23821 gcacgtcgac cagtccgcca aggatgcgaa ggacggtgtg cgctacaccg ctgtcttcgc
23881 cggcgagcgg aagaacgatc tcaacccgca cgagccgatc tccgacgagg cgcacgcgct
23941 gctgcaggcc gaggtggatc gcgtctaccg gctcttcgtc gacaccgtcg cccggcatcg
24001 cggcctcgat gcgcgggcca tcgaggccac cgaggcgggc ctgttcttcg gccaggacgc
24061 cgtgactgcg gggctcgccg atgccgtcgg caccttcgac gacgcgctgt cgcaactcac
24121 cgaatcgctt tcctcatccc cgatggcagt cgccgcgcgg gggccttttc tcaacctcca
24181 gacggagtca cccatgcatg atcgaaccga ccccgctgct gctgatcggc ctgctgccga
24241 tcctgccggc agccctcccc aaccggcaac cgccgccatg gcggtggccg acgccatcga
24301 gatcgcgcag acctgcacgc tcgccggccg cgccgacctg atcgcgggct tccttgaggc
24361 ccggaccccg ccggccaagg tgcgtgctca actgctggcc gcacaggcgg aagccagccc
24421 ggaaatcgtc actcgcatcg cacctgatgc cgctgcatcc gctgccaaca acccgctgct
24481 cgaggccgca aagcagctcg cggcgaagtc cgcgtcgctg aagaaggaga tctgagatgc
24541 ccgtctttgt tgaatcgctg aacctgggcg acctgctcaa gtacgaggcc ccgaacctgt
24601 actcgcgcga ccgcgtcacc gtggccgccg gccagaacct gccgctgggc gccgtggtcg
24661 ggatgatcac cgccaccggc aaggtcaagc agatcgaccc gtctgccacc gacggcagcc
24721 aggttgccgc cggcgtgctg atgcagcgct gtgacgcggc gctggccgag cgtgacgacg
24781 gactgatggt ggcacggcac gccatcgtct ccgaccacgc gctctcgtgg ccgaccggca
24841 tcaccaccgc cgagcagcag gccgccatcg cccagctcaa ggcgctgggc atcctcgtcc
24901 gccagggagt ctgaccatgc agaacatctt cgagaatcct gctttctcga tgtcggcgct
24961 caccgccgcc atcaacctgc tgcccaacaa ctacgaccgc ctggcgcgca tgggcctgtt
25021 tgtcgacaag ccgcagcgct tccgctcggt gatcgtcgag gagcagaacg gcgtgctcac
25081 gttgctgccg accatgccgc cgggctctcc cggcaccgtg ggcgtgcgcg gcaagcgcaa
25141 ggtgcgctcg ttcaccatcc cccacatccc gcacgacgac gtgatcctgc ccgaggaggt
25201 ccagggcatc cgcgccttcg gctccgagac cgaactgcag acggtcgccg gcgtgatggc

25261 gcagcacctg cagaccatgc gcaacaagca cgcgatcacg ctggagcacc tgcgcttcgg
25321 cgcgctcaag ggccagatcc tcgacgccga cggcagcgtg atctatgacc tctacaacga
25381 gttcgagatc acgcccaaga ccttcacctt cgacatcgcc gatccgggca acggcttcga
25441 cgtcaagaag gcctgcctgg acgtcatccg ttacgtcgag gacaacctgc agggcgagcg
25501 gatgaccggc ctgcacgcct tcgtgggcga ggacttcttc gacgcgctga ccggccacga
25561 cgaggtgaag gctgcctacg accgctggca ggacgggcag gcgctgcgca ccgacatgcg
25621 cgccggcttc accttcgccg gcatcacctt cgaggagcat cgcggccgcg ccgtcgcgcc
25681 cggcaatgcc gtgcgccgct tcatcgagcc cgacgaaggc catgcgttcc cgctgggcac
25741 gatggacacg ttcgccacct attacgcgcc cgccgacttc aacgagaccg cgaacaccgt
25801 ggcactgccg ctgtacgcca aacaggagcc gcgcaaattc gaccggggca ccgatctgca
25861 cacccagtcg aacccgctgc cgctgtgcca tcgcccggcg ctgctggtca agctcgtcat
25921 gggtggcgta tgagtctggt ggaacagatc tacgacgcag ccgcccatgc gggcttgctg
25981 aaggagtgcc tctggcgccc cgccgacggc tcgccgccgc agagccatcc ggtcggcttc
26041 gccgcgccgg atggcacggt gctcgacggc ttggccctga gcaccgaata cgcgatctcc
26101 taccccgcat ccgtcttcac cggtcttgcg tcccgtgagg cggtcgagat cgacggcgca
26161 acgttcctgg tgcgcgacat ccgggccgtg ggcgacggct ccgagatgcg cgcaaccctc
26221 acgagggcct gacccatggc cgacaactcg atccgcgagc gcatcctgct cgcggtggcg
26281 gcggctgtcc ggcccgcggc cgaggcgttc ggggccggtc tgcaccgttc gccggcggtg
26341 gccatcagcc gggagcaatg cccggcgctg gtggtgtttc cggaaatcga cgccatcacc
26401 gaacgcgcca acgaccgcgt gacgcgggaa ctcactgtcc gcctcgtggc gctcacgcgc
26461 gccgtgccgc ctgccgtccc cgagaccgcg gccgataggc tgctcacggc agcgcacgcc
26521 gccttgatga cggacggcac cctcggcggt ctggccttgg ggctgcgcga agtcgatgcc
26581 gaatgggaga tcgaggacgc cgatgcggtg gccgtggcgc tgccggcgcg ctaccgcatc
26641 acgtaccgga cgctggccaa cgatctttca acccttggat gaacacccat gaccgcactt
26701 gtcctgaccc gctcgcacac gcatgcgggc aagccctatg cgcccggtga ccggatcgag
26761 gtggacgtgg ccacggccga ctggctgatc gcgcacgaca tcgccaagcc ggggccggcc
26821 gcccctgtcg ccaaccccga caccacaccc gaaccgaaac ctctccaacg caaggaaccc
26881 aagccatgag cacctacgcc agtttccaag gccgcgtctt cctcggcaag cgcgacatcg
26941 ccggccttcc tatcgaagtg cgctcgcccg gcaacgtggc cgagctgaag ctttcgctca
27001 agaccgacgt gctggagcac tacgagagcc agacaggcca gcgctcgctc gaccaccgga
27061 tggtcaagca aaagtcggcc acggtgaacc tcactatcga ggagttcacg aaggagaacc
27121 tcgccctcgc gctgtacggc aaccacgtca ccggctcggg cggcacggtg accggcgaac
27181 cggtaggtgg cgccgcgccg gtcgtgggcg accgctactt cctcgcgcat cccaaggtgt
27241 cgtcgctggt cgtgatggat tcggccggca cgcccgccac cttgaccctg ggcacccact
27301 acaccgccga caccgatttc ggtgccctcc agtttctgga gatcaccggc ttcacgccgc
27361 cgttcaaggc cagctacgcc tacggcgtcg ccaccgagat cggcatcttc acgcaggcac
27421 tgcccgagcg ttacctgcgg ttggaaggcg tcaacaccgc acagggcaat gccaaggtgc
27481 tggtcgagct ctaccgcgtc gcgttcgatc cgctgaagga gatctccttc atctcggacg
27541 agtacaacaa gttcgagctg gagggctcgc tgctggccga ctcgaccaag cccttcgacg
27601 cgctactcgg ccagttcggc cgcatcgtgc agctctgagc cggggtgagc catgagtgat
27661 ctggaaaccc tgattccgca atccgtggag ctggtcatcg acggtgagcc cctggccatc
27721 aagccgttga aggtcggcca gatgccgggc ttcctgcgtg cgatcacgcc ggtgatgcag
27781 catctcaccg gcggcgagat cgactggctc gcgctgttca gcgaacgcgg cgatgacctg
27841 ctgtcggcca tcgccatcgc ggtaggcaag ccgcgcgcat gggtcgacga gctggcagcc
27901 gatgaagcga tcctcctggc tgccaaggtc ctcgaggtga acgccgattt ttttacccgg
27961 acggtgatcc ccaggatcga cggtctgttc gcgagggtca ccgcagttca ggcgaaggcg
28021 gctggttcga caccatccaa cacctgatcg agcacggtca ccgactaccc gacatcctcg
28081 actacaccct ggcgcaggtg cgcggcttcg tggccgccac ggcaagaagc gacgcggccc
28141 gcgatgcgcg gctcctgtcg ctcattgcga ttggtacgcg cggtgagtcc cgccagctcg
28201 accagaccct cgacaccctc accgaccgtg cgcatctccg tccgaatcga tagcgccgcc
28261 gcccaggcgc aactgcgccg ctggggcggc gagttccgcg acaaggtcaa gaaggcggtg
28321 gcgcgcgcca ttgccagcga ggcgaccgaa ctcaagcagg atgtacgcgg ccatgtcgcg
28381 ggtcagatgg cggtgatcaa gaaatccttc ctcaaaggct tcaccgccaa ggtgctcgac

28441 aaagatccga accgcctgcc cgcgctgtac gtcggctcgc gcattccgtg gtcaggcatg
28501 cacgagcgcg gcggcctgat cgccggccgg ctgctgatcc cgctgcacgg gcgggtcggc
28561 aggaaacgct tcaaggccca gatcgccgag ctgatgcgcg gcggcaatgc ctatttcatc
28621 aagaacgcga agggaaacat cgtcttgatg gccgagaaca tcaaggagca cgaccgcgtg
28681 ctttcgggat tcaagcgccg ctatcgcaag gccgagggca tcaagcgcct caagcgcggc
28741 gcggacatcc cgattgccgt gctggtgccc aaggtcgtgc tcaagaagcg cctcgacgtc
28801 gagcgtctgg tcgggggccg catcccgcgt ctgtcggcgg ccatcgagcg ccagatccgc
28861 acggtggatt gaagggctga gaagaatcta tggcgaatcg gatttccgtc ctcgtcgcgc
28921 ttgaaggggc tgacgaggga ctcaagcgcg ccatcgcgtc cgccgagcgc agcctgggcg
28981 agctctcgag cactgccaag acggctggcg agaaggctgc tgccgggatg gctgaggtca
29041 aggccgggat gtcggccttc ggtgatcagg tggccaatgc caggacgcag ttgctggcct
29101 tcctgtcgat caattgggcg gcaggcaagg tgcaggagat cgtccagatc gccgatgcct
29161 ggaacatgat gtcggcgcgc ctcaagctcg ccaccgctgg ccagcgcgag tacgccgttg
29221 cccagaagga attgttcgac atcgcccagc gcagcggctt agccgtccag gaaaccgcca
29281 cgctgtacgg caagctgcag caggcggtac ggatgttggg cggcgagcag aaggacgccc
29341 tcacgatcac cgagagcatc tcgcaggcgc tgcgcctgtc gggcgcgtcc gccgaggagt
29401 cgcgttccgc cttgctgcag ttcggccagg cgctggcgtc gggtgtgctg cgcggcgagg
29461 agttcaactc ggtcgtcgag aacagccccc gtctggcgca ggccttggcc gacggcctga
29521 acgtgcccat cgggcggctg cgcaagctgg ccgaggaagg acagctcacc gccgacgtgg
29581 tggtgaacgc cttgctctcg cagaaggata agctggccag cgagtacgcc caactgccgc
29641 aaaccgtcag tcaatcgttc gagcgcctgc gcaatgcctt cgggcaatgg atcaacaagc
29701 tcgacgaatc gaccggcttc accaagaagc tggccgaagc cctgacgtgg ctggcgcaga
29761 acctcgacac ggtgatgcag tggctgaagc gcatcgccga ggtcgggctc gccgtgctga
29821 tctaccgcct gatcccggca ctgatcaccg cgtggcagac cgccggggcg gcggccgtcg
29881 cggcggccag cgccacctcg gcggcgtggg cgactgcgaa tctttccgtt tccgacgcca
29941 tcgccatagt gggcaagttg cgcgcggcgt tcgcggtgct cggtgccttc ctcgtcggct
30001 gggagatcgg cacgtggctg tcggagaggt tcgagatcgt ccgcaaggcg ggcatcttca
30061 tggtcgaggt gctggtcaag ggcgtcgagc agttgcgcta ccgctgggag gtcttcgccg
30121 ccgtcttcac ctcggacacc atcgccgagg cgaccaagcg ccacgaagcg cggcttgcgg
30181 agatgaaccg catcttcgcc gagatgtacg ccgacgccac caagggggcg gacgcggcca
30241 agggcgcgat gaacaccgcc gcgtcagccg ccgaggagat cgccaagcgg ctggaggccg
30301 tgcgccaggg cacgcaggag gcggtcgggc gcggcatcga ggccgtccac gccgcgttgg
30361 agaagctgaa atcccgcctc ggcgaggtcg aacaggccgt cggcaaggcc aaccagacgg
30421 tcaacgacgc gaccgcgaag atggccgagg cctacaaggg gctgaccgcc gtcgtcgagg
30481 ccaatctgca gcggcagatc gaagccgtga aagcgcgtta cgagcaggag aagagcacgc
30541 tcgaactctc caagcagtcc gaagcggcgc tgatcacgaa gtccacgcag ctgctgaccg
30601 aggcgctgac gcagcagacc acgctgcgcc ggcaggccac gaccgacgcg ctgaagctca
30661 tcgacgacga gtcccgcgcc aagatcgaag cagcacgacg cgacgggcag accgaggccg
30721 agcgcgcggc caacgtcacc cgcgtcgaaa acgaaatcct ggccaccaag cgccagacgc
30781 tcgcgcaggc gctctcggag taccgccagc acatcgacgc gctcaacgcc gaagccaacc
30841 ggcatctggc cgagatcaag cgcatcgagg aggaaaagcg ccagctgtcg atgacgacgg
30901 aggagcgcat ccgcgacatc cgccgtcagg gcatgaccga gttcgaggcg accgaagacc
30961 gcaagcgcca gatcgccgag taccaggaga aggcgcgcga ggcgctggcc aacggcgagt
31021 tcgagcaggc caggcaactg gcccagaaag cgatggacct ggccgcgcag gtggcgagca
31081 gccagaccag cgaggccaag cgcggcgagg aagcccgcaa gcagtccgag caggcggtgt
31141 cgcaggtgac gcaactcgaa gcgcaggcgc gcgaggccac ccgccgccag gagtacgcgc
31201 aggccgaagc gctcatgcgg caggccgacc agctgcgcgc cgaactggcc cagaaggcca
31261 aggacgccga cgcgcagatc gcgcaaggta aggacggcgt caatcaggcg atccagcgca
31321 tccgcgactc cgaggaaatt ctcaaccgcg cgctggatgc ggaagccaag gcgcaccaga
31381 cggcggcaca gtcggctctg acggcccgcg accagatcaa ggagacgctg acgcagaccg
31441 aaacgcagat cgaccagatc accgcgaagt tgaaagacgg tctgaaggtc accatcgatg
31501 ccgacaccgc gcgcttcgac aaggccatcg ccgatctgga caaggctctc gccgagaaag
31561 aggtgctgct caagatccag gccgacctgc aggaggccga gaagaagctg cagcagtacg

31621 agcagctgct caaggagggc aagaccctgc cggtcgatgc cgacgtgtcc aaagcgaagg
31681 atgcgctggc ccggctcaag acctacgccg accagaactc ccagctcgag ctgaaggtgg
31741 ccaccgagaa agcgcaggcg gcgatcacca acgtcgaggg gatgatcaag gcactggatc
31801 gcatccagac cgaatcccgg catcagatca gcaccaacgc cgacgcggcc cgcgcagaga
31861 tcatgagcct gaacggtgcc aacacctcga gcacgcacac gatctacgtg cagaaggtcg
31921 aggtcaacgc cgccggcggt ctggttggtg gcggcgtgcg gcgcttcggc gacggtggcg
31981 cggtcgctcc ggcctttccc cgcatgaccg gcggcacggt acccggctcc ggccaccacg
32041 acaccgttcc gcgcacgctg gaggccggcg cgttcgtcat ccgcaaggcg gcggtgcgca
32101 agtacggcag cggcgcgctg gcgcgtctgg ccaatggcgt cgcccacttc gcggtcggcg
32161 ggcgcgtcgc ctcgttgggc aaccccggct ccacaagtac cgatccggac ggcaagccca
32221 gcacgccgaa acgcaaccgc gaagcggtcg aggccttgaa gatgatcgac ctcggcctgc
32281 agggcatgga cgagtacacc ggctggctgg agtggaacta cggtgcctcg gtcagtctgg
32341 acatgcgtcg caagacgatg gagagctacg gcaagcaggc gcaggaagac cggcgcacgc
32401 tggaaggctt catcgatcgc aagacgctca ccggcaacga acggcagacg ctggagcgga
32461 tcaagcagac gtggcgtcag gcgatggccc agccgctctt gtggggcaaa gacctggagc
32521 gcgagctgat cgactacatg gagcagcacc agggcgagtt ctaccggcgc ggcggcctat
32581 cgaaatcgga caccgtgccg gcgatgctca cgcccggcga attcgtcgtg aacaaggagg
32641 ccgtcgcccg ctacggcgcc ggcttcttcg aggcgatcaa caacctgtca gcacccgcgc
32701 aggccttggc gcaacgggtg cagggcttcg ccgccggtgg actggtgccc ccggcgccgt
32761 cgcgcatcac ccgccctgtg ctgcccagtg aaggtgcgac cacccgcagc gtgcgcgtgg
32821 aactcgccgc gggggaccgc aaggtcgacg cccgcatcga cgcgcgtgac gaagcgcgcc
32881 tgctgcaact cctggacgcc gcccgcaccc ggacggcctg accccaatcc gatgcaactg
32941 aagaacctcg cggacgaggt ggccgtgctg ctgccagacg atctgctgtg gaccgacgag
33001 cacgcctggt caccggctgt ggcctcgacg tcctacctca tcaccggtgc cttgctgatc
33061 cagtcggcgc tccggcaggc ggggcgtccg atcacgttga tgggtgcgcc tgacatggcg
33121 tgggtgacgc gtgccacggt ggagcaactg cgcgcctggg ccgcaattcc ggtgagtgct
33181 gcgtccgggc gttttgccct gaccttcgcc gatgggcgcg ccttcacggt cgccttccgc
33241 cacggcgaca cacccatcga gagcgagccg gtgctgggca tcccggcccg atccggcggt
33301 gacttctacc gcctcaccct gcgattcctg gagatctgac catgcccatt caaacgggcg
33361 acgtgaagct gctcaagtcc gccgtcatgg cggacgtgcc ggagggcggt ggtgcgccga
33421 ccggcaacac catcgcggac ggcgtctcga acgccatctt cccggacatc tcggagctcg
33481 accgcgcggg cggccgcgtc aatctgcgca agaccttcgt ctcggtgcag accgacgaca
33541 ccgacaccta cttcggcgcc aacgtcatcg tggccgagcc gccgaaagac gcgcgggtca
33601 gcgtgacgct cttcagcacg gagcgcacct tcgacaaccg cgagcaggcg caactgcgca
33661 tcgaggccta cctcaacaag gggccggagt gggccgggta tctgttcgag aaccacatcg
33721 ccggccagcg cgtgatccag ctgttccagc gccccaccga caccgtgccg aacgtcggcc
33781 agaccctggt gctggtcgaa agcgagggcc aggccacgca gaaggaacag tatgtgcggg
33841 ccaccgccgt ctcggtggtg gagcgcacct tcacctacga cggcgacaag gactacaagg
33901 ccgccgttgt caccgtcgac atcagcgacg cgctgcgtta cgacttcacc ggctcgccgg
33961 ccagccgcac gttcacgcgc gcagcgaacg gcaccaaggt gcgcgacacc gtggtggccg
34021 acgccggcac ctatgtcggt gtggtgccgt tgacgcaggc tgccgcggtc ggcgacttca
34081 cgatcaaggg cgcttcgatc tacacgcagc tcgtccccag cgcgcagacc gagacgccta
34141 tctccttcgt gccgccctat gcggccgcgg gattgccggt gccgggtgca tcgtctgtca
34201 gctacaccgc gaaccacgcc tggacgcccg gccttcgatt caacttgccg ggcggctgtc
34261 tgccgggttc gctcacgctg cagaccgacg gcatcacgat cttcgacgac gcgggtctgc
34321 tcaagaccgc gagcggcacc atcggcacca tcgactacgc caacggcatc ctggcgctca
34381 acgcgggctc gatgtcgaac gccaaggcgg tcacctacac cccggccgca cagatcctgc
34441 gtgcgccgca aagctcggag atccccgtca cgccggagtc gaggagccag tcctacgtcg
34501 gcacggtgac gccggttccg cagcccggca cgctgtccat cagctacatg gcacaggggc
34561 gttggtacgt gctgtcggat gccggcaacg gcacgctcaa ggggctggac gcgagctatg
34621 gcgccggcac cgtcaaccgc aacaccggcg cattcgtcgt gaccctcggc gcgctgcccg
34681 acgtgggctc ctcactcatc ctcacctgga acgtaccgac gcaggaaacg cagcagcccc
34741 aggtcacgct caaagcgtcg cagacgctgg tcttgagccc gcccgaaggc aaggcggtgc

34801 agccgggctc gctgacggtg agctgggaat acaccggcac caagacggca acggccgcca
34861 ccaacggcgt gctgtcgggc gcagcgaccg gcgtcttgcg catcgccagc aaccgcctcg
34921 agttcgcgcc caatgtgctg ccttcggtgg ggacgcagct caccgtcagc tacgtggctg
34981 ggccgaagca ggaggatgcg ttcgcccatc cgtcacgcaa cggctcgggc ctggtcccgg
35041 tcactgcgac gctcggctcc atcgagccgg gttcgctcga ggtcgaatgg aacacgctga
35101 ccgacacctc cgtgctcggc gcgtacacgc tcgcgcaact gctggagatg ggcgtgcaag
35161 ccgcttggcg cgatccgacg cagatcgcgc gcgacgacgg caacggtcat gtagtgctca
35221 atggcagcag catcggcacg gtcgactacg cgaccggaca ggtgactttc aacccggacg
35281 tcactgtgct gatcccgcgt ccgctctaca cctccaccgc catcaacggc acgggccgct
35341 ggcgcttgaa ctaccggggc ctcgcctacg tcgaggcgcc gtcgctgtac ccgaatgacg
35401 agtcgggcta cgtgaagctg cgctacaaca gcgccggctc gacgagcagc gtgacggaga
35461 ccttccagtt cacgccgtcg ttcaagctgg tgccgggagt caatgctcag atcgtccccg
35521 gcacggtggt gctcacgctg cccggggcgc agccctgggg cgacaacggg caaggaacgc
35581 tgcgcgagtt caccagcagc ggctgggtca cgcgcggcac gatcaactat ctgtcgggcg
35641 acgtcaccct cacgtcgtgg acggcgggaa cgaccaacgc cttcacccgc gccagttgcg
35701 tgaccacagt cggcgagaac atctccagcg agtacgtgtt ccgcaccggc gcggcacctt
35761 tgcgcccagg gtcgctctcg atccagtacg cacgggcggt cggcggcacc cagacggtca
35821 cggcgggcat cgacggcaag atccaggcaa ccggcatcac cggcagcgtg gactacgaga
35881 acggcctggt gcgcgtgcgc tttggcagct tcgtgaccgc cgccggcaac gagagcgagc
35941 cctggtactc cgccgaccgt gtgggcagcg acggcaagat cttccggccg gagccggtcg
36001 cagcgtccac cgtccgctac agcgcggtgg cctacagcta cctgccgctc gacgccgacc
36061 tgctcggcat cgacccggtg cggctgccca gcgacgggcg cgtgccgatc ttccgagcgg
36121 gcggcttcgc cgtcgtcggc cacaccggaa agatcactgc accggtggcc aacgggcaga
36181 ccatcaactg tgggcgcgtg cgcctgtccc gcgtgcgtgt ggtcggccac gacggcacgg
36241 tgatccacag cggctacacg accgacctgg aagcgggcac cgtcagcttc aacaacgtga
36301 cgggctacag ccagccggtg accatcgagc accgcatcga ggacatggcg gtggtgcgcg
36361 acgtccagat caacggcgag atcagcttca ctcgcgcgct cacccacgaa taccccgtcg
36421 ccacggcggg cgaccccgcc tccggcagct acgtgtccag cgcgctcatc gccggcgatc
36481 tgttcgcccg ggtgagcctg gtgttcgacc aggcgacgtg gaatggcgct tgggtcgatg
36541 cgatgtcagg cagcgcggcc acggcgacct tcaacaacac ccagtacccg atccgcgtca
36601 cgaaccgggg cgccgtcacc gagcgctggg tggtgcgctt caccaacagc accgccttcg
36661 aggtcatcgg cgagaacgtc ggcgtcatcg ccaccggcaa caccagcgct gactgcgcgc
36721 cgaacaaccc ggccaccggc gttccctact tccacctgcc cgcgctcggc tggggcagcg
36781 gctgggcgac cggcaacgtg ctgcgcttca acaccatcgg cgcgcagttc ccggtatggg
36841 tggtgcgtac ggtgcaacag ggaccggaat cggtgcccaa cgacaacttc acgttgctga
36901 tccgcggcga cgtggacacc ccctgactcc gcagactgga accaacgaca tgcccgatct
36961 ctccgtcaag tacttcaaca gcggcatggc cggcgccccg cagatctcga acaactgggg
37021 cgacctggtg agcatgctcg acgcctgctt ggtcaacggc ttcgcgctga aagccatcga
37081 cacgctgacc tttgccaacg gaatcgcgac ggccacgatc acctccggcc atgcctatca
37141 gcgcgaccag gtggtgcagg tcgcgggcgc cgagcagccc gagtacaacg ggcagttccg
37201 tgtgctgacg acgaccgcga ccaccttcac ctacgcggtg accggcacgc ccgcgtcgcc
37261 cgccacgacg gcgacgagcc tctccgccaa ggtcgcgcca ctgggctggg aaaagccgtt
37321 cgcgggcacg aacaaggcgg cctaccgcag caagaacccg gcctcgccgc agaacctcct
37381 gctgatcgac gacagcctca agacgcccgg ctacacgact tcgtgggcga agtgggcaaa
37441 cgtcgggatc gtggaggatc tggccgacat cgacaccatc gtcggggcgc aggcgcccta
37501 cgacccgaac aacccgacac agaactggaa acaggtcacc gcgaaccagt ggggctggca
37561 caagtggtac cacgcgcgcc agaccggcta cgacacctac ggcgacagcg ggggcggcaa
37621 ccgcaactgg gtgctggtcg gcgacgaccg cctgttcttc ctgttctgca ccttcgcccc
37681 cggcttcaac tggtacggcc gcagctgcta ctgcttcggc gacatcacga gcttcaagcc
37741 cggcgacaac tacgccaccg tgctcgccgc ccatgacctc tactggagca ataacaacca
37801 gtacatgagc taccccggtg agtacggggg cgcgtcgctg atcgcgtcgc tggacttctc
37861 cggtcatgtg ctgctgcgca accacaccca gctcggcaac ccggtgcgct gggcggcgac
37921 ctcgctcaac accaacaacg gccagcagat ctgcgggcgc ggcccgatgc cgtttccgaa

37981 cggcgccgac tacagcctgt ggctgctgcc cacctacgtg cggcaggagg acggccacat
38041 gcgcggcctg atgcccggga tgtactggat gccgcaggac cgcccgtaca gcgaccagac
38101 catcgtcgac aacgtggtcg gccagaccgg caagcgattc ctgttggtgc gcacccagta
38161 cagctcggag accgaaggcg cccaggtggc cttcgacatc accggcccgt ggaggtgagg
38221 tgtgagctac ccgctctacg acaccttcgc cagtgcgccg gcagcgggct acaccaccgt
38281 cctcggcggg atgtccgcca gccacaacgc cgcgcagcag gcccttgacc tctcggcgtc
38341 gagcacgcaa tccatccttc gcttcaacga agcggcgaac ggggacttct ggttcgaggc
38401 ggacatcgaa ctgttgaccg atccgagtgg gcgcaagcat gtcggcctgt ggatgaccac
38461 cggcaacgcc gcggagggct accgcttcgc ccatctggac agcgcctgga gcgtttcgcg
38521 ctggagcagc ggatttggcg acggctcggc agtcaccggc agcgtcaacg acggtgcccg
38581 gccaatggcc ggcattgctg ccacagcgcc gacgttcaac gtgggacagc gccgggttct
38641 gcgctgcgaa gtcatcaccg gtgcgcccga tgcgaatggc gtgccgtggt cgcggctgct
38701 ccagttttcg gccggcggtg tcgtgctgtt ccaggtggcc gacgcgacgt accggggcaa
38761 gctcgtgccg ggcgtgttcc tgtacggcgc gacggcgcga gtccacgcca tcgcgggcga
38821 cacgccgtcc ggtctgcctg cgtttccggc gacggtggcc gtgaacgccg ccgatgacct
38881 gctgccgctg gcaggagggc ccacctcagt cctgcccgat cccgcctcgc ccatcggcgt
38941 cgcggcggac tgcgatctga tgcgccggaa cagcccggcg tcggatctgt ggaacaggcc
39001 tggcggctac gaccacgatt tccacccgat ccaatccgga agaaaagaca tccacttcag
39061 cgggcatggc gtgatcgccg gtaccgtcaa ggagaagggc cagcccgatc agccgctcgt
39121 gcgccgtgtg cagatcatca gcgagaacgc caacgtcctg gtcgccgaaa cctggagcga
39181 tgccgccggc aattaccgct tcgagttcat cgaccctgcg cagcgctaca ccgtggtcag
39241 ctacgactac aagcatctgt accgcgctgt gatcgcggac aacctcaaac ccgaactgct
39301 gccatgaccg ttgcgatcac cgtcgaacac aatgaggcgc ggctcgcggg cacgctcgca
39361 tttctcgacg ctggccccga gccggcgcgg ctgcgcatct acggcggaac gcgtcctccc
39421 acaccagcca ccgtgccgag cagcgaaatg ctcgtcgaga tccggcttac caagcccgcc
39481 ggcacgatct cgggcgggct gctcacgctc acgcagcagg aggacgggct gatcacggcc
39541 accggcgtgg ccacctgggc gcggctggtc aacggcaacg acgtgaccgc gctcgatctc
39601 gattgcagcg gtacggacgg caatggtgac gtgaagctcg cctcgaccgc gctgtacctt
39661 ggcggcgatg cccggatggt gtcggcgatc ctggggtagg ccgtggcagc ccccgagtct
39721 cagcagaccg acctgctgtt cgaccgaccc gccgccaccg atgcgaatct gctgttcggt
39781 gccgacttcg ccccgccacg caacgatctg agggtcctcg cgaccctgcc ggtcccggcg
39841 gtcgcgatca agttcatccc tccggcgcgt gccgagctgc tggcggaatt gccgcgaccg
39901 acggtgcgct cgctggtgct gcgcccgagc gtgccgctca cgttagcgtc gagcctgccg
39961 gggatcgtgt tcaccggcga ggtgaggtac tactcgcgca tgcagcggcc cacggtcggc
40021 gagatccggc atccctggca gggaacgcga gcgaccgaag aggtcgccac ccagccccag
40081 cagcacgccc acgcgacgcc ggcaggatgg agtggtctct gggagggcgc ctcgggtgcg
40141 ccgcaaggca tcgcgcaccg cctgccgcac gtgctgcaga cggcacacca gcagcgccgc
40201 gcaggtcacc aggatgcatc acgcctccaa gacgcgacct ggtttgcaca tcaggatggg
40261 agtcccctcg gactcgtccg cttcgcagct ttcgagcgcg cgaccggggt gcgacgcgcc
40321 acgtggttcc gtcatcagga cggctccgtt acctgccgtg cggggcgcgc aagcggctgg
40381 caagacgcgc gcgtcctggt gcagcaccag ggctcggacc atcagagcgc cacgccatgt
40441 ccgaaaggat ggcgcgggcg ataccagaac acgaggcggc caccgcccgg gatcagcctg
40501 ctggtgatcc cgcggccgcc gcagccccgg ccttgctata cgccaagccc gcacctgttg
40561 ttcgctgggt tggcggtatc gcagggcgat ctgctgttcg tctgtgagaa ccacatcgat
40621 ccaccgccgc ccgatgggga gccggtggtc gttcccgtcc ggagggtgta cttcgtggtc
40681 aacaacgtga cgctgcaccg cctgcccgat ggtgtgccgg tgcccgtgtt caatctctcg
40741 ctgtcgctcg acgtcgcgtc gtgggcctgg ggcttcgagg cgcagctgcc tgccaaggcc
40801 gaatccttgg tcgcgcccgg caatgcctcc ggcccggtcg aactcgtggc cagcatcaac
40861 ggcacggagt tccgagtgtt cgccgagaac atcagccgcg agcgcatctt cggcgaggcc
40921 agcatccgcg tctccgggcg cgggcacaac gccgtgctgg ccgcgcccta cgcgccggtg
40981 atgaccttcc gcaatgccga ggcgcgcacc gcgcggcagt tgatggacga cgtgctcacg
41041 ctcaatggca ttccgctggg ctggacggtc gattggggcc tcaccgactg gaacgtcccg
41101 gccggagtgt tcgcgcggca aggaacgtgg atcgaggcgc tgaccgccat cgccagtgcc

41161 gccggtggct atctggttcc gcatccgtct gaccagcgta ttcgcgtgcg ccaccgctat
41221 ccggtcgccc cttgggaatg gcacaccgtc acgcctgact tcgtgctgcc ggtcgatgcc
41281 gtcgcccgcg agtcgctgcg ttggatcgag aagcccgcgt acaaccgcgt gttcgtgtcc
41341 gggcaggacg tgggcgtgct cgggcaggtg acgcgcgccg gcactgcagg cgacgtcctg
41401 gcgccgatgg tggtcgacgc cctcatcacc gaggccgccg ccgcgcgtca acgtggcctt
41461 gccgtactgg ctgacaccgg acatcagatc gaggtcagcc tccgcctgcc cgtgctcgct
41521 gagacgggaa tcatcgagcc gggtgccttc gtcgagtacc aggatggcag tgtcacgcgc
41581 ctgggtcttg tccgctcgac gcagatcgag gccggcattc cagaggtctg gcagacgctg
41641 ggggtgcaag gccatgcata acctctacgt gcagttccgc cagttgatcc cagacccgcc
41701 gctgcaggcg gggactgtgg tcgagatcgg ttccggagtg gtcaccgtgc agttgcccgg
41761 tggcggccgg atcaaggccc gcggcactgg cgccattggc cagaacgtgt tcgtccgcga
41821 cgacacggtg gaaggcatcg cgccgacctt gacgctggag ctcatcgaga tttgaagcgc
41881 cgctgatccc accgattcat ccccctgaga cccgcctgat gcccacgagc caggcgggtt
41941 tcgcatttct ggagaccacc catgaccgaa cctgacaaca aacccgctca cgtcgagaac
42001 atgctcctct tgcgcaagga ggacttcgac gagttgctcg accgcgccgc ccaacgtggg
42061 gccgagcgtt gcctcgccca ccttggcctg gagaacggca gcgccgcgcg cgacatccgc
42121 gagctgcgcg acctgctcga agcctggcgc gacgcgcgcc gcaccgcctg gcagaccgtc
42181 gtcaaggtgg tcacgaccgg catcttggcc gcgctgctgg tcggcgccgc catcaagctg
42241 aagctgatgg gaggctccca atgatcgaga ccttgctcgg aggcttcctc ggcggggcct
42301 tccgcctcgc gcccgagatc ctcaagtggc tcgaccgcaa gggcgagcgt ggtcacgagc
42361 tggccatgca ggacaaggcg ctggagtttg agaagttgcg tggcgcccag cggatggccg
42421 agatcggcgc gagtgcggac gccgcgtgga acagcggcgc catcgaggcg ctgcgcgatg
42481 ccgtacgcac ccagggcgag aagaccggcg tgcgttgggc cgacgcgctg tcgtcgagtg
42541 tgcgccctgt catcacctac tggttcatgg cgctgtactg cgcggccaag acggcggcat
42601 tcgcggccgc tgtgactgcc ggtgctggct ggggcgtggc catcctgcac gcctggactg
42661 aggccgatca ggcgctgtgg gccggtgtcc tgaacttctg gttcctcgga cgtgtgttcg
42721 accgggggcg gccgtgatcg cggtgccgca gacagccatc gacctggcca agcgcttcga
42781 gggcttccac cgcgtgccga agaccgatcc gggccgtgcg catccgtaca tctgcccggc
42841 cggctattgg acgatcgggt acggacatct ctgcgactcg acgcatgcgc caatcaccga
42901 ggccgaagcg gaagtctatc tggcccgtga cctgcagatg gcgctcgccg ccacattgcg
42961 ctactgcccg gtgctggcca ccgagtccga ggggcggctt gcagccattg tggatttcac
43021 gttcaacctc ggcgccggtc ggctccagac gtcgacgctg cggcgacggg tcaatcagcg
43081 ggactggatg gccgctggtg acgagatcca acgctgggcc tatgggggtg ggaaggttct
43141 tcctgggctc gtcctgcggc gcaaggccga acgggtattg ctgggcggct aattagtcgt
43201 cgaggtaacg gacgaaggtc ttgtaggccg tgaaatcaaa gtttggatca agcgccaaga
43261 tccgagcacg gagcgcctga tgatcggtcc tggcacgtcc acgaccatac gtctgatcgt
43321 tgccttgaat cccctcgaca agatcgatgc aatcgtcgag ctcgtagcga tccaggttcg
43381 gtcggatgac ctcagaaaat cgccggtcag cagtgttata gctcccgctg gccacgtagt
43441 acttgttgaa cagtcgaacg accaactttg cccattccgg ggaatcagat agttcctcga
43501 tctcatccca gatcgcgcaa tcgatatgcg ggtagtcctt cccactgatc cactcgccaa
43561 gccgatccgc gtgcgcctgc aagttcgtag cagtgaacca ggccaaacaa cgcgctgagg
43621 ggtcgctctc cgcggcgtgc tgtatggcgg tctttgcatg gtctgccagc aggccgtaga
43681 tcgatgcctc gcgcgacaga aagtgaatca ggcagacgac aggcgccccg cctgtcgcga
43741 tggtactgaa gtagtccttc tcggcttcaa tctttgctcg gaactgagca gggttccggc
43801 tatacagcag acgcagcgca ctgtagttga tcgcacggtt cttctcgcag cgctcatcgg
43861 tcagtcgaaa cgcgaatttc cacagagcct tgaaaaccgc cttctccact tcgggattga
43921 agcggttgta gtacttgctc tccaggtatg ccttgagctt cttgtcgtcg atcagaatgc
43981 cggatgcttg ttcgaggtct tcgaccaatt cgttcacgat ctgttttgaa aggatcggcg
44041 ccttggtcag gacgccatcg agcgtgttcc ggatcaacgc cctggcggtc tcccgattcg
44101 gcctgtgcag ctgaaaattc gcgttgacta ccgggtgcgc agcgagatgc cgctgccgtt
44161 gcagatgaag caggttttcg cgctcggcaa tatcgaggag ctgtgtctgc tcggctacag
44221 attccacgag cttcgtttcc cagatcggtg agcgctcgtt ctcctgctgc aacctgccaa
44281 catcttcgag gatttctttg gctttggcgt caccgtggag atcgacgagg ttctgcagct

44341 tgaacagcaa gtcgcaaatg gcaaccgacc acagcatcac gacggccgat ctgtagttgc
44401 ccgcggagta gcagctcagg acctccgaga aatactcctt ggtccgcgcg tcaaaaatct
44461 gcgtagcacg ctgttcaatg gagtactcgt caagcattac gcaccctgct ccacgatctc
44521 tacataacca atcaactcag ccgagtagct gccatcccct ttcacagccc ccttgtgctt
44581 cagctcccgc tgcaacgtcg gctcaacggc tcgcgcgaag agatcgagcc tctgttcggg
44641 cgtaaacctc ggtgacgttg cgggcgcctg gaggtagcgc tccgcctgtc gctcgacagc
44701 cggtacgcgc gtaccgtctt gcttgacggc gatgggttgc accacgatgc ggcgctcgcc
44761 attcccagcc gatgtctcga ccatccagag ggacagcagc acaggaccat caacgtcgcc
44821 cgtcacggca ataccgacat cctctggccg cacaatgcgc cagcgcccca gttcttcctg
44881 gaccagcggg tgatccaagc cgagcagctc cacatcatct tggcccgtgg ccgtgtcgcg
44941 gttcaaagtg aagcgggcac gacgaacgcc atccacggtg acgagatcgt aggtttcgtc
45001 gtcgactttg atcagccttt gctgacgttc cgcagccgct gcggagagga agcgcacgag
45061 tcggtccaag ctggacgaca cgtcagagaa cggcttgtag tcatccaggc tgaagccctc
45121 aaggtcttga aacaggtcga acaccacctg ccgcgcctcg cgggaattgg acaaagcggc
45181 ttccagctcg actcgggtcc gcttgagctc gggatcggac aatgcctcct ggtagaggcg
45241 gtcgtagttc aggcgctcag agagctgccc aagaatctgc gctcgcagat cttcggcgac
45301 attgccctgg tcgtcgacct tgccgaccgt gcgagcgatc tcagtgagct tctcatcgag
45361 caacaggaag atgcggccct cgatggtgtc cgagaggacc aggttgtaga cctgagccgt
45421 gtggttctgg ccatagcggt ggatgcggcc gatacgctgc tcaacatcca tcggattcca
45481 cggcaggtcg aaattgaaca ggatgcgcgc gaactgaaga ttgatgccct cccggccggc
45541 tgcggtgcag accagcaccc gaggaccatc cttctgacgg aagcgacgct cggcagcaac
45601 cttggctccg tggtcgccac cccggagcac gacgactccc tggccagggt aggcttgctc
45661 gatctcgcgg gctatcaggt ctaccgttcc gagatacgtc gcgaatacga cgaccttttc
45721 attcgggttc tgtcgccaga ggtaacccaa tccgtcgagc agcttctgcg ccttcgtttc
45781 ccgttggact ggaaagatct tgagcaggtc cccaatacgc agacgctctt cgggcagatg
45841 catctcgacc accgccgatg ccacatcctc tgcgtgcgtc gcggagtatt cgctgccgta
45901 ggggtctgac gccagatcca gcgcctcttc atccagccgt ttcaccaggc gatacttgag
45961 atcggccagc acccgatcca cctcgctgcg accgatgcta tctcggggaa gctggaactc
46021 ctgatggatg agatcccgcg cctcgtccat gaggcgttcg cgaccctcga tgtcgagttc
46081 cttgtcgcgc agcagcgctt cgtggatcgt cagcatcaac agccgtcgtt tcagtgtccg
46141 ccgaacagcg gcgaagctcg atgcggcgat cttctggaag atcgccatca ggaagccaag
46201 cgcacgtccc tggcttccct ggcgacgcgc gaggtcgaag ccgtcctcca gatactcgcg
46261 cagcctctcg tagaacagcc gctcgccggt gttcatcacg aaggattcgg tatgcaccca
46321 gcgacgcgcg aagagcggcg aaccgtcagg ctggcaggcg tcagccttgg tgcgcctgaa
46381 catcacagtg ttcaggcggt ggcggttctc cagcatttcc tccgggcttc tgaacagcgt
46441 cgggttcagc agttgcacca gcatccagaa ctggaagtgg ttgccctggt ggggcgtcgc
46501 cgacagcagc aagaaatcgc gcgcgtgatc tttcaatgcc tcggccagct tgtagttttc
46561 ggtcttccgc accttgccgc cgttgcggca cgcggtcaga tgatgggcct cgtcgaagac
46621 cacaagatcc cagcgcggcg catctagcag gcgtttgatg cgggccggcc gcttcaaggt
46681 gtcgatgctg gcgatcagac ggtcgtgctt ggcaaaggca ttcgtcttcc ggtcggtgat
46741 gtcgccctcc gagccgaaca cctcgaagtc gaggttgaaa acctcgttca gctcgcggtg
46801 ccagttgttc accagaccgg caggcaccac catcagtgca cgattcagct cgccacggct
46861 cgccagttcc cgcaggatga gcgcggtttc gatggtcttg cctaagccca cttcgtccgc
46921 gatcaagtag cggcgcgggg atgcggtggc gacacgatgc gtcagcacca cttggtgtgg
46981 cagcagatcg atcttggctg aggtcagcgc cgaggcgctc tccataactg gcaatgcgtg
47041 cgcttcgtag gacaaccacg cgcggcgcgc tcggtcagcc cctccgtcga cggcacgcaa
47101 gatgcgctcg gtacgcgtga gctcacgcct gacggagcca accggaacac ggcgctcacc
47161 gacgctgaag aatgcgcgca gatagccgtc gcgcgcaggg tcaagaacaa cgccttgacc
47221 gaattcgtgg tgggtgatcc gctcgccggg ctgaaacgag gtcgctgctt ccacgcgccg
47281 ccctcaggtg atgcgcaggt ggaacaggcc cgccggcttg gcgccctcac gcagaaggat
47341 ctcctgcgga tactcgttgg cctcgcccca aaggatgcgg ccgaagtcga gctggcttcc
47401 tcggtgcggc gcctcgcaaa gccaggtcac tgcatcggtg gctgggttgg tttgcattcg
47461 ctcgcgtgcc gatggcatcc agaagctcag ggccgcctga ccctcgtagt acgactggaa

47521 cgacaggacg gcaggcttga tcacgtcggc tagccaaact tcggcgtctt tgggagagag
47581 caggtcgaga ctgccgtcca gcccggccac taccagcgtg tcgccgttgt tgctgggcag
47641 atcgtcggac cagtgcccaa cggcctgtag aaactggcgc aggctccaca cctcgggcgc
47701 tgtgcagaca atgttgcgcg cctcctcgtc ccaaatccag ctcgtgccgc gccgccgcca
47761 tccggtgtcg agcgtttgcc tcatagctcg tattcctcaa agagcgatcc ctgggtgggc
47821 ttcaccgctt ggctggcgtg ccaagtgttg tagatcgaca cggcgcggga ggctgcgttg
47881 cgcgtggcct gttccgggcc gttgcgatgc agccactcca gcagaggctt cagcgccacg
47941 tgcggcttga agttctcgtt cttgagcgtg tccgacgcat tgatgccgct gccgtcgaag
48001 caggcgccga tcagtaccag cgcctggtcc aagtcggacg tcaggcgccg ccggtgcttg
48061 cccgaccact ctcgggcgaa atccagcgga ttggtgcgcg tgaagacctt gttcttctcc
48121 gcgcaccagc cgcgctggac gaagtcgtcc ggtgtggtga tggtgccgcg cagaaacttc
48181 tggagctggt cgcgcttgag ctcgccggcc gcgccgaagg tgcgcagaaa ctggcgtgtg
48241 atcggctcgg cgttgacggg cggcggctcc ttacccttgt cggcgtcttc gtcgatcagc
48301 tggttgatac caacaagcgc gtccttcacc gtgatggtgc ggccttcgtc cacgtagacc
48361 ttgccgtagt ggcgcgagaa gtactccagt gccttgcctc gccgtatcac ctggatgtcg
48421 gcagcgggca agccctcttt ggcgtggttc tccagcattg cctgaagctg gcgcacatcg
48481 gccatgacct cgcgacgcat gcggccccag ctcacgggct tcggctcttc ggtgcgcttg
48541 cgacagacgt gaatgatgtc gtactcgatg gtcttggagc cgaactctcc ttcgcccttg
48601 gtctcgtccg agcggattgg gtaggtggcc tcaaggtaga agcccgcatc gaacagggac
48661 tccagcactg ccacccaggg ttcgtcctcg ctgtggtgga aggtgaaggc gaggatgcca
48721 ccgggcttga gaatgcggtg cgcctcgcgc cagcactggg tgagcagtcg ctgataaaag
48781 ccgtccgggt cttccggctc gcgggccttg ttggccaccg cctccagtga cttgggcgtg
48841 tagtccgcgc tgaaatactc cgggtacttg cccttgagca ccagacgcag ccaaacgtag
48901 aagaagtcgg ccagttccga gtagtgcagc aggccaccga agggtgggtc ggtgatgacc
48961 agatcaaggc tgccggcttc aatccgtgcg aggtccgtcg aggaaccctg aaatacggtg
49021 acatcgccca ccggatcacc agggaagacc ttttcgctct tgccgcttat ttcaccagcc
49081 agcgcgttgt ccttgcgccg gagggcctcg gcgctcaccg cttcccaggg atcgatggcc
49141 caatcgcgac cttcgagaat gccttcgaca cttgaagccc aattccctcg acccaacgcc
49201 ggaaacacgc agttctcaac cacggtcgac ttagggtggt agttgttgtt cgagaacatc
49261 ggctctgggg tatcgcgctg cggattccag aagctgaaca gacactggtt ccgcaaatac
49321 tgctgaaacg cccccagcac atactcacgc acagtccagt cgtagttacc gacttcgacg
49381 atagctttga gcagctgcgc atgcaccagc agttggcgcg ggttgaacat cgtccaccaa
49441 tgcgtgaagc cgtggccttc tcggatgtcg ccgttggcga tgccggtcat gaagccgtag
49501 ggcacggcgg aacgtggcca gtagtccttg aggtcggtat ccttccgcgc ctcccattcc
49561 tcggaggcgg cgtcgtactg gcgggccagt cgctcatcga gaggtgcgaa gaagcggccg
49621 ttgtagggtt tgcgcgcttc gtcacgtttg ggtgcatagc cctgcaccgc ataggcagcc
49681 atcggcccag tcttaccact ggccttcacc gtggtgagca cgtcctgcgc cgtgccgcag
49741 gcgccgcagg cgtagttgga tttcttgggc accgtgccct tgtcggtgcg gaaggtcacc
49801 cccgtctcgg ggcaggtcac ctgatccggc agggctccgc gcacctccag caggcggatc
49861 ttggcggcgc gcgcatggtt ccagcgcgcg gtagaagccg cgtcatcttg cgccgagccg
49921 ccgtagggct ggccgctgac atcctgcttg gcctcgccgg ccagccactg cggatgcacc
49981 aacagactca gctccacctt cttgttcttg cccttgccga gattgactag ggctgtgtgg
50041 ccacaatgcg ggcagatcac gcccttcttg cggtcgagga cggagaatgg gtattcggat
50101 ggggcgacgt acagcggcac atcgggcgcc atccgtgccg cttcttcctc gacgtggaac
50161 tcgccgccac acttgccgca ggtgtgctcc cagtgtttga cgctgagagt cttcactgcc
50221 atcacggggc tcgacatgat tggtgtccgg tggccgcagc cggtcacctg acagggacca
50281 tgcttggccc agaacgtgta gatgatctcc gggccttcgt agcggtagtc cttgcgctca
50341 tcacgcggga tgacgagcgg gtcaaagtcc gcgggcatca ccttcttagt cggcaggtgt
50401 gtccatgtgc ctttctcacc ttccgggccg tcacagtagt agtaaggcat gatctgcggc
50461 ttgacctcgg cctcgacgtc cgcgaggagt ttctttactt cccccaggtc gacgttcgcg
50521 agttcctgct tgaccacgaa ccacgcgacc gggtttaggt cgttgccgac catctgcatg
50581 cccaggcgcg atccttccac cagcgtggtc ccgccgccca tgaagatgtc ggccaccttc
50641 aggtgcttga acgaaccctt cttctggtgg ttggcgtagt agttgtccca caccagccgg

50701 gccgcgtgag ccgggtcgtc cggcgccttt gccgctgccg cgatgagcat cgagcggaac
50761 acgctggaac gccgccgcgc ccaccacttg gacatctggt agatcggctt gcctgcattg
50821 ccctcaatca ctgccacctg gttgacgggc aggatcggga agtccacctc caggcaggtc
50881 ttgggacggc ccggatcgct gaaatcgacc gattccaagg cgaccgcctt gccggcgccg
50941 acagctttgc cgacttgctc ccgcagttgc gccttcgcgt cttgtttttg ttcccagaaa
51001 ttcatcttct gaccttttat ttcgtgactt ctacgaaggg cgacagcacc tcgagcagcg
51061 aaagaccgcc gtgcgtcagt gttggatagc cgccctggct gcgccacttc catcggccca
51121 gagccattag gtgtgcgcca tgcggactgt tgatttggag cgcgacggga ggaacgaacg
51181 ggccggtgtc gccggaaccc gacgcgctgc gtccactgga gaaggtcttc ttgaggaact
51241 gaccgacctc gccatccgcg tcggggaaat ggccggaggc ggcatagccg tggtcggatg
51301 tgatcaccag ccgacgtcct gtggccaagc gctcgacaaa ggcccagaaa tcatcgctgc
51361 tgagttgctg ggccgcatcg cgggtcagga tctcaaggcc ctgaccggcg cctgcaccgt
51421 catgcacttt gctgtcgggc cagtggtgcc agaacaccca gttgggcgtg ctgttgatca
51481 gcccctcgca gtccttccac ggcatgtcga cgcattccgt gtgggcgggt tgcagcttgt
51541 gcgctaggcc gccgccgttg ttctgcagct ggctgcggct ggtgaagccc aaggcccgcg
51601 cgaactcatt ggtctctccg ggcagctcgg aggcgttggc gccgacctca tgcaaggcaa
51661 agccgcgctc tttggctccc tgcatcagcc atgggagttc gcgcagcgag aggccatcca
51721 agatcagcac cgccttggcg ctgtacggct ctttccacca acgcaccggg cgatctgatg
51781 tgcgttcgac gacgtcaccg aaagagctcc acagatccca gccactggtt gccaagaagt
51841 gatcgagcgc accgatcgcg cgatcacgct tgacgacctc gctgggcgcg ttaccggcag
51901 ccagcggctt ggacgcaatc tcgacggcac tgtcgatgat gactcgccac gcttcggcgg
51961 ctgattcctt ggtcagactg ttgaggatgt tggcgtcgag acccatcagc tgtcttcctt
52021 ctcgaggctg agctcgaacg tcatcccgtc aggcagcttc ttcagcagct ctttgagctg
52081 cgcaccggtc gcggccgaca ctttgatcga tacctccgcg acttgcgtgg ctggaccaat
52141 accccaacct tcgagcttgc cgatcagatt gagcggcgag gttggtgggt tgctcagcgg
52201 aatgcgtggc ttggcccccg cggttgtcgt gccaccgccg aaaatgcctc cgggaggtgg
52261 tgtaccgccg ctacctgtcg tatcgtcacc cggcgtaggc ggtgtcggtg gtgtcgggcc
52321 gccgccaaac agtcctcccc cagtcccgcc ggcgggcggt ggagctgggg gagtggcgcc
52381 gcccgtggtt ggcactgccg agggttccat caggaacacc tcgtcgagtt gcctgccggt
52441 gtagaagagc tttgggcgca ggcgtttcca tgccgcgtct tcgtcttcac cggcgtgggt
52501 ctggaggtac tccaggccgc gcaggttaat ggcaattttg ccgcgcgcgc acaagcgaag
52561 gatgcgctcc ttcatcgccg tttcgccaag ccagggaatg cagtcctggc cggcggggcg
52621 gggttcctgc agttcgcgca gcagcttacc caagggggcg ttgtcggccg cggcttcgag
52681 cacgagggct tcgaagtcct cgggcacgaa gagatcgttc gtcagcgcct cctcgatggc
52741 ctcggggatc tgcgcccctt gctttttcag gctttcgacg ctgaacacac actggtgcgg
52801 gttctgatgg tcgtagcggt gcaagacggc gaagcggtcg aagcgcttct tgaggttgtc
52861 acgcagcgtg ccttcgaact ccttgtgcag cttcttgtac tcagggttct gtccactcca
52921 ctcctgcgcc ttcatctcgg cacgggcgag aatgagcaga tcacgatcca ggaacgcgtt
52981 ggccgaaccg gagcgaggga gcaagaagcg aacggtgttg cggcgcttct gaagatggtc
53041 tttcagccaa cgcccgagcg tctggtcgag cttctcgggc tcttcgggca acactaggat
53101 gggcagccga tcatcccagc gctcgggctg ctcggcttca tcgagcgcgg accacggatc
53161 tgtctgccaa gacttcggca gtgcgatcac gcggaaggac ttggccacct catccgagcc
53221 gccgatcacg taccgaatct gcttggcaag ctgcgcttga tcggagccgt cggtgaacag
53281 cttgtcgttc cgggcgcagg ccataagctt ggcgcgcggg ttttcctctt cgcggaagac
53341 cagcctgggg ccatcctggt ggatgttgaa gctgttctcg acgatggtag ccagctcgac
53401 ctggaaggcg ttgtcgtcca ccggcttgtc gcgcgtgata tcgacttgca gcgtggcggg
53461 ctcagcgccc gcaagattgc ccacggcgat ggagcgaagc cacagcgcgc caacgatctc
53521 ctgaaggtgc ggcgcgcggg tcgcatggtc atagacagcc tcggtgaccg agatgatgtt
53581 ctgctgcgcc ttctcgcgca gggtgcggtg gtgttcgtta gataccgagt cgagcagggc
53641 gccgataccc gaggcatcat cgtccagccg gaaatcggcc gcggtcagca ccggcaccgc
53701 atcaccccgg ctcttgtaca gattggcgag gatgcgaatc atgtcgcgcg tctcctgggc
53761 gtccgtcgcg atcaggactt gttcttcaag cagccgcagc aggtgtgggg cgaagggcca
53821 cgactcgaca aactccctgc gcttgcggtc gtgttccgcc ggcgggacgt cgaggaggcg

53881 gaagtattcc gagacgtgct gcgcgatcaa cgattcaatc gtcccgttcg caatctgcat
53941 gcggttgtca aacagacggt gcagcagcat ccgccgccga tcttgctgga tgcgctcggc
54001 gttgccccct gctttgaagt caatggcgac tgggttgacg cggtgtacct gctggtaggc
54061 gtcgctgccg ccgttgcgca cggaaatcac caacaccagc agatcgggcc gttccttcgc
54121 aatctccgag aggatctgaa tgaagttaaa cgcccagttc ttccagggat actgcttggt
54181 gttcgtcagg ccgtcgtacc aggtctggaa ctcgtccagc agcagcatcg tcggcctatg
54241 ctccagcatc tcgacgatga gcttgtcgga ggggatgtcc gtcttggccg cccccatacc
54301 ctcccactta cccttgatgt aggtgccgtg gggatgacgc tcgaacagca gatcccaaag
54361 aaacttgtac cgctggcgat gcaggctctc gccaatcaca tacatgccgg agcgcaaagc
54421 gatctttccg atattcgcgt cgccaagcgt gcccgcccaa gcattcagcc aagcacctgt
54481 tgaggccgcg tcgttcacag cgtggtacag cgccgccatc agatgcgact taccgaggcc
54541 ccgctcgccg atgacgacaa caggacgccc ctggttggga ccgacggcct cgatgccttt
54601 caacaagtca tgcgtcgggt aggtgatctc caagaactgc ttcgcggcga tctgtgtcgc
54661 gccggtgctc gtgtcgttgg agagctcaat ggccgtccct ttgaggcgct tgcctcgaaa
54721 ttcttctcgt agagtcagtc caagcattac ttacgctccc catcgtgcga ttggtccgtg
54781 gccgcgccgc cctcgtcagg tccggtaccg agcacctccc tcgactgctg cttgatccag
54841 cggtcgatgt caccaagcga gaaccgccac gtaccgccga ccttgaaggc ggggatcttc
54901 ttggctgcag ccagcctgta gatcgtccgc tccgtgacct ttaacaggcc gttgaaaaac
54961 tcccccgcag ccgcccgtgc tgccggtacc atgtgagcgg cggcgacgac gagacagcag
55021 acaccgaaga tgagaggcac acagaacttc caaggggcga tgttcagcta catcagcctt
55081 gaagagcggg taccggccag acacccgctg cgcaagctgc gcgcgctggt cgatgccttg
55141 ctggccagca tgagcgcgga attcgaggcg gtctatgccc gccgtggccg cccttcggtg
55201 ccgcccgaaa tgctgctcaa ggcgttgctg ctgcaaatcc tgttttccat ccgcagcgag
55261 cggctgctgg tggaggccat cgactacaac ctgctgtacc gctggttcgt gggcctgaac
55321 ctggaagaca aggtgtggga ccactccacc ttcagcgcca accgccagcg gctgttcaac
55381 gaagacctcg cccgcgtgtt ctttgagcgg gtcaaataca ccgcggactg ggcgaagttg
55441 atcggtgacg agcacttcag cgtcgacggc acactcatcg aggcctgggc ctcgcaaaag
55501 agcttcaagc gcaaggacgc aagcggcagt gacgacggcg caccgcccca gggtcgcaac
55561 cccgaggtgg atttcaaggg cgagacccgt cgcaacgaca cccacgccag cacgacagat
55621 gccgatgcgc ggctgttcaa gaaagctgca ggcgacaagt cccgcctgtg ccacatgggt
55681 cacatcctca tggacaaccg acacgggctg gtggtggacg tcgaaatcac ccatgccagc
55741 ggcacggccg agcggcaggc cgcactcaag atgctccagc gccaaaagcg caaagccggc
55801 cgactcaccg tgggggcgga caagggctat gactgccgtg ccttcgtgca gggctgccgc
55861 aagctgggga tcaccccgca cgtggcggcc aaagccaagc actcggccat tgacggacgc
55921 acccagcggc acgaaggcta caaggtgagc ctgagggtgc gcaaacgcat cgaggaggcc
55981 ttcggctgga tcaagaccgt gggcggtctg gccaagacca agctcatcgg gcatgccaag
56041 ctggcggggc aggcgctgat gtgctttgcc gcgtacaacc tcgtgcgcat gggctccctc
56101 ggtggctggt gggatgcgca tcatgcgtga ttgcgggggt cagtgcgccc aaaatgggcg
56161 agcagccccc aaagggggag cccaagcggc tgtcggagcc gagaaaaacg gcttgcgccg
56221 gcctcggacc cacgcaaacg ggtacatggc cgcttcgatg gacacttttt caacggcctg
56281 ttaagtactc ggcgacctgc ttgatggtga ggatctcgcc ctcgcgggtg cttataggca
56341 tggtcatagc caagttgagt caaaccgcga caggatagta caaaattttc aaagcagcaa
56401 tgacaggtga ttgatttccg aaagattttc taacccttca agggggaaga ttcatcaggt
56461 gagccagagg gcagagagac ggtccatggc tttcgtgtcc gggccggcgg cggacagccc
56521 gctgatcagg ccgcaagtgg ccaaaccatc gcatccacgc cctcaaggcc gaggcgttac
56581 tatttggatc gggaagcgga cgcgggttcg gttcccgttt tcaccaccaa ttccgggaat
56641 tgtcaagacc attccagacc aactaagacc cttttaaatc agggccttag ttgcctcccc
56701 ccccaacaaa ccaaccggtc tccgttggcc c
//

```
LOCUS       Bath-R2               45605 bp    DNA     linear   PHG 16-FEB-2025
DEFINITION  Bath-R2.
ACCESSION   Bath-R2
VERSION     Bath-R2
KEYWORDS    .
SOURCE      .
  ORGANISM  .
            .
FEATURES             Location/Qualifiers
     CDS             complement(68..982)
                     /ID="LHBQFDQA_CDS_0001"
                     /transl_table=11
                     /phrog="67"
                     /top_hit="No_MMseqs_PHROG_hit"
                     /locus_tag="LHBQFDQA_CDS_0001"
                     /function="other"
                     /product="DNA methyltransferase"
                     /source="PHANOTATE_1.5.1"
                     /score="-2254.479080792154"
                     /phase="0"
                     /translation="MHDNPPASQPASQPASQPASQPASKVCHACGRAIADSIRLVEFSD
                     GRCRLFQNDFREVASLITPGSVAAVITDPPYGSGGFTVKDRLKSSKTKYVSSDASYQKT
                     LPDIDGDSLHPEAWKELMKAACAVARSVLMNGGVLAMFIDWRNKPQLQEIIHGSGLALR
                     GCVAWDKGNGARPMKNGFKNQAEYLLWATQGPTPTREPPVYLPGVLRHSTLSNGKVHIT
                     QKPLALMEDIVQVCPPGGTVFDMFMGSGTTGVAALKHGRRFIGCESVPEYFDASVRRCR
                     EACPEEQTGRPESPSANPAQSYR"
     CDS             complement(1085..1768)
                     /ID="LHBQFDQA_CDS_0002"
                     /transl_table=11
                     /phrog="No_PHROGs_HMM"
                     /top_hit="No_MMseqs_PHROG_hit"
                     /locus_tag="LHBQFDQA_CDS_0002"
                     /function="unknown function"
                     /product="hypothetical protein"
                     /source="PHANOTATE_1.5.1"
                     /score="-16.760604620385724"
                     /phase="0"
                     /translation="MGIQVSATGTASSTGASLSITRPAGIATGDLLLLAVALDAGTFGS
                     GAAIATPSGFTPVPAGSVGFGSNGRSQLAAFYKVASDGEPASYTLNFSAGGFPSYAANA
                     VICAFGGVNTEAPVDASAGVSGGSSSSLAAPSVTPSEGEGDDLLVGLWGGTASVGASTW
                     PAGMGDTLVANAGGGSLLMADQQLVSAGATGTRTLTASAATTWGAVSLLLLPDRSAALP
                     LWENF"
     CDS             complement(1768..2721)
                     /ID="LHBQFDQA_CDS_0003"
                     /transl_table=11
                     /phrog="8147"
                     /top_hit="No_MMseqs_PHROG_hit"
                     /locus_tag="LHBQFDQA_CDS_0003"
                     /function="tail"
                     /product="tail fiber protein"
                     /source="PHANOTATE_1.5.1"
                     /score="-402.25196888253214"
                     /phase="0"
```

/translation="MPTRFDRYRFKDGVTPLSEDTFNAILQDIDLRIAALEEVRISWQA
AVTLLTDQGLLRINEALAPAIETLQYQIDHIVELASQVQVDRILDAPDQVTDVHIGNRT
ADPALVPVNNTGTLTQWLGRLANRLKAITGAANWYDAPATTLAAVAATLASQAAQLATA
AAHASNVSNPHNTTAAQVGALPVGGGNLLGALGLSGYPISGVKTLGFQAEYDNGNSGTA
KTLSLVNGQKQKLKLTASTTLTVSSTGAPVGNYVIRLIQDATGGRAVTWAGLSGSRWLG
RATAPSVNAAANGESLLSIYWDGASMIQSLAKVGAA"
CDS complement(2705..2893)
/ID="LHBQFDQA_CDS_0004"
/transl_table=11
/phrog="No_PHROGs_HMM"
/top_hit="No_MMseqs_PHROG_hit"
/locus_tag="LHBQFDQA_CDS_0004"
/function="unknown function"
/product="hypothetical protein"
/source="PHANOTATE_1.5.1"
/score="-16.29161123295673"
/phase="0"
/translation="MAKQTTVNAIDPTLIDDDGQYRVVLAERIVVDGVVLYPGWDIILK
GDVVKSNREAIEHADPI"
CDS complement(2904..5075)
/ID="LHBQFDQA_CDS_0005"
/transl_table=11
/phrog="17470"
/top_hit="No_MMseqs_PHROG_hit"
/locus_tag="LHBQFDQA_CDS_0005"
/function="unknown function"
/product="hypothetical protein"
/source="PHANOTATE_1.5.1"
/score="-4701891.743990446"
/phase="0"
/translation="MEKTVIFRDRQEFQAADPNALQAYARDSLDHVVADGISAQKHYTG
FGVGAVSATEVEVQPGRYYNGGAVYVAEQPVSINLFQYIPLVAKKIVAVVLWGQEVDTS
VEPRDFLVDLQTGATEPQAVAMQRIRKCEVNPLSGQESADPQPPVIQTGTLAVAHVYLT
PAGIERIEMQTQSLLPNGYDQERRLDGIEIWKAAAEPRIASIATDLAALAKKSSDKADR
AMFVEIAQDLASVKERLQLPSSYSSYQSDAFADTAKSNAGHVGYAARIDHGLLFPFAAS
IQANLALFNPIDAGVSVSGQNVVLPAYTHAARIQTQGYAGDLSISQYQVQTHTVREQKI
VSWQKKYGWHGNFLTRWYARNVWAKLGARYQWTLPWHGYFEQHETTQYVDEVTTTGYNG
AMVAQSFLVANALWLTRLGLFFTQIGANGDVQVIVCETDGGKPNLEKTVASITLPRASL
KAYPSETVIDVPHVLLEGGKRYAIVLITQGDHRVATVSGNAYTQGTLFYGSDGDYFVGD
LTRDLMFTVYGAKFARVRTEVQLQSVSLSGGLTDLAIAAQHVVPEGTELRYEIQPSGSG
AWYPLGDPSLVLSTGPNLVNLRAVLLGTSDLAPAFVLTNNAIQASRPDTSFVHWSTARS
VASTTSVTLKLLVAHWDAANHTLTPRIVTGGSNEVAPSTTVITDEDGAKRFSYTFTIPA
STAYEIKLSGTRNAASQPFAVVERIDVAA"
CDS complement(5091..6401)
/ID="LHBQFDQA_CDS_0006"
/transl_table=11
/phrog="34934"
/top_hit="No_MMseqs_PHROG_hit"
/locus_tag="LHBQFDQA_CDS_0006"
/function="tail"
/product="tail protein"
/source="PHANOTATE_1.5.1"
/score="-3763.3190337264527"

/phase="0"
/translation="MLAALLGQHQAKPMAELLQPSLRDARGLALDALIERISRLDQSAL
LVYLVDQVHPSALPHLADQFHVMGLEGWDTVSTDTERRALIKSAIAYHRLKGTLAGLTW
AGSRVGLSILRAITPPAKTYLAPALTRAERDAFLSGYPQLRLYRYRTRGVRIGSGWYCS
AAFVKGRHYPVVTDAILRLGWRAFLWHRSGNDSKSSGADSKSNGVETPVTTLVREIQRA
GAEAELSLAIRKPGAAGLGTYPGRLVPRSYLVRQDGGGRLFNVRLSTPYLDFDESVHTH
AVRPGLDPIDIRYTTIAEQGIEHGAMLGRFVAGHLADNGARDRLYRRFHLFDPAVPVPR
RGRSTHVGAMKLGMPAYTAELAVSMPARRPPRLIGRFATGYWAPASVSRLERGREAMAL
ASAFRDRVWLDTHTRKQVRAGYAVRSGEILSGQFITV"
CDS complement(6402..6884)
/ID="LHBQFDQA_CDS_0007"
/transl_table=11
/phrog="No_PHROGs_HMM"
/top_hit="No_MMseqs_PHROG_hit"
/locus_tag="LHBQFDQA_CDS_0007"
/function="unknown function"
/product="hypothetical protein"
/source="PHANOTATE_1.5.1"
/score="-30.64779541795652"
/phase="0"
/translation="MTQQRVKIPNGEDWLAGWAVPSAGGQLGFSESVGVADPDTGMAAA
VRDGAPAASDPGLVVRPVPVPLSGGDAAATVVEHSVHTTTLLPANPGRRGALIFNESVE
RLYVKCGGFASASAFTVCLGPFEVWQVPAGYGGRLTGQWSMLSGLADLGRARITEY"
CDS complement(6881..7936)
/ID="LHBQFDQA_CDS_0008"
/transl_table=11
/phrog="6"
/top_hit="No_MMseqs_PHROG_hit"
/locus_tag="LHBQFDQA_CDS_0008"
/function="tail"
/product="baseplate wedge subunit"
/source="PHANOTATE_1.5.1"
/score="-1124.195090151955"
/phase="0"
/translation="MTGKTLYPAQVERLLIDLIAYRETLARIGIQEAAKLNLVHFSRAP
MLDYLGELVGVTRLPAQSARTVLRFSLERPAATSVVIPKGTRVAAGGTDSRSRVAEFAT
DTEVTIPAGAIAVEVNATAAGAGPSANGILPGGIRDLIDVPANGLSVTNLGTSYGGLAA
EDDERLRMRIKLAPERFAAAGPALAYRWHVLSVSQAIVDVGITSPAPGRVNVYPLTASG
LPDPALLDAVRAVLSQDKVRPLTDWVTVLPPSRAPFAVSARVRAFGAVSEAAVVAGVRA
SLEGFAAELRSTLGRDLVPSQWIERAQKVPGVYRVELDAPGYRVLKAHEWPDCEEVSVI
FEGVGETQGFV"
CDS complement(8011..8397)
/ID="LHBQFDQA_CDS_0009"
/transl_table=11
/phrog="43"
/top_hit="No_MMseqs_PHROG_hit"
/locus_tag="LHBQFDQA_CDS_0009"
/function="tail"
/product="baseplate wedge subunit"
/source="PHANOTATE_1.5.1"
/score="-19.44075991295892"
/phase="0"
/translation="MTSVIGTIASVNWQPRLGQPGDIVQDAADIDQAIRIILTTPKGSD

PHRPEFGADILQYVDRPEIEATPYLIAEIADAILLWEPRIELISIRPSFGLAQVTLAIT
WRFRGVDSKSSGGDSQTRTTEVTL"
CDS complement(8394..9872)
/ID="LHBQFDQA_CDS_0010"
/transl_table=11
/phrog="1980"
/top_hit="No_MMseqs_PHROG_hit"
/locus_tag="LHBQFDQA_CDS_0010"
/function="tail"
/product="tail fiber protein"
/source="PHANOTATE_1.5.1"
/score="-2669.531188978879"
/phase="0"
/translation="MASLKQFIANAKAMLPAVTGKPGWLDAPSMHLEQVTGQIKALLGK
ADQFAAPDIDLHQADAALKAVLGEAASTAPPAISLHDTAAALKAVTGKASATAAPDITL
AAAKAHVDAANNPHATTAAQVGAMAITHPANAISGFGNFVYGLGPAQSAGSASTVARSD
HVHPFPNAVQIGAIPDTHFASVLEGFGVSASPLAAAASPGVSYKIAREDHVHPFPTAAQ
VGAMATTHPANAITGFGASAQALAASGSAGSASTVARSDHVHPFPTAAQVGAMATTHPA
NAITGFGSSAQALAATQSAGAATTVARSDHRHPYPTAAQVGAAATYGDAANQFNADFFQ
ATQDVGYRFANSPGSYAMTSTDGGVRVPVGGGFHVRNTSLAYAPCYASAFTVSSNRRLK
RVLGEVRHALERVRALQPIRYRLEADGPQGRIELGLIAEDAREVLPEVVYPVTDGANGP
DGASLSIDYGRLAVLALAAIRELEARVEALEAAR"
CDS complement(9872..10297)
/ID="LHBQFDQA_CDS_0011"
/transl_table=11
/phrog="9992"
/top_hit="No_MMseqs_PHROG_hit"
/locus_tag="LHBQFDQA_CDS_0011"
/function="tail"
/product="baseplate assembly protein"
/source="PHANOTATE_1.5.1"
/score="-25.162598080266633"
/phase="0"
/translation="MPEVGEQVACIMDEHLEDGTVIGAIYSGPDPVPEGVDEKWYGVWF
EDGSILYYRKDSHQLLIDLTHMQGTVLLKAQSVTVEAHTVNVQAGTAQVEADTVAVTAS
TLTADTQTASISGTRVSVAAPAIDLTGLVNVDGDIDL"
CDS complement(10315..10425)
/ID="LHBQFDQA_CDS_0012"
/transl_table=11
/phrog="No_PHROGs_HMM"
/top_hit="No_MMseqs_PHROG_hit"
/locus_tag="LHBQFDQA_CDS_0012"
/function="unknown function"
/product="hypothetical protein"
/source="PHANOTATE_1.5.1"
/score="-0.9979597456364732"
/phase="0"
/translation="MGWSPPSTRRSRGSRRSSPTRTAWCRTGSACPARRA"
CDS complement(10488..11531)
/ID="LHBQFDQA_CDS_0013"
/transl_table=11
/phrog="52"
/top_hit="No_MMseqs_PHROG_hit"

/locus_tag="LHBQFDQA_CDS_0013"
/function="tail"
/product="tail protein"
/source="PHANOTATE_1.5.1"
/score="-23566.804127828385"
/phase="0"
/translation="MTPRLDSKSNGVETPVEDPVYSVIYDNVNITADISGLVTELIYTD
HEHGESDSVELKIEDREQRWKNEWYPDISARLSVSIGYADGRRLDCGDFELDEIEFDVM
PDTVRIKALATVITPKLRTPRSYGYDDTSLRAIVQQVAARNGLTVRGDIEGIALDRVTQ
NHEKDLAFLTRLAEDYGYAFAVRGDALDFHSIATLEAAPSAFSLHRRQLKHCTLSEKSE
ATYPDGKVSHHDPDQKALVYDWDEKGNIKTGDTHALLKRAKHRGIAKRKASSHLHQADK
KQLSGTLGLVGDTRLVAGINLDLTGLFKLDGKYHVTTSTHRLDRSGGYASEAEVYRVVA
IHSLESP"
CDS complement(11528..11773)
/ID="LHBQFDQA_CDS_0014"
/transl_table=11
/phrog="46"
/top_hit="No_MMseqs_PHROG_hit"
/locus_tag="LHBQFDQA_CDS_0014"
/function="tail"
/product="baseplate hub"
/source="PHANOTATE_1.5.1"
/score="-5.328534032815525"
/phase="0"
/translation="MTTYLSYITQEGDRWDGLAYRFYGDPFRYEPLVVANPHVPIVPVL
PSGLTLAVPVLAKSDSTPSVESLPPWKRGVPAGGVA"
CDS complement(11770..12339)
/ID="LHBQFDQA_CDS_0015"
/transl_table=11
/phrog="788"
/top_hit="No_MMseqs_PHROG_hit"
/locus_tag="LHBQFDQA_CDS_0015"
/function="tail"
/product="tail protein"
/source="PHANOTATE_1.5.1"
/score="-113.2776959364072"
/phase="0"
/translation="MSYLQLGNVQLDLITWLGGFEAGYGYAYASHDIIEGKSHLQWTGD
QLETRALSAQLHARFCDPRAEFEKLKTAASRHNALAMFFANGTYEGRFVIEELQRTLER
TDRFGNVYLMQVRIQLKEWAEPTSSSLAGSLNANARAQAEAIAENAPARQLKDPSFGPT
ASQVRLKELAAKYDTFTRQPVPGLIR"
CDS complement(12404..12532)
/ID="LHBQFDQA_CDS_0016"
/transl_table=11
/phrog="No_PHROGs_HMM"
/top_hit="No_MMseqs_PHROG_hit"
/locus_tag="LHBQFDQA_CDS_0016"
/function="unknown function"
/product="hypothetical protein"
/source="PHANOTATE_1.5.1"
/score="-1.7001395058393998"
/phase="0"
/translation="MPGRGRATGEDSKSSVAETVREWLRDDRGIVAPTEAASAGLF"

CDS complement(12504..15485)
/ID="LHBQFDQA_CDS_0017"
/transl_table=11
/phrog="5690"
/top_hit="No_MMseqs_PHROG_hit"
/locus_tag="LHBQFDQA_CDS_0017"
/function="unknown function"
/product="hypothetical protein"
/source="PHANOTATE_1.5.1"
/score="-6819697530.4428835"
/phase="0"
/translation="MAKELAFGIVIGAALSSAFTAAFGNAKKTIDTLGASVRDLTDKQK
TLGATIQKSGRLAQESLAPLHRDYERLGRLIDEIRRRQEALTASLARGAALKQERADLR
GQALETVGTGAALGAPVVASVRLAGNFQDQLRDIAITGEFTTAQENRLGAAVRESALKW
NQTQAEIASGIGVLVAGGVQDAQALDRYTPVLAKAATATRASMADLGSVLLAFDNNLKV
SADQSESALNMLAYAGKRGQFEIRDSAKWLPALAPMFQTLGVTGKEAVAEIGAALQIAR
KGAGTNDEAANNFRNFLAKLTSPDTLKDFDKAGIDLQSSLKNAAKRGISPMEAMMDTIT
AYIGSKGPQAAAAFKQALSLEDAQKRAEALQALSGSFRLGELFQDMQAMSFIRPMLANR
AEYKDIKQGALGAANQDLIGADFQKRTQGFNESLKAFRIGMSEVGLVVGEALLPPLTDL
LQTVRPVIREFGQFASAHPGVIRGVVGLTAGLLGGKLAVLAVRYAVNLLVSPFNALATA
TQLVLGKWTLLKTALQFGPLARAGGALSAAGSAALGAGRALGGILLSGFRLAGAATLGF
GRSIASPLLSGLRLAGAAAAGFGRILIGSFLAGLRLAGVAALELGRFSAGALLSGLRLA
STAAAGLGRILIGSALAGLRLAWLAALELGRILGGALISGLKLAAQAVMFLGRALLFTP
IGAAVAIIAGAAFLIWKNWDALKARFAPLWDQVKGIFGRALSWFKTLPDVFKAIGSNLL
EGLRSGIMAKWESVKAGLSSIASGIKDTFKSALGIHSPSRVFAGYGADIGQGLIEGVTG
QKDAVAETLGKLVRFPQPPVIRVHTELGSSTGPERSGLAPVSLDAGLGRELGRVLPFAK
PDSKGNGADSKGNGVESPAATTDTPARLLGRKFGDAALRVRQGSDVASGADGHARTFRS
VHQPQGRSGEQVVIHFNPTIHVSGAEDAGKAKAAVTEALELSLRDFERVAAQASHARAR
TGYR"
CDS complement(15478..15663)
/ID="LHBQFDQA_CDS_0018"
/transl_table=11
/phrog="No_PHROGs_HMM"
/top_hit="No_MMseqs_PHROG_hit"
/locus_tag="LHBQFDQA_CDS_0018"
/function="unknown function"
/product="hypothetical protein"
/source="PHANOTATE_1.5.1"
/score="-1.5328897130203238"
/phase="0"
/translation="MMDLPATQNATTAPKSAKYAIQGARCSMFSILSASAAKGFGGFTF
QSTLGCTPGEARLTHG"
CDS complement(15765..15896)
/ID="LHBQFDQA_CDS_0019"
/transl_table=11
/phrog="8286"
/top_hit="No_MMseqs_PHROG_hit"
/locus_tag="LHBQFDQA_CDS_0019"
/function="unknown function"
/product="hypothetical protein"
/source="PHANOTATE_1.5.1"
/score="-3.0000715654490726"
/phase="0"

/translation="MALLARWFRFQPSEIDALELEDFLTWWDLANQQIRLEAGPAGA"
CDS complement(15856..16161)
/ID="LHBQFDQA_CDS_0020"
/transl_table=11
/phrog="166"
/top_hit="No_MMseqs_PHROG_hit"
/locus_tag="LHBQFDQA_CDS_0020"
/function="tail"
/product="tail protein"
/source="PHANOTATE_1.5.1"
/score="-7.978973747805623"
/phase="0"
/translation="MNKIKLHEPLKTGDGRTLTELTMRAPKVKDLKAAQRFGGSDADVE
VALIASLVGVVPEDLDELGLADYRRLQDSFRRFSDPDGGAVEGDGAAGPVVPVPAQ"
CDS complement(16204..16551)
/ID="LHBQFDQA_CDS_0021"
/transl_table=11
/phrog="585"
/top_hit="No_MMseqs_PHROG_hit"
/locus_tag="LHBQFDQA_CDS_0021"
/function="unknown function"
/product="hypothetical protein"
/source="PHANOTATE_1.5.1"
/score="-13.514742962280124"
/phase="0"
/translation="MRLDWNLARSVLEAAEALGDEKDRVAPGAFPGVAEEVAIEHFRLL
CEAGLADGYPQGRSPLFLTRLTWSGHQFLATLRSRTLWSRVKAEAKDRGLALSFEVIKA
LAAKLVGQLVD"
CDS complement(16553..17074)
/ID="LHBQFDQA_CDS_0022"
/transl_table=11
/phrog="55"
/top_hit="No_MMseqs_PHROG_hit"
/locus_tag="LHBQFDQA_CDS_0022"
/function="connector"
/product="head closure"
/source="PHANOTATE_1.5.1"
/score="-224.171032097245"
/phase="0"
/translation="MAKIEINRLTNANVYLDGASFLGRAEEVELPDIKHKMEEHMALGM
VGTLEAWSGIEKMTAKFKWSSFYKEVMAKAANPFKSVAVQVRGSLETYATAGRIAETPV
VAHLIGQFKSIPMGNYKQHKNVEQTNEMAVHYAKLVVDGAEVFEFDALANIYKVNGQDM
LANYRANIGG"
CDS complement(17062..17154)
/ID="LHBQFDQA_CDS_0023"
/transl_table=11
/phrog="No_PHROGs_HMM"
/top_hit="No_MMseqs_PHROG_hit"
/locus_tag="LHBQFDQA_CDS_0023"
/function="unknown function"
/product="hypothetical protein"
/source="PHANOTATE_1.5.1"
/score="-2.0886671365632847"

/phase="0"
/translation="MRDLQGQFGVKIRRHRSLAFRSVRTSIWQR"
CDS complement(17162..17368)
/ID="LHBQFDQA_CDS_0024"
/transl_table=11
/phrog="No_PHROGs_HMM"
/top_hit="No_MMseqs_PHROG_hit"
/locus_tag="LHBQFDQA_CDS_0024"
/function="unknown function"
/product="hypothetical protein"
/source="PHANOTATE_1.5.1"
/score="-3.347311775907478"
/phase="0"
/translation="MDEVWLRNLNRFQALTAKMSRKRACFPAGTGRTPCEARLAAVLDL
ALAKLGGSVSPEHPGRLERAFPG"
CDS complement(17368..18825)
/ID="LHBQFDQA_CDS_0025"
/transl_table=11
/phrog="23"
/top_hit="No_MMseqs_PHROG_hit"
/locus_tag="LHBQFDQA_CDS_0025"
/function="tail"
/product="tail sheath"
/source="PHANOTATE_1.5.1"
/score="-148694.63687791376"
/phase="0"
/translation="MPANFLHGVETIELDKGPRPIRGVKTAVIGLVGTAPMLDVDPADR
SLNRIRLVANDRDAARYFGQNRAGFTIPSALNAIFDQGNGPICVVVNVLDPASDTTAVA
DEAKTFDAATGKLTLAHPQVSNVVVKHTAGTPTYVLNTDYTLDAANGTITRKAGGAIAS
GQSVKVSYSWLDPSKAVNSDIIGAVDGAGNRTGMQAWLNAYQELGFFPKILIAPGFSTV
NAVATELNVLAGKLRAIALVDAPIGTTVQQALTGRGPAGAINFNYSSDRLGLCYPHLKV
YDSASGAEILEPMSPRLAGRIAATDQDKGYWWSASNQEIKGIVGLELPLTAMINDPASE
TNLLNEAGIITVFNAFGSGIRTWGNRSAAWPSNTHPRNFINIRRTADVIHESIEYSMLQ
FLDQPITDALIDAITESVNLFLRTLVMRGALIDGHCGYDKAKNPVTEIALGHLTFDITF
MPPPPLERVSFESFIDINLLSQLGGNG"
CDS complement(18832..19029)
/ID="LHBQFDQA_CDS_0026"
/transl_table=11
/phrog="895"
/top_hit="No_MMseqs_PHROG_hit"
/locus_tag="LHBQFDQA_CDS_0026"
/function="unknown function"
/product="hypothetical protein"
/source="PHANOTATE_1.5.1"
/score="-7.872499876169064"
/phase="0"
/translation="MTRYRYDGPVSSVTLPGGLDVNLHPGAEVELPEDNDYVGVLVAKG
FLTAIASNRKPKGADAPPQE"
CDS complement(19026..19562)
/ID="LHBQFDQA_CDS_0027"
/transl_table=11
/phrog="209"
/top_hit="No_MMseqs_PHROG_hit"

/locus_tag="LHBQFDQA_CDS_0027"
/function="head and packaging"
/product="portal protein"
/source="PHANOTATE_1.5.1"
/score="-46.734941807784416"
/phase="0"
/translation="MSVAPNTIEALEAAIVARLGAALPDVEVAPYPADPANYQFLHPVG
ALLVRYHGSHYGALMDTDAVVQERLLAVEITFLFRALNGQDGLYAYLERARRVLTGFKP
AGFGKVYPLRDAFLEEHGGEWRYAVDFCAPTLAIEDGCEEDGPLLKHVTTLDGYERAET
VRQPDGSTTYEEYAQ"
CDS complement(19559..20011)
/ID="LHBQFDQA_CDS_0028"
/transl_table=11
/phrog="195"
/top_hit="No_MMseqs_PHROG_hit"
/locus_tag="LHBQFDQA_CDS_0028"
/function="connector"
/product="head-tail adaptor"
/source="PHANOTATE_1.5.1"
/score="-22.27348607895969"
/phase="0"
/translation="MRYCTLDDLQKKIPPRTLAQLTNDTPPATAPNLDLVEEIVAGQEE
LVDGYLRQRNLLPLAEVPTIVRDVVVQLVRYEIYDRRPEGKDDLPPAVVRGRKDALQTL
DDIRAGRISLGVADDPAQAQPEAGARMRTRIRGERLFTPDLLNRYR"
CDS complement(20021..21010)
/ID="LHBQFDQA_CDS_0029"
/transl_table=11
/phrog="29"
/top_hit="No_MMseqs_PHROG_hit"
/locus_tag="LHBQFDQA_CDS_0029"
/function="head and packaging"
/product="major head protein"
/source="PHANOTATE_1.5.1"
/score="-10638.251557764626"
/phase="0"
/translation="MNLADLFTVTTLTAAVNKLPYATYKIRDLGIFAEAGVRTTTVAIE
EDQGRLHLVPNRSRNDAPEVVRRKRRTRRVFETFHLAEAGVILPEDIQNIAPFGEDMTG
SSLEPQARVINDKLQIMRDSIEITREWQRVGALGGQILDADGTVVYDLYNEFGVTKKTV
DIAFGTNNLDVRAKIVEGKRHAEQKLTGATVTGFACAASKEFMDLLTDHPKVQAAYANW
QAAQDRLGGDMRNDFTFGGVRFFELDVTVSGQRFIPAGKARLFPLGAGVFQMHNAPANY
NETVNTQGQPYYSKGEPRKFNKGWDLEVQANPLALCLFPEALVEFGAV"
CDS complement(21019..21201)
/ID="LHBQFDQA_CDS_0030"
/transl_table=11
/phrog="No_PHROGs_HMM"
/top_hit="No_MMseqs_PHROG_hit"
/locus_tag="LHBQFDQA_CDS_0030"
/function="unknown function"
/product="hypothetical protein"
/source="PHANOTATE_1.5.1"
/score="-0.2867523876706483"
/phase="0"
/translation="VAVLLANAAAAGADVAAPVIARGAVLDANGLVWKSGITDPQKATA

RAALLALGLKVIDSV"
CDS 21235..22530
/ID="LHBQFDQA_CDS_0031"
/transl_table=11
/phrog="No_PHROGs_HMM"
/top_hit="No_MMseqs_PHROG_hit"
/locus_tag="LHBQFDQA_CDS_0031"
/function="unknown function"
/product="hypothetical protein"
/source="PHANOTATE_1.5.1"
/score="-1023.4775852637384"
/phase="0"
/translation="MQFVFAAGADPAEYATESEIGASGDRHAVAGPLLADLVQQDVGND
LRVGMAWHCGLLLSVSGAAFRVGDKGVLERSPVGPVRGGELAEPHHRTAFGEHGLHGVD
QGTGRTVLAGELGQTTLIGAFTGQHEQLDQEVLVLGRQPNPAVAVGAGELLGELDRVVP
APGFRCRGLALTRCGQLGFEPFVFRRQGGGLSLIHGVVLRSGLGKNGLGCGCRIARFIA
DFLASGIFQILQVGTGHDLVGILGSEPDQQPFPESPPEAGVHAVVAFSAEADDAVLVRG
ECRVERGPAQAAHRRRHRAEKGDLADVPLAGNGVAGMIGGVPADRDVRERLFVHQLGEG
RLDLLRLGLEAVRAVAQRLDVAIGRIEPGRVADHQRCPMQSRVVASGRVAQIPLGELDR
LGPAHRQVAARLETVDAVRHAARLGGRKRGFF"
CDS 22721..22810
/ID="LHBQFDQA_CDS_0032"
/transl_table=11
/phrog="No_PHROGs_HMM"
/top_hit="No_MMseqs_PHROG_hit"
/locus_tag="LHBQFDQA_CDS_0032"
/function="unknown function"
/product="hypothetical protein"
/source="PHANOTATE_1.5.1"
/score="-1.0721598648313733"
/phase="0"
/translation="MGALRAVTGRRFAAARLPGGTRLRRPQPA"
CDS complement(22788..23261)
/ID="LHBQFDQA_CDS_0033"
/transl_table=11
/phrog="26"
/top_hit="No_MMseqs_PHROG_hit"
/locus_tag="LHBQFDQA_CDS_0033"
/function="connector"
/product="tail completion or Neck1 protein"
/source="PHANOTATE_1.5.1"
/score="-41.20454255410768"
/phase="0"
/translation="MSNPIEVTLDDTEVRQMLDRIQRAGLNLGPLMKTLAMTLKDETEH
RFENEGPGWPALSDSTKQARTRLGHWPGQMLQVSGQLAASISTESGADFARIGSSKVYA
AIHQFGGSAGRSQRTTIPARPYLPMTAKGELTPQAREAVLDDTMAYLKRAAGV"
CDS complement(23258..23386)
/ID="LHBQFDQA_CDS_0034"
/transl_table=11
/phrog="No_PHROGs_HMM"
/top_hit="No_MMseqs_PHROG_hit"
/locus_tag="LHBQFDQA_CDS_0034"
/function="unknown function"

/product="hypothetical protein"
/source="PHANOTATE_1.5.1"
/score="-2.5886995237144887"
/phase="0"
/translation="MASCYFEDSLATPPHDKDRAAPLDASGGSLYGRNRLQESIPR"
CDS complement(23361..25658)
/ID="LHBQFDQA_CDS_0035"
/transl_table=11
/phrog="220"
/top_hit="No_MMseqs_PHROG_hit"
/locus_tag="LHBQFDQA_CDS_0035"
/function="head and packaging"
/product="minor head protein and DNA pilot"
/source="PHANOTATE_1.5.1"
/score="-62728138.1412966"
/phase="0"
/translation="MTLPAADLKAIFNLEPKAAIAYLQHKGYKLTWNWQEMLDDAHARA
FTIAKAARLDVVQDIRNALDVALRHGQTLKDFQKNLKPTLQAKGWWGKQIIVDGAGNAE
VARLGSPWRLATIYRTNLQSAFMAANYQEMAEATDSHPYWQYVAVLDGRTRPSHRAMNG
RVFRHDDPIWNTIWPPNGFNCFPAETPVRAAARLGLKTWYAGKVVELQTRLGHRLTLTA
NHPVLTVRGWIAACQLQKGDQLIGDASGVNPRLAGVVNDEQPPARAEDLFQTLAAQGFR
IVPMAPHDFHGDAGLRKPEIHIAGPDVHLMDEVQAAPGQFVGQQQLRRADACAIMDADR
PDGPPPARMILADAVAPQNPADVAEAGAELAADGAFGDQPVAVQGQHPAFEMGVAVAGA
LPGGGALASNGGGVLFDGSPFDALGFRAPPQGDVAGTEQPAQGVTAASGLVRQLLEANA
GLIALDEIVQIRQFDWAGHVYDFETETGLIMAGGVIVHNCRCRVRPRSERSLARDGIAW
QSSAGKLRTLETDAGVDKRTGEITHARRTGIDVVDADGKKHFFAPDAGFNFNPGQGWSK
PFTPPPLDTLPKTFSPGQVLPDLPKPEKFAASMIVPDGLGEEDYAKAFLAEFGADLGKP
VVFQDVTGDPMLIDEALFKSGAGEWKATKDGRGPYMRLLAHAIRSPDEIWMRWEESRER
PGTWLLKRRYLKTFEIDGHGSESPQYGLSVFEYGSEGWSGSTAMISQASRGPGARRRYI
ERQRDGFLLFRR"
CDS complement(25655..26416)
/ID="LHBQFDQA_CDS_0036"
/transl_table=11
/phrog="No_PHROGs_HMM"
/top_hit="No_MMseqs_PHROG_hit"
/locus_tag="LHBQFDQA_CDS_0036"
/function="unknown function"
/product="hypothetical protein"
/source="PHANOTATE_1.5.1"
/score="-4.301360894454668"
/phase="0"
/translation="MPTVILTSGTTWTVPHGVAMLDSVECWGGGAPGNNGNNIGSGGGG
AYSKTINLAVIPNQTCYLQIPGTTAISGGSPADCWFSKTSNSAPAATSDGCLAKSGAKS
TGTVGGQGGQASGCVGDVKYSGGNGGYSSGFGRGGSGGGASASPFGAGGDGNTPTSGST
GGNGGNSPSAGSGGTAGSAGDSIADGGGGGGGGNGTTFGSPASGAAGGAPGGGGGQSGA
NVGGTHAASGGPGARGQIRITYRYATRAIVR"
CDS complement(26420..26914)
/ID="LHBQFDQA_CDS_0037"
/transl_table=11
/phrog="29752"
/top_hit="No_MMseqs_PHROG_hit"
/locus_tag="LHBQFDQA_CDS_0037"
/function="unknown function"

/product="hypothetical protein"
/source="PHANOTATE_1.5.1"
/score="-101.1819571975448"
/phase="0"
/translation="MSNKIQHKRNATWGNIPAPEQLEDGELAINTFEGKLFLKRSASDN
KVVEVGTLAPRVVTLLYPTGVDVVPLFYTPTYFPVGYLKAVLVGSGTPSVTFTIKYGTS
LASGTEMVVGGVTCTNTTTGMTLYSTSFDNDTVPAGNWVWLETTAIAGSVIALQVTLVA
G"
CDS complement(26914..28314)
/ID="LHBQFDQA_CDS_0038"
/transl_table=11
/phrog="2647"
/top_hit="No_MMseqs_PHROG_hit"
/locus_tag="LHBQFDQA_CDS_0038"
/function="head and packaging"
/product="head decoration"
/source="PHANOTATE_1.5.1"
/score="-10791.150835184297"
/phase="0"
/translation="MANAVRIKRRASGNAGAPAALLNAELAYNEVDDVLYYGKGDSGGN
ATSIPAIGGPGAFVTKSTAQTIDGAKTFTGANDLGAQTTATTPAAADNSTRVATTAFVK
AQGYLTGNQVVTLTGDATGSGATSIAVTIPNDTVTNAKLANMAAGTLKGNNTGSAADPA
DLTVAQVKTLLAYQATDITGFDTQVRTSRLDQMAAPTAAVSMNSQRITNLADPTGAQDA
ATKAYVDSVASGLDWKQSVRAATTANITLSGTQTVDGVALVAGDRVLVKNQTTASQNGV
YVVAAGAWGRATDFDSNAEVTAGACMFVSEGTVNGDQTWVLTTNDAIALGTTSLTFAQM
SGGASGEANTASNQGAAGVGVFDGKSGVDLQFRNLVAASSKITVTLNAGNKTIEIDLVQ
ANITAVGTVTAGVWQGTAIGLQYGGTGANLSAAADGAIFKKSGTALVAATAGSDYLNNA
STIDGGTF"
CDS complement(28338..28508)
/ID="LHBQFDQA_CDS_0039"
/transl_table=11
/phrog="No_PHROGs_HMM"
/top_hit="No_MMseqs_PHROG_hit"
/locus_tag="LHBQFDQA_CDS_0039"
/function="unknown function"
/product="hypothetical protein"
/source="PHANOTATE_1.5.1"
/score="-2.8575751848047855"
/phase="0"
/translation="MVCEATVEVDCVDVAPTVDVENAPGPRGPVGPQGPPGPEGPAGGI
DPDTVIDGGNF"
CDS complement(28498..28866)
/ID="LHBQFDQA_CDS_0040"
/transl_table=11
/phrog="No_PHROGs_HMM"
/top_hit="No_MMseqs_PHROG_hit"
/locus_tag="LHBQFDQA_CDS_0040"
/function="unknown function"
/product="hypothetical protein"
/source="PHANOTATE_1.5.1"
/score="-31.990336677557863"
/phase="0"
/translation="MGKYLPDIRQGDTYRVRIRYPKGSNIAGYIHWLALRTAIGTEPPL

LLLRSVVGEHPSDQPEDGIAYLEATPEQTAALPANKRCVYGLLAKTPSGEVKTLRPPLK
DPLDDRILIVPRGGADGV"

CDS complement(28859..30349)
/ID="LHBQFDQA_CDS_0041"
/transl_table=11
/phrog="113"
/top_hit="No_MMseqs_PHROG_hit"
/locus_tag="LHBQFDQA_CDS_0041"
/function="head and packaging"
/product="portal protein"
/source="PHANOTATE_1.5.1"
/score="-152634.28771071444"
/phase="0"
/translation="MTRRAPTGLYLPSGEFVAFAQGPALGQQLVTRQTALGQFSGLTWL
PNPDPILKKMGKSIEVYRDLLSDAHVAGCVRRRKAAVRAMERGVERRSAGARTAKNIEA
ILADLPLARIIGEILDAVLYGYQPLEIVWERVGNFVVPADVIGRPPEWFVYSRNNELMF
RSQACPMGEPLPPNSFLCARQEPTYFNPYGAPDLALVFWPTTFKRGGLGFWMQFAEKYG
MPWPVGKYPRGTETGEIDQLLDSLEQMVRDGVAAVPADSSIEFLTHDQSQGADAYEKLL
LFCRSEVAIALLGQNQTTEVSANKASAEAGIMVTRDIRESDAEIVASTLSDLIRMVCEL
NFHEPAPPVYQLWEQEEIDETRAVRDKALHDAGVRPTNVYWMRAYGFQEGELAPEEGSG
APAFAGIDLELFPDQAALDAAVDALAEGGALQAQAERLLAPLIERIESAGGEAELLGAL
AEAYQDMNPDQLVQTLTRLIFAASVAGRLAAEESLQNG"

CDS complement(30346..31677)
/ID="LHBQFDQA_CDS_0042"
/transl_table=11
/phrog="5909"
/top_hit="No_MMseqs_PHROG_hit"
/locus_tag="LHBQFDQA_CDS_0042"
/function="head and packaging"
/product="terminase large subunit"
/source="PHANOTATE_1.5.1"
/score="-44417.90357292736"
/phase="0"
/translation="MTALTLYPYQQRWIADASRFKVGMFARQCGKTFTTTLELVMDCLQ
AEAQGGRRRWVILSRGERQAREAMEEGVKRHLHAFQTGFKALEYDFEASVKALEVVLPG
GSRISALPANPDTARGFTASVLLDEFAFHADSRKIWQALFPVVSRSDLKLRVISTPNGK
GNKFYDLITGDHPVWSRHVTDIYQAVADGLPRDIEELKAGVGDDDAWAQEYELQWLDEA
SAWLSFELINSVEHDHAGIPEHYAGGPCFLGVDIAARNDLFVIWVLEAVGDVYWTREIL
ARRRISFAEQDALLADAFNRYRVIRCCMDQTGMGEKPVEDAQRRFGSSRVEGVLFTGPN
KLALATTGKEAFEDRRIRIPEGNQELRNDLHKLKKETSATGAPRFVADSDSAGHADRAW
ACFLAIHAASNPGTMPIVASRPRTGTVLSGYGDLRRGTSMRGYA"

CDS complement(31674..32237)
/ID="LHBQFDQA_CDS_0043"
/transl_table=11
/phrog="426"
/top_hit="No_MMseqs_PHROG_hit"
/locus_tag="LHBQFDQA_CDS_0043"
/function="head and packaging"
/product="terminase small subunit"
/source="PHANOTATE_1.5.1"
/score="-144.00534591757722"
/phase="0"
/translation="MPPRSSVYQLPPEVQEELNTRLVGSGFGGYRDLSAWLKDQGYHIS

KSALHSYGQEFELEFKSSMEAAFTVEKLSKALVAQRDDSEAAMLEAAAMVGQEILLKLM
IALRQAESDPTKAAKTISLATRSLADLGRMTLDQKKWQDQLRKQLQEEAGAKLDRLEAG
ARAGKGKLDAETLRIVRQEVYGLA"
CDS complement(32237..32533)
/ID="LHBQFDQA_CDS_0044"
/transl_table=11
/phrog="436"
/top_hit="No_MMseqs_PHROG_hit"
/locus_tag="LHBQFDQA_CDS_0044"
/function="unknown function"
/product="hypothetical protein"
/source="PHANOTATE_1.5.1"
/score="-1.048772616445223"
/phase="0"
/translation="VNLENFETEQRRLVILRRLIRAPAYTLDQTVLAKALALEGLAVSR
DRLKTDLAWLAEQDLIVGQQPGGVWVATLTHRGLDVAKGLTVMPGVARPEPGE"
CDS complement(32530..32868)
/ID="LHBQFDQA_CDS_0045"
/transl_table=11
/phrog="736"
/top_hit="No_MMseqs_PHROG_hit"
/locus_tag="LHBQFDQA_CDS_0045"
/function="unknown function"
/product="hypothetical protein"
/source="PHANOTATE_1.5.1"
/score="-61.27031988447617"
/phase="0"
/translation="MFLFVWNLGVSGWLWLGRSHKATLKRIDEVEADFHDRISTYEQRC
LVKHERITRLESQIGKMPNHDDLGRIYDRINAVSGDVREMKGTLEGTAKTVDRLHRYLL
EHDRGSKS"
CDS complement(32906..33181)
/ID="LHBQFDQA_CDS_0046"
/transl_table=11
/phrog="54"
/top_hit="No_MMseqs_PHROG_hit"
/locus_tag="LHBQFDQA_CDS_0046"
/function="other"
/product="DksA-like zinc-finger protein"
/source="PHANOTATE_1.5.1"
/score="-17.49229264401959"
/phase="0"
/translation="MPDLADLSDERIEIERVAGIRAVLDSMRPDTPQVVIPGEDGLPMV
VCYDCLNEYEVITPIPPERLAANEHAIRCIDCQIEHEREAKLQLHG"
CDS complement(33165..33383)
/ID="LHBQFDQA_CDS_0047"
/transl_table=11
/phrog="No_PHROGs_HMM"
/top_hit="No_MMseqs_PHROG_hit"
/locus_tag="LHBQFDQA_CDS_0047"
/function="unknown function"
/product="hypothetical protein"
/source="PHANOTATE_1.5.1"
/score="-6.671312376573183"

/phase="0"
/translation="MLTPQEKCVGGGALLMLAFCIGVLAGCDCLACEPTGYAKFADTLS
VLHGERPIEFGAGLQCEFTERTDAGSR"
CDS complement(33383..33703)
/ID="LHBQFDQA_CDS_0048"
/transl_table=11
/phrog="No_PHROGs_HMM"
/top_hit="No_MMseqs_PHROG_hit"
/locus_tag="LHBQFDQA_CDS_0048"
/function="unknown function"
/product="hypothetical protein"
/source="PHANOTATE_1.5.1"
/score="-6.883130768799169"
/phase="0"
/translation="MRMWKRGWTVRCVSGFPGAAILALPLTLAGCVQVGPKELVPKPIL
TPAFAPESCALPDLPPLASTVFVDIAPGRPPKADAGGKALVVGYGRAREAIQACKGGAE
GH"
CDS complement(33663..34088)
/ID="LHBQFDQA_CDS_0049"
/transl_table=11
/phrog="873"
/top_hit="No_MMseqs_PHROG_hit"
/locus_tag="LHBQFDQA_CDS_0049"
/function="head and packaging"
/product="endolysin"
/source="PHANOTATE_1.5.1"
/score="-56.8398381100516"
/phase="0"
/translation="MSFQKPRGLRNNNPGNLVYSPRNAWEGQVGHDGRFARFEQMEDGV
RALGVTLLNYQRKRQLHTVRQIITRWAPPNENDTATYIRRVAAALDVGADDRIILSDRK
TLTLLVWAISTHENGAMACERWLKHEDVEAGVDRALR"
CDS complement(34085..34231)
/ID="LHBQFDQA_CDS_0050"
/transl_table=11
/phrog="No_PHROGs_HMM"
/top_hit="No_MMseqs_PHROG_hit"
/locus_tag="LHBQFDQA_CDS_0050"
/function="unknown function"
/product="hypothetical protein"
/source="PHANOTATE_1.5.1"
/score="-3.785609749537376"
/phase="0"
/translation="MQFIVQGIGIAGVGHKIEKAATPAPLPQGRGEDMSDEEFAAFVRR
QQK"
CDS complement(34321..34659)
/ID="LHBQFDQA_CDS_0051"
/transl_table=11
/phrog="2609"
/top_hit="No_MMseqs_PHROG_hit"
/locus_tag="LHBQFDQA_CDS_0051"
/function="lysis"
/product="holin"
/source="PHANOTATE_1.5.1"

/score="-25.992901875399784"
/phase="0"
/translation="MGKFWAWLMTAAAKAWPILWRYIAPMLADGMRQQWEILLPVAERM
VRAVEDSLSWAPKSGEAKKSAAIALILKELKEREIAWADRIPDRMMNRAIEMAVGLLPE
KQSAETAP"
CDS complement(34635..34802)
/ID="LHBQFDQA_CDS_0052"
/transl_table=11
/phrog="No_PHROGs_HMM"
/top_hit="No_MMseqs_PHROG_hit"
/locus_tag="LHBQFDQA_CDS_0052"
/function="unknown function"
/product="hypothetical protein"
/source="PHANOTATE_1.5.1"
/score="-0.1018694307401487"
/phase="0"
/translation="LKPLNQSIPCRPKARLVRALFLIRFKRRGAIRAVLCAHIGSWVRR
AASWANSGRG"
CDS complement(34871..35509)
/ID="LHBQFDQA_CDS_0053"
/transl_table=11
/phrog="8809"
/top_hit="No_MMseqs_PHROG_hit"
/locus_tag="LHBQFDQA_CDS_0053"
/function="unknown function"
/product="hypothetical protein"
/source="PHANOTATE_1.5.1"
/score="-291.7611533065413"
/phase="0"
/translation="MRRTFIWAVLMGVTVAAHADDWSGKVVGVTDGDTLTIMKGGRGAK
VRLAEIDAPESHQDYGAKSKQSLSDLCFGKEAKVNDQGADKYGRRLGRVSCAGIDVNLE
QLKRGLAWFYVEYGHDPALRAAEEQSRASRIGLWSDANPTPPWDFRHGTPKKAARSTDE
ADGSASDCGGKSTCKQMTSCAEARHYLNDCGVSRLDRDRDGIPCESICR"
CDS complement(35521..35871)
/ID="LHBQFDQA_CDS_0054"
/transl_table=11
/phrog="532"
/top_hit="No_MMseqs_PHROG_hit"
/locus_tag="LHBQFDQA_CDS_0054"
/function="transcription regulation"
/product="late transcriptional activator"
/source="PHANOTATE_1.5.1"
/score="-24.66010515213617"
/phase="0"
/translation="MAEKKGRQARELLSDLAAKSAALACELGALPKDVAERFGLELSRE
VAAHWGGQLVYIPRNLPGELDRRDLEIFEKFRGDNHAELALEYRLSVQWIYRIVKRVRQ
SEIDRRQRRLFD"
CDS complement(35864..36472)
/ID="LHBQFDQA_CDS_0055"
/transl_table=11
/phrog="270"
/top_hit="No_MMseqs_PHROG_hit"
/locus_tag="LHBQFDQA_CDS_0055"

/function="DNA"
/function=" RNA and nucleotide metabolism"
/product="replication initiation protein"
/source="PHANOTATE_1.5.1"
/score="-78.08092326963222"
/phase="0"
/translation="MSHTKRSFIQACLIRALPALDRVDAGIAHAEALWERLTAKGYGAP
RQTGPRESVDWYARLVEPSRGWFDQFWTAYGLKRDRNGAAMRWYQLGDLTEHEARRIID
AAKQDNRQWRETAQPGQVRKMAQGWLHEKRWMDYAPTPQPPLSGGYSAGLAGDAQLREL
KQQLASLQRLNAAAPSKELQRQINELVQEIGNFQRPGHG"
CDS complement(36469..36675)
/ID="LHBQFDQA_CDS_0056"
/transl_table=11
/phrog="No_PHROGs_HMM"
/top_hit="No_MMseqs_PHROG_hit"
/locus_tag="LHBQFDQA_CDS_0056"
/function="unknown function"
/product="hypothetical protein"
/source="PHANOTATE_1.5.1"
/score="-1.7403291716342297"
/phase="0"
/translation="VSLNLLVVIVFVAGFGWGQWWAERQIRIERASLEIGRLSVSMPMN
PDDAAATFGLLKTLMEQVERERK"
CDS complement(36672..37103)
/ID="LHBQFDQA_CDS_0057"
/transl_table=11
/phrog="286"
/top_hit="No_MMseqs_PHROG_hit"
/locus_tag="LHBQFDQA_CDS_0057"
/function="unknown function"
/product="hypothetical protein"
/source="PHANOTATE_1.5.1"
/score="-26.607872552359744"
/phase="0"
/translation="MARTDPVRRQLVAIHVLKSKAGMDDSSYRAMLREIGGASSSKDLT
ATGRAKVLDHLRRITQPPVAKPRSRPYPGRPHTTDQRPLLQKIEAQLTEAGRPWNYAVG
ILKRVSRGRAERLEFATDEDLRKVVAALTYDARRHGGTT"
CDS complement(37109..37396)
/ID="LHBQFDQA_CDS_0058"
/transl_table=11
/phrog="379"
/top_hit="No_MMseqs_PHROG_hit"
/locus_tag="LHBQFDQA_CDS_0058"
/function="DNA"
/function=" RNA and nucleotide metabolism"
/product="DNA binding protein"
/source="PHANOTATE_1.5.1"
/score="-12.727276868058231"
/phase="0"
/translation="MNQSQLINAIHEDANGSLTKTVIQRVLIALANVAQEALANGDEVT
IPGVAKLTVKERAARTGRNPQTGETVEIPAKRVARASFVKALKERVQGGA"
CDS complement(37430..37927)
/ID="LHBQFDQA_CDS_0059"

/transl_table=11
/phrog="491"
/top_hit="No_MMseqs_PHROG_hit"
/locus_tag="LHBQFDQA_CDS_0059"
/function="DNA"
/function=" RNA and nucleotide metabolism"
/product="Mu Gam-like end protection"
/source="PHANOTATE_1.5.1"
/score="-361.968647878636"
/phase="0"
/translation="MARIRIEGTQFSSWDDVDQALREIGEIERDLSLIDGDVNDQIDRL
KAAAKTQAQPLLDRKTALELAMKEFCEANRAEFAKVKTRQLVFGSVGFRLSTRVLIKRV
ADTLQALKDLGLHGCIRLKEEIDKEALKTLPAETLAEVGAGLKTDNVFGYEIDRARIAE
AA"
CDS complement(37936..38097)
/ID="LHBQFDQA_CDS_0060"
/transl_table=11
/phrog="No_PHROGs_HMM"
/top_hit="No_MMseqs_PHROG_hit"
/locus_tag="LHBQFDQA_CDS_0060"
/function="unknown function"
/product="hypothetical protein"
/source="PHANOTATE_1.5.1"
/score="-0.6322075260143188"
/phase="0"
/translation="VKAIVLACLAFIYLIGLGTGLLLGAVLDINLVGRAERKAQARQSD
TDDINLGI"
CDS complement(38094..38327)
/ID="LHBQFDQA_CDS_0061"
/transl_table=11
/phrog="No_PHROGs_HMM"
/top_hit="No_MMseqs_PHROG_hit"
/locus_tag="LHBQFDQA_CDS_0061"
/function="unknown function"
/product="hypothetical protein"
/source="PHANOTATE_1.5.1"
/score="-7.845893932335907"
/phase="0"
/translation="MIKIQRYPRLGAYGWKALVTVGGKAREVLTVGTWFDIRRLARRAE
EEMAEKPRRSPCVDAARQRQLDVRRGLQGAAP"
CDS complement(38324..38659)
/ID="LHBQFDQA_CDS_0062"
/transl_table=11
/phrog="1035"
/top_hit="No_MMseqs_PHROG_hit"
/locus_tag="LHBQFDQA_CDS_0062"
/function="DNA"
/function=" RNA and nucleotide metabolism"
/product="endonuclease VII"
/source="PHANOTATE_1.5.1"
/score="-16.99155317695442"
/phase="0"
/translation="MAEISTKRCTRCGQTLPKDRFYRQGVRLEATCKACVNAARTAKRR

AAGIAPRPPAKPRQPDYSPEVWDRELRAVNRAFNHTLKTIFGSLHRCNPHPTLSRREMA
LTQGEHA"
CDS complement(38652..38909)
/ID="LHBQFDQA_CDS_0063"
/transl_table=11
/phrog="No_PHROGs_HMM"
/top_hit="No_MMseqs_PHROG_hit"
/locus_tag="LHBQFDQA_CDS_0063"
/function="unknown function"
/product="hypothetical protein"
/source="PHANOTATE_1.5.1"
/score="-7.630745428271122"
/phase="0"
/translation="MHIRDRIILAPTPAWFDAALDRGETRALRVDIAPAEIAGHSVFHL
PFRPDRADWYRTFQVARAFLRGAAFRGGAAPTETGREVHG"
CDS complement(38896..39234)
/ID="LHBQFDQA_CDS_0064"
/transl_table=11
/phrog="2119"
/top_hit="No_MMseqs_PHROG_hit"
/locus_tag="LHBQFDQA_CDS_0064"
/function="unknown function"
/product="hypothetical protein"
/source="PHANOTATE_1.5.1"
/score="-15.297628516031272"
/phase="0"
/translation="MTEERRERALKLLEAAVAAHGSFAAVAKLLGLNRATISTVARRCY
PGDDAKVLARVLEQFDQIACPYLRRPMAPEECRSVWSGATPSHDPALLAHRRACRSCPH
KGDGHAHS"
CDS complement(39227..39814)
/ID="LHBQFDQA_CDS_0065"
/transl_table=11
/phrog="2030"
/top_hit="No_MMseqs_PHROG_hit"
/locus_tag="LHBQFDQA_CDS_0065"
/function="transcription regulation"
/product="transcriptional regulator"
/source="PHANOTATE_1.5.1"
/score="-175.25358186976703"
/phase="0"
/translation="MARKPVTIELAGGKSPRQRIWEMIRKLRIFTVPELRGHLPGPVPL
ATVRTYVESLHAAGILEKTPGLYELIDDRGIEAPRVNKAGQPVTLGQGNERMWGAMEAL
GAFNCRVLARMADVPLATVKTYCAYLQRAGFLTVERAGKGRGAGGVPTTYRLLHSRITG
PRPPMITRLKTVYDPNVGKIVWQQDPQEQLDD"
CDS complement(39814..40566)
/ID="LHBQFDQA_CDS_0066"
/transl_table=11
/phrog="296"
/top_hit="No_MMseqs_PHROG_hit"
/locus_tag="LHBQFDQA_CDS_0066"
/function="integration and excision"
/product="DNA transposition protein"
/source="PHANOTATE_1.5.1"

/score="-1303.7600450732564"
/phase="0"
/translation="MTDKPENDKSTPAGIAPLTNIAIAERAMALAMGRSMHQSGLVVLH
GPSGYGKSMAAAWVKARSRAYYLQLDDFTRSRKVLLKRLGKALGMQLAKSATVDEMADA
VMAQLEQSGRPLIIDEADYLETFKLVDSIRSIYEGSKAAILLIGEEALPFKLKEWERFD
GRILDRFPAQPVSLPDARELAGLYCPGKVMGEDLLERLVEVAKGSVRRVSTNLERMYEE
ALANGWDRIDLATWGNRPIHTGEAPRRG"
CDS complement(40630..42942)
/ID="LHBQFDQA_CDS_0067"
/transl_table=11
/phrog="310"
/top_hit="No_MMseqs_PHROG_hit"
/locus_tag="LHBQFDQA_CDS_0067"
/function="integration and excision"
/product="transposase"
/source="PHANOTATE_1.5.1"
/score="-231914619.7564892"
/phase="0"
/translation="MSAIKTHYSCAELAALKLPGYPGSERGFRDLVEREGWAFREASTK
GGKNGMRREYQPSAAVMKLIQAREKEAQRQASLAAELAALESSPEYRAGQAIARQMAEA
SAREAEARQQDDEALLALFAQATGERGERGRAWLATIQCWRRFYREKFGDQKRSQEKAL
IAFIKAFNAGALDLDGEVYAPVLKSSGRGKNAQGKGLSLRTLKRKLGLIKKSNIGALLD
NHKSPRRGDSLIHQQPRLEQYMLGLLAQQPDIAAVRLHEMAENRFAGDATIKVPSYSAV
DRYVKNWKAKNASAYLHLRNPDAWKNKAMPAVGLADGDVVRLNQRWELDSTLGDLLLSD
GKRHAVIGVIDVYSRRLKLLVAPTSKARAIAAVVRRAMLDWGVPEQAKTDNGADYASDY
LTGIWDAFGVEHVLCPPFSPEYKPHIERALGTFNHDLVELLPGYIGHDVAERKAIEARK
SFARRLMEKDGVLEVRLSPEELQKFCDDWTENRYHHRPHAGLEGETPFQRAASYRGEIR
RIHNERALDILLSPPVDGKTRSLGKKGLEIHGGFYSHPELWRHHTTGDAFRVMVDETDL
GRVYAYDDNGFVCVAICPERLGLPPEAVSRHAMECKRMAAQQRNQGMKTLNRAKKGVDL
NAEINSYLRSKAESAGKLATFPQLSTGYTTPALEGAARAAESTDRPPRSPEAEKLSAEA
KAHFAASGRSAEIIKLPKRKEHPLERMSDEEKYEFWCSLDAAVKAGGGELPDDAQVRRF
YAEFPLSARFKAQAAIR"
CDS complement(42939..43355)
/ID="LHBQFDQA_CDS_0068"
/transl_table=11
/phrog="No_PHROGs_HMM"
/top_hit="No_MMseqs_PHROG_hit"
/locus_tag="LHBQFDQA_CDS_0068"
/function="unknown function"
/product="hypothetical protein"
/source="PHANOTATE_1.5.1"
/score="-4.996260090393858"
/phase="0"
/translation="MKELPAASLARATRMACCWRSSNTRQAPAEMCSQLLSGSSIRSSI
AASASGSKEASYGRERMRVRLSRNRSLNGHSASTPCHPVMNCGSSDSREQSSARSVKTR
NRSKFFIEGSSVMVVWQRQFKAGRNPRQFGMGRA"
CDS complement(43352..43543)
/ID="LHBQFDQA_CDS_0069"
/transl_table=11
/phrog="No_PHROGs_HMM"
/top_hit="No_MMseqs_PHROG_hit"
/locus_tag="LHBQFDQA_CDS_0069"
/function="unknown function"

/product="hypothetical protein"
/source="PHANOTATE_1.5.1"
/score="-4.373543625951236"
/phase="0"
/translation="MMSLLEVTHARAFSVAAAMKESHLLELKSRIDLLVATGASFREAE
TTLRPLCAAIQKDREESV"
CDS complement(43546..43638)
/ID="LHBQFDQA_CDS_0070"
/transl_table=11
/phrog="No_PHROGs_HMM"
/top_hit="No_MMseqs_PHROG_hit"
/locus_tag="LHBQFDQA_CDS_0070"
/function="unknown function"
/product="hypothetical protein"
/source="PHANOTATE_1.5.1"
/score="-2.270527088138536"
/phase="0"
/translation="MSGERKPQGFRAIQHHRASAVRNGKALAEV"
CDS complement(43635..44075)
/ID="LHBQFDQA_CDS_0071"
/transl_table=11
/phrog="2345"
/top_hit="No_MMseqs_PHROG_hit"
/locus_tag="LHBQFDQA_CDS_0071"
/function="transcription regulation"
/product="transcriptional regulator"
/source="PHANOTATE_1.5.1"
/score="-43.89082041773178"
/phase="0"
/translation="MNAAQQDLFGGGAVHLPQPIPIAPGRFALGDRVAVAMSRAIDASG
VDRPDLASRITALTGKRLPLSVLNAQTAMSRPDHTPSLLQAMAFDAVTGKWALLELYAE
AAGGKVIYGDDIAAFEVGRAVMVKKLAGQREREALRSVGVGR"
CDS complement(44176..44610)
/ID="LHBQFDQA_CDS_0072"
/transl_table=11
/phrog="925"
/top_hit="No_MMseqs_PHROG_hit"
/locus_tag="LHBQFDQA_CDS_0072"
/function="DNA"
/function=" RNA and nucleotide metabolism"
/product="HTH DNA binding protein"
/source="PHANOTATE_1.5.1"
/score="-46.337468061524085"
/phase="0"
/translation="MILGYFIYFHLSAYLGAARKQKNLVDGSSEIQYAMSNSRTNTDTT
QPRKRAMSNTNQAAGAQANPFCAFIEAQGGTITPDQFRSWLALQGITMADWARERGFRP
REVSLVLNGQIKARYGRSFSIAVAMGLKPDPNRAQKQHAA"
CDS 44638..45399
/ID="LHBQFDQA_CDS_0073"
/transl_table=11
/phrog="4"
/top_hit="No_MMseqs_PHROG_hit"
/locus_tag="LHBQFDQA_CDS_0073"

```
                     /function="transcription regulation"
                     /product="transcriptional repressor"
                     /source="PHANOTATE_1.5.1"
                     /score="-2755.8354293813836"
                     /phase="0"
                     /translation="MTSEINSEIGAGSSEEWFSAADLARMVLPGWPKSDRGWRDVLERQ
                     HWPFREVPSKGGKRGMKREYQPPPEVLALIRQKLQGEPQAAHAYPTKPGGLNGDKHLQR
                     QPAVYSPDDYVALPLYDVRAAAGGGVVPDTENVVDFLHFKKAWLRTELRSSPDDLYLIY
                     VDGESMEPTLCKGDVILVNHRDKFQGRDGGIYVLRLDGALLVKRLQRKMGGIIKVTSDN
                     PVYEPFEVGAQDLDRADFSIIGRVVWAGRRM"
     CDS             45486..45587
                     /ID="LHBQFDQA_CDS_0074"
                     /transl_table=11
                     /phrog="No_PHROGs_HMM"
                     /top_hit="No_MMseqs_PHROG_hit"
                     /locus_tag="LHBQFDQA_CDS_0074"
                     /function="unknown function"
                     /product="hypothetical protein"
                     /source="PHANOTATE_1.5.1"
                     /score="-1.8255339104316788"
                     /phase="0"
                     /translation="MPKRIFARNSVNSLIWAKKSAVVRASPECQIKN"
ORIGIN
        1 aggcgccgcg caatttggca tagatttgcg cggcttttgg cactgcgcta acccaccgat
       61 cacggcatca gcgataggat tgggcggggt tcgccgatgg ggattccggc ctgccggtct
      121 gctcctccgg acaggcctct cggcagcgtc gtactgacgc gtcgaaatac tccggcacgg
      181 attcgcaacc gatgaaccgt cggccatgtt tgagcgcggc gacaccagtg gttcccgacc
      241 ccatgaacat gtcgaacacc gttccgccag gcgggcagac ctggacgatg tcctccatca
      301 gcgcgagcgg cttctgggtg atgtggacct tgccgttgct gagggtcgaa tgccgcaaca
      361 caccgggcag ataaaccggc ggttcgcggg tcggcgtcgg cccctgggtg gcccagagca
      421 ggtattcagc ctggttcttg aacccgtttt tcatcggccg ggccccgttg cccttgtccc
      481 acgccacgca accccgcagc gccaggccgg acccgtggat gatctcctga agctgcggct
      541 tgttgcgcca gtcgatgaac atggccagca cgccaccgtt catcaacaca ctgcgcgcaa
      601 cggcgcaggc cgccttcatg agttctttcc aggcttccgg gtgcagcgaa tcgccatcga
      661 tgtccggcag cgtcttttga taagacgcat cggagctgac gtacttggtc ttggatgact
      721 tcaggcgatc cttgaccgtg aatccgcccg aaccgtaggg cgggtcggta atcacggccg
      781 caacgctgcc gggggtgatc agcgaggcaa cctcgcgaaa gtcattttga aagaggcggc
      841 accggccatc gctgaattca accaagcgga tcgagtcggc tattgcccgg ccgcaggcgt
      901 ggcacacttt gctggctggc tggctggctg gctggctggc tggctggctg gctggctggc
      961 tggctggggg attgtcatgc attaggtcct cgtcttggtc gttgcgtgtc agtttctgtg
     1021 gtttctagat cggccagtcg ctgtctttga atcactccaa taagtcctcg ccgggctgcc
     1081 gtcgtcagaa gttctcccac agtggcagcg ccgcactgcg atcgggcaag agcagcaggg
     1141 acaccgcgcc ccaggtcgtg gcggccgacg cggtcagggt cctggttccc gtcgcccccg
     1201 ccgatacgag ctgctggtcc gccatcagga gactgccgcc gcctgcgttg gcgaccagcg
     1261 tgtcgcccat cccggcaggc caggtggagg ctccaacgct ggcggtcccg ccccacaggc
     1321 cgaccagaag atcgtcgcct tccccctcgc tcggggtaac ggaaggcgcc gccagggacg
     1381 atgacgatcc gccggagact cccgccgacg cgtcgaccgg cgcttccgta ttcaccccgc
     1441 cgaaggcaca gatcaccgcg ttggcggcat aggacgggaa accgcccgcc gagaagttca
     1501 gggtgtacga ggccggctcg ccgtccgatg ccaccttata gaaagccgca agctgcgacc
     1561 ggccgttgga gccgaagccg acgctgcctg ccggcaccgg tgtgaatccc gacggcgtgg
     1621 cgatcgcggc gccggatccg aaagtgccgg catcgagcgc gacggccagc aggaggagat
     1681 cgccggtggc gattccggcc ggcctggtga tcgacagcga agcgccggtc gacgaggccg
     1741 tcccggtggc actgacctga atgcccatca ggcggcgccc accttggcca gggactggat
```

1801 catgctggcg ccgtcccagt agatcgacag cagcgattcg ccgttcgccg cggcattgac
1861 ggacggcgcc gtggcccggc cgagccagcg ggagccggac aggccagccc aggtcacagc
1921 acggccgccg gtggcgtcct gaatcaggcg gatcacgtaa ttaccgaccg gcgcgccggt
1981 gctggacacg gtcagggtcg tcgaagccgt cagcttgagc ttctgtttct ggccgttcac
2041 caggctgagg gtcttggccg tgccgctgtt gccattgtcg tactcggcct gaaagcccag
2101 cgtcttcacc ccggagatcg gatagccgga cagccccaac gcacccagca ggttgccgcc
2161 cccgaccggc aaggcgccga cctgcgccgc cgtggtgttg tgcgggttgg atacgttgga
2221 ggcatgcgcg gcggccgtgg ccagttgggc ggcctgcgag gccagcgtgg ccgcgaccgc
2281 cgccagggtc gtggccggcg cgtcgtacca gttggcggcg ccggtgatcg ccttgaggcg
2341 gttcgccagg cggccgagcc actgggtcag ggtcccggtg ttgttgacgg gcaccagggc
2401 cggatcggcg gtgcgattgc cgatgtggac gtcggtcact tggtccgggg catcgaggat
2461 gcgatccacc tgcacctgtg atgccagctc gacgatgtgg tcgatctggt attgcagggt
2521 ctcgatcgcc ggcgccaagg cttcgttgat gcggagcagg ccctggtcgg tgagcagggt
2581 cacggccgcc tgccaggaga tccgcacttc ctccagggcc gcgatccgca ggtcgatatc
2641 ctgaaggatc gcattgaagg tgtcctcgga cagcggcgtc acgccatcct tgaagcggta
2701 tcggtcaaat cgggtcggca tgctcgatcg cctcccggtt ggacttcacc acgtcccctt
2761 ttaggatgat gtcccagccc ggatacagga cgacgccatc caccacgatc ctttccgcca
2821 agaccacgcg gtattggccg tcgtcgtcga tgagggtggg atcgattgcg ttaaccgttg
2881 tctgcttggc catggttttc tccttacgct gccacgtcga tccgctcgac taccgcgaac
2941 ggctggctgg cggcgttgcg ggtgccgctc agcttgattt cgtaggcggt cgaggcgggg
3001 atggtgaagg tgtagctgaa gcgcttggcg ccgtcctcgt cggtgatcac cgtggtcgat
3061 ggcgccacct cgttgctgcc gccggtcacg atccggggcg tcaaggtatg gttggcggcg
3121 tcccagtgcg ccaccagcag cttgagcgtc accgaggtgg tggacgccac gctgcgggca
3181 gtggaccaat gcacgaagct ggtgtccggc cggctcgcct ggatcgcgtt gttggtcagc
3241 acgaaggccg gcgccaggtc tgaagtcccg agcagcaccg cgcgcaggtt gaccaggttc
3301 gggccggtgg acagcaccag cgaaggatcg cccagcgggt accaggcacc cgagccggag
3361 ggctggatct cgtagcgcag ctcggtgcct tccggcacca cgtgctgcgc cgcgatggcc
3421 aggtcggtca gtccgccgga cagcgacacc gactgcagct ggacctcggt gcggacacgg
3481 gcgaacttgg cgccgtagac cgtgaacatc agatcgcggg tcaggtcgcc gacgaagtag
3541 tcgccgtccg agccgtagaa cagcgtgccc tgggtgtagg cattgccgga gacggtcgcc
3601 acccggtgat cgccctgggt gatcaggacg atggcatagc gcttgccgcc ttccagcagg
3661 acgtgcggca cgtcgatcac ggtctcggac gggtacgcct tgaggctggc ccgcggcagc
3721 gtgatcgagg ccaccgtctt ttccagattg ggctttccgc cgtcggtttc gcagacgatg
3781 acctggacgt cgccgttggc gccgatttgg gtgaagaaca ggccgagccg ggtcagccaa
3841 agggcattgg cgaccaggaa cgactgcgcc accatcgcgc cgttgtaacc ggtggtcgtg
3901 acctcgtcga cgtactgggt ggtctcgtgc tgttcgaagt agccgtgcca ggggagcgtc
3961 cactgatacc tggcgcccag cttcgcccag acgttgcgtg cgtaccagcg ggtcaggaag
4021 ttgccgtgcc agccgtactt cttctgccag gagacgatct tctgctcccg caccgtgtgg
4081 gtctgcacct ggtactggct gatcgacaga tcccccgcgt aaccctgggt ctggatgcgg
4141 gcggcgtggg tgtaggccgg cagcaccacg ttctggccgg agacgctgac gccggcgtcg
4201 atcggattga acaatgccag attggcctgg atggaggcgg cgaaagggaa cagcaggcca
4261 tggtcgatcc gggccgcgta gccgacgtgc ccagcgttgc tcttggcggt gtcggcgaac
4321 gcatcggact ggtagctgct gtagctggag ggcagttgca gccgctcctt gacgctcgcc
4381 aggtcctggg cgatctccac gaacatcgcg cggtcggcct tgtccgaaga ctttttcgcc
4441 agcgccgcca ggtccgtggc gatcgaagcg atccggggct cggccgccgc cttccagatc
4501 tcgatcccgt ccaggcgccg ctcctggtcg tagccgttcg gcagcagcga ctgggtctgc
4561 atctcgatcc gctcgatgcc ggccggcgtc aggtagacgt gggccacggc cagcgtaccg
4621 gtctggatca ccggcggctg cggatccgcc gactcctgtc cgctcagcgg attcacctcg
4681 catttgcgga tccgctgcat ggccacggcc tggggttcgg tggcgccggt ctgaagatcg
4741 accaggaagt cgcgcggttc gacgctggta tccacttcct ggccccacag cacgacggcg
4801 acgatcttct tcgcgaccag gggaatgtac tggaacaggt tgatcgagac cggctgctcg
4861 gccacgtaga ccgcgccgcc gttgtaatag cggcccggct ggacttcgac ttcggtggcc
4921 gagaccgcgc cgaccccgaa gccggtgtag tgcttctggg cgctgatccc gtcggcgacc

4981 acgtgatcga gggagtcgcg cgcataggcc tgcagggcat tcgggtcggc cgcctggaac
5041 tcctggcggt cgcggaagat gacggttttt tccatggggg gcctcgtggg tcaaacggtg
5101 atgaactgtc cggacaggat ctcgccgcta cgcaccgcgt agccggcccg cacctgcttg
5161 cgggtatggg tgtccagcca cacccggtcg cggaaggcgc tggcgagcgc catggcctcg
5221 cgcccacgct ccagccggga cacactggcc ggcgcccagt accccgtagc aaaacgaccg
5281 atcaaacggg gcgggcggcg cgccggcatg gagacggcaa gctccgccgt gtaggccggc
5341 atccccagct tcatggcgcc gacgtgggtc gaacggccac ggcgcggcac gggaacggcc
5401 ggatcgaaca gatggaagcg gcggtacagc cggtcgcggg cgccgttgtc ggccaagtgt
5461 ccggcgacga aacggcccag catcgcgccg tgctcgatgc cttgttcggc gatggtcgtg
5521 tagcggatgt cgatcgggtc caatcccggc cgcaccgcgt gggtgtgcac ggactcgtcg
5581 aaatcgaggt aaggcgtgga caggcgcacg ttgaacaaac ggccgcctcc gtcctgccgg
5641 accaggtagg aacggggtac gagccggccg ggataggttc ccaggcccgc cgcgcccggc
5701 ttccggatcg ccagcgacag ttccgcctcg gcgccggcac gctggatttc ccgcaccagt
5761 gtcgtcaccg gtgtttcaac tccgttgctc ttcgagtcag ctccgctgct cttcgagtcg
5821 ttccccgacc tgtgccaaag gaaggcgcgc cagccgagcc ggaggatcgc gtcggtgacg
5881 accggatagt ggcggccttt cacgaatgcc gcgctgcagt accagcccga cccgatccgc
5941 acgccgcgcg tccggtagcg gtagaggcgc agctggggat agccggaaag gaacgcgtcg
6001 cgctcggcgc gggtcagcgc tggcgccaga taggtcttcg ccggcggcgt gatggcgcgc
6061 aggatcgaca gccccacccg cgaaccggcc caggtcagac cggccagcgt gcccttgagg
6121 cggtgatagg cgatcgccga cttgatcagc gcacgccgtt cggtatccgt ggaaacggta
6181 tcccagcctt cgagtcccat gacgtggaac tggtcggcca ggtgtggcag ggccgacggg
6241 tgtacctggt cgaccaggta caccaggagc gccgactgat ccagccggga gatgcgctcg
6301 atcagcgcgt ccagggccag gccgcgtgcg tcgcggagtg agggctgcag cagctcagcc
6361 atcggctttg cctgatgctg acccagcagg gcggcgagca tttaatactc cgtgatccgg
6421 gcgcggccca gatcagccag gcccgacagc atcgaccatt gaccggtcag ccgcccgcca
6481 tatcccgccg gcacctgcca aacctcgaac ggccccagac acacggtaaa ggcggatgcc
6541 gacgcgaacc cgccacactt gacgtacaag cgctcgaccg attcgttaaa gatcaacgcc
6601 ccgcgccggc cagggtttgc cggcagcaac gtcgtcgtat ggaccgaatg ctccaccacg
6661 gtcgccgctg catcgccccc gctcaatggt accggcacag gccgcaccac caaccctgga
6721 tcacttgccg ccggcgctcc atcacgaact gccgcggcca tgccggtatc cggatcagcc
6781 acccccaccg actccgaaaa tcccaactga ccgcccgcgc tgggcaccgc ccatcctgcg
6841 agccagtctt cgccgtttgg aatctttacc ctctgctgag tcatacgaac ccctgcgtct
6901 cgccaacgcc ttcaaaaatt acgctcacct cctcgcaatc cggccactca tgcgccttca
6961 gcacccggta accgggcgcg tccagctcca cgcgatagac ccccggcacc ttctgcgccc
7021 gttcgatcca ttgcgaaggc accaggtcgc ggcccagcgt cgaccgcagt tcggcggcga
7081 atccttccag gctggcgcga accccggcca cgacggcggc ttccgacacg gcgccgaagg
7141 cgcgcacccg cgccgagact gcaaacggcg cccggctggg cgggagcacc gtgacccagt
7201 cggtcaaggg acgaaccttg tcctggctca ggaccgcccg caccgcatcc agcagggctg
7261 ggtccggcag gccggaagcc gtcaggggat acacgttcac gcggcccggc gccggcgagg
7321 tgatcccgac gtcgacgatc gcctggctta ccgacagcac gtgccagcgg taggccaacg
7381 cgggaccggc cgcggcgaaa cgctccggcg ccagcttgat gcgcatccgc aatcgctcgt
7441 cgtcctcggc cgcgagcccg ccgtagctcg tcccgaggtt ggtgacggat agcccgttgg
7501 ccggcacgtc gatcagatcc cggatcccgc ccggcaggat gccgttggcg gacggtccgg
7561 caccggccgc agtggcgttc acctccacgg caatcgcccc ggcgggaatc gtcacctcgg
7621 tgtcggtggc gaattcggct acgcggctcc tcgagtcggt gccgccggcg gccacgcggg
7681 tgcctttggg gatgacgacg gacgttgcag cgggacgctc cagactgaac cgcagcacgg
7741 tgcgggccga ttgcgccggc agccgggtca ccccgaccag ctcgcccagg taatccagca
7801 tcggcgcgcg gctgaagtgg accaggttga gcttggccgc ttcctggatg ccgatccgcg
7861 ccagggtctc gcggtaggcg atgagatcga tcaggagccg ctcgacctgg gccgggtaaa
7921 gcgtctttcc ggtcatctgt tcccagccgg cgatcagcgc ctgggtcacg gcttggccgt
7981 cgcggtcgat gaaatcgggc tcgggcaggc tcacaaggtc acctcggtgg tccgggtctg
8041 ggagtcgcct ccgctgctct tcgagtcgac gccgcggaag cgccaggtga tggcgagcgt
8101 cacctgggcc aggccgaagc tgggccggat cgaaatcagt tcgatccgcg gctcccacag

8161 caggatggca tcggcgattt cggcgatgag atacggcgtg gcctcgatct ccggccggtc
8221 gacgtactgc aggatgtccg cgccgaattc gggccggtga gggtccgagc ccttcggcgt
8281 ggtcagaatg atgcggatgg cctggtcgat gtcggccgca tcctgcacga tgtcgccggg
8341 ctggccgagc cggggctgcc agttgaccga ggcgattgtg ccgattacgg acgtcatcgc
8401 gccgcctcca gggcttccac ccgtgcctcc aattcccgga tcgcggcgag ggccagcacg
8461 gccaggcgtc cgtaatcgat gctcaagctg gcgccgtcgg ggccgttcgc cccgtcggtc
8521 acgggataga ccacttccgg cagcacctcc cgcgcatcct cggcgatgag accgagctcg
8581 atccgcccct gcgggccatc ggcttcgagg cggtaccgga tcggctgcag ggcgcggacc
8641 cgctccagcg catgcctcac ctcgcccagg actcgcttga ggcgccgatt ggacgacacg
8701 gtgaacgcgc tggcgtaaca aggcgcgtag gccagggagg tattgcggac gtggaacccg
8761 cccccgaccg gcacccggac cccgccgtcg gtcgacgtca tggcatagct gccgggggag
8821 ttggcgaacc ggtaccccac gtcctgggtc gcctggaaaa agtcggcatt gaactgattc
8881 gccgcgtcgc cgtaggtcgc tgcggcgccg acctgggcgg cggtcggata ggggtgacgg
8941 tgatcgctgc gggcgaccgt cgtcgccgcg cctgccgact gggtggctgc cagggcttgt
9001 gccgagctcc cgaagccggt gatggcattg gcgggatgcg tggtcgccat cgcgccgacc
9061 tgggcagcgg tggggaaagg atggacgtgg tccgaacgcg cgaccgtact tgccgacccc
9121 gctgaaccgc tcgcggcaag ggcctgcgcc gacgcgccga accccgtgat ggcattggcg
9181 ggatgcgtgg tcgccatcgc gccgacctgg gctgcggtgg gaaaaggatg gacgtggtct
9241 tcccttgcga tcttgtacga aacgccgggg gacgccgcgg ccgccagcgg cgaagcgctg
9301 acgccgaagc cctcgaggac gctggcgaag tgcgtgtccg ggatggcccc gatctgcacg
9361 gcattcggga aggggtgcac gtgatccgag cgggcgacgg tcgaggccga tccggcagac
9421 tgggctggcc caagaccgta gacgaagttc ccgaagccgc tgatcgcatt ggcggggtgc
9481 gtgatcgcca tggcgcccac ctgggcggcg gtggtggcgt gcgggttgtt ggcggcatcc
9541 acgtgcgcct tggcggccgc cagcgtgatg tcgggcgcgg ccgtggccga ggctttcccg
9601 gtcacggcct tgagcgccgc cgcggtatca tgcagggaga tggccggcgg cgccgtcgat
9661 gcggcttcgc cgagtaccgc cttcaaggcg gcatccgcct ggtgcaggtc gatgtccggc
9721 gcggcgaact gatccgcttt gcccagcaat gccttgatct ggccggtcac ctgttccaga
9781 tgcatcgacg gcgcgtccag ccagccgggt ttgccggtca cggccgggag catggccttg
9841 gcgttggcga tgaactgctt caggctggcc atcagaggtc gatgtccccg tcgacgttga
9901 cgagaccggt caaatcgatc gccggggcgg cgacgctaac gcgggtcccg ctgatgctgg
9961 cggtctgggt gtcggcggtg agcgtcgatg ccgtcacggc gaccgtgtcc gcttcgacct
10021 gggcggtccc ggcctgcacg ttcactgtgt gggcctcgac ggtcacgctc tgcgccttca
10081 gcagcaccgt cccttgcatg tgcgtcaggt cgatcagcag ctgatgggag tccttgcggt
10141 agtacaggat ggagccgtcc tcgaaccaga cgccgtacca cttttcgtcg acgccttccg
10201 gcaccggatc cgggccgctg tagatcgccc cgatcacggt accgtcctcc aggtgctcgt
10261 ccatgatgca ggccacctgt tcccccacct cgggcatcca gaacgccttg tccttcaggc
10321 tcttctggca ggacacgctg agccagtgcg acaccaggcc gtcctggtcg gggaacttcg
10381 ccttgagccg cgacttcgcc gggtcgatgg cggtgaccag cccatagcgc agatgggcga
10441 tcttaggatt ggacacggga cgcctcactc atcacagtaa cgcccctcta tgggctctcc
10501 agggagtgga ttgcaacgac acggtagacc tccgcttctg aggcatagcc gccgctgcga
10561 tccagccggt gggtcgaagt ggtgacgtga tacttgccgt cgagcttgaa caagccggtc
10621 agatcgagat tgatcccggc caccagccgg gtgtcaccga ccaggccgag ggtgccggac
10681 aactgtttct tgtcggcctg gtgcagatgg gaggacgcct tgcgcttggc gatgccgcgg
10741 tgcttggcgc gcttcaacag cgcatgggta tccccggtct tgatgttgcc cttctcgtcc
10801 cagtcgtaga ccagggcttt ctggtcgggg tcgtggtggc tgaccttgcc gtcgggatag
10861 gtggcttccg atttctccga gagggtgcag tgtttgagct gccggcgatg caggctgaac
10921 gcggacggcg ccgcctcgag cgtcgcgatc gaatggaagt ccagcgcatc gccgcgcacg
10981 gcgaaggcat agccgtagtc ttccgccagc cgggtgagga aggccaggtc cttctcatga
11041 ttctgggtca cgcgatccag cgcgatgccc tcgatgtcgc cgcgcacggt caagccgttg
11101 cgggccgcca cctgttgcac gatcgcgcgg agcgaggtgt cgtcgtaacc gtaggatcga
11161 ggcgtccgca gcttgggtgt gatcaccgtg gccagcgcct tgatgcgcac ggtgtccggc
11221 atgacgtcga attcgatctc gtccagctcg aagtcgccac aatcgagccg tcggccgtcg
11281 gcatagccga tcgagacgga caggcgcgcg gagatgtccg gataccattc gtttttccag

11341 cgctgctcgc gatcctcgat cttgagttcg acgctgtccg attcgccgtg ctcgtgatcg
11401 gtgtagatca gctcggtcac cagacccgag atgtccgccg tgatgttcac gttgtcgtag
11461 atgaccgaat agactgggtc ctcgaccggc gtttcaactc cgttgctctt cgagtccagg
11521 cggggggtca tgccacgcct cccgccggaa cgccgcgctt ccacggaggc aggctttcca
11581 ccgagggcgt gctgtccgac ttcgcgagca ccggtaccgc cagcgtcaaa cccgagggca
11641 gcacgggaac gatcgggacg tgtggattag cgacgaccag cggctcgtag cggaacggat
11701 cgccatagaa gcggtaggcc agcccgtccc agcggtcgcc ttcctgtgtg atgtaggaca
11761 ggtaggtggt catcggatca atccgggaac gggctggcgg gtgaaggtat cgtatttggc
11821 cgcaagctcc ttgagccgga cctggctggc cgtcggcccg aaggagggat ccttgagctg
11881 ccgggccggt gcgttctcgg cgatcgcttc agcctgcgcc cgggcgttgg cgttcaggct
11941 gccggccagc gacgaggacg tgggctcggc ccattccttg agctggatgc gtacctgcat
12001 caggtagacg tttccgaacc ggtcggtccg ctccagcgtg cgctggagct cctcgatgac
12061 gaaccggcct tcgtaggtgc cgttggcgaa gaacatcgca agcgcgttgt ggcgcgaggc
12121 cgctgttttc agcttctcga actcggccct cgggtcgcag aagcgcgcat gcaattgcgc
12181 cgacagcgcc cgcgtttcca gctgatcgcc ggtccattgc agatggctct tgccttcgat
12241 gatgtcgtgg ctggcgtagg catagccgta tcccgcctcg aatccgccga gccaggtgat
12301 cagatcgagc tgtacgttgc ccagttgcag gtagctcatg cctaaccctg taaatccagg
12361 agtcagggta agccgtccga ggggccgagt cgcttaaacc ggattaaaac aacccggcgg
12421 aagcggcttc tgtgggagcg actatgccgc gatcatcacg cagccactcc cgcaccgttt
12481 cagctacgct gctcttcgag tcctcaccgg tagcccgtcc tcgccctggc atggctggcc
12541 tgcgcagcca cgcgttcgaa gtcgcgcagc gacaactcga gcgcttcggt gacggctgcc
12601 ttggccttgc cggcatcctc cgcgccgctg acgtggatcg tcgggttgaa gtggatcacc
12661 acctgttcac cgctgcgccc ctggggctgg tgcacggagc ggaaggtgcg agcgtgtccg
12721 tccgcgccac tggcgacatc gctgccctga cggactcgaa gagcggcgtc gccgaattta
12781 cgccccaaga gacgcgcagg ggtgtcggtg gtcgcggcgg gcgattcaac tccattgccc
12841 ttcgagtcgg cgccgttgcc cttcgagtcg ggtttggcga acggcaatac acgccccagc
12901 tcccggccca ggccggcatc gagggatacc ggcgccagtc cgctgcgctc cgggcctgtc
12961 gaagaaccga gttcggtgtg gacccgaatg acaggcggct ggggaaaccg caccagcttg
13021 ccaagggtct ccgccacggc gtctttctgc ccggtcacgc cctcaatcaa tccctgtccg
13081 atgtcggcgc cgtagccggc gaatacgcgc gaaggcgaat gaatccccag cgcgctcttg
13141 aaggtgtcct tgatgccgga agcaatgctc gacaaccctg ccttgaccga ctcccatttg
13201 gccatgatgc cggacctcag tccttccagc agattcgagc cgatggcttt gaaaacgtct
13261 ggcagcgtct tgaaccagct cagcgcccgg ccaaagatgc ccttgacctg atcccacaaa
13321 ggcgcgaacc tggctttcaa tgcatcccag tttttccaga tcaggaacgc cgctccggcg
13381 atgatggcaa cggctgcgcc aatgggcgtg aacagcagtg cgcggccgag gaacatgacg
13441 gcctgagccg ccagcttgag accggaaatc aatgcgccac caagaatccg tccgagctct
13501 agcgcggcca gccaggccaa ccgaaggccg gcgagagctg agccgatcag aatgcgaccg
13561 agccccgccg cagccgtact tgccaaacga aggccagaaa gcaaggcccc ggcggagaaa
13621 cgtccgagtt ccagcgccgc cacgcccgcc agtcgaaggc cggcaaggaa ggaaccgatc
13681 aggatacgac caaatccagc cgcggccgcg ccggccaaac gaaggccaga aagcaagggg
13741 ctggcaatgc tgcgtccaaa ccccagcgtt gcagcgcctg cgagtcgaaa gccggaaagc
13801 aaaatcccgc ccagggcgcg accggcgccc agcgccgccg aacctgcggc gctcaaagcc
13861 ccgccggctc gagccagcgg cccgaactgc aaagcggtct tgagcagtgt ccatttaccc
13921 aataccaatt gagtcgccgt agccaacgca ttgaagggcg acaccagcag gttgacggca
13981 tatctcaccg ccaaaacggc aagcttccca cccagcaagc cggcggttaa cccgaccaca
14041 ccccgaatca cgcccggatg tgccgaggcg aactgcccga attcccggat gacgggacgg
14101 acggtctgca acagatccgt gagcggcggc agcagggctt cgccgaccac caggccaacc
14161 tcactcatcc caatacggaa ggctttcagg ctttcattga aaccctgggt gcgcttttgg
14221 aagtcggcgc ctatcaagtc ctgattcgcc gcgccgagcg cgccttgctt gatgtccttg
14281 tactcggccc ggttcgccag catcgggcgg atgaaggaca tcgcctgcat gtcctggaac
14341 aattcaccca gcctgaacga accggacaac gcctggaggg cctcggcacg tttctgcgcg
14401 tcttccaggg acagagcctg cttgaatgcc gcggccgcct gcgggccttt gctgccgata
14461 taggcggtga tggtatccat catcgcctcc atgggactga tgccgcgctt ggcggcattc

14521 ttcaagctgc tctgcagatc gataccggcc ttgtcgaagt ccttcaacgt gtccggcgag
14581 gtcagtttcg ccagaaaatt gcggaagttg ttggcggctt cgtcgttggt gcccgcaccc
14641 ttccgggcga tctgtagtgc cgcaccgatc tcggccacgg cttccttgcc cgtcacgccc
14701 agggtctgga acatcggcgc cagcgccggc agccatttgg cggaatcgcg gatctcgaac
14761 tgcccgcgct tgccagcgta ggccagcatg ttgagcgccg actcggattg gtccgcggaa
14821 accttgagat tgttgtcgaa tgccagcagc acgctgccaa gatcggccat gctggcccgc
14881 gtcgccgtgg ccgctttcgc aaggaccggc gtataccggt ccagcgcctg ggcatcctgg
14941 acacctccgg ccaccagcac cccgatgccg gaagcgatct cggcctgggt ctggttccac
15001 ttcagggcgc tctcacgcac cgcggcgccc aagcggtttt cctgcgcggt cgtgaactcg
15061 ccggtgatgg cgatatcgcg gagctggtcc tggaaattgc ccgccagccg gacggacgcc
15121 acgaccggcg cgcccagcgc cgcgccggtt ccgacggttt ccaacgcctg cccccgcaaa
15181 tcggcgcgct cctgtttgag cgcggctccg cgcgccagac tggcggtcag cgcctcctgt
15241 cgccgtcgta tctcgtcgat cagacgcccc agccgttcgt agtcgcgatg aagcggcgcc
15301 agggattctt gcgccagcct gccggacttc tgaatcgtgg cgcccagcgt cttctgcttg
15361 tcagtcaggt cgcgcacgct ggcgcccagc gtgtcgatgg tcttcttggc attgccgaag
15421 gcggcggtaa aggcgcttga cagcgccgcc ccgatcacga tcccgaaggc aagctcttta
15481 gccatgtgtt aaccttgctt cgcccggcgt acagccgaga gtggattgaa acgtgaaccc
15541 gccgaatccc tttgccgcgc ttgccgaaag gattgagaac atcgaacaac gcgccccttg
15601 gattgcgtat ttcgcactct ttggagccgt tgtcgcgttc tgggttgcgg gcaagtccat
15661 catcgccgcg gtgttgttgg cgctgctgct ttcctggctg atcggctatt tgatggccgg
15721 cgccctgttc gtcttgtggg tcatcctgcg gctgttcgac ggcattacgc gcctgctggt
15781 ccggcctcca gcctgatctg ctggttcgcc agatcccacc aggtcaggaa atcctccagc
15841 tccagcgcat cgatctcact gggctggaac cggaaccacc gggccagcag cgccatcccc
15901 ttccacagct ccgccgtccg gatcagagaa ccggcgaaag ctgtcctgca ggcgcctgta
15961 gtccgccagc cccagttcat ccaggtcctc tggcacgacc ccgaccaggg acgcgatcaa
16021 cgccacttcg acgtcggcat ccgaaccgcc gaaacgctgg gccgccttca ggtccttgac
16081 cttgggcgcc cgcatggtca gctccgtcag ggtgcggcca tcgccggtct tcaagggttc
16141 gtgaagcttg atcttgttca tggttcgatc cttttttgca atgcgtcggg gggtaacgat
16201 ccgctagtcc acgagctggc cgaccagctt cgccgcaagc gccttgatga cctcgaacga
16261 caaggcgagc ccgcggtctt tcgcctcggc tttgactcgc gaccagagcg tgcgactgcg
16321 caaggtcgcc aggaattgat gtccgctcca agtcaaccgc gtcagaaaca ggggggatcg
16381 gccctggggg taaccgtcgg ccagtccggc ctcgcacaac agccgaaagt gctcgatcgc
16441 cacttcttcg gccacgccgg ggaaggcgcc cggcgccacc ctatcttttt cgtccccgag
16501 cgcttcggca gcctccagca cagaccgcgc caaattccag tccagacgca tatcaacccc
16561 cgatgttggc gcggtagttg gccagcatgt cctgcccgtt gaccttgtag atgttggcca
16621 gggcgtcgaa ctcgaacacc tcggcgccgt cgacgacgag cttggcgtaa tgcacggcca
16681 tttcgttggt ctgctcgacg ttcttgtgct gcttgtagtt gcccatcggg atcgacttga
16741 actgtccgat caggtgcgcc accaccggcg tttcggcgat ccggccggca gtcgcatacg
16801 tttccagcga gccgcgcacc tggacggcca ccgacttgaa cggattcgcc gctttggcca
16861 tgacctcctt gtagaaggac gaccacttga acttggcggt catcttctcg atgccggacc
16921 aggcctccag ggtgccgacc atccccagcg ccatgtgctc ctccatcttg tgcttgatgt
16981 ccggcagctc gacctcctcg gcccggccga ggaaggatgc gccgtcgagg tagacgttgg
17041 cgttggtgag cctgttgatt tctatctttg ccatatactt gtcctcacac tcctgaacgc
17101 gagacttcga tgacgcctga tctttacgcc aaactggccc tgcaaatcgc gcataccacg
17161 gctaccctgg aaacgctcgt tcaagacgtc ctggatgttc gggcgagact gatccgccaa
17221 gcttcgccag agcgagatct agaactgctg ctagccgagc ttcgcacgga gttcgaccag
17281 tcccggcagg aaaacatgcg cgctttcgtg acatttttgc agtcagcgcc tgaaaccggt
17341 tcagattcct gagccatacc tcatccatca gccgttacct cccaactgcg acagcaggtt
17401 gatgtcgatg aagctctcga agctcacccg ctccagcggg ggcggcggca tgaacgtgat
17461 gtcgaaggtc aggtgcccga gggcgatctc ggtcaccggg ttcttggcct tgtcatagcc
17521 gcagtggccg tcgatcaacg ccccgcgcat caccagggtg cgcaggaaca ggttcacgct
17581 ctcggtgatg gcgtcgatca gggcatcggt gatcggctga tccaggaatt gcagcatcga
17641 atactcgatg ctttcgtgga tcacgtcggc ggtgcggcgg atgttgatga agttgcgcgg

17701 atgcgtgttc gacggccagg ctgccgagcg gttgccccag gtgcggatcc ccgatccgaa
17761 cgcgttgaac accgtgatga tgccggcctc gttcagcaga ttggtttccg aggccggatc
17821 gttgatcatc gccgtcaacg gcagctcgag gccgacgatc cccttgatct cctggttcga
17881 cgcactccac cagtaaccct tgtcctggtc ggtcgcggcg atcctccccg ccaggcgcgg
17941 cgacatcggc tccaggattt cggcgccgga ggcgctgtcg tacaccttca gatgcggata
18001 gcacaggccc agccggtccg agctgtaatt gaagttgatg gcgccggcgg ggccgcgccc
18061 cgttagcgcc tgctgcaccg tggtgccgat cggcgcatcc actaacgcga tggcgcgcag
18121 cttccccgcc aagacgttga gttcggtcgc gacggcgttc acggtcgaga atccgggcgc
18181 gatcaggatc ttcgggaaga atcccagttc ctggtaggca ttgagccacg cctgcatgcc
18241 ggtccggttg cccgcgccat cgaccgcgcc gatgatgtcg ctgttcacgg ccttggaggg
18301 gtccagccag gaatagctca ctttcaccga ctggccgctg gcgatggcgc cgccggcctt
18361 gcgtgtgatc gtgccgttgg cggcatccag ggtgtaatcg gtgttgagca cataggtcgg
18421 cgtgccggcg gtgtgcttga ccaccacgtt cgatacctgg ggatgcgcca gcgtcagctt
18481 gccggtcgcg gcatcgaagg tcttcgcctc gtccgccacg gcggtggtgt cgctcgccgg
18541 atcgagcacg ttcaccacca cgcagatcgg gccgttgccc tggtcgaaga tggcgttgag
18601 cgcgctcgga atggtgaagc cggcacggtt ctggccgaag taccgggcgg catcgcgatc
18661 gttggcgacc agccggatac ggttcaacga gcgatcggcc ggatccacgt ccagcatcgg
18721 cgcggtgccg accagcccga tcacggccgt tttcacaccc cggatcgggc gcggcccctt
18781 gtccagctcg atggtttcga cgccgtgtag aaaattggcg ggcatgttct atcactcctg
18841 ggggggcgcg tcggcgccct tgggtttgcg gttggaggcg atggccgtca ggaaaccctt
18901 ggccaccagg acccctacat agtcgttgtc ttccggcagc tcgacctcgg cgcccgggtg
18961 caggttgaca tcaaggcccc ccggcagcgt gacgctgctc accgggccgt cgtagcggta
19021 tcgtgtcatt gcgcatactc ctcgtaagtc gtgctcccgt ccggctgacg gaccgtctcg
19081 gcccgctcat agccgtccaa cgtggtgaca tgtttcaaga gcggtccgtc ctcctcgcag
19141 ccgtcctcga tcgccagggt cggggcgcag aagtcgaccg cgtagcgcca ctccccgccg
19201 tgctcctcca ggaacgcatc gcggagcggg taaactttgc cgaaccccgc cggcttgaac
19261 ccggtcaaca cccgccgcgc ccgctccaga taggcgtaga gcccgtcctg cccgttgagc
19321 gcccggaaca ggaaggtgat ctcgaccgcc aggaggcgct cctggaccac cgcatccgtg
19381 tccatcagcg cgccgtagtg gctgccgtga taccggacca ggagcgcgcc gaccggatgc
19441 agaaactggt aattggccgg atcggccgga tacggcgcca cctcgacatc cggcagcgcc
19501 gcgcccagcc gggcgacgat cgccgcttcc agcgcctcga tggtattggg cgccacgctc
19561 atcggtaccg gttcagcagg tcgggcgtga acagccgctc accccggatc cgggtccgca
19621 tccgggcgcc ggcctcgggc tgagcctggg cgggatcgtc cgccacgccc aggctgatac
19681 ggccggcgcg gatgtcgtcc agcgtctgca gcgcgtcctt tcggccgcgt accacggcgg
19741 gcggcagatc gtccttgcct tcgggccgcc ggtcgtagat ctcgtaacgc accagctgca
19801 ccacgacgtc ccgtacgatg gtcggcacct ccgccagcgg cagcagattg cgctgccgca
19861 ggtacccgtc gaccagttct tcctggccag ccacgatctc ctcgaccaga tccaggttcg
19921 gcgccgtcgc cggcggcgta tcgttggtca gctgcgccaa cgtacggggc gggatcttct
19981 tctgcagatc gtcgagggtg cagtagcgca tggcaggacg tcagaccgcc ccgaactcga
20041 ccagggcttc cgggaagaga cacagcgcca gcgggttggc ctgtacttcc aggtcccagc
20101 ccttgttgaa cttccgcggt tcgcctttgg aataataggg ttgcccctgg gtattcaccg
20161 tctcgttgta attggccggc gcgttgtgca tctggaatac accggcgcca agcgggaaca
20221 ggcgggcttt gccggccggg atgaaacgct ggccggacac cgtgacgtcc agttcgaaga
20281 agcggacgcc gccgaacgtg aagtcgttgc gcatgtcgcc tcccagccgg tcctgcgccg
20341 cctgccagtt ggcgtaggcg gcctgtacct tggggtggtc ggtcaggagg tccatgaact
20401 ccttcgaggc cgcgcaggca aaccccgtca cggttgcccc cgtcagcttc tgttccgcat
20461 ggcgcttgcc ttccacgatc ttcgcgcgga cgtccaggtt gttggtgccg aaagcgatgt
20521 ccaccgtttt cttggtgacc ccgaactcgt tgtagaggtc gtagaccacc gtgccgtccg
20581 cgtcgaggat ctgcccgccc agcgcgccga cccgctgcca ttcgcgggtg atctcgatgg
20641 aatcccgcat gatctgcagc ttgtcgttga tgacccgcgc ctgcggctcc agtgaagatc
20701 cggtcatgtc ttcaccgaac ggagcgatgt tctggatgtc ctccggcagg atcacgcctg
20761 cctcagccag gtggaaggtc tcgaataccc ggcgtgtccg ccgcttgcgc cgcaccacct
20821 cgggcgcgtc gttgcgggac cggttgggca ccaggtgcag ccgtccctgg tcttcttcga

20881 tcgctacggt cgtggtgcgc acgcctgctt cggcgaagat gccgaggtca cggatcttgt
20941 aggtcgcata cggcagtttg ttgaccgcgg cggtcagggt agtgactgtg aacagatcgg
21001 ctagattcat ggcgtgcctc agacggagtc gatgaccttg agtcccagcg ccagcagcgc
21061 ggcgcgggca gtggctttct ggggatcggt gataccgctt ttccagacca ggccattggc
21121 gtcgagcacg gcgccgcgcg cgatgacggg cgccgcgaca tccgcgccgg cggcggcagc
21181 attggccagc aggaccgcca cggcattctg ggagccgtcg gcagccgcgg gatcatgcag
21241 tttgtatttg ccgctggcgc tgatcctgcc gagtacgcta ccgagagcga gatcggcgcc
21301 agcggcgatc gtcacgcggt cgcgggacca ttgctcgccg acctcgtaca gcaggacgtc
21361 ggaaatgacc ttcgggtcgg tatggcttgg cattgcggcc tcctcttaag cgtttccggc
21421 gcggcgttcc gcgtcggcga caaggggggtt cttgagcgga gcccggtcgg cccggtccgc
21481 ggtggcgaat tggccgaacc gcaccaccgc accgccttcg gcgaacatgg ccttcacggc
21541 gtcgaccagg ggacgggtcg aaccgtcctc gccggcgaac tggggcagac caccctcatc
21601 ggcgccttta ccggccagca tgagcaactc gaccaggagg ttcttgtgct tgggcgccag
21661 ccgaacccgg ccgttgccgt cggggcgggt gagctcctcg gcgaactgga ccgcgtcgtc
21721 ccggcgccgg gcttccgctg ccgcggactc gcgctgacgc gctgcggcca gctcggcttt
21781 gagccgttcg ttttccgccg tcaaggcggc ggcttgagct tgatccacgg tgttgtcctc
21841 cggagtggat tgggaaaaaa cgggttggga tgcggttgcc ggatcgcccg cttcatcgcg
21901 gatttcctcg cgagcggcat cttccagata ctgcaggtcg gcactgggca cgacctggtc
21961 ggcatcctcg gttccgaacc tgaccagcag ccattcccgg aatcgccgcc agaggccggc
22021 gttcacgctg tcgtcgcgtt ctccgctgaa gcggatgatg ccgtcctcgt ccgcggcgaa
22081 tgccgggttg agcgtgggcc ggcgcaggcc gcgcaccgcc ggcggcaccg cgccgagaaa
22141 ggagacctcg cggacgtacc acttgccggg aacggggttg cggggatgat cgggggcgta
22201 ccagccgacc gagacgttcg ggaacgcctg tttgttcacc agctcggcga aggccggctc
22261 gacctcctcc ggctcggcct cgaggccgtc cgggccgtag ctcaacgcct tgacgtggcc
22321 ataggccgga tcgagcctgg tcgggtggcc gatcaccagc ggtgcccgat gcaaagccgg
22381 gtcgtagcaa gcggccgcgt cgcgcagatc ccgctcggag aactcgatcg gctgggaccc
22441 gcccatcggc aggtggcggc ccggcttgaa acagtggatg cggttcgtca tgccgccaga
22501 ttaggcggcc ggaagcgggg tttcttttag cgtggattaa aaacgtgcgg gaccggcgga
22561 acctgatgaa gcctggtagc tccgattagc gcagcgtaat cggaggaaat cccggtcgat
22621 gatcgcagca ggggtaccgg ttgagcaaga acatgccgct gtggcgcgtt aaaccccccgt
22681 tagaaatcgc gcaggcagaa aagacgatcc aaggtagcgc atgggggctt tacgcgccgt
22741 cacgggccgg agattcgcag cggcgcgtct gcccggggga acgcgcctca gacgcccgca
22801 gcccgcttaa ggtatgccat cgtatcatcg agcaccgctt ctctggcctg gggcgtcagc
22861 tcgcctttgg ccgtcatcgg cagataggga cgtgctggaa tcgtcgttcg ttggctacgt
22921 cccgcggacc cgccgaactg atgaatagcg gcatagactt ttgaggagcc gatccgtgcg
22981 aagtccgcgc cggattcggt cgagatcgat gccgcgagct ggccagacac ctggagcatc
23041 tgtcccggcc agtggcccag gcgggtccgc gcctgcttcg tggaatccga aagcgcaggc
23101 catcccggcc cttcgttctc gaaccggtgt tcggtttcgt ccttcagggt catcgccagg
23161 gttttcatca gcggcccgag gtttagtccg gcccgctgga tgcggtccag catttgacgg
23221 acttcggtgt cgtcgagggt gacttcgatc gggttactca tcgcggtata ctctcctgca
23281 accgattgcg gccgtacaag ctcccgcctg acgcatccaa aggagccgca cggtccttgt
23341 catgaggcgg cgtggcaagg ctatcttcga aataacaaga agccatcgcg ctgccgctca
23401 atataacgac gacgtgcccc aggccctcga ctggcttgag aaatcatcgc tgtcgatcca
23461 gaccatcctt cgcttccata ctcaaaaacg gacaatccat attggggcga ttcgcttcca
23521 tgtccgtcga tctcgaaggt cttgagatag cggcgcttaa gcagccatgt cccaggccgc
23581 tctctcgact cttcccagcg catccaaatt tcatcgggtg atctgatcgc atgcgcaaga
23641 aggcgcatgt agggccctcg accgtctttg gttgctttcc actcgccggc gccggatttg
23701 aacaaagcct cgtcgatcag catcggatct ccagtgacgt cctgaaacac caccggcttg
23761 ccgaggtcgg caccgaactc cgctaagaac gccttcgcgt aatcctcctc gcccaaacca
23821 tccggcacga tcatcgacgc ggcgaatttt tccggcttcg gcagatcggg caggacctgc
23881 cccggcgaaa acgtcttcgg cagggtatcc agcggcggcg gggtgaaagg cttgctccag
23941 ccttggccgg gattgaagtt gaacccggca tccggcgcga agaaatgctt tttcccgtcg
24001 gcgtcgacca cgtcgatccc ggtgcgccga gcgtgtgtta tctccccggt acgtttgtcc

24061 acgccggcat cggtttcgag ggttcggagc ttccctgcgg aagactgcca ggcgatcccg
24121 tcacgcgcga ggcttcgttc ggatctcggc cggacccggc aacggcagtt gtgcacaatc
24181 acaccgccgg ccataatcag gcccgtctcc gtttcgaaat catagacatg gcccgcccaa
24241 tcgaattgcc gaatctggac gatctcatcc agcgctatca ggccggcgtt agcctcaagc
24301 aactggcgga ccaggccgga tgcagccgtg acgccctggg ccggctgttc cgtacccgcg
24361 acatccccct gcgggggcgc tcggaagccg agcgcgtcaa atggcgatcc atcaaacagg
24421 accccgccgc cattgctcgc caatgcgccg ccgcctggga gcgcgcccgc aaccgcgact
24481 cccatctcga acgccgggtg ttgtccctgt accgcaacgg gctgatctcc aaacgcgcca
24541 tcggccgcca gctcggcacc agcctcggca acgtcagccg gattctgcgg agcaacggca
24601 tccgccagga tcatacgggc cggcggcggg ccgtcggggc ggtcggcgtc catgatcgcg
24661 caagcgtcag cccggcggag ttgctgctgg ccgacgaatt gacccggcgc ggcctggact
24721 tcgtccatca gatggacatc gggacccgca atgtggattt cgggtttgcg caagcccgcg
24781 tcgccgtgga aatcgtgcgg cgccattgga acgatgcgaa atccctgcgc cgccagcgtc
24841 tggaagagat cttcggcgcg ggctggcggc tgttcgtcgt ttacgacccc agccagacgg
24901 ggattgacac cgcttgcgtc gccgatcagc tgatcgcctt tctggagttg gcaggccgcg
24961 atccagcccc ggacggtcaa tacggggtgg ttggcggtca gggtgagccg gtgcccgagc
25021 cgcgtttgca gctccaccac tttgcccgca taccaggttt ttagccccag ccgggcggcg
25081 gcccggaccg gcgtttccgc cgggaagcag ttgaagccat tgggcggcca gatcgtgttc
25141 cagatgggat cgtcatggcg gaacactcgg ccgttcatgg cccggtggct gggccgggtg
25201 cggccgtcca gcaccgcgac gtattgccaa taggggtggc tgtccgtggc ttcggccatt
25261 tcttggtaat tcgccgccat gaaggcggat tgcaggttcg tccggtagat cgtagccagc
25321 cgccatgggc tgcccagccg ggcgacctcg gcattcccgg cgccgtcaac gatgatctgt
25381 ttgccccacc agcccttggc ttgcagcgtg ggcttcaagt ttttttggaa gtccttgagc
25441 gtttggccgt ggcggagcgc gacgtcgagg gcattccgaa tgtcctgcac cacgtccagc
25501 cgcgccgcct tcgcaatggt aaaggcccgt gcgtgggcat cgtccagcat ctcctgccag
25561 ttccaggtaa gcttgtagcc cttgtgctgg aggtaggcga tcgcggcctt gggctcgaga
25621 ttgaaaatcg ccttgaggtc ggcggcgggc agggtcatcg cacgattgcc cgcgtcgcat
25681 agcgataggt gattcgtatc tgcccccgcg cacctggacc gccactggcc gcgtgggtgc
25741 cgccaacgtt tgcgccggac tggccgccgc cgccgccagg agcgcccccg gcggctccgc
25801 tggccgggga tccaaaggtc gtcccgttac ccccgccgcc accgccacca ccatcggcaa
25861 tgctatcgcc agcagaacca gccgtcccac cgctccccgc gcttggggag ttaccaccgt
25921 tgccgcctgt tgatcccgag gtaggcgtgt taccgtcgcc gccggcgccg aacggagacg
25981 ccgaggcgcc gccgcctgaa ccacccctgc caaagccgga actgtagccg ccattgccgc
26041 cggaatattt gacatcgccg acgcagccag aggcttgtcc gccttgaccc ccgacggtgc
26101 cggtggattt ggcgccgctc ttggccagac agccgtccga cgtggcggcg ggggccgagt
26161 ttgacgtctt ggaaaaccag caatctgctg gactaccccc ggaaattgcc gtcgttccag
26221 ggatttggag atagcaggtc tgattcggta tgacggccag attgatcgtt ttcgaatacg
26281 cgccgccgcc gccggaccca atgttgttgc cgttgttccc cggtgcgccg ccgccccagc
26341 attcgaccga gtccagcatc gcgacgccat gcggaacggt ccaggtcgtg ccactcgtca
26401 ggataaccgt aggcatgcgt cagcctgcca cgagcgtgac ctgaagcgcg atcaccgagc
26461 cggcaatcgc cgtggtctcc agccataccc agttcccggc cgggacggtg tcattgtcaa
26521 agctggtgct gtataaggtc atccccgtgg tggtgttcgt gcatgtcact ccgccgacaa
26581 ccatttcagt gccggaagcc agactggttc catatttgat cgtgaacgtg accgacggcg
26641 tgccgcttcc cacgagcacc gccttcaaat agccaactgg aaaatacgtt ggcgtgtaaa
26701 acaacggcac gacatcgaca ccggtcgggt acaagagcgt taccacacgg ggcgcgagcg
26761 ttcccacctc gaccaccttg ttgtcgctgg ctgacctttt cagaaagagc ttgccctcaa
26821 aggtattgat cgccagctct ccgtcctcca gctgctcggg ggcgggaata ttgccccagg
26881 tggcattgcg cttgtgctga attttgttgg acatcagaac gtaccgccat caatcgtaga
26941 ggcgttgttc agatagtcgc tgcccgccgt tgccgcgacc agcgcggtgc cggatttctt
27001 gaagatcgcg ccatcggcgg cggcggacag gttggcaccg gtgccgccgt attgcagacc
27061 aatggcggtg ccctgccaaa cacccgccgt caccgtaccg acagcggtga tgttggcttg
27121 gaccaagtcg atctcgatgg tcttgttgcc ggcattgagg gtgacggtga ttttgctgga
27181 agccgccacc aggttgcgga attgcagatc cacgccggac ttgccgtcga acacgccgac

27241 acctgcagcc ccctggttcg aggcggtgtt cgcctcgccc gaagcgcccc ccgacatctg
27301 ggcaaacgtc agggaggtcg tgcccaaggc gatagcgtcg ttggtcgtca gcacccaggt
27361 ttggtcgccg ttgaccgtac cttccgagac gaacatgcag gcgccggcag tcacctccgc
27421 attcgaatcg aagtcggtcg cgcggcccca ggctccagcg gcgaccacgt agacgccgtt
27481 ctgcgaggcc gtggtctgat tcttcaccag cacccggtca ccggctacca atgccacacc
27541 atcgactgtc tgggtaccgg atagggtgat gttggcggtg gtcgcggcgc ggaccgattg
27601 tttccagtcc agccccgacg cgaccgagtc cacgtacgcc ttggtggccg catcctgggc
27661 gccggtggga tcggccaggt tggtgatccg ttggctgttc atggataccg ccgcggtcgg
27721 cgccgccatc tgatccagac gggaggttcg cacctgggta tcgaaaccgg tgatgtcggt
27781 ggcctgatat gccagcaggg tcttgacctg agccacggta agatcggccg gatccgccgc
27841 gctgcccgta ttgttgccct tcagcgtgcc ggccgccatg ttggcaagct tggcgttggt
27901 gaccgtatcg ttcggaatgg tcacggcgat cgaggtcgca ccggatcccg tcgcatcgcc
27961 cgtcagggtg acgacctggt tgcccgtcag gtagccttgc gctttgacga aagcggtggt
28021 ggcgaccctg gtactgttgt cggccgccgc cggcgtcgtc gccgtggttt gagcgccgag
28081 gtcattcgcg ccggtaaagg tcttggcgcc gtcgatggtc tgcgcagtcg acttggtgac
28141 gaaggcgcct ggacctccga tcgccggaat cgaggttgcg ttgcctcccg agtcgccttt
28201 gccgtaataa aggacatcgt cgacttcgtt gtaggcgagc tcggcgttga ggagagcggc
28261 cggcgcgccg gcgttgccgc ttgccctgcg tttgatgcgt actgcgtttg ccatggttcg
28321 tttctctcga gttcaggtca aaaattcccg ccgtcgatga cggtatccgg atcgatcccg
28381 ccagccggtc cctccggacc aggcggacct tggggaccga cggggcctcg cggccccggc
28441 gcgttctcga catccacggt tggagcgacg tcgacacaat cgacctcaac ggttgcttca
28501 cacaccatcg gcgccgcccc gtggaacgat cagaatgcgg tcgtcgaggg gatccttcag
28561 tggtggacga agggtcttga cctcgcccga aggtgttttc gccagcagcc catacacaca
28621 gcgcttgttc gccggcaatg cggccgtctg ctcgggcgtg gcttccaggt aggcgatccc
28681 gtcctccggc tggtcagacg gatgttcgcc aaccaccgat ctcagcaaca gcagtggagg
28741 ttcggtaccg atagcggtcc gcaaggccag ccagtggatg taaccggcga tgttggaccc
28801 cttgggatac cggatccgga cccggtaggt atcgccctgg cggatgtcgg gcaggtactt
28861 acccattttg caggctctcc tcggccgcca gccgcccggc gacgctcgcg gcgaagatca
28921 gccgggtcag ggtttgcacc agctggtcgg gattcatgtc ctgataggct tccgccagtg
28981 cgccgagcag ttcggcttcg ccacccgcac tttcgatccg ctcgatgagc ggcgcgagca
29041 gccgttcagc ctgagcctgc agggcgccac cttcggccag cgcgtctact gccgcatcga
29101 gcgcggcctg atcggggaac agttcaaggt cgattcctgc gaaggccgga gcgccgctgc
29161 cttcctcggg cgccagttcg ccctcctgga aaccataggc ccgcatccag tagacattgg
29221 tcgggcgcac cccggcatcg tgcaatgcct tatcccgtac ggcccgtgtt tcgtcgatct
29281 cttcctgttc ccacagctga tagacgggcg gagccggttc gtggaaattg agttcgcaga
29341 ccatccggat caagtcgctc agggtactgg cgacgatctc cgcgtcgctc tcccggatgt
29401 cgcgcgtgac catgattccg gcctccgccg acgctttgtt ggcgctgact tcggtggtct
29461 ggttctggcc gagcagggcg atggcgacct cggagcggca gaacagcagc agcttttcgt
29521 aggcgtcggc cccttggctt tgatcatggg tcaggaactc gatgctggaa tcggccggta
29581 ccgcggctac gccatcgcgg accatttgtt ccaagctgtc gagcagctga tcgatctcgc
29641 ccgtttcggt cccacgggga tacttgccga ccggccaggg catgccgtac ttctcggcga
29701 actgcatcca gaatcccagc ccaccgcgct tgaaggtggt cggccagaac accagcgcca
29761 gatccggcgc gccgtacggg ttgaaatagg tcggctcctg gcgggcgcac aggaatgaat
29821 tgggcggcag cggctcgccc atcgggcagg cctgactgcg gaacattagc tcgttattcc
29881 gggagtagac gaaccattcg ggcggccggc cgatcacatc cgccggcacc acgaaattcc
29941 ccacgcgctc ccagacgatt tccagcggct ggtagccgta cagcacggcg tcgaggatct
30001 cgccgatgat tcgggccagc ggcagatcgg ccaggattgc ctcgatgttc ttggcggtgc
30061 gcgcgccagc tgaccggcgt tcgacacccc gttccatggc ccgtaccgcc gcttttcggc
30121 ggcgaacgca gccggcgacg tgcgcgtccg agagcagatc acgatagacc tcgatggatt
30181 tccccatctt cttgaggatc gggtcggggt tgggcagcca agtcaggccg ctgaactggc
30241 caagcgccgt ttggcgggtg accagttgtt ggccgagcgc gggaccttgg gcgaaggcga
30301 cgaactcgcc ggagggcaga tatagccctg tcggtgcacg acgagtcatg cgtacccccct
30361 catgcttgtg ccacggcgca ggtcaccgta gcctgacagg accgtaccgg tgcgagggcg

30421 ggaggcgaca atcggcatcg ttcccggatt gctcgcggca tggattgcca ggaaacaggc
30481 ccaggcgcga tcggcgtggc cggcgctgtc cgagtcggcg acgaatcgcg gggcgccggt
30541 ggcagaggtc tccttcttca gcttgtgcag atcgttgcgg agctcctggt tcccttcggg
30601 gatccggatc cggcggtcct cgaatgcctc cttccccgtg gtcgctagcg ccagcttgtt
30661 ggggccggtg aacagaacgc cctcaacgcg gctggagccg aagcggcgct gtgcatcttc
30721 gacaggtttc tcgcccatcc cggtctggtc catgcagcag cgtatgaccc ggtagcgatt
30781 gaacgcatcc gccagcaacg catcctgctc ggcaaatgaa atacgtcgcc tggccagaat
30841 ctcacgcgtc cagtacacat cgcctacggc ctccaacacc cagatcacga acaggtcgtt
30901 gcgcgccgcg atgtcgacgc cgagaaaaca aggaccaccg gcatagtgtt cggggatgcc
30961 ggcgtggtcg tgctcgaccg agttgatcag ttcgaacgac agccaggcgg acgcttcgtc
31021 cagccattgc agctcgtatt cctgcgccca ggcgtcgtcg tcgccgacgc cggccttcag
31081 ctcctcgatg tcacgtggca agccgtccgc cacggcctga tagatgtcgg tgacgtgcct
31141 ggaccagaca ggatggtcgc cggtaatcag gtcgtagaac ttgttgccct tgccgttcgg
31201 cgtggagatc acccgcagct tgagatcgga gcgcgacacc accggaaaca gggcctgcca
31261 aatcttgcgg ctgtcggcat gaaaggcgaa ttcgtccagc agcacggagg ccgtgaagcc
31321 ccgcgcggtg tccggattgg ccggcagggc ggagatgcgc gagccgccgg gcagcaccac
31381 ttccagcgcc ttgacgctgg cctcaaagtc gtattccagc gccttgaagc cggtctggaa
31441 ggcgtggaga tggcgcttga cgccttcctc catcgcctcc cgcgcttgcc gctcgccgcg
31501 cgagagaatc acccagcggc ggcggccgcc ctgcgcttcg gcctgcaggc agtccatcac
31561 cagctccagg gtggtggtga aggttttgcc gcactggcgc gcgaacatgc cgaccttgaa
31621 gcggctggca tcggcgatcc agcgctgttg gtaggggtag agggtaagcg cggtcatgcc
31681 agtccgtaga cctcctgccg gacgatccga agggtctcgg catcaagctt gcccttgccg
31741 gctctggcac cggcttcgag ccgatccagc ttggcgccgg cttcttcctg gagctgtttg
31801 cgcagctggt cctgccactt tttctggtcc agggtcatcc ggcccagatc cgccaatgaa
31861 cgggtggcca gcgaaatagt cttggccgcc ttggtgggat cgctttccgc ctgacgcagg
31921 gcgatcatga gcttgagcag gatttcctgc ccgaccatgg cggcggcttc cagcatcgcc
31981 gcttcgctat cgtcacgctg ggcgaccaac gccttcgaga gcttctcgac cgtgaacgcc
32041 gcctccatgg aggacttgaa ctccagctcg aactcctggc cgtagctgtg cagggcgctc
32101 ttgctgatgt ggtagccctg atccttcagc caggccgata ggtcccggta gccgccgaag
32161 ccggagccga ccaggcgggt attgagctcc tcctggacct ccggcggcag ctgatagacc
32221 gacgaacggg gcggcattat tcgcccggct ccggccgcgc cacgcccggc atcaccgtca
32281 gccccttggc gacgtccaga cctcggtggg tcaaagtcgc cacccagacg ccgccgggct
32341 gctggccgac gatcagatcc tgttcggcca gccaggcgag gtccgtcttc aggcggtcgc
32401 gcgagacggc cagtccttcc agcgccagcg ccttggccag cacggtctgg tcgagggtgt
32461 aggcgggcgc ccggatcaat cggcgcagga tgaccaggcg gcgctgttca gtttcgaagt
32521 tctccaggtt cacgatttac tccctcggtc gtgttcgagc aggtagcgat gcaagcggtc
32581 gaccgttttg gccgtacctt ccagggtgcc tttcatctcc cgcacgtcac cgctgacggc
32641 gttgatccgg tcgtagatcc gccccagatc gtcgtgattc ggcatcttgc cgatttggct
32701 ctccagccgc gttatgcgct cgtgcttgac cagacagcgc tgttcatagg tgctgatgcg
32761 gtcgtggaag tcggcctcga cttcgtcgat ccgcttcagg gtcgccttgt gcgaccggcc
32821 cagccagagc cacccgctga cgccgaggtt ccagacgaac aggaacatct cgaagaccag
32881 ccgccacttg ttgtaatcgg tcggctcacc catgcagttg cagcttggcc tcacgctcgt
32941 gttcaatctg gcagtcgata caccgaatgg cgtgttcgtt cgccgccaac cgttcgggcg
33001 ggatcggcgt gatgacttcg tactcgttca gacagtcgta acacaccacc atcggcaggc
33061 cgtcttcacc cggaatgacg acttggggcg tgtccggccg catcgaatcc aatacggcgc
33121 gaataccggc cacgcgttcg atctcgattc gctcgtcgga caggtcagcg agatccggca
33181 tcggtgcgtt ccgtgaattc gcattgcagg ccggcgccga actcgattgg acgctcgccg
33241 tggaggacgc tcagggtgtc cgcaaatttt gcgtaacctg tcggctcgca ggccagacag
33301 tcgcaaccgg caagcacacc gatgcagaag gccagcatca gcaacgcccc gccacccaca
33361 catttttcct gtggcgtcag catcagtgcc cttcggcccc gcctttgcag gcttggatgg
33421 cctcccgcgc ccgcccatag ccgaccacca gcgctttgcc gccggcatcg gctttgggag
33481 gccgacccgg ggcgatatcg acgaacacgg tcgaggccaa cggcggcaga tcgggcagag
33541 cacaagactc cggcgcgaat gccggtgtca ggatcggctt cggaaccagc tctttcggcc

33601 ccacctgcac gcaccccgcc agcgttagcg gtagcgccag aatcgccgcg cccgggaatc
33661 cgctaacgca acgcacggtc cacccccgct tccacatcct catgcttgag ccagcgctcg
33721 cacgccatcg cgccgttctc gtgggtactg atggcccaga ccagcagggt gagcgttttc
33781 cggtcggaca ggatgatccg gtcatccgcc cctacgtcga gcgcggcggc aacccgccgg
33841 atgtaggtgg cggtgtcgtt ctcgttcggc ggcgcccagc gggtgatgat ctggcggacg
33901 gtgtggagct ggcgcttcct ctggtagttg agcagcgtca cccctagcgc ccgcaccccg
33961 tcttccattt gctcgaaccg ggcaaaccgt ccgtcgtggc cgacctggcc ttcccaggcg
34021 ttgcgggggc tgtagacgag attgccgggg ttgttgttgc gcaggcccct tggcttctgg
34081 aagctcattt ctgctgcctc cgcacgaacg ccgcgaattc ctcatcgctc atatcctccc
34141 ctcgcccttg gggaagagga gccggggtgg cggccttttc gatcttgtgg ccgacgcccg
34201 cgatgccgat gccttgcacg atgaactgca tggcaatgtc cgcgccgtgc aggccgagca
34261 gccatcccag cagcccatag gcgaccgaga cgatcgcggc ggtcttggtc ttccagccgg
34321 tcacggcgca gtctccgcag actgcttctc gggcagcagc ccgaccgcca tttcgatggc
34381 gcggttcatc atccggtccg gaatccggtc cgcccaggcg atttcgcgct ctttcagctc
34441 cttgaggatc agcgcgatgg ccgcggactt cttggcttcg ccggacttcg gcgcccagct
34501 cagcgaatcc tcgacggccc gaaccatccg ctccgcgacc ggcagcagga tctcccactg
34561 ctggcgcatg ccatcggcca gcatcggcgc gatataacgc caaaggatcg gccacgcttt
34621 ggcggcggcg gtcatcagcc acgcccagaa tttgcccatg aagcagccct ccgaacccaa
34681 gaacctatgt gggcacagag tacggcgcgg atggcaccgc gtcgcttaaa gcggattaaa
34741 aacaaagccc gcacaaggcg ggctttgggg cgacagggta tggattgatt gagtggcttc
34801 aatcaccaac actcaacatc ctgagcaaag acactaaagc tatcaaacgt ctcatactaa
34861 tctcgctttg ctatctacaa atcgattcac atggaattcc gtcgcgatcc cgatcaagcc
34921 ggctcacacc gcagtcgttg agatagtggc gtgcttcggc gcagctggtc atctgcttgc
34981 acgtcgactt accgccacag tcgcttgcgg aaccgtcggc ctcatccgta gaacgcgcag
35041 ccttctttgg ggtgccgtgc cggaaatccc atgggggcgt cggattggca tcactccaca
35101 gaccgattct actggcgcga gactgttctt ccgcagccct caaagcgggg tcgtggccgt
35161 attcgacgta gaaccaggct aggccgcgct tgagctgttc cagattgacg tcaatacctg
35221 cgcagctcac gcgtcccagt ctccggccat atttgtcggc gccctgatcg ttaactttgg
35281 cttctttgcc gaaacacaag tcgctcaatg attgcttgga ttttgcgccg taatcttgat
35341 gggactccgg agcgtcgatt tctgccaggc gcaccttggc gccgcggccg cctttcatga
35401 tcgtgagcgt atcaccatca gtgacaccga ccactttgcc actccaatcg tctgcatgcg
35461 cagccaccgt cacacccatc agcaccgccc agataaatgt tctcctcatc ggattctcca
35521 ttaatcaaaa aggcgacgtt gtcgccggtc aatttcagat tgccgcaccc tcttgacgat
35581 gcggtagatc cactgtaccg acaaccggta ctccagagcc agctcggcat ggttgtcgcc
35641 ccggaacttc tcgaatatct ccaggtcccg ccggtccaat tcgccgggca gattccgcgg
35701 gatataaacg agctgcccac cccaatgggc tgcaacttcg cgcgacagct ccagtccgaa
35761 gcgctcggcc acatccttgg gcaacgcccc caactcgcag gcaagcgcgg cggactttgc
35821 cgcgaggtcg ctcaggagtt cacgagcctg ccgccctttt ttctcagcca tggcccggcc
35881 gctggaagtt gccgatctcc tgcaccagct cgttgatctg gcgctgcagc tccttgcttg
35941 gggcggcggc attcagccgc tggagtgagg cgagctgttg tttcagctcg cgcagttgag
36001 cgtctccggc caaccccgcg gaatacccc ctgacaaggg gggctggggg gttggcgcat
36061 agtccatcca gcgcttttcg tgcagccagc cctgggccat tttgcgcacc tgtccaggct
36121 gggcggtttc ccgccactgg cggttgtcct gcttggcggc gtcgatgatc cgccgcgctt
36181 catgttcggt gaggtcgccg agctggtacc agcgcatggc cgcgccgttg cggtcccgct
36241 tgaggccgta ggcggtccag aactggtcga accagccgcg ggacggctcg accagcctgg
36301 cgtaccagtc aacggattca cgcgggccgg tttgtcgcgg cgcgccgtag cctttggcgg
36361 tcagcctctc ccacagtgct tcggcgtgag caatgccggc gtcgacgcgg tccagcgcag
36421 gcagtgcgcg gatgaggcac gcctggatga aggagcgctt ggtgtgactc atttccgttc
36481 ccgctccacc tgttccatta acgttttgag gagtccaaaa gtcgctgccg catcatccgg
36541 attcatcggc atggaaacgc tcaagcgccc tatctctaag gacgcgcgct cgatgcgtat
36601 ctgcctctcc gcccaccact ggccccagcc gaatcccgca acgaatacga tcacgactag
36661 caggttcaaa ctcacgtcgt tcccccatgc cgtctagcat cgtaggtgag cgccgcgacg
36721 actttccgca gatcctcgtc ggtggcgaac tccaagcgct cggcgcggcc ccggctcact

36781 cgcttgagaa tgcctacggc gtaattccaa ggccggccag cctcggtgag ctgggcttcg
36841 atcttctgta gcagcggccg ctggtccgtc gtgtgcggcc ggcccggata gggccggctg
36901 cgtggctttg ccaccggtgg ctgggtgatc cgccggaggt gatcgagcac tttggcgcgc
36961 ccggtcgccg tgaggtcttt cgacgacgac gcgccaccga tctcgcgcaa catggcccga
37021 taggacgaat catccatccc cgccttggac ttcaacacat ggatggcgac caactgccgc
37081 cgcacgggat cggtgcgcgc catggcgatc aagccccgcc ttgaacgcgc tctttgagcg
37141 ccttgacgaa cgatgcgcgc gccactcgct tggctggaat ctccaccgtc tcaccggtct
37201 gcggattgcg gccggtgcgc gccgcgcgct ccttgacggt gagcttggca acgccgggga
37261 tcgtcacttc gtcgccgttg gccagggctt cctgagcgac attcgccagg gcgatcagga
37321 cgcgctgaat cacggttttg gtcagcgatc cattggcgtc ttcatggatg gcgttgatga
37381 gttgggattg gttcatggtt tgtcctcttc gaggttgtgg aaaatgggtt tacgccgctt
37441 cggcgatccg ggcgcggtcg atctcgtagc cgaacacgtt gtcggttttg agtccggcgc
37501 caacttcggc cagggtctcg gccggcaagg tcttgagcgc ctctttgtcg atctcctcct
37561 tcagcctgat acagccgtgc agccccaggt ctttcagcgc ctggagcgtg tcggccacgc
37621 gcttgatgag tacgcgagtg gagaggcgga agccgaccga gccgaacacg agctgccggg
37681 ttttaacctt ggcgaactct gcccggttgg cctcgcagaa ctctttcatc gcgagttcca
37741 gcgcggtttt gcggtcgaga agcggctgcg cctgggtttt ggcggcggcc ttgagcctgt
37801 cgatctgatc attgacgtct ccatcgatca ggctcagatc gcgctcgatc tcaccgatct
37861 cgcgcagcgc ctggtcgacg tcgtcccagc tcgagaattg ggtgccttcg attcggatgc
37921 gtgccatgat tcctcctaaa tgccaaggtt gatgtcgtcg gtgtcactct gccgcgcttg
37981 cgctttgcgc tcggcccgac cgaccaggtt gatgtcgagg acggcgccga gcagcaggcc
38041 ggtaccgagc ccaatgaggt agatgaacgc caagcaggcg aggacgatcg ccttcacggc
38101 gcagccccct gcagcccgcg ccgcacgtcg agctgccgct ggcgggctgc gtccacacag
38161 ggcgagcggc ggggcttctc ggccatttcc tcctcggccc gcctggccag ccggcggatg
38221 tcgaaccagg tgccgacggt cagcacctcg cgcgccttgc cgccaacggt gacgagcgct
38281 ttccagccgt aggctccgag ccggggatat cgttgaatct tgatcatgcg tgttctcctt
38341 gggttaaagc catctcccgc cgggagaggg ttgggtgagg gttgcaacgg tgcagcgacc
38401 cgaaaatggt tttcagggtg tgattgaaag cccggttgac tgcccgcagc tccctgtccc
38461 agacttccgg cgaataatcc ggctgccgcg gcttcgcggg cggacggggg gcgatgccgg
38521 ccgctcgccg cttcgcggtc cgggccgcat tgacacaggc tttgcaggtc gcctccaggc
38581 gaacgccctg ccgatagaac cggtccttgg gcagggtctg gccgcaacgc gtgcagcgtt
38641 tggttgaaat ctcagccatg cacctccctc ccggtttctg tgggagcggc tccgccgcga
38701 aaagccgctc cgcgcaggaa tgcccgagcc acttggaacg tccggtacca gtcggcacga
38761 tcgggtcgga acggcaaatg gaagacgcta tgacccgcga tttccgcagg ggcgatgtcg
38821 acccgaagcg ctcgggtttc gccgcgatcg agcgcggcat caaaccaggc cggtgtgggc
38881 gccaggatga ttcggtcacg aatgtgcatg gccgtctcct ttgtgcggac agctccggca
38941 ggcgcgccga tgggcgagca aggccgggtc gtggctcggc gtggcgccgg accacaccga
39001 tcggcattcc tccggcgcca tcggccgccg aagatacgga caggcgatct ggtcaaattg
39061 ctccagcaca cgcgccagca ctttggcgtc gtcgcccgga taacagcgcc gggctacggt
39121 actgatggtg gcccggttga ggccgagcag cttcgccacc gcggcgaagc tgccgtgcgc
39181 cgcgacggcc gcctccaaca gcttcagcgc tctttcccgc cgctcttcag tcatcgagct
39241 gctcctgcgg gtcctgctgc cagacgatct tgccgacatt ggggtcgtag acggtcttca
39301 ggcgggtgat catgggcggc ctggggccag tgatccgcga gtgcaacagg cggtaggtgg
39361 tcggcacgcc gccggcgccc cggcctttgc cggcacgctc caccgttaga aaaccggccc
39421 gctgcagata ggcgcagtag gttttaacgg tggcgagcgg cacgtcggcc atccgcgcca
39481 gcacccggca gttgaacgcg cccaaggctt ccatggcacc ccacatccgt tcgttgcctt
39541 gccccagggt cacgggctgg ccggccttgt tgacccgcgg ggcttcgatg ccgcggtcgt
39601 cgatcaattc atacaagcca ggcgtcttct ccaaaatccc ggcggcatgc aggctttcga
39661 cgtaggtgcg caccgtggcc agcgggaccg ggccgggcag atggccgcgc agttccggca
39721 cggtgaagat ccggagcttc cggatcattt cccagatccg ctggcgcgga ctcttgccgc
39781 cggccagctc gatcgtaacg ggcttgcgcg ccatcaaccc ctcctcggcg cttcgccggt
39841 gtgaatcggc cgattgcccc aggtggccag atcgatccgg tcccagccgt tggccagggc
39901 ttcttcgtac atccgctcca ggttggtgga aacgcgccgc accgacccct tggccacctc

39961 gaccagcctt tccagcaggt cctcgcccat caccttgccg gggcagtaca gcccggccaa
40021 ctcgcgggca tccggcagag acaccggctg cgccgggaat cgatccagga ttctgccgtc
40081 aaagcgttcc cactccttca gcttgaacgg cagcgcctct tcgccgatca gcaggatggc
40141 ggccttgctg ccttcgtaga tactgcggat ggagtccacc agcttgaacg tctccaggta
40201 gtcggcctcg tctatgatca gcggccgccc ggactgctcc agttgggcca tgactgcatc
40261 ggccatctca tcgaccgtgg cggatttcgc cagttgcatc cccagcgctt tacccaaacg
40321 cttgagcagc accttcctgg agcgcgtgaa atcatccagc tgcaggtaat aggcgcgaga
40381 cctggccttg acccaggcgg ccgccatgct tttgccgtaa ccggaaggtc cgtgcaaaac
40441 caccaaaccg gactggtgca tgctgcggcc catggccagg gccatggcgc gctccgcgat
40501 ggcgatattg gtcagggggg caatgccggc aggggtggat ttgtcgtttt cgggtttgtc
40561 tgtcatgctt ttctccgtgt ggttgatctc atcggcccgt cagcgcggca acgcttacgg
40621 gccgttcttt tagcggatcg cggcttgcgc cttgaaccgg gcggaaagcg gaaattcggc
40681 gtaaaacctc cgaacctgcg cgtcgtcagg cagctcgccg ccgccggctt tcacggcggc
40741 atccagcgag caccagaatt cgtatttctc ctcatcgctc atccgttcca acgggtgctc
40801 cttacgcttt ggcagcttga tgatctcggc cgaccggccc gaagcggcga aatgggcctt
40861 ggcttcggcg ctcagctttt ccgcttccgg cgagcggggc ggccgatccg tgctttccgc
40921 cgcccgcgcc gcgccttcca gggctggcgt ggtgtagccg gtggagagct ggggaaaggt
40981 ggccaatttg ccggcggatt ccgccttgga ccgcaggtag ctgttgatct ccgcgttcag
41041 atcgacgcct ttcttggccc ggttgagcgt cttcatgccc tgattgcgct gctgggccgc
41101 catgcgcttg cactccatgg cgtgccggct gactgcttcc ggcggcagcc ccagccgctc
41161 cgggcagatc gcgacgcaga cgaagccgtt gtcgtcgtag gcgtacaccc gacccagatc
41221 ggtttcatcg accatgacgc ggaaggcgtc tcccgtggtg tggtggcgcc acagctcggg
41281 gtgggaatag aacccgccat ggatttccag ccccttcttg cccaggctgc gggttttgcc
41341 gtccacgggc ggagacagca ggatgtccaa tgcccgctcg ttgtgaatgc gccggatttc
41401 cccccggtaa gacgcggcgc gctggaacgg tgtctcacct tccagacccg catgcggccg
41461 gtggtgatag cggttttcgg tccagtcgtc gcagaatttt tgcagctcct cgggcgatag
41521 ccgcacttcg agcacgccgt ccttttccat caggcggcgg gcgaagctct tccgcgcctc
41581 gatcgccttg cgctcggcga cgtcgtgacc gatgtagccc ggcagcagct cgaccaggtc
41641 gtgattgaaa gtgcccagcg cccgctcgat atggggcttg tattcggggg agaagggcgg
41701 gcaaagcaca tgctctaccc cgaaggcatc ccagatgccc gtcaagtaat cgctggcgta
41761 gtccgcgccg ttgtcggttt tggcctgttc gggcacgccc cagtccagca tcgcccgccg
41821 caccacggcg gcaatcgcgc gcgctttgga ggtgggcgcg accagcagct tgagacggcg
41881 tgagtaaacg tcgatcacgc cgatcacggc atggcgcttg ccgtcggaca gcagcagatc
41941 gcccagggtg gaatccagct cccagcgctg gttgagccgc accacgtcgc cgtccgccag
42001 acccaccgcc ggcatcgcct tgtttttcca ggcgtccggg ttgcgcagat gcagataggc
42061 gctggcgttc ttggccttcc agtttttgac gtaccggtcc accgcgctat aggagggcac
42121 cttgatcgtc gcgtccccgg cgaaacggtt ttccgccatc tcatgcaggc ggaccgcagc
42181 gatatcgggc tgctgcgcca gcagccccag catgtactgc tccagccgcg gctgctggtg
42241 gatcagactg tcgccgcggc gcgggctttt atggttgtcg agcagggcgc cgatgttgga
42301 tttcttgatc aagcccagct tccgtttcag cgtgcgcagg ctcaggccct tgccttgggc
42361 gtttttgccg cgcccggacg atttcaggac gggcgcgtag acctccccat cgagatcgag
42421 agccccggca ttgaaggctt taataaaggc gatcagcgcc ttttcctggc tgcgcttctg
42481 gtcgccaaat ttttcccgat agaagcgccg ccagcattga atcgtcgcca gccaggcgcg
42541 tccgcgctcg cctcgctcgc ccgtcgcctg ggcgaacagc gccagcagcg cttcatcgtc
42601 ctgctgccgg gcctcagctt ctcgggccga ggcttcggcc atctgccggg cgatggcttg
42661 gccggcgcgg tattcggggg agctctccag cgccgccagc tcggcggcga ggctggcctg
42721 gcgctgagct tccttttcgc gtgcctggat cagcttcatg acggcggctg agggttggta
42781 ttcgcggcgc atgccgtttt tgcctccctt ggtgctcgcc tcccggaagg cccaaccttc
42841 gcgctcaacc agatcgcgga acccgcgctc cgagccggga tatcccggca gcttgagcgc
42901 agccagctcg gcgcaggaat agtgggtttt gatagcgctc atgcccggcc catcccaaat
42961 tggcgagggt tccggcccgc tttaaactgg cgttgccaaa cgaccattac ggaagaaccc
43021 tcaatgaaaa attttgacct gttccgcgtt tttacagagc gtgcgctgga ttgctcccta
43081 gagtcgctgg agccgcaatt cataacgggg tggcaaggcg ttgaagcgct gtggccattg

43141 agtgaacgat ttcgcgaaag cctgacgcgc attcgttctc taccgtacga cgcctctttt
43201 gaacccgaag cggatgccgc tatcgagctt ctaatcgacg atccagacag tagctgggag
43261 cacatttcag cgggtgcttg gcgtgtattg ctggagcgcc aacaacaggc catcctggtg
43321 gctctagcca atgaagccgc tggcaactcc ttcataccga ttcctcccgg tctttctgaa
43381 tcgcagcgca cagtggccgt agtgttgttt ctgcttcacg gaatgaagct cccgtggcca
43441 ccagaagatc gatccgactt ttcaattcca acaggtggga ttccttcatc gctgcggcca
43501 cactgaaagc tcgcgcatga gtcacctcaa gaagcgacat catgtctaaa cctctgccag
43561 cgctttgcca ttgcgcacag cgctggcgcg gtgatgctgg atggcgcgaa agccttgcgg
43621 cttacgctcg ccactcatcg ccccaccccc acagaccgga gggcttccct ttctcgctgg
43681 cccgccaact ttttcaccat caccgcccga cctacttcaa aggcggcaat gtcgtcgccg
43741 tagatcacct tgccgccagc cgcttcggca tacagctcga gtagcgccca tttcccagtg
43801 acggcatcga atgccatggc ctgcagcaga ctgggggtgt ggtcgggccg gctcatggcg
43861 gtctgggcgt tcagcacgga cagcggcaaa cgcttgccgg tcaacgcggt gatccgcgag
43921 gccagatcgg gccgatcgac ccctgaggcg tcgatggcgc ggctcatggc gaccgcgacc
43981 cggtcaccca gagcgaaacg ccccggcgcg atggggatgg gttgcggcag gtggacggcg
44041 ccgccgccga acaggtcttg ctgtgctgcg ttcatgactg ctcctcttat gtcgtgatga
44101 ccccggcgta ttgccgtcag ccgttccgct aacgtttgcc ttgtggcccg ctaacgcgct
44161 ctgtatcgtt ctccctcaag cggcgtgctg tttctgagcg cggttcggat cgggtttcaa
44221 ccccatcgcc acggcgatgg aaaagctccg gccataacga gccttgatct gcccgttcag
44281 caccaaagaa acctcgcgcg gcctgaaccc gcgctccctg gcccaatccg ccatggtgat
44341 gccctggagc gcgagccaac tacgaaactg gtccggtgtg atagtgccgc cctgcgcttc
44401 gatgaaggcg cagaacgggt ttgcctgagc ccctgcggct tgatttgtat tcgacatggc
44461 gcgtttccgt ggttgtgttg tgtcagtgtt tgttctgctg tttgacatag cgtattgaat
44521 ctcggagctt ccgtcaacaa gatttttttg cttccgagct gctccaagat aggctgatag
44581 gtgaaaataa atgaaatacc ctaaaatcat aaacttggat aactcggaag tggctaaatg
44641 acttccgaga taaattccga gataggtgcg gggagctcgg aagagtggtt ttccgctgcc
44701 gacctcgcga ggatggtttt gccaggctgg cctaaatctg accgcggatg gcgtgacgtg
44761 ctggaaagac agcactggcc gttcagggaa gtgcccagca agggcggtaa gcgggggatg
44821 aagcgcgaat accagccgcc tccagaagtt cttgcgctca tcaggcaaaa gctgcaaggc
44881 gagccccagg ccgcacacgc ctatccgacc aagccaggcg gcctgaacgg cgataagcat
44941 ctgcagcggc agcctgcagt ctattcaccg gatgactacg ttgcgctgcc gctctatgac
45001 gtgcgtgctg cggccggagg tggcgtagtc ccagacacgg aaaacgtcgt cgatttcctg
45061 catttcaaga aagcatggtt gcgcaccgag ctgcgcagct cacccgatga tctttacttg
45121 atctatgtgg acggcgagag catggagccc acactgtgta aaggcgacgt gatcttggtg
45181 aatcatcggg ataagttcca ggggcgcgat ggcggcatct acgtgctgcg gctggacggt
45241 gcgctactgg tcaagcgctt acagcgcaag atgggcggaa tcatcaaggt aaccagcgat
45301 aaccccgtct acgagccgtt tgaggttggg gcgcaggact tggatagagc agacttttct
45361 atcatcggcc gggtggtatg ggccggtcgc cgaatgtagg gcataaaaaa accgctcgaa
45421 agcggtcttg tttgtgccaa atattgcgct aaatccgttc gtttccgctc agagcgcgcg
45481 aatttatgcc aaaacgaatt ttcgcgcgaa attcggtcaa ctcattgatt tgggctaaga
45541 agtccgccgt tgtccgggct agtccggagt gccaaatcaa gaactaactc acaacacctg
45601 tacca
//

```
LOCUS       KN2-R1                71407 bp    DNA     linear   PHG 16-FEB-2025
DEFINITION  KN2-R1.
ACCESSION   KN2-R1
VERSION     KN2-R1
KEYWORDS    .
SOURCE      .
  ORGANISM  .
            .
FEATURES             Location/Qualifiers
     CDS             3..515
                     /ID="DFLLIFOK_CDS_0001"
                     /transl_table=11
                     /phrog="66"
                     /top_hit="No_MMseqs_PHROG_hit"
                     /locus_tag="DFLLIFOK_CDS_0001"
                     /function="integration and excision"
                     /product="excisionase and transcriptional regulator"
                     /source="PHANOTATE_1.5.1"
                     /score="-21.34365859860116"
                     /phase="0"
                     /translation="WGGRLRQTAFILHNRSVAGQRERLRVQADSAGHRFSSCHPSVSLS
                     PGLGGGLGFSTGLVCYPVRFPSPQPNPRPMVGWPLASAANSAVMDSCQLVRKETKMEVV
                     HLNQKQLATRWGVSEATLERWRSEGIGPKFLKLCGRVLYRQIDIEAYEASCLATSTKCR
                     GARVGAE"
     CDS             complement(602..1099)
                     /ID="DFLLIFOK_CDS_0002"
                     /transl_table=11
                     /phrog="6988"
                     /top_hit="No_MMseqs_PHROG_hit"
                     /locus_tag="DFLLIFOK_CDS_0002"
                     /function="unknown function"
                     /product="hypothetical protein"
                     /source="PHANOTATE_1.5.1"
                     /score="-735.3505317947771"
                     /phase="0"
                     /translation="MGGGGGGSIPSSDLKKLEEKAKQTLTTPADTGCHVFISFAFEDED
                     AVNLLRGQAKNEKSELEFDDFSLKQAVNSENEDYIKQKIRERINRASVTAVYLTPDSAK
                     SKWVEWEITESLKRGKGVIGVYKGDAPPAQLPAAFETYGLKTVKWSHQELMNAIAEARK
                     NR"
     CDS             complement(1103..1642)
                     /ID="DFLLIFOK_CDS_0003"
                     /transl_table=11
                     /phrog="1550"
                     /top_hit="No_MMseqs_PHROG_hit"
                     /locus_tag="DFLLIFOK_CDS_0003"
                     /function="moron"
                     /function=" auxiliary metabolic gene and host takeover"
                     /product="QueC-like queuosine biosynthesis"
                     /source="PHANOTATE_1.5.1"
                     /score="-156.28233638482104"
                     /phase="0"
                     /translation="MAREEGVEVYPLFIDYGQRAARKEWDTCQRVHAQLGLPTPTRMDV
                     SGFGRVIHSGLTSTSLDVKEDAFTPGRNLMFLLMGSAYAYQVGAPTVSIGLLAERFSLF
```

PDQRSEFLAQAERAIEAALGRRIRVVTPLAEFSKADVIKLAQEKGISGTYSCHMGGGEP
CGHCISCLEFGLGKGG"
CDS complement(1693..2406)
/ID="DFLLIFOK_CDS_0004"
/transl_table=11
/phrog="11575"
/top_hit="No_MMseqs_PHROG_hit"
/locus_tag="DFLLIFOK_CDS_0004"
/function="unknown function"
/product="hypothetical protein"
/source="PHANOTATE_1.5.1"
/score="-1890.9836336842889"
/phase="0"
/translation="MNMTAVDRTIRVLCAEVRRNDRGVRDWNLRTEEELLHEACVCMFS
SQMVFEVAEAAAGQIRDRGLLRASRYKGSGAEYEQCLRAALSEPLAIERDGKVRHIRPR
FRNRLASLLATTLTTMRDQGQSLRKILFSAGSAREAREILVKQVWGFGPKQASLFLRRV
GYSAQLAVLDTHILDYLRLARGIDPKPSALSRLPSYEQIEAEFQRVAADFGHSVGCVDL
AMWVTMRVAKREAIW"
CDS complement(2403..3383)
/ID="DFLLIFOK_CDS_0005"
/transl_table=11
/phrog="No_PHROGs_HMM"
/top_hit="No_MMseqs_PHROG_hit"
/locus_tag="DFLLIFOK_CDS_0005"
/function="unknown function"
/product="hypothetical protein"
/source="PHANOTATE_1.5.1"
/score="-8924.283778083869"
/phase="0"
/translation="MITRLEEITLGGNRLAMPVFFPSVSTVKTALAPSEYLATLHSLRD
MNQQFLVSAFDLGRSHNSDGLEHWLTAAQDTGATVLMDSGNYESYWKDEQVSWRQADFH
DVLRRFPCTFAFGFDEQNPPADADAHAGLVVERWRQDQIAAGNRVIVPIVHGTPESLPE
LCARVAQETQVPMIAVAERRLGSGVFERTRSVMALRKALDDNGRYVGLHLLGTGNPISI
ALYAWAGADSFDGLEWCQTVVDHDTGLLFHLSQSDFFRRQTAWGEGDISFYARTLAHNL
TFYADWMQRLRQAIHQGDGLNFCRFNFPPYVFSQCAEAFGWEAAA"
CDS complement(3386..3883)
/ID="DFLLIFOK_CDS_0006"
/transl_table=11
/phrog="7395"
/top_hit="No_MMseqs_PHROG_hit"
/locus_tag="DFLLIFOK_CDS_0006"
/function="unknown function"
/product="hypothetical protein"
/source="PHANOTATE_1.5.1"
/score="-329.56136446611845"
/phase="0"
/translation="MIDSQGGRQENQGTMPEAVAEHPAWHRLEDQLQWYDTKSTQCQKW
YKRLKLVQISLAVLIPVLSHLDPMYAKWVTSVAGALIAVLEGVQHMNQYSTLWVTYRAT
AERLKHEKFLFLSAAGPYKGQPEPERLIALAERVEEHVSTEHANWFNETRRIVSAQKKE
AM"
CDS complement(3873..4016)
/ID="DFLLIFOK_CDS_0007"
/transl_table=11

/phrog="No_PHROGs_HMM"
/top_hit="No_MMseqs_PHROG_hit"
/locus_tag="DFLLIFOK_CDS_0007"
/function="unknown function"
/product="hypothetical protein"
/source="PHANOTATE_1.5.1"
/score="-0.2104193667828092"
/phase="0"
/translation="VNISPVSSFIASSPGTKIGAGNGGNLGKDTANLSIFEPVCRRRGD
DR"
CDS 4113..7706
/ID="DFLLIFOK_CDS_0008"
/transl_table=11
/phrog="23807"
/top_hit="No_MMseqs_PHROG_hit"
/locus_tag="DFLLIFOK_CDS_0008"
/function="unknown function"
/product="hypothetical protein"
/source="PHANOTATE_1.5.1"
/score="-129094873416531.22"
/phase="0"
/translation="MALFAPGNRPVDQATDERRHVRGRKQGQRIMHAEELKPNITVHGP
LFPEPVQVIVAIPMGASVKLVGKGTRSNSVYEPILTAEQLALLTASPAQEPFDGDARKF
RLGIEAMRLGLAYEYDPYFSLSIARVDPLPHQLEAVYDYFMRLPRIRFLLADDPGAGKT
IMAGLLIKELKIRGLVKRVLIITPANLTFQWQRELKDKFRENFEVIRSDVLRANYGMNP
WQEKDQVVTSVSWVSRIEDAKDSLLRSRWDLVIVDEAHKMSAYSSEKKTLAYQLGEALS
NMTDHYLLMTATPHKGDPENFCLFLELLDRDVYGDIRSLEEAMARHEAPFYLRRVKEAL
VTFPEPETGHVKALFTRRNVTTSEFQISDEELDFYDRLTRYVEDQSIKAAADDSARGRA
LAFTMAMLQRRFASSLYAVRRSLERMRDKRERILKDPEGYRREQMNKRLPEDFEDLTDE
EQQEIISQLEDVVASVDPAALREEIAELTKLINHARELEAREVETKLTALKDLLTKQGV
FADPKMKLLVFTEHKDTLDFLAGDGRETRPLGKLREWGLSLTQIHGGMKIGDRDTPGTR
IFAEREFRESAQVLVATEAAGEGINLQFCWLMINYDIPWNPVRLEQRMGRIHRYGQEKD
CLIFNFVSTNTREGRVLHKLFERIEAIQDDLDPKRTGKIFNVLGDVFPANQLEKMLRDM
YSHNQMTEELIKQRIVEQVDLRRFEGITQSTLEGLAKKELNLSAIVGKSAEAKERRLVP
EVIEDFFLSAGPLTGVSPKPVKAGTHAYRLGRVPRNLWPTGERLEPRFGKLGREYGNIV
FDKELLKQDATLEWVTPGHPLFEVVREDVLGQVQEHLKRGAVFFDLHSEKPYRLDVFSA
SIRDGRGNVLSRRLFVVQTGMDGKTAIKQPTIFLDLTPAETPPAVPDDAHLPDRGQVEI
VLLEQALQPLLEESRRERERREVATISRHIELSLNTIINRENLLLADLVSQKESNSTEPG
LEGRIKISEDKLLELDHRLQSRRRELEQERQCSIGDIQHIGRAWVLPHPERAAPGIAPM
VSDPEIERIAVQAVIAHEEAEGRVVESVEADNRGFDLISRRPHPEDPKTAIDVRFIEVK
GRAHTGEIALTSNEYKTAQRLANDYWLYVVFHCATKNPSVNILRNPATLDWQPIVKVEH
YRLKADSIRQPVELHEDQAAYRTEKGDEPTC"
CDS 7700..9010
/ID="DFLLIFOK_CDS_0009"
/transl_table=11
/phrog="4747"
/top_hit="No_MMseqs_PHROG_hit"
/locus_tag="DFLLIFOK_CDS_0009"
/function="unknown function"
/product="hypothetical protein"
/source="PHANOTATE_1.5.1"
/score="-809652.581973699"
/phase="0"

/translation="MLTRIHIQGYKSLKNVEVRLSELSVLFGPNAAGKSNFLDCLQLLS
KLATSRTIKEAFEPPYRGKPLESFTFGEQGIKGALEKERLSFSIEVDVRLSNAIVEAVN
RQIKEMRRSSVQGNEAGEEAKTAGAVRERNLRYSIEIEMLPKSGILRVADEYLAALTDK
GEPTARRKPFLSREGNRLHLRLEGQAHPSYYERYLDHSILSLPHYPPHYPHLVAMRKEL
ENWLFFYFEPRERMRAANPVKEVRHIGLMGEELASFLNTLKALDERQFRAVEKALHTIM
PNVQGIGVEVSNLGEVELTLKENGIPVPARILSEGTLRILGLLSLAGAKEQPSLIGFEE
PENGIHPRRIQLVAELLKTRASSGETQYIVTTHSPLLPDLVSDESLFVCKRVDGETRIE
PFAAWGALAKRGEVDRALMGAEDLTVSERMLRGDFDA"
CDS 9003..9590
/ID="DFLLIFOK_CDS_0010"
/transl_table=11
/phrog="No_PHROGs_HMM"
/top_hit="No_MMseqs_PHROG_hit"
/locus_tag="DFLLIFOK_CDS_0010"
/function="unknown function"
/product="hypothetical protein"
/source="PHANOTATE_1.5.1"
/score="-149.52627217436483"
/phase="0"
/translation="MREIALFVEDFAHKEFLAALLQRLAAEYGVTVHLDWRNARRGHGA
VVTELKQFLRDLQRGRDALPDLVVVATDANCKGLVERMRQITEVTGKVDVPAVCAVPDP
HIERWLLVDSAAFKTVFGRGCNAPDQKCERMRYKKMLIDAIRASGVTPSLGGIEFADDI
VAAMDLTRAAAADASLSRLIEDLRAVFKVWQP"
CDS 9587..12565
/ID="DFLLIFOK_CDS_0011"
/transl_table=11
/phrog="10183"
/top_hit="No_MMseqs_PHROG_hit"
/locus_tag="DFLLIFOK_CDS_0011"
/function="other"
/product="DNA methyltransferase"
/source="PHANOTATE_1.5.1"
/score="-90915105016.41254"
/phase="0"
/translation="MTYPKRLIEVDLPIKRISAHSRREKSIRHGHISTLHIWWARRPLA
ACRAVICAALWPDPADEHCPPAFRGAAKAAMLDWARNHLGLASAESFPRFVAIQKDAAR
LDDPIELRHALLDFIADFANWDNSTVKEYLDTSRALTQAAHEALGGAPGTRPLVVDPFA
GGGSIPLEALRVGADAFASDLNPIPVLLNKVVLEYIPKYGQRLADEVRKWGAWIKQEAE
KELAAFYPKDPDGATPIAYLWARTIRCEGPGCGAEVPLIRSPQLSTKGKKSVNLVVRGD
SVTKKVIVEVSSAAAAKPEPTVRRGSVTCPVCGYTTPAGNARQQFKTRAGGTHDARLLS
VITVKANEPGKSYRAPTDGDRAAFLEAMNRWDQIKSDVGAHGLTTIPSEPLPYLRSIFN
INLLGVNQWGQLHTPRQAVALASFAEKIYQAWDRLRELPDGLGGAIHLCLGLALDRSAD
RMTSLCRWISQTEAVGYTFGRQALGIVWDYIEINPLTTDAAWLGAIENICEVIEAVDRA
LLRNGSVQECSATAHPLPDDSTACFVTDPPYYDAVPYADLSDFFYVWLKRTVKQSHSRF
AHELLTPKEDEVVQLSERNPKYAYKTRERFEALMTDAMREGRRVLTPSGIGVVVFAHKT
TAGWETQLKAMIDAGWVITGSWPIDTEKPGRLRALDAAALASSIHLVCRPREHPDGSLR
NTDIGDWRDVLAELPRRIHEWMPRLAEEGVVGADAIFACLGPALEIFSRYSRVEKASGE
VVSLREYLEQVWAAVAKEALAQVFKDADTAGFEPDARLTAMWLWTLNAGAINGDESPAD
EEEADDEEETKGKAARAKGGFVLEYDAARKIAQGLGAHLEDMGHVVEVSGETARLLPVS
ERTSYLFGKEQADAPTGGRKKKAAQIDLFGELTKVGADETAWSEKTVKKMGETTLDRVH
QAMILFAAGRGEALKRFLVEDGAGRDQRFWRLAQALSALYPASTSEKRWVDGVLARKKG
LGL"
CDS 12567..13151

/ID="DFLLIFOK_CDS_0012"
/transl_table=11
/phrog="No_PHROGs_HMM"
/top_hit="No_MMseqs_PHROG_hit"
/locus_tag="DFLLIFOK_CDS_0012"
/function="unknown function"
/product="hypothetical protein"
/source="PHANOTATE_1.5.1"
/score="-255.4069571318534"
/phase="0"
/translation="MVDGLVARFRETFFAIVQADELAGDLKQAASAANLGAWTRALTEA
AIRTCSGLGLMASAKGHKLELLPIHRSEYLALDVMAFAEGEKRWRFPVAVMELENSARE
DQIAYSLWKVLSVRAELRIVFCYRRNGEEIPALLRHLREEVVEAMGLAGRVKLEGATLL
VVGSRSESETFPFGYFAWWSLDTNTGRFERL"
CDS 13161..15980
/ID="DFLLIFOK_CDS_0013"
/transl_table=11
/phrog="38717"
/top_hit="No_MMseqs_PHROG_hit"
/locus_tag="DFLLIFOK_CDS_0013"
/function="unknown function"
/product="hypothetical protein"
/source="PHANOTATE_1.5.1"
/score="-418733533125.9161"
/phase="0"
/translation="MKLKPWYDVVKPREDLREGKPLDASEFAVHLDKVRLGTAPKDYRD
AATFFDRTFLTENLTGLGAEVMRRLSGITTETSAVFNMTTQFGGGKTHALTLLYHLARH
GSAGNGWRGVTKILQRAGIQTIPDHTAVAVFVGTEFDSVTGRGGDDGTPRRRTPWGELA
WQLGGEESFAHVAQHDADFIEPKGDVIEKFLPSDRPCLILMDEVLNYISTYRDRGWHNK
LYNFIQALSETVRGRHNAVLVGSIPASELSYTDKDEADQQRLKNLLDRLGKAVIVSVES
ETSEIIRRRLFEWDERAVTPDGRVILNKDAEDACRTYADWIQENRLQLPNLINPDLARD
EFRATYPFHPMVISVFERKWQTLPRFQQTRGVLRLLALWVSRAYQEGYKGAQRDPLITL
GTAPLDDPMFRAAVFEQLGETKLEAAVTTDIAGKKDAHAVRLDSEAIDAIKKARLHRKV
ATTIFFESNGGQVGAEAQEASVPEIRLAVGQPESDIGNIETVLEALTDACYYLNVEKTR
YKFSLKENLNKRFADRRATVQGPQIDEEVKREIQKIFAPKEFVERVFFPEKSIQISDRP
VISFLVADLDRTMEDEKATRQFAEQMIRECGTTARTFKSALIWVVPDSAQPMREEARKI
LAWQAIQDEADDLKLDEAQKKQLAENIHKAKRDLKESIWRAYKHLFLLAKDNTVKMVDL
GLVHSSAADTPISNILNRLSADGDVEKGVSPNFLVRNWPPAFKEWATKSVRDAFYASPV
FPRLLNAETVKETIARGVESGILAYVGKGTSGKYQPFAFNQSVSPADIEFSDDMFVITA
EVARAYLDAQTTPPPVGVPVEGGGTGVGTGTGGGAEPGGTPIEPQPAPTPPVAGPAKIA
GIRWSGEVPPQKWMNFYTKVLSRFAATPGLKLTISVEVSPSDGLSKQSLEETQNALREL
GLDDRVTEK"
CDS 16085..17869
/ID="DFLLIFOK_CDS_0014"
/transl_table=11
/phrog="6203"
/top_hit="No_MMseqs_PHROG_hit"
/locus_tag="DFLLIFOK_CDS_0014"
/function="unknown function"
/product="hypothetical protein"
/source="PHANOTATE_1.5.1"
/score="-496223260.3502325"
/phase="0"

/translation="MSRAHLRFQVDSRLATLLSQEYSSSERALKELVDNAWDADAQQVT
IVLPKPMSGDPIVIQDDGTGMTEEELRRHYLSIATDRRSRRGERTVGKNRLVKGRKGIG
KFAGLMAAAVMTVETRARGRLCRITLRLDDLSQVEDIEQLDIGLHSEPCGPELHGTTIT
LSDLHQGLAYPDANRLRQILLQDYGRQDDFSITVDGKRLDVDDVSGSYSEQEQDLPNAG
KVKLRFAISDGKSGLRQPGITLRVDGKSVGRPGFFGLDQRDDFPSKLLRKLYGEVEADG
LRDHITAGWDSAVENSELLKEVEGYVQPILREAYEQQYRREIQLAQARLQRAILARLSA
LPEHKRMFADRAIKKILDKYYGEPESKVEPVVNVLLEAMESSDYRILLEHIAEATPGDV
AGIAERLDEFGLAEMAFLVQQAAARQVFLDQLDALARDAATTEAVLHKALERNLWVFGA
EYSLFSSNSTLRRQVEDILGKTYTGDKADKRPDLLLNENLSGQYLLIEFKRPNHALNHG
DYVQAIGYRHELSKYLSSPIQVLLVGGRSSDFPVENREPQVDARIFGQVISTARRQIEW
LLRTSNGESDLRIGSGVA"

CDS 17884..18609
/ID="DFLLIFOK_CDS_0015"
/transl_table=11
/phrog="No_PHROGs_HMM"
/top_hit="No_MMseqs_PHROG_hit"
/locus_tag="DFLLIFOK_CDS_0015"
/function="unknown function"
/product="hypothetical protein"
/source="PHANOTATE_1.5.1"
/score="-1849.3529947270767"
/phase="0"
/translation="MNWVVIFNRLFELIDTDGTPDYFSGPRFLRKVREVDPYYPTYQQF
IDQRRSQGASTSRRDFFFDILQALPEPARISLVNSILGELETSASDRAGEIRALLGGAV
PGPVAVVPGHGWNADRLNQYLRDIDQSITAGEFPRAVTLAYTCLEGFYRAFVTQCIPEQ
AKETEIIALSKIIKDYLRTTIVAYPDEALNMVTHVSHTVDRARNRFSEAHFEQEAARWL
SVYVRDLVNTQIRLLLHFF"

CDS complement(18606..19139)
/ID="DFLLIFOK_CDS_0016"
/transl_table=11
/phrog="7"
/top_hit="No_MMseqs_PHROG_hit"
/locus_tag="DFLLIFOK_CDS_0016"
/function="lysis"
/product="endolysin"
/source="PHANOTATE_1.5.1"
/score="-29.80698480984644"
/phase="0"
/translation="MGRRAELLVPRPRVRPGAPVIEVPTAAVDLAKRFEGFHRVPKHDP
QRRAHPYICPAGYWTIGYGHLCKPDHPPITEDEGEIYLAQDLRTALAATLRYCPVLATE
PEERLAAIVDFTFNLGAGRLQTSTLRRRVNQRDWPSAAKELRRWVYGGGKVLPGLVSRR
EAEASWLLRSALMA"

CDS complement(19079..19555)
/ID="DFLLIFOK_CDS_0017"
/transl_table=11
/phrog="6637"
/top_hit="No_MMseqs_PHROG_hit"
/locus_tag="DFLLIFOK_CDS_0017"
/function="unknown function"
/product="hypothetical protein"
/source="PHANOTATE_1.5.1"
/score="-20.525436686344907"
/phase="0"

/translation="MIETLLGGLLGGAFRLAPEILKWLDRKGERGHELAMQDKALEFEK
LRGAQRMAEIGAAGEAAWNSGAVEALREAVAAQGQRSGVRWADALSTSVRPVITYWFMA
LYCAAKTAAFLGAVDAGVEWIPAIQAAWTDADQALWAGVLNFWFLGRVFDRVRP"
CDS complement(19552..19842)
/ID="DFLLIFOK_CDS_0018"
/transl_table=11
/phrog="10540"
/top_hit="No_MMseqs_PHROG_hit"
/locus_tag="DFLLIFOK_CDS_0018"
/function="unknown function"
/product="hypothetical protein"
/source="PHANOTATE_1.5.1"
/score="-4.3598940981478975"
/phase="0"
/translation="MSPPTLQDGMVVMPRDEFEELLARAAERGARRALADVGLDGEDAA
HDIRELRGLLEAFNAAKHTAWQTVIRLVTTGFLLALVAGAVIKLKVFGGGQ"
CDS complement(19909..20259)
/ID="DFLLIFOK_CDS_0019"
/transl_table=11
/phrog="5363"
/top_hit="No_MMseqs_PHROG_hit"
/locus_tag="DFLLIFOK_CDS_0019"
/function="unknown function"
/product="hypothetical protein"
/source="PHANOTATE_1.5.1"
/score="-10.379398887265715"
/phase="0"
/translation="MALNDPNLGLAYGWAQGEHNWNGGMDTNLKRLGAVVGLSVKDRDL
ATPPATPVDGDRYIVPAAATGAWAGRTDQIAVRIAGAWEYHIPKIGWTCFIEDEGVLSV
YKATGWSPGIAV"
CDS complement(20259..22460)
/ID="DFLLIFOK_CDS_0020"
/transl_table=11
/phrog="1101"
/top_hit="No_MMseqs_PHROG_hit"
/locus_tag="DFLLIFOK_CDS_0020"
/function="tail"
/product="tail protein"
/source="PHANOTATE_1.5.1"
/score="-3504319.2208817634"
/phase="0"
/translation="MSGGGKGSQEYTVGYWYGLGAHLALCHGPLDAITEIRVGERVAWS
GNVTGNTTITIDNPNLFGGEEREGGVQGPVDILMGGPTQGRNAYLQERLGTDIPAFRGV
VSLILRRVWVAAMNPYIKPWSVRAKRVPRQWYAAKAEISGDANPAHIVRECLTNGEWGM
GYPTSDIDDASFTAAADALHAEGFGLSLLWNKEETIEDFILSVLRHVDGLLYVHPRTGL
FTLKLARADYTLSSLPIFDPGNILRIEEFTRPSWGEITNQVTVVYRDGTTDKDGSVTVQ
DIAAVQLNGGVVATTVNYPGISRAELANRVAMRELKQLVSPLAKCTFVANRQASGLNIG
DVVKLSWPPYGIDQMVMRVARIAYGELANGAVRVECVQDIFGLPQSVYSAPPPSGWTEP
TSLPAPCPHQTLFEVPYWSVVKDFTGESQSLLGDIDDLDGLVAACGSRPSSDAFGFKAL
ARVSGSFTDKGFGIFTPTALLTATLPQSAVQLSVGLTSGIGLEEATAGGLTVIDGEWLK
VVSLDLATQTVTLERGMLDTVPASHPAGSRIWFVDGFRHYLTPEYVAGETVRVKLLTRT
ARGTLPEAAATEMSLPLNKRFIRPYCPGNAQINGKRYPTVVAGEINVSWATRNRQSQTA
YLVLQTEGAITPEAGQTTTVRFYNENGSLRRTVSGITGNGTTWPLAQELADSGLGRVNA

HVKVEIEASRDGHVSWQRHVIEFDRTGYGLRYGDYYGGA"
CDS complement(22457..22597)
/ID="DFLLIFOK_CDS_0021"
/transl_table=11
/phrog="1196"
/top_hit="No_MMseqs_PHROG_hit"
/locus_tag="DFLLIFOK_CDS_0021"
/function="tail"
/product="tail assembly chaperone"
/source="PHANOTATE_1.5.1"
/score="-2.4572296531343913"
/phase="0"
/translation="MRRWFEGRSHTWAEFVERGVPADWLRATGDAMAIRVAEEAEKEQP
R"
CDS complement(22651..22890)
/ID="DFLLIFOK_CDS_0022"
/transl_table=11
/phrog="1180"
/top_hit="No_MMseqs_PHROG_hit"
/locus_tag="DFLLIFOK_CDS_0022"
/function="tail"
/product="tail assembly chaperone"
/source="PHANOTATE_1.5.1"
/score="-5.071733877019272"
/phase="0"
/translation="MWQQIIIWVVTTVLSALLAPRPKVQDAQPGQIGDKDVPIASQDAP
IPVLFGTRVISGPNVVWYGDVQVRPIRKSSGGKK"
CDS complement(22900..23697)
/ID="DFLLIFOK_CDS_0023"
/transl_table=11
/phrog="778"
/top_hit="No_MMseqs_PHROG_hit"
/locus_tag="DFLLIFOK_CDS_0023"
/function="tail"
/product="tail assembly protein"
/source="PHANOTATE_1.5.1"
/score="-148.11612731697284"
/phase="0"
/translation="MSYLGIEQSAHDGQPQELYRFSQGAQRWLYTSGQAAVDYQSETYQ
PATISRGGLEQSNELARLGLEIRMPRDLAVASLFLAAPPEGVVSVTLYRRHVGDAEFIT
YWKGRITGARLSGAEANLKCEPIASSLKRPGLRARYQLLCRHVLYSSGCGALKDSFRVD
GAVAAVSGVTVQVAVAASRPDGYFVGGMLATTAGARMIVGHAGIDLTLVAPMVGLTAGD
AVQLYAGCDHTMAHCKDRFGNLDNFGGFPFIPVKNPFTGDAIV"
CDS complement(23707..25398)
/ID="DFLLIFOK_CDS_0024"
/transl_table=11
/phrog="1082"
/top_hit="No_MMseqs_PHROG_hit"
/locus_tag="DFLLIFOK_CDS_0024"
/function="tail"
/product="tail assembly protein"
/source="PHANOTATE_1.5.1"
/score="-8939.427738705548"

/phase="0"
/translation="MPTFPGSILPGAARQEPPTWSRSPEVSERAPVVFPFDPSASSIAR
PGARLDTQPVDELRLGFTGAPLPAFGADWYHRIHVIPSTIDLGNLVSPVERVLEVWNAR
FDAQTLDAIDETGMDGLLLSGQPAPPLAFGPLQSRLYTFAAGTRGAPVIDAAYRFVFAG
GLTALLVVTGRRVVVFGMRPDWSQGITERLEWLTEVLESYDGTEQRVRLRQLPRRGFEY
GFLIEGRDTQVLDHLLFAWGARIYCLPVWTDVSTLAGEAAVGSAALTVQDAANSDYHAG
GLAVLWRSNTRHEAVEILSIAGNTLTLKLPLAGSWPAGTRVFPARLARLEGEVAVARPT
DTIAVGRCRFGVEDITAPAVADAGPAYQGYRVFDWRPNRSTDLEDRWRRKLSIIDYGTG
VPTFDDESGAPLIGRTLTWLLTDRSAATAFRGWLAARAGRANPCWLPTFESDLEVSRTV
AATDAGLIVRNVGYARFVAADPLRRDLRLLTTAGTFHRRITGANEISEDEELLSLDAPL
GVTLEPQQFLQVSYLELARLDQDAVELHWETDTTARVQLSTRTLRS"
CDS complement(25406..26398)
/ID="DFLLIFOK_CDS_0025"
/transl_table=11
/phrog="687"
/top_hit="No_MMseqs_PHROG_hit"
/locus_tag="DFLLIFOK_CDS_0025"
/function="head and packaging"
/product="structural protein"
/source="PHANOTATE_1.5.1"
/score="-434.03340640286586"
/phase="0"
/translation="MAYQTGTSANPDQLLDALRVFAVANGWTQLRWVPDGTGQTLSLAK
GGLYVHLRSAANERLSTRYNTVTGMWLIGSTGFDAGKPWWDQPGSIVNASYVSNTTSYR
AEACGLFEVGTANTYHLLSAATPELIMLVAEVSPGVYHHLAFGDLTKFGSYGGGAFVSG
AFGPDAYTYTYSGFTDYVFGYDHDRHFGLPFNDYKYYGGANFVLAEVDGATDWHSVCKD
WALTGKRAKALWERGTGTPRDSLARYWWGHVPNTLNGVTPMLPFYLFVARPSGFFSPFG
HTAHLRYLNITHYAPAESFALGAEQWMAFPAHSKNGKSGVHGYAVRLVP"
CDS complement(26405..27337)
/ID="DFLLIFOK_CDS_0026"
/transl_table=11
/phrog="687"
/top_hit="No_MMseqs_PHROG_hit"
/locus_tag="DFLLIFOK_CDS_0026"
/function="head and packaging"
/product="structural protein"
/source="PHANOTATE_1.5.1"
/score="-662.9091030340305"
/phase="0"
/translation="MAFEIGTASDYRDLLDRFHAFLTAHPNLVAAGQQWQALHWTTDAA
TKELILKAPGLAGAEEIYCGIRAYENATAGYYMWDLNGFIGFNPANDFYTQPGAISGWL
PMMSLWNTAIPYWFVANGRRAVVVAKISTVYQAAHLGFVLPYATPGQYPYPLLVGGSMT
GQRGRNYSVTSPNHRHFVDPGEDGQNNANTACMLRGPSGAWLPFQNLAYSSSEYRYDGP
RPVWPTNYTYLGNLREAPDGTYVLSPVVLTQYNSGTDHDLFGELEGVYHVSGFNNAAEN
LITVGGVDHLVVQNVYRTSVRDYWALRLE"
CDS complement(27338..29842)
/ID="DFLLIFOK_CDS_0027"
/transl_table=11
/phrog="13338"
/top_hit="No_MMseqs_PHROG_hit"
/locus_tag="DFLLIFOK_CDS_0027"
/function="unknown function"
/product="hypothetical protein"

/source="PHANOTATE_1.5.1"
/score="-2436202.8220236865"
/phase="0"
/translation="MANNRAQLLITAVDQTRGAFDSIKRNLGGLGDAARSLNGLLANLG
VAVSAAGLGAMVKSSLESADALSKLSQRVGITVESLSTLIPAADLSGVSAEKFEGGLRK
LATRMLEAATGTEDAARSFAAIGVAFQNQDGTLRATDQVLLDLADRFKALPDGAEKTAL
AVEIFGKSGADLIPFLNQGREGIGALTAELQSLGVQIGGDTAAQAEVFNDALAQVRLAV
TSIGNRIIETFLPAMNEMALGMVESAKEGGTLRAVLDGILLALKTVALGAATVGKAFVA
LGEAIGGGMAAAVEALSGNVAGAKAIIAELKGSLVQRLDELAQFRDSLFEPKPITVRAP
AIKADPALVERLKAPGKPAADTGAARLGLAKSQADAELKLLKDALDAQGRALERALEDR
LISIRDYYAAKTALETREIDAEIARLQGLLAEQRRLSTTGRDEGTRLRARAEVAKIEAD
LIVLNNRRADVEVANARKALEAERGLREELARVREELLDLTGAATAEDRRAAIARQYQG
LIERLRAEGDSEGVSTVGRLIDVKAAAADLSAYERQFNEALARMRAAEESINLQRQSGL
LTESQARQQILALHRETGAALEGLLPQLEAAATAIGPEAAARVQAWKNEIAQVKLVVDD
VAVAIDGAVQDGFAQMFEAIGSGAKSAKDAFADFARSVLAAINRIASQKLAEALFGSLF
GGGGAGGGSLGSLVSSLFQGFAGGGYVTGPGTSTSDSIPARLSAGEYVLNAAAVKRVGV
AFLEAINGIEGGPRIQGPRLAFAAGGLVPETPPPQPQGQGVRIVNVIDPAMAADYLNSS
AGEKTILNILQRNAGAVRQVLA"
CDS complement(29842..30381)
/ID="DFLLIFOK_CDS_0028"
/transl_table=11
/phrog="6367"
/top_hit="No_MMseqs_PHROG_hit"
/locus_tag="DFLLIFOK_CDS_0028"
/function="unknown function"
/product="hypothetical protein"
/source="PHANOTATE_1.5.1"
/score="-72.28613920547309"
/phase="0"
/translation="MQSGGREVRDAARAQMRTAFQVKRASFVSSMRAKVLDKKPGRLPA
LLVGSKIPWLGVHERGGTVTGNLLIPLLPGRIGPKRFRQVVDALMRSGNAYFVEKDGKV
ILMAENIRENAAPLARFKRAERARTGAKQIKRGQEIPIAVLVKSVALKRRFDLAGSVQR
TLPRLAGAIQNELAKT"
CDS complement(30473..30610)
/ID="DFLLIFOK_CDS_0029"
/transl_table=11
/phrog="13355"
/top_hit="No_MMseqs_PHROG_hit"
/locus_tag="DFLLIFOK_CDS_0029"
/function="unknown function"
/product="hypothetical protein"
/source="PHANOTATE_1.5.1"
/score="-2.3031163978806157"
/phase="0"
/translation="METFLVAEARDERERLNALLVVTAVGSQGDRRAIERLQRELGHAD
"
CDS complement(30628..31044)
/ID="DFLLIFOK_CDS_0030"
/transl_table=11
/phrog="1442"
/top_hit="No_MMseqs_PHROG_hit"
/locus_tag="DFLLIFOK_CDS_0030"
/function="tail"

/product="tail assembly chaperone"
/source="PHANOTATE_1.5.1"
/score="-6.186592447642549"
/phase="0"
/translation="MSAFDAFPPSPVMLDVGGESLEITPIRVGEVPALIKAVRPFAGQL
SAESDWLVLLAEHGESLLCALAVAARRERAWVERLSLDDAVRLAAALFEVNADFFVQRL
APAVQHAAARVNGRLSGATPSTASSAPGTASPTS"
CDS complement(31053..31805)
/ID="DFLLIFOK_CDS_0031"
/transl_table=11
/phrog="807"
/top_hit="No_MMseqs_PHROG_hit"
/locus_tag="DFLLIFOK_CDS_0031"
/function="tail"
/product="major tail protein with Ig-like domain"
/source="PHANOTATE_1.5.1"
/score="-267.911258698146"
/phase="0"
/translation="MPYFSGQGRVYIGARDALGNPQGLAYVGNVPELKVSLSVETLEHQ
ESVSGQRLTDLQLIKTKKGEFACTLEELIATNLALALYGATTAQTPGTVTAEALPNPVT
PGSLYLLAKQDVSSVVVKDSSATPKTLPAAQYSVNAKHGSLVILDATTGGPYVEPFKVD
YAYGTASVTAMFTQPLPERWVRFEGLNTADGNREVVIDLYRVAINPAKELSVITDELLK
FELSGQVLADTLKPAAGDLGQFGRIVLL"
CDS complement(31808..31975)
/ID="DFLLIFOK_CDS_0032"
/transl_table=11
/phrog="3357"
/top_hit="No_MMseqs_PHROG_hit"
/locus_tag="DFLLIFOK_CDS_0032"
/function="unknown function"
/product="hypothetical protein"
/source="PHANOTATE_1.5.1"
/score="-1.6189832999226443"
/phase="0"
/translation="MHIELIEPHTHAGRLHAPGEILDLDEAAAQWLIERGAARPADPQP
QTQIKTRKGD"
CDS complement(31981..32409)
/ID="DFLLIFOK_CDS_0033"
/transl_table=11
/phrog="4204"
/top_hit="No_MMseqs_PHROG_hit"
/locus_tag="DFLLIFOK_CDS_0033"
/function="connector"
/product="tail terminator"
/source="PHANOTATE_1.5.1"
/score="-7.485015202049031"
/phase="0"
/translation="MTPSVRERLIRAVVARIGSAIAPTPLHRQPTVPLPREASPALLLF
IEGDQVLAQANDRLDRALTLRLVALAREGDAFDVADALIVAAHGALMAEPSLGGLALGV
REIDCEWDTEDADSQALAVPARYEIRYRTLASDLTQKG"
CDS complement(32406..32687)
/ID="DFLLIFOK_CDS_0034"
/transl_table=11

/phrog="10473"
/top_hit="No_MMseqs_PHROG_hit"
/locus_tag="DFLLIFOK_CDS_0034"
/function="unknown function"
/product="hypothetical protein"
/source="PHANOTATE_1.5.1"
/score="-5.72218552851791"
/phase="0"
/translation="MAQVTDLYEAAGRAGLLTDVMVGSLTVQCVFSAPDELALDGLALN
RDYHLEYPSAWLTLAAGDTVEIAGSPYRVREVRQLRDGSEMQAKLTRL"
CDS complement(32687..33691)
/ID="DFLLIFOK_CDS_0035"
/transl_table=11
/phrog="29"
/top_hit="No_MMseqs_PHROG_hit"
/locus_tag="DFLLIFOK_CDS_0035"
/function="head and packaging"
/product="major head protein"
/source="PHANOTATE_1.5.1"
/score="-1162.881888310962"
/phase="0"
/translation="MQNPFSNPAFSMASLTAAINLIPNRYGRLETLDLFPIKPVRTRQV
VVEEMHGVLNLLPTLPPGSPGTVGKRGKRAMRAFIVPHIPHDDVVLPEEVQGIRSFGQE
TETESVAGVLARHLETMRNKHAITLEHLRMGALKGEILDADATPLVNLYTEFGITPKTV
NFALGNANTNVKGKCAEVLRHIEDNLSGEFSTGVHCLCSPEFFDALTGHAKVEEAYKNW
QQGAVLINDMRRGFTFGGITFEEYRGQASDASGTTRRFIAAGEAHAFPLGTVDTFATYV
APADFNETVNTLGLPLYAKQEPRKFDRGTDLHTQSNPLPMCHRPGVLVKLTAA"
CDS complement(33694..34068)
/ID="DFLLIFOK_CDS_0036"
/transl_table=11
/phrog="49"
/top_hit="No_MMseqs_PHROG_hit"
/locus_tag="DFLLIFOK_CDS_0036"
/function="head and packaging"
/product="head decoration"
/source="PHANOTATE_1.5.1"
/score="-12.562088666036567"
/phase="0"
/translation="MPTLVEPMNLGDLLKYEAPNLYSRDLATVAAGQNLVLGSVVGRET
ATNKLKALDPAATDGTELPAGVLIVDADATAADLDTVIVARHAIVARHALVWPAGITPA
QQSAAIAALEARGILVREGA"
CDS complement(34072..35415)
/ID="DFLLIFOK_CDS_0037"
/transl_table=11
/phrog="53"
/top_hit="No_MMseqs_PHROG_hit"
/locus_tag="DFLLIFOK_CDS_0037"
/function="head and packaging"
/product="head maturation protease"
/source="PHANOTATE_1.5.1"
/score="-490.4841258400449"
/phase="0"
/translation="MRSASSSIPTRATTATHPRRPPTRCRRKTSRTSDMQLVHLASRLY

GTPLLIARSKLDVILSVLGPRIGLPDVEAAVPAVMPASPEVAAPPGITVIPIHGTLVRR
TLGLEAASGLMSYGEIGARLDAAIADPAVSGILLDVDSPGGEAGGVFELAERIRAADAV
KPVWAIAADSAFSAAYAIACAASHLAVTRTGGVGSVGVIAMHVDQSVRDAQQGYRYTAI
TAGRHKNDFSPHEPLDQEAAERLQAEVDRLYGLFVGHVAAMRGLDPDAVRATEAGLFFG
EQAIGSRLADAVASRDQLLAVFATFLNPQGRSRNPAPRTRPGSVYAKQENDPMQSPDPT
PEVPETALIAAAASAAPGPETPATTVTDRREAVAIAELCQLGGCPQRTAEFLAAGLSEA
DVRRALLAARAHGPEIGSAIHPDAHAGHRASPEHNPLIKAVKHLAGKE"
CDS complement(35315..37327)
/ID="DFLLIFOK_CDS_0038"
/transl_table=11
/phrog="21"
/top_hit="No_MMseqs_PHROG_hit"
/locus_tag="DFLLIFOK_CDS_0038"
/function="head and packaging"
/product="portal protein"
/source="PHANOTATE_1.5.1"
/score="-8456.312881812557"
/phase="0"
/translation="MAPSPPTRSIWVRSTATHCCWLVRAASPRSRKRWRSRARSTACVG
CRRRASIRTRTWPGTSRRTAATTSPSRADPSDASRASPSRTATPPRPSWSSSWPHWRVS
DHGLHSGRSRSAGAGIGARRAARHLRRQDGGVPLGGRVARGAARGRCGARPRGGPTQAA
PDPGHHGQGALMGFWSRIKLAFGTTPTYDGVGSGRRALAWMPGNPGAVAALLTTQTELR
AKSRDLVRRNAWAAAGIEAFVANAIGTGIKPQSLIEDPGLRETIQALWRDWTEEADAQG
LTDFYGLQALACRAMLEGGEALVRLRYRRPDDGLAVALQIQVLEPEHLPVTLNRLADNG
NVIRAGIEFDRLGRRVAYHLYRSHPEDGLLAPMSGGGGMETVRVPAAEIVHLFRPLRPG
QIRGEPWLARALVKLNELDQYDDAELVRKKTAAMFAGFVTRDQPEDALMGEGPADASGV
ALAGLEPGTLQILEPGEDVKFSQPADVGGSYAEFMRQQFRAVAAAMGVTYEQLTGDLTQ
VNYSSIRAGLLEFRRRCEAIQHGVIVHQLCRPVWRAWMAQAVLEGWLKLPGFARDAARR
RAWLACKWIPQGWQWVDPQKEFNAMLTAIRAGLLSRSEAVSSFGYDAEDVDREIAADNA
RADALGLVFDSDPRHDRNPPAAASDPVPPQDIQDL"
CDS complement(37466..39430)
/ID="DFLLIFOK_CDS_0039"
/transl_table=11
/phrog="15"
/top_hit="No_MMseqs_PHROG_hit"
/locus_tag="DFLLIFOK_CDS_0039"
/function="head and packaging"
/product="terminase large subunit"
/source="PHANOTATE_1.5.1"
/score="-212536.99302893775"
/phase="0"
/translation="MNDFVYEGFDAIERAWREGLTPDPLLSVSEWADRHRMLSSKASAE
PGRWRTSRTPYLKAIMDCLSPTSPVERVVFMKGAQVGATETGSNWIGYVIHHAPGPMMA
VWPTVEMAKRNSKQRIDPLIEESPVLSELIAPARSRDSGNTILAKEFRGGVLVMTGANS
AVGLRSMPVRYLFLDEVDGYPVDVEGEGDAISLAEARTRTFARRKIFIVSTPTIAGASA
VEREYEASDQRRYFVPCPHCSHRQWLRFEQLRWERGRPETAAYVCESCDQPIAEHHKTW
MLEHGEWRAMAPENGIKTAGFHLSSLYSPVGWRSWRDIASAWESAVSKESGSAAAIKTF
KNTELGETWVEEGEAPDWQRLIERREDYRIGTVPMGGLLLVGGADVQKDRIEASIWAFG
RGKASWLVEHRVLMGDTARDAVWRRLAELIDETWTHDSGNQVPLARFALDTGFATQEAY
AFVRACRDSRLMAIKGVPRGAALIGTPTAVDVSQGGKKLRRGIKVYSVAVGLAKLEFYN
NLRKSADVADDGTTPIYPPGFVHLPKVDAEYVQQLCAEQLITRRDRNGFPVREWQKLRE
RNEALDCYVYARAAAAASGLDRFEERHWRELERQLGIAAPPEQDEHTVTAEDAPDSGGV
VVSGRRIRRRSVVKSRWMS"

CDS complement(39430..39960)
/ID="DFLLIFOK_CDS_0040"
/transl_table=11
/phrog="57"
/top_hit="No_MMseqs_PHROG_hit"
/locus_tag="DFLLIFOK_CDS_0040"
/function="head and packaging"
/product="terminase small subunit"
/source="PHANOTATE_1.5.1"
/score="-35.55982153462462"
/phase="0"
/translation="MGLSIRAYARHRGVSDTAVHKAIRAGRITPEADGTIDPDKADRDW
ARNSEPPKEGTGAKAAKVRVADDPAPNLAAGLPAGGTSLLQARTVNEVVKAQTNKVRLA
RLKGDLVDRNQAIAHVFKLARTERDAWLNWPARISAQMAARLGMDAHALHVALEAAVRE
HLAELGELKVRVD"
CDS complement(40027..40176)
/ID="DFLLIFOK_CDS_0041"
/transl_table=11
/phrog="No_PHROGs_HMM"
/top_hit="No_MMseqs_PHROG_hit"
/locus_tag="DFLLIFOK_CDS_0041"
/function="unknown function"
/product="hypothetical protein"
/source="PHANOTATE_1.5.1"
/score="-0.3391719921203841"
/phase="0"
/translation="VEVQPVAVARFQGVDEQVIQRDLLQLPVDVLFGLLGHVLLLEVSL
MVMT"
CDS complement(40218..40319)
/ID="DFLLIFOK_CDS_0042"
/transl_table=11
/phrog="No_PHROGs_HMM"
/top_hit="No_MMseqs_PHROG_hit"
/locus_tag="DFLLIFOK_CDS_0042"
/function="unknown function"
/product="hypothetical protein"
/source="PHANOTATE_1.5.1"
/score="-1.974378102720856"
/phase="0"
/translation="MVDGVVMNALFSGEAKRFPLLSQPCRAACRPAW"
CDS 40336..40851
/ID="DFLLIFOK_CDS_0043"
/transl_table=11
/phrog="10315"
/top_hit="No_MMseqs_PHROG_hit"
/locus_tag="DFLLIFOK_CDS_0043"
/function="unknown function"
/product="hypothetical protein"
/source="PHANOTATE_1.5.1"
/score="-35.166508650917"
/phase="0"
/translation="MSTIQLTPAQHAILAYAVEHTGGKIEWFPDNVKGGARKKVLDGLC
NRALITTDGTDWSVAAEGYEALGRPRPAPASVEADADLEAEVAAAEATWAPQSAETKPR

TRENSKQAQVIAMLRRPEGATVRQICELTGWQAHTVRGTFANAFKKKLGLTITSDKPEG
GERIYRIA"
CDS 40950..41162
/ID="DFLLIFOK_CDS_0044"
/transl_table=11
/phrog="No_PHROGs_HMM"
/top_hit="No_MMseqs_PHROG_hit"
/locus_tag="DFLLIFOK_CDS_0044"
/function="unknown function"
/product="hypothetical protein"
/source="PHANOTATE_1.5.1"
/score="-6.123083876656854"
/phase="0"
/translation="MKPTRAILTHSNYDADDYAYLTAKGWSDDEILARWSEEAAHGNGP
CHWESASARAKLAAVTGRQQAMRED"
CDS 41234..41602
/ID="DFLLIFOK_CDS_0045"
/transl_table=11
/phrog="8020"
/top_hit="No_MMseqs_PHROG_hit"
/locus_tag="DFLLIFOK_CDS_0045"
/function="unknown function"
/product="hypothetical protein"
/source="PHANOTATE_1.5.1"
/score="-8.940357044390604"
/phase="0"
/translation="MTTAKTIPVTRNEAWGFWGTMDAHAQAAWPIAMNAISDATGQPFE
AVRAFLDSRHGRHFADEVLNRMHAGHALHDAIRAATRQWMEWTIGRRTSKDYGIPRGLP
YLTGFVIHCEIVEEEVAA"
CDS complement(41566..42831)
/ID="DFLLIFOK_CDS_0046"
/transl_table=11
/phrog="498"
/top_hit="No_MMseqs_PHROG_hit"
/locus_tag="DFLLIFOK_CDS_0046"
/function="other"
/product="DNA methyltransferase"
/source="PHANOTATE_1.5.1"
/score="-6649.358931250953"
/phase="0"
/translation="MNWLAERIEHWPMDKLLPYARNARQHSDEQIAQIAASIAEFGFVN
PCLVGADGVLVAGHGRLAAARKLGLSTVPVVVLDHLTPTQRRALVLADNRLAELSTWDN
DLLRIELEALQDDGFDLDLTGFDADALADLLAGEEPEHEGQTEDDAVPEMPEEPVSKPG
DVWRLGPHRLVCGDATTAETYVQLFPHGERADMVFTDPPYNVNYANSAKDKLRGKHRPI
LNDALGAGFHDFLYDALSLLVAHTRGAIYVAMSSSELDTLQAAFRSAGGHWSTFIIWAK
NTFTLGRADYQRQYEPILYGWPEGAERHWCGDRDQGDVWQIKKPQKNDLHPTMKPVELV
ERAIRNSSRPGDVVLDPFGGSGTTLIAAEKSGRVARLIELDPKYADVIVRRWQDWTGKQ
TTRESDGLAFDQAATSSSTISQ"
CDS complement(42828..44324)
/ID="DFLLIFOK_CDS_0047"
/transl_table=11
/phrog="498"
/top_hit="No_MMseqs_PHROG_hit"

/locus_tag="DFLLIFOK_CDS_0047"
/function="other"
/product="DNA methyltransferase"
/source="PHANOTATE_1.5.1"
/score="-1874.9851451500183"
/phase="0"
/translation="VHSPIHITSPPTVLRRRAFHFEVPILNTLNVEYRKVETLIPYARN
PRTHSDEQVARIAASIAEFGWTNPILVDGDHGVIAGHGRLLAARKLGLTEVPVIELAHL
TPAQKRAYVIADNRLALDAGWDEAMLALEFAELADAGFDLDLTGFSASEIEGLLDQIEE
TEPAADEDERAPEGDADEDDVTPPTVAVTRPGDLWLLGEHRLLCADSSDAAAVARLING
ERAHLLFTSPPYANQRDYTTGGIVDWNALMQGVFGTARGALREDAQILVNLGLVHRDGE
WQPYWDGWIEWMRGQGWRRFGWYVWDQSVTVPGDWAGRLAPRHEFVFHFNRQARKPNKI
VPCKWAGHETHLRADGSSTAMRGKDGKVGEWNHAGQPTQEFRIPDSVVEVTRQRGRIGE
GIDHPAVFPLGLPKFFIEAYTDAGEIVFEPFSGGGTTLLAGQLTDRKVRAIELAPEYVD
VALRRWLQHHPGMEPVLATTGQPFAEVTAERLGETAEAAA"

CDS complement(44246..44563)
/ID="DFLLIFOK_CDS_0048"
/transl_table=11
/phrog="No_PHROGs_HMM"
/top_hit="No_MMseqs_PHROG_hit"
/locus_tag="DFLLIFOK_CDS_0048"
/function="unknown function"
/product="hypothetical protein"
/source="PHANOTATE_1.5.1"
/score="-1.1620096002715719"
/phase="0"
/translation="MCEGHGVIRGLRRFCPRDPTRRARRVLPGRKAMRGARARCFSSVR
VQTGVCTVCGLQAPSLGPAASRPACGCPNPLQADLCTPRYTSRARPRFSVGGLSISRFR
S"

CDS complement(44556..44663)
/ID="DFLLIFOK_CDS_0049"
/transl_table=11
/phrog="No_PHROGs_HMM"
/top_hit="No_MMseqs_PHROG_hit"
/locus_tag="DFLLIFOK_CDS_0049"
/function="unknown function"
/product="hypothetical protein"
/source="PHANOTATE_1.5.1"
/score="-2.7801494382634586"
/phase="0"
/translation="MRADAERCRFRSILDVQHFTRFCARIPLTSREKHV"

CDS complement(44700..45095)
/ID="DFLLIFOK_CDS_0050"
/transl_table=11
/phrog="6836"
/top_hit="No_MMseqs_PHROG_hit"
/locus_tag="DFLLIFOK_CDS_0050"
/function="unknown function"
/product="hypothetical protein"
/source="PHANOTATE_1.5.1"
/score="-12.93319342650996"
/phase="0"
/translation="MWSVDDVAERFREAAQTARRLPPVRVQGYFNTWPAILRQPWETYS

GGDVLYRFPPDPATIDRMEETMRWVLWLTEDQRHLVWMRAEEREWREICRRFGCDRTTA
WRRWQKALDIVARRLNERAPRNVASLS"
CDS complement(45082..45297)
/ID="DFLLIFOK_CDS_0051"
/transl_table=11
/phrog="19772"
/top_hit="No_MMseqs_PHROG_hit"
/locus_tag="DFLLIFOK_CDS_0051"
/function="unknown function"
/product="hypothetical protein"
/source="PHANOTATE_1.5.1"
/score="-5.6932154900009415"
/phase="0"
/translation="MNAPSPHYRCPLGRLQPTRPDVDAIKRDGWRDQGILVVSLDDERL
DWIERELVKRIGERLYGRQGDGHVER"
CDS complement(45294..46073)
/ID="DFLLIFOK_CDS_0052"
/transl_table=11
/phrog="159"
/top_hit="No_MMseqs_PHROG_hit"
/locus_tag="DFLLIFOK_CDS_0052"
/function="DNA"
/function=" RNA and nucleotide metabolism"
/product="RuvC-like Holliday junction resolvase"
/source="PHANOTATE_1.5.1"
/score="-262.1518359187799"
/phase="0"
/translation="MNTHSQVGKTAANFPPIFRDVGKLANACQLESHTDQRVRELSADW
QVGNAANLPTSPNPRGCWVSGDFQVGENSLPPTGGEEHAVSSPPTRRGLPGIDGRNRGP
GTAILSLDLGTRTGWALLGRDGSITSGSESFKPRRFEGGGMRYLRFKRRLTEVKQSADG
LDAVYFEEVRRHAGVDAAHAYGGFMAQLTAWCEHHGIPYQGVPVGTIKKHATGKGNAGK
QEMVAAMQTLGFRPADDNEADALALLMWAIATQEVPA"
CDS complement(46070..48256)
/ID="DFLLIFOK_CDS_0053"
/transl_table=11
/phrog="13004"
/top_hit="No_MMseqs_PHROG_hit"
/locus_tag="DFLLIFOK_CDS_0053"
/function="unknown function"
/product="hypothetical protein"
/source="PHANOTATE_1.5.1"
/score="-748639.3508711789"
/phase="0"
/translation="MADVIWLDYNDAPEQHGETPSDTEALRRGLLDRLEAVLLYLFPQG
RIRGGGFYVGDVDGNRGKSLVVELEGERRGLWKDFASDEGGDVIDLWARSRGLSARHDF
PRLADEIRRWLGIATPTRPPEPRSKIRSAPVDELGPYTAKWDYLTADGRLIACVYRYDP
PTGKEYRPWDVRARMWRAPDPRPLYNLPAVAEARDVVLVEGEKAACALIGTGITATTAM
NGARAPIDKTDWTPLAGKHVLVWPDRDAPGWDYAENAARACVTAGAASVAILVPPTDKP
DKWDAADAVAEGFDCAEFIRDGERRVVKTAAPGLPTFTLGMLLDDDSPLPADLVAPRVL
TPGGLLVFGGAPKVGKSDFLLAWLTHMAAGATFLAMTPPRPLRVFYLQAEVQYHYLRER
VKEIRLPASRLGAARANFVATPQLRLVLDDAGINQVIPAIAQAFGGEPPDIIAIDPIRN
VFDGGDVGGENDNAAMLFFLSQRVERLRDAVNPGAGVVLAHHTKKLGKKQFEEDPFQAL
AGAGSLRGYYTTGMLLFRPDETRTTRQLIFELRNGAAIPSMHVDKIRGEWREVDANERL

VMKDYGERLDAERRRKRDAILQILFDEAAQGRCYTANQFAESFEGKAGLGGERTIRERL
SALSTQGYIKYFRNAQDYGLPPARTKFGYLCVEAMVLRSVVGELDPATGEVSLRELPVL
PTHFKCPQSGAALPVENPEVWVYQDDLNDPEEPA"
CDS complement(48256..49035)
/ID="DFLLIFOK_CDS_0054"
/transl_table=11
/phrog="1990"
/top_hit="No_MMseqs_PHROG_hit"
/locus_tag="DFLLIFOK_CDS_0054"
/function="DNA"
/function=" RNA and nucleotide metabolism"
/product="Cas4-domain exonuclease"
/source="PHANOTATE_1.5.1"
/score="-179.94125529752705"
/phase="0"
/translation="MCRFEGVAMLDYNHRPKFHERVNTVIDEALARERASQTPRHYLGA
SRLGVACERALQYEYTQAPVDSGRELPGRVLRVFEVGHALEDLAIRWLRLAGFELYTRK
ADGGQFGFSVAGGRIQGHVDGILAAGPADIELAYPALWECKTMNDKSWRETVKQGVAAA
RPIYAAQLAVYQAYMEGTVPGIASNPALFTAINKDSEEIWFELVPFDGGLAQRMSDRAV
RVITATEAGELLPRHATTPTHFECKSCPWQDRCWRPA"
CDS complement(49022..49270)
/ID="DFLLIFOK_CDS_0055"
/transl_table=11
/phrog="21342"
/top_hit="No_MMseqs_PHROG_hit"
/locus_tag="DFLLIFOK_CDS_0055"
/function="unknown function"
/product="hypothetical protein"
/source="PHANOTATE_1.5.1"
/score="-7.859392559345592"
/phase="0"
/translation="MRCQDIHLQRLRAGGGVVIDPTHNEKAAMEAVLPHLGEYVASIGM
DRPLSAYKREEVLQLVDVVLTAYFDNLRELTPDDVPF"
CDS complement(49375..51048)
/ID="DFLLIFOK_CDS_0056"
/transl_table=11
/phrog="16"
/top_hit="No_MMseqs_PHROG_hit"
/locus_tag="DFLLIFOK_CDS_0056"
/function="DNA"
/function=" RNA and nucleotide metabolism"
/product="DNA helicase"
/source="PHANOTATE_1.5.1"
/score="-216770.86044879767"
/phase="0"
/translation="MLRPRQALLVERSLAALHQHGNTLAIGPTGSGKTIMLSAVAGGVL
EEPDAKSCILAHRDELTAQNREKFGRVNPGVATSVFDAKEKSWGGRATFAMVQTLTRDA
HLDAMPTLDLLVVDEAHHAASPSYRRVIDRVLSRNPRALIFGATATPARSDGKGLREVF
SNVADQITLGELIASGHLVPPRTFVIDVGAQSALAQVRRTATDFDMTEVEAILNRTPIT
DAVIRHWREKAGERKTIVFCSTVAHAQCVADAFVAAGIRAVLIHGELSDAERKVRLAEY
ESGEAQVVVNVAVLTEGYDYTPTSCVVLLRPSSHKSTLTQMIGRGLRTVDPAEHSGVVK
TDCIVLDFGTATLMHGSLEQEANLDGHQHQGEAPTKECPSCEATVPLGCRECPLCGFEW
AIDPAEQAEAIDDFVMTEIDLLKRSNFRWCDLFGCDDALMATGFGAWGGIFFLNGRWHA

VGGGKDLQPRLLAVGDRTVCMAKADDWLNENESLDTAHKTRRWLNEPPTEKQLRYLPQA
MRSDFGLTRYQASALLAFQFNKSSIQRLVLAANDEHRRAA"
CDS complement(51066..51662)
/ID="DFLLIFOK_CDS_0057"
/transl_table=11
/phrog="2300"
/top_hit="No_MMseqs_PHROG_hit"
/locus_tag="DFLLIFOK_CDS_0057"
/function="unknown function"
/product="hypothetical protein"
/source="PHANOTATE_1.5.1"
/score="-105.25293482033442"
/phase="0"
/translation="MSYFDFNDANEQSSFDLIPKGTLVRVRMAIRPGGFDDASQGWTGG
YATRNDNTGSVYLNCEFVVMEGEYARRKLWSLIGLYSPKGPEWANMGRAFVKAILNSAR
GVHPGDASPQAQNARRIAGFADLDGIEFLGKVDWEKDQNGQDKAVIKQAIQPDHKDYAA
LMGNARPSAPAPTTPNAYAQATGRAPVSGRPSWAQ"
CDS complement(51675..52532)
/ID="DFLLIFOK_CDS_0058"
/transl_table=11
/phrog="124"
/top_hit="No_MMseqs_PHROG_hit"
/locus_tag="DFLLIFOK_CDS_0058"
/function="DNA"
/function=" RNA and nucleotide metabolism"
/product="Sak4-like ssDNA annealing protein"
/source="PHANOTATE_1.5.1"
/score="-444.5771360015801"
/phase="0"
/translation="MSLPIISADQRLAEKRGVKGVLVGKSGIGKTSQLWTLDPAATLFF
DLEAGDLAVEGWAGDTIRPRTWQECRDFAVFIGGPNPALRDDQPFSQSHFDAVCARFGD
PTVLDRYDTVFVDSITVAGRLCLQWCKGQPQAYSEKTGKPDTRGAYGLMGQEMIAWLTH
LQHTRGKNVWFVGILDERLDDFNRRVFQLQIDGSKTGLELPGIVDEVVTLAELKADDGS
AYRAFVCHTLNPWGYPAKDRSGRLDQIEEPHLGRLMAKIAGPARPALERLDFARPAPSE
PVQQ"
CDS complement(52529..52810)
/ID="DFLLIFOK_CDS_0059"
/transl_table=11
/phrog="36009"
/top_hit="No_MMseqs_PHROG_hit"
/locus_tag="DFLLIFOK_CDS_0059"
/function="unknown function"
/product="hypothetical protein"
/source="PHANOTATE_1.5.1"
/score="-13.35813993077415"
/phase="0"
/translation="MFKNRTLLDKLKKQHPYLLESLPESIPANGADVAIEDATLDQIAF
AIIALETEVRPISRRMNALRELYDLARKHGALGAHRIGDAFADKGGRS"
CDS complement(52816..53286)
/ID="DFLLIFOK_CDS_0060"
/transl_table=11
/phrog="2480"
/top_hit="No_MMseqs_PHROG_hit"

/locus_tag="DFLLIFOK_CDS_0060"
/function="unknown function"
/product="hypothetical protein"
/source="PHANOTATE_1.5.1"
/score="-17.970404757630668"
/phase="0"
/translation="MSRLTPDQVLATPAGDLAALQSDALFQLKNDAADLLSAAKAIVEH
LERALELKYADRAHALRLVAGKDTGVVHFDDGRVRITADLPKRVEWDQQRLAEIVRRIA
EGGEDPAEYVETAYRVSETKFNAWPESLKSAFAPARTLKTGKPGFRLALLEE"
CDS complement(53283..53375)
/ID="DFLLIFOK_CDS_0061"
/transl_table=11
/phrog="No_PHROGs_HMM"
/top_hit="No_MMseqs_PHROG_hit"
/locus_tag="DFLLIFOK_CDS_0061"
/function="unknown function"
/product="hypothetical protein"
/source="PHANOTATE_1.5.1"
/score="-0.1965529622556015"
/phase="0"
/translation="VLYRVEDVESYEADCLRKSTSERASAGGVA"
CDS complement(53413..53652)
/ID="DFLLIFOK_CDS_0062"
/transl_table=11
/phrog="No_PHROGs_HMM"
/top_hit="No_MMseqs_PHROG_hit"
/locus_tag="DFLLIFOK_CDS_0062"
/function="unknown function"
/product="hypothetical protein"
/source="PHANOTATE_1.5.1"
/score="-1.8246903419809544"
/phase="0"
/translation="MTAFGRFFVFCGPYFPVSAGPVHPPVHGGGGRPHITNPTLTSRFR
NCPKGEERECQTPQPTPTGRPLGRQRSHPGTLAL"
CDS complement(53681..54688)
/ID="DFLLIFOK_CDS_0063"
/transl_table=11
/phrog="No_PHROGs_HMM"
/top_hit="No_MMseqs_PHROG_hit"
/locus_tag="DFLLIFOK_CDS_0063"
/function="unknown function"
/product="hypothetical protein"
/source="PHANOTATE_1.5.1"
/score="-6010.666277931964"
/phase="0"
/translation="MSSKRLSTKSLLDLIDLFERSRQPLVDVEGQRLRGVPGWDLARMT
RMPPKERDAWLECVGYSGSYPAPCRDEHVPVELEEDDDPSRYRYRCPETFRLKYVAAAE
AGVYAVRPAAFLGTVAELLAIPQALRKGIDAPTINGILWNLGKARIGPAHTDVWFVRGL
EQSVEDVFRHFHAPTLPDQGLILTSGQPLPEFVRPPRNYRFAAVRQVLVDYVTTPTVDM
DLLHRILATPADGTLRPLLPVHFDEYTNTLTIRSKSNKPWMIKGERQAAAVRYMYEQAM
NDRWLLPAGEILGAAYPDKKTARSQRMQNLFSGNTEWEDYIANPEKGKYGFLLD"
CDS complement(54651..55880)
/ID="DFLLIFOK_CDS_0064"

/transl_table=11
/phrog="37634"
/top_hit="No_MMseqs_PHROG_hit"
/locus_tag="DFLLIFOK_CDS_0064"
/function="unknown function"
/product="hypothetical protein"
/source="PHANOTATE_1.5.1"
/score="-169049.32150258915"
/phase="0"
/translation="MNDAENLAKLLGHLKPELFRAFMEDEFGVAMPAPEKKQTKTEQRE
AMAAVLATLPVSERQRIEEVAEKIILLTDGAGQDVVEGFRDGIFDDADREAFDALRDQY
ERALWLYANAAEIFKEALDARQADVFRQSVTCYSGFVAPKALVMATDEKAKDAFHENVA
RQLGCAKDDVAIQIFKRLRPNSQTGEDVDLYQISVHNNRPPEIIDCVQASELVAQEVVR
AVSSHITYEPDNGHLEVLSRETDGREALARIAADCLLQSPFSGERIPLKQYDYQSLAAP
RSFDLSGENVASVKVAELGYSDSNHRSLLVKIWAKDPDDIHTAAKSLIGPSFDFRHHRL
TYARLSIRLKKVGRERARTISVILRDDNKCNIKTKREKDRALCDRLLAKWQLVKEIGNV
VEAPVDEVAA"
CDS complement(55877..56041)
/ID="DFLLIFOK_CDS_0065"
/transl_table=11
/phrog="No_PHROGs_HMM"
/top_hit="No_MMseqs_PHROG_hit"
/locus_tag="DFLLIFOK_CDS_0065"
/function="unknown function"
/product="hypothetical protein"
/source="PHANOTATE_1.5.1"
/score="-6.568312356532316"
/phase="0"
/translation="MHGCMIAQPCEIMQLVVRPGSSQDKLVCGRFRAGKMDTRCPPENT
INKGGRRFR"
CDS 56036..56440
/ID="DFLLIFOK_CDS_0066"
/transl_table=11
/phrog="No_PHROGs_HMM"
/top_hit="No_MMseqs_PHROG_hit"
/locus_tag="DFLLIFOK_CDS_0066"
/function="unknown function"
/product="hypothetical protein"
/source="PHANOTATE_1.5.1"
/score="-23.957810545965874"
/phase="0"
/translation="MHAINVKPQEVFQALADPTRIRIVRLLAETGEEACLCELVDSLLE
PQYKLSRHMKALRQAGLLSAEKDGRWVYHRLVRGVRYLELAYDMLRALPDADGRFAQDL
HNFRERMCLREGGRCRVGIQTASLSTAEGE"
CDS 56440..58347
/ID="DFLLIFOK_CDS_0067"
/transl_table=11
/phrog="12316"
/top_hit="No_MMseqs_PHROG_hit"
/locus_tag="DFLLIFOK_CDS_0067"
/function="other"
/product="ATPase"
/source="PHANOTATE_1.5.1"

/score="-419044.5059834016"
/phase="0"
/translation="MGCSCAADAPAVMPRPWRNAKVVTSVCSGLLLLLGFVGGYLGLRA
ELQTIFYLMSVLVGGYYFGREALEELVKEREIGIELLMSAAAIVAGVMGQWAEAATLVF
LYSISEAAEGYTAERARHAIRALMDLAPKTALVRRGDQETRIPVEQLRVGDIFIVLPGE
SVATDGEVIDGHSSVNQAPVTGESVPVEKLPGGKVFAATLNGEGALTVRAAKTFADNTL
SRIIHLVEAAQASKGRSQRFIERFGKRYSPAVLGAGILIGLLPPLFGLPWQDWITRATV
FVVAAAPCALVISIPITLVAAIGTAGRNGLLIKGGVHLENLAKVRVVALDKTGTLTLGR
PQVTDVVPLDGKSEREVLAAAAALELRSQHPLAQAVLERAKSEGIPLNPAQDFQSLTGA
GAKGLVEGVECFVGNPRLFEGLGVAVAAVTPRIEALQREGKTVMLVGTAHAIHGLIAVA
DPLRPEAARTIADLKRAGIERVVMLTGDNPLAAEAIARQVGVDEVFAELSPEDKTRKVA
ELEARHGKVVMIGDGVNDAPALAAAHVGVAMGAAGTDVALETADVALMSDNLARLPYLI
AFSRRTWQVIRQNLALSAVVIGALIAGAVGGYFTLPVAVLAHEISEFVVIASGLRMLKT
"
CDS 58384..58776
/ID="DFLLIFOK_CDS_0068"
/transl_table=11
/phrog="No_PHROGs_HMM"
/top_hit="No_MMseqs_PHROG_hit"
/locus_tag="DFLLIFOK_CDS_0068"
/function="unknown function"
/product="hypothetical protein"
/source="PHANOTATE_1.5.1"
/score="-111.27898712930998"
/phase="0"
/translation="MNTKYGFGKTVEASFDTAIEKVTQELQKEGFGVLTDIDVAATLKK
KLNQDMPPYRILGACNPPLAHRALTAEPSIGLLLPCNVVVRQDDAGKVQVEFMDPNAVL
DLVDKPDITQLAGEVRQKLERVMQAL"
CDS 58820..59554
/ID="DFLLIFOK_CDS_0069"
/transl_table=11
/phrog="No_PHROGs_HMM"
/top_hit="No_MMseqs_PHROG_hit"
/locus_tag="DFLLIFOK_CDS_0069"
/function="unknown function"
/product="hypothetical protein"
/source="PHANOTATE_1.5.1"
/score="-730.3529211014"
/phase="0"
/translation="METGRGASSVMAGAAVAISLLVSPFAVGHAHADGTTRLAQGMMDP
GMMRPGMMGGMHRNMMQRMMGGALPPGIDPALLPEPHSAGAMVLQDYCTQCHNLPGPGM
HTAAEWPRVLGRMNMRMQMMGGGMMGGMMGVMAPSRAELEILLAYLQKHAQQPINAAAY
PDLGMQAGQAFSATCQQCHTLPDPRQHSAQEWPAVVERMRGHEAAMGKIVPDKTTTAEI
IDFLRQHALTSDIRPLPGKARK"
CDS 59551..59796
/ID="DFLLIFOK_CDS_0070"
/transl_table=11
/phrog="No_PHROGs_HMM"
/top_hit="No_MMseqs_PHROG_hit"
/locus_tag="DFLLIFOK_CDS_0070"
/function="unknown function"
/product="hypothetical protein"
/source="PHANOTATE_1.5.1"

/score="-32.88546483853312"
/phase="0"
/translation="MMMDYGMGWGWGFGWIGMILLWLVPILLVLVAIKYLFGGKGRTGM
GARGDDRALAILEEKYARGEIDREEFLQKRDDLKGD"
CDS 59848..60456
/ID="DFLLIFOK_CDS_0071"
/transl_table=11
/phrog="No_PHROGs_HMM"
/top_hit="No_MMseqs_PHROG_hit"
/locus_tag="DFLLIFOK_CDS_0071"
/function="unknown function"
/product="hypothetical protein"
/source="PHANOTATE_1.5.1"
/score="-239.02102475877933"
/phase="0"
/translation="MNTKQKLAGTLAALTLLGTAAISASAQQGQTGPYYGYGWCPGCGM
GPGMMGMGPGMMGPGMMGGRGMMGMGPGMMWGDWDDWDDRYMGRGMMGPGMMGGYGYGP
ALDLTEQQQAKIAQIQEGFRKKQWDLAAKMDAEQAKLNEIYYSGKRDPAVIDNQYKKIY
DLRRQMIQEQVEAQNRMDAVLTKEQKERLRGYSPGGMMR"
CDS 60476..60733
/ID="DFLLIFOK_CDS_0072"
/transl_table=11
/phrog="No_PHROGs_HMM"
/top_hit="No_MMseqs_PHROG_hit"
/locus_tag="DFLLIFOK_CDS_0072"
/function="unknown function"
/product="hypothetical protein"
/source="PHANOTATE_1.5.1"
/score="-14.102308688081646"
/phase="0"
/translation="MKTAMPDWLHRIVARLLLITMVVSAAGLTFGREILQDGPSGPKSI
VAMANADQHDGQFGNSGGATKVCDYSCYNPSHFLGQLPLK"
CDS 60730..61839
/ID="DFLLIFOK_CDS_0073"
/transl_table=11
/phrog="13642"
/top_hit="No_MMseqs_PHROG_hit"
/locus_tag="DFLLIFOK_CDS_0073"
/function="unknown function"
/product="hypothetical protein"
/source="PHANOTATE_1.5.1"
/score="-8254.936477202358"
/phase="0"
/translation="MKLLPKRALYILLAALGTVAAYLIYHHALGNRALPEGLILANGRI
EGDHVTVASKFAGRIAELRVREGDSVQVNQILAVLDDAQIKARVAQARAAVTVLDAQIK
AGETALVVTQKELPLAIASAQAEVSRAAAAVAKAKAAELQARRDATRMHDLLTQNFVNR
QLAERADLALSVASAEHATARHAELQAQAQFSQAKLGADRIRAKESELAALRSQREQAR
ASLAEAESVLADLVIKAPSAGVIVTRIRQAGDVVGAGGPLFDLVNLDTLYLKVYVPEIE
IGKLRLNLPARIYTDAFPDTPFDAKVGYISARAEFTPKEVQTPDERVKLTYAVKLYLEQ
NPEHKLTPGLPADAVIRWKVGVPWAKPNW"
CDS 62016..63821
/ID="DFLLIFOK_CDS_0074"
/transl_table=11

/phrog="293"
/top_hit="No_MMseqs_PHROG_hit"
/locus_tag="DFLLIFOK_CDS_0074"
/function="moron"
/function=" auxiliary metabolic gene and host takeover"
/product="ABC transporter"
/source="PHANOTATE_1.5.1"
/score="-1238157.2125298316"
/phase="0"
/translation="MKSIAGVLSFEQGKVEVFGVGIDTEAAAERIKHRLGFMPQGLGLN
LYPDLSVEENVDFFARLRLVSEDELVLRKQRLLGITRLDRFRDRPMKNLSGGMKQKLGL
VCTLIHEPELLILDEPTTGVDPVSRRDFWAILSALVAEKGTTVIVSTAYMDEASRFHRM
ALMYDGRIIAQGSPDEIRSLAPGSQVLVWTSAQQEALARLRAGFAQVETLGPSLCVYAD
GLTPEAAAMRVRQVLDGVEVNRLEAGEPDMEDVFIALLRLRRGVSTREVPLLPGGTVAA
PRGETAVAIEARSLTRDFGRFRAVDHASFRVSQGEIFGLLGANGAGKTTLIKMLTGLLR
PTDGDGRVAGEDMRAAGWRIKERIGYMSQAFSLYADLTVAENIGLYAGIYGLGTRERRE
RTAWVLEMAQLAGYEKNPAGRLPMGLRQRLALGCALVHRPQVLFLDEPTSGVDPLGRRQ
FWDILFGLARGEGVAILVTTHYMSEAEHCDRLALMYAGRIVADAPPEVLKREVEMQAGA
VLAVTCAAPAAALARLSAAGFEGLSLHGRRVHLFSSSPEQDRARIAAVLAGAEFGAVEV
SARATGMEDVFIYRVTELERSEVRA"
CDS 63646..66129
/ID="DFLLIFOK_CDS_0075"
/transl_table=11
/phrog="No_PHROGs_HMM"
/top_hit="No_MMseqs_PHROG_hit"
/locus_tag="DFLLIFOK_CDS_0075"
/function="unknown function"
/product="hypothetical protein"
/source="PHANOTATE_1.5.1"
/score="-28581.2265655008"
/phase="0"
/translation="MAGACTCFPRRRSRTGRVSRRSWPVRNSAPSRCRRARPAWKTSSF
TVSPNWSAARCGREPEARRGGRPQGMARGAARQVVLYSRLRRPGHFHGVVRLRADARRG
EHPLRRAGLRSIERQPRLRAPLCRFALLRFQGLRAGRARDGAPPQRQPHTRRHRHSRAF
RPQSFGRSQRGRADPPRRHDTLARRDGERLCRRHHGCGEQRPRGRACRPPGRAQPRSGK
RPCRPGQPGGALPVQPGRAQHLVAGPQAHHRDPAFRAAHPDRRGRGPGKGDGFDLQHLC
LHGEPRGVPVRQARSLCRDLGGQYDRTVGDGRLAVRRAIQGQPAVLLPGLDSVHRVRHG
HRPVGLAPRAHSGRGHFPHCRADDDAGARLFRLSHSHPLDGCRRSADRPLPALHVCHPD
HGRQFSQRPRLRRAVARIAGPDRLYRGFARGRLRPVQEEAVDMSGRGRALRIWWLRLGV
LTLKEFIQLLRDPVLLLVAAFLFTVNIYMQGSTLSMQLKAAPLLVHDADHSAASREIVY
RFRQPYFRFAGEIQDPREGIRLLDEGHALAVLDIPPGFQEALLAGRPAALQLQVDATPT
AQAFLAASYAGRIVGEFGRETALARTGSADGGETALPLIREEPRVWFNPNQEDRWFFPI
SELMEAITIMSILLPGAAMVREKQRGTVEQLLVAPLSPFQIMFPKVIAMTVVILVGLSF
SLFAVAAPLFHVPMRGSLVLFYCVTALYVFTNAGLGLFAATIARNVAQMGLLAILMVVP
LILVSGTWTPPEAMPVWMRIGTYVSPMRHYIDVSYGILFKGAGLDLLWDSVAAIAVLGG
AVFGFGVWRFRRQFG"
CDS 66170..66379
/ID="DFLLIFOK_CDS_0076"
/transl_table=11
/phrog="No_PHROGs_HMM"
/top_hit="No_MMseqs_PHROG_hit"
/locus_tag="DFLLIFOK_CDS_0076"
/function="unknown function"

/product="hypothetical protein"
/source="PHANOTATE_1.5.1"
/score="-0.7570808172427546"
/phase="0"
/translation="VQNKSFFHGETADTFVIGHRPVVRFQKRCHFLDHVRAAAEHDAIM
FRIGGSKAKLGKKRTRTEQVRAAL"
CDS 66434..66577
/ID="DFLLIFOK_CDS_0077"
/transl_table=11
/phrog="No_PHROGs_HMM"
/top_hit="No_MMseqs_PHROG_hit"
/locus_tag="DFLLIFOK_CDS_0077"
/function="unknown function"
/product="hypothetical protein"
/source="PHANOTATE_1.5.1"
/score="-0.1244242480678778"
/phase="0"
/translation="LNGGLFRQNQFSVTCDPQYIALALVLDHQHSRPLEQGMRGQHACL
GD"
CDS 66627..66782
/ID="DFLLIFOK_CDS_0078"
/transl_table=11
/phrog="No_PHROGs_HMM"
/top_hit="No_MMseqs_PHROG_hit"
/locus_tag="DFLLIFOK_CDS_0078"
/function="unknown function"
/product="hypothetical protein"
/source="PHANOTATE_1.5.1"
/score="-3.521928901002481"
/phase="0"
/translation="MIAILKRMIRIGKCCHRMSRITQQHQHSEAARNSLMATAMPRPDN
LAIERV"
CDS 66802..67020
/ID="DFLLIFOK_CDS_0079"
/transl_table=11
/phrog="No_PHROGs_HMM"
/top_hit="No_MMseqs_PHROG_hit"
/locus_tag="DFLLIFOK_CDS_0079"
/function="unknown function"
/product="hypothetical protein"
/source="PHANOTATE_1.5.1"
/score="-6.904162453948642"
/phase="0"
/translation="MHPTNHVPPERMTAGQRRAEVASLLAHGLVRLRDAAFAQSAGCPG
ESEFELGFSGHQRLHTYPVNNTREEAL"
CDS 67017..67514
/ID="DFLLIFOK_CDS_0080"
/transl_table=11
/phrog="11806"
/top_hit="No_MMseqs_PHROG_hit"
/locus_tag="DFLLIFOK_CDS_0080"
/function="unknown function"
/product="hypothetical protein"

/source="PHANOTATE_1.5.1"
/score="-77.1315351898641"
/phase="0"
/translation="MKASTVPPTPPSVVAKIAGLPDLSMDEIKALWRQLFGNENPTPNR
QFLERRIAYKLQEIEFRKVDPNLLDRNKRRIKSLMETGKSRKLDRDIRLVPGTVLTREY
QGVEHRVTVAQDGQYEFEGRRYPSLSMIAREITGTRWSGPLFFGVKAPVKQKNSNKQGG
RR"
CDS 67511..68686
/ID="DFLLIFOK_CDS_0081"
/transl_table=11
/phrog="95"
/top_hit="No_MMseqs_PHROG_hit"
/locus_tag="DFLLIFOK_CDS_0081"
/function="integration and excision"
/product="integrase"
/source="PHANOTATE_1.5.1"
/score="-14577.595106029168"
/phase="0"
/translation="MSEVLKRRLRCAVYTRKSTDEGLDQEYNSIDAQRDAGHAYIASQR
AEGWIPVADDYDDPAFSGGNMERPALKRLLADIEAGKIDIVVVYKIDRLTRSLTDFSRM
IDVFERHGVSFVSVTQQFNTTTSMGRLMLNILLSFAQFEREVTGERIRDKIAASKRKGM
WMGGVPPLGYDVDNRRLVPNEREAKVVRHIFQRFVELGSSTLLVKELRIDGVTSKAWTT
QDGRVREGKPIDKSLVYKILNNRVYLGEIRHRDQWYSGEHPPIVERRLWDAVQAILDQN
PRVRGNNTRARVPFLLKGIVVGIDGRALTPWFTRKKNGRIYRYYLPDVLISCRTRPTSD
EAGVVSMDGKSSFSMSPKVSISTGLPPRFSRNVALSCHAARLPTTFVTAVM"
CDS 68687..68962
/ID="DFLLIFOK_CDS_0082"
/transl_table=11
/phrog="No_PHROGs_HMM"
/top_hit="No_MMseqs_PHROG_hit"
/locus_tag="DFLLIFOK_CDS_0082"
/function="unknown function"
/product="hypothetical protein"
/source="PHANOTATE_1.5.1"
/score="-0.4119831063547431"
/phase="0"
/translation="VNENEPVSSATVVLNAAAHFSLVSSRNPRQASRPETTLFLAMASI
QYSGRKPPCQLLQLTTITRRAPSSRGWMPITSMSLCTASWRVSYSA"
CDS complement(69054..69542)
/ID="DFLLIFOK_CDS_0083"
/transl_table=11
/phrog="No_PHROGs_HMM"
/top_hit="No_MMseqs_PHROG_hit"
/locus_tag="DFLLIFOK_CDS_0083"
/function="unknown function"
/product="hypothetical protein"
/source="PHANOTATE_1.5.1"
/score="-73.6134960434228"
/phase="0"
/translation="MTWNPDLLLEDFRRVAGMAGVSLTHDAIAIERRPAPHVPPKGLPP
GTMAVYVFSFGPHVLRVGKVGPNSAARYTAQHYNAGSAKSTLAASLIKHGERIGVVGLN
ETNVPGWIREHTDRVNFILDAPLGIHVLNLLEAFLQCRLRPEFEGFASQRIDREGGQA"
CDS complement(69539..70465)

/ID="DFLLIFOK_CDS_0084"
/transl_table=11
/phrog="No_PHROGs_HMM"
/top_hit="No_MMseqs_PHROG_hit"
/locus_tag="DFLLIFOK_CDS_0084"
/function="unknown function"
/product="hypothetical protein"
/source="PHANOTATE_1.5.1"
/score="-1298.6963664565203"
/phase="0"
/translation="MNQRNMAASVRARLLNRARETRQDFNLILTRYALERLLYRLSISP
HADQFLLKGALLFDLWFDIPHRPTRDADLLGFGSAEIPHVEAAFRDVCTVELDDGIRFQ
PDSVHAEEIRKEANYSGVRVTLIGLLDGARCHVQVDVGFGDAVTPGPEAVDYPVMLSEM
PAPKLRAYPRYTVIAEKLEALVSLGIANSRMKDYFDLWILSRYTDFDGALLCKAIHATF
ERRRTPLPDGVPFGLSDEFAQDRQKQTQWQAFLRKNALVELQISEVVAVLRVFLSPPLD
ALRQAAAFPQTWLAGSGWASGSEESGR"

CDS complement(70462..71061)
/ID="DFLLIFOK_CDS_0085"
/transl_table=11
/phrog="No_PHROGs_HMM"
/top_hit="No_MMseqs_PHROG_hit"
/locus_tag="DFLLIFOK_CDS_0085"
/function="unknown function"
/product="hypothetical protein"
/source="PHANOTATE_1.5.1"
/score="-58.43933353506104"
/phase="0"
/translation="MDSSHQSILDLAAQRGLIRPRDLNERGLPTVALTRLVRQGLLQRV
GRGLYAIPDRTVSEHGALAEVARKHPQAIVCLLSALRLHELTTQSPFEVWLAIPNKARA
PKMDYPPLRIVRFSGAALTEGVEEHLIDGVPVRVTNVARTVADCFKFRNKIGLDVALEA
LQESWRAKRVSMDELWRFAALCRVANVMRPYMESLS"

CDS complement(71066..71407)
/ID="DFLLIFOK_CDS_0086"
/transl_table=11
/phrog="No_PHROGs_HMM"
/top_hit="No_MMseqs_PHROG_hit"
/locus_tag="DFLLIFOK_CDS_0086"
/function="unknown function"
/product="hypothetical protein"
/source="PHANOTATE_1.5.1"
/score="-3.0463905257481887"
/phase="0"
/translation="GHQYIKVNRSRLAFFLAHSRVFAGTLADACGLPSPAVSPASGPIS
LSPGRFSLRPRSPTKGRSPHRPQRGPIQINGLRADESNGWIAASAGKGNHISCVLSVVL
DSYRRFATL"

ORIGIN
1 ggtggggcgg ccgcctgcgc caaaccgcgt tcatactaca caatcgaagc gttgccggcc
61 agcgcgagcg cctgcgggtt caagcggata gcgccggtca tcgcttttcc tcctgccatc
121 cgtcagtcag tttgtctccc ggattgggtg gcggtctcgg cttctcaacc ggtctggttt
181 gctacccagt tcgattcccg agtccgcaac cgaacccacg tccgatggtt ggatggccgt
241 tggcttccgc ggcgaacagc gccgtcatgg actcctgcca actcgttcgg aaggagacca
301 agatggaagt cgttcatctc aaccagaagc aactggccac ccgctggggt gtcagcgagg
361 ccacgctgga gcgctggcgc agcgagggga tcgggcccaa gttcctgaag ctctgcggcc

421 gggtcctcta ccgccagatc gacatcgaag cctacgaggc atcgtgtctg gcgacctcga
481 ccaagtgccg cggcgcccgc gtgggcgcag agtgacgcct gttgttgtgt tgcgttttgt
541 cggtatatat cagcaggtga cgacgtttcg gaacagtcgc ttgaggcgga acggctcagt
601 atcaccggtt ctttctcgcc tcagcaatgg cgttcatcag ttcctggtgc gaccacttca
661 ccgtctttaa gccatacgtc tcgaatgccg cagggagttg agctggcggg gcatcgcctt
721 tgtaaactcc tatcacgccc ttgccacgtt tgagactttc ggtgatctcc cattcgaccc
781 acttgctctt ggcactgtca ggagtcaggt agaccgctgt caccgatgcc cgattgatgc
841 gctcgcggat tttctgcttg atgtagtcct cattctcgct gttgacggcc tgtttcaacg
901 aaaagtcgtc aaactccagt tcgctcttct cgttcttggc ctgtccgcgg agcaggttca
961 ccgcatcttc gtcctcgaag gcaaagctga tgaatacgtg acagccggtg tcagccggcg
1021 tcgtgagcgt ttgcttcgcc ttttcttcga gtttcttcaa atcagaactt ggaatgcttc
1081 cgcctccacc accgcccatt tttcaacctc cctttccgag accgaattca agacatgaaa
1141 tacagtgccc gcagggctcg cctccgccca tgtggcacga atacgttccg gagatcccct
1201 tctcttgcgc cagtttgatg acatcagcct tgctgaactc agcaagaggt gtcacgaccc
1261 tgattcgacg gccaagtgct gcctcgatgg cgcgttccgc ctgtgcgagg aattccgatc
1321 gctggtctgg aaacaaactg aagcgttcag caagtaggcc gatggatacg gtaggcgccc
1381 ccacctgata tgcataggca cttcccatca gcaagaacat caggttgcgc cccggcgtga
1441 aggcatcctc tttgacgtcc aggctcgtgg aagtcagtcc ggaatgaatc actcggccaa
1501 accccgacac atccattcgg gttggcgtag gcagtcccaa ctgcgcgtga acccgctggc
1561 aggtgtccca ctcctttcgc gcagcccgct ggccgtagtc gataaacagc ggatacacct
1621 ccacgccttc ttcgcgagcc attacaccaa tcaatgttga atccagccca ccggaaacca
1681 gattgacaag gctcaccaga ttgcctcccg cttggcgacc cgcatcgtga cccacatggc
1741 cagatcgacg cagccgaccg aatgaccgaa gtctgctgcg acacgctgaa actccgcctc
1801 aatctgttcg tagctcggca accgtgataa ggcactcggc ttgggatcaa tcccccgcgc
1861 taggcgcagg tagtcgagga tgtgggtgtc cagtaccgcg agttgggcgg agtacccgac
1921 gcggcgtaag aaaagacttg cctgcttcgg gccgaagccc cagacctgct tcaccaggat
1981 ctctctggcc tcgcgcgccg atccggccga aaacagaatc tttcgcaggg actgaccttg
2041 gtcccgcatt gtggtcaggg tggttgccaa cagcgaggcc agccgattcc ggaatcgggg
2101 gcggatgtgc cgaactttgc cgtcccgttc tatcgcgaga ggttcggaca aggccgcccg
2161 caagcattgt tcatactcgg cgccgcttcc tttgtatcgt gaagcccgga gcaatccacg
2221 gtccctgatc tgcccggcgg cggcctcggc gacctcgaac accatctggc tgctgaacat
2281 gcacacgcag gcttcgtgca gcaattcctc ttccgtgcga aggttccaat ctcgaacacc
2341 ccggtcgttt ctccggacct cggcgcagag cacccggatt gttcggtcaa cggctgtcat
2401 gttcatgctg cagcctccca gccgaacgcc tcggcacatt ggctgaaaac gtatggcgga
2461 aaattgaagc ggcagaaatt gaggccatcg ccctggtgga tcgcctggcg caggcgctgc
2521 atccagtctg cgtagaacgt caggttgtga gccaacgtgc gggcataaaa cgaaatgtcg
2581 ccctcgcccc acgccgtctg ccgccgaaag aagtccgact gggagaggtg gaacaagagc
2641 cctgtatcgt gatcgaccac cgtctggcac cattccagac cgtcaaaact gtcggcaccg
2701 gcccatgcat agagcgcgat cgagatagga ttacccgtgc cgaggagatg aagaccgacg
2761 tagcgcccgt tgtcatcgag tgccttacgc agtgccatca ccgaccgggt gcgctcgaat
2821 acaccgctac ccaaacgccg ttcggcaacg gcgatcatcg gtacttgggt ctcctgcgcg
2881 acacgtgcgc acagctccgg cagactttcc ggggttccat gcacgatggg cacaatcaca
2941 cggtttccgg cggcgatctg atcctgtcgc cagcgttcaa ctaccaaccc ggcgtgggca
3001 tccgcatcag caggcggatt ctgctcatcg aagccaaagg cgaacgtgca ggggaagcgc
3061 cgcagcacgt cgtggaaatc cgcttggcgc caacttacct gctcgtcctt ccaatagctt
3121 tcgtagttgc cggaatccat caacacagta gcgccggtgt cttgggcagc cgtcagccag
3181 tgctcaaggc catcactgtt gtgcgagcga cccaggtcaa aggcagatac caggaactgc
3241 tggttcatgt cacgcagcga gtgcagcgtc gccaggtatt cagacggtgc cagcgcggtc
3301 ttgacggtgg atacagaagg gaaaaatacg ggcatcgcca gccgattccc gcccagagtg
3361 atctcctcca gccgggtgat catcgtcaca tcgcctcctt cttctgcgcc gatacgatgc
3421 gccgggtttc gttgaaccag ttcgcgtgct cggtcgaaac gtgctcctca acgcgctcgg
3481 cgagagcgat cagccgctcg ggctcaggct gccccttata gggtcctgcc gcggaaagga
3541 ataggaactt ctcgtgcttc aggcgttcgg cagtggcgcg ataagtaacc cagagggtcg

3601 aatactggtt catgtgctgg accccctcca gaaccgcaat cagcgcaccg gcaactgagg
3661 tgacccactt ggcatacatg ggatcaaggt gactcaggac cgggatcagc accgccaaac
3721 tgatctgcac cagcttcagc cgcttgtacc acttctggca ttgcgtactc ttggtgtcgt
3781 accactggag ctggtcttcc agccgatgcc acgccgggtg ttcggccacc gcttccggca
3841 tagtgccctg gttctcctgg cgaccgccct ggctatcgat catcccccct cctgcggcat
3901 actggttcaa atatagacaa gtttgccgta tccttgccaa gattaccacc gttgccggcc
3961 ccgatcttgg tgccgggtga ggaagcgatg aaagacgaca ccggagagat attcaccctc
4021 gacgaagtgg cggcctacct caaggtgggc aaacgcacgg tctatcgctt ggcggcggcg
4081 aagaagattc ccgccttcaa ggtcggaggg acatggcgct tttcgcgcca ggaaatcgac
4141 cagtggatca agcgacagac gaacgacgac acgtccgggg aaggaagcaa gggcaacgaa
4201 taatgcacgc cgaagagctc aaaccgaaca tcaccgttca cgggcctctg tttccggagc
4261 cggtccaggt catcgtcgcg attccgatgg gcgcctcggt caagctggtc gggaagggaa
4321 cccgctccaa ttcggtttat gaaccgattc tgactgcgga gcagctcgcc ctcctcacgg
4381 cctccccggc ccaagaaccc ttcgacggcg atgcccgcaa gtttcgcctc ggcatcgagg
4441 cgatgcgcct ggggctcgct tacgagtacg acccgtattt ctcgctgtcc atcgcccgcg
4501 tcgacccgct gccccaccaa ttggaagcag tttacgacta cttcatgagg ttgccacgta
4561 tccggttcct cctggccgac gacccgggcg ctggcaagac gatcatggcg ggactcctca
4621 tcaaggagct caagattcgc ggcctggtca agcgggtgct catcatcacg ccagccaacc
4681 tcacgttcca gtggcagcgt gagctgaagg acaagttccg cgagaacttc gaggtcatcc
4741 gcagcgacgt cctgcgcgcc aactatggaa tgaacccctg gcaggagaag gatcaggtgg
4801 tgacctcggt gtcgtgggtc tcccgcatcg aggacgccaa ggattccttg ctgcgcagtc
4861 gctgggatct ggtcatcgtc gacgaggcgc acaagatgag cgcctacagc agcgagaaga
4921 agaccctcgc ctaccagctc ggcgaggcgc tgtcgaacat gacggaccac tacctgctga
4981 tgacggcaac gcctcacaag ggcgaccccg agaacttctg cctgttcctc gagctgctcg
5041 accgtgatgt ctatggcgac atccggagcc tggaggaagc gatggcgcgg cacgaggcac
5101 cgttctatct gcgccgcgtc aaggaggccc tggtcacctt ccccgagcca gaaaccgggc
5161 atgtcaaggc gctgttcacc cgccgcaatg tcaccacctc ggaattccag atctcggacg
5221 aggaactgga tttctacgac cgcttgaccc gctatgttga agaccagtcg atcaaggccg
5281 cggcggacga ctcagcccgc ggccgggcgc tcgccttcac catggccatg ctccagcgcc
5341 gcttcgcctc cagcctctac gccgtgcggc gcagcctgga acgcatgcgc gacaagcgcg
5401 agcgcatcct caaggatccg gagggctacc gtcgagagca gatgaacaag cggctgccgg
5461 aagacttcga ggacctgacc gacgaagagc agcaggagat catctcccag ctcgaggacg
5521 tcgtcgccag cgtcgacccg gcggcgctgc gcgaagagat cgccgagctg accaaactga
5581 tcaaccacgc tcgggagctg gaggcgcggg aggtcgagac caagctcacc gccctgaagg
5641 atctgctgac caagcagggt gtcttcgccg accccaagat gaaactcctg gtgtttacag
5701 agcacaagga caccctggac tttcttgccg gggatgggcg cgagacccgt ccgctcggca
5761 agttgcgcga atggggcctc agcctcacgc agatccacgg cggcatgaag atcggggatc
5821 gggacactcc cggaacccgt atcttcgccg aacgcgaatt ccgcgaatca gcccaggtgc
5881 tggttgccac cgaggccgcc ggcgagggta tcaacctgca gttctgctgg ctgatgatca
5941 actacgacat cccctggaac ccggtgcgcc tggagcagcg catgggccgt atccaccgct
6001 acggccagga gaaggactgc ctgatcttca acttcgtctc gaccaacacc cgcgaggggc
6061 gcgtgctgca caagctcttc gagcgcatcg aggcgatcca ggacgatctg gaccccaagc
6121 gcaccggcaa gatcttcaac gtcctcggcg acgtgttccc ggccaaccag ctcgaaaaga
6181 tgctgcgcga catgtactcc cacaaccaga tgacggagga gctgatcaaa cagcgcatcg
6241 tcgagcaggt cgatctcaga cgtttcgaag gcatcactca atccacgttg gaagggctgg
6301 cgaaaaaaga gctgaacctg tccgccatcg tgggcaagtc cgccgaggcc aaggagcggc
6361 ggctggtgcc ggaggtcatc gaggatttct tcctcagcgc cggcccgctt accggtgtgt
6421 cacccaaacc cgtgaaggcg ggaacccatg cctaccggct gggccgggtc ccgcgcaacc
6481 tgtggcccac cggcgaacgc ctggagcccc gcttcggcaa actcgggcgc gagtacggca
6541 acatcgtctt cgacaaggaa ctgctcaagc aggatgccac cctggaatgg gtgacgccgg
6601 gccatccgct cttcgaggtg gtgcgcgagg atgtactggg acaggtgcag gagcacctca
6661 agcgcggcgc cgtgttcttc gacctgcaca gtgagaagcc ctaccgcctg gatgtcttca
6721 gcgcatccat tcgcgacggc cggggcaatg tcttgagccg tcggctattc gtggtgcaaa

6781 ccggcatgga cgggaagacg gccatcaagc agccgacgat cttcctcgat ctcaccccgg
6841 cggagacccc accggcagtc ccggacgatg cccacttgcc ggatcgaggc caggtcgaaa
6901 tcgtcctgct ggaacaggcg cttcagcccc tgctggaaga atcacgccgc gaacgggaac
6961 gcgaggtggc taccatctcc cgccatatcg agctctcgct caacaccatt atcaaccggg
7021 aaaacctgct gctggccgat ctcgtttcgc aaaaggaaag caactccacc gagcccgggc
7081 tggaaggccg aatcaagatc tccgaagaca agctcctgga actcgatcat cgcctgcaaa
7141 gccgccgccg cgagctggag caggaacggc aatgctccat cggcgacatc cagcacatcg
7201 gccgcgcctg ggtactgccc catccggagc gggccgcgcc gggcatcgcc ccgatggtgt
7261 ccgaccccga gatagagcgc atcgccgtcc aggccgtcat cgcccacgag gaagcggaag
7321 gccgcgtggt ggagagcgtg gaagcggaca accgcggctt cgacctcatt tcgcgccgac
7381 cgcaccccga ggaccccaag accgccatcg acgtgcgctt catcgaggtc aaagggcggg
7441 cgcacacggg cgagatcgcc ctgacttcca acgagtacaa gactgcccaa cgcctcgcca
7501 acgactattg gctgtatgtc gtcttccact gcgcaacaaa gaatccttcg gtcaacatcc
7561 tgcgcaatcc ggcaacgctc gactggcagc ccatcgtcaa ggtggagcat taccggttga
7621 aggccgattc catcaggcag ccggtggagt tgcatgaaga tcaggccgct tatcgaaccg
7681 agaaaggaga cgaaccgaca tgctgacccg aatccacatc caaggctaca agtcgctcaa
7741 gaacgtcgag gtgaggctgt ctgaactgtc cgtgctgttt ggtccgaatg ccgccggcaa
7801 gagcaacttt ctcgactgcc tgcaattgct ctccaagctt gcaacctcca ggacgatcaa
7861 agaagctttc gagccgccct accggggcaa gccgctcgaa tcctttacgt ttggcgaaca
7921 aggcatcaag ggcgcactgg agaaggaacg actttccttc tccatcgagg tagatgtaag
7981 gctttccaat gccatcgtcg aagcggtcaa ccggcagatc aaggaaatgc ggcgatccag
8041 cgtgcaggga aacgaggctg gcgaagaggc gaagaccgct ggagccgtgc gtgagcgcaa
8101 cctccgctat agcatcgaaa tcgagatgct gcccaagtct ggcatccttc gcgtggcgga
8161 tgaatatctc gcggcgctta ccgacaaagg cgaacctacc gccaggcgca agccattcct
8221 ctcccgcgag ggcaaccgcc tgcatctgcg cctggaaggc caggcgcatc cgagctacta
8281 cgagcgctac ctggatcaca gcatcctttc cctgccgcac tacccgcccc actacccgca
8341 tctggtggcc atgcgcaagg aactggagaa ctggctgttc ttctatttcg aacccaggga
8401 gaggatgcgc gcggccaatc ccgtcaagga ggtgcgccac atcggactca tgggcgagga
8461 gctggcgtcc ttcctcaaca cgttgaaggc gctggacgag cggcagttcc gtgccgtcga
8521 gaaagccctg cacacgatca tgcccaatgt gcagggcatc ggcgtggaag tcagcaatct
8581 cggcgaggtc gaactcaccc tgaaggaaaa tggcattccg gttcctgcac gcatcctctc
8641 ggaaggcacc ctgcgcatcc tgggcctgct gtccctggcc ggcgccaagg aacaaccctc
8701 gctgatcggg ttcgaggaac cggagaacgg catccatccg cgcagaatcc agctcgtcgc
8761 cgaactcctg aagacgcgcg cttcctccgg tgaaacccag tacatcgtca ccacccactc
8821 gccgctcctg cccgatctgg tgtcggacga atcactgttc gtctgcaagc gggtcgatgg
8881 cgaaacgcgc atcgagcctt tcgcggcatg gggcgccctg gcgaagaggg gcgaggtgga
8941 ccgcgcgctg atgggagcgg aggatctcac cgtctccgaa cggatgctgc ggggggattt
9001 cgatgcgtga gatcgccctc ttcgtcgagg acttcgccca caaggaattc ctcgcggcgc
9061 tgttgcagcg cttggccgcc gaatacggag tcaccgtcca tctggactgg cgcaacgccc
9121 gtcgcggcca cggcgcggtg gtcactgaac tcaagcagtt cctgcgtgat ctgcaacggg
9181 ggcgggatgc gctgccggac cttgtcgtcg tggccaccga cgccaactgc aaggggctgg
9241 tggagcgcat gaggcaaatc accgaggtca ccggcaaggt cgatgtgccc gccgtctgtg
9301 ccgtacccga cccccatatc gagcgctggt tgctggtgga ttccgctgcg ttcaagaccg
9361 ttttcggtcg cgggtgtaat gcaccagacc agaaatgcga gcggatgcgc tacaagaaaa
9421 tgctcatcga cgccatcagg gcgtccgggg tgacgccaag cctgggcggc atcgagttcg
9481 ccgacgacat cgtcgcggcc atggatctca cccgcgcagc cgcggccgat gcctcgctct
9541 cccggctgat cgaagacctg cgagcggtct tcaaggtatg gcagccatga cctatcccaa
9601 acgcctcatc gaagtcgacc tacccatcaa gcgcatctcc gcccactcgc ggcgtgagaa
9661 aagcatccgt cacggtcaca tctccactct gcacatctgg tgggcgaggc ggccgctggc
9721 ggcgtgccgg gcggtgatat gcgcggcgct gtggccggac ccggccgacg agcattgccc
9781 gccggcgttc cggggggcgg ctaaagcggc catgctggac tgggcgcgga accatctggg
9841 gctggcaagc gccgagagct ttccgcggtt cgtcgccatt caaaaggatg cggcccggct
9901 agacgatccc atcgaactgc gccatgcgct gctcgacttc atcgccgact tcgccaattg

9961 ggacaactcc acggtcaagg aatacctgga caccagtcgc gccctcaccc aggccgcgca
10021 cgaggccttg ggcggggcgc ccggtacgcg gccgctggtc gtggacccct tcgccggcgg
10081 cggctcgatt ccgctggaag ctctgcgcgt aggagcggat gccttcgcca gcgacctgaa
10141 cccgattccc gtgctgctca acaaggtcgt cctcgaatac atccccaagt acggccaacg
10201 gctggccgac gaggtgcgca agtggggggc ttggatcaag caggaagcgg aaaaggaact
10261 ggctgcgttc tacccgaagg acccggacgg ggcgacgccc atcgcctacc tctgggcgcg
10321 gacgatccgc tgtgaagggc cggggtgtgg ggcggaggta ccgcttatta ggtctccaca
10381 actttcaacc aagggaaaga agagcgtaaa cctggtcgtt cgcggggaca gcgtgacaaa
10441 gaaagtcata gttgaagtct caagcgccgc agctgctaaa ccagagccaa ctgtacgtcg
10501 cgggagtgtc acgtgccccg tttgtggata tacaacaccc gccgggaatg caaggcaaca
10561 attcaagact agagccgggg gaacacacga tgcccggctg ttgagcgtca ttacagttaa
10621 agcaaacgag ccaggcaaaa gttatcgtgc gccgactgat ggagatcggg ctgcgttctt
10681 agaagcgatg aaccggtggg accaaattaa gtcggacgtt ggggcacacg gtctcacaac
10741 gattccatct gaacccctac cgtatttgcg gagcatcttt aacatcaatt tgctcggagt
10801 aaatcagtgg gggcaattac acactccgcg tcaagccgtc gcattggctt cattcgcgga
10861 gaagatctat caagcgtggg atcggctaag agaactacct gatgggcttg gcggcgcaat
10921 acacctgtgt ctcggcttag cccttgatcg gtctgctgac cggatgacaa gcctgtgcag
10981 atggatctct caaacagagg ctgtgggcta tacgttcggc aggcaggctc tcggtattgt
11041 atgggactac attgaaatta accccttgac gaccgacgca gcttggcttg gtgcgattga
11101 gaatatctgt gaggttatcg aggcagtaga cagggcactc ttaagaaatg gcagtgttca
11161 agaatgctcg gcaacagccc accccttgcc agacgatagt acggcctgct ttgtgaccga
11221 tccgccctac tacgatgccg ttccgtacgc tgacctttct gatttcttct atgtgtggtt
11281 gaagcgaact gttaagcagt cccattcacg ctttgcgcat gagcttctca ccccgaaaga
11341 agatgaggtt gttcaactct cggagaggaa tccgaaatat gcatataaga cgcgtgaaag
11401 atttgaagct ttgatgaccg acgcgatgcg cgaaggaagg cgcgtattga cgccttctgg
11461 catcggagtg gttgtctttg cacataagac cactgctgga tgggaaaccc aattaaaggc
11521 catgatcgac gcaggatggg tcataaccgg atcatggcca attgatacag agaaaccggg
11581 tcggctacgt gctttagatg cagcagcctt ggcgtcttct attcatcttg tgtgccgccc
11641 gcgcgagcac ccggacggct ccctgcgcaa caccgacatc ggcgactggc gggacgtcct
11701 ggccgagctt ccccgccgta tccacgagtg gatgccgcgc ctggccgagg aaggcgtggt
11761 cggcgcggat gccattttcg cctgcctggg gccggcgctg gaaatctttt cgcgctactc
11821 acgggtggag aaggcgagcg gcgaggtggt gagcttgcgc gagtacctgg agcaggtctg
11881 ggccgccgtg gccaaggagg cgctggccca ggtgttcaag gacgcggata ccgccggctt
11941 tgagcctgac gcccggctga ccgccatgtg gctatggacg ctgaacgcgg gcgcgatcaa
12001 tggagacgag tcgccggcag acgaggagga ggccgacgac gaagaagaaa ccaagggcaa
12061 ggctgcccgg gccaagggcg gcttcgtcct cgaatacgac gccgcgcgca agatcgccca
12121 gggcctgggc gcgcatctgg aggacatggg gcatgtggtc gaggtttcgg gcgaaactgc
12181 ccgcctgcta cccgtttccg agcgcacatc ctatctcttc ggcaaggagc aggccgacgc
12241 tccaaccggc ggacgcaaga aaaaggccgc ccagattgac ctgttcggcg agttgacaaa
12301 agtcggtgct gacgagacgg cgtggagcga gaagaccgtg aagaagatgg gcgagaccac
12361 actggatcgg gtgcatcagg ccatgatcct gttcgccgcc gggcgcggcg aggccctgaa
12421 gcgcttcctg gtcgaggacg gtgccgggcg ggatcaacgc ttctggcggc ttgcgcaggc
12481 gctctccgcc ctgtatccgg cgtcgaccag cgagaaacgc tgggtcgacg gcgtgctggc
12541 gcggaaaaag ggcctggggt tgtgagatgg tggatggcct cgtcgcgcgt ttccgggaga
12601 cattcttcgc catcgtccag gccgacgagc tggccggtga tctgaagcag gcggcttccg
12661 cagcgaacct cggggcctgg acgcgggcgc ttaccgaagc agcgatcaga acctgttcgg
12721 ggctggggct gatggcctcc gccaagggac acaagctgga actgctgccc atccaccgca
12781 gcgagtacct ggcgctggat gtgatggctt tcgctgaggg cgaaaagcgc tggcggtttc
12841 cggtggcggt gatggagctg gagaacagcg cccgggaaga ccagatcgcc tactcgcttt
12901 ggaaggtgct gtcggtgcga gcggagctgc ggatcgtgtt ttgttaccgc aggaacggag
12961 aggagatacc cgccttgctg cgccacctgc gggaggaggt ggtcgaggcg atggggctgg
13021 cgggccgggt gaaactcgaa ggggccacgc tgctggtggt gggttcccgt agcgaatcgg
13081 agacattccc gttcggctac ttcgcctggt ggtcgctgga tacgaatact ggaagatttg

13141 aacggctata gaggacaacg atgaaactca aaccctggta tgacgtagtg aagccgcgtg
13201 aggacttgcg ggagggcaag ccgctcgacg cctctgaatt cgccgttcat ctggacaagg
13261 tgcggttggg caccgcgccc aaggattatc gtgacgccgc gaccttcttt gaccggacat
13321 tcctgaccga gaacctgacg ggcctgggtg cggaagtgat gcgaaggctc tcgggcatta
13381 ccaccgaaac gagcgcggtg ttcaacatga ccacccagtt cggcggcggc aagacccacg
13441 cgctgaccct gctctatcac ctggcgcggc atggatcggc cggcaacggc tggcgtggtg
13501 tcaccaagat tctgcagcgg gcaggcattc agaccatccc cgatcacacc gcggtggcag
13561 tcttcgtcgg cacggagttc gattccgtga ccggccgtgg cggcgacgac ggcacgccgc
13621 ggcgcagaac cccctggggc gagctcgcct ggcaattggg aggggaggaa tcctttgccc
13681 atgtcgccca gcacgacgcg gacttcatcg aacccaaggg cgatgtgatc gagaagttcc
13741 tgccttcgga tcgcccctgc ctcatcctga tggacgaggt gctgaactac atcagcacct
13801 accgcgaccg gggctggcac aacaagctct acaacttcat ccaggcgctg tctgagacgg
13861 ttcgcgggcg ccacaacgcg gtgctggtgg gctcgatccc ggcttcggaa ctctcctaca
13921 cggacaagga tgaagcggat cagcagcgct tgaagaacct cctggatcgg ctgggcaagg
13981 cggtgatcgt gtcggtggaa tcggagacct ccgagatcat ccgccgccgg ctgtttgaat
14041 gggacgagcg ggccgtgacc ccggatggcc gggtgatcct caacaaggac gctgaggacg
14101 cctgccggac ttatgccgac tggattcagg agaatcgact gcagctgccg aacctgatca
14161 atccggactt ggcccgcgac gaattccgcg ccacctaccc gttccatccg atggtgattt
14221 ccgtcttcga gcggaagtgg cagaccctgc cccggttcca acaaacgcgc ggcgtcctgc
14281 gactactggc cctatgggtg tcccgggcct accaagaggg ctacaagggg gcgcagcgcg
14341 atcccttgat cacactggga acggcgccgc tggacgaccc catgttccgt gccgccgtgt
14401 tcgagcaact aggtgaaacc aaactggagg cggcagtcac cacggatatc gccggcaaga
14461 aggacgccca cgccgtgcgg ctcgattccg aggccatcga tgctatcaag aaggccaggc
14521 ttcaccgcaa ggtggccacc acgatctttt ttgaatccaa cggtgggcag gtcggtgctg
14581 aggcgcagga agccagcgtg cccgaaattc ggcttgccgt agggcaaccc gaaagcgaca
14641 tcgggaacat cgagaccgtg ctggaggcgc tcaccgacgc ctgctactac ctgaacgtcg
14701 agaagacccg ctacaagttc agcctgaagg aaaacctcaa caagcgattc gccgaccgcc
14761 gggcgactgt gcagggtccg cagatcgacg aagaggtcaa acgggagatt cagaagatct
14821 tcgcgccgaa ggagtttgtc gagcgggtgt tcttccccga gaagagcatt cagatctccg
14881 accggcctgt gatcagcttc ctcgtcgccg acctggaccg gacgatggaa gacgagaagg
14941 cgacccgcca gttcgcggag cagatgatca gggaatgcgg cacgacggcg cggacgttca
15001 agagcgcgtt gatctgggtg gtgccggatt ccgctcagcc catgcgggaa gaggcacgga
15061 agattctggc ctggcaggca attcaagacg aggcggatga tctcaagctc gatgaggcgc
15121 agaagaagca gctcgccgag aacatccaca aggccaaacg tgacctgaag gagtccatct
15181 ggcgagccta caagcacctg ttcctgctgg ccaaggacaa cacggtgaag atggtcgatc
15241 tcgggctcgt ccactccagc gcggcggata cccctatctc caacatcctc aaccggctgt
15301 ctgccgatgg cgacgtggag aagggtgtga gcccgaactt cctggtgcgg aactggccgc
15361 ccgctttcaa ggaatgggcg accaagtcgg tccgcgatgc gttctacgcg tcgccagtct
15421 ttccgcgctt gctgaacgcg gagacggtca aggaaaccat cgcgcgcggt gtcgagagtg
15481 gaatactggc ctacgtgggg aaaggcacct ccggcaagta tcagccattc gccttcaatc
15541 agagcgtcag cccggcagac atcgagttct ccgatgacat gttcgtcatc acggctgaag
15601 tggcacgagc ctacctggat gctcaaacca caccaccgcc ggtgggggtt cccgtggaag
15661 gaggcggcac gggagttgga acagggacag gtggaggtgc ggaaccaggt ggtacaccga
15721 ttgaaccgca accggcaccg accccgcctg ttgccgggcc agcgaagatt gccggcatcc
15781 gatggtctgg cgaggtgcct ccacagaaat ggatgaattt ctacaccaag gtgctctcac
15841 gctttgccgc taccccaggc ttgaagctca ctatttcggt cgaggtcagc ccatcggatg
15901 gcctctcgaa gcaatccctg gaggagacgc agaatgcttt gcgggaactg gggctggatg
15961 accgggtgac ggagaagtag tcgccacatg atgagatcaa cgagtcgaag tggttctcgg
16021 cttttctgcg ctttgatgtc aaggcgtagt tgagttgccg cggttcccca acggagattc
16081 caacatgagc cgtgctcact tgcgttttca ggtggattcc cgcctcgcta ccttgttgag
16141 tcaggaatac tcctcatcgg agcgagcgct gaaggaactc gtggacaacg cttgggatgc
16201 cgacgcgcag caagtaacca tcgtgctccc gaagcccatg agcggggacc ccatcgttat
16261 ccaggacgac ggcaccggta tgacggagga ggaactgcgc cggcactact tatccatcgc

16321 gacagaccgg cgttcgcggc gtggcgagcg caccgtcggg aaaaatcgct tggttaaggg
16381 gcgcaagggt atcggaaagt ttgccggact catggcggca gcagttatga cggtcgaaac
16441 ccgtgcgcgc ggccggttgt gtcgcatcac gttgcggctg gacgatctct cgcaggtaga
16501 agacatcgaa cagctggata tcgggctgca cagtgagccc tgtgggccgg aactgcatgg
16561 tacgacgatc acgctgagcg atctgcatca gggcttggct tatccggatg ccaaccggtt
16621 gcggcaaatc ctgttgcagg actatgggcg ccaggatgat ttttccatca ccgtcgatgg
16681 caagcggctc gatgtcgatg atgtatccgg aagctactca gagcaggaac aggatctgcc
16741 aaatgctggc aaggtgaagc tgcgtttcgc catcagcgac ggcaagtccg ggttgcgtca
16801 acctggcatc actctgcggg tggacggcaa gtccgttgga cggcctggat tcttcggttt
16861 ggatcagcga gatgactttc catccaagct gctgcgcaag ctatatggcg aagtcgaagc
16921 ggatggactg cgcgaccaca tcacggcggg ctgggactcg gcggtcgaga acagcgagct
16981 tctgaaagag gttgagggct acgtccagcc catcttgcga gaagcctatg agcagcagta
17041 ccggcgcgaa attcagttgg cgcaggctcg cttgcagaga gcgatcctcg cgcgcttgtc
17101 ggctctgcca gaacacaagc gcatgtttgc cgaccgcgcg atcaagaaga ttctcgacaa
17161 gtattacggc gagccggaga gcaaagtcga accggtcgta aatgtgctgt tggaagcgat
17221 ggaaagttcc gactaccgca tccttctgga gcatatcgca gaagcaacac cgggggatgt
17281 cgccgggata gccgagcgct tggacgaatt tggcctggcg gagatggcct tccttgtaca
17341 acaagcggcg gctcgccagg tttttcttga tcaactggac gcgctcgccc gtgacgccgc
17401 cacgacggaa gccgtcttgc acaaagccct ggagcggaac ctgtgggtat tcggtgccga
17461 atactcgtta ttcagctcga acagtacttt gcggcggcaa gtggaagata ttctcggaaa
17521 gacctacaca ggtgacaaag ccgataagcg ccccgatctg ctgctcaacg aaaacctgag
17581 cggacaatac ctattgatcg agttcaagcg gccgaaccat gcgctgaatc acggcgacta
17641 tgtgcaggca attgggtaca ggcatgagct atccaagtac ctgagttcgc cgattcaggt
17701 tctgttggtc gggggcaggt cttccgattt ccccgtcgag aatcgtgaac ctcaggtgga
17761 tgctcggata ttcggccaag tcatctcgac cgcgcggcgg cagatcgaat ggttgttgcg
17821 aacgagtaat ggagaaagcg acttacgcat cgggagcggt gttgcataga gggaacttgg
17881 cacatgaact gggttgtaat cttcaacagg ctctttgagc tgatcgatac agacgggacg
17941 ccggactatt tctccggccc gcgcttcctt cgcaaggtgc gggaggtcga tccgtactac
18001 ccgacgtatc aacagtttat tgatcaacgc cgatcccaag gagcaagcac aagccgacgg
18061 gacttcttct ttgacatcct acaagccctg cctgaaccag caagaatctc gttggtgaac
18121 tcaattttgg gcgaattgga aacatcggcg tctgatcgag ccggggaaat cagagcgctg
18181 ctcggtggcg ctgttcctgg acctgttgcc gtcgtgcccg gacatggctg gaatgccgac
18241 agactcaatc agtatttgcg cgacatcgat caatccatca ccgctggcga atttccacgt
18301 gcggtgacgc tcgcatacac ctgtctggag ggcttctacc gtgcgttcgt cacccaatgc
18361 atcccagagc aggccaagga aaccgaaatt atcgcgctct cgaagatcat caaggactac
18421 ctccgcacga ccatcgttgc gtatcctgat gaagcgctca acatggtcac ccatgtctcg
18481 cacacggtag acagggcgcg taatcggttt agcgaggcac acttcgagca agaggcagct
18541 cgctggctgt cggtctacgt gcgcgacctt gtgaacactc agatcagact gcttctgcac
18601 ttcttctaag ccatcaaggc gctgcgaagc aaccaagaag cctcggcctc gcgtcgtgag
18661 acgagtccgg gaagcacctt cccgccgcca tacacccacc ggcgcagctc cttggcagcg
18721 ctcggccagt cgcgctggtt gacccggcgc cgtagagtcg aggtctgcaa ccgccctgcg
18781 ccgaggttga acgtgaagtc cacgatggcc gcgagccgtt cctcgggctc cgtcgccagc
18841 accggacagt agcgcagtgt ggcggcgagc gcggttcgca gatcctgggc cagatagatc
18901 tcgccctcgt cctcggtgat gggcggatgg tcgggcttgc agaggtgtcc gtagccgatc
18961 gtccaatatc ccgccgggca aatatagggg tgggcgcggc gctgtggatc gtgcttggga
19021 acacgatgga agccctcgaa tcgcttggcc aggtcgaccg cggccgttgg tacttcgatc
19081 acgggcgcac ccggtcgaac acgcggccga ggaaccagaa gttcagcacg ccggcccata
19141 gcgcctggtc ggcgtcggtc caggcggcct ggatggccgg aatccactcg acgccagcgt
19201 cgaccgcgcc gaggaaggcg gcggtcttgg ccgcgcagta cagcgccatg aaccagtagg
19261 tgatcaccgg tcgcacgctg gtggagagcg catcggccca gcggacgccg gaccgttggc
19321 cctgggcggc gacggcttcc cgcagcgcct cgactgcacc gctgttccag gcggcctcgc
19381 cggcggcacc gatctcggcc atgcgctggg caccgcgcag cttctcgaac tccagcgcct
19441 tgtcctgcat cgccagctcg tgaccgcgct cgcccttgcg gtccagccac ttgaggatct

19501 cgggcgcgag gcggaaggcc ccgccgagga ggccgccgag cagggtctcg atcattggcc
19561 acctccgaac accttgagtt tgatgacggc gcccgcgacc agagccagga ggaagccggt
19621 ggtgacgagc cggatcacgg tctgccaggc ggtgtgcttg gcggcgttga aggcttcgag
19681 caggccgcgc agctcgcgga tatcgtgggc ggcgtcctcg ccgtcgaggc cgacgtcggc
19741 cagggcgcgc ctcgcgccgc gttcggcggc gcgtgcgagc agctcttcga actcatcgcg
19801 cggcatgacg accatgccgt cttgcagagt gggtgggctc attgctggtc tccggaaacg
19861 aagaacccgc cgggcggcgg gttcggtgga tgaaggaaag ggaaacgatc agacggcgat
19921 gccggggctc cagccggtcg ccttgtagac ggagagcacg ccctcgtcct cgatgaagca
19981 ggtccagccg atcttgggga tgtggtactc ccacgcgccc gcgatgcgca ccgcgatctg
20041 gtcggtgcga ccggcccagg cgccggtggc cgctgccggg acgatgtagc gatcgccgtc
20101 cacgggtgtc gcgggtggag tggcgaggtc gcggtccttg accgacagcc ccaccacggc
20161 gccgaggcgc ttcaggttgg tgtccatgcc gccgttccag ttgtgttcgc cctgggccca
20221 gccgtaggcg aggccgaggt tcgggtcatt caaggccatc aggcacctcc gtagtagtcg
20281 ccgtaacgca ggccgtagcc ggtacggtcg aattcgatga cgtgtcgctg ccaggacacg
20341 tggccgtcgc ggctcgcctc gatctccacc ttgacgtgcg cgttgacgcg cccgaggccg
20401 gagtcggcga gctcctgtgc cagcggccag gtggtaccgt tgccggtgat gccgctcacc
20461 gtgcggcgca gcgagccgtt ctcgttgtag aagcgcacgg tggtcgtctg gcccgcctcc
20521 ggcgtgattg ccccctcggt ttgcagcacg aggtaggcgg tctgggactg gcggttgcgc
20581 gtggcccagc tcacgttgat ctcgccggcc accaccgtcg gatagcgctt gccgttgatc
20641 tgggcgttgc cggggcagta cggacggatg aagcgcttgt tgagcggcag gctcatctcc
20701 gtggccgccg cctcgggcag cgtgccgcgt gccgtgcggg tgaggagctt cacgcgcacc
20761 gtctcgccgg cgacgtactc gggcgtgagg tagtggcgga agccgtcgac gaaccagatg
20821 cggctgcctg cggggtggct cgccggcacc gtatcgagca tcccgcgctc cagggtgacg
20881 gtctgggtcg cgaggtcgag cgacaccacc ttgagccatt cgccgtcgat caccgtgagt
20941 cctccggcag tcgcctcctc gaggccgatg ccggaggtca gccccacact caactgaacg
21001 gccgactgcg gcagcgttgc ggtgaggagc gcggtgggcg tgaagatgcc gaagcccttg
21061 tcggtgaaac tgccgctgac gcgggccagc gccttgaagc cgaaggcgtc cgacgagggg
21121 cgcgaaccgc aggccgccac caggccgtcg agatcgtcga tgtcgccgag caggctctgg
21181 gactcgcccg tgaagtcttt gaccaccgac cagtacggca cctcgaacag ggtctggtgg
21241 gggcacggtg ccggcaggct ggtcggctcc gtccagcccg aggggggcgg cgccgagtag
21301 accgactgcg gcaggccgaa gatgtcctgc acgcactcca cccgcaccgc cccgttcgcc
21361 agctccccat aggcgatgcg cgcaacacgc atcaccatct ggtcaatgcc gtagggcggc
21421 caggagagct tgaccacgtc gccgatattg aggccggagg cctggcggtt ggcgacaaag
21481 gtgcatttgg cgaggggggct tacgagctgc ttgagctcgc gcatggccac ccggttggcc
21541 agctccgctc ggctgatgcc ggggtagttg accgtggtcg ccaccacgcc gccgttgagc
21601 tgcacggcgg cgatgtcctg caccgtgacg ctgccgtcct tgtccgtggt gccgtcgcgg
21661 tagacgacgg tcacctggtt ggtgatctcg ccccaggagg gccgcgtgaa ctcctcgatg
21721 cgcaggatgt tgcccggatc gaagatcggc aggctagaga gcgtgtaatc ggcgcgcgcg
21781 agcttgaggg tgaagagccc ggtgcgcgga tggacgtaga gcagcccgtc cacgtgccgg
21841 agcaccgaca ggatgaagtc ctcgatggtc tcttccttgt tccacagcag cgacagcccg
21901 aaaccctcgg catggagcgc gtcggcggcc gccgtgaagc tggcatcgtc gatgtcgctc
21961 gtcgggtaac ccatgcccca ctcgccgttg gtgaggcact cgcgcacgat gtgggccggg
22021 ttggcgtcgc cggagatctc ggccttggcc gcgtaccact gcctcggcac ccgcttggcg
22081 cgcaccgacc atggcttgat gtaggggttc atggccgcga cccacacccg gcgcaggatc
22141 agcgacacca cgccccggaa ggccgggatg tcggtgccga gacgctcctg caggtaagcg
22201 ttgcgtccct gggtcggccc gcccatcagg atgtccaccg gcccctgcac gccgccctcg
22261 cgctcctccc cgccgaagag attggggttg tcgatggtga tggtggtgtt gccggtgacg
22321 ttgcccgacc aggcgacgcg ctcgcccacc cggatctcgg tgatggcgtc cagcgggccg
22381 tggcacaggg cgaggtgggc gccgaggccg taccaatagc cgaccgtgta ctcctgactg
22441 cctttgccgc cgccgctcat cgtggttgct ccttctcggc ttcctcggcg acgcggatcg
22501 ccattgcgtc acccgtggcg cgcagccaat cggccggaac acctcgctcc acgaactcgg
22561 cccaggtgtg gctgcgcccc tcgaaccagc gacgcatgcc gcgggcgcag tagcccaggg
22621 cacgggcatg ttccagccgc gccagcacgc tcacttcttg cccccggagg acttgcggat

22681 cggccgcacc tgcacgtcgc cgtaccagac cacgttgggc ccggagatca cccgcgtacc
22741 gaacagcacc gggatggggg cgtcctggga ggcgatgggt acatccttgt cgccgatctg
22801 gccgggctgg gcgtcctgca ccttggggcg cggcgcgagc agcgcggaca gtaccgtggt
22861 gacgacccaa atgatgatct gttgccacat gggaaagcct cacacgatgg cgtcgccggt
22921 gaatgggttc ttcaccggga tgaaggggaa gccgccgaag ttgtcgaggt tgccgaacct
22981 gtccttgcaa tgcgccatgg tgtggtcgca gccggcatag agctggaccg catcgcccgc
23041 cgtgaggccg accatgggcg cgaccagcgt gaggtcgatc ccggcgtgac cgacgatcat
23101 gcgcgcgccg gcggtggtgg cgagcatgcc gcccacgaag tagccgtccg gccggctggc
23161 ggcgacggcg acctggacgg tcacgccgct caccgccgct acggcgccgt ccacgcggaa
23221 gctgtccttc agcgcgccgc atccgctgga gtacagcacg tggcggcaca gcagctggta
23281 gcgggcacgc aagcccggcc gcttcaggct cgaggcgatg ggctcgcact tgaggttggc
23341 ctcggcgccg gacagccgcg cgccggtgat ccgccccttc cagtaggtga tgaactcggc
23401 gtcgccgaca tggcgccggt agagggtcac gctcaccaca ccctcgggcg gggcggcgag
23461 aaacagactc gccacggcca ggtcgcgcgg catgcggatc tccagcccca gccgcgcgag
23521 ctcgttgctc tgctcgaggc cgccgcgcga gatcgtcgcc ggctgatagg tctcggattg
23581 gtagtccacc gccgcctgcc ccgaggtgta gagccagcgc tgcgcaccct gggagaagcg
23641 gtagagctcc tgcggttgtc cgtcgtgggc ggactgttcg atgcccagat agctcatggt
23701 cttagctcat gatctcaacg tccgggtaga gagctgcacg cgcgccgtgg tgtccgtctc
23761 ccaatgcagc tcgaccgcgt cctggtcgag ccgggccaac tccaggtacg agacctgcag
23821 gaattgctgg ggctcaaggg tcacgcccag cggcgcgtcc agggagagca actcctcgtc
23881 ctcggagatc tcgttcgccc cggtgatacg gcgatggaag gtgccggccg tggtgaggag
23941 ccgcaggtcg cgccgcaagg gatcggcagc gacgaagcgg gcgtagccga cgttgcggac
24001 gatgaggccg gcatccgtgg ccgccaccgt gcggctgact tcgaggtcgg actcgaaggt
24061 cggcaaccag cagggattcg cccgccccgc gcgtgcggcg agccagccac ggaaggcggt
24121 cgcggcgctc cggtcggtga gcagccaggt cagcgtgcgg ccgatgagcg gggcgcccga
24181 ctcgtcgtcg aaggtcggca ctcccgttcc gtagtcgatg atcgagagct tcctccgcca
24241 cctgtcctcg aggtcggtgc tccggttggg ccgccagtcg aacacccggt agccctgata
24301 ggctggaccc gcgtcggcga ccgcgggcgc cgtgatgtcc tcgacgccga aacggcagcg
24361 gccgacggcg atggtgtcgg taggacgcgc cacggccacc tcgccctcga ggcgggcgag
24421 ccgcgccgga aacacccgcg taccggccgg ccagctcccg gccaagggga gcttgagggt
24481 cagcgtgttg ccggcgatgg agaggatctc caccgcctcg tgccgtgtgt tggagcgcca
24541 cagcaccgcc aggccgccgg cgtggtagtc gctgttggcg gcgtcctgca cggtgagcgc
24601 ggcgctgccg acggcggcct cgcccgcaag ggtggagacg tcggtccaca ccggcaggca
24661 gtagatgcgt gcgccccagg caaacagcag gtgatccagc acctgggtgt ctcggccctc
24721 gatcaggaat ccatactcga aaccgcgtcg cggcagctgc cgcaggcgca cccgctgctc
24781 ggtgccgtcg taggactcca gcacctcggt gagccattcc agccgctcgg tgatcccttg
24841 cgaccagtcc ggacgcatgc caaagaccac cacgcggcgg ccggtgacca ccagcagcgc
24901 ggtgagcccg ccggcaaaga cgaagcggta ggcggcgtcg atcaccggcg ccccgcgcgt
24961 gcccgctgcg aaggtgtaga ggcgcgactg cagcggcccg aaggcgagcg gcggcgccgg
25021 ctggcccgag agcagcagcc cgtccattcc ggtctcgtcg atggcatcga gcgtctgcgc
25081 gtcgaaccga gcgttccaca cctccagcac ccgctcgacc ggactcacca ggttgccgag
25141 atcgatggtc gaagggatga cgtggatgcg gtggtaccag tcggccccga aggcgggcag
25201 cggagcgcct gtgaagccga gccgcagctc gtcgacgggc tgggtgtcga gccgcgcgcc
25261 cggacgcgcg atcgagctcg cactcgggtc gaaaggaaag acgaccggcg cgcgctcgga
25321 gacctccggc gagcgcgacc aggtcggcgg ctcttggcgg gcggcaccgg gcaggatgga
25381 accgggaaag gtcggcatga tccgcctagg ggacaagtct cacggcatag ccatgcacgc
25441 cgctcttgcc gttcttggag tgggccggga aggccatcca ctgctcggcg cccaacgcga
25501 aggactccgc cggcgcgtag tgggtgatgt tgaggtagcg caggtgagcg gtgtggccga
25561 agggcgagaa gaaaccggaa ggccgcgcca cgaacaggta gaacggcagc atcggcgtca
25621 cgccgttgag agtgttgggc acgtggcccc accagtagcg cgccagactg tcgcgcggcg
25681 tgcccgtgcc ccgttcccac agcgccttgg cgcgcttgcc ggtgagcgcc cagtccttgc
25741 agaccgagtg ccagtcggtg gcgccgtcca cctcggcgag gacgaagttc gcgccgccgt
25801 agtacttgta atcgttgaac ggcaggccga agtgccggtc gtggtcgtag ccgaagacgt

25861 agtcggtgaa gccgctgtac gtataggtgt aggcgtccgg gccgaacgcg cccgacacga
25921 acgccccgcc gccgtagctg ccgaatttcg tgagatcccc gaaggcgaga tggtgataga
25981 cgccgggcga gacctcggcg acgagcatga tcagttcggg cgtggcggcc gagagtaggt
26041 ggtaggtgtt ggcggtgccc acctcgaaca ggccgcaggc ctcggctcgg tagctcgtgg
26101 tgttgctcac gtagctggcg ttgacgatcg agccgggttg atcccaccag ggcttgccgg
26161 catcgaatcc cgtcgagccg atcagccaca tgccggtgac ggtgttgtag cgcgtggaga
26221 ggcgctcgtt cgctgcggag cgcaggtgga cgtagagccc gcccttggcg agcgacaggg
26281 tctggccggt gccgtcgggg acccagcgca gctgggtcca gccgttggcc accgcgaaca
26341 cgcgcagggc gtcgagcagc tgatcggggt tggcggaggt accagtctgg taggccatgg
26401 tcgtttactc caggcgcagc gcccagtagt cgcgcacgct ggtgcgatac acgttctgca
26461 ccacgaggtg gtccacgccg cccaccgtga tcaggttctc ggcggcgttg ttgaatccgg
26521 agacgtgata gacaccctcc aactctccga agaggtcgtg atcggtgccg gagttgtatt
26581 gcgtgagcac cacgggcgag agcacgtagg tgccgtccgg tgcctcgcgc aggttgccga
26641 gataggtgta gttcgtcggc cagaccgggc gcggcccgtc gtagcgatac tcggacgacg
26701 agtaggcgag gttctggaac ggcagccagg cgcccgaggg gccgcgcagc atgcaggcgg
26761 tgttggcgtt gttctgtccg tcctcgcccg ggtcgacgaa atggcggtgg ttgggcgagg
26821 tcacgctgta gttgcggccc cgctggccgg tcatcgagcc gccgacgagc agcgggtacg
26881 ggtactgacc cggcgtcgcg tagggcagga cgaagcccag gtgcgccgcc tggtagacgg
26941 tcgagatctt cgccaccacc acggcacgtc ggccgttggc cacgaaccag tacgggatcg
27001 ccgtgttcca gagcgacatc atcggcagcc agccgctgat cgcacccggt tgcgtgtaga
27061 agtcgttggc cgggttgaag ccgatgaagc cgttcaggtc ccacatgtag tagccggccg
27121 tggcgttctc gtaggcccgg atgccgcagt agatctcctc ggcaccggcc agcccgggcg
27181 ccttgaggat gagctctttc gtggcggcgt ccgtcgtcca gtgcagcgcc tgccactgct
27241 ggcccgcggc gaccagattc gggtgagcgg tgaggaaggc atggaagcgg tccagcagat
27301 cgcggtaatc gctggccgtg ccgatttcga acgccattta cgcaagcacc tgtctgacgg
27361 ctcccgcatt gcgttgcagg atgttgagga tggtcttctc gcccgcggac gagttgaggt
27421 agtcggcggc catcgccggg tcgatcacgt tgacgatgcg cacgccctgg ccctggggtt
27481 gcgggggcgg cgtctcgggc accagcccgc cggcggcgaa ggcgagccgg ggcccctgga
27541 tgcgcggtcc gccttcgatg ccgttgatcg cctccaggaa cgccacgccc acgcgcttca
27601 cggcggcggc gttgagcacg tactcgccgg cggagagccg tgccgggatc gagtcgctgg
27661 tggaggtgcc cggaccggtg acgtagccgc cgccggcgaa gccctggaac aacgacgaga
27721 ccagcgatcc gaggcttcca ccgcccgcgc cgccgcctcc gaacaggctg ccgaacagcg
27781 cctcagcgag cttctgcgag gcgatgcggt tgatggcggc cagcaccgag cgggcgaagt
27841 cggcgaaggc gtccttggcc gatttggcgc cgctgccgat ggcctcgaac atctgcgcga
27901 agccgtcctg caccgccccg tcgatggcaa ccgccacgtc gtccacgacg agcttcacct
27961 gcgcgatctc gttcttccag gcctgcacgc gcgccgccgc ctcggggccg atggcggtgg
28021 cggcggcctc cagttgtggc agtagtcctt ccagcgccgc cccggtttcc cgatgtagcg
28081 cgaggatctg ctggcgggcc tgggattccg tgagcagccc cgactgacgc tgcaggttga
28141 tcgactcctc ggcggcgcgc atgcgcgcca gcgcctcgtt gaactgccgc tcataggcgg
28201 agagatcggc ggccgcagcc ttgacatcga tgaggcgccc caccgttgac acgccttcgc
28261 tgtcgccctc ggcacgcagg cgctcgatga gtccctggta ctgccgcgcg atcgcggcgc
28321 gacggtcctc cgccgtggcg gcacccgtca ggtccagcag ctcctcccgc acgcgggcga
28381 gctcctcgcg caggccgcgc tcggcttcga gtgccttgcg ggcgttggcg acctcgacgt
28441 cggcgcgcct gttgttgagg acgatgaggt cggcctcgat cttggcgacc tccgcccgtg
28501 cgcgcaggcg cgtgccttcg tcccgtccgg tggtcgagag gcgccgctgc tcggccagca
28561 ggccctgcag gcgcgcgatc tccgcatcga tctcgcgcgt ctccaacgcg gtcttggccg
28621 cgtagtagtc gcggatggag atcagccggt cttcgagcgc ccgctcgagg gcgcggcctt
28681 gggcgtcgag cgcatccttg aggagcttca gctccgcgtc ggcctgcgac ttcgctaggc
28741 ccaagcgggc ggcgccggtg tccgcagccg gtttgcccgg ggccttcagg cgctcgacga
28801 gggcggggtc ggccttgatg gcgggagcac ggaccgtgat gggtttgggc tcgaacaggc
28861 tgtcgcggaa ctgcgccagc tcgtccaggc gctggacgag gctccccttg agctcggcga
28921 tgatggcctt ggctcccgcg acgttgccgg agagggcctc gacggcggcc gccatgccgc
28981 ccccgatggc ctcgcccagg gcgacgaagg ccttgcccac ggtggccgcg cccagcgcca

29041 ccgtcttcaa tgccagcagg atgccgtcca gcaccgcccg cagcgtcccg ccttccttgg
29101 ccgactccac catgccgagc gccatctcgt tcatggccgg caggaaggtc tcgatgatgc
29161 ggttgccgat gctggtcacg gcgaggcgca cctgggccaa ggcgtcgttg aagacctccg
29221 cctgggcggc ggtgtcgccc ccgatctgca cgccgaggga ttgcagctcc gcggtcagcg
29281 ccccgatgcc ctcgcgtccc tggttgagga aggggatcag gtccgcgccc gacttgccga
29341 agatctcgac cgcgagcgcc gtcttctcgg caccgtcggg cagcgccttg aagcggtctg
29401 ccaggtcgag cagcacctgg tcggtggcgc gcagcgtgcc gtcctggttc tggaaggcga
29461 caccgatggc cgcgaagctg cgggccgcgt cctccgtgcc ggtggccgcc tccagcatgc
29521 gggtggcgag cttgcgcagc ccgccctcga acttctccgc cgagacgccc gacaggtccg
29581 ccgccggaat cagggtcgac agcgactcca cggtgatgcc gacacgctgg gacagcttgc
29641 tgagcgcgtc cgccgactcc agcgaggact tgaccatcgc cccgagcccc gccgccgaga
29701 cggccacgcc gaggttcgcg agcagcccgt tgagggagcg cgcggcatcg cccagaccgc
29761 cgaggttgcg cttgatcgag tcgaacgcgc cccgggtctg gtcgacggcg gtgatcagga
29821 gttgtgcgcg gttgttggcc atcaggtctt cgccagttcg ttctggatcg cgcccgcgag
29881 gcgcggcagc gtccgctgca cggagccggc gaggtcgaag cgtcgtttga gcgccacgga
29941 cttgaccagc acggcgatgg ggatctcctg gccgcgcttg atctgcttgg cgccggtgcg
30001 ggcgcgctcg gcgcgcttga agcgggcgag cggtgccgcg ttctcgcgga tgttctcggc
30061 catcaggatg accttgccgt ccttctcaac gaagtaggcg ttgccggagc gcatcagtgc
30121 atccaccacc tggcggaagc gcttcgggcc tatgcgtccc ggcagcagcg ggatcagcag
30181 attgcccgtc accgtgccgc cccgctcgtg cacgcccaac caggggatct tgctgccgac
30241 cagcagggcc ggcagccgcc cgggcttctt gtcgaggacc ttcgcccgca tgcttgatac
30301 gaagcttgcg cgcttcacct ggaacgccgt gcgcatctgt gcacgggccg catcgcgcac
30361 ctcacgcccg cccgactgca tgccgcgcgc gaccgccttg tggatggcct tgcggcgctc
30421 ggccgaccag gcagcgagct gcctcgggtc gaacaggccg gtggcggaga gactaatccg
30481 catggccgag ctccctctgc agccgctcga tggcacgccg gtcgccttgg ctgccgacgg
30541 cggtgaccac gagcagtgcg ttgaggcgct cgcgctcgtc acgcgcctcg gccacgagaa
30601 aggtctccat ctgggcgagg gtgtagttca ggatgtcggg gaggcggtgc ccggagcgga
30661 tgaggcggtg gaaggcgtcg ccccactcag ccggccattc acccgcgcgg ccgcgtgctg
30721 cacggccggc gccagccgtt gcacgaaaaa atccgcgttc acctcgaaca gggcggcggc
30781 caggcgaacg gcatcgtcca gggacagccg ctcgacccat gcccgctcgc ggcgcgcggc
30841 gacagccagc gcgcagagca aggactcgcc atgctcggcg agcagcacca gccagtccga
30901 ttccgccgac agttgcccgg cgaacggacg cacggctttg atcagcgccg gcacctcgcc
30961 gacccggatc ggggtgatct ccagggactc gccgccgacg tcgagcatga ccggggacgg
31021 cggaaaggcg tcgaatgcgc tcatcgccca cctcacagca gcacgatgcg gccgaactgg
31081 ccgaggtcgc cggcggcggg cttgagcgtg tccgcgagca cctggcccga gagctcgaac
31141 ttcagcaact cgtcggtgat gaccgagagc tccttggccg ggttgatggc cacgcggtag
31201 aggtcgatca ccacctcgcg gttgccgtcg gcggtgttga gcccctcgaa gcgcacccag
31261 cgctctggca gcggctgggt gaacatcgcg gtgaccgagg ccgtgccgta ggcgtagtcg
31321 accttgaagg gctcgacgta gggtccaccg gtggtggcgt cgaggatcac cagcgagccg
31381 tgcttggcgt tcaccgagta ctgcgcggcc ggcagggtct tgggggtggc gctggagtcc
31441 ttcaccacca ccgaggagac gtcctgcttg gcgagcaggt agaggctgcc gggggtgacc
31501 gggttcggca gcgcctccgc ggtgaccgtg cccggggtct gggcggtggt ggcgccgtag
31561 agggcgagcg ccaggttggt cgcgatgagc tcctccaggg tgcaggcgaa ctcgcccttc
31621 ttggtcttga tgagctgcag atcggtgagg cgctgaccgc tcaccgactc ctggtgctcc
31681 agggtctcca ccgagaggga gaccttgagt tcgggcacgt tgcccacgta ggcgagcccc
31741 tgcgggttgc caagtgcgtc gcgggcgccg atgtagacgc gcccctgtcc ggaaaagtac
31801 ggcatggtca gtctccttta cgggtcttga tctgggtctg gggttggggg tcggccggcc
31861 gtgcggctcc gcgctcgatc agccattggg ccgctgcctc gtcgagatcg aggatctcac
31921 cgggggcgtg gagtcggccg gcgtgggtgt gcggttcgat cagttcgatg tgcatggtga
31981 ctatcctttt tgggtgaggt cggaggccag ggtgcggtag cggatctcgt agcgcgcggg
32041 cacggccagg gcttggctgt cggcgtcctc cgtgtcccac tcgcagtcga tctcgcgcac
32101 gcccagagcc agcccgccga ggctcggctc ggccatcaac gcgccgtgcg ccgcgacgat
32161 gagcgcgtcg gccacgtcga aggcgtcgcc ttcgcgcgcg agcgcgacca gcctcagcgt

32221 cagggcgcgg tcgagccggt cgttcgcctg cgcgaggacc tgatcgcctt cgatgaacag
32281 caggagcgcg gggctcgcct cgcgcgggag cggcacggtc ggctggcgat gcagcggcgt
32341 cggtgcgatc gcggaaccga tgcgcgccac gaccgcccgg atcaggcgct cgcggacgga
32401 gggcgtcata gccgggtcag cttggcctgc atctcggagc cgtcgcgcag ctggcggacc
32461 tcgcgcaccc gatacgggct tcccgcgatc tccaccgtgt cgccggcggc gagggtgagc
32521 caggcgctcg ggtactcgag gtggtagtcg cggttcagtg cgagcccatc gagtgccagc
32581 tcatccgggg cgctgaagac gcactgcacc gtgagcgaac ccaccatgac gtcggtgagc
32641 agcccggcgc ggccggcggc ctcgtagaga tccgtcacct gcgccatcac gctgccgtca
32701 gcttcaccag cacaccgggc cgatggcaca tgggcagcgg gttgctctgg gtgtgcaggt
32761 cggtgccgcg gtcgaacttg cgcggctcct gcttggcgta gagcggcagg cccagggtgt
32821 tgaccgtctc gttgaagtcg gcgggcgcga cgtaggtggc gaaggtgtcg acagtgccga
32881 gcgggaaggc gtgggcctcg cccgccgcga tgaagcgccg ggtggtgccg gaggcgtcgc
32941 tcgcctggcc gcggtactcc tcgaaggtga tgccgccgaa ggtgaagccg cggcgcatat
33001 cgttgatgag caccgcgccc tgctgccagt tcttgtaggc ctcctcgacc ttggcgtggc
33061 cggtcagcgc gtcgaagaac tcgggcgagc acaggcagtg cacgccggtc gagaactcgc
33121 ccgagaggtt gtcctcgatg tggcgcagca cctcggcgca cttgcccttg acgttggtgt
33181 tggcgttgcc gagggcgaag ttcacggtct tgggcgtgat gccgaactcg gtgtagagat
33241 tgaccagcgg cgtggcatcg gcatcgagga tctcgccctt cagcgccccc atgcgcaggt
33301 gctccagggt gatggcgtgc ttgttgcgca tggtctccag gtggcgggcg agcacgccgg
33361 ccacgctctc ggtctcggtt tcctgcccga agctgcggat gccctggacc tcctcgggca
33421 gcaccacgtc gtcgtgcggg atgtggggca cgatgaaggc gcgcatcgcc cgcttgccgc
33481 gcttgcccac ggtgccgggc gagccgggcg gcagggtcgg cagcaggttc agcacgccgt
33541 gcatctcctc caccacgacc tggcgggtac gcaccggctt gatcgggaac aggtccaggg
33601 tctccagccg cccgtagcgg ttggggatca ggttgatggc ggcggtgagg ctcgccatcg
33661 agaaggcggg attggaaaag ggattctgca tggtcaggct ccttcacgaa cgaggatgcc
33721 gcgcgcttcg agggcggcga tggcggcgga ctgctgcgcg ggcgtgatgc cggcaggcca
33781 gaccagcgca tggcgggcga cgatggcgtg gcgcgcgacg atgaccgtat cgaggtcggc
33841 ggcggtggcg tcggcgtcga cgatcagcac gccggcgggc aactcggtgc cgtcggtggc
33901 ggccgggtcg agcgccttca gcttgtttgt ggcggtctcg cggccgacga cgctgcccag
33961 gacgaggttc tggccggcgg cgaccgtggc aaggtcgcgc gagtacaggt tgggcgcctc
34021 gtacttcagc aggtcaccca ggttcatcgg ttcaactagg gtcggcatgg ttcactcctt
34081 tccggcgagg tgcttgaccg ccttgatcag tgggttgtgt tcgggggaag cgcggtgccc
34141 ggcgtgcgca tccggatgga tcgccgagcc gatctcgggg ccatgggcac gggctgcgag
34201 cagcgcgcgc cggacatcgg cttccgaaag gcccgcggcc aggaactcgg cggtgcgctg
34261 cgggcagccg ccgagctggc acaactcggc gatggccacg gcctcgcgcc ggtccgtcac
34321 ggtggtggcg ggcgtctcgg gcccgggcgc cgcagaggca gcagcggcga tgagggccgt
34381 ctcggggact tcgggggtcg gatcggggga ttgcatcggg tcgttctcct gtttggcgta
34441 gacggaaccg ggacgcgtgc ggggggccgg gttccgcgaa cggccctgcg ggttcaagaa
34501 ggtcgcgaag acggcgagca gctggtcgcg gctcgccacg gcgtcggcga gcctgctgcc
34561 tatcgcctgc tcgccgaaga agagcccggc ctccgtggcg cgcacggcgt cggggtccag
34621 cccgcgcatc gcggccacgt gcccgacgaa caggccgtag aggcgatcca cctccgcctg
34681 gaggcgttcc gccgcctcct ggtcgagcgg ctcgtggggc gaaaagtcgt tcttgtgccg
34741 gccggcggtg atcgccgtgt agcggtagcc ctgctgggcg tcgcggaccg actggtcgac
34801 gtgcatggcg atgaccccga ccgagcccac accgccggtg cgcgtgacgg cgagatgcga
34861 ggcggcgcat gcgatggcgt aggcggccga gaaggccgag tcggcggcga tcgcccagac
34921 cggtttcacg gcgtcggccg cacggatgcg ctcggcgagc tcgaacacgc cgcccgcctc
34981 gccgccgggg gagtccacgt cgagcaggat gccggacacg gcagggtcgg cgatcgcggc
35041 atccagacgg gcgccgatct cgccgtagct catgaggccg gaggcggcct ccagtcccag
35101 ggtgcggcgc accagcgtgc cgtggatcgg gatcacggtg atgccgggcg gtgccgcgac
35161 ctccgggctc gcgggcatca ccgccggtac ggcggcttca acgtcggcca gaccgatgcg
35221 gggaccgagc acggagagga tcacgtccag cttcgaacgc gcgatgagaa gtggcgtccc
35281 gtagagacgg gacgccaggt gtacgagctg catgtcagag gtcctggatg tcttgcggcg
35341 gcaccgggtc ggaggccgcc gcgggtgggt tgcggtcgtg gcgcgggtcg gaatcgaaga

35401 cgaggccgag cgcatcggcg cgggcgttgt cggcggcgat ctcccgatcc acgtcctcgg
35461 cgtcgtagcc gaaggaggac accgcctccg agcgggacaa gagccccgcc cggatcgcgg
35521 tgagcatcgc gttgaactcc ttctgcggat cgacccactg ccagccctgg ggaatccact
35581 tgcaggcgag ccaggcgcgg cgccgcgcgg cgtcccgggc gaagccgggg agcttgagcc
35641 atccctccag caccgcctgc gccatccacg cgcgccacac cgggcggcag agctggtgga
35701 cgatgacgcc gtgctggatc gcctcgcagc ggcggcgaaa ctccagcagc cctgcgcgga
35761 tggaggagta gttcacctgg gtgaggtcgc ccgtgagctg ctcgtaggtc acgcccatcg
35821 cggcggccac cgcgcgaaac tgctgacgca tgaactcggc gtaggagccg cccacgtcgg
35881 ccggctgcga gaacttcacg tcctcgcccg gttcgaggat ctgcagggtg cccggctcca
35941 gccccgccag cgccacgccc gaggcatcgg ccggcccttc gcccatcagt gcgtcctccg
36001 gctggtcgcg ggtgacgaag ccggcgaaca tggcggcggt cttcttgcgc accagttccg
36061 cgtcgtcgta ctggtccagc tcgttgagct tcaccaaggc ccgcgcgagc cagggctcgc
36121 cgcggatctg cccgggacgc aagggccgga acagatgcac gatctcggcg gcgggcacgc
36181 gcaccgtctc catgccgccg ccaccggaca tgggcgcgag caggccgtcc tccgggtgcg
36241 agcggtacag gtggtaggcc acgcgccggc cgagccggtc gaactcgatg ccggcgcgga
36301 tcacgttgcc gttgtcggcc agccggttga gcgtcaccgg caggtgctcg ggctccagca
36361 cctggatctg gagcgccacc gccagcccgt cgtccggccg ccggtagcgc agccgcacca
36421 gtgcctcgcc gccttcgagc atggcgcgac aggccaaggc ctgcagaccg tagaagtcgg
36481 tgagcccctg ggcatcggcc tcctcggtcc agtcgcgcca gagggcctgg atcgtctcgc
36541 gcaggcccgg gtcctcgatc agcgactgcg gcttgatacc ggtgccgatg gcgttggcca
36601 cgaaggcctc gatgccggcg gccgcccagg cgttgcggcg cacaaggtca cggctcttcg
36661 cgcgcagctc cgtctgcgtc gtgagcagcg cggccaccgc gccggggttg cccggcatcc
36721 aggcgagcgc gcggcgcccg gagcccaccc cgtcgtaggt gggggtcgtg ccgaaggcga
36781 gcttgatgcg gctccagaag cccatcaaag ccccttgccc gtggtgaccc ggatctggcg
36841 cagcttgggt cggcccgcct cgcgggcgag cgccgcatcg acctcgcgca gcgccgcgcg
36901 caactcgtcc accgagcggt actccaccgt cttgtcgccg aaggtgacgc gccgctcgcc
36961 gcgcgccaat gcccgctcca gcgcttcgcg atcggccgct gtgtaggcca tggtcagaca
37021 cccgccaatg cggccagctc gaggaccagc tcggtctggg cggcgttgcc gttcttgaag
37081 gcgatgcgcg cgaagcgtcc gatgggtcgg ccctggacgg tgaagtagtc gctgccgttc
37141 ggcggcttgt gccaggccac gttctcgtcc gaatagatgc ccgccgccgg cagccaacgc
37201 acgccgtcga tcgagctctg gatctccatc gcttcctgct tcgaggcgac gccgctctta
37261 cgagccagca gcaatgtgta gccgtgctcc tcacccagat cgaccgcgtc ggtggcgacg
37321 gagccatcgg cggacagggc tcccaccgcg aaggcggaag cgagggccgg tacgcccatc
37381 atgtgtcccg acgcatcaca acggatgcag cggatttcgt ctcgaccggt ggtcgggtta
37441 cggtaggtgc cttccaagat catcttcagc tcatccagcg actcttgacg acggaccggc
37501 gccggatgcg tcggcccgaa acgacgacgc ccccggagtc gggggcgtct tctgcggtta
37561 cggtgtgttc gtcctgctcg ggcggtgcgg cgatgcccag ttgtctttcc agctcgcgcc
37621 agtggcgctc ctcgaagcga tcgagaccgc tcgccgcggc ggcggcgcgg gcgtacacgt
37681 agcagtccag cgcctcgttg cgctcgcgca gcttctgcca ctcgcgcacc ggaaagccgt
37741 tgcggtcacg gcgggtgatc agctgctcgg cgcagagctg ctggacgtac tcggcatcga
37801 ccttgggcaa atggacgaag cctggcggat agatcggcgt ggtgccgtcg tcggccacat
37861 ccgcgctctt gcgcaggttg ttgtaaaact cgagcttggc gagccccacc gccaccgagt
37921 acaccttgat gccccggcgc agcttcttgc cgccctgcga cacatcgacg gcggtgggcg
37981 tgccgatcag ggccgcgccg cgcgggacgc ccttgatcgc catcagtcga ctgtcccggc
38041 aggcgcgcac gaaggcgtag gcctcctggg tggcgaagcc ggtgtcgagc gcgaagcgcg
38101 ccagcggaac ctggttgccg gagtcgtgcg tccaggtctc gtcgatcagc tcggccagac
38161 gtctccacac cgcatcccgc gcggtgtcgc ccatcagcac gcggtgctcg acgagccagg
38221 acgccttgcc gcgcccgaag gcccatatcg aggcctcgat gcgatccttc tgcacgtcgg
38281 cgccgcccac cagcagcagg ccgcccatgg ggacggtgcc gatgcggtag tcctcccgcc
38341 gctcgatcag gcgctgccag tcgggcgctt cgccttcctc gacccaggtc tcgccgagct
38401 cggtgttctt gaaggtcttg atcgcggcag ccgagccgga ctccttgctg accgcgctct
38461 cccacgcgct cgcgatgtcg cgccaggagc gccagcctac cgggctgtag agcgacgaaa
38521 gatggaagcc cgccgtcttg atgccgttct ccggcgccat cgcgcgccac tcgccgtgct

38581 ccagcatcca ggtcttgtga tgctcggcga tcggctggtc gcaagactcg cacacatagg
38641 ccgccgtctc cggccggccc ctctcccaac gcagctgctc gaagcgcagc cactggcggt
38701 gggagcagtg cgggcacggc acgaagtagc ggcgctggtc ggatgcctcg tactcgcgct
38761 cgacggcgct cgccccggcg atggtcggtg tcgagacgat gaagatcttg cgccgcgcga
38821 aggtgcgcgt gcgcgcctcg gcgagcgaga tcgcatcgcc ttcgccctcg acgtcgaccg
38881 ggtagccgtc cacttcgtcg aggaagagat accgcaccgg catcgagcgt aggcccacgg
38941 cgctattcgc gccggtcatc accagcacgc cgccgcggaa ctccttggcg aggatggtgt
39001 tgcccgagtc gcgcgagcgt gccggggcga tcagctcgga gagcaccggc gactcttcga
39061 tcagcgggtc gatgcgctgc ttggagttgc gcttggccat ctccacggtg ggccacaccg
39121 ccatcatcgg accgggcgcg tggtggatga cgtagccgat ccagttcgag ccggtttccg
39181 tggcgcccac ctgtgcgcct ttcatgaaca ccacgcgctc gaccggcgag gtcggcgaca
39241 ggcagtccat gatcgccttg aggtaaggcg tgcggctggt gcgccagcgg cccggctcgg
39301 ccgaggcctt gctggaaagc atccggtgcc ggtcggccca ctcggagacg gagagcagcg
39361 gatcgggcgt cagtccttcg cgccaggcgc gctcgatggc gtcgaagccc tcgtagacga
39421 agtcgttcat taatcgacgc ggaccttcaa ttcaccgagc tcggcgaggt gctcgcgcac
39481 cgccgcctcc aacgcgacgt gcagcgcgtg tgcgtccatg ccgagccggg ccgccatctg
39541 cgccgagatg cgcgcgggcc agttgagcca ggcgtcgcgc tcggtgcgcg cgagcttgaa
39601 cacgtgggcg atggcctggt tgcgatcgac cagatcgccc ttgaggcggg cgaggcgcac
39661 tttgttggtc tgcgccttga ccacctcgtt gaccgtgcgc gcctgcagca gcgacgtgcc
39721 gcccgcgggc agcccggcgg cgaggttcgg ggccggatcg tccgccaccc gcaccttcgc
39781 ggctttggcg cccgttccct ccttgggcgg ctcggagttc cttgcccagt cgcggtcggc
39841 cttgtccgga tcgattgtgc cgtccgcctc gggcgtgatc cgcccggcgc ggatggcctt
39901 gtgtacggcg gtgtccgata ccccacggtg gcgggcatag gcgcgaatcg acagtcccat
39961 ggtctccatc aagcattggc gcggcccggc tcggattcag cttggcttct ctcgggaaca
40021 gcgcgttcat gtcatcacca tcaacgacac ctcgaggaga agcacgtgac cgagcaagcc
40081 gaaaaggaca tcgaccggca gctgcagcag atcgcgctgg atcacctgtt catcgacacc
40141 ctggaaacgc gcaacagcga ccggctggac ttccacgagg tcagcgtctg ggccgtcaag
40201 agcgccttga tggccgccta ccaggcaggc cggcaggccg cgcgacaggg ctgagaaagc
40261 agcggaaagc gcttggcttc tccagagaac agcgcgttca tgaccacacc atcaaccatc
40321 acgaaggagc atcagatgag caccatccaa ctcaccccg cccagcacgc catcctggcc
40381 tacgccgtcg agcacaccgg tggcaagatc gagtggttcc ccgacaacgt gaaaggcggc
40441 gcccgcaaga aggtactgga cggtctctgc aatcgggccc tgatcaccac cgacggcacc
40501 gactggtccg tcgccgccga gggttacgaa gccctggggc gcccgcgtcc cgcgccggcc
40561 tcggtggagg cagatgcgga tctcgaggcg gaggtcgcag ccgccgaagc cacctgggca
40621 ccgcagagcg ccgagaccaa gccccgcacc cgcgagaaca gcaagcaggc ccaagtcatc
40681 gcgatgctcc ggcgcccgga gggcgcgacg gtgcgccaga tctgcgaact caccggctgg
40741 caggcgcaca cggtgcgcgg caccttcgcc aacgccttca agaaaaagct gggcctcacc
40801 atcacctcgg acaagcccga gggcggcgag cgcatctacc gaatcgcgtg attcaatcgg
40861 aatgggaagc caagcagaaa acgcttggct tcccgcgccg gcagcgcgtt catacgggtg
40921 tcgcaaccat ccacgccaag gagcacagca tgaaacccac ccgcgccatc ctcacccaca
40981 gcaactacga cgccgacgac tacgcctacc tcaccgccaa gggctggagc gatgacgaaa
41041 tcctggcccg ctggagcgaa gaggctgcgc acggcaacgg accctgccac tgggaaagcg
41101 cgtcggcccg cgccaagctg gccgccgtaa ccggtcgcca gcaggcgatg cgagaggatt
41161 gagattgagc ttggcttccc gatcgaacag cgcgttcata cgtgtgtcgc aacgatcaac
41221 gaaggaaaca acgatgacca ccgccaaaac gatccccgtc acccgcaacg aagcctgggg
41281 cttctggggc acgatggacg cgcacgcgca agccgcctgg cccatcgcga tgaacgccat
41341 ctccgacgcc acgggacagc ccttcgaagc ggtgcgggcc ttcctcgaca gccgccacgg
41401 acgccacttc gcggacgaag tcctcaaccg catgcatgcg ggccatgccc tccacgacgc
41461 gatccgcgcc gccacccggc agtggatgga atggaccatc ggacgccgca ccagtaagga
41521 ctacggcatc ccgcgcgggc tgccttatct gaccgggttc gtgattcact gcgagatcgt
41581 cgaggaggaa gtcgccgcct gatcgaacgc caggccatcc gattcgcggg tggtctgctt
41641 gcccgtccag tcctgccagc gccgcacgat cacgtccgca tacttcgggt cgagttcgat
41701 caggcgcgcc acgcgccccg acttctcggc ggcgatcagg gtggtgccgg aaccgccgaa

41761 ggggtcaagt accacatcgc cgggacgact cgaattgcgg atcgcccgtt cgaccaactc
41821 caccggcttc atggtcgggt gcaggtcgtt cttctgcggc ttcttgatct gccagacgtc
41881 gccctggtcg cggtcgccgc accagtggcg ctccgcgccc tcgggccaac cgtagaggat
41941 cggctcgtac tggcgctggt agtcggcgcg gcccagcgtg aaggtgttct tcgcccagat
42001 gatgaacgtg gaccagtggc cgccggcaga gcggaacgcc gcctgcaggg tgtcgagctc
42061 gctcgacgac atggcgacgt agatcgcgcc gcgcgtgtgg gcaacgagca gcgacagcgc
42121 gtcgtagaga aagtcgtgaa agcctgcgcc gagcgcgtcg ttgagaatgg gacggtgctt
42181 gccccgcagc ttgtccttgg cgctgttggc gtagttcacg ttgtacggcg gatcggtgaa
42241 gaccatgtcc gcccgctcgc cgtgcgggaa cagctgcacg tatgtctcgg ccgtggtcgc
42301 atcgccgcag accaggcggt gcggccccag ccgccagacg tcgcccggct tggacaccgg
42361 ctcttccggc atctccggga cggcgtcatc ctcggtctgg ccttcgtgct cgggctcctc
42421 accggcgagt aggtcggcca gcgcatcggc atcgaaaccg gtcaggtcga gatcgaagcc
42481 atcgtcctgc agcgcctcca gttcgatgcg cagcaggtcg ttgtcccaag tcgagagctc
42541 cgcgaggcgg ttgtccgcga gcaccagtgc ccgtcgttgg gtgggcgtca ggtgatcgag
42601 caccaccacc ggcacggttg acaggcccag cttgcgcgcg gcggcgagcc gcccatggcc
42661 cgcgaccagc acaccgtcgg cgccgaccag gcagggattg acgaagccga actccgcgat
42721 ggatgccgcg atctgggcga tctgctcgtc cgagtgctgg cgggcgttcc gcgcgtaggg
42781 cagtagcttg tccatcggcc agtgctcgat gcgctcggcg agccagttca tgcggccgcc
42841 tccgccgtct cgcccaagcg ctcggcagta acctcggcga agggctggcc ggtggtcgcg
42901 agcaccggct ccatgcccgg gtggtgctgc agccagcggc gcagcgcgac gtcgacgtac
42961 tcgggcgcga gctcgatggc gcgcactttg cggtcggtga gttgcccggc cagcagcgtg
43021 gtgccgccgc cggagaacgg ctcgaagacg atctcccccg cgtcggtgta ggcctcgatg
43081 aagaacttcg gtaggcccag cgggaacacc gccggatggt cgattccctc acctatgcgg
43141 ccgcgctggc gcgtcacctc gacgacggaa tccgggatgc ggaactcctg cgtgggctgt
43201 ccggcgtggt tccattcgcc gaccttaccg tccttgccgc gcatcgcggt ggaggagccg
43261 tcggcgcgca ggtgtgtctc gtggcctgcc cacttgcagg gcacgatctt gttcggcttg
43321 cgcgcctggc ggttgaaatg gaagacgaac tcgtgccggg gcgcgaggcg cccggcccag
43381 tcgccgggca cggtcaccga ctgatcccag acgtaccagc cgaagcggcg ccagccctgg
43441 ccccgcatcc attcgatcca gccgtcccag tacggctgcc actcaccgtc gcgatggacg
43501 aggccgaggt tgaccaggat ttgcgcgtcc tcgcgcaacg cgccgcgggc ggtgccgaac
43561 acgccctgca tgagcgcgtt ccagtcaacg attccgccgg tggtgtagtc gcgctggttc
43621 gcgtagggcg ggctggtaaa gagcaggtga gcgcgctcgc cattgatgag gcgcgcgacg
43681 gcggcagcgt cgctgctgtc ggcgcagagc agccggtgtt cgcccagcag ccacaagtcg
43741 ccgggccgcg tgaccgcaac cgtgggcggc gtgacgtcgt cctcgtccgc gtcgccttcc
43801 ggtgcacgct cgtcctcatc ggctgccggt tccgtctcct cgatctgatc gagcaggcct
43861 tcgatctcgg aggccgagaa gccggtcagg tccagatcga agccggcgtc ggccagctcc
43921 gcgaactcca gcgccaacat ggcctcatcc cagccggcat cgagcgcgag ccggttgtcg
43981 gcgatcacat aggcgcgctt ctgcgccggg gtgaggtgcg cgagctcgat caccggcacc
44041 tcggtcagcc ccagcttgcg tgcggccagc agtcggccat ggccggcgat cacaccatgg
44101 tcgccatcga ccaggatcgg gttggtccag ccgaactcgg cgatgctggc ggcgatgcgc
44161 gcgacctgct cgtcgctgtg cgtgcgcgga ttgcgggcgt agggaatcag cgtctcgacc
44221 ttgcggtact cgacgttgag cgtgttcagg atcggaacct cgaaatggaa agcccgccga
44281 cggagaaccg tgggcgggct cgtgatgtgt atcggggagt gcacaaatcc gcctggagcg
44341 gatttggaca gccgcaggct ggccgcgaag cggcaggccc cagggatggg gcctgcaaac
44401 cgcaaaccgt gcaaaccccg gtttgcactc tgacgctaga aaagcatcgc gctcgcgccc
44461 cccgcatggc ttttcggcca ggaaggaccc gtcgtgcgcg gcgggtcgga tcgcgagggc
44521 agaaacgacg aaggccacgg atcactccgt ggccttcaca catgcttctc tcgcgaggtt
44581 agcggaatcc tagcgcaaaa acgggtgaag tgttgcacgt ccaaaatgga tcgaaaccgg
44641 catcgttccg catccgcccg catggcctgg accgcgttgg aaacgtcccg caacttcact
44701 catgagaggc tggccacgtt ccggggtgcc cgctcgttca ggcggcgggc gacgatgtcg
44761 agcgccttct gccaccgccg ccaagccgtg gtgcgatcac agccgaagcg ccggcagatc
44821 tcgcgccact cccgctcctc ggcacgcatc cagacaagat ggcgctgatc ctcagtgagc
44881 cacagcaccc agcgcatggt ctcctccatg cggtcgatgg tggccgggtc gggtggaaag

44941 cggtacagca cgtcgccgcc cgagtaagtc tcccacggct ggcgcaggat ggccggccag
45001 gtgttgaagt agccctggac gcgcaccggc ggtaggcgcc gcgcggtctg ggctgcctcg
45061 cggaagcgct cggcgacgtc gtcaacgctc cacatggccg tctccctggc gaccgtagag
45121 ccgctcgccg atgcgcttca ccagctcccg ctcgatccag tcgagccgct cgtcgtcgag
45181 cgagaccacc aggatgccct ggtcccgcca gccgtcgcgt tttatggcat cgacgtccgg
45241 gcgagtcggt tgcaggcgcc cgaggggcga acgatagtgg gggctgggcg cattcatgcc
45301 ggcacctcct gcgtcgcgat cgcccacatc agcagtgcca gcgcgtcggc ctcgttgtcg
45361 tccgcgggcc ggaaacccag ggtctgcatg gcggccacca tctcctgctt gccggcgttg
45421 cccttgccgg tggcgtgctt cttgatcgtg cccaccggaa cgccctggta cgggatgccg
45481 tggtgctcgc accaggcggt cagctgggcc atgaagccgc cgtaggcgtg tgcggcatcc
45541 actccggcgt ggcggcgcac ctcctcgaag tacaccgcat ccagcccgtc cgccgattgt
45601 ttgacctcgg tgagccggcg cttgaagcgc aggtaacgca tgccgccgcc ctcgaagcgc
45661 cggggcttga aggactccga gccgctggtg atggagccgt cacggccgag cagcgcccag
45721 ccggtccggg tgcccaggtc gaggctcagg atggccgtgc ccggtccccg attccgaccg
45781 tcgatgccgg gcagaccccg acgggtcggg ggagaggaca ccgcgtgttc ctctcccccc
45841 gtaggggggа gggagttttc gccaacttgg aaatctccgg aaacccagca accacgcggg
45901 tttggggaag ttggcaagtt ggcagcgttg ccaacttgcc aatctgccga caactcccta
45961 acgcgttgat cagtatggga ttcaagttgg caggcgtttg ccaacttgcc aacgtctctg
46021 aaaatcgggg ggaagttggc agcggttttg ccaacttggc tgtgcgtgtt catgcgggct
46081 cctcggggtc gttcaggtcg tcttggtaaa cccacacctc ggggttctcg accggcagcg
46141 cggcccccga ttgcgggcat ttgaagtggg tgggcagcac cggcagttcg cgcagcgata
46201 cttcgcccgt ggctggatcg agctcgccga cgaccgagcg cagcaccatc gcctcgacgc
46261 acaggtagcc gaacttggtg cgggcgggcg gcaggccgta gtcctgcgcg ttgcggaagt
46321 acttgatgta gccctgggtc gagagcgcgg agaggcgctc gcggatggtg cgctcgccgc
46381 cgagccccgc cttgccctcg aagctttccg cgaactggtt ggcggtgtag cagcgccctt
46441 gggccgcctc gtcgaacagg atctgcagga tggcgtcgcg cttgcgccgg cgctcggcat
46501 cgaggcgctc gccgtagtct ttcatcacca gccgctcgtt ggcatcgacc tcccgccact
46561 cgccgcggat cttgtccacg tgcatcgagg ggatggccgc gccgttgcgc agctcgaaga
46621 tcagctggcg cgtggtgcgc gtctcgtccg ggcgaaacag cagcatgccg gtggtgtagt
46681 agccgcgcag actcccggcc ccggccagcg cctggaacgg gtcctcctcg aactgcttct
46741 tgccaagctt cttggtgtgg tgggccagca cgacgccggc gcccgggttc accgcatcgc
46801 gcaggcgctc cacccgctgc gacaggaaga acagcatggc ggcgttgtcg ttctcgccgc
46861 ccacgtcacc gccgtcgaag acgttgcgaa tgggatcgat ggcgatgatg tcgggcggct
46921 cgccgccgaa ggcctgcgcg atggcgggga tgacctgatt gatcccggcg tcgtccagca
46981 ccaggcgcag ctgcggcgtg gcgacgaagt tggcgcgtgc cgcccccagg cgactggccg
47041 ggaggcggat ctccttcacg cgttcgcgca ggtagtggta ctgcacctcg gcctggaggt
47101 agaacacccg cagcgggcgc ggcggcgtca tggcgaggaa ggtcgcgccc gcggccatgt
47161 gcgtgagcca ggccagcagg aagtcgctct tgcccacctt gggcgcgccg ccgaatacca
47221 gcagaccgcc cggggtgagc acgcggggcg cgacgagatc cgccggcagc ggcgagtcgt
47281 cgtccagcag catgcccagg gtgaaggtcg ggagcccggg ggcggcggtc ttcacgaccc
47341 tgcgctcgcc atcacgaatg aactcggcgc aatcgaagcc ctcggccacg gcatcggccg
47401 cgtcccactt gtcgggcttg tcggtgggcg gcacgaggat ggcgaccgac gccgcgcccg
47461 ccgtcacgca ggcacgggcg gcgttctccg cgtagtccca accgggcgca tcgcgatccg
47521 gccagaccag cacgtgcttg ccggcgagcg gcgtccagtc ggtcttgtcg atcggcgccc
47581 gcgcgccgtt catggccgtg gtcgccgtga tgcccgtccc gatcagggcg caagccgcct
47641 tctcgccctc gaccagcacc acgtcgcgcg cctcggccac ggccggcagg ttgtagagcg
47701 gacgcgggtc gggggcgcgc cacatgcggg cgcgcacgtc ccaggggcgg tactccttgc
47761 cggtgggcgg gtcgtagcgg tagacgcagg cgatgagccg accgtccgcc gtaaggtagt
47821 cccacttggc ggtgtagggg ccgagttcgt cgactggcgc gctgcggatt ttggatcggg
47881 gttcgggtgg gcgagtgggc gtcgcgatcc cgagccatcg gcggatctcg tccgcgaggc
47941 ggggaaagtc gtgccgcgcg gaaaggccgc gcgagcgcgc ccatagatcg atgacgtctc
48001 cgcctttcgtc ggaggcgaag tccttccaca aaccgcgccg ctcgccctcg agctcgacta
48061 ccaggctctt gccccggttg ccgtcgacgt cgccgacgta gaacccgccg ccgcggatgc

48121 ggccctgcgg gaacaggtag agcagcacgg cctccaggcg gtcgagcagg ccccggcgca
48181 acgcctcggt gtcggagggc gtctcgccat gctgttcggg ggcgtcgttg taatcgagcc
48241 aaatgacgtc tgccatcaag ccggtctcca gcagcggtcc tgccagggac acgacttgca
48301 ctcgaagtgg gtcggcgtgg tggcgtgccg gggcagcagc tcgccggcct cggtggcggt
48361 gatgacccgc acggcccggt ccgacatgcg ctgcgcgagc ccgccgtcga acggcacgag
48421 ctcgaaccag atctcctcgc tgtccttgtt gatggcggtg aacagcgcgg gattgctcgc
48481 gatgcccggc acggtgcctt ccatgtacgc ttggtagacg gcgagctggg cggcatagat
48541 cgggcgggca gcggccacgc cctgcttgac ggtctcgcgc caggacttgt cgttcatcgt
48601 cttgcactcc cacaacgcgg gataagcaag ctcgatgtcg gcagggcccg cagccaggat
48661 gccgtcgacg tgcccctgga tgcggccgcc ggcgacggaa aaaccgaact gcccgccgtc
48721 ggccttgcgc gtgtagagct cgaagccggc caggcgcagc cagcggatcg ccagatcctc
48781 cagggcatgc ccgacctcga acacccgcag cacccgcccg ggcagctcgc ggccggaatc
48841 gaccggcgcc tgggtgtact catactgcag ggcgcgctcg caggccacgc cgagccgcga
48901 cgcgccgaga taatggcgcg gcgtttgtga ggcacgttca cgcgccagcg cctcgtcgat
48961 aacggtgttg acgcgctcgt ggaacttggg gcggtggttg taatcgagca tggccacccc
49021 ctcaaaacgg cacatcgtcg ggcgtgagct cccgcaagtt gtcgaagtag gccgtgagca
49081 ccacgtcgac cagttgcagg acctcctcgc gcttgtaggc cgacagcggc cggtccatgc
49141 caatcgacgc gacgtactcg ccgaggtggg gcaggacggc ctccatcgcg gccttctcgt
49201 tatgggtggg atcgatcacg acgccgcctc ctgccctcag tcgttgcaga tggatgtcct
49261 ggcagcgcat cgagcagaag cgcttgaaca ggggcttgcc gtccggcgca cgcgggccat
49321 tcctcggcga cagccagcag aagccgcgtc cctctcgtcc gcagatcgcg catatcacgc
49381 tgccctccgg tgctcgtcgt tggccgccag cacgagacgc tggatggacg acttgttgaa
49441 ctggaaggcg agcagcgccg aggcctggta gcgggtgagg ccgaagtcgg aacgcatggc
49501 ctgcggcagg tagcgcagct gcttctcggt gggcggctcg ttgagccagc ggcgggtctt
49561 gtgggcggtg tcgagggact cgttctcgtt caaccagtca tccgccttgg ccatgcagac
49621 cgtgcgatcg ccgaccgcca gcagacgagg ctgcagatcc ttgccgccgc cgaccgcgtg
49681 ccagcggccg ttgaggaaga agatcccgcc ccaggcgccg aagccggtgg ccatcaaggc
49741 gtcgtcgcag ccgaagagat cgcaccagcg gaagttggag cgtttaagca ggtcgatctc
49801 cgtcatgacg aagtcgtcta tggcttccgc ctgctcggcc ggatcaatgg cccactcgaa
49861 gccgcacagt ggacactcgc ggcagccgag tggaaccgtc gcctcgcagg agggacactc
49921 cttggtgggc gcctcgccct ggtgctggtg gccgtcgaga ttggcctcct gctccagcga
49981 gccgtgcatg agcgtcgcgg tgccgaagtc gaggacgatg cagtcggtct tgacgacacc
50041 ggaatgctcg gccggatcga cggtgcgcag cccacgaccg atcatctggg tcagcgtcga
50101 cttgtgcgag ctcgggcgca gcagcaccac gcaggaggtg ggtgtgtagt cgtagccttc
50161 agtcagcacc gccacgttga cgactacttg tgcctcgccg ctttcatatt cggcgagccg
50221 gaccttgcgc tcggcgtccg acaactcgcc gtggatcagc acggcgcgga tgccggcggc
50281 gacgaaggcg tcggccacgc actgcgcgtg cgccacggtg gagcagaaca cgatggtctt
50341 gcgctcgccc gccttctcac gccagtgacg gatcaccgcg tcggtgatgg gcgtcctgtt
50401 gaggatcgcc tccacctcgg tcatgtcgaa gtcggtggcg gtgcggcgca cctgggcgag
50461 cgcagactgc gcgccgacgt cgatgacgaa ggtgcgcggc ggcaccaaat ggccggaggc
50521 gatgagctcg cccagggtga tctggtcggc cacgttgctg aacacttcgc gaagtccctt
50581 gccgtcgctg cgcgcgggcg tggccgtggc accgaagatc agcgcgcggg ggttgcgcga
50641 cagcacgcga tcgatcacgc gccggtagga gggcgaggcg gcatggtgcg cctcgtcgac
50701 caccaacaga tcgagcgtgg gcatcgcgtc gagatgagca tcgcgcgtaa gcgtctgcac
50761 cattgcgaag gtggcgcgcc cgccccagga cttctccttg gcatcgaaca ccgaggtcgc
50821 gacgcccgga ttgacccggc cgaacttctc ccggttctgg gcggtgagct cgtcgcggtg
50881 cgcgaggatg caggacttgg cgtcgggctc ctccaacacg ccgccggcca ccgccgacag
50941 catgatggtc ttgcccgagc cggtcgggcc gatggccagg gtgttgccgt gctggtgcag
51001 cgccgcgagc gagcgctcca ccagcagggc ctgacgggga cgaagcatca tgccggcagt
51061 ccccсttact gcgcccagct cggacggccc gaaaccggag cgcgacccgt ggcctgggca
51121 taggcgttgg gcgtggtggg cgccggggcg gacggacgcg cgttgcccat cagcgcggcg
51181 tagtccttgt gatcgggctg gatcgcctgc ttgatgacgg ccttgtcctg gccgttctgg
51241 tccttctccc agtcgacctt gccgaggaac tcgatgccgt ccagatcggc gaacccggcg

51301 atgcgccgcg cgttctgggc ctgggggctg gcatcgcccg gatggacgcc gcgcgcggag
51361 ttcaggatcg ccttgacgaa ggcgcggccc atgttggccc actcgggccc cttggggctg
51421 tagagaccaa tcagcgacca cagcttgcgg cgggcgtact caccctccat caccacgaat
51481 tcgcagttga ggtacaccga gccggtgttg tcgtttcggg tggcgtagcc gccggtccag
51541 ccctggctcg catcgtcgaa gccgcccggg cggatggcca tgcgcacgcg caccagcgtg
51601 cccttgggga tcaggtcgaa cgaggactgc tcgttggcgt cgttgaaatc gaaatagctc
51661 atggtcgtgg ctccttattg ctgtacgggt tcggaaggcg cggggcgcgc gaagtcgagg
51721 cgttcgagtg cagggcgggc cgggccggcg atcttggcca tcagccggcc gaggtgcggc
51781 tcctcgatct ggtcgaggcg gccggagcgg tccttggcgg gatagcccca ggggttgagc
51841 gtgtggcaga cgaaggcgcg gtaggcgctg ccgtcgtcgg ccttgagctc ggccagcgtc
51901 accacctcgt cgacgatgcc cggcagctcg agcccggtct tggagccgtc gatctgcagt
51961 tggaagacgc ggcgattgaa atcgtcgaga cgctcgtcga ggatgccgac gaaccacacg
52021 ttcttgccgc gggtgtgctg cagatgggtg agccaggcga tcatctcctg gcccatcagg
52081 ccgtaggcgc cgcgggtgtc cggcttgccg gtcttctccg agtaggcctg cggctggccc
52141 ttgcaccact gcaagcaaag acgcccggcg acggtgatcg agtcgacgaa cacagtgtcg
52201 tagcggtcga gcacggtcgg gtcgccgaag cgcgcgcaga ccgcgtcgaa gtgggactgg
52261 ctgaagggtt ggtcgtcgcg tagcgccgga ttcgggccgc cgatgaacac tgcgaagtcg
52321 cggcactcct gccaggtgcg cggccggatt gtgtcgccgg cccagccctc gacggcgagg
52381 tcgccggcct ccaggtcgaa gaacagcgtg gccgcagggt cgagcgtcca gagctgcgag
52441 gtcttgccga tgccgctctt gcctaccagc acgcctttca cgccacgctt ttccgccagg
52501 cgctggtcgg cggagatgat gggaaggctc atgagcggcc tcctttgtcg gcgaacgcgt
52561 ctccgatccg gtgcgcgccg agggcgccgt gtttgcgggc gaggtcgtag agctcgcgca
52621 gagcgttcat gcggcggctg atgggccgga cttccgtttc cagggcgatg atggcgaagg
52681 cgatctggtc caaggtggcg tcttcgatgg ccacgtcggc accattcgcc gggattgatt
52741 ccggcaagga ctccagcaga taggggtgct gctttttcag cttgtcgagc agggtgcggt
52801 tcttgaacat gacggtcact cctccaacag ggcgagacgg aatccgggct tgccggtctt
52861 gagggtgcgg gcgggggcga aggcactctt cagcgactcg ggccaggcgt tgaacttggt
52921 ctcggagacg cggtaggcgg tctcgacgta ctcggccggg tcctcgccgc cctcggcgat
52981 gcggcgcacg atctcggcga gccgctgctg atcccactcg acccgcttgg ggaggtcggc
53041 ggtgatgcgt acgcggccgt cgtcgaaatg cacgacgccg gtgtccttgc cggcgaccag
53101 acgcagggcg tgggcgcggt cggcgtactt cagttccagc gcgcgttcga ggtgctcgac
53161 gatggccttg gcggccgaga gcagatcggc ggcgtcgttc ttgagctgga acagcgcatc
53221 gctttgcagc gcggcgagat cgccggccgg cgtggcgaga acctggtcgg gggtgaggcg
53281 gctcatgcca cgcctcccgc gctggcgcgc tcggaggtgc tcttgcgcag gcagtccgct
53341 tcgtaggact cgacgtcctc gacgcggtag agcacacgcc cctggatttt cagaaaaacc
53401 ggcccgattc cttcagagcg ccagcgttcc agggtggctt cgctgacgcc ccaacggtcg
53461 gccagttggc gttggttgag gtgtttgaca ctcacgttct tctcctttcg ggcagttgcg
53521 aaaacgtgag gtcagtgtcg gattcgtgat gtgcgggcgt ccgccaccac catgtacggg
53581 cggatgtacg ggcccggccg aaacggggaa atacgggcca cagaaaacaa aaaaccgccc
53641 gaaggcggtc atgcggaaca gcgacgggaa gacgctgggg tcagtcgagc aggaacccgt
53701 acttgccctt ctcggggttg gcgatgtagt cctcccactc ggtgttgccg ctgaagaggt
53761 tctgcatgcg ctggctgcgg gcggttttct tgtccgggta tgcggccccc aagatttcgc
53821 cggctggcaa cagccaacgg tcgttcattg cctgctcgta catgtagcgg acggccgccg
53881 cttggcgttc gcccttgatc atccagggct tattgctctt gctgcggatg gtgagcgtgt
53941 tggtgtactc gtcgaagtgg accggtagga gcgggcgaag cgtcccgtcc gcaggcgtcg
54001 ccaggatgcg atggagcagg tccatgtcca cggtgggtgt cgtcacgtag tcgacgagta
54061 cctggcgcac cgccgcgaat cggtagttcc tggggggacg cacgaactcg ggcaacggct
54121 gacccgacgt caggatcagg ccctgatccg gcagtgtcgg tgcatggaaa tgccggaaga
54181 cgtcctccac cgactgctcg aggccacgca cgaaccaaac gtccgtatgc gccgggccga
54241 tccgtgcctt gcctagattc cacaggatgc cgttgatcgt gggcgcatcg atcccttttc
54301 gcagggcttg tgggatcgcg agcagttcgg cgacggtgcc aagaaacgca gcaggtctaa
54361 cggcatagac ccctgcctcc gcggccgcga catacttcag ccggaaggtt tccgggcagc
54421 gataccggta ccgggaggga tcgtcgtctt cctccagttc gacggggacg tgttcgtcgc

54481 ggcatggcgc cgggtaggag ccggagtacc cgacgcattc gagccacgca tcccgctcct
54541 tgggcggcat ccgcgtcata cgtgcgaggt cccagccagg gacgccgcgt aggcgttgtc
54601 cctcgacatc gacgagcggc tgccgagagc gctcgaacag gtcgatcagg tcaagcagcg
54661 acttcgtcga caggcgcttc gacgacattg ccgatctcct tgaccagctg ccatttggcg
54721 agcagccgat cgcacagcgc gcggtccttc tcgcgcttgg tcttgatgtt gcacttgttg
54781 tcgtcgcgca ggatcacgga gatcgttcgc gcccgctccc gcccgacctt cttcaaacgg
54841 atcgacaatc tggcgtaggt caggcggtga tggcggaagt cgaaggatgg gccgatcagc
54901 gacttcgcgg ccgtgtggat gtcgtcgggg tccttggccc agatcttcac caatagcgag
54961 cggtggtttg agtccgagta gccgagctcg gccaccttga ccgaggcgac gttttcgcct
55021 gagaggtcga agctgcgcgg cgccgcgagg ctttggtagt cgtactgctt gagcgggatc
55081 ctctcgcccg agaagggaga ctgcagcaga cagtccgcag cgatacgcgc caacgcctcg
55141 cgcccgtcgg tctccctgga cagcacttcc aggtggccgt tgtccggctc gtaggtaatg
55201 tgggaggaca cggcgcggac cacttcctgg gccaccagtt cgctggcttg cacgcagtcg
55261 atgatctcgg gcggacggtt gttgtggacg ctgatctggt agagatcgac gtcctcgccg
55321 gtctgcgagt tcggccgcaa ccgcttgaag atctgaatgg cgacgtcgtc cttggcgcag
55381 ccgagctgcc gagccacgtt ctcgtggaac gcgtccttcg ccttctcgtc ggtcgccatg
55441 acgagagcct tcggcgcgac gaagcccgag tagcacgtga cgctctggcg gaacacgtcg
55501 gcctgccggg catccagcgc ttccttgaag atctcggcgg cattcgcgta cagccacaag
55561 gcacgctcgt attggtcgcg caaggcatcg aaagcttcac gatccgcatc atcgaagatg
55621 ccgtcccgga acccctcgac gacgtcctga cccgcaccgt cagtcaacag gatgatcttc
55681 tcggcgacct cctcgatcct ctgccgttcc gatacaggca aggtagccag caccgctgcc
55741 atcgcctcgc gctgctcggt cttcgtctgc ttcttttcgg gggccggcat ggcgaccccg
55801 aattcatcct ccatgaaggc gcggaacagc tcgggtttga ggtgcccgag cagcttggcc
55861 aggttctcag catcattcat ctaaatcgtc ttcctccctt attgatcgtg ttctccggcg
55921 ggcatcgtgt gtccattttt cctgcccgga acctgccgca aacaagcttg tcctggctcg
55981 accctggcct cacaacgagt tgcattatct cgcatggttg cgctatcatg caaccatgca
56041 tgcgatcaac gtaaaacccc aggaagtctt tcaggcgctt gccgacccga cccggatccg
56101 catcgtccgt ctgctggccg agaccgggga ggaggcttgc ctttgcgagc tggtggacag
56161 ccttttggag ccccaataca agctctcgcg ccatatgaag gccttgcgac aggcggggct
56221 gctctcggcg gagaaggacg ggcgctgggt gtatcaccgc ctcgtgcgcg gcgtgcgtta
56281 tctggaactg gcctacgaca tgctgcgggc gctgcccgac gccgacgggc gcttcgccca
56341 agacctgcac aacttccgtg agcggatgtg cctgcgcgaa ggcgggcgct gccgggtcgg
56401 catccagacc gcatcgctgt cgacggcgga gggcgagtga tgggctgcag ctgcgccgcc
56461 gatgcccccg cggtcatgcc gcgaccctgg cgcaacgcca aggtcgtcac ctccgtctgc
56521 tccggactat tgctgctgct cggtttcgtc ggcggctatc tcggcctgcg cgccgagcta
56581 cagacgattt tctatctcat gtccgtgctc gtcggcggct actatttcgg ccgcgaggcg
56641 ctggaggagc tggtcaagga acgggagatc ggcatcgaac tgctcatgtc cgccgccgcg
56701 atcgtcgccg gggtcatggg ccagtgggcg gaggccgcga cgctggtgtt cctctattcc
56761 atctccgagg cggcggaagg ctacacggcc gagcgcgccc ggcacgcgat ccgcgccctc
56821 atggacctgg ccccgaagac cgccctggtg cggcgtggcg atcaggaaac ccggattccg
56881 gtcgagcaac tccgggtggg cgatatcttc atcgtcctgc ccggcgagtc ggtcgccacc
56941 gacggcgagg tgatcgacgg ccattccagc gtgaaccagg ccccggtgac cggcgaatcc
57001 gtgccggtgg agaaactgcc gggcggcaag gtgttcgccg ccaccctcaa cggcgagggc
57061 gcactcacgg tgcgcgccgc caagaccttc gccgacaaca cgctgtcccg catcatccat
57121 ctggtggaag cggcgcaggc gagcaagggg cgcagccagc gcttcatcga gcgcttcggc
57181 aagcgctaca gccccgccgt cctgggcgcg ggcatcctca tcggcctgct gccgccgctg
57241 ttcggcctgc cgtggcagga ctggatcacc cgcgccacgg tgttcgtcgt cgccgcggcc
57301 ccctgcgccc tggtgatctc cattcccatc acgctggtcg ccgccatcgg cacggccggt
57361 cgcaacgggt tgttgatcaa gggcggcgtg catctggaga acctcgccaa ggtgcgcgtg
57421 gtggcgctgg acaagaccgg cacgctcacc ttgggccggc cgcaggtcac ggacgtggtt
57481 ccgctggacg ggaagagcga gcgtgaagtg ctggcggcgg ccgcggccct cgaactgcgc
57541 tcgcagcatc ccctggcgca ggccgtgctg gagcgggcca agagcgaggg catcccgctc
57601 aaccccgcgc aggatttcca gtccctcacc ggcgcgggcg ccaagggatt ggtcgaaggc

57661 gtcgagtgct tcgtgggcaa cccgcggctc ttcgaggggc tgggggttgc cgtggccgcg
57721 gtgacccccc gcatcgaggc cctgcaacgg gaaggcaaga ccgtgatgct ggtcggcacc
57781 gcccacgcca tccatggatt gatcgccgtc gccgatccgc tgcggcccga ggcggcccgg
57841 acgatcgccg atctcaagcg cgccggcatc gagcgggtgg tcatgctcac cggcgacaac
57901 ccgctggctg cggaggcgat cgcccggcag gtgggcgtcg acgaggtgtt cgccgagctt
57961 tccccggaag acaagacgcg caaggtcgcg gaactggagg cgcgccacgg caaggtggtg
58021 atgataggcg acggcgtgaa cgatgccccg gcgctggcgg cggcccacgt gggcgtcgcc
58081 atgggcgcgg ccggcaccga tgtggccctg gagactgcgg acgtggcgct gatgtccgac
58141 aacctcgcgc ggctgcccta tctcatcgcc ttcagtcgcc gcacctggca ggtgatccgg
58201 cagaacctcg ccctgtccgc ggtggtgatc ggtgctctga tcgcgggcgc cgtgggcggc
58261 tatttcaccc tgccggtcgc ggtgctggcc cacgagatca gcgagttcgt ggtgatcgcg
58321 agcgggctgc ggatgctcaa gacatgacaa ttcccaacga aacccaatga aaggagcggc
58381 aacatgaata ccaagtacgg ctttggcaag acggtggagg cttctttcga cacggcgatt
58441 gaaaaggtga cgcaggaact gcaaaaggaa ggcttcggcg tgctcaccga catcgacgtg
58501 gcggccaccc taaaaaagaa gctcaaccag gacatgccgc cctaccggat tctcggggca
58561 tgcaatccgc ccctggccca tcgcgcattg acggcggagc cttccatcgg actgctgctg
58621 ccgtgcaatg tcgtggtgcg ccaggacgat gccggcaagg tgcaggtgga gttcatggac
58681 cccaacgccg tgctcgatct ggtcgacaaa ccagacatta ctcagctggc cggcgaggtg
58741 cgccagaagc tggagcgggt gatgcaggcg ctgtagcagg cagccaggca gaaagcacgc
58801 gaacgaggag cggggcgaga tggaaacagg tcgaggcgct tcaagcgtca tggcgggtgc
58861 agccgtcgcg atctcgttgc tcgtatcgcc cttcgccgtt ggccatgccc atgcggatgg
58921 aacaacaaga ttggcgcaag gcatgatgga tccaggaatg atgcgaccgg gaatgatggg
58981 tggcatgcac cgaaacatga tgcagcggat gatgggcggc gccctgccgc ccggcatcga
59041 tcccgcgctc ctgcccgagc cgcattcggc gggcgcgatg gtactgcagg attactgcac
59101 gcaatgccac aacctgccgg gcccgggcat gcataccgct gccgaatggc cgcgggtgct
59161 gggccggatg aacatgcgca tgcagatgat gggcggcggc atgatgggcg gaatgatggg
59221 ggtcatggct cccagccgcg cggagctgga aatcctgctc gcatatctgc aaaagcatgc
59281 ccagcagccg atcaacgccg ccgcgtaccc ggatctgggc atgcaggcgg gccaggcgtt
59341 cagcgccacc tgtcagcagt gccacaccct gccggacccg cggcagcaca gtgcgcagga
59401 gtggccggcg gtggtcgagc gcatgcgcgg ccatgaggcg gcgatgggca agatcgtccc
59461 ggacaagacg acgacggcgg agatcatcga ctttttgcgg caacacgcgc tcacgagtga
59521 tatccggcct ttgccgggaa aggccaggaa atgatgatgg attacgggat gggttggggc
59581 tgggggttcg gctggatcgg gatgatcctg ctgtggctgg tgccgatact gctcgtgctg
59641 gttgcgatca agtacctgtt cggcggcaaa ggccgaacgg gcatgggcgc ccgcggcgat
59701 gaccgggcgt tggctatatt ggaggaaaaa tatgcgcggg gagaaatcga tcgagaggag
59761 ttcttgcaga aacgcgacga cttgaagggg gattagtccc ggtcccagga tcgggttgtg
59821 cagttctcac cagtaaagga gatgcaaatg aacaccaagc aaaaactcgc cggcacgctt
59881 gccgcgctca ccctcctggg caccgccgcc atcagcgcgt cggcccaaca ggggcaaacc
59941 ggaccgtact acggctacgg atggtgtccc ggttgcggca tgggcccggg gatgatgggt
60001 atgggcccag gcatgatggg gcctggaatg atgggcggcc gtggaatgat ggggatggga
60061 ccgggcatga tgtggggcga ctgggacgac tgggacgacc gctacatggg ccgcggcatg
60121 atggggcccg gcatgatggg cggatatggc tacggccccg cgctcgacct cacggagcag
60181 cagcaggcca agatcgcgca gatccaggaa ggcttccgca agaagcagtg ggacctggcg
60241 gcgaagatgg acgccgagca ggcaaagctc aatgagatct actactccgg caagcgcgac
60301 cccgccgtga tcgacaacca gtacaagaaa atatacgacc tgcgccggca gatgatccag
60361 gaacaggtcg aggcgcagaa ccgcatggac gccgtactca ccaaggagca gaaggaacgc
60421 ctccgcggtt acagccccgg cgggatgatg cggtgaccgg ggactgcgct tgccgatgaa
60481 aacagcgatg ccggattggc tgcaccgaat cgtcgcccgc ctgctgctga tcactatggt
60541 ggtcagcgcg gcggggctga cgttcgggcg cgagattctg caagacgggc cgagcgggcc
60601 gaagtcgatc gttgccatgg caaacgcaga tcagcatgat ggccaattcg gcaatagtgg
60661 cggtgccacc aaggtatgcg actacagctg ctataacccg agtcatttcc tgggccagct
60721 gcccttgaaa tgaaactcct cccgaaacgc gcgttgtata tcttgctggc cgccctcggt
60781 accgtcgcgg cctacctgat ttaccaccat gcattgggca atcgtgcctt gcccgaaggg

60841 ctgattctgg cgaacggccg catcgaaggc gaccatgtca cggttgcaag caagttcgcg
60901 gggcgtattg cagaactgcg agtgcgcgag ggcgactcgg tgcaggtgaa tcagatcctg
60961 gccgtgctgg atgatgcgca gatcaaggcc agggttgccc aggcgcgcgc cgcggtcacc
61021 gtgctggatg cgcagatcaa ggctggcgag acggcgctcg tcgtgacgca aaaggaactg
61081 ccgctggcaa tcgccagtgc gcaggctgag gtgtcccgcg ccgctgccgc ggtcgccaag
61141 gcgaaggccg ccgaacttca ggcgcggcgg gacgcgacga gaatgcatga tcttctcacc
61201 cagaacttcg tgaatcgcca gcttgcagag cgggcggacc ttgcgttgag cgtggcctcc
61261 gctgagcatg caacggcgcg tcacgcggaa cttcaggcgc aagcgcagtt ctcccaagcg
61321 aagcttggcg cggaccggat cagggccaag gagtcagagc tggcggcgct ccgttcgcag
61381 cgcgagcagg ccagggcaag cctggccgaa gcagaaagcg tgctcgccga tcttgtcatc
61441 aaggcgccat cggcgggggt catcgtcacg cgcatccgcc aggccggcga tgtggtcggc
61501 gccggcgggc cgctgttcga cctggtgaat ctcgacacgc tctacctcaa ggtgtacgtg
61561 cccgagatcg agatcggcaa actgcgcctc aatctgccgg cgcgcatcta caccgacgct
61621 tttccagaca cgcctttcga tgcgaaggtc ggatacattt ccgcgcgcgc ggagttcacc
61681 cccaaggaag tccagacgcc ggatgagcgc gtcaagctca cctatgcagt gaagctctac
61741 ctggaacaga acccggagca caagctcacg cccgggctgc ctgccgatgc cgtgatccgc
61801 tggaaggtgg gtgtcccgtg ggccaagccg aattggtagg cgccgggcct gcacctttga
61861 caaatccggc cgccgacggc gagaaagtcg tccgcacacg cgcattcggc aagcgctacg
61921 gcgagaccgt cgccgtcgag gggtcgatc tggacatcgg ccgtggcgag attttcggcc
61981 tgatcggccc ggacggctgc ggcaagagca gcctcatgaa atccatcgcc ggggtgctga
62041 gcttcgagca ggggaaagtc gaggtgttcg gtgtcggcat cgacacggag gccgccgccg
62101 aacgcatcaa gcaccggctc ggtttcatgc cccagggcct gggcctcaac ctctatcccg
62161 acctctctgt ggaagagaac gtagactttt tcgcccgcct gcgcctggtg tcggaggacg
62221 agcttgtgtt acgcaagcag cggctgctcg gcatcacacg gctggacagg tttcgtgacc
62281 ggccgatgaa aaacctgtcg ggcggcatga agcagaagct ggggctcgtt tgcaccctga
62341 tccacgagcc cgagctgctg atcctcgatg agccgaccac cggggtggat ccggtatcgc
62401 ggcgcgattt ctgggccatc ctgtcggcac tggtcgcgga aaagggaacc accgtcatcg
62461 tttccactgc gtacatggac gaggcgagtc gctttcaccg catggcgctc atgtacgacg
62521 gccggatcat cgcccaggga tctccggacg agatccgttc gcttgctccc ggcagccagg
62581 tgctggtgtg gacaagtgcg cagcaggagg cgcttgccag gctcagggcg gggtttgccc
62641 aagtggaaac gctcgggcca tctctgtgcg tctatgcgga cgggctgacg ccggaggccg
62701 ctgcgatgcg ggttcggcag gtgctcgacg gcgtcgaagt gaatcggctt gaggccggcg
62761 aaccggacat ggaagatgta ttcatcgcac tgctgcgtct gcgccgaggc gtgtcgacca
62821 gggaggtacc gctcttgccg ggaggaaccg tcgcagctcc gcgcggcgag acggcggtcg
62881 cgatcgaggc gcgctcgctg acccgcgact tcggccgctt ccgggccgtg gatcatgcga
62941 gcttccgggt gtcccaaggc gaaatcttcg gtctgctggg cgcgaacggc gccggcaaga
63001 cgaccttgat caagatgctg acgggcctgc tgcggccaac cgacggcgat gggcgcgtgg
63061 cgggcgaaga catgcgggcg gccggatggc gtatcaagga acgcatcggc tacatgtcgc
63121 aggccttctc cctgtacgcc gatctcaccg tggccgagaa catcggcctc tatgccggca
63181 tctacggtct aggcacccga gaaagacggg agcgcaccgc atgggtgctc gagatggcgc
63241 agctcgccgg gtatgagaag aatccggccg gccgcctgcc gatgggcctg cgtcaaaggc
63301 tggcgctggg ctgcgccctg gtgcatcggc cacaggtgct gttcctcgac gagccgactt
63361 ccggggtcga tccgctgggg cggcgccagt tctgggacat cctgttcggc ctggcgcgcg
63421 gggaaggcgt ggcgatcctc gttaccacgc actacatgag cgaggcggag cactgcgacc
63481 gcctggcgct gatgtatgcg ggccggatcg tcgccgacgc cccgcccgaa gtgctcaagc
63541 gcgaagtgga gatgcaagcc ggggcggtgc tggccgtgac ctgcgccgcg cctgccgcag
63601 ccttggcgcg gctgtcggcc gccggctttg aaggcctgtc gctgcatggc aggcgcgtgc
63661 acctgttttc ctcgtcgccg gagcaggacc gggcgcgtat cgcggcggtc ctggccggtg
63721 cggaattcgg cgccgtcgag gtgtcggcgc gcgcgaccgg catggaagac gtcttcattt
63781 accgtgtcac cgaactggag cgcagcgagg tgcgggcgtg aacccgaggc gcgtcgcggc
63841 ggtcgcccac aaggaatggc gcgaggtgct gcgcgacaag ttgttcttta ttctcgcctt
63901 cgccgtcccg gccattttca tggtgttgtt cggctacggg ctgacgctcg acgtggagaa
63961 catccccttc gccgtgctgg attacgatcg atcgagcgcc agccgcgatt acgtgcaccg

64021 ctttgccggt tcgcgctact tcgatttcaa gggctacgtg caggacgagc gcgagatgga
64081 gcgcctcctc aaaggcaacc gcatacgcgc cgccatcgtc attcccgagc atttcggccg
64141 caatctttcg ggcgatcgca acgcggccgt gcagatcctc ctcgacggca cgataccctc
64201 gcgcgccgcg acggcgaaag gctatgtcgc cgccatcacg gctgcggcga acagcgacct
64261 cgtggccgcg catgccgccc gccggggcgg gctcagcctc gatcaggcaa gagaccttgc
64321 cgccccggtc agcctggagg tgcgttacct gtacaaccag gccgtgcgca gcatctggtc
64381 gctggccccc aagctcatca tcgtgatcct gcttttcgtg ccgcccatcc tgaccgccgt
64441 gggcgtggtc cgggaaaagg agacgggttc gatctacaac atctatgcct ccacggtgag
64501 ccgcggggag ttcctgtgcg gcaagctcgt tccctatgtc gcgatctcgg tggccaatat
64561 gatcgtactg tgggcgatgg ccgtctggct gttcggcgcg ccattcaagg gcaacccgct
64621 gttcttcttc ctggcctcga cagtgttcat cgtgtgcgcc acggccatcg gcctgttggt
64681 ctcgctcctc gtgcacactc aggtcgcggc cattttcctc actgccgtgc tgacgatgat
64741 gccggcgctc gactattccg gctttctcat tcccatccac tcgatggatg ccggcggtca
64801 gctgatcgcc cgctccctgc ccttcatgta tgccacccag atcatggaag gcagttttct
64861 caaaggcctc ggcttcggcg agcagtggcc cgaattgctg gtcctgatcg cctataccgc
64921 ggctttgctc gcggtcggct acgtcctgtt caggaagagg ccgtcgacat gagcgggcgc
64981 gggcgggcgt tgcggatatg gtggctcagg ctgggcgtcc tgacgctcaa ggagttcatc
65041 cagctcctgc gcgatcccgt gctgctgctc gtcgcggcct ttctcttcac cgtcaatatc
65101 tacatgcagg gatcgaccct gagcatgcag ctcaaggccg cgccgctgct ggtccacgac
65161 gcggatcaca gcgccgcttc gcgggagatc gtctatcggt tccgccaacc gtatttccgc
65221 tttgccgggg aaatacagga tccgcgcgaa ggcattcgac tgttggacga ggggcatgcc
65281 ctggccgtgc tcgacatccc gcccggtttc caggaagcac tgctggcggg caggcccgct
65341 gcactccagc ttcaggtgga tgcgacccct acggcacagg catttctcgc cgccagctat
65401 gccgggcgca tcgtcggcga attcgggcgc gagacggcgc tcgcgcgcac cggctcggcg
65461 gacggcggcg agacggcatt gccgctcatc cgcgaggagc cgcgagtctg gttcaacccc
65521 aaccaggaag accgttggtt cttccccatc tcggagctga tggaggcgat caccatcatg
65581 tccatcctgc tgccgggggc ggccatggtg cgggaaaagc agcgcggcac ggtggagcaa
65641 ttgctggttg cgccgctgtc gccgttccag atcatgttcc ccaaagtgat cgcgatgacg
65701 gtggtgatcc tcgttgggct cagcttctcg ctgtttgccg ttgccgcgcc gttgttccat
65761 gtgccgatgc gcggcagcct cgttttgttc tattgcgtta ccgcgctcta cgtgttcacg
65821 aacgccggat tgggcttgtt tgcagccacc atcgcgcgca atgtcgccca aatgggcttg
65881 ctcgccatcc tgatggtcgt acccctgatc ctggtttccg gaacctggac accgcccgag
65941 gcgatgccgg tctggatgag gataggcacc tacgtgtcgc cgatgcggca ctacatcgat
66001 gtgagctacg gcatcctttt caagggcgct ggcttggacc tgctttggga ctccgtggcg
66061 gcaattgctg tattgggcgg tgcggtattt ggcttcggcg tgtggcgctt ccggcgccag
66121 tttggttaat gggatttcca gcgtcggatg atcctgatta gtcgttgttg tgcagaacaa
66181 aagctttttc cacggtgaaa ctgccgacac ctttgtaatt ggccatcgtc cagtcgttcg
66241 cttccagaaa cgctgtcatt tcctggatca tgtgcgggct gccgcagagc atgacgcgat
66301 catgttccgg attggcggga gcaaggccaa gcttggaaaa aagcgcaccc gaacggaaca
66361 ggtccgtgcc gcgctctaag gttcagtagg ctggctagct accggcgtgg agagaaaccc
66421 gttggctggc tgattgaatg gcggactatt tcgtcagaat caattttccg tgacgtgtga
66481 tcctcaatac atagcgctcg ctctcgtact cgatcaccag cattcgcgcc ccttggaaca
66541 aggcatgcgc gggcagcacg cgtgcctcgg cgactgattg ctctcgaacg ggcttgggcg
66601 agacgggtct tgtgcagcga ttcaacatga tagcgatatt aaaaagaatg attcgcattg
66661 gcaaatgctg ccacagaatg agccgaatca cgcaacagca tcagcactcc gaagcagccc
66721 gcaattccct catggccacg gcgatgcccc gaccggacaa tctcgccatc gagcgagttt
66781 gacgtccagg agcgacaacc gatgcaccca accaaccatg tcccacccga gcgaatgaca
66841 gccgggcagc gccgagccga ggtcgcgtcc ttgctggcgc acggcctcgt tcgtctgcgc
66901 gacgccgcgt tcgcccagtc cgcaggctgc cctggggaga gcgagtttga gcttggcttc
66961 tccggccacc agcgccttca tacctacccc gtcaacaaca cccgcgagga ggccctatga
67021 aggcaagcac cgtcccaccc accccgccga gcgtcgtcgc caagatcgcc gggctcccgg
67081 atctctccat ggatgagatc aaggcactct ggcgccagct gttcggcaac gagaacccca
67141 cgcccaaccg ccagttcctg gagcggagga tcgcctacaa gctgcaggaa atcgagttcc

67201 gcaaggtcga tcccaacctg ctggatcgca acaagcggcg catcaagtcc ctgatggaga
67261 ccggcaagtc gcgcaagctc gaccgcgaca tccggctggt gccaggcacc gtgctgaccc
67321 gcgagtacca gggggtcgag caccgggtga cggtcgccca ggacggacag tacgagttcg
67381 agggcaggcg ctatccgagc ctgtccatga ttgcccgtga gatcaccggc acccgctggt
67441 ccgggcccct ctttttcggc gtgaaagcgc cagtcaagca gaagaattcg aataagcagg
67501 gaggccggcg atgagtgaag tcctcaagcg ccgcctgcgc tgcgccgtct acacccgcaa
67561 gtccaccgac gaggggctcg accaggagta caactccatc gacgcccagc gcgacgccgg
67621 ccacgcctat atcgccagcc agcgcgccga gggctggatt ccggtcgcgg acgattacga
67681 cgaccccgcg ttctctggcg gcaacatgga acgcccggcg ctcaagcggc tgctcgccga
67741 cattgaggcc ggcaagatcg acatcgtggt ggtctacaag atcgaccgcc tgacgcgatc
67801 gctgaccgac ttctcgcgga tgatcgacgt cttcgagcgc cacggcgtct ccttcgtctc
67861 ggtcacccag cagttcaaca ccaccacatc gatggggcgg ttgatgctga atatcctgct
67921 atccttcgcc cagttcgagc gcgaggtgac cggcgagcgc atccgcgaca agatcgcggc
67981 cagcaagcgc aaggggatgt ggatgggcgg cgtgccgcca ctcggctacg acgtcgacaa
68041 ccggcggctg gtgcccaacg agcgcgaggc caaggtcgtg cgccacatct tccagcgctt
68101 cgtcgaactc ggctcctcga cgctactcgt gaaggagctg cgcatagacg gcgtgacctc
68161 caaggcctgg accacccagg acgggcgggt gcgtgagggc aagcccatcg acaagagcct
68221 cgtctacaag atcctcaaca accgggtcta cctgggcgag atccggcatc gtgatcagtg
68281 gtattccggc gagcacccgc ccattgtcga gcgcaggctg tgggacgccg tccaggcgat
68341 cctcgaccag aacccgcggg tgcggggcaa caacacccgc gcaagggtgc cgttcctgct
68401 caaagggatc gtcgtgggaa tcgatggccg ggcgctcact ccgtggttca cgcgcaagaa
68461 gaacggccgc atctaccgct actacctgcc ggacgtcttg atcagctgca gaacccgtcc
68521 aacttcagat gaggcagggg tcgtgtcgat ggacggaaag agttctttca gcatgtcgcc
68581 gaaggtcagt atctcaacgg ggttgccgcc gagattctcg cggaacgtcg cgttgtcctg
68641 ccacgccgct cggctgccca cgactttcgt gacggccgtg atgtaggtga acgagaacga
68701 acccgtcagt tcggcgaccg tcgtcctgaa cgctgcggcc cacttctccc tggtcagctc
68761 gcgaaacccg cgccaagcct cacgccccga gaccaccttg ttcttggcga tggcatcgat
68821 ccagtattcg ggacgaaagc caccctgcca gctcttgcag ctcactacca tcacccgacg
68881 cgcgccgtcg agtcgcgggt ggatgccgat cacgtcgatg tcgctatgca ctgcatcctg
68941 gcgggtgtcg tactccgcgt gatcgccggc gggccggaac ttgatgttgt gcctggtgaa
69001 gtaccctttg tgctgcaggt actcgtccac catctgttcg aggatgtcct ctttcatgcc
69061 tgtccgccct cgcgatcgat ccgttggctg gcaaatccct cgaactcagg ccgaaggcgg
69121 cactgcagaa atgcctcgag gaggttgagg acgtggatgc cgagcggcgc atcgaggatg
69181 aagttcaccc gatcggtgtg ctccctgatc cagccgggga cgtttgtctc gttcaaacca
69241 acgacgccga tgcgctcgcc gtgtttgatc aacgaggctg ccagagtgct ctttgcgctg
69301 ccagcgttgt agtgttgagc cgtgtagcga gcagcgctgt tggggccgac cttcccgacc
69361 ctcagcacat gaggcccaaa cgagaagacg tacaccgcca tggtgccggg cggtaatccc
69421 ttggggggca cgtgcggcgc agggcgccgc tcgatcgcga tggcgtcatg cgtcaacgag
69481 acgccagcca tgccagcgac acggcgaaaa tcctccaaga gcagatcagg attccaggtc
69541 atcggcccga ttcctcgctg ccggacgccc acccgctacc ggcaagccat gtttgcggaa
69601 aagcggcagc ttgccggagc gcgtccagcg ggggcgacag gaatacacgc agaacggcaa
69661 cgacctcgga gatctgaagc tcgaccaaag cattcttcct caagaatgcc tgccactggg
69721 tctgcttctg ccgatcctgt gcgaattcgt cgctcaggcc gaaagggacg ccatccggca
69781 gcggcgtcct gcggcgctcg aaggtggcgt ggatcgcctt gcacaggagc gcgccgtcga
69841 agtcagtgta gcgcgacaag atccagagat cgaagtaatc cttcatgcgg ctgttggcga
69901 taccgagcga caccagcgcc tccagcttct cggcgatgac cgtgtagcgg gggtaggcac
69961 gcaacttcgg ggctggcatc tccgacagca tcaccgggta gtcgacggcc tcggggccgg
70021 gcgtcacggc gtcgccgaaa cccacgtcga cctgcacgtg gcatcgggca ccgtccaaca
70081 ggccgatcag cgtgacccgg acaccggagt agttggcttc cttgcggatc tcctccgcgt
70141 gtaccgagtc aggctggaag cgtatgccgt cgtccagttc caccgtacag acgtcccgaa
70201 acgccgcctc gacatgcgga atctcggccg agccgaagcc cagcagatcg gcgtcgcgtg
70261 tcggacgatg cgggatgtcg aaccacagat cgaacaacag cgcacctttc agcaggaact
70321 ggtcggcgtg gggggagatg ctgagccggt aaagcaggcg ctcaagcgca tagcgggtca

70381 agatcagatt gaagtcttgc cgggtctcgc gggcgcggtt gagcaggcgg gcgcgcaccg
70441 aggcggccat gttgcgctgg ttcatgacag gctttccatg taggggcgca tcacattggc
70501 cacacggcac agcgcagcaa aacgccaaag ttcatccatg ctgacgcgct tggcacgcca
70561 ggactcctgt agcgcctcca gcgcgacatc aaggccgatc ttgttgcgga acttgaagca
70621 gtcagccacg gtccgggcga cgttggtcac gcgcaccggc acaccatcga tgaggtgctc
70681 ctcgaccccc tccgtcagcg cagcgccaga gaagcgcacg atgcgcagcg ggggatagtc
70741 catctttggc gcgcgtgcct tgttggggat tgccagccag acctcgaacg gcgactgtgt
70801 ggtgagttca tgtaggcgca gtgccgacag caggcagacg atggcttgcg ggtgcttgcg
70861 tgccacctcg gcgagtgcgc cgtgttccga gacggtccgg tccggaattg cgtacaggcc
70921 ccgtcccacg cgctggagca gcccttgccg caccagccgc gtgagggcga ccgtgggcag
70981 cccacgctca ttgagatcgc gtgggcgaat aaggccgcgc tgggcggcaa gatccagaat
71041 gctctggtgc gaactgtcca tgtcgtcata atgttgcgaa acgtcggtag ctgtcaagaa
71101 ctaccgacaa aacacaactg atatggtttc ctttgcctgc cgatgccgca atccaaccgt
71161 ttgattcgtc agcgcgtaac ccattgatct gtatgggtcc acgttgcggc ctgtgcggac
71221 ttcggccctt cgtcggggag cgaggccgga gagaaaaacg gcccggagag agcgaaatgg
71281 ggcccgaggc aggcgagacg gccggggaag gaagtccgca ggcgtccgcg agagtccccg
71341 cgaacacgcg ggagtgcgca agaaaaaagg ccaaccgaga acggttgacc tttatatatt
71401 ggtggcc

//

LOCUS   KN2-R2   36045 bp   DNA   linear   PHG 16-FEB-2025
DEFINITION KN2-R2.
ACCESSION   KN2-R2
VERSION   KN2-R2
KEYWORDS   .
SOURCE   .
  ORGANISM .
            .
FEATURES   Location/Qualifiers
     CDS   complement(3..746)
                     /ID="YNAZFPKL_CDS_0001"
                     /transl_table=11
                     /phrog="2045"
                     /top_hit="No_MMseqs_PHROG_hit"
                     /locus_tag="YNAZFPKL_CDS_0001"
                     /function="unknown function"
                     /product="hypothetical protein"
                     /source="PHANOTATE_1.5.1"
                     /score="-1522.2877461955727"
                     /phase="0"
                     /translation="MATQSTIEWTEQTWNPTTGCTKISPGCKNCYAEVMSRRLQAMGAP
                     GYEDGFNLASHPDRLAQPLQRKKPTVYFVNSMSDLFHEDIPDEFLDRVFSVVRQTPQHT
                     YQILTKRADRLPVYFRDRDCPRNAWLGVSVEDRKYGVPRIDHLRGVNAHIRFLSVEPLL
                     EDLGTLDLRGIHWVIVGGESGHKARPMREEWVANVKEQADAAGAAFFFKQWGGWGADGV
                     KRHKKANGRVFRGRTWDAYPEVQQPA"
     CDS   complement(760..1158)
                     /ID="YNAZFPKL_CDS_0002"
                     /transl_table=11
                     /phrog="8"
                     /top_hit="No_MMseqs_PHROG_hit"
                     /locus_tag="YNAZFPKL_CDS_0002"
                     /function="transcription regulation"
                     /product="transcriptional regulator"
                     /source="PHANOTATE_1.5.1"
                     /score="-47.90580707896214"
                     /phase="0"
                     /translation="MHIAHFSRYNMRNVHHCRSPFVAITTVAELVRAARSGRSQKEFAR
                     ELGVRQSSVSRYESGRANPPVRVIEHCMRLVHSTGHEHAPTADELAAKVRTELADSDLG
                     EIRLLLARLIDTLASEHAQARVMSPSSS"
     CDS   complement(1265..3436)
                     /ID="YNAZFPKL_CDS_0003"
                     /transl_table=11
                     /phrog="No_PHROGs_HMM"
                     /top_hit="No_MMseqs_PHROG_hit"
                     /locus_tag="YNAZFPKL_CDS_0003"
                     /function="unknown function"
                     /product="hypothetical protein"
                     /source="PHANOTATE_1.5.1"
                     /score="-17957529.362339005"
                     /phase="0"
                     /translation="MAGTIEFPGYPFDLIRLQVRAMGGLYCTPISALSATGWGEPFKDA
                     KGQPINPPWGTPGGAYPQVKLKPDGDGVMHSPEFPDPHAALAGGTLAVEWEGRTYTIQS
                     SGTVWLPTGYCYNTPYPESGNVVFGFEPGALCRDGRVAVNAFTRSHATLDLSRCIGIGV

CNGHLILITAHPDNEGKLLVAQINGARADRIAEVYAGHPTAKLRFFFREDGIRASAVAT
MARESVVLHIDLVRAGDELGRTIPGAGVESASLAVESLGNAPTKTIDQGATQVGGELIP
DPNGGPATTTPLTSATSFYEYDIPETTRVIAIGYVGNAEHRLAVQASSSYRRESSYIAS
YGRDSRGIGTDTTSSQSRTTGRENISFLIDGIPVAAFSTRDDSAFSKATETTATLITVP
GGEGIGKTKQDSNGDWTITYPEGGSSSNAFVAKFRDEITQDDWIWDPVFNSMPPLNLLD
PPGAYVLPSHLGVSPPEFSETASQSHTSHSEYVQLLDVDFTTGSLYYVRWTEDFQWGGA
GPPPGGVKSNYRQQICRNAAVLAERWFSADPSGGLYQTAPGLRFGWYSGKVDSADIGGP
HNRGTFELVHPLNLRGAYTRPTNEFPYEAGFGGLYIRDWVVHNASGPSHHMTYDLTLQS
ALDAQGNWACAHYIPWCITDADSVNVGWFPNHRYNDPSWNANGATVRYPDSDGAKVMLY
RSAEGVMKPLEAVVQQPGVDRFFLGLGTI"

CDS complement(3439..3798)
/ID="YNAZFPKL_CDS_0004"
/transl_table=11
/phrog="7250"
/top_hit="No_MMseqs_PHROG_hit"
/locus_tag="YNAZFPKL_CDS_0004"
/function="unknown function"
/product="hypothetical protein"
/source="PHANOTATE_1.5.1"
/score="-13.258199479806684"
/phase="0"
/translation="MTVQKFFVNDLNAIVDAVRTTSDALPEPLAKPGIGNAVGIGTRRK
TAGIASPLTEKTAQNAKGESWADREYYPARDLKTAGGQFVVKYAAIKTLKFKDGNGDVV
EFHFAEPDPDKPLGV"

CDS complement(3868..4293)
/ID="YNAZFPKL_CDS_0005"
/transl_table=11
/phrog="No_PHROGs_HMM"
/top_hit="No_MMseqs_PHROG_hit"
/locus_tag="YNAZFPKL_CDS_0005"
/function="unknown function"
/product="hypothetical protein"
/source="PHANOTATE_1.5.1"
/score="-38.9239194118681"
/phase="0"
/translation="MPTFALYTDAALTQLLAGNLVATQNADGSTPPVVFTLYLGSPTAN
RKIQANANPGVDPIQATVVDANTGSGHSASEVKLAATQAGLAAAVGGSALDLGTEILSG
AANAKPIWIEVNDATHAVGTATELAVVIANVRETATA"

CDS complement(4297..6729)
/ID="YNAZFPKL_CDS_0006"
/transl_table=11
/phrog="5842"
/top_hit="No_MMseqs_PHROG_hit"
/locus_tag="YNAZFPKL_CDS_0006"
/function="unknown function"
/product="hypothetical protein"
/source="PHANOTATE_1.5.1"
/score="-10823229.769445138"
/phase="0"
/translation="MTLPVGGVTGIGGVASAAATAAPAAEVYSIAASIGQPIYVEASLV
AAIEQTTLDVYAIAASLHSQVYARLDLSAGVAQAVVVFEVFDAIAPIRQPIYAVTGFEA
FVEQVGFDGYAVAAPLEQRLWARFNLTAPVEQSQFRLFDASVVIEQRLRAEAYRLQAPV
THRVKAVFTLSAGLAQTVADPVFDLDATVEQAVYAFIANLTASLSQTQYDAQAVGGGAF

VGQSQAASADITAWAVKAVIGNAAGDGEPVPPEAGAVMGALLGRMTGTLTVDGEEGAAR
IAEFTVLPAPGPIHIHTLTGLPITLFLVTAGVELPIFQGILDVPGWDPVQGLLHLSCTD
NLQSRFDGLKRKEIAGIIGGRWSPYVFDKHADEWQYVLDRLSTLPVSYDLDLNLQGRIT
PWQARATADFTFRPDAVIENSVRIDMASARQLVNAIDVHMDYRYERLMQREYRYHWELS
LDQLIDQGSTAPTPETVMQAIDGTGWAVLAEGGTPLLRMTPLPDPQWLHGVMYLSDCPR
YSLAGGVSAILAQRWTQTVTEQWHVTVDAPDSIRVIGKRHGTTTANFDAQSDKDPRYAH
WDRTTEQTVKLELHDGAPIGGYACDSWIPYHPLQVRTYTVPVPAGAQRFATLDPIANPL
GDLYYDLDDGAADGRAGLSNAYMTGIERARRDILSSHRQTTVTFDTLIAPLIDRTHTVR
MASPRLTAKGKVRRVTHSIDFDHGAAITTLALAVSKSYGVGTVDTGFDFPAPAKPPATQ
APLKHPLATPAIGYDPANGEFTITVPGVEQQHIDATTTAPADHLPIAIPDDELILEA"
CDS complement(6729..7469)
/ID="YNAZFPKL_CDS_0007"
/transl_table=11
/phrog="No_PHROGs_HMM"
/top_hit="No_MMseqs_PHROG_hit"
/locus_tag="YNAZFPKL_CDS_0007"
/function="unknown function"
/product="hypothetical protein"
/source="PHANOTATE_1.5.1"
/score="-115.13160709329497"
/phase="0"
/translation="MLKLAGLDGVKTVLTAALGAADTWLMAAAAQPPWITPPDPLGEYH
YYLTLIDAETPTKWERVKVTARNGAGPYQLTIERNIASSSGGAQAFAAGALLQWSPGAK
EIDRWRMVLSASMTGTNALTSTRFLAPFSGSTTGSMAAAFWVNRTGKTLRLSKLVAVLS
TSSTFSGDSIVLSVETGATAPATPSNLQTQVAPGNYGFNTDGGYVLDVAPDAPVCFMLS
RANPGAAVSGAFQLAHVSVLAEEL"
CDS complement(7471..7818)
/ID="YNAZFPKL_CDS_0008"
/transl_table=11
/phrog="5314"
/top_hit="No_MMseqs_PHROG_hit"
/locus_tag="YNAZFPKL_CDS_0008"
/function="unknown function"
/product="hypothetical protein"
/source="PHANOTATE_1.5.1"
/score="-9.13579078768431"
/phase="0"
/translation="MWSGRSVKQRTWRKLATEISGGGWVPPGLSALDYSQPQVLACASP
LAIVGGGTPPVPVRTEGDFATVTLNVDGVDITYYYPKITVYCDPPEQQLDGTTGNYSWR
LSAEMVDPIGG"
CDS complement(7929..8081)
/ID="YNAZFPKL_CDS_0009"
/transl_table=11
/phrog="No_PHROGs_HMM"
/top_hit="No_MMseqs_PHROG_hit"
/locus_tag="YNAZFPKL_CDS_0009"
/function="unknown function"
/product="hypothetical protein"
/source="PHANOTATE_1.5.1"
/score="-0.0922935154294044"
/phase="0"
/translation="LTLSGCCTLEPRVTLVPAPWRCPARAADLLAHIDEAGLVLRCDEP
EPDER"

CDS complement(8078..8176)
/ID="YNAZFPKL_CDS_0010"
/transl_table=11
/phrog="No_PHROGs_HMM"
/top_hit="No_MMseqs_PHROG_hit"
/locus_tag="YNAZFPKL_CDS_0010"
/function="unknown function"
/product="hypothetical protein"
/source="PHANOTATE_1.5.1"
/score="-3.511396783746092"
/phase="0"
/translation="MIVLTRGEVWCAAAVLVSAGVVSGLWVALCAL"
CDS complement(8176..8574)
/ID="YNAZFPKL_CDS_0011"
/transl_table=11
/phrog="938"
/top_hit="No_MMseqs_PHROG_hit"
/locus_tag="YNAZFPKL_CDS_0011"
/function="lysis"
/product="endolysin"
/source="PHANOTATE_1.5.1"
/score="-47.71889506712256"
/phase="0"
/translation="MNHIPARTAWAEARGEGEAGMQGVMNVIFRRAQRPGWWGRTPDEV
CLNPRQFSCWNEGDPNKAKAEAVDARDRAFSIALKLESQALKGTLPDITGEATHYFDDS
LLKKPPHWARAMVKTAVIGRLHFFKERD"
CDS complement(8571..8837)
/ID="YNAZFPKL_CDS_0012"
/transl_table=11
/phrog="No_PHROGs_HMM"
/top_hit="No_MMseqs_PHROG_hit"
/locus_tag="YNAZFPKL_CDS_0012"
/function="unknown function"
/product="hypothetical protein"
/source="PHANOTATE_1.5.1"
/score="-23.76029971290134"
/phase="0"
/translation="MFEKVKYLYECMQAGKALQDAGVWSRRADLIAKLTALLMAATGLA
EAFGYDLRLSGTDVAAVAQGLGVLAVTLVNVLHVASNKEAGKR"
CDS complement(8880..9155)
/ID="YNAZFPKL_CDS_0013"
/transl_table=11
/phrog="No_PHROGs_HMM"
/top_hit="No_MMseqs_PHROG_hit"
/locus_tag="YNAZFPKL_CDS_0013"
/function="unknown function"
/product="hypothetical protein"
/source="PHANOTATE_1.5.1"
/score="-4.127695231723194"
/phase="0"
/translation="VNPILRWALWFVVQAVSGITKEQWGLVQNKVAELETRTIAGLVDK
TTLNDIKKKEAAAYIGTFVSGVRMNVVHFLIEVALWFVRSFGVKAP"
CDS complement(9184..12222)

/ID="YNAZFPKL_CDS_0014"
/transl_table=11
/phrog="339"
/top_hit="No_MMseqs_PHROG_hit"
/locus_tag="YNAZFPKL_CDS_0014"
/function="tail"
/product="tail length tape measure protein"
/source="PHANOTATE_1.5.1"
/score="-429894243.2574514"
/phase="0"
/translation="MTTFKTVIEIAANTAAAEKGLGNVAAQVDRLTGSLRRIGQYTVGA
FGAAEALQAARDLGKLSDQYRNLEGRVKLAAGSQNQFTEAQRALFAIAQNNAQAMTGVS
QLYARIAKGAGEMGVSQRQVLSVIDSVAKSFRISGASAEEASSATLQFSQALAAGVLRG
DEFNSIMEQSPRLAQAIADGLGVPIGKLRTLAEAGELTTQKVVEALQKAKASIDRDAAG
LPDTIEQAVVRWDNAALKFVGTSSEITRAAEVIADAINGAAGNIDTIATGVEIAGSVLV
AVLAGKGTAAVAAFAGSQARLVQAHLAATAAARDHALNEAYNARATFAAAEAAVANASG
MARLALVETQLVPASQRLAVAQAEVAASAGPLKIALGGLAAFLGGPVGIAVTLLTAVSA
WHLFGSTAESELERVIRKRRELAKATGQDTRGKSEAELSLLQDEAAVKKQEAVVARLTA
RYKEVGQAADQAFMGGKIGRELAGETALLQEMRAELGKRRQAAADSGNAVKAKEKEVQK
SFEDTRNALKDATAGIEDAYQRRTAAIDALEKLAETRIQAQPIEADAAPPIVYAQNGVQ
ATQARSELEQARQLHALTLQAEGARLKAVRDFAAQRLGLVDQVYAREIAKFKDGEARKT
VLERESLEARRAIYADLESAYTATIDKLIGQEQRLLESARAAAQERRNIEQSTARAVAG
IEQAALTPLEASRAQYAQLQQLLAQQRQELANGNLELSRQAGEQAAGLAESLGQQAAAS
LREQRLNALKGEQTPADPGQPQFGTEDLQRLIDARRQAGQALAEAAARQEQADTQAAAA
VSQKIQETLQNLQSVQAEISDIDQQLTKGFALRVDVDPASLQTLQSRLAELLKPETKTI
SVVMQQQGDGSDTGANGADATLPGFRRGGWIKGYGGGDRIPALLEEGEFVLRKEAVRKL
GLDKLYALNNLSLPRFSIGGYVAQAVPAFDASLARASDGGQPVHIHLPGVPGSFALNGD
PQVVAALKREVARAALKHGRIVR"
CDS complement(12227..12322)
/ID="YNAZFPKL_CDS_0015"
/transl_table=11
/phrog="No_PHROGs_HMM"
/top_hit="No_MMseqs_PHROG_hit"
/locus_tag="YNAZFPKL_CDS_0015"
/function="unknown function"
/product="hypothetical protein"
/source="PHANOTATE_1.5.1"
/score="-1.627399285056767"
/phase="0"
/translation="MTALALIERGHAGVWHYPWRLFLQARLMRRR"
CDS complement(12334..12693)
/ID="YNAZFPKL_CDS_0016"
/transl_table=11
/phrog="7608"
/top_hit="No_MMseqs_PHROG_hit"
/locus_tag="YNAZFPKL_CDS_0016"
/function="unknown function"
/product="hypothetical protein"
/source="PHANOTATE_1.5.1"
/score="-8.683130392251906"
/phase="0"
/translation="MAELDTTNPKGLRAERTIEAGGRKIVVRELTVGEVRAWLKDANAE
LDRNDLVALALFADITLDDLTRFSDLSRAELDAMLPSELDKVREAAKSLNPHFFGLRER

LANAAQVAATAPPAT"
CDS complement(12700..13203)
/ID="YNAZFPKL_CDS_0017"
/transl_table=11
/phrog="807"
/top_hit="No_MMseqs_PHROG_hit"
/locus_tag="YNAZFPKL_CDS_0017"
/function="tail"
/product="major tail protein with Ig-like domain"
/source="PHANOTATE_1.5.1"
/score="-154.74088396739694"
/phase="0"
/translation="MTTTAMLLTGTIKGAVGAGTPVDLGNAALDLSIEEDTKELADYQN
PGGGSIASLSRIKSVTLKLKLWSISKENLALATRGTVSGNSIEALTQTAEDWHITFDGV
NEVNGDAVTYDFYKVKFSPASSLPGPGTEDFAVLELTGKVLKDTSKTGAGVSQYFKATI
TPAV"
CDS complement(13263..13700)
/ID="YNAZFPKL_CDS_0018"
/transl_table=11
/phrog="209"
/top_hit="No_MMseqs_PHROG_hit"
/locus_tag="YNAZFPKL_CDS_0018"
/function="head and packaging"
/product="portal protein"
/source="PHANOTATE_1.5.1"
/score="-9.893158046996266"
/phase="0"
/translation="MITSMFEIEPALIRRLRETLPEFVTVDGVGVLAGVQNLEPLCPAA
LVLPLGFAGPVGTPPPNVFLAERQRWQVTVCVAHTPPASTVVTGGAYVLRILRALEHWS
PQPGWGRLKHVGLDDPWFDLGHVEFSLVFEVRPLPLDTGTP"
CDS complement(13697..14209)
/ID="YNAZFPKL_CDS_0019"
/transl_table=11
/phrog="27"
/top_hit="No_MMseqs_PHROG_hit"
/locus_tag="YNAZFPKL_CDS_0019"
/function="connector"
/product="tail completion or Neck1 protein"
/source="PHANOTATE_1.5.1"
/score="-14.771491113108244"
/phase="0"
/translation="MSIGLRVRLHGDTAQDLRRLPAVLRTASRRAVGQTARETREAMVR
AIGATYGIPLRALRSRRVQAYLRLAGLRGRVWTGHAPIQAAYVGRLRQEDWGSSAGAYL
FPGSFVARMQSGHRGIFHRVGHRSLPVLEDVVALPKVPALAEVLATRATLRLAALIREM
TQDALPR"
CDS complement(14220..14546)
/ID="YNAZFPKL_CDS_0020"
/transl_table=11
/phrog="785"
/top_hit="No_MMseqs_PHROG_hit"
/locus_tag="YNAZFPKL_CDS_0020"
/function="lysis"
/product="holin"

/source="PHANOTATE_1.5.1"
/score="-8.207057019328929"
/phase="0"
/translation="MNARPKQLLLDSPHGNGDDLRLALQIILVTIWSGLVAQLNRWRRH
PRPWRLCLWCLLVDVVSCSLVGFSVWLLAEREGLGYYESLWAAIVAGHFGARWFGLLIH
SSRG"
CDS complement(14543..14851)
/ID="YNAZFPKL_CDS_0021"
/transl_table=11
/phrog="82"
/top_hit="No_MMseqs_PHROG_hit"
/locus_tag="YNAZFPKL_CDS_0021"
/function="connector"
/product="head closure Hc1"
/source="PHANOTATE_1.5.1"
/score="-5.05564575198088"
/phase="0"
/translation="MDLEAVNAVCLSTFGEPVILWPDRTEPPPVHLTGIVSVPAGLERP
GPMGAPRPEADPRLTVRSADLPAIHMGDPLKVRGVPYSIASRIDRLDGLTTLILRPR"
CDS complement(14854..15855)
/ID="YNAZFPKL_CDS_0022"
/transl_table=11
/phrog="29"
/top_hit="No_MMseqs_PHROG_hit"
/locus_tag="YNAZFPKL_CDS_0022"
/function="head and packaging"
/product="major head protein"
/source="PHANOTATE_1.5.1"
/score="-2754.074110759066"
/phase="0"
/translation="MQNPFHNPAFSMAALTAAINIIPNRYGRLEDLNLMPVKPVRQRQI
LVEERNGVLNLLPTLPPGAPGTVGVRGKRTLRSFVIPHIPHDDVVLPEEVQGIRAFGSE
TELEAVAGVMARHLETMRNKHAITLEHLRMGALKGVILDADGSVLYDLYDEFDIPPKTV
SFQLGTATTDVKARCMEVLAHIEDSLLGEFMTDVHCLCSPEFFAALTGHKDVKTAFTHW
QQGAILINDVRRGFTFGGITFEEYRGRATDVHGVTRRFIAAGEAHAFPLGTVDTFATYV
APADFNETVNTLGQPLYAKQAPRQFDRGTDLHTQSNPLPMCHRPGLLIKLTV"
CDS complement(15858..16235)
/ID="YNAZFPKL_CDS_0023"
/transl_table=11
/phrog="49"
/top_hit="No_MMseqs_PHROG_hit"
/locus_tag="YNAZFPKL_CDS_0023"
/function="head and packaging"
/product="head decoration"
/source="PHANOTATE_1.5.1"
/score="-7.712742892559039"
/phase="0"
/translation="MPKLREPKNLGDLLKYEAPNRYSRDLAPVALGQKLALGAVVAREP
AGLRLQALDPAATDASAQAVGVLIEAVDATAAEVPQALLLARHAIVSDLGLVWPAGITP
AQKATAIVQLQALGILVRTGA"
CDS complement(16239..17570)
/ID="YNAZFPKL_CDS_0024"
/transl_table=11

/phrog="53"
/top_hit="No_MMseqs_PHROG_hit"
/locus_tag="YNAZFPKL_CDS_0024"
/function="head and packaging"
/product="head maturation protease"
/source="PHANOTATE_1.5.1"
/score="-1841.9666786793025"
/phase="0"
/translation="MTQPLIHLASRLYGTPLLIARPKLEVILAVLGSRIGLPEAATAVP
APMSRTAAPALPGIAVIPIHGTLVRRTLELEAHSGLTSYAEIGARLDAALRDPEVSGIL
LDLDSPGGESGGVFELAAKIRAGTSHKPIWAHANDTAFSAAYAIAAGASRVTLAQTGGA
GSIGVIALHVDQSVKDAREGLTYSALYAGHHKNDLNPHAPLSPQAAAALQAEVDRLYGI
FVAQVAGFRGLPETAVRATEAGLFFGEDAVSAGLADGVLGFDAVLNKFADAIRTRQRLI
SPQARKAAVMSPLPVSLLHSESVMHDHAPGPLDDEAADESRKDADAAGNLTPHAAPSPQ
NDLAAATPVIGEPTAQSLATEAHHAGRSEAQAIAELCLIAGAAPRTAEFLAAGLSEAQV
RRVLLAARAETPEIASRIQVDAGTSVRPEASPVVAAVQKLIARS"
CDS complement(17567..19039)
/ID="YNAZFPKL_CDS_0025"
/transl_table=11
/phrog="21"
/top_hit="No_MMseqs_PHROG_hit"
/locus_tag="YNAZFPKL_CDS_0025"
/function="head and packaging"
/product="portal protein"
/source="PHANOTATE_1.5.1"
/score="-11620.54281132383"
/phase="0"
/translation="MGWWQRLRFGLFGGPTPTYDGIGGGRRALAWQVANPGAVAALAAS
QEELRAKSRDQVRRNVWAAAGVEAYVANAIGTGIKPQSMAADVAVRPAIQTLWTHWGDD
ADAAGLTDVYGLQALACRALVEGGEALVRLRYRRPEDGLSVGLQLQVLEPEHLPAALNR
ELSSGNVIRAGIEFDKLGRRVAYHLYRSHPEDGALAPMSGAGGLDTVRVDAREVIHLFR
PLRPGQIRGEPWLARALVKLHELDQYDDAELVRKKTAAMFAGFITRTAPEDPLMGEGPA
DAQGVALAGLEPGTLQLLEPGEDVRFSQPADVGASYAEFLRMQFRAVAAAMGITYEMLT
GDLTQVNYSSIRAGLLEFRRRCEAIQHGVIVFQLCRPVWRAWMTQAVLEGALDLPGFSR
RERDYLAVKWIPQGWQWVDPKKEFDALQTAIRAGLLSRSEAISAFGYDAEDIDREIAAD
NARADALGLVFDSDPRHDRAAAPPTSTRANAP"
CDS complement(19039..19242)
/ID="YNAZFPKL_CDS_0026"
/transl_table=11
/phrog="2545"
/top_hit="No_MMseqs_PHROG_hit"
/locus_tag="YNAZFPKL_CDS_0026"
/function="connector"
/product="head-tail adaptor Ad1"
/source="PHANOTATE_1.5.1"
/score="-3.979268233045688"
/phase="0"
/translation="MSFTPEHLQALEAAMARGERRVTFQDRSVEYRSVEELQTAIREVK
RGLAAQGQPVARQIRITTNKAT"
CDS complement(19293..21260)
/ID="YNAZFPKL_CDS_0027"
/transl_table=11
/phrog="15"

/top_hit="No_MMseqs_PHROG_hit"
/locus_tag="YNAZFPKL_CDS_0027"
/function="head and packaging"
/product="terminase large subunit"
/source="PHANOTATE_1.5.1"
/score="-195380.28454463813"
/phase="0"
/translation="MLREDEGLEAIAHAWRDGLTPDPLLTVSEWADRHRVLSSKASAEP
GRWRTARTPYLREIMDCLSPASPVERVVFMKGAQLGATEMGSNWIGFCVHHAPGPMMAV
WPTVEMAKRNSRQRIDPLIEESPALAGLIAPARSRDSGNTILAKEFRGGVLVMTGANSA
VGLRSMPVRYLFLDEVDGYPPDVEGEGDAIALAEARTRTFARRKIFIVSTPTIAGASAI
EREYEASDQRRYFVPCPHCGHFQWLRFEQLRWAREQPETVAYVCEGCERPIPEHHKGDM
LAQGEWRAMAPEHGAKTAGFHLSSLYSPLGWRSWRDIAAAWDSAVHPVSGGPSAIKTFK
NTELGETWVEDGETPDWQRLLERREDYPIGKVPQGGLLLVGGADVQKDRIEVSVWAFGR
GKSAWLVEHRVLMGDTARDAVWHALATLVGDTWTHASGARMPLARLALDTGYATQEAYA
FVRRLKDPRIMAVKGVPRGAALIGTPTAVDVTQAGKKLRRGIKVFSVAVGIAKLEFYNA
LRKTAEVGEDGTTLVYPPGYVHLPQMDAEYLQQLCAEQLVTRRDRHGFARREWQKLRER
NEALDCYVYARAAAAAAGLDRFEERHWRALEDQLGVGPPPDPEAPLQPIHPETPTDPGG
RSVSGPGRSSRRVIRSRWLS"
CDS complement(21261..21788)
/ID="YNAZFPKL_CDS_0028"
/transl_table=11
/phrog="57"
/top_hit="No_MMseqs_PHROG_hit"
/locus_tag="YNAZFPKL_CDS_0028"
/function="head and packaging"
/product="terminase small subunit"
/source="PHANOTATE_1.5.1"
/score="-20.232182720668796"
/phase="0"
/translation="MGLSIRAYARHRGVTDTAVHKAIRTGRITPEADGSIDPDKADREW
ARNSDTPKAGTRQPAVRVAMPDASHGGGPTLPGGGASLLQARTVNEVVKAQTNKVRLAK
LKGDLVDRAAAIAQVFTLARAERDAWLNWPARISAPLAAKLGVDPHTLHIALETAVREH
LQELGELRPRVD"
CDS complement(21895..22233)
/ID="YNAZFPKL_CDS_0029"
/transl_table=11
/phrog="No_PHROGs_HMM"
/top_hit="No_MMseqs_PHROG_hit"
/locus_tag="YNAZFPKL_CDS_0029"
/function="unknown function"
/product="hypothetical protein"
/source="PHANOTATE_1.5.1"
/score="-11.073450259004668"
/phase="0"
/translation="MRPAVCSIAWLRIRLWVSVRGSVFMWVLLGVGLLSGVIDASQPLE
IPPEPPSAKPICSSIGLDPASGLDPCLVRRLQRGFEAPDRRVVEIQVVRVLGFQGVDAE
LLLGDLSQ"
CDS 22160..22660
/ID="YNAZFPKL_CDS_0030"
/transl_table=11
/phrog="10315"
/top_hit="No_MMseqs_PHROG_hit"

/locus_tag="YNAZFPKL_CDS_0030"
/function="unknown function"
/product="hypothetical protein"
/source="PHANOTATE_1.5.1"
/score="-133.1171930919211"
/phase="0"
/translation="MNTLPLTDTQSRILNHAIEHTAGRIDGFPDNIKGGARRKVLDGLL
KRGLIALNGGDHCVTAEGYAAVGQTPPTPVSPSEETEMAASNAETKATGKRSTGSREDS
KQAEVIRMLRRPEGATIPQICAETGWQAHTVRGLFAGAFKKKLGLSLVSEKPEGGDRIY
KIG"
CDS 22671..22781
/ID="YNAZFPKL_CDS_0031"
/transl_table=11
/phrog="No_PHROGs_HMM"
/top_hit="No_MMseqs_PHROG_hit"
/locus_tag="YNAZFPKL_CDS_0031"
/function="unknown function"
/product="hypothetical protein"
/source="PHANOTATE_1.5.1"
/score="-0.1886558004519607"
/phase="0"
/translation="VPESRRPLSFNWKMLGFAVAQRVTTGVPTILLKERV"
CDS 22778..23146
/ID="YNAZFPKL_CDS_0032"
/transl_table=11
/phrog="8020"
/top_hit="No_MMseqs_PHROG_hit"
/locus_tag="YNAZFPKL_CDS_0032"
/function="unknown function"
/product="hypothetical protein"
/source="PHANOTATE_1.5.1"
/score="-30.330110542412164"
/phase="0"
/translation="MKTNKPIPASRNEAWGFWGTMDQHAEVAWALAMTAISDATCQPLE
PVRAFLDTRHGRHFADDVLNEMLRGHAIQQAVDAAVARWMGWTIGRRTSHEYGIPRGMP
YLTGFVIHCEIVEETLAA"
CDS complement(23110..24384)
/ID="YNAZFPKL_CDS_0033"
/transl_table=11
/phrog="498"
/top_hit="No_MMseqs_PHROG_hit"
/locus_tag="YNAZFPKL_CDS_0033"
/function="other"
/product="DNA methyltransferase"
/source="PHANOTATE_1.5.1"
/score="-36515.427348896315"
/phase="0"
/translation="MNWLADKIEQWPTTKLLPYARNARTHSDDQVAQIAASIAEFGFTA
PILAGSDGVMVAGHGRWAAARQLGLSQVPVIVLDHLSPTQRRALVIADNRIAENAGWDE
ALLKLELAALQDEDFDLASIGFDADALLDLLADAESVTEGQTEDDVAPEVPGVPISRPG
DVWILGSHRLLCGDAADPDSFARLMDGVKAAMVFTDPPYNVNYANTAKDKRRGKQRAIL
NDNLGTNFSDFLRAALKPMLAHCEGAVYIAMSSSELDTLQSAFRAAGGHWSTFIIWAKH
TFTLGRADYQRQYEPILYGWPEGASRHWCGDRDQGDVWHINKPARNDLHPTMKPVELVE

RALRNSSRPGALVLDPFGGSGTTLIAAEKSGRVARLIELDPKYVDVIVRRWQDWAGRQA
TRLADGVTFDAAVDQAASVSSTISQ"
CDS complement(24381..25769)
/ID="YNAZFPKL_CDS_0034"
/transl_table=11
/phrog="498"
/top_hit="No_MMseqs_PHROG_hit"
/locus_tag="YNAZFPKL_CDS_0034"
/function="other"
/product="DNA methyltransferase"
/source="PHANOTATE_1.5.1"
/score="-64850.02601596069"
/phase="0"
/translation="MLQVEYRPLEPLIPYARNPRTHSAAQVAKIAASIVEFGWTNPILV
DGAQGIIAGHGRLAAARSLGLAEVPVIELAHLSPAQKRAYVLADNRLALDAGWDEELLG
LELAELSGAGFDLALTGFNDDELEALLSIDTEDSDDAEDGEPETADDVPEPPATPVSRP
GDVWQLGRHRLICGDASDPDVVAALMAGELARLCFTSPPYGNQRDYTSGGIGDWDGLMR
TIFAQIPLTDNAQVLVNLGLIHRDNEVMPYWDAWLGWMRSQGWRRFGWYVWDQGPGMPG
DWAGRLAPSFEFVFHFNRQARKPHKIVPCKFAGQETHLRQDGSSTAMRGKDGEVGGWTH
KGQPTQDTRIPDSVIRVMRHKGKIGQDIDHPAVFPVALPKFVIEAYTDAGDLVFEPFGG
SGTTMLAAQRSGRICRSVEIAPEYVDVAIKRFQQNHAGVPVTLIATGQSFDEVAAERDV
EVAA"
CDS complement(25947..26189)
/ID="YNAZFPKL_CDS_0035"
/transl_table=11
/phrog="No_PHROGs_HMM"
/top_hit="No_MMseqs_PHROG_hit"
/locus_tag="YNAZFPKL_CDS_0035"
/function="unknown function"
/product="hypothetical protein"
/source="PHANOTATE_1.5.1"
/score="-0.1891348277389108"
/phase="0"
/translation="VTEGARCPSAANDCGSFPAPEGYGGNARGISLASDRKPRFAGFAV
CTPCRSARPWRPRRYRRSHESAQRPRCKPQCKPAL"
CDS complement(26186..26296)
/ID="YNAZFPKL_CDS_0036"
/transl_table=11
/phrog="No_PHROGs_HMM"
/top_hit="No_MMseqs_PHROG_hit"
/locus_tag="YNAZFPKL_CDS_0036"
/function="unknown function"
/product="hypothetical protein"
/source="PHANOTATE_1.5.1"
/score="-0.0858158105530988"
/phase="0"
/translation="LRGTGERCGRLRKRQAFGLSHLGACRRRVTLTVQQV"
CDS complement(26293..26382)
/ID="YNAZFPKL_CDS_0037"
/transl_table=11
/phrog="No_PHROGs_HMM"
/top_hit="No_MMseqs_PHROG_hit"
/locus_tag="YNAZFPKL_CDS_0037"

/function="unknown function"
/product="hypothetical protein"
/source="PHANOTATE_1.5.1"
/score="-0.0606290730396786"
/phase="0"
/translation="LDEAVCKTAHVLRRSPPLHGGALTEVDSI"
CDS complement(26468..26818)
/ID="YNAZFPKL_CDS_0038"
/transl_table=11
/phrog="11214"
/top_hit="No_MMseqs_PHROG_hit"
/locus_tag="YNAZFPKL_CDS_0038"
/function="unknown function"
/product="hypothetical protein"
/source="PHANOTATE_1.5.1"
/score="-149.71057565771483"
/phase="0"
/translation="MKTIVIGIMPQEDIRKRLLAIARGELKPKAGDPKIWFTSMRSLAE
VLSDENRVLLKVIRETKPESITSLAAATGRKPGNLSRTLKTMSHYGLVEMKREKNHVRP
IAKGTEFRIVAA"
CDS complement(26815..27186)
/ID="YNAZFPKL_CDS_0039"
/transl_table=11
/phrog="No_PHROGs_HMM"
/top_hit="No_MMseqs_PHROG_hit"
/locus_tag="YNAZFPKL_CDS_0039"
/function="unknown function"
/product="hypothetical protein"
/source="PHANOTATE_1.5.1"
/score="-97.83341315963027"
/phase="0"
/translation="MCMEMDPGIETLLDLDDQIIDQGSGYWVKIEAYQVEPTPDVPHGI
RYSLTLHEPYGKRILGYDNAHAVKPPKKFKYAGRRLTFDHMHQHARDPGVPYEFKDAHQ
LLADFFSEVDQVLLEVKKR"
CDS complement(27228..27395)
/ID="YNAZFPKL_CDS_0040"
/transl_table=11
/phrog="6836"
/top_hit="No_MMseqs_PHROG_hit"
/locus_tag="YNAZFPKL_CDS_0040"
/function="unknown function"
/product="hypothetical protein"
/source="PHANOTATE_1.5.1"
/score="-4.9159559599709555"
/phase="0"
/translation="MRWVLWLEEEQRHLIWMRAERQGWKEISRRFACCNRTAQRRWQQA
LRQVADRLNG"
CDS complement(27353..27508)
/ID="YNAZFPKL_CDS_0041"
/transl_table=11
/phrog="No_PHROGs_HMM"
/top_hit="No_MMseqs_PHROG_hit"
/locus_tag="YNAZFPKL_CDS_0041"

/function="unknown function"
/product="hypothetical protein"
/source="PHANOTATE_1.5.1"
/score="-1.1516356987382894"
/phase="0"
/translation="MSTAGRPSPARPGRAMPTKLANAVSSRAPRRSTACWRRCAGCCGW
RKSSGI"
CDS complement(27592..27801)
/ID="YNAZFPKL_CDS_0042"
/transl_table=11
/phrog="19772"
/top_hit="No_MMseqs_PHROG_hit"
/locus_tag="YNAZFPKL_CDS_0042"
/function="unknown function"
/product="hypothetical protein"
/source="PHANOTATE_1.5.1"
/score="-4.765912456254872"
/phase="0"
/translation="MNGPNPHPSCPLGKLQPQATDLEAVKREGWREQHILVVHLTDERL
DFVERELIRQIGERLYGRRESRRG"
CDS complement(27798..28577)
/ID="YNAZFPKL_CDS_0043"
/transl_table=11
/phrog="159"
/top_hit="No_MMseqs_PHROG_hit"
/locus_tag="YNAZFPKL_CDS_0043"
/function="DNA"
/function=" RNA and nucleotide metabolism"
/product="RuvC-like Holliday junction resolvase"
/source="PHANOTATE_1.5.1"
/score="-349.69089747102055"
/phase="0"
/translation="MTTTLIPDGRRSAICPLKSAIWIQMANVRHLGIHRLQSLTVQMAE
FPDGSAANSPSGATDCSYETIYGIPDGGDSLPLRGRGALRALSPSRRGLSSLGGHDEGD
GTTLLALDLGTHTGWALHQRDGTIISGSETFKPHRFEGGGMRFLRFKRWLTEIKQAAGD
LDAVYFEEVRRHAGVDAAHAYGGFLAHLTAWCEHHRMPYQGVPVGTIKKHAAGQGNANK
AAMVAAMRSMGFDPVDDNEADALALLHWALATQRVGI"
CDS complement(28574..30727)
/ID="YNAZFPKL_CDS_0044"
/transl_table=11
/phrog="13004"
/top_hit="No_MMseqs_PHROG_hit"
/locus_tag="YNAZFPKL_CDS_0044"
/function="unknown function"
/product="hypothetical protein"
/source="PHANOTATE_1.5.1"
/score="-551088.4650477531"
/phase="0"
/translation="LLDFNDALPRPANPVDLAAQRETVRADLLARLPSVLKALFPAGKV
RGGAFAIGNVQGDPGDSLEVALSGEKAGLWYDHATGEGGDLFALIAAVHGLDTHGQFAE
VVALAGRLLGTARVEPTPLRGEAPVDRLGPATAKWDYVAADGSLIACVYRYDPPTGKEY
RPWDVRARLWRAPDPRPLYNLPAIAQAKEVVLVEGERCADALIHKGVAATTAMNGAKAP
IDKTDWSPLRGKAVLIWPDRDAPGWDYAENAARACVAAGAASVAILVPPMDKPDKWDAA

DAVSEGFDCVAFLIHGERQVIKAAPPVLPTHTLGALLDDDSPVPPDLIAPRVLTPGGLL
VFGGAPKVGKSDFLLAWLMHMAAGASFLGMTPPRPLRVFYLQAEVQYHYLRERVKTVSL
PPSRLGAARVNFVATPQLRLVLDEAGLAQVIPAIVQAFGSAPPDLIAIDPIRNVFDGGE
AGGENDNGAMLFFLSQRVEQLRMAVNPDAGLVLVHHTKKLGKKPFEEDPFQALAGAGSL
RGYYSTGMLLFRPDEAQTTRQLIFELRNGAALPVKSVDKFNGTWREVVNTGRRVLQDYA
ERLDAERRRKHDVILQMLFDEAAEGRCYSATQFAEAFEGRAGLGAERTIRERLSVLATQ
GYIKFFRNAEDYGLPPLHRSKFGYQCVEGMVLRREGPPDPDTGEIPMWPVSVYPTHYKC
PQTGALLPVEDSTIWLYHDEDNP"
CDS complement(30720..31079)
/ID="YNAZFPKL_CDS_0045"
/transl_table=11
/phrog="No_PHROGs_HMM"
/top_hit="No_MMseqs_PHROG_hit"
/locus_tag="YNAZFPKL_CDS_0045"
/function="unknown function"
/product="hypothetical protein"
/source="PHANOTATE_1.5.1"
/score="-22.26047836440849"
/phase="0"
/translation="MRTRQPYDGKRVQRQLPRPHVRLTVIDKLLRRHVTLPCPEAHLAV
AVITLAIGDCLDPGAVLRAEARHFLAGSALEFWCDAVGLEATFVRAIARKGGYLPSETA
HGAGVKRTPKERGLA"
CDS complement(31076..31840)
/ID="YNAZFPKL_CDS_0046"
/transl_table=11
/phrog="1990"
/top_hit="No_MMseqs_PHROG_hit"
/locus_tag="YNAZFPKL_CDS_0046"
/function="DNA"
/function=" RNA and nucleotide metabolism"
/product="Cas4-domain exonuclease"
/source="PHANOTATE_1.5.1"
/score="-138.2379281369166"
/phase="0"
/translation="MTCRSEGATMLDYNPGALFSERLTALIDAGLQRRQAGEPIRTYLG
ASRLGVACERALQFEYAQAPVDPGREFSGRMLRIFERGHRMEEAVVGWLRAAGFDLRTH
QADGEQFGFSALDGRLQGHVDGVIVGGPEGFDAPALWENKCLGAKSWRELVKHRLAKAK
PVYAAQVAVYQAYLALHERPALFTAVNADSMEISAERVPFDGGLAQRMSDRAVRVITAT
EAGELLPRGFADPAHVECRQCAWGLRCRGGGA"
CDS complement(31824..31988)
/ID="YNAZFPKL_CDS_0047"
/transl_table=11
/phrog="21342"
/top_hit="No_MMseqs_PHROG_hit"
/locus_tag="YNAZFPKL_CDS_0047"
/function="unknown function"
/product="hypothetical protein"
/source="PHANOTATE_1.5.1"
/score="-2.49726719886821"
/phase="0"
/translation="MAAVLPRLGDYVASIGMDRPLSAYSREEILQLVDVVLTTYFDHLR
EHDPDDVPF"
CDS complement(32002..32175)

/ID="YNAZFPKL_CDS_0048"
/transl_table=11
/phrog="No_PHROGs_HMM"
/top_hit="No_MMseqs_PHROG_hit"
/locus_tag="YNAZFPKL_CDS_0048"
/function="unknown function"
/product="hypothetical protein"
/source="PHANOTATE_1.5.1"
/score="-4.214070225877912"
/phase="0"
/translation="MRDLRTRGTRLLLGIAEEWPAQAGRQAPVQTLLLEALPAHPFTTT
EAERRRRDRSHP"
CDS complement(32177..33853)
/ID="YNAZFPKL_CDS_0049"
/transl_table=11
/phrog="16"
/top_hit="No_MMseqs_PHROG_hit"
/locus_tag="YNAZFPKL_CDS_0049"
/function="DNA"
/function=" RNA and nucleotide metabolism"
/product="DNA helicase"
/source="PHANOTATE_1.5.1"
/score="-201829.9109299384"
/phase="0"
/translation="MMLRPRQALLVERSLAALHQHGNTLAIGPTGSGKTIMLSAVAGGV
LEEPDAKACVLAHRDELTAQNRAKFGQVNPGRTTSVFDAQEKSWAGRATFAMVQTLARD
KHLEQMPTLDLLVIDEAHHAASPSYRRVIDRVLSRNPRARIFGATATPARADGKGLREV
FSNVADQITLGELIASGHLVPPRTFVIDVGAQSALAQVRRTATDFDMTEVETILNRTPI
TDAVIHHWREKAGERKTIVFCSTVAHAQCVADAFVAAGIRAVLIHGELSDAERKTRLAE
YETGEAQLVVNVAVLTEGYDYTPTSCVVLLRPSSHKSTLTQMIGRGLRTVDPAEHPGVF
KTDCVVLDFGTATLMHGSLEQEANLDGHRHQGEAPTKACPYCAATVPLGCRECPLCGFE
WIREEAAQAEALDEFVMTEIDLLKRSHFRWCDLFGCDDALMATGFGAWGGIFFLNGRWH
AVGGGRDLLPRLLAVGDRTVCMAKADDWLNENESLDTAHKTRRWLNEPPTEKQLRYLPP
AQRADFGLTRYQASALLAFQFNKSSIQRLVLAANDEHRRAA"
CDS complement(33868..34494)
/ID="YNAZFPKL_CDS_0050"
/transl_table=11
/phrog="2300"
/top_hit="No_MMseqs_PHROG_hit"
/locus_tag="YNAZFPKL_CDS_0050"
/function="unknown function"
/product="hypothetical protein"
/source="PHANOTATE_1.5.1"
/score="-81.36674812353615"
/phase="0"
/translation="MSYFDFNSAEQASFELIPKDTLVRLRLTLKPGGFDDASQGWTGGW
ATRSPETGAVYLACEGVVLDGPYARRKLWWNIGLHSPKGPTWQAMGRSFIRSLLNSARR
IHPADTGPQAQNARRIAGFAELDGLEFAGRIDIEKDGRGNDRNTVRAVIEPDHKDYAQL
MGQHFAPPVPTSPSGAIPAAATPAYAPSAPTASSAIPGGKPAWAQ"
CDS complement(34497..35360)
/ID="YNAZFPKL_CDS_0051"
/transl_table=11
/phrog="124"

/top_hit="No_MMseqs_PHROG_hit"
/locus_tag="YNAZFPKL_CDS_0051"
/function="DNA"
/function=" RNA and nucleotide metabolism"
/product="Sak4-like ssDNA annealing protein"
/source="PHANOTATE_1.5.1"
/score="-904.3827412899434"
/phase="0"
/translation="MTLPIISADQRLAERRGVKGVLVGKAGLGKTSQLWTLEAGSTLFF
DLEAGDLAVEGWAGDTIRPRTWPECRDFAVFIGGPNPALRDDQPFSPAHFDAVCARFGD
PGVLERYQTVFVDSITVAGRLCLQWCKGQPQAYSEKTGKPDSRGAYGLMGQEMIGWLTH
LQHTRNKNVWFVGILDEKLDDFNRRVFALQIDGAKTGLELPGIVDEVVTLAELPADDGS
RYRAFVCQTLNPWGFPAKDRSGRLDPIEPPHLGQLMQKIAGPARPPLERLDFSRPVPVT
IPTQET"
CDS complement(35357..35836)
/ID="YNAZFPKL_CDS_0052"
/transl_table=11
/phrog="2480"
/top_hit="No_MMseqs_PHROG_hit"
/locus_tag="YNAZFPKL_CDS_0052"
/function="unknown function"
/product="hypothetical protein"
/source="PHANOTATE_1.5.1"
/score="-42.27953894325365"
/phase="0"
/translation="MTSMNPDTLLATPAGQLAELPVESLCRLKHDAAAQLAAAKTLNEH
LDRAVEIRYAERARQLRLAMGKDTGVVHFDDGPVRITADLPKKVEWDARQLAALVRRIA
DSGEDPAQYVEISYRVSETKFNAWPAALQQSFAPARTLKTGKPGFRLALLGEDAV"
CDS complement(35833..36045)
/ID="YNAZFPKL_CDS_0053"
/transl_table=11
/phrog="66"
/top_hit="No_MMseqs_PHROG_hit"
/locus_tag="YNAZFPKL_CDS_0053"
/function="integration and excision"
/product="excisionase and transcriptional regulator"
/source="PHANOTATE_1.5.1"
/score="-0.4852122520139903"
/phase="0"
/translation="VSPKHLNPPALAERWGVSLATLDRWRSEGIGPVYLKLQGRVLYRQ
EDIEAYEARHLRRSPGQCVQAGGAV"
ORIGIN
1 taagccggct gctgaacctc cgggtacgcg tcccaggtcc gtccgcggaa cacgcgcccg
61 ttcgctttct tgtgtcgctt cacgccgtcc gcgccccagc cgccccactg cttgaaaaag
121 aaggcagcgc cagcagcatc cgcctgctcc ttcacgttgg ccacccattc ttcccgcatg
181 ggccgtgctt tgtggccgga ttcgcctccg acaatgaccc aatggatgcc tctgaggtcc
241 agtgtaccga ggtcctccag cagcggctcg acagaaagaa accggatatg cgcgttaacc
301 ccacgtagat ggtcgatgcg cggcacaccg tatttccgat cctcgaccga aacaccaagc
361 caggcattcc gagggcaatc cctgtctctg aagtacactg gcagccggtc tgctcgtttc
421 gtgagaattt gataggtgtg ctgcggagtc tggcgcacca cggagaagac ccggtccagg
481 aactcatctg ggatgtcttc gtggaacaga tcgctcatcg agttcacaaa gtagaccgtc
541 ggtttcttgc gctgcagggg ttgtgcaagt cgatccgggt gcgaggccag attgaatcca
601 tcctcgtatc caggcgcccc catggcttgc aggcggcgcg acatcacctc ggcgtagcaa

661 ttcttgcagc caggcgagat cttcgtgcag cccgtcgttg ggttccaggt ctgctcggtc
721 cactcaatcg tcgattgggt tgccatcgta acctccatcc tagcttgatg acgggctcat
781 tacacgagcc tgcgcgtgtt cgctcgccag cgtgtcgata agcctcgcca gtaagaggcg
841 tatttcgccg agatcggagt cggcaagctc ggtccgcacc ttggccgcga gttcatcggc
901 ggtgggcgca tgctcatggc cggttgaatg caccaggcgc atgcagtgct cgatcactct
961 gaccggcggg ttcgcccttc cgctctcata gcggctcacc gacgactgcc tgacacccag
1021 ctctcgggca aattccttct gactacggcc acttcgcgct gcgcggacca attcggccac
1081 agtggtgatt gccacgaacg gactcctgca atgatgcaca ttgcgcatat tatagcgcga
1141 gaaatgcgcg atgtgcatca cgcaaggcac agaactaggg cctgttaaca cattctgagc
1201 gcttcgatta tgagcgcgaa atggctatac gcaatagaga gaggcgggat cctcagcccc
1261 gaactcaaat cgtccccagc cccagaaaaa atcggtccac ccccggctgc tgaacgaccg
1321 cctcgagcgg tttcattacg ccctcggccg accggtacag catcaccttg gcgccatccg
1381 agtcagggta cctgaccgtt gccccgttcg cattccaact gggatcgttg tatctgtggt
1441 tggggaacca gccaacattc acgctatccg catccgtgat gcaccagggg atgtaatgcg
1501 cgcaggccca gttgccctgc gcatccagcg cggattgcag ggtcagatca taagtcatat
1561 ggtggctggg acccgatgcg ttgtgcacta cccagtcgcg aatatagaga ccaccgaaac
1621 cggcctcata cgggaattcg ttcgttggcc tggtataggc tccgcgcagg ttgagcgggt
1681 gcacgagttc gaaggttcct ctgttatgtg ggccgccaat atctgccgag tctacctttc
1741 ccgaatacca cccaaaccgt aatcccggtg cggtctggta gaggcctcca gacgggtcgg
1801 ccgaaaacca ccgctccgcc agcacagcgg cgtttctgca aatctgctgc ctgtagttcg
1861 atttaacccc gccgggaggc ggcccagcgc ccccccattg aaaatcctcg gtccagcgga
1921 cgtaatacag tgaaccggtc gtgaaatcga cgtccagcag ttgcacatat tcggagtggc
1981 tcgtatggga ctgactggcg gtctcgctga actcaggcgg tgatacgcct agatgggatg
2041 gcagcacgta ggcgccgggc gggtcaagca gattcaaggg cggcatcgaa ttgaacaccg
2101 ggtcccatat ccagtcgtcc tgggtgatct cgtccctgaa tttggcaacg aaggcattgc
2161 tcgaggaacc gccttcggga taggtgatcg tccagtcccc gttcgaatcc tgcttggttt
2221 tcccaatccc ctcaccaccc ggcactgtga tcagggtcgc ggtcgtttcc gtggctttgg
2281 agaaagcact gtcgtcccgg gtggagaacg ccgcgacggg gatgccgtcg atcaggaagc
2341 tgatgttttc ccggccggtg gtgcgggatt gggagcttgt cgtgtccgtg ccgatgcccc
2401 ggctgtctct gccgtagctg gcgatatagc tgctttcccg gcggtacgac gaagacgcct
2461 ggacagccag acgatgctcg gcattgccga cgtagccgat cgcaatcacg cgcgtcgtct
2521 cgggtatgtc gtactcatag aatgaggtgg ccgacgtcag cggcgtcgtc gttgcaggcc
2581 ctccgttcgg gtccgggatg agctcgcctc ccacctgcgt agcgccctga tcgatcgtct
2641 tggtgggcgc gttgcccagc gactccaccg ccaggctcgc ggactcgacg ccggcgcctg
2701 gaatcgtgcg cccgagttcg tccccggctc gcaccaagtc gatgtgcagc accaccgact
2761 ccctcgccat ggtggccacg gcgctggcac gaatcccatc ctcccgaaag aagaaccgca
2821 gcttggcggt cggatgaccg gcgtaaacct cggcgatccg gtcggctctg gcgccgttga
2881 tctgcgcgac caacagcttg ccctcgttgt ccgggtgggc ggtgatcagg atcaggtggc
2941 cgttgcacac cccgatgccg atgcagcgcg acaggtcgag ggtggcatgg ctgcgggtga
3001 aagcgttgac cgccacccgg ccgtcccggc acaaggcgcc gggctcgaat ccgaacacca
3061 cattgcctga ctccgggtaa ggcgtgttgt agcagtagcc cgtcggcagc cacaccgtcc
3121 ccgaggactg gatcgtatag gtccgccctt cccactcgac ggccagggtt ccgccggcca
3181 gcgccgcatg cggatcgggg aactcggggc tgtgcatcac gccgtccccg tccggcttga
3241 gcttaacctg gggataggcc ccgcccggcg tcccccaggg cggattgatc ggctggccct
3301 ttgcgtcctt gaacggctcg ccccagccgg tggcggacag tgccgaaatc ggcgtgcagt
3361 acaacccgcc catggcccgg acctggagcc ggatcaggtc gaagggatac ccgggaaatt
3421 cgatcgtacc cgccatcgtc acaccccgag cggcttgtcc ggatccggct cggcgaaatg
3481 gaactccacg acgtcgccgt tgccgtcctt gaacttcagg gtcttgatgg ccgcgtactt
3541 gaccacgaac tgcccgccgg cggtcttcag atcacgggcc gggtagtatt cgcggtccgc
3601 ccaggactcc cctttcgcgt tctgcgccgt cttctcggtc agcggcgagg cgatgccggc
3661 ggtcttcctg cgggtgccga tgccgacggc gttgccgatc cctggcttgg ccaggggctc
3721 gggcagcgcg tcgctggtcg tccgcaccgc gtcgacgatg gcgttgaggt cgttgacgaa
3781 aaacttctgg acagtcatcg ggaggtgccc tgagcggatg gtgggagcgg ctccgccgcg

3841 aaaacctgga tcgcggcgca gccgctccta cgccgtcgcg gtttcccgca cgttggcgat
3901 cacgaccgcc agctccgttg cggtcccgac cgcatgggtg gcgtcgttca cctcgatcca
3961 gatcggcttg gcattggccg ctcccgagag gatttcggtg ccgaggtcca gcgcggaccc
4021 gccgacggcc gcggccaagc cggcctgagt cgccgccagt ttcacttcgg aggcgctatg
4081 gccggagccg gtattggcgt ccaccaccgt ggcctggatc gggtccacgc cgggattggc
4141 gttggcctga atcttccggt tggccgtggg ggaaccgagg tacagcgtga acaccaccgg
4201 cggggtggag ccgtcggcgt tctgggtcgc caccaggttg cccgccagga gctgggtcag
4261 ggcggcatcg gtatagagtg cgaacgtggg cattacttaa gcctccagga tgagttcgtc
4321 gtcggggatc gcgatgggga gatggtcggc cggcgccgtg gtggtggcgt cgatgtgctg
4381 ctgctcgacg cccggcacgg tgatcgtgaa ttcgccgttg gcggggtcgt agccgatcgc
4441 gggcgtcgcc agcggatgct tgagcggcgc ctgggtggcc ggcggcttgg ccggggccgg
4501 gaaatcgaag ccggtgtcca cggtaccgac gccgtaggac ttcgacaccg ccaaggccag
4561 ggtggtgatg gccgcgccgt ggtcgaaatc gatcgaatgg gtcacccggc gcaccttgcc
4621 cttcgcggtc agccggggcg atgccatccg caccgtgtgg gtgcggtcga tcaggggcgc
4681 gatcagagtg tcgaaggtca cggtggtctg gcggtgggag gacaggatgt ccctccgtgc
4741 ccgctcgatc ccggtcatgt aggcgttgga gaggcccgcc cggccgtcgg ccgcaccgtc
4801 gtcgaggtcg tagtacagat cgcccagggg gttggcgatg gggtccagtg tcgcaaaacg
4861 ctgcgccccg gccgggaccg gcacggtgta ggtccggacc tggagcgggt ggtacggaat
4921 ccaggaatcg caggcatagc cgccgatggg cgcgccgtcg tgcaactcga gcttgaccgt
4981 ctgctccgtg gtccggtccc aatgtgcgta gcgcgggtcc ttgtcgctct gggcgtcgaa
5041 gttcgccgtg gtagtgccgt gccgcttgcc gatcacccga atcgaatccg gtgcgtcgac
5101 ggtgacgtgc cattgctcgg tcacggtctg ggtccagcgc tgcgccagga tcgcggacac
5161 cccgcccgcc aggctgtagc gcgggcagtc ggacaggtac atgacgccgt gcagccattg
5221 cgggtccggc agcggcgtca tccggagcag gggtgtcccg ccctccgcca gcaccgccca
5281 gccggtgccg tcgatggcct gcatgaccgt ttccggggtc ggcgccgtgc tgccctggtc
5341 gatgagctgg tcgagactca gctcccagtg gtagcggtac tcccgctgca tcagccgctc
5401 gtagcggtag tccatgtgca cgtcgatcgc gttgaccagc tggcgggccg aggccatgtc
5461 gatgcggacg ctgttctcga tcaccgcgtc ggggcggaag gtgaaatcgg ccgtagccct
5521 ggcctgccag ggcgtgatcc ggccttgcag gttgaggtcc aggtcgtagc tgaccggcag
5581 cgtcgacagc cggtcgagca cgtactgcca ctcgtcggcg tgcttgtcga acacgtaggg
5641 cgaccagcgg ccgccgatga tcccggcgat ctccttgcgc ttgagcccgt cgaaacggct
5701 ctgcagattg tcggtgcagg agagatggag caggccctgc accgggtccc agccgggcac
5761 gtcgaggatg ccctggaaga tcggcagctc cacgcccgcg gtgaccagaa aaagggtgat
5821 cggcagcccg gtcagcgtgt ggatgtggat cggcccgggc gccggcagca cggtgaactc
5881 ggcgatccgg gccgcgccct cttcgccgtc cacggtcagc gtgccggtca tccgccccag
5941 cagcgccccc atgaccgcac cggcttccgg cggcaccggc tcgccgtcgc cggcggcgtt
6001 gccgatgacg gccttgaccg cccaggccgt gatgtccgcg ctcgctgcct gggactggcc
6061 gacgaacgcc ccgccgccga cggcctgggc gtcgtattgg gtctggctga gacttgcagt
6121 caggttcgcg atgaacgcgt agacggcctg ctcgaccgtg gcgtccaggt cgaacaccgg
6181 gtcggcgacc gtctgtgcca ggcctgcgga cagcgtgaac accgccttga cccggtgggt
6241 gacgggcgcc tggagcctgt aggcctcggc ccggaggcgc tgttcgatga cgacggacgc
6301 atcgaaaagg cggaactgcg actgctcgac cggcgccgtg aggttgaacc gcgcccagag
6361 gcgctgctcg aggggcgccg cgaccgcgta accatcgaag ccgacctgtt cgacgaacgc
6421 ctcgaaaccc gtcaccgcgt agatcggctg acggatcggg gcgatcgcgt cgaagacctc
6481 gaagaccacc acggcctggg cgacccccgc cgacaggtcc aggcgcgcgt agacctggct
6541 gtggaggctt gcggcgatgg cgtagacgtc gagggtcgtc tgctcgatgg ccgcgacgag
6601 gctggcctcg acgtagatcg gctgcccgat ggacgccgcg atcgaataga cctcggcggc
6661 cggcgccgcc gtggctgccg cgctggcgac accgccgatg ccggtaacgc cgccgacggg
6721 gagcgtcatt acagctcctc ggccagcacg ctgacgtggg cgagctggaa ggcgccggac
6781 accgcggcgc cggggttggc ccggctgagc atgaagcaca ccggcgcgtc aggcgctacg
6841 tccaggacgt agccgccatc cgtgttgaat ccatagttgc cgggggcgac ttgcgtctgc
6901 agattggaag gggtggctgg cgccgtggcg cccgtttcca ccgagagcac gatgctgtcg
6961 ccgctgaagg tcgaactggt cgagagcacg gcgacgagct tcgacagcct gagggtcttg

7021 ccggtacggt tgacccagaa cgccgccgcc atgctgccgg tggtgctgcc cgaaaacggc
7081 gccaggaacc gggtgctggt caaagcgttg gtccccgtca tcgatgccga caggaccatc
7141 cgccagcggt cgatctcctt cgcgcccggc gaccattgga gcagggcgcc tgcggcgaac
7201 gcctgggcgc cgccgctgga actggcgatg ttgcgctcga tcgtcaactg gtacggtccc
7261 gccccattgc gggcggtcac cttcacccgc tcccacttcg tcggcgtctc ggcgtcgatg
7321 agggtcaggt agtagtggta ttcacccaac gggtcgggcg gcgtgatcca gggcggctgg
7381 gccgcggccg ccatcaacca ggtatcggcc gcccccaggg cggcggtgag cacggttttg
7441 acgccatcga gccccgcgag cttcagcatg tcagcctccg atcggatcga ccatttcggc
7501 gctcaggcgc cagctgtaat tcccggtcgt gccgtccagc tgctgctccg gaggatcgca
7561 gtagacggtg atcttcgggt agtaataggt gatgtccacg ccgtcgacgt tcagcgtgac
7621 ggtggcgaaa tcgccctccg tccgcaccgg taccggaggc gtaccgccgc cgacgatggc
7681 caggggcgaa gcgcaggcca gaacctgcgg ctgcgagtag tcgagcgccg acagccccgg
7741 cggcacccag ccgcctccgc tgatctcggt ggcgagcttg cgccaggtgc gctgcttcac
7801 cgagcggccg gaccacatcc ggtgggtcgt tgcgccttcc agccagcggt aggtctggga
7861 ccattccagc cgggcgtaga gcggcacggc gataccgccg aggatgaagg ccgaatcgcg
7921 gagtgcgctc accgttcatc gggctccggt tcatcgcagc gcaggaccag cccggcctcg
7981 tcgatgtgcg cgaggagatc ggccgcccgc gccgggcagc gccagggggc cggcaccagc
8041 gtcacccgcg gctccagggt gcaacagccg gacagcgtca aagcgcacac agcgccaccc
8101 agagcccgga caccacgccg gcagacacca gcacggcggc cgcgcaccaa acttctccgc
8161 gggtcaaaac gatcatcagt ccctctcctt gaaaaaatgc agccggccga tgaccgcggt
8221 tttcaccatc gctctcgccc agtgcggcgg ttttttaagc aggctgtcgt cgaagtagtg
8281 cgtcgcctcg ccggtgatat cgggcagcgt gcctttcagc gcctgggatt ccagcttcag
8341 cgcaatcgag aacgcgcggt cgcgcgcgtc cacggcttcc gccttcgcct tgttcgggtc
8401 gccttcattc cagcagctga actgccgggg gttcaggcac acctcgtccg gcgtccgccc
8461 ccaccagcca ggccgctgcg cccgccggaa gatcacgttc atcacgccct gcatgccggc
8521 ctcgccttcg ccgcgcgctt cggcccaggc ggtgcgtgcg ggaatgtggt tcatcgcttg
8581 cctgcctcct tgttgctggc gacatgcagc acgttcacca gggtgacggc caggacgccc
8641 agtccctgcg ccaccgcggc gacgtcggtg ccggatagcc gcaagtcgta gccgaacgct
8701 tcggccaatc ccgtcgccgc catcagcaag gccgtcagct tggcgatgag gtccgcgcgg
8761 cgggaccata cgccggcgtc ctgcaatgcc ttgccggcct gcatgcactc gtacagatat
8821 ttcacttttt cgaacatatc gctgtcctcc tgtcgaaatt cacggcgttt ttaggcacat
8881 cacggcgctt tcacgccgaa cgagcgcacg aaccacaacg cgacctcgat gaggaagtgc
8941 acgacgttca tgcggactcc gctgacgaag gtgccgatgt aggccgccgc ttccttcttc
9001 ttgatgtcgt tcagcgtggt cttgtcgacc agccctgcga tcgtccgggt ttccagttcg
9061 gccaccttgt tctggaccag accccattgc tccttggtga tgccggagac ggcctgcacc
9121 acgaaccaga gggcccagcg taggatggga ttcacgggtt gttcctcctg tgggttggga
9181 aactcatcgc acgatccgcc cgtgcttcag tgccgctcgc gccacctcgc gcttgagcgc
9241 cgctaccact tggggatcgc cgttgagcgc gaaggaaccg ggcacgccgg gcagatggat
9301 gtgtaccggc tggccaccgt cggacgcgcg cgccagtgag gcatcgaacg ccggcaccgc
9361 ctgcgccacg tagccgccga tcgaaaagcg cgggaggctc aggttgttga gggcatacag
9421 cttgtcgagg ccgagcttgc gcaccgcctc cttccgcagc acgaattcgc cctcttccaa
9481 cagcgccgga atacggtcgc cgccgccgta gcccttgatc cagccgccgc ggcggaagcc
9541 gggtaacgtg gcatcggccc cgttggctcc agtgtccgaa ccgtctccct gctgctgcat
9601 gacgacagag atcgtcttgg tttcgggttt cagcagttcc gccagccggc tttgcagggt
9661 ctggagcgac gccgggtcga cgtcgacccg cagcgcgaac cccttcgtca gctgctggtc
9721 gatgtcgctg atttcggcct ggacgctctg caggttttga agggtttcct ggatcttctg
9781 gctgaccgcg gcggccgcct gggtgtcggc ctgttcctgc cgggcggcgg cttccgccag
9841 cgcctggccg gcctgacggc gcgcgtcgat cagccgctgc aggtcctcgg tgccgaactg
9901 cggctggccg ggatcggccg gcgtctgttc acccttgagc gcgttcaggc gctgttcccg
9961 caaggaggcg gcagcctgct gcccgaggga ttcggccagg ccggcggcct gctccccggc
10021 ctgccgcgag agttccagat tgccgttcgc gagctcctgc cgctgctgag cgagcagctg
10081 ctggagctgc gcgtactggg cccgtgaggc ttccaacggc gtcagggccg cctgctcgat
10141 gccggcgaca gcccgtgccg tgctctgttc gatgttgcgg cgttcctggg ccgctgcgcg

10201 ggccgattcc agcagccgct gttcctgccc gatcagcttg tcgatggtgg cggtgtaggc
10261 cgattccagg tccgcgtaga tggcccgccg ggcttccagc gattcgcgct ccaggacggt
10321 cttgcgggct tcgccgtcct tgaacttggc gatttcccgg gcatagacct gatcgaccag
10381 cccgagacgc tgggcggcaa agtcccgcac ggccttcagg cgggcaccct cggcttgcag
10441 ggtcaaggca tggagctgac gagcctgctc cagctccgag cgggcttggg tggcttgcac
10501 gccgttctgg gcatacacga tgggcggtgc ggcatcggct tcgatcggct gcgcctggat
10561 gcgcgtctcg gccagcttct cgagggcgtc gatggccgcc gtccggcgct ggtaggcgtc
10621 ttcgatgccc gcggtcgcgt ccttcagggc gttccgggtg tcctcgaagc tcttctgaac
10681 ctccttctcc ttcgccttga cggcattgcc gctgtcggcc gcggcctgcc tgcgcttgcc
10741 cagctcggcc cgcatctcct gcagcagcgc ggtttcaccg gccagctccc gcccgatctt
10801 gccgcccatg aacgcctggt ccgccgcctg gccgacttcc ttgtagcggg cggtcagccg
10861 tgcgaccacg gcctcctgtt tcttcaccgc ggcttcgtcc tggagcaggc tcagctcggc
10921 ttcggatttg ccgcgggtgt cctggccggt tgccttggcg agttcgcggc gcttccggat
10981 gacgcgctcc agttccgatt ccgcggtgct gccgaacaga tgccaggcgg acaccgccgt
11041 cagcagcgtc accgcgatgc cgacgggacc gccgaggaag gcggccagcc cgcccagagc
11101 tatcttgagc ggcccggcgc tggccgccac ttccgcctgg gcgacagcca agcgttgcga
11161 tgcaggtacc agttgggtct ccaccagcgc caaccgcgcc atgccggagg cattcgccac
11221 ggcggcctcg gcggcggcga aggtggcgcg ggcgttgtat gcttcgttga gggcgtggtc
11281 ccgcgccgcg gccgtagcgg ccagatgagc ttgcaccagc cgggcttggc tcccggcgaa
11341 ggctgcgacg gcggccgtgc ctttgcccgc cagcaccgcg accagcaccg atcccgcgat
11401 ctcgaccccc gtggcgatcg tgtcgatgtt gccggccgcg ccgttgatgg cgtcggcgat
11461 gacttccgcg gcgcgggtga tctcggatga ggtgccgacg aacttcaggg cggcattgtc
11521 ccagcgcacc accgcctgtt cgatggtatc gggcagcccg gccgcgtccc ggtcgatgct
11581 ggccttggcc ttctgcagcg cctccacgac cttctgcgtc gtcagttcgc cggcttcggc
11641 cagggtgcgc agcttgccga tcggcacgcc cagcccgtcg gcgatggcct gggccagccg
11701 cggcgattgc tccatgatcg agttgaactc gtcgccccgc agcacgcccg cggccagcgc
11761 ttgcgagaac tgcagggtgg cgctcgaggc ttcctccgcc gaggcgccgg agatgcggaa
11821 ggatttggcg accgagtcga tgacgctcag cacttgtcgc tgagacacgc ccatctcccc
11881 ggcgcccttg gcgatgcggg catacaactg ggagacgccg gtcatcgcct gcgcgttgtt
11941 ctgggcgatg gcgaacagcg cccgctgcgc ctcggtgaac tggttctggg aaccggccgc
12001 gagcttgacc cggccttcga ggttgcggta ctggtccgag agtttgccca gatcccgggc
12061 agcttgcagg gcttcggcgg ccccgaacgc gccgacggta tactgaccga tgcgccggag
12121 cgatcccgtc aaccggtcga cctgcgccgc gacgttgccg agccccttct cggccgcggc
12181 cgtgttcgcc gcgatctcga tgaccgtctt gaaagtcgtc atgggcctag cgccgccgca
12241 tcaaacgcgc ctgcaggaac agccgccagg ggtaatgcca gacgccggcg tggccgcgct
12301 cgatcagcgc cagcgccgtc atctccaggt tctctaggtt gccggcggcg ccgtagcggc
12361 cacctgggcc gcgttggcca ggcgttcgcg gagcccgaaa aaatgcgggt tcaggctctt
12421 ggccgcctcc ctcaccttgt ccagctcgga ggggagcatg gcgtcgagct cggcccggct
12481 caggtccgag aaccgggtca ggtcgtccag ggtgatgtcc gcgaacagcg ccaacgccac
12541 caggtcgttg cgatccagtt cggcgttggc gtccttcagc caggcgcgga cctcgcccac
12601 ggtcagctcg cgcaccacga tcttgcggcc gccggcctcg atggtgcgct cggcgcggag
12661 gcccttgggg ttcgtggtgt cgagctccgc catggggaat tacaccgccg gggtgatggt
12721 ggctttgaag tactggctga cgcccgcacc ggtcttgctg gtgtccttca gcaccttgcc
12781 ggtcagctcc agcaccgcga agtcctccgt accggggccg ggcaggctgc tggccggcga
12841 gaacttcacc ttgtagaagt cgtaagtcac ggcatcgccg ttgacctcgt tgacgccgtc
12901 gaaagtgatg tgccagtcct cggcggtctg ggtcagcgcc tcgatgctgt taccgctcac
12961 cgtgccgcgg gtggccagcg ccaggttctc cttgctgatc gaccacagct tcagtttcag
13021 cgtgaccgac ttgatgcggc tcaaggacgc gatcgagccg ccgcccgggt tctggtaatc
13081 ggccagttcc ttggtgtcct cctcgatgct gaggtccagg gcggcattgc ccaggtccac
13141 cggggtaccg gcgccgacgg cgcccttgat ggtgccggtc aaaagcatgg cggtcgtagt
13201 cataaaaaac tccttgagta acggatggat gaaaagaagg aaagatcgac gccgaccggg
13261 cgtcacgggg tcccggtgtc cagcggcagc ggacgcactt cgaacaccag ggaaaactcg
13321 acgtggccga gatcgaacca gggatcgtcc aggccgacgt gcttcagccg gccccagccc

13381 ggttgcgggg accagtgctc caaggcgcgc agaatccgca gcacgtatgc gccgcccgtc
13441 accaccgtgc tcgccggcgg ggtatgggcc acgcacacgg tgacctgcca gcgctggcgc
13501 tcggccagga aaacgttggg cggcggcgtc cccaccggcc cggcgaagcc cagcggcagc
13561 accagggcgg cggggcagag cggctccagg ttctgcaccc cggccaggac accgacgccg
13621 tccaccgtga cgaactccgg cagggtctcg cgcaggcgcc ggatcagcgc cggctcgatc
13681 tcgaacatgc tggtgatcat cgcggcagcg cgtcctgcgt catttcacgg atcagtgcgg
13741 ccagccgcag cgtggcccgg gtcgccagta cctcggccag ggccggcact ttcggcaggg
13801 ccaccacgtc ctcgaggacg ggcagggagc ggtgcccgac ccggtgaaag atcccgcggt
13861 ggccgctttg catccgggcg acgaagctgc cgggaaaaag gtaagccccg gcgctgctgc
13921 cccagtcctc ctggcgcagg cggccgacat aggcggcctg gatcggggca tggccggtcc
13981 atacccggcc acgcagtcct gccagccgca gataggcttg cacccggcgc gagcgcagcg
14041 ccctcagcgg aatgccgtag gtcgcgccga tggcgcggac catcgcctcg cgggtctcgc
14101 gcgcggtctg gcccacggcc cggcgggagg ctgtgcgtag caccgccggc aagcggcgca
14161 aatcctgagc ggtatcgccg tgcagccgca cgcgcaagcc gatactcatc gcttcgggct
14221 cagccccggc tcgaatggat cagcaaaccg aaccagcgcg ccccgaaatg ccccgcgacg
14281 atcgccgccc acaggctctc gtagtagccc agaccctccc gctcggccag cagccacacc
14341 gagaagccga ccaggctgca ggaaacgacg tcgaccagca ggcaccacag gcacagccgc
14401 cacggccggg gatggcgccg ccagcgattg agctgggcca ccagtcccga ccagatcgtc
14461 accaggatga tctgcagcgc cagccggagg tcatcgccgt tgccgtgcgg cgaatccagc
14521 aggagctgct taggtcgggc gttcatcggg gccgcaggat caaggtggtc aggccgtcca
14581 ggcgatcgat ccgggaggcg atgctgtagg ggacaccacg gaccttcagg ggatcgccca
14641 tgtgaatcgc gggcaggtcc gcactccgca ccgtcaaccg cggatcggcc tcgggccggg
14701 gcgcgcccat cggtcccggg cgttccagcc cggccggcac cgagacgatc cccgtcagat
14761 gcacaggcgg cggctcggtc cggtccggcc acaggatcac cggctcgccg aaggtcgaca
14821 ggcacaccgc attgaccgct tcgaggtcca tgcctagacc gtcaacttga tcagcagccc
14881 cggccggtgg cacatcggca gcgggttgga ctgggtgtgg agatcggtgc cgcggtcgaa
14941 ctggcgcggg gcctgcttgg catagagtgg ctggcccaag gtattcaccg tctcgttgaa
15001 atcggccggc gcgacgtagg tggcgaaggt atcgacggtg cccagcggga aggcatgagc
15061 ctcgccggcg gcgatgaagc gccgggtcac cccgtgcaca tcggtggcgc ggccacggta
15121 ctcctcgaag gtgatgccgc cgaaggtgaa gccgcggcgc acgtcgttga tcaggatcgc
15181 cccctgctgc caatgggtga aggcggtctt gacgtccttg tgtccggtca gcgcggcgaa
15241 gaattccggc gagcacaggc aatgcacgtc cgtcatgaac tcgcccagca ggctgtcctc
15301 gatgtgggcc agcacctcca tgcatcgcgc cttgacgtcg gtggtggcgg tgcccagctg
15361 gaagctcacc gtcttcggcg ggatgtcgaa ctcgtcgtag aggtcgtaca gcaccgaacc
15421 gtcggcatcc aggatcaccc ccttgagcgc acccatgcgc agatgctcca gggtgatcgc
15481 atgcttgttg cgcatggtct ccagatggcg cgccatcacc ccggccaccg cctccagctc
15541 ggtctccgag ccaaaggccc ggatgccctg gacctcctcc ggcagcacca cgtcgtcgtg
15601 ggggatgtgc ggaatcacga acgagcgcag ggtgcgcttg ccgcggacgc ccaccgtccc
15661 cggtgccccg ggcggcaggg tcggcaacag gttcagcacc ccgttgcgct cctcgaccag
15721 gatctggcgc tgccgcaccg gtttcaccgg catcaggttc agatcctcca gccggccgta
15781 gcggttaggg ataatgttga tggcggcggt cagtgccgcc atcgaaaagg cagggttgtg
15841 aaacgggttc tgcatgatca ggctccggtg cggacgagga tgccgagcgc ttgcaactgg
15901 acgatcgccg tggccttctg ggccggggtg ataccggccg gccacaccag acctaagtcg
15961 gacacgatgg catggcgggc gagcagcagg gcttgcggga cttcggccgc ggtggcgtcg
16021 accgcttcga tcagcacccc gaccgcctgg gccgaggcat ccgtcgcggc gggatcgagc
16081 gcctgcaggc gtaaaccggc cggctcacgc gccaccaccg cccccagcgc cagcttctgg
16141 ccgagagcca ccggggcaag gtcgcgggaa tagcggttgg gcgcctcgta cttcaagaga
16201 tcgccgaggt tcttgggttc gcgtagcttg ggcatggctc agctccgggc gatgagtttt
16261 tggacggcgg cgaccaccgg gctcgcctcg ggccggaccg aggtgcccgc atcgacctgg
16321 atgcgcgagg cgatctcggg cgtctcggct cgcgcggcca gcaaaacccg ccgcacctgg
16381 gcctcggaca gtccggccgc caggaactcc gcggtgcgcg gggccgcgcc ggcgatcagg
16441 cagagctcgg cgatggcctg ggcttcgctc cgtcctgcgt ggtgggcttc ggtcgcgagg
16501 gactgggcag tcggttcgcc gatgacaggg gtggccgcag ccaagtcgtt ctggggggac

16561 ggtgcggcat gcggcgtcaa gttgccggcc gcatcggcgt ccttgcggct ttcgtcggcc
16621 gcttcgtcat cgagtggccc aggcgcgtgg tcgtgcataa cagactccga atgaaggaga
16681 gaaacgggta ggggtgacat caccgcagcc ttccgggctt gcggcgagat cagtcgttgc
16741 cgggtgcgga ttgcatcggc gaacttgttc agcacggcat cgaaaccgag cacgccgtcg
16801 gcgagtcccg ccgacacggc gtcctcgccg aagaacagcc cggcctcggt ggcgcgtacc
16861 gccgtttccg ggagtccgcg gaatccagcg acctgcgcca cgaagatccc gtacagccgg
16921 tccacttcgg cctggagcgc ggcagccgcc tgcggcgaca gcggcgcgtg cgggttgagg
16981 tcgttcttgt gatggccggc atagagcgcg ctgtaggtca gtccctcgcg ggcatccttg
17041 accgactggt cgacgtgcag ggcgatcact ccgatcgagc cggccccgcc ggtctgcgcc
17101 agggtcacgc gcgaggcgcc ggcggcgatc gcataggcgg ccgagaacgc cgtgtcgttg
17161 gcgtgggccc agatcggttt gtgcgatgtc cccgcacgaa tcttggcagc caactcgaac
17221 accccgccgg attcgccgcc gggcgagtcg aggtcgagca ggatgccgct cacttcggga
17281 tcccgcagcg cggcatcgag ccgggcgccg atctcggcat aggaagtcag gccggaatgc
17341 gcctccagct ccagcgtccg tcgcaccagg gtgccgtgga tcgggatcac ggcgatgccc
17401 ggaagggctg gcgcggctgt ccgggacatc ggcgccggca ccgcggtagc cgcttccggc
17461 agcccgatgc gggatcccaa caccgccagg atcacctcga gcttcggccg ggcgatgagg
17521 agcggcgttc cgtacaagcg ggacgccaga tggatgagcg gctgggtcat ggtgcgttgg
17581 ctcgagtcga tgtgggtggc gcggcagcgc ggtcatgccg ggggtccgaa tcgaacacca
17641 gccctaaggc atcggcccga gcgttgtcgg cggcgatctc ccggtcgatg tcctcggcgt
17701 cgtaaccgaa ggccgagatc gcctccgatc gcgacagcag tcccgcccgg atcgccgtct
17761 gcaaggcgtc gaactccttc ttggggtcga cccactgcca gccctgcggg atccatttca
17821 ccgccaggta atctctttcc ctgcgggaga agcccggcaa atcgagcgcc ccctccagca
17881 ccgcctgggt catccaggca cgccacaccg gccggcaaag ctggaacacg atgacgccgt
17941 gctggatcgc ctcacagcgg cggcggaact ccaaaagtcc ggcgcggatc gaggaatagt
18001 tgacctgggt caggtcgccg gtcagcatct cgtaggtgat gcccatggcc gccgccaccg
18061 cccggaactg catgcgcagg aactcggcgt agctcgcccc cacatccgcc ggctggctga
18121 accgcacgtc ctcgcccggc tccagcagct gcagggttcc cggttccagc cccgccaggg
18181 ccaccccctg ggcgtcggcc gggccttcgc ccatgagcgg atcctccggc gccgtccggg
18241 tgatgaagcc ggcgaacatc gccgcagtct tcttccgcac caactcggcg tcgtcgtact
18301 ggtccagctc atgcagcttc accagcgccc gcgccagcca gggctcgccc cggatctggc
18361 cgggccgcag cgggcggaac agatggatca cctcgcgggc gtcgacccgc accgtgtcca
18421 agccgcccgc cccggacatc ggtgccaggg cgccgtcctc cggatgggag cggtacaggt
18481 gataagccac tcgtcggccc agcttgtcga actcgatgcc ggcccggatc acgttccccg
18541 acgacagctc ccggttgagg gcggcgggca gatgttccgg ctccagcacc tgcagttgca
18601 ggccgacgct caagccatct tccggccgcc ggtagcgcag ccgcaccagc gcctcgccgc
18661 cttccaccag ggcgcggcag gccagcgcct gcagcccgta gacatcggtc aacccggccg
18721 catcggcgtc atcgccccag tgggtccaga gcgtctggat ggcggggcgg accgccacgt
18781 cggccgccat cgactgcggc ttgatgccgg taccgatggc attggccacg taggcctcca
18841 ccccggcggc ggcccagacg ttgcgccgca cctggtcgcg gctcttggcc cgcagttcct
18901 cctggctggc ggccagggcc gccaccgcac ccggattggc gacctgccag gccagcgccc
18961 ggcggccgcc gccgatgccg tcgtaggtcg gcgtggggcc gccgaacaag ccaaaccgca
19021 accgctgcca ccagcccatc aggtcgcttt gttcgtggtt atgcggattt gtctcgccac
19081 cggctggccc tgggccgcca agccgcgttt cacctcgcgg atcgccgtct gcaactcctc
19141 caccgagcgg tactcgacac tgcggtcctg gaacgtcacc cggcgctcgc cccgggccat
19201 ggcggcttcc agggcttgca ggtgttcagg cgtaaagctc atcggtaggc catgacttat
19261 tgttccgctg tcatcaaaaa gtctgcttgc gatcagctca gccagcggct acgaataacc
19321 cggcgcgaag atcgaccggg gccggaaaca gaacgcccac cggggtcggt gggcgtttcg
19381 gggtgaatcg gttgtaacgg cgcttcgggg tccggcggcg gacccacacc caactggtcc
19441 tccagggccc gccaatggcg ctcctcgaat cggtccagcc ccgcggcggc cgcggcggca
19501 cgggcgtaga cgtagcagtc cagcgcctcg ttgcgctccc tgagtttctg ccattcccgc
19561 cgggcgaagc cgtggcggtc gcggcgggtc accagctgct cggcgcagag ctgctgcagg
19621 tactcggcgt ccatctgcgg cagatggacg tagcccggcg gatagaccag ggtggtgccg
19681 tcctcgccca cctcggcggt cttgcgcagg gcgttgtaga actccagctt ggcgatgccg

19741 actgcgactg aaaacacctt gatgccgcgg cgcagttttt tgccggcttg ggtgacgtcc
19801 accgcggtgg gggtgccgat cagcgccgca ccgcggggca cgcccttgac cgccatgatc
19861 cgcggatcct tgagtcggcg gacgaaggca taggcctcct gggtggcgta gccggtatca
19921 agggcgagcc gtgccagcgg catccgggcg cccgaggcat gggtccaggt gtcaccgacc
19981 agggttgcca aggcgtgcca gacggcatca cgggcggtgt cgcccatcag cacccggtgt
20041 tccaccagcc aggcggactt accgcgcccg aaggcccaga ccgacacctc gatgcgatcc
20101 ttctgcacgt cggcgccgcc caccagcagt aagccgccct ggggtacctt gccgatggga
20161 tagtcctccc tgcgttccag caggcgctgc cagtccggcg tctcgccgtc ctccacccag
20221 gtctcgccca gctcggtgtt cttgaaggtc ttgatcgcgg atgggccacc cgagaccggg
20281 tgcaccgcgc tgtcccaggc tgcggcgatg tcgcgccaac tgcgccagcc caaggggctg
20341 taaagggacg acaggtggaa gccggcggtc ttggcgccat gctcgggcgc catcgcccgc
20401 cattcgccct gtgccagcat gtcccccttg tgatgctcgg gaatcggccg ttcgcagcct
20461 tcacagacgt aagccaccgt ctctggctgt tcccgcgccc agcgcagctg ttcgaagcgc
20521 agccactgaa aatgcccgca atgggggcag ggcacgaagt agcggcgctg gtccgaggcc
20581 tcgtactcgc gctcgatggc gctggctccg gcaatcgtcg gggtcgagac gatgaagatc
20641 ttgcggcggg cgaaggtgcg ggtgcgggcc tcggccaggg cgatcgcatc gccttcgccc
20701 tcgacgtcgg gcggatagcc gtcgacctcg tccaggaaca ggtagcgcac cggcatcgag
20761 cgcaggccca ccgcgctgtt ggccccggtc atcaccagca ccccgccgcg gaactccttg
20821 gccaggatcg tgttgccgga atcgcggctg cgggccgggg cgatcagccc ggccaaggcc
20881 ggcgattcct cgatcagcgg gtcgatgcgc tggcgggagt tgcgcttggc catctccacc
20941 gtcggccaca ccgccatcat cgggcccggc gcgtggtgga cacaaaagcc gatccagttc
21001 gagcccatct cggtcgcgcc gagctgggcc cctttcatga acaccacccg ctccaccggc
21061 gaggccggcg acaggcagtc catgatctcc cgcaggtacg gtgtccgggc ggtgcgccag
21121 cggcccggct cggccgaggc cttgctggac agcacccggt ggcggtcggc ccattcggac
21181 accgtcaaca gcggatccgg cgtcagcccg tcacgccagg cgtgggcaat cgcctccaga
21241 ccctcgtcct ctctcagcat tcagtcgacc cggggccgca gctcgcccaa ttcctgcagg
21301 tgttcccgca cggccgtctc cagggcgatg tgcagggtgt ggggatcgac gccgagcttt
21361 gccgccagcg gcgccgagat tcgcgctggc cagttcagcc aagcgtcgcg ctcggcccgg
21421 gccagggtga acacctgggc gatggccgca gcgcggtcga ccagatcgcc cttgagtttg
21481 gccagccgga ccttgttggt ctgggccttg accacttcgt tgaccgtgcg ggcctgcagc
21541 aacgaggctc cgccgccggg caaggtcggc ccgccgccgt gggaggcgtc gggcatcgca
21601 acccgtaccg cgggttggcg cgttccggcc ttcggtgtgt ccgaattcct ggcccattcc
21661 cgatccgcct tgtccggatc gatgctgcca tcggcctccg gcgtgatgcg gccggtgcgg
21721 atggccttgt gcacggccgt gtcggtgacg ccgcggtggc gggcgtaggc gcggatcgag
21781 agtcccatcg gtgaaatcga tcgcaaatca gcttggcttc cggggtctac agcgcgttca
21841 tggcgtcgtc atccacacca ccacgaggac tacgtcatga ccccgcttga cgccttactg
21901 actgagatcg cccaacagca actcggcatc gacaccctgg aaacccaaaa ctcggacgac
21961 ctggatttcc acgacacggc ggtctggtgc ctcaaagccg cgctggaggc ggcgtaccag
22021 gcagggatcg aggccggacg ccgggtcgag gccgatgctt gaacagatcg gcttggcgct
22081 cggtggttcc ggtggaattt ccaacggctg gctggcgtcg atgacaccgc tcaacaaccc
22141 aactcccagg agcacccaca tgaatacact ccccctgacc gacacccaga gccggatcct
22201 caaccatgcg atcgagcata cggccgggcg catcgacggg tttccggaca acatcaaagg
22261 cggcgcccgc cggaaagtcc tcgatggcct gctcaagcga ggattgatcg cattgaacgg
22321 tggggatcat tgcgtgaccg ccgaaggcta tgccgctgtg gggcaaacgc cgccgacgcc
22381 ggtatctccc agcgaagaaa ccgaaatggc cgccagcaat gccgagacca aagccaccgg
22441 taagcgaagc accggaagcc gcgaggacag caagcaagcc gaagtcatcc ggatgctacg
22501 ccgccccgaa ggcgcgacga tcccgcagat ctgcgccgag accggatggc aggcccacac
22561 cgtgcgcggc ttatttgccg gcgccttcaa gaaaaaactg gggcttagct tggtctcgga
22621 gaagcctgaa gggggtgatc gcatctacaa gatcggctga tcgagccggg gtgccggaaa
22681 gtcggcgccc cttatcgttc aattggaaaa tgcttggctt cgcagtcgca cagcgcgtta
22741 ctacaggcgt tccaacgatc ctgcttaagg agcgagtatg aaaaccaaca aacccatccc
22801 cgccagccga aacgaagcct ggggcttttg gggcacgatg gaccaacacg ccgaggttgc
22861 ctgggccctc gcgatgacgg ccatctccga tgccacctgc cagcccctcg aaccggtcag

22921 ggctttcctc gacacccgcc atggacggca cttcgccgat gacgtcctca acgaaatgct
22981 ccggggccac gcgatccaac aggcggtgga cgcagccgtc gcccggtgga tgggctggac
23041 gatcggccgc cggaccagcc acgagtacgg catcccgcgc gggatgcctt atctcacagg
23101 cttcgtgatt cactgcgaga tcgtcgaaga aacactggct gcctgatcaa ccgcggcatc
23161 gaaagttaca ccgtcagcaa ggcgggtggc ttgcctgccc gcccagtcct gccagcggcg
23221 aacgatgaca tcgacgtact tggggtccag ttcgatcagc cgcgccaccc gtcccgactt
23281 ctcggcggcg atcagcgtcg tgcccgaacc gccgaagggg tcgagcacaa gggcgccggg
23341 ccggctggaa ttgcggaggg cccgctccac cagttccacc ggtttcatgg tcggatgcag
23401 atcgttccgg gccggtttgt tgatgtgcca cacatccccc tggtcgcggt cgccgcacca
23461 gtgacggctg gcgccctcgg gccagccgta caaaatgggt tcgtactggc gctggtagtc
23521 ggcacgcccg agggtgaagg tgtgtttcgc ccagatgatg aaggtcgacc agtggccccc
23581 cgcggcgcgg aaagcggact gcagggtgtc taattcgctg gaactcatgg cgatgtagac
23641 cgcgccctcg cagtgggcga gcatcggctt cagcgctgcc cgcaagaagt cggagaaatt
23701 cgtgcccagg ttgtcgttca ggatagcccg ttgcttgcca cgtcgcttgt ccttagccgt
23761 gttggcgtaa ttcacgttgt acggcgggtc ggtgaagacc atcgcagcct tcacgccgtc
23821 catcagcctg gcgaaactgt ccggatcggc ggcgtccccg cacagcagcc gatgagaccc
23881 caggatccag acatcgcccg gccgggagat cggtaccccc ggcacttcgg gcgccacgtc
23941 gtcctcggtc tgaccttcgg tgacggattc cgcgtcggcc aagagatcca gcagggcgtc
24001 ggcatcgaaa ccgatcgagg ccaggtcgaa gtcctcgtcc tgcagggcgg ccaactcgag
24061 cttgagcagg gcctcatccc agccggcgtt ctcggcgatg cggttgtcgg cgatcaccag
24121 ggcacggcgc tgggtggggc tcaggtgatc cagcacgatc accggtacct gggacaggcc
24181 cagctggcga gccgctgccc atcgcccgtg cccggcgacc atcaccccgt cgctcccggc
24241 caggatcggc gcggtgaagc cgaactcggc gatcgatgcg gcgatctgcg ccacctgatc
24301 atccgagtgg gtccgcgcgt tgcgtgcata cggcagcagt ttggtcgtgg gccactgctc
24361 gatcttgtcg gccagccagt tcatgccgcc acctccacat cacgttcggc agcgacctcg
24421 tcgaaggact ggccggtggc gatcaaagtc accggcacgc cggcatggtt ctgctggaag
24481 cgcttgatcg ccacatccac atactccggc gcaatctcca cgctgcgaca gatgcggccc
24541 gagcgctggg cagccagcat cgtcgtgccg ctgccgccga acggctcgaa cacgaggtca
24601 ccggcatcgg tgtaagcctc gatgacgaac ttcggcagcg ccaccgggaa caccgcggga
24661 tgatcgatgt cctgaccgat tttgcccttg tggcgcatca cgcgaatcac cgagtcgggg
24721 atgcgggtgt cctgcgtggg ctggcccttg tgcgtccagc cgccgacttc gccgtccttg
24781 ccgcgcatcg cggtggagct gccgtcctgg cggaggtgcg tctcctggcc cgcgaacttg
24841 cacggcacga tcttgtgagg tttcctcgct tgccggttga agtggaacac gaactcgaag
24901 ctcggtgcca agcgtcccgc ccagtcaccc ggcatccccg gcccctggtc ccagacgtac
24961 cagccgaagc ggcgccagcc ctggctccgc atccacccca gccaagcatc ccagtacggc
25021 atgacctcgt tgtcgcgatg gatcagtccc aggttgacca acacctgagc attgtcagtc
25081 agcggtattt gagcgaatat ggtgcgcatc aggccgtccc agtcgccgat gcccccgctg
25141 gtgtaatcgc gctgattgcc gtaaggcggc gaggtaaagc agagacgggc cagttcgccg
25201 gccatcaacg ctgcgacgac atccgggtcc gaggcatcgc cgcagatcaa ccgatgccgg
25261 cccaactgcc agacatcgcc aggccgcgac accggggttg ccggcggctc ggggacgtca
25321 tcggccgtct cgggttcgcc gtcttcggcg tcatccgaat cctcggtatc gatcgacagc
25381 agagcttcca gttcatcgtc gttaaacccc gttagcgcca ggtcgaaccc ggcaccggac
25441 aactcggcca gttccagccc cagcagttcc tcgtcccagc cggcatcgag cgccaaacgg
25501 ttgtcggcca gcacataggc ccgcttctgg gccggcgaga ggtgggcgag ttcgatcacc
25561 ggcacttccg ccagccccag gctgcgggcg gcggccaggc ggccgtgccc ggcgatgatc
25621 ccctgcgccc cgtccaccag gatcgggttg gtccagccga attccacgat gctggccgcg
25681 atcttcgcca cctgcgccgc gctgtgggtg cgcggattgc gggcgtaagg gatcaacggc
25741 tccaacggac ggtattcgac ctgcaacatg aagggttcgt agcaatccca aaaaagtacg
25801 gcccggcgcc gggtccgatc ggaacctgcg gcgaaccgta gacacgaggt cacgcagcaa
25861 caaaaaaccc gccgacggtg aaccggggcg ggtcttgggg aaatcaggtg gagccaaggg
25921 tgcaaacctg gatggcagca cccggtttac agggcgggtt tgcactgagg tttgcaccgg
25981 gggcgttggg cgctctcgtg tgagcgcctg taccggcgcg gcctccaggg tcgtgcgctc
26041 cggcaggggg tgcaaaccgc aaaccctgca aacctcggtt tgcggtctga cgctagcgaa

26101 atcccgcgtg cgttcccccc gtagccctca ggggccggga aggacccgca atcgttcgcg
26161 gcggaggggc atctcgcccc ttcggtcaca cctgctgtac tgtgagcgtt actctacgcc
26221 tgcaggcccc cagatgcgac aggccaaaag cctgccgttt tcgcagccgc ccgcagcgtt
26281 ccccggtgcc cctcaaatgc tatcgacttc ggttaaagct cctccatgca gtggaggtga
26341 tcgccggaga acatgggccg ttttacagac ggcttcgtcc aattcggctc tggaaccttt
26401 ccgcctggat taaccctttg tcaacgtctt caaggaaatc cgaaacagca cttctaccga
26461 ttttgaatta ggctgcgacg atcctgaact ccgttccctt ggcgatgggg cggacatgat
26521 ttttttcacg cttcatctct acgaggccgt aatgagacat cgtcttcagc gtgcgagaaa
26581 ggttgccggg tttcctgccc gtcgctgccg caagagacgt gatcgattca ggtttggttt
26641 ctctgatcac cttgagcagc accctgtttt catcgctcag gacctctgca agcgatctca
26701 tcgaagtgaa ccatatcttg ggatcccccg ctttgggctt gagttcgccc cgcgcgatgg
26761 ccagcagacg cttgcgaatg tcctcttgcg gcatgatgcc tatcacaatg gttttcatcg
26821 cttttttacc tccagcagaa cttggtccac ttcagaaaag aagtcagcca gcaattgatg
26881 tgcatccttg aattcatacg gaacgcccgg gtctctggca tgctggtgca tgtggtcgaa
26941 ggttaaacgt cgacccgcat acttgaactt cttcggcggt tttacggcgt gggcattgtc
27001 ataaccgagt attcttttgc cgtagggctc atgcagtgtc agggagtagc ggatgccatg
27061 aggtacatcg ggcgttggct caacctgata agcctcgatc ttgacccaat acccgctgcc
27121 ctgatcaatg atctgatcat ctaaatccag gagggtttca atgccagggt ccatttccat
27181 gcacatatta tatcagcggc tgataaagta tccatgcttt ggaaacgcta gccattgagc
27241 cggtccgcga cctgtcgcaa ggcctgctgc cagcgccgct gggcggtccg gttgcagcag
27301 gcgaaacgcc ggctgatctc tttccagccc tgccgctcgg cccgcatcca gatcagatgc
27361 cgctgctctt cctccagcca cagcacccag cgcatcgtct ccagcatgcg gtcgaccgcc
27421 tcggggctcg gctggatacg gcattcgcga gtttcgtcgg catagccctc ccaggcctgg
27481 cgggcgatgg ccggccagca gttgacatac ccttgcacct tgaccggcgg caggtcctgg
27541 gcggtccagg ccgcctcctc gaagcgggcg gccacgcgtt cgatcgtcca ctcagccacg
27601 gcgggactcc cggcggccgt acaaccgctc gccgatctgc cggatcagtt cccgctcgac
27661 gaaatccagc cgttcgtcag tcagatggac cacgaggata tgctgctcgc gccagccctc
27721 gcgtttgacc gcttcgaggt ccgtggcctg cggctgcagc tttcccagcg ggcaactggg
27781 atgcgggttc ggtccgttca tatgcccacc ctctgggtcg ccagggccca gtgcagcaag
27841 gcgagggcat cggcttcgtt gtcgtccacc ggatcgaaac ccattgaccg catcgcagcg
27901 accatagcgg ccttattggc gttgccctgg ccggcggcgt gtttcttgat cgttcccacc
27961 ggtacgccct ggtagggcat ccggtggtgc tcgcaccacg ctgtgaggtg ggccaggaac
28021 ccgccatagg cgtgggcggc atcgaccccg gcatggcggc ggacctcctc gaagtagacc
28081 gcatccagat cgccggcggc ttgcttgatc tccgtcagcc aacgtttgaa gcgcagaaag
28141 cgcatgccgc cgccttcgaa acggtgtggc ttgaaggttt cggagccgct gatgatcgta
28201 ccgtcccgct gatgcagggc ccagccggta tgcgtgccca gatcgagggc cagcagcgtg
28261 gtcccgtcac cttcgtcgtg acctccgagg ctggagagac ccctacgaga cggggagagg
28321 gcgcgtagcg cgcctctccc ccgtaggggg agggagtctc cgccatctgg aattccataa
28381 attgtttcgt atgaacaatc ggttgcgcca gatggcgagt tggcggcgct gccatctgga
28441 aattccgcca tctggaccgt aagcgattga agtctatgga ttcccagatg gcggacgttt
28501 gccatctgaa tccagatggc agatttcaag gggcagatgg cagagcgtct gccatctgga
28561 atcagggtcg tcgtcatgga ttgtcctcgt cgtggtaaag ccagatcgtg gagtcttcaa
28621 ccggcagcaa ggcgccggtc tgggggcatt tgtagtgggt ggggtagacc gaaaccggcc
28681 acatcgggat ttctcccgtg tcgggatcag ggggaccttc ccgccgcagc accatgccct
28741 cgacgcattg gtagccgaac ttgctgcggt gtaacggggg aagtccgtag tcctcggcgt
28801 tacgaaagaa cttgatgtaa ccttgagtgg cgagcaccga gagccgctcg cggatcgtgc
28861 gttccgcgcc caggccagcg cggccctcga aggcttcggc gaactgggtg gcgctgtagc
28921 agcggccttc cgccgcctcg tcgaacagca tctgcagaat cacgtcatgc ttgcgccggc
28981 gctcggcatc gaggcgttcg gcgtagtcct gcaggacccg ccgcccggtg ttcaccacct
29041 cgcgccaagt gccgttgaac ttgtcgacgg acttcaccgg aagcgccgcg ccattgcgca
29101 gctcgaagat gagttggcgg gtggtctggg cctcgtccgg ccggaacagc agcatcccgg
29161 tgctgtagta gcccctgagg ctgccggcgc cggccagggc ctggaacgga tcctcctcga
29221 agggtttctt cccgagcttc ttggtgtggt gcaccagcac gaggccggca tccgggttga

29281 ccgccatgcg cagctgttcc acccgctgcg acaggaagaa cagcatcgcg ccgttatcgt
29341 tttcgccccc ggcctcgccg ccgtcgaata cgttgcggat cggatcgatg gcgatcagat
29401 cgggcggagc gctgccgaat gcctgaacaa tggccgggat cacctgagcc aggccggcct
29461 cgtccaggac caggcgcagt tgcggggtgg cgacgaagtt gacgcgcgcg gcacccaggc
29521 gactgggcgg cagggagacc gtcttcacgc gctcgcggag gtagtggtac tgcacctccg
29581 cctgcagata gaacacccgc aagggtcgcg gcggcgtcat gccgaggaag ctggccccgg
29641 ccgccatgtg catcagccag gccagcagga agtcgctctt gccgaccttg ggcgccccgc
29701 cgaacaccaa gagacccccg ggcgtgagca cgcggggcgc gatcaggtcc ggcgggacgg
29761 gagagtcatc atcgagcaag gcgcccaagg tatgcgtcgg caataccggg ggtgcggcct
29821 tgatgacctg gcgctcgccg tggatgagga acgcgacgca atcgaagccc tcggacacgg
29881 catcggctgc gtcccacttg tctggcttgt ccatgggcgg tacgaggatg gcgaccgacg
29941 ccgcgcctgc cgccacgcag gcacgggcgg cgttctccgc gtagtcccag cccggcgcat
30001 ccctatccgg ccagatcagc acggccttgc cccggagcgg cgaccagtcg gtcttgtcga
30061 tcggcgcctt ggccccgttc atcgccgtgg tggcggccac acccttatgg atcagcgcgt
30121 cggcgcaccg ttcgccttcg accagcacca cctccttcgc ctgggcgatc gccggcaagt
30181 tgtagagcgg acgcgggtcg ggggcgcgcc acagccgggc acgcacgtcc caaggccggt
30241 attccttgcc ggtgggcggg tcgtagcggt agacgcaggc gatcagcgag ccgtccgccg
30301 cgacgtagtc ccacttcgcc gtggcgggac cgagccgatc caccggcgct tccccccgca
30361 gcggtgtcgg ttcgaccctt gccgtaccca acagccggcc ggccagggcc acgacctcgg
30421 cgaattggcc gtgcgtatcc aggccgtgga ccgcagcgat caaggcgaag aggtccccgc
30481 cctcgccggt cgcgtggtcg taccacagac cggccttctc gcccgaaagc gccacctcca
30541 ggctgtcgcc cggatcgccc tggacgttgc cgatcgcgaa ggcacccccg cgcaccttgc
30601 cggccgggaa caaggctttc agcaccgacg gcagccgggc cagcaaatcg gcgcgcacgg
30661 tttcacgctg ggctgccaga tcgacggggt tggccggtcg cggcagcgcg tcgttgaagt
30721 caagcaaggc cccgctcctt cggcgtgcgc ttcaccccgg ccccgtgcgc ggtctcgctc
30781 ggcagatacc cgcccttgcg ggcaatcgca cgcacgaacg ttgcctccag gccgaccgca
30841 tcgcaccaga actccaacgc ggagccggcg aggaagtgcc gcgcctcggc gcggaggacg
30901 gcgcccggat cgaggcagtc gccgatggcc agggtgatca ccgccaccgc caagtgggct
30961 tcggggcagg gaagggtcac atgccgacgc agcagcttgt cgatgaccgt cagacgcacg
31021 tgaggccggg gcaactgccg ctgcacccgt ttgccatcgt aaggctgccg ggtcctcatg
31081 ccccacctcc ccggcagcgc aggccccaag cacattgccg gcactcgacg tgggccggat
31141 cggcaaagcc ccgcggcaac agctcgccgg cctcggtggc ggtgatcacc ctgactgccc
31201 gatccgacat ccgctgtgcc agcccgccgt cgaaggggac acgctcggcg gagatctcca
31261 tcgagtccgc gttgacggcg gtgaacagcg ccggccgttc gtgcagcgcg aggtaggcct
31321 ggtacacggc gacctgggct gcgtaaaccg gcttggcctt ggccaagcga tgcttcacca
31381 gctcgcgcca ggatttcgcg cccaggcatt tgttctccca caaagccgga gcatcgaagc
31441 cttcgggccc accgacgatg acgccgtcca catggccctg gaggcggcca tccagcgccg
31501 agaacccgaa ctgctcgcca tcggcctgat gggtgcgcag atcgaagccc gctgcgcgca
31561 gccagcccac caccgcctcc tccatgcgat gaccgcgttc gaagatgcgc agcatccgcc
31621 cggaaaactc gcggccggga tcgaccggcg cctgggcgta ttcgaactga agcgcgcgct
31681 cgcaggccac gccgaggcga gacgcgccga gataagtacg gatgggttca ccggcctggc
31741 gacgctgcag accggcatcg atcagcgccg tgagccgttc gctgaacagg gcgccagggt
31801 tgtaatccag catggtcgcc ccctcagaac ggcacgtcat cgggatcgtg ttcccgcagg
31861 tggtcgaaat aggtcgtcag caccacgtcg accagttgca ggatttcctc ccggctgtag
31921 gccgacagcg gccggtccat gccgatggag gcgacgtagt cgccgaggcg gggcagcacg
31981 gccgccatcg ccgccttctc gttaggggtg ggatcgatca cgacgccgtc gctccgcttc
32041 agtcgttgta aatggatgtg ctggcagcgc ctcgagcaga agcgtttgaa caggggcttg
32101 ccgtccggcc tgcgcgggcc attcctcggc gatacccagc aaaagccgcg tccctctcgt
32161 ccgcagatcg cgcatatcac gccgccctcc ggtgctcgtc gttggccgcc agcacgagac
32221 gctggatgga cgacttgttg aactggaatg cgagcagcgc cgaggcctgg tatcgggtga
32281 gtccgaaatc ggcgcgctgg gccggcggca ggtagcgcag ctgtttctcg gtgggcggct
32341 cgttgagcca gcggcgggtc ttgtgggcag tgtcgaggga ctcgttctcg ttcagccagt
32401 cgtccgcctt ggccatgcag acggtgcggt cgccgaccgc cagcagacgg ggcagcaggt

32461 ccctgccgcc tccgaccgcg tgccagcggc cgttgaggaa gaagatcccg ccccaggcgc
32521 caaagccggt ggccatcaag gcgtcgtcgc agccgaatag atcgcaccag cggaagtggg
32581 agcgcttgag caggtcgatc tccgtcatga cgaattcgtc cagggcttcc gcctgagcgg
32641 cttcctcgcg gatccactcg aagccgcaca gggggcactc gcggcagccg agtggaactg
32701 tcgccgcgca gtatggacac gccttggtcg gcgcctcgcc ctggtgccgg tggccgtcca
32761 ggttggcctc ctgctccagc gagccatgca tgagagttgc cgtgccgaag tcgagcacga
32821 cgcaatcggt cttgaacaca ccgggatgtt cggccgggtc gacggtccgc agcccccgcc
32881 cgatcatctg ggtgagcgtc gacttgtggg agctcggacg cagcaggacg acgcaggagg
32941 tgggcgtgta gtcgtagcct tcggtcagca ccgccacgtt gacgacgagc tgcgcctcgc
33001 cggtttcgta ttcggcgagg cgtgtcttgc gctcggcgtc cgacagctcg ccgtggatca
33061 gtacggcgcg gatgccggcg gcgacgaagg cgtcggccac gcattgggcg tgggccacgg
33121 tcgagcagaa gacgatggtc ttgcgctcgc cggccttctc gcgccagtga tggatcaccg
33181 catcggtgat gggcgtcctg ttgaggatcg tctccacctc ggtcatgtcg aagtcggtgg
33241 cggtgcggcg cacctgggcg agggcggact gcgcgccgac atcgatgacg aaggtgcgcg
33301 gtggcaccag atggccggag gcgatgagct cgcccagggt gatctggtcg gccacgttgc
33361 tgaagacctc gcgtagcccc ttgccgtcgg cgcgtgcggg cgtggccgtg gcgccgaaga
33421 tccgcgcccg tgggttgcgc gacaatacgc ggtcgatcac gcgccggtag gatggcgagg
33481 cggcgtgatg cgcctcatcg atcaccagga ggtcgagcgt cggcatctgc tcgagatgct
33541 tgtctcgggc cagggtctgc accatcgcga aggtggcccg gccggcccag gacttctcct
33601 gggcgtcgaa caccgaggtg gtccgccccg gattgacctg gccgaacttg gcgcggttct
33661 gggcggtgag ctcgtcgcgg tgggcgagaa cgcaggcctt ggcgtccggc tcttccagca
33721 caccaccggc caccgccgac agcatgatgg tcttgcccga gccggtggga ccgatggcca
33781 aggtattgcc atgctgatgc agcgccgcga gcgagcgctc caccagcagg gcctgacggg
33841 gacgaagcat catggcgatg tcctccctta ctgggcccag gccggcttgc cgccggggat
33901 ggccgaagat gcggtcggcg ccgaaggcgc ataggccggc gttgccgctg cgggtatcgc
33961 acccgaaggc gaagttggga ccggcggagc gaagtgttgg cccatgagct gggcataatc
34021 tttgtgatcc ggctcgatca ccgcccggac cgtattgcgg tcattgccgc ggccgtcctt
34081 ctcgatgtcg atgcggccgg cgaactccag tccgtccagc tcggcgaagc cggcgatgcg
34141 gcgggcgttc tgggcttggg gcccggtgtc ggcgggatgg atgcggcggg cgctgttcag
34201 cagggaccga atgaagctgc ggcccatcgc ctgccaagtc ggccctttgg gtgagtgcag
34261 gccgatgttc caccacagct tgcggcgagc ataagggcca tccagcacca cgccttcgca
34321 agcgagatag acggcgccgg tctcaggact gcgggtggcc cagccgccgg tccagccctg
34381 gctggcatcg tcgaacccgc ccggcttgag ggtcaggcgg agccgcacca gggtatcctt
34441 cggaatcagc tcgaagctcg cctgctcggc ggaattgaag tcgaaatagc tcatggtcag
34501 gtctcctggg tcgggatcgt tacgggaaca ggacggctga aatccagccg ttccagcggc
34561 ggacgggcgg ggccggcgat cttctgcatc agctggccca ggtgcggcgg ttcgatcgga
34621 tcgagccggc cggagcggtc cttggcggga aatccccagg ggttcagggt ctggcagacg
34681 aacgcccggt agcggctgcc atcgtccgcg ggcagttcgg ccagggtgac cacttcatcg
34741 acgatgccgg ggagttcgag cccggtcttg gcgccgtcga tctgcagggc gaacacccgg
34801 cgattgaagt cgtcgagctt ctcgtccagg atcccgacga accagacgtt cttgttgcgg
34861 gtgtgctgca agtgcgtcag ccagccgatc atctcctggc ccatcaggcc gtaagcgccg
34921 cggctgtccg gcttgccggt cttctcggag taggcctgcg gctgcccctt gcaccattgc
34981 aggcacaagc gtccggcgac ggtgatcgag tcgacgaata cggtctggta tcgctccagc
35041 acaccggggt cgccgaagcg ggcgcacacc gcgtcgaagt gggctgggct gaaaggctgg
35101 tcatcccgca gggccggatt ggggccgccg atgaagaccg cgaagtcgcg gcattcgggc
35161 caagtgcgcg gccggatcgt atccccggcc cagccttcga ccgcgagatc gccggcttcc
35221 aggtcgaaga acagggtcga gccggcttcc agcgtccaca gttgcgaggt cttgccgagc
35281 ccggccttgc cgaccaggac gcctttgacc ccgcgccgtt cggccaagcg ctggtcggcg
35341 ctgatgatgg ggagggtcat accgcgtcct ccccgagcag cgccaaacgg aagccgggct
35401 tgccggtctt gagcgtacgg gccggtgcga agctctgctg gagcgcggcc ggccaggcgt
35461 tgaacttggt ctcggacacc cggtagctga tctcgacgta ctgggcgggg tcctcaccgc
35521 tgtcggcgat ccggcgcacc agggcggcga gttgccgggc atcccactcg accttcttgg
35581 ggaggtcggc cgtgatgcgc acggggccgt cgtcgaagtg gacgacgccg gtgtctttgc

35641 ccatagccaa ccgcagttgg cgggcgcgct cggcgtatct gatctccacg gcgcggtcca
35701 ggtgctcgtt gagggtcttg gcagcggcca gttgcgcggc ggcgtcgtgc ttgaggcggc
35761 acagcgattc taccggcagc tcggccagct ggccggccgg agtggcgagg agcgtgtcgg
35821 ggttcatcga ggtcataccg cacctccggc ctgaacgcat tggccggggc ttctgcggag
35881 gtggcgggcc tcgtaggcct cgatatcttc ctggcgatag agcacccggc cctgcagctt
35941 cagatagacc gggccgatgc cctcggaccg ccagcgatcc agcgtagcga ggctgacacc
36001 ccagcgttca gccagggccg ggggattcaa gtgtttgggg ctcac
//

```
LOCUS       IO1-R1                47296 bp    DNA     linear   PHG 16-FEB-2025
DEFINITION  IO1-R1.
ACCESSION   IO1-R1
VERSION     IO1-R1
KEYWORDS    .
SOURCE      .
  ORGANISM  .
            .
FEATURES             Location/Qualifiers
     CDS             complement(2..1018)
                     /ID="QSXAMBDO_CDS_0001"
                     /transl_table=11
                     /phrog="7301"
                     /top_hit="No_MMseqs_PHROG_hit"
                     /locus_tag="QSXAMBDO_CDS_0001"
                     /function="unknown function"
                     /product="hypothetical protein"
                     /source="PHANOTATE_1.5.1"
                     /score="-10958.721206969012"
                     /phase="0"
                     /translation="MTLSDISIRRPVLATVMSLALVLVGLMCFQRLPVREYPNIDAPVV
                     SVRSVYKGASSEVIESQVTRPLEDSLSGIEGIRTMKSVSREEVSQITLEFNVSRDQDSA
                     AADVRDRVSRVRARLPREMDEPVISKIEADAQPILWLAFSSDRHSPLEITDFADRYVQD
                     RLQTLPGVASVIIGGERRYAMRLWLNRERMAAHGVTVQDVEAALQKRNVELPGGRIESA
                     QREFTVFSETDLKTVEEFNALIVREVNGFLVRLSDIGRAELGAADERNIVRVNGNPAVA
                     LGVVKQATANTLEVARSVRAELPKILQTLPDGMTLEVSFDTSQFIDESIHSVYKTLAE"
     CDS             complement(1015..2097)
                     /ID="QSXAMBDO_CDS_0002"
                     /transl_table=11
                     /phrog="13642"
                     /top_hit="No_MMseqs_PHROG_hit"
                     /locus_tag="QSXAMBDO_CDS_0002"
                     /function="unknown function"
                     /product="hypothetical protein"
                     /source="PHANOTATE_1.5.1"
                     /score="-3040.9922764609864"
                     /phase="0"
                     /translation="MSNKSFGVLVLAASLAAIAGWWRHTATPPAAVPPADVRVPPSVPV
                     FVARVWAESVDDAIDAVGTLLADESVVIRPEVPGRVKAIRFTEGKPVRAGEVLIELEQD
                     EYRAALAQSLAQQELDQANFARMKAMRERAHVSAQQYDEAVSKLKYSNALVERDRVLLQ
                     KTVLRAPFDGIVGIRQVSVGEYVDKGKALVNLEALDPVKLDFKVPEKYAGTVTRGLALT
                     AEVAAYPGRVFSGQVYAVDPRLEEESRALRVRARLPNPDRALRPGMFARIHLALGAARR
                     ALFVPESALVVKGSSASVFRVVAGKAVATPVAIGARYKGKVEIVQGLAEGDSVVTEGQV
                     KLRDGAPVAVPEAKENPPAP"
     CDS             2142..2777
                     /ID="QSXAMBDO_CDS_0003"
                     /transl_table=11
                     /phrog="No_PHROGs_HMM"
                     /top_hit="No_MMseqs_PHROG_hit"
                     /locus_tag="QSXAMBDO_CDS_0003"
                     /function="unknown function"
                     /product="hypothetical protein"
                     /source="PHANOTATE_1.5.1"
```

/score="-85.60013802001805"
/phase="0"
/translation="MSGRSGQQLAQLTGAFQRSQIVEPAHVPVTDENLRHGAAVGDRHH
FLESGGIQVDPDLFDLHDPLGLQQPLGRRAERTHGCAVHADWEHNRRIGSVTGTVSEFN
TRPGPETPPDRSAVPLYKSMFRENIRNYWNDLVNTMTTERWVRIVAGFFILLSLSLGVE
ASPLFVNANWLWFTAFVGANLFQSGFTRFCPLESILRKLGVKSACGTE"
CDS 2780..3199
/ID="QSXAMBDO_CDS_0004"
/transl_table=11
/phrog="22"
/top_hit="No_MMseqs_PHROG_hit"
/locus_tag="QSXAMBDO_CDS_0004"
/function="DNA"
/function=" RNA and nucleotide metabolism"
/product="thioredoxin domain"
/source="PHANOTATE_1.5.1"
/score="-14.077794021484644"
/phase="0"
/translation="MQLVCPACLTRNRVPADRLGEQPKCGRCSTPLLSGHPVELDDGRF
DAYTRHSDLPVLVDFWATWCGPCRSLAPVVAQAADALNGRILVAKVDVDRAPATAQRFN
IRSVPTLVLFRHGQETRRISGALGFGPLMDWLQRG"
CDS complement(3147..3500)
/ID="QSXAMBDO_CDS_0005"
/transl_table=11
/phrog="No_PHROGs_HMM"
/top_hit="No_MMseqs_PHROG_hit"
/locus_tag="QSXAMBDO_CDS_0005"
/function="unknown function"
/product="hypothetical protein"
/source="PHANOTATE_1.5.1"
/score="-3.7482304815240894"
/phase="0"
/translation="MPCQGHELEVRAGSSASITAPSPISSAGGHLRADGQASFVRFSAE
ELYPMRSLAARARRGDRIRLVLGLGGQKAIGIKRTGVSMPSGRGPGNLPGRSFRCINRV
AANPSRGRSRALR"
CDS complement(3543..5000)
/ID="QSXAMBDO_CDS_0006"
/transl_table=11
/phrog="33847"
/top_hit="No_MMseqs_PHROG_hit"
/locus_tag="QSXAMBDO_CDS_0006"
/function="unknown function"
/product="hypothetical protein"
/source="PHANOTATE_1.5.1"
/score="-44201.876955887856"
/phase="0"
/translation="METLHMKNIPTSLVTLACLGFSAAAQAEGATEIVETVVETVQQAA
TPAAPTPDKGDTAWMIVATVLVTLMVIPGLALFYGGMVRAKNMLSVLMQVFVIFSLMAV
LWAVYGYSIAFTEGGVFFGSLDKAFLAGVTPDSTAATFSKGVVIPELIYVAFQLTFACI
TPALIVGAFAERMKFSAVLLFMVLWFTFSYLPIAHMVWYWAGPDAYTDAAAGEKATATA
GFLFQKGALDFAGGTVVHINAGIAGLVGCLLVGKRIGYKKESMAPHSVPMTMIGASLLW
VGWFGFNAGSNLEATGTAALAFVNTMLATAAATLAWSAVEWIARGKPSMLGGASGAVAG
LVAITPACGFIGPMGSIVLGLAAGAVCFWSVTTLKNALGYDDSLDVFGVHGVGGIIGAL

GTGIFASPALGGAGVYDYVANAVAEYDMVSQVISQSWGVGVTIVWSGVVSLIAYKIVDL
LVGLRVSEETEREGLDIREHGETAYHP"
CDS complement(5016..5354)
/ID="QSXAMBDO_CDS_0007"
/transl_table=11
/phrog="28307"
/top_hit="No_MMseqs_PHROG_hit"
/locus_tag="QSXAMBDO_CDS_0007"
/function="unknown function"
/product="hypothetical protein"
/source="PHANOTATE_1.5.1"
/score="-96.95567943361101"
/phase="0"
/translation="MKFVTAILKPFKLDDVREALSEVGITGITVTEVKGFGRQKGHTEL
YRGAEYVVDFLPKVKLEVAITDDQLEAVIDAITKTANTGKIGDGKIFVADLEQVVRIRT
GETGPDAL"
CDS 5457..5867
/ID="QSXAMBDO_CDS_0008"
/transl_table=11
/phrog="No_PHROGs_HMM"
/top_hit="No_MMseqs_PHROG_hit"
/locus_tag="QSXAMBDO_CDS_0008"
/function="unknown function"
/product="hypothetical protein"
/source="PHANOTATE_1.5.1"
/score="-13.89435856205492"
/phase="0"
/translation="MPREIKEQPRAKQTLVPKQIAPLLSNDRNITHQFKARRRTSGTYP
FRTSRRTAMIDKTTLDELSRRLADAVPTGLLGVRDELEKNFNAVLQSTLSRLNLVSREE
FDVQRAVLERARTRLGELEARLSELEKRLPPA"
CDS 5876..7393
/ID="QSXAMBDO_CDS_0009"
/transl_table=11
/phrog="10371"
/top_hit="No_MMseqs_PHROG_hit"
/locus_tag="QSXAMBDO_CDS_0009"
/function="DNA"
/function=" RNA and nucleotide metabolism"
/product="DNA helicase"
/source="PHANOTATE_1.5.1"
/score="-5983.281956744397"
/phase="0"
/translation="MALAIVHSRACQGIDAPEVTVEVHIAPGLPNLTIVGLPETAVRES
KDRVRSALVTTGFEFPAQRITVNLAPADLPKEGGRFDLPIALGILAASKQIRTEHLLAL
ECVGELALTGELRPVPGALPVALQSRRAERSLIVPWDNAGEAALVSGACVLPARHLLDV
CAHLDGSRPLAAAEPCTEASQASEEFPDLAEVRGQFQAKRALEIAAAGRHNLLMLGPPG
TGKSMLASRMPGILPDLTETEALETAAVASVSGMPFDPAVWRRPPYRAPHHTASAPALV
GGGSSPKPGEISLAHNGVLFLDELPEFDRRVLEVLREPLESGTITISRATQRLDFPARF
QLVAAMNPCPCGYLGDASGRCHCSAEQVARYRARISGPLLDRIDIHIDVPRQDPATLMD
GASPSEETSVQVRSRVTAARERALRRSGQPNALLTPRQIERHCAPDSAGRALLERAMTR
LNLSHRAYHRILKLARTIADLDGSEAITATHIGEAIGYRRLDRAAPR"
CDS 7390..9393
/ID="QSXAMBDO_CDS_0010"

```
                /transl_table=11
                /phrog="1967"
                /top_hit="No_MMseqs_PHROG_hit"
                /locus_tag="QSXAMBDO_CDS_0010"
                /function="DNA"
                /function=" RNA and nucleotide metabolism"
                /product="DNA helicase"
                /source="PHANOTATE_1.5.1"
                /score="-14099036.78121132"
                /phase="0"
                /translation="MNGLNPQQHAAVTTLDCPLLVIAGAGSGKTRVITEKIAYLIRQGT
                PARHIVAVTFTNKAAREMKSRVGKLADDKSLRGLTVSTFHALGLEIVRREHKALELKSA
                ISIFDEQDRQALLRELIRHGDHRFDPEQADAYAARISRWKNRFITPDRARSLDEPDAAG
                IAALYEAYMHHMKAYNAVDFDDLILLPVMLFQRHPEVLDKWRNQIRYLLVDEYQDTNQT
                QYELIKLLTGTLGKFTVVGDDDQSIYAWRGAQPENLAQLRNDFPRLKVIKLEQNYRSMG
                RILKAANQLIGNNPHIFEKKLWSAMGQGDPIRVLRHRDEIAEARQIAADIVHHKFRHNT
                RFADYAILYRGNHQSRLFERALREFSVPYHISGGTSFFGYTEVKDVMAYLRLLVNPDDD
                AAFLRIVNVPRREIGPTTLEKLGQYANRRHISLYAACFELGLEQSLPANAVQRLRRFGE
                HIADTADRTARGDVFKIIDDFLGTIGYENWVRENSSDAKAAERKFGNVRELLDWLRRIA
                EKDGENRSLAETVAKATLLDVLDRNQEDDTGDRVSLMTLHAAKGLEFPYVYLVGMEENL
                LPHQTSIDEDNIEEERRLAYVGITRARRDLTLSYCSHRKRYGEMIGCEPSRFLAELPQE
                DLEWPDRQPLDPETKRERGRASLAQLRNLLSD"
tRNA            9420..9496
                /ID="QSXAMBDO_tRNA_0001"
                /transl_table=11
                /trna="tRNA-Arg(CCG)"
                /isotype="Arg"
                /anticodon=CCG
                /locus_tag="QSXAMBDO_tRNA_0001"
                /source="tRNAscan-SE_2.0.12"
                /score="79.0"
tRNA            9554..9630
                /ID="QSXAMBDO_tRNA_0002"
                /transl_table=11
                /trna="tRNA-Pro(GGG)"
                /isotype="Pro"
                /anticodon=GGG
                /locus_tag="QSXAMBDO_tRNA_0002"
                /source="tRNAscan-SE_2.0.12"
                /score="69.7"
CDS             complement(9660..9791)
                /ID="QSXAMBDO_CDS_0011"
                /transl_table=11
                /phrog="No_PHROGs_HMM"
                /top_hit="No_MMseqs_PHROG_hit"
                /locus_tag="QSXAMBDO_CDS_0011"
                /function="unknown function"
                /product="hypothetical protein"
                /source="PHANOTATE_1.5.1"
                /score="-7.101449964965541"
                /phase="0"
                /translation="MILRHRLKTIFRGKVTQGNVRVAVNELDGISRFGHAGNKKSGR"
CDS             complement(9794..10036)
```

/ID="QSXAMBDO_CDS_0012"
/transl_table=11
/phrog="No_PHROGs_HMM"
/top_hit="No_MMseqs_PHROG_hit"
/locus_tag="QSXAMBDO_CDS_0012"
/function="unknown function"
/product="hypothetical protein"
/source="PHANOTATE_1.5.1"
/score="-0.316047657199731"
/phase="0"
/translation="VPNQELTHIRGDRRIRPASIRPQVGDVRAPDLIRPGRSEVALREV
VLRVGRDPETPQPFHALLAGRISLLRDSPSSSFPG"
CDS complement(10043..10162)
/ID="QSXAMBDO_CDS_0013"
/transl_table=11
/phrog="No_PHROGs_HMM"
/top_hit="No_MMseqs_PHROG_hit"
/locus_tag="QSXAMBDO_CDS_0013"
/function="unknown function"
/product="hypothetical protein"
/source="PHANOTATE_1.5.1"
/score="-2.1321637138343683"
/phase="0"
/translation="MARNPVSTAPWAQICAKTNFRSNISQRFDLSGEVQICPG"
CDS complement(10206..10967)
/ID="QSXAMBDO_CDS_0014"
/transl_table=11
/phrog="4"
/top_hit="No_MMseqs_PHROG_hit"
/locus_tag="QSXAMBDO_CDS_0014"
/function="transcription regulation"
/product="transcriptional repressor"
/source="PHANOTATE_1.5.1"
/score="-2702.1904047346"
/phase="0"
/translation="MTSEINSEIGAGSSEEWFSAADLARMVLPGWPKSDRGWRDVLERQ
HWPFREVPSKGGKRGMKREYQPPPEVLALIRQKLQGEPQAAHAYPTKPGGLNGDKHLQR
QPAVYSPDDYVALPLYDVRAAAGGGVVPDTENVVDFLHFKKAWLRTELRSSPDDLYLIY
VDGESMEPTLCKGDVILVNHRDKFQGRDGGIYVLRLDGALLVKRLQRKMGGIIKVTSDN
PVYEPFEVGAQDLDRADFSIIGRVVWAGRRM"
CDS 10995..11429
/ID="QSXAMBDO_CDS_0015"
/transl_table=11
/phrog="925"
/top_hit="No_MMseqs_PHROG_hit"
/locus_tag="QSXAMBDO_CDS_0015"
/function="DNA"
/function=" RNA and nucleotide metabolism"
/product="HTH DNA binding protein"
/source="PHANOTATE_1.5.1"
/score="-55.16844475719332"
/phase="0"
/translation="MILGYFIYFHLSAYLGAARKQKNLVDGSSELQYAMSNSRTNTDTT

QPRKRAMSNTNQAAGAQANPFCAFIEAQGGTITPDQFRSWLALHGITMADWARERGFRP
REVSLVLNGQIKARYGRSFSIAVAMGLKPDPNRAQKQHAA"
CDS 11530..11970
/ID="QSXAMBDO_CDS_0016"
/transl_table=11
/phrog="2345"
/top_hit="No_MMseqs_PHROG_hit"
/locus_tag="QSXAMBDO_CDS_0016"
/function="transcription regulation"
/product="transcriptional regulator"
/source="PHANOTATE_1.5.1"
/score="-34.75382359727734"
/phase="0"
/translation="MNAAQQDLFGGGAVHLPQPIPIAPGRFALGDRVAVAMSRAIDASG
VDRPDLASRITALTGKRLPLSVLNAQTAMSRPDHTPSLLQAMAFDAVTGKWALLELYAE
AAGGKVIYGDDIAAFEVGRAVMVKKLAGQREREALRSVGVGR"
CDS 11967..12059
/ID="QSXAMBDO_CDS_0017"
/transl_table=11
/phrog="No_PHROGs_HMM"
/top_hit="No_MMseqs_PHROG_hit"
/locus_tag="QSXAMBDO_CDS_0017"
/function="unknown function"
/product="hypothetical protein"
/source="PHANOTATE_1.5.1"
/score="-2.257446008745866"
/phase="0"
/translation="MSGERKPQGFRAIQHHRASAVRNGKALAEV"
CDS 12062..12253
/ID="QSXAMBDO_CDS_0018"
/transl_table=11
/phrog="No_PHROGs_HMM"
/top_hit="No_MMseqs_PHROG_hit"
/locus_tag="QSXAMBDO_CDS_0018"
/function="unknown function"
/product="hypothetical protein"
/source="PHANOTATE_1.5.1"
/score="-3.986979611305401"
/phase="0"
/translation="MMSLLEVTHARAFSVAAAMKESHLLELKSRIDLLVATGASFREAE
TTLRPLCAAIQKDREESV"
CDS 12250..12666
/ID="QSXAMBDO_CDS_0019"
/transl_table=11
/phrog="No_PHROGs_HMM"
/top_hit="No_MMseqs_PHROG_hit"
/locus_tag="QSXAMBDO_CDS_0019"
/function="unknown function"
/product="hypothetical protein"
/source="PHANOTATE_1.5.1"
/score="-3.8237886164479984"
/phase="0"
/translation="MKELPAASLARATRMACCWRSSNTRQAPAEMCSQLLSGSSIRSSI

AASASGSKEASYGRERMRVRLSRNRSLNGHSASTPCHPVMNCGSSDSREQSSARSVKTR
NRSKFFIEGSSVMVVWQRQFKAGRNPRQFGMGRA"
CDS 12663..14975
/ID="QSXAMBDO_CDS_0020"
/transl_table=11
/phrog="310"
/top_hit="No_MMseqs_PHROG_hit"
/locus_tag="QSXAMBDO_CDS_0020"
/function="integration and excision"
/product="transposase"
/source="PHANOTATE_1.5.1"
/score="-192624960.1904547"
/phase="0"
/translation="MSAIKTHYSCAELAALKLPGYPGSERGFRDLVEREGWAFREASTK
GGKNGMRREYQPSAAVMKLIQAREKEAQRQASLAAELAALESSPEYRAGQAIARQMAEA
SAREAEARQQDDEALLALFAQATGERGERGRAWLATIQCWRRFYREKFGDQKRSQEKAL
IAFIKAFNAGALDLDGEVYAPVLKSSGRGKNAQGKGLSLRTLKRKLGLIKKSNIGALLD
NHKSPRRGDSLIHQQPRLEQYMLGLLAQQPDIAAVRLHEMAENRFAGDATIKVPSYSAV
DRYVKNWKAKNASAYLHLRNPDAWKNKAMPAVGLADGDVVRLNQRWELDSTLGDLLLSD
GKRHAVIGVIDVYSRRLKLLVAPTSKARAIAAVVRRAMLDWGVPEQAKTDNGADYASDY
LTGIWDAFGVEHVLCPPFSPEYKPHIERALGTFNHDLVELLPGYIGHDVAERKAIEARK
SFARRLMEKDGVLEVRLSPEELQKFCDDWTENRYHHRPHAGLEGETPFQRAASYRGEIR
RIHNERALDILLSPPVDGKTRSLGKKGLEIHGGFYSHPELWRHHTTGDAFRVMVDETDL
GRVYAYDDNGFVCVAICPERLGLPPEAVSRHAMECKRMAAQQRNQGMKTLNRAKKGVDL
NAEINSYLRSKAESAGKLATFPQLSTGYTTPALEGAARAAESTDRPPRSPEAEKLSAEA
KAHFAASGRSAEIIKLPKRKEHPLERMSDEEKYEFWCSLDAAVKAGGGELPDDAQVRRF
YAEFPLSARFKAQAAIR"
CDS 15039..15791
/ID="QSXAMBDO_CDS_0021"
/transl_table=11
/phrog="296"
/top_hit="No_MMseqs_PHROG_hit"
/locus_tag="QSXAMBDO_CDS_0021"
/function="integration and excision"
/product="DNA transposition protein"
/source="PHANOTATE_1.5.1"
/score="-1204.2099503887976"
/phase="0"
/translation="MTDKPENDKSTPAGIAPLTNIAIAERAMALAMGRSMHQSGLVVLH
GPSGYGKSMAAAWVKARSRAYYLQLDDFTRSRKVLLKRLGKALGMQLAKSATVDEMADA
VMAQLEQSGRPLIIDEADYLETFKLVDSIRSIYEGSKAAILLIGEEALPFKLKEWERFD
GRILDRFPAQPVSLPDARELAGLYCPGKVMGEDLLERLVEVAKGSVRRVSTNLERMYEE
ALANGWDRIDLATWGNRPIHTGEAPRRG"
CDS 15791..16378
/ID="QSXAMBDO_CDS_0022"
/transl_table=11
/phrog="2030"
/top_hit="No_MMseqs_PHROG_hit"
/locus_tag="QSXAMBDO_CDS_0022"
/function="transcription regulation"
/product="transcriptional regulator"
/source="PHANOTATE_1.5.1"
/score="-191.05798778746873"

/phase="0"
/translation="MARKPVTIELAGGKSPRQRIWEMIRKLRIFTVPELRGHLPGPVPL
ATVRTYVESLHAAGILEKTPGLYELIDDRGIEAPRVNKAGQPVTLGQGNERMWGAMEAL
GAFNCRVLARMADVPLATVKTYCAYLQRAGFLTVERAGKGRGAGGVPTTYRLLHSRITG
PRPPMITRLKTVYDPNVGKIVWQQDPQEQLDD"
CDS 16371..16709
/ID="QSXAMBDO_CDS_0023"
/transl_table=11
/phrog="2119"
/top_hit="No_MMseqs_PHROG_hit"
/locus_tag="QSXAMBDO_CDS_0023"
/function="unknown function"
/product="hypothetical protein"
/source="PHANOTATE_1.5.1"
/score="-12.17377447008296"
/phase="0"
/translation="MTEERRERALKLLEAAVAAHGSFAAVAKLLGLNRATISTVARRCY
PGDDAKVLARVLEQFDQIACPYLRRPMAPEECRSVWSGATPSHDPALLAHRRACRSCPH
KGDGHAHS"
CDS 16696..16953
/ID="QSXAMBDO_CDS_0024"
/transl_table=11
/phrog="No_PHROGs_HMM"
/top_hit="No_MMseqs_PHROG_hit"
/locus_tag="QSXAMBDO_CDS_0024"
/function="unknown function"
/product="hypothetical protein"
/source="PHANOTATE_1.5.1"
/score="-5.878493523287598"
/phase="0"
/translation="MHIRDRIILAPTPAWFDAALDRGETRALRVDIAPAEIAGHSVFHL
PFRPDRADWYRTFQVARAFLRGAAFRGGAAPTETGREVHG"
CDS 16946..17281
/ID="QSXAMBDO_CDS_0025"
/transl_table=11
/phrog="1035"
/top_hit="No_MMseqs_PHROG_hit"
/locus_tag="QSXAMBDO_CDS_0025"
/function="DNA"
/function=" RNA and nucleotide metabolism"
/product="endonuclease VII"
/source="PHANOTATE_1.5.1"
/score="-13.209997189383207"
/phase="0"
/translation="MAEISTKRCTRCGQTLPKDRFYRQGVRLEATCKACVNAARTAKRR
AAGIAPRPPAKPRQPDYSPEVWDRELRAVNRAFNHTLKTIFGSLHRCNPHPTLSRREMA
LTQGEHA"
CDS 17278..17511
/ID="QSXAMBDO_CDS_0026"
/transl_table=11
/phrog="No_PHROGs_HMM"
/top_hit="No_MMseqs_PHROG_hit"
/locus_tag="QSXAMBDO_CDS_0026"

/function="unknown function"
/product="hypothetical protein"
/source="PHANOTATE_1.5.1"
/score="-6.189518223736422"
/phase="0"
/translation="MIKIQRYPRLGAYGWKALVTVGGKAREVLTVGTWFDIRRLARRAE
EEMAEKPRRSPCVDAARQRQLDVRRGLQGAAP"
CDS 17508..17669
/ID="QSXAMBDO_CDS_0027"
/transl_table=11
/phrog="No_PHROGs_HMM"
/top_hit="No_MMseqs_PHROG_hit"
/locus_tag="QSXAMBDO_CDS_0027"
/function="unknown function"
/product="hypothetical protein"
/source="PHANOTATE_1.5.1"
/score="-0.6866293727233725"
/phase="0"
/translation="VKAIVLACLAFIYLIGLGTGLLLGAVLDINLVGRAERKAQARQSD
TDDINLGI"
CDS 17678..18175
/ID="QSXAMBDO_CDS_0028"
/transl_table=11
/phrog="491"
/top_hit="No_MMseqs_PHROG_hit"
/locus_tag="QSXAMBDO_CDS_0028"
/function="DNA"
/function=" RNA and nucleotide metabolism"
/product="Mu Gam-like end protection"
/source="PHANOTATE_1.5.1"
/score="-235.9112982030016"
/phase="0"
/translation="MARIRIEGTQFSSWDDVDQALREIGEIERDLSLIDGDVNDQIDRL
KAAAKTQAQPLLDRKTALELAMKEFCEANRAEFAKVKTRQLVFGSVGFRLSTRVLIKRV
ADTLQALKDLGLHGCIRLKEEIDKEALKTLPAETLAEVGAGLKTDNVFGYEIDRARIAE
AA"
CDS 18209..18496
/ID="QSXAMBDO_CDS_0029"
/transl_table=11
/phrog="379"
/top_hit="No_MMseqs_PHROG_hit"
/locus_tag="QSXAMBDO_CDS_0029"
/function="DNA"
/function=" RNA and nucleotide metabolism"
/product="DNA binding protein"
/source="PHANOTATE_1.5.1"
/score="-11.816437176446753"
/phase="0"
/translation="MNQSQLINAIHEDANGSLTKTVIQRVLIALANVAQEALANGDEVT
IPGVAKLTVKERAARTGRNPQTGETVEIPAKRVARASFVKALKERVQGGA"
CDS 18502..18933
/ID="QSXAMBDO_CDS_0030"
/transl_table=11

/phrog="286"
/top_hit="No_MMseqs_PHROG_hit"
/locus_tag="QSXAMBDO_CDS_0030"
/function="unknown function"
/product="hypothetical protein"
/source="PHANOTATE_1.5.1"
/score="-31.017509336240444"
/phase="0"
/translation="MARTDPVRRQLVAIHVLKSKAGMDDSSYRAMLREIGGASSSKDLT
ATGRAKVLDHLRRITQPPVAKPRSRPYPGRPHTTDQRPLLQKIEAQLTEAGRPWNYAVG
ILKRVSRGRAERLEFATDEDLRKVVAALTYDARRHGGTT"
CDS 18930..19136
/ID="QSXAMBDO_CDS_0031"
/transl_table=11
/phrog="No_PHROGs_HMM"
/top_hit="No_MMseqs_PHROG_hit"
/locus_tag="QSXAMBDO_CDS_0031"
/function="unknown function"
/product="hypothetical protein"
/source="PHANOTATE_1.5.1"
/score="-1.619332391608152"
/phase="0"
/translation="VSLNLLVVIVFVAGFGWGQWWAERQIRIERASLEIGRLSVSMPMN
PDDAAATFGLLKTLMEQVERERK"
CDS 19133..19741
/ID="QSXAMBDO_CDS_0032"
/transl_table=11
/phrog="270"
/top_hit="No_MMseqs_PHROG_hit"
/locus_tag="QSXAMBDO_CDS_0032"
/function="DNA"
/function=" RNA and nucleotide metabolism"
/product="replication initiation protein"
/source="PHANOTATE_1.5.1"
/score="-80.11619854028497"
/phase="0"
/translation="MSHTKRSFIQACLIRALPALDRVDAGIAHAEALWERLTAKGYGAP
RQTGPRESVDWYARLVEPSRGWFDQFWTAYGLKRDRNGAAMRWYQLGDLTEHEARRIID
AAKQDNRQWRETAQPGQVRKMAQGWLHEKRWMDYAPTPQPPLSGGYSAGLAGDAQLREL
KQQLASLQRLNAAAPSKELQRQINELVQEIGNFQRPGHG"
CDS 19734..20084
/ID="QSXAMBDO_CDS_0033"
/transl_table=11
/phrog="532"
/top_hit="No_MMseqs_PHROG_hit"
/locus_tag="QSXAMBDO_CDS_0033"
/function="transcription regulation"
/product="late transcriptional activator"
/source="PHANOTATE_1.5.1"
/score="-33.05865500106161"
/phase="0"
/translation="MAEKKGRQARELLSDLAAKSAALACELGALPKDVAERFGLELSRE
VAAHWGGQLIYIPRNLPGELDRRDMEIFEKFRGDNIAELAREKGLSVQWIYRIIKRVRA

AEIERRQQRLFD"
CDS 20100..20738
/ID="QSXAMBDO_CDS_0034"
/transl_table=11
/phrog="8809"
/top_hit="No_MMseqs_PHROG_hit"
/locus_tag="QSXAMBDO_CDS_0034"
/function="unknown function"
/product="hypothetical protein"
/source="PHANOTATE_1.5.1"
/score="-270.54232220049954"
/phase="0"
/translation="MKRFLWGAVLAGAVFGACAEEWNGKVVGISDGDTLTIMKDSGGTK
VRLVEIDAPESRQDYGAKSKQSLSDLCFGKVAKVDDQGADKYGRRLGRVHCAGVDANLE
QITRGLAWFYVEYGHDPAMRDAEEQARASRIGLWADTNPTPPWDFRHGTPKKAARSTDE
ADGSASDCGGKSTCKQMTSCAEARHYLNDCGVSRLDRDRDGIPCESICR"
CDS 20807..20974
/ID="QSXAMBDO_CDS_0035"
/transl_table=11
/phrog="No_PHROGs_HMM"
/top_hit="No_MMseqs_PHROG_hit"
/locus_tag="QSXAMBDO_CDS_0035"
/function="unknown function"
/product="hypothetical protein"
/source="PHANOTATE_1.5.1"
/score="-0.1003281921959351"
/phase="0"
/translation="LKPLNQSIPCRPKARLVRALFLIRFKRRGAIRAVLCAHIGSWVRR
AASWANSGRG"
CDS 20950..21288
/ID="QSXAMBDO_CDS_0036"
/transl_table=11
/phrog="2609"
/top_hit="No_MMseqs_PHROG_hit"
/locus_tag="QSXAMBDO_CDS_0036"
/function="lysis"
/product="holin"
/source="PHANOTATE_1.5.1"
/score="-20.155188683467788"
/phase="0"
/translation="MGKFWAWLMTAAAKAWPILWRYIAPMLADGMRQQWEILLPVAERM
VRAVEDSLSWAPKSGEAKKSAAIALILKELKEREIAWADRIPDRMMNRAIEMAVGLLPE
KQSAETAP"
CDS 21378..21524
/ID="QSXAMBDO_CDS_0037"
/transl_table=11
/phrog="No_PHROGs_HMM"
/top_hit="No_MMseqs_PHROG_hit"
/locus_tag="QSXAMBDO_CDS_0037"
/function="unknown function"
/product="hypothetical protein"
/source="PHANOTATE_1.5.1"
/score="-3.7189278591075654"

/phase="0"
/translation="MQFIVQGIGIAGVGHKIEKAATPAPLPQGRGEDMSDEEFAAFVRR
QQK"
CDS 21521..21946
/ID="QSXAMBDO_CDS_0038"
/transl_table=11
/phrog="873"
/top_hit="No_MMseqs_PHROG_hit"
/locus_tag="QSXAMBDO_CDS_0038"
/function="head and packaging"
/product="endolysin"
/source="PHANOTATE_1.5.1"
/score="-43.6418161789316"
/phase="0"
/translation="MSFQKPRGLRNNNPGNLVYSPRNAWEGQVGHDGRFARFEQMEDGV
RALGVTLLNYQRKRQLHTVRQIITRWAPPNENDTATYIRRVAAALDVGADDRIILSDRK
TLTLLVWAISTHENGAMACERWLKHEDVEAGVDRALR"
CDS 21906..22226
/ID="QSXAMBDO_CDS_0039"
/transl_table=11
/phrog="No_PHROGs_HMM"
/top_hit="No_MMseqs_PHROG_hit"
/locus_tag="QSXAMBDO_CDS_0039"
/function="unknown function"
/product="hypothetical protein"
/source="PHANOTATE_1.5.1"
/score="-8.011264374356204"
/phase="0"
/translation="MRMWKRGWTVRCVSGFPGAAILALPLTLAGCVQVGPKELVPKPIL
TPAFAPESCALPDLPPLASTVFVDIAPGRPPKADAGGKALVVGYGRAREAIQACKGGAE
GH"
CDS 22226..22444
/ID="QSXAMBDO_CDS_0040"
/transl_table=11
/phrog="No_PHROGs_HMM"
/top_hit="No_MMseqs_PHROG_hit"
/locus_tag="QSXAMBDO_CDS_0040"
/function="unknown function"
/product="hypothetical protein"
/source="PHANOTATE_1.5.1"
/score="-6.541503437698388"
/phase="0"
/translation="MLTPQEKCVGGGALLMLAFCIGVLAGCDCLACEPTGYAKFADTLS
VLHGERPIEFGAGLQCEFTERTDAGSR"
CDS 22428..22697
/ID="QSXAMBDO_CDS_0041"
/transl_table=11
/phrog="54"
/top_hit="No_MMseqs_PHROG_hit"
/locus_tag="QSXAMBDO_CDS_0041"
/function="other"
/product="DksA-like zinc-finger protein"
/source="PHANOTATE_1.5.1"

/score="-13.849397878223416"
/phase="0"
/translation="MPDLADLSDERIEIERVAGIRAVLDSMQPDTPQVVIPDENGVPQV
VCYDCLNEYEVITPIPPDRLAANEYAIRCIDCQIAYEEEKRRHA"
CDS 22690..23046
/ID="QSXAMBDO_CDS_0042"
/transl_table=11
/phrog="736"
/top_hit="No_MMseqs_PHROG_hit"
/locus_tag="QSXAMBDO_CDS_0042"
/function="unknown function"
/product="hypothetical protein"
/source="PHANOTATE_1.5.1"
/score="-47.50827216729887"
/phase="0"
/translation="MHEFMRDNWNVISDIAHFAAEIAIGAWLYLSRKNDRTQWRIEALQ
SHVDQRLDEQSRRLAQVETDVRHAPTHDDLGKIYDRINTVGGDVREMKGVLESAAKTVD
RLQSYLLEHDRKGS"
CDS 23043..23339
/ID="QSXAMBDO_CDS_0043"
/transl_table=11
/phrog="436"
/top_hit="No_MMseqs_PHROG_hit"
/locus_tag="QSXAMBDO_CDS_0043"
/function="unknown function"
/product="hypothetical protein"
/source="PHANOTATE_1.5.1"
/score="-12.00953976192116"
/phase="0"
/translation="MNYEDFITEERRLVILRRLVQAPAYTLDQTVLTKALALEGLAVSR
DRLKTDLAWLAEQDLIVGQQPGGVWVATLTVRGLDVAQGLTVVPGVARPEPGE"
CDS 23339..23890
/ID="QSXAMBDO_CDS_0044"
/transl_table=11
/phrog="426"
/top_hit="No_MMseqs_PHROG_hit"
/locus_tag="QSXAMBDO_CDS_0044"
/function="head and packaging"
/product="terminase small subunit"
/source="PHANOTATE_1.5.1"
/score="-86.03180737043365"
/phase="0"
/translation="MPPRSLVYQLPPEVQEELNTRLVGSGFGGYRDLSAWLKDQGYHIS
KSALHSYGQEFELEFNACMADARKAQELARAAVAQRDDSGAALLEATSDVAQEILLKLM
IGLRKADHEPDKAAKLVSLTVRSLADLGRMTIDQKKWQEQIRRQASEEAGQIAEKAAAR
AGVSKDGIAAIRAAILTEMA"
CDS 23887..25386
/ID="QSXAMBDO_CDS_0045"
/transl_table=11
/phrog="2"
/top_hit="No_MMseqs_PHROG_hit"
/locus_tag="QSXAMBDO_CDS_0045"
/function="head and packaging"

/product="terminase large subunit"
/source="PHANOTATE_1.5.1"
/score="-684186.8019208096"
/phase="0"
/translation="MSTAVLLPYQQRWIEDRASVKVCEKSRRIGLSYGEAADSVLHASA
EEGGGNVYYISFDKEMTQGFISDCAGWAKAFHAGASEIGEEVLADPKNPARSILKYSIS
FASGKVIHAFSSNPRNLRSKGRPGDRLVIDEAAFVDDLGELLKAAMAMTMWGGEIHIIS
THNGDDNEFAVLINDIRAGRYDYSLHRIDLDDALGDGLYRRICQVSGQIWTPEKEAEWR
AALIKRYRPNEDEELFCIPAFGGGSYLPRALVETCMVDAPVVRFAGSRTFNMAPEPARR
ADMREWIDAELPSLLEGLDRRRRHTLGMDFARSSDTTVIAPMEIGDTLHRRVPFLVELH
NVPHKQQEQVLFALADALPRFCAGAIDASGNGSYIAESAADRYGSRIEQLKFTEDWYRE
NMPKYKAAFEDRLISIPRHDDVLEDHRAIKVVRGVPRVPEGKTDKKGQRHGDSAIAVAL
AYYASYSASGEMPMVVSRARTGTVLSGYGGIDCGASTRGYM"
CDS 25545..26885
/ID="QSXAMBDO_CDS_0046"
/transl_table=11
/phrog="113"
/top_hit="No_MMseqs_PHROG_hit"
/locus_tag="QSXAMBDO_CDS_0046"
/function="head and packaging"
/product="portal protein"
/source="PHANOTATE_1.5.1"
/score="-74412.52031746609"
/phase="0"
/translation="MGKSIEVYRDLLSDAHVAGCVRRRKAAVRAMERGVERKDASARTV
KNVEAILADLPLARIIGEILDAVLYGYQPLEIVWERVGNFVVPGDVIGRPPEWFVYSQN
NELLFRSQSCPMGEPLPPNSFLCARQEPTYFNPYGAPDLALVFWPTTFKRGGLGFWMQF
AEKYGMPWPVGKYPRGAEPGEIDQLLDSLEQMVRDGVAAVPDDSSVEFLTHDQSQGADA
YEKLLLFCRSEVSIALLGQNQTTEVSANKASAEAGIEVTRDIRESDAEIVASTLSDLIR
MVCELNFHEPAPPVYQLWEQEEIDETRAVRDKALYDAGVRPTNAYWMRAYGFKEGELAP
EASPSESYGAPAFAGIDLDLFPDQAALDAAVDALAEGGALQAQAERLLAPLIERIESAG
GEAELLGALAEAYPDMNPDQLVQTLTRLLFSASVAGRLATEESLQNG"
CDS 26878..27246
/ID="QSXAMBDO_CDS_0047"
/transl_table=11
/phrog="No_PHROGs_HMM"
/top_hit="No_MMseqs_PHROG_hit"
/locus_tag="QSXAMBDO_CDS_0047"
/function="unknown function"
/product="hypothetical protein"
/source="PHANOTATE_1.5.1"
/score="-27.14064414948692"
/phase="0"
/translation="MGKYLPDIRQGDTYRVRIRYPKGSNIAGYIHWLALRTAIGTEPPL
LLLRSVVGEHPSDQPEDGIAYLEATPEQTAALPANKRCVYGLLAKTPSGEVKTLRPPLK
DPLDDRILIVPRGGADGV"
CDS 27291..28829
/ID="QSXAMBDO_CDS_0048"
/transl_table=11
/phrog="2647"
/top_hit="No_MMseqs_PHROG_hit"
/locus_tag="QSXAMBDO_CDS_0048"
/function="head and packaging"

/product="head decoration"
/source="PHANOTATE_1.5.1"
/score="-6265.616616330495"
/phase="0"
/translation="MSRTRRGREAPSVPKVRLVRRDRLAGSIRIPSSTAGIFDLNSRET
NHGKRSTHQTQGKRQRRRAGRSPQRRARLQRSRRRPLLRQRRFGSNATSIPAIGGPGAF
VTKSTAQTIDGAKTFTGANDLGAQTTATTPAAADNSNKVATTAFVKGQNYLTGNQVVTL
TGDATGSGATSIAVTIPNDTVSNAKLANMAAGTLKGNNTGSAADPVDLTVAQVKTLLAY
QATDITGFDTLVRTSRLDQMAAPTAAVSMNSQRITNLADPTGAQDAATKAYVDSVASGL
DWKQSVRAATTANITLSGTQTVDGVALVAGDRVLVKNQTTAAQNGVYVVASGAWSRATD
FDSNAEVTAGACMFVSEGTTNGDKTWVLTTNDAIVLGTTALAFAQMSGGASGEVNTASN
QGASGVGVFDNKVGVDLQFRNLVAASSKIAVTLNAGNKTIEIDLAQANITAVGTLTAGV
WQGTAIGLQYGGTGANLSAAADDTIFKKSGTALVAATAGSDYLNNASTIDGGTF"
CDS 28842..29531
/ID="QSXAMBDO_CDS_0049"
/transl_table=11
/phrog="1699"
/top_hit="No_MMseqs_PHROG_hit"
/locus_tag="QSXAMBDO_CDS_0049"
/function="tail"
/product="long tail fiber protein distal subunit"
/source="PHANOTATE_1.5.1"
/score="-97.69747481993069"
/phase="0"
/translation="MTNTVQFKRSNTAGSNPGALASGEPAVNLADHRLFLGGAAAEGYP
LGCQSRPSPRLSGKPKVAGAGGGTKLASATVTAQQQYFVPFVVPRPMMLSQLGAYVVVN
QFSTVIHVGIYGNLFYSGEDHPGPIIAAVTNLSSATTGLKSGSVSATLEPGRLYWASVL
SSGSPGLIGIVTAAANGDYAVSDSDYMTAITHQYLSGVSSMADYASGALGAVVSGTGTA
PAVYLYE"
CDS 29564..29854
/ID="QSXAMBDO_CDS_0050"
/transl_table=11
/phrog="No_PHROGs_HMM"
/top_hit="No_MMseqs_PHROG_hit"
/locus_tag="QSXAMBDO_CDS_0050"
/function="unknown function"
/product="hypothetical protein"
/source="PHANOTATE_1.5.1"
/score="-7.712062532008155"
/phase="0"
/translation="MFNTVSSLSKPLIQSGQVGTTPVEIDLLQHRPPFRITLIPGSGNT
SLCEIKHAGEWFPWPFGNITSKATDVQHGPIEAVRFTRVSGSSTDTYGVMA"
CDS 29851..30837
/ID="QSXAMBDO_CDS_0051"
/transl_table=11
/phrog="11262"
/top_hit="No_MMseqs_PHROG_hit"
/locus_tag="QSXAMBDO_CDS_0051"
/function="tail"
/product="tail protein"
/source="PHANOTATE_1.5.1"
/score="-453.1820617906045"
/phase="0"

/translation="MSGWESERIARRSFEKSNSLTPAWIAPGGWALAVGQEFYAEVGGR
NLIVPANRDIILPESPAPGTDYAIWLSPNGVVQASSNFVTPPNTSVDRKIGGFHYAPGG
NAPAQAGGNTTPAINPYSMWDLNFRPACPDPRGMTLVADAFWIDIYLLGVDHLVNGSSK
YNVTIADGSSPPKKSTKLGGDGTATYADGSWHNMGDALAHHGKRHPFYAEARVAFFGTT
EASSLGADPGSTVLNAPYTSKWGVIQSTGVMYVWGSDFGGGAAGATWFTNTGGRGSTYQ
AENAVVLAGSWGSGSHSGSGCAIWTYSPATSYNNIGARGVCDHMRRD"
CDS 30916..31590
/ID="QSXAMBDO_CDS_0052"
/transl_table=11
/phrog="6034"
/top_hit="No_MMseqs_PHROG_hit"
/locus_tag="QSXAMBDO_CDS_0052"
/function="unknown function"
/product="hypothetical protein"
/source="PHANOTATE_1.5.1"
/score="-78.0712878303745"
/phase="0"
/translation="MTKWPSSKNTRPSSPIFTRSHSRCLVSTAWRGICFCDACSDSPIS
FTRPGNPIRYRGFMPHWRSAMPKTSINTRSDLDAIAGSPEHAEWMAFLAGSLWRLERDD
ANQTWVAVEDNSTIERYGFVRADFPDAIRPELPVWEPGVPHVPQTVSRFQAKAALSNAG
LLAQVDALMSDPATDPIARLAWSDAQEFHRASPTVAAMAQALGLDAAALDELFVTAASI
IA"
CDS 31587..33884
/ID="QSXAMBDO_CDS_0053"
/transl_table=11
/phrog="220"
/top_hit="No_MMseqs_PHROG_hit"
/locus_tag="QSXAMBDO_CDS_0053"
/function="head and packaging"
/product="minor head protein and DNA pilot"
/source="PHANOTATE_1.5.1"
/score="-114339407.7424754"
/phase="0"
/translation="MSLSAADLKAIFNLEPKAAIAYLQHKGYKLTWNWQEMLDDAHARA
FTIAKAARLDVVQDIRNALDVALRHGQTLKDFQKNLKPTLQAKGWWGKQIIVDGAGNAE
VARLGSPWRLATIYRTNLQSAFMAANYQEMAEATDSHPYWQYVAVLDGRTRPSHRAMNG
RVFRHDDPIWNTIWPPNGFNCFPAETPVRAAARLGLKTWYAGKVVELQTRLGHRLTLTA
NHPVLTVRGWIAACQLQKGDQLIGDASGVNPRLAGVVNDEQPPARAEDLFQTLAAQGFR
IVPMAPHDFHGDAGLRKPEIHIAGPDVHLMDEVQAAPGQFVGQRQLRRADACAIMDADR
PDGPPPARMILADAVAPQNPADVAEAGAELAADGAFGDQPVAVQGQHPAFEMGVAVAGA
LPGGGALASNGGGVLFDGSPFDALGFRAPPQGDVAGTEQPAQGVTAASGLVRQLLEANA
GLIALDEIVQIRQFDWAGHVYDFETETGLIMAGGVIVHNCRCRVRPRSERSLARDGIAW
QSSAGKLRTLETDAGVDKRTGEITHARRTGIDVVDADGKKHFFAPDAGFNFNPGQGWSK
PFTPPPLDTLPKTFSPGQVLPDLPKPEKFAASMIVPDGLGEEDYAKAFLVEFGADLGKP
VVFQDVTGDPMLIDEALFKSGAGEWKASKNGRGPYMRLLAQAIKRPDEIWMRWEESRDK
PGTWLLKRRYIKTFEVEGGDRQSPQFGLSVFEFGQDGWSGSTAMMAKPDRTEAARRRYV
NEQRDGFLAYRR"
CDS 33859..33987
/ID="QSXAMBDO_CDS_0054"
/transl_table=11
/phrog="No_PHROGs_HMM"
/top_hit="No_MMseqs_PHROG_hit"
/locus_tag="QSXAMBDO_CDS_0054"

/function="unknown function"
/product="hypothetical protein"
/source="PHANOTATE_1.5.1"
/score="-2.522785976598359"
/phase="0"
/translation="MDSLLIGDKPYAAPQGSPCGSFGRVGRERLYDRNRLQAIIPR"
CDS 33984..34457
/ID="QSXAMBDO_CDS_0055"
/transl_table=11
/phrog="26"
/top_hit="No_MMseqs_PHROG_hit"
/locus_tag="QSXAMBDO_CDS_0055"
/function="connector"
/product="tail completion or Neck1 protein"
/source="PHANOTATE_1.5.1"
/score="-48.18541940903208"
/phase="0"
/translation="MSNPIEVTIDDIEVRQMLDRIQRAGLNLGPLMKTLAMTLKDETEH
RFENEGPGWPALSDSTKQARTRLGHWPGQMLQVSGQLAASISTESGADFARIGSSKVYA
AIHQFGGSAGRSQRTTIPARPYLPMTAKGELTPQAREAVLDDTMAYLKRAAGV"
CDS 34417..34515
/ID="QSXAMBDO_CDS_0056"
/transl_table=11
/phrog="No_PHROGs_HMM"
/top_hit="No_MMseqs_PHROG_hit"
/locus_tag="QSXAMBDO_CDS_0056"
/function="unknown function"
/product="hypothetical protein"
/source="PHANOTATE_1.5.1"
/score="-1.0143188830028862"
/phase="0"
/translation="MIRWHTLSGLRASEARSPGQTRRCESPARDGA"
CDS 34754..35845
/ID="QSXAMBDO_CDS_0057"
/transl_table=11
/phrog="976"
/top_hit="No_MMseqs_PHROG_hit"
/locus_tag="QSXAMBDO_CDS_0057"
/function="head and packaging"
/product="head maturation protease"
/source="PHANOTATE_1.5.1"
/score="-929.3835116830256"
/phase="0"
/translation="MTNRIHCFKPGRHLPMGGSQPIEFSERDLRDAAACYDPALHRAPL
VIGHPTRLDPAYGHVKALSYGPDGLEAEPEEVEPAFAELVNKQAFPNVSVGWYAPDHPR
NPVPGKWYVREVSFLGAVPPAVRGLRRPTLNPAFAADEDGIIRFSGERDDSVNAGLWRR
FREWLLVRFGTEDADQVVPSADLQYLEDAAREEIRDEAGDPATASQPVFSQSTPEDNTV
DQAQAAALTAENERLKAELAAARQRESAAAETRRRDDAVQFAQELTRPDGNGRVRLAPK
HKNLLVELLMLAGKGADEGGLPQFAGEDGSTRPLVDAVKAMFAEGGAVVQFGEFATADR
AGKAPIKNPLVADAERRAAAVKR"
CDS 35852..36226
/ID="QSXAMBDO_CDS_0058"
/transl_table=11

/phrog="49"
/top_hit="No_MMseqs_PHROG_hit"
/locus_tag="QSXAMBDO_CDS_0058"
/function="head and packaging"
/product="head decoration"
/source="PHANOTATE_1.5.1"
/score="-12.393187131616592"
/phase="0"
/translation="MPSHTDPKVISDVLLYEVGEQWSRDRVTIAAGADLALGTVLGKIT
ASGKYKLHDPAAADGSQNAVAVLLANAAAAGADVAAPVIARGAVLDANGLVWKSGITDP
QKATARAALLALGLKVIDSV"
CDS 36235..37224
/ID="QSXAMBDO_CDS_0059"
/transl_table=11
/phrog="29"
/top_hit="No_MMseqs_PHROG_hit"
/locus_tag="QSXAMBDO_CDS_0059"
/function="head and packaging"
/product="major head protein"
/source="PHANOTATE_1.5.1"
/score="-8753.392988978538"
/phase="0"
/translation="MNLADLFTVTTLTAAVNKLPYATYKIRDLGIFAEAGVRTTTVAIE
EDQGRLHLVPNRSRNDAPEVVRRKRRTRRVFETFHLPEAGVILPEDIQNVAPFGEDMTG
SSLEPQARVINDKLQIMRDSIEITREWQRVGALGGQILDADGTVVYDLYNEFGVTKKTV
DIAFGTNNLDVRAKIVEGKRHAEQKLTGAAVTGFACAASKEFMDLLTDHPKVQAAYANW
QAAQDRLGGDMRNDFTFGGVRFFELDVTVSGQRFIPAGKARLFPLGAGVFQMHNAPANY
NETVNTQGQPYYSKGEPRKFNKGWDLEVQANPLALCLFPEALVEFGAV"
CDS 37234..37695
/ID="QSXAMBDO_CDS_0060"
/transl_table=11
/phrog="195"
/top_hit="No_MMseqs_PHROG_hit"
/locus_tag="QSXAMBDO_CDS_0060"
/function="connector"
/product="head-tail adaptor"
/source="PHANOTATE_1.5.1"
/score="-28.184085014845436"
/phase="0"
/translation="MRYCTLDDLQKKIPPRTLAQLTNDTPPATAPNLDLVEEIVAGQEE
LVDGYLRQRNLLPLTEVPTIVRDVVVQLVRYEIYDRRPEGKDDLPPAVVRGRKDALQTL
DDIRAGRISLGVADDPAQAQPEAGARMRTRIQGERLFTPDVLDQYRGSR"
CDS 37791..38243
/ID="QSXAMBDO_CDS_0061"
/transl_table=11
/phrog="209"
/top_hit="No_MMseqs_PHROG_hit"
/locus_tag="QSXAMBDO_CDS_0061"
/function="head and packaging"
/product="portal protein"
/source="PHANOTATE_1.5.1"
/score="-30.584566592106277"
/phase="0"

/translation="MPYPADPANYQFLHPVGALLVRYHGSRYGALMDTDAVVQERLLAV
EITLLFRALDGQDGLYAYLERARRVLTGFKPAGFGKVYPLRDAFLEEHGGEWRYAVDFC
APTLAIEDGCEEDGPLLKHVTTLDGYERAETVRQPDGSTTYEEYAQ"
CDS 38240..38440
/ID="QSXAMBDO_CDS_0062"
/transl_table=11
/phrog="895"
/top_hit="No_MMseqs_PHROG_hit"
/locus_tag="QSXAMBDO_CDS_0062"
/function="unknown function"
/product="hypothetical protein"
/source="PHANOTATE_1.5.1"
/score="-7.3986255925672815"
/phase="0"
/translation="MTRYRYDGPVSSVTLPGGLDVNLHPGAEVELPEDNDYVGVLVAKG
FLTAIVSNRKPKGADAPAQEG"
CDS 38444..39901
/ID="QSXAMBDO_CDS_0063"
/transl_table=11
/phrog="23"
/top_hit="No_MMseqs_PHROG_hit"
/locus_tag="QSXAMBDO_CDS_0063"
/function="tail"
/product="tail sheath"
/source="PHANOTATE_1.5.1"
/score="-401279.4303984034"
/phase="0"
/translation="MPANFLHGVETIELDKGPRPIRGVKTAVIGLVGTAPMLDVDAADR
SLNRIRLVTNDRDAARYFGQNRAGFTIPSALNAIFDQGNGPICVVVNVLDPASDTTAVA
DEAKTFDAATDKLTLAHPQVSNVVVKHTAGTPTYVLNTDYTLDAANGTITRKAGGAIAS
GQSVKVSYSWLDPSKAVNSDIIGAVDGAGNRTGMQAWLNAYQELGFFPKILIAPGFSTV
NAVATELNVLAGKLRAIALVDAPIGTTVQQALTGRGPSGAINFNYSSDRVGLCYPHLKV
YDSASDTEILEPFSPRLAGRIAATDQDKGYWWSASNQEIKGIVGLELPLTAMINDPASE
TNLLNEAGIITVFNAFGSGIRTWGNRSAAWPSNTHPRNFINIRRTADVIHESIEYSMLQ
FLDQPITDALIDAITESVNLFLRTLVMRGALIDGHCGYDKAKNPVTEIALGHLTFDITF
MPPPPLERVSFESFIDINLLSQLGGNG"
CDS 39901..40107
/ID="QSXAMBDO_CDS_0064"
/transl_table=11
/phrog="No_PHROGs_HMM"
/top_hit="No_MMseqs_PHROG_hit"
/locus_tag="QSXAMBDO_CDS_0064"
/function="unknown function"
/product="hypothetical protein"
/source="PHANOTATE_1.5.1"
/score="-2.4891015945535875"
/phase="0"
/translation="MDEVWLRNLNRFQALTAKMSRKRACFPAGTGRTPCEARLAAVLDL
ALAKLGGSVSPEHPGRLERAFPG"
CDS 40115..40207
/ID="QSXAMBDO_CDS_0065"
/transl_table=11
/phrog="No_PHROGs_HMM"

/top_hit="No_MMseqs_PHROG_hit"
/locus_tag="QSXAMBDO_CDS_0065"
/function="unknown function"
/product="hypothetical protein"
/source="PHANOTATE_1.5.1"
/score="-1.7384731019041049"
/phase="0"
/translation="MRDLQGQFGVKIRRHRSLAFRSVRTSIWQR"
CDS 40195..40716
/ID="QSXAMBDO_CDS_0066"
/transl_table=11
/phrog="55"
/top_hit="No_MMseqs_PHROG_hit"
/locus_tag="QSXAMBDO_CDS_0066"
/function="connector"
/product="head closure"
/source="PHANOTATE_1.5.1"
/score="-212.67733501827996"
/phase="0"
/translation="MAKIEINRLTNANVYLDGASFLGRAEEVELPDIKHKMEEHMALGM
VGTLEAWSGIEKMTAKFKWSSFYKEVMAKAANPFKSVAVQVRGSLETYATAGRIAETPV
VAHLIGQFKSIPMGNYKQHKNVEQTNEMAVHYAKLVVDGAEVFEFDALANIYKVNGQDM
LANYRANIGG"
CDS 40718..41065
/ID="QSXAMBDO_CDS_0067"
/transl_table=11
/phrog="585"
/top_hit="No_MMseqs_PHROG_hit"
/locus_tag="QSXAMBDO_CDS_0067"
/function="unknown function"
/product="hypothetical protein"
/source="PHANOTATE_1.5.1"
/score="-13.410473060547284"
/phase="0"
/translation="MRLDWNLARSVLEAAEALGDEKDRVAPGAFPGVAEEVAIEHFRLL
CEAGLADGYPQGRSPLFLTRLTWSGHQFLATLRSRTLWSRVKAEAKDRGLALSFEVIKA
LAAKLVGQLVD"
CDS 41108..41413
/ID="QSXAMBDO_CDS_0068"
/transl_table=11
/phrog="166"
/top_hit="No_MMseqs_PHROG_hit"
/locus_tag="QSXAMBDO_CDS_0068"
/function="tail"
/product="tail protein"
/source="PHANOTATE_1.5.1"
/score="-6.627425469327513"
/phase="0"
/translation="MNKIKLHEPLKTGDGRTLTELTMRAPKVKDLKAAQRFGGSDADVE
VALIASLVGVVPEDLDELGLADYRRLQDSFRRFSDPDGGAVEGDGAAGPVVPVPAQ"
CDS 41373..41504
/ID="QSXAMBDO_CDS_0069"
/transl_table=11

/phrog="8286"
/top_hit="No_MMseqs_PHROG_hit"
/locus_tag="QSXAMBDO_CDS_0069"
/function="unknown function"
/product="hypothetical protein"
/source="PHANOTATE_1.5.1"
/score="-2.948830786837255"
/phase="0"
/translation="MALLARWFRFQPSEIDALELEDFLTWWDLANQQIRLEAGPAGA"
CDS 41606..41791
/ID="QSXAMBDO_CDS_0070"
/transl_table=11
/phrog="No_PHROGs_HMM"
/top_hit="No_MMseqs_PHROG_hit"
/locus_tag="QSXAMBDO_CDS_0070"
/function="unknown function"
/product="hypothetical protein"
/source="PHANOTATE_1.5.1"
/score="-1.55244121497591"
/phase="0"
/translation="MMDLPATQNATTAPKSAKYAIQGARCSMFSILSASAAKGFGGFTF
QSTLGCTPGEARLTHG"
CDS 41784..44765
/ID="QSXAMBDO_CDS_0071"
/transl_table=11
/phrog="5690"
/top_hit="No_MMseqs_PHROG_hit"
/locus_tag="QSXAMBDO_CDS_0071"
/function="unknown function"
/product="hypothetical protein"
/source="PHANOTATE_1.5.1"
/score="-7135445347.564024"
/phase="0"
/translation="MAKELAFGIVIGAALSSAFTAAFGNAKKTIDTLGASVRDLTDKQK
TLGATIQKSGRLAQESLAPLHRDYERLGRLIDEIRRRQEALTASLARGAALKQERADLR
GQALETVGTGAALGAPVVASVRLAGNFQDQLRDIAITGEFTTAQENRLGAAVRESALKW
NQTQAEIASGIGVLVAGGVQDAQALDRYTPVLAKAATATRASMADLGSVLLAFDNNLKV
SADQSESALNMLAYAGKRGQFEIRDSAKWLPALAPMFQTLGVTGKEAVAEIGAALQIAR
KGAGTNDEAANNFRNFLAKLTSPDTLKDFDKAGIDLQSSLKNAAKRGISPMEAMMDTIT
AYIGSKGPQAAAAFKQALSLEDAQKRAEALQALSGSFRLGELFQDMQAMSFIRPMLANR
AEYKDIKQGALGAANQDLIGADFQKRTQGFNESLKAFRIGMSEVGLVVGEALLPPLTDL
LQTVRPVIREFGQFASAHPGVIRGVVGLTAGLLGGKLAVLAVRYAVNLLVSPFNALATA
TQLVLGKWTLLKTALQFGPLARAGGALSAAGSAALGAGRALGGILLSGFRLAGAATLGF
GRSIASPLLSGLRLAGAAAAGFGRILIGSFLAGLRLAGVAALELGRFSAGALLSGLRLA
STAAAGLGRILIGSALAGLRLAWLAALELGRILGGALISGLKLAAQAVMFLGRALLFTP
IGAAVAIIAGAAFLIWKNWDALKARFAPLWDQVKGIFGRALSWFKTLPDVFKAIGSNLL
EGLRSGIMAKWESVKAGLSSIASGIKDTFKSALGIHSPSRVFAGYGADIGQGLIEGVTG
QKDAVAETLGKLVRFPQPPVIRVHTELGSSTGPERSGLAPVSLNASLGRETGRVLPFAK
PDSKGNGADSKGNGVESPAATTDTPARLLGRKFGDAALRVRQGSDVASGADGHARTFRS
VHQPQGRSGEQVVIHFNPTIHVSGAEDAGKAKAAVTEALELSLRDFERVAAQASHARAR
TGYR"
CDS 44737..44865
/ID="QSXAMBDO_CDS_0072"

/transl_table=11
/phrog="No_PHROGs_HMM"
/top_hit="No_MMseqs_PHROG_hit"
/locus_tag="QSXAMBDO_CDS_0072"
/function="unknown function"
/product="hypothetical protein"
/source="PHANOTATE_1.5.1"
/score="-1.7155791118204147"
/phase="0"
/translation="MPGRGRATGEDSKSSVAETVREWLRDDRGIVAPTEAASAGLF"
CDS 44930..45499
/ID="QSXAMBDO_CDS_0073"
/transl_table=11
/phrog="788"
/top_hit="No_MMseqs_PHROG_hit"
/locus_tag="QSXAMBDO_CDS_0073"
/function="tail"
/product="tail protein"
/source="PHANOTATE_1.5.1"
/score="-112.725117633844"
/phase="0"
/translation="MSYLQLGNVQLDLITWLGGFEAGYGYAYASHDIIEGKSHLQWTGD
QLETRALSAQLHARFCDPRAEFEKLKTAASRHNALAMFFANGTYEGRFVIEELQRTLER
TDRFGNVYLMQVRIQLKEWAEPTSSSLAGSLNANARAQAEAIAENAPARQLKDPSFGPT
ASQVRLKELAAKYDTFTRQPVPGLIR"
CDS 45496..45741
/ID="QSXAMBDO_CDS_0074"
/transl_table=11
/phrog="46"
/top_hit="No_MMseqs_PHROG_hit"
/locus_tag="QSXAMBDO_CDS_0074"
/function="tail"
/product="baseplate hub"
/source="PHANOTATE_1.5.1"
/score="-5.3118401888255615"
/phase="0"
/translation="MTTYLSYITQEGDRWDGLAYRFYGDPFRYEPLVVANPHVPIVPVL
PSGLTLAVPVLAKSDSTPSVESLPPWKRGVPAGGVA"
CDS 45738..46781
/ID="QSXAMBDO_CDS_0075"
/transl_table=11
/phrog="52"
/top_hit="No_MMseqs_PHROG_hit"
/locus_tag="QSXAMBDO_CDS_0075"
/function="tail"
/product="tail protein"
/source="PHANOTATE_1.5.1"
/score="-18090.47945604869"
/phase="0"
/translation="MTPRLDSKSNGVETPVEDPVYSVIYDNVNITADISGLVTELIYTD
HEHGESDSVELKIEDREQRWKNEWYPDISARLSVSIGYADGRRLDCGDFELDEIEFDVM
PDTVRIKALATVITPKLRTPRSYGYDDTSLRAIVQQVAARNGLTVRGDIEGIALDRVTQ
NHEKDLAFLTRLAEDYGYAFAVRGDALDFHSIATLEAAPSAFSLHRRQLKHCTLSEKSE

```
                     ATYPDGKVSHHDPDQKALVYDWDEKGNIKTGDTHALLKRAKHRGIAKRKASSHLHQADK
                     KQLSGTLGLVGDTRLVAGINLDLTGLFKLDGKYHVTTSTHRLDRSGGYASEAEVYRVVA
                     IHSLESP"
     CDS             46844..46954
                     /ID="QSXAMBDO_CDS_0076"
                     /transl_table=11
                     /phrog="No_PHROGs_HMM"
                     /top_hit="No_MMseqs_PHROG_hit"
                     /locus_tag="QSXAMBDO_CDS_0076"
                     /function="unknown function"
                     /product="hypothetical protein"
                     /source="PHANOTATE_1.5.1"
                     /score="-1.0155452357883712"
                     /phase="0"
                     /translation="MGWSPPSTRRSRGSRRSSPTRTAWCRTGSACPARRA"
     CDS             46972..47295
                     /ID="QSXAMBDO_CDS_0077"
                     /transl_table=11
                     /phrog="9992"
                     /top_hit="No_MMseqs_PHROG_hit"
                     /locus_tag="QSXAMBDO_CDS_0077"
                     /function="tail"
                     /product="baseplate assembly protein"
                     /source="PHANOTATE_1.5.1"
                     /score="-17.223614042408425"
                     /phase="0"
                     /translation="MPEVGEQVACIMDEHLEDGTVIGAIYSGPDPVPEGVDEKWYGVWF
                     EDGSILYYRKDSHQLLIDLTHMQGTVLLKAQSVTVEAHTVNVQAGTAQVEADTVAVTAS
                     TLTA"
ORIGIN
        1 actcggccag ggtcttgtag acggaatgga tggactcgtc gatgaactgg gaggtgtcga
       61 aggacacttc cagcgtcatc ccgtccggca gggtttgcag gattttcggc agctccgcgc
      121 gcaccgaccg ggccacctcc agggtgttgg ccgtggcctg cttgaccacc cccagcgcca
      181 ccgccggatt gccgttcacc cgcacgatgt tgcgctcatc cgccgccccc agctcggccc
      241 gaccgatgtc cgatagccgc accagaaaac cgttcacctc ccggacgatc agagcgttga
      301 attcttcgac cgttttcagg tcggtctcgc tgaatacggt gaactcgcgc tgtgcacttt
      361 cgatgcgtcc gcctggcagt tcgacgttgc gcttctgcag cgccgcctcg acatcctgca
      421 ccgtcacccc gtgggcagcc atccgctcgc ggttgagcca cagccgcatc gcatagcggc
      481 gttcaccgcc gatgatcaca ctggcgacgc ccggcagagt ctgcagccgg tcctgcacgt
      541 aacgatcggc gaaatcggtg atttccaggg gactatggcg atcgctggaa aaagcaagcc
      601 acaggatggg ctgggcgtcg gcttcgatct tgctgatgac gggttcgtcc atctcgcgcg
      661 gcagccgggc cctgacccgc gacacccggt cgcgcacatc ggccgccgcc gaatcctggt
      721 cgcggctgac gttgaactcg agggtgatct ggctgacttc ttcgcggctg accgacttca
      781 tggtccggat gccttcgatg ccggacagcg agtcctccag cggccgcgtc acctggctct
      841 cgatcacctc cgaactggcg cccttgtaga cgctgcggac cgagaccacg ggtgcatcga
      901 tgttgggata ctcccgcacc ggcaggcgct ggaaacacat cagcccgacc agcaccaaag
      961 cgaggctcat caccgtcgcc agcaccggcc ggcggatgga aatgtcgctc agtgtcatgg
     1021 ggcgggcgga ttttccttcg cttcgggaac cgcgaccggg gccccgtcgc gaagcttgac
     1081 ctggccttcg gtcacgacgc tgtcaccctc ggcgagcccc tgcacgattt cgaccttgcc
     1141 cttgtagcgc gcgccgatcg ccaccggagt cgcgaccgcc ttgccggcca ccacccggaa
     1201 caccgacgcg gagctgccct tcaccaccag ggcggactcc ggcacgaaca gtgccctgcg
     1261 cgccgcgccc agtgccagat ggatacgcgc gaacataccc ggacgcagtg cccgatcggg
     1321 gttcggcagg cgcgccctca ccctcagggc gcggctttcc tcctcgagac gcggatccac
```

1381 ggcgtagacc tggccggaaa aaacccgccc gggataagcc gccacctcgg cagtcagggc
1441 gagccccctc gtcacggtgc cggcatactt ctccggcacc ttgaaatcca gcttgaccgg
1501 gtccagggcc tccaggttga ccagcgcctt gcccttgtct acgtattccc cgacgctgac
1561 ctgccggata ccgacgatgc cgtcgaacgg cgcgcgcagc acggtcttct gcagcagcac
1621 ccggtcccgt tccaccaagg cattcgaata cttgagcttg gacacggcct cgtcgtactg
1681 ctgggcactg acatgcgcgc gctcacgcat cgccttcatg cgcgcgaagt tggcctggtc
1741 cagctcctgc tgggccagac tctgggccaa ggccgcccgg tattcgtcct gctcgagctc
1801 gatcagcacc tcgcccgccc tgaccggttt accctcggtg aaccggatcg ctttgacccg
1861 tcccgggacc tccgggcgga tcacgaccga ctcgtcagcc aagagggtac cgacggcgtc
1921 gatggcatcg tcgacggact cagcccagac ccgcgccacg aacaccggga ccgacggcgg
1981 cacccgcacg tcggcgggcg gcaccgctgc cggcggcgtc gcggtatgtc gccaccatcc
2041 ggcgatggca gccagcgaag cggccaggac cagaacgccg aaacttttgt tggacatggg
2101 gggacgcccc tcgaaccggt ggcaaatgaa gcggaaagga aatgtcaggc cggtccggac
2161 agcagctcgc ccagctcacc ggtgcgttcc agcgcagcca gatcgtcgaa cccgcccacg
2221 tgcctgtcac cgatgaaaat ctgcggcacg gtgcggcggt gggtgatcgt catcatttcc
2281 tggagtctgg cgggatccag gtcgacccgg atcttttcga tctccacgac ccccttggac
2341 ttcaacagcc gctcggccgc cgagcagaaa ggacacatgg ctgtgctgta catgcggact
2401 gggagcataa tcgtcgcatc ggctctgtga caggaacagt ttccgagttt aacacgcgcc
2461 ccgggccgga gacgcctccc gatcgttcag cggtccctct ttacaaatcc atgtttcgtg
2521 agaatattag aaattactgg aatgacctgg tgaataccat gactactgaa cgctgggtac
2581 gtatcgtcgc cggctttttc atcctgctct ccctgtccct cggcgtggaa gccagccccc
2641 tcttcgtcaa cgccaactgg ctctggttca ccgctttcgt cggtgccaat ctgttccaga
2701 gcggctttac ccgcttctgt ccgctggaaa gcatcttgcg gaaactgggc gtgaaaagcg
2761 cctgcggcac ggaatagcca tgcaactggt ctgcccggcc tgcctcaccc gcaaccgcgt
2821 gcctgccgat cggctcggcg agcagcccaa atgcggccgc tgctccacgc cgctgttgag
2881 cggccatccg gtcgaactcg acgacggccg cttcgatgct tacacccgtc actccgatct
2941 gccggtgctg gtggatttct gggcgacttg gtgcggaccc tgccgatccc tcgcaccggt
3001 cgtggcccag gcggcggacg ccctcaatgg ccggatcctg gtcgccaagg tggacgtcga
3061 ccgagcccct gccacggctc agcgtttcaa catccgcagc gtcccgaccc tggtgctgtt
3121 ccggcacggc caggaaacgc gccgaatcag cggagcgctc ggcttcggcc ccttgatgga
3181 ttggctgcaa cgcggttgat gcagcggaaa gagcgacccg gcaaattgcc gggccccctg
3241 cccgatggca tcgatacccc ggtccgtttg atcccgatcg ctttctggcc gcccaggcca
3301 aggactaggc gaatgcgatc gccgcgcctc gcgcgggccg caagcgaacg cattggataa
3361 agttcttcgg cggaaaaccg gacgaaagaa gcctggccat ccgcgcgaag atgacccccg
3421 gcagatgaga ttggagacgg ggccgtgatc gacgccgagg atccggcccg aacctccagc
3481 tcatgcccct ggcagggcat cttccaatac cctgccagga gactacggaa agaaacccgg
3541 cgtcaggggt gataggcggt ttcgccgtgc tcacggatgt ccaatccctc acgctcggtc
3601 tcctcggaaa cccgaaggcc gacgagcaga tcgacgatct tgtaggcgat cagggagacc
3661 acgccgctcc agacgatggt gacgcccaca ccccaggact ggctgatgac ctggctcacc
3721 atgtcgtatt cggcgacggc gttggccaca tagtcgtaga ccccggcacc gccgagggcg
3781 ggagaggcga agatgccggt gcccagcgcg ccgatgatac cgcccacgcc gtggacaccg
3841 aacacgtcga gcgagtcgtc atagcccagc gcattcttga gggtagtcac ggaccagaag
3901 cacaccgcgc cggccgccaa acccagcacg atcgaaccca tcggaccgat gaacccgcag
3961 gccggcgtga tggccaccag gccggcgacc gcacccgaag caccgcccag catgctgggc
4021 ttgccgcgcg cgatccattc gacggccgac cacgccagcg tcgcggcggc ggtggccagc
4081 atggtgttga cgaaggccaa agccgcggta ccggtggcct ccaggttgga accggcgttg
4141 aagccaaacc aaccgaccca aaggagcgag gcgccgatca tggtcatagg aacgctgtgc
4201 ggcgccattg actccttctt gtagccgatg cgtttaccca ccagcagaca gcctaccagg
4261 ccggcgatgc ccgcattgat gtgaaccacg gtgccgccgg cgaaatccag cgcacccttt
4321 tgaaacagga agccggcggt agcggtcgcc ttttcacccg ccgcggcatc ggtataggcg
4381 tcggggccgg cccagtacca caccatgtgg gcgatcggca gataggagaa ggtgaaccac
4441 aacaccatga acagcagcac cgcgctgaac ttcatgcgct cggcaaaggc accgacgatc
4501 agggccggcg tgatgcaggc gaaggtcaac tggaacgcga cgtagatcag ttcggggatc

4561 accacaccct tgctgaaggt ggcggcggtc gaatcgggcg tcacgccggc gaggaaggcc
4621 ttgtccagac tgccgaagaa gacgccccct tcggtaaacg cgatgctgta accgtagacg
4681 gcccagagta cggccatcag cgaaaagatc acgaagacct gcatcagcac agacagcatg
4741 ttcttggcgc ggaccatgcc gccatagaac aacgccagcc ccggaatgac catcagggtc
4801 accagcaccg tggcgacgat catccaggcc gtgtcaccct tgtccggggt cggagcggcg
4861 ggggtagcgg cctgctgcac cgtctcgacc acggtctcga cgatctcggt ggcgccttcg
4921 gcctgcgcgg cggcactgaa accgagacac gccagagtca ccagggaagt cggaatgttt
4981 ttcatatgaa gggtctccat cgtccgttgt cgttgtcaca gggcatccgg accggtctcg
5041 ccggtgcgaa tccggacgac ttgttcgaga tcggccacga aaatcttgcc atcgccgatc
5101 ttgccggtat tggccgtctt ggtgatcgca tcgatcaccg cctcgagctg gtcgtcggtg
5161 atcgccacct cgagcttcac cttgggcaga aaatccacga cgtactccgc gccacggtat
5221 aattcggtat gccctttctg tctcccgaac cccttgactt cagtgaccgt gatgccggtg
5281 atgccgacct cggacagggc ttccctgacg tcgtcaagct tgaaaggctt gagtattgcg
5341 gtaacgaatt tcatgcaggt ctccttggtt gaagtattac gcggtcggcc tcacgtctcc
5401 tttgcacggc gaattaggcg cgatccgatg gctccactgg atgaaaagca tgagccatgc
5461 cacgggagat aaaagagcag ccaagagcca aacaaaccct ggtccccaaa caaattgcac
5521 ctttgttgtc aaacgaccgc aacattacgc accaatttaa ggcgcgccgg cgcacatccg
5581 gcacttaccc attccgaacc tccaggcgga ccgccatgat cgacaagacc actctcgacg
5641 aactttccag acgcctcgcc gacgccgtgc ccaccgggct gctcggcgtc cgcgacgagc
5701 tggaaaaaaa tttcaatgcc gtgctgcaga gcacgctcag ccgcctcaac ctggtgagcc
5761 gggaagagtt cgatgtgcag cgcgccgtgc tggaacgggc ccgcacccgc ctgggggaac
5821 tcgaagcccg cctctccgaa ctggaaaaac gactgcctcc cgcctgaagc cggacatggc
5881 gctcgcgatc gtccacagcc gggcctgcca gggcatcgac gcaccggagg tgacggtgga
5941 agtccacatc gctccggggc tgcccaatct caccatcgtg ggactgccgg aaaccgcggt
6001 gcgcgagagc aaggaccggg tgcgcagcgc tctcgtcacc accggcttcg aatttcccgc
6061 ccaacgcatc accgtcaatc tggccccggc cgacctgccc aaggaaggcg gccgtttcga
6121 cctgcccatc gccctcggca tactggccgc ttccaagcag atccgtaccg agcatctgct
6181 ggcgctggag tgtgtcggcg agctggcgct taccggtgaa ctgcgtccgg tccccggagc
6241 cttgcccgtc gccctccagt cgcgccgtgc cgagcgcagc ctgatcgtac cctgggacaa
6301 tgccggcgaa gcggcgctgg tctccggcgc ctgcgtgctc ccggcgcgcc atctgctgga
6361 cgtgtgcgcc catctggacg gcagccgccc gctcgccgcc gccgaaccgt gcaccgaagc
6421 gtctcaggca agcgaggaat tccccgatct cgccgaagtc cgcggccagt tccaggccaa
6481 gcgcgccctg gagatcgccg ccgccggccg ccacaacctg ctgatgctgg ggccgccggg
6541 caccggcaaa tccatgctgg cctcgcgcat gcccggcatc ctgcccgacc tgaccgagac
6601 ggaagcattg gaaaccgcgg cggtcgcctc ggtcagcggg atgcccttcg acccggcagt
6661 ttggcgccga ccgccgtacc gcgcccctca tcacactgcc tccgcaccgg cgctggtagg
6721 cggcgggagc agccccaaac ccggggagat ttcgctggcc cataacggcg tgctgttcct
6781 ggacgagttg ccggaattcg accggcgggt gctggaggtc ctgcgcgaac ccctggagag
6841 cggcaccatc accatttccc gcgccaccca acggctggat ttccccgccc gtttccagct
6901 cgtcgccgcg atgaatccct gcccctgcgg ctatctgggc gatgcctccg ggcgctgcca
6961 ttgctcggcc gaacaggtgg cccgctaccg tgcacgcatc tccggacctc tcctggaccg
7021 gatcgacatc cacatcgacg tgccccgcca ggacccggcc acacttatgg acggcgcgtc
7081 cccgagcgag gagaccagcg ttcaggtccg atcccgcgtg actgccgccc gcgagcgggc
7141 gctccgccgc agcggccagc ccaacgcact gctcacgccg cgtcagatcg agcgccattg
7201 cgcccccgac agtgcgggac gcgccttgtt ggaacgggcc atgacacggc tgaatctgtc
7261 gcaccgcgcc taccatcgca ttttgaagct ggcccgcacc atcgccgatc tggatggcag
7321 cgaggcaatc acagccaccc acatcggaga agccatcggc taccggcgtc tcgaccgcgc
7381 cgcgccccga tgaatggtct caacccccag caacacgcgg cggtcaccac cctggactgt
7441 ccgctgctcg tgatcgccgg cgccggcagc ggcaaaaccc gcgtcatcac cgaaaagatc
7501 gcctacctga tccggcaggg cacaccggcc cgccatatcg tcgccgtcac tttcaccaac
7561 aaggcggcgc gcgagatgaa aagccgggtc ggcaagctgg ccgacgacaa gtcactgcgg
7621 ggtctcacgg tctccacctt ccatgctctg ggactggaga tcgtccggcg tgaacacaag
7681 gcgctggaac tcaagtccgc catttccatc ttcgatgagc aggaccggca ggctttgctg

7741 cgggaactga tccgccacgg cgaccaccgc ttcgacccgg agcaagccga cgcctacgcc
7801 gcgcgcatct cgcgctggaa aaaccgcttc atcacccccg accgtgcccg ctcgctcgac
7861 gagcccgatg cggccgggat cgccgccctg tacgaagcct acatgcacca catgaaggct
7921 tacaacgccg tcgatttcga tgacctgatc ctgctgccgg tgatgctgtt ccagcggcac
7981 ccggaggtac tggacaaatg gcgcaaccag atccgttacc tgctggtcga cgaataccag
8041 gacaccaacc agacccagta cgaactcatc aagctgctca ccggcacctt gggcaagttc
8101 accgtggtcg gcgacgacga ccagtccatc tatgcctggc gcggtgccca gccggaaaac
8161 ctggcccagc tccgcaacga cttcccccgc ctcaaggtca tcaagctcga acagaattac
8221 cgctccatgg gacgcatcct caaggccgcc aaccagctca tcggcaacaa tccgcacatt
8281 ttcgaaaaga aactgtggag cgccatgggc caaggcgacc cgatccgggt gctcaggcac
8341 cgcgacgaaa tcgccgaagc acgccagatc gccgccgata tcgtgcatca caaattccgc
8401 cacaacaccc gcttcgccga ctacgccatt ctctaccgcg gcaaccacca gtcgcggctg
8461 ttcgagcgcg cgctgcggga attcagcgtg ccgtatcaca tcagcggcgg cacgtccttc
8521 ttcggctaca cggaggtcaa ggacgtcatg gcctacctgc gcctgctggt gaatccggac
8581 gacgacgccg ccttcctgcg catcgtcaac gtcccgcgcc gcgagatcgg ccccaccact
8641 ctggaaaaac tcggccagta cgccaaccgc cgtcatatca gcctgtacgc cgcctgcttc
8701 gaactgggcc tggaacaatc ccttccggcc aacgccgtcc agcgcctgcg ccgcttcggg
8761 gagcacatcg ccgataccgc cgaccgcacc gcacgcggag acgtcttcaa gatcatcgac
8821 gattttctcg gcacgatcgg ttacgaaaac tgggtgcggg aaaacagcag tgacgccaag
8881 gccgccgaac gcaaattcgg caacgtgcgc gaactgctcg actggctccg gcgcatcgcc
8941 gaaaaagacg gcgaaaaccg cagcctggcc gaaaccgtgg ccaaggccac gctgctcgac
9001 gtactggacc gcaaccagga agacgacacc ggcgaccggg tcagcctcat gaccctgcac
9061 gccgccaagg ggctggaatt tccctatgtc tacctggtcg gcatggagga aaatctcctc
9121 ccccaccaaa ccagcatcga cgaggacaac atcgaagaag agcgccgcct cgcctacgtc
9181 ggcatcaccc gcgcccgccg cgacctcacc ctgagctact gcagccaccg gaagcgttac
9241 ggcgaaatga tcggctgcga gcccagccgc tttctcgccg aactgccgca ggaagacctc
9301 gaatggcccg accgccagcc gctcgatccc gaaaccaagc gcgaacgcgg ccgcgcctca
9361 ctggcgcagc tgcggaattt gttaagcgac tgatgatctt gtataatggt ccgctcagcg
9421 cgcccgtagc tcagttggat agagcgttgg cctccggagc caaaggtcgg acgttcgagt
9481 cgtctcgggc gcgccatttt gagactgaac gaatgcccag ccaggggaca ttcgcagcaa
9541 atgcggtaaa tctcggggcg tagcgcagcc tggtagcgca cctgtatggg gtgcaggtgg
9601 tcggaggttc gaatcctctc gccccgacca attaaatcaa gaaagcggct tgggtgattt
9661 cacctgccgc tttttttatt gcccgcgtga ccaaatcgtg agatcccatc gagctcgttc
9721 accgccacgc ggacatttcc ttgagtgacc ttccctcgaa aaatggtctt caaacggtga
9781 cgtagaatca tcctcaccct ggaaaggaag acgatggaga atcccgaagc agggatatcc
9841 ggccggccag cagcgcgtgg aacggctgcg gcgtttccgg gtcacgaccg actcgaagca
9901 cgacctcccg gagcgcaacc tcactccgac cgggccgaat caggtccgga gctcggacat
9961 cacctacctg tggacggatg gatgccggcc gtatccggcg atcaccccgg atgtgggtta
10021 gttcttgatt tggcactctg gactagcccg gacaaatttg gacttcgccg gacaaatcaa
10081 agcgttggga gatgttggag cggaaattcg ttttggcaca aatttgcgcc cacggagcag
10141 tagaaaccgg atttcgcgcc atctttggca caaacaagac cgctttcgag cggttttttt
10201 atgccctaca ttcggcgacc ggcccatacc acccggccga tgatagaaaa gtctgctcta
10261 tccaagtcct gcgccccaac ctcaaacggc tcgtagacgg ggttatcgct ggttaccttg
10321 atgattccgc ccatcttgcg ctgtaagcgc ttgaccagta gcgcaccgtc cagccgcagc
10381 acgtagatgc cgccatcgcg cccctggaac ttatcccgat gattcaccaa gatcacgtcg
10441 cctttacaca gtgtgggctc catgctctcg ccgtccacat agatcaagta aagatcatcg
10501 ggtgagctgc gcagctcggt gcgcaaccat gctttcttga aatgcaggaa atcgacgacg
10561 ttttccgtgt ctgggactac gccacctccg gccgcagcac gcacgtcata gagcggcagc
10621 gcaacgtagt catccggtga atagactgca ggctgccgct gcagatgctt atcgccgttc
10681 aggccgcctg gcttggtcgg ataggcgtgt gcggcctggg gctcgccttg cagcttttgc
10741 ctgatgagcg caagaacttc tggaggcggc tggtattcgc gcttcatccc ccgcttaccg
10801 cccttgctgg gcacttccct gaacggccag tgctgtcttt ccagcacgtc acgccatccg
10861 cggtcagatt taggccagcc tggcaaaacc atcctcgcga ggtcggcagc ggaaaaccac

10921 tcttccgagc tccccgcacc tatctcggaa tttatctcgg aagtcattta gccacttccg
10981 agttatccaa gtttatgatt ttagggtatt tcatttattt tcacctatca gcctatcttg
11041 gagcagctcg gaagcaaaaa aatcttgttg acggaagctc cgagcttcaa tacgctatgt
11101 caaacagcag aacaaacact gacacaacac aaccacggaa acgcgccatg tcgaatacaa
11161 atcaagccgc aggggctcag gcaaacccgt tctgcgcctt catcgaagcg cagggcggca
11221 ctatcacacc ggaccagttt cgtagttggc tcgcgctcca tggcatcacc atggcggatt
11281 gggccaggga gcgcgggttc aggccgcgcg aggtttcttt ggtgctgaac gggcagatca
11341 aggctcgtta tggccggagc ttttccatcg ccgtggcgat ggggttgaaa cccgatccga
11401 accgcgctca gaaacagcac gccgcttgag ggagaacgat acagagcgcg ttagcgggcc
11461 acaaggcaaa cgttagcgga acggctgacg gcaatacgcc ggggtcatca cgacataaga
11521 ggagcagtca tgaacgcagc acagcaagac ctgttcggcg gcggcgccgt ccacctgccg
11581 caacccatcc ccatcgcgcc ggggcgtttc gctctgggtg accgggtcgc ggtcgccatg
11641 agccgcgcca tcgacgcctc aggggtcgat cggcccgatc tggcctcgcg gatcaccgcg
11701 ttgaccggca agcgtttgcc gctgtccgtg ctgaacgccc agaccgccat gagccggccc
11761 gaccacaccc ccagtctgct gcaggccatg gcattcgatg ccgtcactgg gaaatgggcg
11821 ctactcgagc tgtatgccga agcggctggc ggcaaggtga tctacggcga cgacattgcc
11881 gcctttgaag taggtcgggc ggtgatggtg aaaaagttgg cgggccagcg agaaagggaa
11941 gccctccggt ctgtgggggt ggggcgatga gtggcgagcg taagccgcaa ggctttcgcg
12001 ccatccagca tcaccgcgcc agcgctgtgc gcaatggcaa agcgctggca gaggtttaga
12061 catgatgtcg cttcttgagg tgactcatgc gcgagctttc agtgtggccg cagcgatgaa
12121 ggaatcccac ctgttggaat tgaaaagtcg gatcgatctt ctggtggcca cgggagcttc
12181 attccgtgaa gcagaaacaa cactacggcc actgtgcgct gcgattcaga aagaccggga
12241 ggaatcggta tgaaggagtt gccagcggct tcattggcta gagccaccag gatggcctgt
12301 tgttggcgct ccagcaatac acgccaagca cccgctgaaa tgtgctccca gctactgtct
12361 ggatcgtcga ttagaagctc gatagcggca tccgcttcgg gttcaaaaga ggcgtcgtac
12421 ggtagagaac gaatgcgcgt caggctttcg cgaaatcgtt cactcaatgg ccacagcgct
12481 tcaacgcctt gccaccccgt tatgaattgc ggctccagcg actctaggga gcaatccagc
12541 gcacgctctg taaaaacgcg gaacaggtca aaatttttca ttgagggttc ttccgtaatg
12601 gtcgtttggc aacgccagtt taaagcgggc cggaaccctc gccaatttgg gatgggccgg
12661 gcatgagcgc tatcaaaacc cactattcct gcgccgagct ggctgcgctc aagctgccgg
12721 gatatcccgg ctcggagcgc gggttccgcg atctggttga gcgcgaaggt tgggccttcc
12781 gggaggcgag caccaaggga ggcaaaaacg gcatgcgccg cgaataccaa ccctcagccg
12841 ccgtcatgaa gctgatccag gcacgcgaaa aggaagctca gcgccaggcc agcctcgccg
12901 ccgagctggc ggcgctggag agctcccccg aataccgcgc cggccaagcc atcgcccggc
12961 agatggccga agcctcggcc cgagaagctg aggcccggca gcaggacgat gaagcgctgc
13021 tggcgctgtt cgcccaggcg acgggcgagc gaggcgagcg cggacgcgcc tggctggcga
13081 cgattcaatg ctggcggcgc ttctatcggg aaaaatttgg cgaccagaag cgcagccagg
13141 aaaaggcgct gatcgccttt attaaagcct tcaatgccgg ggctctcgat ctcgatgggg
13201 aggtctacgc gcccgtcctg aaatcgtccg ggcgcggcaa aaacgcccaa ggcaagggcc
13261 tgagcctgcg cacgctgaaa cggaagctgg gcttgatcaa gaaatccaac atcggcgccc
13321 tgctcgacaa ccataaaagc ccgcgccgcg gcgacagtct gatccaccag cagccgcggc
13381 tggagcagta catgctgggg ctgctggcgc agcagcccga tatcgctgcg gtccgcctgc
13441 atgagatggc ggaaaaccgt ttcgccgggg acgcgacgat caaggtgccc tcctatagcg
13501 cggtggaccg gtacgtcaaa aactggaagg ccaagaacgc cagcgcctat ctgcatctgc
13561 gcaacccgga cgcctggaaa aacaaggcga tgccggcggt gggtctggcg gacggcgacg
13621 tggtgcggct caaccagcgc tgggagctgg attccaccct gggcgatctg ctgctgtccg
13681 acggcaagcg ccatgccgtg atcggcgtga tcgacgttta ctcacgccgt ctcaagctgc
13741 tggtcgcgcc cacctccaaa gcgcgcgcga ttgccgccgt ggtgcggcgg gcgatgctgg
13801 actggggcgt gcccgaacag gccaaaaccg acaacggcgc ggactacgcc agcgattact
13861 tgacgggcat ctgggatgcc ttcggggtag agcatgtgct ttgcccgccc ttctcccccg
13921 aatacaagcc ccatatcgag cgggcgctgg gcactttcaa tcacgacctg gtcgagctgc
13981 tgccgggcta catcggtcac gacgtcgccg agcgcaaggc gatcgaggcg cggaagagct
14041 tcgcccgccg cctgatggaa aaggacggcg tgctcgaagt gcggctatcg cccgaggagc

14101 tgcaaaaatt ctgcgacgac tggaccgaaa accgctatca ccaccggccg catgcgggtc
14161 tggaaggtga gacaccgttc cagcgcgccg cgtcttaccg gggggaaatc cggcgcattc
14221 acaacgagcg ggcattggac atcctgctgt ctccgcccgt ggacggcaaa acccgcagcc
14281 tgggcaagaa ggggctggaa atccatggcg ggttctattc ccaccccgag ctgtggcgcc
14341 accacaccac gggagacgcc ttccgcgtca tggtcgatga aaccgatctg ggtcgggtgt
14401 acgcctacga cgacaacggc ttcgtctgcg tcgcgatctg cccggagcgg ctggggctgc
14461 cgccggaagc agtcagccgg cacgccatgg agtgcaagcg catggcggcc cagcagcgca
14521 atcagggcat gaagacgctc aaccgggcca agaaaggcgt cgatctgaac gcggagatca
14581 acagctacct gcggtccaag gcggaatccg ccggcaaatt ggccaccttt ccccagctct
14641 ccaccggcta caccacgcca gccctggaag gcgcggcgcg ggcggcggaa agcacggatc
14701 ggccgccccg ctcgccggaa gcggaaaagc tgagcgccga agccaaggcc catttcgccg
14761 cttcgggccg gtcggccgag atcatcaagc tgccaaagcg taaggagcac ccgttggaac
14821 ggatgagcga tgaggagaaa tacgaattct ggtgctcgct ggatgccgcc gtgaaagccg
14881 gcggcggcga gctgcctgac gacgcgcagg ttcggaggtt ttacgccgaa tttccgcttt
14941 ccgcccggtt caaggcgcaa gccgcgatcc gctaaaagaa cggcccgtaa gcgttgccgc
15001 gctgacgggc cgatgagatc aaccacacgg agaaaagcat gacagacaaa cccgaaaacg
15061 acaaatccac ccctgccggc attgcccccc tgaccaatat cgccatcgcg gagcgcgcca
15121 tggccctggc catgggccgc agcatgcacc agtccggttt ggtggttttg cacggacctt
15181 ccggttacgg caaaagcatg gcggccgcct gggtcaaggc caggtctcgc gcctattacc
15241 tgcagctgga tgatttcacg cgctccagga aggtgctgct caagcgtttg ggtaaagcgc
15301 tggggatgca actggcgaaa tccgccacgg tcgatgagat ggccgatgca gtcatggccc
15361 aactggagca gtccgggcgg ccgctgatca tagacgaggc cgactacctg gagacgttca
15421 agctggtgga ctccatccgc agtatctacg aaggcagcaa ggccgccatc ctgctgatcg
15481 gcgaagaggc gctgccgttc aagctgaagg agtgggaacg ctttgacggc agaatcctgg
15541 atcgattccc ggcgcagccg gtgtctctgc cggatgcccg cgagttggcc gggctgtact
15601 gccccggcaa ggtgatgggc gaggacctgc tggaaaggct ggtcgaggtg gccaaggggt
15661 cggtgcggcg cgtttccacc aacctggagc ggatgtacga agaagccctg gccaacggct
15721 gggaccggat cgatctggcc acctggggca atcggccgat tcacaccggc gaagcgccga
15781 ggaggggttg atggcgcgca agcccgttac gatcgagctg gccggcggca agagtccgcg
15841 ccagcggatc tgggaaatga tccggaagct ccggatcttc accgtgccgg aactgcgcgg
15901 ccatctgccc ggcccggtcc cgctggccac ggtgcgcacc tacgtcgaaa gcctgcatgc
15961 cgccgggatt ttggagaaga cgcctggctt gtatgaattg atcgacgacc gcggcatcga
16021 agccccgcgg gtcaacaagg ccggccagcc cgtgaccctg gggcaaggca acgaacggat
16081 gtggggtgcc atggaagcct tgggcgcgtt caactgccgg gtgctggcgc ggatggccga
16141 cgtgccgctc gccaccgtta aaacctactg cgcctatctg cagcgggccg gttttctaac
16201 ggtggagcgt gccggcaaag gccggggcgc cggcggcgtg ccgaccacct accgcctgtt
16261 gcactcgcgg atcactggcc ccaggccgcc catgatcacc cgcctgaaga ccgtctacga
16321 ccccaatgtc ggcaagatcg tctggcagca ggacccgcag gagcagctcg atgactgaag
16381 agcggcggga aagagcgctg aagctgttgg aggcggccgt cgcggcgcac ggcagcttcg
16441 ccgcggtggc gaagctgctc ggcctcaacc gggccaccat cagtaccgta gcccggcgct
16501 gttatccggg cgacgacgcc aaagtgctgg cgcgtgtgct ggagcaattt gaccagatcg
16561 cctgtccgta tcttcggcgg ccgatggcgc cggaggaatg ccgatcggtg tggtccggcg
16621 ccacgccgag ccacgacccg gccttgctcg cccatcggcg cgcctgccgg agctgtccgc
16681 acaaaggaga cggccatgca cattcgtgac cgaatcatcc tggcgcccac accggcctgg
16741 tttgatgccg cgctcgatcg cggcgaaacc cgagcgcttc gggtcgacat cgcccctgcg
16801 gaaatcgcgg gtcatagcgt cttccatttg ccgttccgac ccgatcgtgc cgactggtac
16861 cggacgttcc aagtggctcg ggcattcctg cgcggagcgg cttttcgcgg cggagccgct
16921 cccacagaaa ccgggaggga ggtgcatggc tgagatttca accaaacgct gcacgcgttg
16981 cggccagacc ctgcccaagg accggttcta tcggcagggc gttcgcctgg aggcgacctg
17041 caaagcctgt gtcaatgcgg cccggaccgc gaagcggcga gcggccggca tcgccccccg
17101 tccgcccgcg aagccgcggc agccggatta ttcgccggaa gtctgggaca gggagctgcg
17161 ggcagtcaac cgggctttca atcacaccct gaaaaccatt ttcgggtcgc tgcaccgttg
17221 caaccctcac ccaaccctct cccggcggga gatggcttta acccaaggag aacacgcatg

17281 atcaagattc aacgatatcc ccggctcgga gcctacggct ggaaagcgct cgtcaccgtt
17341 ggcggcaagg cgcgcgaggt gctgaccgtc ggcacctggt tcgacatccg ccggctggcc
17401 aggcgggccg aggaggaaat ggccgagaag ccccgccgct cgccctgtgt ggacgcagcc
17461 cgccagcggc agctcgacgt gcggcgcggg ctgcaggggg ctgcgccgtg aaggcgatcg
17521 tcctcgcctg cttggcgttc atctacctca ttgggctcgg taccggcctg ctgctcggcg
17581 ccgtcctcga catcaacctg gtcggtcggg ccgagcgcaa agcgcaagcg cggcagagtg
17641 acaccgacga catcaacctt ggcatttagg aggaatcatg gcacgcatcc gaatcgaagg
17701 cacccaattc tcgagctggg acgacgtcga ccaggcgctg cgcgagatcg gtgagatcga
17761 gcgcgatctg agcctgatcg atggagacgt caatgatcag atcgacaggc tcaaggccgc
17821 cgccaaaacc caggcgcagc cgcttctcga ccgcaaaacc gcgctggaac tcgcgatgaa
17881 agagttctgc gaggccaacc gggcagagtt cgccaaggtt aaaacccggc agctcgtgtt
17941 cggctcggtc ggcttccgcc tctccactcg cgtactcatc aagcgcgtgg ccgacacgct
18001 ccaggcgctg aaagacctgg ggctgcacgg ctgtatcagg ctgaaggagg agatcgacaa
18061 agaggcgctc aagaccttgc cggccgagac cctggccgaa gttggcgccg gactcaaaac
18121 cgacaacgtg ttcggctacg agatcgaccg cgcccggatc gccgaagcgg cgtaaaccca
18181 ttttccacaa cctcgaagag gacaaaccat gaaccaatcc caactcatca acgccatcca
18241 tgaagacgcc aatggatcgc tgaccaaaac cgtgattcag cgcgtcctga tcgccctggc
18301 gaatgtcgct caggaagccc tggccaacgg cgacgaagtg acgatccccg gcgttgccaa
18361 gctcaccgtc aaggagcgcg cggcgcgcac cggccgcaat ccgcagaccg gtgagacggt
18421 ggagattcca gccaagcgag tggcgcgcgc atcgttcgtc aaggcgctca aagagcgcgt
18481 tcaaggcggg gcttgatcgc catggcgcgc accgatcccg tgcggcggca gttggtcgcc
18541 atccatgtgt tgaagtccaa ggcggggatg gatgattcgt cctatcgggc catgttgcgc
18601 gagatcggtg gcgcgtcgtc gtcgaaagac ctcacggcga ccgggcgcgc caaagtgctc
18661 gatcacctcc ggcggatcac ccagccaccg gtggcaaagc cacgcagccg gccctatccg
18721 ggccggccgc acacgacgga ccagcggccg ctgctacaga agatcgaagc ccagctcacc
18781 gaggctggcc ggccttggaa ttacgccgta ggcattctca agcgagtgag ccggggccgc
18841 gccgagcgct tggagttcgc caccgacgag gatctgcgga aagtcgtcgc ggcgctcacc
18901 tacgatgcta gacggcatgg gggaacgacg tgagtttgaa cctgctagtc gtgatcgtat
18961 tcgttgcggg attcggctgg ggccagtggt gggcggagag gcagatacgc atcgagcgcg
19021 cgtccttaga gatagggcgc ttgagcgttt ccatgccgat gaatccggat gatgcggcag
19081 cgacttttgg actcctcaaa acgttaatgg aacaggtgga gcgggaacgg aaatgagtca
19141 caccaagcgc tccttcatcc aggcgtgcct catccgcgca ctgcctgcgc tggaccgcgt
19201 cgacgccggc attgctcacg ccgaagcact gtgggagagg ctgaccgcca aaggctacgg
19261 cgcgccgcga caaaccggcc cgcgtgaatc cgttgactgg tacgccaggc tggtcgagcc
19321 gtcccgcggc tggttcgacc agttctggac cgcctacggc ctcaagcggg accgcaacgg
19381 cgcggccatg cgctggtacc agctcggcga cctcaccgaa catgaagcgc ggcggatcat
19441 cgacgccgcc aagcaggaca accgccagtg gcgggaaacc gcccagcctg gacaggtgcg
19501 caaaatggcc cagggctggc tgcacgaaaa gcgctggatg gactatgcgc caacccccca
19561 gccccccttg tcaggggggt attccgcggg gttggccgga gacgctcaac tgcgcgagct
19621 gaaacaacag ctcgcctcac tccagcggct gaatgccgcc gccccaagca aggagctgca
19681 gcgccagatc aacgagctgg tgcaggagat cggcaacttc cagcggccgg gccatggctg
19741 agaaaaaagg gcggcaggct cgtgaactcc tgagcgacct cgcggcaaag tccgccgcgc
19801 ttgcctgcga gttgggggcg ttgcccaagg atgtggccga gcgcttcgga ctggagctgt
19861 cgcgcgaagt tgcagcccat tggggtgggc aactgattta tatcccgcgg aatctgcccg
19921 gcgaattgga ccggcgggat atggagatat tcgaaaagtt caggggcgac aacattgctg
19981 aactcgcccg cgaaaagggt ctgtcggtgc agtggatata tcgcatcatc aagcgggtgc
20041 gcgcagccga gatcgagcgg cgtcagcagc ggctatttga ctgagattag ggggatcgca
20101 tgaaacgctt cctatgggga gctgtattgg ctggggctgt cttcggtgcc tgcgccgaag
20161 aatggaacgg aaaggtcgtc ggcatcagcg acggcgatac tctgacgata atgaaagaca
20221 gtggtggcac caaagtccgg ctggtcgaaa tcgatgcacc ggaatcccgg caggattatg
20281 gagccaagtc gaaacaatca ctgagcgatc tgtgttttgg caaagttgcc aaggtcgacg
20341 accaaggggc cgacaaatac ggtcggagac tgggccgtgt gcactgcgcc ggcgtggatg
20401 caaacctgga acagattacg cgcggtctgg cctggttcta tgtcgaatat ggccacgacc

20461 ccgctatgag ggacgcggaa gaacaggcac gcgccagtag aatcggcctg tgggctgata
20521 ccaatccgac gcccccatgg gatttccggc acggcacccc aaagaaggct gcgcgttcta
20581 cggatgaggc cgacggttcc gcaagcgact gtggcggtaa gtcgacgtgc aagcagatga
20641 ccagctgcgc cgaagcacgc cactatctca acgactgcgg tgtgagccgg cttgatcggg
20701 atcgcgacgg aattccatgt gaatcgattt gtagatagca aagcgagatt agtatgagac
20761 gtttgatagc tttagtgtct ttgctcagga tgttgagtgt tggtgattga agccactcaa
20821 tcaatccata ccctgtcgcc ccaaagcccg ccttgtgcgg gctttgtttt taatccgctt
20881 taagcgacgc ggtgccatcc gcgccgtact ctgtgcccac ataggttctt gggttcggag
20941 ggctgcttca tgggcaaatt ctgggcgtgg ctgatgaccg ccgccgccaa agcgtggccg
21001 atcctttggc gttatatcgc gccgatgctg gccgatggca tgcgccagca gtgggagatc
21061 ctgctgccgg tcgcggagcg gatggttcgg gccgtcgagg attcgctgag ctgggcgccg
21121 aagtccggcg aagccaagaa gtccgcggcc atcgcgctga tcctcaagga gctgaaagag
21181 cgcgaaatcg cctgggcgga ccggattccg gaccggatga tgaaccgcgc catcgaaatg
21241 gcggtcgggc tgctgcccga gaagcagtct gcggagactg cgccgtgacc ggctggaaga
21301 ccaagaccgc cgcgatcgtc tcggtcgcct atgggctgct gggatggctg ctcggcctgc
21361 acggcgcgga cattgccatg cagttcatcg tgcaaggcat cggcatcgcg ggcgtcggcc
21421 acaagatcga aaaggccgcc accccggctc ctcttcccca agggcgaggg gaggatatga
21481 gcgatgagga attcgcggcg ttcgtgcgga ggcagcagaa atgagcttcc agaagccaag
21541 gggcctgcgc aacaacaacc ccggcaatct cgtctacagc ccccgcaacg cctgggaagg
21601 ccaggtcggc cacgacggac ggtttgcccg gttcgagcaa atggaagacg gggtgcgggc
21661 gctaggggtg acgctgctca actaccagag gaagcgccag ctccacaccg tccgccagat
21721 catcacccgc tgggcgccgc cgaacgagaa cgacaccgcc acctacatcc ggcgggttgc
21781 cgccgcgctc gacgtagggg cggatgaccg gatcatcctg tccgaccgga aaacgctcac
21841 cctgctggtc tgggccatca gtacccacga gaacggcgcg atggcgtgcg agcgctggct
21901 caagcatgag gatgtggaag cgggggtgga ccgtgcgttg cgttagcgga ttcccgggcg
21961 cggcgattct ggcgctaccg ctaacgctgg cggggtgcgt gcaggtgggg ccgaaagagc
22021 tggttccgaa gccgatcctg acaccggcat tcgcgccgga gtcttgtgct ctgcccgatc
22081 tgccgccgtt ggcctcgacc gtgttcgtcg atatcgcccc gggtcggcct cccaaagccg
22141 atgccggcgg caaagcgctg gtggtcggct atgggcgggc gcgggaggcc atccaagcct
22201 gcaaaggcgg ggccgaaggg cactgatgct gacgccacag gaaaaatgtg tgggtggcgg
22261 ggcgttgctg atgctggcct tctgcatcgg tgtgcttgcc ggttgcgact gtctggcctg
22321 cgagccgaca ggttacgcaa aatttgcgga caccctgagc gtcctccacg gcgagcgtcc
22381 aatcgagttc ggcgccggcc tgcaatgcga attcacggaa cgcaccgatg ccggatctcg
22441 ctgacctgtc cgacgagcga atcgagatcg aacgcgtggc cggtattcgc gccgtattgg
22501 attcgatgca accggatacg ccccaggtgg tgatccccga tgaaaacggc gtgccccagg
22561 tggtctgtta cgactgcctg aacgaatacg aagtgatcac gccgatcccg cccgatcggc
22621 tggcggcgaa cgaatacgcg atccgctgca tcgactgcca gatcgcgtac gaggaggaga
22681 agcggcggca tgcatgagtt catgcgggac aactggaacg tgatcagcga catcgcccat
22741 ttcgcggcgg agatcgccat cggtgcgtgg ctgtacctga gccgcaaaaa cgaccggacg
22801 caatggcgta tcgaggcgct acaaagccat gtggaccagc gcctcgacga gcaaagccgg
22861 cggctggccc aggtggaaac cgacgtgcgc cacgccccca cccacgacga cctgggaaaa
22921 atttacgacc ggatcaacac ggtcggcggc gacgtgcggg agatgaaagg cgtgctggag
22981 agtgccgcca aaacggtcga ccgcctgcaa agttacttat tggaacatga tcggaaaggc
23041 tcatgaacta cgaggacttc ataaccgaag agcgccgcct ggtgattctg cgccggctgg
23101 tccaggcacc ggcctacacc ctcgaccaga ccgtgctgac caaggcgctg gcgttggaag
23161 gcttggcagt ctcgcgcgac cgcctgaaga ctgacctagc ctggctggcc gaacaggatt
23221 tgatcgtcgg ccaacagccc ggcggcgtct gggtggcgac attgaccgtg cgcggtctgg
23281 acgtcgccca agggctgaca gtggtgccgg gcgtggcgcg gccggagccg ggcgaataat
23341 gccgccccgt tcgttggtct atcaactacc gccggaggtc caggaggagc tcaatacccg
23401 cctggtcggc tccggcttcg gcggctaccg ggatctatcg gcctggctga aggatcaggg
23461 ataccacatc agcaagagcg ccctgcacag ctacggccag gaatttgaac tggagttcaa
23521 cgcctgtatg gccgacgccc gcaaggccca ggaactggcg cgggccgccg tggcccagcg
23581 cgacgactcc ggcgcggcgc tgctggaagc gacctccgac gtcgcccagg aaatcctgct

23641 caagctgatg atcggcctgc gcaaggctga tcacgaaccg gacaaggccg ccaagctggt
23701 atcgctcacg gtgcgttccc tcgctgatct gggccggatg acgatcgacc agaagaagtg
23761 gcaggaacag attcgcaggc aagccagcga agaggctggc cagatcgctg agaaagctgc
23821 cgcgcgtgca ggtgtttcga aggacggtat cgcggcgatc cgtgccgcca tcctcacgga
23881 gatggcatga gcacggccgt cctcctgcca taccagcagc gctggatcga ggacagggct
23941 tccgtcaagg tttgcgagaa gtcgcgccgg atcggcttgt cctatggcga ggcggcggat
24001 tcggtactcc atgcctcggc ggaggaaggc ggcggcaacg tctattacat ctctttcgac
24061 aaggagatga cccagggctt catttcagac tgcgccggct gggcaaaagc ctttcatgcc
24121 ggcgcttcgg aaatcggcga ggaagtgctg gccgatccga agaatccggc ccgctccatc
24181 ctgaaataca gcatcagctt tgcctcgggc aaggtcatcc atgccttttc cagcaaccct
24241 cgcaatcttc gctccaaggg gcggcccggc gaccggctcg tcatcgacga agcggccttc
24301 gtcgacgatc tcggcgagct gctgaaggcc gccatggcca tgacgatgtg gggtggtgaa
24361 atccacatca tcagcaccca caacggcgac gacaacgagt tcgcggtcct catcaacgac
24421 atccgagccg gccgctacga ctatagcctg caccggatcg acctggacga tgcgctgggc
24481 gacggcctgt accggcgcat ttgccaagtg tccggtcaaa tctggacgcc ggagaaggaa
24541 gccgagtggc gggcggcact gatcaagcgc taccgcccca acgaagacga ggagctgttc
24601 tgcattccgg cgttcggcgg cggctcctat ctgccgcgcg ccttggtgga gacctgcatg
24661 gtcgacgcgc cggtcgtgcg cttcgccggc tcgaggacct tcaacatggc ccccgaaccc
24721 gcccgccgag ccgacatgcg ggagtggatc gatgccgagt tgccttcgct attggagggt
24781 ctggatcggc gacggcgcca taccctggga atggacttcg cgcgctcgag cgacaccacg
24841 gtgatcgcgc ccatggagat cggcgacacc ttgcatcgtc gggttccctt tctggtcgag
24901 ctccataacg taccccacaa gcagcaggag caggtgctgt tcgccctggc cgatgccttg
24961 ccgcgattct gcgccggggc gatcgatgcg agcggtaatg gctcctacat cgccgagtcc
25021 gctgcggatc gctatggctc tcggatcgag caattgaagt tcacggagga ttggtaccgg
25081 gagaacatgc ccaagtacaa ggcggcgttc gaggatcggc tcatttccat tccacgccac
25141 gacgacgtgc tcgaagatca ccgtgcgatc aaggtggttc gtggcgtgcc acgggtcccc
25201 gaaggcaaga cggacaaaaa ggggcagcgc cacggcgaca gcgccatcgc tgtggcattg
25261 gcgtattacg cctcctatag cgcttcaggt gagatgccaa tggtcgtctc gcgcgcacgt
25321 accggtacgg tcctgtcagg ctacggcggc atcgactgtg gcgcaagcac aaggggttac
25381 atgtgacccg ccgcgccccg accggacttt acctcccctc tggcgagttc gtcgcattcg
25441 ctcaaggtcc cgcgctcggc cagcaactgg tcacccgcca aacggcgctt ggccagttca
25501 gtgggctgac ttggctgccc aacccggatc cgatcctcaa gaagatgggg aaatccatcg
25561 aggtctatcg cgatctgctc tcggacgcgc acgtcgccgg ctgtgttcgc cgccggaaag
25621 cggcggtacg agccatggaa cggggcgtcg aacgcaagga cgccagcgcg cggaccgtca
25681 agaacgtcga ggcgatcctg gccgatctgc cgctggcccg gatcatcggc gagatcctcg
25741 acgccgtgct atacggttac cagcccctgg agatcgtctg ggagcgagtg gggaatttcg
25801 tggtgccggg agacgtcatt ggccggccgc ccgagtggtt cgtctactcc cagaataacg
25861 agctgctgtt ccggagtcag tcctgcccga tgggcgagcc actaccgccc aattcattcc
25921 tgtgcgcccg ccaggagccg acctatttca acccgtacgg cgcgccggat ctggcactgg
25981 tgttctggcc gaccaccttc aagcgcggtg ggctgggatt ctggatgcag ttcgccgaga
26041 agtacggcat gccctggccc gtgggcaagt atcctcgtgg cgccgaaccg ggcgagatcg
26101 atcagctgct cgacagcctg gaacaaatgg tccgcgatgg cgtagcagcg gtaccggacg
26161 attccagcgt cgagttcctg acccatgatc aaagccaagg ggccgacgcg tacgagaagc
26221 tgctgctgtt ctgccgctcc gaggtctcca tcgccctgct cggacagaac cagaccaccg
26281 aagtcagcgc caacaaagcg tcggcggaag ccggaatcga agtgacgcgc gacatccgag
26341 agagcgacgc ggagatcgtc gccagtaccc tgagcgattt gatccggatg gtctgcgaac
26401 tcaatttcca cgaaccggct ccgcccgtct accagctgtg ggaacaggaa gagatcgacg
26461 aaacgcgggc cgtgcgggat aaagccttgt acgatgccgg ggtgcgcccg accaatgcct
26521 attggatgcg ggcctatggc ttcaaggagg gcgaactggc gccagaagct tcacccagtg
26581 agagctacgg cgctccggcc ttcgcaggaa tcgaccttga tctgttcccc gaccaggccg
26641 cgctcgatgc ggcagtagac gcgctggccg aaggtggcgc cttacaggct caggctgaac
26701 ggctgctcgc gccgctcatc gagcggatcg aaagtgcggg tggcgaagcc gaactgctcg
26761 gcgcgctggc ggaagcctat ccggacatga atcccgacca gctggtgcaa acactgaccc

26821 ggctgctgtt ttccgcgagc gtcgccgggc ggctggcgac cgaggagagc ctgcaaaatg
26881 ggtaagtacc tgcccgacat ccgccagggc gatacctacc gggtccggat ccggtatccc
26941 aaggggtcca acatcgccgg ttacatccac tggctggcct tgcggaccgc tatcggtacc
27001 gaacctccac tgctgttgct gagatcggtg gttggcgaac atccgtctga ccagccggag
27061 gacgggatcg cctacctgga agccacgccc gagcagacgg ccgcattgcc ggcgaacaag
27121 cgctgtgtgt atgggctgct ggcgaaaaca ccttcgggcg aggtcaagac ccttcgtcca
27181 ccactgaagg atcccctcga cgaccgcatt ctgatcgttc cacggggcgg cgccgatggt
27241 gtgtgaagca accgttgagg tcgattgtgt cgacgtcgct ccaaccgtgg atgtcgagaa
27301 cgcgccgggg ccgcgaggcc ccgtcggtcc ccaaggtccg cctggtccgg agggaccggc
27361 tggcgggatc gatccggata ccgtcatcga cggcgggaat ttttgacctg aactcgagag
27421 aaacgaacca tggcaaacgc agtacgcatc aaacgcaggg caagcggcaa cgccggcgcg
27481 ccggccgctc tcctcaacgc cgagctcgcc tacaacgaag tcgacgacgt cctttattac
27541 ggcaaaggcg attcgggagc aacgcgacct cgatcccggc gatcggaggt ccaggcgcct
27601 tcgtcaccaa gtcgactgcg cagaccatcg acggcgccaa gacctttacc ggcgcgaatg
27661 acctcggcgc tcaaaccacg gcgacgacgc cggcggcggc cgacaacagc aacaaggtcg
27721 ccacgaccgc tttcgtcaaa gggcaaaact acctgacggg caaccaggtc gtcaccctga
27781 caggcgatgc gacgggatcc ggtgcgacct cgatcgccgt gaccattccg aacgatacgg
27841 tcagcaacgc caagcttgcc aacatggcgg ccggcacgct gaagggcaac aacacgggca
27901 gcgcggcgga cccggtcgat ctcacggtgg cccaggtcaa aaccctgctg gcgtaccagg
27961 ccaccgatat caccggtttc gacaccctgg tgcgaacgtc ccgtctggat cagatggcgg
28021 cgccgaccgc ggcggtatcc atgaacagcc agcggatcac caacctggcc gatcccacgg
28081 gcgcccagga tgcggccacc aaggcgtacg tggactccgt cgcctcgggg ctggactgga
28141 aacaatcggt ccgggccgcg accaccgcca acatcacctt gtccggtacc cagacggtcg
28201 atggcgtggc gttggtggcc ggtgaccggg tgctggtgaa gaaccagacc acggccgcgc
28261 agaatggcgt ctatgtcgtc gcttccggcg cctggagccg cgcgaccgat ttcgattcga
28321 acgccgaggt caccgccggc gcctgcatgt tcgtttccga gggcacgacc aacggtgata
28381 aaacctgggt gctgacgacc aacgatgcga tcgtgctggg tacgaccgcg ttggcgttcg
28441 cccagatgtc cggaggcgcc tcgggcgaag tgaacaccgc atcgaaccag ggggcttccg
28501 gtgtcggcgt tttcgacaac aaggtgggtg tcgatctgca gttccgcaac ctggtggcgg
28561 cttccagcaa gattgccgtt accctcaatg ccggcaacaa gaccatcgag atcgacctgg
28621 cccaagccaa catcaccgct gtcggcacgc tgacggcggg tgtttggcag ggcaccgcca
28681 ttggtctgca atacggcggc accggtgcca acctgtccgc cgccgccgat gacacgatct
28741 tcaagaaatc cggcaccgcg ctggttgcgg caacggcggg cagcgactat ctgaacaacg
28801 cctcgaccat cgacggcggg acgttttgac gagcttccgc catgaccaat accgtccagt
28861 tcaaacgatc gaacacagcg gggtccaatc ctggggctct tgcgtctggt gagcctgccg
28921 tcaacctggc tgatcatcgc ttgttcctgg gtggcgccgc cgccgagggg tatccgcttg
28981 gttgccagtc taggccttcg ccgcggttgt ccgggaagcc caaagtcgcg ggcgcgggtg
29041 gcgggacaaa gttggccagc gccacggtaa cggcgcaaca gcaatatttc gtgccgttcg
29101 tggtgccgcg gccgatgatg ctgtcgcaac ttggcgccta tgtggtcgtg aaccagttca
29161 gcaccgtcat ccatgtcggg atatacggca acctgttcta ttcgggcgag gatcatcccg
29221 gcccaatcat cgcagcggtg acgaatttga gttcggcgac gaccggactt aaaagcggct
29281 cggtgagtgc gacgctggag cctgggcggt tgtactgggc gagcgtgctg agcagtggta
29341 gtcctggctt gattggcatc gtgacggccg ccgccaacgg cgactacgcc gtcagcgaca
29401 gcgactacat gaccgcaatc actcaccagt atttgtctgg cgtgtcatcg atggcagatt
29461 acgccagcgg cgctctgggt gctgtcgttt ccggtacagg gacggccccg gcggtgtacc
29521 tatacgagta acgctatgta tttgatgtag agccgaggtt gaaatgttta atacagtttc
29581 ttctcttagt aagccactca tccagtccgg gcaggtcggg accactccgg tcgaaattga
29641 cctgcttcag catcgccccc catttcggat aacgctgatt ccgggctctg gaaacacttc
29701 gttgtgtgaa atcaaacacg ccggggaatg gtttccgtgg ccttttggga atatcacgtc
29761 caaagccacc gatgtccagc atggaccgat tgaggccgtg aggtttacac gcgtgtctgg
29821 gtcatcgaca gacacttacg gagtgatggc atgagcggct gggaaagcga gcgcattgct
29881 cgccgatcgt tcgaaaaatc caattccttg acgcctgctt ggattgctcc gggcggctgg
29941 gcattggcgg tcgggcagga attctatgcg gaggttgggg ggcggaatct gatcgtcccc

30001 gccaaccgcg acatcatcct gccggaatcg ccggcgccag ggacggacta tgcgatctgg
30061 ttgagtccaa acggggttgt gcaggcctcg tccaatttcg tgacgccgcc gaatacatca
30121 gtcgaccgaa agatcggtgg attccattac gcgcctggcg gcaatgctcc tgcgcaagcg
30181 ggcggcaata cgacgcctgc tatcaacccc tactcaatgt gggacctcaa tttccgcccc
30241 gcctgtcccg acccgagagg catgacgcta gtagcagatg cgttctggat agacatctac
30301 ctgctcggcg ttgatcacct ggtgaatgga tccagcaagt acaacgtcac aatagccgac
30361 ggctcgtctc ctcccaaaaa gtctacgaag ttgggcggcg acgggaccgc cacctatgcc
30421 gacggctcgt ggcacaacat gggggatgcg ctcgcgcatc acggcaagcg gcatcccttt
30481 tacgctgaag cgcgcgttgc gttctttggc acgaccgagg cgtcgtctct tggtgcggac
30541 cctgggtcca ctgtcctcaa cgcgccatac acttctaaat ggggcgtgat tcaatcgact
30601 ggcgtgatgt atgtatgggg gtccgatttc ggtggtggtg ctgctggcgc gacatggttc
30661 actaatacgg gcggccgagg cagtacatac caggccgaga acgccgtggt gctggcgggc
30721 agctggggca gtgggagcca ctccggttcc ggctgcgcga tctggaccta ctcgcccgcg
30781 acctcgtaca acaacatcgg ggcgcggggc gtctgtgacc acatgagacg tgattgagct
30841 gggcgatagc ccagccgacg cgattttttt ggatggaagg ctagatgaaa cccgtcgcgg
30901 acgctgttca gtcctatgac caaatggcca tcgtcgaaaa atacgaggcc gtcatcgcct
30961 atctttaccc gatcgcacag tcgatgcctc gtaagcacag cgtggcgagg gatatgtttt
31021 tgcgatgctt gctcggacag cccgatctct tttaccaggc cgggaaatcc aatcaggtat
31081 cgaggattta tgccgcattg gagatctgct atgccaaaaa ccagcattaa cacgcgctca
31141 gacctggatg ccatcgcggg ctcaccggag cacgccgagt ggatggcgtt tctagctgga
31201 tcgctgtggc gactggagag agacgacgca aaccagacgt gggtcgccgt ggaggacaat
31261 tcgacgatcg agcgctacgg gttcgttcga gctgattttc ctgatgcaat tcggcccgag
31321 ttgccggttt gggagccggg cgttccgcat gtgccgcaaa ccgtctcgcg ttttcaagcc
31381 aaggcggcat tgagcaacgc cgggcttctg gcgcaggttg acgcactgat gtcggacccg
31441 gcaaccgatc cgattgcgcg gctggcgtgg tcggatgcgc aagaatttca ccgcgccagt
31501 ccgacggttg ccgctatggc gcaggcgctg gggttggacg ccgccgcgct tgacgaatta
31561 tttgtgacgg cagcatcgat cattgcatga gtttgtcagc cgccgacctc aaggcgattt
31621 tcaatctcga gcccaaggcc gcgatcgcct acctccagca caagggctac aagctcacct
31681 ggaactggca ggagatgctg gacgatgccc acgcacgggc ctttaccatt gcgaaggcgg
31741 cgcggctgga cgtggtgcag gacattcgga atgccctcga cgtcgcgctc cgccacggcc
31801 aaacgctcaa ggacttccaa aaaaacttga agcccacgct gcaagccaag ggctggtggg
31861 gcaaacagat catcgttgac ggcgccggga atgccgaggt cgcccggctg ggcagcccat
31921 ggcggctggc cacgatctac cggacgaacc tgcaatccgc cttcatggcg gcgaattacc
31981 aagaaatggc cgaagccacg gacagccacc cctattggca atacgtcgcg gtgctggacg
32041 gccgcacccg gcccagccac cgggccatga acggccgagt gttccgccat gacgatccca
32101 tctggaacac gatctggccg cccaatggct tcaactgctt cccggcggaa acgccggtcc
32161 gggccgccgc ccggctgggg ctaaaaacct ggtatgcggg caaagtggtg gagctgcaaa
32221 cgcggctcgg gcaccggctc accctgaccg ccaaccaccc cgtattgacc gtccggggct
32281 ggatcgcggc ctgccaactc cagaaaggcg atcagctgat cggcgacgca agcggtgtca
32341 atccccgtct ggctggggtc gtaaacgacg aacagccgcc agcccgcgcc gaagatctct
32401 tccagacgct ggcggcgcag ggatttcgca tcgttccaat ggcgccgcac gatttccacg
32461 gcgacgcggg cttgcgcaaa cccgaaatcc acattgcggg tcccgatgtc catctgatgg
32521 acgaagtcca ggccgcgccg ggtcaattcg tcggccagcg gcaactccgc cgggctgacg
32581 cttgcgcgat catggacgcc gaccgccccg acggcccgcc gccggcccgt atgatcctgg
32641 cggatgccgt tgctccgcag aatccggctg acgttgccga ggctggtgcc gagctggcgg
32701 ccgatggcgc gtttggagat cagcccgttg cggtacaggg acaacacccg gcgttcgaga
32761 tgggagtcgc ggttgcgggc gcgctcccag gcggcggcgc attggcgagc aatggcggcg
32821 gggtcctgtt tgatggatcg ccatttgacg cgctcggctt ccgagcgccc ccgcaggggg
32881 atgtcgcggg tacggaacag ccggcccagg gcgtcacggc tgcatccggc ctggtccgcc
32941 agttgcttga ggctaacgcc ggcctgatag cgctggatga gatcgtccag attcggcaat
33001 tcgattgggc gggccatgtc tatgatttcg aaacggagac gggcctgatt atggccggcg
33061 gtgtgattgt gcacaactgc cgttgccggg tccggccgag atccgaacga agcctcgcgc
33121 gtgacgggat cgcctggcag tcttccgcag ggaagctccg aaccctcgaa accgatgccg

33181 gcgtggacaa acgtaccggg gagataacac acgctcggcg caccgggatc gacgtggtcg
33241 acgccgacgg gaaaaagcat ttcttcgcgc cggatgccgg gttcaacttc aatcccggcc
33301 aaggctggag caagcctttc accccgccgc cgctggatac cctgccgaag acgttttcgc
33361 cggggcaggt cttgcccgac ctgccgaagc cggaaaaatt cgccgcgtcg atgatcgtgc
33421 cggatggttt gggcgaggag gattacgcga aggcgttctt ggtagagttc ggtgccgacc
33481 tcggcaagcc ggtggtgttt caggacgtca ctggagatcc gatgctgatc gacgaggctt
33541 tgttcaaatc cggtgctggg gaatggaaag cctccaagaa tggacgcggc ccttatatga
33601 ggctcctggc gcaggcgatc aaacggccag acgagatatg gatgcgctgg gaggagtcac
33661 gagacaaacc cggcacctgg cttctcaagc gtcgctacat caagaccttc gaagttgaag
33721 gcggggatcg ccaatcgccg cagtttggct tgtcggtgtt tgaattcggg caggacggtt
33781 ggagcgggtc gacagccatg atggcaaagc ccgaccgtac tgaagccgca agacgccgtt
33841 acgtcaatga acagcgggat ggattccttg cttatcggcg ataaacccta cgccgccccg
33901 cagggttcac cgtgcggctc ctttggacgc gtcgggcggg agcgattgta cgaccgcaat
33961 cggttgcagg cgattatacc gcgatgagta atccgatcga agtcaccatc gacgacatcg
34021 aagtccgcca aatgctggac cgcatccagc gggccgggct aaacctcggg ccgctgatga
34081 aaaccctggc gatgaccctg aaggacgaaa ccgaacaccg gttcgagaac gaagggccgg
34141 gatggcctgc gctttcggat tccacgaagc aggcgcggac ccgcctgggc cactggccgg
34201 gacagatgct ccaggtgtct ggccagctcg cggcatcgat ctcgaccgaa tccggcgcgg
34261 acttcgcacg gatcggctcc tcaaaagtct atgccgctat tcatcagttc ggcgggtccg
34321 cgggacgtag ccaacgaacg acgattccag cacgtcccta tctgccgatg acggccaaag
34381 gcgagctgac gccccaggcc agagaagcgg tgctcgatga tacgatggca taccttaagc
34441 gggctgcggg cgtctgaggc gcgttccccc gggcagacgc gccgctgcga atctccggcc
34501 cgtgacggcg cgtaaagccc ccatgcgcta ccttggatcg tcttttctgc ctgcgcgatt
34561 tctaacgggg gtttaacgcg ccacagcggc atgttcttgc tcaaccggta cccctgctgc
34621 gatcatcgac cgggatttcc tccgattacg ctgcgctaat cggagctacc aggcttcatc
34681 aggttccgcc ggtcccgcac gtttttaatc cacgctaaaa gaaaccccgc ttccggccgc
34741 ctaatctggc ggcatgacga accgcatcca ctgtttcaag ccgggccgcc acctgccgat
34801 gggcgggtcc cagccgatcg agttctccga gcgggatctg cgcgacgcgg ccgcttgcta
34861 cgacccggct ttgcatcggg caccgctggt gatcggccac ccgaccaggc tcgatccggc
34921 ctatggccac gtcaaggcgt tgagctacgg cccggacggc ctcgaggccg agccggagga
34981 ggtcgagccg gccttcgccg agctggtgaa caaacaggcg ttcccgaacg tctcggtcgg
35041 ctggtacgcc cccgatcatc cccgcaaccc cgttcccggc aagtggtacg tccgcgaggt
35101 ctcctttctc ggcgcggtgc cgccggcggt gcgcggcctg cgccggccca cgctcaaccc
35161 ggcattcgcc gcggacgagg acggcatcat ccgcttcagc ggagaacgcg acgacagcgt
35221 gaacgccggc ctctggcggc gattccggga atggctgctg gtcaggttcg gaaccgagga
35281 tgccgaccag gtcgtgccca gtgccgacct gcagtatctg gaagatgccg ctcgcgagga
35341 aatccgcgat gaagcgggcg atccggcaac cgcatcccaa cccgtttttt cccaatccac
35401 tccggaggac aacaccgtgg atcaagctca agccgccgcc ttgacggcgg aaaacgaacg
35461 gctcaaagcc gaactggccg cagcgcgtca gcgcgagtcc gcggcagcgg aaacccggcg
35521 ccgggacgac gcggtccagt tcgcccagga gctcacccgc ccggacggca acggccgggt
35581 tcggctggcg cccaagcaca agaacctcct ggtcgagttg ctcatgctgg ccggcaaagg
35641 cgccgatgag ggcggtctgc cccagttcgc cggcgaggac ggttcgaccc gtcccctggt
35701 cgacgccgtg aaggccatgt tcgccgaagg cggtgcggtg gtgcagttcg gcgaattcgc
35761 cactgcggac cgggccggca aggcaccgat caagaatccc ctcgtcgccg acgcggaacg
35821 ccgcgcggca gccgttaaga ggtaggccgc gatgccaagc cataccgacc cgaaggtcat
35881 ttccgacgtc ctgctgtacg aggtcggcga gcaatggtcc cgcgaccgcg tgacgatcgc
35941 cgccggcgcc gatctcgctc tcggtaccgt actcggcaag atcaccgcca gcggcaaata
36001 caaactgcat gatcccgcgg ctgccgacgg ctcccagaat gccgtggcgg tcctgctggc
36061 caatgctgcc gccgccggcg cggatgtcgc ggcgcccgtc atcgcgcgcg gcgccgtgct
36121 cgacgccaat ggcctggtct ggaaaagcgg tatcaccgat ccccagaaag ccactgcccg
36181 cgccgcgctg ctggcgctgg gactcaaggt catcgactcc gtctgaggca cgccatgaat
36241 ctagccgatc tgttcacagt cactaccctg accgccgcgg tcaacaaact gccgtatgcg
36301 acctacaaga tccgtgacct cggcatcttc gccgaagcag gcgtgcgcac cacgaccgta

36361 gcgatcgaag aagaccaggg acggctgcac ctggtgccca accggtcccg caacgacgca
36421 cccgaagtgg tgcgccgtaa gcggcgtacc cgccgggtgt tcgagacctt ccacctgccg
36481 gaggccggcg tgattctgcc ggaagacatc cagaacgtcg ccccgttcgg tgaagacatg
36541 accggttctt cactggagcc gcaggcgcgc gtcatcaacg acaagctgca gatcatgcgg
36601 gactccatcg agatcactcg cgaatggcag cgggtcggcg ccctgggcgg gcagatcctc
36661 gacgcggacg gcacggtggt ctacgacctc tacaacgagt tcggggtcac caagaaaacg
36721 gtggacatcg ctttcggcac caacaacctg gacgtccggg cgaagatcgt ggaaggcaag
36781 cgccatgcgg aacagaagct gacgggagcg gcggtgacgg ggtttgcctg cgccgcctcg
36841 aaggagttca tggacctcct gaccgaccac cccaaggtac aggccgccta cgccaactgg
36901 caggcggcgc aggaccggct gggaggcgac atgcgcaacg acttcacgtt cggcggcgtc
36961 cgcttcttcg aactggacgt cacggtgtcc ggccagcgtt tcatcccggc cggcaaagcc
37021 cgcctgttcc cgcttggcgc cggtgtattc cagatgcaca acgcgccggc caattacaac
37081 gagacggtga atacccaggg gcaaccctat tattccaaag gcgaaccgcg gaagttcaac
37141 aagggctggg acctggaagt acaggccaac ccgctggcgc tgtgtctctt cccggaagcc
37201 ctggtcgagt tcggggcggt ctgacgtcct gccatgcgct actgcaccct cgacgatctg
37261 cagaagaaga tcccgccccg tacgttggcg cagctgacca acgatacgcc gccggcgacg
37321 gcgccgaacc tggatctggt cgaggagatc gtggctggcc aggaagaact ggtcgacggg
37381 tacctgcggc agcgcaacct gttgccgctg acggaggtgc cgaccatcgt acgggacgtc
37441 gtggtacagc tggtgcgcta cgagatctac gaccggcggc ccgagggcaa ggacgatctg
37501 ccgcccgccg tggtacgggg ccgaaaggac gccctgcaga cgctggacga cattcgcgcc
37561 ggccgcatca gcctgggcgt ggcggacgat cccgcccagg ctcagcccga ggccggcgcc
37621 cggatgcgga cccggatcca gggtgagcgg ctgttcacgc cggacgtgct ggaccagtac
37681 cgaggctcgc ggtgagcgga gtcgaaccga acgtggcggc caacaccatc gaggcgctgg
37741 aggcggcgat cgtcgcccgg ctcggtgcgg tactgacgga tgtcgaagtg atgccgtatc
37801 cggccgatcc cgctaactac cagtttctgc atccggtcgg cgctctccta gtccggtatc
37861 acggcagccg ctacggcgcg ctgatggaca cggatgcggt ggtccaggag cgcctcctgg
37921 cagtcgagat cactctcctg ttccgggcgc tcgacggcca ggacgggctc tacgcctatc
37981 tggagcgagc gcggcgggtg ttgaccgggt tcaagccggc ggggttcggc aaagtttacc
38041 cgctccgcga cgcgttcctg gaggagcacg gcggggagtg gcgctacgca gtcgacttct
38101 gcgccccgac cctggcgatc gaggacggct gcgaggagga cggaccgctc ttgaaacatg
38161 tcaccacgtt ggacggctat gagcgggccg aaacggtccg tcagccagac gggagcacga
38221 cttacgagga gtatgcgcaa tgacacgata ccgctacgac ggcccggtga gcagcgtcac
38281 gctgccgggg ggccttgatg tcaacctgca cccgggcgcc gaggtcgagc tgccggaaga
38341 caacgactat gtcggggtcc tggtggccaa gggtttcctg acggccatcg tctccaaccg
38401 caaacccaag ggtgccgacg cgcccgccca ggagggatag aacatgccgg cgaattttct
38461 acacggcgtc gaaaccatcg agctggacaa ggggccgcgc ccgatccggg gtgtgaaaac
38521 ggccgtgatc ggcttggtcg gcaccgcgcc gatgctggac gtcgatgcgg ccgatcgctc
38581 gttgaaccgt atccggctgg tcaccaacga tcgcgatgcc gcccggtact tcggccagaa
38641 ccgtgccggc ttcaccattc cgagcgcgct caacgccatc ttcgaccagg gcaacggccc
38701 gatctgcgtg gtggtgaacg tgctcgatcc ggcgagcgac accaccgccg tggcggacga
38761 ggcgaagact ttcgatgccg cgaccgacaa gctgacgctg gcgcatcccc aggtatcgaa
38821 cgtggtggtc aagcacactg ccggcacgcc gacctatgtg ctcaacaccg attacaccct
38881 ggatgccgcc aacggcacga tcacccgcaa ggccggcggc gccatcgcta gcggccagtc
38941 ggtgaaagtg agctattcct ggctggaccc ctccaaggcc gtgaacagcg acatcatcgg
39001 tgcagtcgat ggcgcgggca accggaccgg catgcaggcg tggctcaatg cctaccagga
39061 actgggattt ttcccgaaga tcctgatcgc gcccggattc tcgaccgtga acgccgtcgc
39121 gaccgaactc aacgtattgg cggggaagct gcgcgccatt gccctggtgg atgcgccgat
39181 cggcacgacc gtgcaacaag cgttaaccgg ccgaggtccc tccggcgcca ttaacttcaa
39241 ttacagctcg gaccgggtgg ggctgtgcta tccgcatctg aaggtgtacg acagcgcctc
39301 cgacacagaa atcctggagc cgttctcgcc acgcctggcg gggaggatcg ccgcgaccga
39361 ccaggacaag ggttactggt ggagtgcgtc gaaccaggag atcaagggga tcgtcggcct
39421 cgagctgccg ttgacggcga tgatcaacga tccggcctcg gaaaccaatc tgctgaacga
39481 ggccggcatc atcacggtgt tcaacgcgtt cggatcgggg atccgcacct ggggcaaccg

39541 ctcggcagcc tggccgtcga acacgcatcc gcgcaacttc atcaacatcc gccgcaccgc
39601 cgacgtgatc cacgaaagca tcgagtattc gatgctgcaa ttcctggatc agccgatcac
39661 cgatgccctg atcgacgcca tcaccgagag cgtgaacctg ttcctgcgca ccctggtgat
39721 gcgcggggcg ttgatcgacg gccactgcgg ctatgacaag gccaagaacc cggtgaccga
39781 gatcgccctc gggcacctga ccttcgacat cacgttcatg ccgccgcccc cgctggagcg
39841 ggtgagcttc gagagcttca tcgacatcaa cctgctgtcg cagttgggag gtaacggctg
39901 atggatgagg tatggctcag gaatctgaac cggtttcagg cgctgactgc aaaaatgtca
39961 cgaaagcgcg catgttttcc tgccgggact ggtcgaactc cgtgcgaagc tcggctagca
40021 gcagttctag atctcgctct ggcgaagctt ggcggatcag tctcgcccga acatccagga
40081 cgtcttgaac gagcgtttcc agggtagccg tggtatgcgc gatttgcagg gccagtttgg
40141 cgtaaagatc aggcgtcatc gaagtctcgc gttcaggagt gtgaggacaa gtatatggca
40201 aagatagaaa tcaacaggct caccaacgcc aacgtctacc tcgacggcgc atccttcctc
40261 ggccgggccg aggaggtcga gctgccggac atcaagcaca agatggagga gcacatggcg
40321 ctggggatgg tcggcaccct ggaggcctgg tccggcatcg agaagatgac cgccaagttc
40381 aagtggtcgt ccttctacaa ggaggtcatg gccaaagcgg cgaatccgtt caagtcggtg
40441 gccgtccagg tgcgcggctc gctggaaacg tatgcgactg ccggccggat cgccgaaacg
40501 ccggtggtgg cgcacctgat cggacagttc aagtcgatcc cgatgggcaa ctacaagcag
40561 cacaagaacg tcgagcagac caacgaaatg gccgtgcatt acgccaagct cgtcgtcgac
40621 ggcgccgagg tgttcgagtt cgacgccctg gccaacatct acaaggtcaa cgggcaggac
40681 atgctggcca actaccgcgc caacatcggg ggttgatatg cgtctggact ggaatttggc
40741 gcggtctgtg ctggaggctg ccgaagcgct cggggacgaa aaagataggg tggcgccggg
40801 cgccttcccc ggcgtggccg aagaagtggc gatcgagcac tttcggctgt tgtgcgaggc
40861 cggactggcc gacggttacc cccagggccg atcccccctg tttctgacgc ggttgacttg
40921 gagcggacat caattcctgg cgaccttgcg cagtcgcacg ctctggtcgc gagtcaaagc
40981 cgaggcgaaa gaccgcgggc tcgccttgtc gttcgaggtc atcaaggcgc ttgcggcgaa
41041 gctggtcggc cagctcgtgg actagcggat cgttaccccc cgacgcattg caaaaaagga
41101 tcgaaccatg aacaagatca agcttcacga acccttgaag accggcgatg gccgcaccct
41161 gacggagctg accatgcggg cgcccaaggt caaggacctg aaggcggccc agcgtttcgg
41221 cggttcggat gccgacgtcg aagtggcgtt gatcgcgtcc ctggtcgggg tcgtgccaga
41281 ggacctggat gaactggggc tggcggacta caggcgcctg caggacagct ttcgccggtt
41341 ctctgatccg gacggcggag ctgtggaagg ggatggcgct gctggcccgg tggttccggt
41401 tccagcccag tgagatcgat gcgctggagc tggaggattt cctgacctgg tgggatctgg
41461 cgaaccagca gatcaggctg gaggccggac cagcaggcgc gtaatgccgt cgaacagccg
41521 caggatgacc cacaagacga acagggcgcc ggccatcaaa tagccgatca gccaggaaag
41581 cagcagcgcc aacaacaccg cggcgatgat ggacttgccc gcaacccaga acgcgacaac
41641 ggctccaaag agtgcgaaat acgcaatcca aggggcgcgt tgttcgatgt tctcaatcct
41701 ttcggcaagc gcggcaaagg gattcggcgg gttcacgttt caatccactc tcggctgtac
41761 gccgggcgaa gcaaggttaa cacatggcta aagagcttgc cttcgggatc gtgatcgggg
41821 cggcgctgtc aagcgccttt accgccgcct tcggcaatgc caagaagacc atcgacacgc
41881 tgggcgccag cgtgcgcgac ctgactgaca agcagaagac gctgggcgcc acgattcaga
41941 agtccggcag gctggcgcaa gaatccctgg cgccgcttca tcgcgactac gaacggctgg
42001 ggcgtctgat cgacgagata cgacggcgac aggaggcgct gaccgccagt ctggcgcgcg
42061 gagccgcgct caaacaggag cgcgccgatt tgcgggggca ggcgttggaa accgtcggaa
42121 ccggcgcggc gctgggcgcg ccggtcgtgg cgtccgtccg gctggcgggc aatttccagg
42181 accagctccg cgatatcgcc atcaccggcg agttcacgac cgcgcaggaa aaccgcttgg
42241 gcgccgcggt gcgtgagagc gccctgaagt ggaaccagac ccaggccgag atcgcttccg
42301 gcatcggggt gctggtggcc ggaggtgtcc aggatgccca ggcgctggac cggtatacgc
42361 cggtccttgc gaaagcggcc acggcgacgc gggccagcat ggccgatctt ggcagcgtgc
42421 tgctggcatt cgacaacaat ctcaaggttt ccgcggacca atccgagtcg gcgctcaaca
42481 tgctggccta cgctggcaag cgcgggcagt tcgagatccg cgattccgcc aaatggctgc
42541 cggcgctggc gccgatgttc cagaccctgg gcgtgacggg caaggaagcc gtggccgaga
42601 tcggtgcggc actacagatc gcccggaagg gtgcgggcac caacgacgaa gccgccaaca
42661 acttccgcaa ttttctggcg aaactgacct cgccggacac gttgaaggac ttcgacaagg

42721 ccggtatcga tctgcagagc agcttgaaga atgccgccaa gcgcggcatc agtcccatgg
42781 aggcgatgat ggataccatc accgcctata tcggcagcaa aggcccgcag gcggccgcgg
42841 cattcaagca ggctctgtcc ctggaagacg cgcagaaacg tgccgaggcc ctccaggcgt
42901 tgtccggttc gttcaggctg ggtgaattgt tccaggacat gcaggcgatg tccttcatcc
42961 gcccgatgct ggcgaaccgg gccgagtaca aggacatcaa gcaaggcgcg ctcggcgcgg
43021 cgaatcagga cttgataggc gccgacttcc aaaagcgcac ccagggtttc aatgaaagcc
43081 tgaaagcctt ccgtattggg atgagtgagg ttggcctggt ggtcggcgaa gccctgctgc
43141 cgccgctcac ggatctgttg cagaccgtcc gtcccgtcat ccgggaattc gggcagttcg
43201 cctcggcaca tccgggcgtg attcggggtg tggtcgggtt aaccgccggc ttgctgggtg
43261 ggaagcttgc cgttttggcg gtgagatatg ccgtcaacct gctggtgtcg cccttcaatg
43321 cgttggctac ggcgactcaa ttggtattgg gtaaatggac actgctcaag accgctttgc
43381 agttcgggcc gctggctcga gccggcgggg ctttgagcgc cgcaggttcg gcggcgctgg
43441 gcgccggtcg cgccctgggc gggattttgc tttccggctt tcgactcgca ggcgctgcaa
43501 cgctggggtt tggacgcagc attgccagcc ccttgctttc tggccttcgt ttggccggcg
43561 cggccgcggc tggatttggt cgtatcctga tcggttcctt ccttgccggc cttcgactgg
43621 cgggcgtggc ggcgctggaa ctcggacgtt tctccgccgg ggccttgctt tctggccttc
43681 gtttggcaag tacggctgcg gcggggctcg gtcgcattct gatcggctca gctctcgccg
43741 gccttcggtt ggcctggctg gccgcgctag agctcggacg gattcttggt ggcgcattga
43801 tttccggtct caagctggcg gctcaggccg tcatgttcct cggccgcgca ctgctgttca
43861 cgcccattgg cgcagccgtt gccatcatcg ccggagcggc gttcctgatc tggaaaaact
43921 gggatgcatt gaaagccagg ttcgcgcctt tgtgggatca ggtcaagggc atctttggcc
43981 gggcgctgag ctggttcaag acgctgccag acgttttcaa agccatcggc tcgaatctgc
44041 tggaaggact gaggtccggc atcatggcca aatgggagtc ggtcaaggca gggttgtcga
44101 gcattgcttc cggcatcaag gacaccttca agagcgcgct ggggattcat tcgccttcgc
44161 gcgtattcgc cggctacggc gccgacatcg gacagggatt gattgagggc gtgaccgggc
44221 agaaagacgc cgtggcggag acccttggca agctggtgcg gtttccccag ccgcctgtca
44281 ttcgggtcca caccgaactc ggttcttcga caggcccgga gcgcagcgga ctggcaccgg
44341 tatccctcaa tgccagtctg gggcgggaaa ctgggcgtgt gctgccgttc gccaaacccg
44401 actcgaaggg caacggcgcc gactcgaagg gcaatggagt tgaatcgccc gccgcgacca
44461 ccgacacccc tgcgcgtctc ttggggcgta aattcggcga cgccgctctt cgagtccgtc
44521 agggcagcga tgtcgccagt ggcgcggacg gacacgctcg caccttccgc tccgtgcacc
44581 agccccaggg gcgcagcggt gaacaggtgg tgatccactt caacccgacg atccacgtca
44641 gcggcgcgga ggatgccggc aaggccaagg cagccgtcac cgaagcgctc gagttgtcgc
44701 tgcgcgactt cgaacgcgtg gctgcgcagg ccagccatgc cagggcgagg acgggctacc
44761 ggtgaggact cgaagagcag cgtagctgaa acggtgcggg agtggctgcg tgatgatcgc
44821 ggcatagtcg ctcccacaga agccgcttcc gccgggttgt tttaatccgg tttaagcgac
44881 tcggcccctc ggacggctta ccctgactcc tggatttaca gggttaggca tgagctacct
44941 gcaactgggc aacgtacagc tcgatctgat cacctggctc ggcggattcg aggcgggata
45001 cggctatgcc tacgccagcc acgacatcat cgaaggcaag agccatctgc aatggaccgg
45061 cgatcagctg gaaacgcggg cgctgtcggc gcaattgcat gcgcgcttct gcgacccgag
45121 ggccgagttc gagaagctga aaacagcggc ctcgcgccac aacgcgcttg cgatgttctt
45181 cgccaacggc acctacgaag gccggttcgt catcgaggag ctccagcgca cgctggagcg
45241 gaccgaccgg ttcggaaacg tctacctgat gcaggtacgc atccagctca aggaatgggc
45301 cgagcccacg tcctcgtcgc tggccggcag cctgaacgcc aacgcccggg cgcaggctga
45361 agcgatcgcc gagaacgcac cggcccggca gctcaaggat ccctccttcg ggccgacggc
45421 cagccaggtc cggctcaagg agcttgcggc caaatacgat accttcaccc gccagcccgt
45481 tcccggattg atccgatgac cacctacctg tcctacatca cacaggaagg cgaccgctgg
45541 gacgggctgg cctaccgctt ctatggcgat ccgttccgct acgagccgct ggtcgtcgct
45601 aatccacacg tcccgatcgt tcccgtgctg ccctcgggtt tgacgctggc ggtaccggtg
45661 ctcgcgaagt cggacagcac gccctcggtg gaaagcctgc ctccgtggaa gcgcggcgtt
45721 ccggcgggag gcgtggcatg accccccgcc tggactcgaa gagcaacgga gttgaaacgc
45781 cggtcgagga cccagtctat tcggtcatct acgacaacgt gaacatcacg gcggacatct
45841 cgggtctggt gaccgagctg atctacaccg atcacgagca cggcgaatcg gacagcgtcg

45901 aactcaagat cgaggatcgc gagcagcgct ggaaaaacga atggtatccg gacatctccg
45961 cgcgcctgtc cgtctcgatc ggctatgccg acggccgacg gctcgattgt ggcgacttcg
46021 agctggacga gatcgaattc gacgtcatgc cggacaccgt gcgcatcaag gcgctggcca
46081 cggtgatcac acccaagctg cggacgcctc gatcctacgg ttacgacgac acctcgctcc
46141 gcgcgatcgt gcaacaggtg gcggcccgca acggcttgac cgtgcgcggc gacatcgagg
46201 gcatcgcgct ggatcgcgtg acccagaatc atgagaagga cctggccttc ctcacccggc
46261 tggcggaaga ctacggctat gccttcgccg tgcgcggcga tgcgctggac ttccattcga
46321 tcgcgacgct cgaggcggcg ccgtccgcgt tcagcctgca tcgccggcag ctcaaacact
46381 gcaccctctc ggagaaatcg gaagccacct atcccgacgg caaggtcagc caccacgacc
46441 ccgaccagaa agccctggtc tacgactggg acgagaaggg caacatcaag accggggata
46501 cccatgcgct gttgaagcgc gccaagcacc gcggcatcgc caagcgcaag gcgtcctccc
46561 atctgcacca ggccgacaag aaacagttgt ccggcaccct cggcctggtc ggtgacaccc
46621 ggctggtggc cgggatcaat ctcgatctga ccggcttgtt caagctcgac ggcaagtatc
46681 acgtcaccac ttcgacccac cggctggatc gcagcggcgg ctatgcctca gaagcggagg
46741 tctaccgtgt cgttgcaatc cactccctgg agagcccata gaggggcgtt actgtgatga
46801 gtgaggcgtc ccgtgtccaa tcctaagatc gcccatctgc gctatgggct ggtcaccgcc
46861 atcgacccgg cgaagtcgcg gctcaaggcg aagttccccg accaggacgg cctggtgtcg
46921 cactggctca gcgtgtcctg ccagaagagc ctgaaggaca aggcgttctg gatgcccgag
46981 gtgggggaac aggtggcctg catcatggac gagcacctgg aggacggtac cgtgatcggg
47041 gcgatctaca gcggcccgga tccggtgccg gaaggcgtcg acgaaaagtg gtacggcgtc
47101 tggttcgagg acggctccat cctgtactac cgcaaggact cccatcagct gctgatcgac
47161 ctgacgcaca tgcaagggac ggtgctgctg aaggcgcaga gcgtgaccgt cgaggcccac
47221 acagtgaacg tgcaggccgg gaccgcccag gtcgaagcgg acacggtcgc cgtgacggca
47281 tcgacgctca ccgccg

//

```
LOCUS       McNor-R1             29097 bp    DNA     linear   PHG 16-FEB-2025
DEFINITION  McNor-R1.
ACCESSION   McNor-R1
VERSION     McNor-R1
KEYWORDS    .
SOURCE      .
  ORGANISM  .
            .
FEATURES             Location/Qualifiers
     CDS             complement(35..241)
                     /ID="LFPVWAMN_CDS_0001"
                     /transl_table=11
                     /phrog="No_PHROGs_HMM"
                     /top_hit="No_MMseqs_PHROG_hit"
                     /locus_tag="LFPVWAMN_CDS_0001"
                     /function="unknown function"
                     /product="hypothetical protein"
                     /source="PHANOTATE_1.5.1"
                     /score="-6.345246725698409"
                     /phase="0"
                     /translation="MQPFSITLVATTGDPEGNSAGRQIEPVGLRRRLHQGVVPSVEAGD
                     GLLEQLGKLHPVFGHRVLLGGEV"
     CDS             complement(242..340)
                     /ID="LFPVWAMN_CDS_0002"
                     /transl_table=11
                     /phrog="No_PHROGs_HMM"
                     /top_hit="No_MMseqs_PHROG_hit"
                     /locus_tag="LFPVWAMN_CDS_0002"
                     /function="unknown function"
                     /product="hypothetical protein"
                     /source="PHANOTATE_1.5.1"
                     /score="-4.72665646396931"
                     /phase="0"
                     /translation="MAFLSEAEVESALLDQLHALGYCIEREEDRSN"
     CDS             complement(340..1002)
                     /ID="LFPVWAMN_CDS_0003"
                     /transl_table=11
                     /phrog="31613"
                     /top_hit="No_MMseqs_PHROG_hit"
                     /locus_tag="LFPVWAMN_CDS_0003"
                     /function="unknown function"
                     /product="hypothetical protein"
                     /source="PHANOTATE_1.5.1"
                     /score="-2235.318741323504"
                     /phase="0"
                     /translation="MSLGAALTADDEIAIRAEMIRDYGILVSIVVQGDILPALEVLLLE
                     HRLREIDFIALARSSPIVPKDRAGLFGKALFAGYERDFVTALHLLIPQIEHLVRVHLKQ
                     AGARTTNIDKNGIQNENGMSTLLELPEAVQVFGKDLTFELKSLFCDAFGPNLRNELAHG
                     LLDEDGCNSPFAIYAWWLALQLIFNTWWNSANPAAGQQKVNDGQIPPAEPVEEQGEP"
     CDS             complement(1124..1660)
                     /ID="LFPVWAMN_CDS_0004"
                     /transl_table=11
                     /phrog="26"
```

/top_hit="No_MMseqs_PHROG_hit"
/locus_tag="LFPVWAMN_CDS_0004"
/function="connector"
/product="tail completion or Neck1 protein"
/source="PHANOTATE_1.5.1"
/score="-232.60248335242383"
/phase="0"
/translation="MAGTFIRVDDENARKALAELVRLIGNPEPALRDIGAYLERSHDER
FAAGEAPDGSKWAPLSESYRAIKPQHRDKVLVLEGHLRNSLHYQVEDDGLLFGTDRIYG
AVHQFGAAMGEFGRYYQLSRLKYDKNDFRRHAGSKKGHPIPWGDIPARPFVGLSDADRE
EIAAILEEHIQAAIG"
CDS complement(1665..1856)
/ID="LFPVWAMN_CDS_0005"
/transl_table=11
/phrog="No_PHROGs_HMM"
/top_hit="No_MMseqs_PHROG_hit"
/locus_tag="LFPVWAMN_CDS_0005"
/function="unknown function"
/product="hypothetical protein"
/source="PHANOTATE_1.5.1"
/score="-3.677605741730489"
/phase="0"
/translation="MNKPLDQRQPDDLVVFDGPDAFADTERAQREFDDWYRDVWLNLPP
EEQDRQREEARGRLARKG"
CDS complement(1853..3178)
/ID="LFPVWAMN_CDS_0006"
/transl_table=11
/phrog="220"
/top_hit="No_MMseqs_PHROG_hit"
/locus_tag="LFPVWAMN_CDS_0006"
/function="head and packaging"
/product="minor head protein and DNA pilot"
/source="PHANOTATE_1.5.1"
/score="-93489.79845924371"
/phase="0"
/translation="MSAQYGSLPFDEAIRFFRGKDLVPTDRWADVWKEQHDVGFMVSGA
AKADLLADLHGAMLKGVAKGTTLAEFRKDFDAIVTRHGWTGWKGESSDAGRAWRTRLIY
DTNLRTAYQAGRWAQVQAGKQWRPYLEYKHSDLSAHPRPLHKSWDGLVVAVDDPWVQTH
WPPNGWGCKCRMFALSERDLRRMGKTGPDTPPDDGSYDWTDKVTGEVHRVPNGIDPGWN
YAPGASRTDLMRKEIERKVAKLPAGIGRQLKDELSLRFTQPAGEFTPYDTDYPSANPDI
STPLKAAIIRFEDAVRSDLATEHAALLDSQGAILLQRSGQSDRVTFTHAELTPMAGATF
SHNHPGGASFSLEDIALAAAYDFAEIRAVTALFRHSMAGPWPAEADLARVYAEEFPKAE
SEVRDRVKTGQLRAIHYGFEVRHRVWVRVASRLGLVYRREKS"
CDS complement(3178..4512)
/ID="LFPVWAMN_CDS_0007"
/transl_table=11
/phrog="22899"
/top_hit="No_MMseqs_PHROG_hit"
/locus_tag="LFPVWAMN_CDS_0007"
/function="unknown function"
/product="hypothetical protein"
/source="PHANOTATE_1.5.1"
/score="-99144.6041886328"

/phase="0"
/translation="MSLLSGGLLGSRRRNPGSAVASWARHPDWLPLPVIGPTEEKFVGL
FGVTNDSANFVALLAQGDYTVDWGDGTVENHDSFAVASHQYDYAAIPNGTLCSRGYKQV
IITVTPREWATFTRIDLQRRHPSMTQNYAPMWLDLALGSPNLVWMKLGGWDLSLQFVER
VKIVSVGNVTSTSDSLYNSMTALEVVNDFNTANVTDASYMYMSCDRIKNVPAHNLSSCLY
FTSFFENCSCLEVAPAINTAMGQDFSRFYYNCKALREAPALNTANGTNFNEMHRNNHAL
LDIPAYTLASAQTARYMLSACNSAKACPNFNTPNLTDAEGMFDNMHNLQFGPVLDLSQA
TNCARLFNNCYSLVHIPPYELSSSLANGFILEACTSLSKGAMTGTKVSIEYKNMRLGAA
ELNAIFAGLATGVTGQTIDITGNFGASDPACDRSIATLKGWTVVG"
CDS complement(4509..4805)
/ID="LFPVWAMN_CDS_0008"
/transl_table=11
/phrog="No_PHROGs_HMM"
/top_hit="No_MMseqs_PHROG_hit"
/locus_tag="LFPVWAMN_CDS_0008"
/function="unknown function"
/product="hypothetical protein"
/source="PHANOTATE_1.5.1"
/score="-5.1458519790261645"
/phase="0"
/translation="MNMGQQINDSGVFSGEKVVDVSGIHPPITVALYPGAGNTSRWEYS
ATPGAADAPAAAKWWPGSGDVTAPTIDTIEGACHAIRFARASGSSEDEYEVTA"
CDS complement(4802..6361)
/ID="LFPVWAMN_CDS_0009"
/transl_table=11
/phrog="113"
/top_hit="No_MMseqs_PHROG_hit"
/locus_tag="LFPVWAMN_CDS_0009"
/function="head and packaging"
/product="portal protein"
/source="PHANOTATE_1.5.1"
/score="-278117.188076876"
/phase="0"
/translation="MGFWDFIARAAAPLPPFAELAETAGPEFDEIATTHDGRDVTRGYI
HPDMILPSSDEILQAKGGDLRIYQEVLRDDQVQTSFGQRRSAVTSAEWDVEPGGTKRID
KQAAEFVKAQLEHIGWDQRTDKMLYGVFYGWAVAECLWARDGAQIVMDAVKVRDRRRFA
FDGDFRLRLLTGSNAQGELMPERKFWVFATGADHDDEPYGLGLGHWLYWPTFFKKNDIK
FWLIFLEKFGMPTGKGTYQPGATDAEKRRLLSALRAIQRDSGVIIPEGMTIELIEAARS
GTADYTALYDRMNAAISKVVLGHTGSTDATPGRLGGESNAADVRRDLVKADADLICESA
NRSFIRWLVDWNYDGAAYPKVWRDVEEPEDLKARSERDKNLFAIGFKPTLKEVKETYGG
EWEEVKPEPPPDTGSLPALPAPASDPAQADLAEGDPAPDVVDQYVERLGKEAEPLIEKG
LLDPIRTALDKAIADGQTLAEFAESLPSLFAEMDARDFAELMAQGMFAAGMAGRYELQQ
GE"
CDS complement(6363..7826)
/ID="LFPVWAMN_CDS_0010"
/transl_table=11
/phrog="5909"
/top_hit="No_MMseqs_PHROG_hit"
/locus_tag="LFPVWAMN_CDS_0010"
/function="head and packaging"
/product="terminase large subunit"
/source="PHANOTATE_1.5.1"
/score="-1347036.7402225295"

/phase="0"
/translation="MGAAKVIPPNPAGLFLPFQADWVKDRSRLKLMEKSRQIGLSWSTA
YAADERTAEVGAKWDQWVSSRDDLQARLFIEDCKRFAQLLHLAAEDLGERVIDEDKKIS
AYVLHFANGRRIHSMSSNPDAQAGKRGGRILDEFALHPDPRKLWSIAYPGITWGGSMEI
ISTHRGSHNFFNQLIREIRENGNPKKISLHRVTLQNALDQGFLYKLQQSLPEDHEVQEM
DEAAYFDFIRSGCADEESFLQEYMCEPADDDAAFLEYDLIASCEYPAGEAWEFDLDALS
VARGRLYAGLDIGRTKDLTVLWVLELLGDVLYTRLIVELRNMPKPDQEAILWPIIAIMQ
RTCIDKTGLGIGWVDDAQRKFGETRVEGVTFTASIKEALAYPVRGAMQDKRLRIPYRPE
IRADLRAVTKVTTAAGNVRFTAERSENGHADRFWALALAKHAASTPAAPIDFQSIGGRR
AVIDAGENELALTDSGFGTVSGLNDFEGY"
CDS complement(7826..8383)
/ID="LFPVWAMN_CDS_0011"
/transl_table=11
/phrog="426"
/top_hit="No_MMseqs_PHROG_hit"
/locus_tag="LFPVWAMN_CDS_0011"
/function="head and packaging"
/product="terminase small subunit"
/source="PHANOTATE_1.5.1"
/score="-134.11799029744577"
/phase="0"
/translation="MPPRPAIDLLPDAIRDELNARLVSNGFGGLEALSAWLGEQGYKIS
RSALGRHNISLKEAMDKAMDRARTRLECAKALKGMSDGDKAALLEANEMIALDKLMDLW
DDWDAHEPEAKAELLPKLVRASADLNRSAVGTAKWKRDFEARIRAEERAKAADVATKAA
KSQGVSPETIALIRRDVLGMAT"
CDS complement(8383..8673)
/ID="LFPVWAMN_CDS_0012"
/transl_table=11
/phrog="436"
/top_hit="No_MMseqs_PHROG_hit"
/locus_tag="LFPVWAMN_CDS_0012"
/function="unknown function"
/product="hypothetical protein"
/source="PHANOTATE_1.5.1"
/score="-15.599030506127672"
/phase="0"
/translation="MNRGDVRFHILKILREQPNYTTNQEVLLAALRNAGHAINRDQLKI
ELAWLSANANAVLDTACQGVHIATLTMEGLEALEGTRAIPGIRRPLPGETG"
CDS complement(8670..9068)
/ID="LFPVWAMN_CDS_0013"
/transl_table=11
/phrog="736"
/top_hit="No_MMseqs_PHROG_hit"
/locus_tag="LFPVWAMN_CDS_0013"
/function="unknown function"
/product="hypothetical protein"
/source="PHANOTATE_1.5.1"
/score="-157.93285380664665"
/phase="0"
/translation="MTVSFDFNFWTVLMFLMNFGVVAWVQISNRSKAATEELKAVQKSA
ASETKTLEEKAMHWLGQHSERISALEMAAENAIKHDDLAAVHRRVDGLMTQLSRMEGQV
GQMAGQVTQVLTNLDRLTGLMMHQGSKL"
CDS complement(9065..9376)

/ID="LFPVWAMN_CDS_0014"
/transl_table=11
/phrog="54"
/top_hit="No_MMseqs_PHROG_hit"
/locus_tag="LFPVWAMN_CDS_0014"
/function="other"
/product="DksA-like zinc-finger protein"
/source="PHANOTATE_1.5.1"
/score="-19.26044489458551"
/phase="0"
/translation="MANRRRNSGPACRVISEIRMDAADIAEERIEHELELNRRAALDMM
KPDMPQVVVAGEDGAPTIICYDCEEPIPMGRLDAYPTAIRCIDCQIEHERYLAAEARR"
CDS complement(9394..9498)
/ID="LFPVWAMN_CDS_0015"
/transl_table=11
/phrog="No_PHROGs_HMM"
/top_hit="No_MMseqs_PHROG_hit"
/locus_tag="LFPVWAMN_CDS_0015"
/function="unknown function"
/product="hypothetical protein"
/source="PHANOTATE_1.5.1"
/score="-1.1268161144050572"
/phase="0"
/translation="MCGWRGVADAGLLHRCACRLRLSGLRADRLPDVP"
CDS complement(9518..9832)
/ID="LFPVWAMN_CDS_0016"
/transl_table=11
/phrog="No_PHROGs_HMM"
/top_hit="No_MMseqs_PHROG_hit"
/locus_tag="LFPVWAMN_CDS_0016"
/function="unknown function"
/product="hypothetical protein"
/source="PHANOTATE_1.5.1"
/score="-6.121947528014704"
/phase="0"
/translation="MWKRGWTVRCVSGFPGAAILALRLTLAGCVQVGPKELVPKPILTP
AFAPESCALPDLPPLASTVFVDIAPGRPPKADAGGKALVVGYGRAREAIQACKGGAEGH
"
CDS complement(9798..10223)
/ID="LFPVWAMN_CDS_0017"
/transl_table=11
/phrog="873"
/top_hit="No_MMseqs_PHROG_hit"
/locus_tag="LFPVWAMN_CDS_0017"
/function="head and packaging"
/product="endolysin"
/source="PHANOTATE_1.5.1"
/score="-41.731176856635045"
/phase="0"
/translation="MSFQKPRGLRNNNPGNLVYSPRNAWEGQVGHDGRFARFEQMEDGV
RALGVTLLNYQRKRQLHTVRQIITRWAPPNENDTATYIRRVAAALDVGADDRIILSDRK
TLTLLVWAISTHENGAMACERWLKHEDVEAGVDRALR"
CDS complement(10220..10369)

/ID="LFPVWAMN_CDS_0018"
/transl_table=11
/phrog="No_PHROGs_HMM"
/top_hit="No_MMseqs_PHROG_hit"
/locus_tag="LFPVWAMN_CDS_0018"
/function="unknown function"
/product="hypothetical protein"
/source="PHANOTATE_1.5.1"
/score="-0.3729412240802983"
/phase="0"
/translation="VPGVSAETRSHLKVTDRYSKAATPAPLPQGRGEDMSDEEFAAFVR
RQQK"
CDS 10407..11306
/ID="LFPVWAMN_CDS_0019"
/transl_table=11
/phrog="290"
/top_hit="No_MMseqs_PHROG_hit"
/locus_tag="LFPVWAMN_CDS_0019"
/function="tail"
/product="minor tail protein"
/source="PHANOTATE_1.5.1"
/score="-183.44154781438692"
/phase="0"
/translation="MAETASAAAVTGTAQSGSTTTTLKLAAAASSIDGVYVGARVRITG
GAGSGQSATIIGYVGSTKVATVDRPWTVTPDATSLYSVDPFVRYNPVSDILTMESATFY
YNEGNQVRHKLLGCRGNVKIDASAKDTGMLDFELIGLYGGVADAAETGVAVSNWVDPWE
VGYGRTYGQVFGKPLTGGASGLQMGKFSLDLGQKAAYRSVVGYQGVIITDRAASGSLTL
DATTVAQFDPWTLIAGNGTGAIGIEQPDSNNGSVRIDVPKAAPTEIDYGDDQGISTNTI
QFDCQRALGNDEVFITCR"
CDS 11319..11729
/ID="LFPVWAMN_CDS_0020"
/transl_table=11
/phrog="569"
/top_hit="No_MMseqs_PHROG_hit"
/locus_tag="LFPVWAMN_CDS_0020"
/function="tail"
/product="tail length tape measure protein"
/source="PHANOTATE_1.5.1"
/score="-19.495578067452108"
/phase="0"
/translation="MAFKLGTQEVLGKPIARKVMLPVCNEAGTPVNHQITLLYNRMTVE
EIGAAYAALAEDARMLEALTGPELTAAVLDMHATHVLRLASGWREVQAEDGAEAAFTFD
NVRALLNSVPKAYDRITEEFQKANRGGAALGN"
CDS 11807..12106
/ID="LFPVWAMN_CDS_0021"
/transl_table=11
/phrog="680"
/top_hit="No_MMseqs_PHROG_hit"
/locus_tag="LFPVWAMN_CDS_0021"
/function="tail"
/product="tail length tape measure protein"
/source="PHANOTATE_1.5.1"
/score="-6.116817989881506"

/phase="0"
/translation="MAAPDGDGEPGDGDFPVMAENWDTVMAFLACRTAWRLYFPPLGGA
PVWLGLSYPDVDVVIRHLGHEGEAARRIFAGIQVMEAAALAVFHPPAAESAAEE"
CDS 12162..12254
/ID="LFPVWAMN_CDS_0022"
/transl_table=11
/phrog="No_PHROGs_HMM"
/top_hit="No_MMseqs_PHROG_hit"
/locus_tag="LFPVWAMN_CDS_0022"
/function="unknown function"
/product="hypothetical protein"
/source="PHANOTATE_1.5.1"
/score="-1.5413066435527039"
/phase="0"
/translation="MAAPYLRNPSANSRKPSTIRLIKDIWPALS"
CDS 12236..18946
/ID="LFPVWAMN_CDS_0023"
/transl_table=11
/phrog="339"
/top_hit="No_MMseqs_PHROG_hit"
/locus_tag="LFPVWAMN_CDS_0023"
/function="tail"
/product="tail length tape measure protein"
/source="PHANOTATE_1.5.1"
/score="-4.653279167529952e+23"
/phase="0"
/translation="MAGPIVVGIKLNLDEKGALVGVEKVESGFKRIGSAASESASRIDA
SYKKVETGIAEVENGFKRVGSAASESAGRIDAAYGKTRQGLDSISTQLATAKRQFLEFI
TAQAALGAGRELADVADRYANLSARLKLATQSELEYAKAQAQVFAISQRYSTALDDNAK
LYSRIAPAVRDAGKSQADLVKVIEAVNASLKTSGASAAEQSSTITQLSQALASSTVQWE
DFGQLADTNMRLVDAVAKRLAGNMGELKQKMSDGLISNTQLFDAIIAASEQLKAEAATM
PNTIGGAVQQIENAWERYIGKVDQAAGASRDVANAISGVATHFDQIADAAVRVGEVVAV
AIGVKAVAAMTAYVAEAAAAANGALLFSRAMGALGGPVGAATFAVLAGAEALSVWRANM
IAANDEALKIRSQGNAGALNTTDPTKVMGADLKAKVDQAKQSVGELTVTVNASSAALAA
AAETTKKYTATEREDIQSKIRWLEAHGQQADALRLEGALHGFTGKALEAYVKQELDTIA
ARKANAEATKAHNKALSEEASAVKKSATEHQNALEASDRFIQSLKDELAMLQDRTGASE
AEIAARKASARLTGEEAEARARLARQIKEEIDALEGAERIRQLEIKTQNDIAEAIQRKE
EAAARALQSAQDEEQKQLALLAQIQAAHEAGYGPEALARMRAYLSAVYDVTRGMGEEEA
AATAAAMAHTEAATHMMDEMTRTGQSMLDDLGGVARSMMSGFSSLFFEPFGAATESMGA
RFSRVLQQMAAQLMQSAVTRLFTGGLGLVAGGEAGAGGWLGGLLGLGGSVVGADGLITR
MPQVGEAMVLPSGSMIINSGGNAVSSFPQYVNYAGTGASVAGYGNGLLGGPGWATAALG
YAGAGLGGAGLGYLIGGLIGDRYNYGQAGGAIGGLAGGIGGYAAATSMGLSGAMAGLAA
TGVGAIAAAVIAVIASQVKVGKPDAQLIANVTPGEGYDITALNSRHGGKTGLFGLLEGL
ANITFDPADPFHFSRDNSPNKGAWAPGEKFLGDALTALFGPLGKLEESLGVMIPAFQFI
GRIHDDRTGIQIPGIYASGPFKFDDNAAQYMQQAVSIATMRLLQQPAVLNQMTDPVTKA
VVMLSGDLQAFQANLEKVSEVRKYAGGSFEEVGGRKALGKALDLSGGDIDQYIQNMKEL
GALRTFLGEWTPDVGAWTQTVDGFDAMEKAAIKFGVSVKKVSEQIDKTIQAQQFGIQDQ
VNQLLGKPQSTRSKLFAANQTFDQLGAKESGTLAELAQYNKEERIRGKNALAQLDVKTV
SAIDRQSVKSMLDRLKDPKLASDPAMLAEWQAGWDRFWENELTQAESIKNPKKRKEAIA
KIKALQQQTDPHATEQIDIEAARAQYVEQQKKAILDQAQAYAGQGASQYAAKIKEINDF
FADANEAASELGISTSELAEYQKKATAALRDDFSNALKQFAGETVSGAAAALKSIDDQE
KTFEKDAAALGISLDKVHDAAEKARQKLKDDFVKSLDSMAGVLDANTQAFNSLSDQFKT
LYEDAQTLGVGMDKVGAALVQSMKNLWEQMTAGIVSARQAIAQQIAELQGPQAVATLAG

QNVDTAKGKLADYYASGGNDVNKEISLIQNVQSAVMARYNAELALIQETIQKEVEAIQA
KAQAEVDAVNETLNVQIEAENERLSAAIEAENERLNAVLEAESERLNAQIEAINAASQA
QVDAIQEQADAAAEARQEAQQAELDALSEQLQVANQLKSAIKNIADYAKSLLTAADSPL
SPEARLAAAKQQYQDTLAKAQAGDAEAAQRYQQDAEAYKNAAKDYYASSAGYGDVYNQI
QSDATALGNTTVTSPDSIESQMKELRKQQAAENKEAQKLVQEQIKAVQQGAQDQIKAAQ
EASQDYIKSLQDASKDTIAQMQKDSQATVEGMRDSANAQIKAIQDAAAQATKDAQDLTK
REDVAALKKDTIDNLTALDGHLQAAYDAAQKQYQTFIDALAGVVGSNTTAISALVDWLN
KQGVAVTQPQTKNTIIQVNADGSQTALQEGNSGTTEYLPPPHYAAGGVARPGWALVGEL
GPELVNFDRPGRVYTAQDTAQMMAGLNAANDSGAYADFVARQSESVARIQEWIQSWIAN
ARAANDAHGAYANVLDMPPTAQNAWNAVQSLMLKPAQTQELPDYIPAVVERQTARLEER
LARIESGQGGQQSEAVREEIRGLRDDLRALIVTQSGTNPRMLEKFDALIARFEDMVSET
KLARGRAA"

CDS 18943..20445
/ID="LFPVWAMN_CDS_0024"
/transl_table=11
/phrog="8917"
/top_hit="No_MMseqs_PHROG_hit"
/locus_tag="LFPVWAMN_CDS_0024"
/function="unknown function"
/product="hypothetical protein"
/source="PHANOTATE_1.5.1"
/score="-286081.09863004903"
/phase="0"
/translation="MILLQEFDYYDGSEKTLYLTYGAGYDNDAGIFYRPAIIQPANYSR
DASLLQFGGRTSSSYGETTIANGDGYFDYLNGAYFEGREMRMYTLEDGQPYSSRVLVMR
AIIDAVDWKMSEISFRLTDNSKSLDKPLQPNKYGGTNALPNGIDGTPDDIKGQSKPRIY
GRISLMGGVEVNTSKLIFQVNDRAVAQIINVFDKGAYLTRDGSTYANQTAMETTAPASG
AFKACPSIGCFRTGSTPFGAVSICVAESFVHTEISAAGVIKRVLDDMAISASAYSLQDF
ADLDGKNCGPVGVIVQDQETAWSVIERIAASVGAWVGFDALNVFRVARLDAPTGSYSVV
IDDTVIDSITKPVPAVAPAWKMTLEGDTNYLVQDKNALAGIVPTARAAWFEAATRSASA
SDTSVKTIRINSQDYTASSVQVSVSQLQAEATRRLNLLKNRLDVVTVTLRDSPTSWLGQ
VDIGTEVLLQTRWYGYDAGRPMIVTGLRTNFLRNQLDLTLMG"

CDS 20449..21243
/ID="LFPVWAMN_CDS_0025"
/transl_table=11
/phrog="8610"
/top_hit="No_MMseqs_PHROG_hit"
/locus_tag="LFPVWAMN_CDS_0025"
/function="unknown function"
/product="hypothetical protein"
/source="PHANOTATE_1.5.1"
/score="-470.70566360222256"
/phase="0"
/translation="MAFFLLGHTNRIDAATLSGGTWNSLYPLTNLQDAALARVARSVGT
TSGASTIVIDCGAAKDIRTLGLVNHNIRSTGTVRLEGSNAVGFSPLVADSGAVTVYADT
VAANPTFTLDLGAVYLARYWRITISDSGNPAGYIRLGRVFLGDGWQPTETNFSWGKILG
IENPSVVSVLPSGRRVVDVRPPSRNQKYQIKDLTKSDALREVFQIAWTHGLDKEVLLFE
DPADNTYADVNNFLATIRQLPAIEYPYLDAYSAAFEFSEIIA"

CDS 21256..21774
/ID="LFPVWAMN_CDS_0026"
/transl_table=11
/phrog="No_PHROGs_HMM"
/top_hit="No_MMseqs_PHROG_hit"

/locus_tag="LFPVWAMN_CDS_0026"
/function="unknown function"
/product="hypothetical protein"
/source="PHANOTATE_1.5.1"
/score="-85.89506665349802"
/phase="0"
/translation="MSYTIQDEVNVDFAAEIRGGCIVFESEAETAKRSLLASDGSVIAE
AIAHNGVKVLDKPAARLWFESTGGGEFTRLALESALAETLAGADELLAAAQTPAHVEAV
RGMLKSGGWTCYRVAGNDGATTALLLIAIGAQPNEDLEYELPGNPVEIDADDWANAWAD
FRDRVYILQ"
CDS 21784..22473
/ID="LFPVWAMN_CDS_0027"
/transl_table=11
/phrog="8123"
/top_hit="No_MMseqs_PHROG_hit"
/locus_tag="LFPVWAMN_CDS_0027"
/function="unknown function"
/product="hypothetical protein"
/source="PHANOTATE_1.5.1"
/score="-65.68026665064218"
/phase="0"
/translation="MNRKTSKSLNRELAQAKAMSLAKAVLAASIIEAATAHGAFACWAV
GPVEHMRAEYVEMLAEARDLEEGGAIQLAANLRAQAERMLEEKWHDAAANVVCTGGKND
ALDKYLAGSAYTAAWYLGLISSTSYTGVAAGDTMASHAGWTESTAYSQATRVAPSFSAA
SSGSKATSAAASFSINAAATIKGPFLTSNNTKSGTTGILYSAGLFSSGDRTLANGDTLN
VTYTAGL"
CDS 22486..24258
/ID="LFPVWAMN_CDS_0028"
/transl_table=11
/phrog="No_PHROGs_HMM"
/top_hit="No_MMseqs_PHROG_hit"
/locus_tag="LFPVWAMN_CDS_0028"
/function="unknown function"
/product="hypothetical protein"
/source="PHANOTATE_1.5.1"
/score="-256.8022579452835"
/phase="0"
/translation="VATAGPNYASTAVSDSTLAGNAWSNPSNGTASDNSYATASSNGTQ
YLKATGFGFSIPTGATIDGIVVEWERKASSSTVKDKAVRIVKGGVVGATDKSAAGDWPT
TDTFASYGSSSDLWGETWTAADINAANFGAAIATQSTFVRTASVDAVRITVYYTDVSAI
TESATATESSDAAATVSDGVTETATASESGDATSALVADITESATATESSDSALGPKQG
DITESATAAESSDAVAFSADSVTESASATDTQDASATANASIIETASASESSSASVIVY
ASASESATATESSDGIAVGQLYREDATGFLVVKSGAGAGYTALAAPTTAAAWRVLDIYG
STSAWHPSDDPPAQESAAPTVDYSNVTGTKPPADATRNQIYIQSGTPSSPVAGDLWFDT
ANKTWATWTGSLWQVVSDVTAYKTAASIAGQGAFATLNLLTSANIDSYLSDAALGTLKL
ALNALSVLQGASGLVSTTYNTWVQTSTWNIPAVASASLRGKFLLLLWRQWNDLGTDYTG
ASAIKRDGATLVDVYDRITTNASGIGKSQTIAWIDSSPPATAAFSYSVHVFQNYNGSAP
NINSNFAILELKRS"
CDS 24255..24659
/ID="LFPVWAMN_CDS_0029"
/transl_table=11
/phrog="18216"
/top_hit="No_MMseqs_PHROG_hit"

/locus_tag="LFPVWAMN_CDS_0029"
/function="unknown function"
/product="hypothetical protein"
/source="PHANOTATE_1.5.1"
/score="-24.715355495242537"
/phase="0"
/translation="MNAIWTVYQPSTGRILELPKSIPDAYAHLYLGPDRHVIEGAWYPD
RHYIGPVGALERPPQETSLDTITIRADGEDVATLSRLPDPCTVEIYGPVRDVIEVEGGV
LEFCADVPGRYELVVKAFPWLDFTGMIHAL"
CDS 24649..25011
/ID="LFPVWAMN_CDS_0030"
/transl_table=11
/phrog="2837"
/top_hit="No_MMseqs_PHROG_hit"
/locus_tag="LFPVWAMN_CDS_0030"
/function="tail"
/product="tail protein"
/source="PHANOTATE_1.5.1"
/score="-23.65314923555232"
/phase="0"
/translation="MRYKATLPPEDFKRLMRARVNAEREARLRAGFEFQGHTYDTDPQS
VANMTAVLAALGAGLPLPEGFAWRDRANIDVPMDEATFKAFTGSLLVHVNSIYVESWRK
KDEIKTSGNPASIDAD"
CDS 25038..25145
/ID="LFPVWAMN_CDS_0031"
/transl_table=11
/phrog="No_PHROGs_HMM"
/top_hit="No_MMseqs_PHROG_hit"
/locus_tag="LFPVWAMN_CDS_0031"
/function="unknown function"
/product="hypothetical protein"
/source="PHANOTATE_1.5.1"
/score="-0.1295466816425838"
/phase="0"
/translation="VLRTPGQPADPQRIPASQPKAATEWPLGVASLVDF"
CDS 25171..25722
/ID="LFPVWAMN_CDS_0032"
/transl_table=11
/phrog="56"
/top_hit="No_MMseqs_PHROG_hit"
/locus_tag="LFPVWAMN_CDS_0032"
/function="other"
/product="DNA methyltransferase"
/source="PHANOTATE_1.5.1"
/score="-36.16938939994192"
/phase="0"
/translation="MQCKPIVPWIGGKRRLARHILPLFPPHDCYVEPFCGAAALYFLKE
PAKCEVINDINGELVNLYRVVQHHLEEFVRQFKWALTSRQIYEWMKATPEQTLTDIQRG
ARFFYLQKLAFGGKVESMTFGTSTTSGPPAQPAAARGGAVGGAPAAVTNHHRTSAVGGM
HPPLRPAAHAVLSRPALLGH"
CDS 25763..25942
/ID="LFPVWAMN_CDS_0033"
/transl_table=11

/phrog="56"
/top_hit="No_MMseqs_PHROG_hit"
/locus_tag="LFPVWAMN_CDS_0033"
/function="other"
/product="DNA methyltransferase"
/source="PHANOTATE_1.5.1"
/score="-4.630924578422289"
/phase="0"
/translation="MAELARTIEGRMVISVNDIPEMRAAFEGLPTKHADLCYSVGGGKR
SRRSSDELIIRSWA"
CDS complement(26011..27195)
/ID="LFPVWAMN_CDS_0034"
/transl_table=11
/phrog="31613"
/top_hit="No_MMseqs_PHROG_hit"
/locus_tag="LFPVWAMN_CDS_0034"
/function="unknown function"
/product="hypothetical protein"
/source="PHANOTATE_1.5.1"
/score="-65895.17918118968"
/phase="0"
/translation="MWLALSSAARTAIEQGRVAHGKVLWLLADACSMMLVPSSTNEPFK
PFAVFHDRRSVIPDDLLDTDIAFFAEIVDAVDDNWLKARLSDLLWLKGEPRNTAFALKA
IDAYRRLPLDADTWVHGGCECWSRAISLARMLKTAAGDRLQQMEASIVTAFNAAKRDDG
FLGLWLADLLKSNGLGRDHRVGVARKLEALAREFDGAGDLHRAREYFSSAAEWYRTIPD
AAKAAEMTVAVAEGWVKEAVAQTASESPSHMVAASIFENVIQTYRTVPRAERSTHQVDA
RIAELRDHLNDSGERALGEMVLIQTPGVDITQLIESARKSVTGKSAQLALLAFANLHRG
ANTEELRKNAIERMRRYPLQSLFAAMGMSRDGRVIAKCPPMMKPRAINEHEIVR"
CDS complement(27300..28115)
/ID="LFPVWAMN_CDS_0035"
/transl_table=11
/phrog="2668"
/top_hit="No_MMseqs_PHROG_hit"
/locus_tag="LFPVWAMN_CDS_0035"
/function="unknown function"
/product="hypothetical protein"
/source="PHANOTATE_1.5.1"
/score="-584.5167591357603"
/phase="0"
/translation="VLEAIFQAIVEQRSPSSPSPPAPLPEGEGELWGTLDADGVWHWPQ
HILDLFPDRLVESELGEIPEGWRHSTIGEEVTVCVGSTPSTKEPEFWEGGQHCWATPKD
LSALKFPVLLDTDRKITDAGLAKISSGLLPVGTVLLSSRAPIGYLAIAEVPTAINQGFI
AMKCDGTLPNVFVLLWCKENMDAIVGNANGLTFLEISKSNFRPLRIVVLSDQVLTNFTK
MVGPLYRQLVENERESRTLAHLRDTLLPKLIFGKLRVKDAEDFVTERGL"
CDS complement(28178..29095)
/ID="LFPVWAMN_CDS_0036"
/transl_table=11
/phrog="2668"
/top_hit="No_MMseqs_PHROG_hit"
/locus_tag="LFPVWAMN_CDS_0036"
/function="unknown function"
/product="hypothetical protein"
/source="PHANOTATE_1.5.1"

/score="-3171.0617087389237"
/phase="0"
/translation="TKCRIEEVAERVAMGPFGSSIKVETFVPEGVPVISGQHLHGFRLD
DAPGFNFITREHAERLRNANVQRGDVVFTHAGNIGQVAYIPSTSAFDRYVISQRQFYLR
PDPTKVLPEFLVAYFRSPEGQHKLLANTSQVGVPSIAQPVTYLRSLEIPLPPLPEQRAI
AHILSTLDDKIELNRRQNETLEAMARALFRAWFVDFEPVRAKMNLPSPFGRGAGGEGKK
PKLPADILDFARELRHRMTDAEALLWRLLRNRQLAGVKFRRQHPFPPYVLDFYCHDHKL
VVEIDGGQHNEEAGQRHDARRDAF"

ORIGIN

1 atcgaagact ccggtgaggc aggagttccg accctcaaac ctcaccaccg aggagtactc
61 gatgaccgaa gacaggatgc agctttccga gctgctcgag aaggccgtct cctgcttcaa
121 ccgatggaac aacccccttgg tgaagacgac gccggagccc gaccggttcg atttggcgac
181 ctgccgaatt cccttcgggg tcaccggtgg tggcaaccag tgtgatggaa aacggctgca
241 tttagttgct cctgtcctcc tcgcgttcga tgcagtaccc gagcgcgtgc agctgatcca
301 agagggcgct ttcgacttcg gcctccgaca gaaacgccat tacggttctc cttgttcttc
361 gacgggctca gcaggaggga tttgcccgtc attgactttc tgctgtccag cggcagggtt
421 cgcggagttc caccatgtat tgaaaatgag ctgcaatgcc aaccaccacg catagatcgc
481 gaagggcgag ttacatccat cctcgtcaag cagtccatgg gccaactcgt tgcgcaaatt
541 ggggccgaag gcatcgcaga acagcgattt gagttcgaaa gtcagatcct tgccaaacac
601 ctgcacggcc tccggcaatt cgagcaaggt gctcatcccg ttttcattct ggatgccgtt
661 cttgtcaatg ttcgtagtcc tggcacccgc ctgcttgaga tgcacccgca ccaaatgctc
721 gatctgtgga atcagtaaat gcagtgcggt aacgaaatca cgttcataac cggcaaacag
781 cgctttgccg aaaaggcccg cccgatcttt gggtacgatg ggggaacttc ttgccaatgc
841 aatgaaatcg atttcccgta acctatgctc aagcaggagc acttccaagg ctggcaaaat
901 atcaccttgc accacaatgc taaccaggat gccgtagtca cggatcatct cagcgcggat
961 cgcaatttca tcgtctgccg tcagcgctgc gcccaagctc atcgtgagtg agagtgagat
1021 aaatgaacgg gaatgtgtga gataaacggg aagaccaggg atgaagaagg aagaaactgg
1081 acgtcaaaac gaaaaaacgt ttcacacgac gcaacaacac aaatcagccg atcgcggcct
1141 gaatgtgctc ctcaagaatc gcggcgatct cttcccggtc ggcatcggac aagccgacaa
1201 acggcctcgc tgggatatcg ccccagggaa tcggatgtcc tttcttcgag ccggcatggc
1261 gcctgaagtc gttcttgtcg tacttcaaac gcgacagctg gtagtacctt ccgaactcgc
1321 ccatcgccgc accgaactga tgaacagccc cgtagattct gtcggtaccg aacagcaggc
1381 cgtcgtcctc gacctggtaa tgaagggaat tgcgcagatg cccctcgagc accagcacct
1441 tgtcccggtg ttgcggcttg atagcgcggt aggactctga aagcggcgcc cacttcgagc
1501 catccggcgc ttcccccgcg gcgaagcgtt catcgtggga ccgctccaga taagcgccga
1561 tgtcacggag tgcgggttcc ggattgccga tcaggcgcac cagttccgcc agggccttcc
1621 tggcgttctc gtcatcgact cgaatgaacg tgccggccat gcgttcaccc cttccgcgcg
1681 agccggcccc gagcctcttc ccgctgccga tcttgctctt cagggggaag gtttagccag
1741 acgtcccgat accaatcatc gaactcccgc tgcgcgcgct cggtatcggc aaaagcgtca
1801 gggccgtcga atacgacaag atcatcgggt tggcgctgat cgagcggctt attcatgatt
1861 tctctctacg gtaaaccagg ccaagacggg atgcgacgcg cacccaaaca cggtgccgca
1921 cctcgaatcc gtaatgtatg gcgcgcagtt gtccggtttt tacccggtcc cgaacttcgc
1981 tttcggcctt cggaaactct tcggcgtaga cgcgcgccaa atccgcttcg gccggccatg
2041 gcccggccat tgagtgtctg aatagcgcag tcacggcccg gatttcggca aaatcatacg
2101 ccgcggcgag cgcgatgtct tcgagactga acgacgcgcc gccgggatga ttatggctga
2161 acgtcgcgcc agccatgggt gtcagctcgg catgcgtgaa agtgacccga tccgattgcc
2221 cgcttcgctg caggaggatg gcgccctgac tatccagcag ggcggcatgt tctgttgcca
2281 gatcgctccg gacagcgtct tcgaatcgga tgatggccgc cttcaacggc gtcgaaatat
2341 ccggattcgc tgacggatag tctgtatcgt aaggcgtaaa ttcccccgcc ggctgggtga
2401 atcgcaagga aagctcatct ttcaactgtc gcccaattcc cgccggcagc ttcgccacct
2461 tccgctcgat ctccttgcgc atcagatccg tgcgcgaggc gcccggcgcg tagttccagc
2521 caggatcgat gccgttcggc acccggtgca cttcgccggt caccttgtcc gtccagtcgt
2581 agctgccatc gtccggcggg gtatccggtc ctgtcttgcc catccgccgc agatcccgct

2641 cggacagcgc gaacatccgg cacttgcagc cccagccgtt cggtggccaa tgggtttgca
2701 cccagggatc gtcgaccgcg accaccagcc cgtcccagga tttgtgcagc ggacgcggat
2761 gggcggacag atcggagtgc ttgtattcca ggtacggtcg ccattgcttg cccgcctgca
2821 cctgtgccca gcgaccagct tggtaggcgg tgcgcagatt ggtgtcgtag atcagccggg
2881 tgcgccaagc cctgccggca tcggatgatt ccccсttcca gcccgtccag ccatggcggg
2941 tcacgatcgc atcgaaatcc ttccggaatt ccgccagcgt cgtacccttg gccacccccct
3001 tgagcatagc cccatgcaga tcggccagca ggtccgcctt ggcagcgccg gacaccatga
3061 agccgacatc gtgctgttcc ttccagacgt ccgcccagcg gtcggtcggg accaggtcct
3121 tcccgcggaa gaaccggatc gcctcgtcga atggcaggga gccgtattgg gcggacatca
3181 gccgacgacg gtccagccct tgagcgttgc gatcgagcgg tcacaggcag gatcggacgc
3241 cccgaagttc cctgtgatgt cgatcgtctg cccggtcacc ccggtcgcca aaccggcgaa
3301 gatggcgttc aactcggccg ctccgagccg catgttcttg tattcgatac tgaccttggt
3361 gccggtcatc gcccccctttg agagtgatgt acaggcttcg aggatgaaac cgttcgccaa
3421 gctgctggac agttcatatg gagggatgtg aacgagcgag tagcaattgt tgaatagccg
3481 cgcgcaattg gttgcctgac tcagatcgag caccggtcca aactgcaggt tgtgcatgtt
3541 gtcgaacatg ccttcagcgt ccgtgaggtt tggcgtattg aaattcgggc aggcttttgc
3601 ggaattgcac gccgacaaca tatatcgagc cgtctgcgcg ctcgccagag tgtacgcggg
3661 aatatcgagg agagcgtggt tgttgcggtg catctcgttg aaattcgtgc cgttcgcggt
3721 gttgagcgcc ggagcttcac ggagcgcctt gcagttgtag tagaaccggg agaagtcctg
3781 ccccatcgcc gtattgatcg ccggggctac ttcaagacac gaacagttct cgaagaacga
3841 ggtgaagtac aggcagctcg acaggttatg ggctggaacg tttttgatcc ggtcgcagga
3901 catatacatg tagctggcat ccgtgacgtt cgccgtgttg aagtcgttca ccacctccag
3961 cgcggtcatg gagttgtaga gcgaatccgt gctggtgacg ttaccgaccg acacgatttt
4021 gacgcgctcc acgaactgaa gactcaagtc ccagccgccc aacttcatcc agaccaggtt
4081 gggagaacca agcgctagat ccagccacat gggggcatag ttctgcgtca tcgacggatg
4141 ccggcgctga aggtcaatgc gagtgaacgt cgcccactcc ctgggcgtaa cggtgatgat
4201 cacctgcttg tagccacggc tacacagcgt gccgttcggt atagccgcat agtcgtattg
4261 atgggatgcg accgcgaacg aatcgtggtt ctcgaccgtg ccatcccccc agtccaccgt
4321 gtaatccccc tgggcgagca gggcaacgaa gttggcggag tcattggtaa ctccgaatag
4381 tccgacgaac ttttcttctg tggggccgat caccggcaac ggcagccagt ccggatgcct
4441 ggcccacgac gcgacggccg atccgggatt tctgcgccgg ctccccaaca gcccccсgct
4501 gaggaggctc atgcggttac ctcgtattca tcctccgaac tgccggacgc gcgggcaaac
4561 cggatcgcgt ggcacgcacc ttcgatggtg tcgattgtcg gggcggttac atctccggag
4621 ccgggccacc acttcgccgc tgctggagcg tcggcggcac ccggtgttgc ggagtattcc
4681 cagcgcgatg tgttgccagc gcccggatag agcgcgactg tgatcggtgg atggattccg
4741 gaaacatcga cgaccttctc cccggaaaag acaccgctgt cgttgatctg ttgtcccatg
4801 ttcattcccc ttgctgaagt tcgtaccgtc ccgccatgcc agccgcgaac atcccctgcg
4861 ccatcagctc ggcgaaatcg cgggcgtcca tctcggcgaa gagagacggc agcgattccg
4921 cgaattccgc cagcgtctgg ccgtcggcga tggccttgtc gagcgccgtg cggatcggat
4981 cgagcagccc tttttcgatc agcggttcgg cctctttgcc caggcgctcg acgtattggt
5041 cgaccacgtc gggggcggga tcgccctcgg ccaaatccgc ctgagccggg tccgacgctg
5101 gcgcaggcag cgccggcaag ctgccggtgt ccggcggcgg ctcaggcttc acttcctccc
5161 actccccgcc ataggtttcc ttgacctctt tcagggtcgg tttgaaacca atggcgaaca
5221 ggttcttgtc gcgctcggag cgggccttga ggtcttccgg ctcctcgacg tcgcgccaga
5281 ccttcggata ggccgcgccg tcatagttcc agtcgaccag ccaccggatg aaggaccggt
5341 tggccgactc acagatcaga tcggcatcgg ctttgaccag atcgcggcgg acgtcggcgg
5401 cattgctctc accgcccagc cggccgggcg tggcgtcggt gctgccggtg tggcccaaca
5461 ccactttgct gattgcggcg ttcatgcggt cgtacagcgc ggtgtagtcg gccgtgccgg
5521 agcgagcggc ctcgatcagc tcgatggtca tgccttccgg gatgatgacg cccgagtcgc
5581 gctggatcgc ccgcagcgcg gacagcagcc tgcgtttttc cgcgtcggtg gcgccgggct
5641 gataggtccc tttgcccgtg ggcatcccga acttctccag gaagatcagc cagaacttga
5701 tgtcgttctt cttgaagaag gtcggccaat acagccagtg tcccagcccc aggccgtagg
5761 gttcgtcgtc gtggtcggcg ccggtggcaa atacccagaa cttgcgctcg ggcatcagct

5821 cgccctgggc gttgctgccg gtcagcaacc gcaggcggaa atccccgtcg aaggcaaacc
5881 ggcgccggtc gcgcaccttc accgcatcca tcacgatctg ggcaccgtcg cgcgcccaca
5941 ggcactcggc caccgcccag ccatagaaca ccccgtacag catcttgtcg gtgcgctggt
6001 cccagccgat gtgctcgagc tgcgccttga cgaactcggc ggcctgcttg tcgatccgct
6061 tggtgccgcc gggttccacg tcccattcgg cggaggtcac ggcactgcga cgctggccga
6121 acgaggtctg cacctggtcg tcccgcagga cttcctggta gatcctcaag tctccgccct
6181 tggcctgcag gatctcgtcc gaggacggca ggatcatgtc ggggtggatg tatccgcggg
6241 tgacatcgcg gccgtcgtgg gtggtcgcga tttcgtcgaa ttccgggcca gcggtctccg
6301 ccagttcggc gaagggcggc aagggtgcgg cggctcgagc gatgaaatcc cagaagccca
6361 tctcaatacc cctcgaagtc gttcagcccg gacacggtac cgaagccgct gtcggtgagg
6421 gccagctcgt tttcaccggc gtcgatcaca gcacgacggc cgccgatgga ttggaaatcg
6481 atcggtgccg caggcgtcga cgcggcatgc ttcgccagcg ccagcgccca gaaccggtcg
6541 gcatggccgt tctccgatcg ctcggcggta aaccggacgt tgccggctgc agtggtcact
6601 ttggtcacgg cccggaggtc cgcgcgaatt tccggccggt acgggatgcg caggcgctta
6661 tcctgcattg cgccacgcac cggatacgcc agcgcctctt tgatactggc agtaaacgtc
6721 acgccctcca cccgtgtttc tccgaatttt cgttgcgcgt cgtccaccca gccgataccg
6781 agcccggttt tatcgatgca ggtgcgctgc atgatcgcga tgatcggcca caggatggct
6841 tcctggtcgg gcttcggcat attgcgcaat tcgacgatca ggcgggtata gagcacgtcg
6901 ccgagcagct ccaacaccca taacaccgtc aggtctttgg tgcggccgat atcgagaccg
6961 gcatacagcc ggccgcgggc gacggataac gcgtcgaggt cgaattccca ggcttcgccg
7021 gcggggtatt cgcagctcgc gatcaggtca tattcaagga aagccgcatc gtcgtcggcc
7081 ggctcgcaca tgtactcctg cagaaacgac tcctcgtcgg cgcagccgga gcggatgaaa
7141 tcgaaatacg ccgcctcgtc catctcctgg acttcatggt cctcaggcag cgactgctga
7201 agcttgtaga ggaagccctg gtcgagtgcg ttttgcagcg tgacgcgatg caggctgatc
7261 tttttcggat tgccgttttc ccgaatttcc cggatcagct ggttgaaaaa gttatggctg
7321 ccgcggtggg tggaaatgat ctccatcgaa ccgccccagg tgatgccggg gtacgcgatc
7381 gaccagagct tgcgcgggtc gggatgcagg gcgaattcgt ccagaatgcg gccgccccgc
7441 ttgccggcct gggcatccgg attactggac atggagtgga tgcgccggcc gttggcgaaa
7501 tgcagcacat aggccgaaat cttcttgtcc tcgtcgatga cccgctcgcc gagatcctcc
7561 gccgccaggt gcaggagctg ggcaaaccgc ttgcagtctt cgataaacag ccgcgcttgc
7621 agatcgtcgc ggctggagac ccactggtcc cacttggcgc ccacttcggc cgtgcgctcg
7681 tcggcggcat acgccgtgct ccaggacaag ccgatctgcc tcgacttctc catcagcttc
7741 agacggctgc ggtccttcac ccaatcggcc tggaacggca gaaacagccc ggcgggattc
7801 ggcggaatga ctttggcggc gcccattacg tggccatccc cagcacatcc cgccggatca
7861 gggcgatggt ttcgggcgat acgccttgag acttcgctgc tttcgtggcc acgtcggccg
7921 ctttggctcg ttcctccgcg cgaattcgtg cctcgaaatc acgtttccat ttcgcggtac
7981 ccaccgccga gcggttcagg tcggctgaag cgcgcaccag ctttggcaat agctcggcct
8041 tagcttccgg ctcatgagca tcccagtcgt cccatagatc catgagctta tccagcgcga
8101 tcatctcgtt cgcttccagc agcgccgcct tatcgccgtc cgacattcct ttcagcgctt
8161 ttgcacattc caatcgagtg cgtgcacgat ccatcgcttt gtccatcgcc tctttgagac
8221 tgatgttatg ccgacctagc gccgatctcg atattttgta cccctgttcg cccagccacg
8281 cacttagcgc ttccagaccg ccgaaaccat tgctcaccag tcgcgcgttc agctcgtcgc
8341 gtatagcgtc gggcagtaga tcgattgctg ggcgtggcgg catcagcccg tctcgcccgg
8401 caacggccgc cgaatgcccg gaatcgcccg cgtgccttcc agcgcttcca gcccttccat
8461 cgtcaacgtg gcgatatgca cgccctggca cgcggtatcg agtaccgcat tggcattggc
8521 gctaagccag gccagttcga tcttgagctg gtcgcggtta atcgcgtggc cagcgttacg
8581 caaggcggcg agcagcacct cttgattcgt ggtgtagttg ggctgctccc gtagaatctt
8641 caggatatgg aaacgcacgt ccccgcgatt cataacttgc tcccttggtg catcatcaat
8701 cctgtgagcc ggtccaaatt ggtcagcact tgagtgacct ggccagccat ttgcccaacc
8761 tgaccctcca tgcgactgag ctgcgtcatc aggccatcca cgcgccggtg tactgctgcc
8821 aggtcgtcat gtttgatagc gttctcggcc gccatttcca gcgcagaaat gcgctcggag
8881 tgctgtccca accaatgcat ggctttttcc tccagcgttt tggtttcgct ggccgcactc
8941 ttttgcaccg ccttgagctc ttcggtagcc gccttgctgc gattgctgat ctgaacccag

9001 gccacgacgc cgaagttcat cagaaacatc aagacggtcc agaagttgaa gtcgaacgaa
9061 actgtcatcg ccgcgcctcc gctgctaaat agcgctcgtg ctcaatctgg cagtcgatac
9121 accgaatcgc cgtcggatag gcatcgagcc ggcccattgg aatcggctcc tcgcagtcat
9181 agcaaatgat cgtcggcgca ccgtcttcgc cggcgaccac gacctgcggc atgtccggct
9241 tcatcatgtc cagcgcagcg cgccggttca gctcgagctc gtgctcgatg cgctcttccg
9301 caatgtcagc ggcgtccatc ctgatctccg atatcacacg acaggccggc cccgaattcc
9361 gtcggcggtt cgccatgaac aaatcccgat gcgtcaggga acgtcaggta gccggtcggc
9421 tcgcaagcca gacagtcgca accggcaagc acaccgatgc agaaggccag catcagcaac
9481 gccccgccac ccacacattt ttcctgtggc gtcagcatca gtgcccttcg gccccgcctt
9541 tgcaggcttg gatggcctcc cgcgcccgcc catagccgac caccagcgct ttgccgccgg
9601 catcggcttt gggaggccga cccggggcga tatcgacgaa cacggtcgag gccaacggcg
9661 gcagatcggg cagagcacaa gactccggcg cgaatgccgg tgtcaggatc ggcttcggaa
9721 ccagctcttt cggccccacc tgcacgcacc ccgccagcgt tagccgtagc gccagaatcg
9781 ccgcgcccgg gaatccgcta acgcaacgca cggtccaccc ccgcttccac atcctcatgc
9841 ttgagccagc gctcgcacgc catcgcgccg ttctcgtggg tactgatggc ccagaccagc
9901 agggtgagcg ttttccggtc ggacaggatg atccggtcat ccgcccctac gtcgagcgcg
9961 gcggcaaccc gccggatgta ggtggcggtg tcgttctcgt tcggcggcgc ccagcgggtg
10021 atgatctggc ggacggtgtg gagctggcgc ttcctctggt agttgagcag cgtcacccct
10081 agcgcccgca ccccgtcttc catttgctcg aaccgggcaa accgtccgtc gtggccgacc
10141 tggccttccc aggcgttgcg ggggctgtag acgagattgc cggggttgtt gttgcgcagg
10201 ccccttggct tctggaagct catttctgct gcctccgcac gaacgccgcg aattcctcat
10261 cgctcatatc ctcccctcgc ccttggggaa gaggggccgg ggtggcggcc ttgctgtacc
10321 ggtcggtcac cttcaagtgc gacctggtct cggcgctcac gcccggcacc gcgccgccgt
10381 acggcttcgc gctgcgcgcc tgcggcatgg cggagacggc ctctgccgcg gcggtcaccg
10441 gcaccgcgca gagcgggtcc accaccacca cgctgaagct ggcggccgcc gcctccagca
10501 tcgacggcgt ctatgtcggc gcccgggtgc ggatcaccgg cggcgccggc agcggccagt
10561 ccgccaccat catcggctac gtcggcagca ccaaggtggc gacggtcgac cggccgtgga
10621 ccgtcacgcc ggacgcgacc tcgctctatt cggtcgatcc gttcgtccgc tataacccgg
10681 tgtctgacat cctgaccatg gagtcggcga ccttctacta caacgaaggc aaccaggttc
10741 ggcacaagct gctgggctgc cgcggcaacg tcaagatcga cgcctcggcc aaggacacag
10801 gcatgctcga cttcgagctg atcggcctgt acggcggcgt ggcggacgcc gcggaaaccg
10861 gcgtggccgt gtccaactgg gtcgatccct gggaggtcgg ctacgggcgc acctacgggc
10921 aggtcttcgg caagccgctc acgggcgggg cctccgggct gcagatgggc aagttttccc
10981 tcgacctggg ccagaaggcc gcctaccgct cggtggtcgg ctaccagggc gtcatcatca
11041 ccgaccgcgc agcctccggc tccctcacgc tggacgcgac gaccgtcgcg cagttcgatc
11101 cctggacgct gatcgccggc aacggcacgg gcgccatcgg catcgagcag ccggacagca
11161 acaacggctc ggtccgcatc gacgtcccca aggccgcgcc caccgagatc gactatggcg
11221 acgaccaggg catctcgacc aacacgatcc agttcgactg ccagcgcgcg ctgggcaacg
11281 acgaggtctt cattacctgc cgctaaggag gcggattcat ggcattcaag ctaggcaccc
11341 aggaagtact gggcaaaccc attgcccgca aggtgatgct gccggtctgc aacgaggccg
11401 gcacgcccgt caaccaccag atcaccctgc tctacaaccg catgacggtg gaggagatcg
11461 gcgccgccta tgccgcgctg gcggaggacg cccggatgct cgaggcgctg accgggccgg
11521 agctgaccgc cgcggtgctg gacatgcacg ccacccacgt cctgcggctg gcctcgggat
11581 ggcgggaggt gcaggcggag gatggcgccg aggcggcgtt caccttcgac aacgtccgcg
11641 ccctgctcaa cagcgtgccg aaggcttacg accggatcac cgaggagttc cagaaggcca
11701 accggggcgg ggccgcgctg ggaaactgat cgaggcggcg gagtactggg cggcgggcgg
11761 ccggcggccc gaccacgcgc tcagggacga cctggcggcg ttcggcatgg ccgcgccgga
11821 cggggacggg gagcccggcg acggggattt ccccgtcatg gcggagaact gggacacggt
11881 catggccttc ctggcctgcc ggaccgcctg gcggctgtac ttcccgccgc tgggcggcgc
11941 gccggtctgg ctcggcctgt cctacccgga cgtggacgtg gtgatacgcc acctgggcca
12001 cgaaggcgag gcggcccgcc ggatattcgc cggcatccag gtcatggaag cggcagcctt
12061 ggccgtgttc catccgccgg cggcggagtc agcggctgag gaataaccgc ccggcggagg
12121 tcaggcaagc caggacgaaa acggcgatac cggcccacac catggcggcg ccgtatttga

12181 ggaatcccag cgcgaacagc agaaagccca gcaccatcag actgattaag gacatatggc
12241 cggccctatc gtagttggta tcaaactcaa tctcgacgag aaaggagcgc tggtcggggt
12301 tgagaaggtc gaaagcggat tcaagcgcat cggctcggcc gccagcgaat ccgccagccg
12361 tatcgacgca tcatacaaga aagtcgaaac cggcattgcc gaagtcgaga acggcttcaa
12421 gcgcgttggc tctgccgcca gcgaatccgc tggccgtatc gacgcagctt acggcaagac
12481 ccgccagggc ctggattcca tcagcacgca gctggcgacg gccaagcgcc agtttctcga
12541 attcatcacc gctcaggcgg cgctcggcgc tggccgggaa ctggccgatg tggccgaccg
12601 ctacgccaac ctctccgccc ggctcaagct cgccacccaa agcgagctgg aatacgccaa
12661 ggcgcaggcg caagtcttcg ccatttccca gcgctacagc accgccctcg acgacaacgc
12721 caagctgtac tcccgcattg ctcccgcggt gcgcgatgcc ggcaagagcc aggccgacct
12781 cgttaaagtc atcgaggccg ttaacgccag cctgaagact tccggcgcca gcgcagcgga
12841 gcaatcctcc accatcaccc agctgtccca ggcgctggcc tcctccacgg tgcagtggga
12901 ggacttcggc cagctcgccg acacgaacat gcggctggtc gatgccgtcg ccaagcggct
12961 ggctggcaat atgggggagc tcaagcaaaa gatgtcggac gggctgatca gcaacacgca
13021 gctgttcgac gccatcatcg cggcctccga gcagctcaag gccgaagccg ccacgatgcc
13081 caacaccatc ggcggcgcgg tgcagcagat cgaaaacgcc tgggagcgtt acatcggcaa
13141 agtcgaccag gcggccggcg ccagccgtga cgtggccaat gccatcagcg gggtagccac
13201 ccacttcgac cagattgccg acgccgcggt ccgggtcggc gaagtcgtcg cggtcgccat
13261 cggtgtcaag gccgtggccg cgatgactgc ctatgtggcg gaggccgcgg cggccgccaa
13321 tggcgcgctg ctgttcagcc gggcaatggg agcgctcggc gggccggtcg gtgccgccac
13381 ctttgctgtc ctcgctggcg cggaagccct ctcggtctgg cgcgccaaca tgatcgcggc
13441 caacgacgag gcgctcaaga tccgcagtca gggcaatgcc ggcgcgctga ataccaccga
13501 tcccaccaaa gtcatgggcg ccgatctcaa ggccaaggtc gaccaggcca agcagtcggt
13561 aggcgagctg actgtcacgg tcaacgcgtc gagcgccgcg ctggccgccg ccgcggagac
13621 gacgaagaag tacaccgcca ccgagcgcga ggacatccag tccaagatcc gctggctcga
13681 agcccacggc cagcaggcgg acgcgctgcg gctcgaaggc gccctgcacg gcttcaccgg
13741 caaggcgctc gaggcctacg tcaagcagga actggacacg attgcggcca ggaaggccaa
13801 cgccgaagcc accaaggccc acaacaaggc gctgtccgag gaggccagcg cggtcaagaa
13861 gtccgcgacg gaacaccaga acgcgctcga ggccagcgac cgcttcatcc agtcgctgaa
13921 ggacgagctg gcgatgctgc aggaccgcac cggcgcgtcc gaggccgaga tcgcggcgcg
13981 caaggcgtcg gcccggctca ccggcgagga ggccgaggcg cgcgcccggc tcgcccggca
14041 gatcaaggag gagatcgacg cgctcgaggg cgcggagcgc atccgccagc tggagatcaa
14101 gacccagaac gacatcgccg aggcgatcca gcgcaaggag gaggccgcgg cccgggcgct
14161 gcagagcgcc caggacgagg aacagaagca gctcgccctg ctggcccaga tccaggccgc
14221 gcacgaggcc ggctacgggc cggaggcgct ggcccggatg cgcgcctacc tgagcgcggt
14281 ctacgacgtg acccgcggca tgggcgagga ggaggccgcg gccacggcgg ccgccatggc
14341 ccacaccgag gccgccaccc acatgatgga cgagatgacc cggaccggcc agtccatgct
14401 ggacgacctg ggcggcgtcg cccgcagcat gatgagcggc ttctcctcgc tgttcttcga
14461 gcctttcggc gcggccaccg agagcatggg ggcgcggttc agccgggtgc tgcagcagat
14521 ggcggcgcag ctcatgcaga gcgccgtgac gcggctcttc accggcggtc tcggtctcgt
14581 ggccggaggc gaggccggcg cgggcggctg gctcggcggg ctgctcggcc tgggcgggtc
14641 cgtggtcggc gcggacggcc tcatcacccg catgccgcag gtcggcgagg ccatggtgct
14701 gccctccggc agcatgatca tcaacagcgg cggcaacgcc gtgtccagct tcccgcagta
14761 cgtcaactac gccggcaccg gggccagcgt ggccggctac ggcaacggcc tgctcggcgg
14821 ccccggctgg gccaccgcgg cgctgggcta tgccggggcc gggctgggcg gcgcgggtct
14881 gggctacctg atcggcgggc tgatcggcga ccggtacaac tacggccagg cgggcggcgc
14941 gatcggcggt ctcgcgggcg gcatcggcgg ctatgccgcg gccacctcga tgggcttgag
15001 cggcgccatg gccggcctcg ccgcgaccgg cgttggcgcc attgccgcgg ccgtcatcgc
15061 ggtcattgca agccaggtca aggtaggcaa accggatgcc cagctgatcg ccaacgtcac
15121 gcccggcgag ggttacgaca tcacagcgct taactctcgg catggtggca agaccggcct
15181 attcggcctg ctcgagggac tggcaaacat caccttcgac ccggcggatc cgttccactt
15241 ctcgcgcgac aacagtccga acaaaggcgc ctgggcgccc ggcgagaaat tcctcggcga
15301 cgccctgacg gccctgttcg gcccgctggg caagctcgag gagtccctcg gcgtgatgat

15361 cccggcgttc cagttcatcg ggcgcatcca cgacgacagg accggcatcc agatccccgg
15421 catctacgcc tccggaccgt tcaagttcga cgacaacgcc gcccagtaca tgcagcaggc
15481 cgtgagcatc gccaccatgc ggctgctcca gcagccggcg gtgctgaacc agatgaccga
15541 cccggtgaca aaggccgtgg tcatgctgtc gggggatctc caggcgtttc aagccaacct
15601 cgaaaaggtc tccgaggtcc gcaaatatgc cggcggatcg ttcgaagaag tcggcggacg
15661 caaagcactg ggcaaagccc tagatttgag cggcggcgac atcgaccagt acattcagaa
15721 catgaaggaa ctgggcgcgt tgaggacatt tctcggcgag tggaccccgg atgtaggcgc
15781 atggacccag accgtcgatg gattcgacgc aatggagaaa gccgccatca agttcggcgt
15841 gtcggtcaag aaggtttccg agcagatcga taaaaccatc caggctcagc agttcggtat
15901 ccaagaccag gtcaatcagc tactcggcaa gccgcagtcc acgcgctcga agctgtttgc
15961 cgcgaaccag acatttgacc agctcggggc gaaggaatcc ggcacgctgg ctgagttggc
16021 gcagtacaac aaagaggagc gaatccgcgg caagaacgcg ctcgcccagc tcgacgtcaa
16081 aacggtaagc gcgatcgacc gccagtcggt caagtcgatg ctcgacagac tcaaagatcc
16141 gaagctcgcc agcgatccgg caatgctggc ggagtggcag gctggatggg accggttctg
16201 ggaaaacgag ctgacccaag ccgagagcat caagaatccg aagaagcgca aggaggctat
16261 cgccaaaatc aaggcgctcc agcagcagac cgacccgcac gcgactgagc agatcgacat
16321 cgaagctgcg cgggcgcaat acgtcgagca gcagaaaaag gccatccttg accaggccca
16381 ggcttacgcc ggtcaaggtg cgtcgcaata tgccgcgaag atcaaggaga tcaacgattt
16441 cttcgccgac gcgaacgaag ccgcgtcgga gctcggaatc tctacgagcg aactggccga
16501 ataccagaag aaggcgacgg cggcgctccg ggacgatttc agcaacgcac tgaagcagtt
16561 tgccggcgag accgtatcgg gcgccgctgc tgcgctgaag tccatcgacg accaggagaa
16621 gacgttcgag aaggatgctg ccgcgcttgg catctcgctc gacaaggtgc acgacgctgc
16681 cgagaaagcc cgccaaaagc tcaaggatga tttcgtcaag agcctcgaca gcatggctgg
16741 cgtgctcgac gccaacaccc aagcgttcaa ttccctgagc gaccagttca agacgctgta
16801 cgaggacgcg cagaccctcg gtgtcggtat ggacaaggtg ggcgcggcgc tggtccagtc
16861 catgaagaat ttgtgggagc aaatgaccgc cgggattgtc tctgcccgcc aggccattgc
16921 ccagcagatt gccgagttgc aaggcccgca ggcggtcgcg acgctggccg gtcagaacgt
16981 cgataccgcc aaggggaaat tggccgacta ctacgcctcc ggcggcaatg acgtcaataa
17041 ggaaatctcc ctcatacaga acgtccagtc ggcggtcatg gcccggtaca acgccgagct
17101 ggcgctgata caggagacga ttcagaagga agtcgaggcc atccaggcca aggcccaagc
17161 cgaagtcgat gcggtcaacg agacgctgaa cgtccagata gaggccgaga acgagcggct
17221 gagcgccgct atcgaagctg aaaacgaacg actcaatgca gtcctcgagg ccgagagcga
17281 gcggttgaac gcccagattg aggctatcaa cgcagccagc caggcgcagg tagacgccat
17341 ccaggagcag gccgacgccg cagcggaagc gaggcaggag gcgcagcagg ccgagctgga
17401 cgctctgagc gaacaattgc aggtcgcgaa tcagctcaag tctgccatca agaatatcgc
17461 cgactatgcg aaatcgcttc tgaccgccgc cgattcgccg ctgtctccgg aagcccgcct
17521 tgcggccgcc aagcagcaat atcaagacac gcttgccaag gcgcaggccg gcgacgccga
17581 ggcggcgcag cgctatcagc aggacgccga ggcttacaag aacgccgcga aggactacta
17641 cgcctcgtct gccggctacg gcgacgtcta caaccagatt cagtccgacg cgacggcact
17701 gggcaacacg actgtcacta gcccggacag catcgaatcc cagatgaagg agctgcgcaa
17761 gcagcaggcc gccgagaaca aagaagcgca aaagctggtc caggaacaga tcaaggccgt
17821 ccagcaaggg gcgcaagacc agatcaaggc cgcgcaagag gcgtcgcagg actacataaa
17881 gtcgctccag gatgcctcga aagacaccat cgcgcagatg cagaaggact ctcaggcgac
17941 ggtagagggt atgcgcgact ccgctaatgc ccaaatcaag gccatccagg acgccgctgc
18001 tcaggccacg aaagacgcgc aagacctcac aaagcgcgaa gacgtcgcag cgctgaagaa
18061 ggacaccatc gacaatctga ccgccctgga cggtcatttg caggccgcct acgacgccgc
18121 ccagaagcag tatcagacgt tcatcgatgc cttggccggt gtggtcggca gcaacacgac
18181 cgcgatttcc gcgctggtcg actggctcaa caagcagggt gtcgccgtca cccagccgca
18241 gacgaaaaac accatcatcc aggtcaacgc cgacggcagc cagaccgcgc tccaggaagg
18301 caacagcggt acgaccgaat acctcccgcc gccgcattac gctgccggcg gcgtggctcg
18361 cccgggctgg gcgctggtcg gcgagctggg tccggagctg gtcaatttcg accggcccgg
18421 tcgcgtctac acggcccagg acacggccca aatgatggcc ggtctcaacg ccgcgaatga
18481 cagtggagcc tatgcagatt tcgtggcgcg gcaatccgag tccgtagcga gaatccagga

18541 gtggattcaa tcctggattg ccaacgcccg ggcggcgaac gacgcccacg gcgcctatgc
18601 caacgtactc gacatgccgc ccacggccca gaacgcctgg aatgcggtgc agtcactcat
18661 gctcaagccg gcccagaccc aggagctacc ggactacatc ccggcagtag tggagcggca
18721 gacggcgcgg ctcgaagaac gactcgccag gattgagagc ggccagggcg gacagcagtc
18781 ggaagccgtg cgcgaggaaa tccgcgggct ccgggatgac ctccgcgcgc tgatcgtcac
18841 ccagtccggc accaacccac gcatgctcga aaaattcgac gcgctcattg cccgattcga
18901 ggacatggtg agcgagacca agctggcgag ggggcgcgcg gcatgatcct cctccaggaa
18961 ttcgactact acgacggcag cgagaaaacg ctgtacttga cctacggcgc cggctacgac
19021 aacgacgccg ggattttcta ccggcccgcc atcatccagc cggcgaacta cagtcgcgac
19081 gccagcctgt tgcaatttgg cgggcgcacc agctccagct acggcgagac cacgattgcg
19141 aacggcgacg gctatttcga ctacctgaat ggcgcctatt tcgaagggcg cgagatgcgc
19201 atgtacaccc ttgaagacgg gcagccgtac tcgtcgcgcg tgctggtcat gcgggcgatc
19261 atcgacgctg tcgactggaa gatgagtgag atttcgttcc gcctcaccga caacagcaag
19321 tcgctcgaca aacccctgca accgaacaag tacggcggca ccaacgccct gccgaacggc
19381 atcgacggca cgccggacga tatcaagggc cagagcaagc cccgcattta cgggcgcatc
19441 tcactcatgg gcggagtcga ggtcaacacg tcgaagctga ttttccaggt caacgaccgc
19501 gccgtcgcgc agatcatcaa cgtgttcgat aagggcgctt acctgaccag ggacggcagt
19561 acctacgcca accaaacggc aatggagacg accgctccgg catcgggtgc attcaaggct
19621 tgcccgtcca tcggttgctt ccgcaccggc tccacgccat tcggcgcagt ttcaatctgc
19681 gttgccgaga gcttcgtcca cacagaaatc agtgctgctg gagtcatcaa gcgcgtgctc
19741 gacgacatgg caatttcggc atccgcctac agcctccagg atttcgcgga cctcgacggc
19801 aaaaactgcg ggccggtcgg tgtcatcgtc caggaccagg aaaccgcgtg gtccgtcatc
19861 gagcgcatcg ctgcatcggt cggagcctgg gtaggtttcg atgcgctcaa cgtatttcgt
19921 gtcgcccggc ttgatgctcc gaccgggagc tattcggtcg tcatcgacga caccgtgatc
19981 gactccatca ccaagccggt tccggctgtc gcaccggcct ggaagatgac cttggagggc
20041 gacaccaact atctggtgca ggacaaaaat gccctggccg gcatcgtacc gaccgcccgg
20101 gcagcctggt tcgaggccgc gacccgcagc gcctcggcgt cggatacctc cgtcaagacc
20161 atccgcatca actcgcagga ctacaccgcc tcatcggtgc aggtttccgt gtcgcagctc
20221 caggccgaag ccacgcgccg gctgaacttg ttgaaaaacc gcctggacgt cgtgaccgtg
20281 acgctccgcg actcgccgac ctcctggctc ggacaagtgg acatcggcac cgaagtcctg
20341 ctgcaaacgc gctggtacgg ctacgacgcg ggccgcccga tgatcgtcac cgggctgcgg
20401 accaacttcc tccgcaatca actcgacctg accttgatgg ggtaatggat ggcctttttc
20461 ctgctcgggc acaccaacag aatcgacgcc gccacgctct cgggcggtac gtggaacagc
20521 ctgtatccgc tcacgaactt gcaggacgcg gcgctggccc gcgtcgcccg cagcgtcggc
20581 acgacatcgg gcgccagcac gattgtcatc gactgcggcg cggcgaagga catccgcacg
20641 ctcgggctcg tcaaccacaa catccgcagc accggcacgg tccggctcga aggctccaac
20701 gcggtcggct tctcgccgct cgtcgccgat agcggcgcgg tcacggtcta cgccgacacg
20761 gtagcggcca atcccacctt caccctcgac ctgggcgcgg tctacttggc ccggtactgg
20821 cgcatcacga tctccgactc cgggaatcct gcgggctata tccggctcgg gcgggtattc
20881 ctgggcgacg gctggcagcc cacggagacg aatttctcgt ggggcaagat tctcggcatc
20941 gagaatccgt ctgttgtttc cgttttgccg agcggccgtc gcgtcgtcga tgtgcgcccc
21001 cctagccgca atcagaaata ccaaattaaa gaccttacga aatctgacgc gctgcgagag
21061 gttttccaga tcgcgtggac gcacggtctc gacaaagagg tgctgctgtt cgaagacccc
21121 gccgacaaca cttacgccga cgtgaacaac ttcctggcca cgatacgcca gctcccggct
21181 atcgagtacc cctatctcga cgcctacagc gccgcttttg agttttccga aatcattgcc
21241 tgataggagg gctgaatgag ctacaccatc caagatgaag tcaacgtcga ttttgctgcc
21301 gaaattcgcg gcggctgcat cgttttcgaa tccgaagccg agacggcaaa gcggagcctg
21361 ctggcgtccg acggctcagt catcgccgaa gccatcgccc acaacggcgt caaggtgctc
21421 gacaagcccg ccgcgcggct ttggttcgaa tcaaccggag ggggcgagtt tacccggctg
21481 gcgctcgaat ctgcattggc tgaaaccttg gccggtgccg acgaactgct cgccgcggcg
21541 caaacgccgg cccatgtcga agccgtgcgc ggcatgctca agtctggcgg atggacctgc
21601 taccgcgtcg ctggcaacga cggcgctacc acagcacttc tgctcatcgc catcggcgcg
21661 cagccgaatg aagacctcga atacgaactc cctggcaacc ccgtcgaaat cgatgccgac

21721 gactgggcga atgcctgggc agatttccgt gaccgcgttt acatcctgca gtgaggtagt
21781 cagatgaatc gcaaaacctc gaaatcctta aaccgggagc tggcgcaggc caaggccatg
21841 tcgctcgcta aggcggtttt ggcggccagc atcatcgaag cggcgacagc ccatggcgca
21901 ttcgcctgct gggcggtcgg cccggtcgag catatgcggg ccgagtatgt ggagatgctg
21961 gccgaggcgc gcgacctgga ggagggcggc gccatccaac ttgccgcaaa tctccgggcg
22021 caggccgagc gcatgctgga ggagaaatgg cacgacgcag cggcgaacgt ggtctgcacc
22081 ggcggcaaga atgatgcgct cgacaagtac ctggccggca gcgcctatac ggccgcttgg
22141 tatctcgggc tgatctcatc gaccagctat accggcgtcg cggccggaga caccatggcg
22201 tcgcatgccg gctggactga aagcacggca tactcgcagg ccacgcgagt ggcgccatcg
22261 ttctcggcgg ccagcagcgg cagcaaagcg acctcggcgg cggcctcgtt tagcatcaac
22321 gccgcagcga ccatcaaggg gccgttcctg accagcaaca acaccaagag cggaacgacg
22381 gggattctgt attccgccgg cctgttcagc tcgggcgacc gtacattagc gaacggtgat
22441 acgctcaatg taacgtatac ggcgggcctc tgacggtaac gctaagtggc aacggcaggc
22501 cctaattacg catcgacggc agtatccgat agcacgctgg cgggtaatgc ctggagcaat
22561 ccgagcaatg gcacggccag cgacaactcg tatgcaacgg ccagctcgaa cggaactcag
22621 tatctcaaag ccacagggtt cggattttca attccgacag gggcgaccat cgacggcatc
22681 gtcgtcgagt gggagcgcaa ggcgtcgagc tcaaccgtca aagacaaagc tgttcgcatt
22741 gtcaaaggcg gagttgtcgg agctacggac aagtcagcgg ccggcgactg gccgacgacg
22801 gataccttcg ccagctatgg cagctcgtcc gacctgtggg gcgagacctg gaccgccgct
22861 gatatcaatg cagccaattt cggcgcggcg attgcgacgc agagcacatt cgtccggacc
22921 gcgtcggtcg atgcggtacg gatcacggtc tattacaccg atgtttcggc gataacggaa
22981 agcgctaccg ctacggaatc atcggatgcg gctgcaacgg tctcggatgg cgtcaccgag
23041 accgcaacgg ccagcgaatc cggcgatgcg acaagcgcgc tggtggctga catcaccgag
23101 tcggcaaccg cgacagaatc atccgatagc gcgctcggcc cgaagcaggg cgacatcacg
23161 gagtcggcga ccgccgccga atcatcggat gccgtcgcat tctcggccga tagcgtaacc
23221 gagtcggcat ctgctaccga cactcaggat gcgtcggcaa ccgcgaacgc cagcatcatc
23281 gaaacggcgt cggcctccga gtcgtccagc gcgtcggtca tcgtttacgc cagtgccagc
23341 gaatcagcga cggcgacgga atcctccgat ggtattgccg ttggccagct ttaccgcgag
23401 gatgctaccg ggttcctggt tgtcaaatct ggggccggag cgggctatac cgcgctcgcc
23461 gctcccacaa ccgccgcagc ctggcgagtc ctcgacatct atggatcgac cagcgcatgg
23521 cacccatccg acgacccgcc cgcccaggaa agcgccgccc ccaccgtgga ctacagcaac
23581 gtcaccggca ccaagccgcc ggcggacgcc acccgcaacc agatatacat ccagtcgggc
23641 acgccctcgt cgccagtcgc cggcgacctc tggttcgaca ccgccaacaa gacctgggcc
23701 acctggaccg gctcgctgtg gcaggtggta tccgacgtga ccgcctacaa gaccgccgcc
23761 tccatcgccg ggcagggcgc gttcgccacg ctcaatctcc tgacctcggc caacatcgac
23821 agctacctct cggacgccgc cctcggcacg ctcaagctgg cgctcaacgc gctctcggtg
23881 ctgcagggcg cgtcggggct ggtatcgacc acgtacaaca cctgggtgca gaccagcacc
23941 tggaacatcc ccgccgtggc cagcgcctcg ctgcgcggca agttcctgct gcttctgtgg
24001 cggcagtgga acgacctcgg caccgactac accggagcca gcgccatcaa gcgcgatggc
24061 gcgaccctcg tggacgtgta cgaccgcatc accaccaacg ccagcggcat cggcaagtcc
24121 cagaccatcg cctggatcga ctcctcgccc cccgccacgg cggccttcag ctactcggtg
24181 cacgtgttcc agaactacaa tggctccgcg cccaacatca actcaaactt cgccatactg
24241 gagctgaagc gctcatgaac gcgatctgga ccgtctatca accctcaacg ggccgtatcc
24301 tggaacttcc caagtcgatc ccggacgcct acgcccatct ctatctcggc cccgaccggc
24361 acgtgatcga gggcgcatgg tacccggacc gccactacat cggccccgtg ggggccctgg
24421 agcgtccgcc gcaggaaacg agcctcgaca ccatcacgat ccgggccgac ggcgaggatg
24481 tcgccacgct gagccggctg cccgaccccct gcacggtcga aatctacggc ccggtgcggg
24541 acgtcatcga ggtcgagggg ggcgtgctgg agttctgcgc cgacgtgccg ggccgctacg
24601 agctggtcgt gaaggccttt ccctggctgg acttcacggg gatgatccat gcgctataag
24661 gccacccttc cgccggaaga cttcaagcgg ctgatgcgcg ccagggtaaa cgccgagagg
24721 gaggcgcggc tgcgggccgg gttcgagttc cagggccaca cctacgacac cgacccgcag
24781 tcggtcgcca acatgaccgc ggtgctcgcg gccctggggg ccggcctacc gctccccgag
24841 ggcttcgcct ggcgcgaccg cgccaacatt gacgtgccga tggacgaggc gaccttcaag

24901 gcttttaccg gctctctgct tgtccatgtg aactcgatct atgtcgagtc atggcgcaag
24961 aaggacgaga tcaagaccag cgggaaccca gcttcgattg atgcggattg aagacttgaa
25021 agacagcgac tggccgagtg ctgcgaacac ccggccagcc agctgacccg cagcgtatac
25081 ctgcaagcca acccaaggct gccaccgaat ggccactcgg tgtggcaagc ctagtggatt
25141 tctaacgttt ctgaaagagg cttgcgtccc atgcaatgta aacccatcgt gccgtggatc
25201 ggcggcaagc gccgtctggc gcggcatatc ctgcccttgt tcccgcccca tgactgctac
25261 gtggagccgt tctgcggtgc ggccgcgttg tatttcctca aggagccggc caagtgcgag
25321 gtcatcaacg acatcaacgg cgagctggtc aacctgtacc gggtggtgca gcaccacctc
25381 gaggagttcg tccgccagtt caagtgggcg ctgacttccc ggcagatcta tgaatggatg
25441 aaggctacgc ccgagcaaac actgaccgac attcagcggg gcgcccggtt cttctacctg
25501 cagaagctgg cgttcggcgg caaggtggag tccatgacct tcggcacgtc gaccacgtcc
25561 ggccccccgg ctcaacctgc tgcggctcga ggaggagctg tcggcggcgc acctgcggct
25621 gtcacgaacc accatcgaac atctgccgtg ggaggaatgc atccgccgct acgaccggcc
25681 gcacacgctg ttctatctcg acccgcccta ttggggcact gaaggttatg gggtggagtt
25741 cgggttggag aattacgtcc gtatggcgga actggctcgg acgatcgagg gacggatggt
25801 tatctcggtg aacgatatcc cagagatgcg ggcggcattc gaagggctgc cgacgaaaca
25861 cgccgacctc tgttactcag tcggtggcgg taagcggtcc cgcagatcga gcgacgaact
25921 gatcattcgg agttgggcgt gatctacgcg gcacatgcgt tgctcatgaa gttattatgt
25981 ttccccacgc gcaaaaccgt catggacgat ttatctcaca atttcgtgtt cgtttatcgc
26041 gcgcggcttc atcatcggcg ggcacttcgc aattacccgg ccatcacggc tcatccccat
26101 cgcagcaaaa agcgattgca aggggtaccg acgcatcctt tcaatcgcgt tttttcggag
26161 ttcttccgta ttggctccgc gatgcaggtt cgcaaatgcc agcaaggcga gttgcgccga
26221 tttaccggtg acggacttcc tggcgctctc gatgagttga gtaatatcga cgcctggcgt
26281 ctgaataagc accatctctc ccagagctcg ctcgccagaa tcgttcaagt gatcacgcag
26341 ttcagcaatc cttgcatcga cttgatgcgt ggagcgctcg gctctgggta ctgttcgata
26401 cgtctggatg acgttttcaa atatgctcgc agccaccatg tgactggggc tctctgatgc
26461 ggtttgtgca accgcttcct tgacccagcc ttcggctacc gcaaccgtca tttctgccgc
26521 tttagccgcg tcaggaatgg tcctgtacca ttcggcagcc gaagaaaaat actcccgcgc
26581 cctgtgcaga tcaccggcac catcaaattc tcgggctagc gcttctagct ttctcgccac
26641 acccactcgg tgatctcgcc ccaaaccatt cgatttcaga aggtcggcca gccaaagtcc
26701 gaggaatccg tcgtcccgct tggctgcgtt aaacgcagta actatggagg cctccatttg
26761 ctgaagccta tctccggccg ccgtcttgag catccgtgcc agactaatcg cacgtgacca
26821 gcattcgcag cctccgtgta cccaggtatc tgcgtcgagt ggaaggcggc ggtaagcgtc
26881 aatggctttc agggcgaatg cagtgttgcg aggctcgccc ttcaaccaga gcaagtcgct
26941 gagccgtgcc ttcagccagt tgtcgtcgac cgcatcgacg atttcggcaa agaacgcaat
27001 gtcggtatcg agcagatcat ccggtatgac tgaccgccgg tcatggaaaa cagcaaaggg
27061 cttgaagggt tcgttcgtac ttgatggtac gagcatcatc gaacaggcat cggcaagcag
27121 ccataggacc ttgccgtgcg caacacgacc ttgctctatg gctgtgcgtg cggcactcga
27181 cagtgccagc cacatcgccg agtagccttc tcgggtggct ggggcaatcg cgtctttcca
27241 acctgacgcc tcaaagtctt gaagcgaaac ggccaaatcc tgcgggtagc gttcgttgct
27301 cacagcccac gctccgtaac aaaatcctcg gcatccttca cgcgcagctt gccgaagatg
27361 agcttgggca gcagggtgtc gcggaggtgg gcgagggtgc gggattcgcg ttcgttttcc
27421 accagctgtc gataaagggg gccaaccatc ttcgtgaaat ttgtcagcac ctggtcggaa
27481 agaaccacaa tccgaagagg ccggaagttg cttttgctga tctcaagaaa cgtcaagccg
27541 ttggcgttac cgacaatcgc atccatattt tccttgcacc acaacagcac gaagacgttc
27601 ggcagggtgc catcacattt catcgcgatg aagccctggt tgatcgctgt cggcacttcg
27661 gcaatcgcca aataaccgat gggggcgcgt gacgagagca acacagtgcc gacgggcaga
27721 aggcctgaac tgatctttgc aagaccggca tccgtaattt ttcgatctgt gtctagcaaa
27781 acgggaaact tcagggcgga caggtcttta ggcgttgccc agcagtgttg cccaccttcc
27841 caaaactccg gttccttggt gctgggtgtc gatccgacac agaccgtcac ttcttcgcca
27901 atcgtcgaat gacgccaccc ctccggaatc tcccccaatt cggattcgac gaggcggtcg
27961 gggaagaggt cgaggatatg ttgcggccaa tgccataccc cgtccgcatc gagcgtgccc
28021 caaagctccc cctctccctc tgggagaggg gccgggggtg agggcgaaga aggcgaacgt

```
   28081 tgctccacga tcgcctgaaa aatcgcctcc agcaccgcct cggtttgctg caagacatcg
   28141 tgattccaaa aacgcaagac gcgcaggcca tgctcggcta gaaggcatcc cggcgagcgt
   28201 cgtgccgctg cccggcctct tcgttgtgct gccctccgtc gatctccacg accagcttgt
   28261 gatcgtggca atagaagtcg agcacatacg gcggaaaggg atgctgacgg cgaaacttca
   28321 cgcccgcaag ctgacggttg cgtagcaggc gccacagcag cgcttcggca tcggtcatgc
   28381 ggtggcgcag ctcgcgtgcg aagtcaagga tgtcggcggg aagtttgggc tttttgccct
   28441 cacccccctgc ccctctccca aagggagagg ggagattcat cttggcgcgt accggctcga
   28501 agtccacgaa ccacgctcgg aacaaggcgc gggccatcgc ctccagcgtt tcgttctggc
   28561 ggcggttgag ttcgatcttg tcgtccagcg tgctgaggat gtgggcgatt gcacgttgtt
   28621 cgggaagggg cggcagcgga atctccagcg agcgcagata agtcaccggc tgcgcaatcg
   28681 acggcacgcc aacttgcgag gtattggcta aaagcttgtg ctggccttcc ggcgagcgga
   28741 agtaggcaac cagaaactcc ggcagcacct tggttggatc ggggcggagg taaaactggc
   28801 gctgggaaat gacgtacctg tcaaaagcag atgtgctcgg tatgtaagca acttgaccaa
   28861 tgttgccagc gtgagtgaac acaacatcac cccgctgcac gtttgcattg cgcagtcgtt
   28921 ctgcatgttc gcgggttatg aagttgaacc ccggcgcatc gtccaggcga aaaccgtgca
   28981 aatgctgtcc actgatcacg ggcacgccct cggggacaaa cgtctcgacc ttgatcgaag
   29041 agccgaacgg tcccatggcc acgcgctcgg caacctcttc gatccgacat ttcgtcc
//
```

```
LOCUS       McNor-R2             45876 bp    DNA     linear   PHG 16-FEB-2025
DEFINITION  McNor-R2.
ACCESSION   McNor-R2
VERSION     McNor-R2
KEYWORDS    .
SOURCE      .
  ORGANISM  .
            .
FEATURES             Location/Qualifiers
     CDS             1..270
                     /ID="WDFLIPAM_CDS_0001"
                     /transl_table=11
                     /phrog="No_PHROGs_HMM"
                     /top_hit="No_MMseqs_PHROG_hit"
                     /locus_tag="WDFLIPAM_CDS_0001"
                     /function="unknown function"
                     /product="hypothetical protein"
                     /source="PHANOTATE_1.5.1"
                     /score="-7.573913271799362"
                     /phase="0"
                     /translation="PFFLRAPACSRGLLRIPADFLPPVVSPISDPFRSLRAVILSSLAP
                     RLRAEVHRGRNADPYRSMGYALTNQTVGLRHRQAKETMSVVFCR"
     CDS             310..909
                     /ID="WDFLIPAM_CDS_0002"
                     /transl_table=11
                     /phrog="No_PHROGs_HMM"
                     /top_hit="No_MMseqs_PHROG_hit"
                     /locus_tag="WDFLIPAM_CDS_0002"
                     /function="unknown function"
                     /product="hypothetical protein"
                     /source="PHANOTATE_1.5.1"
                     /score="-57.943566195386886"
                     /phase="0"
                     /translation="MDSSHQSILDLAAQRGLIRPRDLNERGLPTVALTRLVRQGLLQRV
                     GRGLYAIPDRAVSEHGALAEVARKHPQAIVCLLSALRLHELTTQSPFEVWLAIPNKARA
                     PKMDYPPLRIVRFSGAALTEGVEEHLIDGVPVRVTNVARTVADCFKYRNKIGLDVALEA
                     LREAWKAKRVGMDELWQFAKLCRVANVMRPYLESLT"
     CDS             906..1832
                     /ID="WDFLIPAM_CDS_0003"
                     /transl_table=11
                     /phrog="No_PHROGs_HMM"
                     /top_hit="No_MMseqs_PHROG_hit"
                     /locus_tag="WDFLIPAM_CDS_0003"
                     /function="unknown function"
                     /product="hypothetical protein"
                     /source="PHANOTATE_1.5.1"
                     /score="-199.3493917846585"
                     /phase="0"
                     /translation="VSSRNTAASVRARLLAKARTDKQDFNLVLTRYALERLLYRLSVSA
                     HADHFLLKGALLFDLWFDIPHRPTRDADLLGFGSAEIPHVEAAFRDICAVELDDGIRFQ
                     ADSVHAEEIRKEANYSGVRVTLIGLLDGARCHVQVDVGFGDAVTPGPETVDYPVMLSEM
                     PAPKLRAYPRYTVIAEKLEALVSLGIANSRMKDYFDLWILSRYTDFDGKLLCNAIHATF
                     ERRRTPLPDGVPFGLSDEFAQDRQKQTQWQAFLRKNALVELALSEVVAGLRAFLSVPLD
```

ALQQGAAFPQTWLAGNGWTSSSEEFSR"
CDS 1781..2317
/ID="WDFLIPAM_CDS_0004"
/transl_table=11
/phrog="No_PHROGs_HMM"
/top_hit="No_MMseqs_PHROG_hit"
/locus_tag="WDFLIPAM_CDS_0004"
/function="unknown function"
/product="hypothetical protein"
/source="PHANOTATE_1.5.1"
/score="-99.90945395584541"
/phase="0"
/translation="MACRKWVDVEQRGIQPMTWNPDLLLEDFRRVAGMAGVSLAPDAIA
IERRSAPHVPPKSLPLGKMAVYVFSFGQHVLKVGKVGPNSAARYTAQHYNAGSAKSTLA
ASLIKHGERIGVAGLDETNVAGWIREHTDRVNFILDAPLGVHVLNLLEAFLQCRLRPEF
EGFASQRIDREGGQE"
CDS 2314..2901
/ID="WDFLIPAM_CDS_0005"
/transl_table=11
/phrog="No_PHROGs_HMM"
/top_hit="No_MMseqs_PHROG_hit"
/locus_tag="WDFLIPAM_CDS_0005"
/function="unknown function"
/product="hypothetical protein"
/source="PHANOTATE_1.5.1"
/score="-291.76814097679465"
/phase="0"
/translation="MKEDILEQMVDEYLQHKGYFTRHNIKFRPAGDHAEYDTRQDAVHS
DIDVIGIHPRLDGARRVMVVSCKSWQGGFRPEYWIDAIAKNKVVSGREAWRGFRELTRE
KWAAAFRMTVAELTGSSSFTYITAVTKVIGSRSVWEDNAAFREHLGGNPIEILTFGDML
KELFPFIDTTPASSEVGRVLQLIKASGWSLDK"
CDS complement(2934..3437)
/ID="WDFLIPAM_CDS_0006"
/transl_table=11
/phrog="14161"
/top_hit="No_MMseqs_PHROG_hit"
/locus_tag="WDFLIPAM_CDS_0006"
/function="other"
/product="recombinase"
/source="PHANOTATE_1.5.1"
/score="-59.66831527516468"
/phase="0"
/translation="MNEILIDKTGQPDVITASDGSLTVTVPIRIKRRGSRKAVTLPDGG
AVQPRPWDDTPTPIQLALARGHRWLAMVDKFAGSEFGRRSRPRRGEPQGRGEQSGEART
LSEVAERERMDRAYVSRMVNLTTLAPDIVAAILDETLPPEVTLFDLASGTPLLWEEQRA
IIDR"
CDS complement(3434..4114)
/ID="WDFLIPAM_CDS_0007"
/transl_table=11
/phrog="95"
/top_hit="No_MMseqs_PHROG_hit"
/locus_tag="WDFLIPAM_CDS_0007"
/function="integration and excision"

/product="integrase"
/source="PHANOTATE_1.5.1"
/score="-13.551640360459562"
/phase="0"
/translation="VREGKPIDKSLVYKLLNNRVYLGEIRHRDQWYAGEHPTIVERKLW
DAVQAILAQNSRVRGNNTRARVPFLLKGIVVGIDGRALTPWSTRKKNGRIYRYYLPTRE
NKEHAGASGLPRLPAGELEAAVLEQMRRVLRAPDMVAGVAERAARLDPSLDEAQVAVAM
TRLDAIWDQLFPAEQQRIVRLLIDKVIVSPNDIEVRFRPNGIEVLALELRPEPAPETLE
EAVA"
CDS complement(4225..4791)
/ID="WDFLIPAM_CDS_0008"
/transl_table=11
/phrog="95"
/top_hit="No_MMseqs_PHROG_hit"
/locus_tag="WDFLIPAM_CDS_0008"
/function="integration and excision"
/product="integrase"
/source="PHANOTATE_1.5.1"
/score="-59.27809047916872"
/phase="0"
/translation="MSEALKRRLRCAVYTRKSTDEGLDQEYNSIDAQRDAGHAYIASQR
AEGWIPVADDYDDPAFSGGNMDRPALKRLLADIEAGRIDIVVVYKIDRLTRSLTDFSRM
IDVFERHGVSFVSVTQQFNTTTSMGRLMLNILLSFAQFEREVTGERIRDKIAASKRKGM
WMGGCRRSATTSRTGDWCPTSARPS"
CDS complement(4788..5285)
/ID="WDFLIPAM_CDS_0009"
/transl_table=11
/phrog="11806"
/top_hit="No_MMseqs_PHROG_hit"
/locus_tag="WDFLIPAM_CDS_0009"
/function="unknown function"
/product="hypothetical protein"
/source="PHANOTATE_1.5.1"
/score="-117.30753832234828"
/phase="0"
/translation="MKASIVPPTPPSVVARIAGLPDLSIEEMRALWRELFGSENPTPNR
QFMERRIAYKLQEIEFRKVDPNLLERNKRRIKALLETGKARKLDRDIRLMPGTVLTREY
QGVEHRVTVAQDGQYEFEGRRYPSLSMIAREITGTRWSGPLFFGVKAPAKQKHPKKQGG
RR"
CDS complement(5282..5500)
/ID="WDFLIPAM_CDS_0010"
/transl_table=11
/phrog="No_PHROGs_HMM"
/top_hit="No_MMseqs_PHROG_hit"
/locus_tag="WDFLIPAM_CDS_0010"
/function="unknown function"
/product="hypothetical protein"
/source="PHANOTATE_1.5.1"
/score="-4.402026768301565"
/phase="0"
/translation="MCTINHLPPERMTPEQRRREVASLLAHGLVRLREAGFAQSAGGPA
ESEFELGFSGHQRLHSHPVNNTLEEAP"
CDS complement(5628..6842)

/ID="WDFLIPAM_CDS_0011"
/transl_table=11
/phrog="No_PHROGs_HMM"
/top_hit="No_MMseqs_PHROG_hit"
/locus_tag="WDFLIPAM_CDS_0011"
/function="unknown function"
/product="hypothetical protein"
/source="PHANOTATE_1.5.1"
/score="-47316.89926945367"
/phase="0"
/translation="MSAHKWQFASRFRRHAFGWRSDTPVQRIKEAIAEIKQVARKEPVL
AAEGAITLLEKLSPALEQVDSSSGALGSAVNKAIETLVPLIAKADVDTPLRQRWLERLW
QALQDDEMPYIELLGDYWGELCVTPELASHWADEFLPLVEHVWSRTASGHGFFKGTSAC
LASLYAAGRHQELLALIDKAPFKWWHDRRWGVKALSAMGKKAEAIRYAEDSRGLNDPGW
QIAQDCEAILLSSGLLDEAYRRYALEANQGTTHLATFRAISKKYPNKQPEEILRDLIAS
TPRAEGKWFAAAKDAGLFVVAAELATRSPTDPRTLTRAARDFAEKHPDFALAAGLAALH
WISHGYGYEITGVDVLDAYSAVTQAAHGAGVPTQRINEQIREMTAGTQPGNSLMRTMLA
RHLSE"
CDS complement(6860..7771)
/ID="WDFLIPAM_CDS_0012"
/transl_table=11
/phrog="1914"
/top_hit="No_MMseqs_PHROG_hit"
/locus_tag="WDFLIPAM_CDS_0012"
/function="unknown function"
/product="hypothetical protein"
/source="PHANOTATE_1.5.1"
/score="-5409.908818919105"
/phase="0"
/translation="MTEAKKPMAEANRISAMLNAVLGTDRFPVKVDELAIEYSRQCFAD
SPVDKVRGEALDGFDGMLAANKTRSKWLILYNSATRSEGRKRFTIAHEFGHYILHRHQK
DRFECGDDDIETGNDDECDIEAEADLFASTLLMPLDDFRRQVDGQPVSFDLLGHCADRY
GVSLTAAALRWTEIAPKRAVLIASRDDHMLWAKSNKAALRSGAYFATRRNIIELPRDAL
AHSSNALDIGQQQTTAARHWFPREPETMMVNEMIRDAGQYEYTLTLLLLPEAEWQGAHH
DDEESEEDTYDRFIRNGQYPVR"
CDS complement(7768..8088)
/ID="WDFLIPAM_CDS_0013"
/transl_table=11
/phrog="8"
/top_hit="No_MMseqs_PHROG_hit"
/locus_tag="WDFLIPAM_CDS_0013"
/function="transcription regulation"
/product="transcriptional regulator"
/source="PHANOTATE_1.5.1"
/score="-13.192453828501243"
/phase="0"
/translation="VPSPLGDKIRSLRKQKKLSLEQLAELTDSSKSYIWELENKDEPKP
SAEKIGKIAAVLEVTTEFLLTESATTPDEEVLDEAFFRKYKNMSEPDKKKIRKILDAWE
DE"
CDS 8123..8257
/ID="WDFLIPAM_CDS_0014"
/transl_table=11
/phrog="No_PHROGs_HMM"

/top_hit="No_MMseqs_PHROG_hit"
/locus_tag="WDFLIPAM_CDS_0014"
/function="unknown function"
/product="hypothetical protein"
/source="PHANOTATE_1.5.1"
/score="-1.7547772467397205"
/phase="0"
/translation="MLPLRRTISYLLDKPGISPRQSETFGTPNVRVLGGMWRRRASSV"
CDS 8275..9498
/ID="WDFLIPAM_CDS_0015"
/transl_table=11
/phrog="37634"
/top_hit="No_MMseqs_PHROG_hit"
/locus_tag="WDFLIPAM_CDS_0015"
/function="unknown function"
/product="hypothetical protein"
/source="PHANOTATE_1.5.1"
/score="-36917.29241903252"
/phase="0"
/translation="MTAFNPRHLARQVPATTWQAYLSSRSIAIPESFDWNAEEKAFSDA
LIALLEELEPDQQALLHAELRHVYALATQKGIDAILNASDNDVAIREDFGQLRNHAERA
MWVLVNWPQTFMTAEALLQFDLGVGKRSWKRQAIKVTEPVSREAADIEGLQAALSEVLS
KRKGPRRACHVDVCDRHLDGGVQISVYVEDDPNDLVEFVEEGMRRRTTRPATNLALVYF
PASGIVDTVGRGGAKVHQPLVTLFARHLLKQEVKPEAVKQPMFYLNRLRHGLDLPEDSD
IDLAAHGIDRIRLRRARLRSTRAPICDFWVGVPADQAEHCVLAASSAHLKDHDLFRGPF
NIVEALISIYFAPAEVGKRGRVLNIDIKQSGISNLQDMAEEDAKLAERLLRAWRVSEPT
EVELALVA"
CDS 9606..10463
/ID="WDFLIPAM_CDS_0016"
/transl_table=11
/phrog="No_PHROGs_HMM"
/top_hit="No_MMseqs_PHROG_hit"
/locus_tag="WDFLIPAM_CDS_0016"
/function="unknown function"
/product="hypothetical protein"
/source="PHANOTATE_1.5.1"
/score="-586.9271979993363"
/phase="0"
/translation="MDAYRHLLDLGALGAGDGVGSSILCPWCGMGDLEGIRFEQGGRQG
YCTDCGWVDLASDQVTRLRVDITRIVRWLASALGLAGRYQHEEMVPTALWRLGEIEHRR
KRRTIFFGRRLSDPALTPIIEARLRTACAPGCGVLITTTPDVPQAIQAAGHLVVPLRAV
AHLRKAGFVIENLDAYLDGSAGIGERDSETSLRLMHSGRIAVIDGERHALSPQVYGFLS
VLAQGGGDPVHKRTIADALEIDVDKCKGADICKRHKAVYRTFVAHDNDGHYWLKPEFVN
DQRR"
CDS 10463..10753
/ID="WDFLIPAM_CDS_0017"
/transl_table=11
/phrog="3220"
/top_hit="No_MMseqs_PHROG_hit"
/locus_tag="WDFLIPAM_CDS_0017"
/function="unknown function"
/product="hypothetical protein"
/source="PHANOTATE_1.5.1"

/score="-55.11880150717688"
/phase="0"
/translation="MHPTKRNPFIRQFNFDLDEVNDMSGKNQWVVRNGDKWGVRGEGND
RLTSVHDTQQEAFERARDIARNQGSEVLIQGEDGKIRERNSYGKDPFPPPG"
CDS 10837..10932
/ID="WDFLIPAM_CDS_0018"
/transl_table=11
/phrog="No_PHROGs_HMM"
/top_hit="No_MMseqs_PHROG_hit"
/locus_tag="WDFLIPAM_CDS_0018"
/function="unknown function"
/product="hypothetical protein"
/source="PHANOTATE_1.5.1"
/score="-0.0650834655371287"
/phase="0"
/translation="LHDLPAHPARFARRFAHPLKPRMTSRFRNNP"
CDS 10943..11155
/ID="WDFLIPAM_CDS_0019"
/transl_table=11
/phrog="66"
/top_hit="No_MMseqs_PHROG_hit"
/locus_tag="WDFLIPAM_CDS_0019"
/function="integration and excision"
/product="excisionase and transcriptional regulator"
/source="PHANOTATE_1.5.1"
/score="-1.714845947089749"
/phase="0"
/translation="VSIKHLNQRQLADRWGVSEATLERWRSEGIGPVFLKIQGRVLYRV
EDVESYEADCLRKSTSERVGAGGVA"
CDS 11152..11622
/ID="WDFLIPAM_CDS_0020"
/transl_table=11
/phrog="2480"
/top_hit="No_MMseqs_PHROG_hit"
/locus_tag="WDFLIPAM_CDS_0020"
/function="unknown function"
/product="hypothetical protein"
/source="PHANOTATE_1.5.1"
/score="-16.82610118692036"
/phase="0"
/translation="MSRLTPDQVLATPAGELAALQSDALFQLKNDAADLLSAAKAIVEH
LERALELKYADRAQALRLAAGKDTGVVHFDDGRVRVTADLPKRVEWDQKRLAEIVRRIA
EGGEDPAEYVETAYRISETKFNAWPESLKSAFAPARTLKTGKPGFRLALLEE"
CDS 11628..11909
/ID="WDFLIPAM_CDS_0021"
/transl_table=11
/phrog="36009"
/top_hit="No_MMseqs_PHROG_hit"
/locus_tag="WDFLIPAM_CDS_0021"
/function="unknown function"
/product="hypothetical protein"
/source="PHANOTATE_1.5.1"
/score="-21.5142212681002"

/phase="0"
/translation="MFKNRTLLDKLKKQHPYLLESLPERIERNGADVAIEDATLDQIAF
AVIALENEVRPISRRMNALRELYDLARKHGALGAHRIGDAFADKGGRS"
CDS 11906..12763
/ID="WDFLIPAM_CDS_0022"
/transl_table=11
/phrog="124"
/top_hit="No_MMseqs_PHROG_hit"
/locus_tag="WDFLIPAM_CDS_0022"
/function="DNA"
/function=" RNA and nucleotide metabolism"
/product="Sak4-like ssDNA annealing protein"
/source="PHANOTATE_1.5.1"
/score="-403.94945894518526"
/phase="0"
/translation="MSLPIISADQRLAEKRGVKGVLVGKSGIGKTSQLWTLAPEATLFF
DLEAGDLAVEGWAGDTIRPRTWQECRDFAVFIGGPNPALREDQPFSQAHFDAVCARFGD
PTVLDRYDTVFVDSITVAGRLCLQWCKGQPQAYSEKTGKPDTRGAYGLMGQEMIAWLTH
LQHTRGKNVWFVGILDERLDDFNRRVFQLQIDGSKTGLELPGIVDEVVTLAELKADDGT
AYRAFVCHTLNPWGYPAKDRSGRLDQIEEPHLGRLMAKIAGPARPALERLDFARPAPSE
PVQQ"
CDS 12776..13372
/ID="WDFLIPAM_CDS_0023"
/transl_table=11
/phrog="2300"
/top_hit="No_MMseqs_PHROG_hit"
/locus_tag="WDFLIPAM_CDS_0023"
/function="unknown function"
/product="hypothetical protein"
/source="PHANOTATE_1.5.1"
/score="-151.1342025420588"
/phase="0"
/translation="MSYFDFNDANEQSSFDLIPKGTLVHVRMTIRPGGFDDASQGWTGG
YATRNDNTGSVYLNCEFVVMEGEYARRKLWSLIGLYSPKGPEWANMGRAFVKAILNSAR
GVHPGDASPQAQNARRIAGFADLDGIEFLGKVDWEKDQNGQDKAVIKQAIQPDHKEYAA
LMGNARPSAPAPTTPNAYAQATGRAPVTGRPSWAQ"
CDS 13387..15063
/ID="WDFLIPAM_CDS_0024"
/transl_table=11
/phrog="16"
/top_hit="No_MMseqs_PHROG_hit"
/locus_tag="WDFLIPAM_CDS_0024"
/function="DNA"
/function=" RNA and nucleotide metabolism"
/product="DNA helicase"
/source="PHANOTATE_1.5.1"
/score="-119539.80873347308"
/phase="0"
/translation="MMLRPRQALLVERSLAALHQHGNTLAIGPTGSGKTIMLSAVAGGV
LEEPDAKSCILAHRDELTAQNREKFGRVNPGLTTSVFDAKEKSWAGRATFAMVQTLSRD
AHLDAMPTLDLLVVDEAHHAASPSYRRVIDRVLSRNPRALIFGATATPARSDGKGLREV
FSNVADQIHLGELIASGHLVPPHTFVIDVGAQSALAQVRRTATDFDMTEVEAILNRTPI
TDAVIRHWREKAGDRKTIVFCSTVAHAQCVADAFVAAGTRAVLIHGELSDAERKARLAE

YETGDAQVVVNVAVLTEGYDYTPTSCVVLLRPSSHKSTLTQMIGRGLRTVDPAEHPGVV
KTDCIVLDFGTATLMHGSLEQEANLDGHQHQGEAPTKECPSCEATVPLGCRECPLCGFE
WTIDPAEQAEAMDDFVMTEIDLLKRSNFRWCDLFGCDDALMATGFGAWGGIFFLNGRWH
AVGGGKDLQPRLLAVGDRTVCQACADDWLNENESLDTAHKTRRWLNEPPTEKQLRYLPQ
AMRSDFGLTRYQASALLAFQFNKSSIQRLVLAANDEHRRAA"

CDS 15168..15416
/ID="WDFLIPAM_CDS_0025"
/transl_table=11
/phrog="21342"
/top_hit="No_MMseqs_PHROG_hit"
/locus_tag="WDFLIPAM_CDS_0025"
/function="unknown function"
/product="hypothetical protein"
/source="PHANOTATE_1.5.1"
/score="-7.581415532789847"
/phase="0"
/translation="MRCQDIHLRRLKAGGGVVIDPTHNEKAAMEAVLPHLGEYVAAIGM
DRPLSAYSRKEVLQLVDVVLTAYFDNLREITPDDVPF"

CDS 15428..16195
/ID="WDFLIPAM_CDS_0026"
/transl_table=11
/phrog="1990"
/top_hit="No_MMseqs_PHROG_hit"
/locus_tag="WDFLIPAM_CDS_0026"
/function="DNA"
/function=" RNA and nucleotide metabolism"
/product="Cas4-domain exonuclease"
/source="PHANOTATE_1.5.1"
/score="-71.0324955642194"
/phase="0"
/translation="MLDYNHRPKFHERVGAVIDEALARQRASQTPRRYLGASRLGVACE
RALQYEYAQAAVDPGRELPGRVLRVFEVGHALEDLAIRWLRLAGFELYTRKADGGQFGF
SVAGGRIQGHVDGILAAGPADIELDFSMRWPALWECKTMNDKSWRETVKQGVARAKPIY
AAQLAVYQAYMEGTVPGIASNPALFTAINKDSEEIWFELVPFDGGLAQRMSDRAVRVIT
ATEAGELLPRHATTPTHFECKSCPWQDRCWRPA"

CDS 16195..18381
/ID="WDFLIPAM_CDS_0027"
/transl_table=11
/phrog="13004"
/top_hit="No_MMseqs_PHROG_hit"
/locus_tag="WDFLIPAM_CDS_0027"
/function="unknown function"
/product="hypothetical protein"
/source="PHANOTATE_1.5.1"
/score="-794956.1334237866"
/phase="0"
/translation="MADVIWLDYNDAPEQHGETPSDTEALRRGLLDRLEAVLLYLFPQG
RIRGGRFYVGDVDGNRGKSLVVELEGERRGLWKDFASDEGGDVIDLWARSRGLSARHDF
PRLADEIRRWLGIATPTRPPEPRSKIRSAPVDELGPYTAKWDYLTADGRLIACVYRYDP
PTGKEYRPWDVRARMWRAPDPRPLYNLPAVAEAREVVLVEGEKAACALIGTGITATTAM
NGARAPIDKTDWTPLAGKHVLVWPDRDAPGWDYAENAARACVTAGAASVAILVPPMDKP
DKWDAADAVAEGFDCAAFVHEGERRVVKTAAPGLPTFTLGMLLDDDSPLPADLVAPRVL
TPGGMLVFGGAPKVGKSDFLLAWLTHMAAGATFLAMTPPRPLRVFYLQAEVQYHYLRER

VKEIRLPPSRLGAARANFVATPQLRLVLDDAGINQVIPAIAQAFGGEPPDIIAIDPIRN
VFDGGDAGGENDNAAMLFFLSQRVERLRDAVNPDAGVVLTHHTKKLGKKQFEEDPFQAL
AGAGSLRGYYTTGMLLFRPDETRTTRQLIFELRNGAAIPSMHVDKIRGEWREVDANERL
VMKDYGERLDAERRRKRDAILQILFDEAAQGRCYTANQFAESFEGKAGLGGERTIRERL
SALSTQGYIKYFRNAQDYGLPPARTKFGYLCVEAMVLRSVVGEPDPDTGEVSLRELPVL
PTHFKCPQSGAALPVENPEVWVYQDDLNDPEEPA"
CDS 18378..19157
/ID="WDFLIPAM_CDS_0028"
/transl_table=11
/phrog="159"
/top_hit="No_MMseqs_PHROG_hit"
/locus_tag="WDFLIPAM_CDS_0028"
/function="DNA"
/function=" RNA and nucleotide metabolism"
/product="RuvC-like Holliday junction resolvase"
/source="PHANOTATE_1.5.1"
/score="-327.7821329333364"
/phase="0"
/translation="MNTHSQVGKTAANFPPIFRDVGKLANACQLESHTDQRVRELSADW
QVGKAANLPTSPNPRGCWVSGDFQVGENSLPPTGGEEHAVSSPPTRRGLPGIDGRDRGP
GTAILSLDLGTRTGWALLGRDGSITSGSESFKPRRFEGGGMRYLRFKRWLTEVKPSADG
LDAVYFEEVRRHAGVDAAHAYGGFMAQLTAWCEHHGIPYQGVPVGTIKKHATGKGNAGK
QEMVAAMQALGFRPEDDNEADALALLMWAIATQEVPA"
CDS 19154..19369
/ID="WDFLIPAM_CDS_0029"
/transl_table=11
/phrog="19772"
/top_hit="No_MMseqs_PHROG_hit"
/locus_tag="WDFLIPAM_CDS_0029"
/function="unknown function"
/product="hypothetical protein"
/source="PHANOTATE_1.5.1"
/score="-6.448207234712385"
/phase="0"
/translation="MNAPSPNYRCPLGRLQPTRPDVDAIKRDGWRDQGILVVSLDDERL
DWIERELVKRIGERLYGRQGDGHVER"
CDS 19356..19751
/ID="WDFLIPAM_CDS_0030"
/transl_table=11
/phrog="6836"
/top_hit="No_MMseqs_PHROG_hit"
/locus_tag="WDFLIPAM_CDS_0030"
/function="unknown function"
/product="hypothetical protein"
/source="PHANOTATE_1.5.1"
/score="-12.38370765955751"
/phase="0"
/translation="MWSVDDVAERFREAAQTARRLPPVRVQGYFNTWPAILRQPWETYS
GDDVLYRFPPDPAAIDRMEETMRWVLWLTEEQRHLVWMRAERGWREICRRFGCDRTTA
WRRWQKALDIVACRLNEQTRRNVASLS"
CDS 19788..20015
/ID="WDFLIPAM_CDS_0031"
/transl_table=11

/phrog="No_PHROGs_HMM"
/top_hit="No_MMseqs_PHROG_hit"
/locus_tag="WDFLIPAM_CDS_0031"
/function="unknown function"
/product="hypothetical protein"
/source="PHANOTATE_1.5.1"
/score="-3.055341406091592"
/phase="0"
/translation="MRADAERCRFRAILDVQHFTRFCARISLTSREKHVRRPRSDPWPS
SFLPSRSDPPSAMGPSWPKSHAGGASAMLF"
CDS 20127..21623
/ID="WDFLIPAM_CDS_0032"
/transl_table=11
/phrog="498"
/top_hit="No_MMseqs_PHROG_hit"
/locus_tag="WDFLIPAM_CDS_0032"
/function="other"
/product="DNA methyltransferase"
/source="PHANOTATE_1.5.1"
/score="-1644.1074698172597"
/phase="0"
/translation="VHSPIHITSPPTVLRRRAFHFEVPILNTLNVEYRKVETLIPYARN
PRTHSDEQVARIAASIAEFGWTNPILVDGDHGVIAGHGRLLAARKLGLTEVPVIELAHL
TPAQKRAYVIADNRLALDAGWDEAMLALEFAELADAGFDLDLTGFSASEIEGLLDHIEE
TEPSADEDERAPEGDADEDDVTPPTVAVTRPGDLWLLGEHRLLCADSSDAAAVARLING
ERAHLLFTSPPYANQRDYTTGGITDWNALMQGVFGAARSALHEDAQILVNLGLVHRDGE
WQPYWDGWIEWMRTQGWRRFGWYVWDQSVTVPGDWAGRLAPRHEFVFHFNRQARKPNKI
VPCKWAGHETHLRADGSSTAMRAKDGKVGEWNHAGQPTQEFRIPDSVVEVTRQRGRIGD
GIDHPAVFPLGLPKFFIEAYTDVGEIVFEPFSGGGTTLLAGQLTDRRVRAIELAPEYVD
VALRRWLQHHPGMEPVLAATGQPFAEVSAERLGETAEAAA"
CDS 21620..22885
/ID="WDFLIPAM_CDS_0033"
/transl_table=11
/phrog="498"
/top_hit="No_MMseqs_PHROG_hit"
/locus_tag="WDFLIPAM_CDS_0033"
/function="other"
/product="DNA methyltransferase"
/source="PHANOTATE_1.5.1"
/score="-757.9114792838658"
/phase="0"
/translation="VNWFAERIEHWPIDKLLPYARNARQHSDEQIAQIAASIAEFGFVN
PCLVGADGVLVAGHGRLAAARKLGLSTVPVVVLDHLTPTQRRALVLADNRLAELSTWDD
VLLRIELEALQDEGFDLDLTGFDADALAELLAGEEPEHEGQTEDDAVPEIPEEPVSKPG
DVWRLGPHRLVCGDATAAEAYAQLFPDGERADMVFTDPPYNVNYANSAKDKLRGKHRPI
LNDALGAGFHDFLYDALSLLVAHTRGAIYVAMSSSELDTLQAAFRSAGGHWSTFIIWAK
NTFTLGRADYQRQYEPILYGWPEGAERHWCGDRDQGDVWQIKKPQKNDLHPTMKPVELV
ERAIRNSSRPGDVVLDPFGGSGTTLIAAEKSGRVARLIELDPKYADVIVRRWQDWTGKQ
TTRESDGLAFDQAATSSSTISQ"
CDS complement(22849..23217)
/ID="WDFLIPAM_CDS_0034"
/transl_table=11
/phrog="8020"

/top_hit="No_MMseqs_PHROG_hit"
/locus_tag="WDFLIPAM_CDS_0034"
/function="unknown function"
/product="hypothetical protein"
/source="PHANOTATE_1.5.1"
/score="-21.517031702400583"
/phase="0"
/translation="MTTAKTIPATRNEAWGFWGTMDAHAQAAWPIAMNAISDATGQPFE
AVRAFLDSRHGRHFADEVLNRKHAGHALHDAIRAATRQWMEWTIGRRTSKDYGIPRGLP
YLTGFVIHCEIVEEEVAA"
CDS complement(23289..23501)
/ID="WDFLIPAM_CDS_0035"
/transl_table=11
/phrog="No_PHROGs_HMM"
/top_hit="No_MMseqs_PHROG_hit"
/locus_tag="WDFLIPAM_CDS_0035"
/function="unknown function"
/product="hypothetical protein"
/source="PHANOTATE_1.5.1"
/score="-6.595476621149975"
/phase="0"
/translation="MKPTRAILTHSNYDADDYAYLTAKGWSDDEILARWSEEAAHGNGP
CHWESASARAKLAAVTGRQQTTRDD"
CDS complement(23600..24115)
/ID="WDFLIPAM_CDS_0036"
/transl_table=11
/phrog="10315"
/top_hit="No_MMseqs_PHROG_hit"
/locus_tag="WDFLIPAM_CDS_0036"
/function="unknown function"
/product="hypothetical protein"
/source="PHANOTATE_1.5.1"
/score="-44.49267011864057"
/phase="0"
/translation="MSTIQLTPAQHAILAYAVEHTGGKIEWFPDNVKGGARKKVLDGLC
NRALITTIGPDWFVAADGYEALGRPRPAPAPVEANTDLEAEVAAAEATWAPQRAETKPR
TRENSKQAQVIAMLRRPEGATVRQICELTGWQAHTVRGTFANAFKKKLGLTITSDKPEG
GERIYRIA"
CDS complement(24197..24385)
/ID="WDFLIPAM_CDS_0037"
/transl_table=11
/phrog="2555"
/top_hit="No_MMseqs_PHROG_hit"
/locus_tag="WDFLIPAM_CDS_0037"
/function="unknown function"
/product="hypothetical protein"
/source="PHANOTATE_1.5.1"
/score="-8.601188623688218"
/phase="0"
/translation="MTEQAEKDIDRQLQQIALDHLFIDTLETRNSDRLDFHEVSVWAVK
SALMAAYQAGRQAARQG"
CDS complement(24382..24492)
/ID="WDFLIPAM_CDS_0038"

/transl_table=11
/phrog="No_PHROGs_HMM"
/top_hit="No_MMseqs_PHROG_hit"
/locus_tag="WDFLIPAM_CDS_0038"
/function="unknown function"
/product="hypothetical protein"
/source="PHANOTATE_1.5.1"
/score="-2.1388596675153715"
/phase="0"
/translation="MVSIKHWRRPARIPLGFAREQRVHVITINDTPRRST"
CDS 24482..25021
/ID="WDFLIPAM_CDS_0039"
/transl_table=11
/phrog="57"
/top_hit="No_MMseqs_PHROG_hit"
/locus_tag="WDFLIPAM_CDS_0039"
/function="head and packaging"
/product="terminase small subunit"
/source="PHANOTATE_1.5.1"
/score="-24.430367755907913"
/phase="0"
/translation="METMGLSIRAYARHRGVSDTAVHKAIRAGRITPEADGTIDPDKAD
RDWARNSEPPKEGTGAKAAKVRVSDDPAPNLAAGLPAGGTSLLQARTVNEVVKAQTNKV
RLARLKGELVDRNQAIAHVFKLARTERDAWLNWPARISAQMAARLGMDAHTLHVALEAA
VREHLAELGELKVRVD"
CDS 25095..27059
/ID="WDFLIPAM_CDS_0040"
/transl_table=11
/phrog="15"
/top_hit="No_MMseqs_PHROG_hit"
/locus_tag="WDFLIPAM_CDS_0040"
/function="head and packaging"
/product="terminase large subunit"
/source="PHANOTATE_1.5.1"
/score="-239643.5004742173"
/phase="0"
/translation="MDDFVYEGFDAIERAWREGLTPDPLLSVSEWADRHRMLSSKSSAE
PGRWRTSRTPYLKAIMDCLSPTSPVERVVFMKGAQVGATETGSNWIGYVIHHAPGPMMA
VWPTVEMAKRNSKQRIDPLIEESPVLSELIAPARSRDSGNTILAKEFRGGVLVMTGANS
AVGLRSMPVRYLFLDEVDGYPLDVEGEGDAISLAEARTRTFARRKIFIVSTPTIAGASA
VEREYEASDQRRYFVPCPHCSHRQWLRFEQLRWERGRPETAAYVCESCDQPIAEHHKTW
MLEHGEWRAMAPENGIKTAGFHLSSLYSPVGWRSWRDIASAWESAVSKESGSAAAIKTF
KNTELGETWVEEGEAPDWQRLIERREDYRIGTVPMGGLLLVGGADVQKDRIEASIWAFG
RGKASWLVEHRVLMGDTARDAVWKRLSELIDETWTHDSGNQVPLARFALDTGFATQEAY
AFVRACRDSRLMAIKGVPRGAALIGTPTAVDVSQGGKKLRRGIKVYSVAVGLAKLEFYN
NLRKSADVADDGTTPIYPPGFVHLPKVDAEYVQQLCAEQLITRRDRNGFPVREWQKLRE
RNEALDCYVYARAAAAASGLDRFEERHWRELERQLGIAAPPEQDEHTVTAEDAPDSGGV
VVSGRRIRRRSVVKSRWMS"
CDS 27198..29210
/ID="WDFLIPAM_CDS_0041"
/transl_table=11
/phrog="21"
/top_hit="No_MMseqs_PHROG_hit"

/locus_tag="WDFLIPAM_CDS_0041"
/function="head and packaging"
/product="portal protein"
/source="PHANOTATE_1.5.1"
/score="-10043.946880824184"
/phase="0"
/translation="MAPSPPTRSIWVRSTATHCCCLVRAASPRSRKRWRSRARSTACVG
CRRRASIRTRTWPGTSRRTAATTSPSRADPSDASRASPSRTATPPRPSWSSSWPHWRVS
DHGLHSGRSRSAGAGIGARRAARHLRRQDGGVPLGGRVARGAARGRCGARPRGGPTQAA
PDPGHHGQGALMGFWSRIKLAFGTTPTYDGVGSGRRALAWMPGNPGAVAALLTTQTELR
AKSRDLVRRNAWAAAGIEAFVANAIGTGIKPQSLIEDPGLRETIQALWRDWTEEADAQG
LTDFYGLQALACRAMLEGGEALVRLRYRRPDDGLAVALQIQVLEPEHLPVTLNLLADNG
NVIRAGIEFDRLGRRVAYHLYRSHPEDGLLAPMSGGGGMETVRVPAAEIVHLFRPLRPG
QIRGEPWLARALVKLNELDQYDDAELVRKKTAAMFAGFVTRDQPEDALMGEGPADASGV
ALAGLEPGTLQILEPGEDVKFSQPADVGGSYAEFMRQQFRAVAAAMGVTYEQLTGDLTQ
VNYSSIRAGLLEFRRRCEAIQHGVIVHQLCRPVWRAWMAQAVLEGWLKLPGFARDAARR
RAWLACKWIPQGWQWVDPQKEFNAMLTAIRAGLLSRSEAVSSFGYDAEDVDREIAADNA
RADALGLVFDSDPRHDRNPPAAASDPVPPQDIQDL"
CDS 29110..30450
/ID="WDFLIPAM_CDS_0042"
/transl_table=11
/phrog="53"
/top_hit="No_MMseqs_PHROG_hit"
/locus_tag="WDFLIPAM_CDS_0042"
/function="head and packaging"
/product="head maturation protease"
/source="PHANOTATE_1.5.1"
/score="-496.2649833476468"
/phase="0"
/translation="MRSASSSIPTRATTATHPRRPPTRCRRKTSRTSDMQLVHLASRLY
GTPLLIARSKLDVILSVLGPRIGLPDVEAAVPAVMPASPEVAAPPGITVIPIHGTLVRR
TLGLEAASGLMSYGEIGARLDAAIADPAVSGILLDVDSPGGEAGGVFELAERIRAADAV
KPVWAIAADSAFSAAYAIACAASHLAVTRTGGVGSVGVIAMHVDQSVRDAQQGYRYTAI
TAGRHKNDFSPHEPLDQEAAERLQAEVDRLYGLFVGHVAAMRGLDPDAVRATEAGIFFG
EQAIGSRLADAVASRDQLLAVFATFLNPQGRSRNPAPRTRPGSAYAKQENDPMQSTDPT
PETPETTLVAAAASAAPVPETPATAADRREAVAIAELCQLGGCPERTAEFLAAGLSETD
VRRALLAARAHGPEIGSAIHPDAHAAHRASPEHNPLIKAVKHLTGKE"
CDS 30454..30828
/ID="WDFLIPAM_CDS_0043"
/transl_table=11
/phrog="49"
/top_hit="No_MMseqs_PHROG_hit"
/locus_tag="WDFLIPAM_CDS_0043"
/function="head and packaging"
/product="head decoration"
/source="PHANOTATE_1.5.1"
/score="-13.876676188119088"
/phase="0"
/translation="MPTLVEPMNLGDLLKYEAPNLYSRDLATVAAGQNLVLGSVVGRET
ATNKLKALDPAATDGTELPAGVLIVDADATAADLDTVIVARHAIVARHALVWPAGITPA
QQSAAIAALEARGILVREGA"
CDS 30831..31835
/ID="WDFLIPAM_CDS_0044"

/transl_table=11
/phrog="29"
/top_hit="No_MMseqs_PHROG_hit"
/locus_tag="WDFLIPAM_CDS_0044"
/function="head and packaging"
/product="major head protein"
/source="PHANOTATE_1.5.1"
/score="-1242.9328509144225"
/phase="0"
/translation="MQNPFSNPAFSMASLTAAINLIPNRYGRLETLDLFPIKPVRTRQV
VVEEMHGVLNLLPTLPPGSPGTVGKRGKRAMRAFVVPHIPHDDVVLPEEVQGIRSFGQE
TETESVAGVLARHLETMRNKHAITLEHLRMGALKGEILDADATPLVNLYTEFGITPKTV
NFALGNANTNVKGKCAEVLRHIEDNLSGEFSTGVHCLCSPEFFDALTGHAKVEEAYKNW
QQGAVLINDMRRGFTFGGITFEEYRGQASDASGTTRRFIAAGEAHAFPLGTVDTFATYV
APADFNETVNTLGLPLYAKQEPRKFDRGTDLHTQSNPLPMCHRPGVLVKLTAA"
CDS 31835..32116
/ID="WDFLIPAM_CDS_0045"
/transl_table=11
/phrog="10473"
/top_hit="No_MMseqs_PHROG_hit"
/locus_tag="WDFLIPAM_CDS_0045"
/function="unknown function"
/product="hypothetical protein"
/source="PHANOTATE_1.5.1"
/score="-5.710566386475198"
/phase="0"
/translation="MAQVTDLYEAAGRAGLLTDVMVGSLTVQCVFSAPDELALDGLALN
RDYHLEYPSAWLTLAAGDTVEIAGSPYRVREVRQLRDGSEMQAKLTRL"
CDS 32113..32541
/ID="WDFLIPAM_CDS_0046"
/transl_table=11
/phrog="4204"
/top_hit="No_MMseqs_PHROG_hit"
/locus_tag="WDFLIPAM_CDS_0046"
/function="connector"
/product="tail terminator"
/source="PHANOTATE_1.5.1"
/score="-8.054708238533369"
/phase="0"
/translation="MTPSVRERLIRAVVARIGSAIAPTPLHRQPTVPLPREASPALLLF
IEGDQVLAQANDRLDRALTLRLVALAREGDAFDVADAIIVAAHGALMAEPSLGGLALGV
REIDCEWDTEDADSQALAVPARYEIRYRTLASDLTQKG"
CDS 32547..32714
/ID="WDFLIPAM_CDS_0047"
/transl_table=11
/phrog="3357"
/top_hit="No_MMseqs_PHROG_hit"
/locus_tag="WDFLIPAM_CDS_0047"
/function="unknown function"
/product="hypothetical protein"
/source="PHANOTATE_1.5.1"
/score="-1.6813501063483864"
/phase="0"

/translation="MHIELIEPHTHAGRLHAPGEILDLDEAAAQWLIERGAARPADPQP
QTQIKTRKGD"
CDS 32717..33469
/ID="WDFLIPAM_CDS_0048"
/transl_table=11
/phrog="807"
/top_hit="No_MMseqs_PHROG_hit"
/locus_tag="WDFLIPAM_CDS_0048"
/function="tail"
/product="major tail protein with Ig-like domain"
/source="PHANOTATE_1.5.1"
/score="-299.1280022318275"
/phase="0"
/translation="MPYFSGQGRVYIGARDALGNPQGLAYVGNVPELKVSLSVETLEHQ
ESVSGQRLTDLQLIKTKKGEFACTLEELIATNLALALYGATTAQTPGTVTAEALPNPVT
PGSLYLLAKQDVSSVVVKDSSATPKTLPAAQYSVNAKHGSLVILDATTGGPYVEPFKVD
YAYGTASVTAMFTQPLPERWVRFEGLNTADGNREVVIDLYRVAINPAKELSVITDELLK
FELSGQVLADTLKPAAGDLGQFGRIVLL"
CDS 33475..33870
/ID="WDFLIPAM_CDS_0049"
/transl_table=11
/phrog="1442"
/top_hit="No_MMseqs_PHROG_hit"
/locus_tag="WDFLIPAM_CDS_0049"
/function="tail"
/product="tail assembly chaperone"
/source="PHANOTATE_1.5.1"
/score="-10.577490760962444"
/phase="0"
/translation="MTMSASDLDVLVPQPQVVDLAGQRLAISPLVLGELPAMLKAVQPF
AQRLADEPDWLALLSDHGDALLSGLAIASRQPREWVDALALDDAITLAATVFEVNADFF
VRRIAPKVGDLAQRLNGRLAGLTPSPA"
CDS 33987..34082
/ID="WDFLIPAM_CDS_0050"
/transl_table=11
/phrog="No_PHROGs_HMM"
/top_hit="No_MMseqs_PHROG_hit"
/locus_tag="WDFLIPAM_CDS_0050"
/function="unknown function"
/product="hypothetical protein"
/source="PHANOTATE_1.5.1"
/score="-1.372060619198341"
/phase="0"
/translation="MTAAAQGSRDGIRELQAELHRGMRDEDRSGR"
CDS 34060..34707
/ID="WDFLIPAM_CDS_0051"
/transl_table=11
/phrog="6367"
/top_hit="No_MMseqs_PHROG_hit"
/locus_tag="WDFLIPAM_CDS_0051"
/function="unknown function"
/product="hypothetical protein"
/source="PHANOTATE_1.5.1"

/score="-141.50704528088409"
/phase="0"
/translation="MKIDLVADGLLDRRRFSAWQGDTRKAIHTAVARAMRDTGKAMAER
ARGEMRAGFKVVKPKFLRSMHAKVFDRKAEEFPALYIGSKVPWLGIHEQGGTIRGRMLI
PLLPQHRRIGRKAFARVIDALMRSGNAFFIEKNGRQILMAENIAENARPLARFRRAERE
RTGAKRVRRGQEIPIAVLVRRVSLRKRFDLARSVRGDLPRLTAAIRKAMSKV"
CDS 34710..37907
/ID="WDFLIPAM_CDS_0052"
/transl_table=11
/phrog="2335"
/top_hit="No_MMseqs_PHROG_hit"
/locus_tag="WDFLIPAM_CDS_0052"
/function="tail"
/product="tail length tape measure protein"
/source="PHANOTATE_1.5.1"
/score="-37584701.223580286"
/phase="0"
/translation="VAGNRAQILITAVDETRRAFQSVQGNLTRLRGEAAQVGEVLSRIG
GAIGIGLGVRELVEVADQYKNLQARLKLAVTSQEEFNRADAALFEIAQKNRAPLAETVT
LYARLAPSVQALGRSQADVLAATDAIGQAVSLSGASSDAAAGALLQLGQAFASGQLRGE
EFNSVIEQTPRLAQAIADGMGVPLGSLRALAQEGKITSKAVLDVLLKERTRLAEEYASL
PDTVSGALTRLRNAFQRAFGERDASSGLTAGLAQAIQLVAGHLELLIDLAGVVLVAAFG
RMAGAFATSVAAARAEAAARLANLRTLEAEALARVRLADAALAQARAQGLATGALVADA
AKARLQATAASGAVAQAVASTSLLGRAAGLLRGVLALLGGPIGVIVTAAGLLAGALYSA
RDAVVEFGGRTASIKQIVAAAWDLVVEKVGEVVRALGRLVGTNDLSWVRVRAAMVGALN
AIGTAVRAMVNVVIGAFNAIGSVVGITAAFLVERFRNAFSDIGELAKALGQDVAAAFSG
DFSMQALRAALGRQLGEVRDFGKELAGAVRDAVTRDYVGEAAQAIARRIRPEQTQPGVF
GRPQPQALSAPDKGGEAAKLALVQAQAEAEFKLLKDALDRQARALDASLEDRLISLKDY
YAAKTRIEQQEIDAEIRRVQVSLAEQQRLQKTGKDEPARLKAKAEVAKLEAELTVLNNK
RADVEVANARKATQAERELREELAKVRDELLDLTGAATSQDRRAAIERQYQTLIERLRA
EGDTEGVVTVGRLIDVKSAAADLADYERQFNDALARMRASEESINLQRQSGLLTESQAR
SQILALHRQTGESLDALLPQLEAAAAAIGPEAVARVQTWKNEIAQVKLVVDDVAVAIDG
AVQDGFAQMFEAIGSGAKSAKDAFADFARSVLAAINRIASQKLAEALFGSLFGGSGAGG
FSLGSLVSSLFQGFAGGGYVTGPGTSTSDSIPARLSAGEYVLNAAAVKRVGVAFLEAIN
GIEGGPRIQGPRLAFAAGGLVPEAPPPQPQGQGQAVRIVNVIDPAMAADYLNSSAGEKT
ILNILQRNAGAVRQVLA"
CDS 37908..38840
/ID="WDFLIPAM_CDS_0053"
/transl_table=11
/phrog="687"
/top_hit="No_MMseqs_PHROG_hit"
/locus_tag="WDFLIPAM_CDS_0053"
/function="head and packaging"
/product="structural protein"
/source="PHANOTATE_1.5.1"
/score="-602.7572861053907"
/phase="0"
/translation="MAFEIGTASDYRDLLDRFHAFLTTHPNLVAAGQQWQALHWTTDAA
TKELILKAPGLAGAEEIYCGIRAYENATAGYYMWDLNGFIGFNPANDFYTQPGAISGWL
PMMSLWNTAIPYWFVANGRRAVVVAKISTVYQAAHLGFVLPYATPGQYPYPLLVGGSMT
GQRGRNYSVTSPNHRHFVDPGEDGQNNANTACMLRGPSGAWLPFQNLAYSSSEYRYDGP
RPVWPTNYTYLGNLREAPDGTYVLSPIVLTQNNSGTDHDLFGELEGVYHVSGFNNAAEN
LITVGGVDHLVVQNVYRTSVRDYWALRLE"

CDS 39098..41545
/ID="WDFLIPAM_CDS_0054"
/transl_table=11
/phrog="1082"
/top_hit="No_MMseqs_PHROG_hit"
/locus_tag="WDFLIPAM_CDS_0054"
/function="tail"
/product="tail assembly protein"
/source="PHANOTATE_1.5.1"
/score="-11097.10220563985"
/phase="0"
/translation="VGSTRIDRQRQLREQHHELPRRSLRPVRGGHRQHLPPTLGRHART
DHVCGRGLARRLSPSRLRGSHEVRQLRRWGVRVGRVRPGRLHLYVQRLQRLCLRLRPRP
ALRPAVQRLQVLRRRELRSRRGGRRHRLALGLQGLGAHRQARQGAVGTGHGHAARQSGA
LLVGARAQHLERRDADAAVLPVRGAAFRLLLALRPHRSPALPQHHPLRAGGGLRVGRRA
VDGLPGPLQERQKRRARLCRETHPLSPKRIMPTFPGSILPGAARQEPPTWSRSPEVSER
VPVAFPFDQSASSVMRPGARLDTQPVDELRLGFAGALLPTFGADWYHRIHVIPSTIDLG
NLVSPVERVLEVWNARFDAQTLDAIDETGTDGLLLSGQPAPPLAFGPLQSRLYTFAAGT
RGAPVIDAAYRFVFAGGLTALLVVTGRRVVVFGMRPDWSQGITERLEWLTEVLEAYDGT
EQRVRLRQLPRRGFEYGFLIEGRDAQVLDHLLFAWGARIYCLPVWTDVSTLAGEAAVGS
TALTVQDAANSDYHAGGLAVLWRSNTRHEAVEILSIAGNTLTLKLPLAGSWPAGTRVFP
ARLARLEGEVAVARPTDTIAVGRCRFGIEDITAPAVADYGPAYQGYRVFDWRPNRSTDL
EDRWLRRLSIIDYGTGLPTFDDESGSPLIGRTLTWLLTDRSKATAFRGWLAARAGRANP
CWLPTFESDLEVSRTVAATDAGLVVRNVGYARFVAADPLRRDLRLLTTAGTFHRRITGA
NEISEDEELLSLDAPLGVTLDPQQFLQVSYLELARLDQDAVELHWETDATARVQLSTRT
LRS"
CDS 41555..42352
/ID="WDFLIPAM_CDS_0055"
/transl_table=11
/phrog="778"
/top_hit="No_MMseqs_PHROG_hit"
/locus_tag="WDFLIPAM_CDS_0055"
/function="tail"
/product="tail assembly protein"
/source="PHANOTATE_1.5.1"
/score="-130.869112589392"
/phase="0"
/translation="MSYLGIEQSAHGGQPQELYRFSQGAQRWLYTSGQVAVDYQSETYQ
PATISRGGLEQSNELARLGLEIRMPRSLPVASLFLAAPPEGVVSVTLYRRHVGDAEFIT
YWKGRITGARLSGAEATLKCEPIASSLKRPGLRARYQLLCRHVLYSSGCGALKDSFRVD
GTVAAVSGVTVQVAVAASRPDGYFVGGMLATTAGARMIVGHAGIDLTLVAPMVGLTAGD
AVQLYAGCDHTMAHCKDRFGNLDNFGGFPFIPVKNPFTGDAIV"
CDS 42362..42601
/ID="WDFLIPAM_CDS_0056"
/transl_table=11
/phrog="1180"
/top_hit="No_MMseqs_PHROG_hit"
/locus_tag="WDFLIPAM_CDS_0056"
/function="tail"
/product="tail assembly chaperone"
/source="PHANOTATE_1.5.1"
/score="-5.174552420805932"
/phase="0"

/translation="MWQQIIIWVVTTVLSALLAPRPKVQDAQPGQIGDKDVPIASQDAP
IPVLFGTRVISGPNVVWYGDVQVRPIRKSSGGKK"
CDS 42658..42870
/ID="WDFLIPAM_CDS_0057"
/transl_table=11
/phrog="1196"
/top_hit="No_MMseqs_PHROG_hit"
/locus_tag="WDFLIPAM_CDS_0057"
/function="tail"
/product="tail assembly chaperone"
/source="PHANOTATE_1.5.1"
/score="-2.939724956622834"
/phase="0"
/translation="MRRWFEGRSHTWAEFVERGVPADWLRATGDAMAIRVAEEADKAQR
AAGQHEVLVPSAARDARSAQKEQAG"
CDS 42867..45068
/ID="WDFLIPAM_CDS_0058"
/transl_table=11
/phrog="1101"
/top_hit="No_MMseqs_PHROG_hit"
/locus_tag="WDFLIPAM_CDS_0058"
/function="tail"
/product="tail protein"
/source="PHANOTATE_1.5.1"
/score="-7217033.317206187"
/phase="0"
/translation="MSGGGKGSQEYTVGYWYGLGAHLALCHGPLDAITEIRVGERVAWS
GNVTGNTTITIDNPNLFGGEEREGGVQGPVDILMGGPTQGRNAYLQERLGADIPAFRGV
VSLILRRVWVAAMNPYIKPWSVRAKRVPRQWYAAKAEISGDANPAHIVRECLTNGEWGM
GYPTSDIDDASFMAAADALHAEGFGLSLLWNKEETIEDFILSVLRHVDGLLYVHPRTGL
FTLKLARDDYTISSLPIFDPSNILRIEEFTRPSWGEITNQVTVVYRDGVTDKDGSVTVQ
DIAAVQLNGGVVATTVNYPGISRAELANRVAMRELKQLVSPLAKCTFVANRQASGLNIG
DVVKLSWPPYGIDQMVMRVARIAYGELANGAVRVECVQDIFGLPQSVYSAPPPSGWTEP
TSLPAPCPHQTLFEVPYWSVVKDFTGESQSLLGDIDDLDGLVAACGSRPSSDAFGFKAL
ARVSGSFTEKGFGIFTPTAVLTAMLPQSAAQVSVGLTSGIGLEEVTAGGLTVIDGEWLK
VVSLNLATQTVTLERGMLDTVPASHPAGSRIWFVDGFRHYLTPEYVAGETVRVKLLTRT
ARGTLPEAAATEMSLPLDKRFIRPYCPGNAQINGKRYPTVVAGEINVSWATRNRQSQTA
YLVLQTEGAITPEAGQTTTVRFYNENGQLARTVSGITGNGTTWPLAQELADSGLGRVNA
HVKVEIEASRDGHVSWQKHVIEFDRTGYGLRYGDYYGGV"
CDS 45068..45418
/ID="WDFLIPAM_CDS_0059"
/transl_table=11
/phrog="5363"
/top_hit="No_MMseqs_PHROG_hit"
/locus_tag="WDFLIPAM_CDS_0059"
/function="unknown function"
/product="hypothetical protein"
/source="PHANOTATE_1.5.1"
/score="-9.708768440593602"
/phase="0"
/translation="MALNDPNLGLAYGWAQGEHNWNGGMDANLKRLGAVVGLSVKDRDL
ATPPATPVDGDRYIVPAAATGAWAGRTDQIAVRIAGAWEYHAPKIGWTCFVEDEGVLSV
YKATGWSPGIAV"

```
     CDS             45486..45776
                     /ID="WDFLIPAM_CDS_0060"
                     /transl_table=11
                     /phrog="10540"
                     /top_hit="No_MMseqs_PHROG_hit"
                     /locus_tag="WDFLIPAM_CDS_0060"
                     /function="unknown function"
                     /product="hypothetical protein"
                     /source="PHANOTATE_1.5.1"
                     /score="-4.893379216509172"
                     /phase="0"
                     /translation="MSPPTLQDGMVVMPRDEFEELLARAAERGARRALADVGLDGEDAA
                     HDIRELRGLLEAFNAAKHTAWQTVIRLVTTGFLLALVAGAVIKLKVFGGGQ"
     CDS             45773..45874
                     /ID="WDFLIPAM_CDS_0061"
                     /transl_table=11
                     /phrog="6637"
                     /top_hit="No_MMseqs_PHROG_hit"
                     /locus_tag="WDFLIPAM_CDS_0061"
                     /function="unknown function"
                     /product="hypothetical protein"
                     /source="PHANOTATE_1.5.1"
                     /score="-2.382693151628733"
                     /phase="0"
                     /translation="MIETLLGGLLGGAFRLAPEILKWLDRKGERGHEL"
ORIGIN
        1 cctttttct tgcgcgctcc cgcgtgttcg cggggactcc tgcggattcc tgcggacttc
       61 cttcctccgg tcgtctcgcc catctcggac ccatttcgct ctctccgcgc cgttattctc
      121 tccagcctcg ctccccgact aagggccgaa gtccacagag gccgcaacgc ggacccatac
      181 agatcaatgg gttacgcgct gacgaatcaa acggttggat tgcggcatcg gcaggcaaag
      241 gaaaccatgt cagttgtgtt ttgtcggtag ttcttgacag ctaccgacgt ttcgcaacat
      301 tatgacgaca tggacagttc gcaccagagc attctggatc ttgccgccca gcgcggcctt
      361 attcgcccac gcgatctcaa tgagcgtggg ctgcccacgg tcgccctcac gcggctggtg
      421 cggcaagggc tgctccagcg cgtgggacgg ggcctgtacg caattccgga ccgggccgtc
      481 tcggaacacg gcgcactcgc cgaggtggca cgcaagcacc cgcaagccat cgtctgtctg
      541 ctgtcggcac ttcgcctaca tgaactcacc acacagtcgc cgttcgaggt ctggctggca
      601 atccccaaca aggcacgtgc gccaaagatg gactatcccc cgctgcgcat cgtgcgcttc
      661 tccggcgccg cgctgacgga gggggtcgag gagcacctca tcgatggtgt gcctgtgcgc
      721 gtgaccaacg tcgcccggac ggtggccgac tgcttcaagt accgcaacaa gatcggcctg
      781 gatgtcgcgc tggaggcgct gcgtgaggcc tggaaagcaa agcgcgtcgg catggacgaa
      841 ctgtggcagt ttgccaagct gtgccgggtg gccaacgtca tgcgccccta cctggagagc
      901 ctgacgtgag cagccggaac accgccgcct cggtccgtgc acgactgctc gccaaggccc
      961 gcaccgacaa gcaggacttc aacctcgtcc tcacccgcta cgccctggag cggctcctgt
     1021 atcgcctgag tgtttccgct catgcggatc acttcctgct caagggcgcc ttgctgttcg
     1081 atctgtggtt cgacatcccg catcgtccga cacgcgacgc cgatctgctg ggcttcggct
     1141 cggccgagat cccgcatgtc gaggcggcgt tccgggatat ctgtgcggtg gaactggacg
     1201 acggcatacg ctttcaagcc gactcggtac acgcggagga gatccgcaag gaagccaact
     1261 actccggtgt ccgggtcacg ctgatcggcc tgttggacgg tgcccgatgc cacgtgcagg
     1321 tcgacgtggg tttcggcgac gccgtgacgc ccggccccga gaccgtcgac tacccggtga
     1381 tgctgtcgga gatgccagcc ccgaagttgc gtgcctaccc ccgctacacg gttatcgccg
     1441 agaagctgga ggcgctggtg tcgctcggca tcgccaacag ccgcatgaag gattacttcg
     1501 atctctggat cttgtcgcgc tacaccgact tcgacggcaa gcttctgtgc aacgcgatcc
     1561 acgccacctt cgagcgccgc aggacgccgc tgccggatgg cgttccgttc ggcctgagtg
```

1621 acgaattcgc gcaggatcgg cagaagcaga cccagtggca ggcattcctg aggaagaacg
1681 ccctggtcga gcttgcgctt tccgaggtcg ttgccggtct gcgtgccttc ctgtcagtcc
1741 cgctggatgc gctccagcaa ggtgctgcat ttccgcaaac atggcttgcc ggaaatgggt
1801 ggacgtcgag cagcgaggaa ttcagccgat gacatggaat cccgatctgc tcctggagga
1861 ttttcgccgt gtcgccggca tggccggcgt ctcgttggca cccgatgcca tcgcgatcga
1921 gcggcgctct gcgccgcatg tgccgcccaa gtcactgccg ctcggcaaga tggccgtcta
1981 cgtcttctca ttcgggcagc atgtgctgaa ggtcgggaag gtcggcccca acagcgctgc
2041 tcgctacacg gctcaacact acaacgccgg cagcgcgaag agcactctgg cagcctcgtt
2101 gatcaaacac ggcgagcgca tcggcgtcgc cggcctggac gaaacgaacg tcgccggctg
2161 gatcagggag cacaccgatc gggtgaactt catcctcgat gcgccgctcg gtgtccacgt
2221 cctcaacctc ctcgaggcat ttctgcagtg ccgcctccgg cctgagttcg agggattcgc
2281 cagccaacgg atcgatcgcg agggtggaca ggaatgaaag aagacatcct cgaacaaatg
2341 gtggacgaat acctgcagca caaagggtac ttcacccggc acaacatcaa gttccgaccc
2401 gccggcgatc acgcggagta cgacacccgc caggatgccg tgcatagcga catcgacgtg
2461 atcggcatcc acccgcgact cgacggcgcg cgtcgggtga tggtggtgag ctgcaagagc
2521 tggcagggtg gctttcgtcc cgaatactgg atcgatgcga tcgccaagaa caaggtggtc
2581 tcggggcgtg aggcctggcg cgggtttcgc gaactgacca gggagaagtg ggccgcagcg
2641 ttcaggatga cggtcgccga actgacgggt tcgtcctcgt tcacctacat cacggccgtc
2701 acgaaagtca tcggcagccg atcggtgtgg gaggacaacg cggcgttccg tgagcatctc
2761 ggcggcaacc ccatcgagat cttgaccttc ggcgacatgc tgaaggaact cttcccgttc
2821 atcgatacca ctccggcatc atccgaagtc ggacgggttc tgcaactgat caaggcctcc
2881 ggctggtctc tcgacaagta atccaaaccc aaagggttta cagccgatgg caatcagcga
2941 tcaatgatgg cccgctgctc ctcccacaac aacggcgtcc ccgacgccag atcgaacagg
3001 gtcacctccg gcggcagggt ctcgtcgagg atggcggcga cgatgtccgg cgccagggtg
3061 gtgaggttca ccatccgact cacataggcc cgatccatcc tctcgcgttc ggccacctcg
3121 gatagcgtgc gcgcctcgcc ggattgttcg ccccgtccct ggggctcacc ccttcggggc
3181 cggctacgcc gtccaaattc gctcccggcg aatttgtcca ccatcgccag ccagcggtgg
3241 ccgcgggcga gcgcgagctg gatcggcgtc ggcgtgtcat cccacgggcg tggttgcacg
3301 gcgccgccgt ccggcagggt cacggccttg cggctgccgc gacgcttgat ccggattggc
3361 acggtgacgg tgaggctgcc gtcgctcgcg gtgatcacgt ctggctgacc ggtcttgtcg
3421 atcagaattt cattcatgcc acggcctcct caagcgtctc cggggcgggc tcggggcgca
3481 gctccagggc gagcacctcg atgccgttgg gccggaaccg cacctcgatg tcgttcggcg
3541 agacaatcac cttgtcgatg aggagccgca cgatgcgctg ctgctcggcc ggaaagagct
3601 gatcccagat cgcatccagc cgggtcatgg ccaccgcgac ctgggcctcg tccagggagg
3661 gatcgagccg ggcggcgcgc tcggccaccc cggcgaccat gtccggggcg cgcagcacgc
3721 ggcgcatctg ctccagcacg gcggcctcca gctcaccggc cggcaggcgc ggcaatccgg
3781 aggcaccggc gtgctccttg ttctcccgcg tcggcaggta gtagcggtag atgcgcccgt
3841 tcttcttgcg cgtggaccag ggcgtgagcg cccggccatc gattcccacg acgatcccct
3901 tgagcaggaa cggcacccgg gcgcgggtgt tgttgcctcg cacccgcgag ttctgcgcga
3961 ggatcgcctg gacggcgtcc cacagtttgc gttcgacgat ggtcggatgc tcgcctgcat
4021 accactgatc gcgatggcgg atctcgccca ggtagacccg gttgttgagc agcttgtaga
4081 cgaggctctt gtctatgggc ttgccctcgc gcacccgccc gtcctaggta gtccaggcct
4141 tggaggtcac gccgtcgagg cgcagctcct tcacgagcag ggtcgaggac ccgagttcga
4201 cgaagcgctg aaagatgtgc cggatcagct tggcctcgcg ctcgttgggc accaatcgcc
4261 ggttctcgac gtcgtagccg agcggcggca gccgcccatc cacatgccct tgcgcttgct
4321 ggcggcgatc ttgtcgcgga tgcgctcacc ggtcacctcg cgctcgaact gggcgaagga
4381 gagcaggata ttgagcatca accggcccat cgaggtggtg gtgttgaact gctgggtgac
4441 cgagacgaag gagaccccgt ggcgctcgaa gacgtcgatc atgcgcgaga agtcggtcag
4501 cgatcgcgtc agccggtcga tcttgtagac caccacgatg tcgatcctgc cggcctcgat
4561 gtcggcgagc agccgcttga gcgcgggccg gtccatgttg ccgcccgaga acgcgggatc
4621 gtcgtagtcg tcggccaccg gaatccagcc ctccgcgcgc tggctggcga tgtaggcgtg
4681 gccggcgtcg cgctgggcgt cgatggagtt gtactcctgg tcgagcccct cgtcggtgga
4741 cttccgagtg tagacggcgc agcgcaggcg gcgcttcagg gcttcgctca tcgccgacct

4801 ccctgcttct tcggatgctt ctgcttggcc ggtgccttca cgccgaagaa gagcggcccg
4861 gaccagcggg tgccggtgat ctcgcgggcg atcatggaca ggctcgggta gcgcctgccc
4921 tcgaactcgt actgcccgtc ctgggcgacc gtcacccggt gctcgacccc ctggtactca
4981 cgggtgagca cggtgccggg catcagccgg atgtcgcggt cgagcttgcg cgccttcccg
5041 gtctccagca gggccttgat gcgccgcttg ttgcgctcca gcaggttggg atcgaccttg
5101 cggaactcga tctcctgcag cttgtaggcg atccgcctct ccatgaactg gcggttgggt
5161 gtcgggttct cgctgccgaa gagttctcgc caaagcgccc tcatttcctc tatcgagaga
5221 tccgggagcc cggcgatccg ggcgacgacg ctcggcgggg tgggtgggac gatgcttgcc
5281 ttcatggagc ctcctccaaa gtgttgttga cggggtggct atgaaggcgc tggtggccgg
5341 agaagccaag ctcaaactcg ctctccgcgg ggccgcctgc ggactgagcg aacccggcct
5401 cgcgcagtcg gacgaggccg tgggccagca aggatgcgac ttcgcgccgg cgctgctccg
5461 gcgtcatccg ttcgggtggt aggtggttaa tcgtgcacat cggtatcggt cctgaacgtc
5521 caactcgctt gatgacgaga ttgtccagtc aggccgtgac ctcggccatc agggagtttc
5581 gggccggtgc ggactggtgc ggcctactgc gagaaggcgg tgccggctca ctccgatagg
5641 tgccgggcca gcatcgtcct catgagcgag tttccgggct gcgtgccggc tgtcatctcc
5701 cggatctgct cattgatgcg ctgagtagga acgcctgcgc cgtgagccgc ctgcgtcacc
5761 gccgagtagg catccagcac atcaacgccg gtgatttcgt acccataccc atgagagatc
5821 caatgcagcg cagccaatcc agccgcaagt gcgaagtcag gatgcttctc agcaaaatca
5881 cgggcggcgc gagtcaaggt gcgaggatcg gtcggactgc gcgtagccag ttcagcagca
5941 acgacgaaga gacctgcatc cttggctgcg gcaaaccatt tgccctcggc gcgaggcgtg
6001 ctggcaatca agtcgcgcag gatttcttcc ggctgcttgt ttggatactt cttggaaatg
6061 gcgcgaaacg tggcgagatg agtcgtgcct tgattggctt ccagcgcgta gcgacggtag
6121 gcctcgtcga gcaaaccgga tgacaacagg atggcttcac agtcctgggc tatttgccag
6181 cccgggtcat tgagcccgcg tgaatcctct gcatagcgga tcgcctctgc cttcttgccc
6241 atcgccgata aggctttcac gccccagcgc cggtcgtgcc accacttgaa tggcgccttg
6301 tcgatcaacg ccagcaattc ctggtgacgg cctgccgcgt acagggatgc caggcaagca
6361 ctcgtgccct tgaagaatcc gtggccggac gcagtccggc tccagacatg ctcgacaagc
6421 ggcagaaact catcggccca gtgtgatgcc aactccgggg tcacgcacag ttccccccaa
6481 tagtcgccta gcagttcgat gtagggcatc tcgtcgtcct gcagcgcttg ccataaccgt
6541 tcgagccaac gctggcgcaa gggcgtgtcg acgtccgcct tggcgatgag aggtaccagt
6601 gtctcgatgg ccttgttgac ggcggaaccc aaggcgcccg acgagctatc gacttgctcc
6661 agggctggcg agagtttttc cagcagagta atggcgccct cggccgccaa caccggctcc
6721 ttgcgggcga cctgcttgat ctccgcgatg gcttccttga tccgctgcac cggcgtgtcg
6781 gatcgccaac cgaacgcgtg tcggcggaaa cgggaggcaa actgccactt gtgggcactc
6841 atgatcggcg acccattgcc tatcgcaccg ggtactggcc attgcggatg aaacgatcgt
6901 aggtgtcctc ctccgactcc tcatcgtcgt gatgggcacc ctgccactcg gcttccggca
6961 gcaggagcag ggtcagggtg tattcgtact ggccagcgtc cctgatcatc tcattgacca
7021 tcatggtttc cggctcgcgc ggaaaccaat gccgtgccgc tgtcgtttgc tgctggccga
7081 tgtccaaggc gttactgctg tgcgccagcg catcacgcgg cagctcgatg atgttcctgc
7141 gggttgcgaa gtaggcgcca gacctgagcg ccgccttgtt cgacttggcc cacagcatat
7201 ggtcgtcgcg gctggcgatg aggaccgccc gcttgggcgc gatctcggtc cagcgcaaag
7261 ctgccgcagt cagcgagacg ccgtagcggt cggcgcagtg acccagcaga tcgaagctga
7321 ctggctgccc gtccacctgt cgccggaagt cgtccagtgg catcagcagg gtcgaggcga
7381 aaagatccgc ttcggcctcg atgtcgcact cgtcgtcatt gccggtctca atgtcgtcgt
7441 cgccgcactc gaagcggtct ttctggtgac ggtgcaggat gtagtgccca aactcatgcg
7501 cgatcgtgaa gcgcttacgg ccttctgatc gggtggcgct gttgtacagg atcagccact
7561 tcgaccgcgt cttgttcgcc gccagcatgc cgtcgaaccc gtccagcgct tccccccgga
7621 ccttgtctac gggcgaatcc gcgaaacact ggcgcgagta ctcgatggcg agctcatcga
7681 ccttgaccgg aaatcgatcc gtgcccagaa ccgcgttgag catggccgag atgcggttgg
7741 cctcggccat gggctttttc gcctctgtca ttcatcttcc cacgcgtcga ggatcttgcg
7801 gatcttcttc ttgtccggct cggacatgtt cttgtactta cggaagaagg cctcatcgag
7861 cacttcttcg tccggcgtgg tcgccgactc ggtgagcagg aattctgtag tcacctcgag
7921 tacggcagcg atcttgccga tcttctcagc cgagggcttc ggttcgtcct tgttctccag

7981 ctcccagatg tagctcttgc tggagtcggt cagttcggcc aactgctcaa ggctgagctt
8041 cttctgtttc cgcaatgagc ggatcttgtc ccccaagggc gatggcaccg gtatctccta
8101 atcgttctgc gtcaggccga agatgttacc actacgccga acgatttcgt acctgcttga
8161 caaacccggc atcagtccga gacaatccga aacgttcggc acaccgaacg ttcgcgtcct
8221 tggtgggatg tggcggcgcc gggcatcgtc cgtttgaaga aggagaagaa aggcatgacc
8281 gcgttcaacc ctcgtcacct cgcccgtcag gttccggcca ccacttggca ggcgtatctg
8341 tcctcccgtt ccatcgctat ccccgaatct ttcgactgga acgccgagga aaaggcattt
8401 tccgatgccc tgatcgccct cctggaggaa ctcgaaccgg atcaacaggc cctgctgcac
8461 gccgagctgc gccatgtcta cgcccttgcg acccagaaag gcatcgatgc aatcctcaac
8521 gccagcgaca acgacgtggc cattcgggag gacttcgggc agctccgcaa ccacgccgag
8581 cgggccatgt gggtgttggt gaactggccg caaaccttca tgacggccga ggcgctgctc
8641 caattcgatc tcggcgtcgg caagcggtcg tggaaacggc aagccatcaa ggtgaccgag
8701 ccggtgtccc gagaggctgc cgacatcgag ggattgcagg ccgccttgtc cgaggtgctc
8761 tcgaaacgca agggaccgcg gcgcgcctgc cacgtggacg tctgcgaccg tcacctggac
8821 ggcggtgtgc agatcagcgt ctacgtggag gacgatccca acgatctggt ggagttcgtc
8881 gaggagggca tgcgccgccg gacgacccgc ccggcgacca atctcgcgct ggtctacttc
8941 cccgcatccg gcatcgtcga caccgtcggc cggggcggcg ccaaggtcca ccagccgctg
9001 gtgacgctct tcgcacgcca tctactgaaa caggaggtca agcccgaggc agtgaagcag
9061 ccgatgttct atctcaaccg gttgcggcac ggcttggacc ttccggagga cagcgacatc
9121 gacctcgccg cccacggcat tgatcgcatt cgcctccgcc gcgcccggct caggagcacg
9181 agggcgccga tctgcgactt ctgggtaggc gtaccggcgg atcaggccga acactgcgtg
9241 ctcgctgcat cgagcgccca cctgaaggac cacgacctgt tccgcggtcc gttcaatatc
9301 gtcgaggccc tcatcagcat ctacttcgcc cctgccgagg tgggcaagcg cggtcgcgta
9361 ctgaacatcg acatcaagca atcggggatc tccaacctcc aggacatggc agaggaggac
9421 gccaagctcg ctgagcgcct cctgcgtgcc tggcgcgtct ccgaaccgac cgaggtcgaa
9481 ctggccctgg tcgcgtagaa gccgccgtgc aagcgcgcaa cgacatcgcc ttcggcctgc
9541 tctgcgggct cgtcgaggaa tccgagggaa gcctccatcc ggatgccgcc cggtattccg
9601 gacaaatgga tgcctatcgg catctcctcg acctcggcgc cctcggggcg ggtgatggtg
9661 tcggatcgag catcctctgt ccctggtgcg gaatgggcga tctagagggg attcgcttcg
9721 agcaaggagg ccgccagggg tactgcacgg actgcggctg ggtggatctg gccagtgatc
9781 aagtgacacg gctcagggtg gacatcacgc gcatcgtccg gtggctcgcc tcggccctgg
9841 gccttgcggg acggtatcaa catgaagaga tggttccgac agcgctgtgg cgcctgggcg
9901 agatcgaaca tcgacgaaag cggcgcacta tcttcttcgg tcggaggctg agcgacccgg
9961 ccttgacgcc catcatcgaa gctcgtttgc gcaccgcctg cgcacccggc tgcggcgtcc
10021 tgatcactac cacacctgac gtccctcaag cgatccaggc cgccggccat ctcgtcgttc
10081 cgctgcgtgc cgtggcgcat ctccgcaagg ccggcttcgt catcgagaat ctcgacgcat
10141 acctcgacgg ctccgccggg atcggtgaac gggattcgga aacctccctg cggctcatgc
10201 actccggccg catcgcagtg atcgacggag agcggcacgc actgtcgccc caggtctacg
10261 gctttctttc ggtactcgcg cagggcggcg gcgatcccgt ccataaacgc accatcgccg
10321 acgccttgga aatcgatgtc gacaagtgca agggcgcgga catctgcaag cggcacaagg
10381 ccgtctaccg caccttcgtc gcccacgaca acgacggtca ctattggctg aaacccgaat
10441 tcgtcaacga ccaaaggagg tgatgcaccc aacgaagcgc aatcctttca tccggcagtt
10501 caacttcgat ctcgatgagg taaatgacat gagtggtaag aaccagtggg tcgtccgcaa
10561 cggcgacaag tggggtgtcc ggggggaagg caacgaccgc ctgacctccg tacacgatac
10621 gcaacaggaa gccttcgagc gcgcacgcga catcgcgcgc aaccagggca gcgaggtgct
10681 gatccagggc gaggacggga agatccgcga gcgcaacagc tacggcaaag atccgttccc
10741 gcctcccggc tgaccacccc gcatttccac ccccgataag ggcccggagc ccaccactcc
10801 gggccttttt cttttccggc tctgcgaaga gccgatttgc acgatttgcc cgcccatcct
10861 gcccgctttg cccgccggtt tgcccacccc ctgaaaccca gaatgacctc acgtttccgc
10921 aacaacccgt aaggagcgaa tcgtgagtat caaacacctc aaccaacgcc aactggccga
10981 ccgttggggc gtcagcgaag ccaccttgga acgctggcgc tccgaaggaa tcgggccggt
11041 ctttctcaag atccaggggc gcgtgctcta ccgcgtcgag gacgtcgagt cctacgaagc
11101 ggattgtctg cgcaagagca cctccgagcg cgtcggcgcg ggaggtgtgg catgagccgc

11161 ctcaccccag accaggttct cgccacgccg gccggcgagc tcgccgcgct gcaaagcgat
11221 gcgctgttcc agctcaagaa cgatgccgcc gatctgctct cggccgccaa ggccatcgtc
11281 gagcacctcg agcgcgcatt ggagctgaag tacgccgacc gcgcccaggc cctgcgcctg
11341 gccgccggca aggacaccgg tgtcgtgcat ttcgacgacg gccgcgtgcg cgtcaccgcc
11401 gacctcccca agcgggtcga gtgggaccag aagcgtctcg ccgagatcgt gcgccgcatc
11461 gccgagggcg gcgaggaccc ggccgagtac gtcgagaccg cctaccgcat ctccgagacc
11521 aagttcaacg cctggcccga gtcgctgaag agcgccttcg cccccgcccg caccctcaag
11581 accggcaagc ccggattccg tctcgccctg ctggaggagt gaccgtcatg ttcaagaacc
11641 gcactttgct cgacaagctg aaaaagcagc acccctatct gctggagtcc ctgccggaac
11701 ggatcgagag gaatggcgcc gacgtggcca tcgaagatgc caccctggat cagatcgcct
11761 tcgccgtcat cgccctcgaa aacgaggtcc ggcccatcag ccgccgcatg aacgctctgc
11821 gggagctcta cgacctcgcc cgcaaacacg gcgcactcgg cgcgcaccgg atcggagacg
11881 ccttcgccga caaaggaggc cgctcatgag ccttcccatc atctccgccg accagcgtct
11941 ggccgagaag cgcggcgtga aaggcgtgct ggtcggcaag agcggcatcg gcaagacctc
12001 tcagctctgg accctggcgc ccgaggccac cctgttcttc gatctcgagg cgggcgacct
12061 cgccgtcgag ggtgggccg gcgacaccat ccgcccgcgc acctggcagg agtgccgcga
12121 cttcgcggtg ttcattggcg gtccgaaccc ggcgctgcgt gaggaccagc cttttagcca
12181 ggcccacttc gacgccgtct gcgcgcgctt cggcgacccg accgtgctcg atcgctacga
12241 caccgtgttc gtcgactcga tcaccgtcgc cggacgtctg tgcctgcagt ggtgcaaggg
12301 ccagccgcag gcctactcgg agaagaccgg caagccggat acccgcggcg cctacggcct
12361 gatgggccag gagatgatcg cgtggctcac ccacctgcag cacacccgcg gcaagaacgt
12421 gtggttcgtc ggcatcctcg acgagcgtct cgacgacttc aatcgccgcg tcttccagct
12481 gcaaatcgac ggctccaaga ccgggctcga gctgcccggc atcgtcgacg aggtggtgac
12541 gctggccgag ctcaaagccg acgacggcac ggcctaccgc gccttcgtct gccacacgct
12601 caacccctgg ggctaccccg ctaaggaccg ctccggccgc ctcgaccaga tcgaggagcc
12661 gcacctcggc cggctgatgg ccaagatcgc cggccccgcc cgccccgccc tcgaacgcct
12721 cgacttcgcg cgccccgcgc cttccgaacc cgtacagcaa taaggagcta cgaccatgag
12781 ctatttcgat ttcaacgatg ccaacgagca gtcctcgttc gacctgatcc ccaaaggcac
12841 gctggtgcac gtgcgcatga ccatccgccc gggcggtttc gacgatgcga gccagggctg
12901 gaccggcggc tacgccaccc gcaacgacaa caccggctcg gtgtacctca attgcgaatt
12961 cgtggtgatg gagggtgagt acgcccgccg caagctgtgg tcactgatcg gtctctacag
13021 ccccaagggt cccgagtggg ccaacatggg ccgcgccttc gtcaaggcga tcctgaactc
13081 cgcgcgcggc gtccatccgg gcgacgccag cccccaggcc cagaacgcgc ggcgcatcgc
13141 cgggttcgcc gacctcgacg gcatcgagtt cctcggcaag gtcgattggg agaaggatca
13201 gaacggccag gacaaggccg tcatcaagca ggcgatccag ccggatcaca aggaatacgc
13261 cgcgctgatg ggcaacgcgc gtccgtccgc cccggcgccc accacgccga acgcctatgc
13321 ccaagccacg ggtcgcgctc cggtcacggg ccgtccgagc tgggcgcagt aagggagact
13381 gccggcatga tgcttcgtcc ccgtcaggcc ctgctggtgg agcgctcgct cgcggcgctg
13441 caccagcacg gcaacaccct ggccatcggc cccaccggtt cgggcaaaac catcatgctg
13501 tcggcggtcg ccggcggcgt gttggaggag cccgacgcca agtcctgcat cctcgcacac
13561 cgcgacgagc tcaccgccca gaaccgggag aagttcggcc gggtgaatcc cggcctcacc
13621 acctcggtgt tcgatgccaa ggagaagtcc tgggccgggc gcgccacctt cgcgatggtg
13681 cagacgctct cgcgcgatgc gcatctcgac gcgatgccca cgctcgatct gctggtggtc
13741 gacgaggcgc accatgctgc ctcgccctcc taccggcgcg tgatcgatcg cgtgctgtcg
13801 cgcaacccac gggcgctgat cttcggtgcc acggccacgc ccgcgcgcag cgacggcaaa
13861 gggctgcgcg aagtcttcag caacgtggcc gaccagatcc acctgggcga gctcatcgcc
13921 tcgggccatc tggtgccgcc gcacaccttc gtcatcgacg tcggcgcgca gtctgcgctc
13981 gcccaggtgc gccgcaccgc caccgacttc gacatgaccg aggtggaggc gatcctcaac
14041 aggacgccga tcaccgacgc ggtgatccgt cattggcgcg agaaggccgg cgaccgcaag
14101 accatcgtgt tctgctccac cgtggcccac gcgcagtgcg tggccgacgc cttcgtcgcc
14161 gccggcaccc gcgccgtact gatccacggc gagttgtcgg acgccgagcg caaggcgcgg
14221 ctcgccgaat acgagaccgg agatgcgcag gtcgtggtca acgtggcggt gctcaccgaa
14281 ggttacgact acacgcccac ctcctgcgtg gtgctgctgc gaccgagttc gcacaagtcg

14341 acgctcaccc agatgatcgg gcgggggctg cgcaccgtcg atccggccga gcatcccggc
14401 gtggtcaaga ccgattgcat cgtcctcgac ttcggcaccg cgaccctcat gcacggctcg
14461 ctggagcagg aggccaacct ggacggccac cagcaccagg gggaggcgcc caccaaggag
14521 tgtccgtcct gcgaggcgac ggtgccgctc ggctgccgcg aatgcccact gtgcggcttc
14581 gaatggacca ttgatccagc cgagcaggcg gaagccatgg acgacttcgt catgacggag
14641 atcgacctgc tcaagcgctc caacttccgc tggtgcgatc tcttcggctg cgacgacgcg
14701 ttgatggcca ccggcttcgg tgcttggggc gggatcttct tcctcaacgg ccgctggcac
14761 gcggtcggcg gcggcaagga tctgcagcct cgtctgctgg cggtcggtga tcgcaccgtc
14821 tgtcaggcct gcgcggacga ctggttgaac gagaacgagt ccctcgacac tgcccacaag
14881 acccgccgct ggctcaacga gccgcccacc gagaagcagc tgcgctacct gccgcaggcc
14941 atgcgttccg acttcgggct cacccgctac caggcctcgg cgctgctcgc cttccagttc
15001 aacaagtcgt ccatccagcg cctcgtactg gcagccaacg acgaacaccg gagggcggcg
15061 tgatatgcgc gatctgcgga cgagagggac gcggcctctg ctggctgtcg ccgaggaatg
15121 gcccgcgtgc gccggacgga aggcctctgt tcaagcgctt ctgctcaatg cgctgccagg
15181 acatccatct gcgcaggctg aaggcgggag gtggcgtcgt gattgatccc acccacaacg
15241 agaaggccgc gatggaggcc gtactgcccc acctcggcga atacgtcgcc gctatcggga
15301 tggaccggcc gctgtcggcc tacagccgca aggaggtcct gcaactggtc gacgtggtgc
15361 tcaccgccta cttcgacaac ctgcgggaga tcacgcccga cgacgtgccg ttctgagggg
15421 ggtgtccatg ctcgattaca accaccgccc caagttccac gagcgcgtcg gcgccgtcat
15481 cgacgaggcg ctggcgcgtc aacgtgcctc acaaacgccg cgccgttatc tcggcgcgtc
15541 acgactcggc gtggcctgcg agcgcgccct gcagtacgag tacgcccagg ccgcggtcga
15601 tcccggccgc gagctgcccg ggcgggtgct gcgggtgttc gaggtcgggc atgccctgga
15661 ggatctggcg atccgctggc tgcgcctggc cggcttcgag ctctacacgc gcaaggcgga
15721 cggcgggcag ttcggttttt ccgtcgccgg cgggcgtatc caggggcacg tcgacggcat
15781 cctcgccgcc ggccccgccg acatcgagct cgacttctcc atgcgttggc ccgcgctgtg
15841 ggagtgcaag acgatgaacg acaagtcctg gcgcgagacc gtcaagcagg gcgtggcccg
15901 cgccaagccg atctatgccg cccagctcgc cgtctaccaa gcgtacatgg aaggcaccgt
15961 gccgggcatc gcgagcaatc ccgcgctgtt cacggccatc aacaaggaca gcgaggagat
16021 ctggttcgag ctcgtgccgt tcgacggcgg gctcgcgcag cgcatgtcgg accgggccgt
16081 gcgggtcatc accgccaccg aggccggcga gctgctgccc cggcacgcca ccacgccgac
16141 ccacttcgag tgcaagtcgt gtccctggca ggaccgctgc tggagaccgg cttgatggca
16201 gacgtcattt ggctcgatta caacgacgcc cccgaacagc atggcgagac gccctccgac
16261 accgaggcgt tgcgccgggg cctgctcgac cgcctggagg ccgtgctgct ctacctgttc
16321 ccgcagggcc gcatccgcgg cggcaggttc tacgtcggcg acgtcgacgg caaccggggc
16381 aagagcctgg tggtcgagct cgagggcgag cggcgcggtt tgtggaagga cttcgcctcc
16441 gacgaaggcg gagacgtcat cgatctatgg gcgcgctcgc gcggcctttc cgcgcggcac
16501 gactttcccc gcctcgcgga cgagatccgc cgatggctcg ggatcgcgac gcccactcgc
16561 ccacccgaac cccgatccaa aatccgcagc gcgccagtcg acgaactcgg cccgtacacc
16621 gccaagtggg actaccttac ggcggacggt cggctcatcg cctgcgtcta ccgctacgac
16681 ccgcccaccg gcaaggagta ccgcccctgg gacgtgcgcg cccgcatgtg gcgcgccccc
16741 gacccgcgtc cgctctacaa cctgccagcc gtggccgagg cgcgcgaggt ggtgctggtc
16801 gagggcgaga aggcggcttg cgccctgatc gggacgggca tcacggcgac cacggccatg
16861 aacggcgcgc gggcgccgat cgacaagacc gactggacgc cgctcgcggg caagcacgtg
16921 ctggtctggc cggatcgcga tgcgcccggt tgggactacg cggagaacgc cgcccgtgcc
16981 tgcgtgacgg cgggcgcggc gtcggtcgcc atcctcgtgc cgcccatgga caaaccggac
17041 aagtgggacg cggccgacgc cgtggccgag ggcttcgatt gcgccgcgtt cgtgcatgag
17101 ggcgagcgca gggtcgtgaa gaccgccgcc cccgggctcc cgaccttcac cctgggcatg
17161 ctgctggacg acgactcgcc cctgccggcg gatctcgtcg cgccgcgcgt gctgaccccg
17221 ggcgggatgc tcgtcttcgg cggcgcgccc aaggtcggca agagcgactt cctgctggcc
17281 tggctcacgc acatggccgc gggcgcgacc ttcctcgcca tgacaccgcc gcgcccgctg
17341 cgggtgttct acctgcaagc cgaggtgcag taccactacc tgcgcgagcg cgtgaaggag
17401 atccgcctcc cgcccagtcg cctgggtgcg gcacgcgcca acttcgtcgc cacgccccag
17461 ctgcgcctgg tgctggacga cgccgggatc aatcaggtca tccccgccat cgcgcaggcc

17521 ttcggcggcg agccgcccga catcatcgcc atcgatccca tccgcaatgt cttcgacggc
17581 ggggacgcgg gcggcgagaa cgacaacgcc gccatgctgt tcttcctgtc acagcgggtc
17641 gagcgcctgc gcgatgcggt gaacccggac gccggcgtcg tgctgaccca ccacaccaag
17701 aagctcggca agaagcagtt cgaggaggac ccgttccagg ccctggccgg ggccgggagt
17761 ctgcgcggct actacaccac cggcatgctg ctgtttcgcc cggacgagac acgcaccacg
17821 cgccagctga tcttcgagtt gcgcaacggc gcggccatcc cctcgatgca cgtggacaag
17881 atccgcggcg agtggcggga ggtcgatgcc aacgagcggc tggtgatgaa ggactacggc
17941 gagcgcctcg atgccgagcg ccggcgcaag cgcgacgcca tcctgcagat cctgttcgac
18001 gaggcggccc aggggcgctg ctacaccgcc aaccagttcg cggaaagctt cgagggcaag
18061 gcggggctcg gcggcgagcg caccatccgc gagcgcctct ccgcgctctc gacccagggc
18121 tacatcaagt acttccgcaa cgcgcaggac tacggcctgc cgcccgcccg caccaagttc
18181 ggctacctgt gcgtcgaggc gatggtgctg cgctcggtcg tcggcgagcc cgatccagac
18241 accggcgaag tgtcgctgcg cgaactgccg gtgctgccca cccacttcaa atgcccgcaa
18301 tcgggggccg cgctgccggt cgagaacccc gaggtgtggg tttaccaaga cgacctgaac
18361 gaccccgagg agcccgcatg aacacgcaca gccaagttgg caaaaccgct gccaacttcc
18421 ccccgatttt cagagacgtt ggcaagttgg caaacgcctg ccaacttgaa tcccatactg
18481 atcaacgcgt tagggagttg tcggcagatt ggcaagttgg caaggctgcc aacttgccaa
18541 cttccccaaa cccgcgtggt tgctgggttt ccggagattt ccaagttggc gaaaactccc
18601 tcccccctac ggggggagag gaacacgcgg tgtcctctcc cccgacccgt cggggtctgc
18661 ccggcatcga cggtcgggat cggggaccgg gcacggccat cctgagcctc gacctgggca
18721 cccggaccgg ctgggcgctg ctcggccgtg acggctccat caccagcggc tcggagtcct
18781 tcaagccccg gcgcttcgag ggcggcggca tgcgttatct gcgcttcaag cgctggctca
18841 ccgaggtcaa gccatcggcg gacgggctgg atgcggtgta cttcgaggag gtccgccgcc
18901 acgccggagt ggatgccgcc cacgcctacg gcggcttcat ggcccagctg accgcctggt
18961 gcgagcacca cggcatcccg taccagggcg ttccggtggg cacgatcaag aagcacgcca
19021 ccggcaaggg caatgccggc aagcaggaga tggtggccgc catgcaggcc ttgggtttcc
19081 ggcccgagga cgacaacgag gccgacgcgc tggcactgct gatgtgggcg atcgcgacgc
19141 aggaggtgcc ggcatgaatg cacccagccc caactatcgt tgccccctcg ggcgcctgca
19201 accgactcgc ccggacgtcg atgccataaa acgcgacggc tggcgggacc agggcatcct
19261 ggtggtctcg ctcgacgacg agcggctcga ctggatcgag cgggagctgg tgaagcgcat
19321 cggcgagcgg ctctacggtc gccagggaga cggccatgtg gagcgttgac gacgtcgccg
19381 agcgcttccg ggaggccgcc cagaccgcgc ggcgtctgcc gccggtacgc gtccagggct
19441 acttcaacac ctggccggcc atcctgcgcc agccgtggga gacctactcg ggcgacgacg
19501 tgctgtaccg ctttccgccc gacccggccg ccatcgaccg catggaggag acgatgcgct
19561 gggtgctgtg gctcaccgag gagcagcgcc atctcgtctg gatgcgcgcc gaggaacgcg
19621 ggtggcggga gatctgccgg cgcttcggct gcgaccgcac tacggcctgg cggcggtggc
19681 agaaggcgct cgacatcgtc gcctgccgtc tgaacgagca gacccgccgg aacgtggcca
19741 gcctttcatg agtgaagttg cgggtcgttt ccaaagtgat ccaagccatg cgggcggatg
19801 cggaacgatg ccggtttcga gccattttgg acgtgcaaca cttcacccgt ttttgcgcta
19861 ggatttcgct aacctcgcga gagaagcatg tgcgaaggcc acggagcgat ccgtggcctt
19921 cgtcgtttct gccttcgcga tccgacccgc cgagcgcgat gggtccttcc tggccgaaaa
19981 gccatgcggg gggcgcgagc gcgatgcttt tctagcgtca gagtgcaaac cggggtttgc
20041 acggtttgcg gtttgcaggc cccatccctg gggcctgccg cttcgcggcc agcctgcggc
20101 tgtccaaatc cgctccaggc ggatttgtgc actccccgat acacatcacg agcccgccca
20161 cggttctccg tcggcgggct ttccatttcg aggttccgat cctgaacacg ctcaacgtcg
20221 agtaccgcaa ggtcgagacg ctgattccct acgcccgcaa tccgcgcacg cacagcgacg
20281 agcaggtcgc gcgcatcgcc gccagcatcg ccgagttcgg ctggaccaac ccgatcctgg
20341 tcgatggcga ccatggtgtg atcgccggcc atggccgact gctggccgca cgcaagctgg
20401 ggctgaccga ggtgccggtg atcgagctcg cgcacctcac cccggcgcag aagcgcgcct
20461 atgtgatcgc cgacaaccgg ctcgcgctcg atgccggctg ggatgaggcc atgttggcgc
20521 tggagttcgc ggagttggcc gacgccggct tcgatctgga cctgaccggc ttctctgcct
20581 ccgagatcga aggcctgctc gaccacatcg aggagacgga accgtccgcc gatgaggacg
20641 agcgtgcgcc ggaaggcgac gcggacgagg acgacgtcac gccgcccacg gttgcggtca

20701 cgcggcccgg cgacttgtgg ctgctgggcg aacaccggct gctctgcgcc gacagcagcg
20761 acgcggccgc cgtcgcgcgc ctcatcaatg gcgagcgcgc tcacctgctc tttaccagcc
20821 cgccctacgc gaaccagcgc gactacacca ccggcgggat cactgactgg aacgcgctca
20881 tgcagggcgt gttcggcgcc gcccgcagcg cgctgcacga ggacgcgcaa atcctggtca
20941 acctcggcct cgtccatcgc gacggcgagt ggcagccgta ctgggacggc tggatcgaat
21001 ggatgcgcac tcagggctgg cgccgcttcg gctggtacgt ctgggatcag tcggtgaccg
21061 tgcccggcga ctgggccggg cgcctcgcgc cgcgccacga gttcgtgttc cacttcaacc
21121 gccaggcgcg caagccgaac aagatcgtgc cctgcaagtg ggccggtcac gagacgcatc
21181 tgcgcgccga tgggtcatcc accgcgatgc gcgccaagga tggcaaggtc ggcgaatgga
21241 accacgccgg acagcccacg caggagttcc gcatcccgga ttccgtcgtc gaggtgacgc
21301 gccagcgcgg ccgcatcggt gatggtatcg accatccggc cgtattcccg ctgggcctgc
21361 cgaagttctt catcgaggcc tacaccgacg tgggcgagat cgtcttcgag ccgttctccg
21421 gcggcggcac cacgctgctg gccgggcaac tcaccgaccg cagggtccgc gccatcgagc
21481 tcgcgcccga gtacgtcgac gtcgcgctgc gccgctggct gcagcaccac ccgggcatgg
21541 agccggtgct cgcggccacc ggccagccct tcgccgaggt ctcggccgag cgcttgggcg
21601 agacggcgga ggcggccgcg tgaactggtt cgccgagcgc atcgagcact ggccgatcga
21661 caagctactg ccctacgcgc ggaacgcccg ccagcactcg gacgagcaga tcgcccagat
21721 cgcggcctcc atcgccgagt tcggcttcgt caatccctgc ctggtcggcg ccgacggtgt
21781 gctggtcgcg ggccatgggc ggctcgccgc cgcgcgcaag ctgggcctgt ccaccgtgcc
21841 ggtggtggtg ctcgatcacc tgacgccgac gcagcgccgg gcgctggtcc tcgccgataa
21901 ccggctcgcg gagctctcga cctgggacga tgtgctactg cgcatcgaac ttgaggcact
21961 gcaggacgaa ggcttcgacc tcgatttgac cggtttcgat gccgatgcgt tggccgagct
22021 actggccggt gaggagccag agcacgaagg ccagaccgag gacgacgccg tcccggagat
22081 tccggaagag ccggtgtcca agccgggcga cgtctggagg ctggggccgc accgtctggt
22141 ctgcggcgat gcgaccgcgg cagaggccta cgcgcagctg ttcccggacg gcgagcgggc
22201 ggacatggtc ttcaccgatc cgccgtacaa cgtgaactac gccaacagcg ccaaggacaa
22261 gctgcggggc aagcaccgtc ccattctcaa cgacgcgctc ggcgcaggct ttcacgactt
22321 tctctacgac gcgctgtcgc tgctcgttgc ccacacccgc ggcgcgatct acgtcgccat
22381 gtcgtcgagc gagctcgaca ccctgcaggc ggcgttccgc tctgccggcg gccactggtc
22441 tacgttcatc atctgggcga agaacacctt cacgctgggc cgcgccgact accagcgcca
22501 gtacgagccg atcctctacg gttggcccga gggcgcggag cgccactggt gcggcgaccg
22561 cgaccagggc gacgtctggc agatcaagaa gccgcagaag aacgacctgc acccgaccat
22621 gaagccggtg gagttggtcg aacgggcgat ccgcaattcg agtcgtcccg gcgacgtggt
22681 gctggacccc ttcggcggtt ccggcaccac cctgatcgcc gccgagaagt cggggcgcgt
22741 ggcgcgcctg atcgaactcg acccgaagta tgcggacgtg atcgtgcggc gctggcagga
22801 ctggacgggc aagcagacca cccgcgaatc ggatggcctg gcgttcgatc aggcggcgac
22861 ttcctcctcg acgatctcgc agtgaatcac gaacccggtc agataaggaa gcccgcgcgg
22921 gataccgtag tccttactgg tgcggcgtcc gatggtccat tccatccact gccgggtggc
22981 ggcgcggatc gcgtcgtgga gggcatggcc cgcatgcttg cggttgagga cttcgtccgc
23041 gaagtggcgt ccgtggcggc tgtcgaggaa ggcccgcacc gcttcgaagg gctgtcccgt
23101 ggcgtcggag atggcgttca tcgcgatggg ccaggcggct tgcgcgtgcg cgtccatcgt
23161 gccccagaag ccccaggctt cgttgcgggt ggcggggatc gttttggctg tggtcatcgt
23221 tgtctccttc gttgatcgtt gcgacacacg tatgaacgcg ctgttcgatc aggaagccaa
23281 gctcaatctc aatcatctcg cgtcgtctgt tggcgaccgg ttacggcggc cagcttggcg
23341 cgggccgacg cgctttccca gtggcagggt ccgttgccgt gcgcagcctc ttcgctccag
23401 cgggccagga tttcgtcatc gctccagccc ttggcggtga ggtaggcgta gtcgtcggcg
23461 tcgtagttgc tgtgggtgag gatggcgcgg gtgggtttca tgctgtgctc cttggcgtgg
23521 atggttgcga cacccgtatg aacgcgctgc cggcgcggga agccaagcgt tttctgcttg
23581 gcttcccatt ccgctcgaat cacgcgatcc ggtagatgcg ctcgccgccc tcgggcttgt
23641 ccgaggtgat ggtcagcccc agcttcttct tgaaggcgtt ggcaaaggtg ccgcgcaccg
23701 tgtgcgcctg ccagccggtg agttcgcaga tctggcgcac cgtcgcgccc tccgggcgcc
23761 ggagcatcgc gatgacttgg gcctgcttgc tgttctcgcg ggtgcggggc ttggtctcgg
23821 cgcgctgcgg tgcccaggtg gcttcggcgg ctgcgacctc cgcctcgaga tccgtgtttg

23881 cctccaccgg cgccggcgcg ggacgcgggc gccccagggc ctcgtaaccg tcggcggcga
23941 caaaccagtc gggaccaatg gtggtgatca gggcccgatt gcagaggccg tccagcacct
24001 tcttgcgggc gccgcctttc acgttgtcgg ggaaccactc gatcttgcca ccggtgtgct
24061 cgacggcgta ggccaggatg gcgtgctggg cgggggtgag ttggatggtg ctcatctgat
24121 gctccttcgt gatggttgat ggtgtggtca tgaacgcgct gttctctgga gaagccaagc
24181 gctttccgct gctttctcag ccctgtcgcg cggcctgccg gcctgcctgg taggcggcca
24241 tcaaggcgct cttgacggcc cagacgctga cctcgtggaa gtccagccgg tcgctgttgc
24301 gcgtttccag ggtgtcgatg aacaggtgat ccagcgcgat ctgctgcagc tgccggtcga
24361 tgtccttttc ggcttgctcg gtcatgtgct tctcctcggg gtgtcgttga tggtgatgac
24421 atgaacgcgc tgttcccgag cgaagccaag cggaatccga gcgggacggc gccaatgctt
24481 gatggagacc atgggactgt cgattcgcgc ctacgcccgc caccgtgggg tgtcggacac
24541 cgccgtacac aaggccatcc gcgccgggcg catcacgccc gaggcggacg gcacgatcga
24601 tccggacaag gccgaccgcg actgggcaag gaactccgag ccgcccaagg agggaacggg
24661 cgccaaagcc gcgaaggtgc gggtgtcgga cgatccggcc ccgaacctcg ccgccgggct
24721 acccgcgggc ggcacgtcgc tgctgcaggc gcgcacggtc aacgaggtgg tcaaggcgca
24781 gaccaacaag gtgcgcctcg cccgcctcaa gggcgagctg gtcgatcgca accaggccat
24841 cgcccacgtg ttcaagctcg cgcgcaccga gcgcgacgcc tggctcaact ggccggcgcg
24901 catctcggcg cagatggcgg ccaggctcgg catggacgcg cacacgctgc acgtcgcgtt
24961 ggaggcggcg gtgcgcgagc acctcgccga gctcggtgaa ctgaaggtcc gcgtcgattg
25021 atgaaacaca tccctgtgtt tcaccctgcc gggcgcgctg gcgcgcgtcc aaattcgttc
25081 ccgacgaatt tgtgatggac gacttcgtct acgagggctt cgacgccatc gagcgcgcct
25141 ggcgcgaagg actgacgccc gatccgctgc tctccgtctc cgagtgggcc gaccggcacc
25201 ggatgctctc cagcaagtcc tcggccgagc cgggccgctg gcgcaccagc cgcacgccgt
25261 atctcaaggc gatcatggac tgcctgtcgc cgacctcgcc ggtcgagcgc gtggtgttca
25321 tgaaaggcgc acaggtgggc gccacggaaa ccggctcgaa ctggatcggc tacgtcatcc
25381 accacgcgcc tgggccgatg atggcggtct ggcccaccgt ggagatggcc aagcgcaact
25441 ccaagcagcg catcgacccg ctgatcgaag agtcgccggt gctctccgag ctgatcgccc
25501 cggcacgctc gcgcgactcg ggcaacacca tcctcgccaa ggagttccgc ggcggcgtgc
25561 tggtgatgac cggcgcgaat agcgccgtgg gcctgcgctc gatgccggtg cggtatctct
25621 tcctcgacga agtggacggc tacccgctcg acgtcgaggg cgaaggcgat gcgatctcgc
25681 tcgccgaggc gcgcacgcgc accttcgcgc ggcgcaagat cttcatcgtc tcgacgccga
25741 ccatcgccgg ggcgagcgcc gtcgagcgcg agtacgaggc atccgaccag cgccgctact
25801 tcgtgccgtg cccgcactgc tcccaccgcc agtggctgcg cttcgagcag ctgcgttggg
25861 agaggggccg gccggagacg gcggcctatg tgtgcgagtc ttgcgaccag ccgatcgccg
25921 agcatcacaa gacctggatg ctggagcacg gcgagtggcg cgcgatggcg ccggagaacg
25981 gcatcaagac ggcgggcttc catctttcgt cgctctacag cccggtaggc tggcgctcct
26041 ggcgcgacat cgcgagcgcg tgggagagcg cggtcagcaa ggagtccggc tcggctgccg
26101 cgatcaagac cttcaagaac accgagctcg gcgagacctg ggtcgaggaa ggcgaagcgc
26161 ccgactggca gcgcctgatc gagcggcggg aggactaccg catcggcacc gtccccatgg
26221 gcggcctgct gctggtgggc ggcgccgacg tgcagaagga tcgcatcgag gcctcgatat
26281 gggccttcgg gcgcggcaag gcgtcctggc tcgtcgagca ccgcgtgctg atgggcgaca
26341 ccgcgcggga tgcggtgtgg aagcggctgt ccgagctgat cgacgagacc tggacgcacg
26401 actccggcaa ccaggttccg ctggcgcgct tcgcgctcga caccggcttc gccacccagg
26461 aggcctacgc cttcgtgcgc gcctgccggg acagtcgact gatggcgatc aagggcgtcc
26521 cgcgcggcgc ggccctgatc ggcacgccca ccgccgtcga tgtgtcgcag ggcggcaaga
26581 agctgcgccg gggcatcaag gtgtactcgg tggcggtggg gctcgccaag ctcgagtttt
26641 acaacaacct gcgcaagagc gcggatgtgg ccgacgacgg caccacgccg atctatccgc
26701 caggcttcgt ccatttgccc aaggtcgatg ccgagtacgt ccagcagctc tgcgccgagc
26761 agctgatcac ccgccgtgac cgcaacggct ttccggtgcg cgagtggcag aagctgcgcg
26821 agcgcaacga ggcgctggac tgctacgtgt acgcccgcgc cgccgccgcg gcgagcggtc
26881 tcgatcgctt cgaggagcgc cactggcgcg agctggaaag acaactgggc atcgccgcac
26941 cgcccgagca ggacgaacac accgtaaccg cagaagacgc ccccgactcc gggggcgtcg
27001 tcgtttcggg ccgacgcatc cggcgccggt ccgtcgtcaa gagtcgctgg atgagctgaa

27061 gatgatcttg gaaggcacct accgtaaccc gaccaccggt cgagacgaaa tccgctgcat
27121 ccgttgtgat gcgtcgggac acatgatggg cgtaccggcc ctcgcttccg ccttcgcggt
27181 gggagccctg tccgccgatg gctccgtcgc caccgacgcg gtcgatctgg gtgaggagca
27241 cggctacaca ttgctgctgt ctcgtaagag cggcgtcgcc tcgaagcagg aagcgatgga
27301 gatccagagc tcgatcgacg gcgtgcgttg gctgccggcg gcgggcatct attcggacga
27361 gaacgtggcc tggcacaagc cgccgaacgg cagcgactac ttcaccgtcc agggccgacc
27421 catcggacgc ttcgcgcgca tcgccttcaa gaacggcaac gccgcccaga ccgagctggt
27481 cctcgagctg gccgcattgg cgggtgtctg accatggcct acacagcggc cgatcgcgaa
27541 gcgctggagc gggcattggc gcgcggcgag cggcgcgtca ccttcggcga caagacggtg
27601 gagtaccgct cggtggacga gttgcgcgcg gcgctgcgcg aggtcgatgc ggcgctcgcc
27661 cgcgaggcgg gccgacccaa gctgcgccag atccgggtca ccacgggcaa ggggctttga
27721 tgggcttctg gagccgcatc aagctcgcct tcggcacgac ccccacctac gacggggtgg
27781 gctccgggcg ccgcgcgctc gcctggatgc cgggcaaccc cggcgcggtg gccgcgctgc
27841 tcacgacgca gacggagctg cgcgcgaaga gccgtgacct tgtgcgccgc aacgcctggg
27901 cggccgccgg catcgaggcc ttcgtggcca acgccatcgg caccggtatc aagccgcagt
27961 cgctgatcga ggacccgggc ctgcgcgaga cgatccaggc cctctggcgc gactggaccg
28021 aggaggccga tgcccagggg ctcaccgact tctacggtct gcaggccttg gcctgtcgcg
28081 ccatgctcga aggcggcgag gcactggtgc ggctgcgcta ccggcggccg gacgacgggc
28141 tggcggtggc gctccagatc caggtgctgg agcccgagca cctgccggtg acgctcaacc
28201 tgctggccga caacggcaac gtgatccgcg ccggcatcga gttcgaccgg ctcggccggc
28261 gcgtggccta ccacctgtac cgctcgcacc cggaggacgg cctgctcgcg cccatgtccg
28321 gtggcggcgg catggagacg gtgcgcgtgc ccgccgccga gatcgtgcat ctgttccggc
28381 ccttgcgtcc cgggcagatc cgcggcgagc cctggctcgc gcgggccttg gtgaagctca
28441 acgagctgga ccagtacgac gacgcggaac tggtgcgcaa gaagaccgcc gccatgttcg
28501 ccggcttcgt cacccgcgac cagccggagg acgcactgat gggcgaaggg ccggccgatg
28561 cctcgggcgt ggcgctggcg gggctggagc cgggcaccct gcagatcctc gaaccgggcg
28621 aggacgtgaa gttctcgcag ccggccgacg tgggcggctc ctacgccgag ttcatgcgtc
28681 agcagtttcg cgcggtggcc gccgcgatgg gcgtgaccta cgagcagctc acgggcgacc
28741 tcacccaggt gaactactcc tccatccgcg cggggctgct ggagtttcgc cgccgctgcg
28801 aggcgatcca gcacggcgtc atcgtccacc agctctgccg cccggtgtgg cgcgcgtgga
28861 tggcgcaggc ggtgctggag ggatggctca agctccccgg cttcgcccgg gacgccgcgc
28921 ggcgccgcgc ctggctcgcc tgcaagtgga ttccccaggg ctggcagtgg gtcgatccgc
28981 agaaggagtt caacgcgatg ctcaccgcga tccgggcggg gctcttgtcc cgctcggagg
29041 cggtgtcctc cttcggctac gacgccgagg acgtggatcg ggagatcgcc gccgacaacg
29101 cccgcgccga tgcgctcggc ctcgtcttcg attccgaccc gcgccacgac cgcaacccac
29161 ccgcggcggc ctccgacccg gtgccgccgc aagacatcca ggacctctga catgcagctc
29221 gtacacctgg cgtcccgtct ctacgggacg ccacttctca tcgcgcgttc gaagctggac
29281 gtgatcctct ccgtgctcgg tccccgcatc ggtctgcccg acgttgaagc cgccgtaccg
29341 gcggtgatgc ccgcgagccc ggaggtcgcg gcaccgcccg gcatcaccgt gatcccgatc
29401 cacggcacgc tggtgcgccg caccctggga ctggaggccg cctccggcct catgagctac
29461 ggcgagatcg gcgcccgtct ggatgccgcg atcgccgacc ctgccgtgtc cggcatcctg
29521 ctcgacgtgg actcccccgg cggcgaggcg ggcggcgtgt tcgagctcgc cgagcgcatc
29581 cgtgcggccg acgccgtgaa accggtctgg gcgatcgccg ccgactcggc cttctcggcc
29641 gcctacgcca tcgcatgcgc cgcctcgcat ctcgccgtca cgcgcaccgg cggtgtgggc
29701 tcggtcgggg tcatcgccat gcacgtcgac cagtcggtcc gcgacgccca gcagggctac
29761 cgctacacgg cgatcaccgc cggccggcac aagaacgact tttcgcccca cgagccgctc
29821 gaccaggagg cggcggaacg cctccaggcg gaggtggatc gcctctacgg cctgttcgtc
29881 gggcacgtgg ccgcgatgcg cgggctggac cccgacgccg tgcgcgccac ggaggccggg
29941 atcttcttcg gcgagcaggc gataggcagc aggctcgccg acgccgtggc gagccgcgac
30001 cagctgctcg ccgtcttcgc gaccttcttg aacccgcagg gccgttcgcg gaacccggcc
30061 ccccgcacgc gtcccggttc cgcgtacgcc aaacaggaga acgacccgat gcaatccacc
30121 gatccgaccc ccgaaacccc cgagacgacg ctcgtcgccg ctgctgcctc cgcggcgccc
30181 gtgcccgaga cgcccgccac cgctgcggac cggcgcgagg ccgtggccat cgccgagctg

30241 tgccagctcg gcggctgccc ggagcgcacc gccgagttcc tggccgcagg tctttcggaa
30301 accgatgtcc gccgcgcgct gctcgccgcc cgcgcccatg gccccgagat cggctcggcg
30361 atccatccgg atgcgcatgc cgcgcaccgc gcttcacccg aacacaaccc gctgatcaag
30421 gcggtcaagc acctcaccgg aaaggagtga accatgccga ccctagttga accgatgaac
30481 ctgggtgacc tgctgaagta cgaggcgccc aacctgtact cgcgcgacct tgccacggtc
30541 gccgccggcc agaacctcgt cctgggcagc gtcgtcggcc gcgagaccgc cacaaacaag
30601 ctgaaggcgc tcgacccggc cgccaccgac ggcaccgagt tgcccgccgg cgtgctgatc
30661 gtcgacgccg acgccaccgc cgccgacctc gatacggtca tcgtcgcgcg ccacgccatc
30721 gtcgcccgcc atgcgctggt ctggcctgcc ggcatcacgc ccgcgcagca gtccgccgcc
30781 atcgccgccc tcgaagcgcg cggcatcctc gttcgtgaag gagcctgacc atgcagaatc
30841 ccttttccaa tcccgccttc tcgatggcga gcctcaccgc cgccatcaac ctgatcccca
30901 accgctacgg gcggctggag accctggacc tgttcccgat caagccggtg cgtacccgcc
30961 aggtcgtggt ggaggagatg cacggcgtgc tgaacctgct gccgaccctg ccgcccggct
31021 cgcccggcac cgtgggcaag cgcggcaagc gggcgatgcg cgccttcgtc gtgccccaca
31081 tcccgcacga cgacgtggtg ctgcccgagg aggtccaggg catccgcagc ttcgggcagg
31141 aaaccgagac cgagagcgtg gccggcgtgc tcgcccgcca cctggagacc atgcgcaaca
31201 agcacgccat caccctggag cacctgcgca tgggggcgct gaagggcgag atcctcgatg
31261 ccgatgccac gccgctggtc aatctctaca ccgagttcgg catcacgccc aagaccgtga
31321 acttcgccct cggcaacgcc aacaccaacg tcaagggcaa gtgcgccgag gtgctgcgcc
31381 acatcgagga caacctctcg ggcgagttct cgaccggcgt gcactgcctg tgctcgcccg
31441 agttcttcga cgcgctgacc ggccacgcca aggtcgagga ggcctacaag aactggcagc
31501 agggcgcggt gctcatcaac gatatgcgcc gcggcttcac cttcggcggc atcaccttcg
31561 aggagtaccg cggccaggcg agcgacgcct ccggcaccac ccggcgcttc atcgcggcgg
31621 gcgaggccca cgccttcccg ctcggcaccg tcgacacctt cgccacctac gtcgcgcccg
31681 ccgacttcaa cgagacggtc aacaccctgg gcctgccgct ctacgccaag caggagccgc
31741 gcaagttcga ccgcggcacc gacctgcaca cccagagcaa cccgctgccc atgtgccatc
31801 ggcccggtgt gctggtgaag ctgacggcag cgtgatggcg caggtgacgg atctctacga
31861 ggccgccggc cgcgccgggc tgctcaccga cgtcatggtg ggttcgctca cggtgcagtg
31921 cgtcttcagc gccccggatg agctggcgct cgatgggctc gcactgaacc gcgactacca
31981 cctcgagtac ccgagcgcct ggctcaccct cgccgccggc gacacggtgg agatcgcggg
32041 aagcccgtat cgggtgcgcg aggtccgcca gctgcgcgac ggctccgaga tgcaggccaa
32101 gctgacccgg ctatgacgcc ctccgtccgc gagcgcctga tccgggcggt cgtggcgcgc
32161 atcggttccg cgatcgcacc gacgccgctg catcgccagc cgaccgtgcc gctcccgcgc
32221 gaggcgagcc ccgcgctcct gctgttcatc gaaggcgatc aggtcctcgc gcaggcgaac
32281 gaccggctcg accgcgccct gacgctgagg ctggtcgcgc tcgcgcgcga aggcgacgcc
32341 ttcgacgtgg ccgacgcgat catcgtcgcg gcgcacggcg cgttgatggc cgagccgagc
32401 ctcggcgggc tggctctggg cgtgcgcgag atcgactgcg agtgggacac ggaggacgcc
32461 gacagccaag ccctggccgt gcccgcgcgc tacgagatcc gctaccgcac cctggcctcc
32521 gacctcaccc aaaaaggata gtcaccatgc acatcgaact gatcgaaccg cacacccacg
32581 ccggccgact ccacgccccc ggtgagatcc tcgatctcga cgaggcagcg gcccaatggc
32641 tgatcgagcg cggagccgca cggccggccg acccccaacc ccagacccag atcaagaccc
32701 gtaaaggaga ctgaccatgc cgtacttttc cggacagggg cgcgtctaca tcggcgcccg
32761 cgacgcactt ggcaacccgc aggggctcgc ctacgtgggc aacgtgcccg aactcaaggt
32821 ctccctctcg gtggagaccc tggagcacca ggagtcggtg agcggtcagc gcctcaccga
32881 tctgcagctc atcaagacca agaagggcga gttcgcctgc accctggagg agctcatcgc
32941 gaccaacctg gcgctcgccc tctacggcgc caccaccgcc cagaccccgg gcacggtcac
33001 cgcggaggcg ctgccgaacc cggtcacccc cggcagcctc tacctgctcg ccaagcagga
33061 cgtctcctcg gtggtggtga aggactccag cgccaccccc aagaccctgc cggccgcgca
33121 gtactcggtg aacgccaagc acggctcgct ggtgatcctc gacgccacca ccggtggacc
33181 ctacgtcgag cccttcaagg tcgactacgc ctacggcacg gcctcggtca ccgcgatgtt
33241 cacccagccg ctgccagagc gctgggtgcg cttcgagggg ctcaacaccg ccgacggcaa
33301 ccgcgaggtg gtgatcgacc tctaccgcgt ggccatcaac ccggccaagg agctctcggt
33361 catcaccgac gagttgctga agttcgagct ctcgggccag gtgctcgcgg acacgctcaa

33421 gcccgccgcc ggcgacctcg gccagttcgg ccgcatcgtg ctgctgtgag ggggatgacg
33481 atgagtgctt ccgatctgga tgttctcgtg ccgcagcctc aagttgtgga tctggccggc
33541 caacgcctcg cgatcagccc gctggtgctc ggcgagctgc cggcgatgct caaggccgtg
33601 cagcccttcg cgcaacgact ggcggacgaa ccggactggc tcgccttgct ctcggaccac
33661 ggcgatgcgt tgctctccgg gttggcgatc gccagccgcc aaccgcgcga gtgggtggac
33721 gcgctggccc tcgacgatgc gatcaccctg gcggcgaccg tgttcgaggt gaacgcggat
33781 tttttcgtgc gccggatcgc gccgaaagtg ggcgatctgg cgcagcgtct gaacggccgt
33841 ttggctgggc tgacgccatc gcccgcctga ttcggggcgg tcaccgctac ccggacatcc
33901 tcggctacac gttgggccag ctgaacgcct tcctcgccgc cgatcgccgc ctcgaacacg
33961 aacggctcgc cacccggctt gccgtcatga ccgctgccgc ccagggcagt cgcgacggca
34021 tccgtgagct ccaggccgaa ctccatcgag gcatgcgtga tgaagatcga tctggtcgct
34081 gacggtttgc tggatcggcg gcgcttcagc gcctggcagg gcgacacccg caaggcgatc
34141 cacaccgccg tggcccgcgc gatgcgcgac accggcaagg cgatggccga gcgggcgcgg
34201 ggcgaaatgc gcgccggttt caaggtcgtg aagccgaagt tcctccgctc gatgcacgcc
34261 aaggtgttcg accgcaaggc cgaggaattc ccggccctct acatcggctc gaaagtgccc
34321 tggctgggta tccacgaaca gggcggaacg atccgggggc ggatgctcat cccgctgctg
34381 ccgcagcacc ggcgcatcgg gcgcaaggcg ttcgcccggg tgatcgatgc cttgatgcgc
34441 tccggcaatg ccttcttcat cgaaaagaac ggccggcaga tcctgatggc cgagaacatc
34501 gccgagaacg cccggccgct cgcgcgcttt cgccgtgccg agcgggagcg caccggcgcc
34561 aagcgcgtgc ggcgcggcca ggagattccc atcgccgtgc tcgtgcgacg cgtgagcttg
34621 agaaaacggt tcgacctcgc ccgctcagtg cgaggcgatc ttccccgcct gacggcggcc
34681 atccgtaaag caatgtcgaa ggtttgaacg tggccggtaa ccgtgcccag atcctcatca
34741 ccgccgtcga tgagacgcga cgggccttcc agtccgtaca gggaaatctc acccgcctgc
34801 gcggcgaagc cgcccaggtc ggagaagtgc tctcccgcat cggcggcgcg atcggcatcg
34861 ggctgggggt gcgcgaactg gtcgaggtcg ccgaccagta caagaacctg caggcgcgcc
34921 tcaagctcgc ggtcacctcg caagaggagt tcaaccgcgc cgacgcggcc ctcttcgaga
34981 tcgcccagaa aaaccgcgcg cccctggcag agaccgtcac gctctatgcg cggctcgcac
35041 cctcggtgca ggcgttgggg cgttcgcagg cggacgtgct ggcggctacc gatgccatcg
35101 gtcaggccgt gtcgctctcc ggcgcatcca gcgacgcggc ggcaggtgcc ttgctgcagc
35161 tggggcaggc cttcgcctcg ggccagctgc gcggtgagga gttcaattcc gtcatcgagc
35221 agacgccgcg cctggcgcag gccatcgccg acggcatggg cgtgccgctc ggctcactgc
35281 gggccctggc ccaggaaggc aagatcacct cgaaggccgt gctcgacgtc ctgctcaagg
35341 agcggacgcg cctcgccgag gagtacgcga gcctccccga tacggtgtcg ggtgcgctca
35401 cccgcctcag gaacgccttc cagcgagcct tcggcgaacg cgacgcgagc tcgggtctga
35461 cggcgggact ggcgcaggcg atccagctcg tcgccgggca tctcgagctg ctgatcgact
35521 tggccggtgt cgtgctggtt gccgcgttcg ggcggatggc cggcgccttc gcgaccagtg
35581 ttgctgccgc ccgggcggaa gcggccgcgc gcctggccaa cctgcgcacg ctggaagccg
35641 aggcgctcgc ccgggtgcgg ctcgccgatg ccgccttggc tcaggcacgt gcgcaagggc
35701 ttgccaccgg cgcgctggtc gcggatgcgg ccaaggctcg gctgcaagcc accgctgctt
35761 ccggtgccgt ggctcaggca gtagcgtcca cgtccctgct cggtcgcgcc gcgggtctgt
35821 tgcgcggggt gctcgcgctc ctgggcgggc ccatcggcgt catcgtgacc gccgcggggc
35881 tgctcgcggg cgcactctat tcggcgcgcg acgccgtggt cgagttcggc ggcaggaccg
35941 cctcgatcaa gcagatcgtc gccgccgcct gggacctggt cgtcgagaag gtcggcgaag
36001 tcgtcagagc cttaggaagg ctggtcggca ccaacgatct ctcctgggtc cgcgtgcgcg
36061 cggcgatggt cggcgcgctg aacgccatcg gcacggccgt ccgcgcgatg gtgaacgtcg
36121 tcatcggcgc gttcaacgcc atcggcagcg tcgtgggcat cacggccgcc ttcctggtcg
36181 agcgctttcg taacgccttc tccgacatcg gagagctggc gaaggccttg ggccaggacg
36241 tggcagcggc cttcagcggc gacttttcga tgcaggcgct gcgcgcagca ctcggccgcc
36301 agctcggcga ggtgcgggat ttcgggaagg agttggctgg agccgtgcgc gacgccgtca
36361 cgcgtgacta cgtcggggag gccgcgcaag ccatcgcccg gcgcatccgc cccgagcaaa
36421 cccagccagg cgtcttcggc cgtccgcagc cgcaggcctt gtccgccccc gacaagggag
36481 gcgaagcagc gaagctcgcc ctcgtgcagg cccaggccga ggccgaattc aagctcctca
36541 aggacgcgtt ggaccgtcag gcgcgggcat tggatgcctc cctcgaagac cggctgatct

36601 cgctcaagga ctactacgcg gccaagaccc ggatcgagca gcaggagatc gatgcggaga
36661 tccggcgcgt gcaggtctcg ctcgcggagc agcagcgcct gcaaaagacc ggcaaggacg
36721 aaccggcacg cctcaaggcg aaagccgagg tcgccaagct cgaagcggag ctcaccgtcc
36781 tcaacaacaa gcgcgcggac gtcgaggtcg ccaatgcccg caaggccacc caggccgagc
36841 gcgagctgcg cgaggagctt gccaaggtgc gcgacgaact gctcgacctc accggggcgg
36901 cgaccagcca ggatcgacgc gcggcgatcg agcggcagta ccagacgctg atcgagcggc
36961 tgcgtgccga gggcgacacc gaaggcgtgg tcacggtggg gcgcctcatc gacgtcaagt
37021 cggcggccgc cgatctcgcc gactacgagc gccagttcaa cgacgcgctc gcccggatgc
37081 gtgcatccga ggagtcgatc aacctgcagc gccagtcggg gctactcacc gaatcccagg
37141 cacgaagcca aatccttgcg ctgcatcgcc agacgggcga atcgctcgat gcgctcctgc
37201 cgcagttgga ggccgccgcc gcggccattg gcccggaggc cgtcgcccgg gtacagacct
37261 ggaagaacga gatcgcgcag gtgaagctcg tggtggacga cgtggcggtc gccatcgacg
37321 gggcggtgca ggacggcttc gcgcagatgt tcgaggccat cggcagcggc gccaagtcgg
37381 ccaaggacgc cttcgccgac ttcgcccgct cggtgctcgc cgccatcaac cgcatcgcct
37441 cgcagaagct cgccgaggcg ctgttcggca gcctgttcgg gggcagcggc gcgggcgggt
37501 tcagcctagg atcgctggtc tcgtcgttgt tccagggctt cgccggcggc ggctacgtca
37561 ccggtccggg cacctccacc agcgactcga tcccggcacg gctctccgcc ggcgagtacg
37621 tgctcaacgc cgccgccgtg aagcgcgtgg gcgtggcgtt cctggaggcg atcaacggca
37681 tcgaaggcgg accgcgcatc caggggcccc ggctcgcctt cgccgccggc gggctggtgc
37741 ccgaggcgcc gccaccgcaa ccgcagggcc agggccaggc ggtacgcatc gtcaacgtga
37801 tcgacccggc gatggccgcc gactacctca actcgtccgc gggcgagaag accatcctca
37861 acatcctgca acgcaatgcg ggagccgtca gacaggtgct tgcgtaaatg gcgttcgaaa
37921 tcggcacggc cagcgattac cgcgatctgc tggaccgctt ccatgccttc ctcaccactc
37981 acccgaatct ggtcgccgcg ggccagcagt ggcaggcgct gcactggacg acggacgccg
38041 ccacgaaaga gctcatcctc aaagcgcccg gcctggccgg tgccgaggag atctactgcg
38101 gcatccgggc ctacgagaac gccacggccg gctactacat gtgggacctg aacggcttca
38161 tcggcttcaa cccggccaac gacttctata cgcaaccggg cgcgatcagc ggctggctgc
38221 cgatgatgtc gctctggaac acagcgatcc cgtactggtt cgtggccaac ggccggcgtg
38281 ccgtggtggt ggcgaagatc tcgaccgtct accaggcggc gcacctgggc ttcgtcctgc
38341 cctacgcgac gccgggtcag tacccgtacc cgctgctcgt cggcggctcg atgaccggcc
38401 agcggggccg caactacagc gtgacctcgc ccaaccaccg ccatttcgtc gacccgggcg
38461 aggatggaca gaacaacgcc aacaccgcct gcatgctgcg cggccccctcg ggcgcctggc
38521 tgccgttcca gaacctcgcc tactcgtcgt ccgagtatcg ctacgacggg ccgcgcccgg
38581 tctggccgac gaactacacc tacctcggca acctgcgcga ggcgccggat ggcacctacg
38641 tgctctcgcc catcgtgctc acgcagaaca actccggcac cgatcacgac ctcttcggcg
38701 agttggaagg cgtctatcac gtctccgggt tcaacaacgc cgccgagaac ctgatcacgg
38761 tgggcggcgt ggaccacctc gtggtgcaga acgtgtatcg caccagcgtg cgcgactact
38821 gggcgctgcg cctggagtaa acgaccatgg cctaccagac tggtacctcc gccaacgccg
38881 atcagctgct cgacgccctg cgcgtgttcg cggtggccaa cggctggacc cagctgcgct
38941 gggcccccga cggcaccggg cagaccctgt cgctcgccaa gggcgggctc tacgtccatc
39001 tgcgctccgc ggtgaatgag cgcctctcca cacgctacaa caccatcacc ggcatctggc
39061 tgatcggctc gacgggattc gatgccggaa agccctggtg ggatcaaccc ggatcgatcg
39121 tcaacgccag ctacgtgagc aacaccacga gctaccgcgc cgaagcctgc ggcctgttcg
39181 aggtgggcac cgccaacacc taccacctac tctcggccgc cacgcccgaa ctgatcatgt
39241 gtgtggccga ggtctcgccc ggcgtctatc accatctcgc cttcggggat ctcacgaagt
39301 tcggcagcta cggcggtggg gcgttcgtgt cgggcgcgtt cggcccggac gcctacacct
39361 atacgtacag cggcttcaac gactatgtct tcggctacga ccacgaccgg cacttcggcc
39421 tgccgttcaa cgattacaag ttctacggcg gcgcgaactt cgttctcgcc gaggtggacg
39481 gcgccaccga ctggcactcg gtctgcaagg actgggcgct caccggcaag cgcgccaagg
39541 cgctgtggga acggggcacg ggcacgccgc gcgacagtct ggcgcgctac tggtgggggc
39601 acgtgcccaa caccttgaac ggcgtgacgc cgatgctgcc gttctacctg ttcgtggcgc
39661 ggccttccgg cttcttctcg cccttcggcc acaccgctca cctgcgctac ctcaacatca
39721 cccactacgc gccggcggag gccttcgcgt tgggcgccga gcagtggatg gccttcccgg

39781 cccactccaa gaacggcaaa agcggcgtgc acggctatgc cgtgagactc atcccctaag
39841 ccctaagcgg atcatgccga cctttcccgg ttccatcctg cccggtgccg cccgccagga
39901 gccgccgacc tggtcgcgct cgccggaggt ctccgagcgc gtgccggtcg cctttccgtt
39961 cgaccagagt gcgagctcgg tcatgcgtcc gggcgcgcgg ctcgacaccc agcccgtcga
40021 cgagctgcgg ctcggcttcg caggcgctct gctgcccacc tttggggccg actggtatca
40081 ccgcatccac gtcatccctt cgaccatcga tctcggcaac ctggtgagtc cggtcgagcg
40141 ggtgctggag gtgtggaacg ctcggttcga cgcgcagacg ctcgatgcca tcgacgagac
40201 cggaacggac gggctgctgc tctcgggcca gccggcgccg ccgctcgcct tcgggccgct
40261 gcagtcgcgc ctctacacct tcgcagcggg cacgcgcggg gcgccggtga tcgacgccgc
40321 ctaccgcttc gtctttgccg gcgggctcac cgcattgctg gtcgtcaccg gccgccgcgt
40381 ggtggtcttc ggcatgcgtc cggactggtc gcaagggatc accgagcggc tggaatggct
40441 caccgaggtg ctggaggcct acgacggcac cgagcagcgg gtgcgcctgc ggcagctgcc
40501 gcgacgcggt ttcgagtatg gattcctgat cgagggccgg gacgcccagg tgctggatca
40561 cctgctgttc gcctggggcg cacgcatcta ctgcctgccg gtgtggaccg acgtctccac
40621 ccttgcgggc gaggctgccg tcggcagcac cgcgctcacc gtgcaggatg ccgccaacag
40681 cgactaccac gcgggcggtc tggcggtact gtggcgctcc aacacgcggc acgaggcggt
40741 ggagatcctc tccatcgccg gcaacacgct gaccctcaag ctccccctgg ccgggagctg
40801 gccggccggc acgcgggtgt tcccggcgcg gctcgcccgc ctcgagggcg aggtggccgt
40861 ggcgcgtccc accgacacca tcgccgtcgg ccgctgccgt ttcggcatcg aggacatcac
40921 ggcgcccgcg gtcgccgact atggcccggc ctatcagggc taccgggtgt tcgactggcg
40981 gcccaaccgg agcaccgacc ttgaggacag gtggcttcgg cgcctctcca tcatcgacta
41041 cgggacggga ctgccgacct tcgacgacga gtcgggctcc ccgctcatcg gacgcacgct
41101 gacctggctg ctcaccgacc ggagcaaggc gaccgccttc cggggctggc tcgccgcacg
41161 cgcgggacgg gcgaatccct gctggttgcc gaccttcgag tccgacctcg aagtcagccg
41221 cacggtggcg gccacggatg ccggcctcgt cgtccgcaac gtcggctacg cccgcttcgt
41281 cgccgccgat cccttgcggc gcgacctgcg gctcctcacc acggccggca ccttccatcg
41341 ccgtatcacc ggggcgaacg agatctccga ggacgaggag ctgctctccc tggacgcgcc
41401 gctgggcgtg accctcgatc cccagcagtt cctgcaggtc tcgtacctgg agttggcccg
41461 gctcgaccag gacgcggtcg agctgcattg ggagacggac gccacggcgc gcgtgcagct
41521 ctccacccgg acgttgagat catgagctaa gaccatgagc tatctgggca tcgaacagtc
41581 cgcccacggc ggacaaccgc aggagctcta ccgcttctcc cagggcgcgc agcgctggct
41641 ctacacctcg gggcaggtgg cggtggacta ccaatccgag acctatcagc cggcgacgat
41701 ctcgcgcggc ggcctcgagc agagcaacga gctcgcgcgg ctggggctgg agatccgcat
41761 gccgcggagc cttcccgtgg cgagtctatt tctcgccgcc ccgcccgagg gtgtcgtgag
41821 cgttaccctc taccggcgcc atgtcggcga cgccgagttc atcacctact ggaaggggcg
41881 gatcaccggc gcgcggctgt ccggcgccga ggccaccctc aagtgcgagc ccatcgcctc
41941 gagcctgaag cggccgggct tgcgcgcccg ctaccagctg ctgtgccgcc acgtgctgta
42001 ctccagcgga tgcggcgcgc tgaaggacag cttccgcgtg gacggcaccg tagcggcggt
42061 gagcggcgtg accgtccagg tcgccgtcgc cgccagccgg ccggacggct acttcgtggg
42121 cggcatgctc gccaccaccg ccggcgcgcg catgatcgtc ggtcacgccg ggatcgacct
42181 caccctggtc gctcccatgg tcggcctcac ggcgggcgat gcggtccagc tctatgccgg
42241 ctgcgaccac acgatggcgc attgcaagga caggttcggc aacctggaca acttcggcgg
42301 cttccccttc atcccggtga agaacccctt caccggcgac gccatcgtgt gaggctttcc
42361 catgtggcaa cagatcatca tttgggtcgt caccacggta ctgtccgcgc tgctcgcgcc
42421 gcgccccaag gtgcaggacg cccagcccgg ccagatcggc gacaaggacg tgcccatcgc
42481 ctcccaggac gcccccatcc cggtgctgtt cggtacgcgg gtgatctccg ggcccaacgt
42541 ggtctggtac ggcgacgtgc aggtgcggcc gatccgcaag tcctccgggg gcaagaagtg
42601 accgaggtca tcgctcggct ggagcatgcc cgtgccctgg gctactgcgc ccgcggcatg
42661 cgtcgctggt tcgaggggcg cagccacacc tgggccgagt tcgtggagcg aggggtcccg
42721 gccgactggc tgcgcgccac gggtgacgcg atggcgatcc gcgtcgccga ggaagccgac
42781 aaggcccaaa gggccgcagg acagcacgaa gtgctggtcc cgagcgcagc gagggatgcg
42841 cgaagcgcac agaaggagca ggcaggatga gcggcggcgg caaaggcagt caggagtaca
42901 cggtcggcta ttggtacggc ctcggcgccc acctcgccct gtgccacggc ccgctggacg

42961 ccatcaccga gatccgggtg ggcgagcgcg tcgcctggtc gggcaacgtc accggcaaca
43021 ccacgatcac catcgacaac cccaatctct tcggcgggga ggagcgcgag ggcggcgtgc
43081 aggggccggt ggacatcctg atgggcgggc cgacccaggg acggaacgcc tacctgcagg
43141 agcgcctcgg cgccgacatc ccggccttcc gcggcgtggt gtcgctgatc ctgcgccggg
43201 tgtgggtcgc ggccatgaac ccctacatca agccctggtc ggtgcgcgcc aagcgggtgc
43261 cgaggcagtg gtacgcggcc aaggccgaga tctccggcga cgccaacccg gcccacatcg
43321 tgcgcgagtg cctcaccaac ggcgagtggg gcatgggtta cccgacgagc gacatcgacg
43381 atgccagctt catggcggcc gccgacgcgc tccatgccga gggcttcggg ctgtcgctgc
43441 tgtggaacaa ggaagagacc atcgaggact tcatcctgtc ggtgctccgg cacgtggacg
43501 ggctgctcta cgtccatccg cgcaccgggc tcttcaccct caagctcgcg cgcgacgatt
43561 acacgatctc cagtctaccg atcttcgacc ccagtaacat cctgcgcatc gaggagttca
43621 cgcggccctc ctggggtgag atcaccaacc aggtgaccgt cgtctaccgc gacggcgtca
43681 cggacaagga cggcagcgtc acagtgcagg acatcgccgc cgtgcagctc aacggcggcg
43741 tggtggcgac cacggtcaac taccccggca tcagccgagc ggagctggcc aaccgggtgg
43801 ccatgcgcga gttgaagcaa ctcgtaagcc ccctcgccaa atgcaccttc gtcgccaacc
43861 gccaggcctc cggcctcaac atcggcgacg tggtgaagct ctcctggccg ccctacggca
43921 tcgaccagat ggtgatgcgc gtcgcgcgca tcgcctatgg ggagctggcg aacggggcgg
43981 tgcgggtgga gtgcgtgcag gacatcttcg gcctgccgca gtcggtctac tcggcgccgc
44041 ccccctcggg ctggacggag ccgaccagcc tgccggcgcc gtgccccccac cagaccctgt
44101 tcgaggtgcc gtactggtcg gtggtcaagg acttcaccgg cgagtcccag agcctgctcg
44161 gcgacatcga cgatctcgac ggcctggtgg cagcctgcgg ttcgcgcccc tcgtcggacg
44221 ccttcggctt caaggcactg gcccgcgtca gcggcagttt caccgagaag ggcttcggca
44281 tcttcacgcc cacggcggtt ctcacggcaa tgctgccgca gtcggccgcg caggtgagtg
44341 tggggctgac ctccggcatc ggcctcgagg aggtgactgc cggaggactc acggtgatcg
44401 acggcgaatg gctcaaggtg gtgtcgctca acctcgcgac ccagaccgtc accctggagc
44461 gcgggatgct cgatacggtg ccggcgagcc accccgcagg cagccgcatc tggttcgtcg
44521 acggcttccg ccactacctc acgcccgagt acgtcgccgg cgagacggtg cgcgtgaagc
44581 tcctcacccg cacggcacgc ggcacgctgc ccgaggcggc ggcaacggag atgagcctgc
44641 cactcgacaa gcgcttcatc cgtccgtact gccccggcaa cgcccagatc aacggcaagc
44701 gctatccgac cgtggtggcc ggcgagatca acgtaagctg ggccacgcgc aaccgccagt
44761 cccaaaccgc ctacctcgtc ctgcagaccg agggggcgat cacgccggag gcgggccaga
44821 ccacaaccgt gcgcttctac aacgagaatg ggcagctcgc gcgcacggtg agcggcatca
44881 cgggtaacgg taccacctgg ccgctggcgc aggagctcgc cgactccggc ctcgggcgcg
44941 tcaacgcgca cgtcaaggtg gagatcgagg cgagccgcga cggccacgtg tcctggcaga
45001 aacacgtcat cgaattcgac cgtaccggct acggcctgcg ttacggcgac tactacggag
45061 gtgtctgatg gccttgaatg acccgaacct cggcctcgcc tacggctggg cccagggcga
45121 acacaactgg aacggcggca tggacgccaa cctgaagcgg ctcggcgccg tggtgggcct
45181 gtcggtcaag gaccgcgacc tcgccactcc acccgcgaca cccgtggacg gcgatcgcta
45241 catcgtcccg gcagcggcca ccggcgcctg ggcgggtcgc accgaccaga tcgccgtgcg
45301 catcgccggt gcgtgggagt accacgcccc caagatcggc tggacctgct tcgtcgagga
45361 cgagggcgtg ctctccgtct acaaggcgac cggctggagc cccggcatcg ccgtctgatc
45421 gcattctctt tactgcatcc accgaacccg ccgcccggcg ggttcttcgt ttctggagac
45481 cagcaatgag cccacccact ctgcaagacg gcatggtcgt catgccgcgc gacgagttcg
45541 aggagctgct cgcacgcgca gccgaacgcg gcgcgaggcg cgcccttgcc gacgtcggcc
45601 ttgacggcga ggacgccgcc cacgatatcc gcgagctgcg cggcctgctc gaagccttca
45661 acgccgccaa gcacaccgcc tggcagaccg tgatccggct cgtcaccacc ggcttcctcc
45721 tggctctggt cgcgggcgcc gtcatcaaac tcaaggtgtt cggaggtggc caatgatcga
45781 gaccctgctc ggcggcctcc tcggcggggc cttccgcctc gcgcccgaga tcctcaagtg
45841 gctggaccgc aagggcgagc gcggtcacga gctggc
//

```
LOCUS       McNor-R3             41154 bp    DNA     linear   PHG 16-FEB-2025
DEFINITION  McNor-R3.
ACCESSION   McNor-R3
VERSION     McNor-R3
KEYWORDS    .
SOURCE      .
  ORGANISM  .
            .
FEATURES             Location/Qualifiers
     CDS             complement(3..362)
                     /ID="RKPNQLQJ_CDS_0001"
                     /transl_table=11
                     /phrog="7250"
                     /top_hit="No_MMseqs_PHROG_hit"
                     /locus_tag="RKPNQLQJ_CDS_0001"
                     /function="unknown function"
                     /product="hypothetical protein"
                     /source="PHANOTATE_1.5.1"
                     /score="-13.066090625074477"
                     /phase="0"
                     /translation="MTVQKFFVNDLNAIVDAVRTTSDALPEPLAKPGIGNAVGIGTRRK
                     TAGIASPLTEKTAQNAKGESWADREYYPARDLKTAGGQFVVKYAAIKTLKFKDGNGDVV
                     EFHFAEPDPDKPLGV"
     CDS             complement(432..857)
                     /ID="RKPNQLQJ_CDS_0002"
                     /transl_table=11
                     /phrog="No_PHROGs_HMM"
                     /top_hit="No_MMseqs_PHROG_hit"
                     /locus_tag="RKPNQLQJ_CDS_0002"
                     /function="unknown function"
                     /product="hypothetical protein"
                     /source="PHANOTATE_1.5.1"
                     /score="-36.65581052126252"
                     /phase="0"
                     /translation="MPTFALYTDAALTQLLAGNLVATQNADGSTPPVVFTLYLGSPTAN
                     RKIQANANPGVDPIQATVVDANTGSGHSASEVKLAATQAGLAAAVGGSALDLGTEILSG
                     AANAKPIWIEVNDATHAVGTATELAVVIANVRETATA"
     CDS             complement(861..3293)
                     /ID="RKPNQLQJ_CDS_0003"
                     /transl_table=11
                     /phrog="5842"
                     /top_hit="No_MMseqs_PHROG_hit"
                     /locus_tag="RKPNQLQJ_CDS_0003"
                     /function="unknown function"
                     /product="hypothetical protein"
                     /source="PHANOTATE_1.5.1"
                     /score="-9309711.596147688"
                     /phase="0"
                     /translation="MTLPVGGVTGIGGVASAAATAAPAAEVYSIAASIGQPIYVEASLV
                     AAIEQTTLDVYAIAASLHSQVYARLDLSAGVAQAVVVFEVFDAIAPIRQPIYAVTGFEA
                     FVEQVGFDGYAVAAPLEQRLWARFNLTAPVEQSQFRLFDASVVIEQRLRAEAYRLQAPV
                     THRVKAVFTLSAGLAQTVADPVFDLDATVEQAVYAFIANLTASLSQTQYDAQAVGGGAF
                     VGQSQAASADITAWAVKAVIGNAAGDGEPVPPEAGAVMGALLGRMTGTLTVDGEEGAAR
```

IAEFTVLPAPGPIHIHTLTGLPITLFLVTAGVELPIFQGILDVPGWDPVQGLLHLSCTD
NLQSRFDGLKRKEIAGIIGGRWSPYVFDKHADEWQYVLDRLSTLPVSYDLDLNLQGRIT
PWQARATADFTFRPDAVIENSVRIDMASARQLVNAIDVHMDYRYERLMQREYRYHWELS
LDQLIDQGSTAPTPETVMQAIDGTGWAVLAEGGTPLLRMTPLPDPQWLHGVMYLSDCPR
YSLAGGVSAILAQRWTQTVTEQWHVTVDAPDSIRVIGKRHGTTTANFDAQSDKDPRYAH
WDRTTEQTVKLELHDGAPIGGYACDSWIPYHPLQVRTYTVPVPAGAQRFATLDPIANPL
GDLYYDLDDGAADGRAGLSNAYMTGIERARRDILSSHRQTTVTFDTLIAPLIDRTHTVR
MASPRLTAKGKVRRVTHSIDFDHGAAITTLALAVSKSYGVGTVDTGFDFPAPAKPPATQ
APLKHPLATPAIGYDPANGEFTITVPGVEQQHIDATTTAPADHLPIAIPDDELILEA"

CDS complement(3293..4033)
/ID="RKPNQLQJ_CDS_0004"
/transl_table=11
/phrog="No_PHROGs_HMM"
/top_hit="No_MMseqs_PHROG_hit"
/locus_tag="RKPNQLQJ_CDS_0004"
/function="unknown function"
/product="hypothetical protein"
/source="PHANOTATE_1.5.1"
/score="-106.02926590380032"
/phase="0"
/translation="MLKLAGLDGVKTVLTAALGAADTWLMAAAAQPPWITPPDPLGEYH
YYLTLIDAETPTKWERVKVTARNGAGPYQLTIERNIASSSGGAQAFAAGALLQWSPGAK
EIDRWRMVLSASMTGTNALTSTRFLAPFSGSTTGSMAAAFWVNRTGKTLRLSKLVAVLS
TSSTFSGDSIVLSVETGATAPATPSNLQTQVAPGNYGFNTDGGYVLDVAPDAPVCFMLS
RANPGAAVSGAFQLAHVSVLAEEL"

CDS complement(4035..4382)
/ID="RKPNQLQJ_CDS_0005"
/transl_table=11
/phrog="5314"
/top_hit="No_MMseqs_PHROG_hit"
/locus_tag="RKPNQLQJ_CDS_0005"
/function="unknown function"
/product="hypothetical protein"
/source="PHANOTATE_1.5.1"
/score="-9.02885880823914"
/phase="0"
/translation="MWSGRSVKQRTWRKLATEISGGGWVPPGLSALDYSQPQVLACASP
LAIVGGGTPPVPVRTEGDFATVTLNVDGVDITYYYPKITVYCDPPEQQLDGTTGNYSWR
LSAEMVDPIGG"

CDS complement(4493..4645)
/ID="RKPNQLQJ_CDS_0006"
/transl_table=11
/phrog="No_PHROGs_HMM"
/top_hit="No_MMseqs_PHROG_hit"
/locus_tag="RKPNQLQJ_CDS_0006"
/function="unknown function"
/product="hypothetical protein"
/source="PHANOTATE_1.5.1"
/score="-0.0876250087549826"
/phase="0"
/translation="LTLSGCCTLEPRVTLVPAPWRCPARAADLLAHIDEAGLVLRCDEP
EPDER"

CDS complement(4642..4740)

/ID="RKPNQLQJ_CDS_0007"
/transl_table=11
/phrog="No_PHROGs_HMM"
/top_hit="No_MMseqs_PHROG_hit"
/locus_tag="RKPNQLQJ_CDS_0007"
/function="unknown function"
/product="hypothetical protein"
/source="PHANOTATE_1.5.1"
/score="-3.0138682373231727"
/phase="0"
/translation="MIVLTRGEVWCAAAVLVSAGVVSGLWVALCAL"
CDS complement(4740..5138)
/ID="RKPNQLQJ_CDS_0008"
/transl_table=11
/phrog="938"
/top_hit="No_MMseqs_PHROG_hit"
/locus_tag="RKPNQLQJ_CDS_0008"
/function="lysis"
/product="endolysin"
/source="PHANOTATE_1.5.1"
/score="-33.19580475791103"
/phase="0"
/translation="MNHIPARTAWAEARGEGEAGMQGVMNVIFRRAQRPGWWGRTPDEV
CLNPRQFSCWNEGDPNKAKAEAVDARDRAFSIALKLESQALKGTLPDITGEATHYFDDS
LLKKPPHWARAMVKTAVIGRLHFFKERD"
CDS complement(5135..5401)
/ID="RKPNQLQJ_CDS_0009"
/transl_table=11
/phrog="No_PHROGs_HMM"
/top_hit="No_MMseqs_PHROG_hit"
/locus_tag="RKPNQLQJ_CDS_0009"
/function="unknown function"
/product="hypothetical protein"
/source="PHANOTATE_1.5.1"
/score="-16.703221044618562"
/phase="0"
/translation="MFEKVKYLYECMQAGKALQDAGVWSRRADLIAKLTALLMAATGLA
EAFGYDLRLSGTDVAAVAQGLGVLAVTLVNVLHVASNKEAGKR"
CDS complement(5444..5719)
/ID="RKPNQLQJ_CDS_0010"
/transl_table=11
/phrog="No_PHROGs_HMM"
/top_hit="No_MMseqs_PHROG_hit"
/locus_tag="RKPNQLQJ_CDS_0010"
/function="unknown function"
/product="hypothetical protein"
/source="PHANOTATE_1.5.1"
/score="-2.901722903051364"
/phase="0"
/translation="VNPILRWALWFVVQAVSGITKEQWGLVQNKVAELETRTIAGLVDK
TTLNDIKKKEAAAYIGTFVSGVRMNVVHFLIEVALWFVRSFGVKAP"
CDS complement(5748..8786)
/ID="RKPNQLQJ_CDS_0011"

/transl_table=11
/phrog="339"
/top_hit="No_MMseqs_PHROG_hit"
/locus_tag="RKPNQLQJ_CDS_0011"
/function="tail"
/product="tail length tape measure protein"
/source="PHANOTATE_1.5.1"
/score="-378287922.42001617"
/phase="0"
/translation="MTTFKTVIEIAANTAAAEKGLGNVAAQVDRLTGSLRRIGQYTVGA
FGAAEALQAARDLGKLSDQYRNLEGRVKLAAGSQNQFTEAQRALFAIAQNNAQAMTGVS
QLYARIAKGAGEMGVSQRQVLSVIDSVAKSFRISGASAEEASSATLQFSQALAAGVLRG
DEFNSIMEQSPRLAQAIADGLGVPIGKLRTLAEAGELTTQKVVEALQKAKASIDRDAAG
LPDTIEQAVVRWDNAALKFVGTSSEITRAAEVIADAINGAAGNIDTIATGVEIAGSVLV
AVLAGKGTAAVAAFAGSQARLVQAHLAATAAARDHALNEAYNARATFAAAEAAVANASG
MARLALVETQLVPASQRLAVAQAEVAASAGPLKIALGGLAAFLGGPVGIAVTLLTAVSA
WHLFGSTAESELERVIRKRRELAKATGQDTRGKSEAELSLLQDEAAVKKQEAVVARLTA
RYKEVGQAADQAFMGGKIGRELAGETALLQEMRAELGKRRQAAADSGNAVKAKEKEVQK
SFEDTRNALKDATAGIEDAYQRRTAAIDALEKLAETRIQAQPIEADAAPPIVYAQNGVQ
ATQARSELEQARQLHALTLQAEGARLKAVRDFAAQRLGLVDQVYAREIAKFKDGEARKT
VLERESLEARRAIYADLESAYTATIDKLIGQEQRLLESARAAAQERRNIEQSTARAVAG
IEQAALTPLEASRAQYAQLQQLLAQQRQELANGNLELSRQAGEQAAGLAESLGQQAAAS
LREQRLNALKGEQTPADPGQPQFGTEDLQRLIDARRQAGQALAEAAARQEQADTQAAAA
VSQKIQETLQNLQSVQAEISDIDQQLTKGFALRVDVDPASLQTLQSRLAELLKPETKTI
SVVMQQQGDGSDTGANGADATLPGFRRGGWIKGYGGGDRIPALLEEGEFVLRKEAVRKL
GLDKLYALNNLSLPRFSIGGYVAQAVPAFDASLARASDGGQPVHIHLPGVPGSFALNGD
PQVVAALKREVARAALKHGRIVR"
CDS complement(8791..8886)
/ID="RKPNQLQJ_CDS_0012"
/transl_table=11
/phrog="No_PHROGs_HMM"
/top_hit="No_MMseqs_PHROG_hit"
/locus_tag="RKPNQLQJ_CDS_0012"
/function="unknown function"
/product="hypothetical protein"
/source="PHANOTATE_1.5.1"
/score="-1.6067045396904793"
/phase="0"
/translation="MTALALIERGHAGVWHYPWRLFLQARLMRRR"
CDS complement(8898..9257)
/ID="RKPNQLQJ_CDS_0013"
/transl_table=11
/phrog="7608"
/top_hit="No_MMseqs_PHROG_hit"
/locus_tag="RKPNQLQJ_CDS_0013"
/function="unknown function"
/product="hypothetical protein"
/source="PHANOTATE_1.5.1"
/score="-9.592736407801237"
/phase="0"
/translation="MAELDTTNPKGLRAERTIEAGGRKIVVRELTVGEVRAWLKDANAE
LDRNDLVALALFADITLDDLTRFSDLSRAELDAMLPSELDKVREAAKSLNPHFFGLRER
LANAAQVAATAPPAT"

CDS complement(9264..9767)
/ID="RKPNQLQJ_CDS_0014"
/transl_table=11
/phrog="807"
/top_hit="No_MMseqs_PHROG_hit"
/locus_tag="RKPNQLQJ_CDS_0014"
/function="tail"
/product="major tail protein with Ig-like domain"
/source="PHANOTATE_1.5.1"
/score="-133.79008741407753"
/phase="0"
/translation="MTTTAMLLTGTIKGAVGAGTPVDLGNAALDLSIEEDTKELADYQN
PGGGSIASLSRIKSVTLKLKLWSISKENLALATRGTVSGNSIEALTQTAEDWHITFDGV
NEVNGDAVTYDFYKVKFSPASSLPGPGTEDFAVLELTGKVLKDTSKTGAGVSQYFKATI
TPAV"
CDS complement(9827..10252)
/ID="RKPNQLQJ_CDS_0015"
/transl_table=11
/phrog="209"
/top_hit="No_MMseqs_PHROG_hit"
/locus_tag="RKPNQLQJ_CDS_0015"
/function="head and packaging"
/product="portal protein"
/source="PHANOTATE_1.5.1"
/score="-9.5761484070296"
/phase="0"
/translation="MFEIEPALIRRLRETLPEFVTVDGVGVLAGVQNLEPLCPAALVLP
LGFAGPVGTPPPNVFLAERQRWQVTVCVAHTPPASTVVTGGAYVLRILRALEHWSPQPG
WGRLKHVGLDDPWFDLGHVEFSLVFEVRPLPLDTGTP"
CDS complement(10261..10773)
/ID="RKPNQLQJ_CDS_0016"
/transl_table=11
/phrog="27"
/top_hit="No_MMseqs_PHROG_hit"
/locus_tag="RKPNQLQJ_CDS_0016"
/function="connector"
/product="tail completion or Neck1 protein"
/source="PHANOTATE_1.5.1"
/score="-14.464554929520139"
/phase="0"
/translation="MSIGLRVRLHGDTAQDLRRLPAVLRTASRRAVGQTARETREAMVR
AIGATYGIPLRALRSRRVQAYLRLAGLRGRVWTGHAPIQAAYVGRLRQEDWGSSAGAYL
FPGSFVARMQSGHRGIFHRVGHRSLPVLEDVVALPKVPALAEVLATRATLRLAALIREM
TQDALPR"
CDS complement(10784..11110)
/ID="RKPNQLQJ_CDS_0017"
/transl_table=11
/phrog="785"
/top_hit="No_MMseqs_PHROG_hit"
/locus_tag="RKPNQLQJ_CDS_0017"
/function="lysis"
/product="holin"
/source="PHANOTATE_1.5.1"

/score="-8.15092988641933"
/phase="0"
/translation="MNARPKQLLLDSPHGNGDDLRLALQIILVTIWSGLVAQLNRWRRH
PRPWRLCLWCLLVDVVSCSLVGFSVWLLAEREGLGYYESLWAAIVAGHFGARWFGLLIH
SSRG"
CDS complement(11107..11415)
/ID="RKPNQLQJ_CDS_0018"
/transl_table=11
/phrog="82"
/top_hit="No_MMseqs_PHROG_hit"
/locus_tag="RKPNQLQJ_CDS_0018"
/function="connector"
/product="head closure Hc1"
/source="PHANOTATE_1.5.1"
/score="-4.996207309135008"
/phase="0"
/translation="MDLEAVNAVCLSTFGEPVILWPDRTEPPPVHLTGIVSVPAGLERP
GPMGAPRPEADPRLTVRSADLPAIHMGDPLKVRGVPYSIASRIDRLDGLTTLILRPR"
CDS complement(11418..12419)
/ID="RKPNQLQJ_CDS_0019"
/transl_table=11
/phrog="29"
/top_hit="No_MMseqs_PHROG_hit"
/locus_tag="RKPNQLQJ_CDS_0019"
/function="head and packaging"
/product="major head protein"
/source="PHANOTATE_1.5.1"
/score="-2935.893705664318"
/phase="0"
/translation="MQNPFHNPAFSMAALTAAINIIPNRYGRLEDLNLMPVKPVRQRQI
LVEERNGVLNLLPTLPPGAPGTVGVRGKRTLRSFVIPHIPHDDVVLPEEVQGIRAFGSE
TELEAVAGVMARHLETMRNKHAITLEHLRMGALKGVILDADGSVLYDLYDEFDIPPKTV
SFQLGTATTDVKARCMEVLAHIEDSLLGEFMTDVHCLCSPEFFAALTGHKDVKTAFTHW
QQGAILINDVRRGFTFGGITFEEYRGRATDVHGVTRRFIAAGEAHAFPLGTVDTFATYV
APADFNETVNTLGQPLYAKQAPRQFDRGTDLHTQSNPLPMCHRPGLLIKLTV"
CDS complement(12422..12799)
/ID="RKPNQLQJ_CDS_0020"
/transl_table=11
/phrog="49"
/top_hit="No_MMseqs_PHROG_hit"
/locus_tag="RKPNQLQJ_CDS_0020"
/function="head and packaging"
/product="head decoration"
/source="PHANOTATE_1.5.1"
/score="-7.965537474983696"
/phase="0"
/translation="MPKLREPKNLGDLLKYEAPNRYSRDLAPVALGQKLALGAVVAREP
AGLRLQALDPAATDASAQAVGVLIEAVDATAAEVPQALLLARHAIVSDLGLVWPAGITP
AQKATAIVQLQALGILVRTGA"
CDS complement(12803..14134)
/ID="RKPNQLQJ_CDS_0021"
/transl_table=11
/phrog="53"

/top_hit="No_MMseqs_PHROG_hit"
/locus_tag="RKPNQLQJ_CDS_0021"
/function="head and packaging"
/product="head maturation protease"
/source="PHANOTATE_1.5.1"
/score="-1740.8802188910872"
/phase="0"
/translation="MTQPLIHLASRLYGTPLLIARPKLEVILAVLGSRIGLPEAATAVP
APMSRTAAPALPGIAVIPIHGTLVRRTLELEAHSGLTSYAEIGARLDAALRDPEVSGIL
LDLDSPGGESGGVFELAAKIRAGTSHKPIWAHANDTAFSAAYAIAAGASRVTLAQTGGA
GSIGVIALHVDQSVKDAREGLTYSALYAGHHKNDLNPHAPLSPQAAAALQAEVDRLYGI
FVAQVAGFRGLPETAVRATEAGLFFGEDAVSAGLADGVLGFDAVLNKFADAIRTRQRLI
SPQARKAAVMSPLPVSLLHSESVMHDHAPGPLDDEAADESRKDADAAGNLTPHAAPSPQ
NDLAAATPVIGEPTAQSLATEAHHAGRSEAQAIAELCLIAGAAPRTAEFLAAGLSEAQV
RRVLLAARAETPEIASRIQVDAGTSVRPEASPVVAAVQKLIARS"
CDS complement(14131..15603)
/ID="RKPNQLQJ_CDS_0022"
/transl_table=11
/phrog="21"
/top_hit="No_MMseqs_PHROG_hit"
/locus_tag="RKPNQLQJ_CDS_0022"
/function="head and packaging"
/product="portal protein"
/source="PHANOTATE_1.5.1"
/score="-11359.46646775446"
/phase="0"
/translation="MGWWQRLRFGLFGGPTPTYDGIGGGRRALAWQVANPGAVAALAAS
QEELRAKSRDQVRRNVWAAAGVEAYVANAIGTGIKPQSMAADVAVRPAIQTLWTHWGDD
ADAAGLTDVYGLQALACRALVEGGEALVRLRYRRPEDGLSVGLQLQVLEPEHLPAALNR
ELSSGNVIRAGIEFDKLGRRVAYHLYRSHPEDGALAPMSGAGGLDTVRVDAREVIHLFR
PLRPGQIRGEPWLARALVKLHELDQYDDAELVRKKTAAMFAGFITRTAPEDPLMGEGPA
DAQGVALAGLEPGTLQLLEPGEDVRFSQPADVGASYAEFLRMQFRAVAAAMGITYEMLT
GDLTQVNYSSIRAGLLEFRRRCEAIQHGVIVFQLCRPVWRAWMTQAVLEGALDLPGFSR
RERDYLAVKWIPQGWQWVDPKKEFDALQTAIRAGLLSRSEAISAFGYDAEDIDREIAAD
NARADALGLVFDSDPRHDRAAAPPTSTRANAP"
CDS complement(15603..15806)
/ID="RKPNQLQJ_CDS_0023"
/transl_table=11
/phrog="2545"
/top_hit="No_MMseqs_PHROG_hit"
/locus_tag="RKPNQLQJ_CDS_0023"
/function="connector"
/product="head-tail adaptor Ad1"
/source="PHANOTATE_1.5.1"
/score="-3.929076405361307"
/phase="0"
/translation="MSFTPEHLQALEAAMARGERRVTFQDRSVEYRSVEELQTAIREVK
RGLAAQGQPVARQIRITTNKAT"
CDS complement(15857..17824)
/ID="RKPNQLQJ_CDS_0024"
/transl_table=11
/phrog="15"
/top_hit="No_MMseqs_PHROG_hit"

/locus_tag="RKPNQLQJ_CDS_0024"
/function="head and packaging"
/product="terminase large subunit"
/source="PHANOTATE_1.5.1"
/score="-184733.64800748735"
/phase="0"
/translation="MLREDEGLEAIAHAWRDGLTPDPLLTVSEWADRHRVLSSKASAEP
GRWRTARTPYLREIMDCLSPASPVERVVFMKGAQLGATEMGSNWIGFCVHHAPGPMMAV
WPTVEMAKRNSRQRIDPLIEESPALAGLIAPARSRDSGNTILAKEFRGGVLVMTGANSA
VGLRSMPVRYLFLDEVDGYPPDVEGEGDAIALAEARTRTFARRKIFIVSTPTIAGASAI
EREYEASDQRRYFVPCPHCGHFQWLRFEQLRWAREQPETVAYVCEGCERPIPEHHKGDM
LAQGEWRAMAPEHGAKTAGFHLSSLYSPLGWRSWRDIAAAWDSAVHPVSGGPSAIKTFK
NTELGETWVEDGETPDWQRLLERREDYPIGKVPQGGLLLVGGADVQKDRIEVSVWAFGR
GKSAWLVEHRVLMGDTARDAVWHALATLVGDTWTHASGARMPLARLALDTGYATQEAYA
FVRRLKDPRIMAVKGVPRGAALIGTPTAVDVTQAGKKLRRGIKVFSVAVGIAKLEFYNA
LRKTAEVGEDGTTLVYPPGYVHLPQMDAEYLQQLCAEQLVTRRDRHGFARREWQKLRER
NEALDCYVYARAAAAAAGLDRFEERHWRALEDQLGVGPPPDPEAPLQPIHPETPTDPGG
RSVSGPGRSSRRVIRSRWLS"
CDS complement(17825..18352)
/ID="RKPNQLQJ_CDS_0025"
/transl_table=11
/phrog="57"
/top_hit="No_MMseqs_PHROG_hit"
/locus_tag="RKPNQLQJ_CDS_0025"
/function="head and packaging"
/product="terminase small subunit"
/source="PHANOTATE_1.5.1"
/score="-20.22541187737344"
/phase="0"
/translation="MGLSIRAYARHRGVTDTAVHKAIRTGRITPEADGSIDPDKADREW
ARNSDTPKAGTRQPAVRVAMPDASHGGGPTLPGGGASLLQARTVNEVVKAQTNKVRLAK
LKGDLVDRAAAIAQVFTLARAERDAWLNWPARISAPLAAKLGVDPHTLHIALETAVREH
LQELGELRPRVD"
CDS complement(18459..18797)
/ID="RKPNQLQJ_CDS_0026"
/transl_table=11
/phrog="No_PHROGs_HMM"
/top_hit="No_MMseqs_PHROG_hit"
/locus_tag="RKPNQLQJ_CDS_0026"
/function="unknown function"
/product="hypothetical protein"
/source="PHANOTATE_1.5.1"
/score="-10.405760962733028"
/phase="0"
/translation="MRPAVCSIAWLRIRLWVSVRGSVFMWVLLGVGLLSGVIDASQPLE
IPPEPPSAKPICSSIGLDPASGLDPCLVRRLQRGFEAPDRRVVEIQVVRVLGFQGVDAE
LLLGDLSQ"
CDS 18724..19224
/ID="RKPNQLQJ_CDS_0027"
/transl_table=11
/phrog="10315"
/top_hit="No_MMseqs_PHROG_hit"
/locus_tag="RKPNQLQJ_CDS_0027"

/function="unknown function"
/product="hypothetical protein"
/source="PHANOTATE_1.5.1"
/score="-112.13238956913436"
/phase="0"
/translation="MNTLPLTDTQSRILNHAIEHTAGRIDGFPDNIKGGARRKVLDGLL
KRGLIALNGGDHCVTAEGYAAVGQTPPTPVSPSEETEMAASNAETKATGKRSTGSREDS
KQAEVIRMLRRPEGATIPQICAETGWQAHTVRGLFAGAFKKKLGLSLVSEKPEGGDRIY
KIG"
CDS 19235..19345
/ID="RKPNQLQJ_CDS_0028"
/transl_table=11
/phrog="No_PHROGs_HMM"
/top_hit="No_MMseqs_PHROG_hit"
/locus_tag="RKPNQLQJ_CDS_0028"
/function="unknown function"
/product="hypothetical protein"
/source="PHANOTATE_1.5.1"
/score="-0.1777567353840541"
/phase="0"
/translation="VPESRRPLSFNWKMLGFAVAQRVTTGVPTILLKERV"
CDS 19342..19710
/ID="RKPNQLQJ_CDS_0029"
/transl_table=11
/phrog="8020"
/top_hit="No_MMseqs_PHROG_hit"
/locus_tag="RKPNQLQJ_CDS_0029"
/function="unknown function"
/product="hypothetical protein"
/source="PHANOTATE_1.5.1"
/score="-26.05582764512276"
/phase="0"
/translation="MKTNKPIPASRNEAWGFWGTMDQHAEVAWALAMTAISDATCQPLE
PVRAFLDTRHGRHFADDVLNEMLRGHAIQQAVDAAVARWMGWTIGRRTSHEYGIPRGMP
YLTGFVIHCEIVEETLAA"
CDS complement(19674..20948)
/ID="RKPNQLQJ_CDS_0030"
/transl_table=11
/phrog="498"
/top_hit="No_MMseqs_PHROG_hit"
/locus_tag="RKPNQLQJ_CDS_0030"
/function="other"
/product="DNA methyltransferase"
/source="PHANOTATE_1.5.1"
/score="-33267.78282758025"
/phase="0"
/translation="MNWLADKIEQWPTTKLLPYARNARTHSDDQVAQIAASIAEFGFTA
PILAGSDGVMVAGHGRWAAARQLGLSQVPVIVLDHLSPTQRRALVIADNRIAENAGWDE
ALLKLELAALQDEDFDLASIGFDADALLDLLADAESVTEGQTEDDVAPEVPGVPISRPG
DVWILGSHRLLCGDAADPDSFARLMDGVKAAMVFTDPPYNVNYANTAKDKRRGKQRAIL
NDNLGTNFSDFLRAALKPMLAHCEGAVYIAMSSSELDTLQSAFRAAGGHWSTFIIWAKH
TFTLGRADYQRQYEPILYGWPEGASRHWCGDRDQGDVWHINKPARNDLHPTMKPVELVE
RALRNSSRPGALVLDPFGGSGTTLIAAEKSGRVARLIELDPKYVDVIVRRWQDWAGRQA

TRLADGVTFDAAVDQAASVSSTISQ"
CDS complement(20945..22333)
/ID="RKPNQLQJ_CDS_0031"
/transl_table=11
/phrog="498"
/top_hit="No_MMseqs_PHROG_hit"
/locus_tag="RKPNQLQJ_CDS_0031"
/function="other"
/product="DNA methyltransferase"
/source="PHANOTATE_1.5.1"
/score="-61649.43592552871"
/phase="0"
/translation="MLQVEYRPLEPLIPYARNPRTHSAAQVAKIAASIVEFGWTNPILV
DGAQGIIAGHGRLAAARSLGLAEVPVIELAHLSPAQKRAYVLADNRLALDAGWDEELLG
LELAELSGAGFDLALTGFNDDELEALLSIDTEDSDDAEDGEPETADDVPEPPATPVSRP
GDVWQLGRHRLICGDASDPDVVAALMAGELARLCFTSPPYGNQRDYTSGGIGDWDGLMR
TIFAQIPLTDNAQVLVNLGLIHRDNEVMPYWDAWLGWMRSQGWRRFGWYVWDQGPGMPG
DWAGRLAPSFEFVFHFNRQARKPHKIVPCKFAGQETHLRQDGSSTAMRGKDGEVGGWTH
KGQPTQDTRIPDSVIRVMRHKGKIGQDIDHPAVFPVALPKFVIEAYTDAGDLVFEPFGG
SGTTMLAAQRSGRICRSVEIAPEYVDVAIKRFQQNHAGVPVTLIATGQSFDEVAAERDV
EVAA"
CDS complement(22511..22753)
/ID="RKPNQLQJ_CDS_0032"
/transl_table=11
/phrog="No_PHROGs_HMM"
/top_hit="No_MMseqs_PHROG_hit"
/locus_tag="RKPNQLQJ_CDS_0032"
/function="unknown function"
/product="hypothetical protein"
/source="PHANOTATE_1.5.1"
/score="-0.1860092811112893"
/phase="0"
/translation="VTEGARCPSAANDCGSFPAPEGYGGNARGISLASDRKPRFAGFAV
CTPCRSARPWRPRRYRRSHESAQRPRCKPQCKPAL"
CDS complement(22750..22860)
/ID="RKPNQLQJ_CDS_0033"
/transl_table=11
/phrog="No_PHROGs_HMM"
/top_hit="No_MMseqs_PHROG_hit"
/locus_tag="RKPNQLQJ_CDS_0033"
/function="unknown function"
/product="hypothetical protein"
/source="PHANOTATE_1.5.1"
/score="-0.0838318659192101"
/phase="0"
/translation="LRGTGERCGRLRKRQAFGLSHLGACRRRVTLTVQQV"
CDS complement(22857..22946)
/ID="RKPNQLQJ_CDS_0034"
/transl_table=11
/phrog="No_PHROGs_HMM"
/top_hit="No_MMseqs_PHROG_hit"
/locus_tag="RKPNQLQJ_CDS_0034"
/function="unknown function"

/product="hypothetical protein"
/source="PHANOTATE_1.5.1"
/score="-0.0574278397299375"
/phase="0"
/translation="LDEAVCKTAHVLRRSPPLHGGALTEVDSI"
CDS complement(23032..23382)
/ID="RKPNQLQJ_CDS_0035"
/transl_table=11
/phrog="11214"
/top_hit="No_MMseqs_PHROG_hit"
/locus_tag="RKPNQLQJ_CDS_0035"
/function="unknown function"
/product="hypothetical protein"
/source="PHANOTATE_1.5.1"
/score="-136.7596028415392"
/phase="0"
/translation="MKTIVIGIMPQEDIRKRLLAIARGELKPKAGDPKIWFTSMRSLAE
VLSDENRVLLKVIRETKPESITSLAAATGRKPGNLSRTLKTMSHYGLVEMKREKNHVRP
IAKGTEFRIVAA"
CDS complement(23379..23750)
/ID="RKPNQLQJ_CDS_0036"
/transl_table=11
/phrog="No_PHROGs_HMM"
/top_hit="No_MMseqs_PHROG_hit"
/locus_tag="RKPNQLQJ_CDS_0036"
/function="unknown function"
/product="hypothetical protein"
/source="PHANOTATE_1.5.1"
/score="-84.81433834839902"
/phase="0"
/translation="MCMEMDPGIETLLDLDDQIIDQGSGYWVKIEAYQVEPTPDVPHGI
RYSLTLHEPYGKRILGYDNAHAVKPPKKFKYAGRRLTFDHMHQHARDPGVPYEFKDAHQ
LLADFFSEVDQVLLEVKKR"
CDS complement(23792..23959)
/ID="RKPNQLQJ_CDS_0037"
/transl_table=11
/phrog="6836"
/top_hit="No_MMseqs_PHROG_hit"
/locus_tag="RKPNQLQJ_CDS_0037"
/function="unknown function"
/product="hypothetical protein"
/source="PHANOTATE_1.5.1"
/score="-5.3852291346154955"
/phase="0"
/translation="MRWVLWLEEEQRHLIWMRAERQGWKEISRRFACCNRTAQRRWQQA
LRQVADRLNG"
CDS complement(23917..24072)
/ID="RKPNQLQJ_CDS_0038"
/transl_table=11
/phrog="No_PHROGs_HMM"
/top_hit="No_MMseqs_PHROG_hit"
/locus_tag="RKPNQLQJ_CDS_0038"
/function="unknown function"

/product="hypothetical protein"
/source="PHANOTATE_1.5.1"
/score="-1.1222272257694133"
/phase="0"
/translation="MSTAGRPSPARPGRAMPTKLANAVSSRAPRRSTACWRRCAGCCGW
RKSSGI"
CDS complement(24156..24365)
/ID="RKPNQLQJ_CDS_0039"
/transl_table=11
/phrog="19772"
/top_hit="No_MMseqs_PHROG_hit"
/locus_tag="RKPNQLQJ_CDS_0039"
/function="unknown function"
/product="hypothetical protein"
/source="PHANOTATE_1.5.1"
/score="-4.904751810374213"
/phase="0"
/translation="MNGPNPHPSCPLGKLQPQATDLEAVKREGWREQHILVVHLTDERL
DFVERELIRQIGERLYGRRESRRG"
CDS complement(24362..25141)
/ID="RKPNQLQJ_CDS_0040"
/transl_table=11
/phrog="159"
/top_hit="No_MMseqs_PHROG_hit"
/locus_tag="RKPNQLQJ_CDS_0040"
/function="DNA"
/function=" RNA and nucleotide metabolism"
/product="RuvC-like Holliday junction resolvase"
/source="PHANOTATE_1.5.1"
/score="-348.1527894041882"
/phase="0"
/translation="MTTTLIPDGRRSAICPLKSAIWIQMANVRHLGIHRLQSLTVQMAE
FPDGSAANSPSGATDCSYETIYGIPDGGDSLPLRGRGALRALSPSRRGLSSLGGHDEGD
GTTLLALDLGTHTGWALHQRDGTIISGSETFKPHRFEGGGMRFLRFKRWLTEIKQAAGD
LDAVYFEEVRRHAGVDAAHAYGGFLAHLTAWCEHHRMPYQGVPVGTIKKHAAGQGNANK
AAMVAAMRSMGFDPVDDNEADALALLHWALATQRVGI"
CDS complement(25138..27291)
/ID="RKPNQLQJ_CDS_0041"
/transl_table=11
/phrog="13004"
/top_hit="No_MMseqs_PHROG_hit"
/locus_tag="RKPNQLQJ_CDS_0041"
/function="unknown function"
/product="hypothetical protein"
/source="PHANOTATE_1.5.1"
/score="-471050.69993766496"
/phase="0"
/translation="LLDFNDALPRPANPVDLAAQRETVRADLLARLPSVLKALFPAGKV
RGGAFAIGNVQGDPGDSLEVALSGEKAGLWYDHATGEGGDLFALIAAVHGLDTHGQFAE
VVALAGRLLGTARVEPTPLRGEAPVDRLGPATAKWDYVAADGSLIACVYRYDPPTGKEY
RPWDVRARLWRAPDPRPLYNLPAIAQAKEVVLVEGERCADALIHKGVAATTAMNGAKAP
IDKTDWSPLRGKAVLIWPDRDAPGWDYAENAARACVAAGAASVAILVPPMDKPDKWDAA
DAVSEGFDCVAFLIHGERQVIKAAPPVLPTHTLGALLDDDSPVPPDLIAPRVLTPGGLL

VFGGAPKVGKSDFLLAWLMHMAAGASFLGMTPPRPLRVFYLQAEVQYHYLRERVKTVSL
PPSRLGAARVNFVATPQLRLVLDEAGLAQVIPAIVQAFGSAPPDLIAIDPIRNVFDGGE
AGGENDNGAMLFFLSQRVEQLRMAVNPDAGLVLVHHTKKLGKKPFEEDPFQALAGAGSL
RGYYSTGMLLFRPDEAQTTRQLIFELRNGAALPVKSVDKFNGTWREVVNTGRRVLQDYA
ERLDAERRRKHDVILQMLFDEAAEGRCYSATQFAEAFEGRAGLGAERTIRERLSVLATQ
GYIKFFRNAEDYGLPPLHRSKFGYQCVEGMVLRREGPPDPDTGEIPMWPVSVYPTHYKC
PQTGALLPVEDSTIWLYHDEDNP"
CDS complement(27284..27643)
/ID="RKPNQLQJ_CDS_0042"
/transl_table=11
/phrog="No_PHROGs_HMM"
/top_hit="No_MMseqs_PHROG_hit"
/locus_tag="RKPNQLQJ_CDS_0042"
/function="unknown function"
/product="hypothetical protein"
/source="PHANOTATE_1.5.1"
/score="-20.84084169521233"
/phase="0"
/translation="MRTRQPYDGKRVQRQLPRPHVRLTVIDKLLRRHVTLPCPEAHLAV
AVITLAIGDCLDPGAVLRAEARHFLAGSALEFWCDAVGLEATFVRAIARKGGYLPSETA
HGAGVKRTPKERGLA"
CDS complement(27640..28404)
/ID="RKPNQLQJ_CDS_0043"
/transl_table=11
/phrog="1990"
/top_hit="No_MMseqs_PHROG_hit"
/locus_tag="RKPNQLQJ_CDS_0043"
/function="DNA"
/function=" RNA and nucleotide metabolism"
/product="Cas4-domain exonuclease"
/source="PHANOTATE_1.5.1"
/score="-123.1323261217031"
/phase="0"
/translation="MTCRSEGATMLDYNPGALFSERLTALIDAGLQRRQAGEPIRTYLG
ASRLGVACERALQFEYAQAPVDPGREFSGRMLRIFERGHRMEEAVVGWLRAAGFDLRTH
QADGEQFGFSALDGRLQGHVDGVIVGGPEGFDAPALWENKCLGAKSWRELVKHRLAKAK
PVYAAQVAVYQAYLALHERPALFTAVNADSMEISAERVPFDGGLAQRMSDRAVRVITAT
EAGELLPRGFADPAHVECRQCAWGLRCRGGGA"
CDS complement(28388..28552)
/ID="RKPNQLQJ_CDS_0044"
/transl_table=11
/phrog="21342"
/top_hit="No_MMseqs_PHROG_hit"
/locus_tag="RKPNQLQJ_CDS_0044"
/function="unknown function"
/product="hypothetical protein"
/source="PHANOTATE_1.5.1"
/score="-3.2361535702965862"
/phase="0"
/translation="MAAVLPRLGDYVASIGMDRPLSAYSREEILQLVDVVLTTYFDHLR
EHDPDDVPF"
CDS complement(28566..28739)
/ID="RKPNQLQJ_CDS_0045"

/transl_table=11
/phrog="No_PHROGs_HMM"
/top_hit="No_MMseqs_PHROG_hit"
/locus_tag="RKPNQLQJ_CDS_0045"
/function="unknown function"
/product="hypothetical protein"
/source="PHANOTATE_1.5.1"
/score="-3.740786999430088"
/phase="0"
/translation="MRDLRTRGTRLLLGIAEEWPAQAGRQAPVQTLLLEALPAHPFTTT
EAERRRRDRSHP"
CDS complement(28741..30417)
/ID="RKPNQLQJ_CDS_0046"
/transl_table=11
/phrog="16"
/top_hit="No_MMseqs_PHROG_hit"
/locus_tag="RKPNQLQJ_CDS_0046"
/function="DNA"
/function=" RNA and nucleotide metabolism"
/product="DNA helicase"
/source="PHANOTATE_1.5.1"
/score="-188679.57367568504"
/phase="0"
/translation="MMLRPRQALLVERSLAALHQHGNTLAIGPTGSGKTIMLSAVAGGV
LEEPDAKACVLAHRDELTAQNRAKFGQVNPGRTTSVFDAQEKSWAGRATFAMVQTLARD
KHLEQMPTLDLLVIDEAHHAASPSYRRVIDRVLSRNPRARIFGATATPARADGKGLREV
FSNVADQITLGELIASGHLVPPRTFVIDVGAQSALAQVRRTATDFDMTEVETILNRTPI
TDAVIHHWREKAGERKTIVFCSTVAHAQCVADAFVAAGIRAVLIHGELSDAERKTRLAE
YETGEAQLVVNVAVLTEGYDYTPTSCVVLLRPSSHKSTLTQMIGRGLRTVDPAEHPGVF
KTDCVVLDFGTATLMHGSLEQEANLDGHRHQGEAPTKACPYCAATVPLGCRECPLCGFE
WIREEAAQAEALDEFVMTEIDLLKRSHFRWCDLFGCDDALMATGFGAWGGIFFLNGRWH
AVGGGRDLLPRLLAVGDRTVCMAKADDWLNENESLDTAHKTRRWLNEPPTEKQLRYLPP
AQRADFGLTRYQASALLAFQFNKSSIQRLVLAANDEHRRAA"
CDS complement(30432..31058)
/ID="RKPNQLQJ_CDS_0047"
/transl_table=11
/phrog="2300"
/top_hit="No_MMseqs_PHROG_hit"
/locus_tag="RKPNQLQJ_CDS_0047"
/function="unknown function"
/product="hypothetical protein"
/source="PHANOTATE_1.5.1"
/score="-68.7278087200874"
/phase="0"
/translation="MSYFDFNSAEQASFELIPKDTLVRLRLTLKPGGFDDASQGWTGGW
ATRSPETGAVYLACEGVVLDGPYARRKLWWNIGLHSPKGPTWQAMGRSFIRSLLNSARR
IHPADTGPQAQNARRIAGFAELDGLEFAGRIDIEKDGRGNDRNTVRAVIEPDHKDYAQL
MGQHFAPPVPTSPSGAIPAAATPAYAPSAPTASSAIPGGKPAWAQ"
CDS complement(31061..31924)
/ID="RKPNQLQJ_CDS_0048"
/transl_table=11
/phrog="124"
/top_hit="No_MMseqs_PHROG_hit"

/locus_tag="RKPNQLQJ_CDS_0048"
/function="DNA"
/function=" RNA and nucleotide metabolism"
/product="Sak4-like ssDNA annealing protein"
/source="PHANOTATE_1.5.1"
/score="-841.0290600862095"
/phase="0"
/translation="MTLPIISADQRLAERRGVKGVLVGKAGLGKTSQLWTLEAGSTLFF
DLEAGDLAVEGWAGDTIRPRTWPECRDFAVFIGGPNPALRDDQPFSPAHFDAVCARFGD
PGVLERYQTVFVDSITVAGRLCLQWCKGQPQAYSEKTGKPDSRGAYGLMGQEMIGWLTH
LQHTRNKNVWFVGILDEKLDDFNRRVFALQIDGAKTGLELPGIVDEVVTLAELPADDGS
RYRAFVCQTLNPWGFPAKDRSGRLDPIEPPHLGQLMQKIAGPARPPLERLDFSRPVPVT
IPTQET"
CDS complement(31921..32400)
/ID="RKPNQLQJ_CDS_0049"
/transl_table=11
/phrog="2480"
/top_hit="No_MMseqs_PHROG_hit"
/locus_tag="RKPNQLQJ_CDS_0049"
/function="unknown function"
/product="hypothetical protein"
/source="PHANOTATE_1.5.1"
/score="-39.737789355863576"
/phase="0"
/translation="MTSMNPDTLLATPAGQLAELPVESLCRLKHDAAAQLAAAKTLNEH
LDRAVEIRYAERARQLRLAMGKDTGVVHFDDGPVRITADLPKKVEWDARQLAALVRRIA
DSGEDPAQYVEISYRVSETKFNAWPAALQQSFAPARTLKTGKPGFRLALLGEDAV"
CDS complement(32397..32609)
/ID="RKPNQLQJ_CDS_0050"
/transl_table=11
/phrog="66"
/top_hit="No_MMseqs_PHROG_hit"
/locus_tag="RKPNQLQJ_CDS_0050"
/function="integration and excision"
/product="excisionase and transcriptional regulator"
/source="PHANOTATE_1.5.1"
/score="-0.8723945378228268"
/phase="0"
/translation="VSPKHLNPPALAERWGVSLATLDRWRSEGIGPVYLKLQGRVLYRQ
EDIEAYEARHLRRSPGQCVQAGGAV"
CDS complement(32606..32785)
/ID="RKPNQLQJ_CDS_0051"
/transl_table=11
/phrog="No_PHROGs_HMM"
/top_hit="No_MMseqs_PHROG_hit"
/locus_tag="RKPNQLQJ_CDS_0051"
/function="unknown function"
/product="hypothetical protein"
/source="PHANOTATE_1.5.1"
/score="-0.0777312519854731"
/phase="0"
/translation="LTRHLLTPPAQPPPGGFLLSGVRYRLRPYIRPYMAVPDARTSRRP
NCPQVPQPLPRRQT"

CDS complement(32782..33783)
/ID="RKPNQLQJ_CDS_0052"
/transl_table=11
/phrog="No_PHROGs_HMM"
/top_hit="No_MMseqs_PHROG_hit"
/locus_tag="RKPNQLQJ_CDS_0052"
/function="unknown function"
/product="hypothetical protein"
/source="PHANOTATE_1.5.1"
/score="-3270.8698988283804"
/phase="0"
/translation="MTTSLSTPSLLELIELFERASHPILGSEGRPLHGVPAWSLRADAA
LSKESQAQWFQRIGFAGHYPAERGDELLPVELEEDDDPQWYRYRCPETFRMKSVPASQV
AIYEVISARFLSMIADLLDIPRALQGAIHRPAIEGALWYLGSARIGSSHTDVWFARGLT
RRLAEIFRYFHAMPLPDQGLVLTSGPPLGEFIPPPRNYRFASLRELLIADTRTPSIDRD
LLQRILAAPADAGLRPNLEVQFDEYTRTLRIRSNPKPWVIRGDRQAAAVRYMYTQALHD
RWELSAGEILRAAYPNRTSARSLRMQNLFSGNIEWEDYIARRRKGFYGFRVD"
CDS complement(33749..34981)
/ID="RKPNQLQJ_CDS_0053"
/transl_table=11
/phrog="37634"
/top_hit="No_MMseqs_PHROG_hit"
/locus_tag="RKPNQLQJ_CDS_0053"
/function="unknown function"
/product="hypothetical protein"
/source="PHANOTATE_1.5.1"
/score="-132640.11111939885"
/phase="0"
/translation="MNDAENLAKLLTHLHPGLFRAFIDDEFGIDLPPPEDQERKRKQRE
RITKALAMLAVPTRQKIEEVAEKIVLLTDGPGQDVVEGFREGIFDEADRKAFDALKDQY
ERALWLYRHAPTVFKEALDARQADVFRQSTACYSGFMAPANLAVATDAESRQAFLGAVA
EHLGCLPTEVAIQIFLRLRPDTESGEEVDLYQISIHCNRPPETIDCVQSSELIAQHIIR
SEPSHITYEPANGHLEVLSRHGSDREALALIAAQSLLQSPITGERIPIKQYDYQSLAAS
RTFDLSGEEIVASVKVVELGYTDSNHRSLLVRIWSKDIDDIYTAAQSLIGPAFDFRHYH
LNYAKLSLRLKKVGRERARTISIVLRDANKCNIKTKREKDRALCDRLLAQWKLVKELGG
DDHKPLHAVAA"
CDS 34918..35631
/ID="RKPNQLQJ_CDS_0054"
/transl_table=11
/phrog="No_PHROGs_HMM"
/top_hit="No_MMseqs_PHROG_hit"
/locus_tag="RKPNQLQJ_CDS_0054"
/function="unknown function"
/product="hypothetical protein"
/source="PHANOTATE_1.5.1"
/score="-110.23451744419727"
/phase="0"
/translation="MRGTGQGGDGLGAWPGFRRRSCVRTLQGDERCGLRSADRRSAPKL
TEMCQTLVDFGVWQVGSGSLQHAIEYRNQPTVIDDGVFGQRIDHPEHAMNLDAFFLGQR
PHALVSRQQRPAVGDRQCHREAVVDGQFGLGQAVGQRLRHSGRVQGFYAQAKPLELFAV
DCTEIQQFVFVQSVGDSKFEGQLEDVFQKGPVSQLNQNRRIAHQDPHARSLQRARHGSA
NLAGTLAQQLRRFRQ"
CDS complement(35554..36606)

/ID="RKPNQLQJ_CDS_0055"
/transl_table=11
/phrog="2439"
/top_hit="No_MMseqs_PHROG_hit"
/locus_tag="RKPNQLQJ_CDS_0055"
/function="transcription regulation"
/product="transcriptional regulator"
/source="PHANOTATE_1.5.1"
/score="-22449.829441518417"
/phase="0"
/translation="MFGERLKLARKRAGLSLRGLAEAVDHAVSAQAIGRYERGEMLPGS
SVALKLAQALGVTLSYLFSPSEIRLEGVEFRKTAATRARERAMVEAAVLDHVDRYLLIE
DLLEIGSHEWDKPVGAPIPVSTLEEAEDATDQIRTAWNLGGDAIPHMTELLEEHGIKVL
KLDFPLSVDGLTCLVARPGQEKVPVIVGAMGKSVERRRFTLAHELGHMVMAIVGDIDEE
KACQRFASALLMPKQDLLFEVGQRRHAFGYTEVIQIKRMYGVSAAALIVRLRDLGVIGD
GTVQTLFRGIGRTWRKREPEPLTEDETPKRFQRLVLRALAEDVISLPKAAELLGKSTGE
VSRTMSGPLE"
CDS complement(36622..36717)
/ID="RKPNQLQJ_CDS_0056"
/transl_table=11
/phrog="No_PHROGs_HMM"
/top_hit="No_MMseqs_PHROG_hit"
/locus_tag="RKPNQLQJ_CDS_0056"
/function="unknown function"
/product="hypothetical protein"
/source="PHANOTATE_1.5.1"
/score="-1.3619416236134836"
/phase="0"
/translation="MRCPEGRCQRRESDIRPALARFTYQTSISTL"
CDS 36798..36986
/ID="RKPNQLQJ_CDS_0057"
/transl_table=11
/phrog="No_PHROGs_HMM"
/top_hit="No_MMseqs_PHROG_hit"
/locus_tag="RKPNQLQJ_CDS_0057"
/function="unknown function"
/product="hypothetical protein"
/source="PHANOTATE_1.5.1"
/score="-3.0484476905936813"
/phase="0"
/translation="MLATYLPSERLSPAQRAHEITTILAGALLRTYPTEPGKKTGCDLG
LLPGKRVHTTSSQPESV"
CDS 36986..37438
/ID="RKPNQLQJ_CDS_0058"
/transl_table=11
/phrog="11806"
/top_hit="No_MMseqs_PHROG_hit"
/locus_tag="RKPNQLQJ_CDS_0058"
/function="unknown function"
/product="hypothetical protein"
/source="PHANOTATE_1.5.1"
/score="-30.25071682201382"
/phase="0"

/translation="MNDLKLSVAAQVASLPSLKIKDLWTLWDKYFPRRPAHPNRPYLES
RIAYKIQEAAYGGLSPETRRRLEQIGQRHSKIKARRPSSTIHLPPGTVLVREWGEQDHK
VIVTAEGRFDYAGQSFKSLTAVARHITGTAWSGPLFFGLRQAGEGT"
CDS 37435..38832
/ID="RKPNQLQJ_CDS_0059"
/transl_table=11
/phrog="95"
/top_hit="No_MMseqs_PHROG_hit"
/locus_tag="RKPNQLQJ_CDS_0059"
/function="integration and excision"
/product="integrase"
/source="PHANOTATE_1.5.1"
/score="-74323.80111116557"
/phase="0"
/translation="MRASNRMEVATSAPSKPRQRCAVYCRVSSDERLDQEFNSIDAQKE
AGHAYIASQRIEGWIPVADDYDDPGYSGGNTERPALRRLLADIKAGRIDIVVVYKIDRL
TRSLADFSRMVEVFERQGVSFVSVTQQFNTTTSMGRLMLNVLLSFAQFEREVTGERIRD
KIAAAKRKGLWMGGVPPLGYDVADRQLVVNPREAAVVRRIFEDMLSVGSTTRIAAALNA
EGITTKAWTTQDGKQRPGARIDKKHLHHLLRNRIYLGEISHKGSWHPGAHPAIIEATLW
DAVHAVLARDARSRATETRQRERTDALLRGLLYDAEGEKMYPTYVKKNGRQYRYYFSKA
EARFGAGQKTSVRLPAEEIEAATLAQIRTVLASPEAIAAIWLAVQSQTSDIDEAQVVVA
LGNLGSVWEQLFPAERHRIVQLMIERVELADGGLRIRWRPLGWKALLGEFTPKTLGAEL
VEAEAAA"
CDS 38829..39194
/ID="RKPNQLQJ_CDS_0060"
/transl_table=11
/phrog="14161"
/top_hit="No_MMseqs_PHROG_hit"
/locus_tag="RKPNQLQJ_CDS_0060"
/function="other"
/product="recombinase"
/source="PHANOTATE_1.5.1"
/score="-40.344423066467115"
/phase="0"
/translation="MKADALESFVQITFQRRGVQRLAATTAPAHDLPFLVGLGRALYWQ
HLLDNGVVRSGSDIARREGLHPSTVNELLRLTLLAPDLIERLMAGRQARALTLKWFQRH
PLPVDWAEQRNFMASFE"
CDS 39203..39673
/ID="RKPNQLQJ_CDS_0061"
/transl_table=11
/phrog="14161"
/top_hit="No_MMseqs_PHROG_hit"
/locus_tag="RKPNQLQJ_CDS_0061"
/function="other"
/product="recombinase"
/source="PHANOTATE_1.5.1"
/score="-77.97652098857725"
/phase="0"
/translation="MAKKDRGIIIGKPRTVSLPQPAGGVQLETFLPWTLVKRGVKREIL
TPLGTPAAFREEVQRERKQRQAEQDTPLIRALGLAHYWQRLLDEGKLESLSDIAAAEGM
NLSQVSRIARLGRLAPGIVEACLAEKDSGLTLEDLNRWANSFRWPSREWPSA"
CDS 39707..39865
/ID="RKPNQLQJ_CDS_0062"

/transl_table=11
/phrog="No_PHROGs_HMM"
/top_hit="No_MMseqs_PHROG_hit"
/locus_tag="RKPNQLQJ_CDS_0062"
/function="unknown function"
/product="hypothetical protein"
/source="PHANOTATE_1.5.1"
/score="-5.721702405397223"
/phase="0"
/translation="MGAYDCIIQKDFFRVGLLRQFGEKAMGGVRISVCEARVAFSTVTS
TEGPFRP"
CDS complement(39899..41152)
/ID="RKPNQLQJ_CDS_0063"
/transl_table=11
/phrog="2331"
/top_hit="No_MMseqs_PHROG_hit"
/locus_tag="RKPNQLQJ_CDS_0063"
/function="integration and excision"
/product="transposase"
/source="PHANOTATE_1.5.1"
/score="-38521.16293320821"
/phase="0"
/translation="PSKTSKKNPAAALPSLPKELIDHFISGPMSAEAVNAASMAFKKAL
IERALGAELSHHLGYPPGADKPGEVCNHRNGATKKTVLTEEGPLRIDIPRDRQGSFEPM
LIPKHERRFTGFDDKIVAMYARGMTVREIQGFLAEQYGTEVSPEFISSVTDVVMAEVTA
WQSRPLEPMYPVVFFDALRVKIREDAVVRNKAIYLALGVLPDGTRDILGLWIENTEGAK
FWMKVFNDLKTRGVADILIAVTDGLKGMPEALAAVFPATTLQTCIVHLIRNSLDYASWK
DRKALAAAIKPIYTAPSAEAALAELEAFARGPWGEKFPTVAAAWRRAWDRVIPFFAFPP
AIRRVIYTTNAIESINARLRKIIKTRGHFPSDDAATKLIWLALRNITADWGRAAKDWKE
AMNQFAILYAERFEAARG"
ORIGIN
1 cgtcacaccc cgagcggctt gtccggatcc ggctcggcga aatggaactc cacgacgtcg
61 ccgttgccgt ccttgaactt cagggtcttg atggccgcgt acttgaccac gaactgcccg
121 ccggcggtct tcagatcacg ggccgggtag tattcgcggt ccgcccagga ctcccctttc
181 gcgttctgcg ccgtcttctc ggtcagcggc gaggcgatgc cggcggtctt cctgcgggtg
241 ccgatgccga cggcgttgcc gatccctggc ttggccaggg gctcgggcag cgcgtcgctg
301 gtcgtccgca ccgcgtcgac gatggcgttg aggtcgttga cgaaaaactt ctggacagtc
361 atcgggaggt gccctgagcg gatggtggga gcggctccgc cgcgaaaacc tggatcgcgg
421 cgcagccgct cctacgccgt cgcggtttcc cgcacgttgg cgatcacgac cgccagctcc
481 gttgcggtcc cgaccgcatg ggtggcgtcg ttcacctcga tccagatcgg cttggcattg
541 gccgctcccg agaggatttc ggtgccgagg tccagcgcgg acccgccgac ggccgcggcc
601 aagccggcct gagtcgccgc cagtttcact tcggaggcgc tatggccgga gccggtattg
661 gcgtccacca ccgtggcctg gatcgggtcc acgccgggat tggcgttggc ctgaatcttc
721 cggttggccg tgggggaacc gaggtacagc gtgaacacca ccggcggggt ggagccgtcg
781 gcgttctggg tcgccaccag gttgcccgcc aggagctggg tcagggcggc atcggtatag
841 agtgcgaacg tgggcattac ttaagcctcc aggatgagtt cgtcgtcggg gatcgcgatg
901 gggagatggt cggccggcgc cgtggtggtg gcgtcgatgt gctgctgctc gacgcccggc
961 acggtgatcg tgaattcgcc gttggcgggg tcgtagccga tcgcgggcgt cgccagcgga
1021 tgcttgagcg gcgcctgggt ggccggcggc ttggccgggg ccgggaaatc gaagccggtg
1081 tccacggtac cgacgccgta ggacttcgac accgccaagg ccagggtggt gatggccgcg
1141 ccgtggtcga aatcgatcga atgggtcacc cggcgcacct tgcccttcgc ggtcagccgg
1201 ggcgatgcca tccgcaccgt gtgggtgcgg tcgatcaggg gcgcgatcag agtgtcgaag
1261 gtcacggtgg tctggcggtg ggaggacagg atgtccctcc gtgcccgctc gatcccggtc

1321 atgtaggcgt tggagaggcc cgcccggccg tcggccgcac cgtcgtcgag gtcgtagtac
1381 agatcgccca gggggttggc gatggggtcc agtgtcgcaa aacgctgcgc cccggccggg
1441 accggcacgg tgtaggtccg gacctggagc gggtggtacg gaatccagga atcgcaggca
1501 tagccgccga tgggcgcgcc gtcgtgcaac tcgagcttga ccgtctgctc cgtggtccgg
1561 tcccaatgtg cgtagcgcgg gtccttgtcg ctctgggcgt cgaagttcgc cgtggtagtg
1621 ccgtgccgct tgccgatcac ccgaatcgaa tccggtgcgt cgacggtgac gtgccattgc
1681 tcggtcacgg tctgggtcca gcgctgcgcc aggatcgcgg acaccccgcc cgccaggctg
1741 tagcgcgggc agtcggacag gtacatgacg ccgtgcagcc attgcgggtc cggcagcggc
1801 gtcatccgga gcaggggtgt cccgccctcc gccagcaccg cccagccggt gccgtcgatg
1861 gcctgcatga ccgtttccgg ggtcggcgcc gtgctgccct ggtcgatgag ctggtcgaga
1921 ctcagctccc agtggtagcg gtactcccgc tgcatcagcc gctcgtagcg gtagtccatg
1981 tgcacgtcga tcgcgttgac cagctggcgg gccgaggcca tgtcgatgcg gacgctgttc
2041 tcgatcaccg cgtcggggcg gaaggtgaaa tcggccgtag ccctggcctg ccagggcgtg
2101 atccggcctt gcaggttgag gtccaggtcg tagctgaccg gcagcgtcga cagccggtcg
2161 agcacgtact gccactcgtc ggcgtgcttg tcgaacacgt agggcgacca gcggccgccg
2221 atgatcccgg cgatctcctt gcgcttgagc ccgtcgaaac ggctctgcag attgtcggtg
2281 caggagagat ggagcaggcc ctgcaccggg tcccagccgg gcacgtcgag gatgccctgg
2341 aagatcggca gctccacgcc cgcggtgacc agaaaaaggg tgatcggcag cccggtcagc
2401 gtgtggatgt ggatcggccc gggcgccggc agcacggtga actcggcgat ccgggccgcg
2461 ccctcttcgc cgtccacggt cagcgtgccg gtcatccgcc ccagcagcgc ccccatgacc
2521 gcaccggctt ccggcggcac cggctcgccg tcgccggcgg cgttgccgat gacggccttg
2581 accgcccagg ccgtgatgtc cgcgctcgct gcctgggact ggccgacgaa cgccccgccg
2641 ccgacggcct gggcgtcgta ttgggtctgg ctgagacttg cagtcaggtt cgcgatgaac
2701 gcgtagacgg cctgctcgac cgtggcgtcc aggtcgaaca ccgggtcggc gaccgtctgt
2761 gccaggcctg cggacagcgt gaacaccgcc ttgacccggt gggtgacggg cgcctggagc
2821 ctgtaggcct cggcccggag gcgctgttcg atgacgacgg acgcatcgaa aaggcggaac
2881 tgcgactgct cgaccggcgc cgtgaggttg aaccgcgccc agaggcgctg ctcgaggggc
2941 gccgcgaccg cgtaaccatc gaagccgacc tgttcgacga acgcctcgaa acccgtcacc
3001 gcgtagatcg gctgacggat cggggcgatc gcgtcgaaga cctcgaagac caccacggcc
3061 tgggcgaccc ccgccgacag gtccaggcgc gcgtagacct ggctgtggag gcttgcggcg
3121 atggcgtaga cgtcgagggt cgtctgctcg atggccgcga cgaggctggc ctcgacgtag
3181 atcggctgcc cgatggacgc cgcgatcgaa tagacctcgg cggccggcgc cgccgtggct
3241 gccgcgctgg cgacaccgcc gatgccggta acgccgccga cggggagcgt cattacagct
3301 cctcggccag cacgctgacg tgggcgagct ggaaggcgcc ggacaccgcg gcgccggggt
3361 tggcccggct gagcatgaag cacaccggcg cgtcaggcgc tacgtccagg acgtagccgc
3421 catccgtgtt gaatccatag ttgccggggg cgacttgcgt ctgcagattg gaaggggtgg
3481 ctggcgccgt ggcgcccgtt tccaccgaga gcacgatgct gtcgccgctg aaggtcgaac
3541 tggtcgagag cacggcgacg agcttcgaca gcctgagggt cttgccggta cggttgaccc
3601 agaacgccgc cgccatgctg ccggtggtgc tgcccgaaaa cggcgccagg aaccgggtgc
3661 tggtcaaagc gttggtcccc gtcatcgatg ccgacaggac catccgccag cggtcgatct
3721 ccttcgcgcc cggcgaccat tggagcaggg cgcctgcggc gaacgcctgg gcgccgccgc
3781 tggaactggc gatgttgcgc tcgatcgtca actggtacgg tcccgcccca ttgcgggcgg
3841 tcaccttcac ccgctcccac ttcgtcggcg tctcggcgtc gatgagggtc aggtagtagt
3901 ggtattcacc caacgggtcg ggcggcgtga tccagggcgg ctgggccgcg gccgccatca
3961 accaggtatc ggccgccccc agggcggcgg tgagcacggt tttgacgcca tcgagccccg
4021 cgagcttcag catgtcagcc tccgatcgga tcgaccattt cggcgctcag gcgccagctg
4081 taattcccgg tcgtgccgtc cagctgctgc tccggaggat cgcagtagac ggtgatcttc
4141 gggtagtaat aggtgatgtc cacgccgtcg acgttcagcg tgacggtggc gaaatcgccc
4201 tccgtccgca ccggtaccgg aggcgtaccg ccgccgacga tggccagggg cgaagcgcag
4261 gccagaacct gcggctgcga gtagtcgagc gccgacagcc ccggcggcac ccagccgcct
4321 ccgctgatct cggtggcgag cttgcgccag gtgcgctgct tcaccgagcg gccggaccac
4381 atccggtggg tcgttgcgcc ttccagccag cggtaggtct gggaccattc cagccgggcg
4441 tagagcggca cggcgatacc gccgaggatg aaggccgaat cgcggagtgc gctcaccgtt

4501 catcgggctc cggttcatcg cagcgcagga ccagcccggc ctcgtcgatg tgcgcgagga
4561 gatcggccgc ccgcgccggg cagcgccagg gggccggcac cagcgtcacc cgcggctcca
4621 gggtgcaaca gccggacagc gtcaaagcgc acacagcgcc acccagagcc cggacaccac
4681 gccggcagac accagcacgg cggccgcgca ccaaacttct ccgcgggtca aaacgatcat
4741 cagtccctct ccttgaaaaa atgcagccgg ccgatgaccg cggttttcac catcgctctc
4801 gcccagtgcg gcggtttttt aagcaggctg tcgtcgaagt agtgcgtcgc ctcgccggtg
4861 atatcgggca gcgtgccttt cagcgcctgg gattccagct tcagcgcaat cgagaacgcg
4921 cggtcgcgcg cgtccacggc ttccgccttc gccttgttcg ggtcgccttc attccagcag
4981 ctgaactgcc gggggttcag gcacacctcg tccggcgtcc gcccccacca gccaggccgc
5041 tgcgcccgcc ggaagatcac gttcatcacg ccctgcatgc cggcctcgcc ttcgccgcgc
5101 gcttcggccc aggcggtgcg tgcgggaatg tggttcatcg cttgcctgcc tccttgttgc
5161 tggcgacatg cagcacgttc accagggtga cggccaggac gcccagtccc tgcgccaccg
5221 cggcgacgtc ggtgccggat agccgcaagt cgtagccgaa cgcttcggcc aatcccgtcg
5281 ccgccatcag caaggccgtc agcttggcga tgaggtccgc gcggcgggac catacgccgg
5341 cgtcctgcaa tgccttgccg gcctgcatgc actcgtacag atatttcact ttttcgaaca
5401 tatcgctgtc ctcctgtcga aattcacggc gtttttaggc acatcacggc gctttcacgc
5461 cgaacgagcg cacgaaccac aacgcgacct cgatgaggaa gtgcacgacg ttcatgcgga
5521 ctccgctgac gaaggtgccg atgtaggccg ccgcttcctt cttcttgatg tcgttcagcg
5581 tggtcttgtc gaccagccct gcgatcgtcc gggtttccag ttcggccacc ttgttctgga
5641 ccagacccca ttgctccttg gtgatgccgg agacggcctg caccacgaac cagagggccc
5701 agcgtaggat gggattcacg ggttgttcct cctgtgggtt gggaaactca tcgcacgatc
5761 cgcccgtgct tcagtgccgc tcgcgccacc tcgcgcttga gcgccgctac cacttgggga
5821 tcgccgttga gcgcgaagga accgggcacg ccgggcagat ggatgtgtac cggctggcca
5881 ccgtcggacg cgcgcgccag tgaggcatcg aacgccggca ccgcctgcgc cacgtagccg
5941 ccgatcgaaa agcgcgggag gctcaggttg ttgagggcat acagcttgtc gaggccgagc
6001 ttgcgcaccg cctccttccg cagcacgaat tcgccctctt ccaacagcgc cggaatacgg
6061 tcgccgccgc cgtagccctt gatccagccg ccgcggcgga agccgggtaa cgtggcatcg
6121 gccccgttgg ctccagtgtc cgaaccgtct ccctgctgct gcatgacgac agagatcgtc
6181 ttggtttcgg gtttcagcag ttccgccagc cggctttgca gggtctggag cgacgccggg
6241 tcgacgtcga cccgcagcgc gaaccccttc gtcagctgct ggtcgatgtc gctgatttcg
6301 gcctggacgc tctgcaggtt ttgaagggtt tcctggatct tctggctgac cgcggcggcc
6361 gcctgggtgt cggcctgttc ctgccgggcg gcggcttccg ccagcgcctg gccggcctga
6421 cggcgcgcgt cgatcagccg ctgcaggtcc tcggtgccga actgcggctg gccgggatcg
6481 gccggcgtct gttcaccctt gagcgcgttc aggcgctgtt cccgcaagga ggcggcagcc
6541 tgctgcccga gggattcggc caggccggcg gcctgctccc cggcctgccg cgagagttcc
6601 agattgccgt tcgcgagctc ctgccgctgc tgagcgagca gctgctggag ctgcgcgtac
6661 tgggcccgtg aggcttccaa cggcgtcagg gccgcctgct cgatgccggc gacagcccgt
6721 gccgtgctct gttcgatgtt gcggcgttcc tgggccgctg cgcgggccga ttccagcagc
6781 cgctgttcct gcccgatcag cttgtcgatg gtggcggtgt aggccgattc caggtccgcg
6841 tagatggccc gccgggcttc cagcgattcg cgctccagga cggtcttgcg ggcttcgccg
6901 tccttgaact tggcgatttc ccgggcatag acctgatcga ccagcccgag acgctgggcg
6961 gcaaagtccc gcacggcctt caggcgggca ccctcggctt gcagggtcaa ggcatggagc
7021 tgacgagcct gctccagctc cgagcgggct tgggtggctt gcacgccgtt ctgggcatac
7081 acgatgggcg gtgcggcatc ggcttcgatc ggctgcgcct ggatgcgcgt ctcggccagc
7141 ttctcgaggg cgtcgatggc cgccgtccgg cgctggtagg cgtcttcgat gcccgcggtc
7201 gcgtccttca gggcgttccg ggtgtcctcg aagctcttct gaacctcctt ctccttcgcc
7261 ttgacggcat tgccgctgtc ggccgcggcc tgcctgcgct tgcccagctc ggcccgcatc
7321 tcctgcagca gcgcggtttc accggccagc tcccgcccga tcttgccgcc catgaacgcc
7381 tggtccgccg cctggccgac ttccttgtag cgggcggtca gccgtgcgac cacggcctcc
7441 tgtttcttca ccgcggcttc gtcctggagc aggctcagct cggcttcgga tttgccgcgg
7501 gtgtcctggc cggttgcctt ggcgagttcg cggcgcttcc ggatgacgcg ctccagttcc
7561 gattccgcgg tgctgccgaa cagatgccag gcggacaccg ccgtcagcag cgtcaccgcg
7621 atgccgacgg gaccgccgag gaaggcggcc agcccgccca gagctatctt gagcggcccg

7681 gcgctggccg ccacttccgc ctgggcgaca gccaagcgtt gcgatgcagg taccagttgg
7741 gtctccacca gcgccaaccg cgccatgccg gaggcattcg ccacggcggc ctcggcggcg
7801 gcgaaggtgg cgcgggcgtt gtatgcttcg ttgagggcgt ggtcccgcgc cgcggccgta
7861 gcggccagat gagcttgcac cagccgggct tggctcccgg cgaaggctgc gacggcggcc
7921 gtgcctttgc ccgccagcac cgcgaccagc accgatcccg cgatctcgac ccccgtggcg
7981 atcgtgtcga tgttgccggc cgcgccgttg atggcgtcgg cgatgacttc cgcggcgcgg
8041 gtgatctcgg atgaggtgcc gacgaacttc agggcggcat tgtcccagcg caccaccgcc
8101 tgttcgatgg tatcgggcag cccggccgcg tcccggtcga tgctggcctt ggccttctgc
8161 agcgcctcca cgaccttctg cgtcgtcagt tcgccggctt cggccagggt gcgcagcttg
8221 ccgatcggca cgcccagccc gtcggcgatg gcctgggcca gccgcggcga ttgctccatg
8281 atcgagttga actcgtcgcc ccgcagcacg cccgcggcca gcgcttgcga gaactgcagg
8341 gtggcgctcg aggcttcctc cgccgaggcg ccggagatgc ggaaggattt ggcgaccgag
8401 tcgatgacgc tcagcacttg tcgctgagac acgcccatct ccccggcgcc cttggcgatg
8461 cgggcataca actgggagac gccggtcatc gcctgcgcgt tgttctgggc gatggcgaac
8521 agcgcccgct gcgcctcggt gaactggttc tgggaaccgg ccgcgagctt gacccggcct
8581 tcgaggttgc ggtactggtc cgagagtttg cccagatccc gggcagcttg cagggcttcg
8641 gcggccccga acgcgccgac ggtatactga ccgatgcgcc ggagcgatcc cgtcaaccgg
8701 tcgacctgcg ccgcgacgtt gccgagcccc ttctcggccg cggccgtgtt cgccgcgatc
8761 tcgatgaccg tcttgaaagt cgtcatgggc ctagcgccgc cgcatcaaac gcgcctgcag
8821 gaacagccgc caggggtaat gccagacgcc ggcgtggccg cgctcgatca gcgccagcgc
8881 cgtcatctcc aggttctcta ggttgccggc ggcgccgtag cggccacctg ggccgcgttg
8941 gccaggcgtt cgcggagccc gaaaaaatgc gggttcaggc tcttggccgc ctccctcacc
9001 ttgtccagct cggaggggag catggcgtcg agctcggccc ggctcaggtc cgagaaccgg
9061 gtcaggtcgt ccagggtgat gtccgcgaac agcgccaacg ccaccaggtc gttgcgatcc
9121 agttcggcgt tggcgtcctt cagccaggcg cggacctcgc ccacggtcag ctcgcgcacc
9181 acgatcttgc ggccgccggc ctcgatggtg cgctcggcgc ggaggccctt ggggttcgtg
9241 gtgtcgagct ccgccatggg gaattacacc gccggggtga tggtggcttt gaagtactgg
9301 ctgacgcccg caccggtctt gctggtgtcc ttcagcacct tgccggtcag ctccagcacc
9361 gcgaagtcct ccgtaccggg gccgggcagg ctgctggccg gcgagaactt caccttgtag
9421 aagtcgtaag tcacggcatc gccgttgacc tcgttgacgc cgtcgaaagt gatgtgccag
9481 tcctcggcgg tctgggtcag cgcctcgatg ctgttaccgc tcaccgtgcc gcgggtggcc
9541 agcgccaggt tctccttgct gatcgaccac agcttcagtt tcagcgtgac cgacttgatg
9601 cggctcaagg acgcgatcga gccgccgccc gggttctggt aatcggccag ttccttggtg
9661 tcctcctcga tgctgaggtc cagggcggca ttgcccaggt ccaccggggt accggcgccg
9721 acggcgccct tgatggtgcc ggtcaaaagc atggcggtcg tagtcataaa aaactccttg
9781 agtaacggat ggatgaaaag aaggaaagat cgacgccgac cgggcgtcac ggggtcccgg
9841 tgtccagcgg cagcggacgc acttcgaaca ccagggaaaa ctcgacgtgg ccgagatcga
9901 accagggatc gtccaggccg acgtgcttca gccggcccca gcccggttgc ggggaccagt
9961 gctccaaggc gcgcagaatc cgcagcacgt atgcgccgcc cgtcaccacc gtgctcgccg
10021 gcggggtatg ggccacgcac acggtgacct gccagcgctg gcgctcggcc aggaaaacgt
10081 tgggcggcgg cgtccccacc ggcccggcga agcccagcgg cagcaccagg gcggcggggc
10141 agagcggctc caggttctgc accccggcca ggacaccgac gccgtccacc gtgacgaact
10201 ccggcagggt ctcgcgcagg cgccggatca gcgccggctc gatctcgaac atgctggtga
10261 tcatcgcggc agcgcgtcct gcgtcatttc acggatcagt gcggccagcc gcagcgtggc
10321 ccgggtcgcc agtacctcgg ccagggccgg cactttcggc agggccacca cgtcctcgag
10381 gacgggcagg gagcggtgcc cgacccggtg aaagatcccg cggtggccgc tttgcatccg
10441 ggcgacgaag ctgccgggaa aaaggtaagc cccggcgctg ctgccccagt cctcctggcg
10501 caggcggccg acataggcgg cctggatcgg ggcatggccg gtccataccc ggccacgcag
10561 tcctgccagc cgcagatagg cttgcacccg gcgcgagcgc agcgccctca gcggaatgcc
10621 gtaggtcgcg ccgatggcgc ggaccatcgc ctcgcgggtc tcgcgcgcgg tctggcccac
10681 ggcccggcgg gaggctgtgc gtagcaccgc cggcaagcgg cgcaaatcct gagcggtatc
10741 gccgtgcagc cgcacgcgca agccgatact catcgcttcg ggctcagccc cggctcgaat
10801 ggatcagcaa accgaaccag cgcgccccga aatgccccgc gacgatcgcc gcccacaggc

10861 tctcgtagta gcccagaccc tcccgctcgg ccagcagcca caccgagaag ccgaccaggc
10921 tgcaggaaac gacgtcgacc agcaggcacc acaggcacag ccgccacggc cggggatggc
10981 gccgccagcg attgagctgg gccaccagtc ccgaccagat cgtcaccagg atgatctgca
11041 gcgccagccg gaggtcatcg ccgttgccgt gcggcgaatc cagcaggagc tgcttaggtc
11101 gggcgttcat cggggccgca ggatcaaggt ggtcaggccg tccaggcgat cgatccggga
11161 ggcgatgctg taggggacac cacggacctt caggggatcg cccatgtgaa tcgcgggcag
11221 gtccgcactc cgcaccgtca accgcggatc ggcctcgggc cggggcgcgc ccatcggtcc
11281 cgggcgttcc agcccggccg gcaccgagac gatccccgtc agatgcacag gcggcggctc
11341 ggtccggtcc ggccacagga tcaccggctc gccgaaggtc gacaggcaca ccgcattgac
11401 cgcttcgagg tccatgccta gaccgtcaac ttgatcagca gccccggccg gtggcacatc
11461 ggcagcgggt tggactgggt gtggagatcg gtgccgcggt cgaactggcg cggggcctgc
11521 ttggcataga gtggctggcc caaggtattc accgtctcgt tgaaatcggc cggcgcgacg
11581 taggtggcga aggtatcgac ggtgcccagc gggaaggcat gagcctcgcc ggcggcgatg
11641 aagcgccggg tcaccccgtg cacatcggtg gcgcggccac ggtactcctc gaaggtgatg
11701 ccgccgaagg tgaagccgcg gcgcacgtcg ttgatcagga tcgccccctg ctgccaatgg
11761 gtgaaggcgg tcttgacgtc cttgtgtccg gtcagcgcgg cgaagaattc cggcgagcac
11821 aggcaatgca cgtccgtcat gaactcgccc agcaggctgt cctcgatgtg ggccagcacc
11881 tccatgcatc gcgccttgac gtcggtggtg gcggtgccca gctggaagct caccgtcttc
11941 ggcgggatgt cgaactcgtc gtagaggtcg tacagcaccg aaccgtcggc atccaggatc
12001 accccccttga gcgcacccat gcgcagatgc tccagggtga tcgcatgctt gttgcgcatg
12061 gtctccagat ggcgcgccat caccccggcc accgcctcca gctcggtctc cgagccaaag
12121 gcccggatgc cctggacctc ctccggcagc accacgtcgt cgtgggggat gtgcggaatc
12181 acgaacgagc gcagggtgcg cttgccgcgg acgcccaccg tccccggtgc cccgggcggc
12241 agggtcggca acaggttcag caccccgttg cgctcctcga ccaggatctg gcgctgccgc
12301 accggtttca ccggcatcag gttcagatcc tccagccggc cgtagcggtt agggataatg
12361 ttgatggcgg cggtcagtgc cgccatcgaa aaggcagggt tgtgaaacgg gttctgcatg
12421 atcaggctcc ggtgcggacg aggatgccga gcgcttgcaa ctggacgatc gccgtggcct
12481 tctgggccgg ggtgataccg gccggccaca ccagacctaa gtcggacacg atggcatggc
12541 gggcgagcag cagggcttgc gggacttcgg ccgcggtggc gtcgaccgct tcgatcagca
12601 ccccgaccgc ctgggccgag gcatccgtcg cggcgggatc gagcgcctgc aggcgtaaac
12661 cggccggctc acgcgccacc accgccccca gcgccagctt ctggccgaga gccaccgggg
12721 caaggtcgcg ggaatagcgg ttgggcgcct cgtacttcaa gagatcgccg aggttcttgg
12781 gttcgcgtag cttgggcatg gctcagctcc gggcgatgag tttttggacg gcggcgacca
12841 ccgggctcgc ctcgggccgg accgaggtgc ccgcatcgac ctggatgcgc gaggcgatct
12901 cgggcgtctc ggctcgcgcg gccagcaaaa cccgccgcac ctgggcctcg gacagtccgg
12961 ccgccaggaa ctccgcggtg cgcggggccg cgccggcgat caggcagagc tcggcgatgg
13021 cctgggcttc gctccgtcct gcgtggtggg cttcggtcgc gagggactgg gcagtcggtt
13081 cgccgatgac aggggtggcc gcagccaagt cgttctgggg ggacggtgcg gcatgcggcg
13141 tcaagttgcc ggccgcatcg gcgtccttgc ggctttcgtc ggccgcttcg tcatcgagtg
13201 gcccaggcgc gtggtcgtgc ataacagact ccgaatgaag gagagaaacg ggtaggggtg
13261 acatcaccgc agccttccgg gcttgcggcg agatcagtcg ttgccgggtg cggattgcat
13321 cggcgaactt gttcagcacg gcatcgaaac cgagcacgcc gtcggcgagt cccgccgaca
13381 cggcgtcctc gccgaagaac agcccggcct cggtggcgcg taccgccgtt tccgggagtc
13441 cgcggaatcc agcgacctgc gccacgaaga tcccgtacag ccggtccact tcggcctgga
13501 gcgcggcagc cgcctgcggc gacagcggcg cgtgcgggtt gaggtcgttc ttgtgatggc
13561 cggcatagag cgcgctgtag gtcagtccct cgcgggcatc cttgaccgac tggtcgacgt
13621 gcagggcgat cactccgatc gagccggccc cgccggtctg cgccagggtc acgcgcgagg
13681 cgccggcggc gatcgcatag gcggccgaga acgccgtgtc gttggcgtgg gcccagatcg
13741 gtttgtgcga tgtccccgca cgaatcttgg cagccaactc gaacaccccg ccggattcgc
13801 cgccgggcga gtcgaggtcg agcaggatgc cgctcacttc gggatcccgc agcgcggcat
13861 cgagccgggc gccgatctcg gcataggaag tcaggccgga atgcgcctcc agctccagcg
13921 tccgtcgcac cagggtgccg tggatcggga tcacggcgat gcccggaagg gctggcgcgg
13981 ctgtccggga catcggcgcc ggcaccgcgg tagccgcttc cggcagcccg atgcgggatc

14041 ccaacaccgc caggatcacc tcgagcttcg gccgggcgat gaggagcggc gttccgtaca
14101 agcgggacgc cagatggatg agcggctggg tcatggtgcg ttggctcgag tcgatgtggg
14161 tggcgcggca gcgcggtcat gccgggggtc cgaatcgaac accagcccta aggcatcggc
14221 ccgagcgttg tcggcggcga tctcccggtc gatgtcctcg gcgtcgtaac cgaaggccga
14281 gatcgcctcc gatcgcgaca gcagtcccgc ccggatcgcc gtctgcaagg cgtcgaactc
14341 cttcttgggg tcgacccact gccagccctg cgggatccat ttcaccgcca ggtaatctct
14401 ttccctgcgg gagaagcccg gcaaatcgag cgccccctcc agcaccgcct gggtcatcca
14461 ggcacgccac accggccggc aaagctggaa cacgatgacg ccgtgctgga tcgcctcaca
14521 gcggcggcgg aactccaaaa gtccggcgcg gatcgaggaa tagttgacct gggtcaggtc
14581 gccggtcagc atctcgtagg tgatgcccat ggccgccgcc accgcccgga actgcatgcg
14641 caggaactcg gcgtagctcg cccccacatc cgccggctgg ctgaaccgca cgtcctcgcc
14701 cggctccagc agctgcaggg ttcccggttc cagccccgcc agggccaccc cctgggcgtc
14761 ggccgggcct tcgcccatga gcggatcctc cggcgccgtc cgggtgatga agccggcgaa
14821 catcgccgca gtcttcttcc gcaccaactc ggcgtcgtcg tactggtcca gctcatgcag
14881 cttcaccagc gcccgcgcca gccagggctc gccccggatc tggccgggcc gcagcgggcg
14941 gaacagatgg atcacctcgc gggcgtcgac ccgcaccgtg tccaagccgc ccgccccgga
15001 catcggtgcc agggcgccgt cctccggatg ggagcggtac aggtgataag ccactcgtcg
15061 gcccagcttg tcgaactcga tgccggcccg gatcacgttc cccgacgaca gctcccggtt
15121 gagggcggcg ggcagatgtt ccggctccag cacctgcagt tgcaggccga cgctcaagcc
15181 atcttccggc cgccggtagc gcagccgcac cagcgcctcg ccgccttcca ccagggcgcg
15241 gcaggccagc gcctgcagcc cgtagacatc ggtcaacccg gccgcatcgg cgtcatcgcc
15301 ccagtgggtc cagagcgtct ggatggcggg gcggaccgcc acgtcggccg ccatcgactg
15361 cggcttgatg ccggtaccga tggcattggc cacgtaggcc tccaccccgg cggcggccca
15421 gacgttgcgc cgcacctggt cgcggctctt ggcccgcagt tcctcctggc tggcggccag
15481 ggccgccacc gcacccggat tggcgacctg ccaggccagc gcccggcggc cgccgccgat
15541 gccgtcgtag gtcggcgtgg ggccgccgaa caagccaaac cgcaaccgct gccaccagcc
15601 catcaggtcg ctttgttcgt ggttatgcgg atttgtctcg ccaccggctg gccctgggcc
15661 gccaagccgc gtttcacctc gcggatcgcc gtctgcaact cctccaccga gcggtactcg
15721 acactgcggt cctggaacgt cacccggcgc tcgccccggg ccatggcggc ttccagggct
15781 tgcaggtgtt caggcgtaaa gctcatcggt aggccatgac ttattgttcc gctgtcatca
15841 aaaagtctgc ttgcgatcag ctcagccagc ggctacgaat aacccggcgc gaagatcgac
15901 cggggccgga aacagaacgc ccaccggggt cggtgggcgt ttcggggtga atcggttgta
15961 acggcgcttc ggggtccggc ggcggaccca cacccaactg gtcctccagg gcccgccaat
16021 ggcgctcctc gaatcggtcc agccccgcgg cggccgcggc ggcacgggcg tagacgtagc
16081 agtccagcgc ctcgttgcgc tccctgagtt tctgccattc ccgccgggcg aagccgtggc
16141 ggtcgcggcg ggtcaccagc tgctcggcgc agagctgctg caggtactcg gcgtccatct
16201 gcggcagatg gacgtagccc ggcggataga ccagggtggt gccgtcctcg cccacctcgg
16261 cggtcttgcg cagggcgttg tagaactcca gcttggcgat gccgactgcg actgaaaaca
16321 ccttgatgcc gcggcgcagt tttttgccgg cttgggtgac gtccaccgcg gtgggggtgc
16381 cgatcagcgc cgcaccgcgg ggcacgccct tgaccgccat gatccgcgga tccttgagtc
16441 ggcggacgaa ggcataggcc tcctgggtgg cgtagccggt atcaagggcg agccgtgcca
16501 gcggcatccg ggcgcccgag gcatgggtcc aggtgtcacc gaccagggtt gccaaggcgt
16561 gccagacggc atcacgggcg gtgtcgccca tcagcacccg gtgttccacc agccaggcgg
16621 acttaccgcg cccgaaggcc cagaccgaca cctcgatgcg atccttctgc acgtcggcgc
16681 cgcccaccag cagtaagccg ccctggggta ccttgccgat gggatagtcc tccctgcgtt
16741 ccagcaggcg ctgccagtcc ggcgtctcgc cgtcctccac ccaggtctcg cccagctcgg
16801 tgttcttgaa ggtcttgatc gcggatgggc cacccgagac cgggtgcacc gcgctgtccc
16861 aggctgcggc gatgtcgcgc caactgcgcc agcccaaggg gctgtaaagg gacgacaggt
16921 ggaagccggc ggtcttggcg ccatgctcgg gcgccatcgc ccgccattcg ccctgtgcca
16981 gcatgtcccc cttgtgatgc tcgggaatcg gccgttcgca gccttcacag acgtaagcca
17041 ccgtctctgg ctgttcccgc gcccagcgca gctgttcgaa gcgcagccac tgaaaatgcc
17101 cgcaatgggg gcagggcacg aagtagcggc gctggtccga ggcctcgtac tcgcgctcga
17161 tggcgctggc tccggcaatc gtcggggtcg agacgatgaa gatcttgcgg cgggcgaagg

17221 tgcgggtgcg ggcctcggcc agggcgatcg catcgccttc gccctcgacg tcgggcggat
17281 agccgtcgac ctcgtccagg aacaggtagc gcaccggcat cgagcgcagg cccaccgcgc
17341 tgttggcccc ggtcatcacc agcaccccgc cgcggaactc cttggccagg atcgtgttgc
17401 cggaatcgcg gctgcgggcc ggggcgatca gcccggccaa ggccggcgat tcctcgatca
17461 gcgggtcgat gcgctggcgg gagttgcgct tggccatctc caccgtcggc cacaccgcca
17521 tcatcgggcc cggcgcgtgg tggacacaaa agccgatcca gttcgagccc atctcggtcg
17581 cgccgagctg ggcccctttc atgaacacca cccgctccac cggcgaggcc ggcgacaggc
17641 agtccatgat ctcccgcagg tacggtgtcc gggcggtgcg ccagcggccc ggctcggccg
17701 aggccttgct ggacagcacc cggtggcggt cggcccattc ggacaccgtc aacagcggat
17761 ccggcgtcag cccgtcacgc caggcgtggg caatcgcctc cagaccctcg tcctctctca
17821 gcattcagtc gacccggggc cgcagctcgc ccaattcctg caggtgttcc cgcacggccg
17881 tctccagggc gatgtgcagg gtgtggggat cgacgccgag ctttgccgcc agcggcgccg
17941 agattcgcgc tggccagttc agccaagcgt cgcgctcggc ccgggccagg gtgaacacct
18001 gggcgatggc cgcagcgcgg tcgaccagat cgcccttgag tttggccagc cggaccttgt
18061 tggtctgggc cttgaccact tcgttgaccg tgcgggcctg cagcaacgag gctccgccgc
18121 cgggcaaggt cggcccgccg ccgtgggagg cgtcgggcat cgcaacccgt accgcgggtt
18181 ggcgcgttcc ggccttcggt gtgtccgaat tcctggccca ttcccgatcc gccttgtccg
18241 gatcgatgct gccatcggcc tccggcgtga tgcggccggt gcggatggcc ttgtgcacgg
18301 ccgtgtcggt gacgccgcgg tggcgggcgt aggcgcggat cgagagtccc atcggtgaaa
18361 tcgatcgcaa atcagcttgg cttccggggt ctacagcgcg ttcatggcgt cgtcatccac
18421 accaccacga ggactacgtc atgaccccgc ttgacgcctt actgactgag atcgcccaac
18481 agcaactcgg catcgacacc ctggaaaccc aaaactcgga cgacctggat ttccacgaca
18541 cggcggtctg gtgcctcaaa gccgcgctgg aggcggcgta ccaggcaggg atcgaggccg
18601 gacgccgggt cgaggccgat gcttgaacag atcggcttgg cgctcggtgg ttccggtgga
18661 atttccaacg gctggctggc gtcgatgaca ccgctcaaca acccaactcc caggagcacc
18721 cacatgaata cactccccct gaccgacacc cagagccgga tcctcaacca tgcgatcgag
18781 catacggccg ggcgcatcga cgggtttccg gacaacatca aaggcggcgc ccgccggaaa
18841 gtcctcgatg gcctgctcaa gcgaggattg atcgcattga acggtgggga tcattgcgtg
18901 accgccgaag gctatgccgc tgtggggcaa acgccgccga cgccggtatc tcccagcgaa
18961 gaaaccgaaa tggccgccag caatgccgag accaaagcca ccggtaagcg aagcaccgga
19021 agccgcgagg acagcaagca agccgaagtc atccggatgc tacgccgccc cgaaggcgcg
19081 acgatcccgc agatctgcgc cgagaccgga tggcaggccc acaccgtgcg cggcttattt
19141 gccggcgcct tcaagaaaaa actggggctt agcttggtct cggagaagcc tgaagggggt
19201 gatcgcatct acaagatcgg ctgatcgagc cggggtgccg gaaagtcggc gccccttatc
19261 gttcaattgg aaaatgcttg gcttcgcagt cgcacagcgc gttactacag gcgttccaac
19321 gatcctgctt aaggagcgag tatgaaaacc aacaaaccca tccccgccag ccgaaacgaa
19381 gcctggggct tttggggcac gatggaccaa cacgccgagg ttgcctgggc cctcgcgatg
19441 acggccatct ccgatgccac ctgccagccc ctcgaaccgg tcagggcttt cctcgacacc
19501 cgccatggac ggcacttcgc cgatgacgtc ctcaacgaaa tgctccgggg ccacgcgatc
19561 caacaggcgg tggacgcagc cgtcgcccgg tggatgggct ggacgatcgg ccgccggacc
19621 agccacgagt acggcatccc gcgcgggatg ccttatctca caggcttcgt gattcactgc
19681 gagatcgtcg aagaaacact ggctgcctga tcaaccgcgg catcgaaagt tacaccgtca
19741 gcaaggcggg tggcttgcct gcccgcccag tcctgccagc ggcgaacgat gacatcgacg
19801 tacttggggt ccagttcgat cagccgcgcc acccgtcccg acttctcggc ggcgatcagc
19861 gtcgtgcccg aaccgccgaa ggggtcgagc acaagggcgc cgggccggct ggaattgcgg
19921 agggcccgct ccaccagttc caccggtttc atggtcggat gcagatcgtt ccgggccggt
19981 ttgttgatgt gccacacatc cccctggtcg cggtcgccgc accagtgacg gctggcgccc
20041 tcgggccagc cgtacaaaat gggttcgtac tggcgctggt agtcggcacg cccgagggtg
20101 aaggtgtgtt tcgcccagat gatgaaggtc gaccagtggc cccccgcggc gcggaaagcg
20161 gactgcaggg tgtctaattc gctggaactc atggcgatgt agaccgcgcc ctcgcagtgg
20221 gcgagcatcg gcttcagcgc tgcccgcaag aagtcggaga aattcgtgcc caggttgtcg
20281 ttcaggatag cccgttgctt gccacgtcgc ttgtccttag ccgtgttggc gtaattcacg
20341 ttgtacggcg ggtcggtgaa gaccatcgca gccttcacgc cgtccatcag cctggcgaaa

20401 ctgtccggat cggcggcgtc cccgcacagc agccgatgag accccaggat ccagacatcg
20461 cccggccggg agatcggtac ccccggcact tcgggcgcca cgtcgtcctc ggtctgacct
20521 tcggtgacgg attccgcgtc ggccaagaga tccagcaggg cgtcggcatc gaaaccgatc
20581 gaggccaggt cgaagtcctc gtcctgcagg gcggccaact cgagcttgag cagggcctca
20641 tcccagccgg cgttctcggc gatgcggttg tcggcgatca ccagggcacg gcgctgggtg
20701 gggctcaggt gatccagcac gatcaccggt acctgggaca ggcccagctg gcgagccgct
20761 gcccatcgcc cgtgcccggc gaccatcacc ccgtcgctcc cggccaggat cggcgcggtg
20821 aagccgaact cggcgatcga tgcggcgatc tgcgccacct gatcatccga gtgggtccgc
20881 gcgttgcgtg catacggcag cagtttggtc gtgggccact gctcgatctt gtcggccagc
20941 cagttcatgc cgccacctcc acatcacgtt cggcagcgac ctcgtcgaag gactggccgg
21001 tggcgatcaa agtcaccggc acgccggcat ggttctgctg gaagcgcttg atcgccacat
21061 ccacatactc cggcgcaatc tccacgctgc gacagatgcg gcccgagcgc tgggcagcca
21121 gcatcgtcgt gccgctgccg ccgaacggct cgaacacgag gtcaccggca tcggtgtaag
21181 cctcgatgac gaacttcggc agcgccaccg ggaacaccgc gggatgatcg atgtcctgac
21241 cgattttgcc cttgtggcgc atcacgcgaa tcaccgagtc ggggatgcgg gtgtcctgcg
21301 tgggctggcc cttgtgcgtc cagccgccga cttcgccgtc cttgccgcgc atcgcggtgg
21361 agctgccgtc ctggcggagg tgcgtctcct ggcccgcgaa cttgcacggc acgatcttgt
21421 gaggtttcct cgcttgccgg ttgaagtgga acacgaactc gaagctcggt gccaagcgtc
21481 ccgcccagtc acccggcatc cccggcccct ggtcccagac gtaccagccg aagcggcgcc
21541 agccctggct ccgcatccac cccagccaag catcccagta cggcatgacc tcgttgtcgc
21601 gatggatcag tcccaggttg accaacacct gagcattgtc agtcagcggt atttgagcga
21661 atatggtgcg catcaggccg tcccagtcgc cgatgccccc gctggtgtaa tcgcgctgat
21721 tgccgtaagg cggcgaggta aagcagagac gggccagttc gccggccatc aacgctgcga
21781 cgacatccgg gtccgaggca tcgccgcaga tcaaccgatg ccggcccaac tgccagacat
21841 cgccaggccg cgacaccggg gttgccggcg gctcggggac gtcatcggcc gtctcgggtt
21901 cgccgtcttc ggcgtcatcc gaatcctcgg tatcgatcga cagcagagct tccagttcat
21961 cgtcgttaaa ccccgttagc gccaggtcga acccggcacc ggacaactcg gccagttcca
22021 gccccagcag ttcctcgtcc cagccggcat cgagcgccaa acggttgtcg gccagcacat
22081 aggcccgctt ctgggccggc gagaggtggg cgagttcgat caccggcact tccgccagcc
22141 ccaggctgcg ggcggcggcc aggcggccgt gcccggcgat gatcccctgc gccccgtcca
22201 ccaggatcgg gttggtccag ccgaattcca cgatgctggc cgcgatcttc gccacctgcg
22261 ccgcgctgtg ggtgcgcgga ttgcgggcgt aagggatcaa cggctccaac ggacggtatt
22321 cgacctgcaa catgaagggt tcgtagcaat cccaaaaaag tacggcccgg cgccgggtcc
22381 gatcggaacc tgcggcgaac cgtagacacg aggtcacgca gcaacaaaaa acccgccgac
22441 ggtgaaccgg ggcgggtctt ggggaaatca ggtggagcca agggtgcaaa cctggatggc
22501 agcacccggt ttacagggcg ggtttgcact gaggtttgca ccgggggcgt tgggcgctct
22561 cgtgtgagcg cctgtaccgg cgcggcctcc agggtcgtgc gctccggcag gggtgcaaa
22621 ccgcaaaccc tgcaaacctc ggtttgcggt ctgacgctag cgaaatcccg cgtgcgttcc
22681 ccccgtagcc ctcaggggcc gggaaggacc cgcaatcgtt cgcggcggag gggcatctcg
22741 ccccttcggt cacacctgct gtactgtgag cgttactcta cgcctgcagg cccccagatg
22801 cgacaggcca aaagcctgcc gttttcgcag ccgcccgcag cgttccccgg tgcccctcaa
22861 atgctatcga cttcggttaa agctcctcca tgcagtggag gtgatcgccg gagaacatgg
22921 gccgttttac agacggcttc gtccaattcg gctctggaac ctttccgcct ggattaaccc
22981 tttgtcaacg tcttcaagga aatccgaaac agcacttcta ccgattttga attaggctgc
23041 gacgatcctg aactccgttc ccttggcgat ggggcggaca tgattttttt cacgcttcat
23101 ctctacgagg ccgtaatgag acatcgtctt cagcgtgcga gaaaggttgc cgggtttcct
23161 gcccgtcgct gccgcaagag acgtgatcga ttcaggtttg gtttctctga tcaccttgag
23221 cagcaccctg ttttcatcgc tcaggacctc tgcaagcgat ctcatcgaag tgaaccatat
23281 cttgggatcc cccgctttgg gcttgagttc gccccgcgcg atggccagca gacgcttgcg
23341 aatgtcctct tgcggcatga tgcctatcac aatggttttc atcgcttttt tacctccagc
23401 agaacttggt ccacttcaga aaagaagtca gccagcaatt gatgtgcatc cttgaattca
23461 tacggaacgc ccgggtctct ggcatgctgg tgcatgtggt cgaaggttaa acgtcgaccc
23521 gcatacttga acttcttcgg cggttttacg gcgtgggcat tgtcataacc gagtattctt

23581 ttgccgtagg gctcatgcag tgtcagggag tagcggatgc catgaggtac atcgggcgtt
23641 ggctcaacct gataagcctc gatcttgacc caatacccgc tgccctgatc aatgatctga
23701 tcatctaaat ccaggagggt ttcaatgcca gggtccattt ccatgcacat attatatcag
23761 cggctgataa agtatccatg ctttggaaac gctagccatt gagccggtcc gcgacctgtc
23821 gcaaggcctg ctgccagcgc cgctgggcgg tccggttgca gcaggcgaaa cgccggctga
23881 tctctttcca gccctgccgc tcggcccgca tccagatcag atgccgctgc tcttcctcca
23941 gccacagcac ccagcgcatc gtctccagca tgcggtcgac cgcctcgggg ctcggctgga
24001 tacggcattc gcgagtttcg tcggcatagc cctcccaggc ctggcgggcg atggccggcc
24061 agcagttgac atacccttgc accttgaccg gcggcaggtc ctgggcggtc caggccgcct
24121 cctcgaagcg ggcggccacg cgttcgatcg tccactcagc cacggcggga ctcccggcgg
24181 ccgtacaacc gctcgccgat ctgccggatc agttcccgct cgacgaaatc cagccgttcg
24241 tcagtcagat ggaccacgag gatatgctgc tcgcgccagc cctcgcgttt gaccgcttcg
24301 aggtccgtgg cctgcggctg cagctttccc agcgggcaac tgggatgcgg gttcggtccg
24361 ttcatatgcc caccctctgg gtcgccaggg cccagtgcag caaggcgagg gcatcggctt
24421 cgttgtcgtc caccggatcg aaacccattg accgcatcgc agcgaccata gcggccttat
24481 tggcgttgcc ctggccggcg gcgtgtttct tgatcgttcc caccggtacg ccctggtagg
24541 gcatccggtg gtgctcgcac cacgctgtga ggtgggccag gaacccgcca taggcgtggg
24601 cggcatcgac cccggcatgg cggcggacct cctcgaagta gaccgcatcc agatcgccgg
24661 cggcttgctt gatctccgtc agccaacgtt tgaagcgcag aaagcgcatg ccgccgcctt
24721 cgaaacggtg tggcttgaag gtttcggagc cgctgatgat cgtaccgtcc cgctgatgca
24781 gggcccagcc ggtatgcgtg cccagatcga gggccagcag cgtggtcccg tcaccttcgt
24841 cgtgacctcc gaggctggag agacccctac gagacgggga gagggcgcgt agcgcgcctc
24901 tcccccgtag ggggagggag tctccgccat ctggaattcc ataaattgtt tcgtatgaac
24961 aatcggttgc gccagatggc gagttggcgg cgctgccatc tggaaattcc gccatctgga
25021 ccgtaagcga ttgaagtcta tggattccca gatggcggac gtttgccatc tgaatccaga
25081 tggcagattt caaggggcag atggcagagc gtctgccatc tggaatcagg gtcgtcgtca
25141 tggattgtcc tcgtcgtggt aaagccagat cgtggagtct tcaaccggca gcaaggcgcc
25201 ggtctggggg catttgtagt gggtggggta gaccgaaacc ggccacatcg ggatttctcc
25261 cgtgtcggga tcagggggac cttcccgccg cagcaccatg ccctcgacgc attggtagcc
25321 gaacttgctg cggtgtaacg ggggaagtcc gtagtcctcg gcgttacgaa agaacttgat
25381 gtaaccttga gtggcgagca ccgagagccg ctcgcggatc gtgcgttccg cgcccaggcc
25441 agcgcggccc tcgaaggctt cggcgaactg ggtggcgctg tagcagcggc cttccgccgc
25501 ctcgtcgaac agcatctgca gaatcacgtc atgcttgcgc cggcgctcgg catcgaggcg
25561 ttcggcgtag tcctgcagga cccgccgccc ggtgttcacc acctcgcgcc aagtgccgtt
25621 gaacttgtcg acggacttca ccggaagcgc cgcgccattg cgcagctcga agatgagttg
25681 gcgggtggtc tgggcctcgt ccggccggaa cagcagcatc ccggtgctgt agtagcccct
25741 gaggctgccg gcgccggcca gggcctggaa cggatcctcc tcgaagggtt tcttcccgag
25801 cttcttggtg tggtgcacca gcacgaggcc ggcatccggg ttgaccgcca tgcgcagctg
25861 ttccacccgc tgcgacagga agaacagcat cgcgccgtta tcgttttcgc ccccggcctc
25921 gccgccgtcg aatacgttgc ggatcggatc gatggcgatc agatcgggcg gagcgctgcc
25981 gaatgcctga acaatggccg ggatcacctg agccaggccg gcctcgtcca ggaccaggcg
26041 cagttgcggg gtggcgacga agttgacgcg cgcggcaccc aggcgactgg gcggcaggga
26101 gaccgtcttc acgcgctcgc ggaggtagtg gtactgcacc tccgcctgca gatagaacac
26161 ccgcaagggt cgcggcggcg tcatgccgag gaagctggcc ccggccgcca tgtgcatcag
26221 ccaggccagc aggaagtcgc tcttgccgac cttgggcgcc ccgccgaaca ccaagagacc
26281 cccgggcgtg agcacgcggg gcgcgatcag gtccggcggg acgggagagt catcatcgag
26341 caaggcgccc aaggtatgcg tcggcaatac cgggggtgcg gccttgatga cctggcgctc
26401 gccgtggatg aggaacgcga cgcaatcgaa gccctcggac acggcatcgg ctgcgtccca
26461 cttgtctggc ttgtccatgg gcggtacgag gatggcgacc gacgccgcgc ctgccgccac
26521 gcaggcacgg gcggcgttct ccgcgtagtc ccagcccggc gcatccctat ccggccagat
26581 cagcacggcc ttgccccgga gcggcgacca gtcggtcttg tcgatcggcg ccttggcccc
26641 gttcatcgcc gtggtggcgg ccacaccctt atggatcagc gcgtcggcgc accgttcgcc
26701 ttcgaccagc accacctcct tcgcctgggc gatcgccggc aagttgtaga gcggacgcgg

26761 gtcgggggcg cgccacagcc gggcacgcac gtcccaaggc cggtattcct tgccggtggg
26821 cgggtcgtag cggtagacgc aggcgatcag cgagccgtcc gccgcgacgt agtcccactt
26881 cgccgtggcg ggaccgagcc gatccaccgg cgcttccccc cgcagcggtg tcggttcgac
26941 ccttgccgta cccaacagcc ggccggccag ggccacgacc tcggcgaatt ggccgtgcgt
27001 atccaggccg tggaccgcag cgatcaaggc gaagaggtcc ccgccctcgc cggtcgcgtg
27061 gtcgtaccac agaccggcct tctcgcccga aagcgccacc tccaggctgt cgcccggatc
27121 gccctggacg ttgccgatcg cgaaggcacc cccgcgcacc ttgccggccg ggaacaaggc
27181 tttcagcacc gacggcagcc gggccagcaa atcggcgcgc acggtttcac gctgggctgc
27241 cagatcgacg gggttggccg gtcgcggcag cgcgtcgttg aagtcaagca aggccccgct
27301 ccttcggcgt gcgcttcacc ccggccccgt gcgcggtctc gctcggcaga tacccgccct
27361 tgcgggcaat cgcacgcacg aacgttgcct ccaggccgac cgcatcgcac cagaactcca
27421 acgcggagcc ggcgaggaag tgccgcgcct cggcgcggag gacggcgccc ggatcgaggc
27481 agtcgccgat ggccagggtg atcaccgcca ccgccaagtg ggcttcgggg cagggaaggg
27541 tcacatgccg acgcagcagc ttgtcgatga ccgtcagacg cacgtgaggc cggggcaact
27601 gccgctgcac ccgtttgcca tcgtaaggct gccgggtcct catgccccac ctccccggca
27661 gcgcaggccc caagcacatt gccggcactc gacgtgggcc ggatcggcaa agccccgcgg
27721 caacagctcg ccggcctcgg tggcggtgat caccctgact gcccgatccg acatccgctg
27781 tgccagcccg ccgtcgaagg ggacacgctc ggcggagatc tccatcgagt ccgcgttgac
27841 ggcggtgaac agcgccggcc gttcgtgcag cgcgaggtag gcctggtaca cggcgacctg
27901 ggctgcgtaa accggcttgg ccttggccaa gcgatgcttc accagctcgc gccaggattt
27961 cgcgcccagg catttgttct cccacaaagc cggagcatcg aagccttcgg gcccaccgac
28021 gatgacgccg tccacatggc cctggaggcg gccatccagc gccgagaacc cgaactgctc
28081 gccatcggcc tgatgggtgc gcagatcgaa gcccgctgcg cgcagccagc ccaccaccgc
28141 ctcctccatg cgatgaccgc gttcgaagat gcgcagcatc cgcccggaaa actcgcggcc
28201 gggatcgacc ggcgcctggg cgtattcgaa ctgaagcgcg cgctcgcagg ccacgccgag
28261 gcgagacgcg ccgagataag tacggatggg ttcaccggcc tggcgacgct gcagaccggc
28321 atcgatcagc gccgtgagcc gttcgctgaa cagggcgcca gggttgtaat ccagcatggt
28381 cgccccctca gaacggcacg tcatcgggat cgtgttcccg caggtggtcg aaataggtcg
28441 tcagcaccac gtcgaccagt tgcaggattt cctcccggct gtaggccgac agcggccggt
28501 ccatgccgat ggaggcgacg tagtcgccga ggcggggcag cacggccgcc atcgccgcct
28561 tctcgttagg ggtgggatcg atcacgacgc cgtcgctccg cttcagtcgt tgtaaatgga
28621 tgtgctggca gcgcctcgag cagaagcgtt tgaacagggg cttgccgtcc ggcctgcgcg
28681 ggccattcct cggcgatacc cagcaaaagc cgcgtccctc tcgtccgcag atcgcgcata
28741 tcacgccgcc ctccggtgct cgtcgttggc cgccagcacg agacgctgga tggacgactt
28801 gttgaactgg aatgcgagca gcgccgaggc ctggtatcgg gtgagtccga aatcggcgcg
28861 ctgggccggc ggcaggtagc gcagctgttt ctcggtgggc ggctcgttga gccagcggcg
28921 ggtcttgtgg gcagtgtcga gggactcgtt ctcgttcagc cagtcgtccg ccttggccat
28981 gcagacggtg cggtcgccga ccgccagcag acggggcagc aggtccctgc cgcctccgac
29041 cgcgtgccag cggccgttga ggaagaagat cccgccccag gcgccaaagc cggtggccat
29101 caaggcgtcg tcgcagccga atagatcgca ccagcggaag tgggagcgct tgagcaggtc
29161 gatctccgtc atgacgaatt cgtccagggc ttccgcctga gcggcttcct cgcggatcca
29221 ctcgaagccg cacagggggc actcgcggca gccgagtgga actgtcgccg cgcagtatgg
29281 acacgccttg gtcggcgcct cgccctggtg ccggtggccg tccaggttgg cctcctgctc
29341 cagcgagcca tgcatgagag ttgccgtgcc gaagtcgagc acgacgcaat cggtcttgaa
29401 cacaccggga tgttcggccg ggtcgacggt ccgcagcccc cgcccgatca tctgggtgag
29461 cgtcgacttg tgggagctcg gacgcagcag gacgacgcag gaggtgggcg tgtagtcgta
29521 gccttcggtc agcaccgcca cgttgacgac gagctgcgcc tcgccggttt cgtattcggc
29581 gaggcgtgtc ttgcgctcgg cgtccgacag ctcgccgtgg atcagtacgg cgcggatgcc
29641 ggcggcgacg aaggcgtcgg ccacgcattg ggcgtgggcc acggtcgagc agaagacgat
29701 ggtcttgcgc tcgccggcct tctcgcgcca gtgatggatc accgcatcgg tgatgggcgt
29761 cctgttgagg atcgtctcca cctcggtcat gtcgaagtcg gtggcggtgc ggcgcacctg
29821 ggcgagggcg gactgcgcgc cgacatcgat gacgaaggtg cgcggtggca ccagatggcc
29881 ggaggcgatg agctcgccca gggtgatctg gtcggccacg ttgctgaaga cctcgcgtag

29941 ccccttgccg tcggcgcgtg cgggcgtggc cgtggcgccg aagatccgcg cccgtgggtt
30001 gcgcgacaat acgcggtcga tcacgcgccg gtaggatggc gaggcggcgt gatgcgcctc
30061 atcgatcacc aggaggtcga gcgtcggcat ctgctcgaga tgcttgtctc gggccagggt
30121 ctgcaccatc gcgaaggtgg cccggccggc ccaggacttc tcctgggcgt cgaacaccga
30181 ggtggtccgc cccggattga cctggccgaa cttggcgcgg ttctgggcgg tgagctcgtc
30241 gcggtgggcg agaacgcagg ccttggcgtc cggctcttcc agcacaccac cggccaccgc
30301 cgacagcatg atggtcttgc ccgagccggt gggaccgatg gccaaggtat tgccatgctg
30361 atgcagcgcc gcgagcgagc gctccaccag cagggcctga cggggacgaa gcatcatggc
30421 gatgtcctcc cttactgggc ccaggccggc ttgccgccgg ggatggccga agatgcggtc
30481 ggcgccgaag gcgcataggc cggcgttgcc gctgcgggta tcgcacccga aggcgaagtt
30541 gggaccggcg gagcgaagtg ttggcccatg agctgggcat aatctttgtg atccggctcg
30601 atcaccgccc ggaccgtatt gcggtcattg ccgcggccgt ccttctcgat gtcgatgcgg
30661 ccggcgaact ccagtccgtc cagctcggcg aagccggcga tgcggcgggc gttctgggct
30721 tggggcccgg tgtcggcggg atggatgcgg cgggcgctgt tcagcaggga ccgaatgaag
30781 ctgcggccca tcgcctgcca agtcggccct ttgggtgagt gcaggccgat gttccaccac
30841 agcttgcggc gagcataagg gccatccagc accacgcctt cgcaagcgag atagacggcg
30901 ccggtctcag gactgcgggt ggcccagccg ccggtccagc cctggctggc atcgtcgaac
30961 ccgcccggct tgagggtcag gcggagccgc accagggtat ccttcggaat cagctcgaag
31021 ctcgcctgct cggcggaatt gaagtcgaaa tagctcatgg tcaggtctcc tgggtcggga
31081 tcgttacggg aacaggacgg ctgaaatcca gccgttccag cggcggacgg gcggggccgg
31141 cgatcttctg catcagctgg cccaggtgcg gcggttcgat cggatcgagc cggccggagc
31201 ggtccttggc gggaaatccc caggggttca gggtctggca gacgaacgcc cggtagcggc
31261 tgccatcgtc cgcgggcagt tcggccaggg tgaccacttc atcgacgatg ccggggagtt
31321 cgagcccggt cttggcgccg tcgatctgca gggcgaacac ccggcgattg aagtcgtcga
31381 gcttctcgtc caggatcccg acgaaccaga cgttcttgtt gcgggtgtgc tgcaagtgcg
31441 tcagccagcc gatcatctcc tggcccatca ggccgtaagc gccgcggctg tccggcttgc
31501 cggtcttctc ggagtaggcc tgcggctgcc ccttgcacca ttgcaggcac aagcgtccgg
31561 cgacggtgat cgagtcgacg aatacggtct ggtatcgctc cagcacaccg gggtcgccga
31621 agcgggcgca caccgcgtcg aagtgggctg ggctgaaagg ctggtcatcc cgcagggccg
31681 gattggggcc gccgatgaag accgcgaagt cgcggcattc gggccaagtg cgcggccgga
31741 tcgtatcccc ggcccagcct tcgaccgcga gatcgccggc ttccaggtcg aagaacaggg
31801 tcgagccggc ttccagcgtc cacagttgcg aggtcttgcc gagcccggcc ttgccgacca
31861 ggacgccttt gaccccgcgc cgttcggcca agcgctggtc ggcgctgatg atggggaggg
31921 tcataccgcg tcctccccga gcagcgccaa acggaagccg ggcttgccgg tcttgagcgt
31981 acgggccggt gcgaagctct gctggagcgc ggccggccag gcgttgaact tggtctcgga
32041 cacccggtag ctgatctcga cgtactgggc ggggtcctca ccgctgtcgg cgatccggcg
32101 caccagggcg gcgagttgcc gggcatccca ctcgaccttc ttggggaggt cggccgtgat
32161 gcgcacgggg ccgtcgtcga agtggacgac gccggtgtct ttgcccatag ccaaccgcag
32221 ttggcgggcg cgctcggcgt atctgatctc cacggcgcgg tccaggtgct cgttgagggt
32281 cttggcagcg gccagttgcg cggcggcgtc gtgcttgagg cggcacagcg attctaccgg
32341 cagctcggcc agctggccgg ccggagtggc gaggagcgtg tcggggttca tcgaggtcat
32401 accgcacctc cggcctgaac gcattggccg gggcttctgc ggaggtggcg ggcctcgtag
32461 gcctcgatat cttcctggcg atagagcacc cggccctgca gcttcagata gaccgggccg
32521 atgccctcgg accgccagcg atccagcgta gcgaggctga caccccagcg ttcagccagg
32581 gccgggggat tcaagtgttt ggggctcacg tctgtctcct tgggaggggt tgtggaactt
32641 gagggcagtt tgggcgtcgg gatgtgcggg cgtctggtac cgccatgtac gggcggatgt
32701 acgggcgtag gcgatagcgg acgccggaca acaaaaaacc gcccggaggc ggttgtgcag
32761 gaggagtgag gaggtgccgc gtcaatctac gcggaaaccg tagaagccct tgcgtcgacg
32821 ggcgatatag tcctcccatt caatgttgcc gctgaagaga ttttgcatcc tgagactgcg
32881 ggccgaggtt cgattgggat aggcggccct gagaatctcc cccgctgaaa gctcccagcg
32941 gtcgtgaagc gcttgggtgt acatgtaccg aaccgcggcc gcctggcgat caccccggat
33001 cacccagggc ttgggattcg atcgaatccg cagcgttcgg gtgtactcgt cgaactgaac
33061 ctccaagttg ggacgcaaac cggcatcggc tggcgccgcg aggatacgtt gcaggaggtc

33121 ccgatcaatg ctgggagtcc gcgtgtccgc gatgagtaac tcgcgcaggg aggcgaagcg
33181 gtaatttcgt ggtggaggga taaactcgcc cagtggaggg cccgacgtca ggaccagccc
33241 ctggtcgggg aggggcatcg catggaaata tcgaaagatc tccgcaagtc ttcgggtaag
33301 ccctcgggcg aaccagacgt cggtgtgcga cgacccgatc cgggcgctgc cgagatacca
33361 cagggcgcct tcgatggccg gtcggtggat agcgccctgg agcgcccgtg gaatgtcgag
33421 caaatcggcg atcatgctca ggaatcttgc cgatatgact tcgtagatcg ccacctggct
33481 ggcagggacc gatttcatcc ggaacgtctc cgggcaacgg tagcgatacc actgcgggtc
33541 gtcgtcctct tcgagctcga cgggcaggag ttcatcgccg cgctcggctg gataatgacc
33601 tgcaaagcca atgcgctgaa accactgtgc ctgcgactct ttggagagcg cggcgtccgc
33661 ccggagcgac catgcgggca cgccgtggag cggccggcct tcgctgccga ggatggggtg
33721 gctggcgcgt tcgaacagtt cgatcagctc aagcagcgac ggcgtggaga ggcttgtggt
33781 catcgccacc gagttctttc acgagtttcc actgggcgag cagacgatcg cacaaggcgc
33841 ggtctttctc ccgcttggtc ttgatgttgc acttgttggc atcgcgtaag acgatggaga
33901 tcgtgcgagc tcgctcccgg cccaccttct tcagccggag ggaaagtttg gcgtagttga
33961 ggtggtaatg gcggaagtcg aaggccgggc cgatcagcga ttgcgcggcg gtgtagatgt
34021 catcgatgtc cttgctccag attctgacca acagggagcg atggttcgaa tccgtgtagc
34081 cgagttccac gaccttgacg gaggccacga tctcctcgcc ggacaaatcg aaagtgcgtg
34141 acgcggccaa gctttggtag tcgtattgct tgatggggat ccgctcgccg gtgatcggag
34201 attggagcag cgactgggcc gcaatcaggg ccagcgcctc ccggtcactg ccgtgtctcg
34261 acaggacttc caggtgcccg ttggcaggtt cgtaggtgat gtgggaaggc tcagaccgga
34321 tgatgtgctg ggcgatgagc tcgctcgatt ggacacagtc gatggtttca ggcggcctat
34381 tgcaatggat gctgatctgg tagagatcga cctcttctcc ggattcggta tccgggcgca
34441 gccgcaggaa tatctggatg gcgacctctg tgggcaaaca gcccaagtgt tctgccactg
34501 ccccaaggaa ggcttgccgg ctctcagcgt cggttgccac tgcaagattt gccggcgcca
34561 tgaagccgga gtagcacgcc gtactctggc ggaacacgtc agcctgacgg gcatccaacg
34621 cttccttgaa gacggtcgga gcgtgccggt agagccagag ggctcgttcg tactgatcct
34681 tcaacgcgtc gaaagctttg cggtccgcct cgtcgaagat gccctcgcga aagccttcga
34741 cgacatcctg tccggggccg tccgtgagca gaacgatctt ctcggcaacc tcctcgatct
34801 tctggcgagt gggtacggcg agcatggcca acgcctttgt gattcgttcg cgctgttttc
34861 gcttgcgttc ctgatcttcc ggaggcggca gatcgatgcc aaactcgtcg tcgatgaatg
34921 cgcggaacag gccagggtgg agatgggtta ggagcttggc caggttttcg gcgtcgttca
34981 tgcgttcgaa ctctccaagg ggatgaaagg tgtggcttgc gcagtgcgga ccggcggtca
35041 gcgccgaaac tcaccgaaat gtgtcagacg cttgtggatt tcggcgtgtg gcaggtgggt
35101 tcggggagcc tccagcatgc cattgagtat cgcaaccagc cgacagtcat cgacgacggc
35161 gtattcggcc agcgtatcga tcacccagag cacgccatga acctcgacgc attcttcctc
35221 ggccagcgcc cgcatgcgct tgtctcccgt cagcagcggc cagccgtggg tgaccgccag
35281 tgtcaccgcg aagcagtcgt ggatggtcag tttgggcttg gccaagcggt aggccaacgc
35341 ctgcgtcatt ccggccgggt ccagggcttc tacgcgcagg ccaagcctct tgagctgttc
35401 gcggtggact gcaccgagat ccagcagttc gtcttcgtac aaagcgtcgg ggacagcaaa
35461 ttcgaagggc agctggaaga tgtcttccag aagggaccag ttagccagct caatcagaat
35521 cgacgcatcg ctcaccagga cccgcacgct cgatcactcc agcgggcccg acatggttcg
35581 gctaacctcg ccggtactct tgcccagcag ctccgccgct ttcggcagtg agatgacgtc
35641 ttctgccagg gcgcggagca ccagccgttg gaaccgtttg ggcgtttcat cctccgtcag
35701 cggttcgggt tcgcgcttgc gccaagtgcg gccgataccc cgaaacaggg tttgcaccgt
35761 accatcgccg atgaccccga gatcccgcag tcgcacgatc agcgcggcgg cactgacgcc
35821 gtacatgcgt ttgatctgga tgacctcggt gtagccgaaa gcgtggcggc gctggccgac
35881 ctcgaacaaa agatcctgct tcggcatcaa caaggcactg gcgaaacgct ggcaggcctt
35941 ctcttcatcg atatcgccga cgattgccat gaccatgtga cccaactcgt gcgccagggt
36001 aaagcggcgc cgttccacgg acttccccat cgcgcccacg atgactggga ctttttcctg
36061 acccggccgg gcgaccaggc aggtcaagcc gtcgaccgac aaggggaagt cgagcttgag
36121 caccttgatg ccgtgctctt cgagcagttc ggtcatatgc gggatggcat caccgccgag
36181 attccaggcc gtccggatct ggtcggtagc gtcctcggct tcctccaagg tggacaccgg
36241 gatcggcgct cccaccggtt tgtcccactc gtggctgccg atctcgagca ggtcctcaat

36301 caggaggtaa cggtcgacgt ggtcgaggac ggcagcttcc accatggcgc gctcccgcgc
36361 ccgggttgct gccgttttgc ggaactctac cccctcgagg cggatctccg aggggctgaa
36421 caggtaactc agcgtgactc caagggcctg ggcgagcttc agtgccacgc ttgatccggg
36481 cagcatctcg cctcgttcgt aacggccgat cgcctgagcg ctgaccgcat gatcgacggc
36541 ttcggccagt ccccgcaggg acaagccggc acgcttgcgg gccagtttca atcgctcacc
36601 gaacatgggg ttcctccgtg tttacagtgt acttatagag gtttgatatg taaacctagc
36661 gagcgctggg cgaatgtccg attctcgtct ctggcatcgg ccctcaggac accgcatcgg
36721 ccctcaactc cctcgtggtg cagggatgga atgcgtcgga caatggctca cggaatcatt
36781 tgcacctcgg gaacgttatg cttgccacct atcttccgtc ggaacgcctg tcccccgccc
36841 aacgcgccca cgagatcacc acgatcctgg ccggcgccct ccttcgtact tatccgaccg
36901 aacccggaaa aaagaccggg tgtgaccttg gcttactgcc cgggaagcgc gtccatacga
36961 cttcctctca accggagtct gtgtgatgaa cgatttgaaa ctctcggtgg cggcccaagt
37021 ggcctcgctc ccgagcctga agatcaaaga cctctggacg ctgtgggata aatatttccc
37081 gcgccgcccg gcccatccca accgccccta cctggaatcc cggattgcct ataagatcca
37141 ggaagccgcc tacggcgggc tttcgcccga gactcgccgc cggctcgagc agatcggcca
37201 gcgccattcc aagatcaagg cccggcgccc ctcgtcgacg atccacctgc cgcccggcac
37261 cgtgctggtg cgggaatggg gcgagcagga ccacaaggtc atcgtcaccg ccgaagggcg
37321 gttcgactac gccgggcaat cgttcaagag cctgaccgcg gtggcacggc acatcaccgg
37381 caccgcctgg tccggtccgt tgttcttcgg cctacgccaa gccggggagg ggacatgagg
37441 gcatcgaacc ggatggaagt ggcaaccagc gccccgtcca agccccgcca acgctgcgcc
37501 gtctactgcc gggtgtcctc cgacgaacgg ctggaccagg agttcaactc catcgacgcc
37561 cagaaagagg ccggccacgc ctacatcgcc agccagcgga tcgaaggctg gatcccggtg
37621 gccgacgact acgacgatcc cggctactcc ggcggcaaca ccgagcggcc ggcgttgcga
37681 cgcctcctgg ccgacatcaa ggccggccga atcgacatcg tggtggtgta caagatcgac
37741 cggctgaccc ggagcctggc ggacttctcc cgcatggtcg aagtgttcga gcgccagggc
37801 gtctccttcg tctcggtcac ccagcagttc aacaccacca cctcgatggg gcggctgatg
37861 ttgaacgtgt tgctgtcctt tgcccagttc gaacgcgaag tcaccggcga gcgcatccgc
37921 gacaagatcg ccgcggccaa gcgcaagggg ctatggatgg gcggggtacc gcccttaggg
37981 tatgacgtcg ccgaccggca gttggtcgtc aacccaaggg aggctgccgt ggtccgccgg
38041 attttcgaag acatgctcag cgtcggctct accacccgga tcgccgcggc gctgaatgcc
38101 gaagggatca ccaccaaagc ctggaccacc caggacggca agcagcgccc cggcgcccgg
38161 atcgacaaga aacacctgca ccacctgctg cgcaaccgga tctacctcgg cgaaatctcc
38221 cacaaaggca gttggcatcc cggcgcgcac ccggcaatca tcgaagccac cttgtgggac
38281 gctgtccacg ccgtcctggc ccgggatgcc cgcagccgcg ccaccgaaac ccggcagcgg
38341 gaacggaccg acgcgctgct gcgcggcctg ctctacgacg ccgagggcga gaagatgtac
38401 ccgacctacg tgaagaaaaa cggccgccaa taccgctatt acttctccaa ggccgaagcg
38461 cgtttcggcg caggccaaaa gaccagcgtc cggctcccgg ccgaggaaat cgaagcggcc
38521 accctcgccc agatccggac cgtcctggcc agccccgaag ccatcgcggc catatggctg
38581 gcggtgcagt cgcaaacctc cgacatcgac gaagcccagg tggtggtcgc cttgggcaac
38641 ctcggcagcg tctgggaaca gctgttccca gccgaacggc accggatcgt ccagctcatg
38701 atcgagcggg tcgagctcgc cgacggcggt ctcaggatcc gctggcgtcc cctcggctgg
38761 aaagcgctgc taggcgaatt cactccgaaa acccttggcg ccgaactggt ggaagcggag
38821 gcggccgcat gaaagccgat gccttggaat ccttcgtgca gatcactttt cagcggcgcg
38881 gggtgcaacg gctggccgcg accaccgcgc cagcgcatga cctcccgttc ctggtcggcc
38941 tgggcagggc gttgtactgg cagcatctcc ttgacaacgg cgtcgtccgc agcggctccg
39001 acatcgcccg ccgggaaggg ctgcacccgt ccacggtgaa cgaactgctg cggttgaccc
39061 tgctcgcgcc ggacctcatc gaacggctca tggctggacg ccaagctcgg gctctgacgc
39121 tgaagtggtt ccagcggcat ccgctgccgg tcgattgggc agagcagcgg aatttcatgg
39181 cgagtttcga atagaggcga caatggccaa gaaagaccgc ggcatcatca tcggcaaacc
39241 ccgcacggta tcccttcccc agccggccgg tggggttcag ttggaaacct tcctgccctg
39301 gacgctggtg aagcgtgggg tgaagcgcga gatcctcacg ccgctgggga cgccagcggc
39361 gtttcgggag gaggtgcagc gggaacggaa gcaacgccaa gccgagcagg acacgccgct
39421 cattcgagcg ctgggtctgg cgcactactg gcagcggttg ttggacgagg gaaagctcga

39481 gtcgctgtcc gacatcgcgg cggcggaggg catgaatctg agtcaagtca gccgaatcgc
39541 acggctgggg agattggcgc cggggatcgt ggaggcttgt ctggccgaga aggactcagg
39601 attgaccctg gaagacttga atcggtgggc gaactctttc cgctggccat cgcgcgagtg
39661 gccgagcgct tgatccccat ttttggggag caccggcggc atgctgatgg gcgcgtacga
39721 ctgtattatc cagaaagact tcttcagagt cggcctcttg cgccagttcg gcgaaaaggc
39781 gatggggggt gtcagaattt ctgtgtgtga ggcgagagta gccttttcca cggtgacgtc
39841 taccgaaggt ccgttcaggc cgtaggcagg cggcggggtg ggaaaggtgg tggtcatttt
39901 atccgcgggc cgcttcaaag cgttcggcgt agaggatggc aaattggttc atggcctcct
39961 tccagtcctt ggccgcccgt ccccaatccg ccgtgatgtt gcgcaaggcg agccagatca
40021 gcttggtggc ggcatcgtcg ctggggaaat gcccgcgggt cttgatgatc ttgcgcagcc
40081 gggcgttgat gctctcgatg gcattggtcg tgtagatcac ccggcggatc gccggcggga
40141 aggcgaagaa ggggatgacc cggtcccagg ctcgccgcca ggcagcggcc acggtgggga
40201 atttctcgcc ccacggccct cgcgcgaagg cctccagttc ggccagggcc gcttcggcgc
40261 tgggggcggt gtagatcggc ttgatcgccg ccgccagcgc cttgcggtcc ttccaactcg
40321 cgtagtcgag gctgttgcgg atcagatgca cgatgcaggt ctgcagcgtg gtagccggga
40381 agacggcagc caaggcctcc ggcatgccct tcaggccgtc ggtgacggcg atcaggatgt
40441 cggccacgcc gcgggtcttc aaatcgttga agaccttcat ccagaacttg gccccttcgg
40501 tgttctcgat ccacaacccg aggatgtcgc gcgtgccgtc gggcaacaca ccgagggcca
40561 gatagatcgc cttgttccgg accacggcat cttcgcgaat cttgacccgc agggcatcga
40621 agaacacgac cgggtacatc ggttccagcg ggcgcgattg ccaggcggtg acttcggcca
40681 tcacgacatc ggtgaccgaa ctgatgaatt cgggcgaaac ctcggtgcca tactgctccg
40741 ccaggaaacc ctgaatttcg cggacagtca tgccccgggc gtacatggcg acgatcttgt
40801 cgtcgaagcc ggtgaagcgc cgttcgtgct tggggatgag catgggctcg aaactcccct
40861 gccggtcgcg cgggatgtcg attcgcaatg ggccctcttc ggtcagtacg gtcttcttgg
40921 tggcgccgtt gcggtggttg cagacttcgc cgggcttgtc ggcgccaggc gggtaaccca
40981 ggtgatggct gagttcggca cccagcgcgc gttcgatcaa cgccttcttg aacgccatcg
41041 acgcggcatt gaccgcctcg gcgctcatcg ggccgctgat aaaatggtca atgagttctt
41101 tgggaaggct gggcagcgcc gctgccgggt tcttcttgct cgtcttgctt ggca
//

```
LOCUS       16-5-R1              67726 bp    DNA     linear   PHG 16-FEB-2025
DEFINITION  16-5-R1.
ACCESSION   16-5-R1
VERSION     16-5-R1
KEYWORDS    .
SOURCE      .
  ORGANISM  .
            .
FEATURES             Location/Qualifiers
     CDS             complement(1..1038)
                     /ID="CZTTGXGG_CDS_0001"
                     /transl_table=11
                     /phrog="1"
                     /top_hit="No_MMseqs_PHROG_hit"
                     /locus_tag="CZTTGXGG_CDS_0001"
                     /function="integration and excision"
                     /product="integrase"
                     /source="PHANOTATE_1.5.1"
                     /score="-78837.5869224805"
                     /phase="0"
                     /translation="MSLYKRKDSPHWWIKLHVNGRRLQESTGTADRKRAQELHDKLKAE
                     LWEQSRLGVKPGYSWQQAVLRWLDDTEEKASRRDDINRLRWLHVHLEGVMLSEVDRYKL
                     DAVISAKKGEGASNATINRHLAIVRSILRKAVHEWGWLDSMPKVRMLPEPKQRIRFLTK
                     DEAERLLAELPAHLADMVRFSLETGLRQANVTGLLWNQLDLVRKVAWIHPDQAKARKAI
                     AVPLSDVAVEIIRRQLGKHHTHVFSYRGQSVSRVNNHAWTKALGRAGIENFRWHDLRHT
                     WASWHVQRGTPLHVLQELGGWESVEMVKRYAHLSGEHLAQYVTSMTGCPRRVVTNRLRS
                     ETKKG"
     CDS             complement(1013..1114)
                     /ID="CZTTGXGG_CDS_0002"
                     /transl_table=11
                     /phrog="No_PHROGs_HMM"
                     /top_hit="No_MMseqs_PHROG_hit"
                     /locus_tag="CZTTGXGG_CDS_0002"
                     /function="unknown function"
                     /product="hypothetical protein"
                     /source="PHANOTATE_1.5.1"
                     /score="-0.0563972570856619"
                     /phase="0"
                     /translation="LIWPPGCADNTPRGRCKVTARKRKTHVTLQTQR"
     CDS             complement(1274..2563)
                     /ID="CZTTGXGG_CDS_0003"
                     /transl_table=11
                     /phrog="1159"
                     /top_hit="No_MMseqs_PHROG_hit"
                     /locus_tag="CZTTGXGG_CDS_0003"
                     /function="unknown function"
                     /product="hypothetical protein"
                     /source="PHANOTATE_1.5.1"
                     /score="-508205.82625183265"
                     /phase="0"
                     /translation="MADLKLFQRYSYSSLSKGTEEDPKGRIFKTPRITWDLSHILICGT
                     FVDTVRQNYHGQLKPELYQKIKEAAEGSGEFRFLEGEHPWAARRMGKACGYRYALQDNT
                     RGIILLIGSYYQAEVDLLEGRPAHHLKIELSPQLLAEANANAIQTTLDSFACQLMSAHE
```

ASGVAVHLAVDVQQWSPDPGFQNRFITRARITRRFDGISDAEFHGLSEVAVTYGDKAAE
SLTFGKAASLQLCLYDKSKQIVHADKVDFMQAQWGEFTEGEFDPSQPVWRIEARFHHSV
VNEIGEGMGKPLKRWLDVVPHLTDMWRYALTINRAKDGTYIDPVWQLLRDDPEFKVPAS
GYKIVRKKKEDVSAVARNFGNVLGNLISLYAREGRNADYILIQIKRLSIYPKLLEHLAR
RGQAKDQVEQDLKDFIREGLWKRRTLGKAA"

CDS complement(2630..2857)
/ID="CZTTGXGG_CDS_0004"
/transl_table=11
/phrog="No_PHROGs_HMM"
/top_hit="No_MMseqs_PHROG_hit"
/locus_tag="CZTTGXGG_CDS_0004"
/function="unknown function"
/product="hypothetical protein"
/source="PHANOTATE_1.5.1"
/score="-1.3227739207397726"
/phase="0"
/translation="MPATAKGRAVPLRALRPGCRAPAGALHRSAHAACRARAGARPRRA
AAQPVRRACNTSDLGGCKASFEHSEAQKTV"

CDS complement(2841..3074)
/ID="CZTTGXGG_CDS_0005"
/transl_table=11
/phrog="No_PHROGs_HMM"
/top_hit="No_MMseqs_PHROG_hit"
/locus_tag="CZTTGXGG_CDS_0005"
/function="unknown function"
/product="hypothetical protein"
/source="PHANOTATE_1.5.1"
/score="-7.06343044343244"
/phase="0"
/translation="MAAPDFSSLIPSIDIDFHSVASAVMYVGTAIAGVLVVVRGVGLVH
SAVLGKTLLYEEKPEKADDFDLTQRLKDAGYR"

CDS complement(3083..4207)
/ID="CZTTGXGG_CDS_0006"
/transl_table=11
/phrog="197"
/top_hit="No_MMseqs_PHROG_hit"
/locus_tag="CZTTGXGG_CDS_0006"
/function="moron"
/function=" auxiliary metabolic gene and host takeover"
/product="Zot-like toxin"
/source="PHANOTATE_1.5.1"
/score="-8346.683180581751"
/phase="0"
/translation="MSILAYVGLPGAGKSYGVIENVILPSVKRLRPIWTNIPLVMQEWE
IVWPDVEIRVFDNAQAQQPGFWAGLPGGAVIVIDEAWRFWPAGIKSADLTEEHKSFFAE
HRHKVGANQYSQEIVLIVQDLTNLCSYVRNLVDKTFVATKLDTLGADKRYRVDIYGGPQ
RGPRYPVKAAIRQIFGSYKPEVYRLYKSHTKALDDMVGIESKPDGRATIWGNNLLKFGV
PAAVLFGVLAIRGVWGFFHPEPKAALAEKPKVLPEYTGEPVAVAATPTPSPLPQATSPP
VAEATPLPTPEPKVSDAWRLVGFSGEGESRRVYLQDKRRKFRVISARRCVDLEVEPQCL
VDGEIVARWSGPAPVQTPFHIAESAFQPPAHPGP"

CDS complement(4291..4683)
/ID="CZTTGXGG_CDS_0007"
/transl_table=11

/phrog="974"
/top_hit="No_MMseqs_PHROG_hit"
/locus_tag="CZTTGXGG_CDS_0007"
/function="head and packaging"
/product="minor head protein"
/source="PHANOTATE_1.5.1"
/score="-9.616077632029054"
/phase="0"
/translation="MSGPSSRFSFCSALSLWVLALVYAGQAHAEGLFPDMAQFVQWIWD
ALLYVPKWIWSHLLEILGDALAGSFLLGCCSALIAVPTVVSGLMGAVAASGVGFFLAPF
QAAYGLGMICCAYGVRFLIRRLPFIG"
CDS complement(4640..6214)
/ID="CZTTGXGG_CDS_0008"
/transl_table=11
/phrog="11232"
/top_hit="No_MMseqs_PHROG_hit"
/locus_tag="CZTTGXGG_CDS_0008"
/function="unknown function"
/product="hypothetical protein"
/source="PHANOTATE_1.5.1"
/score="-34206.92100592641"
/phase="0"
/translation="MRLRHLLTVLSLVSVLALLPGPAVAVPSLTNLVAPLNALSTAFSN
GAKAWEALSGFRGPFSPVGAAIGLGIGIGLYELCDNLNCYQYLPGENLPYPAVPGWSDP
DTPPASVPGTSNYTYYDSRGWIKGTFTDPAAACVQFCANAYGPTVTECLPRSGYSNPDG
SGYYDCNGPGGCNCIIAVSSASTTCGTCYVDDGKGHCLLQSACPNGAKAQWPSDGKPTY
RAGRTGWVAAERDPDVGTHQDTRTGADAHANSVRETIKPTPDGGADYVRDTESVGSDGK
PVVNRDQVHTDANGNVTSSTTTSYNNTTITNVDNTTGAGTTIDTSSLNQEATQQRVAAA
SEATKTNTDAVKQDVALSRVAVEGAKVDLAAIRGDSAASRASLDSIDRTLKAQPTVDIR
GLDLEVTQQRVATATEAAKESLDNLDKKLIKDTTKYGQVPQIDDFDTTWQIFIQKVQTG
PLGAMFLVSASEPSGGCVTYPVDLAWFNRAFVIDQHCTIWAEVSPTIASFMPYVWSLIA
VFILFSA"
CDS complement(6184..6321)
/ID="CZTTGXGG_CDS_0009"
/transl_table=11
/phrog="No_PHROGs_HMM"
/top_hit="No_MMseqs_PHROG_hit"
/locus_tag="CZTTGXGG_CDS_0009"
/function="unknown function"
/product="hypothetical protein"
/source="PHANOTATE_1.5.1"
/score="-1.908387649507784"
/phase="0"
/translation="MTKTTLTMNSMTKTTSMRWSVMADLYYWTFFVMGAACAYGIFSRF
"
CDS complement(6432..6710)
/ID="CZTTGXGG_CDS_0010"
/transl_table=11
/phrog="2186"
/top_hit="No_MMseqs_PHROG_hit"
/locus_tag="CZTTGXGG_CDS_0010"
/function="head and packaging"
/product="virion structural protein"

/source="PHANOTATE_1.5.1"
/score="-4.630133846768551"
/phase="0"
/translation="MFATARKFCSESSAKVVNAARSHKAEVVTATGTGLAAANDLVFAA
GPDFTTLTTGIDFGTAATAIMAVGVAVAAALIALRGTRMVLSAIGGR"
CDS complement(6741..6992)
/ID="CZTTGXGG_CDS_0011"
/transl_table=11
/phrog="No_PHROGs_HMM"
/top_hit="No_MMseqs_PHROG_hit"
/locus_tag="CZTTGXGG_CDS_0011"
/function="unknown function"
/product="hypothetical protein"
/source="PHANOTATE_1.5.1"
/score="-3.919868546565547"
/phase="0"
/translation="MRYVPICDGLLVDTGGPVSPTCAGTWLLNIDASGIPGAFDISTLD
PIACLSAFSSGFVIVGSVLLVGLAVAMVLDAIRGDRRD"
CDS complement(7022..8296)
/ID="CZTTGXGG_CDS_0012"
/transl_table=11
/phrog="22642"
/top_hit="No_MMseqs_PHROG_hit"
/locus_tag="CZTTGXGG_CDS_0012"
/function="unknown function"
/product="hypothetical protein"
/source="PHANOTATE_1.5.1"
/score="-207.24898531040887"
/phase="0"
/translation="MLGLPLLPRRSRKEPRQELPAPTSPALVLGTGRKPRHPGRQLPLR
TLIPLHRRPARRDRPRHRRTPPRLRRSPRLVPGLRPQPPGSGQSQWRHAAPGSGSGSGF
SSSVSVSFQFPQLLPPARCPYAHTGQTGCTPKRVNPKRRKPMQIVISAPVIGANRYHID
GSKGGSVYVLQPAQDDAQNHLGLQVQKFNAPYDLVEQLSQKPLPGHYDLLVDIERGSQD
SDKESVLSLAGSGHFELAALYDLFHIDPNAPVSKPHPDSQRPLAGALRGLVVSATRYDM
TDDGGGKGGRLYILQPTSGRNPNQFGLELIRLRMPYEIFAQLQALQVPGEYLIDVQLGR
GAGDKAQLRVTGLRPDNLLDKARLHELFKLGKDGQPLQPGQAGNGSGTASAAKPEPNPS
GAGAKPGAAPSPGALSGPEPAPVRA"
CDS complement(8558..9064)
/ID="CZTTGXGG_CDS_0013"
/transl_table=11
/phrog="No_PHROGs_HMM"
/top_hit="No_MMseqs_PHROG_hit"
/locus_tag="CZTTGXGG_CDS_0013"
/function="unknown function"
/product="hypothetical protein"
/source="PHANOTATE_1.5.1"
/score="-5.059731879841407"
/phase="0"
/translation="MIVLTYATPGSGKVAPRALGPTVRALLTASAPRSFGTRWPAHGNR
PTRRERPATPVTCCDQPTPWRIPSRQAQKSCAPVPQSQGADPTPKGPLFRSPGRSHRPP
VISQAGRPTGTSPVPTVRASFIPPADRARPGLDAPKPVEPDAVSELLASALWLWRLNRE
LACAY"
CDS complement(9061..9186)

/ID="CZTTGXGG_CDS_0014"
/transl_table=11
/phrog="No_PHROGs_HMM"
/top_hit="No_MMseqs_PHROG_hit"
/locus_tag="CZTTGXGG_CDS_0014"
/function="unknown function"
/product="hypothetical protein"
/source="PHANOTATE_1.5.1"
/score="-1.1821569709833144"
/phase="0"
/translation="MFPQPPRTRPPFASPTSNPSGPLGWGRGPETYSYRQPEVRP"
CDS complement(9190..9411)
/ID="CZTTGXGG_CDS_0015"
/transl_table=11
/phrog="No_PHROGs_HMM"
/top_hit="No_MMseqs_PHROG_hit"
/locus_tag="CZTTGXGG_CDS_0015"
/function="unknown function"
/product="hypothetical protein"
/source="PHANOTATE_1.5.1"
/score="-3.9943223831498655"
/phase="0"
/translation="MRTDHRTPQCECLTRTGERCVLPGTWFQELPNGREVRICSTHIQR
TRRGADLHFIPAPAAVIGTGNAQIPGRT"
CDS complement(9413..9742)
/ID="CZTTGXGG_CDS_0016"
/transl_table=11
/phrog="No_PHROGs_HMM"
/top_hit="No_MMseqs_PHROG_hit"
/locus_tag="CZTTGXGG_CDS_0016"
/function="unknown function"
/product="hypothetical protein"
/source="PHANOTATE_1.5.1"
/score="-8.475523159111532"
/phase="0"
/translation="MTPHPPIAPMPYGIPDTPDRETVGGLVDRVDLISQDLAGVLQRVT
QLAMEFPLETTLLISQVHLKGTLRRVQHVRDRFQILVDDHADTRAEAEADAQAQARTFN
PEEDS"
CDS complement(9744..10199)
/ID="CZTTGXGG_CDS_0017"
/transl_table=11
/phrog="No_PHROGs_HMM"
/top_hit="No_MMseqs_PHROG_hit"
/locus_tag="CZTTGXGG_CDS_0017"
/function="unknown function"
/product="hypothetical protein"
/source="PHANOTATE_1.5.1"
/score="-30.37506049503954"
/phase="0"
/translation="MNTQTVPQSLAVPTTGYHRRVFDCILSEVADTERYCRSGHCVGQS
DGFSRALQACDHSATLIHGMVLPYLREANDPDRQLYAFAELASGYVRTLWKALGGVVGP
SPEDLTTRQRYRAEVERLTGASAPEAAPAEASAPNANGRRVGDCEAA"
CDS complement(10196..10438)

/ID="CZTTGXGG_CDS_0018"
/transl_table=11
/phrog="1864"
/top_hit="No_MMseqs_PHROG_hit"
/locus_tag="CZTTGXGG_CDS_0018"
/function="unknown function"
/product="hypothetical protein"
/source="PHANOTATE_1.5.1"
/score="-14.174922094011684"
/phase="0"
/translation="MDLEALRQALLALPSDTKTAEKFAEETGLTQDTVRGLVERGQVKT
IRMGRRVLINMVDIRVRCLADLDLSFLNLQGEAER"
CDS 10562..10840
/ID="CZTTGXGG_CDS_0019"
/transl_table=11
/phrog="147"
/top_hit="No_MMseqs_PHROG_hit"
/locus_tag="CZTTGXGG_CDS_0019"
/function="transcription regulation"
/product="transcriptional regulator"
/source="PHANOTATE_1.5.1"
/score="-41.94155447689193"
/phase="0"
/translation="MIGQRIKELREFKGLSRRELERLTGIPDYKWVSIETERQQTNGEH
IAALAKIWPEYKHWIVFGETIPEVGQISPELEETREKLAKAGGSPNG"
CDS 10837..10929
/ID="CZTTGXGG_CDS_0020"
/transl_table=11
/phrog="No_PHROGs_HMM"
/top_hit="No_MMseqs_PHROG_hit"
/locus_tag="CZTTGXGG_CDS_0020"
/function="unknown function"
/product="hypothetical protein"
/source="PHANOTATE_1.5.1"
/score="-0.3461563645437169"
/phase="0"
/translation="VIERWGTGWVILGGLSSFSLEAKFNIPQDR"
CDS 10950..11480
/ID="CZTTGXGG_CDS_0021"
/transl_table=11
/phrog="36926"
/top_hit="No_MMseqs_PHROG_hit"
/locus_tag="CZTTGXGG_CDS_0021"
/function="unknown function"
/product="hypothetical protein"
/source="PHANOTATE_1.5.1"
/score="-328.23101616743287"
/phase="0"
/translation="MKPDQRTLLKSWRDEIGTMMTTHHQLSTKIRRLNYLLGIPTIVMA
MLIATYVFFTVNRDPDLWIRMLVGLIALLVAILSSLQTFLKYSEQAENHRNASARYQAL
FNALDQSLVIPPKDEAALGDWCDKLRERWDELNLEAPTVPNRLEVRSILDFEATAIHPS
QKHDTENSERQTT"
CDS complement(11534..11746)

/ID="CZTTGXGG_CDS_0022"
/transl_table=11
/phrog="No_PHROGs_HMM"
/top_hit="No_MMseqs_PHROG_hit"
/locus_tag="CZTTGXGG_CDS_0022"
/function="unknown function"
/product="hypothetical protein"
/source="PHANOTATE_1.5.1"
/score="-3.489627720553108"
/phase="0"
/translation="MASLEQVSARFVQAFETGWPQSARINSETEEQAWKTLTPKRGSLR
SPFKDSRSWVLGISGMFFPRRFRTG"
CDS complement(11830..12081)
/ID="CZTTGXGG_CDS_0023"
/transl_table=11
/phrog="No_PHROGs_HMM"
/top_hit="No_MMseqs_PHROG_hit"
/locus_tag="CZTTGXGG_CDS_0023"
/function="unknown function"
/product="hypothetical protein"
/source="PHANOTATE_1.5.1"
/score="-39.8991864684472"
/phase="0"
/translation="MVTSNLISLRTRAEIPEFESVKSYIDTIIPPYTLEDIYGFNRVYK
EAYPYLNPRQRRVIENFVDIMIDNLSDKALAKRIFGVV"
CDS complement(12275..12379)
/ID="CZTTGXGG_CDS_0024"
/transl_table=11
/phrog="No_PHROGs_HMM"
/top_hit="No_MMseqs_PHROG_hit"
/locus_tag="CZTTGXGG_CDS_0024"
/function="unknown function"
/product="hypothetical protein"
/source="PHANOTATE_1.5.1"
/score="-1.1243870928758004"
/phase="0"
/translation="MHVFQKKTIRTSPDSVISQFALILTTPALFRAFF"
CDS complement(12399..12551)
/ID="CZTTGXGG_CDS_0025"
/transl_table=11
/phrog="No_PHROGs_HMM"
/top_hit="No_MMseqs_PHROG_hit"
/locus_tag="CZTTGXGG_CDS_0025"
/function="unknown function"
/product="hypothetical protein"
/source="PHANOTATE_1.5.1"
/score="-8.633958420588833"
/phase="0"
/translation="MGLVLQSMAQRLDTLLQNLILGEALQDSSLRLHISCVTLHPCCYK
YSNIM"
CDS complement(12579..12908)
/ID="CZTTGXGG_CDS_0026"
/transl_table=11

/phrog="No_PHROGs_HMM"
/top_hit="No_MMseqs_PHROG_hit"
/locus_tag="CZTTGXGG_CDS_0026"
/function="unknown function"
/product="hypothetical protein"
/source="PHANOTATE_1.5.1"
/score="-6.989868784932562"
/phase="0"
/translation="MKYRNQIVLILAATVVNYSAVLHARDPGVNQVGAAGNVHRDSGIN
QPGAVGNVGTPGVGRGAGVNPLGPAGNVHRDVGVNQPGAVGKLNAPAAGRNPGVTPPGP
AGNWR"
CDS complement(12984..13184)
/ID="CZTTGXGG_CDS_0027"
/transl_table=11
/phrog="No_PHROGs_HMM"
/top_hit="No_MMseqs_PHROG_hit"
/locus_tag="CZTTGXGG_CDS_0027"
/function="unknown function"
/product="hypothetical protein"
/source="PHANOTATE_1.5.1"
/score="-14.417837966924505"
/phase="0"
/translation="MSRQVIDHGLTISLIQKIVRTSNVLRQFGNRGKHSLNVSQWLSSR
KVLFLTTPYETAHVDFVRKAA"
CDS 13083..13427
/ID="CZTTGXGG_CDS_0028"
/transl_table=11
/phrog="5869"
/top_hit="No_MMseqs_PHROG_hit"
/locus_tag="CZTTGXGG_CDS_0028"
/function="unknown function"
/product="hypothetical protein"
/source="PHANOTATE_1.5.1"
/score="-82.74619332208181"
/phase="0"
/translation="MLTTVTELPEYIRCANDLLDEADRKAVIDYLAAHPRAGDVMEGTG
GIRKLRWGRGNRGKSGGVRVIYYYHDERLPLFLLTVFGKNEQANLTKAERNSLAKLIDV
LVTTALEKKR"
CDS 13424..13708
/ID="CZTTGXGG_CDS_0029"
/transl_table=11
/phrog="2104"
/top_hit="No_MMseqs_PHROG_hit"
/locus_tag="CZTTGXGG_CDS_0029"
/function="unknown function"
/product="hypothetical protein"
/source="PHANOTATE_1.5.1"
/score="-9.216189180790144"
/phase="0"
/translation="MSRAFESIAQGLKEAIALTEGQPVPAKTHRPDEVDVAELRHSLGL
TQMEFAAKLRISVGTLRHWERGDRQPHGPALTLLHVVAKEPGAVLRALG"
tRNA 13793..13887
/ID="CZTTGXGG_tRNA_0001"

/transl_table=11
/trna="tRNA-Lys(CTT)"
/isotype="Lys"
/anticodon=CTT
/locus_tag="CZTTGXGG_tRNA_0001"
/source="tRNAscan-SE_2.0.12"
/score="39.2"
CDS 13920..14048
/ID="CZTTGXGG_CDS_0030"
/transl_table=11
/phrog="No_PHROGs_HMM"
/top_hit="No_MMseqs_PHROG_hit"
/locus_tag="CZTTGXGG_CDS_0030"
/function="unknown function"
/product="hypothetical protein"
/source="PHANOTATE_1.5.1"
/score="-0.0588727930077742"
/phase="0"
/translation="LCPSTGHGWRGSRIWPLDQPSPKKPVSGQNRLNFSLCSLSSS"
CDS 14045..14347
/ID="CZTTGXGG_CDS_0031"
/transl_table=11
/phrog="No_PHROGs_HMM"
/top_hit="No_MMseqs_PHROG_hit"
/locus_tag="CZTTGXGG_CDS_0031"
/function="unknown function"
/product="hypothetical protein"
/source="PHANOTATE_1.5.1"
/score="-0.1000541497247414"
/phase="0"
/translation="LTVKRPNPGKSRVPACRVATVNWSAVGIENRNFGTTITLGSITPR
AQRWRAIQWTPPTMPPTSWPAAAKPPCSWPTGRTRARACEMAEPHRDCRRDHRHD"
CDS 14370..14810
/ID="CZTTGXGG_CDS_0032"
/transl_table=11
/phrog="27171"
/top_hit="No_MMseqs_PHROG_hit"
/locus_tag="CZTTGXGG_CDS_0032"
/function="other"
/product="hydrolase"
/source="PHANOTATE_1.5.1"
/score="-43.70145774289792"
/phase="0"
/translation="MLGATAPDWLEWLITPVRKVRHRTVTHYLVLWIAAVLFFFLVWDF
HHLGQAFSLGGLFHLLTDAMTISGIPVGWWSDRKFYLFGGRLKTGSAEEYMIAGVVLMI
CALIVWHRPDNRFTPFFFDWGGYYEQGLIDGREWKENRFRWL"
CDS 14874..15230
/ID="CZTTGXGG_CDS_0033"
/transl_table=11
/phrog="No_PHROGs_HMM"
/top_hit="No_MMseqs_PHROG_hit"
/locus_tag="CZTTGXGG_CDS_0033"
/function="unknown function"

/product="hypothetical protein"
/source="PHANOTATE_1.5.1"
/score="-53.33118250412286"
/phase="0"
/translation="MGYSCADIPEPRVQTSREVEGLEGVTVWLVAGEGENPKAYFLAAK
FVASHCERNSTSGSKFPYLIAGPGSLYKKSIPLGGTSVLAQIRKESANFVRGFYETKNP
SIVSALNALAPTKS"
CDS complement(15268..15945)
/ID="CZTTGXGG_CDS_0034"
/transl_table=11
/phrog="8809"
/top_hit="No_MMseqs_PHROG_hit"
/locus_tag="CZTTGXGG_CDS_0034"
/function="unknown function"
/product="hypothetical protein"
/source="PHANOTATE_1.5.1"
/score="-134.87886380428617"
/phase="0"
/translation="MRFLFLWLCLTVTASAAAESLAGRVVSVLDGDTITLLSVGNVQTR
VRLAQIDAPEKRQDYGQASKRALSDWVSGKKVTVEVADTDRYGRSVGKVLIGGTDVNLE
QVRAGMAWVYRKYAYDPGYFSAEDEARAVRRGLWSQPNPVPPWEFRHKSRGPSFWDDFN
PTRKPTQTPRPLSGVAASACGSKRTCQEMTTCDEARYYLTRCGVKSLDRDGDGVPCEGL
CGG"
CDS 16016..16735
/ID="CZTTGXGG_CDS_0035"
/transl_table=11
/phrog="1599"
/top_hit="No_MMseqs_PHROG_hit"
/locus_tag="CZTTGXGG_CDS_0035"
/function="unknown function"
/product="hypothetical protein"
/source="PHANOTATE_1.5.1"
/score="-326.7791717676275"
/phase="0"
/translation="MCGRYTLTTPADKYKAQLKYERARAFLEKLFARYNIAPTQDVAAV
RTGTEGVRELVALHWGLIPSWSKAPKTEYSTINARAETVAEKPAFRSAFRYRRCLILAD
GYYEWQARPGSKLKQPWYIRRADGEPFAFAGLWERWESHLGEEGEPVESCSIIVTDANA
LTRPIHDRMPVILDPATYDVWLDPECRDKTGLLSLLRPFPPDEMTAWKVSTHVNSPRHD
DPTCVEPAEPSALDSVD"
CDS 16744..16845
/ID="CZTTGXGG_CDS_0036"
/transl_table=11
/phrog="No_PHROGs_HMM"
/top_hit="No_MMseqs_PHROG_hit"
/locus_tag="CZTTGXGG_CDS_0036"
/function="unknown function"
/product="hypothetical protein"
/source="PHANOTATE_1.5.1"
/score="-1.090828625011296"
/phase="0"
/translation="MPATPEGRGAIPAHSGRCPCPPAERIGPRVRAG"
CDS 16842..16994
/ID="CZTTGXGG_CDS_0037"

/transl_table=11
/phrog="No_PHROGs_HMM"
/top_hit="No_MMseqs_PHROG_hit"
/locus_tag="CZTTGXGG_CDS_0037"
/function="unknown function"
/product="hypothetical protein"
/source="PHANOTATE_1.5.1"
/score="-0.2207346282467836"
/phase="0"
/translation="VSLEGGAGFLPEQSDRPARLRPAGLPARRHCSLWNLRTDPYVCVC
EYVKA"
CDS 17140..17262
/ID="CZTTGXGG_CDS_0038"
/transl_table=11
/phrog="No_PHROGs_HMM"
/top_hit="No_MMseqs_PHROG_hit"
/locus_tag="CZTTGXGG_CDS_0038"
/function="unknown function"
/product="hypothetical protein"
/source="PHANOTATE_1.5.1"
/score="-1.580891355131832"
/phase="0"
/translation="MRSSSSEEALSGPPFNCSILSSGSWVIAGCVVYRSAHFAD"
CDS 17310..17588
/ID="CZTTGXGG_CDS_0039"
/transl_table=11
/phrog="No_PHROGs_HMM"
/top_hit="No_MMseqs_PHROG_hit"
/locus_tag="CZTTGXGG_CDS_0039"
/function="unknown function"
/product="hypothetical protein"
/source="PHANOTATE_1.5.1"
/score="-15.486370346626313"
/phase="0"
/translation="MFQYIVRVRDKTYVWGSNDLEVALSLPIVLVVNNDAFGDAVEIAS
ESPQGAPAVLGILQPGQCWTLVLTGLRGVTATCETDTTLACAILSPS"
CDS 17605..18786
/ID="CZTTGXGG_CDS_0040"
/transl_table=11
/phrog="No_PHROGs_HMM"
/top_hit="No_MMseqs_PHROG_hit"
/locus_tag="CZTTGXGG_CDS_0040"
/function="unknown function"
/product="hypothetical protein"
/source="PHANOTATE_1.5.1"
/score="-423256.6876423799"
/phase="0"
/translation="MATGRSVNLRVRPYQSVDLSFPVDGVISKQSDTILLGRPVLGIDV
EHLYAMLGEMRNVGLQVHGGRLKWDSRQISEYLSNVAGVAAGQNGTTLLGKLRNATEAA
DLDRALMMRQNAYLTSYSPEVLAEVRRVYCGEPRDDSLARYRLLQKVEEDITTMHNGLA
GAYNHRGWSGKVIEYTRSENTNKATQYSGSGHIQFEGLTDTNSWGYEFRYPSAENDLRY
HQARAGIRQEILNAQRMAEMCRHGDVTFPNEIGAIDLSIRKLQAAYIDTFLFAPFNGIV
TGVFRGPGDYVRAGEPVCRVENYLLAYLVGTVKYRGMLGIGTKVDVSTTLFEAAGTTPV

KVSGEIVAVRGHDAVSEQWDVLIFCENYTATGDPVLPLNYNFDFESTTVGVLP"
CDS 18935..20623
/ID="CZTTGXGG_CDS_0041"
/transl_table=11
/phrog="No_PHROGs_HMM"
/top_hit="No_MMseqs_PHROG_hit"
/locus_tag="CZTTGXGG_CDS_0041"
/function="unknown function"
/product="hypothetical protein"
/source="PHANOTATE_1.5.1"
/score="-32966.6667577313"
/phase="0"
/translation="MVGQAFADAGPVGQYLGMLYGSPGTGIPRVEWGTDYASQVDYGDY
LDFTQSQVFADSPDYSASVRVVDTSDSAPSRPAEPRTLEVLDRGDSGAVPAAEPIEVVH
VFGTPPDRPSKNDWFESFLSTPRNNTFSGGEIKLWVDNPLPRPPRPPRPQRSAGRKGSP
VETTSPLPELPPPERDPVDSGPYFNVEGTPLTVPGSDQATNPWMSDAAVPERFLPWGSW
DRPENKPSPRWERGDTGRIEAVPTVPVERSFWSRGGTGLAAGAVTAGLGVAALMWWNPV
GWVALGTALAIAGGVAATTASAVELTASYSGATSLEQDAEMNRAISATLGYSSPGGVIG
SVLGTVVADDPQEGFAQGALWGGLTEGVTSLPGALRAVPGLWRAALPWTKSLLLTPFWF
FMSAGGGGGRARSLARVFAAQGRIASRVRSVEYLGTTPLLERDADWARFQVFATRTRNE
SVFRITYANGQQRIVLADRAQPTGRAILEAKYGDMGQMWNPTREAHIIGQANNYLDIAT
VTGGRVGYLVSTERGAYRLTQRFGLEFPAEMASGQLWIDWVPWRR"
CDS 20799..20888
/ID="CZTTGXGG_CDS_0042"
/transl_table=11
/phrog="No_PHROGs_HMM"
/top_hit="No_MMseqs_PHROG_hit"
/locus_tag="CZTTGXGG_CDS_0042"
/function="unknown function"
/product="hypothetical protein"
/source="PHANOTATE_1.5.1"
/score="-0.0609157697890264"
/phase="0"
/translation="LQRAQSYLWASLQSIPTRCRRRPCRLSLC"
CDS 20916..21290
/ID="CZTTGXGG_CDS_0043"
/transl_table=11
/phrog="No_PHROGs_HMM"
/top_hit="No_MMseqs_PHROG_hit"
/locus_tag="CZTTGXGG_CDS_0043"
/function="unknown function"
/product="hypothetical protein"
/source="PHANOTATE_1.5.1"
/score="-0.4912934913260574"
/phase="0"
/translation="VLVPRRSGLRKGRGLSCPPSLTGRPGCAQRARRALSLPKPDRLPC
PPRNLSFLIPPSPHGGMSAYPCATNGAWVPKTKKPKREKKPRLLPGLRAQGVRFASRGR
RFCAAHDDPACLDSIGGSSG"
CDS 21244..21969
/ID="CZTTGXGG_CDS_0044"
/transl_table=11
/phrog="811"
/top_hit="No_MMseqs_PHROG_hit"

/locus_tag="CZTTGXGG_CDS_0044"
/function="DNA"
/function=" RNA and nucleotide metabolism"
/product="UmuD-like protein"
/source="PHANOTATE_1.5.1"
/score="-114.8222216048277"
/phase="0"
/translation="MTILRAWIQSVALRGELTAIDPRSLPNNGHIDYRTEIISSDSVFP
MTSIPRGGLRPGAGRPRGTGAFGEPTTPVRIPESRLAEVRTLIARPRRPAAPELEWTGE
PDASAPGLSLPLFASRIQAGFPSPADDYVEGKLDLNQHLIRNPPATFFLRVQGESMLGA
GIHPGDLLVVDRSIEPKHGAVVIAVVDGELTVKRLWLATPERVELRAENPAYAPIVIGE
FQQFELWGVVTSVIHPLLP"
CDS 21966..23249
/ID="CZTTGXGG_CDS_0045"
/transl_table=11
/phrog="1268"
/top_hit="No_MMseqs_PHROG_hit"
/locus_tag="CZTTGXGG_CDS_0045"
/function="DNA"
/function=" RNA and nucleotide metabolism"
/product="DNA polymerase"
/source="PHANOTATE_1.5.1"
/score="-6735.595948943284"
/phase="0"
/translation="MSVFALVDCNNFYVSCERVFDPKLEGRPVVVLSNNDGCVVARSPE
VKALGVPMGAPWFQYQTLARKQRIVALSSNYALYADLSDRVMRILADLSPAHEVYSIDE
CFCDWRGLAGVDLIEYGQRTRQRIRQWVGLPVCVGLGPTKTLAKLANHVAKKQPQHDSV
FDIARLTETERTELLDRIAVGEVWGVGRRIEERLREQGITTVRALRDADTRMIRARYGV
VLERTVRELRGSSCLPLEAVTPPRKQILCSRSFGQRVEDLDSLRQAVTAYTARAAEKLR
RQGSSAGAIQVFIETNRFNDEPRYAAQRTIPLATPTADTHRLLHAAHHALVDIYRPGYR
YQKAGVILLDLDPAENAQRDCFASLAADAIDPARAKLMSAIDAINRNHGRGALRWAAEG
YTRPWAMRTDRRTQGYTTRWNELATARA"
CDS 23316..23609
/ID="CZTTGXGG_CDS_0046"
/transl_table=11
/phrog="No_PHROGs_HMM"
/top_hit="No_MMseqs_PHROG_hit"
/locus_tag="CZTTGXGG_CDS_0046"
/function="unknown function"
/product="hypothetical protein"
/source="PHANOTATE_1.5.1"
/score="-35.98869451029493"
/phase="0"
/translation="MDSFCTWDGETLVLNILGQPSAKQDAIGKAKGNQLKVSVTAAPVG
GKATDHMVRFLAKEFGVATQDIEVVFGRYNVNKQLRIKSPKKLPSVIDKQLP"
CDS 23656..23748
/ID="CZTTGXGG_CDS_0047"
/transl_table=11
/phrog="No_PHROGs_HMM"
/top_hit="No_MMseqs_PHROG_hit"
/locus_tag="CZTTGXGG_CDS_0047"
/function="unknown function"
/product="hypothetical protein"

/source="PHANOTATE_1.5.1"
/score="-2.2827411815525624"
/phase="0"
/translation="MSALFQPLSKTENRSVRLAGLGQIPLIWAR"
CDS complement(23731..24213)
/ID="CZTTGXGG_CDS_0048"
/transl_table=11
/phrog="14161"
/top_hit="No_MMseqs_PHROG_hit"
/locus_tag="CZTTGXGG_CDS_0048"
/function="other"
/product="recombinase"
/source="PHANOTATE_1.5.1"
/score="-69.83481919920148"
/phase="0"
/translation="MPRKSQGRITGRPVTTTIPSPADGVRLETFLPWTLVKRGVKREVI
TPLGTPAAFREEARQEVEKRRAEQDTPLIRALGLAHFWQRLLDEGKFESLSEIANAEGL
DLSQASRVGRLVQLAPEIVEACLAGKGGGRVSESRCRSPFSLDWQAQRVEILARPD"
CDS complement(24224..24580)
/ID="CZTTGXGG_CDS_0049"
/transl_table=11
/phrog="14161"
/top_hit="No_MMseqs_PHROG_hit"
/locus_tag="CZTTGXGG_CDS_0049"
/function="other"
/product="recombinase"
/source="PHANOTATE_1.5.1"
/score="-24.18944172929174"
/phase="0"
/translation="MDNPETLVPFCLRRRQWHLVNTQRTAHDPGLLISIGRAMYWQHLL
DTGVVHSCAELAEREGLTPPSVSHVLRLALLAPEQVERCLAGHQPRTLTQRWLKRHRLP
LDWEAQRETIDRFE"
CDS complement(24580..25977)
/ID="CZTTGXGG_CDS_0050"
/transl_table=11
/phrog="95"
/top_hit="No_MMseqs_PHROG_hit"
/locus_tag="CZTTGXGG_CDS_0050"
/function="integration and excision"
/product="integrase"
/source="PHANOTATE_1.5.1"
/score="-92941.96838713494"
/phase="0"
/translation="MRASNRMEVQTGAPSKPRQRCAVYCRVSSDERLDQEFNSIDAQKE
AGHAYIASQRIEGWIPVADDYDDPGYSGGNTERPGLKRLLSDIEAGRIDIVVVYKIDRL
TRSLADFSRMVEVFERQGVSFVSVTQQFNTTTSMGRLMLNVLLSFAQFEREVTGERIRD
KIAAAKRKGLWMGGVPPLGYDVRDRQLVVNENEAAIVRRIFKDMLSVGSTTRIAAALNA
EGITTKAWTTQDGRHRPGARIDKKHLHHLLRNRIYLGEISHKGSWHPGAHPAIIEATLW
DAVHAVLARDARSRATETRQRERTDALLRGLLYDTEGEKMYPTYVRKNGRQYRYYFSKA
EARFGAGHKTSVRLPAEEIEAATLAQIRTVLASPEAIAAIWLAVQAQTADLDEAQVVVA
VGQLGSVWEQLFPAERHRIVQLMIERVELAEGGLRIRWRALGWKALLGEFTPKTIGAEL
IEMEAVA"
CDS complement(25974..26426)

/ID="CZTTGXGG_CDS_0051"
/transl_table=11
/phrog="11806"
/top_hit="No_MMseqs_PHROG_hit"
/locus_tag="CZTTGXGG_CDS_0051"
/function="unknown function"
/product="hypothetical protein"
/source="PHANOTATE_1.5.1"
/score="-24.11537612131596"
/phase="0"
/translation="MNDLKLSVAAQVASLSNLPIKDLWALWDKYFPRRPTHPNRNYLES
RIAYKIQEAAYGGLAPETRRRLELIGQRHSKIKSRRVSPAIHLPPGTVLVREWGEQDHK
VTVTAEGQFDYAGQSFKSLTAVARHITGTAWSGPLFFGLRQAGEGT"
CDS complement(26426..26635)
/ID="CZTTGXGG_CDS_0052"
/transl_table=11
/phrog="No_PHROGs_HMM"
/top_hit="No_MMseqs_PHROG_hit"
/locus_tag="CZTTGXGG_CDS_0052"
/function="unknown function"
/product="hypothetical protein"
/source="PHANOTATE_1.5.1"
/score="-5.192669418812251"
/phase="0"
/translation="MIVELPMTMPSFPSPERLSPAQRAHEITTILAAAIVRTLQKESEK
KTGLDLGLLAGKRVHTTPSQPERV"
CDS complement(26707..26808)
/ID="CZTTGXGG_CDS_0053"
/transl_table=11
/phrog="No_PHROGs_HMM"
/top_hit="No_MMseqs_PHROG_hit"
/locus_tag="CZTTGXGG_CDS_0053"
/function="unknown function"
/product="hypothetical protein"
/source="PHANOTATE_1.5.1"
/score="-0.175108340939541"
/phase="0"
/translation="VSKPGLPGRRKHPPQRFPSRKIRLVFAMACNDP"
CDS complement(26835..27746)
/ID="CZTTGXGG_CDS_0054"
/transl_table=11
/phrog="1914"
/top_hit="No_MMseqs_PHROG_hit"
/locus_tag="CZTTGXGG_CDS_0054"
/function="unknown function"
/product="hypothetical protein"
/source="PHANOTATE_1.5.1"
/score="-9405.101659705226"
/phase="0"
/translation="MTERKWPTAEANRLNVMLGQVLGRERFPVDVEALALEYSKQCFPH
APITQIKGADLPGFEGMLAAHPSKTQWKIVYNSAVRSRGRIRFTLAHEFGHYLLHRDRQ
ELFSCSQQDMEEWDAEERQLETEADTFASYLLMPLDDFRQQIGKERISFELLAHCAERY
GVSLTAAALKWIEIAENRAVLVAVRDDHLLWARSNQAAFKSGAVFATRKRTYAVPLESL

AHGHNGGIPIQEGSLSAKTWFPKEPRDMPLTELTFVNEHYDYTLALLLMPKAEPRWQRE
EEEDDDPAERMDSAIRQGRFRR"
CDS complement(27743..28066)
/ID="CZTTGXGG_CDS_0055"
/transl_table=11
/phrog="2439"
/top_hit="No_MMseqs_PHROG_hit"
/locus_tag="CZTTGXGG_CDS_0055"
/function="transcription regulation"
/product="transcriptional regulator"
/source="PHANOTATE_1.5.1"
/score="-15.556178307228008"
/phase="0"
/translation="VPSPLGEKIRGLRKQKKLSLDQLADLTESSKSYLWELENKEAPNP
SAEKIARIAAVLEVTTEFLMNDQEMTPDAAVADEAFFRKYKKMPEETKKKLRQLIDVWD
DDP"
CDS complement(28088..28177)
/ID="CZTTGXGG_CDS_0056"
/transl_table=11
/phrog="No_PHROGs_HMM"
/top_hit="No_MMseqs_PHROG_hit"
/locus_tag="CZTTGXGG_CDS_0056"
/function="unknown function"
/product="hypothetical protein"
/source="PHANOTATE_1.5.1"
/score="-0.0581224047180523"
/phase="0"
/translation="LRAAECSVKPVRIRSARCYIPKVACGDMN"
CDS complement(28214..28540)
/ID="CZTTGXGG_CDS_0057"
/transl_table=11
/phrog="No_PHROGs_HMM"
/top_hit="No_MMseqs_PHROG_hit"
/locus_tag="CZTTGXGG_CDS_0057"
/function="unknown function"
/product="hypothetical protein"
/source="PHANOTATE_1.5.1"
/score="-14.794396643998036"
/phase="0"
/translation="MPCVEQRVDAFALGQPVNPAHFRVDRRALFAVLETLEAGDQGICQ
RGFFRLPIEIVTDLDRPLCQIVAPGFPGDLPDEVTKIECSQDDFSCTEEKTRNERPEQR
TLTQ"
CDS 28478..29509
/ID="CZTTGXGG_CDS_0058"
/transl_table=11
/phrog="37634"
/top_hit="No_MMseqs_PHROG_hit"
/locus_tag="CZTTGXGG_CDS_0058"
/function="unknown function"
/product="hypothetical protein"
/source="PHANOTATE_1.5.1"
/score="-25866.39332794964"
/phase="0"

/translation="MRRVHRLAQRKGIYALLNAGHGQSEIQEGFEQLHNHAERALWTWV
HWPAVFQIGERLLQFDLSAGTRAWKRQAIKVSEPVSKEEENIRALETALSALMSRRKGP
RRACRIDVCERCLDGGVQVNIYIEDDPNDLVEFVEENMKRRTTRPAGNLALVYYPISGI
VDSVGRGGARIHVTLVTLFAKHLLNRDVKPEAVKQPMFHLNRLRYGLAFVEGDAIDLAT
HGVDRIRLRQVRVRATIPPCCDFWVETPADPNEACAFTASSTHLQERDFFRGPFNLVEV
VISVYFVPSEPGKIGHVLNIVLKQSGVSNLRDLSESDAQLADALLRAWQVTEPSEFEAK
LAA"
CDS 29517..30476
/ID="CZTTGXGG_CDS_0059"
/transl_table=11
/phrog="No_PHROGs_HMM"
/top_hit="No_MMseqs_PHROG_hit"
/locus_tag="CZTTGXGG_CDS_0059"
/function="unknown function"
/product="hypothetical protein"
/source="PHANOTATE_1.5.1"
/score="-3673.80646707674"
/phase="0"
/translation="MPACNDIAFGLLCELIEAPHADIHPDTVRCSGRREAYEHLHELQA
LQVGTGLAGSVLCPWCGDDELCSLSFSEEGYRGYCSDCGWLNLATHQVKPLRVEYQRIV
RWIASALGLQGRFRLEERVPARLWRLGDIEHRRKRRTVFFGRRLNESSDAQTIDAGIRA
VAAPGTEILITTTPQDSIAPPLANGRLVPLRAVSHLRKAGFVVENLESYLDAPLIADED
VAETSLRLLHSGRLALIDGKQIKVSPQIYRFLSILIDADGKPVHKRVLADALEMDVDAC
KGSEIFKRHKAVYRTFIEHDTEGRYWIKPEFLSRRGGE"
CDS 30542..30763
/ID="CZTTGXGG_CDS_0060"
/transl_table=11
/phrog="3220"
/top_hit="No_MMseqs_PHROG_hit"
/locus_tag="CZTTGXGG_CDS_0060"
/function="unknown function"
/product="hypothetical protein"
/source="PHANOTATE_1.5.1"
/score="-7.045804768146209"
/phase="0"
/translation="MSKNQWVVKNGDGWGVRGEGNSRLTSKHDTQHEAIERARDIARNQ
SSEVIIQGEDGKIRERNSFGNDPFPPPG"
CDS 30845..31162
/ID="CZTTGXGG_CDS_0061"
/transl_table=11
/phrog="66"
/top_hit="No_MMseqs_PHROG_hit"
/locus_tag="CZTTGXGG_CDS_0061"
/function="integration and excision"
/product="excisionase and transcriptional regulator"
/source="PHANOTATE_1.5.1"
/score="-0.3387746031416692"
/phase="0"
/translation="LQVLPTPAARFAPRFVHPLTPRICSRFRNNPQERLVNVKHLNQRQ
LADRWGVSEATLERWRSEGIGPVYLKLHGRVLYRQEDIEAYETRHLRRSPGQCVQAGGA
A"
CDS 31159..31638
/ID="CZTTGXGG_CDS_0062"

/transl_table=11
/phrog="2480"
/top_hit="No_MMseqs_PHROG_hit"
/locus_tag="CZTTGXGG_CDS_0062"
/function="unknown function"
/product="hypothetical protein"
/source="PHANOTATE_1.5.1"
/score="-29.34578176094415"
/phase="0"
/translation="MTSMNPDILLATPAGQLAELPAESLCRFKLDADAQLAAAKTLNEH
LDRALEIRYAERARQLRLATGKDTGIVHFDDGPVRVTADLPKKVEWDARQLAALVRRIA
DSGEDPAQYVEIGYRVSETKFNAWPAGLQQSFAPARTLRTGKPGFRLALLGEDAV"
CDS 31635..32498
/ID="CZTTGXGG_CDS_0063"
/transl_table=11
/phrog="124"
/top_hit="No_MMseqs_PHROG_hit"
/locus_tag="CZTTGXGG_CDS_0063"
/function="DNA"
/function=" RNA and nucleotide metabolism"
/product="Sak4-like ssDNA annealing protein"
/source="PHANOTATE_1.5.1"
/score="-545.010935971359"
/phase="0"
/translation="MTLPIISADQRLAERRGVKGVLVGKAGLGKTSQLWTLEAASTLFF
DLEAGDLAVEGWAGDTIRPRTWPECRDFAVFIGGPNPALRDDQPFSPAHFDAVCARFGD
PGALERYQTVFVDSITVAGRLCLQWCKGQPQAYSEKTGKPDSRGAYGLMGQEMIGWLTH
LQHTRNKNVWFVGILDEKLDDFNRRVFALQIDGAKTGLELPGIVDEVVTLAELPADDGS
RHRAFVCQTLNPWGYPAKDRSGRLDPIEPPHLGQLMQKIAGPARPLAERLDFSRPAPAT
IPTQET"
CDS 32501..33121
/ID="CZTTGXGG_CDS_0064"
/transl_table=11
/phrog="2300"
/top_hit="No_MMseqs_PHROG_hit"
/locus_tag="CZTTGXGG_CDS_0064"
/function="unknown function"
/product="hypothetical protein"
/source="PHANOTATE_1.5.1"
/score="-61.63890564558896"
/phase="0"
/translation="MSYFDFNSAEQASFELIPKDTLVRLRLTLKPGGFDDASQGWTGGW
ATRSPETGAVYLACEGVVLDGPYARRKLWWNIGLHSPKGPTWQAMGRSFIRSLLNSARR
IHPADIGPQAQNARRIAGFAELDGLEFAGRIDIEKDGRGNDRNTIRAVIEPDHKDYAQL
MGQHFAPPVPTSPSGAIPAAAAYAASTPATSPGVPGGKPAWAQ"
CDS 33136..34821
/ID="CZTTGXGG_CDS_0065"
/transl_table=11
/phrog="16"
/top_hit="No_MMseqs_PHROG_hit"
/locus_tag="CZTTGXGG_CDS_0065"
/function="DNA"
/function=" RNA and nucleotide metabolism"

/product="DNA helicase"
/source="PHANOTATE_1.5.1"
/score="-1116290.520054204"
/phase="0"
/translation="MMLRPRQTLLVERSLAALHQHGNTLAIGPTGSGKTIMLSAVAGSV
LEEPDAKACILAHRDELTAQNREKFGRVNPGLTTSVFDAQEKSWAGAATFAMVQTLARG
RHLEQMPTLDLLVIDEAHHAASPSYRAVIDRVRSRNPKALLCGLTATPNRGDGKGLREV
FSNVADQITLGEMIASGHLVPPRTFVIDIGVQAALQQVRRTAIDFDMDEVASIFDKQLV
TDAVIQHWRDKASGRKSIVFCSTVAHARHVCEAFIAAGIHAVLIHGDLSDAERKARLAN
YESGPAQVVVNVAVLTEGYDYPPTGCVVLLRPSSHQSTFIQMVGRGLRTVDPEVFPGAI
KTDCIVLDFGTASLMHGTLEQTVDLDGHLGQGEAPTKDCPECGAIVPAACLECPLCGHL
WERQPKDRGVLSDFVMSEIDLLKRSHFRWCDLFGSDDALMAAGFSAWGGVFFLTGRWYA
VGGGKGLPTHLLGVGERTVCMAKADDWLNEHESVDTAHKTRRWLNEPPTEKQLRYLPPQ
WQSDFGLSRYQASALLSFQFNKRAIQSLVLGADAATHDTQRRAA"
CDS 34886..35251
/ID="CZTTGXGG_CDS_0066"
/transl_table=11
/phrog="21342"
/top_hit="No_MMseqs_PHROG_hit"
/locus_tag="CZTTGXGG_CDS_0066"
/function="unknown function"
/product="hypothetical protein"
/source="PHANOTATE_1.5.1"
/score="-14.077496434363704"
/phase="0"
/translation="MHAMICAICGREGRGFCWASSHDAPHDSGDKRLFKRFCSRRCQDI
HLQRLKRSDGVVIDPTHNEKAAMAAVLPRLGDYVAAVGMDRPLSAYSRAEILQLVDVVL
TAYFDHLREHAPDDVPF"
CDS 35235..35999
/ID="CZTTGXGG_CDS_0067"
/transl_table=11
/phrog="1990"
/top_hit="No_MMseqs_PHROG_hit"
/locus_tag="CZTTGXGG_CDS_0067"
/function="DNA"
/function=" RNA and nucleotide metabolism"
/product="Cas4-domain exonuclease"
/source="PHANOTATE_1.5.1"
/score="-145.33179409191808"
/phase="0"
/translation="MTCRSEGATMLDYNPGALFNERLTALIDAGLQHRQAGEPRRTYLG
ASRLGVACERALQYEYAQAPVDPGREFSGRMLRIFERGHRMEEAVVGWLRAAGFGLRTH
QADGEQFGFSALDGRLQGHVDGVIVGGPEGFDTPALWENKCLGAKSWRELVKHRLAKAK
PVYAAQIAVYQAYLALHEQPALFTAVNADSMEIYAERVPFDGGLAQRMSDRAVRVITAT
EAGELLPRGFADPAHIECRQCAWGLRCRGGGA"
CDS 35996..36355
/ID="CZTTGXGG_CDS_0068"
/transl_table=11
/phrog="No_PHROGs_HMM"
/top_hit="No_MMseqs_PHROG_hit"
/locus_tag="CZTTGXGG_CDS_0068"
/function="unknown function"
/product="hypothetical protein"

/source="PHANOTATE_1.5.1"
/score="-19.671051558021748"
/phase="0"
/translation="MKTRQPSDDKRAQRQPPRPLVRLTVIDKLLRRHIVFACPEAHLAV
AVITLAIGDCIDPDDALRAEARYFLAGPALEFWCDAVGLEPAFVRAIAHKGGYLPSETA
HGVGVKLTPKELGLA"
CDS 36490..36615
/ID="CZTTGXGG_CDS_0069"
/transl_table=11
/phrog="No_PHROGs_HMM"
/top_hit="No_MMseqs_PHROG_hit"
/locus_tag="CZTTGXGG_CDS_0069"
/function="unknown function"
/product="hypothetical protein"
/source="PHANOTATE_1.5.1"
/score="-0.1216668946353027"
/phase="0"
/translation="VPSRSATSRAIRATAWKWRFRAKRPGCGMTMRPARAGISSP"
CDS 36663..38501
/ID="CZTTGXGG_CDS_0070"
/transl_table=11
/phrog="13004"
/top_hit="No_MMseqs_PHROG_hit"
/locus_tag="CZTTGXGG_CDS_0070"
/function="unknown function"
/product="hypothetical protein"
/source="PHANOTATE_1.5.1"
/score="-1096879.0706984731"
/phase="0"
/translation="MALAGQLLGTARVEPTPLREKAPVDRLGPATAKWDYVAADGTLIA
CVYRYDPPTGKEFRPWDVRARLWRAPDPRPLYNLPAIAQAKEVVLVEGERCADALVHQG
VAATTAMNGAKAPIDKTDWSPLRGKAVLIWPDRDAPGWDYAENAARACVTAGAASVAIL
VPPTDRPDKWDAADAVSEGFDCVAFITHGERRVIGVAPPVLPTHTLGALLDDDSPVPPD
LIAPRVLTPGGLLVFGGAPKVGKSDFLLAWLMHMAAGASFLGMTPPRPLRVFYLQAEVQ
YHYLRERVKGIPLPSHRIAEARVNFVATPQLRLVLDEAGLAQVIPAIVQAFGNTPPDLI
AIDPIRNVFDGGEAGGENDNGAMLFFLSQRVEQLRTAVNPDAGLVLVHHTKKLGKKPFE
EDPFQALAGAGSLRGYYSTGMLLFRPDETQTTRQLIFELRNGAALPVKSVDKFNGTWRE
VVNTGRRVLQDYAERLDAERRRKHDVILQILFDEAADGRCYSATQFAEAFEGRAGLGAE
RTIRERLSVLATQGYIKFFRNAEDYGLPPLHRSKFGYQCVEGMVLRREDPPDPDTGEIP
MWPVSVYPSHYKCPQTGALLPVEDATIWLYHDEDHP"
CDS 38498..39277
/ID="CZTTGXGG_CDS_0071"
/transl_table=11
/phrog="159"
/top_hit="No_MMseqs_PHROG_hit"
/locus_tag="CZTTGXGG_CDS_0071"
/function="DNA"
/function=" RNA and nucleotide metabolism"
/product="RuvC-like Holliday junction resolvase"
/source="PHANOTATE_1.5.1"
/score="-258.00157908357414"
/phase="0"
/translation="MTTTLIPDGGRSAICPLKSAIWIQTANVRHLGIHILQSLTVQMAE

FPDGSAANSPSGATDCLYETIYGIPDGGDSLPLRGRGALRALYPSRRGLSGLGDNDEGD
RTTLLALDLGTHTGWALHQRDGTVISGSETFKPQRFEGGGMRFLRFKRWLTEIKQAAGD
LDAVYFEEVRRHAGVDAAHAYGGFLAHLTAWCEHHQIPYQGVPVGMIKKHATGQGNANK
AAMIAAMRSLGFDPVDDNEADALALLHWARVTQGGGV"
CDS 39274..39483
/ID="CZTTGXGG_CDS_0072"
/transl_table=11
/phrog="19772"
/top_hit="No_MMseqs_PHROG_hit"
/locus_tag="CZTTGXGG_CDS_0072"
/function="unknown function"
/product="hypothetical protein"
/source="PHANOTATE_1.5.1"
/score="-5.511445085842217"
/phase="0"
/translation="MSGPNPNPRCPLGKLQPQVTDLEAVKREGWREQHILVVHLTDERL
DFVERELIRQIGERLYGRQESRRG"
CDS 39476..39847
/ID="CZTTGXGG_CDS_0073"
/transl_table=11
/phrog="6836"
/top_hit="No_MMseqs_PHROG_hit"
/locus_tag="CZTTGXGG_CDS_0073"
/function="unknown function"
/product="hypothetical protein"
/source="PHANOTATE_1.5.1"
/score="-3.1549889628046346"
/phase="0"
/translation="VAEWTIERVAARFEEAAWTAQDLPPVKVQGYFNCWPAIARQAWEG
YADETRECRVQPSPDAVDRMLETMRWVLWLEEEQRHLVWMRAERRGWKIIARRFGCCTR
TAQRRWQQALRQVVDRLNH"
CDS 39895..40260
/ID="CZTTGXGG_CDS_0074"
/transl_table=11
/phrog="No_PHROGs_HMM"
/top_hit="No_MMseqs_PHROG_hit"
/locus_tag="CZTTGXGG_CDS_0074"
/function="unknown function"
/product="hypothetical protein"
/source="PHANOTATE_1.5.1"
/score="-120.1127126899948"
/phase="0"
/translation="MERDPGIETLLDLQDQIIDQGSGYWVKIEAYQVEPTPDVPHGIRY
SLTLHEPYGKRILGYDNAHAVKPPKKFKYAGRRLTFDHMHRHARDPGVPYEFKNAHQLL
ADFFSEVDQVLLEVKKR"
CDS complement(40197..41135)
/ID="CZTTGXGG_CDS_0075"
/transl_table=11
/phrog="No_PHROGs_HMM"
/top_hit="No_MMseqs_PHROG_hit"
/locus_tag="CZTTGXGG_CDS_0075"
/function="unknown function"
/product="hypothetical protein"

/source="PHANOTATE_1.5.1"
/score="-204.4139871300788"
/phase="0"
/translation="MAAPGLQGGVCTQVCTGGVGRSRVSASTGAASRVVRSGWGCKPQT
LQTLVCSLTLAKSRARSPRSPQEPGRTRNRSRRRGVSPLRSHLLYFERYSTPVGPRMRQ
PKSRLFSQPPAAFPDDLQTLSTSVKFPHAVEAIAGADGRSGSPVHRRSSKSLRSIRLSH
FKEIRNSASVVFELGSDDPVLRSLDDGADMILFTLHLYEAVMGHRLQRARKVAGLPARR
CRERRDRFRFGFSDHLEQRPVFIAQDLCKRSHRSEPYLGIPCFGLELAPRDGQQTLANV
LLRHDAYHNGFHRFFTSSRTWSTSEKKSASN"
CDS 41142..41333
/ID="CZTTGXGG_CDS_0076"
/transl_table=11
/phrog="No_PHROGs_HMM"
/top_hit="No_MMseqs_PHROG_hit"
/locus_tag="CZTTGXGG_CDS_0076"
/function="unknown function"
/product="hypothetical protein"
/source="PHANOTATE_1.5.1"
/score="-0.124670343380105"
/phase="0"
/translation="LHPWAYLISQDPPRFAVGGFFVAALPRVYGSLQVPIGPCAGPYFF
GIATNPSCCRSNTVRWKS"
CDS 41297..42691
/ID="CZTTGXGG_CDS_0077"
/transl_table=11
/phrog="498"
/top_hit="No_MMseqs_PHROG_hit"
/locus_tag="CZTTGXGG_CDS_0077"
/function="other"
/product="DNA methyltransferase"
/source="PHANOTATE_1.5.1"
/score="-42419.919009745405"
/phase="0"
/translation="MLQVEYRPLEKLIPYARNPRTHSVDQVAKIAASIVEFGWTNPILV
DGAQGIIAGHGRLAAARSLGLAEVPVIELGHLSPAQKRAYVLADNRLALDAGWDEELLA
LELAELSGAGFDLALTGFNDDELEALLSIDTEDSDDAEDREPETADDVPEPPAAPLSRP
GDVWQLGRHRLICGDASDPDVVAALMGGELARLCFTSPPYDNQRDYTSGGIGDWDGLMR
GVFAQLPLTDDAQVLVNLGLIHRDNEVIPYWDPWLGWMRTQGWRRFGWYVWDQGPGMPG
DWAGRLAPSFEFVFHFNRQSRKPNKTVACKFAGQETHLRQDGSSTALRGKDGEVGGWTH
AGQPTQDRRIPDSVIRIMRHKGKIGRDIDHPAVFPVALPEFVLTAYSDPDDPVYEPFGG
SGTSLLAAERTGRRGYAVEIAPGYVDVAILRFRQSFPAIPVTLLDTGETWEVVASQRQA
IQTTAG"
CDS 42688..43038
/ID="CZTTGXGG_CDS_0078"
/transl_table=11
/phrog="No_PHROGs_HMM"
/top_hit="No_MMseqs_PHROG_hit"
/locus_tag="CZTTGXGG_CDS_0078"
/function="unknown function"
/product="hypothetical protein"
/source="PHANOTATE_1.5.1"
/score="-2.067697846710333"
/phase="0"

/translation="MIIACRSSFGSRYSANCSTLRPMALASLVSVALRGVLSPRSRRAN
AGCCMQARCATSSCVSPKYSRQARISGISCRTWASTTAWGMASRTLRRSSRGRTRSKSA
TGTNTKAGCPSW"
CDS 43039..43317
/ID="CZTTGXGG_CDS_0079"
/transl_table=11
/phrog="No_PHROGs_HMM"
/top_hit="No_MMseqs_PHROG_hit"
/locus_tag="CZTTGXGG_CDS_0079"
/function="unknown function"
/product="hypothetical protein"
/source="PHANOTATE_1.5.1"
/score="-7.266082074820301"
/phase="0"
/translation="MVWYSVGAFIAFLDLLDADHLDGVQDIEQNPVIAHAQPVTTGMID
QRLDATHCREPLQRCRCSQYPLLSRLVAQLLKLLQSSRLPNDRFHGA"
CDS 43485..44951
/ID="CZTTGXGG_CDS_0080"
/transl_table=11
/phrog="498"
/top_hit="No_MMseqs_PHROG_hit"
/locus_tag="CZTTGXGG_CDS_0080"
/function="other"
/product="DNA methyltransferase"
/source="PHANOTATE_1.5.1"
/score="-84714.33640361091"
/phase="0"
/translation="MRGFHQAFQLIGRNHGDGPTGTAADDDHLTIIDGAIHQRFELLAG
PAVGDFERHGGKHFRQNAFNVQRMRHSWVADRVESWPVDKLLPYACNARTHSDAQIAQI
AASIAEFGFTAPILAGADGVIVAGHGRWAAARQLGLSQVPVIVLDHLSPTQRRALVIAD
NRIAENAGWDESLLKLELTALQDEDFNLGLTGFDADALLDLLADEASVTEGQTEDDVAP
DVPVQPVSRPGDVWILGSHRLLCGDATLAEHYDRLLAGESVDMVFTDPPYNVNYANSAK
DKLRGKHRAILNDNLGEGFSDFLLAALTPMLAHCKGAVYIAMSSSELDTLQSAFRTAGG
HWSTFIIWAKNTFTLGRADYQRQYEPILYGWAAGAQRHWCGDRDQGDVWQINKPVKNDL
HPTMKPVELVERALRNSSRPGDVILDPFGGSGTTLIAAEKSGRVARLIELDPKYVDVIV
RRWQDWTGKQATREADGLAFDALVSETAGG"
CDS complement(44958..45236)
/ID="CZTTGXGG_CDS_0081"
/transl_table=11
/phrog="1213"
/top_hit="No_MMseqs_PHROG_hit"
/locus_tag="CZTTGXGG_CDS_0081"
/function="moron"
/function=" auxiliary metabolic gene and host takeover"
/product="RelE-like toxin"
/source="PHANOTATE_1.5.1"
/score="-14.676258620832089"
/phase="0"
/translation="MRLQVTPTFDRAAKKLNRPQKLDLDEVVRAIAADPEIGVAKVGDL
AGVRVYKFRLSNALCLLAYRILAPDTLKLLTFGPHENFYRDIKRQDV"
CDS complement(45236..45559)
/ID="CZTTGXGG_CDS_0082"
/transl_table=11

```
                /phrog="16013"
                /top_hit="No_MMseqs_PHROG_hit"
                /locus_tag="CZTTGXGG_CDS_0082"
                /function="unknown function"
                /product="hypothetical protein"
                /source="PHANOTATE_1.5.1"
                /score="-14.711596590253082"
                /phase="0"
                /translation="MIELGRGTTCAPHPHRRDAEGSVRAPLGLHCDFAPESAMSVTVKL
                SEHLVEQARRYAHIEHRSVPKQIEHWSMIGKIAEENPDLPFTLIRDILVADEEEAIGEY
                RFG"
CDS             complement(45556..46065)
                /ID="CZTTGXGG_CDS_0083"
                /transl_table=11
                /phrog="10315"
                /top_hit="No_MMseqs_PHROG_hit"
                /locus_tag="CZTTGXGG_CDS_0083"
                /function="unknown function"
                /product="hypothetical protein"
                /source="PHANOTATE_1.5.1"
                /score="-95.93207279261667"
                /phase="0"
                /translation="MNTLSLTDTQRRILDHAIEHTAGRIDWFPDNIKGGARRKVLDGLL
                KRGLIALNGGDHCVTAEGFAAVGRTPPVPISPSAETEMPASNAEAAEANATGKRVRKSR
                EDSKQAEVIRMLHRPEGATIPQICAETGWQAHSVRGLFAGAFKKKLRLTLVSDKPEGGD
                RIYKIG"
CDS             complement(46077..46166)
                /ID="CZTTGXGG_CDS_0084"
                /transl_table=11
                /phrog="No_PHROGs_HMM"
                /top_hit="No_MMseqs_PHROG_hit"
                /locus_tag="CZTTGXGG_CDS_0084"
                /function="unknown function"
                /product="hypothetical protein"
                /source="PHANOTATE_1.5.1"
                /score="-0.0576106006793947"
                /phase="0"
                /translation="LNTSIARLADPVGRPLTEGRRRRCPKPKH"
CDS             complement(46163..46438)
                /ID="CZTTGXGG_CDS_0085"
                /transl_table=11
                /phrog="2555"
                /top_hit="No_MMseqs_PHROG_hit"
                /locus_tag="CZTTGXGG_CDS_0085"
                /function="unknown function"
                /product="hypothetical protein"
                /source="PHANOTATE_1.5.1"
                /score="-8.176843344164116"
                /phase="0"
                /translation="MAVDFAWLVFGTARSWRRHPRHHENYAMTPRDALLTRNAQQHLGI
                DTLETQNSDHLDFHDMAVWCLKAALEAAYQAGIEAGRRTKSAAANT"
CDS             46451..46978
                /ID="CZTTGXGG_CDS_0086"
```

/transl_table=11
/phrog="57"
/top_hit="No_MMseqs_PHROG_hit"
/locus_tag="CZTTGXGG_CDS_0086"
/function="head and packaging"
/product="terminase small subunit"
/source="PHANOTATE_1.5.1"
/score="-17.495288725382846"
/phase="0"
/translation="MGLSIRAYARHRGVTDTAVHKAIRTGRITPEADGSIDPDKADREW
ARNSDAPKAGTRQPAVRVAVPDASHDSGPSMPAGGTSLLQARTVNEVVKAQTNKVRLAK
LKGDLVDRAAAIAQVFTLARAERDAWLNWPARISAPLAAELGIDPHTLHVALETAVREH
LQELGELRPRID"
CDS 46979..49030
/ID="CZTTGXGG_CDS_0087"
/transl_table=11
/phrog="15"
/top_hit="No_MMseqs_PHROG_hit"
/locus_tag="CZTTGXGG_CDS_0087"
/function="head and packaging"
/product="terminase large subunit"
/source="PHANOTATE_1.5.1"
/score="-414580.0638321301"
/phase="0"
/translation="MPNDDDGADAIARAWRDGLTPDPLLTVSEWADRHRVLSSKASSEP
GRWRTARTPYLREIMDCLSPASPVERVVFMKGAQLGATEMGSNWIGFCVHHAPGPMMAV
WPTVEMAKRNSRQRIDPLIEESPALAALIAPARSRDSGNTMLAKEFRGGVLVMTGANSA
VGLRSMPVRYLFLDEVDGYPPDVEGEGDAIALAEARTRTFARRKIFVVSTPTISGASAI
EREYEASDQRRYFVPCPHCGQFQWLRFEQLRWERGRPETVGYVCEGCERPIPEHHKGDM
LAQGEWRALAPEHGAKTAGFHLSSLYSPLGWRSWRDIAAAWDSAVHPVSGGPSAIKTFK
NTELGETWVEDGETPDWQRLLERREDYTIGTVPLGGLLLAGGADVQKDRIEVSIWAFGR
GKSAWLVEHRVLMGDTGREPVWAELASMLEERWSHAVGALLPLARLALDTGYATQEAYA
FVRRLKDPRVMAVKGVPRGAALIGTPTAVDLTQGGRKLRRGIKVFAVAVGIAKLEFYNA
LRKTAEVGEDGTTLVYPPGYIHLPRMDAEYLQQLCAEQLVTRRDRHGFARREWQKLRER
NEALDCYVYARAAAAAAGLDRYEERHWRALEEQLGVGPPPDSEPPLQPIDPQRPPTPVG
VPFPARSIFAPGDPQPLAGLRPKKAYRWPIPQNTCKPWKPPWPGASVG"
CDS 49027..49167
/ID="CZTTGXGG_CDS_0088"
/transl_table=11
/phrog="2545"
/top_hit="No_MMseqs_PHROG_hit"
/locus_tag="CZTTGXGG_CDS_0088"
/function="connector"
/product="head-tail adaptor Ad1"
/source="PHANOTATE_1.5.1"
/score="-0.2604526349998108"
/phase="0"
/translation="VTFQDRSVEYRSVEELKAAIREVKRGLAAQVGPVARQLRITTRKA
T"
CDS 49167..50657
/ID="CZTTGXGG_CDS_0089"
/transl_table=11
/phrog="21"

/top_hit="No_MMseqs_PHROG_hit"
/locus_tag="CZTTGXGG_CDS_0089"
/function="head and packaging"
/product="portal protein"
/source="PHANOTATE_1.5.1"
/score="-9396.028637926433"
/phase="0"
/translation="MGWWQRLRLGLFGGPAPTYDGTGGGRRALAWQVGNPGAVAALAYS
QDELRAKSRDQVRRNVWAAAGVEAYVANAIGTGIKPQSMAADVTVRSAIQALWAHWGDD
ADAAGLTDVYGLQALACRALVEGGEALVRLRYRRPEDGLSVGLQLQVLEPEHLPVALNR
ELPSGNVIRAGIEFDRLGRRVAYHLYRSHPEDGALAPMSGAGGLDTVRVDASEVIHLFR
PLRPGQIRGEPWLARALVKLHELDQYDDAELVRKKTAAMFAGFITRLAPEDPLMGEGPA
DAQGVALAGLEPGTLQLLEPGEDVRFSQPADVGASYAEFLRMQFRAVAAAMGITYEMLT
GDLTQVNYSSIRAGLLEFRRRCEAIQHGVIVFQLCRPVWRAWMTQAVLEGALELPGFSR
RTREYLAVKWVPQGWQWVDPKKEFDALQTAIRAGLLSRSEAISAFGYDAEDVDREIAAD
NARADALGLVFDSDPRHDRAAAPARGSAPAAGGNRVSV"
CDS 50590..51207
/ID="CZTTGXGG_CDS_0090"
/transl_table=11
/phrog="No_PHROGs_HMM"
/top_hit="No_MMseqs_PHROG_hit"
/locus_tag="CZTTGXGG_CDS_0090"
/function="unknown function"
/product="hypothetical protein"
/source="PHANOTATE_1.5.1"
/score="-18.332951222829895"
/phase="0"
/translation="MTALPRRPGDRRRQPVETGSAFEVAIEILVRPHGLEIDQAQAVIA
GVAEQVELHLAADFIDPQPGQIAFLFLTDARIAGDHRDRRIDVGRHWIVQFLVGARETA
RLQHLPVHRRVRGTNGVACSGDSFSAIRLSAMKEAGRSGLSSAARPTLAQNSICPCTVR
NSALAAARASSIKVSSIRTETLMIGSQVVRHNCSQSGRGGQG"
CDS 51207..52496
/ID="CZTTGXGG_CDS_0091"
/transl_table=11
/phrog="53"
/top_hit="No_MMseqs_PHROG_hit"
/locus_tag="CZTTGXGG_CDS_0091"
/function="head and packaging"
/product="head maturation protease"
/source="PHANOTATE_1.5.1"
/score="-2229.934909481673"
/phase="0"
/translation="MLPHLAGRLYGTPLLIARPKLEVILAVLGARIGLPAADTPVPAPM
PRAAAQALPGIAVILIHGTLVRRTLGLDANSGLTSYAEIGAQLDAALRDPEVAGILLDI
DSPGGESGGVFELAAKIRAGTSHKPIWAHANDTAFSAAYAIAAGASRVTLAQTGGVGSI
GVIALHVDQSVKDAREGLTYTALYAGHHKNDLNPHAPLSPQAAAALQTEVDRLYAIFVR
DVAVLRQLPETAVRATEAGLFFGEDAVSAGLADGVLGFEAVLSEFADALQAQRRLSVIQ
AHQTPVISTRIMENPMLENPAEPIAPAQLGEETSRDPNPGPSATTDGSVPQVASSLPPS
PETGQADAHHEARAEAQAIAELCLIAGAASRTAEFLAAGMSEAQVRRALLAARAETPEI
ASRIQVDAGTSVRPEASPVVAAVQKLIARS"
CDS 52500..52877
/ID="CZTTGXGG_CDS_0092"
/transl_table=11

/phrog="49"
/top_hit="No_MMseqs_PHROG_hit"
/locus_tag="CZTTGXGG_CDS_0092"
/function="head and packaging"
/product="head decoration"
/source="PHANOTATE_1.5.1"
/score="-5.436531734776032"
/phase="0"
/translation="MPKLREPKNLGDLLKYEAPNRYSRDLAPVALGQKLALGAVVAREP
AGARLQALDPAATDSLAQAVGVLIEAVDATAAEVPQALLLARHAIVSDPSLAWPAGITP
AQKTTAIAQLQALGILVRTGA"
CDS 52880..53881
/ID="CZTTGXGG_CDS_0093"
/transl_table=11
/phrog="29"
/top_hit="No_MMseqs_PHROG_hit"
/locus_tag="CZTTGXGG_CDS_0093"
/function="head and packaging"
/product="major head protein"
/source="PHANOTATE_1.5.1"
/score="-1792.6490012980169"
/phase="0"
/translation="MQNPFHNPAFSMAALTAAINIIPNRYGRLEDLNLMPVKPVRQRQI
LVEERNGVLNLLPTLPPGAPGTVGVRGKRTLRSFVIPHIPHDDVVLPEEVQGIRAFGSE
TELEAVAGVMARHLETMRNKHAITLEHLRMGALKGVILDADGSVLYDLYDEFDIPPKTV
GFQLGTATTDVKARCMEVLAHIEDSLLGEFMTEVHCLCSPEFLAALTGHKDVKTAFTHW
QQGAILINDVRRGFTFGGITFEEYRGRATDVHGVTRRFIAAGEAHAFPLGTVDTFATYV
APADFNETVNTLGQPLYAKQAPRQFDRGTDLHTQSNPLPMCHRPGLLVKLTV"
CDS 53884..54192
/ID="CZTTGXGG_CDS_0094"
/transl_table=11
/phrog="82"
/top_hit="No_MMseqs_PHROG_hit"
/locus_tag="CZTTGXGG_CDS_0094"
/function="connector"
/product="head closure Hc1"
/source="PHANOTATE_1.5.1"
/score="-3.8295138664683095"
/phase="0"
/translation="MDLDAVNAVCLSTFGETVILWPDRTDPPPVHLTGIVSVPAGLERP
GPMGAPRPEADPRLTVRSADLPAVRMGDPLKLRGLPYGIASRLDRLDGLTTLILRPR"
CDS 54189..54515
/ID="CZTTGXGG_CDS_0095"
/transl_table=11
/phrog="785"
/top_hit="No_MMseqs_PHROG_hit"
/locus_tag="CZTTGXGG_CDS_0095"
/function="lysis"
/product="holin"
/source="PHANOTATE_1.5.1"
/score="-7.560138075381887"
/phase="0"
/translation="MNAPPKKLLLVSPHGNGDEVRLALQIILVTIWSGLVAQLNRWRRH

PRPWRPCLWCLLVDVVSCSLVGFSVWLLAEREGLGYYESLWAAIVAGHLGARWFGLLIH
SSRG"
CDS 54525..55037
/ID="CZTTGXGG_CDS_0096"
/transl_table=11
/phrog="27"
/top_hit="No_MMseqs_PHROG_hit"
/locus_tag="CZTTGXGG_CDS_0096"
/function="connector"
/product="tail completion or Neck1 protein"
/source="PHANOTATE_1.5.1"
/score="-15.449912319074132"
/phase="0"
/translation="MSLGLRVQLHGDTAQDLRRLPAVLRKASRRAVGQTARETREAMVR
AIGATYGIPLRALRSRRVQAYLRLAGVRARVWTGHAPIKAAYVGRLRQEEWGSSAGAYL
FPGSFVARMPSGHRGVFHRAGHASLPIIEDVVALPKVPALAEALATRATLRLAALIREM
TQDALPR"
CDS 55034..55471
/ID="CZTTGXGG_CDS_0097"
/transl_table=11
/phrog="No_PHROGs_HMM"
/top_hit="No_MMseqs_PHROG_hit"
/locus_tag="CZTTGXGG_CDS_0097"
/function="unknown function"
/product="hypothetical protein"
/source="PHANOTATE_1.5.1"
/score="-11.722828420427204"
/phase="0"
/translation="MITSMFEIEPALILRLRDTLPEFVTVDSVGVLAGVQNLEPLCPAA
LVLPLGFAGPVGTPPPNVFLAERQRWQITVCVAHAPPASTVVTGGEYVLRILRALEHWS
PQPGWGRLKHVGLDDPWFDLGHVEFSLVFEVRPLPLDTGTP"
CDS 55533..56036
/ID="CZTTGXGG_CDS_0098"
/transl_table=11
/phrog="807"
/top_hit="No_MMseqs_PHROG_hit"
/locus_tag="CZTTGXGG_CDS_0098"
/function="tail"
/product="major tail protein with Ig-like domain"
/source="PHANOTATE_1.5.1"
/score="-82.75385484192005"
/phase="0"
/translation="MTTTAMLLTGTIKGAVGAGAPVDLGNAALDLSIEEDTKELADYQN
PGGGSIASLSRIKSVTLKLKLWSISKENLALATRGTVSGNSIEALTQTAEDWHITFDGV
NEVNGDAVAYDFYKVKFSPASSLPGPGTEDFAVLELTGKVLKDTSKTGAGVSQYFKATI
TPAV"
CDS 56043..56402
/ID="CZTTGXGG_CDS_0099"
/transl_table=11
/phrog="7608"
/top_hit="No_MMseqs_PHROG_hit"
/locus_tag="CZTTGXGG_CDS_0099"
/function="unknown function"

/product="hypothetical protein"
/source="PHANOTATE_1.5.1"
/score="-11.109970514456904"
/phase="0"
/translation="MAELDTTNPKGLRQERTIEAGGRKVVVRELTVGEVRAWLKDANAE
LDRNDLVALALFADITLDDLTRFSDLSRAELDAMLPSELDKVREAAKSLNPHFFGLRER
LANAAQAAATAPPAT"
CDS 56414..56509
/ID="CZTTGXGG_CDS_0100"
/transl_table=11
/phrog="No_PHROGs_HMM"
/top_hit="No_MMseqs_PHROG_hit"
/locus_tag="CZTTGXGG_CDS_0100"
/function="unknown function"
/product="hypothetical protein"
/source="PHANOTATE_1.5.1"
/score="-1.523143471256939"
/phase="0"
/translation="MTALALIERGHAGVWHYPWRLFLQARLMRRR"
CDS 56514..59681
/ID="CZTTGXGG_CDS_0101"
/transl_table=11
/phrog="339"
/top_hit="No_MMseqs_PHROG_hit"
/locus_tag="CZTTGXGG_CDS_0101"
/function="tail"
/product="tail length tape measure protein"
/source="PHANOTATE_1.5.1"
/score="-13344447162.407766"
/phase="0"
/translation="MTTFKTVIEIAANTAAAEKGLGNVAAQVDRLTGSLRRIGQYTVGA
FGAAEALQAARDLGKLSDQYRNLEGRVKLAAGSQSQFTEAQRALFAIAQNNAQALNGVS
QLYSRIAKGAGEMGVSQQQVLSIIDSVAKSFRISGASAEEASGATLQFSQALASGVLRG
DEFNSIMEQSPRLAQAIADGLDVPIGKLRAMAEAGELTTQKVVGALQKAKASIDADAAG
LPDTIEQAMVRWDNAALKFVGTSPEITRAAETIAAAINTAAGNIDTIANGVEIAGSLLV
AVLAGKGTAAVAAFAGSQARLVQANLAAATAARNHALNEEYNARAMLASAEAAVANASG
MARLAAVETALVPASQRLAVAQAEVAASAGPLKIALGGLASFLGGPTGIAVLLLTAVSA
WHLFGSAAESELERVIRKRRELAKETGKDTRGKSETDLSLLQDEAAVKKQEAVVARLAA
RYKEVGQAADQAFMGGKLGRELVGETALLREMQAELVKRKQAATDSGDALKAKEKQVQQ
SLNDTKYALKDATTQAETYYKRRVDAIDAIEQTGITRIIQTPPTQPVPGESSTLGSTAQ
PNNKAKTELEQAQAVFRLQQQAEQARLTAARDYANQRLALVDQVYGREIAKYKDGEDKK
TALERESLQARRAIYTELEAAYTASIDKLVAQERRLRDEAVAAAKARQDIELETAQAVK
SMAQGAASTEEAARAQYLELQRLIAEQQSALAAGNLDLSKQAGNQAKGIAEALGQQIAS
ALRDVKQKIAEQGKTADAANPPIDHQGNWWDQEFGAAAKDQSGQFIRNAPAQPEPLKPD
LGTQQDLQKLIQLRQEIGQLLADAETKTGQTATHQAEQTAQQIQTAMQSAQSVGAEIDK
IDQQLRHGFNLDIKADPAALQALQTQLAELLRPETKIINIKVVKDGNSITATPDGSALP
DTGLTVPGYRRGGWIKGYGGGDRIPALLEEGEFVLRKEAVRKLGLDKLYALNNLALPRF
SIGGYVAQALPAFDASLTRSSDGGQPVHIHLPGIPGSFPLNGDPQVVAALKREVARAAL
KHGRIVR"
CDS 59710..59985
/ID="CZTTGXGG_CDS_0102"
/transl_table=11
/phrog="No_PHROGs_HMM"

/top_hit="No_MMseqs_PHROG_hit"
/locus_tag="CZTTGXGG_CDS_0102"
/function="unknown function"
/product="hypothetical protein"
/source="PHANOTATE_1.5.1"
/score="-38.962786560930006"
/phase="0"
/translation="MNPILRWALWLVVQAVSGITKEQWGLIQDKVAALETRTIKGIVDK
TTLNDIKKKEAAAYIGTFVSGVRTNVVHFLIEAALWFVRSFGVKAP"
CDS 60028..60294
/ID="CZTTGXGG_CDS_0103"
/transl_table=11
/phrog="No_PHROGs_HMM"
/top_hit="No_MMseqs_PHROG_hit"
/locus_tag="CZTTGXGG_CDS_0103"
/function="unknown function"
/product="hypothetical protein"
/source="PHANOTATE_1.5.1"
/score="-14.040229763127083"
/phase="0"
/translation="MFEKVKYLYECMQAGKSLQNPRLWSQRASLIAVLTALLTAGVGLA
RAFGYDVDATGTDIAAVAQGLGVLGVIVVDVIHRASNKEAGKR"
CDS 60291..60764
/ID="CZTTGXGG_CDS_0104"
/transl_table=11
/phrog="7"
/top_hit="No_MMseqs_PHROG_hit"
/locus_tag="CZTTGXGG_CDS_0104"
/function="lysis"
/product="endolysin"
/source="PHANOTATE_1.5.1"
/score="-43.950681508479214"
/phase="0"
/translation="MRRTKAATMSVSALIVGMIALHEGYRGEAYDDGVGVQTIGFGSTA
GVKRGDRTDPVRAVQRLAADATRVSQAVARCVGEVPLYQHEFDAYVSLTYNIGVNAFCG
STLVKRLRSTPPGYAEACREILRWNKAGGRIQKGLVRRREAEYALCVGTAQGG"
CDS 60730..61038
/ID="CZTTGXGG_CDS_0105"
/transl_table=11
/phrog="No_PHROGs_HMM"
/top_hit="No_MMseqs_PHROG_hit"
/locus_tag="CZTTGXGG_CDS_0105"
/function="unknown function"
/product="hypothetical protein"
/source="PHANOTATE_1.5.1"
/score="-4.740256288537627"
/phase="0"
/translation="MRCASAPPRAARHDRTDAAGNHLVDGRRAGFPFGSAQDRRRIGRG
AAGVSLVLTLSGCCSLEPWVTLAPRACAWPVRLSGLVAGVDEVGLALRCDELEPDEQ"
CDS 61149..61496
/ID="CZTTGXGG_CDS_0106"
/transl_table=11
/phrog="5314"

/top_hit="No_MMseqs_PHROG_hit"
/locus_tag="CZTTGXGG_CDS_0106"
/function="unknown function"
/product="hypothetical protein"
/source="PHANOTATE_1.5.1"
/score="-9.662583989528235"
/phase="0"
/translation="MWSGRSVKQQTWRKLTTEISGGGWVPPGLSALDYSQPLVLACAAP
LSVVGGGSPPVPVRTEPDFATVTIDVDGVAITYYYPKITVYCEPPEQQLDGVSGNYSWR
LNAEMVDPLGA"
CDS 61498..62247
/ID="CZTTGXGG_CDS_0107"
/transl_table=11
/phrog="No_PHROGs_HMM"
/top_hit="No_MMseqs_PHROG_hit"
/locus_tag="CZTTGXGG_CDS_0107"
/function="unknown function"
/product="hypothetical protein"
/source="PHANOTATE_1.5.1"
/score="-294.9906900936422"
/phase="0"
/translation="MLNLAGLNGVKAMVTDALVAPSDFTMNVSALGAPWVLPPVLASGE
YYYLTIIDQDYPTKWERVKVTACQGMGANCLLAITRNVASSTGVAQTFTQGAFVQWSPG
VEEMESRWRMILSASMTGTNTMTSTRFVAPFSGSTTGSMGTAYWINRTGKTLRLSNLVA
ALSTGSSFSGDSIVVSVEAGSTAPTSPSALQVVVSPGDYGFKSNNVNTVTVGPDSLVCF
MLSRSNTGAAVSGGFQLANVSVLAEEL"
CDS 62247..64562
/ID="CZTTGXGG_CDS_0108"
/transl_table=11
/phrog="5842"
/top_hit="No_MMseqs_PHROG_hit"
/locus_tag="CZTTGXGG_CDS_0108"
/function="unknown function"
/product="hypothetical protein"
/source="PHANOTATE_1.5.1"
/score="-288447872.1155633"
/phase="0"
/translation="MTLPVGGVTGIGGVAAGPDVGERYALTAVLEQAVFSVYGLGTGIT
QAVVDIDAYDVFCVIDQPVFERFGLSAAVEQTLLDVYGMTAPLRFEVLAVAGNSASIQQ
VQFSISSLAAPLQQSIFFKPYRRAAWITQWIGEVYGVVAPITCTVDHLPFDLAAQVELA
VYEVVAALNVPFRHTVFDAQAVAGGAFVAQAMAASADITSWAVLVNIGNAAGDGEPLNA
GTGAVMGALLGRLTGQMTVDAEEGAARIAEFSVLPEPGPISINALTGLPITIYLVAGTT
HLPIFQGILDIPGWDPVQGVLQLSCTDNLQNRFDGIKRKDIAAIIGGYWSEYVFDKKAD
EWQYLQDRLSTLPASYDLDLNLQGVVTPWRAKTAPDFTFTRDAVIEHSIRIDMASARQL
VNTIEVKMQYRYERLMQRNVGIFWEMDMHELVAGGAAPSVETVFQAVDGTGWACYGLTM
KAVPTGSFEGVGLLGDIPRYSLTAGMQCALAQRWTQTVTEDWSVTVDAPDSIKIIGKRK
GSTTANFDSNADKDPRYTHWDRITEKEIKLTLIGQDMVSPIAGDSSFIPYHPLNIKQYT
VAVPDSAKRLPSQDPAANPQGDLYYDLDDGAQDNRTGLSNGYMTGIARAQRDILSSHRQ
TTLSFTGLIAPLLDRMHTVRMLSARLTAKGKVRRVTHHLDFDAGSALTEVAIAVSKSYG
IGMVDTPYDLQAPAKPAVVQPPLKYPMERPSATYDSGTGEFIVTVPGVAQEHIDATHSA
PADHVSIFIPDDELLLEA"
CDS 64567..64992
/ID="CZTTGXGG_CDS_0109"

/transl_table=11
/phrog="No_PHROGs_HMM"
/top_hit="No_MMseqs_PHROG_hit"
/locus_tag="CZTTGXGG_CDS_0109"
/function="unknown function"
/product="hypothetical protein"
/source="PHANOTATE_1.5.1"
/score="-21.768736379027107"
/phase="0"
/translation="MSSFKLYTDAALTTPLTGSLVAAQNADGSTPAIQFSLYLGSTTAS
RMIQANANPGVDQIQATVVDAGAGSGHDVSEVKLAATQAGLASAVGGAALDLGTSILSG
AANAKPIWIEVNDATHVVGTATELSVVIADVRETAIV"
CDS 64992..65357
/ID="CZTTGXGG_CDS_0110"
/transl_table=11
/phrog="7250"
/top_hit="No_MMseqs_PHROG_hit"
/locus_tag="CZTTGXGG_CDS_0110"
/function="unknown function"
/product="hypothetical protein"
/source="PHANOTATE_1.5.1"
/score="-18.843820051611452"
/phase="0"
/translation="MTVQKFFVNDINAIVDAVRSSGEDLPEPAAKPGIGARTGVGAPKR
GGGIASPLTETTAPNAKGEFWADREYYPARDMETTDGEFVIKYAAIKTLKFTDANGEAV
EFHFAEPDPDKALGTTA"
CDS 65354..67726
/ID="CZTTGXGG_CDS_0111"
/transl_table=11
/phrog="No_PHROGs_HMM"
/top_hit="No_MMseqs_PHROG_hit"
/locus_tag="CZTTGXGG_CDS_0111"
/function="unknown function"
/product="hypothetical protein"
/source="PHANOTATE_1.5.1"
/score="-34275189415.69069"
/phase="0"
/translation="MKTIARQSDPFDLVRIVDIQACGLLCTPTAGNYPDGWGEPFKDAQ
DQPIHPPLGTYAPASGAIGVELSAETDPKKPKALQVKRRASGRKVGNCLVERKIGKRWI
RVSFDGPFNRVFVDNDLQPYGWDVGTDRYSEEPWSNYIYLRGKRLYIDAKKNVYDSDFF
GRIVGVGIRDENTLVLAAIHRNEIAFAPLDISKPYADGQTVSVTYYKLTPQVQDLPGYT
FYPGFTVKPTPPGFVFHGPIYPQIDTPFEFNSDCTQASCIAKSYRFPDTSMYVARRHWR
IANGYKSEEEGDVDSEFSDGVPYNWANSDLADDVEAVITFLNESPMESVINKLGAARLN
PNFKRVPFFQRYLHDVELIWSQDDKNKTNKKKVLSAVQGRATRVDAHQGGESFWTPNSA
RVETLNPHTATVSWSTYKIYNTNPDPAQLIATGSPGDYDPGRNNNQRHAGESFVDYTIE
RGYESHFDLPVYVYYDDEDMKSFIRFFGEDTYQYTEKIRFHASGVVPVGESGMGSTGYG
EEISGSSIVGIQSHDGANQKVYYFGSVDTVYGEDYTDNTCRKARSLKVYASHPYAGYPV
MERVTIRDGTGEPPPGPPYDTAYGMNGVPGEFFFISEGLIYVESDLLARIIWMDPRQNN
YLFAKSTHQGAWYGTTVQNSTDPFFDARLSSILYGVAPGLASGEIVNIDTLEKFLGGKL
MTEVLHTGWPETKGALVKCKSFKSIDYGGCVTLPESGIAVASYKVPVWHESADPPQRLR
YRTFSAISTGQQLREMTGLSAGAPNSYADFYAPLNLF"
ORIGIN
1 ttaacccttt tttgtttctg agcgtagccg attcgtaacc acccgacgcg ggcagccggt

61 catgctggtg acatattgcg cgaggtgttc accgctcaga tgggcgtagc gcttcaccat
121 ttccaccgac tcccagccgc ccagctcctg gagcacgtgt aacggtgttc cccgctgtac
181 gtgccacgat gcccaggtat ggcggagatc atgccagcgg aaattctcga ttccggcccg
241 tcccaaggct ttcgtccaag catggttatt cacccggctg acagactgcc cacgatagct
301 gaacacgtgg gtgtgatgct ttccgagctg gcgccggatg atttcgaccg cgacgtccga
361 aagcggtacg gcgatggcct tacgcgcctt ggcctggtcc gggtgaatcc aggcgacctt
421 gcggacaagg tcgagctggt tccagagcag gccggtgacg ttggcctgac gcagacccgt
481 ttccaggctg aaacgcacca tatcagcaag gtgcgccggc agttcggcca ggagcctttc
541 cgcttcgtct ttcgtcagga agcggatgcg ctgtttcggc tcgggcagca tccgaacttt
601 cggcatggaa tccaaccaac cccactcgtg cacagccttt ctcagtatcg atcggacaat
661 ggcgagatgc cggttaatgg tcgcatttga agcgccctcc cctttcttcg ccgatataac
721 cgcgtcgagc ttgtaccggt cgacctcaga gagcattacc ccttccagat ggacatgaag
781 ccaccgcagg cggttgatgt cgtcacgccg ggaggctttc tcttccgtgt catcgagcca
841 ccgcaaaacc gcctgttgcc aggaataccc cggtttcacc ccgaggcggc tttgttccca
901 cagttccgcc ttgagcttgt cgtggagttc ctgggcgcgt ttccggtcgg cagtcccagt
961 gctttcctgt aggcgtcgtc cgttgacgtg gagcttgatc caccaatggg gactatcttt
1021 gcgtttgtaa agtgacatgg gttttcctct tgcgtgctgt caccttgcaa cgcccgcgag
1081 gggtattgtc cgcgcagcca ggcggccaga tcaacgtcga ggaacagcca gcgcttgccg
1141 accttggcgg cgggaatctc gccggctcgg gccttgtccc ggagggtgac cgggtgcagc
1201 tgcaggaact cggcggcctg ctggaggtta agggtttgca tgggaccgga tcggttcggt
1261 tttgtttgag cggtcaagcc gccttcccga gcgttcgccg cttccatagc ccttcgcgga
1321 taaagtcctt caaatcctgc tccacctggt ccttggcctg gccccggcgg gcgaggtgtt
1381 ccagcagctt ggggtagatc gacaggcgct tgatctggat caggatgtaa tcggcattcc
1441 tgccctcacg ggcatagagc gaaatgaggt tgccgagcac gttgccgaag ttgcgggcga
1501 ccgcggacac gtcctctttt tttttacgca cgatcttgta gccggaggcc gggactttga
1561 attccggatc gtcgcgcagc agctgccaaa cggggtcgat gtaggtgccg tccttggcgc
1621 ggttgatggt cagggcatag cgccacatgt cggtgaggtg cggcacgacg tccagccagc
1681 gcttgagggg tttacccatg ccttcgccga tttcgttcac caccgagtga tggaaccggg
1741 cttcgatccg ccacaccggt tggctgggat cgaattcgcc ttcggtgaac tcgccccatt
1801 gcgcttgcat gaagtccacc ttgtcggcat ggacgatctg cttggacttg tcgtagaggc
1861 agagctggag cgatgccgcc ttgccgaagg tgaggctttc ggcggcctta tcgccgtagg
1921 tcaccgccac ttccgacagg ccgtggaatt cggcatccga gatcccatcg aagcggcggg
1981 taattcgtgc tcgggtgatg aaacggttct ggaaccccgg atcgggtgac cactgctgga
2041 cgtcgaccgc cagatgcacc gccacacccg aagcctcgtg cgcgctcatg agctggcaag
2101 cgaagctgtc cagggtggtt tgaatggcgt tggcgttggc ttcggccagc agctggggcg
2161 acagctcgat tttcagatga tgggcgggcc ggccttccag caagtccact tcggcctggt
2221 agtagctgcc gatcagcaag atgatgccgc gggtgttgtc ctggagggcg tagcggtaac
2281 cgcaagcctt gcccatcctc cgggctgccc aggggtgttc gccttccagg aaccggaact
2341 ccccggagcc ttccgcggcc tctttgatct tctggtacag ctcgggcttg agctggccgt
2401 ggtagttctg ccgcacggtg tccacgaagg tgccgcagat caggatatgg gacaggtccc
2461 aggtgatgcg gggggttttg aagatacgcc ctttcgggtc ttcttccgtg cccttggaca
2521 ggctggagta cgaataacgc tggaacagct tcagatcggc catggtcttt tctcccttct
2581 cgttgctctt gttgctctga acttcttcaa tccaaaaacc tttaaacgct caaactgtct
2641 tttgtgcttc actgtgctcg aatgaggctt tgcatccccc tagatcagac gtgttacagg
2701 cacgtctgac cggctgcgcc gctgcgcgcc gtgggcgcgc gcccgcacgc gcccggcacg
2761 cagcgtgcgc cgaccggtgg agcgccccgg ccggggctcg gcaacccggt cgcagcgccc
2821 gcagcggcac ggcccggccc ttagcggtag ccggcatctt tcaggcgctg ggtcaggtcg
2881 aagtcgtcgg ctttctcggg cttttcctca tagagcaggg tcttgcccag gacggccgaa
2941 tgcaccaggc cgaccccgcg caccacgacc agcacgccgg cgatggcggt gccgacatac
3001 atgacggccg aagccaccga gtgaaaatcg atatcgatgc tgggaatcag cgaggaaaaa
3061 tcgggtgcag ccatagatca cctcatgggc cagggtgggc gggggggttgg aaagcgcttt
3121 cggcgatatg gaacggagtc tgcaccgggg ccggccccga ccagcgggcg acgatctcgc
3181 cgtccaccag gcactgcggc tcgacctcga gatcgacaca gcggcgggcc gaaatcaccc

3241 ggaacttcct gcgcttgtcc tgcaggtaca cccggcgcga ctcgccctcc ccgctgaagc
3301 cgaccaggcg ccaggcgtcc gacaccttgg gctctggggt ggggaggggg gtggcttcgg
3361 ccaccggcgg cgacgtggcc tggggaagcg gtgaaggcgt gggcgttgcc gctaccgcca
3421 ccggctcccc ggtgtactcg ggcaggacct tcggcttctc ggccagggcg gctttcggct
3481 cgggatggaa gaagccccag accccgcgga tggccagaac cccgaacagg acggccgccg
3541 gcaccccgaa cttcagcagg ttgttgcccc agatggtggc gcgtccgtcc ggcttcgatt
3601 cgatccccac catatcgtcc agggccttgg tgtgggactt gtagagccgg taaacctccg
3661 gcttgtagct gccgaagatt tgccggatcg cggctttgac cgggtagcgc ggcccccgct
3721 gcggcccgcc gtagatatcc acccggtaac gcttgtccgc gccgagggta tcgagcttgg
3781 tggcgacgaa ggtcttatcg accaggttgc ggacataact gcacaggttg gtcaggtcct
3841 gcacgatcag cacgatttcc tgcgaatact gattcgcgcc gaccttgtgg cggtgctcgg
3901 caaaaaacga cttgtgttct tcggtgagat cggcggattt gatgccggcg ggccaaaacc
3961 gccaggcttc gtcgatgacg atgacggcgc cgcccggcag accggcccag aaccccggct
4021 gctgggcctg ggcattatcg aacacccgga tttcgacatc gggccaaacg atctcccatt
4081 cctgcatgac caagggaatg ttggtccaga tggggcgcag ccgcttcacc gacggcagaa
4141 tgacgttctc gatcacgccg taactcttgc cggcaccagg caggcccaca taggcgagga
4201 tggacatggc tcaaggcttc cggaaacgtc cggcaaagcc ggcatcgctt tgggtgggat
4261 tgatctgtcc taccatggca ccggtctccc tcagccgatg aacggcaaac gccggatcag
4321 gaagcggacg ccgtaggcgc agcagatcat gccgagtccg taggcggcct ggaacggcgc
4381 caggaagaag ccgacccccg aggccgccac cgcccccatg aggccagaca ccaccgtcgg
4441 caccgcgatc agcgccgaac agcagccgag gaggaaactc ccggccaggg catcgccgag
4501 gatttccagg aggtgcgacc agatccactt cggcacgtat agcaaggcat cccagatcca
4561 ctggacgaac tgggccatgt ccggaaacag cccctcggcg tgggcctggc cggcataaac
4621 cagcgccagc acccacaggc taagcgctga acagaatgaa aaccgcgatg agggaccaga
4681 catagggcat gaaactcgcg atggtgggag agacttccgc ccagatcgtg caatgctgat
4741 cgatcacgaa cgcgcggttg aaccaggcca agtcgaccgg ataggtgaca cagccgcctg
4801 agggttcgga agcactgaca aggaacatcg cccccaaggg gccggtttgg actttttgga
4861 tgaagatttg ccaggtggtg tcgaagtcat cgatctgggg gacttggccg tatttggtgg
4921 tgtccttgat gagcttctta tccaggttgt ccaacgactc cttggcggct tccgtggcgg
4981 tggccacccg ctgctgggtg acttccaggt cgagaccgcg aatgtcgacg gtgggctggg
5041 ctttaagcgt tcggtcaatc gaatccagcg aagcgcggct cgccgcggag tcgccgcgga
5101 tggcagccag gtcgaccttg gcgccttcca ccgcgacccg gctcagggcc acgtcctgct
5161 tcaccgcgtc ggtgttggtc ttggtggcct cgctggcagc cgccacccgc tgctgggtgg
5221 cttcctggtt caggctgctg gtgtcgatgg tggtgccggc gccggtggtg ttgtcgacgt
5281 tggtgatggt ggtgttgttg tagctggtcg tggtcgaact ggtgacattg ccgttggcgt
5341 cggtgtggac ctggtcgcgg ttgaccaccg gcttgccgtc gctgccgacc gattcggtgt
5401 cgcggacgta gtcggcgccg ccgtcggggg tgggcttgat cgtttcccgc acgctgttgg
5461 catgcgcatc cgcgccggtg cgggtgtcct gatgcgtccc cacatcggga tcgcgctcgg
5521 ccgccaccca gccggtgcgc ccggcccgat aggtgggctt gccgtcggag ggccattgcg
5581 ccttggcgcc gttcgggcaa gcactctgca agagacaatg gcccttgccg tcgtcgacat
5641 aacaagtacc acacgtagtc gaggctgagc tgacagcgat gatgcagtta cagccgcccg
5701 gaccattgca gtcgtaataa cccgacccat ccggattcga atagcccgac cgggggaggc
5761 attcggtcac tgtagggcca tacgcattcg cgcagaactg gacacatgcc gccgcgggat
5821 cagtaaacgt gcccttaatc cagccccggc tgtcgtaata ggtgtaattc gacgtccccg
5881 gcacggacgc gggtggggtg tccggatcgg accagcccgg caccgccgga tacggcaggt
5941 tctcgcccgg cagatactgg tagcagttga ggttgtcgca cagctcatac aacccgatgc
6001 caatcccgag gccgatggcg gcgccgaccg ggctgaaggg accgcggaag ccggacaggg
6061 cttcccaggc tttggcaccg ttcgagaacg ccgtggacag cgcattgagc ggggccacca
6121 ggttggtcag ggaaggcacg gcgacggccg gtcccggcag cagggccaag accgagacca
6181 ggctcagaac cgtgagaaga tgccgtaggc gcatgcggcc cccatcacga agaaggtcca
6241 gtagtagaga tcagccatga cggaccaccg cattgaggtc gtcttcgtca tcgaattcat
6301 cgtcagcgtc gtcttcgtca tcgtcgaaca tggcaaggtc ctcggctcag cgaaatccag
6361 gtgaatcagc gcagcttgag agaaccgggg cggtgacccg ccccgatccc aaccgtcaag

6421 cggctaagcg cttaccggcc gccgatggcg gagagcacca tgcgggtgcc gcgcagggcg
6481 atgagggcgg cggcgacggc gacaccgacc gccatgatgg cggtggcggc ggtgccgaag
6541 tcgatgccgg tggtgagggt ggtgaagtcg ggaccggcgg cgaacaccag gtcattggcg
6601 gccgccaggc cggtgccggt ggcggtgaca acttcggctt tgtgggaccg ggccgcgttg
6661 accaccttgg ccgagctttc cgaacagaac ttacgagcgg ttgcaaacat aaagatcacc
6721 tcattgtcag gtattgcgag ttaatcccgc cggtcgccgc ggatggcatc gagcaccatc
6781 gcgacggcga gaccgacgag caggacactc ccgacgatca cgaagcctga agagaacgcg
6841 gagagacagg cgatcggatc gagcgtggag atatcgaacg cgccgggaat gccggacgcg
6901 tcgatattca atagccacgt cccggcacag gtcgggctga ccggtccgcc ggtgtcgacc
6961 agcagaccgt cgcagatggg gacgtagcgc atgttgggtt tcccttaggg cggcagagcc
7021 gttaagcccg gaccggggcg ggttcggggc cggacagggc gccggggctt ggcgcggcgc
7081 ccggcttggc gcccgctccg cttgggttcg gttcgggctt ggcggcagat gctgtaccgc
7141 taccgttccc ggcctggccg ggctggagcg gctggccgtc cttgccgagc ttgaagagtt
7201 cgtgcagccg ggctttgtcg agcaggttgt cgggccgcag gccggtgacg cggagttggg
7261 ctttgtcgcc ggcgccgcgg ccgagctgga cgtcgatgag gtattcgccg gggacctgga
7321 gggcttggag ctgggcgaat atctcgtagg gcatgcgcag ccggatgagt tcgaggccga
7381 actggttggg gttccttccg ctggtgggct ggaggatgta gagccggccg cctttgccgc
7441 cgccgtcgtc ggtcatgtcg tagcgggtgg cgctgacgac gaggccgcgc agggccccgg
7501 ccaggggggcg ctggctgtcg gggtggggtt tgctgacggg ggcgttggga tcgatatgga
7561 acaggtcata gagcgcggct aactcgaaat ggccgctgcc ggcgagggag aggacgcttt
7621 ctttgtcgct gtcctggctg ccgcgttcga tgtcgaccag caggtcgtaa tggccgggca
7681 ggggcttttg gctgagctgt tcgaccaggt cgtaaggggc gttgaacttc tggacctgga
7741 ggcctaagtg attctgggcg tcgtcctggg cgggctggag gacgtagacg ctgccgcctt
7801 tgctgccgtc gatgtggtag cggttggcgc cgatgacggg ggcgctgatg acgatttgca
7861 tgggttttct ccgtttgggg ttcacgcgct tgggggtgca gccggtctgt ccggtgtgcg
7921 cgtaggggca ccgggccggc ggaaggagtt gcggaaattg gaagctgacg ctgacgctgg
7981 agctgaagcc ggagccggag ccggagccgg gagcggcatg gcgccattgg ctttggcctg
8041 agcctggagg ctgcggtcgt agtcctggaa ccaggcgagg gcttcgacgt agtcggggcg
8101 gagttcgccg gtgtcgcggt cgatcccgtc gcgcagggcg tcgatgtagg ggaataaggg
8161 ttcgcagcgg tagctgtcgg cctggatgac gaggttttcg cccggtcccc agtaccagcg
8221 ctggcgacgt tggagccggc agctcttggc ggggttcttt tcgacttcgc cgcggtagta
8281 ggggcagtcc cagcattgcg ggggcgtgcg ggtgaaggac acgcggcgca ggaggaggcc
8341 gccttggagg ccggcggttt cgggggcgat ctgctcgctt tcggtggagt agcggccgcc
8401 gccgatgggg taggcgcagg cggcgggcgg ggtgcggtag tcgaagcggg cggcgctgtt
8461 gagcagcgcg agcgcggggc cggtgagacg gaagccgcag acggtccagc cgttgggtaa
8521 ggcttggagg tagtcgcagt cggggcagtg gcggaggtta gtacgcacag gcgagctccc
8581 ggttaagacg ccagagccag agggcgctgg cgaggagttc ggagacggcg tcgggctcaa
8641 ccggcttggg agcatccaag ccggggcggg cgcggtcggc aggaggaata aaagacgccc
8701 gtaccgtcgg gacgggcgag gtgccggtgg gtcggccggc ttgggagatg accggggggc
8761 ggtgactccg accagggctg cggaaaagcg gtcctttagg ggtagggtcg gcgccttggg
8821 actgcgggac cggtgcgcac gacttttgcg cctggcgcga agggatgcgc cagggcgtcg
8881 gctgatcgca gcaggttacg ggtgtcgctg gccgctcccg acgggtggga cggttgccgt
8941 gcgcgggcca tctcgtccca aaagatcgtg gggcggaggc ggtaagcagg gcgcggacgg
9001 tcgggccaag ggcgcggggg gcgactttgc cggagcccgg tgtcgcgtag gttaggacga
9061 tcatggccga acctccggtt gccgatacga ataggtttcc gggcctcgcc cccagccgag
9121 gggaccggaa gggttgctgg tgggtgacgc gaagggaggg cgcgtcctgg ggggttgggg
9181 gaacatgggt taggttctcc ccggtatctg ggcgttgccg gtgccgataa cggcagcggg
9241 cgcgggaatg aaatggagat cggcgccgcg ccgggtgcgc tgaatgtgcg tggagcagat
9301 ccggacttcg cgaccgttcg ggagttcctg gaaccaggtg ccgggtagga cgcagcgctc
9361 cccggtccgg gtgaggcatt cgcattgcgg cgtgcggtgg tcggtgcgca tatcagctgt
9421 cctcctcggg gttgaacgtc cgggcttggg cctgggcgtc ggcttccgct tcggcgcgcg
9481 tgtcggcgtg gtcatccacc aggatctgga agcggtcgcg gacgtgctga acgcggcgca
9541 gggtgccttt caaatgcacc tggctgatca gcagcgtggt ttccaggggg aattccatgg

9601 cgagctgggt gacgcgctgc agtacgccgg ccaagtcttg gctgatgagg tcgacacggt
9661 cgaccaggcc gccgacggtc tcgcggtccg gggtgtcggg gatgccgtag ggcatgggtg
9721 caatcggcgg gtgcggggtc atgtcaggcc gcctcgcagt cgccgacccg ccggccattg
9781 gcattcggcg cggacgcttc ggccggcgcg gcttccgggg cgctggcgcc ggtaaggcgt
9841 tcgacttcgg cgcggtagcg ctggcgcgtg gtgaggtctt ccggactcgg cccgacaacg
9901 ccaccaagcg ccttccagag ggttcggaca tagccgctgg cgagttccgc gaacgcatac
9961 agctgccggt ccgggtcgtt ggcttcgcga agataaggca acaccatgcc gtggatcagg
10021 gtggcgctgt gatcgcaggc ttgcaaagct cgggaaaacc cgtccgactg gccgacgcag
10081 tgcccggagc ggcaataacg ctcggtgtcg gccacttccg acaggatgca atcgaacact
10141 ctgcggtgat aaccggtcgt cggcacggcg agagactggg gaacggtttg cgtgttcatc
10201 gttcggcctc cccttgcagg ttgagaaagg acagatcgag gtcggcgagg cagcggacac
10261 ggatgtcgac catgttgatc agcacccggc ggcccatgcg gatggtcttg acttgacccc
10321 gttcgaccag gccccggacg gtgtcttggg tcaggccggt ctcttcggcg aatttctccg
10381 ccgtcttggt gtccgatggc agcgctaaca gcgcttgacg caacgcttcc aaatccataa
10441 gttccccctt taagacctca ctggaccaca gtgacacaca gaacccgcac tagaacttgc
10501 gttttgcaaa tattctcaaa gtctatttgc gaatgcaata gttcttcgag aatatttaca
10561 aatgattggt cagcgaatca aagaacttag agaattcaaa ggcttaagcc ggagggagct
10621 ggagcggtta acgggaatcc cggactacaa atgggtctca atcgagaccg agcgacagca
10681 gacgaacggc gagcacattg cagcactcgc gaaaatttgg ccggagtaca agcactggat
10741 agtgttcggc gagacgatcc ctgaagtcgg gcagatcagc cccgaactgg aggaaaccag
10801 agaaaaacta gccaaggctg gcggatcgcc caacgggtga tcgaacgatg gggaacaggg
10861 tgggtgattc taggaggact ttctagtttt tctttagagg ccaagttcaa cataccgcaa
10921 gacagatagc agtattggga gccaaaacca tgaaacctga tcagagaacc ctattgaaat
10981 cctggcgaga tgaaatcggc acgatgatga ccactcatca ccagttatca acaaaaaatta
11041 ggcgtttgaa ctatttattg ggcatcccga ctatcgtaat ggcgatgctt atcgcgacct
11101 acgtgttttt tacagtcaac cgagacccag atttatggat cagaatgctc gtcgggttga
11161 tcgcactgct tgtcgcaatc ctttcttcgt tgcaaacctt cttaaaatat tccgagcagg
11221 cggagaatca tcgtaatgcc agcgcccgtt accaagcctt gttcaacgcc cttgatcagt
11281 ctctagttat cccgcctaaa gacgaggcgg cccttgggga ttggtgcgat aagttacgcg
11341 aacgttggga cgagcttaac ctggaagcac cgaccgtgcc gaaccggcta gaagtccgtt
11401 ctattctgga tttcgaagcg acggctatac atccgtcgca gaaacacgat acagaaaaca
11461 gtgagcggca gaccacgtag cccgcgtagg gtgcgctgaa gaatgaagcg caccgtccag
11521 atgaaagcag acctcacccg gtacgaaatc gacgaggaaa gaacatgcca gaaatcccaa
11581 gcacccaact cctggagtcc ttgaaggggc ttcgtaagct cccccgtttg ggggtgaggg
11641 ttttccaggc ctgttcttcg gtctcggagt tgattcgagc gctctggggc cagccggtct
11701 cgaaggcttg aacgaaccgg gcggatacct gttcaaggct cgccattcct aaacgctatc
11761 gctagtggaa aggtggctcc agcgtcagac aggtgaacct gaggtctgat ttctaatcaa
11821 atctttacgc tagacgacgc cgaatattct ctttgcaagc gccttgtcgg agaggttatc
11881 gatcataata tcgacaaaat tttctatgac gcgtctctgt cgcgggttga ggtaaggata
11941 tgcctcttta taaacgcgat taaaaccata aatgtcttca agcgtatagg gcggtattat
12001 ggtatcaata tagctcttga cgctctcgaa ctcggggatt tccgctcgag ttcgaagtga
12061 gataagattg ctcgtgacca tacgattcta cctttgtttt gatatttcag ccaattaggc
12121 gcgaaggcag ataatccttg ttcgttagta ttttttgatg tgtcttcttg attcagttaa
12181 tacatatttg cctatcagaa gatgatgttg cggttgggtg cgggaagtag caaaaatttg
12241 gtcaaacttg tgtatattag gacaagggta ttgttcagaa gaaggctctg aacaaggcag
12301 gagtagttaa aattaacgca aattgtgaaa ttacggaatc cggagatgta cggattgttt
12361 ttttctggaa aacgtgcatg ctcaatgtat gttttatgtt acataatgtt gctatatttg
12421 taacagcagg gatgtaatgt aacacagctt atatgcagac gtagcgagga gtcctgtaag
12481 gcctcgccta gaattaggtt ttgcaataac gtatctagcc tttgtgccat ggactgaagt
12541 accaggccca tcaagcagag cggacccgtt taccgatttc aacgccagtt tccggccggg
12601 ccgggcggag tgacgccagg gttacgaccg gctgcagggg cgttcaattt tccgacggct
12661 cccggttggt tgacgcccac gtcgcgatgg acgttacctg ccgggccgag cgggttgact
12721 cccgcgcctc gccccacccc tggggtaccc acgtttccga cagcgccggg ctggtttatc

12781 cccgaatccc gatggacatt gccggcggca ccgacctggt tgacgccggg gtcgcgtgcg
12841 tgaagcacgg cagaataatt gacgaccgtg gcggccagga tcaagacgat ttgattacgg
12901 tatttcatcg gtccctcctc tctccgaatt ttccaacgct gcccgcatag cgtgcgctgg
12961 gctacgaagc gcaccggcaa tattcacgcc gccttcctga cgaagtcaac atgcgcggtt
13021 tcgtacggag tcgtaaggaa aaggaccttg cgtgaactaa gccattggct tacattgagg
13081 ctatgcttac cacggttacc gaactgccgg agtacattcg atgtgcgaac gatcttctgg
13141 atgaggctga tcgtaaggcc gtgatcgatt acctggcggc tcatcctcgc gccggggatg
13201 tgatggaggg cacgggcgga atccgcaagc tgcgctgggg gcgaggtaat cggggcaaga
13261 gcggcggggt ccgggtgatt tattactacc acgacgaacg gctgccgctg ttcttgctga
13321 cggtgttcgg taagaacgaa caggcgaacc ttacgaaagc agagcggaac agcctcgcga
13381 agctgataga cgtgctggtg acaacggcat tggagaagaa acgatgagca gggcttttga
13441 aagtatcgcg caggggctga aggaggctat tgcgctgacg gagggacaac cggttccggc
13501 gaagacgcat cgcccggatg aagtggacgt ggcggagctg cgccatagct tgggtctgac
13561 gcagatggag ttcgctgcga aattgcggat cagtgttggg acacttcggc attgggagcg
13621 gggagaccgt cagcctcacg gtccggcttt gactttgctc catgtggtgg cgaaggagcc
13681 gggggcggtg ttgcgggctt tgggttgaaa catcaaacgt agccatttcg taaccgcgtt
13741 aagcttgcaa ctccactaaa tcaatacttt acaaaacttt gttgcgtgat gcgggcgttt
13801 gtaaggcttt gatttgtaat ccaatatcta gagagtggcg gactcttaat ccgtttgtcg
13861 taggttcgat ccctacatgg cccaccagac accaagccgg agaatattct ccggcttttt
13921 tgtgcccatc tacaggccac ggctggcgcg gttcccgcat ttggcctctt gaccagccat
13981 ccccgaaaaa gccggtttcg ggccaaaata ggctaaattt ctctctctgt tctctgtcga
14041 gctcttgact gtcaagaggc cgaacccagg aaaatcaaga gttccagcgt gtagggttgc
14101 gactgtcaat tggtctgcgg tggggattga gaacagaaat ttcggtacaa cgatcaccct
14161 cggatccatc acgccccgcg cgcagcgctg gcgcgcgatc cagtggacgc cgccaacgat
14221 gccgccgacc tcctggccgg ccgctgcgaa gccgccttgt tcctggccta ctggcaggac
14281 gagggcgcgg gcctgtgaaa tggcagaacc acatcgtgat tgccggcgcg atcaccgcca
14341 cgattgaccc agtgcttgcc cccggcgcca tgctcggcgc caccgccccg gactggctgg
14401 aatggctcat caccccggtg cgcaaggtcc gccaccgcac cgtgacccat tacctggtgc
14461 tgtggatcgc cgccgtcctg ttcttcttcc tagtttggga cttccaccac ctggggcaag
14521 ccttcagcct cggcggcctg ttccacctcc tcaccgacgc catgaccatt tccggcatcc
14581 cggtcggctg gtggtccgac cgcaagttct acctgttcgg cggccggctc aaaacgggca
14641 gcgcggagga atacatgatt gccggcgtgg tgctgatgat ctgcgccctc atcgtctggc
14701 atcggccgga caaccgcttt accccgttct tcttcgactg gggcgggtat tacgagcagg
14761 gcttgattga tggcagggaa tggaaggaga atcggtttcg gtggctgtag tgtcacaagc
14821 cctcgaagga gaaatcgttg cgtcatttca ttgcgtatca caatcagcag aagatgggct
14881 actcctgtgc cgacatcccc gagcctcgtg ttcaaactag cagggaggtt gaagggcttg
14941 aaggagtaac ggtctggttg gttgctggag agggcgagaa tccaaaggcc tacttcctcg
15001 cggcaaagtt cgtcgcctcc cactgtgaaa gaaactcaac atcgggttcc aaatttcctt
15061 atctcatcgc cggtccaggc tccctttaca aaaagagcat tccgctggga ggcacatccg
15121 tgcttgccca gattcgcaaa gagtcggcaa atttcgttcg ggggttctac gaaacaaaga
15181 acccttcgat cgtgtccgcg ctcaatgcgc ttgcaccaac taagagctaa cccggatttc
15241 gtatccggca agctgctgaa ctcaccgtca ccccccacac aacccctcgc acggcactcc
15301 atcaccgtct cgatccaagc ttttgacgcc gcagcgggtg aggtaatacc gggcttcgtc
15361 gcaggtggtc atttcctggc aggtccgctt cgagccgcag gcggacgcgg caaccccgga
15421 caaaggacgt ggtgtctgag tgggttttcg ggtggggttg aaatcatccc agaacgacgg
15481 gccacggctc ttgtggcgga actcccacgg cgggaccggg ttcggctgtg accatagccc
15541 gcgccggacg gcacgcgctt cgtcttcggc gctgaagtag ccgggatcgt aggcgtattt
15601 gcggtagacc caggccatgc cggcgcgcac ttgctccagg ttcacatcag ttccaccaat
15661 cagcaccttg ccgacgctgc ggccataacg gtcggtgtcg gcgacttcga ccgtgacctt
15721 tttgccagac acccagtccg acaaggcgcg cttggaggct tgaccgtagt cctgccgctt
15781 ctccggcgcg tcgatctgcg ccagccgcac gcgggtttga acgttgccaa cggacagcag
15841 ggtgatcgtg tcgccatcga gcacactgac cacccggccg gcgagggatt cggccgcggc
15901 tgaggcggtg acggtcaggc agagccaaag gaacaagaag cgcatggaga gttcgggcgg

15961 tggtcaagga agcaccggcc gcgctacgat cggccggtag gggtgaggtg gaacgatgtg
16021 cggacgatac acgctgacga cgccagcgga caaatacaag gcgcaactga aatacgagcg
16081 ggccagggcg tttttggaaa aactgttcgc ccgctacaac atcgccccga cccaggacgt
16141 ggcggccgtg cggaccggga ccgagggggt gcgggagctg gtggcgctgc actggggctt
16201 gattccgagc tggtcgaagg cgccgaagac cgaatacagc accatcaatg cccgcgcgga
16261 gacggtggcg gagaaaccgg ccttccgctc ggccttccgg taccgccgct gtctgatcct
16321 cgccgatggc tactacgagt ggcaggcgcg gccgggctcg aaactcaagc agccctggta
16381 catccgccgg gccgacggtg agccgtttgc cttcgcggga ttgtgggagc gctgggaatc
16441 gcatctgggc gaggagggcg agccggtgga gtcgtgcagc atcatcgtga ccgacgccaa
16501 cgccttgacc cgaccgatcc atgaccgcat gccggtgatc cttgacccgg ccacctacga
16561 tgtctggctc gatccggagt gccgggacaa gaccggcctg ctctcgctgc tgcggccgtt
16621 tccgcccgac gaaatgaccg cctggaaggt cagcacccac gtcaacagtc cgagacatga
16681 cgatcccacg tgtgtggagc cggcggaacc gtccgcgctc gactcggtgg actgacccgc
16741 ccgatgccgg ctacgcccga agggcggggc gctatccctg cccattccgg ccggtgtccg
16801 tgcccaccgg cagagcgaat cggtccccgc gtccgtgcgg ggtgagtctg gagggtggtg
16861 cgggcttcct gcccgagcaa tctgaccggc cggcccgact ccgcccggcg ggcctccctg
16921 cccgccgcca ctgctctcta tggaatttga gaaccgaccc ctatgtatgt gtatgtgaat
16981 atgtgaaagc atagccatcc accgggcatt ggccgtggcg gtgaagtccg aaagcttgac
17041 ataattacgc attgtgttag ctctagtaag gccgtctgcg gcgatagcgc gcaaaggcgt
17101 ggatacgggc gaagtcgcct ggtttacgca tcctcgcaga tgcgatcgtc gagttcggag
17161 gaggccttgt ccggcccgcc cttcaactgc tcgattctat cttcaggcag ttgggtaata
17221 gccggctgcg tggtgtatag gtctgcgcat ttcgccgact gaggcactta gaagatcacg
17281 aacgtcactg gcttcgaagg gaggtctgaa tgtttcagta catcgtccgg gtgcgagaca
17341 agacatacgt ctggggatcg aacgatcttg aggttgccct gtcgttgccg atcgtgctgg
17401 tggtgaacaa cgatgcgttc ggcgatgccg tcgaaattgc ctcggaatca ccacagggcg
17461 ctccggccgt cctgggcata ctccagcccg ggcagtgctg gacgttggtg ctcaccggtc
17521 ttcgcggcgt gacggccacc tgcgagaccg acactacatt ggcgtgcgcg atcctgtccc
17581 ccagttgaac ggaaggtatg gtcaatggct acgggtagat cagtaaacct gcgggttcgc
17641 ccctaccagt cggtcgacct gtcctttccg gtcgatggtg tcatcagcaa gcaatcagac
17701 accatcctac tgggcagacc ggttctcgga attgacgtcg aacaccttta cgcgatgctc
17761 ggcgaaatgc gaaacgtcgg gttgcaggtg cacggcggcc ggctgaagtg ggactcgcgg
17821 cagatctcgg aatatttgtc caatgtcgcg ggcgtagcag cagggcagaa cggtactacg
17881 ctcctgggga agcttcggaa cgctacggaa gccgccgatc tggaccgggc gctgatgatg
17941 cggcagaacg cctatctgac gtcgtattcg cctgaggtgc tcgccgaggt gcggcgggtc
18001 tactgcggtg aaccacgcga cgactcgctg gcacgttacc ggctgctgca gaaggtcgaa
18061 gaggacatca cgaccatgca caacgggcta gccggtgcgt acaatcatcg cggctggtcg
18121 ggcaaggtca tcgaatatac acgctcggaa aacacgaaca aagcgaccca gtactccggg
18181 tccggacaca tccaattcga gggtttgacc gacaccaatt cctgggggta tgagttccgt
18241 tacccctcag cggagaacga tctgagatac caccaggcac gggcgggcat tcggcaggag
18301 attctcaacg cccagcgaat ggcagaaatg tgccggcatg gcgatgtcac tttccccaat
18361 gagatcggcg cgatagacct aagtatccgg aagctgcagg ccgcctacat cgacaccttc
18421 ctcttcgcgc cgtttaacgg gattgtcacc ggcgtattcc gtgggccagg ggattatgtg
18481 cgggccgggg aaccggtatg tcgtgtggag aactatctac tcgcctacct ggtaggcacc
18541 gtgaaatatc gtggcatgct ggggattgga acgaaggtgg atgtgagcac gacattgttc
18601 gaggccgctg gaacgacccc ggtaaaggtc tccggtgaga tcgtcgcggt gcgtggccac
18661 gacgcggtgt ccgagcagtg ggatgtgctg attttctgtg agaactacac cgccacaggg
18721 gatccggtgc tgccgctgaa ctataacttc gatttcgaaa gtaccacggt aggcgtccta
18781 ccctgatcac gaatcgccgc tgacgaacgg agttgttgtg tccgaccctt ttggctttct
18841 cgacgcaatt ttcggcggcg gcggtggcgg tggctactcc tcgacgcagg gcgggccgcg
18901 actcgatcag ggcttcgcac cggtcgcggg atggatggtc ggtcaggcat ttgccgatgc
18961 tggcccggtc gggcagtact tgggaatgct gtacggctcc ccaggcacgg gcataccacg
19021 cgtcgagtgg gggacggact acgcaagcca ggtggactac ggcgactacc tcgatttcac
19081 ccagtcgcag gtgtttgcgg actcccctga ctacagtgcc agcgtgcggg tggtggatac

19141 ctccgactcg gcgccgtcgc ggcccgccga gcctcgaact ctcgaggtac ttgatcgggg
19201 ggactccggg gcagtcccgg cggcagaacc gatagaagtg gtgcatgtct ttggcacccc
19261 gccggatcgc cccagcaaga acgactggtt cgagagtttt ctctcgacgc cgcggaacaa
19321 cacgttctcc ggaggggaga tcaagctttg ggtggacaat ccgctaccgc gtccaccgcg
19381 ccccccgcga ccgcaacgct ccgctggccg caaagggtcg cctgtggaaa ctaccagccc
19441 gctcccggaa ctgccgccac ctgagcgcga cccagtggac agcgggccgt acttcaatgt
19501 cgaggggacg ccgctgaccg tccctggcag cgaccaggcc accaacccgt ggatgtcaga
19561 cgctgccgtt ccggagcggt tcctgccctg gggatcgtgg gaccgccccg agaacaagcc
19621 ctcgccgcgt tgggagcggg gagacaccgg caggatcgaa gccgtcccta ccgtaccggt
19681 cgaacgcagt ttctggagcc gtggcggcac cgggctggcc gcgggcgcag tgaccgccgg
19741 cctcggcgtg gccgcgttga tgtggtggaa tccggtcggc tgggttgcgc tcgggacggc
19801 attggcgatc gccggcggcg tggcagcaac gaccgccagt gcggtcgagc ttaccgcatc
19861 ctacagcggc gccacatccc tggagcagga tgctgagatg aaccgggcga tctcggcaac
19921 cctcgggtac agctcgcccg gcggagtcat cgggagtgtg ttgggcaccg ttgtcgccga
19981 tgaccctcag gaggggtttg cacagggcgc cctgtggggg gggctgaccg aaggcgtgac
20041 cagtctcccc ggcgcactgc gcgccgtgcc tggactgtgg cgcgcagccc tgccctggac
20101 gaaatccctc ctgctaacgc cattctggtt tttcatgtca gcgggcggcg gtggaggccg
20161 tgcccgctcg ttggcccggg tgttcgccgc gcagggccgg atcgcatccc gggttcgcag
20221 tgttgagtac ctgggtacga cgccgctgct ggagcgcgac gccgactggg ctcgattcca
20281 ggtctttgca acccgaaccc gcaacgagtc cgtcttccga attacctatg cgaacggcca
20341 gcagcggatc gtgctcgccg accgcgccca gccgaccggc cgggcaatct tggaagccaa
20401 atacggcgac atgggccaga tgtggaaccc gacccgtgag gcccacatca tcggccaagc
20461 caacaactac ctcgacatag ccacggtgac cggcggcagg gtcggctacc tcgtcagcac
20521 cgaacgcggc gcgtatcgac tcacgcagcg attcgggctg gagttcccgg cggagatggc
20581 gtcggggcag ctgtggatcg actgggtgcc gtggcggcgg tagacgccca cggtctgcat
20641 ccgaaatagg ttctgcaacc acgcccgtca gcaatgtttg cgtaggttcc acgtaattca
20701 aaccgcctcc gtcagccagt ctttaatttg tatcatttgc gcccgctacc gcgctctcta
20761 atgctatacc gtacctgagc tgtaagcgga ctgcgtcctt gcaacgcgcg caatcctacc
20821 tttgggcttc cctgcaatcc atcccgacaa ggtgccggcg gcgtccgtgc cgtctgagct
20881 tgtgctaatc gcccaccggc gagcggaggc ggcgggtgct tgtgccccgc cggtccggtc
20941 tacgcaaggg gcgcgggctg tcgtgcccgc ctagtctgac cggccggccc ggctgcgccc
21001 agcgggccag aagggcgcta tccttgccta aacccgaccg gcttccctgc ccgccgcgca
21061 atctttcatt tttgatcccc ccaagccccc acggggggat gtcggcatat ccatgtgcca
21121 ctaacggtgc atgggtcccc aaaacaaaaa aaccgaaaag agagaaaaag cctcggttgt
21181 tgccagggct gcgcgcgcaa ggggtacgct tcgccagtcg ggggcggcgg ttctgtgccg
21241 cgcatgacga tcctgcgtgc ctggattcaa tcggtggctc ttcggggtga gttgacggcg
21301 atcgacccgc gaagcttgcc aaacaatggg catattgatt accgtacgga aatcataagc
21361 agcgactcgg tcttccccat gacttcgatt cctcgcggcg gactccgccc cggcgccggc
21421 cgcccgcgtg gcaccggcgc cttcggcgaa ccgaccaccc ctgtacgcat ccccgaaagc
21481 cgcttggccg aggtgcggac gctcattgct cgccctcgcc ggcctgccgc accggaactt
21541 gaatggacag gtgaaccgga cgccagcgcg ccgggcttga gtcttccgct gttcgcctcc
21601 cgcatccagg ccgggtttcc gtctccggcg gatgattacg tcgaaggcaa gctggacctc
21661 aaccagcacc tgatccgcaa tccgcccgcc accttcttcc tgcgggtgca gggtgaatcc
21721 atgctggggg ctgggattca tcccggcgat ctgctagtgg tggatcgctc gatcgagccc
21781 aagcacgggg cggtggtcat cgcggtggtg gatggcgagc tgacggtcaa gcgcttgtgg
21841 ctggcgacac cggagcgggt cgagctgcgc gccgagaacc cggcctatgc gccgatcgtg
21901 atcggcgaat tccagcagtt cgagctgtgg ggcgtggtca ccagcgtgat tcatccgctg
21961 ctgccatgag cgttttcgcc ctggtcgact gcaacaactt ctacgtctcc tgcgagcggg
22021 tgttcgatcc gaagctggaa ggccggccag tggtggtgct gtccaacaat gacggatgcg
22081 tggtcgctcg atcgcccgag gtgaaggcgc tcggcgtgcc gatgggtgcg ccgtggttcc
22141 aatatcagac cctggcccgc aagcagcgca tcgtggcgct gtccagcaat tacgccctgt
22201 acgccgacct gtccgaccgg gtcatgcgca tcctggccga tctcagcccg gcccatgagg
22261 tgtattccat cgacgaatgt ttctgcgact ggcgcggcct ggccggcgtg gatctgatcg

22321 agtacggcca acggacccgg cagcgcatcc gccagtgggt gggcctaccc gtatgtgtcg
22381 gactcggtcc gaccaagacc ctcgccaagc tagctaacca tgtggcgaag aagcagccgc
22441 agcatgacag cgtgttcgac atcgcccggc tgaccgaaac cgagcgcacg gagctgctcg
22501 accgcattgc cgtcggcgaa gtctggggag tcggtcgccg gatcgaagaa cgcttgcggg
22561 aacaaggcat caccacagta cgggcgctgc gcgatgccga tactcgcatg atccgcgccc
22621 gttacggcgt ggtgttggaa cgcaccgtgc gcgaactgcg cggcagctcc tgtctgccgc
22681 tggaagccgt cacgcccccg cgaaaacaaa tcctctgctc gcgttccttc ggccaacggg
22741 tggaagacct cgactcactg cgccaggccg tcaccgccta cactgcgcgg gccgccgaga
22801 aattgcgccg gcagggctcg agtgccgggg ccatccaggt attcatcgaa accaaccgct
22861 tcaacgatga gccccgctac gccgcccagc gcaccatccc gctcgccacg cccacggcag
22921 acacgcaccg cctcctgcat gccgcccacc acgccctggt ggacatctac cgccccggtt
22981 accgctacca aaaggccggg gtcatcctgc tcgacctcga ccccgcagaa aacgcccagc
23041 gcgattgctt cgccagcctc gccgccgacg caatcgaccc tgcccgcgcc aaactcatgt
23101 ccgccatcga cgccatcaac cgcaaccacg gccgcggcgc cctgcgctgg gccgccgaag
23161 gctacacccg cccgtgggcg atgcgcaccg accggcggac gcagggctac actacgcgtt
23221 ggaacgagtt ggcgacggcg agggcttgag gccaatggat cagctcacaa aaatcatcat
23281 tcagtggagc aaaagtggca aaaaaaggct tatcgatgga ttcgttctgt acctgggatg
23341 gcgaaacctt agtgctgaat attctcggac aaccgagcgc aaagcaggat gcgattggca
23401 aagccaaagg caatcagctc aaagtaagcg tgactgccgc gccggtagga ggcaaagcga
23461 ccgaccatat ggttcgcttc ttggcaaagg agttcggagt agcaactcag gatatcgaag
23521 ttgtttttgg ccggtacaac gtcaataagc agctacgcat caagtctccc aagaaactac
23581 cgtctgtaat cgacaagcaa ctgccctgat agacggcacc taggccgcat aaggttcagc
23641 cgagcccgga ttacgatgag cgccttgttt caaccactga gcaagaccga aaacaggtcg
23701 gttcggctag ccggtttggg ccaaataccg ttaatctggg cgcgctaaaa tctctaccct
23761 ctgcgcttgc cagtccagcg agaaaggact ccggcaccga ctctccgaca cccgcccgcc
23821 tcccttcccc gccaagcaag cctctacgat ctccggcgcc aactgcacca gccgccccac
23881 cctgcttgcc tggctcaggt cgaggccttc ggcattggcg atttcactga gcgattcgaa
23941 ctttccctcg tccaacaacc gctgccagaa gtgggccaat ccaagggcgc ggatgagcgg
24001 cgtgtcctgc tcggctctgc gcttttcgac ttcctgcctg gcttcttctc ggaatgcggc
24061 cggcgtgccc agtggcgtga taacctcccg cttcacccca cgcttgacca atgtccaggg
24121 gaggaaggtt tcgagcctca ctccgtccgc ggggcttggg atcgtcgttg taactggacg
24181 gccggtgatc cggccttggc tttttcgggg catgggatgc gtctcactcg aacctgtcga
24241 tggtctcacg ctgggcttcc cagtccagcg gcagtcgatg gcgtttcagc catcgttggg
24301 tcagcgtccg tggctggtgc cctgccagac agcgctccac ttgctccggc gccaacaggg
24361 ccagccggag cacgtgcgat acgctcggcg gcgtcagtcc ttcccgttcc gccaactcgg
24421 cgcaactgtg cacgacaccg gtgtccagca ggtgctgcca gtacatggct cggccgatcg
24481 agatgagcag gccggggtcg tgagccgttc gctgagtgtt caccaggtgc cactgccgcc
24541 gccgcaagca aaacggtacc agggtttcgg ggttgtccat caggccaccg cctccatttc
24601 tatcagttct gccccgatcg ttttaggcgt gaactcaccc agcagcgcct tccaacccag
24661 cgcccgccag cggatcctga gaccaccctc ggcgagctcg acccgctcga tcatgagctg
24721 gacgatgcgg tgccgttcgg ctgggaacag ctgttcccag acgctgccga gctggcccac
24781 ggcaaccaca acctgggctt cgtcgaggtc ggccgtttgc gcctgcaccg ccagccatat
24841 agccgcaatg gcttcgggac tggccaggac ggtccggatc tgggcgaggg tggccgcttc
24901 gatttcttcc gcaggcagcc ggacgctggt cttgtggccg gcgccgaaac gcgcttcggc
24961 cttggagaag taatagcggt attggcggcc gtttttccgc acgtaggtcg ggtacatctt
25021 ttcgccctcg gtgtcgtaga gcaggccgcg cagcagcgcg tcggttcgct cccgctgccg
25081 ggtttcggtg gcacggctgc gggcatcccg ggccaggact gcgtgaacag cgtcccacaa
25141 agtggcttcg ataattgccg ggtgggcgcc gggatgccaa ctgcccttgt gggagatttc
25201 gccgagatag atccggttgc gcagcaggtg gtgcaggtgt ttcttgtcga tccgggcgcc
25261 ggggcggtgc ctgccgtcct gggtggtcca ggccttggtg gtgatgcctt cggcattcag
25321 cgccgcggcg atccgggtgg tcgaaccgac gctgagcatg tccttgaaga tccggcggac
25381 gatggcggcc tcgttttcgt tgaccaccag ttgccgatcc ctgacgtcat agcccaaggg
25441 cggtaccccg cccatccaca gccccttgcg cttggccgcg gcgatcttgt cgcggatgcg

25501 ctcgccggtg acttcgcgtt cgaactgggc gaaggacaac aacacgttca gcatcagccg
25561 tcccatcgag gtggtggtgt tgaactgctg ggtgaccgag acaaaggaca cgccctggcg
25621 ctcgaacact tcgaccatgc gggagaaatc cgccaggctc cgggtcagcc ggtcgatctt
25681 gtacaccacc acgatgtcga tccggccggc ctcgatgtcg gacagcagac gcttgagccc
25741 cggccgctcg gtgttgccgc cggagtagcc gggatcgtcg tagtcgtcag cgaccgggat
25801 ccagccttcg atccgctggc tggcgatgta ggcgtggccg gcttccttct gggcgtcgat
25861 ggagttgaac tcctggtcca accgttcatc ggaggacacc cggcagtaga cggcacagcg
25921 ttggcggggc ttggacgggg cgccggtttg cacttccata cggttcgatg ccctcatgtc
25981 ccctccccgg cttggcgcag gccgaagaag agcgggccgg accaggcggt gccggtgatg
26041 tggcgcgcta ccgcggtcag gctcttgaac gattgcccgg catagtcgaa ctgcccttcg
26101 gcagtgacag tgaccttgtg gtcctgctcg ccccattccc gcaccagcac cgtgccgggc
26161 ggcaggtgaa tcgctggcga cacccgccgg gacttgatct tcgagtggcg ctggccgatc
26221 agctcgaggc gccggcgggt ctcgggcgcg aggccgccgt aggcggcttc ctgaatcttg
26281 taggcgatgc gggattccag gtagttccgg ttgggatggg tggggcggcg cgggaaatac
26341 ttgtcccaca gcgcccagag gtctttgatc ggcaggttcg agagcgaggc cacttgggcc
26401 gccaccgaga gtttcaagtc gttcattaca cacgctccgg ttgagaggga gtggtatgga
26461 cgcgcttccc ggccagtaag ccaaggtcca acccggtctt tttctcggat tctttctgga
26521 gcgtgcggac aatcgcagcg gccaggatcg tagtgatctc gtgggcgcgt tgggcggggg
26581 agagacgttc gggcgacggg aaggatggca tcgtcatcgg caactcaaca atcatttggc
26641 acgaaaggga tgcctaccag tctccgtcac aacccgatcg ccgcaccatc ccgagatttc
26701 gaaggtctac gggtcgttgc aggccattgc aaaaactagt cgaatttttc ggctcggaaa
26761 ccgctgtgga gggtgctttc gacgacctgg cagcccaggt ttcgacacca gaacttgatg
26821 tgataaaaaa cctgctatcg cctgaagcgt ccctggcgga tggctgaatc catccgctcg
26881 gccggatcgt cgtcctcctc ctcttcacgc tgccagcgcg gctcggcctt cggcatcagc
26941 aggagagcga gcgtgtagtc gtagtgctcg ttgacgaagg tcaattctgt caatggcatg
27001 tccctgggct ccttgggaaa ccaggtcttg gccgagaggc tcccttcctg aatcggaatg
27061 ccgccattgt ggccatgggc cagcgactcg aggggaacgg cataggtgcg cttgcgagtg
27121 gcgaataccg cacccgactt gaaggctgcc tgatttgagc gcgcccacaa caaatgatca
27181 tcacggacgg cgacgagcac cgcccggttt tcggcgatct cgatccattt caaggcagcc
27241 gccgtcagcg aaacgccgta gcgctcggcg cagtgggcca gcaactcgaa ggaaatacgc
27301 tccttgccga tctgctgccg gaagtcgtcc aaaggcatca ggaggtaaga cgcgaacgtg
27361 tccgcttcgg tttcgagctg tcgctcttcg gcatcccatt cctccatgtc ctgttggctg
27421 cagctgaaga gctcctgccg gtcgcggtgc agcaggtaat ggccgaattc atgagccagc
27481 gtaaaacgga tccgccccct ggaccggact gccgagttgt agacgatttt ccattgggtc
27541 ttgctgggat gggccgctag catgccttcg aacccaggta gatcggcgcc cttgatctgg
27601 gtgatcggtg cgtgcgggaa gcactgtttc gaatactcca gcgccagtgc ctccacatcg
27661 accggaaacc gttcgcgccc cagtacctgc ccgagcatca cgttgagccg attggcctcg
27721 gccgttggcc acttgcgctc ggtcatggat cgtcgtccca gacatcgatg agttgccgca
27781 gcttcttctt ggtttcctcc ggcatcttct tgtacttccg gaaaaaggcc tcgtccgcga
27841 cggccgcgtc gggcgtcatt tcctggtcat tcatcaagaa ctccgtcgtg acctccaaca
27901 cggcggcgat tctggcgatc ttttctgcag acgggttggg agcctccttg ttttcgagct
27961 cccaaagata gctcttgctg gattcggtta ggtcggcaag ctgatccagg ctcagtttct
28021 tctgcttgcg caacccccgg attttctcac ccaagggcga tggcacggtg tttctcctct
28081 cgtcaggtca attcatgtca ccgcaggcga ccttaggaat atagcaccgt gccgaacgaa
28141 ttcgtaccgg cttgacagaa cattcagcag cccgcaataa ttcgcatcgt tcggtgtacc
28201 gaacctatcc gtcctattgg gtcagtgttc gttgctcagg ccgctcattc ctcgtttttt
28261 cttcagtaca ggagaaatca tcttggctgc attcaatctt cgtcacctca tccgggagat
28321 ccccgggaaa acctggcgcg actatttggc aaagcggtct atcgaggtcg gtgacgattt
28381 cgattgggag gcggaagaat ccgcgctggc aaatgccctg atcgcccgct tcgagagtct
28441 cgaggacagc gaacaacgcg cggcggtcca cgcggaaatg cgcagggttc accggctggc
28501 ccagcgcaaa ggcatctacg cgctgctcaa cgcagggcat ggacagtccg agattcagga
28561 aggtttcgaa caattgcaca accacgccga gcgcgccctt tggacctggg tgcattggcc
28621 ggcggtgttt cagatcggcg agcgtttgtt gcaattcgat ctgagtgccg gcacccgggc

28681 ctggaaacgg caggccatca aggtcagcga acccgtatct aaagaggaag agaatatccg
28741 ggctttggaa acggctttgt cggcattgat gtcgcgccgg aaaggccccc gacgggcgtg
28801 ccggatcgat gtctgcgagc ggtgcctcga tggcggcgtt caggtcaata tctacatcga
28861 ggacgacccg aacgatctgg tggaattcgt cgaggaaaac atgaaacgcc gaaccacccg
28921 gccggcgggc aatctggcgc tggtgtatta cccgatctcc gggatcgtcg acagtgtcgg
28981 tcgaggcggc gcgcggatcc atgtcaccct cgtcaccctt ttcgcaaaac acctcctcaa
29041 ccgagacgtc aagccggagg ctgtcaagca accgatgttc catctcaacc gtctgcgtta
29101 tggactcgca tttgtcgaag gcgatgccat cgatctggcg acccatggcg tggatcgtat
29161 ccggctgcgg caagtccgag tacgtgccac gatcccccca tgctgcgatt tctgggtcga
29221 gacaccagcg gatccgaacg aagcttgcgc attcacggca tccagtacgc acttgcagga
29281 gagagacttt ttccgaggcc ccttcaatct ggtcgaagtg gtgatcagcg tctacttcgt
29341 cccgagtgaa ccgggaaaaa tcggccatgt cctgaacatc gttctgaaac agtccggcgt
29401 gtccaacctg cgcgacctga gcgagagcga cgcacaactc gccgacgccc tgctacgcgc
29461 gtggcaggtc accgaaccga gcgaattcga agccaagctg gccgcctagg tgaccgatgc
29521 cggcctgcaa cgacatcgcc ttcggcctgc tttgcgagct gatcgaggcg ccccatgcgg
29581 atatccaccc cgataccgtc cgatgttccg gtaggcgcga agcctacgag cacctgcacg
29641 aactccaagc cctgcaagtg ggtacgggcc ttgcgggttc cgttctctgc ccttggtgtg
29701 gagacgacga actgtgttcg ctcagtttct ccgaagaagg ctatcgcggc tattgcagcg
29761 attgtggctg gctgaatctt gccacccacc aggtgaaacc cctgcgggtc gagtatcaac
29821 gcatcgtccg ctggatcgcc tccgcgctcg gcctgcaggg acgctttcga ctcgaggaac
29881 gcgtaccggc ccggctttgg cgtttgggtg acatcgaaca tcggcggaaa cgccgcacgg
29941 tgtttttcgg caggcggctg aacgagtcga gcgatgcgca aacgatcgac gccggcatcc
30001 gtgccgtcgc cgcgccgggg accgagatcc tgattacgac caccccgcag gattcgattg
30061 cgccaccgct cgcgaacggt cggctcgtac cccttcgcgc cgtgtcacat ctgcgcaaag
30121 ccggcttcgt cgtcgaaaac ttggagtcct atcttgacgc gcccctgatt gctgatgagg
30181 acgttgccga gacttcgttg cggctgctgc attccggccg gctcgccttg atcgacggca
30241 aacagatcaa agtctcaccc cagatctacc gatttctcag cattttgatc gacgcagacg
30301 gcaagcccgt gcacaaaaga gtgctcgccg atgcgctaga aatggatgtc gacgcctgca
30361 aaggttccga gatcttcaag cggcacaaag cggtctaccg aaccttcatc gaacatgaca
30421 ctgagggccg ctactggatc aaacccgagt tcctgagccg acgaggaggt gaatgaccgt
30481 aatcatcctt attcctattt tcccgacata ttccacaacg tactcaacat gaggtaaacc
30541 gatgagcaag aaccaatggg tcgtcaagaa cggcgacggc tggggcgtcc ggggcgaagg
30601 caatagccga ttgacctcca agcacgacac ccaacacgaa gccatcgagc gcgcacgaga
30661 catcgcgcgc aatcagagca gtgaagtcat catccaaggc gaggatggga agatccgcga
30721 gcgcaacagc ttcggcaacg atccttttcc cccacccgga tgatttcgcc gtaatcgcat
30781 tatcgatcga accggcccgg agcatccctc cgggcttttt cgtttctgtc gcctgaaatt
30841 cgaattgcag gttttgccca cccctgccgc ccgttttgcc ccccgctttg tccaccccct
30901 gacgcccaga atttgttcac gtttccgcaa caacccacag gagcgactcg tgaacgtaaa
30961 acatctgaat caacgccaac tggcggatcg ttggggcgtc agcgaagcca cgctggaacg
31021 ctggcggtcc gaaggcatcg gcccggtcta tctgaagctg cacggccggg tgctctatcg
31081 tcaggaagac atcgaggcct acgagacccg ccacctccgc agaagtcccg gtcaatgcgt
31141 tcaggccgga ggtgcggcat gacctcgatg aaccccgaca tcctcctcgc cactccggcc
31201 ggccagctcg ccgagttgcc ggcggaatcg ctgtgccggt tcaagctcga cgccgacgcg
31261 caactggccg ccgccaagac cctcaacgaa cacttggacc gcgccctcga gatcagatac
31321 gccgagcgcg cccgccaact gcggctggct acgggcaaag acaccggcat cgtccacttc
31381 gacgacggcc cggtgcgggt caccgccgac ctccccaaga aggtcgagtg ggatgcccga
31441 cagctcgccg ccctggtgcg acggatcgcc gacagcggcg aggaccctgc ccaatacgtc
31501 gagatcggct accgggtatc cgagaccaag ttcaacgctt ggccggccgg cctccagcag
31561 agcttcgcac cggcccgtac gctcaggacc ggcaagcccg gcttccgttt ggcgctgctc
31621 ggggaggacg cggtatgact ctccccatca tcagcgccga ccagcgcttg gcggaacggc
31681 gcggcgtcaa gggcgtcctg gtcggcaaag cgggcctcgg caagacctcg caactgtgga
31741 cgctggaagc cgcctcgacc ctgttcttcg acctggaagc cggcgacctc gcggtcgaag
31801 gctgggccgg cgatacgatc cggccgcgca cctggcccga atgccgcgac ttcgcggtct

31861 tcatcggcgg ccccaatccg gccttgcggg atgaccagcc cttcagccca gcccacttcg
31921 acgcggtgtg cgcgcgcttc ggcgaccccg gtgcgctgga acgataccag accgtgttcg
31981 tcgactcgat caccgtcgcc gggcgcttgt gcctgcagtg gtgcaagggg cagccgcagg
32041 cctactccga gaagaccggc aagccggaca gccgcggggc atacggcctg atgggccagg
32101 agatgatcgg ctggctgacg catttgcagc acacccgcaa taagaacgtc tggttcgtcg
32161 ggatcctgga cgagaagctc gacgacttca atcgccgggt gttcgcgctg cagatcgacg
32221 gcgccaagac cgggctcgaa ctgccgggga tcgtcgatga agtggtcacc ctggccgaac
32281 tgcccgcgga cgatggcagc cgccaccggg cgttcgtctg ccagacactg aacccttggg
32341 gctatcccgc caaggaccgc tccggccggc tcgatccgat cgaaccgccg cacctcggcc
32401 agttgatgca gaagatcgcc ggccccgccc gcccgctagc ggaacggctc gatttcagcc
32461 gtcctgcccc cgcaacgatc cccacccagg agacctgacc atgagctatt tcgatttcaa
32521 ttccgccgag caggcgagct tcgagctgat cccgaaggat accctggtgc ggctccgcct
32581 caccctcaag ccgggcgggt tcgacgatgc cagccagggc tggaccggcg gttgggccac
32641 ccgcagtcct gagaccggcg ccgtctatct cgcatgcgaa ggcgtggtgt tggatggccc
32701 ttacgcccgc cgcaagctgt ggtggaacat cggcctgcac tcacccaagg ggccgacctg
32761 gcaggcgatg ggccgcagtt tcattcggtc gctgctgaac agcgcccgcc gcatccatcc
32821 cgccgacatc ggcccccagg cccagaacgc ccgccgcatc gccggcttcg ccgagttgga
32881 cgggctggaa tttgccggcc gcatcgacat cgagaaggac ggccgcggca atgaccgcaa
32941 caccatccgg gcggtcatcg agcctgatca caaggactat gcccagctca tgggccagca
33001 cttcgccccg ccggtcccaa cttcgccttc gggtgcgata ccggcagcgg cagcctatgc
33061 ggcttcgacg ccggctacat ccccaggcgt ccccggcggc aagccggcct gggcccagta
33121 agggaggaca tcgccatgat gcttcgtccc cgtcagaccc tgctggtgga gcgctcgctc
33181 gcggcgctgc atcagcacgg caacaccttg gccatcggtc ccaccggctc gggcaagacc
33241 atcatgctgt cggcggtggc cggcagcgtg ctggaggagc cggacgccaa ggcctgcatc
33301 ctcgcccacc gcgacgaact caccgcccag aaccgggaga agttcggccg cgtcaatccg
33361 ggactgacta cctcggtgtt cgacgcccag gagaaatcct gggccggcgc ggccactttc
33421 gccatggtgc aaaccttggc gcgaggccga catcttgagc agatgccgac gctcgacctt
33481 ctggtgatcg atgaagcgca ccacgccgcc tcgcccagct atcgagcggt gatcgaccgg
33541 gtacggtccc gcaatccgaa ggccctgctc tgcggcctga ccgcgacccc gaaccggggc
33601 gacggcaaag gcctgcgcga ggtgttctcc aacgtagccg accagatcac cctgggcgag
33661 atgatcgcct ccggccactt ggtgccgccc cgcaccttcg tcatcgacat cggtgtacag
33721 gcagccttgc agcaggtccg ccgcaccgcg atcgacttcg acatggacga ggtggcatcg
33781 atcttcgaca agcagctggt caccgacgcg gtcatccaac actggcggga caaggcctcc
33841 gggcggaaat ccatcgtgtt ctgctcgacg gtggcacatg cccgccatgt ctgcgaggcc
33901 ttcatcgccg ctgggatcca cgcggtgctg atccatggcg acttgtccga cgccgagcgc
33961 aaggcgcgct tggccaacta tgagtccggg ccggcccaag tcgtggtcaa cgtggcagtg
34021 ctgacggaag gctacgacta cccaccgacc ggctgtgtgg tgctgttgcg gccaagctcc
34081 caccagtcca ccttcatcca gatggtgggc cggggcctgc gcacggtgga cccggaagtt
34141 tttcccggtg ccatcaagac cgactgcatc gtgctggatt tcggcaccgc cagcctgatg
34201 cacggcaccc tcgaacagac ggtcgatctc gacggccatc tgggccaggg cgaggcgccg
34261 accaaagact gccccgaatg cggcgcgatc gttccggcag cctgtctgga gtgtccgctg
34321 tgcggccacc tttgggagcg gcagcctaaa gaccgcggag tgctcagcga tttcgtcatg
34381 agcgagatcg atctgctgaa acgctcgcac ttccgctggt gcgacctgtt cggtagtgac
34441 gacgccctga tggccgccgg cttcagtgcc tggggcgggg tgttcttcct gaccggccgt
34501 tggtacgcgg tgggcggcgg caagggtctg cccacccatc tcctcggcgt aggcgaacgc
34561 accgtctgca tggccaaggc cgacgattgg ctgaacgagc acgagtccgt cgacaccgcc
34621 cacaagaccc gtcgctggct caacgaaccg cccaccgaga aacaactccg ctacctgcca
34681 ccccagtggc aaagcgactt cggattgtcg cgctaccaag cctccgcgct gctttcgttc
34741 cagttcaaca agcgggccat tcaatcctta gtgctgggcg cagatgccgc gacccacgat
34801 acccaacgga gggcggcttg acaaatgatc aggacagaat atttgcacag ccgcgcagcg
34861 gctgcccgaa gggtggcgta cagggatgca cgccatgatt tgcgctatct gcggaagaga
34921 aggccggggc ttttgctggg catcgtccca cgacgctccc cacgactcag gcgacaagcg
34981 actgttcaag cgcttctgct cgaggcgctg ccaggacatc catctgcaac gactgaaacg

35041 gagcgacggc gtcgtgatcg atcccaccca taacgagaag gcggcgatgg cggccgtgct
35101 gccccgcctc ggcgactacg tcgcggcggt cggcatggac cggccgctgt cggcctacag
35161 ccgggcggaa atcctgcaac tggtcgacgt ggtgctgacc gcctatttcg accacctgcg
35221 ggaacacgct cccgatgacg tgccgttctg agggggcgac catgctggat tacaaccctg
35281 gcgccttgtt caacgaacgt ctcacggcgc tgatcgatgc cggtctgcag catcgccagg
35341 ccggtgagcc acgccgcact tatctcggcg cgtctcgcct cggcgtggcc tgcgagcgcg
35401 cgcttcagta cgaatacgcc caggcgccgg tcgatcccgg ccgtgagttt tcaggccgga
35461 tgctgcgcat cttcgaacgt ggccaccgca tggaggaggc ggtggtgggc tggctgcgcg
35521 cagcgggctt cggcctccgc actcatcagg ccgacggcga gcagttcggg ttctcggcgc
35581 tggacggccg cctccagggc catgtcgacg gcgtcatcgt cggcgggccc gaaggcttcg
35641 acaccccggc tttgtgggag aacaaatgcc tgggcgcgaa atcctggcgc gagctggtga
35701 agcaccgcct ggccaaagcc aagccggtct acgcagccca gatcgcggta taccaggcct
35761 acctcgcact gcacgaacag ccggcgctgt tcaccgccgt caacgcggat tcgatggaga
35821 tctacgccga gcgcgtcccc ttcgacggcg ggctggcgca gcggatgtcg gatcgggccg
35881 tgcgtgtcat tactgcgacc gaagccggcg agctgttgcc gcggggtttt gccgatccgg
35941 cccacatcga gtgccggcaa tgtgcttggg gcctgcgctg ccggggaggc ggggcatgaa
36001 gacccggcag ccttccgatg acaaacgggc gcagcggcag ccgcccaggc ctctggtgcg
36061 tctgacggtc atcgacaaac tgctgcgccg gcatatcgtg ttcgcctgcc ccgaagccca
36121 cctggcagtg gcggtgatca ccctggccat cggcgactgc atcgatccgg acgacgccct
36181 ccgcgccgag gcgcggtact tcctcgccgg cccggccctg gagttctggt gcgatgcggt
36241 cggcctggaa ccggcgttcg tgcgtgcgat tgcgcacaag ggcgggtatc tgccgagcga
36301 gacggcgcac ggagtcggag tgaagctcac gccgaaggag ctaggacttg cttgacttca
36361 acgacgcgct gcccaggccg gccaaccccg ccgatctgac gctccagcgc gaagccgtgc
36421 gcgccgatct gctggcccgg ttgccgtcgg tgctgaagac cttgttcccg gccggcaagg
36481 tgcgcggggg tgccttcgcg atcggcaacg tccagggcga tccgggcgac agcctggaag
36541 tggcgctttc gggcgaaaag gccgggttgt ggtatgacca tgcgaccggc gagggcgggg
36601 atctcttcgc cttgatcgcc gctgtccacg gcctggagac gcacggccaa ttcgccgagg
36661 tgatggctct ggccggtcag ctgttgggta cggcgcgggt cgaaccgaca ccgctgcggg
36721 agaaagcccc ggtggatcgg ctcggccccg ccacggcgaa gtgggactac gtcgcggcgg
36781 acggcacgct gatcgcctgc gtctaccgct acgacccgcc caccggcaag gaattccggc
36841 cgtgggacgt gcgcgcccgc ctgtggcgcg cccccgaccc gcgtccgctc tacaacttgc
36901 cggcgatcgc ccaggcgaag gaggtggtgc tggtcgaggg cgaacggtgc gccgacgcgc
36961 tggtccatca gggtgtagcc gccaccacgg cgatgaacgg ggccaaggcg ccgatcgaca
37021 agaccgactg gtcgccgctc cggggcaagg ccgtgctgat ctggccggat cgggatgcgc
37081 cgggctggga ctatgcggag aacgccgccc gcgcttgtgt gacggctggc gcggcctcgg
37141 tcgcgatcct cgtgccgccg acggacaggc ccgacaagtg ggacgcagcc gatgccgtgt
37201 ccgagggctt cgattgcgtc gcgttcatca cccacggcga gcgccgcgtc atcggggtcg
37261 cacccccggt actgccgacg cataccttgg gcgccttgct cgatgatgac tctcccgtcc
37321 cgccggacct gatcgcgccg cgcgtactca cgcccggcgg cctcttggtg ttcggcgggg
37381 cgcccaaggt cggcaagagc gacttcctgc tggcctggct gatgcacatg gcggcggggg
37441 ccagcttcct cggcatgaca ccgccgcgtc ccttgcgggt gttctatctg caggcggagg
37501 tgcagtacca ctacctgcgg gaacgggtga agggaatccc cttaccgtcg caccggatag
37561 ccgaggcgcg ggtgaacttc gtcgccaccc cgcaactgcg tctggtcctg gacgaggccg
37621 gcctggccca ggtgatcccg gccattgttc aggcgttcgg caacactccg cccgatctga
37681 tcgccatcga tccgatccgc aatgtgttcg acggcggcga ggccgggggc gagaacgata
37741 acggcgcgat gctgttcttc ctgtcgcagc gggtggaaca gctgcgcacg gcagtcaacc
37801 cggacgccgg cctcgtgctg gtgcaccaca ccaagaagct cggcaagaaa cccttcgagg
37861 aggatccgtt ccaggccctg gccggcgccg gcagcctcag gggctactac agcaccggga
37921 tgctgctgtt ccggccggac gagacccaaa ccacccgcca actcatcttt gagctgcgca
37981 atggcgcggc gctcccggtg aagtccgtcg acaagttcaa cggtacttgg cgcgaggtgg
38041 tgaacaccgg gcgacgggtc ctgcaggact acgccgaacg ccttgatgcc gagcgccggc
38101 gcaagcatga cgtgatcctg cagatactgt tcgacgaggc ggcggatggg cgctgctaca
38161 gcgccaccca gttcgccgaa gccttcgagg gtcgcgccgg attgggcgcg gaacgcacga

38221 tccgcgagcg gctctcggtg ctcgccactc aaggttacat caagttcttc cgtaacgccg
38281 aggactacgg acttcccccg ttacaccgca gcaagttcgg ctaccaatgc gtcgagggca
38341 tggtgctacg gcgggaagat cccccggatc ctgacaccgg agaaatcccg atgtggccgg
38401 tttcggtcta cccctcccac tacaaatgcc cccagaccgg cgccttgctg ccggtcgaag
38461 acgccacgat ctggctctac cacgacgagg accatccatg acaacgacct tgattccaga
38521 tggcggacgc tctgccatct gccccttgaa atctgccatc tggatccaga cggcaaacgt
38581 ccgccatctg ggaatccata tacttcaatc gcttacggtc cagatggcgg aatttccaga
38641 tggcagcgcc gccaactcgc catctggcgc aaccgattgt ttatacgaaa caatttatgg
38701 aattccagat ggcggagact ccctccccct acgggggaga ggcgcgctac gcgccctcta
38761 cccgtcccgt aggggcctct ctgggctagg agataacgac gaaggtgacc ggaccacgct
38821 gctggccctc gatctgggta ctcacaccgg ctgggctctg catcagcggg acggtacggt
38881 catcagcggc tccgaaacct tcaagcccca acgcttcgaa ggcggcggga tgcgcttcct
38941 gcgtttcaag cgctggctga ccgaaatcaa gcaagccgcc ggcgatctgg atgcggtcta
39001 tttcgaggag gtccgccgcc atgccggcgt cgatgccgcc cacgcctatg gcgggttcct
39061 ggcccacctc accgcttggt gcgagcacca ccaaattccc taccaggggg taccggtggg
39121 aatgatcaag aaacacgcga ccggccaggg caacgccaac aaggccgcca tgatcgctgc
39181 gatgcggtca ctgggcttcg atccggtgga cgacaacgaa gccgacgccc tggctttgct
39241 gcactgggca cgggtgaccc aaggggggggg cgtatgagcg gaccgaaccc taatccccgt
39301 tgcccgctgg gaaagctgca gccgcaggtc accgacctcg aagcggtcaa gcgcgagggc
39361 tggcgcgagc agcacatcct ggtggtccat ctgaccgacg agcggctgga tttcgtcgag
39421 cgggagttga tccggcagat cggcgagcgg ctgtacggcc gccaggagtc acgccgtggc
39481 tgagtggacg atcgagcgcg tggccgcccg cttcgaggag gcggcctgga ccgcccagga
39541 cttgccgccg gtcaaggtgc aaggctattt caactgctgg ccggccatcg cccgccaagc
39601 ctgggagggc tatgccgacg agacccgcga gtgccgggtc cagccgagcc ccgacgcggt
39661 ggaccgcatg ctggaaacga tgcgctgggt gctgtggctg gaggaggaac aacgtcatct
39721 ggtctggatg cgggccgagc gacggggctg gaagatcatc gcccggcgct tcggatgctg
39781 cacccggacc gcccagcggc gctggcagca ggccttgagg caagtcgtgg accggctcaa
39841 tcactagaga tttttaggcg tggatacttt atcagccgct gatataatat atgcatggaa
39901 agggaccctg gcattgaaac cctcctggat ttacaggacc agatcattga tcagggcagc
39961 ggatattggg tcaagatcga ggcgtatcag gttgagccaa cgcccgatgt gccccatggc
40021 atccgctact cactgacact gcatgagccc tacggcaaaa ggatactcgg ttatgacaat
40081 gcccacgcag ttaaaccgcc gaagaagttc aagtatgcgg ggcgacgttt aaccttcgac
40141 cacatgcatc ggcatgccag agatccaggc gttccgtatg agttcaagaa tgcgcatcaa
40201 ttgctggctg acttcttttc tgaagtggac caagttctgc tggaggtaaa aaagcgatga
40261 aaaccattgt gataggcatc atgccgcaag aggacattcg cgagcgtctg ctggccatcg
40321 cgcggggcga gctcaagccc aaaacagggg atcccaagat atggttcact tcgatgagat
40381 cgcttgcaga ggtcctgagc gatgaaaaca gggcgctgct caaggtgatc agagaaacca
40441 aacctgaatc gatcacgtct ctcgcggcaa cgacgggcag gaagcccggc aacctttctc
40501 gcacgctgaa gacgatgtcc cattacggcc tcgtagagat gaagcgtgaa aagaatcatg
40561 tccgccccat cgtcaaggga acggagtaca ggatcgtcgc tgcctaattc gaaaacgaca
40621 gaagcactgt ttcgaatttc cttgaagtgg ctcaacctga tcgatcgcaa ggacttggaa
40681 cttcttctgt gaaccgggct gcccgagcgg ccatcagcgc cggcgatcgc ctccactgca
40741 tgcgggaact taaccgaagt cgatagcgtt tgaaggtcat cggggaacgc tgcgggcggc
40801 tgcgaaaaca gccggctttt gggctgtcgc atccgagggc cgacgggcgt agagtagcgc
40861 tcaaagtaca gcaggtgtga ccgaaggggc gagacgcccc gccgccgtga gcggttgcgg
40921 gtccttcccg gctcctgagg gctacggggg gaacgcgcgc gggatttcgc tagcgtcaga
40981 ctgcaaacca aggtttgcag ggtttgcggt ttgcaccccc agccggagcg caccaccctg
41041 gaggccgcgc cggtgctggc gctcacacgg gagcgcccaa cgcccccagt gcaaacctgg
41101 gtgcaaaccc caccctgcaa accgggtgct gccatcgagg tttgcaccct tgggcttacc
41161 tcatttccca agacccgccc cggttcgccg tcggcgggtt ttttgttgct gcgttacctc
41221 gtgtctacgg ctcgttgcag gttccgatcg gaccctgcgc cgggccgtac ttttttggga
41281 ttgctacgaa cccttcatgt tgcaggtcga ataccgtccg ctggaaaagt tgatccctta
41341 tgcccgcaat ccgcgcaccc acagcgtgga tcaggtggcg aagatcgcgg ccagcatcgt

41401 ggaattcggc tggaccaacc cgatcctggt ggacggggca caggggatca tcgccgggca
41461 cggccgcctg gcggccgccc gcagcctggg gctggcggag gtgccggtga tcgaactggg
41521 tcacctctcg ccggcccaga agcgggccta tgtgctggcc gacaaccgcc tggctctcga
41581 tgccggctgg gacgaggagt tgctggcgct ggaactggcc gagttgtccg gtgccgggtt
41641 cgacttggcg ctgacggggt ttaacgatga tgagctggaa gctctgctgt cgatcgatac
41701 cgaagattcg gatgacgccg aagaccgcga acccgagacg gccgatgatg tccccgagcc
41761 gccggcggcc cctctgtcgc gtcccggcga tgtctggcag ttgggccgac atcggttgat
41821 ctgcggcgat gcctcggacc cggacgtcgt cgcggcgttg atgggcggcg agttggcccg
41881 tctctgcttt acctcgcctc cgtatgacaa tcagcgcgac tacacctccg gcggcatcgg
41941 cgactgggac ggcctgatgc gcggcgtgtt cgcccagtta ccgctgaccg acgatgccca
42001 ggtgttggtg aatctggggc tgatccatcg cgacaacgag gtgatcccct actgggatcc
42061 ctggctgggg tggatgcgga cccagggctg gcgccgtttc ggctggtacg tctgggacca
42121 ggggccgggg atgccgggcg actgggcggg acgcttggcg ccgagcttcg agttcgtgtt
42181 ccacttcaac cgtcagagcc gcaagcccaa caaaaccgtg gcctgcaagt tcgcaggtca
42241 ggagacgcac ctccgccagg acggcagttc caccgccctg cgtggcaagg acggtgaggt
42301 cggcggttgg acccatgcgg gtcaaccgac ccaggaccgc cggatccccg attccgtcat
42361 ccgcatcatg cgccacaagg gcaagatcgg acgggacatc gaccacccgg cggtgtttcc
42421 cgtggcgctg ccggagtttg tcctgaccgc ctactcggat ccggacgatc cggtgtatga
42481 accttttggc ggttcgggga ccagcctgct ggcagcggag cgcaccgggc gccgggggta
42541 tgcggtggag atcgcgccgg ggtatgtgga tgtcgcgatc ctgcgtttcc gtcagagttt
42601 tccggccatc ccggtgacac tgctagacac cggcgaaact tgggaagtgg ttgcctccca
42661 gcggcaagcg atacagacga cggctggatg atcatcgcct gccgatcgtc ttttggaagc
42721 cgctactcgg ccaactgctc cacgctcaga cccatggcct tggcgagctt ggtcagcgtg
42781 gccttacggg gcgtactgtc gccgcgctcc agacgggcca acgccggttg ctgcatgcag
42841 gcccgttgcg cgacgtcttc ctgcgtcagc cccaagtact cacgccaagc acggatcagc
42901 ggcatatcgt gcaggacatg ggcttccacc accgcttggg gaatggcgtc acggacactg
42961 cggcgttcga gtagggggcg gacccgttcg aaatcggcga ccggcaccaa cacgaaagca
43021 ggctgtccgt catggtagat ggtctggtat tcagtaggtg ctttcatcgc gtttcttgac
43081 ctcctcgatg ctgaccacct cgatggcgtc caggacattg aacagaatcc ggtaatcgcc
43141 cacgcgcagc cggtaaccac aggcatgatt gaccagcgcc ttgacgccac ccactgccgg
43201 gaaccgctcc agcgctgtcg ttgcagtcaa tatccgctgt tgagccgcct tgtcgcccaa
43261 cttcttaagt tgcttcagag ctctcggctg ccaaacgatc gttttcatgg cgcataacaa
43321 attataacca atatccgaaa gctattgtta tattcgccag ccgctcattt ttcaaatccg
43381 agcgcctgct tcaacgccgg gagcggggtc ggttcgactt caccggcccg ggcgcgcgcc
43441 agcgccgcca gcagccgctc ggcgttccgg ggcgaacgca gcagatgcgc ggtttccatc
43501 aggctttcca gctcatcggc cgcaatcatg gcgacggccc caccggaacg gcggcggatg
43561 atgatcacct cacaatcatc gacggtgcga tccatcagcg attcgagttg ctcgcgggcc
43621 cggctgtagg tgatttcgag cgtcatggcg gtaagcactt ccggcaaaat gcattcaatg
43681 tacaacgcat gcgtcattcc tgggtagccg accgggtcga gtcctggccg gtcgacaagt
43741 tgctgcctta cgcctgcaat gcccgcaccc actcggacgc gcagatcgct cagatcgccg
43801 cctccatcgc cgagttcggc ttcaccgcgc cgatcctggc gggcgcggac ggggtgatcg
43861 tggccgggca cgggcgctgg gcggcggccc gccagctggg cctgtcccag gtgccggtga
43921 tcgtgctgga tcacttgagc cccacccagc gccgtgccct ggtgatcgcc gacaaccgca
43981 tcgccgagaa cgcaggctgg gatgagtcct tgctcaagct cgagctgacg gccctgcagg
44041 acgaagactt caatctgggc ttgaccggct tcgatgccga cgccttgctg gaccttttgg
44101 ccgacgaggc atcagtcacc gagggccaga ccgaggacga cgtggcgccc gacgtgccgg
44161 tgcaaccggt ctctcggccg ggcgatgtct ggatcctggg ctcccatcgg ctgctgtgcg
44221 gagacgccac cctggcggag cactacgacc gcctccttgc cggcgaatct gtggacatgg
44281 tgttcaccga cccgccgtac aatgtgaact acgccaactc ggccaaggac aagctgcgcg
44341 gcaagcatcg cgcgatcctg aacgacaacc tgggcgaggg cttctcggac tttctgctgg
44401 cggcgctgac tccgatgctc gcccactgca agggcgccgt ctacatcgct atgtcctcca
44461 gcgaactcga tacgctgcag tctgccttcc gtaccgccgg cggccactgg tcgacgttca
44521 tcatctgggc caagaacacg tttaccctgg gccgtgcaga ttaccagcgc cagtacgaac

44581 cgatcctgta cggttgggcc gcaggcgccc agcgccattg gtgcggcgat cgtgaccaag
44641 gcgatgtgtg gcagatcaac aagccggtga agaacgatct gcacccgacc atgaagccgg
44701 tggaacttgt ggagcgggcc ctccgcaatt ccagccggcc cggcgacgtg atcctggatc
44761 cgttcggcgg ttcgggcacc accttgatcg ccgccgaaaa gtcgggacgg gtggcgcggc
44821 tgatcgaact ggacccgaag tacgtcgatg tcatcgttcg ccgctggcag gattggacgg
44881 ggaaacaagc cacccgcgaa gcagatgggc tcgcgttcga tgccctcgtg tccgagacgg
44941 cggggggtta atcggactca gacgtcttgc cgtttgatgt cgcggtagaa attctcatgc
45001 ggaccgaagg tgagcagctt gagggtgtcc ggtgcgagga tccgataggc caacagacac
45061 agcgcattcg acaagcggaa tttgtaaacc cgaacgccgg ccaaatcgcc gaccttggcg
45121 acaccgattt ccggatcggc ggcgatggcg cgcaccacct cgtcgaggtc cagcttctgg
45181 ggccgattga gtttcttcgc cgcccgatcg aaggtgggcg taacttgtag gcgcatcagc
45241 cgaagcggta ttcgccgatg gcctcttcct catctgcgac caggatgtcg cggatcaggg
45301 taaacggcag atccgggttc tcctcggcga tcttgccgat catcgaccag tgctcgatct
45361 gcttgggcac cgaacggtgc tcgatgtgag catagcgccg ggcctgctcc accaggtgct
45421 cggacagttt gacagtgacg gacatggcgg actccggcgc aaaatcgcag tgtagcccca
45481 aaggtgccct gacggaacct tccgcatcgc gccggtgggg gtgcggggca caggtggtcc
45541 cgcgccccag ctcgatcatc cgatcttgta gatgcgatcg cccccttccg gcttatccga
45601 caccagggtg agcctgagtt tcttcttgaa ggcgccggca aacaagcccc gcacggagtg
45661 ggcctgccaa ccggtctcgg cgcagatctg cggaatcgtg gcgccttcgg gtcggtgcag
45721 catccggatg acttcggctt gcttgctgtc ctcgcggctc tttcggactc gcttcccggt
45781 ggcgttggcc tcggcggcct cggcgttgct ggcgggcatt tcggtttctg cgctgggaga
45841 tatcggcacc ggaggcgttc ggcccacggc ggcaaaacct tcggcggtga cgcaatgatc
45901 cccgccgttc aatgcgatca atcctcgctt gagcagtccg tcgaggactt tccggcgggc
45961 gccgcccttg atgttgtccg gaaaccagtc gatgcgcccc gccgtgtgct cgatcgcatg
46021 gtcgaggatc cggcgctggg tgtcggtcag ggagagtgtg ttcatgtcga tgctcctcag
46081 tgcttgggtt tggggcagcg tcgccgccgt ccctctgtca gtggacgccc gacgggatcc
46141 gccaggcgtg caattgatgt gttcaagtgt tggcggcggc ggacttggtg cgccgtccag
46201 cctcgatccc tgcctggtac gccgcctcca gcgcggcttt gaggcaccag accgccatgt
46261 cgtggaaatc gaggtgatcc gagttttggg tttctagggt atcgatcccg agatgctgct
46321 gggcgttccg ggtcagtaag gcgtcgcgtg gcgtcatggc gtagttctcg tggtggcgtg
46381 gatgacgacg ccatgaacgc gctgttccga acacaagcca agcaaaatcc acagccattc
46441 gaagcggccg atgggactct cgatccgcgc ctacgcccgc caccgcggcg tcaccgacac
46501 cgccgtgcac aaggccatcc ggaccggccg catcacgccc gaagccgatg ggagcatcga
46561 tccggacaag gccgatcggg aatgggccag gaattcggat gcgccgaagg ccgggacgcg
46621 tcaacccgcg gtacgggttg cggtgcccga cgcctcccac gacagcgggc cgtccatgcc
46681 cgccggcggc acctcgctgc tgcaggcccg aacggtcaac gaggtggtca aggcccagac
46741 caacaaggtc cgcctggcca agctcaaggg cgatctggtc gaccgcgctg cggccatcgc
46801 ccaggtgttc accctggccc gggccgagcg cgacgcctgg ctgaactggc ctgcgcggat
46861 ctcggcgccg cttgcggcgg aactgggtat cgatccgcac accctgcacg tcgccctgga
46921 gacggccgtg cgagaacacc tgcaggaact gggcgaactg cggccccgaa tcgactgaat
46981 gccgaacgac gatgacggcg cggacgcgat tgcccgcgcc tggcgcgacg ggctgacgcc
47041 ggatccgctg ctgaccgtgt ccgagtgggc cgaccgccac cgggtgctgt ccagcaaggc
47101 atcaagcgaa ccgggccgct ggcgcaccgc ccggacgcct tacctgcgcg agatcatgga
47161 ctgcctatcg ccggcctcgc cggtggagcg ggtggtgttc atgaaagggg cccaactcgg
47221 cgcgaccgag atgggctcga actggatcgg tttttgtgtc catcacgcgc cgggcccgat
47281 gatggcggtg tggccgacgg tggagatggc caagcgcaac tcccgccagc gcatcgaccc
47341 gctgatcgag gaatcgccgg ctttggccgc gctgatagcg ccggcccgca gccgcgattc
47401 cggcaacacc atgctggcca aggagttccg tggcggggtc ctggtgatga ccggcgccaa
47461 cagcgcggtg gggctgcgat cgatgccggt gcgctacctg ttcctggacg aggtcgacgg
47521 ctatccgccg gacgtcgagg gcgaaggcga tgcgatcgcc ctggccgagg cccgcacccg
47581 caccttcgcc cgccgcaaga tcttcgtcgt ctcgaccccg acgatctcag gagccagcgc
47641 catcgagcgc gagtacgagg cgtcggacca gcgccgctat ttcgtgccgt gtccacactg
47701 tggccagttc caatggctgc gcttcgagca gctgcgctgg gagcgcgggc ggccggagac

47761 ggtcggctac gtctgcgaag gctgcgaacg gccgattccg gaacatcaca agggcgacat
47821 gctggcacag ggtgaatggc gggccttggc gcccgagcac ggcgccaaga ctgccggttt
47881 tcatctgtcc tcgctgtaca gcccgctagg ctggcgcagc tggcgtgaca tcgccgcggc
47941 ctgggacagt gcggtgcacc cggtctcggg tggcccgtcc gcgatcaaga ccttcaagaa
48001 caccgagctg ggcgagacct gggtcgagga cggcgagacg ccggactggc aacgcctgct
48061 ggaacgccgc gaagactata cgatcggcac ggtaccgctc ggcggtttgt tgttggcggg
48121 cggcgccgac gtgcaaaagg atcgcatcga ggtctcgatc tgggccttcg gccgcggcaa
48181 gtccgcctgg ctggtggaac accgggtttt gatgggcgat acgggccggg aaccggtatg
48241 ggcggagctc gcgtcgatgc tcgaggaacg ctggagccat gccgtcgggg cgttgctgcc
48301 cttggcgcgt cttgccctgg acaccggcta cgccacccag gaggcctatg ccttcgtccg
48361 ccgactcaag gatcctcggg tgatggcggt caagggcgtc ccccgcggcg cggccctgat
48421 cggcaccccc accgcggtgg acctcaccca gggcggccgg aaactgcgcc gcggcatcaa
48481 ggtgttcgca gtggcggtcg gcatcgccaa gctggagttc tacaacgcgt tgcgtaagac
48541 cgccgaggtg ggcgaggacg gcaccaccct ggtctatccg ccgggctaca tccatctgcc
48601 gaggatggac gccgagtacc tgcagcagct ctgcgccgag cagctggtga cccgccgcga
48661 ccgccacggc ttcgcccggc gcgagtggca gaagctcagg gagcgcaacg aggcgctgga
48721 ctgctacgtc tacgcccggg ccgctgccgc ggccgccggg ctggaccgct acgaggaacg
48781 acattggcgc gccctggagg aacagctcgg tgtcggtccg ccgccggatt ccgaaccgcc
48841 gttacaaccg atcgatcccc agcgcccacc gactccggtg ggcgttccgt ttccagcccg
48901 gtcgatcttc gcgccgggtg atccgcagcc gctggctggc ttgagaccga agaaagctta
48961 tcgatggcct attccgcaga acacctgcaa gccctggaaa ccgccctggc ccggggcgag
49021 cgtcgggtga cgttccagga ccgcagtgtc gagtaccgct cggtggagga gctgaaggca
49081 gcgatccgcg aggtgaaacg cggcttggcg gcccaggtcg gaccggtggc ccggcagctc
49141 cgcatcacca cgcgcaaggc cacctgatgg gctggtggca gcggttgcgc ttggggctgt
49201 tcggcggacc ggcgccgacc tacgacggca ccggcggcgg ccgccgggcg ctggcctggc
49261 aggtcggcaa tccgggcgcg gtggcggcgc tggcctacag ccaggacgag ctgcgggcca
49321 agagccgcga ccaagtgcgg cgcaacgtct gggccgccgc cggggtggag gcttatgtgg
49381 ccaatgccat cggcaccggc atcaagccgc agtcgatggc ggccgacgtc acggtccgct
49441 ccgccatcca ggcgctctgg gcccactggg gcgatgacgc cgatgcggcc ggactgaccg
49501 atgtctacgg gctgcaggcc ctggcctgcc gcgccctggt ggaaggcggc gaggcgctgg
49561 tgcggctgcg ctaccggcgg ccggaggatg gcttgagcgt aggactccag ctgcaggtgc
49621 tggagccgga acatctgccc gtcgccctga accgggagct gccttcaggg aacgtgatcc
49681 gagccggcat cgaattcgat cggttgggac ggcgggtggc ctatcacctg taccgctcgc
49741 atccggagga cggcgccttg gcgccgatgt ccggggcggg cggcttggac acggtgcggg
49801 tcgacgccag cgaggtaatc catctgttcc gtcccctgcg ccccggccag atccggggcg
49861 aaccctggct ggcgcgggcg ttggtgaagc tgcatgagct ggaccagtac gacgacgccg
49921 agctggtgcg gaagaagacc gcggcgatgt tcgccggctt catcacccgg ctggcgccgg
49981 aggatccgct catgggcgaa ggaccggccg acgcccaggg ggtggccctg gcggggctgg
50041 aaccggggac cctgcagctg ctggagccgg gcgaggacgt gcgcttcagc cagccggccg
50101 acgtcggggc gagctacgcc gagttcctgc gtatgcagtt ccgggcggtg gcggcggcca
50161 tgggcatcac ctacgagatg ctgaccggcg acctgaccca ggtcaactat tcctcgatcc
50221 gcgcggggct cctggagttc cgccgccgct gcgaggcgat ccaacacggc gtcatcgtgt
50281 tccagctgtg ccggccggtg tggcgggcct ggatgaccca ggcggtgctg gaaggggctc
50341 tggaacttcc cggcttcagc cgcaggacac gcgagtactt ggcggtgaaa tgggttccgc
50401 agggctggca gtgggtcgat cccaagaaag agttcgatgc cttgcagacg gcgatccggg
50461 ccggcctcct atcgcgttcg gaggcgatct cggcctttgg ttacgacgcc gaagacgtcg
50521 accgggagat cgccgccgac aacgcccggg cggatgcatt ggggctggtg ttcgattcgg
50581 acccccggca tgaccgcgct gccgcgccgg ccaggggatc ggcgccggca gccggtggaa
50641 accgggtcag cgtttgaggt cgcgatagaa attctcgtgc ggccccacgg cctcgagata
50701 gaccaggctc aagccgtcat cgcgggtgta gccgagcagg tagagctgca cctggctgcg
50761 gattttatag acccacagcc gggccagatc gcctttcttt ttctcaccga cgcccggatc
50821 gctggcgatc accgcgaccg ccgcatcgac gtcggccgcc actggatcgt gcagtttctt
50881 gtaggcgcgc gcgaaacggc gcgtctgcag cacctgccag ttcaccggcg ggtacgcggt

50941 acgaacggtg tggcttgctc aggcgactca ttcagcgcca tcaggctttc ggcgatgaag
51001 gaagccggca gatccgggtt gtccagcgcg gcacgcccaa ctttggccca gaactcgatc
51061 tgcccctgca cggtgcggaa ctcggccttg gccgccgcgc gggcttcctc gatcaaggtt
51121 tcatcgatac ggacggagac gctcatgatc ggttctcaag tagttcgcca caattgtagt
51181 cagagcggcc ggggtgggca aggctgatgc ttccccactt ggccggccgc ctgtacggaa
51241 cgccgctcct catcgcccga ccgaagctcg aggtgatcct ggcggtgttg ggcgcccgga
51301 tcgggctgcc ggcagcggat accccggtgc cggcgccgat gcccagggct gccgcccaag
51361 cccttccggg catcgccgtg atcctgatcc acggcaccct ggtgcgacgg acgctggggc
51421 tggacgcaaa ttccgggttg acctcgtatg ccgagatcgg cgcccagctc gatgccgcgc
51481 tgcgggatcc cgaggtcgcc ggcatcctgc tcgacatcga ctcgcccggc ggcgaatccg
51541 gcggggtgtt cgagctggcg gcgaagatcc gcgcgggcac ttcgcacaaa ccgatctggg
51601 cccatgccaa cgatacggcg ttctcggccg cctatgcgat cgccgccggc gcctcgcgcg
51661 tgaccctggc gcagaccggc ggggtcggct cgatcggggt gatcgccctg cacgtcgacc
51721 agtcggtcaa ggatgcccgc gagggactga cctacaccgc gctctatgcc ggccatcaca
51781 agaacgacct caatccgcac gcgccgctct cgccgcaggc ggccgccgcg ctccaaacgg
51841 aagtggaccg gctctacgcg atcttcgtcc gggatgtggc ggtattgcgg caactgccgg
51901 aaacggcggt acgcgccacc gaggccggac tgttcttcgg cgaggacgcc gtatccgcag
51961 gactcgccga cggcgtgctc ggcttcgagg cagtgctgag cgaattcgcc gacgcgctgc
52021 aggcccagcg ccgactgagc gtcatacagg cccaccaaac cccggtgatt tcaacacgaa
52081 tcatggagaa ccccatgctc gaaaaccctg ccgaaccgat cgcccctgcc cagttgggtg
52141 aggaaacatc cagggatccg aaccccgggc cgtcggccac gacggacgga tccgtcccgc
52201 aggtggcgag ttccttacca ccgtccccgg aaaccggcca agccgacgcc caccatgagg
52261 cccgtgccga agcccaggcc atcgccgagc tctgcctgat cgccggcgcg gcctcgcgca
52321 ccgcggagtt cctggcggcc ggcatgagcg aggcccaggt gcggcgcgcc ttgctggcgg
52381 cgcgggccga gacgcccgag atcgcttccc gcatccaggt cgatgcggga acctcggttc
52441 ggcccgaggc gagcccggtg gtcgccgccg tccagaaact catcgcccgg agctgagcca
52501 tgcccaagct gcgcgaaccc aagaacctcg gcgatctctt gaagtacgag gcgcccaacc
52561 gctattcccg cgacctcgcc ccggtggccc tgggccagaa gctggcactg ggggcagtgg
52621 tggcgcgtga gccggccggc gctcggctgc aggcgctcga tcccgccgcg acggatagct
52681 tggcccaggc ggtgggggtg ctgatcgaag cggtcgatgc caccgcggcc gaagtcccgc
52741 aggccctgct gcttgcccgc catgccatcg tgtccgaccc gtccctggcg tggccggccg
52801 gcatcacccc ggcccagaag accaccgcga tcgcccagtt gcaagcgctg ggcatcctcg
52861 tccgcaccgg agcctgatca tgcagaaccc gtttcacaat cccgcctttt cgatggcggc
52921 gctgacggcc gccatcaaca tcattcccaa tcgctacggc cggctggagg atctgaacct
52981 gatgccggtg aaaccggtgc ggcagcgcca gatcctggtc gaggagcgca acggcgtgct
53041 gaacctgttg ccgaccctgc caccgggggc gccgggcacg gtgggcgtcc gcggcaagcg
53101 caccctgcgc tcgttcgtga ttccgcacat cccccacgac gacgtggtgc tgccggagga
53161 ggtccagggc atccgtgcct tcggctcgga gaccgagctg gaggcggtgg ccggggtgat
53221 ggcgcgccat ctggagacca tgcgcaacaa gcatgcgatc accctggagc atctgcgtat
53281 gggcgcgctc aagggagtga tcctggatgc cgacggctcg gtgctgtacg acctctacga
53341 cgagttcgac atcccgccga agacggtcgg gtttcagctg ggcacggcca ccaccgacgt
53401 caaggcgcga tgcatggagg tgctggccca catcgaggac agcctgctgg gcgagttcat
53461 gaccgaggtg cattgcctgt gctcgccgga gttcctcgct gcgctgaccg ggcacaagga
53521 cgtcaagacg gccttcaccc actggcaaca aggcgcgatc ctcatcaacg acgtgcgccg
53581 cggcttcacc ttcggcggca tcaccttcga ggagtaccgc ggccgcgcca ccgacgtgca
53641 cggggtgacc cggcgcttca tcgccgccgg cgaggcccat gccttcccgc tgggcaccgt
53701 cgataccttc gccacctatg tcgccccggc cgatttcaac gaaacggtga acaccttggg
53761 ccagccgctc tatgccaagc aggccccgcg ccagttcgac cgcggcaccg atctccacac
53821 ccagtccaac ccgctgccga tgtgccaccg gccggggctg ctggtcaagc tgacggtcta
53881 gggatggacc tcgatgcggt caatgcggtg tgcctgtcga ccttcggcga gacggtgatc
53941 ctgtggccgg accggaccga tccgccgccg gtacacctga cggggatcgt ctcggtcccg
54001 gccggactgg aacgcccggg gccgatgggc gcgccccgtc ccgaggcgga cccgcgactg
54061 accgtgcgga gtgcggacct gcctgcggtt cgcatgggcg atcccctgaa gctgcgtggc

54121 ctcccctacg gcatcgcctc ccgcctcgat cgcctggacg gcctgaccac cttgatcctg
54181 cggccccgat gaacgccccg ccgaagaagc tcctgctggt gtcgccccac ggcaatggcg
54241 atgaggtccg gctggccctg cagatcatcc tggtgacgat ctggtcgggg ctggtggccc
54301 agctcaatcg atggcggcgc catccccggc cgtggcggcc gtgcctgtgg tgcctgctgg
54361 tcgacgtcgt gtcctgcagc ctggtcggct tctcggtgtg gctgctggcc gagcgggagg
54421 gcctgggcta ctacgagagc ctgtgggcgg cgatcgtcgc cgggcatctc ggggcgcgct
54481 ggttcggttt gctgatccat tcgagtcggg gctaggcaac aggcatgagt ctcggcttgc
54541 gcgtgcagct gcacggcgat accgctcagg atttgcgccg cttgccggcg gtgctgcgca
54601 aagcgtcccg ccgggcggtg ggccagaccg cgcgggagac ccgcgaggcg atggtccgag
54661 cgatcggagc gacctacggc attccgctga gggcgctgcg ctcgcgccgg gtgcaggcgt
54721 atctgcggct ggcaggagtc cgcgcccggg tctggaccgg ccacgccccg atcaaggccg
54781 cctatgtcgg ccgcctgcgc caggaggagt ggggcagcag cgccggggcc tatctctttc
54841 ccggcagctt cgtcgcccgg atgcccagcg gccatcgcgg tgtgttccac cgggcaggcc
54901 acgcatcgtt accgattatc gaggacgtgg tggccttgcc caaggtgccg gccctggccg
54961 aggcgctggc gacccgggcc acgctgcggc tggccgcgct gatccgcgaa atgacgcagg
55021 acgcgctgcc gcgatgatca ccagcatgtt cgagatcgag ccggcgctga tcctacgcct
55081 gcgcgacacc ctgccggagt tcgtcacggt ggacagcgtc ggcgtcctgg ccggggtgca
55141 gaacctggag ccgctctgtc cggccgccct ggtgctgccc ttgggcttcg ccgggccggt
55201 cgggacgccg ccgcccaacg ttttcctggc cgagcgccag cgctggcaga ttacggtgtg
55261 tgtcgcccac gctccgccgg ccagcaccgt ggtgacgggc ggcgaatacg tgctgcggat
55321 tctgcgtgcg ctggagcact ggtccccgca accgggctgg ggccggctga agcacgtcgg
55381 cctggacgac ccctggttcg atctcggcca tgtcgagttc tccctggtgt tcgaggtgcg
55441 tccgctgccg ctggacaccg ggaccccgtg acgcccggtc ggcgtcaatc ttcctttcct
55501 ttaatccatc catttttcta aggagttgac acatgactac gaccgccatg cttttgaccg
55561 gcaccatcaa gggcgccgtt ggcgccggtg ccccggtgga cctgggcaat gccgccctgg
55621 acctcagcat cgaggaggac accaaggaac tggccgatta ccagaacccg ggcggcggct
55681 cgatcgcgtc cttgagccgc atcaagtcgg tcacgctgaa actgaagttg tggtcgatca
55741 gcaaggagaa cctggcgctg gccacccgcg gcacggtgag cggcaacagc atcgaggcgc
55801 tgacccagac cgcggaggac tggcacatca ctttcgacgg cgtcaacgag gtcaacggcg
55861 atgccgtggc ctacgacttc tacaaggtga agttctcgcc ggccagcagc ctgcccggcc
55921 ccggtacgga ggatttcgcc gtgctggagc tgaccggcaa ggtcctgaag gacaccagca
55981 agaccggtgc gggcgtcagc cagtacttca aggccaccat caccccggcg gtgtagtccc
56041 ccatggcaga gctcgacacc acgaacccga aaggactccg gcaggagcgc accatcgagg
56101 ccggcggccg caaggtcgtg gtgcgcgagc tgaccgtggg cgaggtccgg gcctggctga
56161 aggacgccaa cgccgaattg gatcgcaacg acctggtggc gctggcgctg ttcgcggaca
56221 tcaccctgga cgatctgacc cgattctcgg acctctcccg ggccgagctc gatgccatgc
56281 tgccgtcgga gctggacaag gtgcgggagg cggccaagag cctgaacccg catttttcg
56341 ggctccgcga acgcctggcc aacgcggccc aggcggccgc tacggcgccg ccggcaacct
56401 agagaatttg gagatgacgg cgctggcgct gatcgagcgc ggccacgccg gcgtctggca
56461 ttacccctgg cggctgttcc tgcaggcgcg tttgatgcgg cgacgctagg cccatgacga
56521 ctttcaagac ggtcatcgag atcgcggcga acacggccgc ggccgagaag gggctcggca
56581 acgtcgcggc gcaggtcgac cggttgacgg gatcgctccg gcgcatcggg cagtataccg
56641 tcggcgcgtt cggggccgcc gaagccctgc aggctgcccg cgatctgggc aaactctccg
56701 accagtaccg caacctcgaa ggccgggtca agcttgccgc cggttcccaa tcccagttca
56761 ccgaggcgca gcgggcgctg ttcgccatcg cccagaacaa cgcgcaagcc ctgaacggcg
56821 tctctcaatt gtattcccgc atcgccaagg gcgccgggga aatgggcgtg tcgcagcaac
56881 aagtgctgag catcatcgac tcggtcgcca aatccttccg gatttccggc gcctcggcgg
56941 aagaggcctc gggcgccacc ctgcagttct cccaggcgct ggcctccggc gtgctgcgcg
57001 gcgacgaatt caactcgatc atggagcaat cgccgcggct ggcccaggcc atcgccgacg
57061 gactcgacgt gccgatcggc aagctacgcg ccatggccga agccggcgaa ctgacgactc
57121 agaaggttgt cggcgcgctg caaaaggcta aggccagcat cgacgccgat gccgccgggc
57181 tgcccgatac catcgagcag gccatggtgc gctgggacaa cgccgcgctg aagttcgtcg
57241 gcacctcgcc cgagatcacc cgcgcggccg agaccatcgc cgccgcgatc aataccgcgg

57301 cgggcaacat cgacacgatc gccaacgggg tcgagatagc gggatcgttg ttggtcgcgg
57361 tgctggcggg caaaggtacg gccgccgtgg cggccttcgc cgggagccag gcccggctgg
57421 tgcaagcgaa tctggcggcg gcgaccgcag cgcggaacca cgccctcaat gaggaataca
57481 acgcccgtgc catgctggct tccgccgaag ctgcagtagc gaacgcctcc ggcatggcgc
57541 ggttggccgc ggtcgaaaca gcgctggtgc cggcatcgca gcgcctggcc gtcgcccagg
57601 cggaagtggc ggccagcgcc ggaccgctca agattgcact gggtggtctg gcctccttcc
57661 tcggcggtcc taccggtatc gcagtgttgc tgctgacggc ggtgtccgcc tggcatctat
57721 tcggcagcgc cgcagaatcg gaactggagc gcgtaatccg gaagcgccga gaactcgcca
57781 aggaaaccgg caaggacacc cgcggcaagt ccgaaaccga tctgagcctg ctccaggacg
57841 aagccgcggt gaagaagcag gaagccgtgg tcgcccggtt ggcggctcgt tacaaggagg
57901 tgggccaggc ggcggaccag gcgttcatgg gcggcaagct cgggcgggag ctggtaggcg
57961 aaaccgcgct gctccgagaa atgcaggccg agctggtcaa gcgcaagcag gccgcgaccg
58021 acagtggcga tgcgctgaag gccaaggaaa aacaggtcca gcagagcctc aatgacacca
58081 agtacgccct caaggacgcc actacccagg ccgagaccta ctacaagcgc cgggtcgatg
58141 cgatcgatgc catagaacag accggcatca cgcggatcat ccagaccccg ccgacgcagc
58201 ccgtccccgg cgagtccagc accttaggga gcacggccca gcccaacaac aaggccaaga
58261 ccgagctgga gcaggcccag gccgtgttta ggctccagca gcaggcagaa caggcccggc
58321 tgactgcggc gcgggactac gccaatcagc gcctcgctct ggtagatcag gtctacggca
58381 gggaaatcgc caagtacaag gacggggaag acaagaagac cgcgctggag cgtgaatcgc
58441 tgcaagcccg ccgggcgatc tacaccgagc tggaagcggc ctacacggcc agcatcgaca
58501 agctggtcgc tcaagagcgc aggctgcggg atgaggcggt cgccgcagcg aaggcgcgcc
58561 aggacatcga gctggaaacc gcgcaagcgg tcaaaagcat ggcgcagggc gcggcctcga
58621 ccgaggaggc ggcacgtgcg cagtacctgg aactgcaaag gctgattgcc gaacagcaaa
58681 gcgcgctcgc cgccggcaac ctggaccttt ccaaacaggc cggcaatcag gcgaagggga
58741 tcgccgaggc gctgggccag cagattgcct ccgcgctgcg ggacgtgaag cagaagatcg
58801 ccgaacaggg caaaacggcg gatgccgcca acccgcccat cgaccaccag ggaaactggt
58861 gggaccagga attcggcgcg gccgccaagg atcagagcgg ccagtttatc cgcaatgctc
58921 cggcccagcc cgaaccgctc aagcccgacc tcggcacgca gcaggatttg cagaagctga
58981 tccaactgcg ccaggagatc ggccagctgc tggccgatgc cgagaccaag accgggcaga
59041 ccgcaacgca tcaggccgag cagaccgccc agcaaatcca gaccgccatg cagtccgcgc
59101 aaagcgtggg ggccgagatc gacaagatcg accagcagct ccgacacggc ttcaacctgg
59161 acatcaaggc cgatcctgcc gcgctgcaag ccctgcaaac ccaactcgcc gagctgctac
59221 ggcccgagac caagatcatc aacatcaagg tggtcaagga cggcaactcg atcaccgcaa
59281 cgcccgacgg ctccgccctc cccgataccg gcctgaccgt gcccggctac cgccgcggcg
59341 gctggatcaa gggctacggc ggaggcgacc gcatccccgc cctgttggag gaaggcgaat
59401 tcgtgctgcg caaggaggcg gtgcgcaagc tcggcctcga caagctgtac gccctcaaca
59461 acttagccct cccccgtttc agcatcggcg gctacgtggc gcaagcactg ccggcgttcg
59521 atgcgtcgct cacgcgctcc agtgacggcg gccagccggt gcatatccat ctgcccggca
59581 taccgggttc gttccccctc aacggcgacc cgcaagtggt ggcggcgctt aagcgcgagg
59641 tggcacgggc ggcgctgaag cacgggcgga tcgtgcgatg agtttcccaa ccgacaggag
59701 caatacccga tgaatccgat cttacgctgg gcgctctggc ttgtagtgca ggccgtctcc
59761 ggcatcacca aggagcaatg gggcctgatc caggacaagg tggcggctct ggaaacccgg
59821 acgatcaaag ggatcgtcga caagaccacg ctgaacgaca tcaagaagaa ggaggcggca
59881 gcctacatcg gcaccttcgt cagcggtgtc cgaaccaacg tcgtccattt cctcatcgag
59941 gccgcgctgt ggttcgtgcg gtcgttcggc gtgaaagcgc cctgacgtgc ataaaaacac
60001 ggtgaatttc gacaggaggg cggcgatatg ttcgaaaaag tgaaatatct gtacgagtgc
60061 atgcaggccg gcaagagcct gcagaaccca cggctatgga gccagcgcgc gagcctgatc
60121 gcggtcctga ctgcgctgct gaccgccggc gtggggctgg ctcgcgcgtt cggctacgac
60181 gtggacgcca caggcacgga catcgcggca gtggcccagg gcctcggtgt gctgggcgtc
60241 atcgtggtcg atgtgatcca ccgggccagc aacaaggagg cgggaaagcg atgaggcgca
60301 cgaaggcggc gaccatgtcg gtttctgcgt tgatcgtcgg catgattgcc ctgcatgaag
60361 gctaccgggg cgaagcctac gacgacggag tcggggtcca gacgatcggc ttcggttcga
60421 cggcgggcgt gaagcgcggc gaccggaccg atccggtgcg ggcggtgcag cggctggccg

60481 ccgacgcaac ccgggtgtcc caggcggtag cgcgctgcgt gggcgaagtg ccgctgtatc
60541 agcacgagtt cgacgcctac gtctcgctga cctacaacat cggcgtgaat gcgttctgcg
60601 gatcgaccct ggtcaaacgg ctgcgctcca caccgcccgg ctatgctgag gcctgccgcg
60661 aaatcctgcg ctggaacaag gccggcggac gcattcaaaa agggctggtg cggcgccggg
60721 aggcggaata tgcgctgtgc gtcggcaccg cccagggcgg ctaggcatga tcgtactgac
60781 gctgcgggaa atcaccttgt cgacggtcgc cgtgctggtt ttcccttcgg ctccgctcag
60841 gacaggcggc ggattggccg gggtgctgct ggggtgagtc tcgtgctgac actgtcaggc
60901 tgctgctcgc tggagccgtg ggtgacgctg gcgccgcggg cgtgcgcgtg gccggtgcgc
60961 ctttctggtc ttgtcgccgg cgtcgatgaa gtcggcctgg ccttgcgctg cgacgagctg
61021 gagcccgatg aacagtgagc gatctccgcg acacggcatt caagctcggc ggcgtggtgg
61081 tgccgctgta cgcccggctg gaatggtcgc agacctaccg ttggcttcaa ggggccacca
61141 cccaccggat gtggtccggc cgctcggtga agcagcagac ctggcgcaag ctcacgaccg
61201 aaatcagcgg aggcggctgg gttcctccgg ggctgtcggc gctggactac tcgcagccgc
61261 tggtgctggc ctgtgccgcg cccctgtccg tcgtcggcgg cggatcgccc ccggttccgg
61321 tgcggacgga gcccgatttc gccacggtga cgatcgacgt cgacggcgtg gccatcacct
61381 actactaccc gaagatcact gtctactgcg agccccccga gcagcagttg gacggggtga
61441 gcgggaatta ttcgtggcgc ctgaacgccg aaatggtcga tcccctagga gcatgacatg
61501 ctgaatctcg ccgggctcaa tggcgtcaag gccatggtga cggacgcctt ggtagcgccg
61561 tcggatttca cgatgaacgt cagcgcactg ggtgcgccct gggttttgcc gccggtgctg
61621 gccagcggcg aatactacta cctgacgatc atcgatcagg attacccgac gaagtgggag
61681 cgggtgaaag tcaccgcctg ccaggggatg gggggccaatt gcctgctggc gatcacccgc
61741 aacgtcgcca gttccaccgg ggtggctcag accttcaccc aaggggcctt cgtccaatgg
61801 tcgccggggg tggaggagat ggagtcgcgc tggcggatga tcctgtcggc gtcgatgacc
61861 gggaccaaca cgatgaccag cacccggttc gtggcgccgt tttcgggcag caccacaggg
61921 tccatgggca cggcgtactg gatcaatcgt accggcaaga ccctccggct ctccaatctc
61981 gtcgccgcgc tctcgaccgg ttcgagtttc agcggtgaca gcatcgtggt ctcggtggaa
62041 gcgggctcga cggcgccgac gtccccgtcc gctttgcaag tggtggtatc gcccggcgac
62101 tatggcttca agtcgaataa cgtcaacacg gtgaccgtgg ggcctgacag cctggtctgc
62161 ttcatgctca gccgatccaa cacaggcgct gcggtatccg gcggctttca actcgccaac
62221 gtcagcgtgc tggccgagga gttgtaatga cgctccccgt cggcggcgtt accggcatcg
62281 gcggtgttgc ggcggggccg gatgtcggcg agcggtatgc gcttaccgcg gtactggaac
62341 aagcggtttt ctccgtttac ggcctcggga ccggtatcac ccaggctgtc gtcgacatcg
62401 acgcctacga cgtcttttgt gtcatcgatc agccggtctt cgaacgcttc ggtctgagcg
62461 ccgccgtcga gcagacgctg ctcgacgtct atggcatgac ggcgccgctc cggttcgagg
62521 tcttggcggt ggccggcaac tcggcttcga tccagcaagt ccagttctcg atctcatcgc
62581 ttgcggcccc cctacagcag agcatcttct tcaaacccta ccgccgcgct gcatggatca
62641 cgcagtggat tggcgaggtc tacggtgtcg tcgcaccgat cacttgtacg gtggaccacc
62701 tgccgttcga cctggcggcg caggtcgagc tggccgtata cgaagttgtt gcggccctga
62761 acgtcccgtt ccgtcacacc gtgttcgatg cccaagccgt cgccggcggg gccttcgtgg
62821 cccaagcgat ggccgccagc gccgacatta cctcctgggc ggtactggtc aacatcggca
62881 acgcggccgg cgacggagag ccgctgaatg ccgggaccgg ggcggtgatg ggcgcgctgc
62941 tgggtaggct gaccgggcag atgacggtgg atgcggaaga gggcgcggcc cggatcgccg
63001 aattcagcgt gctgccggag ccaggtccga tcagcatcaa cgcgctgacc ggcttgccga
63061 tcacgatcta cctggtggcg gggacgacgc acctgccgat cttccagggc atcctggata
63121 ttcccggctg ggacccggtg cagggcgtgc tgcaactgtc ctgcaccgac aatctgcaga
63181 accgcttcga cggcatcaag cgcaaggaca tcgccgccat catcggcggc tattggtccg
63241 agtacgtgtt cgacaagaag gccgacgagt ggcaatacct gcaagaccgg ctgtcgacgc
63301 tgccggccag ctacgacctc gacctgaatc tacagggtgt cgtcaccccc tggcgggcca
63361 agacggcccc cgacttcacc ttcacccgcg atgcggtgat cgagcacagc atccgcatcg
63421 acatggcctc ggcgcggcag ctggtgaaca ccatcgaggt gaagatgcag taccgctacg
63481 agcggctgat gcagcgcaac gtcggcattt tctgggaaat ggacatgcac gagctggtgg
63541 ccggcggtgc cgcgccctcg gtggaaacgg tgttccaggc cgtcgacggc accggctggg
63601 cctgctacgg cctgacgatg aaggcggtgc ccaccggatc gttcgaaggc gtggggctgc

63661 tcggcgatat accgcgctac agcctgacgg cgggtatgca atgtgcgctg gcgcagcgct
63721 ggacccagac cgtcaccgag gactggagcg tgacggtgga cgcgccagat tcgatcaaga
63781 tcatcggcaa acgcaagggc agcaccacgg cgaatttcga ttcgaatgcc gacaaagacc
63841 cgcgctacac ccattgggac cggatcacgg aaaaggaaat caagctcacc ctgatcggcc
63901 aggacatggt atcgccgatt gcgggcgact cctcgttcat cccttaccac ccgctgaaca
63961 tcaagcagta caccgtcgca gtgccggaca gcgcaaagcg gctgccgtcc caagacccgg
64021 cggcgaatcc ccagggcgat ctgtactacg acctggacga cggtgcccag gacaaccgca
64081 ccgggctctc caacggctat atgacgggca tcgcccgtgc ccagcgcgac atcctgtcct
64141 cgcaccgcca aaccacgctg agcttcaccg gcctgatcgc gccgctgctc gaccggatgc
64201 atacggtgcg catgctgtct gcgcggctga ccgccaaggg caaggtaagg cgggtcacgc
64261 accatctcga cttcgatgcc ggttcggcgc tgaccgaagt cgccatcgcc gtgtcgaaat
64321 cctacgggat aggcatggtc gacacgccct acgacctgca agccccggcc aagccggcgg
64381 tcgtgcagcc gccattgaaa tatccgatgg agcgcccctc ggcgacctac gacagcggca
64441 ccggcgaatt catcgtgacg gtgccaggcg tggcccagga gcacatcgac gccacccact
64501 cggctccggc cgaccatgtg agcattttca tccccgacga cgaactactt ttggaggcct
64561 aaaaccatgt cctcgttcaa actgtacacc gatgccgccc tgaccacccc gctgacgggc
64621 agcctggtgg ccgcccagaa tgccgacggc tccactccgg ccattcagtt ctccctatac
64681 ctcggttcga ccaccgccag ccggatgatc caggccaacg ccaatcccgg tgtggatcag
64741 attcaggcca ccgtggtgga cgctggcgcc ggtagcgggc acgacgtgag cgaagtgaaa
64801 ttggcggcga ctcaagccgg cctggcgtcg gcagtcggcg gcgccgcgct ggacctgggc
64861 acttccatcc tgtcgggcgc ggccaacgcc aagccgatct ggatcgaggt gaacgacgcc
64921 acccacgtgg tcggcaccgc cacggaactg tcggtggtga tcgccgacgt gcgggaaacg
64981 gcgatcgtgt aatgacagtt caaaagttct tcgtcaacga catcaacgcc atcgtcgatg
65041 cggtgcggag cagcggcgaa gatttgcccg aaccggcggc caaaccgggc atcggcgccc
65101 gcaccggggt gggcgcgccc aagcgcggag gcggcatcgc ctcccccctc accgagacga
65161 cggcgccgaa cgcaaagggc gagttttggg cggatcggga gtattacccg gctcgggata
65221 tggaaaccac cgacggcgag ttcgtcatca agtacgccgc catcaagacg ttgaaattca
65281 cagatgccaa cggcgaggca gtcgagttcc atttcgccga accggatccg gataaagcgc
65341 tggggacgac ggcatgaaaa cgatcgcgcg gcagagtgac ccgttcgacc tggtgcggat
65401 cgtcgatatc caggcctgcg ggctgctgtg cacgccgacc gccggcaact atccggacgg
65461 ctggggcgag ccgttcaagg atgctcagga ccaaccgatc cacccgccgc tgggcactta
65521 cgccccagcg tcgggagcca tcggcgttga gctgtcggcc gagaccgacc ccaaaaagcc
65581 caaggccctg caggtcaagc ggcgcgcttc cggccgcaag gtcggcaatt gcctggttga
65641 gcgcaagatc ggtaagcgct ggattcgggt gtcgttcgac gggccgttca accgggtgtt
65701 cgtcgataac gacctgcagc cctacggctg ggatgtgggc acggaccgct actccgagga
65761 accctggagc aattacatct atctccgcgg aaaacggttg tatatcgacg ccaagaaaaa
65821 cgtttacgac tcggatttct tcggcaggat cgtgggagtc ggcatccgcg acgagaacac
65881 cctggtactg gcggccatcc atcgcaacga aatcgccttc gcccccctcg atatctcgaa
65941 accttatgcc gacggtcaaa ccgtgtcggt gacgtattac aagctgacgc ctcaggtcca
66001 ggatctgccg ggttatacct tctaccccgg tttcacggtc aagcccacgc cgccgggctt
66061 cgtttttcac ggccccattt atccgcagat cgatacgccg tttgaattca attcggattg
66121 cacgcaggcc tcctgcattg ccaaaagcta tcgcttccct gacaccagca tgtatgtggc
66181 gcggcggcac tggcgcattg cgaacggcta taaatccgag gaggaaggcg acgtcgattc
66241 cgagttcagc gacggcgtgc cctacaactg ggcgaatagc gacctggccg acgacgtgga
66301 agcggtcatc acgttcctga acgaatcccc catggagtcg gtcatcaaca agttgggcgc
66361 ggcgcggttg aatcccaatt tcaaacgggt gccgtttttc cagcgctatc tccatgacgt
66421 ggaattgatc tggagccagg atgacaagaa caagacaaat aaaaagaagg tgctatccgc
66481 cgtgcagggc cgggcgacac gggtggatgc gcatcaaggg ggtgagtcgt tctggacgcc
66541 caacagcgcc cgggtcgaaa cgctcaaccc gcacacggcg accgtgtcct ggtcgaccta
66601 caagatatac aacaccaacc ccgatccggc gcagttgatc gcgaccgggt cgcccggcga
66661 ttacgatccg ggccgcaata ataatcagcg ccacgccgga gaatccttcg tggattacac
66721 catcgagcgg ggctacgaaa gccatttcga cctgcccgtc tacgtttatt acgacgacga
66781 ggacatgaag tcctttatcc gattcttcgg ggaagatact tatcaataca ccgagaaaat

66841 aagatttcac gcctctggtg tggtaccggt cggcgagtcc gggatgggat cgaccggcta
66901 cggggaggaa atctcgggaa gcagtatcgt cggaatacag tcgcacgacg gcgccaatca
66961 gaaggtgtac tacttcggca gcgtggatac ggtgtatggc gaggactaca ccgacaatac
67021 ctgccgcaag gcgcgttcgc tcaaggtgta tgcctcgcat ccttatgcgg gctacccggt
67081 catggagcgg gtaacgatca gggacggcac gggagagccg ccccccggtc cgccctacga
67141 caccgcgtac ggcatgaacg gcgtgccggg cgagttcttt ttcatttcgg aagggctgat
67201 ttacgtcgaa tccgacctgt tggcgcgcat catttggatg gacccccggc aaaacaatta
67261 cctcttcgcc aagtcgacgc accagggcgc gtggtatggg acaaccgtcc agaacagcac
67321 ggacccgttt ttcgacgcgc gactctcgag cattctgtat ggcgtggctc cgggcttggc
67381 ctcgggcgag atcgtcaata tcgatacgct ggagaagttc ctgggcggca aactcatgac
67441 cgaggtattg cacaccgggt ggccggagac taagggtgcg ctggtgaagt gcaagtcgtt
67501 caagtcgatc gactatggcg gctgtgtcac gctgccggag tcggggatcg ccgtggcctc
67561 gtacaaggtt cccgtctggc atgaaagcgc agacccgccg cagcggctgc gataccgcac
67621 gttcagcgcg atctccaccg ggcagcagct gcgcgagatg accggattga gcgcgggcgc
67681 cccgaacagc tatgccgatt tctacgcgcc gttgaacctg ttttga
//

```
LOCUS       16-5-R2              45879 bp    DNA     linear   PHG 16-FEB-2025
DEFINITION  16-5-R2.
ACCESSION   16-5-R2
VERSION     16-5-R2
KEYWORDS    .
SOURCE      .
  ORGANISM  .
            .
FEATURES             Location/Qualifiers
     CDS             26..265
                     /ID="QRZSKLUB_CDS_0001"
                     /transl_table=11
                     /phrog="No_PHROGs_HMM"
                     /top_hit="No_MMseqs_PHROG_hit"
                     /locus_tag="QRZSKLUB_CDS_0001"
                     /function="unknown function"
                     /product="hypothetical protein"
                     /source="PHANOTATE_1.5.1"
                     /score="-5.610075691459885"
                     /phase="0"
                     /translation="MLTRKSNWMNGIPPTRDEPAGQERVKRGYGKLCLYPAENGGISGD
                     HSQRRPLAPLQMNASAAAAGKLLPAVGTLKGLAQ"
     CDS             326..445
                     /ID="QRZSKLUB_CDS_0002"
                     /transl_table=11
                     /phrog="No_PHROGs_HMM"
                     /top_hit="No_MMseqs_PHROG_hit"
                     /locus_tag="QRZSKLUB_CDS_0002"
                     /function="unknown function"
                     /product="hypothetical protein"
                     /source="PHANOTATE_1.5.1"
                     /score="-2.440194140616476"
                     /phase="0"
                     /translation="MWADATDSLADDGCHPPAFNDNTLEPEERPERTRAALAP"
     CDS             503..1051
                     /ID="QRZSKLUB_CDS_0003"
                     /transl_table=11
                     /phrog="No_PHROGs_HMM"
                     /top_hit="No_MMseqs_PHROG_hit"
                     /locus_tag="QRZSKLUB_CDS_0003"
                     /function="unknown function"
                     /product="hypothetical protein"
                     /source="PHANOTATE_1.5.1"
                     /score="-111.22766635717664"
                     /phase="0"
                     /translation="MKPESPRLKTPLLRPLLWCCLSLPAWPAQAATDAAFAAKSDASSE
                     ILAEIGQHGFSRVLVEFRRPATASRLTPSGAGLETYKASIKDGRDAIVSSLFGNPDHPM
                     DGRGFARALRNFEISPIFAVNVDQAELEALAADARVIRIHYDRLLQPTLNDSVPLVGMS
                     GPAGAYVKGSEINERECVR"
     CDS             complement(1130..1666)
                     /ID="QRZSKLUB_CDS_0004"
                     /transl_table=11
                     /phrog="26"
```

/top_hit="No_MMseqs_PHROG_hit"
/locus_tag="QRZSKLUB_CDS_0004"
/function="connector"
/product="tail completion or Neck1 protein"
/source="PHANOTATE_1.5.1"
/score="-361.5967395615029"
/phase="0"
/translation="MAGAFIEVDDQNARKALAELTRLIGNPEPALHDIGAYLERSHDER
FAAGEAPDGSKWAPLSESYRAIKPQHRDKVLVLEGHLRNSLHYQIEDDGLLFGTDRIYG
AVHQFGAEMGEFGRYYQLSRLKYDKNDFRRHAGSKKGHPIPWGDIPARPFVGLSDADRE
EIASILEEHIQAAIG"
CDS complement(1666..1962)
/ID="QRZSKLUB_CDS_0005"
/transl_table=11
/phrog="No_PHROGs_HMM"
/top_hit="No_MMseqs_PHROG_hit"
/locus_tag="QRZSKLUB_CDS_0005"
/function="unknown function"
/product="hypothetical protein"
/source="PHANOTATE_1.5.1"
/score="-9.950940371504291"
/phase="0"
/translation="MKLDLTLSHHLAGESLSFSWDSDTGTLTGPDADRARRMVERADGK
VTLPAWPEMDAPDPLHNSASFALIFASYGYHLPPELEILLPELTDDLPTDAVA"
CDS complement(1959..3491)
/ID="QRZSKLUB_CDS_0006"
/transl_table=11
/phrog="220"
/top_hit="No_MMseqs_PHROG_hit"
/locus_tag="QRZSKLUB_CDS_0006"
/function="head and packaging"
/product="minor head protein and DNA pilot"
/source="PHANOTATE_1.5.1"
/score="-981216.8235990979"
/phase="0"
/translation="MSAQYGSLPFDEAIRFFRGKDLVPTDRWADVWKAEHDVGFMVAGA
AKADLLADLHGAVLKGVAKGTTLAEFRKDFDTIVARHGWTGWKGESSDAGRAWRTRLIY
DTNLRTSYQAGRWAQVQAGKQWRPYLEYKHSDLSAHPRPLHKSWDGLVVAVDDPWVQTH
WPPNGWGCKCRMFALSERDLRRMEKSGPDTPPDDGSYDWTDKVTGEMHRVPNGIDPGWD
YAPGASRTDLMRKEIERKVAKLPKEIGKSLKAETEQPPPPWRFLSKDELDLETVKTLGQ
ERLDQWLGKSLGSGTIAGVLDAPWEAVAPSAGQIRAILFEDLNTRQSLGGVRPATWNRS
GQGLQVLVRAASRFPDGWVSAANSVPLRVQMAKSRGYYSWKQWNSAERRFEAFIRTDAS
STAEHEYTHHLQRTMPELDAIFQAEHRRRTANDPSEILFAWAKSERGRPDQYVHRYQGR
EYEGHNALEVMTMAFQGLLGTDKFADELLAKMLIFDREMLKVALGLLFHFRP"
CDS complement(3491..4825)
/ID="QRZSKLUB_CDS_0007"
/transl_table=11
/phrog="22899"
/top_hit="No_MMseqs_PHROG_hit"
/locus_tag="QRZSKLUB_CDS_0007"
/function="unknown function"
/product="hypothetical protein"
/source="PHANOTATE_1.5.1"

/score="-341215.77358209726"
/phase="0"
/translation="MNLLGGGLLGSQRRSAGSAVASWARHPDWLPLPAIGPAEEKFIGL
FGVTDDSANFVALLAQGDYTVDWGDGTVENHDSFTVASHQYDYASVPANTLSSRGYKQV
IITVAPRDWTTLSRVELQRRHPSMTQNYAPLWLDIAVGSPNLVWMKLGGWDLSLQFVER
VKIVSVGNVTSTDSLYNSMTALEVVSDFDTSQVTDASYMYRSCDRIKTVPAHNLMNCLT
FTSIFENCSCLEFAPAINTAMGQNFSRFYYNCKALREAPALNTANGTNFNEMHRNNHAL
LDIPAYDLTNAQTARYMFSACNSVKLCPDFNTPALVDTEAMFDNLHNLQFGPMLDLSQS
TNCKLLLNNGYSLTHVPAYDFSSSNDNSFAFNGCTSISKVEATGAKSSIELKNMRLGAA
ELNLIFAGLAAGVTGQTIDITGNFGTSDPACDRSIATLKGWTVVG"
CDS complement(4822..5118)
/ID="QRZSKLUB_CDS_0008"
/transl_table=11
/phrog="No_PHROGs_HMM"
/top_hit="No_MMseqs_PHROG_hit"
/locus_tag="QRZSKLUB_CDS_0008"
/function="unknown function"
/product="hypothetical protein"
/source="PHANOTATE_1.5.1"
/score="-9.420390549647484"
/phase="0"
/translation="MNMGQQINDSGVFSGQKIVDVSGVNPPVTVALYPGPGNTSRWEYS
ATPGAAGNPAAAKWWPGSGDVTGPTIDTIEGACHAIRFIRVSGSDADEFEVTA"
CDS complement(5115..6698)
/ID="QRZSKLUB_CDS_0009"
/transl_table=11
/phrog="113"
/top_hit="No_MMseqs_PHROG_hit"
/locus_tag="QRZSKLUB_CDS_0009"
/function="head and packaging"
/product="portal protein"
/source="PHANOTATE_1.5.1"
/score="-609380.5488217465"
/phase="0"
/translation="MGFWDFIARAAAPFDSAHGRPLPPFAELAETAGPEFDEIATTHDG
RDVTRGYIHPDMILPSSDEILQAKGGDLKVYAEVLRDDQVQTSFGQRRSAVTSAEWDVE
PGGTKRIDKQAAEFIKAQLEHIGWDQRTDKMLYGVFYGWAVAECLWARDGAQLVLDGMK
VRNRRRFVFDGDFRLRLLTGSNAQGELMPERKFWVFATGADHDDEPYGLGLGHWLYWPT
FFKKNDIKFWLIFLEKFGMPTGKGTYQPGATDEEKRRLLSALRAIQRDSGVIIPEGMSI
ELIEAARSGTADYTALYDRMNASISKVVLGHTGSTDATPGRLGGESNAADVRRDLVKAD
ADLVCESANRSFIRWLVDWNYPGAAYPKVWRDVEEPEDLKARSERDKNLFDIGFKPTLK
EVKETYGGEWEEVKPELPPDTGSLPALPAPASDPAQADLAEVDPAPDVVDQYVERLGKE
AEPLIEKGLLDPIRTALDKAIAEGQTLSEFAELLPGLYADMDARDFAEFMAQGMFAAGM
AGRYELQQGE"
CDS complement(6700..8223)
/ID="QRZSKLUB_CDS_0010"
/transl_table=11
/phrog="2"
/top_hit="No_MMseqs_PHROG_hit"
/locus_tag="QRZSKLUB_CDS_0010"
/function="head and packaging"
/product="terminase large subunit"
/source="PHANOTATE_1.5.1"

/score="-2145876.6665998083"
/phase="0"
/translation="MDSSLLLDYQLRWIHDYSPVKIIEKSRRIGLSYGEAADSVLHAAA
ADGGNVYYISFDKEMTQGFISDCADWAKRFEAGAGAIGEEVFPDPKNPDKSICKFTLDF
ASGKEIHAFSSNPRNLRSKGRPKDRLVIDEAAFVDDLAELLKAAMAMTMWGGQIHIIST
HNGEENPFNELINDIRAGRYDYSLHRVTLDDALADGLYRRICQVSEQAWTPEKEAAWRE
SLVKRYRPNEDEELFCVPAFGGGAYLARVLIEACMIDAPLLRFSGDRQFNEAPEPQRRR
IMADWIGDELVPAFALLNKGHRHACGMDFARKGDMSDIVPMEIGEKLNRRVPFIVEMHN
VPHKQQEQVLFAVCDTLPRFGALAIDAGGNGSYIGEAAVDRYGSRVEALQLTEAFYREN
MPRYKALFEDRLISLPMHDDVVEDHRAIRLVRGVPRVPEGKTDKKGQRHGDSAVAGCLA
YYASRMDVGPIDFQSIGGRRAVIDAGENELALTDRGFGTVSGLADFEGY"
CDS complement(8223..8792)
/ID="QRZSKLUB_CDS_0011"
/transl_table=11
/phrog="426"
/top_hit="No_MMseqs_PHROG_hit"
/locus_tag="QRZSKLUB_CDS_0011"
/function="head and packaging"
/product="terminase small subunit"
/source="PHANOTATE_1.5.1"
/score="-60.10582689549568"
/phase="0"
/translation="MPRPARLDELSPEARAELDRQIVARNFKDYAVLVEWIREQGWFLT
DEEPGISVIHRHGAKLKRRLEAVKASTQAARMIAEAAPDDADLRSAAVISMVQSELFEL
LLNLQEADAEDDPATRVELMAKAARGVADLSRASVSQKKWKTEVEARIRAEERAGAEQA
AAAAATKAGVSAEGIAAIRAAIAARM"
CDS complement(8796..9107)
/ID="QRZSKLUB_CDS_0012"
/transl_table=11
/phrog="436"
/top_hit="No_MMseqs_PHROG_hit"
/locus_tag="QRZSKLUB_CDS_0012"
/function="unknown function"
/product="hypothetical protein"
/source="PHANOTATE_1.5.1"
/score="-18.957943520797286"
/phase="0"
/translation="MSNPDMEKARREFIRWSILLTLNNGRPVACHESVLLSVMQTVYSD
ATAIETRRELGYLEGRDLVRITRSPSGPWRAELTRHGVDIAEYTIDCEPGIARPVKYW"
CDS complement(9094..9486)
/ID="QRZSKLUB_CDS_0013"
/transl_table=11
/phrog="462"
/top_hit="No_MMseqs_PHROG_hit"
/locus_tag="QRZSKLUB_CDS_0013"
/function="other"
/product="lipoprotein"
/source="PHANOTATE_1.5.1"
/score="-60.491979599413966"
/phase="0"
/translation="MISIDIWALLTMAVGLLGSIFGAAFTAGKIVAGLYEKRQDERFKA
QEKAREETRRHWDQKFAALEQAANRDTEQWRNVERELLQLKADLPEKYVRHEDYVRGQS
TIEMKIDRVALKIENLQLRTQMHEQP"

CDS complement(9483..9737)
/ID="QRZSKLUB_CDS_0014"
/transl_table=11
/phrog="54"
/top_hit="No_MMseqs_PHROG_hit"
/locus_tag="QRZSKLUB_CDS_0014"
/function="other"
/product="DksA-like zinc-finger protein"
/source="PHANOTATE_1.5.1"
/score="-17.9748200196925"
/phase="0"
/translation="MDAADIAEERIEQELDLNRRAALDMMKPDMPQIVVAGEDGAPTII
CYDCEEPIPQGQLDAYPTAIRCMDCQVEHERYLAAEARR"
CDS complement(9724..9924)
/ID="QRZSKLUB_CDS_0015"
/transl_table=11
/phrog="No_PHROGs_HMM"
/top_hit="No_MMseqs_PHROG_hit"
/locus_tag="QRZSKLUB_CDS_0015"
/function="unknown function"
/product="hypothetical protein"
/source="PHANOTATE_1.5.1"
/score="-3.3330726012891114"
/phase="0"
/translation="MKWRTCPPPQPQPWRSWFAWYPVQTDIGHWVWLEWVERQRFFPYM
HTRFGAFWQYRGPDRSTHGRR"
CDS complement(9921..10439)
/ID="QRZSKLUB_CDS_0016"
/transl_table=11
/phrog="1403"
/top_hit="No_MMseqs_PHROG_hit"
/locus_tag="QRZSKLUB_CDS_0016"
/function="lysis"
/product="Rz-like spanin"
/source="PHANOTATE_1.5.1"
/score="-10.101320339189636"
/phase="0"
/translation="MPISLLLPIAAFLLGGLAGAAGGYRYADAQGTAALAELERQIALR
NFQAEAALRNELEAAHERGNRLAGDLARTKSRVTVRTVEVIRHVPDVTSGQRCLGPDAV
RLLNGALVAAGDGAGLPETAGGIVDPPRSAAASDRDVAGWIATAAGRYRACTAQLNALI
DWHSQSPDE"
CDS complement(10429..11085)
/ID="QRZSKLUB_CDS_0017"
/transl_table=11
/phrog="669"
/top_hit="No_MMseqs_PHROG_hit"
/locus_tag="QRZSKLUB_CDS_0017"
/function="lysis"
/product="endolysin"
/source="PHANOTATE_1.5.1"
/score="-109.41974646008843"
/phase="0"
/translation="MRWRCEFRTACVFNLLLVLALVGCIPEPVQAADAIPHAAARYRGD

LTRNARLIWGLEAPVATLAGQIHQESAWRATAQSKYAAGLAQFTPDTAKWICGAYPSLG
DMGCDRMAPAWAIRALVTYDLHLFDRIDAANACERMAMALSAYNGGLGWLNRDKRLASA
KGADELSWFGEVERFNAGRADWAFTENRGYPRAILRRWEPLYQAAGWGIGVCDAD"
CDS complement(11088..11453)
/ID="QRZSKLUB_CDS_0018"
/transl_table=11
/phrog="1528"
/top_hit="No_MMseqs_PHROG_hit"
/locus_tag="QRZSKLUB_CDS_0018"
/function="lysis"
/product="holin"
/source="PHANOTATE_1.5.1"
/score="-14.075140203562874"
/phase="0"
/translation="MFRMFWTLAVSILLLLAIAYLAPQQLEVSLYKVSLVTIAAVAGYW
IDRELFPYSRPDTFIVVDEPEPEADKVKPADPPGTDGVLVSSIPLTWSAFDLLFAAAQL
RRAIIVGCAMLAIGLGA"
CDS complement(11514..11927)
/ID="QRZSKLUB_CDS_0019"
/transl_table=11
/phrog="836"
/top_hit="No_MMseqs_PHROG_hit"
/locus_tag="QRZSKLUB_CDS_0019"
/function="transcription regulation"
/product="late transcriptional activator"
/source="PHANOTATE_1.5.1"
/score="-42.78768673401984"
/phase="0"
/translation="MTPPIDLPGVLSDIARIAGFPAALAIAERWGGTRLYVPMADKLES
DHPLVEVVGIDAARAIAEYLGGDRPEIAKADRYMTLCRNVLIRTERAQGYSQAALALRH
HLSERQIRNILGEAEEFSPNIDLFESCGGNVSA"
CDS complement(11924..12541)
/ID="QRZSKLUB_CDS_0020"
/transl_table=11
/phrog="286"
/top_hit="No_MMseqs_PHROG_hit"
/locus_tag="QRZSKLUB_CDS_0020"
/function="unknown function"
/product="hypothetical protein"
/source="PHANOTATE_1.5.1"
/score="-312.0402491818318"
/phase="0"
/translation="MNNHDHEKQRLIKLIHVGKRDLKLGDDVYRLLLKSATGKMSTTAM
TVPELEKALEAMKSKGFKVQHKTRPHPNPSPKGRGATSRPVDQSAMVSKLRALWLDLHA
LGVVRDPNESALASWASNSRSPNVTAALQLLDYDQLERAIERAKQWRARYLLGGELYCP
ACGVRTAMSLRLTRQYPGVCCGVCDANPELVWRRKDGGMQAA"
CDS complement(12538..12723)
/ID="QRZSKLUB_CDS_0021"
/transl_table=11
/phrog="No_PHROGs_HMM"
/top_hit="No_MMseqs_PHROG_hit"
/locus_tag="QRZSKLUB_CDS_0021"
/function="unknown function"

/product="hypothetical protein"
/source="PHANOTATE_1.5.1"
/score="-1.6831346146593364"
/phase="0"
/translation="MQGETTRKGGRPGWRPGPDELASNGAGRSIAWGGIVRPRSVSHCI
VLLSLALCSKANQGFL"
CDS complement(12798..13373)
/ID="QRZSKLUB_CDS_0022"
/transl_table=11
/phrog="4652"
/top_hit="No_MMseqs_PHROG_hit"
/locus_tag="QRZSKLUB_CDS_0022"
/function="unknown function"
/product="hypothetical protein"
/source="PHANOTATE_1.5.1"
/score="-631.5168317758735"
/phase="0"
/translation="MLIQATITGTTPLLMNRFTEEAERKVSSGTSAVSIGTKGTPREQA
DPKVYRDDKGGLYIPGPNLFAALIAAGTFHKVGKSKVTTTKTSLVPAGLAVEEIALPLG
TKEFEVDSRSVVIPSTGGRVMCHRPRLDQWHVSFTLSVDTDIFSPQFVRLLVDDAGKKI
GLGDFRPARKGPFGRFVVTKWQETKEKK"
CDS complement(13392..14045)
/ID="QRZSKLUB_CDS_0023"
/transl_table=11
/phrog="846"
/top_hit="No_MMseqs_PHROG_hit"
/locus_tag="QRZSKLUB_CDS_0023"
/function="unknown function"
/product="hypothetical protein"
/source="PHANOTATE_1.5.1"
/score="-869.2007036254149"
/phase="0"
/translation="MTSTWVFRRYDMATAEQIHGGFWRDAEGRLIPENLIKPIDKEREA
LVTALTAAAKEMSEALARFKSKAFADIGAFVELSAEQYGVKLGGKKGNVTLMSFDGRYK
VIRQIQEHLVFDERLQAAKALIDECIQAWTEGSRDEIKALINDAFQVNKEGKINTGRVL
GLKRLDIRDERWQRAMQAIADSVQVAGSKPYFRVYERVGDTDQYQAISLDIAGA"
CDS complement(14023..14184)
/ID="QRZSKLUB_CDS_0024"
/transl_table=11
/phrog="No_PHROGs_HMM"
/top_hit="No_MMseqs_PHROG_hit"
/locus_tag="QRZSKLUB_CDS_0024"
/function="unknown function"
/product="hypothetical protein"
/source="PHANOTATE_1.5.1"
/score="-5.352316451683727"
/phase="0"
/translation="MKTIVFAGMAMIFLFGLGTGLFLGAVLDINLVGRAERKAQARHPA
TDDINLGI"
CDS complement(14181..14471)
/ID="QRZSKLUB_CDS_0025"
/transl_table=11
/phrog="1273"

/top_hit="No_MMseqs_PHROG_hit"
/locus_tag="QRZSKLUB_CDS_0025"
/function="unknown function"
/product="hypothetical protein"
/source="PHANOTATE_1.5.1"
/score="-28.19072542770784"
/phase="0"
/translation="MNLHTSVPWSVCTGKIETSLNKGRLIQAVWAGNDVVAICENQANA
TLIAAVPQMLGALRTVYAYLEDPHPSDAFELDHIIALLRNTIDQATGELSE"
CDS complement(14468..14986)
/ID="QRZSKLUB_CDS_0026"
/transl_table=11
/phrog="2161"
/top_hit="No_MMseqs_PHROG_hit"
/locus_tag="QRZSKLUB_CDS_0026"
/function="unknown function"
/product="hypothetical protein"
/source="PHANOTATE_1.5.1"
/score="-79.69625185290731"
/phase="0"
/translation="MGARLKLACPSCGALMSLDVVVAHDGAREAVQIALQLPAPMGKLL
IQYITLFRPAQRQLTLDRLASLLGELLPLIQSGQITRDGRIWAVPAEVWPAALQEILDR
DRAKPFTRPLKSHGYLLEILVGKASKAEARSEVLTEERRRNGGSGSAAKRQGMADVAGH
LSNLKGALK"
CDS complement(14971..15207)
/ID="QRZSKLUB_CDS_0027"
/transl_table=11
/phrog="No_PHROGs_HMM"
/top_hit="No_MMseqs_PHROG_hit"
/locus_tag="QRZSKLUB_CDS_0027"
/function="unknown function"
/product="hypothetical protein"
/source="PHANOTATE_1.5.1"
/score="-8.567099873590896"
/phase="0"
/translation="MTMIRIYHYPRLGQYGWKSVIKVGDQVREILIVGTWFRAEQLAET
AEKEMREAKRPVNTVAAAQAKQRQARRLAWGRA"
CDS complement(15204..16385)
/ID="QRZSKLUB_CDS_0028"
/transl_table=11
/phrog="296"
/top_hit="No_MMseqs_PHROG_hit"
/locus_tag="QRZSKLUB_CDS_0028"
/function="integration and excision"
/product="DNA transposition protein"
/source="PHANOTATE_1.5.1"
/score="-207324.63186935597"
/phase="0"
/translation="MLKLKRALIEAGIAQAALARDCGVSPATIAQIVNHDQWPKNALML
EKGILHALQDRGLSVDESIFRVADECCNTHQPDSNGQSPTTENEMLTEPEWLSPAAKRH
FKLARDPFHDDVNEVDDIYLGESHAFALAAMRDALRGQRFVAVVGESGAGKSTAKDFFI
ESMREEQVTVITPYVTDIDDVETRGRIFKSTQIHEAILRTLAPRQSVPRTPEARQHRSL
ELLLSNRRAGFLNLLLIEEAHALTVPVLKHLKRFWENKDGLQRLLGIVLIGQPELLAKL

DIRLQWDAREAIQRIERAQLLPIDATGELEGYLRLKCERALRPFEQLCEPDVPDAIRQV
LVGKRKDGRGGFSEISYCYPLAVNNLMTKTMNFCARIGIPKVGGEQIRKVMGA"
CDS complement(16357..16554)
/ID="QRZSKLUB_CDS_0029"
/transl_table=11
/phrog="No_PHROGs_HMM"
/top_hit="No_MMseqs_PHROG_hit"
/locus_tag="QRZSKLUB_CDS_0029"
/function="unknown function"
/product="hypothetical protein"
/source="PHANOTATE_1.5.1"
/score="-28.759344147634042"
/phase="0"
/translation="MDDEGEIKDEMKAAMFTLAQHMPELCAISVWRINGVTCIEIVCEG
EVKRVQYMRGRDAETQARTH"
CDS complement(16569..18374)
/ID="QRZSKLUB_CDS_0030"
/transl_table=11
/phrog="310"
/top_hit="No_MMseqs_PHROG_hit"
/locus_tag="QRZSKLUB_CDS_0030"
/function="integration and excision"
/product="transposase"
/source="PHANOTATE_1.5.1"
/score="-2660272.926823831"
/phase="0"
/translation="MAIVEPYSVTLLDQFRALARRLDAAPHRQRGELIQGFLAEHGWAD
ANRVYRGLRKVGWSSGRKTRSDAGSTALDEQVLRELGAAIRPGIRKNGKVTMEVPNAIS
LMASNGRAIPVGASRVRTLLRRAHLDARSAALDTPHVPMSSLYPNHVHQTDPSLCLLYY
APNGEQRVLRDEEIHRNKPAWVERIGNLKCWRYVLTDHMSSVVIVRYYQAKGETQTNLY
DFLLYAWRQLDALGCPFHGVPELLVWDKGSDAGGAIHNALRALQVETWAHARGQARAKG
QVECANNIVEKLFESRLKFEPVRNVDELNTAAEAWYRAFNANAIPHYDSRLKRIGLAQP
QARYALWQTIRQEQLRTLPDDELCRYLLTAEPEPRKVRATGAGGLTVSFRHPSAKQSLV
YDLSGIAGVFRDMTVTVSPLVYGANQVLVTIADYKGDEQTHVIDPVAFEQTSGFRADAP
VFGQEFRALRDTPIEEAAKTADRTAYPGRSDEEIEKAKARNAAPFGGLNAHSHLREVYV
PEYMKRRGEVVEVAHPVRVAEQILTVTDICARLASVMGRGWRPEYYAAIKDRCPEGAPE
RELATLRAWLEQPAEAPARPALRAV"
CDS complement(18378..18809)
/ID="QRZSKLUB_CDS_0031"
/transl_table=11
/phrog="1748"
/top_hit="No_MMseqs_PHROG_hit"
/locus_tag="QRZSKLUB_CDS_0031"
/function="unknown function"
/product="hypothetical protein"
/source="PHANOTATE_1.5.1"
/score="-56.478225435915"
/phase="0"
/translation="MSVRELRLALRKKDEDHAKAIEKLKTADAGKQRLLESKNLQIDDL
QQKLIERESAVPSWPAVVNGINLDVTVAAGETLQGFDRLTRQLERILTVELPGEPADHD
AALRMMAVHLYDLADQVMQAAQGFADACEANLSGYKTLG"
CDS 18894..19157
/ID="QRZSKLUB_CDS_0032"

/transl_table=11
/phrog="10408"
/top_hit="No_MMseqs_PHROG_hit"
/locus_tag="QRZSKLUB_CDS_0032"
/function="unknown function"
/product="hypothetical protein"
/source="PHANOTATE_1.5.1"
/score="-27.52256162360741"
/phase="0"
/translation="MKKRFTEEQIIGILKEAEAGLKVAELCRKHGLSEATYYNWKAKYG
GLTVSDAQRLKALETENARLKRLLAEAMLDNAALKEVVGRKW"
CDS 19193..19999
/ID="QRZSKLUB_CDS_0033"
/transl_table=11
/phrog="157"
/top_hit="No_MMseqs_PHROG_hit"
/locus_tag="QRZSKLUB_CDS_0033"
/function="integration and excision"
/product="transposase"
/source="PHANOTATE_1.5.1"
/score="-10566.648501366277"
/phase="0"
/translation="MTKHQMGVTRACGLIGISRSLYRYEAKRPVDQELKERLCELAAQK
RRYGYRRLHVLLCREGWEINRKRTYRVYHEAGLMVRKRKRKRIAGVERQIKVAPSAPNE
SWSMDYVSDGLADGRRLRCLNIVDDFTKQCLAIEVDTSLPGRRVVGVLQRLAEIRGLPK
SVTVDNGPEFAGKALDEWADSQGLCLSFIQPGKPQQNAYIESFNGKFRDECLNEHWFVS
MCHARQVIEEWRREYNEQRPHSSLAYLTPDQFADTFLTADSMSVSD"
CDS complement(19996..20463)
/ID="QRZSKLUB_CDS_0034"
/transl_table=11
/phrog="1748"
/top_hit="No_MMseqs_PHROG_hit"
/locus_tag="QRZSKLUB_CDS_0034"
/function="unknown function"
/product="hypothetical protein"
/source="PHANOTATE_1.5.1"
/score="-114.54561398355932"
/phase="0"
/translation="MGREKLPETEMPNLPALPEEQLIANHNRAVLDAAETDRQYGDILP
YQLDRVTQEIRFLLNQSAEAMLEAGRRLIQIKDREPRGEFMDAVQKIGIDQFVASKLIR
AARKFSNLRSNANLMALGKTKLIELAVLDEDEIDELANDQEVRGLVTCSPI"
CDS complement(20465..20590)
/ID="QRZSKLUB_CDS_0035"
/transl_table=11
/phrog="No_PHROGs_HMM"
/top_hit="No_MMseqs_PHROG_hit"
/locus_tag="QRZSKLUB_CDS_0035"
/function="unknown function"
/product="hypothetical protein"
/source="PHANOTATE_1.5.1"
/score="-3.3059056680771883"
/phase="0"
/translation="MSDFQMCVQTHICVQTQFGFVHRIGAIEMRFVVEILNGIGG"

CDS complement(20590..20919)
/ID="QRZSKLUB_CDS_0036"
/transl_table=11
/phrog="1368"
/top_hit="No_MMseqs_PHROG_hit"
/locus_tag="QRZSKLUB_CDS_0036"
/function="transcription regulation"
/product="transcriptional regulator"
/source="PHANOTATE_1.5.1"
/score="-41.966966327178206"
/phase="0"
/translation="MNEKIKNSRRKKTIESGARILRVLKMLKGHTLTGLSNTELAKALG
DSPCNVNRALNTLIAEGLAVKLDNGRFAHSVGMLQIAQAHADHVTKLQGRILELNQRIA
AGASY"
CDS complement(20916..21095)
/ID="QRZSKLUB_CDS_0037"
/transl_table=11
/phrog="No_PHROGs_HMM"
/top_hit="No_MMseqs_PHROG_hit"
/locus_tag="QRZSKLUB_CDS_0037"
/function="unknown function"
/product="hypothetical protein"
/source="PHANOTATE_1.5.1"
/score="-4.502162033379821"
/phase="0"
/translation="MSTNGMNKLMRLKARKQAAKAAAQQAWKRLAAAQREYERLDSARI
RAAIEYRDYAERQV"
CDS complement(21092..21271)
/ID="QRZSKLUB_CDS_0038"
/transl_table=11
/phrog="No_PHROGs_HMM"
/top_hit="No_MMseqs_PHROG_hit"
/locus_tag="QRZSKLUB_CDS_0038"
/function="unknown function"
/product="hypothetical protein"
/source="PHANOTATE_1.5.1"
/score="-8.938789017308503"
/phase="0"
/translation="MSINEEKELIRLNMRKYAALDDLDEARRKLSEAQREYACKRKAHQ
KAVQEYLDYAERRV"
CDS complement(21261..21779)
/ID="QRZSKLUB_CDS_0039"
/transl_table=11
/phrog="101"
/top_hit="No_MMseqs_PHROG_hit"
/locus_tag="QRZSKLUB_CDS_0039"
/function="transcription regulation"
/product="CII-like transcriptional activator"
/source="PHANOTATE_1.5.1"
/score="-49.39344731649085"
/phase="0"
/translation="VLGKINIFRVLTVRNRNWSRVQPLNALHAIKLCNQYARDKRNLSI
PGIENKMGVKADKVYDWQSDGNLPVRLILNYEEVCGVHFISRWLAQSAGYMTIRVPTGR

KSQARDINSLQELLTEAVGQIIKFAAGKAEANDVLGAIQTGMEALAWHRVNVEKHQQPE
FDFGGDDEH"
CDS 21822..22127
/ID="QRZSKLUB_CDS_0040"
/transl_table=11
/phrog="25550"
/top_hit="No_MMseqs_PHROG_hit"
/locus_tag="QRZSKLUB_CDS_0040"
/function="unknown function"
/product="hypothetical protein"
/source="PHANOTATE_1.5.1"
/score="-11.1983624722244"
/phase="0"
/translation="MQSRCVCRECPHFNADRGSIMNTAEFTELAGRIEGLGAAFLCLVA
ELENRRAMDGSRFTSGLHRIAGELCFEQPHLEETKRTVRELAAELDAMRERRIHGR"
CDS 22142..22381
/ID="QRZSKLUB_CDS_0041"
/transl_table=11
/phrog="No_PHROGs_HMM"
/top_hit="No_MMseqs_PHROG_hit"
/locus_tag="QRZSKLUB_CDS_0041"
/function="unknown function"
/product="hypothetical protein"
/source="PHANOTATE_1.5.1"
/score="-15.278369350461633"
/phase="0"
/translation="MKPQGLMVMIAPSVVKQGNQRLIGLRALSARRLTVCFWRWGINVF
ARIYGRVSIVLLGRRFVVGFSHALIRAYRPERAV"
CDS 22383..22604
/ID="QRZSKLUB_CDS_0042"
/transl_table=11
/phrog="No_PHROGs_HMM"
/top_hit="No_MMseqs_PHROG_hit"
/locus_tag="QRZSKLUB_CDS_0042"
/function="unknown function"
/product="hypothetical protein"
/source="PHANOTATE_1.5.1"
/score="-4.298849825935805"
/phase="0"
/translation="MQHWPMALRGLGSTPQPTQSQHLPLMAKRKEAGLRRAFSMGLRRS
ISPAARFSAIAAWAFSRGDRLVMTGPAE"
CDS 22693..22809
/ID="QRZSKLUB_CDS_0043"
/transl_table=11
/phrog="No_PHROGs_HMM"
/top_hit="No_MMseqs_PHROG_hit"
/locus_tag="QRZSKLUB_CDS_0043"
/function="unknown function"
/product="hypothetical protein"
/source="PHANOTATE_1.5.1"
/score="-1.788298247972234"
/phase="0"
/translation="MRPLSRSRTRRGSNDFALAKDSTVTPLESSSLRSCSGV"

CDS 22892..23740
/ID="QRZSKLUB_CDS_0044"
/transl_table=11
/phrog="4"
/top_hit="No_MMseqs_PHROG_hit"
/locus_tag="QRZSKLUB_CDS_0044"
/function="transcription regulation"
/product="transcriptional repressor"
/source="PHANOTATE_1.5.1"
/score="-10417.651900844008"
/phase="0"
/translation="MMSPYGLFVNRLCAHMDTIGFRLRKEREALGFSQEEFAEKGGVQR
RAQVRYESDERCPDGHYFSAIAAVGADVNYILTGEKKEIAEKLKGVGQARDVVVEAVLK
RSGLDREMLADLDRKVFDSNLSAADIEKVLEERFPTSAVVMVPRYDVHGSAGGGAVNHT
EHVIEYVAFDPAWVREVLRVSPDKLCLIGVRGTSMEPTLLNGEVILVDRTLSEIRDSDV
YVIEYEGNLLIKRIQLRLDGTVVIKSDNTAYEPEILTSEDAQRLSVVGRVVPWKFGRFK
L"
CDS 23713..24213
/ID="QRZSKLUB_CDS_0045"
/transl_table=11
/phrog="220"
/top_hit="No_MMseqs_PHROG_hit"
/locus_tag="QRZSKLUB_CDS_0045"
/function="head and packaging"
/product="minor head protein and DNA pilot"
/source="PHANOTATE_1.5.1"
/score="-125.69218858290807"
/phase="0"
/translation="MEIWTIQAVDKEMNHLEEAFWKIADQLIVDRARLEGLWPQFEKHR
NDPKRTYSIIEQCLPEGTWEWPEFIAHQASKGVTPTFKEMCKMLYSRIERVSGALRTAD
QISHVIVRTPYLLFRAGLEPRPECRAADGRVEHYTNEFWEHHTPPCDWLECSCRIFVDR
KMR"
CDS 24315..25436
/ID="QRZSKLUB_CDS_0046"
/transl_table=11
/phrog="4225"
/top_hit="No_MMseqs_PHROG_hit"
/locus_tag="QRZSKLUB_CDS_0046"
/function="DNA"
/function=" RNA and nucleotide metabolism"
/product="endonuclease"
/source="PHANOTATE_1.5.1"
/score="-882124.011532604"
/phase="0"
/translation="MLQHVEHVKNVGPHCDTEETTKQALILPVLDVLGFSPYDPTKVKA
EFAADFPGVKANERVDYALFCNGVPVMFIEAKSYTENLSNHAPQLSRYFNATPEVTIAA
ITNGREWRYFTDLVDKNVMDKEPFLTVDFSSLRDADMEQLGRFRHDRFQPEALRTLAEE
SVFLAAFKAVVKRSVLEGDADFVRYVAGKSTIQRTLTAKFLEQITPIVKQAVAQTMSDM
VVNSLSAPSLPEPSATPQSTDSFDDDIIDPSNSKIVTTAAERALLGIVLDLLAGEPLSG
KDTESYFSVLYSNKVNRWLLRYTGNKRRPVVQFIVPLTPQHRSEIERAGLEIGSGNQIL
LDKPEYLMRISGLLFDALAYCKDDDNFKLKKEK"
CDS 25555..26361
/ID="QRZSKLUB_CDS_0047"

/transl_table=11
/phrog="15511"
/top_hit="No_MMseqs_PHROG_hit"
/locus_tag="QRZSKLUB_CDS_0047"
/function="unknown function"
/product="hypothetical protein"
/source="PHANOTATE_1.5.1"
/score="-1355.9441748306956"
/phase="0"
/translation="MKSFLVVGFLCVFLGAAHAAGPTFPTTHPFDAIYQRLHFGDNESD
PFDAYFWWENGNLWMEADPLPSAHVCSQNFTFVQDGSQYNVALRNKSDDTFCGTLYINA
VFRMVDRRNGAPFNPVLGGKIFVDELDKTQSIDMPAGPAPSPDPTPEPSPTPSPTQAPT
PTPEPTVAPTPAPTVNPTPAPTLGPCPTPAPTGTPDPTPEPTPAPIYDKPQADVYDIDI
KKVVCQRKNGKKFPARQLPGPGNFVCDGIPINAGEKVTITVVGTKK"
CDS 26398..26511
/ID="QRZSKLUB_CDS_0048"
/transl_table=11
/phrog="No_PHROGs_HMM"
/top_hit="No_MMseqs_PHROG_hit"
/locus_tag="QRZSKLUB_CDS_0048"
/function="unknown function"
/product="hypothetical protein"
/source="PHANOTATE_1.5.1"
/score="-1.3213195900493513"
/phase="0"
/translation="MRALCLPDSVARPRTAFEDLERGVWDGPCRAVYSSHS"
CDS 26603..27685
/ID="QRZSKLUB_CDS_0049"
/transl_table=11
/phrog="976"
/top_hit="No_MMseqs_PHROG_hit"
/locus_tag="QRZSKLUB_CDS_0049"
/function="head and packaging"
/product="head maturation protease"
/source="PHANOTATE_1.5.1"
/score="-2646.329872579589"
/phase="0"
/translation="MSQTTPQPIESLKPGRFTAMSGQVVEFSESDLAAMAAAYDPAVYR
APIVAGHPKHDDPAYGWVDALRYSPDTQRLLSVPGDVDPAFAEMVRSKKFRSVSLSVYL
PDSPANPKPGVFYPRHLGFLGAMPPAVKGLREASLSDSEEGVIELSEWDDVTNAGLWRR
LREWLIGQFGQDTADRVIPGYDVQSLEQAAQQEVAEAAAEDAGAQPAFSESQPEVTPTM
TAQTTDLAEREAEIARREAELQRREAAATRQAHADFAESLVQAGKLPPADKAGLVEFMA
SLDGEATLEFSEGEETVTTQSSAWLKSFLSRRLPKTIEFSELSGGAGTGDEISLSEDER
KTCEALNLTAEEYLAAKKGA"
CDS 27687..28103
/ID="QRZSKLUB_CDS_0050"
/transl_table=11
/phrog="1731"
/top_hit="No_MMseqs_PHROG_hit"
/locus_tag="QRZSKLUB_CDS_0050"
/function="head and packaging"
/product="virion structural protein"
/source="PHANOTATE_1.5.1"

/score="-17.85992466539868"
/phase="0"
/translation="MTALAAERNTKERIGDVFDLPVKANTKCYLGGLAVIDAGYAAPGR
TATTLVAAGRFEESVDNTGGAAGDKKARVKRGIYKFANSAAGDLIAQADVGADCYIVDD
QTVAKTNGTNTRSRAGQIVAVDSDGVWVQIGLGW"
CDS 28116..29012
/ID="QRZSKLUB_CDS_0051"
/transl_table=11
/phrog="371"
/top_hit="No_MMseqs_PHROG_hit"
/locus_tag="QRZSKLUB_CDS_0051"
/function="head and packaging"
/product="major head protein"
/source="PHANOTATE_1.5.1"
/score="-2794.187567634167"
/phase="0"
/translation="MQITPQTLKALQNGFNAAFLKGFNSAEAQWPLIAMEVPSQSALEN
YGWMKELPGMREWIGQRVIHNLEMIGYQIINKHWEHTVGVDRDDIDDDKLGLYSTRFSM
QGEVAKRHPDELMWQVLLSGFNSLAMDGQYFFDTDHLGYDAAGTETSWSNYQAGASSPW
FLMDLSRSFMKPLVFQNRSTVKFVNQSNETDDNVFMNRQFRFGVDGRYNGGFGFYQLAY
ASKAALDATNYQAGRLALGTQRRPDGTALPVHVTHLVVGPTNEAAALALLNKELVSGGE
TNIWYKTAQLVVVPQLG"
CDS 29020..29256
/ID="QRZSKLUB_CDS_0052"
/transl_table=11
/phrog="6188"
/top_hit="No_MMseqs_PHROG_hit"
/locus_tag="QRZSKLUB_CDS_0052"
/function="unknown function"
/product="hypothetical protein"
/source="PHANOTATE_1.5.1"
/score="-3.437535752093949"
/phase="0"
/translation="MIRITARRAGFRRCGIAHPAEPIDYPFDRFDEGELKKLQAENMLI
VEVLDDEASESAADDPAPLKPHRGKAAPTGSAA"
CDS 29253..29669
/ID="QRZSKLUB_CDS_0053"
/transl_table=11
/phrog="195"
/top_hit="No_MMseqs_PHROG_hit"
/locus_tag="QRZSKLUB_CDS_0053"
/function="connector"
/product="head-tail adaptor"
/source="PHANOTATE_1.5.1"
/score="-22.38667247929091"
/phase="0"
/translation="MSYCTQADLEKVSAVEFLQLADRDHDSVADADVVAEAIAKADGII
DSYVRHRYAVPLAPDPAIRDCAVKLVWFTLQETNPTEAARKNRDDAVKFLRDLSEGTSH
LGPDQLAPGATVSVDVPQAEAPDRVFSDDTLAGY"
CDS 29679..30107
/ID="QRZSKLUB_CDS_0054"
/transl_table=11
/phrog="209"

/top_hit="No_MMseqs_PHROG_hit"
/locus_tag="QRZSKLUB_CDS_0054"
/function="head and packaging"
/product="portal protein"
/source="PHANOTATE_1.5.1"
/score="-27.629909787534007"
/phase="0"
/translation="MQISPVVAQLQTVPELTGRVEIAGSVRQILTELNSGKGPRWAWVI
SPGDDAGNNTLSAGGIRQRVTDRYGVVLGCYDVSDRRGEAALLKVEALGQAVEPKLLGF
IPAPGYAELTYRSGQMVGGTDGWVLWLHLWQSNHYITK"
CDS 30115..30456
/ID="QRZSKLUB_CDS_0055"
/transl_table=11
/phrog="No_PHROGs_HMM"
/top_hit="No_MMseqs_PHROG_hit"
/locus_tag="QRZSKLUB_CDS_0055"
/function="unknown function"
/product="hypothetical protein"
/source="PHANOTATE_1.5.1"
/score="-4.838799020389705"
/phase="0"
/translation="MHTSEHFGKGGSYTLNPETDQIELAEERIQMAPEFVPVVAFSDGS
ASEEQAAAAEAATVSDAPAPVTADPVAADPASLEAAQDPSATVNDTSTDRPGRRGKRAE
TDPTDGGAA"
CDS 30453..31643
/ID="QRZSKLUB_CDS_0056"
/transl_table=11
/phrog="290"
/top_hit="No_MMseqs_PHROG_hit"
/locus_tag="QRZSKLUB_CDS_0056"
/function="tail"
/product="minor tail protein"
/source="PHANOTATE_1.5.1"
/score="-8491.711845480386"
/phase="0"
/translation="MSGQITRNNRVLVGKLESTYGVDANPTGAVNAIRVTDLSIKPVLG
NQKRINYIRGFLGIGRNVRTELYRSITFKVDMVSALTPGQAPPYGFILRACGMAETLLA
AAVTGTLQAGSTATTLKLPAAASAINGAYIGAKSRITGGLGSGQAASGIAYDGVSKILT
LDRPLATVPDGTSAVSIDPFARYNPISDLGTMEAATFYYYTGNKVRHKLLGCRGNAKWD
ASSKDTGFIEFEMFGLYGGVADASEASVVTTNWVDPWEVGFGRTYGQFFGKQFTGGATG
LQMGKFSLDLGHKPKYRSVVGFQGINSTDRESTGSITLDATLVAEQDIWAIVTGSTTGP
IGIEQPDTNNGSVRLDVPKADPTDADDGDDEGISINNIPFDCMPVLGNDELYYTCR"
CDS 31658..32068
/ID="QRZSKLUB_CDS_0057"
/transl_table=11
/phrog="313"
/top_hit="No_MMseqs_PHROG_hit"
/locus_tag="QRZSKLUB_CDS_0057"
/function="tail"
/product="Tail assembly chaperone"
/source="PHANOTATE_1.5.1"
/score="-66.77341342218824"
/phase="0"

/translation="MAFKLGTQEALDKPIARKVLLPVCNEAGTPVNHQITLLYHRMTVE
EIGAAYAALGEDAKLLEELSGPALTAAVLDMHANHVLRLAVGWKEVQADDGTDAAFTFD
NVRALLNAVPKAYDKITEEFQKANRGGAALGN"
CDS 32131..32457
/ID="QRZSKLUB_CDS_0058"
/transl_table=11
/phrog="680"
/top_hit="No_MMseqs_PHROG_hit"
/locus_tag="QRZSKLUB_CDS_0058"
/function="tail"
/product="tail length tape measure protein"
/source="PHANOTATE_1.5.1"
/score="-14.704834536802833"
/phase="0"
/translation="MEGFGLTQAQEEAAPADTDFYVMEDNWDTVMAFLACRTRWQREFP
PLGGPAIWLGLNYSDVEVVIRNLGHEGEAARQIFAGIQVMEAAALAVFHPPAVVSTAGE
QADE"
CDS 32575..39129
/ID="QRZSKLUB_CDS_0059"
/transl_table=11
/phrog="339"
/top_hit="No_MMseqs_PHROG_hit"
/locus_tag="QRZSKLUB_CDS_0059"
/function="tail"
/product="tail length tape measure protein"
/source="PHANOTATE_1.5.1"
/score="-4.47353219438996e+24"
/phase="0"
/translation="MAGPIVVGIKLNLDEKGALVGVEKVESGFKRIGSAASESASRIDA
SYKKVETGIAEVENGFKRVGSAANESAGRIDAAYGKTRQGLDSISTQLATAKRQFLEFI
TAQAALGAGRELADVADRYANLSARLKLATQSELEYAKAQAQVFAISQRYSTALDDNAK
LYSRIAPAARDAGKSQADLIKVIEGVNASLKTSGASAAEQSSTITQLSQSLASSTVQWE
DFGQLADTNMRLVDAVAKQLGGSMGDLKQKMSDGLISNTQLFDAIIAASEQLKAEAATM
PNTIGGAVQQMSNAWEQYIGKADQAAGASRQVADAISGIAGDFDDIADAAIHVGEVITA
VLAVKAVQAIGSYVIAAEGATLATRTLGVAVSALGGPWGIAAAAITTGGIALHEFMAQQ
QAAMKAQSLLEAQMNVRDLVMAGPDSSGLFGATAMDVAKWQKELAAARAEADALAKSAS
SAGQAAVQSGQAMAGAADAVKKYTATEREDIQSKVRWLEAHGQQTDALRLEGVLHGFSG
KALEAYVTQELDTIAAKKANAEATKVHNKALSEEASTVKKAATEHQNAIEASDKFIQSL
KDQLQNIQDTTGASEAEQAARKASIGLIGKEADERSKLAKQLVEERQAYEGAERIKQLE
LKTQNDIAEAIRRKEETNAQALQSAQAEEQKQLAILAQLQAAHEAGYGPEALARMKAYL
SAVYDVTQGMGEEEAAATAAMAHTESANQMMSEMTQTGQSMLSDLGGIAQNMMGSFTS
MFFDPFGSATANMGQQFSRMLQQMASQLLQSVVMRFFTGGSSGTGSIGGLIGSVGSWFG
GSEAAAAPSVGGIVNTGAAVANWANVGDMFVSTGGGKAMPVAVVDAAGAGTRAAGTASS
WLGGPGWASTALGYAGAGIGGLGTGYLIGNAIGGQYTYGREGGMVGGAIGGMAGYAGAT
IGMEMAAGSIEAALASTGIGAIVAAIMAAVMSSIKKGQPDATAQYSWQNDSWTLNKQAE
RNGGNLAFLGNFATKLFQPLTDLEKTLAVRLPAFNLNMRQHEGYFGMQIPGLYSARMKA
DSEANKYMAQAVSIGVMRLFQQPAVTSQMADPLTKAVVMLSGDLQAFGDNIKAAEDVRK
YAAGASSFKVIGKSLDLSGGSAETFFGNMKEAGALRTFLGELTPNTDAFAKLVQSFQQM
EDIAKKFGVSVDDVHKKVDQFLKAAEYNTQDRVNQLLGKPQSIGSKVFNDMQSFDQIAQ
DQRNNVTELAKQNKAEAQRGKNLLNYGDFVSLTKSDQKEAKGLLNQLNNPELAGNPEML
AQWQAGWDAYWQKQIDEAQATIKNPKKLAKEIKRLENGRDSTDALGIGQVDIDAAKQQY
IEQQRKAYLDEAKSRAGVTVTAGFAERDKANHEFFEQARAGADALGLSLSELADLEHKA
ADQVNKDWSKALEEFAGNAPIGDAAERLQAIADREQALVADAIATGRSVVAVYEAGDQA

RKKAADDFARSQGQIAGTIDQWTAAFQGYMDNFKGWVADAKAYGTDMSVTSKAIIQTMK
DVWENMVSGIESARQAIAQQIAELRGPQAVADLAVQNVDKAKQKLADYRAGGGTDISRE
IALIQGVQSAVMARYNAELALIQQAVQKEVEAIQAKSQADIEAINETLNAQIEAENERL
NAAIEAENAALNARIEAINAASQAQIDAINQQADAAAEARQEAQQAAMDALNEQLQTAN
QLKNAIKGIADYAKSLQTAADSPLSPEARLNAAKNQYEDTLAKAKAGDAEAAARYQQDA
EAYKSAAKDYYASSAGYGQVYGQIQSDATALGNTNVTSPDSVESQLKELRKQQSEENKA
AQKAVQDQIKAVQSSAQDQIKEAQKESQERIKAMQDASKDSIAAMKANAEAQIKAIQTA
SAAEIKKAQDLDSRPDVQALKKDTITQLELLDKDLVAAYDAAAKQYQTFLDSLAGVVGA
NTTVIGSLVEWLRSQGVNVTAPPDPDPGVYEPTSSTAGGSGDPRMPPHHAAGGHYRGGL
ALVGELGPELINFNRPGYVHTADETARILAAVSGSGSAASPDDQINQMLGGLPFDPRQL
LSMFSAANDAHGAYANILDLPPTWPNAAAAVQSLMLKPTAAPQAVDMPSVGYQPPTRPT
VSRQTAQIEDRLSRIEANQSGTELKGLKEALESIRRESKAGVNVQSAGFSQLLAVMNQM
LARLEEIERRSRYAS"

CDS 39145..39783
/ID="QRZSKLUB_CDS_0060"
/transl_table=11
/phrog="240"
/top_hit="No_MMseqs_PHROG_hit"
/locus_tag="QRZSKLUB_CDS_0060"
/function="head and packaging"
/product="head protein"
/source="PHANOTATE_1.5.1"
/score="-74.83095758476317"
/phase="0"
/translation="MATKSNYLENKIIDHMFKATPFTAPAVMAVALFTVNKGQRANSTA
YVTTDYVWLTANDGKTHIYKCTTAGSTAAAQGTLYPGTANEVITDGTAVFTEQSSQFDA
NALTALEVSAGGYARVANNPSATANWLNTQGTASGASTGAGGSTSNQNAITFGTPSASW
GVVAAIGIYDAASAGNLLYWGYMTTPKTINNGDPAPAFAAGALTILEDN"

CDS 39805..42321
/ID="QRZSKLUB_CDS_0061"
/transl_table=11
/phrog="8917"
/top_hit="No_MMseqs_PHROG_hit"
/locus_tag="QRZSKLUB_CDS_0061"
/function="unknown function"
/product="hypothetical protein"
/source="PHANOTATE_1.5.1"
/score="-2974267.6967246355"
/phase="0"
/translation="VASVTLRPGTIVSATGAGSVAWSNPGNAGASDNAYATANVGTSPG
ESQWLRCTNFNFASVIPAGAIINGFTLTIERKGSAINSVYDTRLYLYSGSAAIGSNKAV
ATKWPTSDGIATYGSSSDLWGTTGLTGADVNATGFGIELAVNSSTFTTTASVDDIALTI
DYTPQIDLAAYLSSESFVLADLSTSITFQSDLQAFGGLAATLSTSIPLQAALVSESAFS
GFFSVALSADLWGQSALTADLSTGIACSANLQGNAWTAADLSTQIPLSASCAGQSSLTA
DFTTQIRFSAALSGISQAYAGLTTEIALAAVLSGNAEATAALTIGRVFIPPASQRIYLA
EITCYDPALPGIRTLRYSSGADGYDDAGTFYPPLIEQPATIRREIPADVAGGHVSASYG
ALTLVNASGALDPLRDYYYDGYELTLKVGDAGAGYGTFVTRLRATIDNASFERNRVTVR
LRDRAETLKTPLQTNTYGGTNALPNGLDGTADDLKGKRKPLIFGRIAALQPVLVNTSRL
IYQVNDGAVDAIINIFDSGAYLTRGTDYTAGQQSTFESAAVAAGSFSTYAPAGLIKLGS
KPFGTLAACVAERWDYTQISAAGVTQRMLQMRGYGSADWMAQDFLDLDAVNCGSVGVQT
EDGENVDILIDRTLGSVGAWWGFDALNRFRLRRLDAPTGTAVATITDDHILEYDPVQSS
QRALWQVTLQGDANYAVQDKANLAGIVAADRAAWWEKATRDQSSTSTAVKTTRLLSQDA
TFASVANGISQTQAEAVRRRSLFDGRRDTLGLTVAAPNDWMDLIDLGSEVTIVSAKCGY

PAGRNMIVVMTQPDFQRNRLDLILWG"
CDS 42323..43078
/ID="QRZSKLUB_CDS_0062"
/transl_table=11
/phrog="8610"
/top_hit="No_MMseqs_PHROG_hit"
/locus_tag="QRZSKLUB_CDS_0062"
/function="unknown function"
/product="hypothetical protein"
/source="PHANOTATE_1.5.1"
/score="-774.5138364744919"
/phase="0"
/translation="MQPIVIGYANLIDIATLSGPGWQTSYPLSNLQNRYAGSKARATGL
TGVIDINLGVPDAAIRTFGIKNHNAATIRLQGSSASDFSTVAYDTTVLPTYADTSYLRA
LPVAVVAPYWRLTLTGTSANPELSRIFLGAGMQPAPENNIEWGASLRIESKTEVAESLS
GYEDFNVKPNRRVFRGRFDFLPDADAWGVLLPMMRSQGIHREYLLIADPDDTTYQAQRN
MFCRMRELSEIEDPYLNAHTVGVEFSELL"
CDS 43079..44179
/ID="QRZSKLUB_CDS_0063"
/transl_table=11
/phrog="No_PHROGs_HMM"
/top_hit="No_MMseqs_PHROG_hit"
/locus_tag="QRZSKLUB_CDS_0063"
/function="unknown function"
/product="hypothetical protein"
/source="PHANOTATE_1.5.1"
/score="-1329.414971837503"
/phase="0"
/translation="MALALYRENATGFLVRRDTPGAGYTAIAAMPTGSIAARASWYRDL
DTHGQSSAWHPSQSAPGPENAAPSVDYGNVTGTKPPADATHNKIWPPSTSAPSSGVQSG
DMWQNSNTGVFYTYNGSAWVPVASKNIIWPPSTVAPSGANAGDLWQDSSTKTFYTYNGS
LWVPVSDVTAYNTAAAITGQGSFATISQIVAGNIPALIAAGIIGANYITAGALHSQWGT
VGNSNAPYVAAGNWTAGASGCIWTNVAVSANAYRTSIVVFAFAQYGDGTRQADKPGYVN
VVVGVNSQTLTDQATNEILGGQVQKDNAPTYITMPGIALAPMMYYDVAPGTAGRTYYIG
FRHDARANGTDTMPLRYAILVMEFSK"
CDS 44189..44593
/ID="QRZSKLUB_CDS_0064"
/transl_table=11
/phrog="No_PHROGs_HMM"
/top_hit="No_MMseqs_PHROG_hit"
/locus_tag="QRZSKLUB_CDS_0064"
/function="unknown function"
/product="hypothetical protein"
/source="PHANOTATE_1.5.1"
/score="-98.06918105657226"
/phase="0"
/translation="MPNKQYRRVATRTGEILSVVSIPDTDAARDLYNTHLEEGSGDSRT
EYSVNGILTPRPPMPYVLDTDTITADGADTARMTCLPIPASIRVSGPHGLQVMTIDDGE
FEFQADMPGDYQLEIEAFPYRQAEVVIHAV"
CDS 44583..44954
/ID="QRZSKLUB_CDS_0065"
/transl_table=11
/phrog="2837"

/top_hit="No_MMseqs_PHROG_hit"
/locus_tag="QRZSKLUB_CDS_0065"
/function="tail"
/product="tail protein"
/source="PHANOTATE_1.5.1"
/score="-37.10734793549466"
/phase="0"
/translation="MRFKATLPLPELRRLKQDQVNAKRAERLAEGYAFEGAIFATDPQS
IANMTAVLAAIGAGVDLPDGFAWRDQNNVNVPMDAETFKRFAGGLLARVNQIYTQSWSR
KDEIETSDNPASIDADLML"
CDS 44973..45080
/ID="QRZSKLUB_CDS_0066"
/transl_table=11
/phrog="No_PHROGs_HMM"
/top_hit="No_MMseqs_PHROG_hit"
/locus_tag="QRZSKLUB_CDS_0066"
/function="unknown function"
/product="hypothetical protein"
/source="PHANOTATE_1.5.1"
/score="-0.1279005258681581"
/phase="0"
/translation="VLRTPGQPADPQRIPASQPKAATEWPLGVASLVDF"
CDS 45106..45879
/ID="QRZSKLUB_CDS_0067"
/transl_table=11
/phrog="56"
/top_hit="No_MMseqs_PHROG_hit"
/locus_tag="QRZSKLUB_CDS_0067"
/function="other"
/product="DNA methyltransferase"
/source="PHANOTATE_1.5.1"
/score="-1393.1016098587534"
/phase="0"
/translation="MQNAKPIVPWIGGKRRLARHILPLFPPHECYVEPFCGAAALFFLK
EPAKCEVINDINGELVTLYRVVQHHLEEFVRQFKWALTSRQIYEWMKATPEETLTDIQR
AARFFYLQKLAFGGKVESRTFGTSTTSAPRLNLLRLEEELSAAHLRLSRTTIEHLPWEE
CIRRYDRTHTLFYLDPPYWSTEGYGVEFGLENYVRMAELARTIEGRMVISVNDIPEMRA
AFEGLPTKHAELCYSVGGGKRSRGSSGELIIRSWA"
ORIGIN
1 ggtgacattg gctgtagaga tcaaaatgct taccaggaag agtaattgga tgaatggaat
61 tccgccgacg cgagatgaac cggccggcca agaaagggtg aagcgtggat atgggaaatt
121 atgcctgtat ccggcagaaa acggtggaat ttccggcgat cactcccagc ggcgtcccct
181 tgcgcccttg cagatgaatg cgtccgcagc cgcagccgga aaactcctgc cggcggtggg
241 caccttgaaa ggattggcac aatgatctaa aaacaaaaaa atccacagca ttgacggcag
301 atggtacgga tatccaaagc tctggatgtg ggcggacgct accgattcgt tggctgacga
361 cggatgccat ccgcccgcgt ttaacgataa cactctggaa cccgaggagc gcccggaacg
421 aaccagggcg gcattggcac cgtaaaaccc ggttgccacg gaccagccac atttctgtcg
481 aacatcaggg gaaagcacgc tcatgaaacc ggaatcgccg cgattgaaaa cacccctgct
541 ccggcctttg ctctggtgct gcctcagcct gccggcctgg cccgcccagg cggcaaccga
601 tgccgcattc gcggcgaaat ccgatgcatc gagcgagata ctcgccgaga tcggccagca
661 cggattttcg agggttctcg tggaattccg gcgacccgcc accgccagtc ggctcacccc
721 ctcaggcgcc ggcctggaaa cctataaggc ctcgatcaaa gacggccggg acgcgatcgt
781 ctcatctctt ttcggcaacc cggatcatcc gatggacgga cggggctttg cgcgggcgtt

841 gcgaaatttc gagatcagcc cgatattcgc cgtcaacgtc gatcaggcgg aactcgaagc
901 gctggccgcg gatgcccgcg tcatcaggat acattacgac cggcttctcc agcccacgct
961 gaacgacagc gtgccactgg tcgggatgtc cggcccggcg ggcgcctatg tgaaggggag
1021 tgagataaat gaacgggaat gtgtgagata aacgggaaga acagggatga agaaggaaga
1081 aacggaacgt caaaacgaaa aaacgtttca cacgacgcaa caacgcaaat cagccgatcg
1141 cggcctgaat gtgctcctca agaatcgagg cgatctcttc ccggtcggca tcggacaagc
1201 cgacaaacgg cctcgctggg atatcgcccc agggaatcgg atgtcctttc tttgagccgg
1261 catggcgcct gaagtcgttc ttgtcgtact tcaaacgcga cagctggtag taccttccga
1321 actcgcccat ctccgcaccg aactgatgaa cagccccgta gattctgtcg gtaccgaaca
1381 gcaggccatc gtcctcgatc tggtagtgaa gggaattgcg cagatgcccc tcgagcacca
1441 gcaccttgtc ccggtgttgc ggcttgatgg cccgatagga ctctgacaaa ggcgcccact
1501 tcgagccatc cggcgcttcc cccgcggcaa aacgctcgtc gtgggaccgc tccagatatg
1561 cgccgatgtc atggagcgct ggctccggat tgccgatcag gcgcgtgagt tccgccaaag
1621 ccttcctggc gttttggtca tcgacttcaa tgaacgcgcc agccatcagg ccaccgcgtc
1681 ggtgggcaga tcgtccgtga gctccggcaa aaggatttcc agctcggggg gcaagtgata
1741 cccatagctg gcaaagatca gggcaaacga ggcggaattg tggagcggat cgggggcgtc
1801 catctcgggc catgcgggta gcgtgacttt gccgtcggcg cgctcgacca tccggcgcgc
1861 ccgatcggca tcggggccag tcaaggtacc ggtgtcgcta tcccaggaaa acgacagcga
1921 ttcaccggcc agatgatgag acaaggtcag gtcgagtttc atggccggaa atggaacaac
1981 agaccgagtg ccactttgag catctcgcga tcgaatatta gcatctttgc cagcaattca
2041 tccgcgaatt tgtccgtacc caagaggccc tggaacgcca tggtcatgac ctcaagcgcg
2101 ttgtgtccct cgtactcccg cccctggtag cgatgcacat actgatcggg ccgtccacgc
2161 tcgctcttgg cccaggcgaa caggatttcg gaagggtcgt tggcggtcct gcgccgatgc
2221 tccgcctgaa agatggcatc cagctccggc atcgtgcgtt gcaggtggtg cgtatattca
2281 tgctccgcag tactgctggc gtcggtgcgg atgaaggctt cgaaccgacg ctcggcggaa
2341 ttccactgtt tccacgagta gtagcctcgg cttttggcca tctgaacccg aagcggaaca
2401 ctgttcgcgg ctgaaaccca tccgtcgggg aaccgacttg ccgcccgcac gagtacttgc
2461 aggccttgcc ccgagcggtt ccaagtcgct ggacgaactc cgccgaggct ctgtcgggta
2521 ttgaggtcct cgaacagaat cgcgcggatc tggcccgccg agggagctac ggcctcccaa
2581 ggcgcatcca ggacgccggc aatcgttcca gatcccagcg atttgcccaa ccattggtcc
2641 aaccgctctt ggcccagcgt cttgacggtc tccagatcca gttcgtcctt gctgagaaag
2701 cgccatgggg gtgggggctg ctccgtttcg gccttcaatg acttgccaat ctcttttggc
2761 agtttcgcca ccttccgctc gatctccttg cgcatcaaat ccgtgcgcga agcgcccggc
2821 gcgtagtccc agccgggatc gatgccgttc ggcacccggt gcatttcgcc agtcaccttg
2881 tccgtccagt cgtagctgcc atcgtcgggc ggggtatccg gtccggattt ttccatccgc
2941 cgcagatccc gctcggacag tgcgaacatc cggcacttgc agccccagcc gttgggcggc
3001 caatgggttt gcacccaggg gtcgtcgacc gcgaccacca gcccgtccca ggatttgtgc
3061 agcgggcgcg gatgggcgga cagatcggag tgcttgtatt ccaggtacgg ccgccactgc
3121 ttgcccgcct gcacctgcgc ccagcggccg gcctggtagg aagtgcgcag attggtgtcg
3181 tagatcagcc gggtgcgcca agccctgccg gcatcggatg attccccctt ccagcctgtc
3241 cagccatgac gggccacgat cgtatcgaaa tccttccgga attccgccag cgtcgtaccc
3301 ttggccaccc ccttgagcac agccccatgc aggtcggcca gcaggtccgc cttggcagcg
3361 ccggccacca tgaagccgac atcgtgctct gccttccaga cgtccgccca gcggtcggtt
3421 gggaccaggt ccttcccgcg gaagaaccgg atcgcctcgt cgaatggcag ggagccgtac
3481 tgggcggaca tcagccgacg acggtccagc ccttgagcgt tgcgatcgag cggtcacagg
3541 ccggatcgga cgtcccgaaa ttgcccgtga tgtcgatcgt ctgcccggtc accccggccg
3601 ccaaaccggc gaagatgaga ttcagctcgg ctgccccgag tcgcatgttc ttcaactcaa
3661 tactcgattt tgctcccgtt gcctcaacct tcgaaatcga cgtgcagccg ttgaacgcaa
3721 acgaattatc gttgctggac gagaaatcgt aggcgggaac atgcgtcaac gagtagccgt
3781 tgttgagcag cagcttgcag ttcgtagact gactcaagtc aagcatgggg ccgaactgaa
3841 gattgtgcag gttgtcgaac atggcctcgg tgtctacaag cgccggcgta ttgaaatctg
3901 gacagagctt taccgagttg cacgccgaaa acatgtagcg tgccgtttgc gcgtttgtca
3961 ggtcgtaggc cgggatatcg aggagagcgt ggttgttgcg gtgcatctcg ttgaagttcg

4021 tgccgttcgc ggtgttgagc gccggagctt cacggagcgc cttgcagttg tagtagaacc
4081 gggagaagtt ctgtcccatc gccgtattga ttgccggggc aaactccagg caggaacaat
4141 tctcgaaaat tgatgtgaaa gtcaaacagt tcatcaggtt gtgggcagga acagtcttga
4201 tcctgtcgca ggaccgatac atgtagctgg cgtcggttac ctgggatgta tcaaagtcgc
4261 tcactacctc caacgcggtc attgagttgt agagcgaatc cgtgctggtg acgttgccaa
4321 ccgatacgat tttgacgcgc tcgacgaact ggagactcaa gtcccagccg ccaagtttca
4381 tccacactaa gttcggcgat cccacggcaa tgtcgagcca cagcggggcg tagttctgcg
4441 tcatcgacgg gtgccggcgc tgaagttcta ctcggctcag ggtcgtccaa tcacgaggcg
4501 ccaccgtgat gatgacctgt ttgtagccac ggcttgacag cgtatttgca gggaccgacg
4561 catagtcgta ttgatgggat gcgaccgtga acgaatcgtg gttctcgacc gtgccgtcgc
4621 cccagtccac cgtgtagtca ccttgagcga gcagggcaac gaaattggct gagtcgtcgg
4681 tgacaccgaa caacccgata aacttctcct ccgcgggtcc aatagccggc aacggcagcc
4741 agtccggatg cctggcccac gacgcgacgg ccgatccagc gcttctgcgc tgactgccga
4801 gcaagccgcc gccgagtaga ttcatgccgt cacctcgaat tcgtcggcgt cgctgccgga
4861 tacgcggata aaccggatcg cgtgacacgc accttcgatg gtatcgatag tggggccggt
4921 gacatccccg gaaccgggcc accacttcgc tgcagccggg ttgccggcgg caccgggggt
4981 tgccgaatat tcccagcgcg atgtgttgcc gggtccggga tagagcgcga ctgtgaccgg
5041 cggattgacg ccggaaacgt caacgatctt ctgcccggaa aagacaccgc tgtcgttgat
5101 ctgctgtccc atgttcattc cccttgctga agttcgtacc gtcccgccat gccagccgca
5161 aacatcccct gcgccatgaa ctccgcgaaa tcgcgggcat ccatatcggc atacagaccc
5221 ggcaataact ccgcgaattc agacagcgtc tggccttcgg caatggcctt gtcgagcgcc
5281 gtgcggatcg ggtcaagcag ccctttttcg atcagcggct cggcctcttt gcccaggcgc
5341 tcgacgtatt ggtcgaccac gtcgggggcg ggatcgacct cggccaaatc cgcctgagcc
5401 gggtccgacg ctggcgcagg cagcgccggc aagctgccgg tgtccggtgg cagctcaggc
5461 ttcacttcct cccactcccc gccataggtt tccttaacct ctttcagggt cggcttgaaa
5521 ccgatgtcga acaggttctt gtcgcgctcg gaacgggcct tcaggtcttc tggttcctcg
5581 acgtcgcgcc aaacctttgg ataggccgcg ccggggtagt tccagtcgac cagccagcgg
5641 atgaaggacc ggtttgccga ttcacagacc agatcggcat cggccttgac caggtcgcgg
5701 cggacgtcgg cggcattgct ctctccgccc agccggcctg gcgtcgcgtc ggtgctgccg
5761 gtgtggccca gcaccacctt gctgatcgag gcgttcatgc ggtcgtacag tgcggtgtag
5821 tcggcggtgc ccgaacgggc agcctcgatc agttctatgg acatgccttc cgggatgatg
5881 acgcccgagt cgcgctggat cgcccgcagt gcggacagaa gtctacgttt ttcctcatcg
5941 gtggcgccgg gctgataggt ccccttgccg gtgggcatgc cgaacttctc gaggaagatc
6001 agccagaact tgatgtcgtt ctttttgaag aaggtcggcc aatacagcca gtgtcccagc
6061 cccaggccgt agggttcgtc gtcatggtcg gcgccggtgg cgaataccca gaacttgcgc
6121 tcgggcatca gctcaccctg agcgttgctg ccggtcagca accgcaggcg gaagtcgccg
6181 tcgaagacga accggcgccg gttgcgcacc ttcatcccat ccagtacgag ctgggcaccg
6241 tcgcgcgccc acaggcactc ggccaccgcc cagccgtaga acaccccgta cagcatcttg
6301 tcggtgcgct ggtcccagcc gatgtgctcg agctgggcct tgatgaactc ggcggcctgc
6361 ttgtcgatcc gcttggtgcc gccgggttcg acgtcccatt cggcggaggt cacggcactg
6421 cggcgctggc cgaacgaggt ctgcacctgg tcatcgcgca gcacttctgc gtagaccttc
6481 aggtccccgc ccttggcctg caggatttcg tccgaggacg gcaggatcat gtcggggtgg
6541 atgtagccgc gggtgacgtc gcggccgtcg tgcgtggtcg caatttcgtc gaattccggg
6601 ccagcggtct ccgccagttc ggcgaagggc ggcaagggcc tgccgtgagc ggagtcgaat
6661 ggtgcggcgg ctcgagcgat gaagtcccag aagcccatct caataccct cgaagtcggc
6721 caagccagac acggtgccga agccgcggtc ggtgagggcc agctcgtttt caccggcgtc
6781 gatcacggca cgacggccgc cgatggattg gaagtcgatc ggtccgacat ccatacggct
6841 tgcgtaataa gccaaacaac ccgctaccgc gctgtcgcca tggcgttggc cctttttatc
6901 ggtctttccc tcgggtactc gcggcacgcc gcgcaccaag cggatcgccc gatgatcttc
6961 cacaacgtcg tcgtgcatcg gcagggagat cagccggtcc tcgaatagcg ccttgtatcg
7021 cggcatgttc tcccgataga acgcctccgt gagctgcagt gcctccactc gcgaaccata
7081 gcggtccacg gctgcctcgc cgatgtagga gccgttgccg ccggcgtcga ttgccagcgc
7141 accgaacctg gggagcgtat cgcacaccgc aaatagaacc tgctcctgct gcttgtgcgg

7201 cacgttgtgc atttccacta tgaatggcac cctccggttc agcttctcgc ctatctccat
7261 gggcacgatg tccgacatgt cacccttgcg cgcgaagtcc atgccgcagg cgtggcgatg
7321 gcccttgttc agcaacgcga acgccggcac cagctcgtcg ccgatccagt ccgccatgat
7381 ccgccgccgc tgcggctccg gggcttcgtt gaactggcga tcgccggaaa atcgcagcaa
7441 cggggcatct atcatgcagg cttcgattag cacccgcgct agataagcgc ccccgccgaa
7501 cgcgggcacg cagaacagct cctcgtcctc gttcgggcgg tagcgcttca cgagcgattc
7561 gcgccaggcg gcctctttct ccggggtcca ggcctgttct gacacctggc agatgcgccg
7621 ataaagccca tcggccagcg catcgtccag ggtcacccgg tgcaggctgt agtcgtagcg
7681 gccggcgcgg atatcgttga tcagctcgtt gaacgggttc tcttcgccgt tgtgggtgct
7741 gatgatgtgg atctgaccgc cccacatcgt catggccatg gccgccttga gcagctccgc
7801 cagatcgtcc acgaaggccg cctcatcgat caccaggcga tctttcgggc ggcccttgga
7861 gcgcagattg cggggattgg aactaaaggc gtggatctcc ttaccgctgg cgaagtccag
7921 agtgaacttg cagatggact tgtccggatt cttcgggtcc ggaaacactt cctccccgat
7981 ggcgccagcc cccgcttcga agcgcttggc ccagtccgcg cagtcggaga tgaagccttg
8041 ggtcatctcc ttgtcgaagg agatgtaata gacattgccg ccgtcggcgg cggcggcgtg
8101 cagcaccgag tctgccgcct ccccgtagga caggccgatc cggcgcgact tttcaatgat
8161 cttgacgggg gaatagtcat ggatccagcg cagttggtaa tccagcaaca gcgacgaatc
8221 catcacatcc tcgccgcgat ggccgcgcgg atcgccgcga ttccctcggc cgaaacgccc
8281 gccttagtcg ccgccgccgc tgcagcttgt tcagcgccag cgcgttcctc tgcgcgaatt
8341 ctcgcctcga cctcggtctt ccatttcttc tggcttacgc ttgcccggct taaatccgca
8401 acccccgcg cggccttggc catgagctcc acccgggttg ccgggtcgtc ctccgcgtcc
8461 gcctcctgca ggttcagcag cagttcgaac agctccgact gcaccatcga tatcaccgcc
8521 gccgagcgta gatcggcatc gtccggcgcg gcctccgcga tcatccgggc cgcttgggtg
8581 ctggccttga cggcttccag ccggcgtttc aattttgcgc cgtggcgatg gatgacggag
8641 atgcccggtt cctcgtcggt cagaaaccag ccctgctcgc ggatccattc gacgagcacg
8701 gcgtagtctt tgaagttccg ggcgacgatc tgccgatcca gttcggcgcg agcctccggg
8761 ctcaattcgt ccaggcgggc gggcctaggc atggctcacc aatacttcac aggccgggca
8821 atgcctggct cgcagtcgat ggtgtactcg gcgatgtcga ccccgtggcg tgtcaattcg
8881 gcgcgccatg gcccggacgg ggaacgggta atccttacca ggtcgcgccc ttcgagatag
8941 cccagttcgc ggcgggtctc gatagccgtc gcatccgaat agacggtttg catcacgctg
9001 agcaacacgg attcatggca tgcgaccggg cggccgttgt tgagcgtcag caaaatgctc
9061 cagcgaatga attcgcggcg agctttttcc atgtcagggt tgctcatgca tttgtgtcct
9121 cagctgcagg ttttcgattt tcagcgccac tcggtcgatc ttcatttcga tcgtcgactg
9181 ccctcggacg tagtcctcgt gccgcacata tttttcaggc aggtcagctt tgagctgtag
9241 cagttcgcgt tcaacgtttc gccactgctc ggtatcccga ttggctgcct gttccaatgc
9301 cgcgaacttc tgatcccagt gccggcgcgt ctcttccctg gctttttcct gggccttgaa
9361 tcgttcatcc tggcgttttt catagagccc ggcgacgatc ttgccggcgg taaacgccgc
9421 gccgaaaatg ctacccagca gtccgacggc catcgtcagc agcgcccaga tatcgatcga
9481 gatcatcgcc gcgcctccgc tgccagatag cgctcgtgct caacctggca gtccatacag
9541 cgaatcgccg tcggataggc atcgagctga ccctgtggaa tcggctcctc gcagtcatag
9601 caaatgatcg tcggcgcacc gtcttcgccg gcgaccacga tctgcggcat gtccggcttc
9661 atcatgtcca gcgcagcgcg ccggttcaga tcgagctctt gctcgatgcg ctcttccgca
9721 atgtcagcgg cgtccatgag tgctcctatc gggtccgcgg tactgccaaa atgccccgaa
9781 ccgcgtgtgc atgtagggga agaaccgttg ccgttcgacc cattccagcc atacccaatg
9841 gccgatgtcc gtttgtacgg gataccaggc gaaccaggat cgccacggct gcggctgggg
9901 tggtgggcag gtgcgccatt tcattcgtcg ggagactggc tgtgccaatc gatcagcgcg
9961 ttgagctggg cggtgcaggc ccggtatcgg ccggcggcgg tggcgatcca gccggcaacg
10021 tcgcggtcag aggcggcagc ggatcggggc ggatcaacaa ttccgccggc ggtttcgggc
10081 aggccagcac cgtcaccggc ggcaacgagg gcgccgttga gcaggcgcac agcatcaggg
10141 ccgagacaac gctggccgga cgtaacatcg ggaacatggc ggatcacctc gacagtgcga
10201 accgtgacgc gggatttggt gcgggccagg tcgcccgcca ggcggttgcc gcgctcgtgg
10261 gcggcctcca gctcgttgcg cagcgccgct tcggcttgga aattccgcaa ggcgatttgc
10321 cgttccaact cggccagtgc cgcggtgccc tgagcgtcgg cgtagcgata gccgccggca

10381 gcgccagcca gccctccgag caaaaacgct gcgatcggca gcagcagact aatcggcatc
10441 gcaaaccccg atgccccagc ccgcagcctg gtatagcggc tcccaccggc gcagtatggc
10501 ccgtgggtag ccgcggttct cggtgaacgc ccagtccgct cggccggcat tgaagcgttc
10561 gacctcgcca aaccaactca gctcatcggc tccctttgcc gacgccaaac gtttgtcgcg
10621 gttgagccag ccgagtcctc cgttataggc ggacagcgcc atggccatgc gttcgcaggc
10681 attggcggcg tctattctgt cgaacaggtg caggtcgtag gtcaccaggg cacggatggc
10741 ccaggccggc gccatccgat cgcagcccat atcccctagg gaggggtagg cgccgcaaat
10801 ccatttcgcc gtatccgggg tgaactgagc caggcccgcg gcgtattttg actgcgccgt
10861 ggcgcgccag gcgctctcct ggtgaatctg cccggctaag gtcgccaccg gggcctccag
10921 gccccagatc agccgggcat tgcgggtcag atcacctcgg tagcgcgccg cggcgtgcgg
10981 tatcgcatcc gccgcctgta ccggctccgg aatgcagcct accagggcca gcaccagcag
11041 cagattgaac acgcaggctg tgcggaactc gcagcgccag cgcatcgtca ggcacccagg
11101 ccgatggcca gcatcgcaca gccgacgatg atcgctcggc gtagttgagc ggcggcaaaa
11161 agcagatcga acgcactcca cgtcagcggg atggacgaaa cgagtacgcc atcggttccg
11221 ggcggatcgg caggcttaac cttgtccgcc tcgggttccg gctcgtcgac gacgatgaac
11281 gtatcgggcc ggctgtacgg gaacagctcc cgatcgatcc agtaaccggc cacggcggca
11341 atggtcacca gagagacctt gtacagggac acttcgagtt gctgaggcgc caggtaggca
11401 atagctaaga gcagcagaat ggacacagcc agtgtccaga acatgcgaaa catggttgga
11461 gtactccggg ttgtcgactt caaccgcaga gtaaaggagc gccggcaatc ggctcaggcg
11521 gaaacgtttc cgccgcagga ttcgaacaga tcgatgttgg gggagaattc ctcggcctca
11581 ccgaggatgt tgcggatctg gcgctcgctg agatgatgcc gcagcgccag ggcggcctgg
11641 ctgtaaccct gtgcgcgctc ggtgcggatc agcacattgc ggcacagcgt catgtaccgg
11701 tcggccttcg cgatttctgg ccggtcgcca cccaaatact cggcgatggc gcgcgccgcg
11761 tcaatgccga ccacctccac cagcggatga tcggattcca gtttatcggc catcggcaca
11821 tacagccgcg taccgcccca gcgctccgca atcgccagcg ccgccgggaa accggcgatg
11881 cgggcgatgt cggagaggac gccggggagg tcgatgggtg gggtcatgcc gcctgcatgc
11941 ctccgtcctt acggcgccaa accagctccg gatttgcatc gcaaacgccg cagcagacgc
12001 cgggatattg ccgggttaac ctcaaactca tggcggtacg gacgccgcac gccgggcaat
12061 acagctcccc acccagtagg tatcgggcgc gccattgctt cgcccgctca atcgcccgct
12121 cgagctggtc gtagtccagc agctgcaggg ctgcggtcac gttcgggctg cggctgttcg
12181 aggcccatga cgccagcgcg gactcgttgg gatcacgcac cacgcccagc gcatgcaggt
12241 ccagccacag cgcccggagt ttgctgacca tggcagactg gtccacgggc cggcttgttg
12301 cccctctccc tttgggagag gggttggggt gagggcgcgt cttgtgctga accttgaacc
12361 ccttggactt catcgcctcc agggcttttt ccagctccgg cacggtcatg gccgttgtcg
12421 acattttccc ggtggccgac ttcagcagta gacggtaaac gtcgtcgccc agtttcaaat
12481 cccgcttgcc gacgtggatc aacttgatca ggcgttgttt ctcgtggtca tgattattca
12541 taagaatcct tggtttgcct tggaacacag cgccagactt aacaatacaa tgcagtggct
12601 aaccgagcgc ggccggacaa tgcctcccca cgctatgctt cgccccgccc cgttactggc
12661 tagctcatca ggcccaggtc gccagcctgg acgaccaccc tttcgggtgg tttcaccttg
12721 catcgcttta ccttgccgag ccggacaata cgcggcacag gatttcaata ccttgcaaaa
12781 gagctcaaag cgcggcttta tttcttctcc ttcgtctcct gccacttggt cacgacaaat
12841 cgtccgaacg gccccttacg ggccggccgg aaatcgccga gtccgatctt cttaccggca
12901 tcgtccacca ggagccggac gaactgaggc gagaaaatat cggtatccac cgacagggtg
12961 aaactcacgt gccactggtc cagccgcggt ctgtgacaca tcacccggcc accggtcgag
13021 ggaatgacta cagatcggga gtccacctcg aactccttgg tgcctagcgg gagggcgatt
13081 tcttcaactg ctaggccagc cggaaccaac gaggttttgg tggtcgtcac cttgcttttg
13141 cccaccttgt ggaaggttcc cgctgcgatc agcgccgcga aaaggtttgg gcctggaata
13201 tagagtccgc ccttgtcatc gcggtaaacc ttgggatcgg cctgctcccg cggcgtgccc
13261 ttggtgccaa tagatacggc agaggtgcca gaagagacct tgcgctcggc ctcctcggtg
13321 aatcggttca tgagcagcgg tgtggttccg gtgatcgtgg cttggatcag cattctgttt
13381 ctcctgttga tttatgcgcc ggcaatgtcc aggctgatcg cctggtactg atcggtgtcg
13441 ccgacgcgct cgtagacgcg gaaatagggc ttgctgccgg ccacttggac gctgtcagcg
13501 atggcctgca tcgcccgctg ccagcgctcg tcgcggatgt ccagacgctt gaggccgagc

13561 acccggccgg tgttgatctt gccctccttg ttcacctgga aggcatcgtt gatcaaggcc
13621 ttgatctcgt cccggctgcc ttcggtccag gcctgtatgc attcgtcgat caacgccttg
13681 gcggcttgca ggcgttcgtc gaacaccaag tgttcctgaa tctgtcgaat gaccttgtag
13741 cgtccgtcga acgacatgag ggtgacgttg cccttcttgc cgccgagctt gacgccgtac
13801 tgttcggcgc tcagttcgac gaaggcgccg atgtcggcga aggctttgga cttgaatcgc
13861 gccagggcct cggacatttc cttagcggca gcggtcaagg cggtcaccaa tgcttcacgc
13921 tccttgtcga tgggtttgat caggttttcc gggatcagcc ggccttcggc gtcgcgccaa
13981 aagccgccgt ggatttgctc tgcagtagcc atgtcgtacc tcctaaatac ccaggttgat
14041 gtcatcggtg gccgggtgcc tcgcctgtgc tttccgctcg gcccggccaa ccaggttgat
14101 gtccagcacc gcaccgagaa acagcccggt accgagtcca aacaggaaga tcatcgccat
14161 gccggcgaat acgatggttt tcattcggac agttcccctg ttgcttggtc gatggtgttg
14221 cgcagcagag cgatgatgtg gtccagttca aatgcgtctg atggatgagg gtcctccagg
14281 taggcgtaca cagtgcgtag agcgcccagc atctgcggta ctgccgcgat caacgtcgcg
14341 tttgcctggt tttcgcagat agccacgaca tcattccccg cccaaaccgc ctggatcagg
14401 cgccctttat taagacttgt ttcaattttg ccggtgcaga ctgaccacgg cactgacgtg
14461 tgtagattca tttcaatgct cctttcagat tgctcaaatg cccggccacg tccgccatgc
14521 cctggcgttt cgcggcggag ccgctcccac cgttgcgccg ccgctcttcc gtgagcactt
14581 ccgatcgggc ttccgcttta ctcgccttgc cgaccaggat ttccagcaga tagccgtgcg
14641 atttcaacgg ccgggtaaag ggcttggcgc ggtcgcggtc gagaatttcc tgcagcgccg
14701 ccggccagac ctccgccggt accgcccaga tccgcccgtc ccgggtaatc tgtccggact
14761 ggatcaacgg cagcagctcg ccgagcaggc tggccagtcg atccagcgtg agctggcgct
14821 gagctggacg gaacagggtg atgtactgga tcagcagctt ccccatcggc gccggcagct
14881 gcagcgcgat ctgcaccgcc tcccgcgcgc cgtcgtgggc caccaccacg tccaggctca
14941 tcaatgcgcc acagctgggg caggccaatt tcaagcgcgc ccccatgcca gcctgcgcgc
15001 ctggcgttgc ttggcttggg cagcggccac ggtattgacc ggccgctttg cctcgcgcat
15061 ttccttctcc gcggtttccg cgagctgctc ggcacggaac caggtgccta cgatgaggat
15121 ttccctaacc tggtcgccga ccttgatgac ggacttccag ccatactggc ccaggcgtgg
15181 atagtgatag atgcggatca tggtcatgcc cccattactt tccggatttg ctccccaccg
15241 accttgggaa taccgatgcg cgcgcagaaa ttcatggttt ttgtcatcag gttgttgacc
15301 gccagcggat agcaatagga gatttccgag aatccgccgc gaccatcctt tcgctttccg
15361 actaggacct ggcggatcgc atccggaaca tccggctcgc agagctgctc gaatggccgc
15421 agcgcccgtt cgcacttcag ccgcaaatag ccctccagct cgcccgttgc gtcgatgggc
15481 agcagttgtg cacgctcgat gcgctggatg gcctcgcggg catcccattg cagccggata
15541 tccagtttgg ccaacagctc gggctggccg atcagcacga tgccgagcag ccgttgcagc
15601 ccatctttgt tttcccagaa ccgctttaga tgtttgagca cgggaaccgt cagggcatgc
15661 gcctcttcaa tgagcagcag gttcaggaat ccggcgcgcc gattggataa cagcaattcc
15721 agtgagcggt gctggcgcgc ttcaggcgtt ctcggcaccg actgtctcgg ggccagcgtg
15781 cgcagaatcg cctcatggat ttgggtgctc ttgaatatcc gcccgcgagt ttcgacgtcg
15841 tcgatatcgg tgacgtaggg ggtgatcacc gtcacctgtt cctccctcat cgattcaatg
15901 aaaaaatctt tcgccgtgga cttgcccgcg ccggactcgc cgacgacggc cacgaaccgc
15961 tgtcctcgca gggcatcgcg catggccgct agcgcgaacg catgcgactc gcccagataa
16021 atgtcgtcca cctcgttcac atcgtcatgg aacggatcgc gggccagctt gaaatggcgt
16081 ttggccgctg gtgacagcca ctcgggttct gttaacatct cattctccgt tgtgggcgat
16141 tgcccattgc tatccggctg atgggtgttg cagcactcat cagccactct aaatatcgat
16201 tcgtcgacgc tcagcccgcg atcctgcaac gcatgcagga ttcctttttc cagcatcagg
16261 gcatttttcg gccactgatc gtggtttacg atctgggcga tcgtggcggg gctgacccca
16321 caatcccgcg ccagtgcggc ttgggcgatg ccggcctcaa tgagtgcgcg cttgagtttc
16381 agcatcgcgt cccctcatgt actgcacccg cttcacctcg ccttcgcaga cgatttcaat
16441 gcaggtcacc ccattgatac gccacacgct gatggcgcac agctccggca tgtgctgagc
16501 gagcgtaaac atcgcggcct tcatttcgtc tttgatctcg ccttcatcat ccatcgttgt
16561 gtctcctctc aaaccgcccg caacgcaggc cttgccggcg cctctgcggg ctgttccagc
16621 caggcccgca gcgttgccag ttcccgttcc ggcgcgcctt cagggcaccg gtctttgatg
16681 gcggcgtaat attccggccg ccagcctcgg cccatgaccg aggcgagccg cgcgcaaata

16741 tccgtcaccg tcaaaatctg ttccgccacc cgcaccgggt gagcgacttc caccacctcg
16801 ccacgccgct tcatgtactc gggcacgtac acctcccgca ggtgagagtg tgcattcagc
16861 ccgccgaagg gcgccgcgtt cctggctttg gccttttcga tctcctcgtc cgatcgtccc
16921 ggatacgcgg tgcggtcggc cgtttttgcc gcctcttcta tcggggtatc ccgtagcgcg
16981 cggaactcct gaccgaacac cggcgcatcg gcgcggaatc cgctcgtctg ctcaaatgcc
17041 accggatcga tcacatgggt ttgctcatcg cccttgtaat ccgcgatcgt caccagtacc
17101 tggttggcgc cataaaccag cgggctgacc gtgacggtca tgtcccggaa cacgccggca
17161 atgccggaca ggtcgtacac tagggactgt ttcgcagacg ggtgccggaa actgacggtt
17221 agccccccgg cgccggtcgc ccgcaccttg cgcggctccg gctcggccgt cagcagatag
17281 cggcacagct cgtcatccgg caacgtgcgc agctgctcct ggcggatggt ttgccagagc
17341 gcatagcggg cctgcggttg agccaggccg atacgcttca ggcgcgagtc gtagtgcggt
17401 atcgcattgg cgttgaacgc ccggtaccac gcctccgctg cggtattgag ttcgtccacg
17461 ttccgcaccg gctcgaattt gagccggctt tcgaacagct tttcgacgat gttgttggca
17521 cattccacct ggcccttggc ccgcgcctgg ccgcgggcat gcgcccaggt ctccacttgc
17581 agggcgcgca gcgcgttgtg gatggcgccg ccggcatcgg atcctttgtc ccataccagc
17641 agctcgggca cgccatggaa ggggcagccc agcgcatcga gctggcgcca ggcgtagagc
17701 aggaaatcgt agagattggt ctgcgtttca cctttggcct ggtagtagcg aacgatgacg
17761 acgctggaca tgtgatcggt cagcacatag cgccagcact tcaggttgcc gatccgctcc
17821 acccaggcgg gtttgttgcg gtggatttcc tcgtcgcgca gtacccgctg ctcgccattg
17881 ggcgcgtaat acagcaggca gagcgagggg tcggtctgat gcacgtggtt ggggtacagg
17941 ctgctcatgg gcacatgcgg tgtgtccagc gcggccgacc gggcatccag gtgcgcccgc
18001 cgcaacagcg tccgcacccg gctcgcgccg accggtatcg cccggccatt gctggccatc
18061 agcgaaatgg cgttcggcac ctccatcgtc accttgccgt tcttgcggat gccgggccgg
18121 atcgccgcgc ccaactcacg caacacctgt tcgtccagtg ccgtggagcc cgcgtccgag
18181 cgggtcttgc gtccgctgct ccagcccact ttgcgcagcc cccggtacac ccggttggca
18241 tcggcccagc cgtgctcggc cagaaagccc tggatcaact cgccccgctg ccggtgcggc
18301 gccgcgtcca gccgacgcgc caacgcgcgg aactggtcca acagggtgac ggaatacggt
18361 tcgacgatgg ccatgggtta ccccagtgtt ttgtatccgg agaggtttgc ctcgcaggca
18421 tcggcaaacc cttgagccgc ctgcatcacc tggtcggcca ggtcgtacag gtgcacggcc
18481 atcatgcgca gcgccgcatc gtggtcggcg ggctcgcccg gcagctcgac ggtgaggatg
18541 cgttccaact gccgggtgag ccgatcgaat ccctgcagcg tctctccggc cgcgaccgtc
18601 acgtcgaggt tgatgccgtt gaccaccgcc ggccagctcg ggaccgcgct ttcgcgctct
18661 atcaacttct gttgcaggtc gtcgatctgc aggtttttgg actccagcag ccgttgttta
18721 ccggcatctg cggtcttgag cttttcgatc gccttggcgt ggtcttcgtc tttcttgcgc
18781 agcgctaggc gcagctcgcg gacggacatg caggcaattt catcgagctc cagtgacctg
18841 ctccccgaga ttagtccggt cgtgatgtag agtctgcctc gataggaggc agcatgaaga
18901 agcgtttcac cgaagaacag atcattggca tcctgaagga agccgaagcc ggcctaaaag
18961 tagcggagct gtgccgcaag cacgggctca gcgaggcgac gtactacaac tggaaagcga
19021 aatacggcgg cctgacagtg tcggatgcgc agcggctcaa ggcactggag accgagaatg
19081 cccggctcaa gcgcctgctg gcggaggcga tgctggacaa tgctgcgttg aaagaggttg
19141 taggccgaaa gtggtaagcc cacaagccaa gagggtggcg gtctcccatc tgatgacaaa
19201 gcaccagatg ggcgtcacgc gggcttgtgg gctgatcggt atttctcggt cgctgtatcg
19261 ctacgaagct aagcggccag tagaccagga gctcaaggaa cgactgtgcg aattggcagc
19321 gcagaagcgg cgctatgggt atcgccggct gcacgtgcta ctttgccgag agggttggga
19381 aatcaaccga aagcgcacct atcgcgtgta tcacgaggcc ggcctgatgg tccgcaaacg
19441 aaagcggaag cgcattgccg gcgtggagcg ccaaatcaag gtcgcgccat cggcgcctaa
19501 cgagagttgg tctatggact atgtttcgga cggtttggcc gatggtcggc ggctgcggtg
19561 cctgaatatc gtcgatgact tcacgaagca gtgcttggcc atcgaagtcg atacttcgct
19621 gcctggcaga cgtgtagtcg gtgtgctgca acggctggca gagatccgcg gattgcccaa
19681 atcagtcacc gtcgacaacg gccccgagtt tgccggcaag gctttggatg aatgggccga
19741 tagccaagga ctgtgcttga gcttcatcca gccaggtaag ccacagcaga acgcctacat
19801 cgaaagcttc aacggcaaat tccgggatga atgcctgaac gagcattggt tcgtctcgat
19861 gtgccatgct cgccaagtca ttgaggagtg gcgtcgggaa tacaatgagc aacgccccca

19921 cagttcgttg gcttacctga cgccagatca gtttgcagac acatttttaa ccgcagactc
19981 tatgtccgtt tcggactaaa tcggggagca ggtcaccaga cctcgcacct cctggtcgtt
20041 ggcgagctcg tcgatctcgt cctcgtccag caccgcgagt tcgatcagct tggttttgcc
20101 gagagccatc aaatttgcgt ttgaacgcaa atttgagaat ttgcgagcgg cccgaatcag
20161 cttcgacgcg acgaactggt cgatcccaat tttttgaact gcatccatga actcgccgcg
20221 gggttcgcgg tctttgatct gaatcaatct ccgcccagct tccagcatgg cttcggcgga
20281 ttgattcagc agaaatcgta tttcctgggt cacccgatcc agctgatacg ggagaatgtc
20341 gccgtattgc ctgtctgttt cggcggcatc caacacagcc cgattgtggt ttgctatgag
20401 ctgttcttcg gggagtgccg gcagattggg catttcggtt tcaggcagct tctcgcgtcc
20461 catctcaacc ccctatgcca ttcagaattt caactacaaa tctcatctct attgctccaa
20521 tgcggtggac gaaaccaaat tgcgtttgaa cgcaaatgtg cgtttgaacg cacatctgaa
20581 aatccgacat caatagctag cgccagcagc gatgcgctga ttcagctcca aaatccgacc
20641 ctgcaacttg gtcacgtgat cggcatgggc ctgggcgatc tgcagcatgc ccaccgaatg
20701 ggcaaatcga ccgttgtcca gtttcaccgc cagcccttcg gcaatcagcg tgttcagtgc
20761 ccggttgacg ttgcagggcg agtcgcccag cgccttggcc agctctgtgt tagagagacc
20821 ggtcagcgtg tgccccttca gcatcttgag cacccgcaga atgcgggcgc cggactcgat
20881 ggttttcttg cgccgcgagt tcttgatctt ttcgttcata cctgccgctc cgcgtagtcg
20941 cggtattcaa tggctgccct gatacgcgcg ctgtccagcc gctcgtattc acgctgcgct
21001 gcagccagcc ttttccatgc ctgctgggcc gcggccttgg cggcctgctt gcgcgccttg
21061 aggcgcatca atttattcat gccgttagtg ctcatacccg ccgctccgcg tagtcgaggt
21121 attcctggac cgctttctga tgcgccttgc gcttgcaggc atactcccgc tgagcctcgc
21181 tcagcttccg ccgtgcctcg tccagatcat ccagggccgc gtacttgcgc atgttcaggc
21241 gtatcaattc cttctcctcg ttaatgctca tcgtcgcctc cgaaatcgaa ttcgggttgt
21301 tggtgcttct ctacgtttac ccggtgccag gccagggcct ccatgccggt ctgaatcgcg
21361 ccgagcacgt cattcgcttc ggctttgcct gcggcaaatt tgatgatttg gcccaccgcc
21421 tcggtcagca gctcttgcag cgagttgatg tctcgcgcct gcgatttcct gcctgtaggc
21481 acccggatcg tcatgtagcc ggcggattgc gccagccatc ggctgatgaa atggacgccg
21541 caaacctcct cgtaattcag gatcagccgg accggtaaat ttccgtcgga ctgccagtcg
21601 taaaccttgt cggccttgac acccatcttg ttctcgatgc cagggatcga cagattgcgt
21661 ttgtcgcggg cgtactggtt gcacagcttg atcgcgtgca gagcgttcag cggctgtact
21721 cgtgaccaat ttcgattgcg cactgtcaag acccgaaaaa tatttatttt tcccaacacc
21781 gcatcaggcg gtgcgccgaa acaaaaaaac ttttaccctc tatgcaatct cgttgcgtct
21841 gccgagaatg tccgcacttc aacgcagaca ggggctcgat catgaacacc gccgaattca
21901 cagaactggc cggccgtatt gaaggcctgg gtgctgcttt cctttgcctg gttgcggagc
21961 tggaaaatcg cagagccatg gacggttccc gcttcacttc gggcctacat cggatcgcgg
22021 gggaattgtg tttcgagcag ccacacctgg aggagacgaa acggacggtg cgcgaactgg
22081 cggccgagct ggatgcgatg cgcgagcgtc gtattcacgg ccgataattg cggcgtcggg
22141 gatgaaaccc caggggttga tggtcatgat tgctccaagt gtcgttaagc aaggaaatca
22201 gcgtttaatc ggcttgaggg ccctcagtgc taggcgcctg actgtttgtt tctggcgttg
22261 gggcataaat gtgtttgcca ggatttacgg acgcgtcagc atcgtcctcc tcgggcgccg
22321 attcgttgtc ggattcagcc atgcgttgat aagggcgtac cggccagagc gtgcagtctg
22381 agatgcagca ttggccaatg gctttgcgag gattgggatc aacaccgcag ccgacgcagt
22441 cccagcattt gccgttgatg gccaagcgca aggaagccgg gttacggcgc gccttttcga
22501 tggggctaag gcgttcgatt tcaccggcgg cacgcttttc ggccatagcc gcctgggctt
22561 tttcgagggg agacaggttg gtcatgacgg gtcctgctga atgaggcgga tcaggcggcg
22621 tgctggctaa cttcgccgtc cttcagaccc atgatgacgg catgatcgtg ggccttgcct
22681 cggcgtccgc ggatgcggcc gttgagcaga tcgcgcacga gacgcggatc caatgatttc
22741 gccttggcaa aggactcgac ggtaacgcct ttggagtcca gctccttgcg tagctgttcg
22801 ggcgtgtaga gagccatagg gttgctcctg tggtttggtt tgcgagaacg tccgcgctca
22861 tcgcttttga tgtgcgcgtt ttgtgcgata gatgatgagt ccgtacggac tctttgtcaa
22921 taggctatgc gctcatatgg acactattgg ttttcgatta cgcaaagaac gcgaagctct
22981 tgggttcagc caagaggagt ttgccgagaa aggaggtgtc caacggcgcg cccaagttcg
23041 ttatgaatcg gacgaaaggt gtccggatgg gcactatttc tcagcaatag cggcggtcgg

23101 tgccgacgtg aactacatcc ttaccggaga gaagaaggag attgctgaaa agctcaaggg
23161 cgtcgggcag gccagagatg tcgtagtgga ggcggtcctg aaacgttccg gattagacag
23221 ggagatgctg gccgatttgg atcggaaggt gtttgactcg aacttgtcgg cggccgacat
23281 cgagaaagtg ctggaagaac gttttccgac ttccgcagtc gtcatggttc cgcgctatga
23341 cgtccatgga agcgctggcg gtggagcggt caaccacacc gagcacgtca tcgaatacgt
23401 ggcgtttgac ccagcatggg tacgtgaggt cctacgcgtg tcgcccgata agctgtgcct
23461 gatcggcgtg cgtggcacga gcatggagcc gaccctgctc aatggcgaag tgatcctcgt
23521 ggatcgtact ctgtcggaaa tccgggacag cgatgtctat gtcatcgagt acgaggggaa
23581 tttactcatc aaacggattc agctccgcct ggatggcact gtagtcatca aatcggataa
23641 cacggcctac gaaccagaga ttctcacctc ggaagatgcc cagcggctca gcgtggttgg
23701 gcgagtagtc ccatggaaat ttggacgatt caagctgtag acaaagaaat gaaccatctg
23761 gaagaggcat tctggaaaat cgccgatcag ctgatcgtgg atcgagccag attggagggc
23821 ttgtggccac agtttgaaaa acatcgcaac gatcctaagc gtacctattc gatcatcgag
23881 cagtgtttgc ccgaggggac gtgggaatgg cccgagttta ttgcccacca agcatcgaaa
23941 ggcgtcacgc cgacattcaa agagatgtgc aagatgctct acagccggat cgagcgcgtc
24001 tccggtgcgc tacgaactgc agatcaaatc agccacgtca tcgtacgaac gccctatttg
24061 ctgtttcgcg ccggacttga gccccggcca gagtgtcggg ctgccgatgg acgagtagag
24121 cattacacga atgaattctg ggaacatcac acaccgccgt gtgattggct cgaatgcagc
24181 tgccgaatat tcgtagaccg gaaaatgcga taagcacgtc attgaggcgg acgccagaat
24241 gacgcaagaa attgaaatga cgaagtgaat ttgaccgctg gaaggagaag caagtgagcg
24301 aatttcgtag ccggatgttg cagcatgtgg agcacgtgaa gaatgtaggg cctcactgcg
24361 acaccgagga gaccaccaaa caggcgttaa ttttgccggt gttggacgtt cttggcttta
24421 gtccctatga ccccacaaaa gtaaaagcgg aatttgccgc cgattttcct ggcgtgaagg
24481 caaatgagcg ggtggactac gccttattct gtaatggcgt acccgtgatg ttcatcgagg
24541 ccaagagcta tacggaaaac ctgagcaacc atgcgccgca gttgtcccgt tatttcaatg
24601 cgacgccgga agtgaccatt gccgcgatca ccaacggtcg ggaatggcgt tacttcacgg
24661 atctggtcga caagaacgtg atggacaaag agccgttcct gacggtggac ttctcgagct
24721 tgcgcgatgc cgacatggag cagcttggcc gcttccgtca cgatcggttt cagcccgaag
24781 cactacggac tcttgcggag gaaagtgtat tcctcgccgc attcaaggcc gtcgttaagc
24841 ggagcgtact ggaaggggat gccgatttcg tgcgctatgt cgctggcaag tcgacaattc
24901 aacgcacact gacagcgaaa ttcctcgaac aaatcacgcc gatcgtgaaa caggccgtcg
24961 cgcagaccat gagcgatatg gtggtcaaca gtctttcagc gccaagcttg ccggagccgt
25021 ccgcaacgcc gcagtccacc gatagctttg acgacgacat cattgacccg agcaatagca
25081 agattgtcac tacagcggca gagcgggctc tgctcggcat cgtgcttgac cttctggcgg
25141 gagagccgtt gtccgggaaa gacacggaaa gttatttctc cgtgttgtat tcgaacaaag
25201 ttaatcgttg gctcctacga tacacaggaa acaagcgtcg cccggtcgtg caatttattg
25261 tgccgctgac tccccagcat cgcagcgaga tcgagcgggc cgggttggaa attgggtctg
25321 ggaatcaaat cctgctcgat aagcctgaat acctgatgcg catctccgga ctgctgtttg
25381 acgccctggc gtattgcaag gatgacgata acttcaagct taagaaggaa aagtagggag
25441 tacattgaat gagcaaagac aacctggagc aatgtggcct gtcgcggctg gatcgcgatc
25501 atgatggcgt gccttgcgag tcgatttgcc gttaagaaaa aataaggaga gacgatgaaa
25561 agcttcctgg tagtgggttt cctttgcgtg tttctaggcg cagcacatgc cgccggacca
25621 acgtttccaa cgacccaccc ctttgacgcg atctaccaga gattacattt tggcgataac
25681 gagtcagacc cgtttgacgc ctatttctgg tgggagaacg ggaatctgtg gatggaagca
25741 gaccccttgc cgagtgctca cgtctgtagc cagaatttca ctttcgtgca agatggatca
25801 cagtacaacg tcgcgttacg gaacaagagc gacgacacgt tttgcggcac gttgtatatc
25861 aatgctgtct tccggatggt cgaccggcgc aatggcgcgc cgtttaatcc agtgctcggc
25921 gggaaaatct ttgtcgatga gctggacaag actcaatcga ttgatatgcc ggctgggcca
25981 gcgccatcgc cggaccccac tcccgaacct tccccaactc catctccgac tcaggctccg
26041 accccgaccc ctgaaccgac tgtggctcca actcctgcgc ccacagtcaa cccaactcca
26101 gcgcctactc tcggaccatg tccgacgccg gcgcctacgg gcacaccaga tcctacaccc
26161 gagccgaccc cggcccccat atacgacaag ccgcaagcgg acgtatatga catcgacatc
26221 aagaaagtgg tttgccagcg caagaatgga aagaaattcc ctgctcggca gctgcccgga

26281 cctggcaact tcgtttgtga cgggataccg atcaatgccg gagagaaagt aacgatcacc
26341 gtcgtgggca cgaagaagta atccgacgag gggcgcgtgt ggcttcattt gattgctatg
26401 cgcgccctat gcctaccgga tagcgttgct cggcctcgaa cggctttcga ggatttggaa
26461 cgcggtgttt gggatggccc ttgccgcgcc gtatactcta gccactctta acccggttaa
26521 cagctccttt tctcccttct gccaagcagg cggaaacgtt tccgcctgat gcctctcggc
26581 tccggtccgt aacctacgga ccatgagcca gaccacacct caacccatcg aatccctcaa
26641 gcccggccgg tttacggcca tgagcggcca ggtcgttgag ttttccgaat ccgatcttgc
26701 tgcgatggcg gcggcctacg atccagcggt ttaccgcgcg ccgatcgtcg ccgggcaccc
26761 gaaacacgac gatccggcct acggctgggt cgatgccctg cgctattcgc cggacaccca
26821 gcgcctgctg tccgtgcccg gcgatgtcga tccggcgttc gccgaaatgg tgcgcagtaa
26881 gaaattccgc tctgtcagcc tgtccgtcta cctccctgac tctcccgcca atccgaaacc
26941 gggcgtgttc tatccgcgcc acctcggatt tctcggcgcc atgccgccgg cggtgaaggg
27001 cctgcgcgaa gcctccctgt ccgattccga agaaggcgtc atcgagctga gcgaatggga
27061 cgacgtgacc aacgccggcc tgtggcgccg tttgcgcgaa tggctcatcg gccaatttgg
27121 ccaggacacc gccgatcggg tgatccccgg ttacgacgtg caatccctgg agcaagccgc
27181 tcaacaggaa gtggccgaag ccgccgccga ggatgccggc gcccagcccg ccttttcaga
27241 atctcaaccc gaggtaacgc ccacgatgac tgctcaaacc accgatctgg ccgaacgcga
27301 ggcggaaatt gcccgccgcg aggccgaact gcaacgccgc gaggcggcgg cgacgcggca
27361 ggcgcatgcc gatttcgccg aaagcctggt gcaggccggc aagttgccgc cggcggacaa
27421 ggcgggcctg gtcgaattca tggccagcct ggacggcgaa gcgacgctgg aattcagcga
27481 aggcgaggaa acggtcacga cgcaatccag cgcctggctc aagtccttcc tgagccgtcg
27541 cctgccgaaa accatcgaat tcagcgagct cagcggcggt gccggcacgg gtgacgagat
27601 cagcctgtcg gaagacgagc ggaagacctg tgaggcgctg aacctcacgg cggaagagta
27661 cctggcggca aagaaggggg cgtgacatga ccgcattggc agcagagcgc aacaccaaag
27721 aacggatcgg cgacgtcttc gacctgccgg tcaaggccaa caccaaatgc tatctcggcg
27781 gcctggccgt catcgatgcc ggctacgcgg cgcccggacg taccgcaacc accctggtcg
27841 ccgccggccg gttcgaagag agcgtcgaca acaccggcgg tgcggccggc gacaagaagg
27901 cgcgggtcaa gcgcggcatc tacaagttcg ccaacagcgc cgcgggcgat ctgatcgcgc
27961 aggccgacgt cggcgccgac tgctacatcg tcgacgacca gaccgtcgcc aagaccaacg
28021 gcaccaacac ccgctcccgc gcgggccaaa tcgtagccgt cgacagcgac ggcgtctggg
28081 tccagatcgg tctgggctgg taatcggaga cacatatgca gatcacgccg caaaccctga
28141 aagccctcca gaacggcttc aatgccgcct tcctcaaggg cttcaactcc gccgaggccc
28201 agtggcccct gatcgcgatg gaggttccct cccagtcggc cctggaaaac tacggctgga
28261 tgaaagaact gcccggcatg cgggaatgga tcggccagcg cgtcatccac aacctggaga
28321 tgatcggtta ccagatcatc aacaaacatt gggagcacac cgtcggcgtc gaccgcgacg
28381 acatcgatga cgacaaactg ggcctgtatt ccacccgctt ctcgatgcag ggcgaggtcg
28441 ccaaacgcca cccggacgaa ctgatgtggc aggtgctgct gagcggattc aattcgctcg
28501 cgatggacgg ccagtatttc ttcgacaccg accatctggg ctacgacgcc gccggcacgg
28561 aaacctcctg gagcaactac caggccggtg ccagttcgcc ctggttcctg atggacctct
28621 cgcgcagctt catgaagccg ctggtgttcc agaaccgttc gaccgtgaag ttcgtcaatc
28681 agtccaacga gaccgacgac aacgtgttca tgaaccggca gttccggttc ggtgtggacg
28741 gccgctacaa cggcggcttc ggtttctatc agcttgccta tgcctccaaa gccgcgctcg
28801 atgccaccaa ctaccaggca ggccggctgg cgctgggcac tcagcgccgg ccggacggga
28861 cggcgctgcc tgtgcatgtc actcatctag tcgttggccc gaccaatgaa gccgccgcgc
28921 tcgcgctgct caacaaggag ctggtctccg gcggtgaaac caacatctgg tacaagaccg
28981 cgcagctcgt ggtcgtgccg cagctggggt aagcaaacca tgatccgaat cactgcacga
29041 cgcgccggat tccgccgctg tggcatcgcc cacccggcgg aacccatcga ttaccccttc
29101 gatcgtttcg acgaaggcga gctgaagaag ctccaggccg aaaacatgct catcgtcgaa
29161 gtgctggacg atgaggcatc ggaatccgcc gcagacgatc cggcgccgct caagccgcat
29221 cgcggcaaag ccgctcccac agggtccgcg gcatgagcta ttgcacccaa gccgacctgg
29281 aaaaggtctc ggccgtcgag ttcctgcagc tggccgatcg cgaccacgac agtgtggcgg
29341 atgcggacgt cgtagccgag gccatcgcga aggcggacgg gatcatcgac agctacgtgc
29401 gccaccgcta cgccgtgccg ctggcgcccg atccggctat ccgggactgc gcggtgaagc

29461 tggtctggtt caccctgcag gaaaccaatc cgaccgaagc cgcccggaag aaccgcgacg
29521 acgcggtgaa gttcctgcgt gacctatcag aaggcacgag ccacctgggg ccggatcagc
29581 tcgcaccggg cgcgaccgtc agcgtcgacg ttccgcaggc cgaagcgccg gaccgggtgt
29641 tcagcgacga cacgctggcc ggctactaga gcgcgtcgat gcaaatctca cccgtcgtcg
29701 cccagctcca gaccgtgccg gaacttaccg gccgggtcga gatcgccggc agcgtccgtc
29761 agatcctgac cgagctgaac agcgggaaag gcccgcgatg ggcctgggtg atttcgccgg
29821 gtgacgacgc cgggaataac acactgtcgg ccggtggcat ccggcaacgg gtgaccgacc
29881 gctacggcgt cgtcctcggc tgctacgacg tcagcgaccg ccgcggggaa gccgcactgc
29941 tcaaggtcga agcgctcggc caggccgtgg aaccgaagct cttgggcttc atcccggcgc
30001 cgggttacgc ggaactcacc taccgatccg gccaaatggt cggcggcacc gacggctggg
30061 tgctgtggct gcacctctgg caatcgaacc actacatcac caagtgagga cgtgatgcac
30121 acgagtgaac atttcggcaa gggcggcagt tacaccctga acccggaaac cgatcagatc
30181 gaactggccg aggaacgcat ccagatggcc ccggaattcg tgccggttgt tgccttctcg
30241 gacggttcgg cctcggagga acaggctgcg gctgccgaag cggcgactgt gtccgacgcg
30301 ccggcgccgg tgaccgctga tccagttgcc gcggatccgg cgtccctcga agccgcgcag
30361 gacccgtcag cgaccgtgaa cgacacctca acggaccggc ctggccggcg cggcaaacga
30421 gccgagaccg atccgactga cggaggcgca gcatgagtgg ccaaatcacc cgcaacaacc
30481 gagtcctggt cggcaaactc gaatccacct atggcgtcga cgccaacccc accggcgcgg
30541 tcaatgcgat ccgggtcacc gacctgagca tcaagccggt cctgggcaac cagaagagga
30601 tcaattacat ccggggtttc ctcggtatcg gtcgcaatgt ccgcaccgaa ctgtaccgct
30661 cgatcacctt caaggtagac atggtctcgg cgctgacgcc gggccaggca ccgccctatg
30721 ggttcatcct gcgggcctgc ggcatggccg agaccttact ggcggctgcg gtgaccggca
30781 ccttacaagc aggctccacc gccaccacgc tcaagctgcc ggcagcggcc tccgccatca
30841 acggtgccta catcggcgcc aagagccgga tcaccggcgg actcggcagc ggtcaggccg
30901 cctctggcat cgcctacgac ggcgtctcga agatcctgac gctcgaccgt cccttggcca
30961 ccgtgccgga cggcacctcg gcggtatcga tcgatccctt cgcccgctac aacccgatca
31021 gcgacctcgg aaccatggaa gcggcgacgt tctactacta cacgggcaac aaagtccggc
31081 acaagctgct cggctgccgt ggcaacgcca agtgggatgc ctcgtcgaag gacaccggct
31141 tcattgagtt cgagatgttc gggctctacg gcggcgtggc ggacgcctcg gaagccagcg
31201 tggtgacgac caactgggtc gatccctggg aggtcggttt cggccgcacc tacggccaat
31261 tcttcgggaa gcagttcacc ggcggcgcta ccggcctgca gatgggcaag ttctcgctcg
31321 atctgggcca caagcccaaa taccgcagcg tggtgggttt ccagggcatc aacagtacgg
31381 atcgggagtc caccggctcg atcacgctcg acgccaccct ggtggccgag caggacatct
31441 gggccatcgt caccggcagc acgaccggcc cgatcggcat tgagcagccc gacaccaaca
31501 atggctcagt ccgtctcgac gtgccgaagg ctgacccgac cgacgccgat gacggcgacg
31561 acgagggcat ctcgattaac aacatcccgt tcgattgtat gccggtcctc ggcaacgacg
31621 aactctacta cacctgccgc taaggagaca tgaccccatg gcattcaagc tcggtaccca
31681 ggaagcactc gacaagccca tcgcccgcaa ggtgctgctc ccggtctgta acgaggccgg
31741 caccccggtc aatcaccaga tcacgctgct ctatcaccgt atgacggttg aagagatcgg
31801 cgccgcctac gctgccctgg gggaagacgc caagctgctc gaagagctca gcgggccggc
31861 gctgaccgcc gcggtgcttg atatgcacgc caaccacgtc ctgcgcctgg cggtcgggtg
31921 gaaggaggtt caggcggacg acggtaccga cgcggcattc accttcgaca atgtccgcgc
31981 tttgctgaac gccgtgccga aagcctacga caagattacg gaggagttcc agaaagccaa
32041 ccggggcggc gccgcactgg gaaactgatc gaggccgccg agcactgggc gcgaggcggc
32101 gaccagcccg atcaagcctt ccaggacgac atggaaggct tcggtttgac gcaggcacaa
32161 gaggaagcgg cgccggcgga taccgacttc tatgtcatgg aggacaactg ggacacggtg
32221 atggcgttcc tcgcctgccg aacccggtgg cagcgggaat ttccaccccct cggcggcccc
32281 gcgatctggc tcggtttgaa ctattcagac gtggaagtgg tgattcgcaa cctcgggcac
32341 gaaggcgagg cggcccgcca gatcttcgcc ggcattcagg tgatggaagc ggcggcactg
32401 gccgtgttcc atccgccggc ggttgtctca acggccggag aacaggcgga cgaatgacgt
32461 cagagcgaaa accacaacga tagcggcccc taccatcgcc acgccggcgc cgtatttcag
32521 ccaccccagc gcaaatagca gaaagcccag caccatcaga ctgattacgg acatatggcc
32581 ggccctatcg tagttggcat caaactcaat ctcgacgaga aaggagcgct ggtcggggtt

32641 gagaaggtcg aaagcggatt caagcgcatc ggctcggccg ccagcgaatc cgccagccgt
32701 atcgacgcat catacaagaa agtcgaaacc ggcatcgccg aagtcgagaa cggcttcaag
32761 cgcgttggtt cggctgccaa cgaatccgcc ggccggatcg atgccgctta cggtaagacc
32821 cgccagggcc tggattccat cagcacgcag ctggcgacgg ccaagcgcca gtttctcgaa
32881 ttcatcaccg cccaggcggc gctcggcgct ggccgggaac tggccgatgt ggccgaccgc
32941 tacgccaacc tatccgcccg cctcaagctc gccacccaaa gcgaactgga atatgccaag
33001 gcgcaggcgc aggtgttcgc catttcccag cgctacagca ctgccctcga cgacaacgcc
33061 aagctgtact cccgcattgc ccctgcggcg cgcgatgccg gcaagagcca ggccgacctg
33121 atcaaggtca tcgaaggcgt caacgccagc ctgaagactt ccggcgccag cgcagcggag
33181 caatcctcca ccatcaccca gctgtcccag tccctggcct cctccacggt gcagtgggag
33241 gacttcggcc agctcgccga caccaacatg cggctggtgg atgccgtcgc caagcaattg
33301 ggcggcagca tgggggacct caagcaaaag atgtcggacg ggctgatcag caacacgcaa
33361 ctgttcgacg ccatcatcgc ggcctccgaa cagctcaaag ccgaagccgc cacgatgccc
33421 aacaccatcg gcggcgcggt gcagcagatg tcaaatgcct gggagcaata catcggcaaa
33481 gccgaccagg cggccggcgc cagccggcaa gtggcggacg ccatcagtgg tatcgccggg
33541 gattttgatg atatcgccga tgcggctatc catgtcggcg aagtcatcac ggcggtgctg
33601 gcggtcaaag ccgtccaggc gattggcagt tatgtgatcg cggcagaagg cgccacgttg
33661 gcaacccgca cgctgggcgt tgcggtgtcc gcgttgggcg gcccgtgggg tatcgcggcg
33721 gcggcaatca ccacgggagg tatcgctctc cacgagttca tggcgcagca acaggcggcg
33781 atgaaagcgc aatcgctgct ggaagcgcag atgaacgtcc gcgacctggt catggctggt
33841 ccggattcat cgggtctgtt cggtgcgacc gcgatggacg tggccaaatg gcagaaggaa
33901 ttagccgccg cccgcgccga ggcggatgcc ctggcgaaaa gcgcatccag cgccggacag
33961 gcggccgtgc aatccggaca agccatggcg ggcgccgccg atgctgtcaa gaaatacacg
34021 gccaccgagc gcgaagacat ccagtccaag gtccgctggc tcgaagctca cggccagcag
34081 acggatgcct tgcgcctcga aggtgtcctt cacggattca gcggcaaggc gctcgaggcc
34141 tacgtcacgc aggagctgga cacgatcgcg gccaagaagg ccaacgccga agccaccaag
34201 gtccacaaca aggccctgtc cgaggaagcc agcacggtca aaaaggcggc gaccgagcat
34261 cagaacgcga tcgaagccag cgacaagttc atccagtcgc tgaaagacca gctccagaac
34321 atccaggaca ccaccggcgc cagcgaggcc gagcaagccg cgcgcaaggc ttccatcggg
34381 ctgatcggca aagaggcgga cgaacggtcc aagctggcca agcagctggt cgaagaacgc
34441 caggcctacg aaggcgccga gcggatcaag cagctcgaac tcaaaaccca gaacgacatc
34501 gccgaggcga tccggcggaa agaagagacg aacgcccagg ccctgcaaag cgcccaggcc
34561 gaagagcaga agcagctcgc tatcctggct caacttcagg ctgcgcacga ggccggctac
34621 ggacccgaag ccctggccag gatgaaagcc tacctgagcg cggtctatga cgtgacccag
34681 ggcatgggcg aggaggaggc cgcggcgact gcagccgcaa tggcccacac cgagtcggcc
34741 aaccagatga tgagcgagat gacccagacc ggccagtcca tgctgtccga tttgggtggc
34801 atcgcccaga acatgatggg cagcttcacc tcgatgttct tcgacccgtt cggctccgcc
34861 acggcgaaca tggggcagca gttcagccgg atgttgcagc agatggcgtc gcaattgctg
34921 caaagcgtgg tgatgcggtt cttcacgggc ggctcgtcgg ggactggctc tatcggtggg
34981 ctgataggct ccgtcggcag ctggttcggt ggttcggaag ccgctgccgc gccttcggtg
35041 ggtgggatcg tcaataccgg ggcggccgtt gcgaactggg ccaacgtcgg cgatatgttc
35101 gtctcgaccg gtggcggcaa ggccatgccg gtggccgtcg tggatgccgc cggcgccggg
35161 acacgggcgg caggtacggc aagcagttgg ctgggcggcc ccggctgggc cagcaccgcg
35221 ctcggctatg ccggtgcggg tatcggagga ttagggacgg gctatctgat cggcaatgcg
35281 atcggcggtc aatacaccta cggccgtgaa ggcggcatgg tcggcggggc cataggcgga
35341 atggctggct atgctggcgc cacgattggc atggagatgg cggcgggctc catcgaagcg
35401 gcgcttgcct cgactggtat tggcgcaatc gtggccgcga tcatggccgc ggtgatgtcc
35461 tcaatcaaaa agggacagcc cgacgccacg gcccagtatt cctggcagaa cgatagctgg
35521 accctgaata agcaagcgga gcgcaatggc ggcaatcttg cctttctggg gaatttcgcc
35581 accaaactgt tccagcctct gacggatctc gaaaagacgc tggccgtccg cctgcccgcc
35641 ttcaatctca acatgcggca gcatgaaggc tatttcggca tgcagatacc ggggctctat
35701 tcggcccgga tgaaagctga ttcagaggcc aataaataca tggcccaggc ggtcagcatc
35761 ggcgtcatgc ggctgtttca gcaaccggcc gtcacgagcc agatggccga cccgctgacg

35821 aaagcggtgg tgatgctgtc gggtgacctc caagcgttcg gcgacaacat caaggcagcg
35881 gaggacgtgc gcaaatatgc ggccggcgct tcctccttca aagtgatcgg caaatccctg
35941 gacctgtccg gcggcagcgc cgagaccttc ttcggcaata tgaaggaggc cggcgcgctg
36001 cgcaccttcc tcggcgagct gacgccaaac accgatgcgt tcgccaagct ggtgcagtcg
36061 ttccagcaga tggaggacat cgccaagaaa ttcggcgtgt cggtcgacga cgtgcacaag
36121 aaggtcgacc aattcctgaa ggcagcggaa tacaacacgc aagaccgcgt caatcagctg
36181 ctcggcaagc cgcaatcgat cggctcgaag gtcttcaacg acatgcagag cttcgaccag
36241 attgcccagg accagcgcaa caacgtcacc gaactcgcga agcagaacaa agcggaggct
36301 caacggggca aaaacctgct gaattacggc gatttcgtct cattgacgaa gtccgaccag
36361 aaggaagcca aagggctcct caaccagctc aacaaccccg agctggccgg caatccggaa
36421 atgctggcgc aatggcaggc cggctgggat gcctactggc agaaacagat cgacgaagcc
36481 caggccacca tcaagaaccc gaagaagctg gctaaggaaa tcaagcggct ggagaacggg
36541 cgcgattcga ccgatgcctt gggtatcggg caggtggata tcgatgcggc aaagcagcaa
36601 tacatcgagc agcagcgcaa agcctacctg gacgaggcca aatcgcgcgc cggtgtgacc
36661 gtcaccgcgg gctttgccga gcgcgacaaa gccaaccatg aattcttcga gcaagcacgg
36721 gccggcgccg atgcgctggg gctctccctg agtgagctag cggatctaga gcataaggcg
36781 gcagaccaag taaataagga ttggagcaag gcgctcgagg aattcgccgg caacgccccg
36841 atcggcgacg ccgccgaacg attgcaggcg atcgcggacc gcgagcaggc cctggttgcc
36901 gatgctattg cgaccggtag atcggtggtg gcggtgtacg aggcggggga tcaggcgcgt
36961 aagaaagccg ccgacgattt cgcccggagt caggggcaga tcgccgggac gatcgaccag
37021 tggaccgcgg ccttccaggg ctacatggac aatttcaaag gctgggttgc ggacgccaag
37081 gcctacggca ccgacatgtc ggtgaccagc aaagccatca tccagaccat gaaggatgtc
37141 tgggaaaaca tggtctccgg tatcgaatcc gcccgccagg cgattgctca gcagatcgcc
37201 gagcttcgag ggccccaggc cgtcgccgat ctggcggtgc agaacgtcga caaggccaag
37261 cagaagctcg ccgactaccg ggccggcggc ggtaccgaca tcagccggga aatcgctctg
37321 attcagggcg tgcaatcggc ggtaatggct cggtataacg ccgagctggc gctgattcag
37381 caggcggtac agaaagaagt cgaagcgatc caggcgaaat cgcaagcgga catcgaagcg
37441 atcaatgaaa cgctgaacgc ccagatcgag gccgaaaacg agcgcctgaa tgccgcgatc
37501 gaagccgaga acgcggcgct gaatgcccgg atcgaagcga tcaacgccgc cagccaggct
37561 cagatcgatg cgatcaacca acaggccgat gccgctgccg aggcgcgcca ggaagcccag
37621 caggcggcga tggacgcgct caacgagcag ctgcagaccg cgaatcagct caaaaacgcc
37681 atcaaaggaa ttgccgacta cgcgaaatcg ctgcaaacgg cagcggattc gccgctgtcg
37741 cccgaggccc ggctgaacgc ggccaaaaat cagtacgaag acacgctggc caaggcgaag
37801 gcgggcgatg ctgaagcggc cgctcgctat cagcaggacg ccgaagccta taaaagtgcc
37861 gctaaggact actacgccag cagcgcgggt tatggtcagg tctatggcca gatccaatct
37921 gatgccacgg cgctgggcaa taccaacgtc accagccccg acagcgtcga atcccagctc
37981 aaggagttgc gcaaacaaca atccgaagaa aacaaggctg cccagaaggc cgtccaggat
38041 cagatcaaag ccgttcagtc ctcagcgcag gatcagatca aggaggcgca gaaagagtcc
38101 caggaacgca tcaaggcgat gcaggatgcg tcgaaagaca gcatcgccgc aatgaaggcg
38161 aatgccgaag cacagatcaa agcgatccag accgcatcgg cagccgagat caagaaggcc
38221 caggatctgg actcgcgtcc ggacgtgcag gcgctaaaga aggacacgat cacccagctt
38281 gaattgctgg ataaggacct ggtcgccgcc tacgacgccg ccgccaagca atatcagacg
38341 ttcctcgact cgctcgcggg tgtcgtcggc gccaatacga cggtgatcgg gtcacttgtc
38401 gagtggctgc gtagccaggg cgtcaacgtc accgcgccgc ctgaccccga tccgggcgta
38461 tacgagccaa cgtcctcgac cgcgggcggc tcgggcgatc cccgcatgcc gccgcatcat
38521 gcagccggcg gccattatcg cggcggtctc gctctcgtcg gcgaactcgg cccggagctg
38581 atcaatttca accgccccgg ctacgtccac accgcggacg aaaccgcgcg gatcctcgcc
38641 gccgtgagtg ggagcggctc cgccgcgagt ccggatgacc aaatcaacca gatgctcggc
38701 ggcctgccgt tcgacccgcg ccagctgcta tcgatgttct cggcggccaa cgacgcccac
38761 ggcgcctacg ccaacatcct cgacctgccg ccaacatggc cgaacgccgc cgcggccgtc
38821 caaagcctga tgctcaagcc gaccgccgcg ccgcaggcgg tagacatgcc ctccgtcggc
38881 tatcagccgc cgaccaggcc caccgtatcc cgccaaaccg cccagatcga ggatcgcctc
38941 agccgcatcg aagccaacca atccggcacc gaactcaagg gcttgaaaga agcgctggaa

39001 tcgatccgcc gtgagtcgaa agccggggtc aacgtgcaaa gcgcgggatt ctcgcagttg
39061 ctcgcagtga tgaaccaaat gctggcgcgc ttggaagaaa ttgagcgccg gtcgagatac
39121 gcgtcataac caccggagac acccatggca acgaagtcga attacctgga aaataagatc
39181 atcgatcaca tgttcaaagc gacacccttt acggctcccg ctgtcatggc ggtggcgctg
39241 ttcactgtga acaaggggca gcgcgccaac tcgaccgcat acgtcactac agactacgta
39301 tggctcacgg ccaacgacgg caaaacccat atctataagt gcaccacggc tggcagcacg
39361 gctgccgctc aggcaccct gtatcccggc accgccaacg aggtcatcac ggacggcacc
39421 gccgtgttca ccgagcagtc gtcgcaattt gacgccaatg cgctcaccgc tctggaggtt
39481 tctgccggcg gctacgcgcg agtcgccaat aacccttcgg ccacagccaa ttggctgaac
39541 acccaaggca cggcatccgg ggcgtccacg ggtgccggag gctcaacgag caaccagaac
39601 gccatcacgt tcgggacgcc gtcagcctct tggggcgtag tcgccgcgat cggcatttac
39661 gatgcggcga gcgcaggcaa cctgctgtat tggggttaca tgacgacgcc gaaaacgatc
39721 aacaacggcg acccagcgcc ggcgtttgct gcgggcgccc tcacgatcct ggaagacaac
39781 tagccctgag tcggcggcgt cgcagtggca agcgtaaccc tacgcccggg cacgatcgta
39841 tcagcgaccg gggctggatc ggtcgcatgg tccaatcccg gcaatgccgg cgcgtcggat
39901 aatgcctatg ccacggcgaa tgtcggcacc tcgcccggcg aatcccagtg gctgcgctgc
39961 accaacttca atttcgccag cgtcatcccc gccggtgcga tcatcaacgg atttacgctg
40021 acgatcgaac gcaaagggtc ggcgatcaac tcggtatacg acacccggct ttacctctat
40081 tccggctccg ccgcaatcgg cagcaacaag gccgtcgcaa ccaaatggcc gacctccgac
40141 ggcatagcta cctacggcag ctcgtccgat ctgtggggaa ccaccgggct gaccggggca
40201 gacgtcaatg cgaccgggtt cggcatcgag ctcgcggtta actcgtcgac gtttaccacc
40261 acggcgtcgg tcgatgacat cgcgctcacg atcgactaca cgccccaaat cgatctcgcc
40321 gcttacctca gcagtgaatc gttcgtcctt gccgatctct ccacgtcgat aacgtttcag
40381 agtgatctgc aggcgttcgg aggcttggca gcgacgctct cgacctcgat tccgcttcag
40441 gctgccctgg tctccgagtc agcgttttcg ggcttcttct cggtggccct gtcggccgac
40501 ctttggggtc aatccgctct caccgctgat ctatcgaccg gcatcgcttg cagcgcgaat
40561 ttgcagggca atgcctggac cgctgcagat ttgtccaccc agatcccgct cagcgccagc
40621 tgcgccgggc agtcctcgct gacggcggac ttcaccacgc aaatccgttt ctccgcggcc
40681 ctctccggaa tctctcaagc ctacgcggga ctgactaccg agatcgcctt ggcagccgtg
40741 ctgtcgggca atgcggaggc aaccgcggcc ctcaccatcg gccgagtctt catcccgccc
40801 gcgtcccagc gcatctacct cgccgagatc acctgctatg acccggcctt gccgggtatc
40861 cgcaccttgc gctacagctc gggggcggat ggctacgacg atgccggcac gttctatccg
40921 ccgctgatcg agcaaccggc gacgatccgg cgggaaatcc cggccgatgt cgccggcggg
40981 cacgtgtcgg ccagttatgg cgcgctgacc ctggtcaacg ccagtggcgc gctcgatccg
41041 ctgcgggact actactatga cggttacgaa ctgacgctta aggtgggcga tgccggtgcg
41101 ggctatggca cgttcgtcac tcggcttaga gcgacgatcg acaacgcgag cttcgagcgc
41161 aatcgcgtca cggtccgact gcgcgatcgg gccgagaccc tgaaaacgcc gctgcagacc
41221 aatacctacg gcggcaccaa cgcgctcccc aatggcctgg acggtaccgc cgacgacctc
41281 aagggcaagc gcaagccgct cattttcggt cggatcgccg cgctgcagcc ggtgctggtc
41341 aacacctccc gcctgatcta ccaggtcaac gatggtgcag tggatgccat catcaacatc
41401 ttcgattcgg gcgcttatct cacccgcggt accgactaca ccgccggtca gcagtcgacg
41461 tttgaaagcg ccgcggtcgc ggccggatcg ttttccactt atgcgccggc cgggctcatc
41521 aaactcggct ccaagccgtt cggtaccctg gccgcctgcg tcgccgagcg gtgggattac
41581 acccagatat ccgcggccgg cgtcacccag cgcatgctgc aaatgcgggg ctatggatcg
41641 gccgactgga tggcgcagga ttttctcgac ctcgatgccg tcaactgcgg gtcagtcggc
41701 gttcagacag aagatggcga gaacgttgac atcctcatcg accggacgct gggatcggtt
41761 ggggcgtggt ggggattcga cgcattgaac aggttccgcc tgcgccgcct ggatgcgccg
41821 accggtaccg ctgtggccac gattaccgac gaccatatcc tcgaatacga ccctgtgcag
41881 tccagccagc gggcgctctg gcaggtcacc ctccagggcg atgccaacta tgccgtacag
41941 gacaaggcga acctggccgg catcgtcgcg gcggaccggg cggcctggtg ggagaaggcg
42001 acgcgcgacc aatcgtcgac cagcactgca gtgaaaacga cccgattgct ctctcaggac
42061 gcaacatttg ccagcgtcgc gaacggcatc agccagaccc aggccgaagc cgtccgccgt
42121 cgcagcctgt tcgacggccg acgcgacacc ctggggctga cggtcgcagc cccgaatgac

42181 tggatggacc tcatcgatct cggcagcgag gtgacgatcg tatccgccaa atgcggctat
42241 cccgccggcc gcaacatgat cgtcgtaatg acccagccgg acttccaacg caaccgcctc
42301 gacctgatcc tgtggggatg acatgcagcc tatcgtgatc ggctacgcca atctgatcga
42361 catcgccacg ctgagcggtc cgggctggca gacgagttat ccgctgagca acctgcagaa
42421 ccgttatgcc ggcagcaagg cgcgcgcgac cggactgact ggtgtgatcg atatcaatct
42481 cggggtgccc gacgcggcca ttcggacgtt tgggatcaaa aaccacaacg cggctacgat
42541 acggctgcag ggctcgtccg cgagtgattt ctcgaccgtc gcctacgaca cgacggtgct
42601 gccgacctac gccgacacct cctacctgcg ggcactgccc gtggccgttg tcgcccccta
42661 ttggcggtta actctaacgg ggacgtcggc caatccggaa ctctcgcgga tttttctcgg
42721 cgccggcatg cagccggctc ctgagaacaa cattgaatgg ggcgcctcac tccggatcga
42781 gagtaaaacg gaggtggccg agtccctgag cggctacgag gatttcaacg tcaagccgaa
42841 tcgccgggtg ttccgcggcc ggttcgattt tctgcccgat gccgatgcct ggggtgtgct
42901 gctgccgatg atgcggtcgc agggcatcca ccgcgagtat ctcctgatcg ccgatccgga
42961 cgatacgacc taccaggcgc agcgcaacat gttctgcagg atgcgcgaac tcagcgagat
43021 cgaggatccg tacctcaacg cccatacggt gggcgtggaa ttttcggagc tgctgtagat
43081 ggcgcttgca ctctaccggg aaaatgcaac cggattcctg gtgcggcggg atacgcccgg
43141 cgctggctat accgccatcg ccgccatgcc gaccggcagc atcgccgccc gcgcgagctg
43201 gtaccgggac ctggacaccc acggccagtc cagcgcctgg cacccgtcgc aaagcgcgcc
43261 ggggccggaa aatgcggcgc caagcgtcga ttatggcaac gtcaccggca caaaaccgcc
43321 agcggatgcg acgcataaca agatctggcc cccctcgacg tccgcgccct catcgggagt
43381 gcagtcggga gacatgtggc aaaacagcaa taccggcgtg ttctacacct acaacggttc
43441 ggcctgggtg ccggtcgcct cgaagaacat catctggcct ccatcgacgg tcgctccctc
43501 cggcgcaaac gcgggagatt tgtggcagga cagcagcacc aaaacgttct acacctacaa
43561 cggttcactc tgggtgcccg tatccgatgt cacggcgtat aacacggccg cagcaattac
43621 cgggcaaggc tcgtttgcga ccatcagcca gatcgtcgca gggaacattc cggcattgat
43681 tgccgccgga atcatcgggg cgaattacat cacggcaggc gccctgcact cgcagtgggg
43741 gaccgttggc aactcaaacg cgccatacgt ggcggctgga aattggacag cgggagcgtc
43801 cgggtgcatc tggaccaatg tcgccgtgtc tgccaatgcc taccgaacat ccatagttgt
43861 attcgcgttc gcacaatacg gcgacggcac tcggcaagcc gacaagccgg ggtacgtcaa
43921 tgtcgtcgtc ggcgtgaatt cgcagacact gaccgaccaa gccacgaacg aaatccttgg
43981 cgggcaagtg caaaaagaca atgcgccaac ttacatcacc atgccgggca tcgcattagc
44041 gcccatgatg tattacgacg tagcgcctgg gactgcgggc cgaacctatt acatcggctt
44101 tcggcatgat gccagggcca acggcacgga cacgatgccg cttcggtacg ccattctggt
44161 tatggaattt tcgaagtagg agaaagagat gccaaacaag caatatcgcc gcgttgctac
44221 tcgcaccgga gaaatcctct ctgtagtgtc gattcccgac accgatgcgg ccagggattt
44281 gtacaacacg cacctcgaag agggttccgg agacagtcgg acagagtatt ccgtgaacgg
44341 catactgacg cctcgcccgc cgatgcccta cgtgctcgac acggacacga tcactgccga
44401 cggagcggat accgcccgca tgacgtgcct tccaatccca gcatcaatca gggtgtccgg
44461 cccccacgga cttcaggtaa tgaccatcga cgacggagaa ttcgagttcc aagccgatat
44521 gccgggagac tatcagctgg agatcgaggc gttcccctac cggcaagcgg aggtcgtaat
44581 tcatgcggtt taaggccacc ctgccgctgc cggagctgcg gcggctgaag caggaccagg
44641 tcaatgcaaa acgcgcagag cgcctggccg aaggctatgc gttcgagggc gccattttcg
44701 ccaccgatcc gcagtcgatc gccaacatga ccgcggtgct ggccgccata ggcgccggcg
44761 ttgacctacc ggacgggttc gcgtggcgcg atcagaacaa tgtcaacgtg ccgatggacg
44821 ccgagacgtt caagcgcttc gccggcggtc tcctcgcacg ggtgaaccag atttataccc
44881 agtcctggag caggaaggat gagatcgaga ccagcgacaa tccggcgtct atcgatgcgg
44941 atttgatgct ttgaaagaca gcgactggcc gagtgctacg aacacccggc cagccagctg
45001 acccgcagcg tatacctgca agccaaccca aggctgccac cgaatggcca ctcggtgtgg
45061 caagcctagt ggatttctaa cgtttctgaa agaggcttgc gtcctatgca aaatgcgaaa
45121 cctatcgtcc cctggatcgg cggcaagcgt cgtctggcgc ggcatatcct tcctttgttc
45181 ccgcctcatg agtgctatgt cgagccgttc tgcggtgcgg cggcactgtt tttcctgaag
45241 gagccggcca agtgcgaggt tatcaacgac ataaacggcg agctggtcac cctgtaccgg
45301 gtggtgcagc atcacctcga ggagttcgtc cgccagttca agtgggcgct gacgtcccgg

45361 cagatctatg aatggatgaa ggccacgccc gaggaaacac tgaccgacat ccagcgggcg
45421 gcccggttct tctatctgca gaagctggcg ttcggcggca aggtggagtc caggactttc
45481 ggcacgtcga ccacgtcggc gccgcggctt aacctgctac ggctcgagga ggagctgtcg
45541 gcggcacacc tgcggctgtc acgaaccacc atcgagcatc tgccgtggga ggagtgcatc
45601 cgccgctacg accggacgca cacactgttc tatctcgacc cgccctattg gagcacggaa
45661 ggttacgggg tggagttcgg gttggagaat tacgtccgta tggcggagct ggctcggacg
45721 atcgagggac ggatggttat ctcggtgaac gatatcccag agatgcgggc ggcattcgaa
45781 gggctgccga cgaaacacgc cgagctctgt tactcagtcg gtggcggtaa gcgatcccgc
45841 ggatcgagcg gtgaactgat cattcggagc tgggcatga
//

LOCUS   16-5-R3   52455 bp   DNA   linear   PHG 16-FEB-2025
DEFINITION 16-5-R3.
ACCESSION   16-5-R3
VERSION   16-5-R3
KEYWORDS   .
SOURCE   .
  ORGANISM .
            .
FEATURES   Location/Qualifiers
     CDS   2..4294
                     /ID="VIXMOERF_CDS_0001"
                     /transl_table=11
                     /phrog="25411"
                     /top_hit="No_MMseqs_PHROG_hit"
                     /locus_tag="VIXMOERF_CDS_0001"
                     /function="unknown function"
                     /product="hypothetical protein"
                     /source="PHANOTATE_1.5.1"
                     /score="-2.1668098886861348e+18"
                     /phase="0"
                     /translation="SDNGRSAKSMGWATNMARKRISKSTLSMFLRTKCDRELYLSLHED
                     SELDANGMPVPLQARPGIGVLQTAGRDFEDERNDQLIQSFGNLVMYQPDKGGANKPVKA
                     PLASLLGKVTALPSIILQGKFEPSAFQNAVMANIGLQPAQVAQVPRIAGLIPDIIVVRQ
                     ATVDDEEVGADGCRRPIDPTTETRRALSIIDVKHTSEANPSYSAEVALYAIFLANWIVD
                     QGLQNSYFVTIRSYLWTRFKQGQSALGALMSGSSPATPDQYLDVLIADSEDANLRFYLP
                     TVLHFFREDLLRVITIGDASANGWENLEWHVDGRCSACDWLGHEKWANSKDKARIAAQP
                     AHYCYPAAKLTGHLSQIAGMTRGARKTLQINAIQNTAGAASAPSAHPAFQQHSHLKKER
                     SRIPVRAQALISSTTSVDSSAVLASLAPWPQLHVAITVNFDPSAGLLTGLSIFGRATAY
                     VSGQTPRQFATKCFVVDQKSLSDEWVALEGLLSTLSDMVDQSEAFVRAAGKTPLTAQIA
                     FWEKRQFEELCAAMGRHLPKVLSLTNRKTKALAWLFPADELIEKPDGAVSPCVVFVDEI
                     VQRVVFAPTPHVITLFDTAEAYYSGSGPIRQGDAFYREFLTNGIPRERIYEIWSNVTTI
                     KRGSVTVPRNTVIQEFGNALEKQCRALSSVVEKLRTDFRGQLKANAPKLTLSIPQGARD
                     VAFDSKLWIWWEELQYHTRKLESHQRLALDAEALEASYEAVRLTNGQPTGIPTEYNFDV
                     LAGSTEAKLDDNEGYLALGKEAHPGLPLLRAKDIVAAGSPPYSGSDQTITTPLWSSLSV
                     TLVSFDRSTRKAVLNLSNWREAAFFPYLRDNSTIDLLNDIFITKGQGSFKWYETAKNIL
                     TTVGNPSLAVADSNAATAMGARPPRPGTDPVTPLARVLWEADILHATSVVAATPAASVA
                     AYAKAKHNLNSSQTDAVAHATEKQLTIIWGPPGTGKTQTLAGCIHGLVHDAAPNRQPLK
                     LLVAGPTYKAVEEIIGRVVDALDNDATCPAEVYVGYSSSQAPKAFPAGSSHLRVESFKL
                     DRTNQETQDCLASLAKADAVTIVGTAMMQAYKLAEWTCGSSVGPIFDVVIIDESSQVQV
                     TAAISALATLKEDARLIIAGDHLQMPPIMALEPPNGAEYLVGSIQKYLLDRPFGSQIVS
                     CPLEENYRSAEDIVAYARTIGYRATLKASNAATALHLLAAVPTSASGFPGGMPWSTLWP
                     QILDPKKKVLTLLHDDDLSSQSNSFEAKIVAALTWCLRQTVSAELDGRGAVTHAAPTPD
                     QFWGQCIGIVTPHRAQRALVVRELRSIFPSDPPDLIDSAVDTVEKFQGGQRHTIIVTFG
                     VGDADVIMGEEAFLMQLERTNVAISRAMAKCLVIMPMTLAGHVPHDKKALETAHAIKDY
                     VDEFCNQEMTDQITSGPTPKQAKLRYHQ"
     CDS   complement(4307..5458)
                     /ID="VIXMOERF_CDS_0002"
                     /transl_table=11
                     /phrog="18146"
                     /top_hit="No_MMseqs_PHROG_hit"
                     /locus_tag="VIXMOERF_CDS_0002"
                     /function="other"
                     /product="DNA methyltransferase"

/source="PHANOTATE_1.5.1"
/score="-18715.781696601527"
/phase="0"
/translation="MTHNTRTKNVALAYSKENALNAICPYFTMFPLEYPAKVLNKHRKE
RPVVLDPFCGRGTTLFAARTLGLAAWGIDTSPVAVAIARAKLASCDTEEPLRLAERLIS
EVDPSEIPDTAFFRSAYHRDTLRNVCALREGLLNLGAETDASAILRAAALGCLHGPVPK
HMENAGYFSNQMPRTYASKPDYAVRFWKERKLKAPRIDVIRVLRRKIERLVGLEQESPC
PISQVLHGDAQSVEVFRSVSTAPSVVITSPPYYGMRTYVQDQWLRNWFLGGPDFIDYSA
GPQLDHGGQSVFATSLGRVWRNIADSADSSETLHMYVRFGIIPSAAVDAKSIFKNSLEE
SGVPWRLVSTRPAKSADAGKRQADQMKAESAAAVEFDFHVERV"
CDS complement(5474..5752)
/ID="VIXMOERF_CDS_0003"
/transl_table=11
/phrog="7"
/top_hit="No_MMseqs_PHROG_hit"
/locus_tag="VIXMOERF_CDS_0003"
/function="lysis"
/product="endolysin"
/source="PHANOTATE_1.5.1"
/score="-0.6974708969487556"
/phase="0"
/translation="VIRTRLTAIEDFTFNLGGGRLQTSALRRKVNQRDWIAAASEQGRW
VYGGTGCSPDWSRGEPQNVCSYSGKTDVKRSRNVTLLRSPSAVGRVN"
CDS complement(5786..5917)
/ID="VIXMOERF_CDS_0004"
/transl_table=11
/phrog="No_PHROGs_HMM"
/top_hit="No_MMseqs_PHROG_hit"
/locus_tag="VIXMOERF_CDS_0004"
/function="unknown function"
/product="hypothetical protein"
/source="PHANOTATE_1.5.1"
/score="-4.923951958251154"
/phase="0"
/translation="MRLRAGEVLYRSEEDLRKPLESVVKNRGWSSSFWIWGRFEKGA"
CDS complement(5980..6822)
/ID="VIXMOERF_CDS_0005"
/transl_table=11
/phrog="No_PHROGs_HMM"
/top_hit="No_MMseqs_PHROG_hit"
/locus_tag="VIXMOERF_CDS_0005"
/function="unknown function"
/product="hypothetical protein"
/source="PHANOTATE_1.5.1"
/score="-4916.985226054536"
/phase="0"
/translation="MGSTGYGEEISGSSIVGIQSHDGANQKVYYFGSVDTVYGEDYTDN
TCRKARSLKVYASHPYAGYPVMERVTIRDGTGEPPPGPPYDTAYGMNGVPGEFFFISEG
LIYVESDLLARIIWMDPRQNNYLFAKSTHQGAWYGTTVQNSTDPFFDARLSSILYGVAP
GLASGEIVNIDTLEKFLGGKFMTEVLHTGWPETEGALVKCKSFKSIDYGGCVTLPDSGI
TVASFKLPVWHESADPPQRLRYRTFSAISTGQQLREVTGLSAGAPGSYADFYAPLNLY"
CDS complement(6864..7070)
/ID="VIXMOERF_CDS_0006"

/transl_table=11
/phrog="No_PHROGs_HMM"
/top_hit="No_MMseqs_PHROG_hit"
/locus_tag="VIXMOERF_CDS_0006"
/function="unknown function"
/product="hypothetical protein"
/source="PHANOTATE_1.5.1"
/score="-0.8172850385577193"
/phase="0"
/translation="LIATGSPGDYDPGRNNNQRHAGESFVDYTIERGYESHFDLPVYVY
YDDEDMKSFIRFFGKILINTPRK"
CDS complement(7067..8350)
/ID="VIXMOERF_CDS_0007"
/transl_table=11
/phrog="No_PHROGs_HMM"
/top_hit="No_MMseqs_PHROG_hit"
/locus_tag="VIXMOERF_CDS_0007"
/function="unknown function"
/product="hypothetical protein"
/source="PHANOTATE_1.5.1"
/score="-111710.86557035348"
/phase="0"
/translation="MKTIERPGDPFDLVRIVDIRSQGLLCTPTAGNYPDGWGEPFKDAE
GAPIHPPLGTYAPAAGAIGVELSAETDPKKPKALRVQRRASGRKVGNCLVQRKIGKRWI
RVSFDGPFNRVFVDNDLQPYGWDVGTDRYSEEPWSNYIYLRGKRLYIDAKKNVYDSDFF
GKIVGVGIRDENTLVLAAIHRNEIAFAPLDISKPYADGQTVSVTYYKLTPQVQDLPGYT
FYPGFTVKPTPPGFVFHGPIYPQIDTPFEFNSDCTQASCIAKSYRFPDTSMYVARRHWR
IANGYKSEEEGDVDSEFSDGVPYNWANSDLADDVEAVITFLNESPMESVINKLGAARLN
PNFKRVPFFQRYLHDVELIWSQDDKNKTNKKKVLSAVQGRATRVDAHQGVSRSGRPTAP
GSKRSTRTRRPCPGRPTRYTTPTPIRRS"
CDS complement(8347..8712)
/ID="VIXMOERF_CDS_0008"
/transl_table=11
/phrog="7250"
/top_hit="No_MMseqs_PHROG_hit"
/locus_tag="VIXMOERF_CDS_0008"
/function="unknown function"
/product="hypothetical protein"
/source="PHANOTATE_1.5.1"
/score="-23.266232186943856"
/phase="0"
/translation="MTVQKFFVNDINAIVDAVRSGGDDLPEPAAKPGIGARTGVGAPKR
GGGIASPLSETTAPNATGEFWADREYYPARDMQTTDGEFVIKYAAIKTLKFQDANGEAV
EFHFAEPDPDKALGTTA"
CDS complement(8737..9162)
/ID="VIXMOERF_CDS_0009"
/transl_table=11
/phrog="No_PHROGs_HMM"
/top_hit="No_MMseqs_PHROG_hit"
/locus_tag="VIXMOERF_CDS_0009"
/function="unknown function"
/product="hypothetical protein"
/source="PHANOTATE_1.5.1"

/score="-30.816145355174385"
/phase="0"
/translation="MSSFKLYTDAALTTPLTGSLVATQNADGSTPAIQFTLYLGSTTAS
RMIQANANPGVDQIQATVVDAGAGSGHDVSEVKLAATQAGLASAVGGAALDLGTSVLSD
AANAKPIWIEVNDATHVVGTATELSVVIADVRETAVV"
CDS complement(9167..11482)
/ID="VIXMOERF_CDS_0010"
/transl_table=11
/phrog="5842"
/top_hit="No_MMseqs_PHROG_hit"
/locus_tag="VIXMOERF_CDS_0010"
/function="unknown function"
/product="hypothetical protein"
/source="PHANOTATE_1.5.1"
/score="-232860598.58947396"
/phase="0"
/translation="MTLPVGGVTGIGGVAAGPDVGERYALTAVLEQAVFSVYGLGTGIT
QAVVDIDAYDVFCVIDQPVFERFGLSVAVEQTLLDVYGMTAPLRFEVLAVAGNSAPIQQ
VQFSISLLAAPLQQSIFFKPYRRAAWLTQQIADVYGIAAPITLVVDDLPFDLAAQVELT
VYEVLTAMDVPIRHTVFDAQAVGGGAFVAQAMSASADITAWAVLVNIGNAAGDGEPLNA
GTGAVMGALLGRLTGQMTVDAEEGAARIAEFSVLPEPGPININALTGLPITIYLVAGTT
HLPIFQGILDIPGWDPVQGVLQLSCTDNLQNRFDGIKRKDIAKIIGGFWSEYVFDKKAD
EWQYLQDRLSTLPASYDLDLNLQGVVTPWRAKTAPDFTFRPDAVLENSIRIDMASARQL
VNTIDVKMQYRYERLMQRDVGIFWEMDMHELVAGGAAPSVETVFQAVDGTGWACYGLEM
LPVPTGAFEGVGLLGDVPRYSLTVGLHCALAQRWTQTVTEDWSVTVDAPDSIQVIGKRK
GSTTANFDSNADKDPRYTHWDRITERETKFTLAGQGTISPIAGDTSFIPYHPLNIKQYT
VATPDGAKRLPSQDVLANPRGDLYYDLDDGVADNRAGLSNGYMTGIARAQRDILSSHRQ
TTLSFTGLIAPLLDRTHTVRMLSARLTAKGKVRRVTHNLDFDAGSALTEVAIAVSKAYG
VGMVDTPYDLQAPAKPAVVQPPLKYPMERPSASYDSSTGEFMVTVPGVAQEHIDATHSA
PADHVSIFIPDDELLLEA"
CDS complement(11482..12231)
/ID="VIXMOERF_CDS_0011"
/transl_table=11
/phrog="No_PHROGs_HMM"
/top_hit="No_MMseqs_PHROG_hit"
/locus_tag="VIXMOERF_CDS_0011"
/function="unknown function"
/product="hypothetical protein"
/source="PHANOTATE_1.5.1"
/score="-349.63093854832425"
/phase="0"
/translation="MLNLAGLNGVKAMVTDALVAPSDFTMNVSALGAPWVLPPVLASGE
YYYLTIIDQDYPTKWERVKVTACQGMGANCLLAITRNVASSTGVAQTFTQGAFVQWSPG
VEEMESRWRMILSASMTGTNTMTSTRFVAPFSGSTTGSMGTAYWINRTGKTLRLSNLVA
ALSTGSSFSGDSIVVSVEAGSTAPTSPSALQVVVSPGDYGFKSNNVNTVTVGPDSLVCF
MLSRSNTGAAVSGGFQLANVSVLAEEL"
CDS complement(12233..12580)
/ID="VIXMOERF_CDS_0012"
/transl_table=11
/phrog="5314"
/top_hit="No_MMseqs_PHROG_hit"
/locus_tag="VIXMOERF_CDS_0012"
/function="unknown function"

/product="hypothetical protein"
/source="PHANOTATE_1.5.1"
/score="-9.468999951354942"
/phase="0"
/translation="MWSGRSVKQQTWRKLATEISGGGWVPPGLSALDYSQPLVLACAAP
LSVVGGGSPPVPVRTEPDFATVTIDVDGVAITYYYPKITVYCEPPEQQLDGVSGNYSWR
LNAEMVDPLGA"
CDS complement(12691..12975)
/ID="VIXMOERF_CDS_0013"
/transl_table=11
/phrog="No_PHROGs_HMM"
/top_hit="No_MMseqs_PHROG_hit"
/locus_tag="VIXMOERF_CDS_0013"
/function="unknown function"
/product="hypothetical protein"
/source="PHANOTATE_1.5.1"
/score="-6.3674218912455185"
/phase="0"
/translation="MRCASALPGSARHDRTDATGNHVVHGRRAGIRRACRLTAGVGVVL
TLTGCCSLELWVTLAPRACAWPVRATDLVAGVDEVGLALRCDEPEPDER"
CDS complement(12941..13414)
/ID="VIXMOERF_CDS_0014"
/transl_table=11
/phrog="7"
/top_hit="No_MMseqs_PHROG_hit"
/locus_tag="VIXMOERF_CDS_0014"
/function="lysis"
/product="endolysin"
/source="PHANOTATE_1.5.1"
/score="-42.48661920411991"
/phase="0"
/translation="MRRTTAAAMSASALIVGMIALHEGYRGEAYDDGVGVQTIGFGSTA
GVKRGDRTDPVRAVQRLAADATRVSQAVARCVGEVPLYQHEFDAYVSLTYNIGVNAFCG
STLVKKLRSTPPGYAEACREILRWNKAGGRVQNGLVRRREAEYALCVGTARVG"
CDS complement(13411..13677)
/ID="VIXMOERF_CDS_0015"
/transl_table=11
/phrog="No_PHROGs_HMM"
/top_hit="No_MMseqs_PHROG_hit"
/locus_tag="VIXMOERF_CDS_0015"
/function="unknown function"
/product="hypothetical protein"
/source="PHANOTATE_1.5.1"
/score="-15.361863189404902"
/phase="0"
/translation="MFEKVKYLYECMQAGKSLQNPRLWSQRASLIAALTALLTAGVGLA
RALGYDVDATGTDIAAVAQGLGVLGVIVVDVLHRASNKEAGRR"
CDS complement(13720..13995)
/ID="VIXMOERF_CDS_0016"
/transl_table=11
/phrog="No_PHROGs_HMM"
/top_hit="No_MMseqs_PHROG_hit"
/locus_tag="VIXMOERF_CDS_0016"

/function="unknown function"
/product="hypothetical protein"
/source="PHANOTATE_1.5.1"
/score="-41.56902776876613"
/phase="0"
/translation="MNPILRWALWLVVQAVSGITKEQWGLIQDKVAALETRTIKGIVDK
TTLNDIKKKEAAAYIGTFVSGVRTNVVHFLIEAALWFVRSFGVKAP"
CDS complement(14024..17047)
/ID="VIXMOERF_CDS_0017"
/transl_table=11
/phrog="339"
/top_hit="No_MMseqs_PHROG_hit"
/locus_tag="VIXMOERF_CDS_0017"
/function="tail"
/product="tail length tape measure protein"
/source="PHANOTATE_1.5.1"
/score="-550746831.5416873"
/phase="0"
/translation="MTTFKTVIEIAANTAAAEKGLGNVAAQVDRLTGSLRRIGQYTVGA
FGAAEALQAARDLGKLSDQYRNLEGRVKLAAGSQNQFTEAQRALFAIAQNNAQALTGVS
QLYSRIAKGAGDMGVSQQQVLSIIDSVAKSFRISGASAEEASGATLQFSQALASGVLRG
DEFNSIMEQSPRLAQAIADGLDVPIGKLRAMAEAGELTTQKVVGALQKAKASIDADAAG
LPDTIEQAVVRWDNAALKFVGTSTEITRAAEAIAGAINTAAGNIDTIANGVEIAGSLLV
AVLAGKGTAAVAAFAGSQARLVQANLAAATAARNHALNEEYNARATLAAAEAAVTNASG
MARLALVETQLVPASQRLAVAQAEVAASAGPLKIALGGLASFLGGPAGIAVTLLTAVSA
WHLFGQAGTSEIERIIQKRRELAKETGKDTRGKSEADLSLLQDEAAVKKQEAVVARLAA
RYKEVGQAADQAFMGGRIGRELAGETTLLQEMRAELAKRKQAAAESGDTVKAKEREVQQ
SFKDTQNALKDATAGLEDAYQRRTAAIDALEKLAETRIQAQPIEADAAPPIVYARNGVQ
ATQARSELEQARQLHALTLQAEGARLKAVRDYAAQRLGLVDQVYAQEIAKFKDGEARKA
ALERESLQARRAIYADLESAYTATIDKLIGQEQRLLESARASAQERRSIEQSTARAVAG
IEQAALTPLQAARAQFAQLQQLLAQQRQELANGNLELSRQAGEQAAGLAEALGQQAAAS
LREQRLNALKGEQTPADPGQPQFSTEDLQRLIEARRQAGQVLAEAAARQEQADTQAAAA
VSQKIQETLQNLQSVQAEISDIDQQLTKGFALRVDVDPASLQTLQSQLAELLKPETKTI
TVVMNSQGGATGGDAALPGFRRGGWIKGYGGGDRIPALLEEGEFVLRKEAVRKLGLDKL
YALNNLALPRFSIGGYVAQAVPAFDASLARSSNGGQAVHIHLPGIPGSFPLNGDPQVVA
ALKREVARAALKHGRIVR"
CDS complement(17052..17147)
/ID="VIXMOERF_CDS_0018"
/transl_table=11
/phrog="No_PHROGs_HMM"
/top_hit="No_MMseqs_PHROG_hit"
/locus_tag="VIXMOERF_CDS_0018"
/function="unknown function"
/product="hypothetical protein"
/source="PHANOTATE_1.5.1"
/score="-1.557734974862574"
/phase="0"
/translation="MTALALIERGHAGVWHYPWRLFLQARLMRRR"
CDS complement(17159..17518)
/ID="VIXMOERF_CDS_0019"
/transl_table=11
/phrog="7608"
/top_hit="No_MMseqs_PHROG_hit"

/locus_tag="VIXMOERF_CDS_0019"
/function="unknown function"
/product="hypothetical protein"
/source="PHANOTATE_1.5.1"
/score="-10.558604759800332"
/phase="0"
/translation="MAELDTTHPKGLRAERTIEAGGRKVVVRELTVGEVRAWLKDANAE
LDRNDLVALALFADITLDDLTRFSDLSRAELDAMLPSELDKVREAAKSLNPHFFGLRER
LANAAQAAATAPPAT"
CDS complement(17525..18028)
/ID="VIXMOERF_CDS_0020"
/transl_table=11
/phrog="807"
/top_hit="No_MMseqs_PHROG_hit"
/locus_tag="VIXMOERF_CDS_0020"
/function="tail"
/product="major tail protein with Ig-like domain"
/source="PHANOTATE_1.5.1"
/score="-105.2904133435738"
/phase="0"
/translation="MTTTAMLLTGTIKGAVGAGTPVDLGNAALDLSIEEDTKELADYQN
PGGGSIASLSRIKSVTLKLKLWSISKENLALATRGTVSGNSIEALTQTAEDWHITFDGV
NEVNGDAVAYDFYKVKFSPASSLPGPGTEDFAVLELTGKVLKDTSKTGAGVSQYFKATI
TPAV"
CDS complement(18092..18529)
/ID="VIXMOERF_CDS_0021"
/transl_table=11
/phrog="209"
/top_hit="No_MMseqs_PHROG_hit"
/locus_tag="VIXMOERF_CDS_0021"
/function="head and packaging"
/product="portal protein"
/source="PHANOTATE_1.5.1"
/score="-11.006465536888353"
/phase="0"
/translation="MIASLFEIEPALIRRLRETLPEFVTVDSVGILAGVQNLEPLCPAA
LVLPLGFAGPVGTPPPNVFLAERQRWQVTVCVAHAPPASTVVTGGEYVLRILRALEHWS
PQPGWGRMKHVGLDDPWFDLGHVEFSLVFEVRPLPLDTGTP"
CDS complement(18526..19038)
/ID="VIXMOERF_CDS_0022"
/transl_table=11
/phrog="27"
/top_hit="No_MMseqs_PHROG_hit"
/locus_tag="VIXMOERF_CDS_0022"
/function="connector"
/product="tail completion or Neck1 protein"
/source="PHANOTATE_1.5.1"
/score="-16.734043423372007"
/phase="0"
/translation="MSLGLRVQLHGDTAQDLRRLPAVLRKASRRAVGQTARETREAMVR
AIGATYGIPLRALRSRRVQAYLRLAGFSARVWTGHAPIKAAYVGRLRQEEWGSSAGAYL
FPGSFVARMPSGHRGIFHRVGRGSLPILEDVVALPQVPALAEALAGRATLRLAAMIREM
TQDALPR"

```
CDS             complement(19048..19374)
                /ID="VIXMOERF_CDS_0023"
                /transl_table=11
                /phrog="785"
                /top_hit="No_MMseqs_PHROG_hit"
                /locus_tag="VIXMOERF_CDS_0023"
                /function="lysis"
                /product="holin"
                /source="PHANOTATE_1.5.1"
                /score="-7.683588363727942"
                /phase="0"
                /translation="MNAPPKKLLLVSPHGNGDEVRLALQIILVTIWSGLVAQLNRWRRH
                PRPWRPCLWCLLVDVVSCSLVGFSVWLLAEREGLGYYESLWAAIVAGHLGARWFGLLIH
                SSRG"
CDS             complement(19371..19679)
                /ID="VIXMOERF_CDS_0024"
                /transl_table=11
                /phrog="82"
                /top_hit="No_MMseqs_PHROG_hit"
                /locus_tag="VIXMOERF_CDS_0024"
                /function="connector"
                /product="head closure Hc1"
                /source="PHANOTATE_1.5.1"
                /score="-3.9645901495950255"
                /phase="0"
                /translation="MDLDAVNAVCLSTFGETVILWPDRTDPPPVHLTGIVSVPAGLERP
                GPMGAPRPEADPRLTVRSADLPAVRMGDPLKLRGLPYGIASRLDRLDGLTTLILRPR"
CDS             complement(19682..20683)
                /ID="VIXMOERF_CDS_0025"
                /transl_table=11
                /phrog="29"
                /top_hit="No_MMseqs_PHROG_hit"
                /locus_tag="VIXMOERF_CDS_0025"
                /function="head and packaging"
                /product="major head protein"
                /source="PHANOTATE_1.5.1"
                /score="-1602.813394547418"
                /phase="0"
                /translation="MQNPFHNPAFSMAALTAAINIIPNRYGRLEDLNLMPVKPVRQRQI
                LVEERNGVLNLLPTLPPGAPGTVGVRGKRTLRSFVIPHIPHDDVVLPEEVQGIRAFGSE
                TELEAVAGVMARHLETMRNKHAITLEHLRMGALKGVILDADGSVLYDLYDEFDIPPKTV
                GFQLGAATTDVKARCMEVLAHIEDSLLGEFMTEVHCLCSPEFLAALTGHKDVKTAFTHW
                QQGAILINDVRRGFTFGGITFEEYRGRATDVHGVTRRFIAAGEAHAFPLGTVDTFATYV
                APADFNETVNTLGQPLYAKQAPRQFDRGTDLHTQSNPLPMCHRPGLLVKLTV"
CDS             complement(20686..21063)
                /ID="VIXMOERF_CDS_0026"
                /transl_table=11
                /phrog="49"
                /top_hit="No_MMseqs_PHROG_hit"
                /locus_tag="VIXMOERF_CDS_0026"
                /function="head and packaging"
                /product="head decoration"
                /source="PHANOTATE_1.5.1"
```

/score="-6.8423754845673805"
/phase="0"
/translation="MPKLREPKNLGDLLKYEAPNRYSRDLAPVALGQKLALGAVVAREP
DGIRLQALDPAATDASAQAVGVLIEAVDATAAEVPQALLLARHAVVSDSALVWPAGINP
AQKATAIAQLQALGILVRTGA"
CDS complement(21067..22392)
/ID="VIXMOERF_CDS_0027"
/transl_table=11
/phrog="53"
/top_hit="No_MMseqs_PHROG_hit"
/locus_tag="VIXMOERF_CDS_0027"
/function="head and packaging"
/product="head maturation protease"
/source="PHANOTATE_1.5.1"
/score="-2065.896345732396"
/phase="0"
/translation="MLPHLAGRLYGTPLLVARPKLEVILAVLGSRIGLPAADALVPPAP
MPRTAAQALPGIAVIPIHGTLVRRTQGLDAHSGLTSYAEIGVRLDAALRDPEVAGLLLD
IDSPGGESGGVFELAAKIRAGTSHKPIWAHANDTAFSAAYAIAAGASRVTLAQTGGVGS
IGVIALHVDQSVKDTREGLTYTALYAGHHKNDLNPHAPLSPQAAEALQSEVDRLYGIFI
RDVAALRRLPEAAVRATEAGLFFGEDAVSAGLADGVLGFDAVLSEFADAINARQRLISP
PSPKAAVMSPLPVSLLHSESVMHDHAPGPLNDEAAAEIHKDTDAAGNPTSPAAPSPPND
LAAATPVLGDPTAQSLATEAHHAGRSEAQAIAELCLIAGAALRTAEFLAAGLSEAQVRR
ALLAARAETPEIASRIAVDAGTSIRPETGPVVAAVQKLIARS"
CDS complement(22392..23009)
/ID="VIXMOERF_CDS_0028"
/transl_table=11
/phrog="No_PHROGs_HMM"
/top_hit="No_MMseqs_PHROG_hit"
/locus_tag="VIXMOERF_CDS_0028"
/function="unknown function"
/product="hypothetical protein"
/source="PHANOTATE_1.5.1"
/score="-26.072395144556392"
/phase="0"
/translation="MTALPRRPGDRRRQPVETGSAFEVAIEILVRPHGLEIDQAQAVIA
GVAEQVELHLAADFIDPQPGQIAFLFLTDARIAGDHLDRRIDVGRHWIVQFLVGTRETA
RLQHLPVHRRVRGTNGVACSRDSFSAIRLSAMKEAGKSGLSSAARPTLAQNSICPCTVR
NSALAAARASSIKVSSIRTETLMISSQVVRHNCSQSDRGGQG"
CDS complement(22942..24432)
/ID="VIXMOERF_CDS_0029"
/transl_table=11
/phrog="21"
/top_hit="No_MMseqs_PHROG_hit"
/locus_tag="VIXMOERF_CDS_0029"
/function="head and packaging"
/product="portal protein"
/source="PHANOTATE_1.5.1"
/score="-8288.541515146338"
/phase="0"
/translation="MGWWQRLRLGLFGGPAPTYDGTGGGRRALAWQVGNPGAVAALAYS
QDELRAKSRDQVRRNVWAAAGVEAYVANAIGTGIKPQSMAADVTVRSAIQALWAHWGDD
ADAAGLTDVYGLQALACRALVEGGEALVRLRYRRPEDGLSVGLQLQVLEPEHLPVALNR

ELPSGNVIRAGIEFDRLGRRVAYHLYRSHPEDGALAPMSGAGGLDTVRVDASEVIHLFR
PLRPGQIRGEPWLARALVKLHELDQYDDAELVRKKTAAMFAGFITRLAPEDPLMGEGPA
DAQGVALAGLEPGTLQLLEPGEDVRFSQPADVGASYAEFLRMQFRAVAAAMGITYEMLT
GDLTQVNYSSIRAGLLEFRRRCEAIQHGVIVFQLCRPVWRAWMTQAVLEGALELPGFSR
RPRDYLAVKWIPQGWQWVDPKKEFDALQTAIRAGLLSRSEAISAFGYDAEDVDREIAAD
NARADALGLVFDSDPRHDRAAAPARGSAPAAGGNRVSV"
CDS complement(24432..24635)
/ID="VIXMOERF_CDS_0030"
/transl_table=11
/phrog="2545"
/top_hit="No_MMseqs_PHROG_hit"
/locus_tag="VIXMOERF_CDS_0030"
/function="connector"
/product="head-tail adaptor Ad1"
/source="PHANOTATE_1.5.1"
/score="-3.4395491352922094"
/phase="0"
/translation="MAYSAEHLQALETALARGERRVTFQDRSVEYRSVEELKAAIREVK
RGLAAQVGPVARQLRITTRKAT"
CDS complement(24655..26622)
/ID="VIXMOERF_CDS_0031"
/transl_table=11
/phrog="15"
/top_hit="No_MMseqs_PHROG_hit"
/locus_tag="VIXMOERF_CDS_0031"
/function="head and packaging"
/product="terminase large subunit"
/source="PHANOTATE_1.5.1"
/score="-179421.2712607626"
/phase="0"
/translation="MLGDEEGLTAIARAWRDGLTPDPLLTVSKWADRHRVLSSKASAEP
GRWRTARTPYLREIMDCLSPASPVERVVFMKGAQLGATEMGSNWIGFCVHHAPGPMMAV
WPTVEMAKRNSRQRIDPLIEESPSLVELIAPARSRDSGNTILAKEFRGGVLVMTGANSA
VGLRSMPVRYLFLDEVDGYPPDVEGEGDAIALAEARTRTFARRKIFIVSTPTISGASAI
EREYEASDQRRYFVPCPHCGQFQWLRFEQLRWERGRPETVGYVCEGCERPIPEHHKGDM
LAQGEWRALAPEHGAKTAGFHLSSLYSPLGWRSWRDIAAAWDSAVHPVSGGPSAIKTFK
NTELGETWVEDGETPDWQRLLERREDYTIGTVPLGGLLLAGGADVQKDRIEVSIWAFGR
GKSAWLVEHRVLMGDTGREPVWAELASMLEERWSHAVGALLPLARLALDTGYATQEAYA
FVRRLKDPRVMAVKGVPRGAALIGTPTAVDLTQGGRKLRRGIKVFAVAVGIAKLEFYNA
LRKTAEVGEDGTTLVYPPGYIHLPRMDAEYLQQLCAEQLVTRRDRHGFARREWQKLRER
NEALDCYVYARAAAAAAGLDRYEERHWRALEEQLGVGPPPDSEPPLQPIDPPAPTDSGG
RSVSSPGRSSRRVIRSRWLA"
CDS complement(26623..27150)
/ID="VIXMOERF_CDS_0032"
/transl_table=11
/phrog="57"
/top_hit="No_MMseqs_PHROG_hit"
/locus_tag="VIXMOERF_CDS_0032"
/function="head and packaging"
/product="terminase small subunit"
/source="PHANOTATE_1.5.1"
/score="-17.758510853089497"
/phase="0"

/translation="MGLSIRAYARHRGVTDTAVHKAIRTGRITPEADGSIDPDKADREW
ARNSDAPKAGTRQPAVRVAVPDASHDGGPSLPGGGTSLLQARTVNEVVKAQTNKVRLAK
LKGDLVDRAAAIAQVFTLARAERDAWLNWPARISAPLAAELGIDPHTLHVALETAVREH
LQELGELRPRVD"
CDS 27163..27438
/ID="VIXMOERF_CDS_0033"
/transl_table=11
/phrog="2555"
/top_hit="No_MMseqs_PHROG_hit"
/locus_tag="VIXMOERF_CDS_0033"
/function="unknown function"
/product="hypothetical protein"
/source="PHANOTATE_1.5.1"
/score="-8.526201967040032"
/phase="0"
/translation="MAVDFAWLVFGTARSWRRHPRHHENYAMTPRDALLTRNAQQHLGI
DTLETQNSDHLDFHDMAVWCLKAALEAAYQAGIEAGRRTKSAAANT"
CDS 27435..27524
/ID="VIXMOERF_CDS_0034"
/transl_table=11
/phrog="No_PHROGs_HMM"
/top_hit="No_MMseqs_PHROG_hit"
/locus_tag="VIXMOERF_CDS_0034"
/function="unknown function"
/product="hypothetical protein"
/source="PHANOTATE_1.5.1"
/score="-0.0585528949150736"
/phase="0"
/translation="LNTSIARLADPVGRPLTEGRRRRCPKPKH"
CDS 27536..28045
/ID="VIXMOERF_CDS_0035"
/transl_table=11
/phrog="10315"
/top_hit="No_MMseqs_PHROG_hit"
/locus_tag="VIXMOERF_CDS_0035"
/function="unknown function"
/product="hypothetical protein"
/source="PHANOTATE_1.5.1"
/score="-119.77074160679572"
/phase="0"
/translation="MNTLSLTDTQRRILDHAIEHTAGRIDWFPDNIKGGARRKVLDGLL
KRGLIALNGGDHCVTAEGFAAVGRTPPVPISPSAETEMPASNAEAAEANATGKRIQKSR
EDSKQAEVIRMLHRPEGATIPQICAETGWQAHTVRGVFAGAFKKKLGLTLVSEKPEGGD
RIYKIA"
CDS 28156..28365
/ID="VIXMOERF_CDS_0036"
/transl_table=11
/phrog="16013"
/top_hit="No_MMseqs_PHROG_hit"
/locus_tag="VIXMOERF_CDS_0036"
/function="unknown function"
/product="hypothetical protein"
/source="PHANOTATE_1.5.1"

/score="-21.414432146988776"
/phase="0"
/translation="MSVTVKLSEHLVEQARRYAHIEHRSVPKQIEHWSMIGKIAEENPD
LPFTLIRDILIADEEEAVGEYHFG"
CDS 28365..28643
/ID="VIXMOERF_CDS_0037"
/transl_table=11
/phrog="1213"
/top_hit="No_MMseqs_PHROG_hit"
/locus_tag="VIXMOERF_CDS_0037"
/function="moron"
/function=" auxiliary metabolic gene and host takeover"
/product="RelE-like toxin"
/source="PHANOTATE_1.5.1"
/score="-19.304598008848423"
/phase="0"
/translation="MRLEVTPTFDRTAKKLNRHQKMDLDEAVRAIADDPEIGVAKVGDL
AGVRVYKFRLSNAQCLLAYRILAPDTLKLLTFGPHENFYRDLKRLDV"
CDS complement(28650..30116)
/ID="VIXMOERF_CDS_0038"
/transl_table=11
/phrog="498"
/top_hit="No_MMseqs_PHROG_hit"
/locus_tag="VIXMOERF_CDS_0038"
/function="other"
/product="DNA methyltransferase"
/source="PHANOTATE_1.5.1"
/score="-80503.53228474224"
/phase="0"
/translation="MRGFHQAFQLIGRNHGDGPTGTAADDDHLTIIDGAIHQRFELLAG
PAVGDFERHGVKHFRQNAFNVQRMRHSWVADRVESWPVDKLLPYACNARTHSDAQIAQI
AASIAEFGFTAPILAGADGVIVAGHGRWAAARQLGLSQVPVIVLDHLSPTQRRALVIAD
NRIAENAGWDESLLKLELTALQDEDFDLGLTGFDADALLDLLADEASVTEGQTEDDVAP
DVPVQPVSRPGDVWILGSHRLLCGDATLAEHYDRLLAGESVDMVFTDPPYNVNYANSAK
DKLRGKHRAILNDNLGEGFSDFLLAALTPMLAHCKGAVYIAMSSSELDTLQSAFRTAGG
HWSTFIIWAKNTFTLGRADYQHQYEPILYGWAAGAQRHWCGDRDQGDVWQINKPARNDL
HPTMKPVELVERALRNSSRPGDLVLDPFGGSGTTLIAAEKSGRVARLIELDPKYVDVIV
RRWEDWTGKQATREADGLAFDALASEATAV"
CDS complement(30284..30562)
/ID="VIXMOERF_CDS_0039"
/transl_table=11
/phrog="No_PHROGs_HMM"
/top_hit="No_MMseqs_PHROG_hit"
/locus_tag="VIXMOERF_CDS_0039"
/function="unknown function"
/product="hypothetical protein"
/source="PHANOTATE_1.5.1"
/score="-7.729487997536262"
/phase="0"
/translation="MVWYSVGAFIAFLDLLDADHLDGVQDIEQNPVIAHAQPVTTGMID
QRLDATHCREPLQRCRCSQYPLLSRLVAQLLKLLQSSRLPNDRFHGA"
CDS complement(30563..30913)
/ID="VIXMOERF_CDS_0040"

/transl_table=11
/phrog="No_PHROGs_HMM"
/top_hit="No_MMseqs_PHROG_hit"
/locus_tag="VIXMOERF_CDS_0040"
/function="unknown function"
/product="hypothetical protein"
/source="PHANOTATE_1.5.1"
/score="-2.1379765410680918"
/phase="0"
/translation="MIIACRSSFGSRYSANCSTLRPMALASLVSVALRGVLSPRSRRAN
AGCCMQARCATSSCVSPKYSRQARISGISCRTWASTTAWGMASRTLRRSSRGRTRSKSA
TGTNTKAGCPSW"
CDS complement(30910..32304)
/ID="VIXMOERF_CDS_0041"
/transl_table=11
/phrog="498"
/top_hit="No_MMseqs_PHROG_hit"
/locus_tag="VIXMOERF_CDS_0041"
/function="other"
/product="DNA methyltransferase"
/source="PHANOTATE_1.5.1"
/score="-45134.041736163235"
/phase="0"
/translation="MLQVEYRPLEKLIPYARNPRTHSVDQVAKIAASIVEFGWTNPILV
DGAQGIIAGHGRLAAARSLGLAEVPVIELGHLSPAQKRAYVLADNRLALDAGWDEELLA
LELAELSGAGFDLALTGFNDDELEALLSIDTEDSDDAEDGEPETADDVPEPPATPVSRP
GDVWQLGRHRLICGDASDPDVVAALMGGELARLCFTSPPYDNQRDYTSGGIGDWDGLMR
GVFAQLPLTDDAQVLVNLGLIHRDNEVIPYWDPWLGWMRTQGWRRFGWYVWDQGPGMPG
DWAGRLAPSFEFVFHFNRQSRKPNKTVACKFAGQETHLRQDGSSTALRGKDGEVGGWTH
AGQPTQDRRIPDSVIRIMRHKGKIGRDIDHPAVFPVALPEFVLTAYSDPDDPVYEPFGG
SGTSLLAAERTGRRGYAVEIAPGYVDVAILRFRQSFPAIPVTLLDTGETWEAVASQRQA
IQTTAG"
CDS complement(32268..32591)
/ID="VIXMOERF_CDS_0042"
/transl_table=11
/phrog="No_PHROGs_HMM"
/top_hit="No_MMseqs_PHROG_hit"
/locus_tag="VIXMOERF_CDS_0042"
/function="unknown function"
/product="hypothetical protein"
/source="PHANOTATE_1.5.1"
/score="-0.2668303687412284"
/phase="0"
/translation="LHPQPERTTLEAAPVLALTRERPTPPVQTWVQTPHCKPGAAIEVC
TLGAYLISQDPPRFAVGGFFVAALPRVYGSLQVPIGPCAGPYFFGIATNPSCCRSNTVR
WKS"
CDS complement(32722..32832)
/ID="VIXMOERF_CDS_0043"
/transl_table=11
/phrog="No_PHROGs_HMM"
/top_hit="No_MMseqs_PHROG_hit"
/locus_tag="VIXMOERF_CDS_0043"
/function="unknown function"

/product="hypothetical protein"
/source="PHANOTATE_1.5.1"
/score="-0.126568158164322"
/phase="0"
/translation="LKVIGERCGRLRKQQDFGLSHLGVGRRRVALKVQQV"
CDS complement(32910..33233)
/ID="VIXMOERF_CDS_0044"
/transl_table=11
/phrog="2104"
/top_hit="No_MMseqs_PHROG_hit"
/locus_tag="VIXMOERF_CDS_0044"
/function="unknown function"
/product="hypothetical protein"
/source="PHANOTATE_1.5.1"
/score="-54.29122138023074"
/phase="0"
/translation="MTQTAKANSRILEAVHETADDLHRLGFIDKCKMREFDVLCLRPVP
VYDSSSIRALRERCQISQAVLANLLNTSLSTVQKWEIGDKKPSGPSLKLLNLIDRKGLE
LLL"
CDS complement(33347..33697)
/ID="VIXMOERF_CDS_0045"
/transl_table=11
/phrog="11214"
/top_hit="No_MMseqs_PHROG_hit"
/locus_tag="VIXMOERF_CDS_0045"
/function="unknown function"
/product="hypothetical protein"
/source="PHANOTATE_1.5.1"
/score="-114.8540332785114"
/phase="0"
/translation="MKTIVIGIMPQEDIRKRLLAIARGELKPKAGDPKIWFTSMRSLAE
VLSDENRALLKVIRETKPESITSLAAATGRKPGNLSRTLKTMSHYGLVEMKREKNHVRP
IAKGTEFRIVAA"
CDS complement(33694..34065)
/ID="VIXMOERF_CDS_0046"
/transl_table=11
/phrog="No_PHROGs_HMM"
/top_hit="No_MMseqs_PHROG_hit"
/locus_tag="VIXMOERF_CDS_0046"
/function="unknown function"
/product="hypothetical protein"
/source="PHANOTATE_1.5.1"
/score="-87.82788516043458"
/phase="0"
/translation="MCMEVDPGIETLLDLQDQIIDQGSGYWVKIEAYQVAPTPDVPHGI
RYSLTLHEPYGKRILGYDNAHAVKPPKKFKYAGRRLTFDHMHRHARDPGVPYEFKDAHQ
LLADFFSEVDQVLLEVKKR"
CDS complement(34107..34478)
/ID="VIXMOERF_CDS_0047"
/transl_table=11
/phrog="6836"
/top_hit="No_MMseqs_PHROG_hit"
/locus_tag="VIXMOERF_CDS_0047"

/function="unknown function"
/product="hypothetical protein"
/source="PHANOTATE_1.5.1"
/score="-2.12692996871658"
/phase="0"
/translation="VAEWTIERVAARFEEAAWTAQDLPPVKVQGYFNCWPAIARQAWEG
YADETRECRVQPSPDAVDRMLETMRWVLWLEEDQRHLIWMRAERRGWKDIARRFACCTR
TAQRRWQQALRQVADRLNG"
CDS complement(34471..34680)
/ID="VIXMOERF_CDS_0048"
/transl_table=11
/phrog="19772"
/top_hit="No_MMseqs_PHROG_hit"
/locus_tag="VIXMOERF_CDS_0048"
/function="unknown function"
/product="hypothetical protein"
/source="PHANOTATE_1.5.1"
/score="-4.392430589844607"
/phase="0"
/translation="MRGPNPNPRCPLGKLQPQVTDLEAVKREGWREQRILVVHLIDERL
DFVERELIRQIGERLYGRRESRRG"
CDS complement(34677..35456)
/ID="VIXMOERF_CDS_0049"
/transl_table=11
/phrog="159"
/top_hit="No_MMseqs_PHROG_hit"
/locus_tag="VIXMOERF_CDS_0049"
/function="DNA"
/function=" RNA and nucleotide metabolism"
/product="RuvC-like Holliday junction resolvase"
/source="PHANOTATE_1.5.1"
/score="-307.3199677069585"
/phase="0"
/translation="MTTTLIPDGRRSAICPLKSAIWIQMANVRHLRIDILQSLTVQMAE
FPDGSAANSPSGATDCLYETIYGIPDGGDSLPLRGRGALRALSPSRRGLSGLGDNDEGD
RTTLLALDLGTHTGWALHQRDGTVISGSETFKPQRFEGGGMRFLRFKRWLTEIKQAAGD
LDAVYFEEVRRHAGVDAAHAYGGFLAHLTAWCEHHGVPYQGVPVGTIKKHATGQGNANK
AAMIAAMRSLGFDPVDDNEADALALLHWARMTQGVGV"
CDS complement(35453..37291)
/ID="VIXMOERF_CDS_0050"
/transl_table=11
/phrog="13004"
/top_hit="No_MMseqs_PHROG_hit"
/locus_tag="VIXMOERF_CDS_0050"
/function="unknown function"
/product="hypothetical protein"
/source="PHANOTATE_1.5.1"
/score="-748782.5332021726"
/phase="0"
/translation="MALAGQLLGTARVEPTPLREKAPVDRLGPATAKWDYVAADGTLIA
CVYRYDPPTGKEFRPWDVRARLWRAPDPRPLYNLPAIAQAKEVVLVEGERCADALVHQG
VAATTAMNGAKAPIDKTDWSPLRGKAVLIWPDRDAPGWDYAENAARACMAAGAASVAIL
VPPTDKPDKWDAADAVSEDFDCVAFITHGERRVIKAASPALPTHTLGALLDDDSPVPPD

LIAPRVLTPGGLLVFGGAPKVGKSDFLLAWLMHMAAGASFLGMTPPRPLRVFYLQAEVQ
YHYLRERVKGIPLPSHRISEARVNFVATPQLRLVLDEAGLAQVIPAIVQAFGNTPPDLI
AIDPIRNVFDGGEAGGENDNGAMLFFLSQRVEQLRTAVNPDAGLVLVHHTKKLGKKPFE
EDPFQALAGAGSLRGYYSTGMLLFRPDETQTTRQLIFELRNGAALPVKSVDKFNGTWRE
VANTGRRVLQDYAERLDAERRRKHDVILQILFDEAADGRCYSATQFAEAFEGRAGLGAE
RTIRERLSVLATQGYIKFFRNAEDYGLPPLHRSKFGYQCVEGMVLRREGPPDPDTGEIP
MWPVSVYPSHYKCPQTGALLPVEDATIWLYHDEDHP"
CDS complement(37339..37464)
/ID="VIXMOERF_CDS_0051"
/transl_table=11
/phrog="No_PHROGs_HMM"
/top_hit="No_MMseqs_PHROG_hit"
/locus_tag="VIXMOERF_CDS_0051"
/function="unknown function"
/product="hypothetical protein"
/source="PHANOTATE_1.5.1"
/score="-0.1260100213451203"
/phase="0"
/translation="VPSRSATSRAIRATAWKWRFRAKRPGCGTTMRPARAGISSP"
CDS complement(37599..37958)
/ID="VIXMOERF_CDS_0052"
/transl_table=11
/phrog="No_PHROGs_HMM"
/top_hit="No_MMseqs_PHROG_hit"
/locus_tag="VIXMOERF_CDS_0052"
/function="unknown function"
/product="hypothetical protein"
/source="PHANOTATE_1.5.1"
/score="-26.024403446568186"
/phase="0"
/translation="MKTRQPSDDKRVQRQPPRPLVRLTVIDKLLRRHIVFACPEAHLAV
AVITLAIGDCIDPDDALRAEARYFLAGPALEFWCDAVGLEPAFVRAIAHKGGYLPSETA
QGVGVKHTPKELGLA"
CDS complement(37955..38692)
/ID="VIXMOERF_CDS_0053"
/transl_table=11
/phrog="1990"
/top_hit="No_MMseqs_PHROG_hit"
/locus_tag="VIXMOERF_CDS_0053"
/function="DNA"
/function=" RNA and nucleotide metabolism"
/product="Cas4-domain exonuclease"
/source="PHANOTATE_1.5.1"
/score="-134.94562750729017"
/phase="0"
/translation="MLDYNHGALFNERLTALIDAGLQRRQAGEPRRTYLGASRLGVACE
RALQYEYAQAPVDPGREFSGRLLRIFERGHRIEEAVVGWLRAAGFGLCPHQADGKQFGF
SALNGHLQGHVDGVIVGGPERFDTPALWENKCLGAKSWRELVKHRLAKAKPVYAAQIAV
YQAYLALHEQPALFTAVNADSMEIYAERVPFDGGLAQRMSDRAVRVITATEAGELLPRG
FADPAHVECRQCAWSLRCRGGGA"
CDS complement(38703..39068)
/ID="VIXMOERF_CDS_0054"
/transl_table=11

/phrog="21342"
/top_hit="No_MMseqs_PHROG_hit"
/locus_tag="VIXMOERF_CDS_0054"
/function="unknown function"
/product="hypothetical protein"
/source="PHANOTATE_1.5.1"
/score="-13.725581021107352"
/phase="0"
/translation="MHAMICAICGREGRGFCWASSHDAPRDSGGKRLFKRFCSRRCQDI
HLQRLKRRDGVVIDPTHNEKAAMEAVLPRLGDYVAAIGMDRPLSAYSRAEILQLVDVVL
TAYFDHLREHDPDDVPF"
CDS complement(39056..39181)
/ID="VIXMOERF_CDS_0055"
/transl_table=11
/phrog="No_PHROGs_HMM"
/top_hit="No_MMseqs_PHROG_hit"
/locus_tag="VIXMOERF_CDS_0055"
/function="unknown function"
/product="hypothetical protein"
/source="PHANOTATE_1.5.1"
/score="-1.5060259715700814"
/phase="0"
/translation="MGHIPVAHPPGTPSVVQIRSRRICAQPRSGCPKGGVQGCTP"
CDS complement(39205..40890)
/ID="VIXMOERF_CDS_0056"
/transl_table=11
/phrog="16"
/top_hit="No_MMseqs_PHROG_hit"
/locus_tag="VIXMOERF_CDS_0056"
/function="DNA"
/function=" RNA and nucleotide metabolism"
/product="DNA helicase"
/source="PHANOTATE_1.5.1"
/score="-1176584.4066044665"
/phase="0"
/translation="MMLRPRQTLLVERSLAALHQHGNTLAIGPTGSGKTIMLSAVAGGV
LEEPDAKACILAHRDELTAQNREKFGRVNPGLTTSVFDALEKSWAGAATFAMVQTLARG
RHLEQMPTLDLLVIDEAHHAASPSYRAVIDRVRSRNPKALLCGLTATPNRGDGKGLREV
FSNVADQITLGELIASGHLVPPRTFVIDIGVQAALQQVRRTAIDFDMDEVASIFDKQLV
TDAVIQHWQDKASGRKSIVFCSTVAHARHVYEAFIAAGIHAVLIHGDLSDAERKARLAD
YESGPAQVVVNVAVLTEGYDYPPTGCVVLLRPSSHQSTFIQMVGRGLRTVDPEVFPGAI
KTDCIVLDFGTASLMHGTLEQTVDLDGHLGQGEAPTKDCPECGAIVPAACLACPLCGHL
WERQPKDRGVLSDFVMSEIDLLKRSHFRWCDLFGSDDALMAAGFSAWGGVFFLTGRWYA
VGGGKGLPTHLLGVGERTVCMAKADDWLNEHESVDTAHKTRRWLNEPPTEKQLRYLPPQ
WQSDFGLSRYQASALLSFQFNKRAIQSLVLGADAAAHDTQRRAA"
CDS complement(40905..41531)
/ID="VIXMOERF_CDS_0057"
/transl_table=11
/phrog="2300"
/top_hit="No_MMseqs_PHROG_hit"
/locus_tag="VIXMOERF_CDS_0057"
/function="unknown function"
/product="hypothetical protein"

/source="PHANOTATE_1.5.1"
/score="-76.93704443628954"
/phase="0"
/translation="MSYFDFNSAEQASFELIPKDTLVRLRLTLKPGGFDDASQGWTGGW
ATRSPETGAVYLACEGVVLDGPYARRKLWWNIGLHSPKGPTWQAMGRSFIRSLLNSARR
IHPADTGPQAQNARRIAGFAELDGLEFAGRIDIEKDGRGNDRNTVRAVIEPDHKDYAQL
MGQHFAPPVPTSPSGAIPAAAAPAYAASTPATSPGVPGGKPAWAQ"

CDS complement(41534..42397)
/ID="VIXMOERF_CDS_0058"
/transl_table=11
/phrog="124"
/top_hit="No_MMseqs_PHROG_hit"
/locus_tag="VIXMOERF_CDS_0058"
/function="DNA"
/function=" RNA and nucleotide metabolism"
/product="Sak4-like ssDNA annealing protein"
/source="PHANOTATE_1.5.1"
/score="-753.0021290723366"
/phase="0"
/translation="MTLPIISADQRLAERRGVKGVLVGKAGLGKTSQLWTLEAASTLFF
DLEAGDLAVEGWAGDTIRPRTWPECRDFAVFIGGPNPALRDDQPFSPAHFDAVCARFGD
PGALERYQTVFVDSITVAGRLCLQWCKGQPQAYSEKTGKPDSRGAYGLMGQEMIGWLTH
LQHTRNKNVWFVGILDEKLDDFNRRVFALQIDGAKTGLELPGIVDEVVTLAELPADDGS
RYRAFVCQTLNPWGYPAKDRSGRLDPIEPPHLGQLMQKIAGPARPPLERLDFSRPVPAT
IPTQET"

CDS complement(42394..42873)
/ID="VIXMOERF_CDS_0059"
/transl_table=11
/phrog="2480"
/top_hit="No_MMseqs_PHROG_hit"
/locus_tag="VIXMOERF_CDS_0059"
/function="unknown function"
/product="hypothetical protein"
/source="PHANOTATE_1.5.1"
/score="-36.17274846516688"
/phase="0"
/translation="MTSMNPDILLATPAGQLAELPAESLCRFKHDAAAQLAAAKTLNEH
LDRALEIRYAERARQLRLATGKDTGIVHFDDGPVRVTADLPKKVEWDARQLAALVRRIA
DSGEDPAQYVEIGYRVSETKFNAWPAGLQQSFAPARTLRTGKPGFRLALLGEDAV"

CDS complement(42870..43187)
/ID="VIXMOERF_CDS_0060"
/transl_table=11
/phrog="66"
/top_hit="No_MMseqs_PHROG_hit"
/locus_tag="VIXMOERF_CDS_0060"
/function="integration and excision"
/product="excisionase and transcriptional regulator"
/source="PHANOTATE_1.5.1"
/score="-0.3762021565367028"
/phase="0"
/translation="LQVLPTPAARFAPRFAHPLKPRICSRFRNNPQERLVNVRHLNQRQ
LADRWGVSEATLERWRSEGIGPVYLKLHGRVLYRQEDIEAYETRHLRRSPGQCVQAGGA
A"

CDS complement(43269..43490)
/ID="VIXMOERF_CDS_0061"
/transl_table=11
/phrog="3220"
/top_hit="No_MMseqs_PHROG_hit"
/locus_tag="VIXMOERF_CDS_0061"
/function="unknown function"
/product="hypothetical protein"
/source="PHANOTATE_1.5.1"
/score="-8.09683952766589"
/phase="0"
/translation="MSKNQWVVKNGDGWGVRGEGNSRLTSKHETQHEAIERARDIARNQ
SSEVIIQGEDGKIRERNSYGNDPFPPPG"
CDS complement(43557..44516)
/ID="VIXMOERF_CDS_0062"
/transl_table=11
/phrog="No_PHROGs_HMM"
/top_hit="No_MMseqs_PHROG_hit"
/locus_tag="VIXMOERF_CDS_0062"
/function="unknown function"
/product="hypothetical protein"
/source="PHANOTATE_1.5.1"
/score="-3605.22021872557"
/phase="0"
/translation="MPACNDIAFGLLCELIEAPHADIHPDTVRCSGRREAYEHLHELQA
LQVGTGLAGSVLCPWCGDDELCSLSFSEEGYRGYCSDCGWLNLATHQVKPLRVEYQRIV
RWIASALGLQGRFRLEERVPARLWRLGDIEHRRKRRTVFFGRRLNESSDAQTIDAGIRA
VAAPGTEILITTTPQDSIAPPLANGRLVPLRAVSHLRKAGFVVENLESYLDAPLIADED
VAETSLRLLRSGRLALIDGKQIKVSPQIYRFLSILIDADGKPVHKRVLADALEMDVDAC
KGSEIFKRHKAVYRTFIEHDTEGRYWLKPEFLSRRGGE"
CDS complement(44524..45555)
/ID="VIXMOERF_CDS_0063"
/transl_table=11
/phrog="37634"
/top_hit="No_MMseqs_PHROG_hit"
/locus_tag="VIXMOERF_CDS_0063"
/function="unknown function"
/product="hypothetical protein"
/source="PHANOTATE_1.5.1"
/score="-16853.51611994235"
/phase="0"
/translation="MRRVHRLAQRKGIYALLNAGHGQSEIQEGFEQLHNHAERALWTWV
HWPAVFQIGERLLQFDLSAGTRAWKRQAIKVSEPVSKEEENIRALETALSTLMSRRKGP
RRACRIDVCERCLDGGVQVNIYIEDDPNDLVEFVEEGMKRRTTRPAGNLALVYYPISGI
VDSVGRGGARIHVALVTLFAKHLLNRDVKPEAVKQPMFHLNRLRFGLAFVEGDAIDLAA
HGVDRIRLRQVRVRATIPPHCDFWVETPADPNEACAFTASSTHLQERDFFRGPFNLVEV
VISVYFVPNEPGKIGHVLNIVLKQSGVSNLRDMSENDAQLADALLRAWQVTEPSEFEAK
LAA"
CDS 45493..45828
/ID="VIXMOERF_CDS_0064"
/transl_table=11
/phrog="No_PHROGs_HMM"
/top_hit="No_MMseqs_PHROG_hit"

/locus_tag="VIXMOERF_CDS_0064"
/function="unknown function"
/product="hypothetical protein"
/source="PHANOTATE_1.5.1"
/score="-23.90508308894612"
/phase="0"
/translation="MPCVEQRVDAFALGQSVNPAHFRVDRRALFTVLETLEAGNQGICQ
RGFFRLPIEIVTDLDRPLCQIVAPGFSGDLPDEVTKIECSQDDFSCTEEKTRNERLEPR
TLTKWDG"
CDS 45937..46290
/ID="VIXMOERF_CDS_0065"
/transl_table=11
/phrog="8"
/top_hit="No_MMseqs_PHROG_hit"
/locus_tag="VIXMOERF_CDS_0065"
/function="transcription regulation"
/product="transcriptional regulator"
/source="PHANOTATE_1.5.1"
/score="-102.1014426850792"
/phase="0"
/translation="MNRPDERRNTVPSPLGEKIRGLRKQKKLSLDQLADLTESSKSYLW
ELENKEAPNPSAEKIARIAAVLEVTTEFLMNDQEMTPDAAVADEAFFRKYKKMPEETKK
KLRQLIDVWDDDP"
CDS 46287..47198
/ID="VIXMOERF_CDS_0066"
/transl_table=11
/phrog="1914"
/top_hit="No_MMseqs_PHROG_hit"
/locus_tag="VIXMOERF_CDS_0066"
/function="unknown function"
/product="hypothetical protein"
/source="PHANOTATE_1.5.1"
/score="-10414.70101028641"
/phase="0"
/translation="MTERKWPTAEANRLNVMLGQVLGRERFPVDVEALALEYSKQCFPH
APITQIKGADLPGFEGMLAAHPSKTQWKIVYNSAVRSRGRIRFTLAHEFGHYLLHRDRQ
EIFSCSQQDMEEWDAEERQLETEADTFASYLLMPLDDFRQQIGKERVSFELLGHCAERY
GVSLTAAALKWIEIAENRAVLVAVRDDHLLWARSNQAAFKSGAVFATRKRTYAVPPESL
VHGRNGDIPIQAGSLSANTWFPKEPRDMPLTELIFVNEHYDYTLALLLMPKAELRWQGE
EDEDDDPAERMDSAIRQGRFRR"
CDS 47225..47326
/ID="VIXMOERF_CDS_0067"
/transl_table=11
/phrog="No_PHROGs_HMM"
/top_hit="No_MMseqs_PHROG_hit"
/locus_tag="VIXMOERF_CDS_0067"
/function="unknown function"
/product="hypothetical protein"
/source="PHANOTATE_1.5.1"
/score="-0.1835130171846476"
/phase="0"
/translation="VSKPGLPGRRKHPPKRFPSGNIRPVFAMACNDP"
CDS 47416..47607

/ID="VIXMOERF_CDS_0068"
/transl_table=11
/phrog="No_PHROGs_HMM"
/top_hit="No_MMseqs_PHROG_hit"
/locus_tag="VIXMOERF_CDS_0068"
/function="unknown function"
/product="hypothetical protein"
/source="PHANOTATE_1.5.1"
/score="-3.755002470777208"
/phase="0"
/translation="MTMPSLTPPERLSSAQRVHEITTILAAAILRTYLIEPEKKTGLDL
GLLAGKRVHTTPSQPERV"
CDS 47607..48059
/ID="VIXMOERF_CDS_0069"
/transl_table=11
/phrog="11806"
/top_hit="No_MMseqs_PHROG_hit"
/locus_tag="VIXMOERF_CDS_0069"
/function="unknown function"
/product="hypothetical protein"
/source="PHANOTATE_1.5.1"
/score="-25.58009757910637"
/phase="0"
/translation="MNDLKLSVAAQVASLPTLPIKDLWTLWDQYFPRRPTHPNRNYLES
RIAYKIQEAAYGGLAPETRRRLEQVGQRHSKIKSRRVSPAIHLPPGTVLVREWGEQDHK
VTVTAEGRFDYAGQSFKSLTAVARHITGTAWSGPLFFGLRQAGEGT"
CDS 48056..49453
/ID="VIXMOERF_CDS_0070"
/transl_table=11
/phrog="95"
/top_hit="No_MMseqs_PHROG_hit"
/locus_tag="VIXMOERF_CDS_0070"
/function="integration and excision"
/product="integrase"
/source="PHANOTATE_1.5.1"
/score="-149852.45900604993"
/phase="0"
/translation="MRASNRMEVQTGAPSKPRQRCAVYCRVSSDERLDQEFNSIDAQKE
AGHAYIASQRIEGWIPVADDYDDPGYSGGNTERPALRRLLADIEAGRIDIVVVYKIDRL
TRSLADFSRMVEVFERQGVSFVSVTQQFNTTTSMGRLMLNVLLSFAQFEREVTGERIRD
KIAAAKRKGLWMGGVPPLGYDVANRQLVVNDREAALVKRIFQDMLSVGSTTRIAAALNA
EGITTKAWITQDGRHRPGARIDKKHLHHLLRNRIYLGEISHKGSWHSGAHPAIIEATLW
DAVHAVLARDARSRATETRQRERTDALLRGLLYDAEGEKMYPTYVRKNGRQYRYYFSKA
EARFGAGHKTSVRLPAEEIEAATLAQIRTVLASPEAIAAIWQAVQAQTADLDEAQVVVA
LGQLGAVWEQLFPAERHRIVQLMIERVELADGGLRIRWRALGWKELLSEFAPRTIGAEL
VEMETAA"
CDS 49363..49815
/ID="VIXMOERF_CDS_0071"
/transl_table=11
/phrog="14161"
/top_hit="No_MMseqs_PHROG_hit"
/locus_tag="VIXMOERF_CDS_0071"
/function="other"

/product="recombinase"
/source="PHANOTATE_1.5.1"
/score="-27.541166272978323"
/phase="0"
/translation="MAGFGLERTAQRIRAQDDRGRVGRDGDGSMKTDAWESFVPLTFRR
RGVQRLVATAAPAHDLPFLVGLGRALYWQHLLDTGVVHSGSDIARQEDLHPSTVNELLR
LTLLAPDLIERLMAGRQPRPLTLMWFQRHPLPVDWAEQRDLMVSFE"
CDS 49824..50288
/ID="VIXMOERF_CDS_0072"
/transl_table=11
/phrog="14161"
/top_hit="No_MMseqs_PHROG_hit"
/locus_tag="VIXMOERF_CDS_0072"
/function="other"
/product="recombinase"
/source="PHANOTATE_1.5.1"
/score="-125.50650371506389"
/phase="0"
/translation="MAKKDRGIIIGKPRTVSLPQPAGGVQLETFLPWTLVKRGLKREIL
TPLGTPAAFQEEAKREVEKRRGEQDTPLIRALGLAHYWQRLLDEGKFESLSELAAAEGI
DIAQVSRIARLVRLAPGIVEACVAEMAPVLTLEDLNRRAKSVHWDVRQRV"
CDS complement(50285..50398)
/ID="VIXMOERF_CDS_0073"
/transl_table=11
/phrog="No_PHROGs_HMM"
/top_hit="No_MMseqs_PHROG_hit"
/locus_tag="VIXMOERF_CDS_0073"
/function="unknown function"
/product="hypothetical protein"
/source="PHANOTATE_1.5.1"
/score="-2.3290441092012366"
/phase="0"
/translation="MRVFWAAAAEQDRADIIDYISQDNPLAAVTVAPCQAI"
CDS complement(50395..50766)
/ID="VIXMOERF_CDS_0074"
/transl_table=11
/phrog="28287"
/top_hit="No_MMseqs_PHROG_hit"
/locus_tag="VIXMOERF_CDS_0074"
/function="unknown function"
/product="hypothetical protein"
/source="PHANOTATE_1.5.1"
/score="-14.136454560807595"
/phase="0"
/translation="MGHFRDKQTYLQRIFFITTSSAGGSAMSKEAIFTMKLEPELRAEF
MAEAKASHRPASQILRELMREFVQRQREAREYDEFLRGKVADAREQIRAGECASADDVE
ARFAARRAQLLAKAGRAGE"
CDS complement(50794..51114)
/ID="VIXMOERF_CDS_0075"
/transl_table=11
/phrog="No_PHROGs_HMM"
/top_hit="No_MMseqs_PHROG_hit"
/locus_tag="VIXMOERF_CDS_0075"

/function="unknown function"
/product="hypothetical protein"
/source="PHANOTATE_1.5.1"
/score="-7.842343120028602"
/phase="0"
/translation="MSRSRRKTPIFGHTTARSEADDKRLWHKRWRSRERDQLASLGPDG
DPLPVHRQAVSSTWDMAKDGKHWFDPRRQREMAERIATRRSPLKPECKALQARLLAKWR
AK"
CDS complement(51111..51491)
/ID="VIXMOERF_CDS_0076"
/transl_table=11
/phrog="No_PHROGs_HMM"
/top_hit="No_MMseqs_PHROG_hit"
/locus_tag="VIXMOERF_CDS_0076"
/function="unknown function"
/product="hypothetical protein"
/source="PHANOTATE_1.5.1"
/score="-25.494317337042958"
/phase="0"
/translation="MLNRLPSAGKLLRMNRRQRKKLRVGEFQERVFEVRMRFHNPMDDA
AHDDFLDGFIALIESRHLAVGGLGGQLPLMETDGIVSAWGRGSPTEEDRQAVLDWLRRH
PRVAGAEAGDFMDGWYGWDDAP"
CDS complement(51485..52297)
/ID="VIXMOERF_CDS_0077"
/transl_table=11
/phrog="14344"
/top_hit="No_MMseqs_PHROG_hit"
/locus_tag="VIXMOERF_CDS_0077"
/function="DNA"
/function=" RNA and nucleotide metabolism"
/product="nucleotidyltransferase"
/source="PHANOTATE_1.5.1"
/score="-2109.174710216461"
/phase="0"
/translation="MVDSHPIAPALRADIQARLSAIETEHGVRVLYACESGSRGWGFAS
PDSDYDVRFIYVHSLPWYLQVSAQRDVIGVPISDELDINGWELRKALGLLKKGNATLIE
WLDSPVVYRAETDFLHAMREAARQTHQAERSFHHYVHMARKNYREYLRGDTVRLKKYLY
VLRPLLATLWIEQGRGAAPMRFQDLVDAIVTDPALREAIAQLLAIKRAALESEYGQPLP
IINAFIDDELTRLESVLPPLPRDTDFSILDRLLLDTVLQPEARRTPEC"
CDS complement(52303..52455)
/ID="VIXMOERF_CDS_0078"
/transl_table=11
/phrog="No_PHROGs_HMM"
/top_hit="No_MMseqs_PHROG_hit"
/locus_tag="VIXMOERF_CDS_0078"
/function="unknown function"
/product="hypothetical protein"
/source="PHANOTATE_1.5.1"
/score="-3.0069397387905816"
/phase="0"
/translation="MTDTFSVAALQAENARLIALLLLRPRLFVRRTYRKPQPACVGLYA
QFGPD"
ORIGIN

1 gtcggacaac ggcagatcgg ccaaatctat gggatgggcg accaatatgg caagaaagag
61 aatctccaaa agcacactct cgatgttcct tcgcacgaag tgcgaccgcg agctttatct
121 gtcgctacac gaggactcgg agctggacgc taatggcatg ccggttcctc tacaggcacg
181 gcctggcatt ggagttctgc aaaccgcagg ccgcgatttt gaggatgagc ggaatgacca
241 attgatccag tctttcggca acctggtcat gtatcagccc gacaaaggag gggcgaacaa
301 gccggtcaag gcaccgcttg cctcattgct cggcaaggtc acggctttgc cttcgatcat
361 cttgcaaggc aagtttgagc catcggcgtt ccaaaatgcc gtcatggcga acattggtct
421 ccaaccagcc caagttgccc aggttccaag gatcgcagga cttatccctg acatcattgt
481 ggtgcgccag gcgacagtcg acgacgaaga ggtcggcgca gatggatgcc ggagaccaat
541 cgatccaacg accgaaactc gtcgtgcgct gagcattatt gatgtcaagc acaccagcga
601 agccaatccc agctactctg ctgaggttgc gctttacgcg atttttctcg ccaattggat
661 cgtcgaccaa ggcctacaga acagctactt tgtgaccatc cgcagctact tgtggacccg
721 attcaagcaa ggccaatctg cgctaggcgc tttgatgtcg ggctcctctc cggcaacgcc
781 cgaccaatat ctcgacgtgc tgatcgccga cagcgaggat gccaaccttc gtttctacct
841 acctaccgtg ctgcatttct tcagggaaga cttgctccgg gtcatcacca ttggtgacgc
901 ttcagccaat ggctgggaga atctcgaatg gcacgtcgat ggtcgctgta gcgcttgtga
961 ctggctcggg cacgagaagt gggcaaattc gaaggacaag gcgcgaatcg cggcacagcc
1021 agcccattac tgctatccgg cggcgaagct gactgggcac ttgagtcaga ttgctggcat
1081 gactcggggg gcaaggaaaa cgctccagat caatgccatt cagaacacag caggcgcagc
1141 gagtgctcct tcggcgcatc ctgcgtttca gcagcacagc catctcaaga aggaacgaag
1201 ccgtatcccc gttcgtgccc aagctctgat ctcatcgacg accagtgtgg acagttcagc
1261 ggtgctggca agcctggctc cctggccgca attgcatgtc gccataacag ttaacttcga
1321 tcccagcgct ggactgctaa caggcttatc gatatttggc agggcaacag cttacgtaag
1381 cggacagact ccgcgacagt tcgccaccaa gtgttttgtc gtcgaccaga aaagcctcag
1441 cgatgagtgg gtcgcccttg aaggactgct atcgaccctg tccgacatgg ttgaccagtc
1501 tgaagcgttt gtccgcgcag cgggcaagac acctctcacc gcgcaaatcg cgttctggga
1561 aaagcgccag ttcgaggagc tgtgtgcggc aatggggcgg cacctaccca aggtgttgag
1621 tcttaccaac cgcaagacca aggctctggc atggctattc cctgccgatg aactcattga
1681 gaagcctgac ggtgcggtaa gcccgtgtgt cgtcttcgta gacgagattg tccagcgcgt
1741 cgtcttcgca ccgacacctc acgtcatcac gctatttgac accgccgaag cctactattc
1801 cggatctggg ccgattcggc agggagacgc gttctatcga gagtttctga ccaacggtat
1861 tccacgagag cgcatctacg agatctggag caatgtgacc acgattaagc gtggctcggt
1921 tacagtccct cgaaacactg ttattcagga atttggtaat gcgctggaaa agcagtgtcg
1981 cgcgttgagc agtgtcgttg agaagttgcg cacggacttc agagggcaac tcaaggcaaa
2041 cgctcccaag ctaaccctct cgattccgca gggtgcccgg gatgttgctt tcgacagcaa
2101 gctttggatc tggtgggagg agctgcagta ccacaccaga aagctagagt cgcaccagcg
2161 cttggcgctc gatgccgaag cattggaagc cagctatgag gcagttcgac tcaccaacgg
2221 ccaaccgacc ggaattccga ccgaatacaa tttcgacgtg ttggcggggt caacagaagc
2281 gaagcttgat gataacgaag ggtaccttgc tctgggaaaa gaagctcatc ccggacttcc
2341 tctcctgcgc gcaaaggaca ttgtcgccgc cggttcgcct ccatattcgg gatcggatca
2401 gacgatcact acgccattgt ggtcgtcgct gtcggtgacc ctcgtgtcgt tcgatcgcag
2461 cacgcgtaag gccgtattga atctgtctaa ctggcgtgaa gccgcatttt tcccgtacct
2521 gcgggacaat tcgaccatcg acctcctgaa cgatatcttc atcactaaag gtcaaggatc
2581 attcaaatgg tacgagacgg ccaagaatat tctgactact gtcggcaacc cttcgctcgc
2641 ggtcgctgat agcaatgcgg ccactgcgat gggggcgagg ccgccgcgac ccggcacaga
2701 cccagttacc cctcttgccc gagtgctctg ggaggccgac attcttcacg caacgtccgt
2761 cgtggctgca acgccagctg catcggttgc tgcctacgcc aaggccaagc acaacctgaa
2821 cagtagtcag accgatgctg tcgcccacgc gacggaaaag cagctgacga tcatctgggg
2881 tcctccaggc actggaaaga cccagaccct tgccggatgc attcatggtc ttgtccatga
2941 tgccgcgccg aaccggcagc cattgaagct actggtcgcc gggccaacgt acaaagctgt
3001 tgaagagatt attggtcgcg ttgttgatgc gctggataac gacgcaacat gccctgccga
3061 ggtatatgtt ggatattcct cgtcgcaagc tccaaaggca tttccagcgg gtagctcgca
3121 tctacgcgtc gagtcattca agctcgaccg gaccaatcag gaaacacagg actgtcttgc

3181 cagtctggca aaggccgatg ctgtcacgat tgtcggcact gcgatgatgc aggcctacaa
3241 gctcgcggag tggacgtgtg gtagcagtgt cgggccgatt ttcgacgttg tcatcatcga
3301 cgaaagctct caggttcagg taaccgcagc aatctcagca ttggccactc tgaaagagga
3361 cgcacgattg atcattgccg gcgaccacct tcagatgccg ccaatcatgg cgcttgagcc
3421 acccaacggt gccgaatact tggtgggcag catccagaaa tacctgctcg accgtccgtt
3481 tggcagccaa atcgtttcat gtccgctcga agagaattac cgttcggcgg aggacatagt
3541 ggcctatgct cgtacgatcg gctaccgagc gacgttgaag gcgtcaaacg ccgcgacggc
3601 acttcatctc ttggcggcag tacctacttc cgcatccggc tttccaggcg gcatgccttg
3661 gtcgactttg tggccgcaaa ttctggaccc caagaagaag gtgctgacgt tgctccatga
3721 cgatgacctg tcgtcgcaaa gcaattcctt tgaagcaaag atcgtcgccg ccttgacctg
3781 gtgccttagg cagacagtga gcgcggaact tgatgggcga ggtgccgtga cgcatgcggc
3841 gccaacgccc gatcagttct gggggcagtg tattggaatc gtgacgccac atcgcgccca
3901 acgcgcgttg gtggttcgcg aactgaggtc gatatttccg tctgatccgc cggatttgat
3961 cgactcggcg gttgataccg tggaaaagtt ccagggtggt cagcggcaca caatcatcgt
4021 gacttttggt gtcggggatg cggacgtgat catgggggag gaagcgttcc tcatgcagtt
4081 agagaggaca aacgtggcca tctctcgcgc gatggctaag tgcctggtga ttatgccgat
4141 gacgcttgcc gggcacgtcc ctcacgacaa gaaggctctt gagacagctc atgcgatcaa
4201 agattatgtc gacgagttct gtaatcaaga aatgacggac cagatcacgt ccggaccgac
4261 gccgaagcaa gcgaagttgc gctaccacca gtaatggctg ctgaaattaa actcgttcga
4321 cgtgaaaatc gaattcaacg gcggcggcgg attcagcttt catctgatcg gcttgccgct
4381 tgccagcatc tgcggacttt gcagggcggg tcgacaccaa gcgccacggg acaccggatt
4441 cctccaatga gttcttgaaa atacttttcg cgtcgactgc ggcagaagga atgatgccga
4501 agcggacgta catgtgcagt gtttcagaag aatctgcgct atcagcaata tttcgccata
4561 cccttccgag tgaagtcgcg aagacgctct gtccgccgtg gtcaagctgg gggccggccg
4621 aatagtcaat gaaatcaggg ccgccgagga accagtttct caaccactga tcctgaacgt
4681 acgtccgcat tccgtagtag ggaggggatg tgattacaac tgacggtgca gtactaaccg
4741 atcgaaacac ttcgaccgac tgagcgtcgc catgcaagac ttgagatatc ggacatggag
4801 attcttgctc tagtcctact aaacgctcga tcttgcgacg gagcacccga ataacgtcaa
4861 tcctaggggc ttttagcttt ctttctttcc agaaacgcac cgcgtaatca ggcttcgacg
4921 cgtaggttct gggcatttgg ttcgagaaat agcccgcgtt ctccatgtgc ttggggacag
4981 ggccgtgcag gcagcccagg gccgcagcgc gcaggatggc ggatgcatcg gtctccgccc
5041 cgaggtttag cagcccttct cgcaacgcac agacatttct aagcgtgtcg cggtgatatg
5101 cagatcgaaa gaatgctgtg tcggggatct ccgatggatc gacttctgag attagccttt
5161 cggcaagccg gagaggttct tcagtatcgc agcttgcgag cttggccctg gcaatggcaa
5221 cagcgaccgg agatgtgtca ataccccatg cggcaagccc gagcgtgcga gcggcaaaca
5281 aagtagtgcc tcgaccacaa aaagggtcga gaacaacggg cctttctttg cgatgcttat
5341 tgagtacctt ggcggggtac tccaacggga acatcgtgaa gtaaggacaa atcgcgttca
5401 gtgcattttc tttgctatat gcaagggcca cgtttttggt tcttgtgttg tgcgtcattt
5461 cgggatataa gggtcagttt acacggccga ccgccgacgg ggaacggagt agggtcacgt
5521 ttctgctcct cttgacatca gttttgcccg agtagctgca aacattctgc ggttcgccgc
5581 gcgaccaatc cggggagcac cctgtcccgc catacaccca ccgcccctgc tcgctcgcgg
5641 cggcgatcca gtcccgctga ttcacttttc gccgcagggc cgacgtctgt agccttcccc
5701 ctccgagatt gaatgtgaaa tcttcgatgg ccgtgagacg cgtccgtatc acagggatag
5761 ccgtactcca aatagaggcg tgatcctagg ccccctttttc aaatcgtccc cagatccaga
5821 atgaactgct ccaccctcga ttcttcacga ccgattcgag cggtttcctg aggtcctctt
5881 ccgaccggta cagtacctcc ccggctctta ggcgcatccg gatgccccag gtacggattt
5941 cgtagcgatt gcggtcaaaa ttcggccagc cgcttcccct taatacaaat tcaacggcgc
6001 gtaaaaatcg gcgtagctgc ccggggcgcc cgcgctcaat ccggtcactt cgcgcagctg
6061 ctgcccggtg gagatcgcgc tgaacgtccg gtaccgcaac cgctgcggcg ggtccgcact
6121 ttcgtgccag acgggaagct tgaacgaggc cacggtgatc cccgagtccg gcagcgtgac
6181 acagccgcca tagtcgatcg acttgaacga cttgcacttc accagcgcac cctccgtctc
6241 cggccacccg gtgtgcaata cctcggtcat gaatttgccg cccaggaact tctccagcgt
6301 atcgatattg acgatctcgc ccgaggccaa gcccggagcc acgccataca gaatgctcga

6361 gagtcgcgcg tcgaaaaacg ggtccgtgct gttctggacg gttgtcccat accacgcgcc
6421 ctggtgcgtc gacttggcga agaggtaatt gttttgccgg gggtccatcc aaatgatgcg
6481 cgccaacagg tcggattcga cgtaaatcag cccttccgaa atgaaaaaga actcgcccgg
6541 cacgccgttc atgccgtacg cggtgtcgta gggcggaccg gggggcggct ctcccgtgcc
6601 gtccctgatc gttacccgct ccatgaccgg gtagcccgca taaggatgcg aggcatacac
6661 cttgagcgaa cgcgccttgc ggcaggtatt gtcggtgtag tcctcgccat acaccgtatc
6721 cacgctgccg aagtagtaca ccttctgatt ggcgccgtcg tgcgactgta ttccgacgat
6781 actgcttccc gagatttcct ccccgtagcc ggtcgatccc atcccggact cgccgaccgg
6841 taccacacca gaggcgtgaa atcttatttt ctcggtgtat tgataagtat cttcccgaag
6901 aatcggataa aggacttcat gtcctcgtcg tcgtaataaa cgtagacggg caggtcgaaa
6961 tggctttcgt agccccgctc gatggtgtaa tccacgaagg attctccggc gtggcgctga
7021 ttattattgc ggcccggatc gtaatcgccg ggcgacccgg tcgcgatcaa ctgcgccgga
7081 tcggggttgg tgttgtatat cttgtaggtc gaccaggaca cggtcgccgt gtgcgggttg
7141 agcgtttcga cccgggcgct gttgggcgtc cagaacgact cacccccttga tgcgcatcca
7201 cccgtgtcgc ccggccctgc acggcggata gcaccttctt tttatttgtc ttgttcttgt
7261 catcctggct ccagatcaat tccacgtcat ggagatagcg ctggaaaaac ggcacccgtt
7321 tgaaattggg attcaaccgc gccgcgccca atttgttgat gaccgactcc atgggggatt
7381 cgttcaggaa cgtgatgacc gcttccacgt cgtcggccag gtcgctattc gcccagttgt
7441 agggcacgcc gtcgctgaac tcggaatcga cgtcgccttc ctcctcggat ttatagccgt
7501 tcgcaatgcg ccagtgccgc cgcgccacat acatgctggt gtcagggaag cgatagcttt
7561 tggcaatgca ggaggcctgc gtgcaatccg aattgaattc aaacggcgta tcgatctgcg
7621 gataaatggg gccgtgaaaa acgaagcccg gcggcgtggg cttgaccgtg aaaccggggt
7681 agaaggtata acccggcaga tcctggacct gaggcgtcag cttgtaatac gtcaccgaca
7741 cggtttgacc gtcggcataa ggtttcgaga tatcgagggg ggcgaaggcg atttcgttgc
7801 gatggatggc cgccagcacc agggtgttct cgtcgcggat gccgaccccg acgatcttgc
7861 cgaagaaatc cgagtcgtaa acgtttttct tggcgtcgat atacagccgt tttccgcgca
7921 ggtagatgta attgctccag ggttcctcgg agtagcggtc cgtgcccaca tcccagccgt
7981 agggctgcag gtcgttatcg acgaacaccc ggttgaacgg cccgtcgaac gacacccgaa
8041 tccagcgctt accgatcttg cgctgaacca ggcaattgcc caccttgcgg ccggaagcgc
8101 gccgctggac ccgcagggcc ttgggctttt tgggatcggt ctcggccgac agctcgacgc
8161 cgatggctcc cgccgccggg gcgtaagtgc ccagcggcgg gtggatcggc gctccttcag
8221 catccttgaa cggctcgccc cagccgtccg gatagttgcc ggcggtcggc gtgcacagca
8281 gcccttggga ccggatatcg acgatccgca ccaggtcgaa cggatcgccg ggccgctcga
8341 tcgttttcat gccgtcgtcc ccagtgcttt atccgggtcc ggctcggcga aatggaactc
8401 gaccgcctcg ccgttggcat cctggaactt caacgtcttg atggcggcgt actttatgac
8461 gaactcgccg tcggtggttt gcatatcccg agccgggtaa tactcccgat ccgcccagaa
8521 ctcgccagtt gcgttcggcg ccgtcgtctc gctgaggggg gaggcgatgc cgccgccgcg
8581 cttgggcgct cccaccccgg tgcgggcgcc gatacccggc ttggcggccg gttcgggcaa
8641 atcatctccc ccgctccgca ctgcatcgac gatggcgttg atatcgttga cgaagaactt
8701 ctgaaccgtc atgaatactc caccgatcga tccggattac acgacagccg tttcccgcac
8761 gtcggcgatc accaccgaca gctccgtcgc cgtgccgacc acgtgggtgg catcgttcac
8821 ctcgatccag atcggcttgg cgttggccgc gtccgacaaa acggacgtac ccaggtccag
8881 cgcggcgccg ccgaccgccg acgccaggcc ggcttgagtc gctgccaatt tcacttcgct
8941 tacgtcgtgc ccgctacccg cgccagcgtc caccacggtg gcctgaatct gatccacacc
9001 gggattggcg ttgcttgga tcatccggct ggcggtggtc gagcccaggt atagggtgaa
9061 ctgaatggcc ggggtggagc cgtcggcatt ctgggtggcc accaggctgc ccgtcagcgg
9121 ggtggtcaag gcggcatcgg tgtacagctt gaacgaggac atggttttag gcctccaaaa
9181 gtagttcgtc gtcggggatg aaaatgctca catggtcggc cggggccgag tgggtggcgt
9241 cgatgtgctc ctgcgctacg cccggcaccg tgaccatgaa ttctccggtg ctgctgtcgt
9301 agctcgccga ggggcgctcc atcggatatt tcaatggcgg ctgcaccacc gccggcttgg
9361 ccggggcctg cagatcgtag ggcgtgtcga ccatgccgac cccgtaggct ttcgacacgg
9421 cgatggcgac ttcggtcagc gccgagccgg catcgaaatc gaggttgtgc gtcacccgcc
9481 gcaccttgcc cttggcggtc agccgcgcgg acagcatccg taccgtatgc gtccggtcca

9541 gcagcggcgc gatcagaccg gtaaaactca gcgtggtttg gcggtgcgaa gacaggatgt
9601 cgcgctgggc gcgggcgatg ccggtcatgt agccgttgga gagtccggcc cggttgtcgg
9661 ccacgccgtc gtccaagtcg tagtacaggt cgccgcgggg attggctaaa acgtcctggg
9721 acggcagccg tttcgcgccg tccggcgtgg cgacggtgta ctgtttgatg ttcagcgggt
9781 ggtaggggat gaacgaggtg tcgccggcaa tgggcgatat cgtgccctgg ccggccaggg
9841 tgaacttcgt ttcccgctcg gtgatccggt cccaatgggt gtagcgcgga tccttgtcgg
9901 cattggagtc gaagttggcc gtggtgctgc ctttgcgctt gccgatgacc tggatcgagt
9961 ccggcgcgtc caccgtcacg ctccagtctt ccgtgaccgt ctgggtccag cgctgcgcca
10021 gcgcgcaatg tagccccacc gtcaggctgt agcgcggcac gtcgcccagc aggccgacgc
10081 cctcgaatgc ccccgtgggc accggcagca tttccaggcc gtagcaggcc cagccggtgc
10141 catcgacggc ctggaacacc gtttccaccg agggcgcggc accgccggcc accagctcgt
10201 gcatgtccat ttcccagaaa atgccgacgt cgcgctgcat caggcgttca tagcggtact
10261 gcatcttcac gtcgatggtg ttcaccaact gccgggccga agccatgtcg atgcggatgc
10321 tgttctccag cacggcatcg gggcggaagg tgaaatcggg ggccgtcttg gcacgccagg
10381 gggtgacgac gccttgcaga ttcaggtcga ggtcgtagct ggccggcagc gtcgacagcc
10441 ggtcctgcag gtattgccac tcgtcggcct tcttgtcgaa cacgtactcg gaccagaatc
10501 cgccgatgat cttggcgatg tccttgcgct tgatgccgtc gaagcggttt tgcaggttgt
10561 cggtgcagga cagctgcagc acaccctgca ccgggtccca accgggaatg tccaggatgc
10621 cttggaagat cggcaggtgc gtcgtccccg ccaccaggta gatcgtgatc ggcaagccgg
10681 tcagcgcgtt gatgttgatc gggcccggct ccggcagcac gctgaattcg gcgatccgcg
10741 ccgcgccttc ttccgcatcc accgtcatct gcccggtcag ccggcccagc agcgcgccca
10801 tcaccgcccc tgtccccgca ttcagcggtt cgccgtcgcc ggcggcattg ccgatgttca
10861 ccaagaccgc ccaggcggtg atatccgcgc tggcgctcat cgcctgggcg acgaaggccc
10921 cgccgccgac ggcttgggcg tcgaacaccg tgtggcggat cggcacatcc atggctgtga
10981 gaacttcgta cacggtcaac tcgacctgcg ccgccagatc gaacgggagg tcatccacca
11041 ccaaggtgat cggcgcggca atgccgtaga cgtcggcgat ctgctgtgta agccaagctg
11101 cgcggcggta gggtttgaag aagatgcttt gctgtagggg agccgcaagc aatgagatcg
11161 agaactggac ttgctggatc ggcgccgagt tgccggcgac cgccaagacc tcgaaccgga
11221 gcggcgccgt catgccatag acgtcgagca acgtctgctc gacggcgacg ctcaggccga
11281 agcgttcgaa gaccggctga tcgatgacac aaaagacgtc gtaggcgtcg atgtcgacga
11341 cagcctgggt gataccggtc ccgaggccgt aaacggagaa gaccgcttgt tccagtaccg
11401 cggtaagcgc ataccgctcg ccgacatccg gccccgccgc aacaccgccg atgccggtaa
11461 cgccgccgac ggggagcgtc attacaactc ctcggccagc acgctgacgt tggcgagttg
11521 aaagccgccg gataccgcag cgcctgtgtt ggatcggctg agcatgaagc agaccaggct
11581 gtcaggcccc acggtcaccg tgttgacgtt attcgacttg aagccatagt cgccgggcga
11641 taccaccact tgcaaagcgg acggggacgt cggcgccgtc gagcccgctt ccaccgagac
11701 cacgatgctg tcaccgctga aactcgaacc ggtcgagagc gcggcgacga gattggagag
11761 ccggagggtc ttgccggtac gattgatcca gtacgccgtg cccatggacc ctgtggtgct
11821 gcccgaaaac ggcgccacga accgggtgct ggtcatcgtg ttggtcccgg tcatcgacgc
11881 cgacaggatc atccgccagc gcgactccat ctcctccacc cccggcgacc attggacgaa
11941 ggccccttgg gtgaaggtct gagccacccc ggtggaactg gcgacgttgc gggtgatcgc
12001 cagcaggcaa ttggccccca tcccctggca ggcggtgact ttcacccgct cccacttcgt
12061 cgggtaatcc tgatcgatga tcgtcaggta gtagtattcg ccgctggcca gcaccggcgg
12121 caaaacccag ggcgcaccca gtgcgctgac gttcatcgtg aaatccgacg gcgctaccaa
12181 ggcgtccgtc accatggcct tgacgccatt gagcccggcg agattcagca tgtcatgctc
12241 ctaggggatc gaccatttcg gcgttcaggc gccacgaata attcccgctc accccgtcca
12301 actgctgctc gggggctcg cagtagacag tgatcttcgg gtagtagtag gtgatggcca
12361 cgccgtcgac gtcgatcgtc accgtggcga aatcgggctc cgtccgcacc ggaaccgggg
12421 gcgatccgcc gccgacgacg gacaggggcg cggcgcaggc cagcactaaa ggctgcgagt
12481 agtccagcgc cgacagcccc ggcggcaccc agccgcctcc gctgatttcg gtcgccagct
12541 tgcgccaggt ctgctgcttc accgagcggc cggaccacat ccggtgggtg gtggccccct
12601 gcagccagcg gtaggtctgc gaccattcca gccgggcata cagcggcacc accacgccgc
12661 cgagcttgaa tgccgtgtcg cggagatcgc tcaccgttca tcgggctccg gctcgtcgca

12721 gcgcaaggcc aggccgactt catcgacgcc ggcgacgagg tcggtagccc gaaccggcca
12781 cgcgcacgcc cgtggcgcca gcgtgaccca cagctccagc gagcagcagc ccgtcagtgt
12841 cagcacgacg cccaccccag cagtaagccg gcaagcccgc cgaataccag cacggcgacc
12901 gtggacaacg tgatttcccg tagcgtcagt acgatcatgc ctagccgacc cgggcagtgc
12961 cgacgcacag cgcatattcc gcctcccggc gccgcaccag gccgttttga acgcggccgc
13021 cggccttgtt ccagcgcagg atttcgcggc aggcctcggc atagccgggc ggtgtggagc
13081 gcagcttttt gaccagggtc gatccgcaga acgcattcac gccgatgttg taggtcagcg
13141 aaacataggc gtcgaactcg tgctgataca gcggtacctc gcccacgcag cgcgctaccg
13201 cctgcgacac ccgagttgcg tcggcggcca gccgctgcac cgcccgcacc ggatcggtcc
13261 ggtctccgcg cttcactccc gccgtcgagc cgaagccaat cgtctggacc ccgactccgt
13321 cgtcgtaggc ctcgccccgg tagccttcat gcagggcaat catgccgacg atcagcgcgg
13381 aggctgacat ggccgccgcc gtcgtgcgcc tcatcgcctc cccgcctcct tgttgctcgc
13441 ccggtggagc acatcgacca cgatgacgcc cagcacaccg aggccctggg ccactgcggc
13501 gatatccgta cccgtggcgt cgacgtcgta gcccaacgcc cgcgcaaggc cgactccagc
13561 ggtcagcaac gcagtcagcg ctgcaatcag actcgcgcgc tggctccaca ggcgagggtt
13621 ctgcaggctc ttgccggcct gcatgcactc gtacagatat ttcacttttt cgaacatatc
13681 gccgccctcc tgtcgaaatt caccgtgttt ttatgcacgt cagggcgctt tcacgccgaa
13741 cgagcgcacg aaccacagcg cggcctcgat gaggaaatgg acgacgttgg tccggacacc
13801 gctgacgaag gtgccgatgt aggccgccgc ctccttcttc ttgatgtcgt tcagcgtggt
13861 cttgtcgacg atccctttga tcgtccgggt ttccagagcc gccaccttgt cctggatcag
13921 gccccattgc tccttggtga tgccggagac ggcctgcact acaagccaga gcgcccagcg
13981 taagatcgga ttcatcgggt attgctcctg tcggttggga aactcatcgc acgatccgcc
14041 cgtgcttcag cgccgcccgc gccacctcgc gcttaagcgc cgccaccact tgcgggtcgc
14101 cgttgagggg gaacgatccc ggtatgccgg gcaaatggat gtgcaccgcc tggccgccgt
14161 tggacgaacg cgccagcgag gcgtcgaatg ccggcaccgc ttgcgcgacg tagccgccga
14221 tgctgaaacg ggggagggct aagttgttga gggcgtacag cttgtcgagg ccgagtttgc
14281 gcaccgcctc cttgcgcagc acgaactcgc cttcctccaa cagcgccggg atgcggtcgc
14341 cgccgccata gcccttgatc cagccgccgc ggcggaagcc gggcaaagcg gcgtcgccgc
14401 cggtggctcc cccttggctg ttcatcacca ccgtgatggt cttggtttcg ggtttcagca
14461 gctccgccag ttggctttgc agggtctgga gcgaagccgg gtcgacgtcg actcgcaagg
14521 cgaacccttt cgtcagctgc tggtcgatgt cgctgatttc ggcctggacg ctctgcaggt
14581 tttgaagggt ttcctggatc ttctggctga ccgcggcggc cgcctgggtg tcggcctgtt
14641 cctgccgggc ggcggcttcc gccaggacct gcccggcctg ccggcgcgcc tcgatcagcc
14701 gctgcaggtc ctcggtgctg aactgcggct ggccgggatc ggccggggtc tgttcgcctt
14761 tgagcgcgtt caggcgctgt tcccgtaaag aggccgcggc ctgctgcccg agggcttcgg
14821 cgaggccggc agcttgctct ccggcctgtc gcgagagttc caggttgccg ttcgcgagtt
14881 cctgccgctg ctgagcgagc aactgctgga gctgcgcgaa ctgggcccgt gcggcctgca
14941 acggcgtcag ggccgcctgc tcgatgccgg ccaccgcccg tgcggtgctc tgctcgatgc
15001 tgcggcgttc ctgggctgag gcgcgcgccg attccagcag ccgctgttcc tgcccgatca
15061 gcttgtcgat ggtggcggtg taggccgatt ccaggtccgc gtagatggcc cgccgggctt
15121 gcagcgattc gcgctccagg gcggccttgc gggcttcgcc gtccttgaac ttggcgattt
15181 cctgggcata gacctgatcg accagcccga ggcgctgggc ggcatagtcc cgcacggcct
15241 tcaggcgggc gccctcggct tgcagggtca aggcatggag ctgacgggcc tgctccagct
15301 ccgaacgggc ttgggtggct tgcacgccgt tccgggcgta gacgatgggc ggtgcggcat
15361 cggcttcgat cggttgcgcc tggatgcgcg tctcggccag cttctcgagg gcgtcgattg
15421 ccgccgtccg gcgctggtag gcatcctcga ggcctgccgt cgcatctttc agcgcgttct
15481 gggtgtcctt gaagctctgc tggacctctc tttccttggc cttgaccgtg tcgccgctct
15541 cggccgcggc ctgcttgcgc ttggccagct cggcccgcat ctcctggagc agcgtggttt
15601 cgccggccag ctcccgcccg atcctgccgc ccatgaaggc ctggtccgcc gcctgaccga
15661 cttccttgta acgcgccgcc agccgggcga cgacggcttc ctgtttcttc actgcggctt
15721 cgtcctggag caggctgaga tcggcttccg atttgccccg ggtgtccttg ccggtttcct
15781 tggccaactc gcggcgtttt tggatgatgc gctcgatttc tgaggtaccg gcctggccga
15841 acagatgcca ggcggacact gccgtcagca gcgtcaccgc gatcccggcg gggccgccga

15901 ggaaagaagc caagccaccc agtgcaatct tgagcggtcc ggcactggcc gccacttccg
15961 cctgggcgac ggccaggcgc tgcgatgcgg gaacaagctg ggtttccacc agcgccaacc
16021 gcgccatgcc ggaggcattc gtcacggcag cttcggcggc ggcgagcgtg gcccgagcgt
16081 tgtattcttc gttgagggcg tggttccgcg ccgcggtcgc cgccgccaga ttcgcttgca
16141 ccagccgcgc ctggctcccg gcgaaggccg ccacggcggc cgtgcctttg cccgccagca
16201 ccgccaccag gagggagccc gcgatctcga ccccgttggc gatcgtgtcg atgttgcccg
16261 ccgcggtatt gatcgcgccg gcgatggcct cggccgcgcg ggtgatctcg gtcgaggtgc
16321 cgacgaactt cagggcggcg ttatcccagc gcactaccgc ctgctcgatg gtatcgggca
16381 gcccggcggc atcggcgtcg atgctggcct tggccttttg cagcgcgcct acgaccttct
16441 gcgtggtcag ctcgccggct tcggccatgg cgcgcagctt gccgatcggc acatcgagtc
16501 cgtcggcgat ggcctgggcc agccgcggcg attgctccat gatcgagttg aattcgtcgc
16561 cgcgcagcac gccggaggcc agcgcctggg agaactgcag ggtggcgccc gaggcctctt
16621 ccgccgaggc gccggaaatc cggaaggatt tggcgaccga gtcgatgatg ctcagcactt
16681 gttgctgaga tacacccatg tccccggcac ccttggcgat gcgggaatac aattgagaga
16741 cgccggtcag ggcttgcgcg ttgttctggg cgatggcgaa cagcgcccgc tgcgcctctg
16801 tgaactggtt ctgggagccc gccgccagct tgacccggcc ctcgaggttg cggtactggt
16861 ccgagagttt gcccagatcg cgggcagcct gcagggcttc ggcggcgccg aacgcgccga
16921 cggtatactg cccgatgcgc cggagcgatc cggtcagccg gtcgacctgc gccgcgacgt
16981 tgccgagccc cttctcggcc gcggccgtgt tcgccgcgat ctcgatgacc gtcttgaaag
17041 tcgtcatggg cctagcgccg ccgcatcaaa cgcgcctgca ggaacagccg ccaggggtaa
17101 tgccagacgc cggcgtggcc gcgctcgatc agcgccagcg ccgtcatctc cagattctct
17161 aggttgccgg cggcgccgta gcggccgcct gggccgcgtt ggccaggcgt tcgcggagcc
17221 cgaaaaaatg cgggttcagg ctcttggccg cctcccgcac tttgtcgagc tccgacggca
17281 gcatggcgtc gagctcggcc cgggagaggt ccgagaaccg ggtcaaatcg tccagggtga
17341 tgtccgcgaa cagcgccagc gccaccaggt cgttccgatc caattcggcg ttggcgtcct
17401 tcagccaagc ccggacctcg cccacagtca gctcgcgcac cacgaccttg cggccgccgg
17461 cctcgatggt gcgttcggcg cggaggccct tggggtgcgt ggtgtcgagc tctgccatag
17521 ggaattacac cgccggcgtg atggtggcct tgaagtactg gctgacgccc gccccggtct
17581 tgctggtgtc cttcagcacc ttgccggtca gctccagcac tgcgaaatcc tccgtaccgg
17641 ggccgggcag gctgctggcc ggcgagaact tcaccttgta gaagtcgtag gccacggcat
17701 cgccgttgac ctcgttgacg ccgtcgaagg tgatgtgcca gtcctccgcg gtctgggtca
17761 gcgcctcgat gctgttgccg ctcaccgtgc cgcgggtggc cagcgccagg ttctccttgc
17821 tgatcgacca caacttcagt ttcagcgtga ccgacttgat gcggctcaag gaggcgatcg
17881 agccgccgcc cgggttctgg taatcggcca gttccttggt gtcctcttcg atgctgaggt
17941 ccagggcggc attgcccagg tccaccgggg tgccggcgcc gacggcgccc ttgatggtgc
18001 cggtcaaaag catggcagtc gtagtcatgt gtcaactcct tagaaaaagg gatggatgaa
18061 aaaagaaggg aatatcgacg ccgaccgggc gtcacggggt cccggtgtcc agcggcagcg
18121 gacgcacctc gaacaccagg gaaaactcga catggccgag atcgaaccag gggtcgtcca
18181 ggccgacgtg cttcatgcgg ccccagcccg gttgcgggga ccagtgctcc agcgcgcgca
18241 ggatccgcag cacgtattcg ccgcccgtca ccacggtgct ggccggcgga gcgtgggcga
18301 cgcacaccgt gacctgccag cgctggcgct cggccaggaa aacgttgggc ggcggcgtcc
18361 cgaccggccc ggcgaagccc aagggcagca ctaaggcggc ggggcagagc ggttccaggt
18421 tctgcacccc ggccaggatg ccgacgctgt ccaccgtgac gaactccggc agggtctcgc
18481 gcaggcgccg gatcagcgcc ggctcgatct cgaacaggct ggcgatcatc gcggcagcgc
18541 gtcctgcgtc atttcacgga tcatcgcggc cagccgcagc gtggcccggc ccgccagcgc
18601 ttcggccagg gccggcacct gcggcaaggc caccacgtcc tcgaggatgg gcaaggatcc
18661 tcgcccgacc cggtgaaaga tcccgcgatg gccgctgggc atccgggcga cgaagctgcc
18721 gggaaagaga taggccccgg cgctgctgcc ccattcctcc tggcgcaggc ggccgacata
18781 ggcggccttg atcggggcgt ggccggtcca gacccgggcg ctgaacccgg ccagccgcag
18841 atacgcctgt acccggcgcg agcgcagcgc cctcagcgga atgccgtagg ttgctccgat
18901 cgcccggacc atcgcctcgc gggtctcccg cgcggtctgg cccaccgccc ggcgggacgc
18961 tttgcgcagc accgccggca agcggcgcaa atcctgagcg gtatcgccgt gcagctgcac
19021 gcgcaagccg agactcatgc ctgttgccta gccccgactc gaatggatca gcaaaccgaa

19081 ccagcgcgcc ccgagatgcc cggcgacgat cgccgcccac aggctctcgt agtagcccag
19141 gccctcccgc tcggccagca gccacaccga gaagccgacc aggctgcagg acacgacgtc
19201 gaccagcagg caccacaggc acggccgcca cggccgggga tggcgccgcc atcgattgag
19261 ctgggccacc agccccgacc agatcgtcac caggatgatc tgcagggcca gccggacctc
19321 atcgccattg ccgtggggcg acaccagcag gagcttcttc ggcggggcgt tcatcggggc
19381 cgcaggatca aggtggtcag gccgtccagg cgatcgaggc gggaggcgat gccgtagggg
19441 aggccacgca gcttcagggg atcgcccatg cgaaccgcag gcaggtccgc actccgcacg
19501 gtcagtcgcg ggtccgcctc gggacggggc gcgcccatcg gccccgggcg ttccagtccg
19561 gccgggaccg agacgatccc cgtcaggtgt accggcggcg gatcggtccg gtccggccac
19621 aggatcaccg tctcgccgaa ggtcgacagg cacaccgcat tgaccgcatc gaggtccatc
19681 cctagaccgt cagcttgacc agcagccccg gccggtggca catcggcagc gggttggact
19741 gggtgtggag atcggtgccg cggtcgaact ggcgcggggc ctgcttggca tagagcggct
19801 ggcccaaggt gttcaccgtt tcgttgaaat cggccggggc gacataggtg gcgaaggtat
19861 cgacggtgcc cagcgggaag gcatgggcct cgccagcggc gatgaagcgc cgggtcaccc
19921 cgtgcacgtc ggtggcgcgg ccgcggtact cctcgaaggt gatgccgccg aaggtgaagc
19981 cgcggcgcac gtcgttgatg aggatcgcgc cttgttgcca gtgggtgaag gccgtcttga
20041 cgtccttgtg cccggtcagc gcggcgagga actccggcga gcacaggcaa tgcacctcgg
20101 tcatgaactc gcccagcagg ctgtcctcga tgtgggccag cacctccatg catcgcgcct
20161 tgacgtcggt ggtggccgcg cccagctgaa agccgaccgt cttcggcggg atgtcgaact
20221 cgtcgtagag gtcgtacagc accgagccgt cggcatccag gatcaccccc ttgagcgcgc
20281 ccatgcgcaa atgctccagg gtgatcgcat gcttgttgcg catggtctcc agatggcgcg
20341 ccatcacgcc ggctaccgcc tccagctcgg tctccgagcc gaaggcccgg atgccctgga
20401 cctcctccgg cagcactacg tcgtcgtggg ggatgtgcgg aatcacgaac gagcgcaggg
20461 tgcgcttgcc gcggacgccc accgtgcccg gcgcccccgg cggcagggtc ggcaacaggt
20521 tcagcacgcc gttgcgctcc tcgacgagga tctggcgctg ccgcaccggt ttcaccggca
20581 tgagattcag atcctccagc cggccgtagc ggttggggat gatgttgatg gcggcggtca
20641 gcgccgccat ggaaaaggcg ggattgtgaa acgggttctg catgatcagg ctccggtgcg
20701 gacgaggatg cccagcgctt gcagctgggc gatcgcggtg gccttctggg ccgggttgat
20761 gccggccggc cacaccaggg ccgaatcgga cacgacggca tggcgggcga gcagcagggc
20821 ctgcgggact tcggccgcgg tggcatcgac cgcttcgatc agcaccccca ccgcctgagc
20881 cgaggcatcc gtcgcggcgg gatcgagcgc ctgcagccga atgccgtccg gctcacgcgc
20941 taccactgcc cccagcgcca gcttctggcc cagggccact ggggcgaggt cgcgggaata
21001 gcggttgggc gcctcgtact tcaagagatc gccgaggttc ttcggttctc gcagcttggg
21061 catggctcag ctccgggcga tgagtttctg gacggcggcg accacgggac ccgtctccgg
21121 tcgaatggac gtgccggcat cgacggcgat gcgggaagcg atctcgggcg tctcggcccg
21181 cgccgccagc aaggcccgcc gcacctgggc ctcggacagt ccggccgcca ggaactccgc
21241 ggtgcgcaag gccgcgccgg cgatcaggca gagctcggcg atagcctggg cttcgctccg
21301 ccctgcgtgg tgggcttcgg tcgcgaggga ttgggcagtc ggatcgccga ggacaggggt
21361 agccgcagcc aagtcgttcg ggggggacgg tgcggcaggc gacgtaggt tgccggccgc
21421 atcggtgtcc ttgtggattt cggcggccgc ttcgtcattg agtggaccag gcgcgtgatc
21481 gtgcataact gactccgaat gaaggagaga aacgggtagg ggtgacatca ccgccgcctt
21541 ggggcttggc ggcgagatca gtcgttgtcg agcgttgatt gcatcggcga actcgctcag
21601 cacggcatcg aagccgagca cgccgtcggc gagtcccgcg gacacggcgt cctcgccgaa
21661 gaacagtccg gcctcggtgg cgcgtaccgc ggcctccggc agtcgccgca acgccgccac
21721 gtcccggatg aagatcccgt agagccggtc cacctcggac tggagcgctt cggccgcctg
21781 cggcgacagc ggcgcgtgcg gattgaggtc gttcttgtga tgaccggcat agagcgcggt
21841 gtaggtcagt ccctcgcggg tatccttgac cgactggtcg acgtgcaggg cgatcactcc
21901 gatcgagccg accccgccgg tctgcgccag ggtcacgcgc gaggcgccgg cggcgatcgc
21961 ataggcggcc gagaacgccg tgtcgttggc atgggcccag atcggtttgt gagaagtccc
22021 cgcacggatc ttggccgcca gctcgaacac cccgccggat tcgcccccgg gcgagtcgat
22081 gtcgagcagg agacctgcga cctcgggatc ccggagcgcg gcatcgagcc ggacgccgat
22141 ctcggcatag gaagtcaggc cggaatgcgc gtccagtccc tgggtgcgtc ggaccagggt
22201 gccgtggatc gggatcacgg cgatgcccgg aagcgcttgg gcggctgtcc ggggcatcgg

22261 tgccggcggc accagagcat ccgctgccgg caacccgatc cgggatccca acaccgccag
22321 gatcacctcg agcttcggtc gggcgacgag gagcggtgtc ccatacagcc ggccggccaa
22381 gtggggaagc atcagccttg cccacctcgg tcgctctgac tacaattgtg gcgaaccact
22441 tgagaactga tcatgagcgt ctccgtccgt atcgatgaaa ccttgatcga ggaagcccgc
22501 gcggcggcca aggccgagtt ccgcaccgtg caggggcaga tcgagttctg ggccaaagtc
22561 gggcgcgccg cgctggacaa cccggatttg ccggcttcct tcatcgccga aagcctgatg
22621 gcgctgaatg agtcgcgtga gcaagccacg ccgttcgtgc cgcgcacccg tcggtgaact
22681 ggcaggtgct gcagacgcgc cgtttcgcgc gtgcctacaa gaaactgcac gatccagtgg
22741 cggccgacgt cgatgcggcg gtcgaggtga tcgccagcga tccgggcgtc ggtgagaaaa
22801 agaaaggcga tctggcccgg ctgtgggtct ataaaatccg cagccaggtg cagctctacc
22861 tgctcggcta cacccgcgat gacggcttga gcctggtcta tctcgaggcc gtggggccgc
22921 acgagaattt ctatcgcgac ctcaaacgct gacccggttt ccaccggctg ccggcgccga
22981 tcccctggcc ggcgcggcag cgcggtcatg ccgggggtcc gaatcgaaca ccagccccaa
23041 tgcatccgcc cgggcgttgt cggcggcgat ctcccggtcg acgtcttcgg cgtcgtaacc
23101 aaaggccgag atcgcctccg aacgcgacag gaggccggcc cggatcgccg tctgcaaggc
23161 atcgaactcc ttcttgggat cgacccactg ccagccctgc gggatccatt tcaccgccag
23221 gtagtcgcgt ggcctgcggc tgaagccggg aagttccagg gcaccctcca gcaccgcctg
23281 ggtcatccag gcccgccata ccggccggca cagctggaac acgatgacgc cgtgctggat
23341 cgcctcgcag cggcggcgga actccagcag ccccgcgcgg atcgaggagt agttgacctg
23401 ggtcaggtcg ccggtcagca tctcgtaggt gatgcccatg gccgccgcca ccgcccggaa
23461 ctgcatacgc aggaactcgg catagctcgc ccccacatcg gccggctggc tgaaccgcac
23521 gtcctcgccc ggctccagca gctgcagggt ccccggttcc agccccgcca gggccacccc
23581 ctgggcgtcg gccggtcctt cgcccatgag cggatcctcc ggcgccagcc gggtgatgaa
23641 gccggcgaac atcgccgcgg tcttcttccg caccagctcg gcgtcgtcgt actggtccag
23701 ctcatgcagc ttcaccaacg cccgcgccag ccagggctcg ccccggatct ggccggggcg
23761 caggggacgg aacagatgga ttacctcgct ggcgtcgacc cgcaccgtgt ccaagccgcc
23821 cgccccggac atcggcgcca aggcgccgtc ctccggatgc gagcggtaca ggtgataggc
23881 cacccgccgt cccaaccgat cgaattcgat gccggctcgg atcacgttcc ctgaaggcag
23941 ctcccggttc agggcgacgg gcagatgttc cggctccagc acctgcagct ggagtcctac
24001 gctcaagcca tcctccggcc gccggtagcg cagccgcacc agcgcctcgc cgccttccac
24061 cagggcgcgg caggccaggg cctgcagccc gtagacatcg gtcagtccgg ccgcatcggc
24121 gtcatcgccc cagtgggccc agagcgcctg gatggcggag cggaccgtga cgtcggccgc
24181 catcgactgc ggcttgatgc cggtgccgat ggcattggcc acataagcct ccaccccggc
24241 ggcggcccag acgttgcgcc gcacttggtc gcggctcttg gcccgcagct cgtcctggct
24301 gtaggccagc gccgccaccg cgcccggatt gccgacctgc caggccagcg cccggcggcc
24361 gccgccggtg ccgtcgtagg tcggcgccgg tccgccgaac agccccaagc gcaaccgctg
24421 ccaccagccc atcaggtggc cttgcgcgtg gtgatgcgga gctgccgggc caccggtccg
24481 acctgggccg ccaagccgcg tttcacctcg cggatcgctg ccttcagctc ctccaccgag
24541 cggtactcga cactgcggtc ctggaacgtc acccgacgct cgccccgggc cagggcggtt
24601 tccagggctt gcaggtgttc tgcggaatag gccatcgata agctttcttc ggtctcaagc
24661 cagccagcgg ctgcggatca cccggcgcga agatcgaccg gggctggaaa cggaacgccc
24721 accggagtcg gtgggcgctg ggggatcgat cggttgtaac ggcggttcgg aatccggcgg
24781 cggaccgaca ccgagctgtt cctccagggc gcgccaatgt cgttcctcgt agcggtccag
24841 cccggcggcc gcggcagcgg cccgggcgta gacgtagcag tccagcgcct cgttgcgctc
24901 cctgagcttc tgccactcgc gccgggcgaa gccgtggcgg tcgcggcggg tcaccagctg
24961 ctcggcgcag agctgctgca ggtactcggc gtccatcctc ggcagatgga tgtagcccgg
25021 cggatagacc agggtggtgc cgtcctcgcc cacctcggcg gtcttacgca acgcgttgta
25081 gaactccagc ttggcgatgc cgaccgccac tgcgaacacc ttgatgccgc ggcgcagttt
25141 ccggccgccc tgggtgaggt ccaccgcggt gggggtgccg atcagggccg cgccgcgggg
25201 gacgcccttg accgccatca cccgaggatc cttgagtcgg cggacgaagg cataggcctc
25261 ctgggtggcg tagccggtgt ccagggcaag acgcgccaag ggcagcaacg ccccgacggc
25321 atggctccag cgttcctcga gcatcgacgc gagctccgcc cataccggtt cccggcccgt
25381 atcgcccatc aaaacccggt gttccaccag ccaggcggac ttgccgcggc cgaaggccca

25441 gatcgagacc tcgatgcgat ccttttgcac gtcggcgccg cccgccaaca acaaaccgcc
25501 gagcggtacc gtgccgatcg tatagtcttc gcggcgttcc agcaggcgtt gccagtccgg
25561 cgtctcgccg tcctcgaccc aggtctcgcc cagctcggtg ttcttgaagg tcttgatcgc
25621 ggacgggcca cccgagaccg ggtgcaccgc actgtcccag gccgcggcga tgtcacgcca
25681 gctgcgccag cctagcgggc tgtacagcga ggacagatga aaaccggcag tcttggcgcc
25741 gtgctcgggc gccaaggccc gccattcacc ctgtgccagc atgtcgccct tgtgatgttc
25801 cggaatcggc cgttcgcagc cttcgcagac gtagccgacc gtctccggcc gcccgcgctc
25861 ccagcgcagc tgctcgaagc gcagccattg gaactggcca cagtgtggac acggcacgaa
25921 atagcggcgc tggtccgagg cctcgtactc gcgctcgatg gcgctggccc ccgagatcgt
25981 cggggtcgag acgatgaaga tcttgcggcg ggcgaaggtg cgggtgcggg cctcggccag
26041 cgcgatggca tcgccttcgc cctcgacgtc cggcggatag ccgtcgacct cgtccaggaa
26101 caggtagcgt accggcatcg agcgcaggcc taccgcgctg ttggccccgg tcatcaccaa
26161 aactccgccg cggaactcct tggccaggat cgtgttgccg gagtcgcggc tgcgggccgg
26221 cgcgatcagc tcgaccaagg acggcgactc ctcgatcagc ggatcgatgc gctggcggga
26281 gttgcgcttg gccatctcca ccgtcggcca caccgccatc atcgggccag gcgcgtgatg
26341 gacacaaaaa ccgatccagt tcgagcccat ctcggtggcg ccgagttgag cccctttcat
26401 gaacaccacc cgctccaccg gcgaggccgg cgacaggcaa tccatgatct cgcgcaggta
26461 cggcgtccgg gcggtgcgcc agcggcccgg ctcggccgag gctttgctgg acagcacccg
26521 gtggcggtcg gcccacttgg acacggtcaa cagcgggtcc ggcgtcagcc cgtcgcgcca
26581 ggcgcgggca atggcggtaa ggccctcctc gtcaccgagc attcagtcga cccgcgggcg
26641 cagttcgccc agttcctgca ggtgttctcg cacggccgtc tccagggcga cgtgcagggt
26701 gtgcggatcg atacccagtt ccgccgcaag cggcgccgag atccgcgcag gccagttcag
26761 ccaggcgtcg cgctcggccc gggccagggt gaacacctgg gcgatggccg cagcgcggtc
26821 gaccagatcg cccttgagtt tggccagccg gaccttgttg gtctgggcct tgaccacctc
26881 gttgaccgtg cgggcctgca gcagggaggt accgccgccg ggcaaggacg gcccgccgtc
26941 gtgggaggcg tcgggcaccg caacccgtac cgcgggttga cgcgtcccgg ccttcggcgc
27001 atccgaattc ctggcccatt cccgatcggc cttgtccgga tcgatgctcc catcggcttc
27061 gggcgtgatg cggccggtcc ggatggcctt gtgcacggcg gtgtcggtga cgccgcggtg
27121 gcgggcgtag gcgcggatcg agagtcccat cggccgcttc gaatggctgt ggattttgct
27181 tggcttgtgt tcggaacagc gcgttcatgg cgtcgtcatc cacgccacca cgagaactac
27241 gccatgacgc cacgcgacgc cttactgacc cggaacgccc agcagcatct cgggatcgat
27301 accctagaaa cccaaaactc ggatcacctc gatttccacg acatggcggt ctggtgcctc
27361 aaagccgcgc tggaggcggc gtaccaggca gggatcgagg ctggacggcg caccaagtcc
27421 gccgccgcca acacttgaac acatcaattg cacgcctggc ggatcccgtc gggcgtccac
27481 tgacagaggg acggcggcga cgctgcccca aacccaagca ctgaggagca tcgacatgaa
27541 cacactctcc ctgaccgaca cccagcgccg gatcctcgac catgcgatcg agcacacggc
27601 ggggcgcatc gactggtttc cggacaacat caagggcggc gcccgccgga aagtcctcga
27661 cggactgctc aagcgaggat tgatcgcatt gaacggcggg gatcattgcg tcaccgccga
27721 aggttttgcc gccgtgggcc gaacgcctcc ggtgccgata tctcccagcg cagaaaccga
27781 aatgcccgcc agcaacgccg aggccgccga ggccaacgcc accgggaagc gaatccaaaa
27841 gagccgcgag gacagcaagc aagccgaagt catccggatg ctgcaccgac ccgaaggcgc
27901 cacgattccg cagatctgcg ccgagaccgg atggcaggcc cacaccgtgc ggggcgtgtt
27961 tgccggcgcc ttcaagaaaa aactcgggct cactctggtg tcggagaagc cggaaggggg
28021 cgatcgcatc tacaagatcg cctgaacgag cgggggcgcg ggaccgcctg cgccccgcag
28081 ccccaccggc gtgatgcgga aagttccttc aaggcacctt tggggctaca ctgcgatttt
28141 gcgctggagg ccgccatgtc cgtcaccgtc aaactgtccg agcacttggt ggagcaggcc
28201 cgtcgctatg ctcacattga gcaccgttcg gtgcccaagc agatcgagca ctggtcgatg
28261 atcggcaaga tcgccgagga gaacccggat ctgccgttta ccctgatccg cgacatcctg
28321 atcgccgatg aggaagaggc tgtcggcgaa tatcacttcg gctgatgcgc ctggaagtta
28381 cgccgacctt cgatcggacg gcgaaaaaac tcaatcggca tcagaagatg gacctcgacg
28441 aggcggtgcg cgccattgcg gacgatccgg aaatcggcgt cgccaaggtc ggcgatctgg
28501 ccggtgttcg ggtctacaaa ttccgcttgt cgaatgcgca gtgtctgttg gcttatcgga
28561 tcctcgcacc ggacaccctc aagctgctca ccttcggtcc gcacgagaat ttctaccgcg

28621 atctcaaacg gctagacgtc tgagtccgat taaaccgcgg tcgcctcgga ggcgagggca
28681 tcgaacgcga gcccatccgc ttcgcgggtg gcttgtttcc ccgtccaatc ctcccatcta
28741 cgcacgatga catcgacgta tttcgggtcc agctcgatca gccgcgccac ccgtcccgac
28801 ttctccgcgg cgatcagcgt ggtgcccgaa ccgccgaagg ggtcgagcac aaggtcgccg
28861 ggccgactgg agttgcggag ggctcgctcc accagttcca ccggcttcat ggtcgggtgc
28921 agatcgttcc gggccggctt gttgatctgc cacacatcgc cttggtcacg atcgccgcac
28981 caatggcgct gggcgcctgc ggcccaaccg tacaggatcg gttcgtactg gtgctggtaa
29041 tctgcacggc ccagggtaaa cgtgttcttg gcccagatga tgaacgtcga ccagtggccg
29101 ccggcggtac ggaaggcaga ctgcagcgta tcgagttcgc tggaggacat agcgatgtag
29161 acggcgccct tgcagtgggc gagcatcgga gtcagcgccg ccagcagaaa gtccgagaag
29221 ccctcgccca ggttgtcgtt caggatcgcg cgatgcttgc cgcgcagctt gtccttggcc
29281 gagttggcgt agttcacatt gtacggcggg tcggtgaaca ccatgtccac agattcgccg
29341 gcaaggaggc ggtcgtagtg ctccgccagg gtggcgtctc cgcacagcag ccgatgggag
29401 cccaggatcc agacatcgcc cggccgagag accggttgca ccggcacgtc gggcgccacg
29461 tcgtcctcgg tctggccctc ggtgactgat gcctcgtcgg ccaaaaggtc cagcaaggcg
29521 tcggcatcga agccggtcaa gcccagatcg aagtcttcgt cctgcagggc cgtcagctcg
29581 agcttgagca aggactcatc ccagcctgcg ttctcggcga tgcggttgtc ggcgatcacc
29641 agggcgcggc gctgggtggg gctcaggtga tccagcacga tcaccggcac ctgggacagg
29701 cccagctggc gggccgccgc ccagcgcccg tgcccggcca cgatcacccc gtccgcgccc
29761 gccaggatcg gcgcggtgaa gccgaactcg gcgatggagg cggcgatctg agcgatctgc
29821 gcgtccgagt gggtgcgggc attgcatgcg taaggcagca acttgtcgac cggccaggac
29881 tcgacccggt cggctaccca ggaatgacgc atgcgttgta cattgaatgc attttgccgg
29941 aagtgcttaa cgccatgacg ctcgaaatca cctacagccg ggcccgcgag caactcgaat
30001 cgctgatgga tcgcaccgtc gatgattgtg aggtgatcat catccgccgc cgttccggtg
30061 gggccgtcgc catgattgcg gccgatgagc tggaaagcct gatggaaacc gcgcatctgc
30121 tgcgttcgcc ccggaacgcc gagcggctgc tggcggcgct ggcgcgcgcc cgggccggtg
30181 aagtcgaacc gaccccgctc ccggcgttga agcaggcgct cggatttgaa aaatgagcgg
30241 ctggcgaata taacaatagc tttcggatat tggttataat ttgttatgcg ccatgaaaac
30301 gatcgtttgg cagccgagag ctctgaagca acttaagaag ttgggcgaca aggcggctca
30361 acagcggata ttgactgcaa cgacagcgct ggagcggttc ccggcagtgg gtggcgtcaa
30421 ggcgctggtc aatcatgcct gtggttaccg gctgcgcgtg ggcgattacc ggattctgtt
30481 caatgtcctg gacgccatcg aggtggtcag catcgaggag gtcaagaaac gcgatgaaag
30541 cacctactga ataccagacc atctaccatg acggacagcc tgctttcgtg ttggtgccgg
30601 tcgccgattt cgaacgggtc cgccccctac tcgaacgccg cagtgtccgt gacgccattc
30661 cccaagcggt ggtggaagcc catgtcctgc acgatatgcc gctgatccgt gcttggcgtg
30721 agtacttggg gctgacgcag gaagacgtcg cgcaacgggc ctgcatgcag caaccggcgt
30781 tggcccgtct ggagcgcggc gacagtacgc cccgtaaggc cacgctgacc aagctcgcca
30841 aggccatggg tctgagcgtg gagcagttgg ccgagtagcg gcttccaaaa gacgatcggc
30901 aggcgatgat catccagccg tcgtctgtat cgcttgccgc tgggaggcaa ccgcttccca
30961 agtttcgccg gtgtctagca gtgtcaccgg gatggccgga aaactctgac ggaaacgcag
31021 gatcgcgaca tccacatacc ccggcgcgat ctccaccgca tacccccggc gcccggtgcg
31081 ctccgccgcc agcaggctgg tccccgaacc gccaaaaggt tcatataccg gatcgtccgg
31141 atccgagtag gcggtcagga caaactccgg cagcgccacg ggaaacaccg ccgggtggtc
31201 gatgtcccgt ccgatcttgc ccttgtggcg catgatgcgg atgacggaat cggggatccg
31261 gcggtcctgg gtcggttgac ccgcatgggt ccaaccgccg acctcaccgt ccttgccacg
31321 cagggcggtg gaactgccgt cctggcggag gtgcgtctcc tgacctgcga acttgcaggc
31381 cacggtcttg ttgggcttgc ggctctgacg gttgaagtgg aacacgaact cgaagctcgg
31441 cgccaagcgt cccgcccagt cgcccggcat ccccggcccc tggtcccaga cgtaccagcc
31501 gaaacggcgc cagccctggg tccgcatcca ccccagccag ggatcccagt aggggatcac
31561 ctcgttgtcg cgatggatca gccccagatt caccaacacc tgggcatcgt cggtcagcgg
31621 taactgggcg aacacgccgc gcatcaggcc gtcccagtcg ccgatgccgc cggaggtgta
31681 gtcgcgctga ttgtcatacg gaggcgaggt aaagcagaga cgggccaact cgccgcccat
31741 caacgccgcg acgacgtccg ggtccgaggc atcgccgcag atcaaccgat gtcggcccaa

31801 ctgccagaca tcgccgggac gcgacacagg agtcgccggc ggctcgggga catcatcggc
31861 cgtctcgggt tcgccgtctt cggcgtcatc cgaatcttcg gtatcgatcg acagcagagc
31921 ttccagctca tcatcgttaa accccgtcag cgccaagtcg aacccggcac cggacaactc
31981 ggccagttcc agcgccagca actcctcgtc ccagccggca tcgagagcca ggcggttgtc
32041 ggccagcaca taggcccgct tctgggccgg cgagaggtga cccagttcga tcaccggcac
32101 ctccgccagc cccaggctgc gggcggccgc caggcggccg tgcccggcga tgatcccctg
32161 tgccccgtcc accaggatcg ggttggtcca gccgaattcc acgatgctgg ccgcgatctt
32221 cgccacctga tccacgctgt gggtgcgcgg attgcgggca taagggatca acttttccag
32281 cggacggtat tcgacctgca acatgaaggg ttcgtagcaa tcccaaaaaa gtacggcccg
32341 gcgcagggtc cgatcggaac ctgcaacgag ccgtagacac gaggtaacgc agcaacaaaa
32401 aacccgccga cggcgaaccg gggcgggtct tgggaaatga ggtaagcacc aagggtgcaa
32461 acctcgatgg cagcacccgg tttgcagtgt ggggtttgca cccaggtttg cactgggggc
32521 gttgggcgct cccgtgtgag cgccagcacc ggcgcggcct ccagggtggt gcgctccggc
32581 tgggggtgca aaccgcaaac cctgcaaacc ttggtttgca gtctgacgct agcgaaatcc
32641 cgcgtgcgtt ccccccgtag ccctcagggg ccgggaagga cccgcgaccg ttcgcggcgg
32701 cggggcgtct cgccccttgg gtcacacctg ctgtactttg agcgctactc tacgcctgcc
32761 gacccccaga tgcgacaggc caaaatcctg ctgttttcgc agccgcccgc agcgttcccc
32821 gatgaccttc aaacgctatc gacttcggtt aagttcccgc atgcagtgga ggcgatcacc
32881 ggctctgatg gccgctcagg cagcctggtt cacagaagaa gttccaagcc cttgcgatcg
32941 atcaggttga gtaacttcaa ggacggaccg ctcggcttct tgtcgccgat ctcccatttt
33001 tgcacggtcg acaggctggt gttgagaagg tttgccagaa cggcttggct gatctggcat
33061 cgctcccgta gcgcacgaat gctgctgctg tcatacaccg ggacggggcg gaggcacagg
33121 acatcgaatt cccgcatctt gcatttgtcg atgaatccca aacggtggag atcatcggcc
33181 gtttcatgga cggcctcgag gattcggcta ttggctttag cggtctgggt catgacatat
33241 ttcctgtaac gtgccgtcca attcggctct ggaacctttc cgcctggatc aattcttagt
33301 caacgccttc aaggaaatcc gcaacagtac ttctaccgtt ttcgaattag gctgcaacga
33361 tcctgaactc cgttcccttg gcgatggggc ggacatgatt tttttcacgc ttcatctcta
33421 cgaggccgta atgagacatc gtcttcagcg ttcgagaaag gttgccgggc ttcctgcccg
33481 tcgctgccgc aagagacgtg atcgattcag gttttgtttc tctgatcacc ttgagcagtg
33541 ccctgttttc atcgctcagg acctcggcaa gcgatctcat cgaagtgaac catatcttgg
33601 gatccccgc tttgggcttg agttcgcccc gcgcgatggc cagcagacgc ttgcgaatgt
33661 cctcttgcgg catgatgcct atcacaatgg ttttcatcgc ttttttacct ccagcagaac
33721 ttggtccact tcagaaaaga agtcagccaa caattgatgt gcatccttga attcatacgg
33781 aacgcccggg tctctggcat gccggtgcat gtggtcgaag gttaaacgtc gacccgcata
33841 cttgaacttc ttcggcggct ttacggcgtg ggcattgtca taaccgagta ttcttttgcc
33901 gtagggctca tgcagtgtca gggagtagcg gatgccatga ggtacatcgg gcgttggcgc
33961 aacctgataa gcctcgatct tgacccaata tccgctgccc tgatcaatga tctggtcctg
34021 taaatccagg agggtttcaa tgccagggtc cacttccatg cacatattat atcagcggct
34081 aataaagtat ccacgcttgg gaagcgctag ccattgagcc ggtccgcgac ctgtcgcaag
34141 gcctgctgcc aacgccgctg ggcggttcgg gtgcagcatg cgaaccgccg ggcgatgtcc
34201 ttccatcccc gtcgctcggc ccgcatccag atcagatggc gctgatcttc ctccagccac
34261 agcacccagc gcatcgtttc cagcatgcga tccaccgcat cggggctcgg ctggacccgg
34321 cactcgcggg tctcgtcggc ataccccctcc caggcctggc gggcgatggc cggccagcag
34381 ttgaaatacc cttgcacctt gaccggcggc aagtcctggg cggtccaggc cgcctcctcg
34441 aagcgggcgg ccacgcgctc aatcgtccac tcagccacgg cgggactccc gtcggccgta
34501 cagccgctcg ccgatctgcc ggatcaactc ccgctcgacg aaatccagcc gctcgtcgat
34561 cagatggacc accaggatgc gctgctcgcg ccagccctcg cgcttgaccg cttcgaggtc
34621 ggtgacctgc ggctgcagct ttcccagcgg gcaacgggga ttagggttcg gtcctctcat
34681 acgcccaccc cttgggtcat ccgtgcccag tgcagcaaag ccagggcgtc ggcttcgttg
34741 tcgtccaccg gatcgaagcc cagtgaccgc atcgcagcga tcatggcggc cttgttggca
34801 ttgccctggc cggtcgcgtg tttcttgatc gttcccaccg gtacgccttg atagggaacc
34861 ccgtggtgct cgcaccaagc ggtgaggtgg gccaggaacc cgccataggc gtgggcggca
34921 tcgacgccgg catggcggcg gacctcctcg aaatagaccg catccagatc gccggcggct

34981 tgcttgattt cggtcagcca gcgcttgaaa cgcaggaagc gcatcccgcc gccttcgaag
35041 cgttggggct tgaaggtttc ggagccgctg atgaccgtac cgtcccgctg atgcagagcc
35101 cagccggtgt gagtacccag atcgagggcc agcagcgtgg tccggtcacc ttcgtcgtta
35161 tctccgagcc cagagaggcc cctacgggac ggggagaggg cgcgtagcgc gcctctcccc
35221 cgtaggggga gggagtctcc gccatctgga attccataaa ttgtttcgta taaacaatcg
35281 gttgcgccag atggcgagtt ggcggcgctg ccatctggaa attccgccat ctggaccgta
35341 agcgattgaa ggatatcgat tctcagatgg cggacgtttg ccatctggat ccagatggca
35401 gatttcaagg ggcagatggc ggagcgtctg ccatctggaa ttagggtcgt cgtcatggat
35461 ggtcctcgtc gtggtagagc cagatcgtgg cgtcttcgac cggcagcaag gcgccggtct
35521 gggggcattt gtagtgggag gggtagaccg aaaccggcca catcgggatt tctccggtgt
35581 caggatccgg gggaccttcc cgccgtagca ccatgccctc gacgcattgg tagccgaact
35641 tgctgcggtg taacggggga agtccgtagt cctcggcgtt acggaagaac ttgatgtaac
35701 cttgagtggc gagtaccgag agccgctcgc ggatcgtgcg ttccgcgccc aatccggcgc
35761 gaccctcgaa ggcttcggcg aactgggtgg cgctgtagca gcgcccatcc gccgcctcgt
35821 cgaacagtat ctgcaggatc acgtcatgct tgcgccggcg ctcggcatcg aggcgttcgg
35881 cgtagtcctg caggacccgt cgcccggtgt tcgccacctc gcgccaagta ccgttgaact
35941 tgtcgacgga cttcaccggg agcgccgcgc cattgcgcag ctcgaagatg agttggcggg
36001 tggtttgggt ctcgtccggc cggaacagca gcatcccggt gctgtagtag cccctgaggc
36061 tgccggctcc ggccagggcc tggaacggat cctcctcgaa gggtttcttg ccgagcttct
36121 tggtgtggtg caccagcacg aggccggcgt ccgggttgac tgccgtgcgc agctgttcca
36181 cccgctgcga caggaagaac agcatcgcgc cgttatcgtt ctcgcccccg gcctcgccgc
36241 cgtcgaacac attgcggatc ggatcgatgg cgatcagatc gggcggagtg ttgccgaacg
36301 cctgaacaat ggccgggatc acctgggcca ggccggcctc gtccaggacc agacgcagtt
36361 gcggggtggc gacgaagttc acccgcgcct cggatatccg gtgcgacggt aaggggattc
36421 ccttcacccg ttcccgcagg tagtggtact gcacctccgc ctgcagatag aacacccgca
36481 agggacgcgg cggtgtcatg ccgaggaagc tggcccccgc cgccatgtgc atcagccagg
36541 ccagcaggaa gtcgctcttg ccgaccttgg gcgccccgcc gaacaccaag aggccgccgg
36601 gcgtgagtac gcgcggcgca atcaggtccg gcgggacggg agagtcatca tcgagcaagg
36661 cgcccaaggt atgcgtcggc aatgccgggg aggcggcctt gatgacccgg cgctcgccgt
36721 gggtgatgaa cgcgacgcaa tcgaagtcct cggacacggc atcggctgcg tcccacttgt
36781 cgggcttgtc cgtcggcggc acgaggatgg ccaccgacgc cgcgcctgcc gccatgcagg
36841 cacgggcggc gttctccgcg tagtcccagc ccggcgcatc ccgatccggc cagatcagca
36901 cggctttgcc ccggagcggc gaccagtcgg tcttgtcgat cggcgccttg gccccgttca
36961 tcgccgtggt ggcggccaca ccctgatgga ccagcgcgtc ggcgcaccgt tcgccctcga
37021 ccagcaccac ctccttcgcc tgggcgatcg ccggcaagtt gtagagcgga cgcgggtcgg
37081 gggcgcgcca caggcgggcg cgcacgtccc acggccggaa ttccttgccg gtgggcgggt
37141 cgtagcggta gacgcaggcg atcagcgtgc cgtccgccgc gacgtagtcc cacttcgccg
37201 tggcggggcc gagccgatcc accggggctt tctcccgcag cggtgtcggt tcgacccgcg
37261 ccgtacccaa cagctgaccg gccagagcca tcacctcggc gaattggccg tgcgtctcca
37321 ggccgtggac agcggcgatc aaggcgaaga gatccccgcc ctcgccggtc gcatggtcgt
37381 accacaaccc ggccttttcg cccgaaagcg ccacttccag gctgtcgccc ggatcgccct
37441 ggacgttgcc gatcgcgaag gcaccccgc gcaccttgcc ggccgggaac aaggtcttca
37501 gcaccgacgg caaccgggcc agcaaatcgg cgcgcagggc ttcgcgctgg agcgtcagat
37561 cggcggggtt ggcaggcctg ggcagcgcgt cgttgaagtc aagcaagtcc tagctccttc
37621 ggcgtgtgct tcactccgac tccctgcgcc gtctcgctcg gcagataccc gcccttgtgc
37681 gcaatcgcac gcacgaacgc cggttccagg ccgaccgcat cgcaccagaa ctccagggcc
37741 gggccggcga ggaagtaccg cgcctcggcg cggagggcgt cgtccggatc gatgcagtcg
37801 ccgatggcca gggtgatcac cgccactgcc aggtgggctt cggggcaggc gaacacgata
37861 tgccggcgca gcagtttgtc gatgaccgtc agacgcacca gaggcctggg cggctgccgc
37921 tgcacccgtt tgtcatcgga aggctgccgg gtcttcatgc cccgcctccc cggcagcgca
37981 ggctccaagc acattgccgg cactcgacgt gggccggatc ggcaaaaccc cgcggcaaca
38041 gctcgccggc ttcggtcgca gtaatgacac gcacggcccg atccgacatc cgctgcgcca
38101 gcccgccgtc gaaggggacg cgctcggcgt agatctccat cgaatccgcg ttgacggcgg

38161 tgaacagcgc cggctgttcg tgcagtgcga ggtaggcctg gtataccgcg atctgggctg
38221 cgtagaccgg cttggctttg gccaagcggt gcttcaccag ctcgcgccag gatttcgcgc
38281 ccaggcattt gttctcccac aaggccggag tatcgaagcg ttcgggcccg ccgacgatga
38341 cgccgtcgac atggccctgc aggtggccgt tcagcgccga gaacccgaac tgcttgccgt
38401 cggcctgatg ggggcagagg ccgaagccag ccgcgcgcag ccagcccact accgcctcct
38461 cgatgcgatg accgcgttcg aagatgcgca acagccgtcc tgaaaactca cggccgggat
38521 cgaccggcgc ctgggcgtat tcgtactgaa gcgcgcgctc gcaggccacg ccgaggcgag
38581 acgcgccgag ataagtgcgg cgtggctcac cggcctggcg acgctgcaga ccggcatcga
38641 tcagcgccgt gagccgttcg ttgaacaagg cgccatggtt gtaatccagc atggtcgccc
38701 cctcagaacg gcacgtcatc gggatcgtgt tcccgcaggt ggtcgaaata ggcggtcagc
38761 accacgtcga ccagttgcag gatttccgcc cggctgtagg ccgacagcgg ccggtccatg
38821 ccgatcgccg cgacgtagtc gccgaggcgg ggcagcacgg cctccatcgc cgccttctcg
38881 ttatgggtgg ggtcgatcac gacgccgtcc ctccgtttca gtcgttgcag atggatgtcc
38941 tggcagcgtc gcgagcagaa gcgcttgaac agtcgcttgc cgcctgagtc gcggggagcg
39001 tcgtgggacg atgcccagca aaagccccgg ccttctcttc cgcagatagc gcaaatcatg
39061 gcgtgcatcc ctgtacgcca cccttcgggc agccgctgcg cggctgtgca caaatccgcc
39121 gggagcggat ttgaacaacc gaaggggtgc ccggagggtg ggccacaggg atgtggccca
39181 tagatgttct gtcctgatca tttgtcaagc cgccctccgt tgggtatcgt gggccgcggc
39241 atctgcgccc agcaccaagg attgaatggc ccgcttgttg aactggaacg aaagcagcgc
39301 ggaggcttgg tagcgcgaca atccgaaatc gctttgccac tggggtggca ggtagcggag
39361 ttgtttctcg gtgggcggtt cgttcagcca gcgacgggtc ttgtgggcgg tgtcgacgga
39421 ctcgtgctcg ttcagccagt cgtcggcctt ggccatgcag acggtgcgtt cgcctacgcc
39481 gaggagatgg gtgggcagac ccttgccgcc gcccaccgcg taccaacggc cggtcaggaa
39541 gaacaccccg ccccaggcac tgaagccggc ggccatcagg gcgtcgtcac taccgaacag
39601 atcgcaccag cggaagtgcg agcgtttcag cagatcgatc tcgctcatga cgaaatcgct
39661 gagcacaccg cggtctttag gctgccgctc ccaaaggtgg ccgcacagcg gacacgccag
39721 acaggctgca ggaacgatcg cgccgcattc ggggcagtct ttggtcggcg cctcgccctg
39781 gcccagatgg ccgtcgagat cgaccgtctg ttcaagggtg ccgtgcatca ggctggcggt
39841 gccgaaatcc agcacgatgc agtcggtctt gatggcaccg ggaaagactt ccgggtccac
39901 cgtgcgcagg ccccggccca ccatctggat gaaggtggac tggtgggagc ttggccgcaa
39961 caacaccaca cagccggtcg gtgggtagtc gtagccttcc gtcagcaccg ccacgttgac
40021 tacgacttgg gccggcccgg actcatagtc ggccaagcgc gccttgcgct cggcgtcgga
40081 caagtcgcca tggatcagca ccgcgtggat cccagcggcg atgaaggcct cgtagacatg
40141 gcgggcatgt gccaccgtcg agcagaacac gatggacttc cgcccggagg ccttgtcctg
40201 ccagtgttgg atgaccgcgt cggtgaccag ctgcttgtcg aagatcgatg ccacctcgtc
40261 catgtcgaag tcgatcgcgg tgcggcggac ctgctgcaag gctgcctgta caccgatgtc
40321 gatgacgaag gtgcggggcg gcaccaagtg gccggaggcg atgagctcgc ccagggtgat
40381 ctggtcggct acgttggaga acacctcgcg caggcctttg ccgtcgcccc ggttcggcgt
40441 cgcggttagg ccacaaagca gggccttcgg attgcgggac cgtacccggt cgatcaccgc
40501 acgatagctg ggcgaggcgg cgtggtgcgc ttcatcgatc accagaagat cgagcgtcgg
40561 catctgctcg agatgtcggc ctcgcgccaa ggtttgcacc atggcgaaag tggccgcacc
40621 ggcccaggat ttctccaggg cgtcgaacac cgaggtggtc agccccggat tgacgcggcc
40681 gaacttctcc cggttctggg cggtgagttc gtcgcggtgg gcgaggatgc aggccttggc
40741 gtccggctcc tccagcacgc cgccggccac cgccgacagc atgatggtct tgcccgagcc
40801 ggtgggaccg atggccaagg tgttgccgtg ctgatgcagc gccgcgagcg agcgctccac
40861 cagcagggtc tgacggggac gaagcatcat ggcgatgtcc tcccttactg ggcccaggcc
40921 ggcttgccgc ctgggacgcc tggggatgta gccggcgtcg aagccgcata ggccggcgct
40981 gccgctgccg gtatcgcacc cgaaggcgaa gttgggaccg gcggggcgaa gtgctggccc
41041 atgagctggg catagtcctt gtgatcaggc tcgatgaccg cccggacggt gttgcggtca
41101 ttgccgcgac cgtctttctc gatgtcgatg cggccggcaa actccagccc gtccagctcg
41161 gcgaagccgg cgatgcggcg ggcgttctgg gcctgtgggc cggtgtcggc gggatggatg
41221 cggcgggcgc tgttcagcag cgaccgaatg aaactgcggc ccatcgcctg ccaggtcggc
41281 cccttgggtg agtgcagacc gatgttccac cacagcttgc ggcgggcgta agggccatcc

41341 agcaccacgc cttcgcatgc gagatagacg gcgccggtct caggactgcg ggtggcccaa
41401 ccaccggtcc agccctggct ggcatcgtcg aacccgcccg gcttgagggt caggcggagc
41461 cgcaccaggg tatccttcgg gatcagctcg aagctcgcct gctcggcgga attgaaatcg
41521 aaatagctca tggtcaggtc tcctgggtgg ggatcgttgc ggggacagga cggctgaaat
41581 ccagccgttc cagcggcgga cgggcggggc cggcgatctt ctgcatcagc tggccgaggt
41641 gcggcggttc gatcggatcg agccggccgg agcggtcttt ggcgggatag ccccaagggt
41701 tcagtgtctg gcagacgaac gcccggtagc ggctgccatc gtccgcaggc agttcggcca
41761 gggtgaccac ttcatcgacg atccccggca gttcgagccc ggtcttggcg ccgtcgatct
41821 gcagcgcgaa cactcggcga ttgaagtcgt cgagcttctc gtccaggatc ccgacgaacc
41881 agacgttctt gttgcgggta tgctgcaaat gcgtcagcca gccgatcatc tcctggccca
41941 tcaggccgta ggcgccgcgg ctgtccggct tgccggtctt ctcggagtag gcctgcggct
42001 gccccttgca ccactgcagg cacaagcgcc cggcgacggt gatcgagtcg acgaacacgg
42061 tctggtatcg ttccagcgca ccggggtcgc cgaagcgcgc gcacaccgcg tcgaagtggg
42121 ctgggctgaa gggctggtca tcccgcaagg ccggattggg gccgccgatg aagaccgcga
42181 agtcgcggca ttcgggccag gtgcgcggcc ggatcgtatc gccggcccag ccttcgaccg
42241 cgaggtcgcc ggcttccagg tcgaagaaca gggtcgaggc ggcttccagc gtccacagtt
42301 gcgaggtctt gccgaggccc gctttgccga ccaggacgcc cttgacgccg cgccgttccg
42361 ccaagcgctg gtcggcgctg atgatgggga gagtcatacc gcgtcctccc cgagcagcgc
42421 caaacggaag ccgggcttgc cggtcctgag cgtacgggcc ggtgcgaagc tctgctggag
42481 gccggccggc caagcgttga acttggtctc ggatacccgg tagccgatct cgacgtattg
42541 ggcagggtcc tcgccgctgt cggcgatccg tcgcaccagg gcggcgagct gtcgggcatc
42601 ccactcgacc ttcttgggga ggtcggcggt gacccgcacc gggccgtcgt cgaagtggac
42661 gatgccggtg tctttgcccg tagccagccg cagttggcgg gcgcgctcgg cgtatctgat
42721 ctcgagggcg cggtccaagt gttcgttgag ggtcttggcg gcggccagtt gcgcggcggc
42781 gtcgtgcttg aaccggcaca gcgattccgc cggcaactcg gcgagctggc cggccggagt
42841 ggcgaggagg atgtcggggt tcatcgaggt catgccgcac ctccggcctg aacgcattgg
42901 ccgggacttc tccggaggtg gcgggtctcg taggcctcga tgtcttcctg acgatagagc
42961 acccggccgt gcagcttcag atagaccggg ccgatgcctt cggaccgcca gcgttccagc
43021 gtggcttcgc tgacgcccca acgatccgcc agttggcgtt gattcagatg tcttacgttc
43081 acgagtcgct cctgtgggtt gttgcggaaa cgtgaacaaa ttctgggctt cagggggtgg
43141 gcaaagcggg gggcaaaacg ggcggcaggg gtgggcaaaa cctgcaattc gaatttcagg
43201 cgacagaaac gaaaaagccc ggagggatgc tccgggccgg ttcgatcgat aatgcgatta
43261 cggcgaaatc atccgggtgg gggaaaagga tcgttgccgt agctgttgcg ctcgcggatc
43321 ttcccatcct cgccttggat gatgacttca ctgctctgat tgcgcgcgat gtctcgtgcg
43381 cgttcgatgg cttcgtgttg ggtttcgtgc ttcgaggtca atcggctatt gccttcgccc
43441 cggacgcccc agccgtcgcc gttcttgacg acccattggt tcttgctcat cggtttacct
43501 catgtcgagt acgctgtggg aaatgtcgga taaatatgga attaggatga ttacggtcat
43561 tcacctcctc gtcggctcag gaactcgggt ttgagccagt agcggccctc ggtgtcatgt
43621 tcgatgaagg ttcggtagac cgctttgtgc cgcttgaaga tctcggaccc tttgcaggca
43681 tcgacatcca tttccagtgc atcggcgagc actcttttgt gcacgggctt gccgtctgcg
43741 tcgatcaaaa tgctgagaaa tcggtagatc tggggtgaga ctttgatctg tttgccgtcg
43801 atcaaggcga gccggccgga acgcagcagc cgcaacgaag tctcggcaac gtcctcatca
43861 gcaatcaggg gcgcgtcaag ataggactcc aagttttcga cgacgaagcc ggctttgcgc
43921 agatgtgaca cggcgcgaag gggtacgagc cgaccgttcg cgagcggtgg cgcgatcgaa
43981 tcctgcgggg tggtcgtaat caggatctcg gtccccggcg cggcgacggc acggatgccg
44041 gcgtcgatcg tttgcgcatc gctcgactcg ttcagccgcc tgccgaaaaa caccgtgcgg
44101 cgtttccgcc gatgttcgat gtcacccaaa cgccaaagcc gggccggtac gcgttcctcg
44161 agtcgaaagc gtccctgcag gccgagcgcg gaggcgatcc agcggacgat gcgttgatac
44221 tcgacccgca ggggtttcac ctggtgggtg gcaagattca gccagccaca atcgctgcaa
44281 tagccgcgat agccttcttc ggagaaactg agcgaacaca gttcgtcgtc tccacaccaa
44341 gggcagagaa cggaacccgc aaggcccgta cccacttgca gggcttggag ttcgtgcagg
44401 tgctcgtagg cttcgcgcct accggaacat cggacggtat cggggtggat atccgcatgg
44461 ggcgcctcga tcagctcgca aagcaggccg aaggcgatgt cgttgcaggc cggcatcggt

44521 cacctaggcg gccagcttgg cttcgaattc gctcggttcg gtgacctgcc acgcgcgtag
44581 cagggcgtcg gcgagttgcg cgtcgttctc gctcatgtcg cgcaggttgg acacgccgga
44641 ctgtttcagc acgatgttca ggacatggcc gatttttccc ggttcattcg ggacgaagta
44701 gacgctgatc accacttcga ccaagttgaa ggggcctcgg aagaagtctc tctcctgcaa
44761 gtgcgtactg gatgccgtga atgcgcaagc ttcgttcgga tccgctggcg tctcgaccca
44821 gaaatcgcag tgcgggggga tcgtggcacg tactcggact tgccgcagcc ggattcgatc
44881 cacgccatgg gccgccagat cgatggcatc gccttcgaca aacgcgagtc caaaacgcag
44941 acggttgaga tggaacatcg gctgcttgac agcctctggt ttgacgtcgc ggttgaggag
45001 atgttttgcg aacagggtga cgagggcgac atggatccgt gcgccgcctc gaccgacact
45061 gtcgacgatc ccggagatcg ggtaatacac cagcgccaga ttgcccgccg gtcgggtggt
45121 ccggcgtttc atgccttcct cgacgaattc caccagatcg ttcgggtcgt cctcgatgta
45181 gatgttgacc tgaacgccgc catcgaggca ccgctcgcag acatcgatcc ggcacgcccg
45241 tcgggggcct ttccggcgcg acatcaacgt cgacagcgcc gtttccaacg cccggatatt
45301 ctcttcctct ttagatacgg gttcgctgac tttgatggcc tgccgtttcc aggcccgggt
45361 gccggcactc agatcgaatt gcaacaaccg ctcgccgatc tgaaacaccg ccggccaatg
45421 cacccaggtc caaagggcgc gctcggcgtg gttgtgcaat tgttcgaaac cttcctgaat
45481 ctcggactgt ccatgccctg cgttgagcag cgcgtagatg cctttgcgct gggccagtcg
45541 gtgaaccctg cgcatttccg cgtggaccgc cgcgcgttgt tcactgtcct cgagactctc
45601 gaagcgggca atcagggcat ttgccagcgc ggtttcttcc gcctcccaat cgaaatcgtc
45661 accgacctcg atagaccgct ttgccaaata gtcgcgccag gtttttccgg ggatctcccg
45721 gatgaggtga cgaagattga atgcagccaa gatgatttct cctgtactga agaaaaaacg
45781 aggaatgagc ggcttgagcc acgaacactg accaaatggg acggataggt tcggtacacc
45841 gaacgatgcg aattattgcg ggctgctgaa tgttctgtca agccgatacg aattcgttcg
45901 gcacggtgct atattcctaa ggtcgcctgc ggtgacatga atagacctga cgagaggaga
45961 aacaccgtgc catcgccctt gggtgagaaa atccgggggt tgcgcaagca gaagaaactg
46021 agcctggatc agcttgccga cctaaccgaa tccagcaaga gctatctttg ggagctcgaa
46081 aacaaggagg ctcccaaccc gtctgcagaa aagatcgcca gaatcgccgc cgtgttggag
46141 gtcacgacgg agttcttgat gaatgaccag gaaatgacgc ccgacgcggc agtcgcggat
46201 gaggcctttt tccggaagta caagaagatg ccggaggaaa ccaagaagaa gctgcggcaa
46261 ctcatcgatg tctgggacga cgatccatga ccgagcgcaa gtggccaacg gccgaggcca
46321 atcggctcaa cgtgatgctc gggcaggtac tggggcgcga acggtttccg gtcgatgtgg
46381 aggcactggc gctggagtat tcgaaacagt gcttcccgca cgcaccgatc acccagatca
46441 agggcgccga tctaccgggg ttcgaaggca tgctagcggc ccatcccagc aagacccaat
46501 ggaaaatcgt ctacaactct gcagtccggt ccagggggcg gatccgtttt acgctggctc
46561 atgaattcgg ccattacctg ctgcaccgcg accggcagga gatcttcagc tgcagccaac
46621 aggacatgga ggaatgggat gccgaagagc gacagctcga aaccgaagcg gacacgttcg
46681 cgtcctacct cctgatgcct ttggacgact tccggcagca gatcggcaag gagcgtgttt
46741 ccttcgagct gctgggccac tgcgccgagc gctacggcgt ttcgctgacg gcagctgcct
46801 tgaaatggat cgagatcgcc gaaaaccggg cggtgctcgt cgccgtccgt gatgatcatt
46861 tgttgtgggc tcgatccaat caggccgcat tcaagtcggg tgcggtattc gccactcgca
46921 agcgtaccta tgccgttccc cccgagtccc tggtccatgg tcgcaatggc gacattccga
46981 tacaggcagg gagcctctcg gccaatacct ggtttcccaa ggagccccgg gatatgccat
47041 tgacggagtt gatcttcgtc aacgagcact acgactacac gctcgccctc ctgctgatgc
47101 cgaaggccga gctgcgctgg cagggtgaag aggacgagga cgacgatccg gccgagcgga
47161 tggattcggc catccgccag ggacgattca ggcgatagca ggttctttat cacagcaagt
47221 tctggtgtcg aaacctgggc tgccaggtcg tcgaaagcac cctccaaagc ggtttccgag
47281 cggaaacatt cgccccgttt ttgcaatggc ctgcaacgac ccgtagacct tcgaaatctc
47341 gggatggtgc ggcgatcggg ttgtgacgga gactggtagg catccctttc gtgccaaacg
47401 attgttgagt tgcctatgac gatgccatcc ctcacaccgc ccgaacgtct ctcctccgct
47461 caacgcgtcc acgagatcac cacgatcctg gccgccgcga tccttcgtac ctacctgatc
47521 gaacccgaga aaaagaccgg gttggacctt ggcttactgg ccgggaagcg cgtccatacc
47581 actccctctc aaccggagcg tgtgtgatga acgacctcaa actctcggtg gcggcccaag
47641 tagcctcgct cccgaccttg ccgatcaaag acctctggac gttgtgggat caatatttcc

47701 cgcgccgtcc gacccatccc aatcggaact acctggaatc ccgcatcgcc tacaagattc
47761 aggaagccgc ctacggcggc ctcgcgcccg agacccgtcg acgcctggag caggtcggcc
47821 agcgccactc gaagatcaag tcccggcggg tgtcgccggc gattcacctc ccgcccggca
47881 ccgtgctggt gcgggaatgg ggcgagcagg accacaaggt caccgtcacc gccgaagggc
47941 ggttcgacta tgccggccag agcttcaaga gcctgaccgc ggtagcccgt cacatcaccg
48001 gcaccgcctg gtccggcccg ctcttcttcg gcctgcgcca agccggggag gggacatgag
48061 ggcatcgaac cgtatggaag tgcaaaccgg cgccccgtcc aagccccgcc aacgctgtgc
48121 cgtctactgc cgggtgtcct ccgatgaacg gttggaccag gagttcaact ccatcgacgc
48181 ccagaaagag gccggccacg cctacatcgc cagccagcgg atcgaaggct ggatcccggt
48241 cgctgacgac tacgacgacc ccggctactc cggcggcaac accgagcggc cggctttgcg
48301 gcgcttgctg gccgacattg aggccggccg gatcgacatc gtggtggtgt acaagatcga
48361 ccggctgacc cggagcttgg cggatttctc acgcatggtc gaagtgttcg agcgccaggg
48421 cgtgtccttc gtctcggtga cacaacagtt caacactacc acctcgatgg gacggctgat
48481 gctgaacgtg ttgttgtcct tcgcccagtt cgaacgcgaa gtcaccggcg agcgcatccg
48541 cgacaagatc gccgcggcca agcgcaaggg gttatggatg ggcggggtgc cgcccttggg
48601 ctacgacgtc gccaaccggc agttggtcgt caacgacagg gaagcggccc tcgtcaaacg
48661 gattttccag gacatgctca gcgtcggctc caccacccgg atcgccgcgg cgctgaatgc
48721 cgaaggcatc accaccaagg cctggatcac ccaggacggc agacatcgcc ccggcgcccg
48781 gatcgacaag aaacacctgc accacctgct gcgcaaccgg atctatctcg gcgaaatctc
48841 ccacaagggc agttggcata gcggcgccca cccggcgatc atcgaagcca ctttgtggga
48901 cgctgtccac gccgtcctgg cccgggatgc ccgcagccgc gccaccgaaa cccggcaacg
48961 ggaacggacc gacgcgctcc tgcgcggtct gctctacgac gccgagggcg aaaagatgta
49021 cccgacctac gtgcggaaaa acggccgcca ataccgctat tacttctcca aggccgaagc
49081 gcgtttcggc gccggacaca agaccagcgt tcggctgccg gccgaagaaa tcgaagcggc
49141 caccctcgcc cagatccgga ccgtcctggc cagtcccgaa gccattgcgg ccatatggca
49201 ggcggtgcag gcgcaaacgg ccgacctcga cgaagcccag gtcgtggttg ccctgggaca
49261 gctcggtgct gtctgggagc aactgttccc ggccgagcgg caccgcatcg ttcagctcat
49321 gatcgagcgg gtcgaactcg ccgacggcgg tctcaggatc cgatggcggg ctttgggttg
49381 gaaagaactg ctcagcgaat tcgcgcccag gacgataggg gccgagttgg tagagatgga
49441 gacggcagca tgaaaaccga cgcctgggaa tccttcgtgc cgctgacctt ccgacgacgt
49501 ggagtgcaac ggctggtagc gacggccgcc ccggcgcatg atctcccgtt cctggtcggc
49561 ctgggccggg cgttgtactg gcagcatctc ctcgacaccg gtgtcgtcca cagcgggtcc
49621 gacatcgccc gccaggaaga cttgcacccc tccacggtga acgaactgct gcgcctcacc
49681 ctgttggcgc ccgacctcat cgaacggctg atggccggac gacaaccccg gcccctgacc
49741 ttgatgtggt tccagcggca tccactgccg gtcgattggg cagagcagcg ggatctcatg
49801 gtgagtttcg agtagaggcg acgatggcga agaaagaccg cgggatcatc attggcaaac
49861 cccgcaccgt atcccttccc cagccggcag gcggggttca gttggaaacc tttctgccct
49921 ggacgctggt gaagcgaggg ctgaaacggg agatcctgac gcccttggga actccggcgg
49981 cgtttcagga agaagccaag cgggaagtgg aaaagcgtag aggcgagcag gacacgccgc
50041 tcatccgagc actcggcctc gcgcattact ggcagcggtt attggatgag gggaagttcg
50101 aatcgttgtc agaactcgcg gcggcggagg gcatcgatat agcgcaggtc agccggattg
50161 cgcggctggt gcgcttggcg ccgggaatcg tggaggcttg cgtagccgag atggctccgg
50221 ttctgacgct ggaagacttg aatcggcggg cgaaatctgt ccactgggat gtgcggcagc
50281 gtgtttagat ggcttgacaa ggagcgacag ttacggcggc gagcgggttg tcctggctga
50341 tgtagtcgat gatgtcggcg cggtcttgct ctgcagcagc ggcccagaag accctcattc
50401 gccagcacgc cctgccttgg ccagcaactg cgcacgtcgt gcagcgaacc gggcctccac
50461 atcgtcagcg ctggcgcact ctcctgcacg gatctgttcc cgagcatcgg caaccttacc
50521 gcgcagaaac tcgtcgtact cccgcgcctc gcgctggcgt tgcacgaact cgcgcatcaa
50581 ttcacgtagg atctgtgagg cgggtcgatg gctggccttt gcctcggcca tgaactcggc
50641 acgtagctct ggctccagct tcatggtgaa aatagcttct ttggacatag cggaacctcc
50701 ggcacttgac gtagtaatga agaatatacg ttgtaagtat gtctgtttgt ctcggaagtg
50761 acccatcacc acacatcact ccctgccgat ggattatttc gcccgccact tggctaacag
50821 ccgggcctgc agcgctttgc attccggctt caatgggcta cggcgggtgg cgatgcgttc

50881 cgccatctca cgttggcggc gcggatcgaa ccagtgtttt ccatccttgg ccatatccca
50941 agtgctcgac acggcttggc gatgcaccgg caacggatcg ccgtctggac cgagggatgc
51001 cagttgatcg cgttcgcgcg agcgccagcg cttgtgccac aggcgtttgt catcggcttc
51061 cgagcgggcg gtggtgtggc cgaaaatcgg tgtcttgcgg cggctgcgac tcatggcgca
51121 tcgtcccagc cgtaccagcc atccatgaaa tcaccggctt ctgcgccggc cacacggggg
51181 tggcgccgca gccaatcgag cacggcctgc cggtcctctt cggtgggcga accacgtccc
51241 cacgcggaca cgatgccatc tgtttccatc aagggaagtt ggccgcccag cccaccaacg
51301 gcgaggtggc gcgactcgat cagcgcgatg aacccatcca gaaaatcatc gtgggcggca
51361 tcatccatgg ggttgtggaa tcgcattcgg acctcaaaca cccgttcctg gaactcgccc
51421 acgcgcagct ttttgcgttg ccggcgattc atgcgcagaa gttttccggc agaaggcaag
51481 cggttcaaca ttcgggtgtc ctccgggctt ccggctgcag tacggtgtcc agcagcaacc
51541 ggtccaaaat cgaaaagtca gtgtcgcgcg gcaagggtgg caacaccgat tcgagccggg
51601 tcagttcgtc atcgatgaaa gcattgatga tcggcagtgg ctggccgtat tccgactcca
51661 gcgccgcgcg cttgatggcc agcaactggg cgatggcctc gcgcaaggct ggatcggtca
51721 cgatggcatc gaccagatcc tgaaaccgca tcggcgccgc accgcgtcct tgctcgatcc
51781 acagcgtggc cagcagtggg cgcagcacgt acagatattt cttcaggcgc acagtgtcgc
51841 cgcgcaggta ctcgcggtag ttcttacgcg ccatgtgcac gtaatggtgg aaagagcgtt
51901 cggcctgatg ggtctggcgg gccgcctccc gcatcgcgtg cagaaagtcc gtttcagccc
51961 ggtacaccac cggcgagtcc agccactcga tcagcgtggc gttccccttt ttcaacaggc
52021 ccaacgcctt gcgcagttcc cagccgttga tgtccagctc gtcagaaatc ggcaccccga
52081 tcacatcgcg ctgcgcactg acctgcaagt accagggcag tgaatgaacg tagatgaagc
52141 ggacatcgta atcgctgtcg ggcgaggcaa aaccccatcc ccggctgccg gactcgcagg
52201 catagagtac gcgcacgccg tgttcggttt cgatggccga tagacgggcc tggatgtcgg
52261 cacggagggc tggggctatg ggatgcgaat cgaccatatc tattaatccg gcccgaattg
52321 agcgtacaag ccaacgcacg ccggctgcgg cttacggtag gttcggcgta caaataaacg
52381 gggccggagt aatagcagcg cgatcaggcg agcgttttcg gcctgaaggg ccgcaacgga
52441 aaacgtgtca gtcat
//
