# Supplementary material for: Identification and comparative genomic analysis of prophage sequences and CRISPR‒Cas immunity in Methylococcus genomes: insights into industrial methane bioconversion
Source: Biotechnol Biofuels Bioprod. 2026 Jan 29;19:21. doi: 10.1186/s13068-026-02738-6 (PMC12924245; doi:10.1186/s13068-026-02738-6)
Supplement: Supplementary file 3 — Supplementary Material 3. [file 13068_2026_2738_MOESM3_ESM.pdf]

spacer	phage	strain	CRISPR_type	value	col4	col5	start	end	PAM_3_5	PAM_5_3	ID_0	transl_table_0	phrog_0	top_hit_0	locus_tag_0	function_0	product_0	source_0	score_0	phase_0	phase_1
>spacer1_Bath_2	16-5-R1	Bath	CAS-TypeIE	1,442E-07	2	31	8511	8482	-|-	"-|TTGGGT "											
>spacer23_IO1_2	16-5-R1	IO1	CAS-TypeIE	2.25e-12	31	2	8118	8089	-|-	"TCG|- "	CZTTGXGG_CDS_0012	11.0	22642	No_MMseqs_PHROG_hit	CZTTGXGG_CDS_0012			PHANOTATE_1.5.1	-	207.24898531040887	0.0
>spacer25_IO1_2	16-5-R1	IO1	CAS-TypeIE	2.25e-12	31	2	8118	8089	-|-	"TCG|- "	CZTTGXGG_CDS_0012	11.0	22642	No_MMseqs_PHROG_hit	CZTTGXGG_CDS_0012			PHANOTATE_1.5.1	-	207.24898531040887	0.0
>spacer40_IO1_2	16-5-R1	IO1	CAS-TypeIE	6,773E-02	30	4	2288	2314	-|-	"-|- "	CZTTGXGG_CDS_0003	11.0	1159	No_MMseqs_PHROG_hit	CZTTGXGG_CDS_0003			PHANOTATE_1.5.1	-	508205.8262518327	0.0
>spacer56_IO1_2	16-5-R1	IO1	CAS-TypeIE	4,247E-11	1	33	7948	7916	-|-	"-|- "	CZTTGXGG_CDS_0012	11.0	22642	No_MMseqs_PHROG_hit	CZTTGXGG_CDS_0012			PHANOTATE_1.5.1	-	207.24898531040887	0.0
>spacer10_KN2_5	16-5-R1	KN2	CAS-TypeIC	2,889E-09	36	1	1828	1793	CTC|-	"-|- "	CZTTGXGG_CDS_0003	11.0	1159	No_MMseqs_PHROG_hit	CZTTGXGG_CDS_0003			PHANOTATE_1.5.1	-	508205.8262518327	0.0
>spacer11_KN2_5	16-5-R1	KN2	CAS-TypeIC	3,372E-03	32	3	2290	2261	TTG|-	"-|- "	CZTTGXGG_CDS_0003	11.0	1159	No_MMseqs_PHROG_hit	CZTTGXGG_CDS_0003			PHANOTATE_1.5.1	-	508205.8262518327	0.0
>spacer15_KN2_5	16-5-R1	KN2	CAS-TypeIC	2,045E-03	33	1	1777	1745	TTC|-	"-|- "	CZTTGXGG_CDS_0003	11.0	1159	No_MMseqs_PHROG_hit	CZTTGXGG_CDS_0003			PHANOTATE_1.5.1	-	508205.8262518327	0.0
>spacer3_KN2_6	16-5-R1	KN2	CAS-TypeIE	0.0006381	33	1	7414	7382	-|-	"-|- "	CZTTGXGG_CDS_0012	11.0	22642	No_MMseqs_PHROG_hit	CZTTGXGG_CDS_0012			PHANOTATE_1.5.1	-	207.24898531040887	0.0
>spacer31_BH_1	16-5-R1	BH	CAS-TypeIE	4,247E-11	1	33	7948	7916	-|-	"-|- "	CZTTGXGG_CDS_0012	11.0	22642	No_MMseqs_PHROG_hit	CZTTGXGG_CDS_0012			PHANOTATE_1.5.1	-	207.24898531040887	0.0
>spacer48_BH_1	16-5-R1	BH	CAS-TypeIE	6,773E-02	30	4	2288	2314	-|-	"-|- "	CZTTGXGG_CDS_0003	11.0	1159	No_MMseqs_PHROG_hit	CZTTGXGG_CDS_0003			PHANOTATE_1.5.1	-	508205.8262518327	0.0
>spacer74_BH_1	16-5-R1	BH	CAS-TypeIE	1,467E-02	33	1	1822	1790	-|-	"-|-											

" CZTTGXGG_CDS_0003 11.0 1159 No_MMseqs_PHROG_hit
CZTTGXGG_CDS_0003 PHANOTATE_1.5.1 -
508205.8262518327 0.0
>spacer78_BH_1 16-5-R1 BH CAS-TypeIE 2.25e-12 31 2 8118
8089 -|- "TCG|-
" CZTTGXGG_CDS_0012 11.0 22642 No_MMseqs_PHROG_hit
CZTTGXGG_CDS_0012 PHANOTATE_1.5.1 -
207.24898531040887 0.0
>spacer86_BH_1 16-5-R1 BH CAS-TypeIE 2.25e-12 31 2 8118
8089 -|- "TCG|-
" CZTTGXGG_CDS_0012 11.0 22642 No_MMseqs_PHROG_hit
CZTTGXGG_CDS_0012 PHANOTATE_1.5.1 -
207.24898531040887 0.0
>spacer26_McNor_1 16-5-R1 McNor CAS-TypeIE 0.0006381 33 1
7414 7382 -|- "-|-
" CZTTGXGG_CDS_0012 11.0 22642 No_MMseqs_PHROG_hit
CZTTGXGG_CDS_0012 PHANOTATE_1.5.1 -
207.24898531040887 0.0
>spacer12_MIR_1 16-5-R1 MIR CAS-TypeIE 4,247E-11 1 33 7948
7916 -|- "-|-
" CZTTGXGG_CDS_0012 11.0 22642 No_MMseqs_PHROG_hit
CZTTGXGG_CDS_0012 PHANOTATE_1.5.1 -
207.24898531040887 0.0
>spacer53_MIR_1 16-5-R1 MIR CAS-TypeIE 1,467E-02 33 1 1822
1790 -|- "-|-
" CZTTGXGG_CDS_0003 11.0 1159 No_MMseqs_PHROG_hit
CZTTGXGG_CDS_0003 PHANOTATE_1.5.1 -
508205.8262518327 0.0
>spacer57_MIR_1 16-5-R1 MIR CAS-TypeIE 2.25e-12 31 2 8118
8089 -|- "TCG|-
" CZTTGXGG_CDS_0012 11.0 22642 No_MMseqs_PHROG_hit
CZTTGXGG_CDS_0012 PHANOTATE_1.5.1 -
207.24898531040887 0.0
>spacer3_Mc7_1 16-5-R1 Mc7 CAS-TypeIE 8,211E-03 32 3 7258
7229 -|- "CC|-
" CZTTGXGG_CDS_0012 11.0 22642 No_MMseqs_PHROG_hit
CZTTGXGG_CDS_0012 PHANOTATE_1.5.1 -
207.24898531040887 0.0
>spacer14_Mc7_1 16-5-R2 Mc7 CAS-TypeIE 2.25e-12 3 32 13549
13578 -|- "-|-
" QRZSKLUB_CDS_0023 11.0 846 No_MMseqs_PHROG_hit
QRZSKLUB_CDS_0023 PHANOTATE_1.5.1 -
869.2007036254149 0.0
>spacer31_Bath_1 16-5-R2 Bath CAS-TypeIC 8.07e-07 4 33 15950
15921 TTC|- "-|-
" QRZSKLUB_CDS_0028 11.0 296 No_MMseqs_PHROG_hit
QRZSKLUB_CDS_0028 integration and excision DNA transposition
protein PHANOTATE_1.5.1 -207324.631869356 0.0
>spacer3_McNor_2 16-5-R2 McNor CAS-TypeIC 0.0009829 33 1 1675
1643 -|- "-|-
" QRZSKLUB_CDS_0004 11.0 26 No_MMseqs_PHROG_hit
QRZSKLUB_CDS_0004 connector tail completion or Neck1 protein
PHANOTATE_1.5.1 -361.5967395615029 0.0 0.0
>spacer4_McNor_2 16-5-R2 McNor CAS-TypeIC 8.07e-07 33 4 27441
27470 -|- "-|-
" QRZSKLUB_CDS_0049 11.0 976 No_MMseqs_PHROG_hit
QRZSKLUB_CDS_0049 head and packaging head maturation
protease PHANOTATE_1.5.1 -2646.329872579589 0.0
>spacer5_McNor_2 16-5-R2 McNor CAS-TypeIC 2.02e-05 3 32 28806
28835 ATG|- "-|-

"	QRZSKLUB_CDS_0051	11.0	371	No_MMseqs_PHROG_hit
	QRZSKLUB_CDS_0051	head and packaging	major head protein
	PHANOTATE_1.5.1	-2794.187567634167	0.0
>spacer9_McNor_2	16-5-R2	McNor	CAS-TypeIC	2,694E-09	2	34	28473
	28505	-|-	"-|-
"	QRZSKLUB_CDS_0051	11.0	371	No_MMseqs_PHROG_hit
	QRZSKLUB_CDS_0051	head and packaging	major head protein
	PHANOTATE_1.5.1	-2794.187567634167	0.0
>spacer13_McNor_2	16-5-R2	McNor	CAS-TypeIC	7,194E-06	1	36
	29080	29115	-|-	"CC|-
"	QRZSKLUB_CDS_0052	11.0	6188	No_MMseqs_PHROG_hit
	QRZSKLUB_CDS_0052		PHANOTATE_1.5.1	-
3.437535752093949	0.0
>spacer14_McNor_2	16-5-R2	McNor	CAS-TypeIC	8,111E-06	1	33
	28119	28151	ATG|-	"-|-
"	QRZSKLUB_CDS_0051	11.0	371	No_MMseqs_PHROG_hit
	QRZSKLUB_CDS_0051	head and packaging	major head protein
	PHANOTATE_1.5.1	-2794.187567634167	0.0
>spacer15_McNor_2	16-5-R2	McNor	CAS-TypeIC	3,264E-02	4	33
	28659	28688	-|-	"-|-
"	QRZSKLUB_CDS_0051	11.0	371	No_MMseqs_PHROG_hit
	QRZSKLUB_CDS_0051	head and packaging	major head protein
	PHANOTATE_1.5.1	-2794.187567634167	0.0
>spacer16_McNor_2	16-5-R2	McNor	CAS-TypeIC	4,247E-11	2	34
	28277	28309	CCG|-	"-|CGG
"	QRZSKLUB_CDS_0051	11.0	371	No_MMseqs_PHROG_hit
	QRZSKLUB_CDS_0051	head and packaging	major head protein
	PHANOTATE_1.5.1	-2794.187567634167	0.0
>spacer20_McNor_2	16-5-R2	McNor	CAS-TypeIC	8,358E-06	3	32
	27458	27487	-|-	"-|-
"	QRZSKLUB_CDS_0049	11.0	976	No_MMseqs_PHROG_hit
	QRZSKLUB_CDS_0049	head and packaging	head maturation
protease	PHANOTATE_1.5.1	-2646.329872579589	0.0
>spacer12_Mc7_7	16-5-R2	Mc7	CAS-TypeIF_1	2.25e-12	1	30
	32831	32860	-|-	"-|-
"	QRZSKLUB_CDS_0059	11.0	339	No_MMseqs_PHROG_hit
	QRZSKLUB_CDS_0059	tail	tail length tape measure protein
	PHANOTATE_1.5.1	-4.47353219438996e+24	0.0
>spacer17_Mc7_2	16-5-R2	Mc7	CAS-TypeIC	5.76e-05	3	35	41833
	41865	-|-	"-|CGG
"	QRZSKLUB_CDS_0061	11.0	8917	No_MMseqs_PHROG_hit
	QRZSKLUB_CDS_0061		PHANOTATE_1.5.1	-
2974267.6967246355	0.0
>spacer20_Mc7_2	16-5-R2	Mc7	CAS-TypeIC	3,717E-02	2	34	1935
	1903	-|-	"-|-
"	QRZSKLUB_CDS_0005	11.0	No_PHROGs_HMM	No_MMseqs_PHROG_hit
	QRZSKLUB_CDS_0005		PHANOTATE_1.5.1	-
9.950940371504291	0.0
>spacer21_Mc7_2	16-5-R2	Mc7	CAS-TypeIC	8.07e-07	1	30	32782
	32811	-|-	"-|-
"	QRZSKLUB_CDS_0059	11.0	339	No_MMseqs_PHROG_hit
	QRZSKLUB_CDS_0059	tail	tail length tape measure protein
	PHANOTATE_1.5.1	-4.47353219438996e+24	0.0
>spacer27_Mc7_2	16-5-R2	Mc7	CAS-TypeIC	8,111E-06	2	34	41635
	41667	-|-	"-|-
"	QRZSKLUB_CDS_0061	11.0	8917	No_MMseqs_PHROG_hit
	QRZSKLUB_CDS_0061		PHANOTATE_1.5.1	-
2974267.6967246355	0.0
>spacer29_Mc7_2	16-5-R2	Mc7	CAS-TypeIC	3.86e-08	34	8	34035
	34009	-|-	"-|-

" QRZSKLUB_CDS_0059 11.0 339 No_MMseqs_PHROG_hit
QRZSKLUB_CDS_0059 tail tail length tape measure protein
PHANOTATE_1.5.1 -4.47353219438996e+24 0.0
>spacer31_Mc7_2 16-5-R2 Mc7 CAS-TypeIC 2,694E-09 2 34 11561
11529 -|- "-|GAGAAT
" QRZSKLUB_CDS_0019 11.0 836 No_MMseqs_PHROG_hit
QRZSKLUB_CDS_0019 transcription regulation late
transcriptional activator PHANOTATE_1.5.1 -42.78768673401984 0.0
>spacer33_Mc7_2 16-5-R2 Mc7 CAS-TypeIC 1,562E-07 2 34 34777
34809 -|- "-|TGG
" QRZSKLUB_CDS_0059 11.0 339 No_MMseqs_PHROG_hit
QRZSKLUB_CDS_0059 tail tail length tape measure protein
PHANOTATE_1.5.1 -4.47353219438996e+24 0.0
>spacer12_Mc7_7 McNor-R1 Mc7 CAS-TypeIF_1 2.25e-12 3 32
12494 12523 -|- "-|-
" LFPVWAMN_CDS_0023 11.0 339 No_MMseqs_PHROG_hit
LFPVWAMN_CDS_0023 tail tail length tape measure protein
PHANOTATE_1.5.1 -4.653279167529952e+23 0.0
>spacer21_Mc7_2 McNor-R1 Mc7 CAS-TypeIC 6,599E-02 1 30 12443
12472 -|- "-|-
" LFPVWAMN_CDS_0023 11.0 339 No_MMseqs_PHROG_hit
LFPVWAMN_CDS_0023 tail tail length tape measure protein
PHANOTATE_1.5.1 -4.653279167529952e+23 0.0
>spacer29_Mc7_2 McNor-R1 Mc7 CAS-TypeIC 3,812E-04 33 1 13650
13618 -|- "-|-
" LFPVWAMN_CDS_0023 11.0 339 No_MMseqs_PHROG_hit
LFPVWAMN_CDS_0023 tail tail length tape measure protein
PHANOTATE_1.5.1 -4.653279167529952e+23 0.0
>spacer33_Mc7_2 McNor-R1 Mc7 CAS-TypeIC 0.006195 2 31 14393
14422 -|- "-|TGG
" LFPVWAMN_CDS_0023 11.0 339 No_MMseqs_PHROG_hit
LFPVWAMN_CDS_0023 tail tail length tape measure protein
PHANOTATE_1.5.1 -4.653279167529952e+23 0.0
>spacer1_Mc7_2 Bath-R1 Mc7 CAS-TypeIC 0.0003453 1 27 19868
19894 TCG|GGG "CCC|-
" YLSDQRHL_CDS_0032 11.0 15 No_MMseqs_PHROG_hit
YLSDQRHL_CDS_0032 head and packaging terminase large
subunit PHANOTATE_1.5.1 -640999.0411576249 0.0
>spacer1_Bath_1 Bath-R2 Bath CAS-TypeIC 1,769E-05 1 33 32896
32934 -|- "-|-
"
>spacer24_McNor_2 Bath-R2 McNor CAS-TypeIC 4,247E-11 2 34
32661 32629 -|- "-|-
" LHBQFDQA_CDS_0045 11.0 736 No_MMseqs_PHROG_hit
LHBQFDQA_CDS_0045 PHANOTATE_1.5.1 -
61.27031988447617 0.0
>spacer58_MIR_2 Bath-R2 MIR CAS-TypeIC 1,769E-05 34 2 32896
32934 -|- "-|-
"
>spacer8_Mc7_11 Bath-R2 Mc7 noCAS_1 4,247E-11 1 33 44056
44088 -|- "-|-
"
>spacer24_McNor_2 IO1-R1 McNor CAS-TypeIC 0.003535 2 34
22918 22950 -|- "-|-
" QSXAMBDO_CDS_0042 11.0 736 No_MMseqs_PHROG_hit
QSXAMBDO_CDS_0042 PHANOTATE_1.5.1 -
47.50827216729887 0.0
>spacer8_Mc7_11 IO1-R1 Mc7 noCAS_1 4,247E-11 1 33 11549
11517 -|- "-|-

"	QSXAMBDO_CDS_0016	11.0	2345	No_MMseqs_PHROG_hit
	QSXAMBDO_CDS_0016	transcription regulation	transcriptional regulator	PHANOTATE_1.5.1	-34.75382359727734	0.0
>spacer1_Mc7_2	KN2-R1	Mc7	CAS-TypeIC	1,708E-03	1	27	39166
	39140	-|-	"CC|-
"	DFLLIFOK_CDS_0039	11.0	15	No_MMseqs_PHROG_hit
	DFLLIFOK_CDS_0039	head and packaging	terminase large subunit	PHANOTATE_1.5.1	-212536.99302893772	0.0
>spacer1_Mc7_2	McNor-R2	Mc7	CAS-TypeIC	1,708E-03	1	27	25359
	25385	-|GGG	"CCC|-
"	WDFLIPAM_CDS_0040	11.0	15	No_MMseqs_PHROG_hit
	WDFLIPAM_CDS_0040	head and packaging	terminase large subunit	PHANOTATE_1.5.1	-239643.5004742173	0.0
>spacer73_IO1_2	McNor-R1	IO1	CAS-TypeIE	4,247E-11	33	1	15407
	15439	-|-	"-|-
"	LFPVWAMN_CDS_0023	11.0	339	No_MMseqs_PHROG_hit
	LFPVWAMN_CDS_0023	tail	tail length tape measure protein
	PHANOTATE_1.5.1	-4.653279167529952e+23	0.0
>spacer32_KN2_6	McNor-R1	KN2	CAS-TypeIE	2.25e-12	31	2	18515
	18544	-|-	"-|-
"	LFPVWAMN_CDS_0023	11.0	339	No_MMseqs_PHROG_hit
	LFPVWAMN_CDS_0023	tail	tail length tape measure protein
	PHANOTATE_1.5.1	-4.653279167529952e+23	0.0
>spacer34_KN2_6	McNor-R1	KN2	CAS-TypeIE	2,694E-09	33	1	15341
	15373	-|-	"-|-
"	LFPVWAMN_CDS_0023	11.0	339	No_MMseqs_PHROG_hit
	LFPVWAMN_CDS_0023	tail	tail length tape measure protein
	PHANOTATE_1.5.1	-4.653279167529952e+23	0.0
>spacer35_KN2_6	McNor-R1	KN2	CAS-TypeIE	4,247E-11	1	33	16537
	16505	-|-	"-|-
"	LFPVWAMN_CDS_0023	11.0	339	No_MMseqs_PHROG_hit
	LFPVWAMN_CDS_0023	tail	tail length tape measure protein
	PHANOTATE_1.5.1	-4.653279167529952e+23	0.0
>spacer57_BH_1	McNor-R1	BH	CAS-TypeIE	1,562E-07	1	33	18961
	18993	-|-	"-|-
"	LFPVWAMN_CDS_0024	11.0	8917	No_MMseqs_PHROG_hit
	LFPVWAMN_CDS_0024		PHANOTATE_1.5.1	-286081.09863004903	0.0
>spacer36_MIR_1	McNor-R1	MIR	CAS-TypeIE	1,562E-07	1	33	18961
	18993	-|-	"-|-
"	LFPVWAMN_CDS_0024	11.0	8917	No_MMseqs_PHROG_hit
	LFPVWAMN_CDS_0024		PHANOTATE_1.5.1	-286081.09863004903	0.0
>spacer28_MIR_2	McNor-R1	MIR	CAS-TypeIC	4,247E-11	1	33	19649
	19617	-|-	"TTC|-
"	LFPVWAMN_CDS_0024	11.0	8917	No_MMseqs_PHROG_hit
	LFPVWAMN_CDS_0024		PHANOTATE_1.5.1	-286081.09863004903	0.0
>spacer12_Mc7_1	McNor-R1	Mc7	CAS-TypeIE	8,111E-06	1	33	14915
	14947	-|-	"-|-
"	LFPVWAMN_CDS_0023	11.0	339	No_MMseqs_PHROG_hit
	LFPVWAMN_CDS_0023	tail	tail length tape measure protein
	PHANOTATE_1.5.1	-4.653279167529952e+23	0.0
>spacer28_Mc7_2	16-5-R2	Mc7	CAS-TypeIC	0.007979	3	35	44757
	44789	-|-	"-|-
"	QRZSKLUB_CDS_0065	11.0	2837	No_MMseqs_PHROG_hit
	QRZSKLUB_CDS_0065	tail	tail protein	PHANOTATE_1.5.1	-37.10734793549466	0.0
>spacer37_Mc7_2	16-5-R2	Mc7	CAS-TypeIC	0.01279	33	1	31593
	31625	-|-	"-|-

"	QRZSKLUB_CDS_0056	11.0	290	No_MMseqs_PHROG_hit
	QRZSKLUB_CDS_0056	tail	minor tail protein	PHANOTATE_1.5.1
	-8491.711845480386	0.0
>spacer11_Mc7_2	McNor-R1	Mc7	CAS-TypeIC	2.02e-05	32	3	15092
	15121	-|-	"-|CGG
"	LFPVWAMN_CDS_0023	11.0	339	No_MMseqs_PHROG_hit
	LFPVWAMN_CDS_0023	tail	tail length tape measure protein
	PHANOTATE_1.5.1	-4.653279167529952e+23	0.0
>spacer19_Mc7_2	McNor-R1	Mc7	CAS-TypeIC	1,562E-07	1	33	17648
	17680	-|-	"-|-
"	LFPVWAMN_CDS_0023	11.0	339	No_MMseqs_PHROG_hit
	LFPVWAMN_CDS_0023	tail	tail length tape measure protein
	PHANOTATE_1.5.1	-4.653279167529952e+23	0.0
>spacer22_Mc7_2	McNor-R1	Mc7	CAS-TypeIC	0.0002671	3	35	21583
	21615	TCA|-	"-|-
"	LFPVWAMN_CDS_0026	11.0	No_PHROGs_HMM	No_MMseqs_PHROG_hit
	LFPVWAMN_CDS_0026			PHANOTATE_1.5.1	-
85.89506665349802	0.0
>spacer24_Mc7_2	McNor-R1	Mc7	CAS-TypeIC	7,403E-02	36	1	19858
	19893	CC|-	"-|-
"	LFPVWAMN_CDS_0024	11.0	8917	No_MMseqs_PHROG_hit
	LFPVWAMN_CDS_0024			PHANOTATE_1.5.1	-
286081.09863004903	0.0
>spacer28_Mc7_2	McNor-R1	Mc7	CAS-TypeIC	2.37e-05	3	35	24823
	24855	-|-	"-|-
"	LFPVWAMN_CDS_0030	11.0	2837	No_MMseqs_PHROG_hit
	LFPVWAMN_CDS_0030	tail	tail protein	PHANOTATE_1.5.1	-
23.65314923555232	0.0
>spacer37_Mc7_2	McNor-R1	Mc7	CAS-TypeIC	6,395E-03	33	1	11256
	11288	-|-	"-|-
"	LFPVWAMN_CDS_0019	11.0	290	No_MMseqs_PHROG_hit
	LFPVWAMN_CDS_0019	tail	minor tail protein	PHANOTATE_1.5.1
	-183.4415478143869	0.0
